
JOURNAL INFORMATION
==============================
NLM Title Abbreviation: Insights Imaging
Journal ID: Insights Imaging
NLM Journal ID: 101532453

Insights into Imaging

EISSN: 1869-4101
Publisher: Springer

ARTICLE INFORMATION
==============================
PMCID: PMC11408449
DOI: 10.1186/s13244-024-01766-w
Article ID: 1766
Article version: 1
Subjects: Meeting Abstracts

ECR 2024 Book of Abstracts

Electronic publication date: 2024 Sep 18
Publication date: 2024 Sep
Volume: 15
Issue: Suppl 2
Issue sponsor: Publication of this supplement was funded by the European Congress of Radiology.
Electronic Location ID: 223
Copyright: © The Author(s) 2024
Copyright year: 2024
License: Open Access This article is licensed under a Creative Commons Attribution 4.0 International License, which permits use, sharing, adaptation, distribution and reproduction in any medium or format, as long as you give appropriate credit to the original author(s) and the source, provide a link to the Creative Commons licence, and indicate if changes were made. The images or other third party material in this article are included in the article's Creative Commons licence, unless indicated otherwise in a credit line to the material. If material is not included in the article's Creative Commons licence and your intended use is not permitted by statutory regulation or exceeds the permitted use, you will need to obtain permission directly from the copyright holder. To view a copy of this licence, visit http://creativecommons.org/licenses/by/4.0/.
License URL: https://creativecommons.org/licenses/by/4.0/

Conference name: European Congress of Radiology 2024
Conference location: Vienna, Austria
Conference date: 28 February - 3 March 2024
issue-copyright-statement: © European Society of Radiology (ESR) 2024

==============================

EUROPEAN CONGRESS OF RADIOLOGY

ECR2024

Insights into Imaging
Volume 15
Supplement 2

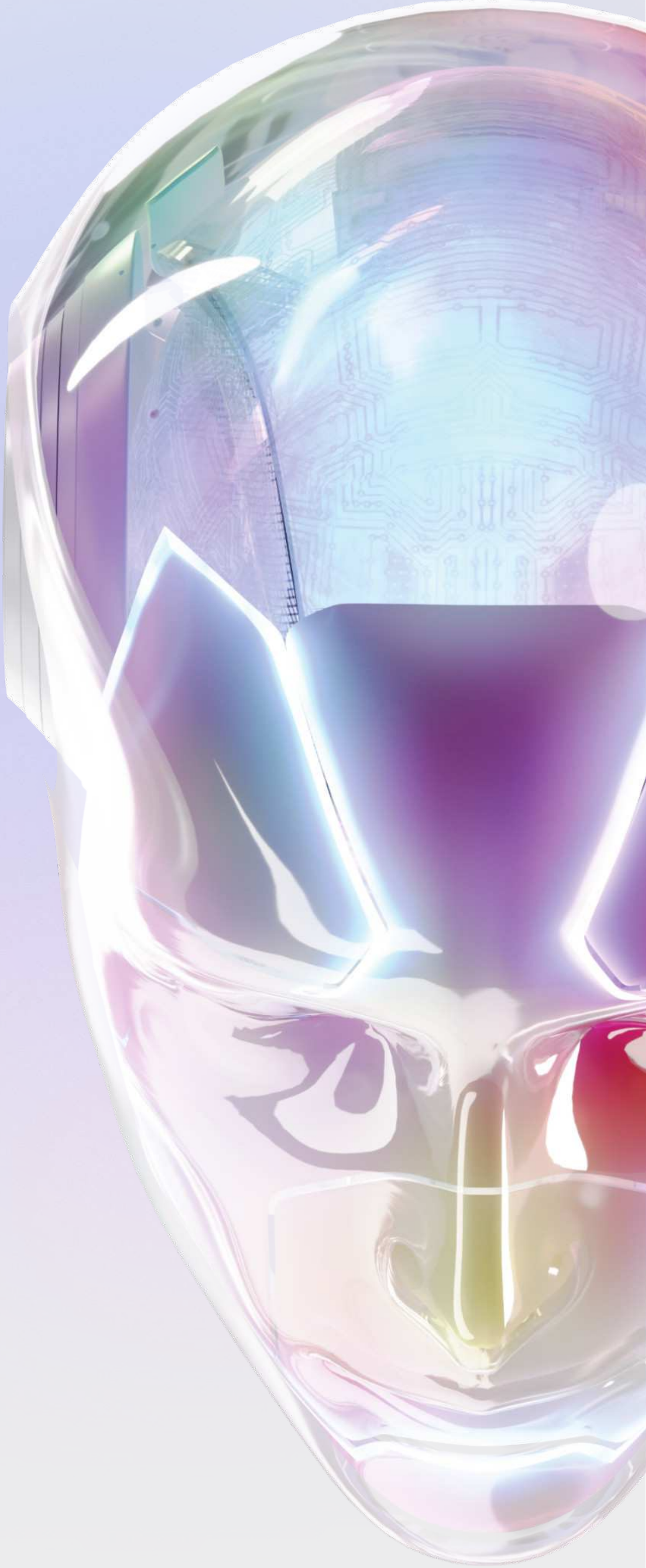

### Book of Abstracts

Contents

3 - 284 ECR 2024 Abstract based Programme February 28 - March 3, 2024

ESR EUROPEAN SOCIETY OF RADIOLOGY × Springer

**Disclaimer**

The ECR 2024 Book of Abstracts is published by the European Society of Radiology (ESR) and summarises the presentations accepted to be held at the European Congress of Radiology 2023 (Vienna, Austria, February 28 - March 3, 2024).

Abstracts were submitted by the authors warranting that good scientific practice, copyrights and data privacy regulations have been observed and relevant conflicts of interest declared.
Abstracts reflect the authors' opinions and knowledge. The ESR does not give any warranty about the accuracy or completeness of medical procedures, diagnostic procedures or treatments contained in the material included in this publication. The views and opinions presented in ECR abstracts and presentations, including scientific, educational and professional matters, do not necessarily reflect the views and opinions of the ESR.

In no event will the ESR be liable for any direct or indirect, special, incidental, consequential, punitive or exemplary damages arising from the use of these abstracts.

The Book of Abstracts and all of its component elements are for general educational purposes for health care professionals only and must not take the place of professional medical advice. Those seeking medical advice should always consult their physician or other medical professional.

In preparing this publication, every effort has been made to provide the most current, accurate, and clearly expressed information possible. Nevertheless, inadvertent errors in information can occur. The ESR is not responsible for typographical errors, accuracy, completeness or timeliness of the information contained in this publication.

The ECR 2024 Book of Abstracts is a supplement to Insights into Imaging (1869-4101) and published under Creative Commons Attribution 4.0 International License.

Insights Imaging (2024) Vol 15 (Suppl 2)
https://doi.org/10.1186/s13244-024-01766-w
Published: September 2024

### ECR 2024
### Abstract-based Programme

Research Presentation Sessions (RPS)

**Wednesday, February 28 .............. 3**
**Thursday, February 29................ 65**
**Friday, March 1 .........................129**
**Saturday, March 2 ......................189**
**Sunday, March 3........................249**

### Wednesday, February 28

08:00-09:00 **Research Stage 1**

### Research Presentation Session: Abdominal Viscera & GI Tract

### RPS 101

### Pre- and post-treatment imaging in cirrhosis and HCC

**Moderator**
J. Podgorska; Warsaw/PL
*(jpodgo@gmail.com)*
**Author Disclosures:**
Joanna Podgorska: Founder: Bayer; Speaker: Bayer, Siemens

### RPS 101-2

**Abbreviated liver MRI with second shot arterial phase image to assess the viability of treated HCC**
S. Kim, N. K. Lee, S-B. Hong, *J. Han*; Busan/KR
*(mickypanda7@gmail.com)*

**Purpose:** The purpose of this study was to evaluate the feasibility of abbreviated liver MRI (AMRI) with second shot arterial phase (SSAP) for the viability of treated HCC.
**Methods or Backround:** We retrospectively identified 95 patients (70 men, 25 women; mean age, 68.7 years) between March 2021 and February 2022 meeting the following inclusion criteria; (1) patients with locoregional therapy for HCC who performed the modified gadoxetic acid-enhanced liver MRI protocol including the routine dynamic imaging after the first injection of 6 mL and SSAP imaging after the second injection of 4 mL and (2) available reference standard for tumour viability in treated observation. Two radiologists independently reviewed two MRI sets: full-protocol MRI and AMRI with SSAP. In the full-protocol MRI set, both reviewers assigned observations according to the liver imaging reporting and data system treatment response (LR-TR) algorithm (TR-viable, TR-equivocal, or TR-nonviable). In AMRI with SSAP set, we assigned a abbLR-TR category (abbLR-TR viable, abbLR-TR equivocal, or abbLR-TR nonviable) according to arterial hyperenhancement in SSAP and Hepatobiliary phase hypointensity, using the modified version of LI-RADS treatment response algorithm. We compared the diagnostic performance between two MRI sets.
**Results or Findings:** In 95 patients, 42 patients (44.2%) had viable lesions, and 53 patients (55.8%) had nonviable lesions. There was no significant difference in sensitivity for treated lesions between full-protocol MRI assigned according to the LR-TR algorithm and AMRI with SSAP assigned according to abbLR-TR category (Full-protocol, 73.8 % (31/42); AMRI with SSAP, 71.4 % (30/42); p=1.00). There was also no significant difference in specificity between two protocols (Full-protocol, 98.1 % (52/53); AMRI with SSAP, 96.2 % (51/53); p=1.00).
**Conclusion:** The abbLR-TR score in AMRI with SSAP showed the non-inferior diagnostic performance to the full-protocol MRI in evaluating the viability for the treated HCCs.
**Limitations:** No limitations were identified.
**Ethics committee approval:** The study was approved by PNUH - 2309-013-131.
**Funding for this study:** No funding was received for this study.
**Author Disclosures:**
Suk Kim: Nothing to disclose
Seung-Baek Hong: Nothing to disclose
Jaewon Han: Nothing to disclose
Nam Kyung Lee: Nothing to disclose

### RPS 101-3

**The imaging features of LI-RADS nonviable or equivocal lesions in the first follow-up evaluation after TACE for HCC to predict recurrence**
*S-H. Zhang*, Y-C. Wang; Jiangsu/CN
*(shuhangzhang@outlook.com)*

**Purpose:** The purpose of this study was to investigate whether the imaging features of lesions evaluated as LI-RADS nonviable or equivocal during the first post-TACE imaging follow-up can predict recurrence in HCC patients.
**Methods or Backround:** A total of 171 lesions from 149 patients were included in this study. All patients underwent at least three enhanced magnetic resonance imaging examinations, with the first one conducted before TACE and the second one during the first follow-up visit the one to two months after TACE. The lesions were divided into two groups, no recurrence during long-term follow-up (n = 128) and recurrence during long-term follow-up (n = 43). Imaging features evaluated included irregular shape, size, internal homogeneity, non-smooth margin, arterial peritumoural enhancement, rim enhancement in portal venous phase or delay phase, peritumoural hyperintensity in T2WI or DWI. Using univariate and multivariate logistic regression analysis to investigate which imaging features at the first follow-up can predict lesion recurrence at 6-12 months after TACE.
**Results or Findings:** After univariate and multivariate analysis showed that non-smooth margin (OR, 3.96, 95% CI, 1.66, 9.44, P = 0.002) and peritumoural hyperintensity in T2WI or DWI (OR, 7.74, 95% CI, 3.32, 18.06, P < 0.001) were independent risk factors for recurrence of LI-RADS nonviable lesions at 6 to 12 months. The area under the ROC curve using these two factors to predict the recurrence of LI-RADS nonviable lesions at 6 to 12 months was 0.754.
**Conclusion:** Non-smooth border and peritumoural hyperintensity in T2WI or DWI are imaging features to predict recurrence in HCC patients with LI-RADS nonviable lesions evaluated on the first post-TACE imaging follow-up.
**Limitations:** This is a retrospective study where selection bias is an inherent flaw. It can be included in subsequent research for analysis of patient baseline imaging features.
**Ethics committee approval:** This is a retrospective study. It was approved for retrospective analyses by the institutional review board of this hospital, with an informed consent waiver.
**Funding for this study:** No funding was received for this study.
**Author Disclosures:**
Yuan-Cheng Wang: Nothing to disclose
Shu-Hang Zhang: Nothing to disclose

### RPS 101-4

**MRI evaluations of focal liver reactions (FLR) following stereotactic body radiotherapy (SBRT) for small hepatocellular carcinoma: relationship between MRI features and dose-volumetric parameters**
*R. Huang*; Guangzhou/CN
*(huangrq1@sysucc.org.cn)*

**Purpose:** The aim of this study was to assess the MRI features of FLR for patients with small hepatocellular carcinoma (HCC) treated with stereotactic body radiotherapy (SBRT), and to investigate the relationship between MRI features of FLR and dose–volumetric parameters.
**Methods or Backround:** Between September 2018 and April 2021, 102 patients with small HCC treated with SBRT were evaluated according to the entry criteria. After SBRT, all patients underwent regular follow-ups with blood tests and dynamic MR scans at a median follow-up of more than 18 months. Two radiologists assessed imaging features of FLR in MRI sequences at 3 to 6 months. To assess the deterioration of hepatic function, we evaluated the ALBI score. To identify the parameters of predicting the radiation-induced hepatic toxicity, univariate and multivariate analyses were used to select independent risk factors and a nomogram model was established.
**Results or Findings:** The MRI features of FLR were divided into three types according to T1WI, T2WI, arterial phase, portal-venous phase and delayed phase signals: low/high/high/high/high in 27 patients (Type A), low/low/low/low/low in 48 patients (Type B) and low/low/iso (or high)/iso (or high)/high in 27 patients (Type C). ALBI score at 3 to 6 months after SBRT was the significant factor that differed between Type A (worse prognosis) and alternatives (Type B and C). Multivariate analysis showed that PTV (P=0.028) and V5Gy (normal liver volumes receiving from 5Gy) (P=0.003) were the significant dose–volumetric parameters for predicting Type A. Both factors were used to construct the nomogram model, which showed good performance (AUC=0.772).
**Conclusion:** The MR features of FLR can reflect the deterioration of the hepatic function for small HCC by SBRT. The PTV and V5Gy were the significant parameters that predicted the risk of the deterioration of hepatic function for different MR types of FLR.
**Limitations:** No limitations were identified.
**Ethics committee approval:** No information provided by the submitter.
**Funding for this study:** No funding was received for this study.
**Author Disclosures:**
Runqian Huang: Nothing to disclose

### RPS 101-5

**Ascites in patients with hepatocellular carcinoma undergoing transarterial chemoembolisation: the more, the worse?**
*L. Müller*[1], D. Bender[1], F. Stöhr[1], A. Mähringer-Kunz[1], J. Mittler[1], A. Weinmann[1], R. Klöckner[2], F. Hahn[1]; [1]Mainz/DE, [2]Lübeck/DE

**Purpose:** Preliminary work has shown that portal hypertension plays a key role for the prognosis in patients with hepatocellular carcinoma (HCC) undergoing transarterial chemoembolisation (TACE). Specifically, the presence of ascites appears to be a strong negative predictor for these patients. However, it remains unclear whether different ascites volumes influence prognosis. Therefore, the aim of this work was to investigate the influence of different ascites volumes on survival for patients with HCC undergoing TACE.
**Methods or Backround:** A total of 351 patients with HCC treated at our tertiary referral centre between 2010 and 2020 were included. In patients with ascites, the fluid was segmented, and the volume quantified by slice-wise addition using contrast-enhanced CT imaging. The patients with ascites were subclassified using cut-off values previously suggested for patients with liver cirrhosis: A0 = 0ml, A1 ≤ 300ml, and A2 > 300ml ascites. Median overall survival (OS) was calculated.
**Results or Findings:** Ascites was present in 102 (29.1%) patients. Median OS without ascites was 16.5 months, and therefore significantly longer than in patients with ascites (6.4 months, P<0.001). Patients were further classified as n(A0)=249(70.9%), n(A1)=43(12.3%), n(A2)=59(16.8%). The corresponding median OS yielded 16.5, 9.9 and 4.0 months for A0-A2, respectively (overall P<0.001, pairwise comparison A0 vs A1 P=0.03, A1 vs A2 P<0.001).
**Conclusion:** Ascites in patients with HCC undergoing TACE is associated with a bad prognosis. Our results indicate an association between the amount of ascites and OS. Not only the presence but also the amount of ascites is relevant. The quantitative assessment applied here can help improve clinical decision making.
**Limitations:** The retrospective design and single centre nature of the study were identified as limitations.
**Ethics committee approval:** The study has been approved by the local ethics committee responsible (Rhineland-Palatinate, Germany).
**Funding for this study:** This study was funded by the Deutsche Forschungsgemeinschaft (DFG, German Research Foundation); project number: 518477942.
**Author Disclosures:**
Roman Klöckner: Nothing to disclose
Fabian Stöhr: Nothing to disclose
Felix Hahn: Nothing to disclose
Jens Mittler: Nothing to disclose
Daniel Bender: Nothing to disclose
Lukas Müller: Nothing to disclose
Arndt Weinmann: Nothing to disclose
Aline Mähringer-Kunz: Nothing to disclose

### RPS 101-6

**Adding estimates for central venous pressure boosts performance of non-invasive assessment of portosystemic gradient prior to TIPS implantation**
F. Stoehr[1], L. Müller[1], T. Loew[1], C. Labenz[1], J. Mittler[1], R. Klöckner[2], M. B. B. Pitton[1], *F. Hahn*[1]; [1]Mainz/DE, [2]Lübeck/DE

**Purpose:** We aimed to evaluate the accuracy of non-invasive scores to predict markedly increased portosystemic gradient (PSG) in patients with clinically significant portal hypertension (CSPH) undergoing TIPS procedure and to further investigate patients without markedly increased PSG at the time of intervention.
**Methods or Backround:** We included patients who underwent TIPS implantation at our tertiary care center between 2010 and 2022 with CT imaging and complete laboratory workup prior to TIPS and without history of splenectomy. Liver and spleen volume were automatically assessed using a commercially available in-house trained segmentation software. Scores using CT data by Iranmanesh et al. and Kihira et al. were assessed with regard to classification accuracy. Moreover, differences between the subgroups of patients with and without PSG>10mmHg were analysed.
**Results or Findings:** Preliminary results after assessing 200 patients show a mean PSG prior to TIPS of 16.5±4.8mmHg. Out of these patients, 22 with CSPH had a PSG≤10mmHg at the time of the TIPS procedure. Both scores showed high sensitivity (89% and 87% resp.), but poor specificity (36% and 41% resp.), in predicting a markedly increased PSG. ROC-analysis yielded an AUC of 0.67 and 0.70. In the subgroup of patients with low PSG, central venous pressure (CVP) was significantly elevated compared to patients with high PSG (13.4±5.9mmHg vs 7.7±3.6mmHg, p=0.003), while distribution of portal venous pressure was not significantly different (p=0.51). Adding IVC diameter as an estimator for CVP in linear regression resulted in a significant AUC increase to 0.78 and 0.79 resp. (p=0.04 and p=0.01).
**Conclusion:** Including IVC diameter boosted the accuracy of CT morphological scores for assessing elevated PSG, especially increasing specificity.
**Limitations:** This was a single-centre, retrospective study.
**Ethics committee approval:** The study was approved by the local ethics committee (Medical Association of Rhineland Palatinate, Mainz, Germany). Informed consent was waived due to the retrospective nature of the study.
**Funding for this study:** No funding was received for this study.
**Author Disclosures:**
Roman Klöckner: Nothing to disclose
Michael Bernhard B. Pitton: Nothing to disclose
Fabian Stoehr: Nothing to disclose
Christian Labenz: Nothing to disclose
Felix Hahn: Nothing to disclose
Jens Mittler: Nothing to disclose
Lukas Müller: Nothing to disclose
Tilla Loew: Nothing to disclose

### RPS 101-7

**The comparison of preoperative and intraoperative graft volumes in living donor liver transplantation: effect of graft type on accuracy**
*H. Özen Atalay*, T. Kanmaz, A. Durur Karakaya; Istanbul/TR
*(handeozen15@gmail.com)*

**Purpose:** The purpose of this study was to conduct a retrospective evaluation of the difference between the preoperative estimated and the actual intraoperative graft volume detected in living liver transplantation donors in terms of graft types.
**Methods or Backround:** The present retrospective study was performed at a single centre, involving a total of 219 donors. The analysis of total, right, left lobe and left lateral segment liver volumes were conducted in the venous phase abdominal CT by using Myrian software. The preoperative graft volumes were compared to the intraoperative graft volumes. Intraclass correlation coefficient, Blant Altman and one-sample tests were used to calculate correlation coefficient and to detect error of estimation.
**Results or Findings:** 128 donors underwent right hepatectomy, 81 donors had left lateral segmentectomy and 10 donors had left hepatectomy. The estimated mean volumes of the right lobe, left lobe, and left lateral segment were 885.39 mL, 402.2 mL, and 241.14 mL, respectively. The mean intraoperative weights of the right lobe, left lobe, and left lateral segment were 820.65 mL, 504.2 mL, and 250.82 mL, respectively. The automated preoperative right lobe volume assessment had a higher correlation with intraoperative graft weight (r=0,753) than the left lobe and left lateral segment volume assessment (r=0.468 and r=0.007, respectively). The mean percentages of error in volume estimation were calculated at 12,20%, 23,79% and 68,53% for the right lobe, left lobe and left lateral segment, respectively.
**Conclusion:** This study underscores the need for more accurate preoperative volume estimations, particularly for the left lobe and left lateral segment. These areas showed lower correlation, higher error ratios, suggesting that extra caution is needed when planning transplantation procedures involving these segments.
**Limitations:** Our retrospective study included a limited number of patients from a single institution.
**Ethics committee approval:** This study was approved by Koc University Biomedical Research Ethics Committee, Istanbul/Turkey.
**Funding for this study:** No funding was received for this study.
**Author Disclosures:**
Afak Durur Karakaya: Nothing to disclose
Hande Özen Atalay: Nothing to disclose
Turan Kanmaz: Nothing to disclose

**08:00-09:00** **Research Stage 2**

### Research Presentation Session: Breast

### RPS 102

### Breast imaging biomarkers

**Moderator**
F. Kilburn-Toppin; Cambridge/UK
**Author Disclosures:**
Fleur Kilburn-Toppin: Advisory Board: Genesis Care

### RPS 102-2

**MRI radiomics analysis predicting early recurrence in breast cancer patients who are candidates for neoadjuvant chemotherapy**
*C. M. L. Trombadori*, A. D'Angelo, E. Boccia, L. Boldrini, G. Franceschini, D. Giannarelli, A. Franco, A. Fabi, P. Belli; Rome/IT
*(charlotte.trombadori@gmail.com)*

**Purpose:** The aim of this study was to assess the role of pure radiomic predictive models and combined models with clinical/radiological variables applied to Magnetic Resonance Imaging (MRI) in predicting early recurrence (ER: disease-free survival <3 years after surgery) in breast cancer patients undergoing neoadjuvant chemotherapy (NAT). Identifying tools for non-invasive pre-treatment predictors of clinical outcomes, particularly recurrence, is necessary for better patient stratification and treatment selection.
**Methods or Backround:** Patients with breast cancer who underwent staging MRI, NAT, and surgery at our centre (from 2012 to 2021) were included. Clinical variables evaluated included pathological complete response, ER, and tumour subtype. Radiological variables included tumour response according to RECIST criteria. Four breast radiologists reviewed MRI, annotated regions of interest, and extracted radiomic features. Pure radiomic models and combined models (clinical-radiological, radiological-radiomic, and clinical-radiomic) were developed. The area under the curve (AUC) was calculated for each model, and the models were compared in terms of accuracy, sensitivity, and specificity.
**Results or Findings:** A total of 211 patients were included, with an ER prevalence of 11.34%. Patients with complete or partial response to NAT and Luminal tumour subtype had a lower likelihood of developing ER (p = 0.001 and p = 0.037, respectively). Two radiomic features were statistically significant associated with ER: F_cm_2.5D.energy and F_cm_2.5D.joint.entr. The AUC values for combined models were 0.77 (radiological-radiomic model), 0.68 (clinical-radiomic model), and 0.74 (clinical-radiological model). The radiological-radiomic model was significantly more accurate in predicting ER than the pure radiological and radiomic models (p<0.001 and p<0.03, respectively).
**Conclusion:** The radiological-radiomic model, combining radiomic features and RECIST criteria, showed the most promising results in predicting ER.
**Limitations:** The small sample size and monocentric nature of the study were identified as limitations.
**Ethics committee approval:** This study was approved by an ethics committee; ID cineca: 6081.
**Funding for this study:** No funding was received for this study.
**Author Disclosures:**
Antonio Franco: Nothing to disclose
Luca Boldrini: Nothing to disclose
Anna D'Angelo: Nothing to disclose
Alessandra Fabi: Nothing to disclose
Gianluca Franceschini: Nothing to disclose
Charlotte Marguerite Lucille Trombadori: Nothing to disclose
Diana Giannarelli: Nothing to disclose
Edda Boccia: Nothing to disclose
Paolo Belli: Nothing to disclose

### RPS 102-3

**PET/CT radiomics integrated with clinical indexes as a tool to predict Ki67 in breast cancer: a pilot study**

*C. Shen*[1], Y. Liao[2], X. Yu[2]; [1]Xi'an/CN, [2]Shanghai/CN
*(1187385519@qq.com)*

**Purpose:** This study aims to assess the predictive value of radiomics features extracted from 18F-FDG PET/CT, in combination with clinical characteristics, for estimating Ki67 expression in patients with breast cancer.
**Methods or Backround:** A total of 114 patients diagnosed with breast cancer and examined using 18F-FDG PET/CT were included. Patients were randomly assigned to a training set (n = 79, including 55 cases of Ki67+ and 24 cases of Ki67-) and a validation set (n = 35, comprising 24 cases of Ki67+ and 11 cases of Ki67-). Thirteen clinical characteristics and 704 radiomics features were extracted. Feature selection involved univariate logistic analysis, Max-Relevance and Min-Redundancy, least absolute shrinkage and selection operator regression, and Spearman test. Three models were developed, including the clinical model, the radiomics model, and the combined mode. Model performance was evaluated using receiver operating characteristic (ROC) curve, and clinical utility was assessed through decision curve analysis (DCA).
**Results or Findings:** The N stage, tumour morphology, maximal standard uptake, and the longest diameter were significantly differed between Ki67+ and Ki67- groups (all P<0.05). Seven radiomics features were selected for the radiomics model. The area under the ROC curve (AUC) of the combined model in the training and test group was 0.90 (95% CI: 0.82–0.97) and 0.81 (95% CI: 0.64–0.99), respectively. The combined model significantly outperformed both the radiomics model and the clinical model alone (both P<0.05). The DCA curve demonstrated the superior clinical utility of the combined model compared to the clinical model and radiomics model.
**Conclusion:** PET/CT image-based radiomics features combined with clinical features have the potential to predict Ki67 expression in BC.
**Limitations:** The retrospective nature of the study and its small sample size were identified as limitations.
**Ethics committee approval:** This retrospective study was conducted at the First Affiliated Hospital of Xi 'an Jiaotong University (NCT05826197), and the study protocol was approved by the Ethics Committee of Xi 'an Jiaotong University (IRB-SOP-AF-16).
**Funding for this study:** This study was funded by the National Natural Science Foundation of China (No. 82272073), the Key Research and Development Plan of Shaanxi Province (2023-YBSF-480), and the Natural Science and basic research project of Shaanxi Province (2023-JC-QN-0903).
**Author Disclosures:**
Xiao Yu: Nothing to disclose
Cong Shen: Nothing to disclose
Yuting Liao: Nothing to disclose

### RPS 102-4

**Automatic breast segmentation-based radiomics for classifying breast composition and detecting neoplastic lesions on chest CT**

*G. Dasegowda*[1], M. Frölich[2], S. Dalal[3], S. R. Digumarthy[3], P. Kaviani[3], L. Karout[3], R. Fahimi[3], E. Garza Frias[3], M. K. Kalra[3]; [1]Little Rock, AR/US, [2]Munich/DE, [3]Boston, MA/US
*(giridhar.dasegowda@gmail.com)*

**Purpose:** The aim of this study was to evaluate if automatic breast segmentation-based radiomics can differentiate between benign and malignant breast lesions and classify the breast based on tissue composition on contrast-enhanced chest CT.
**Methods or Backround:** Our retrospective study included 882 female patients (mean age 55 ± 13 years) who underwent both contrast-enhanced chest CT and mammography within one year. Patients with surgical clips, prior breast surgeries, and those with artifacts projecting over the breast tissue on CT images were excluded. The tissue composition (dense, fibroglandular, fatty) and BIRADS score reported on mammography examinations were recorded. Furthermore, when suspicious for malignancy, the pathology report was used as the gold standard for classifying benign and malignant breast tissues. Thin-section CT images (1-1.25 mm) were reconstructed and processed with a Radiomics software prototype (Frontier, Siemens Healthineers) for segmentation and feature extractions of the left and right breast (1688 radiomic features) were analysed with multiple logistic regression and area under the curve for precision-recall curve analysis (R Statistical software).
**Results or Findings:** Automated segmentation-based radiomics differentiated the breast tissue as dense (n=779), fibroglandular (n=876), and fatty (n=108) with an 0.90 AUC (p<0.001). Of the 1764 breasts with a BIRADS score and pathology confirmation of malignancy, there were 1545 benign and 219 malignant breast tissues. To differentiate benign and malignant lesions in all breast tissue, radiomics had an AUC of 0.78 (p<0.001). To differentiate benign and malignant lesions in fatty and fibro glandular breast tissue (excluding dense breast tissue), radiomics had an AUC of 0.82 (p<0.001).
**Conclusion:** Radiomics can reliably differentiate breast tissue composition as well as predict malignancy in fibroglandular and fatty breast tissues with high accuracy. Radiomics can help assess breast tissues and suspicious breast lesions on contrast-enhanced chest CT examinations.
**Limitations:** This was a single centre study.
**Ethics committee approval:** Mass General Brigham IRB approved this study.
**Funding for this study:** No funding was received for this study.
**Author Disclosures:**
Giridhar Dasegowda: Nothing to disclose
Lina Karout: Nothing to disclose
Emiliani Garza Frias: Nothing to disclose
Mannudeep K. Kalra: Research/Grant Support: Siemens Healthineers, Riverrain technologies
Matthias Frölich: Research/Grant Support: Siemens Healthineers
Parisa Kaviani: Nothing to disclose
Sophia Dalal: Nothing to disclose
Roshan Fahimi: Nothing to disclose
Subba Rao Digumarthy: Research/Grant Support: Vuno, Lunit, GE, Qure AI

### RPS 102-5

**Prediction of human epidermal growth factor receptor 2 (HER2) status in breast cancer by mammographic radiomics features and clinical characteristics: a multicentre study**

*Y. Deng*, Y. Lu; Shanghai/CN
*(20211230041@fudan.edu.cn)*

**Purpose:** The study aimed to preoperatively evaluate the human epidermal growth factor 2 (HER2) status in breast cancer using radiomics features extracted from digital mammography (DM) and clinical characteristics.
**Methods or Backround:** This study included a cohort of 1512 Chinese women with invasive ductal carcinoma of no special type (IDC-NST) from two different hospitals. 1332 from Institution A, used for training and testing the models, and 180 women from Institution B, as the external validation cohort. The Gradient Boosting Machine (GBM) algorithm was employed to establish a radiomics model and multiomics model using radiomics features and clinical characteristics. Model efficacy was evaluated by the area under the curve (AUC).
**Results or Findings:** The number of HER2-positive patients in the training, testing and external validation cohort was 245 (26.3%), 105 (26.3.8%) and 51 (28.3%) respectively, with no statistical differences among the three cohorts (P = 0.842, Chi-square test). The radiomics model, based solely on radiomics features, achieved an AUC of 0.814 (95% CI: 0.784-0.844) in the training cohort, 0.776 (95% CI: 0.727-0.825) in the testing cohort and 0.702 (95% CI: 0.614-0.790) in the external validation cohort. The multiomics model, incorporating radiomics features with clinical characteristics, consistently outperformed the radiomics model with AUC values of 0.838 (95% CI: 0.810-0.866) in the training cohort, 0.788 (95% CI: 0.741-0.835) in the testing cohort, and 0.722 (95% CI: 0.637-0.811) in the external validation cohort.

**Conclusion:** Our study demonstrates that a model based on radiomics features and clinical characteristics has the potential to accurately predict the HER2 status of breast cancer patients across different centres.
**Limitations:** The limitations were (1) ROIs were manually delineated, (2) the focus was exclusively on the relationship between radiomics features and HER2 status without analyzing other prognostic factors, and (3) the relationship between imaging equipment and radiomics model efficacy was not explored.
**Ethics committee approval:** This study was approved by the Ethics Committee of Fudan University Affiliated Cancer Hospital.
**Funding for this study:** Funding for this study was received from:
1. Clinical Research Plan of SHDC (No. SHDC2020CR4069)
2. Medical Engineering Fund of Fudan University (No. yg2021-029)
3. Shanghai Sailing Program (No. 21YF1404800)
4. Youth Medical Talents –Medical Imaging Practitioner Program (No. 3030256001)
5. Shanghai Municipal Science and Technology Major Project (No. 2018SHZDZX01)
**Author Disclosures:**
Yiping Lu: Nothing to disclose
Yalan Deng: Nothing to disclose

### RPS 102-6

**Performance of radiomic features in STIR sequences in predicting histopathological outcomes of breast cancer**

*G. Rona*[1], M. Arifoğlu[1], T. A. Serel[2], Ö. Adıgüzel Karaoysal[1], Ş. Kökten[1]; [1]Istanbul/TR, [2]Isparta/TR
*(gunayrona@gmail.com)*

**Purpose:** This study aimed to investigate the performance of radiomic features in STIR sequences in predicting the results of histopathological outcomes of invasive breast cancer.
**Methods or Backround:** Patients who underwent MRI before treatment were evaluated retrospectively. Histologic grade, ER, PR, HER-2, Ki-67 expressions and molecular subtypes were noted. Lesions were manually segmented from STIR sequences in the 3D Slicer program and volume of interest (VOI) was obtained. Machine learning (ML) analysis was performed using Python 2.3, the Pycaret library program. Datasets were randomly divided into training (70%) and independent testing set (30%). The performances of ML algorithms were evaluated by area under curve (AUC), accuracy, recall and precision values.
**Results or Findings:** 197 patients with a mean age of 50.72±46 (range 28-82) years were included in the study. The mean lesion size was 23.71±14.86 (5-120) mm. 156 of the patients were luminal A+B (79.2%), 17 were HER-2 positive (8.6%), and 24 were TN BC (12.2%). 156 (79.2%) of the patients were ER +, 41 (20.8%) were ER -, 126 (63.9%) were PR +, 71 (36.1%) were PR -, 58 (29.4%) were HER-2 +, 139 (70.6%) were HER-2 -. 43 (21.8%) of the patients were grade 1, 104 (52.8%) were grade 2, and 50 (25.4%) were grade 3. The best results were obtained in predicting ER status and luminal A+B tumours. In the test set, AUC, accuracy, recall and precision values in ER+/- discrimination were 0.7518, 0.8048, 0.9628, and 0.8194, respectively. AUC, accuracy, recall and precision values in predicting luminal A+B tumours were 0.7229, 0.7958, 0.7958, and 0.6490 respectively.
**Conclusion:** Radiomic features obtained from STIR sequences have the potential to predict ER receptor status and luminal A+B tumours.
**Limitations:** The limitations were that it was a retrospective study and a small patient population.
**Ethics committee approval:** This study was approved with the approval code: 202351425620.
**Funding for this study:** No funding was provided for this study.
**Author Disclosures:**
Tekin Ahmet Serel: Nothing to disclose
Şermin Kökten: Nothing to disclose
Özge Adıgüzel Karaoysal: Nothing to disclose
Günay Rona: Nothing to disclose
Meral Arifoğlu: Nothing to disclose

### RPS 102-7

**Radiogenomics model based on quantitative spatial heterogeneity for predicting pathological complete response and prognosis of triple-negative breast cancer**

*J. Zhou*, Y. Chao, Y. Gu; Shanghai/CN
*(zhoujiayin988@163.com)*

**Purpose:** The study aimed to characterize the spatial heterogeneity of triple-negative breast cancer (TNBC) on MRI and develop a radiogenomics model for predicting both pathological complete response (pCR) and prognosis.
**Methods or Backround:** In this prospective study, TNBC patients undergoing neoadjuvant chemotherapy were enrolled as the radiomics development cohort (n=315); among these patients, those with genetic data were enrolled as the radiogenomics development cohort (n=98). The external validation cohort (n=50) included patients from the DUKE database. Spatial heterogeneity was characterized using features from the tumour body, intratumoral subregions, and peritumoral region. Three radiomics models were developed by logistic regression after selecting features. Two fusion models were developed by further integrating pathological and genomics features (PRM: pathology-radiomics model; GPRM: genomics-pathology-radiomics model). Model performance was assessed with the AUC and decision curve analysis. Prognostic implications were assessed with Kaplan–Meier curves and multivariate Cox regression.
**Results or Findings:** For radiomics models, the multiregional model representing spatial heterogeneity (Model 3) exhibited better pCR prediction with AUCs of 0.87, 0.79, and 0.74 in the training, internal validation, and external validation sets, respectively. GPRM showed the best performance for predicting pCR in the training (AUC=0.97, P=0.015) and validation sets (AUC=0.93, P=0.019). Model 3, PRM and GPRM could stratify patients by disease-free survival, and a predicted non-pCR was associated with poor prognosis (P=0.034, 0.001 and 0.019, respectively).
**Conclusion:** Imaging spatial heterogeneity could effectively predict pCR and prognosis of TNBC. The radiogenomics model could potentially serve as a valuable biomarker to improve the prediction performance.
**Limitations:** No limitations were identified.
**Ethics committee approval:** No information provided by submitter.
**Funding for this study:** No information provided by submitter.
**Author Disclosures:**
Jiayin Zhou: Nothing to disclose
Yajia Gu: Nothing to disclose
You Chao: Nothing to disclose

### RPS 102-8

**Prediction of cancer aggressiveness based on breast MRI features**

*V. Magni*[1], A. Benedek[2], A. Colarieti[2], F. Sardanelli[1]; [1]Milan/IT, [2]San Donato Milanese/IT
*(veronica.magni@unimi.it)*

**Purpose:** This study aimed to investigate the value of breast MRI features for the prediction of tumour aggressiveness, considering the association of Ki67 expression and tumour grade with perilesional oedema, rim enhancement, necrosis sign, and adjacent vessel sign.
**Methods or Backround:** Patients with histologically confirmed malignant breast lesions at preoperative breast MRI and available results of final pathology on surgical specimens were included in this retrospective study. Exclusion criteria were incomplete or suboptimal MRI examinations, incomplete histopathological data, breast implants, and neoadjuvant therapy. Pearson correlation coefficient was calculated to evaluate the strength of association between variables, dichotomising Ki67 expression as low (when <20% positive cells) and as high (when ≥20% positive cells). Multivariable binary logistic regression was then performed to identify significant predictors of Ki67 expression and histological tumour grade.
**Results or Findings:** Among 50 malignant lesions included in the study, 23/50 (48%) showed high-Ki67 expression, while 27/50 (54%) showed low-Ki67 expression. Seven (14%) lesions were grade 1, 26 (52%) were grade 2, and 17 (34%) were grade 3. Ki67 expression showed a positive association with perilesional edema (ρ=0.729, p<0.001), rim enhancement (ρ=0.382, p=0.006), necrosis sign (ρ=0.341, p=0.015), and adjacent vessel sign (ρ=0.327, p=0.020). At multivariable binary logistic regression, perilesional oedema and rim enhancement were significant predictors for high-Ki67 expression, showing odds ratios of 39.7 (p=0.002) and 13.6 (p=0.040) respectively. Perilesional oedema was significantly correlated with histological tumour grade (ρ=0.465, p<0.001).
**Conclusion:** Breast MRI features may have the potential to predict tumour aggressiveness, serving as prognostic and predictive biomarkers usable in clinical practice. The systematic and standardised reporting of these findings in radiological reports should be encouraged to obtain an initial assessment of tumour biological behaviour.
**Limitations:** The limitations were that it was a single-centre retrospective study with a small sample size.
**Ethics committee approval:** This study was approved by the Ethics Committee of IRCCS Ospedale San Raffaele. The protocol code SenoRetro was approved on November 9th, 2017 and amended on April 4th, 2021.
**Funding for this study:** No funding was received for this study.
**Author Disclosures:**
Anna Colarieti: Nothing to disclose
Francesco Sardanelli: Grant Recipient: General Electric, Bayer, and Bracco
Veronica Magni: Nothing to disclose
Adrienn Benedek: Nothing to disclose

**RPS 102-9**

**Quantitative radiomic analysis in contrast-enhanced mammography for breast lesions characterisation**

*G. Della Pepa*[1], C. Depretto[1], W. Carli[1], G. Irmici[1], E. D'Ascoli[1], C. De Berardinis[1], C. Cazzella[2], D. Ballerini[1], G. P. Scaperrotta[1]; [1]Milan/IT, [2]Bergamo/IT
*(gianmarco.dellapepa@gmail.com)*

**Purpose:** This study aimed to investigate the potential of radiomic quantitative texture analysis for characterising breast lesions on contrast-enhanced mammography (CEM) and correlate them with their biological phenotypes.
**Methods or Backround:** Patients who underwent CEM procedures at our institution since 2018 were considered. Among them, all CEM-detected malignant lesions, confirmed via core needle biopsy and surgical intervention, were included in our study. These lesions were firstly subjected to a semi-automatic segmentation, and then 93 radiomic features were extracted for each of them, using the open-source Python package Py-Radiomics. The association between each feature and the predetermined endpoints was evaluated through univariate logistic regression analysis. The correlation was performed either with the singular molecular characteristics: the presence of estrogen (ER) and progesterone (PR) receptors, HER2 status, Ki67 level either with the specific immunophenotype: Luminal A, Luminal B, HER2+ and Triple Negative (TN).
**Results or Findings:** In our preliminary results, 86 patients were selected, with a total of 89 breast lesions analysed. The logistic regression isolated a subset of radiomic features correlating robustly with the biological phenotype. Second-order statistics textural features of Neighbouring Gray Tone Difference Matrix (NGTDM) demonstrated a stronger correlation with the presence of both ER and PR receptors, and multiple combinations of them resulted in a better correlation with Luminal A and Luminal B immunophenotype. The Gray Level Run Length Matrix GLSZM contrast and first-order uniformity both correlate with the TN immunophenotype.
**Conclusion:** Radiomic quantitative texture analysis of breast lesions on CEM demonstrates promising capability in characterising biological phenotype. Looking forward, it could lead to the construction of a nomogram to be used in clinical practice, potentially helping decision-making processes before biopsy.
**Limitations:** The study is constrained by a limited sample size and by the lack of a distinct validation cohort.
**Ethics committee approval:** No information provided by the submitter.
**Funding for this study:** No funding was received for this study.
**Author Disclosures:**
Gianmarco Della Pepa: Nothing to disclose
Elisa D'Ascoli: Nothing to disclose
Walter Carli: Nothing to disclose
Claudia De Berardinis: Nothing to disclose
Daniela Ballerini: Nothing to disclose
Gianfranco Paride Scaperrotta: Nothing to disclose
Catherine Depretto: Nothing to disclose
Caterina Cazzella: Nothing to disclose
Giovanni Irmici: Nothing to disclose

**08:00-09:00** **Research Stage 3**

### Research Presentation Session: Artificial Intelligence & Machine Learning & Imaging Informatics

### RPS 105

### Automatic segmentation techniques

**Moderator**
L. Caldeira; Cologne/DE
*(liliana.caldeira@uk-koeln.de)*

**RPS 105-2**

**Deep learning for segmentation and classification of cardiac implantable electronic devices on chest x-rays**

*F. Busch*[1], A. Zhukov[1], P. Suwalski[1], S. Niehues[1], D. Poddubnyy[1], M. Makowski[2], K. K. Bressem[1], L. C. Adams[2]; [1]Berlin/DE, [2]Munich/DE
*(felix.busch@charite.de)*

**Purpose:** The accurate classification of cardiac implantable electronic devices (CIEDs) on chest x-rays is crucial for effective patient care. The aim of this study was to create an open-access deep learning algorithm capable of both segmenting and classifying CIEDs on DICOM as well as smartphone-acquired images for bedside use.
**Methods or Backround:** This retrospective study included patients with implantable pacemakers, cardioverter defibrillators, cardiac resynchronisation therapy devices, and cardiac monitors who had undergone anterior-posterior or posterior-anterior chest radiography from January 2012 to January 2022 at Charité – University Medicine Berlin. Utilising a U-Net architecture with a ResNet-50 backbone, we developed a model to segment and classify CIEDs based on their manufacturer and model, using both DICOM and smartphone images. Performance metrics included the Dice coefficient for the segmentation model on the validation set (70-30 training/validation set split) and balanced accuracy for manufacturer and model classification on the test set (70-20-10 training/validation/test set split).
**Results or Findings:** The study encompassed 897 patients with 2,322 unique chest radiographs featuring 25 CIED models from six manufacturers. To prevent misclassification of models less represented or not included in the training data, an "other" category was implemented. Additionally, 11,072 images were captured using five different smartphones. The segmentation algorithm attained an average Dice coefficient of 0.936 (interquartile range: 0.068), while the classification model achieved an overall accuracy of 0.927 (95% confidence interval (CI): 0.890-0.965) for manufacturer and 0.847 (95% CI: 0.799-0.888) for model classification.
**Conclusion:** We present a publicly accessible deep learning framework for the high-accuracy segmentation and classification of CIEDs on chest x-rays. Notably, this research introduces the first classification algorithm specifically designed for accurate CIED model identification based on both DICOM and smartphone images.
**Limitations:** The retrospective design of the study and the unequal representation of CIEDs were identified as limitations.
**Ethics committee approval:** This study was approved by an ethics committe; IRB-approval number: EA4/042/20.
**Funding for this study:** No funding was received for this study.
**Author Disclosures:**
Keno K. Bressem: Nothing to disclose
Denis Poddubnyy: Nothing to disclose
Lisa C. Adams: Nothing to disclose
Stefan Niehues: Nothing to disclose
Felix Busch: Nothing to disclose
Phillip Suwalski: Nothing to disclose
Marcus Makowski: Nothing to disclose
Andrei Zhukov: Nothing to disclose

**RPS 105-3**

**Advancing total tumour volume estimation in colorectal liver metastases: development and evaluation of a self-learning auto-segmentation model**

J. I. Bereska[1], M. Zeeuw[1], L. Wagenaar[1], M. G. Besselink[1], H. Marquering[1], J. Stoker[1], Å. Fretland[2], G. Kazemier[1], *I. M. Verpalen*[1]; [1]Amsterdam/NL, [2]Oslo/NO

**Purpose:** Total tumour volume (TTV) assessments have been shown to be prognostic of overall and recurrence-free survival in patients with colorectal cancer liver metastases (CRLM). However, the labor-intensive nature of these assessments has hampered their clinical adoption. This study aimed to develop an auto-segmentation model for CRLM on contrast-enhanced portal venous phase CT scans to facilitate the clinical adoption of TTV assessments.
**Methods or Backround:** We developed a self-learning-based segmentation model to segment CRLM using 760 portal venous phase CTs (CT-PVP) of 363 patients with 13,739 CRLM from the Amsterdam University Medical Centre. We used a self-learning setup in which we first trained a teacher model on 99 manually segmented CT-PVPs segmented by three radiologists and combined using the Simultaneous Truth and Performance Level Estimation (STAPLE) algorithm. The teacher model was then used to segment the remaining 661 CT-PVPs for training the student model. We used Intraclass Correlation Coefficient (ICC) to compare the TTV obtained from the student model's segmentations against that obtained from the STAPLE-combined radiologist's segmentations.
**Results or Findings:** We evaluated the student model in an external test set of 50 CT-PVPs from 35 patients with 72 CRLM from the Oslo University hospital. The student model reached a DICE similarity score of 0.83 for segmenting CRLM. There was no significant difference between the student model's DICE scores and interrater DICE scores. The ICC between the student model's and the STAPLE-combined TTV was 0.97, signifying near perfect agreement.
**Conclusion:** Segmentation models can provide accurate and efficient assessments of TTV in CRLM patients.
**Limitations:** Our study's limitations include its retrospective design, lack of global data, and an external test cohort that differs from the training set, underlining the need for prospective, internationally diverse studies for more robust validation.
**Ethics committee approval:** The Medical Ethics Review Committee of the Amsterdam UMC, the Regional Ethical Committee of South Eastern Norway, and the Data Protection Officer of Oslo University Hospital approved this study protocol.

**Funding for this study:** This study was funded by the KWF (project number 14002).
**Author Disclosures:**
Jaap Stoker: Nothing to disclose
Åsmund Fretland: Nothing to disclose
Luuk Wagenaar: Nothing to disclose
Geert Kazemier: Nothing to disclose
Inez Margaretha Verpalen: Nothing to disclose
Henk Marquering: Founder: Trianect, Nicolab
Michiel Zeeuw: Nothing to disclose
Marc G. Besselink: Nothing to disclose
Jacqueline Isabel Bereska: Nothing to disclose

### RPS 105-4

**A deep learning-based pipeline for cervical spinal cord segmentation and labelling on heterogeneous T1w brain images**

*R. Magalhaes*, A. Liseune, S. van Eyndhoven, T. Billiet, N. Barros, D. Smeets, D. M. Sima; Leuven/BE
*(ricardo.lazarus@gmail.com)*

**Purpose:** The aim of this study was to develop a robust and automated deep learning-based method for cervical spinal cord measurements on T1w brain images.
**Methods or Backround:** Measuring spinal cord (SC) cross-sectional area (CSA) is valuable for monitoring multiple sclerosis (MS), but challenging in daily clinical practice. We propose a fully automated processing pipeline that performs this measurement in mere minutes and is robust for a wide range of imaging protocols. The pipeline was developed using T1w brain scans from MS patients, with ground truth masks generated using an in-house semi-automated pipeline that ensures SC coverage and segmentation quality (187 training, 44 validation). Starting from an input T1w image, the pipeline uses icobrain to perform neck cropping and subsequently applies three cascaded U-net deep learning models that respectively segment, smooth and label the spinal cord, from which the measurements are derived. We report performance on an independent data set containing 10 MS subjects with 53 scans from different scanners.
**Results or Findings:** Dice scores for the segmentation of the full SC and for labelling vertebrae C1 to C4 were 0.89 and 0.85, 0.87, 0.85 and 0.83, respectively. Intra-scanner measurement reproducibility on the test set was high, with an average relative CSA error of 1.5% (intrascanner) and 4.6% (interscanner).
**Conclusion:** Trained on a heterogeneous set of T1w brain scans, the pipeline enables reliable and accurate quantification of cervical SC using standard brain scans, extending icobrain software's capabilities.
**Limitations:** The method requires brain images covering at least a portion of the cervical spine.
**Ethics committee approval:** The test data set was acquired from 10 MS patients who participated in a study at the University Hospital Brussels, Belgium. The study was approved by the local ethics committee and all patients signed informed consent forms. Development data comes from subjects for which icometrix analysed MR scans as part of clinical practice who had agreed to allow icometrix to use an anonymised version of the already analysed MR images for post-market research purposes. Icometrix processes personal data received from the hospitals in conformity with the applicable data protection and privacy legislation.
**Funding for this study:** This study is partly funded by Flanders Innovation & Entrepreneurship (VLAIO) project HeKDiscoMS (HBC.2021.0500) and by the CLAIMS project, supported by the Innovative Health Initiative Joint Undertaking (JU) under grant agreement No 101112153. The JU is supported by the European Union's Horizon Europe research and innovation programme and COCIR, EFPIA, EuropaBio, MedTech Europe, Vaccines Europe, AB Science SA and icometrix NV.
**Author Disclosures:**
Nuno Barros: Employee: icometrix
Diana M. Sima: Employee: icometrix
Simon van Eyndhoven: Employee: icometrix
Arno Liseune: Employee: icometrix
Thibo Billiet: Employee: icometrix
Dirk Smeets: Employee: icometrix
Ricardo Magalhaes: Employee: icometrix

### RPS 105-5

**Multi-organ CT-based automatic segmentation via semi-supervised learning**

*A. Vergara*, A. Jimenez-Pastor, C. Kerckhaert, A. Alberich-Bayarri; Valencia/ES
*(alejandrovergara@quibim.com)*

**Purpose:** To overcome the scarcity of manually annotated data sets for multi-organ segmentation, we propose a novel approach that combines fragmented data sets to train a single model capable of performing multiorgan segmentation.
**Methods or Backround:** Four distinct data sets were collected: AbdomenCT1k (liver, kidneys, pancreas, spleen), CT-org (lungs, bladder), VerSe (spine), and CTPelvic1K (hips, sacrum), collecting a total of 1543 cases. Each data set was used to train a 2D U-Net with deep supervision. These submodels generated pseudo-labels for data sources that were not included in their training, resulting in a combination of both original strong labels and soft labels. Finally, a unified model was trained using all data sets and labels.
**Results or Findings:** The submodels achieved a mean Dice Score Coefficient (DSC) of 0.91. The final model improved the DSC for each structure by a mean of 0.15, attaining a maximum DSC of 0.98 for liver and a minimum of 0.78 for bladder.
**Conclusion:** Our study introduces an innovative method for training a single model using diverse data sources, leveraging a pseudo-label semi-supervised strategy to achieve robust multiorgan segmentation. This approach enables the generation of a larger annotated data set from smaller, specialised ones lacking all desired labels. Additionally, employing a unified model, as opposed to separate models for each data source, offers advantages in terms of reduced inference time and resource efficiency.
**Limitations:** The accuracy of the pseudo-labels used for training the final model is crucial. Gross errors or inaccuracies in these labels could propagate through the subsequent stages of training, affecting the final model's performance.
**Ethics committee approval:** No information provided by the submitter.
**Funding for this study:** The IMAS project (High Sensitivity and Low Dose Molecular Imaging) was funded by the Spanish Ministry of Science and Innovation and European Funds.
**Author Disclosures:**
Alejandro Vergara: Nothing to disclose
Camiel Kerckhaert: Nothing to disclose
Ana Jimenez-Pastor: Nothing to disclose
Angel Alberich-Bayarri: Nothing to disclose

### RPS 105-6

**ACMA-net and unscented Kalman filter-based accurate coronary artery segmentation: an application of deep learning to computed tomography angiography image**

*B. Li*, C. Wen, H. Sun, W. Wang, J. Liu, Y. Liu; Beijing/CN

**Purpose:** Accurate coronary computed tomography angiography (CCTA) image segmentation is a prerequisite for high-precision reconstruction of three-dimensional (3D) coronary artery models, which can visually demonstrate stenotic lesion information and develop treatment plans. However, due to the complex structure and small size of coronary arteries, and the interference in image acquisition, the 3D models reconstructed by existing segmentation technology present insufficient precision. This results in the 3D coronary artery models being noisy and prone to disconnection. To overcome the challenges in accurate segmentation of coronary arteries, this study proposes a deep learning-based two-stage algorithm.
**Methods or Backround:** In the first stage, we added an atrous convolution feature fusion module (ACFFM) and a multiaxis attention module (MAM) to 3D U-Net, called ACMA-Net, to enhance the feature expression ability of the network and effect the preliminary segmentation of coronary arteries. CCTA images of 323 patients were clinically collected to train the network. In the second stage, the preliminary segmentation results were skeletonised and endpoint detection was performed. The regions of coronary artery disconnection were determined by finding minimum distance between the main trunk branch and the endpoint of each disconnected branch. The disconnected skeleton was repaired after reconnection by the unscented Kalman filter (UKF) algorithm.
**Results or Findings:** We evaluated the proposed method on the constructed test set of 50 patients, and the Dice and Jaccard scores were 0.940 and 0.888, respectively, outperforming existing deep learning methods.
**Conclusion:** This study proposed a coronary segmentation method that effectively reduces the phenomenon of coronary disconnection and improves the accuracy and continuity of coronary segmentation using a small-size data set. This provides excellent technical support for the patient-specific 3D demonstration of coronary arteries.
**Limitations:** More CTA images will be clinically collected to further validate the segmentation method.
**Ethics committee approval:** The Ethics Committee of Peking University People's Hospital approved this study.
**Funding for this study:** Funding was provided by the National Key Research and Development Program of China (Grant No. 2021YFA1000201), and the National Natural Science Foundation of China (Grant No. 12202022, 11832003, 32271361, 12102014).
**Author Disclosures:**
Jian Liu: Nothing to disclose
Bao Li: Nothing to disclose
Hao Sun: Nothing to disclose
Chuanqi Wen: Nothing to disclose
Youjun Liu: Nothing to disclose
Wenxin Wang: Nothing to disclose

**RPS 105-7**

**Deep learning-based bowel automatic segmentation and visualisation of Crohn's disease using multilabelled continuous MRE images**

*L. Huang*[1], Z. Zhong[2], B. Huang[2], S-T. Feng[1], X. Li[1]; [1]Guangzhou/CN, [2]Shenzhen/CN
*(guitar_sun@163.com)*

**Purpose:** Recognition of bowel segments from magnetic resonance enterography (MRE) images is quite challenging and time-consuming due to unclear boundary, shape, size, and appearance variations. We established a publicly available whole bowel segments MR data set with benchmark results and visualisation.
**Methods or Backround:** We retrospectively collected T2-weighted coronal MRE data from 70 patients with Crohn's disease (CD). The bowel images per patient were divided into ten segments (stomach, duodenum, small intestine, appendix, caecum, ascending colon, transverse colon, descending colon, sigmoid colon, and rectum), with fine pixel level annotations labelled by two experienced radiologists. Then, nnU-Net model, a deep learning-based segmentation method that automatically configures all hyperparameters based on the data set characteristics, was employed on this data set (training set, n=56; test set, n=14). To reinforce the mutually exclusive relationship between tags, a topological interaction loss function was utilised. The segmentation algorithm was assessed using the dice similarity coefficient (DSC).
**Results or Findings:** Evaluating the performance of bowel segmentation, the mean DSC in the test set was 0.778. Our nnU-Net method in segmenting digestive tract can achieve DSC of 0.963 ± 0.042 in stomach, 0.886 ± 0.049 in duodenum, 0.936 ± 0.024 in small intestine, 0.378 ± 0.441 in appendix, 0.598 ± 0.294 in cecum, 0.825 ± 0.131 in ascending colon, 0.819 ± 0.231 in transverse colon, 0.819 ± 0.186 in descending colon, 0.801 ± 0.220 in sigmoid colon and 0.859 ± 0.130 in rectum, respectively. Segmentation results with predicted bowel boundary can be shown by two- or three-dimensional visual representation.
**Conclusion:** We presented a new data set containing labels for all digestive tract segments on MRE images. Accurate deep learning-based bowel automatic segmentation and visualisation of CD can facilitate the application of artificial intelligence in CD.
**Limitations:** No limitations were identified.
**Ethics committee approval:** No information provided by the submitter.
**Funding for this study:** No funding was received for this study.
**Author Disclosures:**
Shi-Ting Feng: Nothing to disclose
Li Huang: Nothing to disclose
Bingsheng Huang: Nothing to disclose
Xuehua Li: Nothing to disclose
Zhangnan Zhong: Nothing to disclose

**RPS 105-8**

**Replicability of outcome prediction across IPF patient cohorts based on machine learning features learned without supervision**

*J. Pan*, J. Hofmanninger, K-H. Nenning, F. Prayer, S. Röhrich, N. Sverzellati, V. Poletti, H. Prosch, G. Langs; Vienna/AT
*(jeanny.pan@meduniwien.ac.at)*

**Purpose:** Idiopathic pulmonary fibrosis (IPF) is most common interstitial lung disease. This study validated the transferability of an outcome prediction method based on previously identified disease patterns on a new cohort.
**Methods or Backround:** Four lung CT patterns associated with disease progression had been previously identified from 74 IPF patients using unsupervised machine learning. We studied a different multicentre cohort (various manufacturers, slice thickness and reconstruction kernels) of 164 patients to investigate the transferability of the progression patterns. We tested outcome prediction based on patterns in a single CT scan, and based on additional pattern changes in subsequent scan pairs. In both experiments, patients were clustered based on similarities in their progression pattern profiles, and Kaplan-Meier survival curves were analysed for each cluster to test if the outcome was significantly different.
**Results or Findings:** Of the 164 patients, 59 died and 16 had transplants before the censoring date, with an average time of 211.73 weeks from the baseline scan, while for the remaining 89, it was 197.34 weeks. Predicting survival outcomes with a single scan profile yields a hazard ratio (HR) of 5.39 ($p<0.01$). Consistent with the results on the initial cohort, incorporating the change of pattern profiles between two scans further improved the prediction, yielding an HR of 6.03 ($p <0.01$).
**Conclusion:** The replication of outcome prediction with previously identified progression markers in a new cohort of IPF patients demonstrated significant predictive value for outcome. The dynamic changes in marker profiles between scans enhanced the hazard ratio. This underscores the potential of quantitative marker profiles in disease monitoring for IPF patients. Future studies may explore the broader applicability of the method to other interstitial lung diseases.
**Limitations:** We did not perform specific analysis to differentiate the impact of centres and manufacturers.
**Ethics committee approval:** No information was provided by the submitter.
**Funding for this study:** Funding for this project was received via the FWF, ONSET Project P35189.
**Author Disclosures:**
Karl-Heinz Nenning: Nothing to disclose
Georg Langs: Nothing to disclose
Helmut Prosch: Nothing to disclose
Florian Prayer: Nothing to disclose
Sebastian Röhrich: Nothing to disclose
Nicola Sverzellati: Nothing to disclose
Johannes Hofmanninger: Nothing to disclose
Jeanny Pan: Nothing to disclose
Venerino Poletti: Nothing to disclose

**09:30-11:00** **Research Stage 1**

### Research Presentation Session: Musculoskeletal

### RPS 210

### Exploring knee imaging

**Moderator**
R. Sutter; Zurich/CH
**Author Disclosures:**
Reto Sutter: Author: Reto Sutter is book editor for Breitenseher publisher; Other: Balgrist University Hospital has an academic resarch agreement with Siemens Healthineers, Bayer, and Balzano Informatik.

**RPS 210-2**

**Assessing lateral posterior meniscal root insertion variations of the knee on a clinical 7 Tesla MRI in an asymptomatic cohort**

*R. P. Marcus*, A. A. Marth, S. Zimmermann, R. Sutter; Zurich/CH
*(roy.marcus@outlook.com)*

**Purpose:** The aim of this study was to assess variations of posterolateral meniscal root insertions using a high-resolution sequence on a clinical 7 Tesla (T) MRI scanner.
**Methods or Backround:** Fifty-eight knees of healthy subjects were examined on a clinical 7 T MRI scanner (Magnetom Terra, Siemens Healthineers). Multiplanar reconstructions of a sagittal isotropic 0.24 mm double echo steady state 3D-sequence were used. The number of root insertion reins and insertion localisations was recorded for the lateral meniscus by two radiologists.
**Results or Findings:** The majority of subjects presented with double root insertion reins (66.7%), followed by single (31.6%) and triple insertion reins (1.8%). The majority of single posterior lateral rein roots inserted into the intercondylar area (77.8%) and the remaining 22.2% inserted into the posterior slope of the posteromedial tubercle of the tibia. The majority of double reins inserted with a major rein along the intertubercular area to the posteromedial insertion of the ACL and a minor rein to the lateral tubercle (78.9%). The inverted variant (major rein inserts into the lateral tubercle and the minor rein inserts next to the tibial ACL-insertion) was visible in 5.3%. The remaining double reins inserted into the posterolateral slope of the medial and lateral eminence. Interrater reliability for describing the meniscal root reins was perfect ($\kappa = 1$) and identifying the insertion sites was strong ($\kappa = 0.808$).
**Conclusion:** Posterolateral meniscal roots consisted mostly of double root insertion reins, followed by single and triple reins. Five insertion locations were detected, with the majority inserting with a major rein next to the posteromedial insertion of the ACL and a minor rein to the lateral tubercle of the tibia.
**Limitations:** Small sample size.
**Ethics committee approval:** This study was approved by the Local Ethics committee of the Canton of Zurich.
**Funding for this study:** This was an in-house study and no funding was received for it.
**Author Disclosures:**
Stefan Zimmermann: Nothing to disclose
Adrian Alexander Marth: Nothing to disclose
Reto Sutter: Nothing to disclose
Roy P. Marcus: Nothing to disclose

### RPS 210-3

**Stability of deep learning-based image quality improvement in MRI of the knee: correlation with arthroscopy**

*S. Park*, S. H. Choi, H. Lee, S. Kim, J-Y. Jung; Seoul/KR
*(unluckyskeleton@naver.com)*

**Purpose:** The study aimed to validate whether deep learning (DL)-based image-quality improvement changes the diagnostic performance and visibility of core features for meniscal, cartilage, and ligament knee lesions.
**Methods or Backround:** This retrospective study include 106 patients, who underwent knee MRI and arthroscopy within a three-month interval. Fat-suppressed 2D fast spin-echo (FSE) were processed with pre-trained DL. Two musculoskeletal radiologists independently reviewed both the original and DL-processed MRI for medial meniscus (MM), lateral meniscus (LM), articular cartilage (AC) and cruciate ligaments (CL). Sensitivity and specificity were compared with arthroscopic results used as the reference standard. Additionally, two radiologists compared the target lesions between arthroscopic images and original or DL-processed MRI with different denoising levels using pe-defined similarity score.
**Results or Findings:** In reader 1, the sensitivity and specificity (%) of original vs. DL-IQI-processed MRI were 91.0 vs. 92.5 (P>0.99) and 92.3 vs. 97.4 (P=0.50) for MM, 88.9 vs. 93.3 (P=0.50) and 90.1 vs. 91.8 (P>0.99) for LM, 87.2 vs. 94.8 (*P<0.05) and 92.8 vs. 85.7 (P=0.625) for AC, and 91.3 vs. 91.3 (Non-Applicable, NA) and 98.8 vs. 98.8 (NA) for CL. In reader 2, the sensitivity and specificity of original vs. DL-IQI-processed MRI were 86.6 vs. 91.4 (P=0.25) and 97.4 vs. 97.4 (NA) for MM, 86.7 vs. 86.7 (P>0.99) and 88.5 vs. 88.5 (P>0.99) for LM, 80.8 vs. 84.6 (P=0.37) and 96.4 vs. 89.3 (P=0.5) for AC, and 78.3 vs. 78.3 (NA) and 98.8 vs. 98.8 (NA) for CL. The mean agreement scores between arthroscopy and MRI for target lesions were 7.18(O) and 7.33-7.36(DL) for meniscal tears (n=130), and 2.53(O) and 3.23-3.24(DL) for cartilage lesions (n=84).
**Conclusion:** DL-processing of knee MRIs improved the identification of cartilage lesions, without affecting overall diagnostic performances as correlated with arthroscopic results.
**Limitations:** This was a retrospective, single-centre study.
**Ethics committee approval:** This study was approved by the Institutional review board in our hospital: 20230822-F-261.
**Funding for this study:** No funding was obtained for this study.
**Author Disclosures:**
Joon-Yong Jung: Nothing to disclose
Seung Hoon Choi: Nothing to disclose
Heerin Lee: Nothing to disclose
Seokhee Park: Nothing to disclose
Sohyun Kim: Nothing to disclose

### RPS 210-4

**M score as an alternative to T score in the evaluation of femoral osteoporosis?**

*M. B. Gökhan*, N. Çay; Ankara/TR
*(mbgokhan@gmail.com)*

**Purpose:** This study aimed to contribute to the diagnosis of osteoporosis-osteopenia with MRI-based “M-scores” obtained from T1 weighted hip MRI.
**Methods or Backround:** Between February 2019 and May 2023, patients aged 18 years or older, who underwent DXA and also had hip MRI within six months before or after DXA, were included. A reference group consisting of 190 females with a normal BMI who had hip MRI and aged between 20 and 29 years was also included. Images were retrospectively evaluated by two radiologists, blinded to the DXA scores. Signal intensities of locations corresponding to DXA measurement sites, subcutaneous fat intensities, muscle intensities, and noise from artifact-free areas, were measured. Signal-to-noise(SNR), signal-to-fat (SFR) and signal-to-muscle ratios(SMR) were calculated. M scores for SNR, SMR and SFR were calculated based on the measurement values of the reference group, similar to the T score. Correlation and ROC analysis were performed for all M-scores.
**Results or Findings:** Included 330 patients (mean age 57.61); 30(%9.1) osteoporotic for hip at DXA. There were a strong negative correlation between M-scores and T-scores (SNR-M-r=0.722, SFR-M-r=0.750, SMR-M-r=0.662) for neck and moderate negative correlation for total hip (SNR-M-r=0.599, SFR-M-r=0.625, SMR-M-r=0.568). Cut-off points for femoral neck osteoporosis detection were determined as follows: SNR-M-score at 2.23 (sensitivity 93%, specificity 71%), SFR-M-score at 3.052 (sensitivity 84%, specificity 85%), and SMR-score at 1.538 (sensitivity 100%, specificity 78%). And cut-off points for hip were determined as following: SNR-M-score at 1.22 (sensitivity 85%, specificity 68%), SFR-M-score at 1.313 (sensitivity 75%, specificity 82%), and SMR-M-score at 1.576 (sensitivity 67%, specificity 83%).
**Conclusion:** Our study suggests the potential of MRI-based M scores for diagnosing hip osteoporosis, but further validation on a larger population is needed.
**Limitations:** The limitations of the study is sample size.
**Ethics committee approval:** The study was approved by Ankara Bilkent City Hospital Ethics Committee: E2-23-4074.
**Funding for this study:** No funding was received for this study.
**Author Disclosures:**
Muhammet Batuhan Gökhan: Nothing to disclose
Nurdan Çay: Nothing to disclose

### RPS 210-5

**Extension and costs of low-value MRI of the knee in Norway**

*B. M. Hofmann*[1], V. Håvik[2], I. Ø. Brandsæter[1], E. R. Andersen[1], E. Kjelle[1]; [1]Gjøvik/NO, [2]Oslo/NO
*(bjoern.hofmann@ntnu.no)*

**Purpose:** As low-value imaging is a challenge for the quality of care and patient safety, we need more evidence about the extension and costs of low-value imaging. Accordingly, the purpose of this study is to provide knowledge about the extension of knee MRIs that are of low value and to estimate their opportunity costs.
**Methods or Backround:** Contingent extraction of imaging and diagnostic data from the control and reimbursement of healthcare claims (Helfo) registry in Norway in 2021 analysed with descriptive statistics.
**Results or Findings:** Between 60 and 80 per cent of knee MRIs in Norway could be of low value, as they yield no relevant diagnoses or actions prior to or after the MRI examination. This corresponds to between 24,000 and 35,000 MRIs, at a cost of USD 8 to 12 million annually. Fewer than 7% of the patients had a relevant diagnosis code or a prior imaging examination when having the MRI, and less than 15% got a knee-related diagnosis code from specialist care within six months after the MRI.
**Conclusion:** Most MRIs of the knee in Norway could be of low value to the patient. Reducing low-value MRIs can free resources for high-value imaging, reduce wait times, increase the quality of care, in addition to improve patient safety, healthcare efficiency, and professional integrity.
**Limitations:** Quality of registry data, inclusiveness of disease and treatment codes, investigative time span.
**Ethics committee approval:** This is a register study where no patient data are used outside data provider organization and so no ethical approval was sought for this study.
**Funding for this study:** This study was funded by the Research Council of Norway: Project number 302503.
**Author Disclosures:**
Vegard Håvik: Nothing to disclose
Ingrid Øfsti Brandsæter: Nothing to disclose
Bjørn Morten Hofmann: Nothing to disclose
Elin Kjelle: Nothing to disclose
Eivind R. Andersen: Nothing to disclose

### RPS 210-6

**Investigation of the anatomic risk factors in acute anterior cruciate ligament rupture coexisted with ramp lesions of the medial meniscus by quantitative MRI**

*Z. Tang*, Y. Luo, D. Liu, S. Zhou, H. Yang; Chongqing, China/CN
*(tangziyi98@126.com)*

**Purpose:** This study aimed to investigate the predictive anatomic risk factors of knees in patients with acute non-contact anterior cruciate ligament (aACL) rupture that coexisted with ramp lesions of medial meniscus.
**Methods or Backround:** A total of 202 subjects were retrospectively divided into three groups: (1) an aACL rupture with ramp lesions group (n=76); (2) an isolated ACL rupture group (n=56) and (3) a normal control group (n=70). Quantitative morphological parameters on MRI were measured: the diameter of medial femoral condyle (MFC), the anterior-posterior length and depth of medial tibial plateau (MTP AP length and MTP depth), the lateral posterior tibial slope (LPTS) and medial posterior tibial slope (MTPS), the asymmetry of LPTS and MPTS (LMPTS), the lateral meniscal slope (LMS), and medial meniscal slope (MMS).
**Results or Findings:** The MTP AP length, MTP AP length/MFC diameter ratio, MTP depth, LPTS and the asymmetry of LMPTS showed significant differences among the three groups (p<0.001). The risk factors associated with the ramp lesions including a longer MTP AP length (OR 1.17, 95% CI 1.00-1.44, p=0.044), deeper MTP depth (OR 1.91, 95% CI 1.22-3.00, p=0.005) and larger MTP AP length/MFC diameter ratio (OR 1.11, 95% CI 1.01-1.22, p = 0.036). The highest AUC was the MTP AP length/MFC diameter ratio (0.74; 95% CI, 0.66-0.82), with a sensitivity of 73% and specificity of 68% to predict ramp lesions.
**Conclusion:** Several occult anatomic characteristics of the knee, especially the morphology of the medial tibia plateau, can be additional risk factors for aACL rupture that coexisted with ramp lesions.
**Limitations:** Not all cases were diagnosed with ramp lesions under arthroscopy. However, the ramp lesions exhibited the typical MRI characteristics with specificity as high as 98% in previous studies.

**Ethics committee approval:** The study was approved by The First Affiliated Hospital of Chongqing Medical University (No.2021-203).
**Funding for this study:** No funding was received for this study.
**Author Disclosures:**
Suying Zhou: Nothing to disclose
Yuxi Luo: Nothing to disclose
Ziyi Tang: Nothing to disclose
Dan Liu: Nothing to disclose
Haitao Yang: Nothing to disclose

### RPS 210-7

**Leveraging subtraction STIR-T1 MAVRIC sequences for precise discrimination of artefacts vs peri-implant infections in total knee replacement prostheses**

*S. Rajan*, V. K. Venugopal, H. Mahajan; New Delhi/IN
*(drsriramrajan@gmail.com)*

**Purpose:** This study aimed to explore the utility of a new subtraction technique which involved subtracting STIR from T1 images to unveil the persisting infective signals on STIR images while reducing the T1 artefacts.
**Methods or Backround:** Our study encompassed 80 patients suspected of peri-implant infections surrounding total knee replacement prostheses, all of whom underwent MRI scans with the standard T1 and STIR MAVRIC protocol. Subsequently, the anonymised and randomised images were evaluated by two radiologists, possessing 8 and 21 years of experience, who arrived at a consensus regarding the presence or absence of peri-implant infections or marked them as indeterminate cases. Further, subtraction images, derived from STIR minus T1 weighted images, were meticulously reconstructed and colour-coded (hot-iron) before being fused with conventional T1 images. These fused images underwent a second evaluation by consensus to ascertain the presence or absence of peri-implant infections or indeterminate cases.
**Results or Findings:** The initial assessment revealed 38 cases detected with infection, 25 cases absent of infection, and 17 cases marked as indeterminate. However, upon implementing the subtraction technique, the numbers shifted to 34 cases with infection, 32 cases without infection, and 14 indeterminate cases. To ascertain the statistical significance of these results, a chi-square goodness-of-fit test was conducted, revealing a Chi-square value of 18.66. This value signifies a significant difference between the observed and expected frequencies, further affirming the utility of the subtraction technique.
**Conclusion:** Our study underscores the substantial advantages of employing subtraction STIR-T1 MAVRIC sequences in distinguishing between infections and artefacts, outperforming conventional sequences.
**Limitations:** This study was limited by the small size of the dataset
**Ethics committee approval:** This study was IRB approved.
**Funding for this study:** No funding was received for this study.
**Author Disclosures:**
Sriram Rajan: Nothing to disclose
Harsh Mahajan: Nothing to disclose
Vasantha Kumar Venugopal: Consultant: Carpl.ai

### RPS 210-8

**Deep learning-based MRI quantification of the thigh muscles in sarcopenia**

*Z. Zhang*[1], W. Yu[1], F. Santini[2], J. Zhu[1], F. Wang[1]; [1]Beijing/CN, [2]Basel/CH
*(zaizhuzhang@163.com)*

**Purpose:** This study aimed to explore whether MRI markers of cross-sectional volume (CSV), fat fraction (FF) and T2 values are affected in sarcopenia and to examine associations between MRI markers and components of sarcopenia (muscle mass, handgrip strength, and gait speed) in Chinese subjects.
**Methods or Backround:** Postmenopausal women were prospectively recruited for this cross-sectional study. Muscle mass was measured by dual-energy X-ray absorptiometry (DXA). Jamar hydraulic hand dynamometer was used to assess muscle strength and gait speed was used to assess physical performance. A 3.0T MRI machine was used to scan bilateral thigh muscles. A DL-based automated segmentation software (Deep Anatomical Federated Network, DAFNE) was used for the segmentation of anatomical images. MRI measures were compared between sarcopenic and non-sarcopenic groups and correlations with other variables were assessed. Their optimal cut-off points (Youden index) for sarcopenia diagnosis were identified in relation to the AWGS2 criteria.
**Results or Findings:** 68 participants (mean age, 56.6 years ± 5.7 [SD]) were studied, and 25 (36.8%) were diagnosed as sarcopenic. The sarcopenic group had lower CSV (737.8 cm3 vs 862.2 cm3; P<0.001) and elevated T2 values (45.8 ms vs 44.4 mol/L; P=0.018) than the non-sarcopenic group, although there was no evidence of a difference in FF. CSV was positively associated with DXA-derived muscle mass and handgrip strength (P<0.001), whereas T2 values were inversely associated with gait speed (P<0.05). The CSV and T2 value optimal cut-off points in discriminating sarcopenic patients were identified at 798.7 cm3 (AUC-ROC = 0.883) and 44.8 ms (AUC-ROC = 0.666) respectively.
**Conclusion:** CSV and T2 values can differentiate sarcopenic versus non-sarcopenic subjects, representing an imaging biomarker of sarcopenia.
**Limitations:** This preliminary study was limited in size, and therefore could not account for all confounders in the sarcopenic group.
**Ethics committee approval:** This study was approved by the institutional review board of the Peking Union Medical College Hospital, Beijing, China.
**Funding for this study:** No funding was received for this study.
**Author Disclosures:**
Francesco Santini: Nothing to disclose
Jinxia Zhu: Employee: at Siemens Healthineers
Fengdan Wang: Nothing to disclose
Zaizhu Zhang: Nothing to disclose
Wei Yu: Nothing to disclose

### RPS 210-9

**Five-minute knee MRI: an AI-based super resolution reconstruction approach for compressed sensing. A validation study on healthy volunteers**

*R. A. Terzis*, T. M. Dratsch, R. Hahnfeldt, L. Basten, P. Rauen, K. Sonnabend, D. Maintz, A-I. Iuga, G. Bratke; Cologne/DE

**Purpose:** This study aimed to investigate the feasibility of a complete five-minute 2D knee MRI protocol obtained by combining Compressed Sensing (CS) and a new AI-aided super-resolution reconstruction method.
**Methods or Backround:** 20 volunteers were examined on a 3T-MRI scanner (Ingenia Elition X, Philips) using a 2D knee protocol similar to clinical standards: a sagittal fat-saturated proton-density-, and a T1-weighted-sequence in coronal, sagittal and transversal orientation. Images were acquired in standard and low resolution and reconstructed with a traditional CS and a new, AI-aided technique (CS-SuperRes). Two experienced radiologists assessed subjective image quality using 8 criteria on a 5-point scale, and signal-to-noise ratio was measured as an objective parameter.
**Results or Findings:** CS-SuperRes showed superior ratings over equivalent CS reconstructions, particularly for low-resolution images (overall image quality: 4.3±0.4 vs. 3.4±0.4, p<0.05). For the low-resolution scans, CS-SuperRes reconstructions matched the quality of standard-resolution traditional CS across all metrics. Overall image impression scored 4.3±0.4 with CS-SuperRes vs. 4.0±0.5 with traditional CS (p<0.05). This allowed a significant decrease in scan duration without image quality loss, from 11:01 minutes to just 4:46 minutes (a 57% reduction) for the entire protocol.
**Conclusion:** The AI-aided CS-SuperRes reconstruction method can reduce MRI scan times by 57%, retaining image quality comparable to traditional CS. This allows for a complete 2D knee MRI scan in just 5 minutes with preserved image excellence, facilitating increased scanner throughput and improved patient workflow.
**Limitations:** This was a single-centre study which used a single MRI scanner and a small sample of healthy volunteers. Nevertheless, the multiscale network ensures data consistency and objective findings corroborate subjective interpretations. Moreover, some of the scans showed incidental findings, which were visible with all reconstruction methods.
**Ethics committee approval:** This prospective single-centre study was carried out in accordance with the ethical standards in the 1964 Declaration of Helsinki and its later amendments and was approved by the institutional review board.
**Funding for this study:** This research did not receive any specific grant from funding agencies in the public, commercial, or not-for-profit sectors.
**Author Disclosures:**
Lajos Basten: Nothing to disclose
David Maintz: Speaker: Philips Healthcare
Philip Rauen: Nothing to disclose
Andra-Iza Iuga: Nothing to disclose
Robert Hahnfeldt: Nothing to disclose
Thomas Markus Dratsch: Nothing to disclose
Robert Angelo Terzis: Nothing to disclose
Kristina Sonnabend: Nothing to disclose
Grischa Bratke: Speaker: Philips Healthcare

### RPS 210-10

**dGEMRIC evaluation of autologous conditioned adipose tissue with leukocyte-poor platelet rich plasma for treatment of mild knee osteoarthritis**

*V. Matišić*, V. Molnar, E. Pavelić, P. Brlek, Ž. Jeleč, E. Rod, I. Borić, D. Primorac; Zagreb/HR

**Purpose:** This study was conducted to assess the clinical and radiomorphologic effects of autologous conditioned adipose tissue (ACA) with leukocyte-poor, platelet-rich (LP-PRP) plasma knee injections in treating patients with mild knee osteoarthritis (KOA). The main objective of this study was to compare the results of the treatment to the control group treated with the standard hyaluronic acid injection.
**Methods or Backround:** 16 patients (8 male and 8 female) with KOA were included in this study. Patients received an intraarticular, ultrasound-guided injection of ACA and LP-PRP. Contralateral knees of the same patients were treated with hyaluronic acid (HA) to compare the dGEMRIC indices with knees with KOA. Clinical results and dGEMRIC scores were noted by filling out questionnaires (VAS, WOMAC, KOOS) and imaging at 3-time points occurring prior to intervention and 3 and 6 months after the intervention. Each subject received 0.2 mmol/kg gadolinium diethylene triamine penta-acetic acid for the purpose of dGEMRIC per visit.
**Results or Findings:** Clinical scores demonstrated improvement at both 3 and 6-month follow-ups. However, dGEMRIC indices showed no significant change at both time points. Furthermore, no difference between the affected and the unaffected knees treated with HA was demonstrated.
**Conclusion:** The lack of objective findings after ortho-biologic treatment is a common finding across the published literature. Hence, the major orthopaedic guidelines do not include or advise against the use of these treatment modalities. Further research should standardise the dose of given mesenchymal stem cells and PRP products to try and produce higher-quality evidence for their use and identify the best patients to receive these treatments.
**Limitations:** This study was limited by the small patient size and the undetermined amount of MSCs given to any of the patients.
**Ethics committee approval:** This study was approved by the Ethics Committee of St. Catherine Specialty Hospital (protocol code 21/3-1, 9 March 2021)
**Funding for this study:** No funding was received for this study.
**Author Disclosures:**
Vilim Molnar: Nothing to disclose
Eduard Pavelić: Nothing to disclose
Vid Matišić: Nothing to disclose
Željko Jeleč: Nothing to disclose
Dragan Primorac: Nothing to disclose
Igor Borić: Nothing to disclose
Petar Brlek: Nothing to disclose
Eduard Rod: Nothing to disclose

### RPS 210-11

**Performance of low-field MR for knee cartilage quantification: results on comparison with arthroscopy**

*F. Pucciarelli*, M. Zerunian, M. Polici, B. Masci, A. Del Gaudio, G. Argento, D. De Santis, D. Caruso, A. Laghi; Rome/IT
*(pucciarelli.fra@gmail.com)*

**Purpose:** This study aimed to evaluate the reliability of T2-mapping on knee cartilage on low-field (0.3 T) MRI to detect low-grade chondropathy and to compare it to arthroscopy.
**Methods or Backround:** 55 patients with planned arthroscopic surgery were prospectively enrolled. MRI examination was performed with a low-field scanner (0.3 T) with a dedicated sequence (3D SHARC) for the evaluation of cartilage T2 relaxation time. Image analysis was performed by two radiologists with dedicated experimental software specifically set on MatLab. A colour map, superimposed on the cartilage, consisted of different colours reflecting different T2 values. Each knee was divided into 14 regions, according to the Whole-Organ Magnetic Resonance Imaging Score (WORMS) classification, and a single free-hand region of interest (ROI) was drawn in each region. Arthroscopic knee surgery was performed by an orthopaedic surgeon blinded to MRI data and chondropathy grade was assessed by Outerbridge classification. The T2-relaxation time of each ROI was annotated and compared to arthroscopy results.
**Results or Findings:** A total of 52 participants were included and a total of 728 ROIs were analysed. Comparison of negative patients (grade 0) and positive patients (grade I, II, III and IV) showed significant ($p<0.001$) lower T2-values for grade 0 (42,35±11,67 ms vs 48,36±12,36 ms, respectively). Sub-analysis of different grades showed significant ($p<0.001$) lower T2-values between grade 0 and I (42,35±11,67 ms vs 46,38±9,09 ms, respectively) and grade 0 and II (42,35±11,67 ms vs 47,42±10,51 ms, respectively). No significant difference ($p=0.089$) was found between grades I and II (46,38±9,09 ms vs 47,42±10,51 ms, respectively). No significant difference (all $p>0.05$) was found between the two readers.
**Conclusion:** T2 mapping applied to low-field magnetic resonance allows discrimination between negative and positive patients with chondropathy and allows the detection of patients with low degrees of chondropathy.
**Limitations:** This was a single-centre study with a small sample size.
**Ethics committee approval:** Written informed consent was acquired for all patients and Institutional Review Board approval was obtained.
**Funding for this study:** No funding was received for this study.
**Author Disclosures:**
Damiano Caruso: Nothing to disclose
Benedetta Masci: Nothing to disclose
Francesco Pucciarelli: Nothing to disclose
Marta Zerunian: Nothing to disclose
Domenico De Santis: Nothing to disclose
Andrea Laghi: Nothing to disclose
Antonella Del Gaudio: Nothing to disclose
Michela Polici: Nothing to disclose
Giuseppe Argento: Nothing to disclose

### RPS 210-12

**To evaluate the impact of cartilage damage on knee joint functionality through dynamic CT imaging**

*M. Acke*, T. Scheerlinck, B. Keelson, N. Van Vlasselaer, S. Héréus, G. Van Gompel, E. Cattrysse, N. Buls; Brussels/BE
*(manou.acke@gmail.com)*

**Purpose:** This study aimed to evaluate the impact of cartilage damage on knee joint functionality through dynamic CT imaging.
**Methods or Backround:** In an ex-vivo thiel embalmed leg, cartilage was removed progressively from the patellofemoral joint in three stages: lateral facet, crista and medial facet. After each stage, dynamic CT scans were acquired while inducing a cyclic flexion-extension motion of the leg. Dynamic CT scans were acquired in cine mode with 16 cm z-coverage, 50 cm FOV, 80 kVp, 452 mA, 280 ms rotation time and 6 s total scan duration. Bones were segmented and registered through a multi-atlas approach using SimpleITK-Elastix. Surface proximity maps of the joint were created to visualise the intra-articular joint space. The intra-articular contact area was computed as the area with a distance to the adjacent bone smaller than the cartilage thickness (5,7 mm). Differences in contact areas between normal and damaged stages were evaluated using a Wilcoxon Signed Rank test.
**Results or Findings:** The contact area after maximum intervention increased from 241 $mm^2$ to 613 $mm^2$ for extension, and from 67 $mm^2$ to 480 $mm^2$ for maximum flexion ($p=0.014$). Apart from the first stage, each intervention resulted in an increased intra-articular contact area ($p=0.02 – 0.014$).
**Conclusion:** Dynamic CT scans can indicate an increasing trend of intra-articular contact area with escalating cartilage degeneration.
**Limitations:** The feasibility was shown for a single, ex-vivo sample.
**Ethics committee approval:** Prior to commencing this study, ethical approval (B.U.N. 1432023000100) was obtained from the VUB-UZ Brussels University Hospital's ethical committee after submitting the necessary documentation. The cadaver used in the study was legally donated, and either the subject or their legal next of kin explicitly consented to the use for research purposes.
**Funding for this study:** Funding for this study was provided by the UZBrussels.
**Author Disclosures:**
Manou Acke: Nothing to disclose
Savanah Savanah Héréus: Nothing to disclose
Gert Van Gompel: Nothing to disclose
Nicolas Nicolas Van Vlasselaer: Nothing to disclose
Erik Cattrysse: Nothing to disclose
Nico Buls: Nothing to disclose
Thierry Scheerlinck: Nothing to disclose
Benyameen Keelson: Nothing to disclose

**09:30-11:00** **Research Stage 2**

### Research Presentation Session: Interventional Radiology

### RPS 209

### Advances in liver loco-regional treatment

**Moderator**
N. Villard; Lausanne/CH

### RPS 209-2

**Salvage therapy outcomes of degradable starch microspheres transarterial chemoembolisation (DSM-TACE) in patients with uveal melanoma liver metastases**

*J. M. Ludwig*, H. Steinberg, J. Haubold, L. Umutlu, M. Forsting, J. T. Siveke, B. M. Schaarschmidt, J. Theysohn; Essen/DE
*(ludwigjohannes@hotmail.com)*

**Purpose:** The aim of this study was to assess DSM-TACE as salvage therapy in patients with unresectable uveal melanoma liver metastases and to identify prognostic factors for survival.
**Methods or Backround:** Fifty-five patients (49.1% male, median age 65 yrs) first treated between 08/2016-06/2021 were assessed retrospectively. One hundred and sixty two DSM-TACE (median of 2/patient, range: 1-12) with melphalan (in 98%) were performed. Cox Proportional Hazard Model for uni- (UVA) & multivariate (MVA) analyses (Hazard ratio;95%CI,p-value) and Kaplan-Meier analysis for determining median overall survival and time to progression (OS/TTP in months; 95%CI) were performed. Response assessment was performed according to the RECIST criteria.
**Results or Findings:** The median OS of the study cohort was 7.97 (6.7-9.3) months following the first DSM-TACE. UVA identified low lactate dehydrogenase (LDH <2x the upper level of normal) (0.26;0.12-0.57,p=<0.001), normal serum protein (0.32;0.2-0.7,p=0.008), hepatic tumour burden ≦25% (0.39;0.19-0.7,p=0.007), and monthly tumour growth rate ≦20% before the first DSM-TACE (0.32;0.14-0.7,p=0.005) as predictors of prolonged OS. MVA confirmed low LDH (median OS: 11.4 vs. 4.3 months, p=0.021) and low TGR (median OS: 9.9 vs. 6.4 months, p=0.005) as independent predictors. Patients with low LDH and TGR (40.9%) survived longer, with a median OS of 20.4 (8.4-1.6) months compared to patients with high LDH or high TGR (46%) with 6,4 months (4,5-8,9) or patients with high TGR and high LDH (14%) with 2.4 months (1,1-7,2). Median TTP was four months (3.1-5.7). The best response observed was PR in 14%, SD in 67%, and PD in 19%, with a median OS of 25 (13.5-31.6), 8 (7.1-.8), and 4.4 (2.1-20.4) months, respectively.
**Conclusion:** DSM-TACE is a promising salvage therapy for patients with unresectable uveal melanoma liver that can achieve disease control and prolong survival. Low pretreatment LDH and low prior tumor growth rate are independent predictors of prolonged survival.
**Limitations:** Single-centre, retrospective study is its limitation.
**Ethics committee approval:** This study is a retrospective single-centre database analysis that has been approved by the local institutional review board with waived informed consent (5: 20-9799-BO).
**Funding for this study:** No funding was received for this study.
**Author Disclosures:**
Michael Forsting: Nothing to disclose
Jens Theysohn: Consultant: PharmaCept
Benedikt Michael Schaarschmidt: Nothing to disclose
Johannes Maximilian Ludwig: Nothing to disclose
Hannah Steinberg: Nothing to disclose
Johannes Haubold: Nothing to disclose
Jens T. Siveke: Nothing to disclose
Lale Umutlu: Nothing to disclose

### RPS 209-3

**Percutaneous combined treatment (transarterial chemoembolisation + thermal ablation) in patients with hepatocellular carcinoma enlisted for liver transplant: pathological findings and follow-up**

*F. Piccione*, M. Calandri, A. Doriguzzi Breatta, P. Fonio, M. M. E. Fronda, C. Gazzera, E. Susanna; Turin/IT
*(federica.piccione@unito.it)*

**Purpose:** The study aimed at evaluating the efficacy of a combined thermal ablation (TA) and trans-arterial chemoembolisation (TACE) treatment as a bridge or downstaging method for liver transplantation (LT) in patients with hepatocellular carcinoma (HCC) > 3 cm in size.
**Methods or Backround:** A retrospective review encompassed 700 consecutive patients subjected to OLT for HCC, focusing on 36 patients who underwent combined TA and TACE as bridge or downstaging. Primary objectives included assessing explanted liver pathology, emphasising necrosis of the targeted lesion, post-OLT overall survival (OS) and post-OLT recurrence free survival (RFS). A comparison in terms of post-OLT OS and RFS with 170 patients subjected to TA alone for nodules < 3 cm in size was also performed.
**Results or Findings:** Out of the 36 patients, 63.9% underwent the combined therapy as a bridge, while 36.1% required downstaging to meet the Milan criteria. The average treated node size was 4.25 cm (+/- DS). Half received radiofrequency (RF) treatment and the other half underwent microwave (MW) treatment. All nodes underwent drug-eluting beads (DEB) TACE. The mean necrosis percentage was 65.9% in the RF+TACE group and 83.3% in the MW+TACE group (p-value = 0.099). OS was 100% at 1 year, 100% at 3 years, and 94.7% at 5 years. RFS was 97.2% at 1 year, 94.4% at 3 years, and 90% at 5 years. Despite the different sizes of the lesions, OS and RFS did not show significant differences between the study cohort and the cohort of patients subjected to TA alone.
**Conclusion:** Combined therapy (TA and TACE) is effective for HCC > 3 cm, particularly for bridging and downstaging to OLT, achieving OS and RFS rates exceeding 80% at 1, 3, and 5 years.
**Limitations:** Retrospective single-centre study limits the scope of this study.
**Ethics committee approval:** Waiting for a response.
**Funding for this study:** No funds were received for this study.
**Author Disclosures:**
Andrea Doriguzzi Breatta: Nothing to disclose
Marco Calandri: Nothing to disclose
Carlo Gazzera: Nothing to disclose
Eleonora Susanna: Nothing to disclose
Federica Piccione: Nothing to disclose
Marco Mario Edgardo Fronda: Nothing to disclose
Paolo Fonio: Nothing to disclose

### RPS 209-4

**Two-dimensional perfusion-angiography permits direct visualisation of redistribution of flow in hepatocellular carcinoma during balloon-occluded TACE**

P. Lucatelli, *P. Vetri*, S. Ciaglia, B. Rocco, E. Damato, M. G. Travaglini, P. Ricci, C. Catalano; Rome/IT
*(paolovvetri@gmail.com)*

**Purpose:** The purpose of this study was to demonstrate in vivo redistribution of the blood flow towards HCC's lesions by utilizing two-dimensional perfusion-angiography in balloon occluded-TACE procedures.
**Methods or Backround:** Thirty patients with 35 HCC nodules treated in the period between January 2019 and November 2021. For each patient, a post-processing software leading to a two-dimensional perfusion-angiography was applied on each angiography performed via balloon microcatheter, before and after inflation. On the color map obtained, reflecting the evolution of contrast intensity change over time, five regions of interests (ROIs) were assessed: one on the tumour (ROI-t), two in the immediate peritumoural healthy liver parenchyma (ROI-ihl) and two in the peripheral healthy liver parenchyma (ROI-phl). The results have been interpreted with a novel in-silico model that simulates the haemodynamics of the hepatic arterial system.
**Results or Findings:** Among the ROIs drawn inside the same segment of target lesion, the time-to-peak of the ROI-t and of the ROI-ihl have a significantly higher mean value when the balloon was inflated compared with the ROIs obtained with deflated balloon (10.33 ± 3.66s vs 8.87 ± 2.60s (p=0.015) for ROI-t; 10.50 ± 3.65s vs 9.23 ± 2.70s (p=0.047) for ROI-ihl). The in-silico model prediction time-to-peak delays when balloon was inflated, matches with those observed in vivo. The numerical flow analysis shows how time-to-peak delays are caused by the obstruction of the balloon-occluded artery and the opening of intra-hepatic collateral.
**Conclusion:** The measurements identify predictively the flow redistribution in the hepatic arteries during b-TACE, supporting a proper positioning of the balloon microcatheter, in order to provide the operator with an advantageous flow distribution for chemoembolization.
**Limitations:** Limitations to the study are the fact that it is a retrospective analysis and that it is monocentric.
**Ethics committee approval:** Not applicable for this study.
**Funding for this study:** No funding was obtained for this study.
**Author Disclosures:**
Bianca Rocco: Nothing to disclose
Paolo Vetri: Nothing to disclose
Paolo Ricci: Nothing to disclose
Elio Damato: Nothing to disclose
Pierleone Lucatelli: Nothing to disclose
Maria Giulia Travaglini: Nothing to disclose
Carlo Catalano: Nothing to disclose
Simone Ciaglia: Nothing to disclose

### RPS 209-5

**Interventional treatment of unresectable or recurrent hepatic cholangiocarcinoma: transarterial chemoembolisation (TACE) with combined thermal ablation vs TACE alone**

T. J. Vogl, M. H. H. Albrecht, N-E. A. N-E. Mohammed, *A. M. Bucher*; Frankfurt a. Main/DE

**Purpose:** The purpose of this study was to retrospectively evaluate local tumour control and survival rates after targeted local therapy using transarterial chemoembolisation (TACE) with or without local thermal ablation in patients with unresectable or recurrent hepatic cholangiocarcinoma (CCC).
**Methods or Backround:** From 01/07 to 12/17, 152 patients (69 males/83 females; mean: 58.7 years) with CCC were retrospectively evaluated. The study included patients with both unresectable (80.2%) and recurrent lesions (19.8%). Patients were treated with at least three cycles of TACE (range; 3-26). 32 patients received combined TACE and local thermal ablation. Local tumour response was assessed by contrast-enhanced MRI (CE-MRI) based on the modified RECIST criteria (mRECIST) and the survival evaluated using the Kaplan-Meier method.
**Results or Findings:** The mean survival for all patients was 28.7 months (range:21.8-35.7). Patients with additional thermal ablation showed significantly longer survival vs. those with TACE as monotherapy (median: 27 vs. 18 months). Tumour response after three cycles of TACE was either stable (35.5%), partial response (41.4%), progressive (23%), or complete response (0%) and the response at the last follow-up was 25.7%, 15.2%, 59.2%, and 3.5%, respectively. Significant prognostic factors were found: nodal and/or systemic metastases, pre-therapeutic tumour load, initial local tumour response and associated application of local thermal ablation.
**Conclusion:** Targeted therapy of unresectable or recurrent CCC using TACE with an added value of thermal ablation may provide a therapeutic option for local tumour control and may improve patient's survival.
**Limitations:** Retrospective non-randomised study design limits the scope of this study.
**Ethics committee approval:** Institutional review board approval was obtained prior to commencing this retrospective study.
**Funding for this study:** No funding was obtained for this study.
**Author Disclosures:**
Thomas J. Vogl: Nothing to disclose
Moritz Hans Hans Albrecht: Nothing to disclose
Nour-Eldin Abdelrehim Nour-Eldin Mohammed: Nothing to disclose
Andreas Michael Bucher: Nothing to disclose

### RPS 209-6

**Cone beam-assisted transarterial chemoembolisation in malignant liver tumours: evaluation of parenchymal blood volume**

T. J. Vogl, E. Emrich, S. Bernatz, T. Gruber-Rouh, *A. M. Bucher*; Frankfurt a. Main/DE

**Purpose:** The study was aimed to assess the prognostic value of parenchymal blood volume (PBV) after transarterial chemoembolisation (TACE).
**Methods or Backround:** One hundred and thirty seven patients (60 females/77 males; median age 61; range 22–86) with malignant liver tumours of hepatocellular carcinoma (HCC) (n=27), colorectal cancer (CRC) (n=30) or other primaries (n=80) treated with TACE between 07/2016 and 07/2018 were retrospectively evaluated. Examinations were performed using Artis Pheno- and/or Artis Zeego-Dyna CT. CTs were reworked at a dedicated workstation to create a PBV map, which was overlapped with the associated MRI to determine tumour diameter and PBV. Patients were divided into 2 groups: PBV<50 or >50ml/1000 ml and subdivided regarding the primaries. Median survival was calculated using Kaplan-Meier and compared using the log-rank test.
**Results or Findings:** One hundred and eleven patients with 2-6 TACEs (average: 3) were included for measuring reduction PBV and tumor size: In 27/111 patients with initial PBV <50 ml/1000 ml, tumour volume, independent of the primary, was reduced by 13.26%, PBV by 23.11%. In 84/111 patients with PBV>50 ml/1000 ml, tumour volume was reduced by 24.01%, PBV by 44.69%. In the overall study population (n=137) patients with PBV>50 ml/1000 ml (n=101) survived on average 15.05 months, patients with PBV<50 ml/1000 ml (n=36) 10.01 months (p<0.002). The subgroup analysis showed a longer median survival in the HCC group at PBV>50 ml/1000 ml of 18.09 months. For CRC and other primaries survival time for high and low PBV is almost identical with 11.64 months for CRC and 12.03 months for other primaries.
**Conclusion:** High initial PBV values in the HCC group showed a prolonged survival time. In the other subgroups (CRC, other primaries) there was no effect on survival time regarding the initial PBV value.
**Limitations:** Retrospective study design, preparation and analysis of PBV cards is very time-consuming, different pretreatments of the primary tumours, partly no longer traceable, all these are the limitations of this study.
**Ethics committee approval:** A consenting ethics vote was issued by the Frankfurt Ethics Committee on January 17, 2019 (business no: 376 / 18).
**Funding for this study:** There was no funding for this study.
**Author Disclosures:**
Eileen Emrich: Nothing to disclose
Thomas J. Vogl: Nothing to disclose
Tatjana Gruber-Rouh: Nothing to disclose
Simon Bernatz: Nothing to disclose
Andreas Michael Bucher: Nothing to disclose

### RPS 209-7

**Long-term outcome of transarterial radioembolisation for patients with hepatocellular carcinoma**

*R. Muglia*, P. Marra, C. Prussia, M. De Giorgio, M. Viganò, A. Gerali, G. L. Poli, S. Fagiuoli, S. Sironi; Bergamo/IT
*(r.7@hotmail.it)*

**Purpose:** Transarterial radioembolisation (TARE) is a treatment indicated across many stages of hepatocellular carcinoma (HCC). We aimed to assess radiological response, safety and overall survival (OS) of TARE for HCC, identifying predictors of OS.
**Methods or Backround:** We included consecutive patients treated with TARE from 2012 to 2021 in a single centre with following criteria: presence of at least 1 measurable HCC without extrahepatic metastases, Child-Pugh score (CPS) A/B, ECOG performance status 0/1. Only the first TARE was considered in those patients who received >1 procedure. The radiological response by mRECIST criteria was evaluated 3/4 months after TARE. Uni- and multivariable analyses were used to explore the features at time of TARE and post-TARE radiological evaluation.
**Results or Findings:** Among the 142 patients (median age 67 years, 85% males, 92% cirrhotics, BCLC-A 29%, BCLC-B 35%, BCLC-C 36%, HCV+ 46%, CPS-A 85%, median alpha-fetoprotein 27 ng/mL) the median OS was 16.7 months with a 3-yrs cumulative survival rate of 28%. 31%, 39%, 9% and 21% of patients had complete response (CR), partial response (PR), stable disease (SD) and progressive disease (PD), respectively. BCLC stage and alpha-fetoprotein levels at time of TARE, delta alpha-fetoprotein (from TARE to post-TARE evaluation) and radiological response were statistically related to OS. Alpha-fetoprotein >21.4 ng/mL and BCLC-C at time of TARE were significantly related with death [HR 1.48 (95%CI 1.00-2.18, p=0.048) and 1.71 (95%CI 1.05-2.79, p=0.031),respectively] although only radiological incomplete response (PR, SD, PD) had higher HR for death (3.34, 95%CI 2.03-5.79, p<0.0001), at multivariate analysis. Adverse events occurred in 27% of patients (1 severe).
**Conclusion:** TARE is effective for HCC patients across different disease stages, and response to treatment remains the most important predictor of OS.
**Limitations:** Possible biases in patients selection leading to cohort inomogeneity limit this study.
**Ethics committee approval:** All procedures performed were in accordance with the ethical standards of the institutional and/or national research committee and with the 1964 Helsinki declaration and its later amendments or comparable ethical standards.
**Funding for this study:** This study was not supported by any funding.
**Author Disclosures:**
Sandro Sironi: Nothing to disclose
Massimo De Giorgio: Nothing to disclose
Paolo Marra: Nothing to disclose
Mauro Viganò: Nothing to disclose
Carolina Prussia: Nothing to disclose
Alberto Gerali: Nothing to disclose
Riccardo Muglia: Nothing to disclose
Gian Luca Poli: Nothing to disclose
Stefano Fagiuoli: Nothing to disclose

### RPS 209-8

**Percutaneous thermal segmentectomy for liver malignancies over 3 cm: retrospective multicentric study of mid-term oncological performance and predictors of sustained complete response**

*B. Rocco*[1], R. Argirò[1], V. Semeraro[1], Q. Lai[1], C. Catalano[1], R. Iezzi[1], L. Crocetti[2], P. Lucatelli[1]; [1]Rome/IT, [2]Pisa/IT
*(biancarocco.br@gmail.com)*

**Purpose:** Report the mid-term oncological results of a multicenter retrospective experience on percutaneous thermal segmentectomy (single-step combination of balloon-occluded MWA -bMWA- followed by balloon-occluded TACE -bTACE-) in patients with liver malignancies >3 cm and to identify risk factors for the loss of sustained complete response in the target lesion.
**Methods or Backround:** Sixty-three patients (40/23, male/female) with liver primary malignancies (hepatocellular carcinoma,HCC=49; intra-hepatic cholangiocarcinoma,iCC=4) and metastasis (n=10) were treated. Median diameter of the target lesion was 4.5 cm (range: 3-7 cm). bMWA was performed, in a single-step procedure, after balloon-microcatheter inflation, followed eventually by bTACE (with epirubicin or irinotecan). Oncological results at 1 month and at 3 months interval until 12-15 months, were evaluated using m-RECIST (HCC) and RECISTv1.1 (metastasis/iCC).

**Results or Findings:** Median follow-up was 9.2 months. At one month follow-up 79.4% of patients presented a complete response and the remaining 20.6% of patients were partial responders. The 3-6 months follow up was reached by 59/63 patients: 83.3%(48/59) of patients showed a sustained complete response, while 10.2%(6/59) and 8.5%(5/59) of patients had respectively a partial response and a progressive disease. At the last available follow-up, the global median time of sustained complete response was 9 months, with 65.9% of cases showing a complete response. While, no relevant risk factors were identified for obtaining a complete response after the bMWA-bTACE, an initial diameter of the target lesion ≥ 5cm showed to be the unique independent variable for the risk of failure in maintaining a complete response at 6 months (OR=8.58, 95%CI=1.38-53.43; P=0.02).
**Conclusion:** Percutaneous thermal segmentectomy allows to achieve promising oncological results in patients with >3 cm tumors, with tumour dimension ≥5 cm being the only risk factor associated with the failure of a sustained complete response.
**Limitations:** Retrospective nature
**Ethics committee approval:** Sapienza ethics committee approved of this study.
**Funding for this study:** No funding was obtained for this study.
**Author Disclosures:**
Bianca Rocco: Nothing to disclose
Vittorio Semeraro: Nothing to disclose
Quirino Lai: Nothing to disclose
Roberto Iezzi: Nothing to disclose
Pierleone Lucatelli: Nothing to disclose
Renato Argirò: Nothing to disclose
Carlo Catalano: Nothing to disclose
Laura Crocetti: Nothing to disclose

### RPS 209-9

**Radiomics analysis of pre-treatment imaging in hepatocellular carcinoma (HCC) undergoing trans-arterial chemoembolisation (TACE): correlations with treatment response and survival**
P. Lucatelli, *E. Damato*, E. P. Perrone, B. Rocco, M. Forlino, A. De Santis, C. Catalano; Rome/IT

**Purpose:** Radiomics uses radiological imaging to generate multi-dimensional data, defined as features. The novelty of radiomics is the possible correlation with clinical endpoints, mostly in oncological diseases. This a retrospective study investigate correlations between pre-treatment imaging radiomics and clinical outcomes in patients with hepatocellular carcinoma (HCC) undergoing transarterial chemoembolisation (TACE).
**Methods or Backround:** We selected pretreatment data (clinical, CT scan and laboratory) of 64 HCC Patients treated with TACE. With an open-source software we extract 68 features. Therapeutic outcome was divided in "response" (complete and partial response) and "non-response" (stable disease). Response to TACE was assessed with mRECIST criteria. Primary endpoint was correlation with clinical response to treatment. Secondary endpoint was overall survival.
**Results or Findings:** Primary endpoint: clinical data related to response (Chi-Square test) were age (upper median, p=0.027), Child-Pugh Score (A vs B, P=0.009) and albumin (upper median, P=0.009). Twelve radiomics features were related with response (Mann-Whitney test, p<0.05), namely: CONVENTIONAL, SHAPE, GLRLM, NGLDM, GLZLM family. Secondary endpoint: clinical data related to survival (Cox regression model) were: age, number of HCC nodules, albumin, history of ascites and hepatic encephalopathy. Kaplan Meier curve showed that Patients that had at least two of the aforementioned clinical variable experienced inferior survival, Log Rank: p<0,01. Radiomic parameters related to survival were: GLCM_Entropy_median, GLZLM_SZHGE_median. Kaplan Meier curve showed that Patients with high median scores of GLCM_entropy and GLZLM_SZHGE experienced inferior survival, Log Rank: p=0.022.
**Conclusion:** Our study showed that some radiomic features have predictive or prognostic value in HCC Pts undergoing TACE. Clinical and laboratory data are always essential. Integration of the two models can help management of HCC patients.
**Limitations:** Limited sample size. Further studies are needed to develop standardised models for performing radiomic analysis.
**Ethics committee approval:** Not applicable for this study.
**Funding for this study:** No funding was obtained for this study.
**Author Disclosures:**
Mariana Forlino: Nothing to disclose
Bianca Rocco: Nothing to disclose
Elio Damato: Nothing to disclose
Adriano De Santis: Nothing to disclose
Pierleone Lucatelli: Nothing to disclose
Carlo Catalano: Nothing to disclose
Elio Pietro Perrone: Nothing to disclose

### RPS 209-10

**European multicentre retrospective study of balloon-occluded transarterial chemoembolisation (B-TACE) for hepatocellular carcinoma (HCC) long term (31 months) follow-up**
P. Lucatelli[1], *E. Damato*[1], B. Rocco[1], T. Debaere[2], G. Verset[3], F. Fucilli[4], A. Paccapelo[5], C. Catalano[1], C. Mosconi[5]; [1]Rome/IT, [2]Villejuif/FR, [3]Brussels/BE, [4]Triggiano/IT, [5]Bologna/IT

**Purpose:** The aim of this retrospective multicentric European study was to evaluate the long-term tumor response rates of balloon-occluded transarterial chemoembolisation (B-TACE) in HCC patients.
**Methods or Backround:** B-TACE procedures response were evaluated according to m-RECIST criteria with multiphasic CT/MRI imaging at 1 months after the procedure and then every 3-6 months. Data were collected from six European centers from 2015 to 2022. Values assessed at latest available follow-up were disease control (DC), progressive disease (PD) and Progression free survival (PFS), for both target lesions and patient overall.
**Results or Findings:** Of the 91 patients of the original cohort, 19 patients were lost at follow-up, resulting in a final cohort of 72 patients. Mean maximum diameter of target lesion was 36.5±19.4 mm, with a median follow-up of 31.6 months. Overall Response was DC 33.3% (24 patients) and PD 66.7% (48 patients). Target lesion long-term response was DC 69.4% (50 patients) and PD 30.6% (22 patients). Median Progression-Free Survival was 9.3 months for Overall Response and not Reached for Target Lesion Response (Local Recurrence-Free), among the 22 patients with local recurrence the mean time observed for PD was 9.8±19.4 months (median 7.7, range 1-31).
**Conclusion:** The study proves that B-TACE permits to achieve an high sustained disease control rate of the target lesion whereas progression occurred within first 7 months from initial treatment.
**Limitations:** Small sample size
**Ethics committee approval:** Not applicable for this study.
**Funding for this study:** No funding was received for this study.
**Author Disclosures:**
Fabio Fucilli: Nothing to disclose
Bianca Rocco: Nothing to disclose
Gontran Verset: Nothing to disclose
Thierry Debaere: Nothing to disclose
Cristina Mosconi: Nothing to disclose
Elio Damato: Nothing to disclose
Pierleone Lucatelli: Nothing to disclose
Carlo Catalano: Nothing to disclose
Alexandro Paccapelo: Nothing to disclose

### RPS 209-11

**MRI-based investigation of dynamic changes in viable hepatocellular carcinoma following transarterial chemoembolisation**
W. Wang, Y-C. Wang, *S-H. Zhang*; Nanjing/CN
*(shuhangzhang@outlook.com)*

**Purpose:** The aim of this study was to analyse the dynamic changes in MRI radiological features of viable tumours at different follow-up times after the initial Tanscatheter arterial chemoembolisation (TACE) treatment for hepatocellular carcinoma (HCC) to improve the understanding and diagnostic accuracy of viable tumours.
**Methods or Backround:** The study analysed a prospective, single-arm, multicentre clinical trial data set (NCT03113955) and another three centres of clinically diagnosed patients with intermediate-stage HCC who received TACE as their first treatment. Viable tumours in two follow-up MRI were evaluated according to the Liver Imaging Reporting and Data System (LI-RADS). Radioligical features were evaluated including signal characteristics of T1WI, T2WI, and DWI, the presence or absence of wash in and wash out, as well as the size, location, and shape of the viable tumours.
**Results or Findings:** There were 157 HCCs in multicentre data set and 297 HCCs in another three centers met inclusion criteria. After exclusion, there were total 160 viable tumours on both follow-up MRI. Compared with the radiological features of the baseline, the T2WI hyperintensity (P= .002), wash out (P< .001) and the tumour short diameter (P< .001) of the first follow-up viable tumours significantly decreased. Compared with the radiological features of the first follow-up viable tumours, only the DWI hyperintensity of the second follow-up viable tumours increased (P= .009). For the shape features, both follow-ups showed mostly regular shapes (66.9%, 60.6%). Another location feature, the first follow up viable tumour showed mostly internal location (51.3%), but the second follow up viable tumour showed mostly non-internal location (66.9%).
**Conclusion:** The radiological features of viable tumours at early follow up post-TACE showed more variability compared to the baseline HCC, while those at second follow-up remained variable but less than the first follow up.
**Limitations:** Not applicable for this study.
**Ethics committee approval:** Not applicable for this study.

**Funding for this study:** No funding was received for this study.
**Author Disclosures:**
Yuan-Cheng Wang: Nothing to disclose
Shu-Hang Zhang: Nothing to disclose
Weilang Wang: Nothing to disclose

**09:30-11:00** **Research Stage 3**

### Research Presentation Session: Cardiac

### RPS 203

### Strain analysis in cardiac MRI

**Moderator**
U. Reiter; Graz/AT
*(ursula.reiter@medunigraz.at)*
**Author Disclosures:**
Ursula Reiter: Board Member: Scientific Editorial Board of European Radiology, Insights Into Imaging

### RPS 203-2

**The additive effects of coronary obstruction on right ventricular diastolic and systolic dysfunction in type 2 diabetes mellitus patients: evaluated by CMR feature tracking**

*J. Wang*, Z-G. Yang, Y. Li, Y-K. Guo, H. Fang, W-F. Yan, Y. Jiang; Chengdu/CN

**Purpose:** Obstructive coronary artery disease and type 2 diabetes mellitus (T2DM) commonly coexist, and coexisting OCAD are at increased risk for adverse cardiovascular events in T2DM patients. This study aimed to investigate the impact of coronary obstruction on right ventricle (RV) systolic and diastolic function in T2DM patients, and explore independent predictors of reduced RV function.
**Methods or Backround:** Cardiac magnetic resonance (CMR) scanning was performed on 274 T2DM patients {161 patients without OCAD [T2DM(OCAD−)] and 113 with [T2DM(OCAD+)]} and 83 control subjects. CMR-derived parameters, including RV structure, function, global strains [including systolic peak strain (PS), peak systolic (PSSR) and diastolic strain rate (PDSR) in radial, circumferential and longitudinal directions] were measured and compared among observed groups. Univariable and multivariable linear regression analyses were performed to identify independent predictors of RV dysfunction.
**Results or Findings:** Compared to the control subjects, T2DM (OCAD-) patients exhibited reduced RV systolic and diastolic function, as evidenced by a decrease in all RV global strains except for radial PS, PSSR, and PDSR (all $P < 0.05$). T2DM(OCAD+) patients showed a significantly more severe impairment of RV systolic and diastolic function than T2DM(OCAD−) patients and control subjects (all $P < 0.05$). The presence of OCAD was independently correlated with reduced GCPS ( $\beta = -0.149$, $P < 0.05$), the radial ( $\beta = -0.204$, $P < 0.001$) and the longitudinal PS ( $\beta = -0.155$, $P < 0.05$) in the context of T2DM. Among T2DM(OCAD+) patients, Gensini score was associated with decreased GCPS ($\beta = -0.388$, $P < 0.001$).
**Conclusion:** In the context of T2DM, coronary artery obstruction exacerbated RV diastolic and systolic dysfunction. The presence of OCAD and Gensini score were independent predictors of decreased RV function.
**Limitations:** Not applicable
**Ethics committee approval:** Not applicable
**Funding for this study:** No funding was obtained for this study.
**Author Disclosures:**
Yuan Li: Nothing to disclose
Han Fang: Nothing to disclose
Jin Wang: Nothing to disclose
Ying-Kun Guo: Nothing to disclose
Yu Jiang: Nothing to disclose
Wei-Feng Yan: Nothing to disclose
Zhi-Gang Yang: Nothing to disclose

### RPS 203-3

**Preoperative left atrial strain predicts outcome in patients with HOCM after myectomy**

*S. Yang*, S. Zhao, S. Wang; Beijing/CN
*(yshj6769@126.com)*

**Purpose:** This study aims to investigate the prognostic value of preoperative FT-derived LA strain among patients with HOCM following septal myectomy.
**Methods or Backround:** In this retrospective, single-center study, 802 adult patients with HOCM who underwent septal myectomy from January 2010 through December 2017 were included. All patients underwent preoperative CMR with complete clinical outcome follow-up. Preoperative LA phasic strains were measured using CMR-FT. Primary endpoint included all-cause mortality, heart transplantation, unscheduled hospitalization for heart failure, and stroke. Secondary endpoint included all-cause mortality and heart transplantation.
**Results or Findings:** Of the 802 patients in this study, 116 experienced primary endpoints, 38 experienced secondary endpoints, and 18 experienced SCD/aborted SCD during a median follow-up of 56.7 (IQR, 40.2-78.6) months. LA reservoir strain held the best performance for predicting the occurrence of adverse outcomes. Compared with the group with LA reservoir strain > 22.9% (median), the annual event rate of primary endpoint(4.2% vs. 1.7%, $P < .0001$), secondary endpoint (1.5% vs. 0.4%, $P = .0005$), and sudden cardiac death (SCD) (0.7% vs. 0.2%, $P = .047$) was significantly higher in the group with LA reservoir strain ≤ 22.9%. In multivariable Cox proportional hazards regression analysis, LA reservoir strain≤22.9% was independently associated with primary endpoint (HR 1.98, 95%CI 1.26-3.11, P=.003) and secondary endpoint(HR 3.71, 95%CI 1.73-7.92, P=.001). Incorporating LA reservoir strain, the performance of the 2014 HCM Risk-SCD score improved for sudden cardiac death prediction (C-statistic: from 0.62 to 0.69; log-likelihood: from -110.31 to -108.27, P=.04).
**Conclusion:** In patients with HOCM undergoing septal myectomy, reduced preoperative LA reservoir strain assessed by CMR-FT is independently associated with postoperative adverse outcomes.
**Limitations:** Selection bias and referral bias might be induced in this observational study.
**Ethics committee approval:** The study complied with the Declaration of Helsinki and was approved by the institutional review boards of Fuwai Hospital.
**Funding for this study:** This study is supported by grants from the National Key Research and Development Program of China (No. 2021YFF0501404, No. 2021YFF0501400) and the key projects of the National Natural Science Foundation of China (No. 81930044).
**Author Disclosures:**
Shuiyun Wang: Nothing to disclose
Shihua Zhao: Nothing to disclose
Shujuan Yang: Nothing to disclose

### RPS 203-4

**Subacute and convalescent phases of Takotsubo syndrome: CMR Feature-Tracking evaluation of left atrial and ventricular involvement**

*G. C. Pambianchi*, L. Ruoli, G. Cundari, L. Marchitelli, C. Catalano, N. Galea; Rome/IT
*(giacomo.pambianchi@gmail.com)*

**Purpose:** The aim of this study was to evaluate the ventricular and atrial contractile dysfunction during the subacute and convalescent phases of Takotsubo (TTS), assessing myocardial strain by Cardiac Magnetic Resonance Feature-Tracking technique (CMR-FT).
**Methods or Backround:** We retrospectively selected 50 patients with clinical-radiological diagnoses of TTS who underwent CMR within 30 days since the onset of specific symptoms; 19 patients were studied during the subacute phase (saTTS: ≤7 days) and 31 during the convalescence (cTTS: 8–30 days). We measured the left ventricular (LV) longitudinal, circumferential, radial systolic strain (lvGLS, lvGCS, lvGRS), the left atrial (LA) reservoir (laS_r), conduit (laS_cd), and booster pump strain (laS_bp). CMR parameters were compared with those of 30 healthy subjects.
**Results or Findings:** All the patients were female (100%), with a mean age of 69±13 years for TTS and 63±11 years for controls. When compared to controls, TTS patients showed reduced values of LV and LA strain parameters (p<0.05 for all). The saTTS showed increased laS_bp (12.7±2.6% vs 9.8±1.9%; p<0.001), reduced lvEF (47.4±11.9% vs 54.8±9.9%; p:0.028), lvGLS (-11.9±3.9% vs 14.6±3.8%; p:0.044), as compared to cTTS. The days passed from the onset of symptoms and CMR was inversely related to laS_bp (r: -0.484; p<0.001), with a direct correlation with the lvGLS (r: 0.470; p:0.001) with Pearson. In ROC analysis, laS_bp showed the best discriminatory power between saTTS and cTTS, with an AUC of 0.815 (95% CI: 0.684-0.945, p<0.001).
**Conclusion:** During the early phases of the TTS LA Booster Pump, function is significantly increased and gradually decreases over time. laS_bp could be an early marker of cardiac function impairment, even better the LV Strain and EF.
**Limitations:** The population under analysis was numerically limited and exclusively female.
CMR follow-up of the patients wasn't included in the study. Single-centre study.
**Ethics committee approval:** This study was approved
**Funding for this study:** No funding was obtained for this study.
**Author Disclosures:**
Giacomo Carlo Pambianchi: Nothing to disclose
Giulia Cundari: Nothing to disclose
Livia Marchitelli: Nothing to disclose
Nicola Galea: Nothing to disclose
Carlo Catalano: Nothing to disclose
Letizia Ruoli: Nothing to disclose

### RPS 203-5

**Impairment of left atrial and ventricular strain as early markers of myocyte dysfunction in hereditary transthyretin (h-TTR) mutation carriers**

*L. Conia*, L. Ruoli, G. Stancanelli, G. Cundari, C. Catalano, N. Galea; Rome/IT
*(luca.conia@uniroma1.it)*

**Purpose:** The aim of the study was to investigate left atrial and ventricular contractile function and myocardial tissue characteristics by CMR in carriers of an hereditary transthyretin (h-TTR) mutation related with cardiac amyloidosis (CA) in pre-hypertrophic phase, compared with CA patients with left ventricular hypertrophy (LVH) and healthy controls.
**Methods or Backround:** Twenty patients with definite diagnosis of CA (myocardial biopsy and/or genetic test and/or myocardial scintigraphy) and ten subjects with h-TTR mutation related to CA without LVH, who performed CMR in a 3T scanner between 2020-2023 were enrolled and compared with ten healthy age- and sex- matched controls. CMR image analysis was performed using a dedicated software (Cvi42, circle cardiovascular imaging) and LV volumes, maximal wall thickness, native-T1, LV global longitudinal (GLS), circumferential (GCS), atrial reservoir (ARS), conduit (ACS) and booster-pump (ABPS) strain were measured.
**Results or Findings:** Native-T1 values were higher in patients with hypertrophic CA (1348±87 ms) compared to h-TTR-mutation carriers (1227±33 ms) and healthy controls (1225±35 ms) with a p-value < 0,0003, whereas no significant difference was found between TTR-mutation carriers and healthy controls (p-value > 0,999). ARS, GLS and GCS were higher in healthy controls (respectively 25.6±2; 20.3±2; 21.9±5 %) compared to TTR-mutation carriers (respectively 22.0±4; 15.0±5; 15.5±5 %) with p-values < 0,05; there was non significant difference of ACS and ABPS between the two groups (p-value> 0,5).
**Conclusion:** In h-TTR mutation carriers CMR evaluation showed no significant alterations in T1 mapping values compared to healthy subjects, whereas a significant reduction was found in atrial and ventricular strain features, indicating a potential role as early markers of myocyte dysfunction.
**Limitations:** Small study population. Single center study. Lack of histologically evaluations in h-TTR carriers. Lack of a clinical and CMR follow-up.
**Ethics committee approval:** No information provided by the submitter.
**Funding for this study:** No funding was obtained for this study.
**Author Disclosures:**
Giulia Cundari: Nothing to disclose
Giuseppe Stancanelli: Nothing to disclose
Luca Conia: Nothing to disclose
Nicola Galea: Nothing to disclose
Carlo Catalano: Nothing to disclose
Letizia Ruoli: Nothing to disclose

### RPS 203-6

**Acute cardiac injury after postoperative left-sided breast cancer radiotherapy assessed by CMR feature-tracking strain**

*L. Zheng*, M-X. Yang, C. Luo, J. Xu, H. Wang, J. Li, J. Zhang, P. Zhou, J. Yin; Chengdu/CN
*(linlinleslie@163.com)*

**Purpose:** Chest radiotherapy could lead to cardiac toxicities; this study aims to assess the acute cardiac injury after left-side breast cancer radiotherapy using CMR feature-tracking strain.
**Methods or Backround:** Patients treated with breast/chest-wall radiotherapy for left-side breast cancer were prospectively enrolled, and the mean heart dose (MHD) was calculated. CMR scans, consisting of cardiac cine and T2-weighted imaging, were performed within two weeks before and after radiotherapy. Left ventricular ejection fraction (LVEF), global radial strain (GRS), global circumferential strain (GCS), global longitudinal strain (GLS), and myocardial edema were analyzed by CVI 42 post-processing software.
**Results or Findings:** Thirty women patients aged 46±9 years were included (16 received breast radiotherapy and 14 received chest-wall radiotherapy). The MHD in all patients was 5.33 (3.13-9.19) Gy; and the MHD of chest-wall group was higher compared with that of breast group (9.65±2.05Gy vs. 3.45±1.15Gy, P<0.001). After radiotherapy, LVEF showed no significant changes but myocardial edema was observed in nine patients in the whole cohort. Compared with baseline scan, a decrease of GCS (-20.04±1.72 vs. -18.98±2.04, P=0.029) and GLS (-15.64±2.49 vs. -14.32±2.19, P=0.001) was observed in all patients after radiotherapy, while no differences of GRS were shown. In the chest-wall group, GRS [37.67±6.89 vs. 34.97 (29.95-38.49), P=0.038], GCS (-20.33±1.29 vs. -18.61±2.59, P=0.028) and GLS (-16.29±2.68 vs. -14.41±2.46, P=0.007) all demonstrated a decrease after radiotherapy; however, no change of GRS, GCS and GLS was observed in the breast group.
**Conclusion:** Left-side breast cancer radiotherapy could cause acute cardiac injury, which is more pronounced in patients with higher cardiac radiation dose. CMR strain allows the sensitive detection of radiation-related cardiac dysfunction soon after radiotherapy.
**Limitations:** The changes of left ventricular strain after radiotherapy in long-term follow-up and its clinical significance require further studies.
**Ethics committee approval:** The clinical protocol in this study was approved by the institutional ethics review board of our hospital.
**Funding for this study:** This study was supported by National Natural Science Foundation of China (82202094) the Science and Technology Department of Sichuan Province (2022NSFSC1600)
**Author Disclosures:**
Junjie Li: Nothing to disclose
Linlin Zheng: Nothing to disclose
Peng Zhou: Nothing to disclose
Cheng Luo: Nothing to disclose
Jia Xu: Nothing to disclose
Jun Yin: Nothing to disclose
Meng-Xi Yang: Nothing to disclose
Hao Wang: Nothing to disclose
Jianhui Zhang: Nothing to disclose

### RPS 203-7

**Unveiling subclinical myocardial changes in a cohort of treatment-naive cancer population with diffuse large B-cell lymphoma (DLBCL) and breast cancer: is advanced cancer a cardiovascular syndrome?**

*C. Lisi*, S. Figliozzi, F. Catapano, R. Levi, L. Monti, M. Francone; Milan/IT

**Purpose:** Pre-chemotherapy patients with advanced cancer may experience myocardial alterations due to inflammation and neuro-hormonal activation. These changes could serve as subclinical indicators of latent heart dysfunction, allowing for enhanced cardiotoxicity assessment and patient management. Our study sought to explore cardiac MR (CMR) potentials in shedding light on the cancer-associated uncontrolled immune activation hypothesis and to provide early biomarkers of myocardial damage.
**Methods or Backround:** In this prospective single-center study, we examined 70 individuals, including 35 healthy controls and 35 patients with de novo DLBCL or breast cancer. All patients underwent CMR before starting chemotherapy. Univariate and multivariate analyses were performed to detect differences in myocardial tissue characterisation and functional parameters between the two groups.
**Results or Findings:** Significant differences were found in native T1 mapping times (p<0.01), with oncologic T1 at 998 msec vs. healthy T1 at 979.5 msec. Global longitudinal strain (GLS) values also differed significantly (p<0.01), with oncologic GLS at 15.06 and healthy GLS at 19.9. No significant differences were found in global biventricular function ad T2 mapping.
**Conclusion:** Advanced cancer is a multifaceted syndrome with profound involvement of the cardiovascular system. It initiates tissue inflammation, oxidative stress, and neuro-hormonal activation, resulting in substantial myocardial tissue changes. These changes manifest as myocardial fibrosis and apoptosis, potentially accounting for the increase in native T1 values and the decrease in GLS. This preliminary study underscores the importance of early tissue modifications as subclinical biomarkers of cardio-toxicity and prompts a reconsideration of the significance attributed to chemotherapy-induced myocardial alterations.
**Limitations:** This study is limited by its monocentric design and the relatively small sample size.
**Ethics committee approval:** No additional info was provided by the submitter.
**Funding for this study:** No funding was obtained for this study.
**Author Disclosures:**
Lorenzo Monti: Nothing to disclose
Costanza Lisi: Nothing to disclose
Federica Catapano: Nothing to disclose
Marco Francone: Nothing to disclose
Riccardo Levi: Nothing to disclose
Stefano Figliozzi: Nothing to disclose

### RPS 203-8

**Atrial and ventricular strain using cardiovascular magnetic resonance in the prediction of outcomes of pericarditis patients: a pilot study**

*R. Cau*[1], F. Pisu[1], G. Muscogiuri[2], R. Montisci[1], L. Saba[1]; [1]Cagliari/IT, [2]Milan/IT
*(riccardocau00@gmail.com)*

**Purpose:** The aims of our study were to explore with cardiovascular magnetic resonance (CMR) the impact of left atrial (LA) and ventricular myocardial strain in patients with acute pericarditis and to investigate their possible prognostic significance for adverse outcomes.
**Methods or Backround:** In this retrospective study, 33 consecutive patients with acute pericarditis were studied with CMR. The primary endpoint was the combination of recurrent pericarditis, constrictive pericarditis, and surgery for pericardial diseases defined as pericardial events. Atrial and ventricular strain functions were performed on conventional cine SSFP sequences.
**Results or Findings:** After a median follow-up time of 16 months (interquartile range [13-24]), 11 patients (eight males) acute pericarditis patients reached the primary endpoint. In multivariable Cox regression analysis, the left atrial (LA) reservoir, LA conduit, and LA conduit strain rate parameters were all independent determinants of adverse pericardial events. Adjusted models of

Wednesday

LA conduit and LA reservoir showed strong predictive performance, achieving significantly higher time-dependent AUCs than the LA conduit rate-based model (0.848 [95% CI, 0.7 – 1.0] and 0.851 [95% CI, 0.69 – 1.0], P < 0.05), for outcome prediction at 12 months.
**Conclusion:** LA reservoir and conduit mechanisms on CMR are independent predictor in patients with acute pericarditis. Including atrial strain parameters in the management of acute pericarditis may improve risk stratification.
**Limitations:** Retrospective single-center study
**Ethics committee approval:** This study was ethically approved by the IRB.
**Funding for this study:** No funding was obtained for this study.
**Author Disclosures:**
Roberta Montisci: Nothing to disclose
Riccardo Cau: Nothing to disclose
Francesco Pisu: Nothing to disclose
Giuseppe Muscogiuri: Nothing to disclose
Luca Saba: Nothing to disclose

### RPS 203-9

**Atrial and ventricular involvement in acute myocarditis patients with preserved ejection fraction**
*R. Cau*[1], F. Pisu[1], G. Muscogiuri[2], J. S. Suri[3], R. Montisci[1], L. Saba[1];
[1]Cagliari/IT, [2]Milan/IT, [3]Roseville, CA/US
*(riccardocau00@gmail.com)*

**Purpose:** To evaluate ventricular and atrial strain parameters using cardiovascular magnetic resonance (CMR) in patients with acute myocarditis (AM) and preserved ejection fraction (EF) in comparison with control subjects.
**Methods or Backround:** This retrospective study collected 126 consecutive patients (99 males, 27 females; mean age of 44.72 ± 18.22 years) with AM that fulfilled the Lake Louise criteria and with preserved EF and 52 age- and sex-matched control subjects. Left atrial (LA) and left ventricular (LV) strain functions were assessed on conventional cine SSFP sequences.
**Results or Findings:** AM patients with preserved ejection fraction had impaired global longitudinal strain (LS), global radical strain (RS), and global circumferential strain (CS) in comparison with control subjects. LA reservoir and conduit were significantly reduced in the myocarditis group compared to the control group. In cardiovascular risk factors-adjusted multivariable analysis, atrial and ventricular strain mechanisms remained significantly impaired in patients with AM with preserved EF compared to control subjects. Most importantly, a combined model of atrial and ventricular functions exhibited superior discriminatory ability when compared to base models of atrial or ventricular strain alone.
**Conclusion:** Besides ventricular strain parameters dysfunction, patients with AM and preserved EF demonstrated impaired reservoir and conduit mechanisms. A combined analysis of both atrial and ventricular function may improve the diagnostic accuracy for patients with AM and preserved EF.
**Limitations:** Retrospective single-center study
**Ethics committee approval:** The study was approved by the IRB.
**Funding for this study:** No funding was obtained for this study.
**Author Disclosures:**
Roberta Montisci: Nothing to disclose
Riccardo Cau: Nothing to disclose
Francesco Pisu: Nothing to disclose
Giuseppe Muscogiuri: Nothing to disclose
Jasjit S. Suri: Nothing to disclose
Luca Saba: Nothing to disclose

### RPS 203-10

**Strain analysis using feature-tracking CMR: a predictor of cardiac involvement in hypereosinophilia patients?**
*C. V. Gkizas*, B. Saal, M. Dubois, A. L. Rodriguez Musso, G. Lefevre, D. Launay, B. Longere, F. A. Pontana; Lille/FR
*(chgkizas@gmail.com)*

**Purpose:** The aim of this study was to assess the role of cardiac strain analysis using feature-tracking cardiac magnetic resonance (CMR) in patients with hypereosinophilia (HE).
**Methods or Backround:** Sixty-one consecutive patients (aged 52±18 years, 35 males) diagnosed with HE and referred for CMR were enrolled. Based on clinical evaluations, patients were classified into a “symptomatic group, Group1” (n=20), identified by evidence suggestive of potential HE-induced cardiopathy, and a “screening group, Group2” (n=41), where CMR was executed to exclude cardiac involvement despite the absence of any other cardiac abnormality. The CMR protocol included conventional cine imaging, T1 and T2 parametric mapping, and late gadolinium enhancement (LGE) imaging. Global longitudinal strain (GLS), global circumferential strain (GCS) and global radial strain (GRS) of left ventricle (LV) were measured (cvi42, Circle Cardiovascular Imaging).
**Results or Findings:** Despite preserved LV ejection fraction (LVEF), GLS, GCS and GRS were decreased in both groups but were more impaired in Group1 compared to Group2 (GLS= -10.2±3.9 vs -14.5±4.2, p=0.0004; GCS= -12.0±4.1 vs -14.9±4.2, p=0.015; GRS= 17.6±9.1 vs 24.1±9.6, p=0.0165). No differences were found in all strain parameters when comparing the two different patterns of LGE (ie. myocarditis or subendocardial fibrosis).
**Conclusion:** In symptomatic patients, strain parameters are diminished even when the LVEF remains within the normal range. Utilising feature-tracking CMR may offer a valuable tool in the early identification of cardiac abnormalities in asymptomatic HE individuals.
**Limitations:** Relatively small population of patients (rare disease). No endomyocardial biopsies
**Ethics committee approval:** This study was approved by the IRB: HP1907
**Funding for this study:** Authors did not receive a fund for this study
**Author Disclosures:**
Benjamin Longere: Nothing to disclose
Morgan Dubois: Nothing to disclose
Christos Vasileiou Gkizas: Nothing to disclose
Guillaume Lefevre: Nothing to disclose
David Launay: Nothing to disclose
Benjamin Saal: Nothing to disclose
Aimée Leilen Rodriguez Musso: Nothing to disclose
François Ascagne Pontana: Nothing to disclose

### RPS 203-11

**The additive effect of metabolic syndrome on left ventricular deformation and function in patients with obstructive coronary artery disease assessed by 3.0T cardiac magnetic resonance feature tracking**
*C. Min*, Y. Gao, Y. Zhigang, Y. Li; Chengdu/CN
*(2607046636@qq.com)*

**Purpose:** Metabolic syndrome (MetS) can increase the risk of morbidity and mortality of cardiovascular disease and obstructive coronary artery disease (OCAD). This study aimed to explore the impact of MetS on left ventricular (LV) deformation and function in OCAD patients and investigate the independent factors of impaired LV function and deformation.
**Methods or Backround:** One hundred and twenty one patients with OCAD and 52 sex- and age-matched controls who underwent cardiac magnetic resonance scanning were enrolled in the study. All OCAD patients were divided into two groups: OCAD with MetS [OCAD(MetS+), n = 83] and OCAD without MetS [OCAD(MetS−), n = 38]. LV functional and global strain parameters were measured and compared among the three groups. Multivariable linear regression analyses were constructed to investigate the independent factors of LV deformation and impaired LV strain in OCAD patients. The logistic regression analysis and receiver operating characteristic curve (ROC) were performed to test the prediction efficiency of MetS for LV deformation and impaired LV strain.
**Results or Findings:** From controls to the OCAD(MetS-)group to the OCAD(MetS+) group, LV mass (LVM) increased, and LV global function index (LVGFI) and LV global longitudinal peak strain (GLPS) decreased (all p < 0.05). Compared with the OCAD(MetS-) group, the LV GLPS declined significantly (p=0.027), the LVM increased (p=0.006), and the LVGFI decreased (p=0.043) in the OCAD(MetS+) group. MetS was an independent factor of decreased LV GLPS (β=−0.211, p=0.002) and increased LVM (β=0.221, p=0.003). The logistic multivariate regression analysis and ROC analysis showed that combined MetS improved the efficiency of predicting LV GLPS reduction (AUC = 0.88) and LVM (AUC = 0.89) increase.
**Conclusion:** MetS aggravated the damage of LV deformation and function in OCAD patients and was independently associated with LV deformation and impaired LV strain.
**Limitations:** Not applicable
**Ethics committee approval:** Not applicable for this study.
**Funding for this study:** No funding was obtained for this study.
**Author Disclosures:**
Yuan Li: Nothing to disclose
Chenyan Min: Nothing to disclose
Yue Gao: Nothing to disclose
Yang Zhigang: Nothing to disclose

### RPS 203-12

**Biventricular dysfunction and ventricular interdependence in patients with pulmonary hypertension: a 3.0 T cardiac MRI feature tracking study**
*H. Fang*, J. Li, Z-G. Yang; Chengdu/CN

**Purpose:** The objective of this study was to explore the right ventricular (RV) and left ventricular (LV) dysfunction and the ventricular interdependence in patients with pulmonary hypertension (PH), using cardiac MRI feature tracking (MRI-FT).
**Methods or Backround:** One hundred and seven PH patients (mean pulmonary artery pressure >20 mm Hg) and 72 age- and sex- matched controls were recruited. Strains were compared between controls, PH patients with preserved RV ejection fractions (EF) (≥ 40%, n = 48) and PH patients with reduced RVEF (<40%, n = 59) and multivariable linear regression analysis were performed to explore the RV-LV interdependence in patients with PH.
**Results or Findings:** RV strain decreased sequentially from controls, through PH with preserved RVEF, to PH with reduced RVEF. PH patients with reduced

Wednesday

RVEF had significantly lower LV strain, especially septal strain and LV peak diastolic strain rate compared with both controls and PH patients with preserved RVEF. Multivariable analyses showed that RVEF was independently correlated with LV strain, furthermore, independent of RVEF, RV strain was significantly correlated with LV strain(LVGRS: $\beta = 0.416$; LVGCS: $\beta = -0.371$; LVGLS: $\beta = 0.283$).
**Conclusion:** Subclinical impairment of RV function was found in PH with preserved RVEF. LV strain was impaired when RV was dysfunctional, which was associated with worsening RV strain. Therefore, while focusing on improving RV function, LV dysfunction in PH patients should also be monitored and treated early in order to slow the progression of the disease.
**Limitations:** First, this was a single-centre and cross-sectional study conducted in a large tertiary care hospital, and there might be potential selection bias. Second, because our study was a retrospective study, the inherent design limitations hindered our ability to consider changes in biventricular interdependence at different stages of PH.
**Ethics committee approval:** The current study received approval from the Biomedical Research Ethics Committee of West China Hospital, Sichuan University: 2019-648.
**Funding for this study:** This work was supported by the 1·3·5 project for disciplines of excellence, West China Hospital, Sichuan University (ZYGD18013).
**Author Disclosures:**
Han Fang: Nothing to disclose
Jiang Li: Nothing to disclose
Zhi-Gang Yang: Nothing to disclose

**09:30-11:00** **Research Stage 4**

### Research Presentation Session: Neuro

### RPS 211

### Normal brain development and psychiatric disorders

**Moderator**
P. M. Parizel; Perth, WA/AU
*(paul.parizel@gmail.com)*

### RPS 211-2

**Brain responses to complex addition problems in children, adolescents and adults**

*A. Istomina*[1], A. Faber[1], M. Ublinskiy[1], A. Manzhurtsev[1], M. Arsalidou[2]; [1]Moscow/RU, [2]Toronto, ON/CA
*(asya-istomina2014@ya.ru)*

**Purpose:** Addition is a basic mathematical operation that is taught first in school, and it is the most popular math operation in functional magnetic resonance imaging (fMRI) studies. fMRI studies consistently show that solving addition problems elicit fronto-parietal and cingulo-opercular areas in adults. Results in children is less consistent and no study to our knowledge examines addition problem-solving with 2-digit and 3-digit problems in children, adolescents, and adults. This current fMRI study highlights developmental effect in complex addition problem-solving.
**Methods or Backround:** Structural (TR=8.4 ms, matrix=240×222, voxel size=1.0×1.0×1.0 mm; FOV=240×240×170 mm; TE=3.9 ms; flip angle=8°) and functional (TR=2500 ms; TE=35 ms; FOV=230×230×150; 260 measurements per run; voxel size=3.0×3.0×3.0 mm) brain data of 20 children (9 female, 11-13 years), 20 adolescents (9 female, 14-16 years) and 20 adults (12 females; 18–29 years) were acquired using a magnetic resonance Philips Achieva dStream 3.0T scanner. Participants performed 2-digit and 3-digit addition tasks in a block design that lasted 32 seconds each. Participants were instructed to provide as many correct answers as possible. Data processing and analysis were carried out using AFNI software.
**Results or Findings:** Solving difficult addition problems elicits activity in common and distinct regions across age groups. Common areas include middle and superior frontal gyri, inferior parietal lobule, insular cortex. Adults also engaged the bilateral cerebellar and supplementary motor areas, whereas in children and adolescents, cerebellar activation was in the right hemisphere.
**Conclusion:** Results suggest that cognitive strategies may not be fully developed in children and adolescents. The agreement in brain areas among adults, contrasted with their absence in children and adolescents, offers insights into neural processing during math tasks. This highlights developmental distinctions in brain function and cognitive capacities across these groups.
**Limitations:** Motion is the most prevalent artifact, especially in pediatric samples.
**Ethics committee approval:** The local ethics committee approved all materials and procedures.
**Funding for this study:** The study was funded by the Brain Program of the IDEAS Research Center.
**Author Disclosures:**
Asya Istomina: Nothing to disclose
Maxim Ublinskiy: Nothing to disclose
Andrei Faber: Nothing to disclose
Marie Arsalidou: Nothing to disclose
Andrei Manzhurtsev: Nothing to disclose

### RPS 211-3

**Association of variability in body size with imaging metrics of brain structural health**

*J. Sun*, H. Lv, Z. Wang; Beijing/CN

**Purpose:** This study aimed to examine the associations of long-term variability in body mass index (BMI) and waist-to-hip ratio (WHR) with neuroimaging metrics that approximate brain structural health.
**Methods or Backround:** This cohort study recruited 1,114 participants aged 25-83 years from the subset of brain MRI acquisition within Kailuan study from 2020 onward. BMI and WHR variability during 2006-2018 were calculated as the average slope incorporating seven biennial measurements separately. We investigated the associations of BMI and WHR variability with MRI markers of brain tissue volumes, white matter microstructural integrity, white matter hyperintensity, and cerebral small vessel diseases.
**Results or Findings:** Progressive weight gain during follow-up was associated with decreased global fractional anisotropy (beta = -0.19, 95% CI -0.35 to -0.02) and increased mean diffusivity (beta = 0.16, 95% CI 0.01 to 0.31) and radial diffusivity (beta = 0.18, 95% CI 0.03 to 0.33). Weight loss was associated with lower periventricular white matter hyperintensity load (beta = -0.24, 95% CI -0.47 to -0.02) and lower risk of moderate-to-severe basal ganglia enlarged perivascular spaces (OR = 0.41, 95% CI 0.21 to 0.83). In adults with central obesity, WHR loss was associated with larger volumes of gray matter (beta = 0.50, 95% CI 0.11 to 0.89), hippocampus (beta = 0.62, 95% CI 0.15 to 1.09), and parahippocampal gyrus (beta = 0.85, 95% CI 0.34 to 1.37).
**Conclusion:** Progressive weight gain is associated with impaired white matter integrity. Weight and WHR losses are associated with improved general brain structural health. The present study contributes to a better understanding of the integrated associations between variations in general and central adiposity and brain structural health, especially in the early stages of the prevention of adiposity-related Alzheimer's disease and dementia.
**Limitations:** None
**Ethics committee approval:** The Medical Ethics Committee of Kailuan General Hospital approved the META-KLS study (IRB number: 2021002). Written informed consent was obtained from all the participants.
**Funding for this study:** This study was supported by grants 62171297 and 61931013 from the National Natural Science Foundation of China (No. [2015] 160 from the Beijing Scholars Program, No. ZYLX202101 from Beijing Hospitals Authority Clinical Medicine Development of Special Funding Support, No. 2021-135 from Beijing Municipal Health Commission-Beijing Key Clinical Discipline Funding.
**Author Disclosures:**
ZhenChang Wang: Nothing to disclose
Han Lv: Nothing to disclose
Jing Sun: Nothing to disclose

### RPS 211-4

**Resolving heterogeneity in post-traumatic stress disorder using individualised structural covariance network analysis**

*L. Chen*[1], N. Pan[1], L. Li[2], G. J. Kemp[3], S. Wang[1], Q. Gong[1], X. Suo[1]; [1]Chengdu/CN, [2]Changsha/CN, [3]Liverpool/UK

**Purpose:** The heterogeneity of post-traumatic stress disorder (PTSD) is an obstacle to both understanding and therapy, and this has prompted a search for internally-homogeneous biological subgroups within the broad clinical diagnosis. Our goal was to do this using the individual differential structural covariance network (IDSCN).
**Methods or Backround:** We constructed cortical thickness-based IDSCN using T1-weighted images of 89 individuals with PTSD (mean age 42.8 years, 60 female) and 89 demographically-matched trauma-exposed non-PTSD (TENP) controls (mean age 43.1 years, 63 female). The IDSCN metric quantifies how the structural covariance edges in a patient differ from those in the controls. We examined the structural diversity of PTSD and variation among subtypes using a hierarchical clustering analysis, the correlation between IDSCN and individually clinical symptoms, and sex-by-subtype interactions.
**Results or Findings:** PTSD patients exhibited notable diversity in distinct structural covariance edges, but mainly affecting three networks: default mode, ventral attention and sensorimotor. These changes predicted individual PTSD symptom severity. We identified two neuroanatomical subtypes: the one with greater PTSD symptom severity showed lower structural covariance edges in the frontal cortex and between frontal, parietal, and occipital cortex regions which are functionally implicated in selective attention, response selection and learning tasks. And there was no significant PTSD subtype x sex interaction.
**Conclusion:** Deviations in structural covariance in large-scale networks are common in PTSD, but fall into two subtypes. This work sheds light on the neurobiological mechanisms underlying the clinical heterogeneity, and may aid in personalised diagnosis and therapeutic interventions.
**Limitations:** First, this was one dataset and a single modality; future studies could use multimodal imaging and validation in an independent dataset. Second, it was a cross-sectional design; longitudinal research would be needed to establish whether these two PTSD subtypes showed different responses to treatment.
**Ethics committee approval:** The study protocol was reviewed and approved by the Sichuan University Research Ethics Committee. Each participant provided full-informed written consent. This study conforms to the provisions of the Declaration of Helsinki.
**Funding for this study:** This work was supported by the National Natural Science Foundation of China (Grant Nos. 82001800, 81761128023, 81820108018, and 82027808), and the Young Elite Scientists Sponsorship Program by CAST (Grant No. 2022QNRC001).
**Author Disclosures:**
Li Chen: Nothing to disclose
Song Wang: Nothing to disclose
Graham J. Kemp: Nothing to disclose
Lingjiang Li: Nothing to disclose
Xueling Suo: Nothing to disclose
Qiyong Gong: Nothing to disclose
Nanfang Pan: Nothing to disclose

### RPS 211-5

**Default-mode network overlap with the mirror-neuron system activation task in trauma-exposed veterans**

*O. Zakomornyi*, L. Myroniak, I. Kobzar, O. Omelchenko, I. Dykan; Kyiv/UA
*(doctorzak911@gmail.com)*

**Purpose:** The purpose of this study was to detect functional connectivity of the mirror-neuron system and the overlap with the default-mode network as a potential marker of PTSD in trauma-exposed veterans.
**Methods or Backround:** Fifteen trauma-exposed veterans (mean PCL5 value = 24) were studied with the fMRI using the event-related paradigm of different right-hand motion types observation with subsequent execution. A 3T scanner was used with the 16ch head-neck coil. Invivo fMRI stimulation hardware with mirror goggles and a display screen was used. EPI pulse sequence with TR/TE=3000/100. FSL software library was used for the analysis.
**Results or Findings:** Mirror neuron system fMRI activation was detected during the hand motion observation periods. In particular, activation of the posterior inferior frontal gyrus of the right and left hemispheres and extensive activation of inferior parietal lobules bilaterally were observed. In addition to the described activation of the precuneus, the inferior parietal lobules region was detected during the described motion observation phases. Default-mode network deactivation was not detected in its typical regions.
**Conclusion:** "Social-brain" networks like default-mode network and mirror-neuron system were detected to function coherently, while the deactivation of the default-mode network was decreased, which might be the imaging signature of PTSD in veterans and might be used for the detection of the PTSD severity markers.
**Limitations:** The study applies to the patient population with subacute trauma exposure period.
**Ethics committee approval:** Local ethics committee approval was received before the study.
**Funding for this study:** No additional funding was received.
**Author Disclosures:**
Oleksandr Zakomornyi: Nothing to disclose
Iryna Dykan: Nothing to disclose
Liudmyla Myroniak: Nothing to disclose
Oleksii Omelchenko: Consultant: fMRI consultant
Ihor Kobzar: Nothing to disclose

### RPS 211-6

**Abnormal reward circuits in adolescents with first-episode non-suicidal self-injury based on subcortical volume and lateralisation index**

R. Yu, *N. Liu*; Nanchong/CN
*(232402432@qq.com)*

**Purpose:** This study aims to investigate the structural characteristics of the reward circuit in adolescents with non-suicidal self-injury (NSSI) in the absence of comorbidities using subcortical volume and lateralisation index(LI). The objective is to elucidate the pathophysiological mechanisms of NSSI and provide structural evidence for NSSI.
**Methods or Backround:** This study prospectively enrolled 30 primary NSSI patients and 29 healthy controls (HC). Clinical characteristics included the self-rating anxiety scale (SAS), Beck Scale for Suicidal Ideation (BSI), Brief Assessment of Cognition in Schizophrenia (BACS), and Ottawa Self-Injury Inventory (OSI). Subcortical volume segmentation was conducted using Freesurfer software. LI quantified differences in left and right subcortical volume. Statistical analysis of clinical features, subcortical volume, and LI were performed using wilcoxon rank-sum and independent sample t-tests. Pearson or Spearman correlation was used to explore relationships between subcortical volume or LI and clinical characteristics.
**Results or Findings:** The NSSI group had higher SAS scores compared to the HC group ($P<0.05$). BACS scores in the NSSI group were lower than in the HC group ($P<0.05$). Compared to the HC group, the NSSI group exhibited reduced volume in the left accumbens and putamen, along with an increased LI of the accumbens ($p<0.05$). In the NSSI group, left putamen volume was negatively correlated with OSI scores ($p=0.045$), left accumbens volume was positively correlated with BACS scores ($p=0.033$), and LI of the accumbens was negatively correlated with OSI scores ($p=0.044$).
**Conclusion:** Our study reveals that in primary NSSI adolescents, subcortical volume of the left putamen and left accumbens are diminished, with LI indicating potential left-sided vulnerability. These findings provide a new sight and structural evidence for the reward circuits mechanism of NSSI.
**Limitations:** This is a cross-sectional study, so the exploration of the efficacy is limited.
**Ethics committee approval:** Ethical approval was obtained from the ethics board of the Affiliated Hospital of North Sichuan Medical College (2022ER406-1).
**Funding for this study:** The Bureau of Science & Technology and Intellectual Property Nanchong City (NO. 22SXQT0305). The Opening Project of Functional and Molecular Imaging Key Laboratory of Sichuan Province (NO. SCU-HM-202307001).
**Author Disclosures:**
Rui Yu: Nothing to disclose
Nian Liu: Nothing to disclose

### RPS 211-7

**Network controllability developmental patterns in first-episode drug-naïve schizophrenia**

*S. Li*, W. Zhang, S. Lui; Chengdu/CN

**Purpose:** Schizophrenia typically manifests during late adolescence and early adulthood. Studying early-stage patients is vital for understanding brain function without the influence of antipsychotic medications. While previous research has identified brain region and network deficits, little attention has been given to network controllability. Controllability is a predictor of the brain's capacity to transition between different states, with modal controllability being a phenotype associated with substantial changes in brain states. This study aims to investigate network controllability deficits in the early stages of schizophrenia while controlling for the influence of antipsychotic medication.
**Methods or Backround:** The study included 135 drug-naïve first-episode schizophrenia patients aged 16-30 and 119 matched healthy controls. Resting-state fMRI data were acquired using a 3-Tesla GE scanner. DPASF software processed data and computed voxel-wise functional connectivity. Linear Mixed-Effects Models incorporated regional modal controllability and factors including controllability, category (schizophrenia or healthy), gender, age, education level, and category interactions. Inter-group comparisons assessed regional modal controllability and age-related changes.
**Results or Findings:** Schizophrenia patients exhibited significantly lower regional modal controllability in the left inferior parietal gyrus compared to healthy controls ($p=0.02$, corrected). As schizophrenia patients aged, their regional modal controllability decreased, whereas healthy controls showed increased modal controllability with age. A significant difference in age-related changes in regional modal controllability emerged between diagnostic groups ($p=0.009$, corrected).
**Conclusion:** This study uncovers abnormal network development patterns in early-stage schizophrenia marked by reduced regional modal controllability, notably in the left inferior parietal gyrus. Understanding network controllability in schizophrenia's early stages is crucial and may inform treatment and intervention strategies.
**Limitations:** The study did not include long-term follow-up data to track how network controllability deficits evolve over time in early-stage schizophrenia. Longitudinal data could provide a more comprehensive understanding of disease progression.
**Ethics committee approval:** This study was approved by the Ethics Committee of West China Hospital, Sichuan University.
**Funding for this study:** This study was funded by the Miaozi Project in Science and Technology Innovation Program of Sichuan Province: Grant No. 2021028.
**Author Disclosures:**
Su Lui: Nothing to disclose
Wenjing Zhang: Nothing to disclose
Siyi Li: Nothing to disclose

### RPS 211-8

**Altered cerebellar effective connectivity in first-episode schizophrenia and their long-term changes after treatment**

*X. Wei*[1], H. Cao[2], C. Luo[1], Q. Zhao[1], C. Xia[1], Z. Li[1], Z. Liu[1], Q. Gong[1], S. Lui[1]; [1]Chengdu/CN, [2]Manhasset, NY/US
*(weixia.1013@qq.com)*

**Purpose:** We aimed to the find the detailed directional connectivity disruption of cerebellar cortex.
**Methods or Backround:** Cerebellar functional dysconnectivity plays a key role in schizophrenia (SZ) pathology. One hundred and eighty drug naïve first-episode patients with SZ (54 were reassessed after one-year treatment) and 166 healthy controls (HCs) were included. Their resting-state functional magnetic resonance images were used to perform Granger causal analysis, in which nine functional systems of the cerebellum were defined as seeds. The observed directional connectivity alterations at baseline were further assessed at follow-up and associated with psychotic symptom changes.
**Results or Findings:** We observed increased information projections in the first episode SZ from cerebellum to cerebrum (i.e., from the cerebellar attention and cingulo-opercular systems to the bilateral angular gyri, and from the cerebellar cingulo-opercular system to the right inferior frontal gyrus). In contrast, decreased information projections in the first episode SZ were mainly from cerebrum to cerebellum (i.e., from the right inferior temporal gyrus, the left middle temporal gyrus, the left putamen, and the right angular gyrus to the cerebellar language system) and inside the cerebellum (i.e., from the cerebellar cingulo-opercular system to the right cerebellum Crus II). With symptom remission after one-year antipsychotic treatment, the information projections from cerebrum to cerebellum were partly restored. The normalization extent of the information projection from the left middle temporal gyrus to the cerebellar language system was positively correlated with the change in general psychopathological symptoms.
**Conclusion:** These findings suggest that the decreased information projections from the cerebrum into the cerebellum during the acute phase of schizophrenia may be a state-dependent alteration that is related to symptoms and medication. While the increased information projections from the cerebellum may reflect a persistent pathologic trait.
**Limitations:** Not applicable.
**Ethics committee approval:** This study was approved by the Ethics Committee on Biomedical Research, West China Hospital of Sichuan University
**Funding for this study:** This study was supported by the National Natural Science Foundation of China (Project Nos. 82120108014, 82071908), National Key R&D Program of China (Project Nos.2022YFC2009901, 2022YFC2009900/2022YFC209903), China Postdoctoral Science Foundation (Project No. 2022M722270), the Youth Science Fund of the Natural Science Foundation of Sichuan Province, China (Project No. 2022NSFSC1435), CAMS Innovation Fund for Medical Sciences (CIFMS) (Project No. 2021-I2M-C&T-A-022), Chengdu Science and Technology Office, major technology application demonstration project (Project Nos. 2022-YF09-00062-SN, 2022-GH03-00017-HZ), Sichuan Science and Technology Program (Project No. 2021JDTD0002), Sichuan University Postdoctoral Interdisciplinary Innovation Fund (Project No. JCXK2209), the Fund of the Beijing Medical Award Foundation (Project No. YXJL-2022-0665-0189), Dr. Su Lui acknowledges the support from Humboldt Foundation Friedrich Wilhelm Bessel Research Award and Chang Jiang Scholars (Program No. T2019069).
**Author Disclosures:**
Xia Wei: Nothing to disclose
Zhiqin Liu: Nothing to disclose
Ziyu Li: Nothing to disclose
Hengyi Cao: Nothing to disclose
Chunyan Luo: Nothing to disclose
Qiyong Gong: Nothing to disclose
Qiannan Zhao: Nothing to disclose
Chao Xia: Nothing to disclose
Su Lui: Nothing to disclose

### RPS 211-9

**Evaluating diffusion tensor imaging of corpus callosum in patients with psychosis**

*R. Rastogi*, D. Goel; Moradabad/IN
*(rajulrst@yahoo.co.in)*

**Purpose:** Psychosis is a complex group of disease showing significant alterations in corpus callosum affecting the cognitive behavior of the patient. Diffuse tensor imaging (DTI) is an advanced MR imaging tool that depicts the microstructural changes in the white matter of the brain. Since DTI can predict callosal changes before visible thinning, hence it can be used to determine the prognosis of patients and manage patients accordingly.
**Methods or Backround:** Twenty patients with proven psychosis for more than one year underwent noncontrast MRI brain and DTI on 1.5T scanner followed by evaluation of callosal MD, FA & ADC measurement as well as changes in its thickness. This data was compared to 20 age & sex matched subjects who were normal healthy volunteers.
**Results or Findings:** Fourteen out of 20 patients revealed alterations in MD, ADC and FA of the corpus callosum with only 3 out of 14 revealed thinning of corpus callosum with ventriculomegaly.
**Conclusion:** Callosal DTI MR imaging in psychotic patients may be a novel screening tool of disease progression and prognostication allowing early identification of patients who may require adjuvant psychotherapy or more aggressive management. It may also serve as an objective tool for evaluating the effects of various drug in the management of retarding or cessation of the progression of psychosis.
**Limitations:** Small sample volume, single centre study
**Ethics committee approval:** This study was ethically approved.
**Funding for this study:** No funding was received for this study.
**Author Disclosures:**
Rajul Rastogi: Nothing to disclose
Deepti Goel: Nothing to disclose

### RPS 211-10

**Resting state functional MRI in patients with obsessive-compulsive disorder to detect the areas of activation**

*A. N. Parimalai*, A. Chellathurai, M. K. Logudoss, D. Lakshmi; Chennai/IN
*(anand7687@gmail.com)*

**Purpose:** The aim of the study is to analyse the various functionally connected networks in obsessive-compulsive disorder using Rs-fMRI (resting state functional MRI) and to compare the same with normal individuals, thereby defining the pathophysiology of obsessive-compulsive disorder.
**Methods or Backround:** All the patients who were diagnosed under DSM-5 criteria and were registered and undergoing treatment in the psychiatry Department in Government Stanley Medical College, Chennai. All our images were acquired using a 1.5 T MRI system and the resting state functional images were acquired using an echo-planar imaging (EPI) sequence.
**Results or Findings:** OCD patients showed significant decrease/absence of functional connectivity in executive control network and significant increase/presence of functional connectivity in orbito-fronto-striatal thalamic network
**Conclusion:** The altered functional connectivity in executive control network and orbito-fronto -striatal thalamic network forms the root cause for the obsessions & compulsions in OCD
**Limitations:** Not applicable
**Ethics committee approval:** It is an educational study and no ethical approval was sought.
**Funding for this study:** No funding was received for this study
**Author Disclosures:**
Murali Krishna Logudoss: Nothing to disclose
Dhana Lakshmi: Nothing to disclose
Amarnath Chellathurai: Nothing to disclose
Anand Narsingam Parimalai: Nothing to disclose

### RPS 211-11

**The association among individual grey matter volume of frontal-limbic circuitry, fatigue susceptibility, and comorbid neuropsychiatric symptoms following COVID-19**

*W. Bao*, Z. Luo, M. Zhang, X. Niu; Xi'an, China/CN

**Purpose:** Our study aimed to identify brain structural markers of post-COVID fatigue and to further investigate the correlation between individual grey matter volume, fatigue susceptibility, and concomitant neuropsychiatric symptoms.
**Methods or Backround:** Individuals following a mild SAS-COV2 infection (COV+) underwent neuropsychological measurements (n = 335) and MRI scans (n = 271) within one month, and 191 (70.5%) of the individuals were followed up at 3three months after infection. 67 healthy controls (COV-) completed the same protocol on recruitment to the study.
**Results or Findings:** The analysis of the whole brain revealed that there was no difference in grey matter volume during the acute phase between the study groups. In the COV+ group, however, fatigue severity was linked only to the volume of the right dorsal anterior cingulate cortex (dACC) and dorsolateral prefrontal cortex (DLPFC), which are both part of the frontal-limbic system. Furthermore, it should be noted that fatigue acted as a mediator in the associations between the volume of identified brain regions and COVID-related sleep, PTSD, and anxiety. It is important to highlight that the initial structural differences in the DLPFC observed during the early stage can predict fatigue symptoms three months after COVID-19 infection.
**Conclusion:** Our findings provide novel evidence on the neuroanatomical basis of fatigue vulnerability and emphasize fatigue as an important link from GMV in frontal-limbic regions to comorbid neuropsychiatric symptoms in the early recovery after infection. This may facilitate public health interventions by early identifying individuals at risk of developing post-COVID fatigue and implement treatment targeted at fatigue, further reducing the long-term adverse effects of the disease.

**Limitations:** Our data were collected in adults with mild COVID-19 who were in good health, which may limit the generalisability of the findings to a broader population.
**Ethics committee approval:** This study was approved by the Ethics Committee of The First Affiliated Hospital of Xi'an Jiaotong University: XJTU1AF2023LSK-013.
**Funding for this study:** This study was funded by the Shaanxi Province health brain science and imaging new technology research and innovation platform-2023TD-09.
**Author Disclosures:**
Wenrui Bao: Nothing to disclose
Zhaoyao Luo: Nothing to disclose
Xuan Niu: Nothing to disclose
Ming Zhang: Nothing to disclose

### RPS 211-12

**Neural correlates of auditory verbal hallucinations: a combined structural and resting state fMRI**

*F. Aldhafeeri*, K. Al Enazi; Hafar al-Batin/SA
*(aldhafeeri8@hotmail.com)*

**Purpose:** Auditory verbal hallucinations (AVH) refer to the perception of voices by subjects in the absence of external auditory stimulation. The objective of this study was to investigate the underlying neural mechanisms associated with experiencing AVH while excluding any presence of psychological or neurological condition.
**Methods or Backround:** This study was approved by the local research ethics committee. A total of 14 subjects suffering from AVH and 15 age and sex matched healthy controls were recruited in this study. All participants underwent structural magnetic resonance imaging (MRI), diffusion tensor imaging (DTI), and resting-state functional MRI (rs-fMRI). The findings acquired from each modality were utilised for statistical comparison between the control group and AVH group.
**Results or Findings:** Structural MRI revealed a remarkable cortical thickness reductions in the frontal and temporal lobes and cingulate cortex in the AVH group compared to the controls. Rs-fMRI exhibited reduced functional connectivity (FC) in the AVH group compared to the controls in the following regions: inferior frontal gyrus, anterior cingulate, and temporal cortex bilaterally. AVH subjects have demonstrated increased FC in the right thalamus, left hippocampal region and amygdala. Compared to the healthy control group, AVH demonstrated reduced mean fractional anisotropy (FA) in the right fronto-occipital fasciculus and the corpus callosum and higher mean diffusivity (MD) in the left inferior fronto-occipital fasciculus and anterior thalamic radiation.
**Conclusion:** Using a multimodel MRI techniques, current findings shed the light on the brain mechanisms of AVH by revealing the causal involvement of key brain areas involving the auditory, language, and memory networks.
**Limitations:** This study is limited by a small sample size; therefore, we recommend further studies with larger sample sizes to better understand the neural correlates of AVH.
**Ethics committee approval:** This study was approved by the Local Research Ethics Committee at University of Hafr Albatin, SA.
**Funding for this study:** This study did not receive any funding.
**Author Disclosures:**
Faten Aldhafeeri: Nothing to disclose
Khaled Al Enazi: Nothing to disclose

**11:30-12:30** **Research Stage 1**

### Research Presentation Session: Chest

### RPS 304

### MRI in thoracic imaging

**Moderator**
J. Biederer; Heidelberg/DE
*(juergen.biederer@gmx.net)*
**Author Disclosures:**
Jürgen Biederer: Equipment Support Recipients: Siemens, Bayer; Speaker: Roche, Boehringer Ingelheim, Fuji

### RPS 304-2

**Elexacaftor/Tezacaftor/Ivacaftor improves bronchial artery dilatation detected by magnetic resonance imaging in patients with cystic fibrosis**

*L. Wucherpfennig*[1], S. Wege[1], H-U. Kauczor[1], C. P. Heussel[1], O. Sommerburg[1], M. Stahl[2], M. Mall[2], M. Eichinger[1], M. Wielpütz[1]; [1]Heidelberg/DE, [2]Berlin/DE

**Purpose:** It was previously shown that magnetic resonance imaging (MRI) detects improvements in mucus plugging and bronchial wall thickening, but not lung perfusion in patients with cystic fibrosis (CF) treated with elexacaftor/ tezacaftor/ ivacaftor (ETI). It is still unclear, whether bronchial artery dilatation (BAD), a key feature of advanced lung disease, indicates irreversibility of perfusion abnormalities and whether BAD could be reversed in CF patients treated with ETI.
**Methods or Backround:** 59 adults with CF underwent longitudinal chest MRI including MR angiography (MRA) twice, 35 CF patients (mean age 31±7 years) before (MRI1) and after (MRI2) at least one month (mean duration 8±4 months) on ETI therapy and 24 control CF patients (mean age 31±7 years) without ETI. MRI was assessed using the validated chest MRI score, and presence and total lumen area of BAD were assessed with commercial software.
**Results or Findings:** The MRI global score was stable in the control group from MRI1 to MRI 2 (mean difference: 1.1±3.4, P=0.054), and was reduced in the ETI group (-10.1±4.2, P<0.001). In the control as well as in the ETI group, BAD was present in almost all patients at baseline (95% and 94%, respectively) and presence did not change at MRI2. The BAD total lumen area was stable in the control group from MRI1 to MRI2 (mean difference: 1.0±3.0 mm2, P=0.099), but decreased in the ETI group (-6.6±5.8 mm2, P<0.001). This decrease correlated with improvements in the MRI global score (r=0.540, P<0.001).
**Conclusion:** Our data show that BAD may be partially reversible under ETI therapy in adult CF patients with established disease.
**Limitations:** The number of patients treated with ETI was only moderate. Moreover, our study was conducted in adults with CF, excluding children with milder lung disease.
**Ethics committee approval:** This study was approved by the ethics committee of Heidelberg Medical Faculty.
**Funding for this study:** This study was supported by grants from the German Federal Ministry of Education and Research (82DZL00401, 82DZL004A1, 82DZL009B1), the German Research Foundation (STA 1685/1-1 to M.S.) and the Mukoviszidose e.V. (S02/09, C-H-P 1504). Funders had no involvement in the collection, analysis and interpretation of data, in the writing of the report and in the decision to submit the article for publication.
**Author Disclosures:**
Olaf Sommerburg: Nothing to disclose
Mirjam Stahl: Other: MS received grants from Vertex Pharmaceuticals (Independent Research Innovation Award) and personal fees for participation in advisory boards from Vertex Pharmaceuticals, outside the submitted work. MS is participant of the BIH-Charité (Advanced) Clinician Scientist Program funded by the Charité – Universitätsmedizin Berlin and the BIH.
Lena Wucherpfennig: Nothing to disclose
Claus Peter Heussel: Other: CPH declares advisory board membership with Boehringer Ingelheim unrelated to the present study.
Hans-Ulrich Kauczor: Other: HUK declares relationships with Siemens, Philips, Bayer, Boehringer Ingelheim, Sanofi, Median unrelated to the present study.
Sabine Wege: Nothing to disclose
Mark Wielpütz: Other: MOW declares advisory board membership with Boehringer Ingelheim unrelated to the present study and receives study grants from Vertex Pharmaceuticals unrelated to the present study.
Marcus Mall: Other: MAM received grants from Vertex Pharmaceuticals and personal fees for participation in advisory boards or consulting from Abbvie, Antabio, Arrowhead Pharmaceuticals, Boehringer Ingelheim, Enterprise Therapeutics, Kither Biotech, Pieris Pharmaceuticals, Vertex Pharmaceuticals, outside the submitted work.

Monika Eichinger: Other: ME declares advisory board membership with Boehringer Ingelheim unrelated to the present study and speaker honoraria by Boehringer Ingelheim, Roche Pharma and Vertex Pharmaceuticals unrelated to the present study.

### RPS 304-3

**Magnetic resonance imaging detects delayed perfusion inside perfusion defects in patients with cystic fibrosis**

*P. Leutz-Schmidt*[1], J. Grolig[1], S. Triphan[1], M. Mall[1], O. Sommerburg[1], A. Koeppe[1], B. Nestler[2], H-U. Kauczor[1], M. Wielpütz[1]; [1]Heidelberg/DE, [2]Karlsruhe/DE
*(p.leutz@gmx.de)*

**Purpose:** Dynamic contrast-enhanced perfusion MRI (DCE-MRI) detects perfusion abnormalities in patients with cystic fibrosis (CF) from preschool age related to a reduction in inflow through pulmonary arteries. However, little is known about the alterations of bronchial arterial inflow from the systemic circulation. We hypothesised that in perfusion defect areas a delayed perfusion from bronchial arteries may be observed related to bronchial artery dilatation (BAD).
**Methods or Backround:** Morpho-functional MRI incl. DCE-MRI from 75 patients with CF (mean age 15.3±5.5 years, range 6-29 years) were included. The lungs were segmented on coronal 3D T1 and registered onto DCE-MRI. The arterial input function (AIF) was calculated automatically, in order to quantify pulmonary blood flow (PBF) and mean transit time (MTT). Perfusion defects were classified in percent (QDP). Quantitative perfusion parameters were compared in areas with normal perfusion vs perfusion defects.
**Results or Findings:** Mean QDP was 35.4±23.0%. Mean PBF was 165.2±90.4 ml/100 ml per minute in normal vs 56.9±24.2 ml/100ml per minute in perfusion defect areas (P<0.001). Corresponding MTT was 5.4±1.7 seconds in normal vs 6.9±2.2 seconds in defect areas (P<0.001). 48 CF patients had BAD, and 27 did not show BAD on DCE-MRI. Patients with BAD had higher QDP (46.2±21.3 vs 16.1±9.0, P<0.001), lower PBF (91.9±54.8 vs 178.3±77.4, P<0.001) and tended to have shorter MTT (5.5±1.2 vs 6.2±2.2, P<0.06) for the whole lung. Considering defect areas only, MTT was shorter in patients with BAD vs without (6.3s ±1.8s vs 8.1s ± 2.6s, P<0.001).
**Conclusion:** Lung areas with perfusion defects in the pulmonary arterial phase show delayed perfusion in the systemic arterial phase in patients with CF. Patients with BAD have more severe perfusion defects and reduced PBF. Increased inflow through BAD can potentially be quantified by MTT.
**Limitations:** Ground truth is missing for the validation of quantitative perfusion results.
**Ethics committee approval:** This study was approved by an ethics committee; the vote number is S-211/2011.
**Funding for this study:** This study was funded by the German Center for Lung research (DZL)
This study was supported by grants from the German Federal Ministry of Education and Research (BMBF) (82DZL004A1).
**Author Disclosures:**
Olaf Sommerburg: Nothing to disclose
Britta Nestler: Nothing to disclose
Hans-Ulrich Kauczor: Nothing to disclose
Julian Grolig: Nothing to disclose
Mark Wielpütz: Nothing to disclose
Arnd Koeppe: Nothing to disclose
Simon Triphan: Nothing to disclose
Marcus Mall: Nothing to disclose
Patricia Leutz-Schmidt: Nothing to disclose

### RPS 304-4

**Delta-radiomics features of ADC maps as early predictors of treatment response in lung cancer**

*C. M. Heidt*[1], J. Bohn[1], O. von Stackelberg[1], O. Weinheimer[1], J. Vogel-Claussen[2], F. L. Giesel[3], H-U. Kauczor[1], C. P. Heußel[1], G. Heußel[1]; [1]Heidelberg/DE, [2]Hannover/DE, [3]Düsseldorf/DE

**Purpose:** The purpose of this study was to investigate the feasibility of detecting very early treatment-induced tumour tissue changes in patients with advanced lung adenocarcinoma using diffusion-weighted MRI-derived radiomics features.
**Methods or Backround:** This prospective study included 144 patients receiving TKI (n=64) or chemotherapy (n=80) for lung adenocarcinoma. Patients underwent diffusion-weighted MRI at baseline as well as +1, +7 and +14 days after treatment initiation. Radiomics features quantifying shape, textures and intensities were extracted from tumour ROI segmentations and feature changes over time were analysed for correlation with treatment response (TR) according to RECIST and progression-free survival (PFS).
**Results or Findings:** Out of 14 selected delta-radiomics features, 6 showed significant correlations with PFS or TR. Most significant correlations were found after 14 days. Features quantifying ROI heterogeneity, such as short run emphasis (P = 0.037 (pfs) / 0.006 (tr)), gradient short run emphasis (P = 0.058 (pfs) / 0.012 (tr)) and zone percentage (P = 0.019 (pfs) / 0.013 (tr)) increased in patients with overall better TR. Patients with worse overall response and survival instead showed an increase in features quantifying ROI homogeneity, such as normalised inverse difference (P = 0.012 (pfs)/ 0.043 (tr)). Clustering of these imaging features allows stratification of patients into groups of longer and shorter survival.
**Conclusion:** As early as 2 weeks after initiation of treatment, diffusion MRI of lung adenocarcinoma reveals quantifiable tissue-level insights that correlate well with future treatment (non-)response. Diffusion MRI derived radiomics thus shows promise as an early, radiation-free decision-support to assess efficacy and potentially alter treatment course early.
**Limitations:** The study was performed on a small, single-centre data set, pending validation on external data.
**Ethics committee approval:** Approval was granted by Heidelberg Medical School Ethics committee Ethics Vote S-445/2015.
**Funding for this study:** Funding was provided by the German Center for Lung Research (FKZ 82DZLF14A2).
**Author Disclosures:**
Oliver Weinheimer: Nothing to disclose
Hans-Ulrich Kauczor: Nothing to disclose
Frederik L. Giesel: Nothing to disclose
Oyunbileg von Stackelberg: Nothing to disclose
Jens Vogel-Claussen: Nothing to disclose
Christian Marcel Heidt: Nothing to disclose
Jonas Bohn: Nothing to disclose
Claus Peter Heußel: Nothing to disclose
Gudula Heußel: Nothing to disclose

### RPS 304-5

**3.0T MRI with functional sequences and respiratory motion-resolved compressed sensing reconstruction of free-breathing radial acquisition for immediate response assessment of lung tumour postablation**

*D. Zhao*, L. Liu, X. Ye, k. liu, C. Yiheng, S. Yuanxin, F. Wang, H. Sun; Shanghai/CN

**Purpose:** The purpose of this study was to use serial magnetic resonance imaging (MRI) examinations to observe changes in malignant lung tumours within a short time postablation.
**Methods or Backround:** Patients with primary or metastatic lung tumours eligible for ablation were included in this study. Ablation was performed according to standard procedures. Unenhanced and dynamic contrast-enhanced MRI scans were performed preablation and in one week after ablation. Dynamic imaging were undergone with free-breathing imaging technique combining compressed sensing (CS) and parallel imaging with golden-angle radial sampling instead of conventional breath-hold volumetric interpolated breath-hold examination (VIBE) or liver acquisition volume acceleration (LAVA). At each time point, the signal intensity and parameters of the ablation zone in both perfusion imaging and DCE imaging were examined and analysed.
**Results or Findings:** Using CS technique we obtained T1-weighted imaging with high spatial resolution without requiring patients to hold their breath. A total of 19 nodules in 17 patients were included in the study. Among them, 16 nodules examined parameters of IVIM. The ablation zone in MRI imaging has been shown to have a central area with complete tissue necrosis showed no enhancement (19/19) on ceT1. The value of Ktrans (18/19), D*(10/16), iAUC90(19/19), CER(19/19) were significantly lower in the postablation zone compared to the preablation tumour, which represents decreased blood flow perfusion. The increase in D value (11/16) and ADC value (13/16) indicates that the diffusion of water molecules is less restricted compared to before.
**Conclusion:** Patients who cannot hold their breath autonomously can get high-quality images and stable parameter data by CS technique. The ablative margin and the changes of tumour activity and microvascular perfusion can be analysed via functional MRI images, which can be used to assess immediate response with technical success and ablative margin.
**Limitations:** The limitations of the study are the lack of cases and long-term follow-up data.
**Ethics committee approval:** The study is retrospective.
**Funding for this study:** No funding was provided for this study.
**Author Disclosures:**
Xiaodan Ye: Nothing to disclose
Feihang Wang: Nothing to disclose
Huiyi Sun: Nothing to disclose
Lingxiao Liu: Nothing to disclose
Danyang Zhao: Nothing to disclose
Cai Cai Yiheng: Nothing to disclose
Kai Liu: Nothing to disclose
Sun Yuanxin: Nothing to disclose

### RPS 304-6

**Feasibility of pulmonary MRI for lung nodule detection and quantification using AI-accelerated 3D gradient echo imaging**

*A. W. Marka*[1], M. Steinhardt[1], M. Graf[1], A. Sauter[1], K. Weiss[2], M. Makowski[1], D. C. Karampinos[1], J. Gawlitza[1], S. Ziegelmayer[1]; [1]Munich/DE, [2]Hamburg/DE
*(alexander.marka@gmail.com)*

**Purpose:** Pulmonary MR imaging has made substantial progress in detecting lung nodules through optimisation and developing new sequences in recent years. Nevertheless, the widespread integration of MRI as an alternative method in lung cancer screening is limited either by long scan times or insufficient signal-to-noise ratio.
**Methods or Backround:** In this prospective trial, patients with benign and malignant lung nodules admitted between December 2021 and December 2022 underwent low-dose chest CT and pulmonary MRI using a 3D gradient echo sequence, accelerated with a combination of parallel imaging, compressed sensing, and deep learning. 3D segmentation masks of 192 lung nodules were created in both modalities, and quantitative morphological features were extracted from the segmentation masks. Two readers evaluated all cases in a blinded setting and measured the most extensive lesion. The agreement and relationship between all features in both modalities were assessed.
**Results or Findings:** A total of 38 patients (mean [SD] age, 65±13 years; 19 women [50%]) with 192 pulmonary nodules were included and analysed. The average scan time was 2.5 minutes. The quantitative features showed a strong positive relationship between both modalities (maximum nodule diameter (r = 0.99 95% CI [0.95, 1.02]), surface-area (r = 1.00 95% CI [0.96, 1.04]), nodule volume (r = 1.00 CI [0.96, 1.04]). Mean differences in maximum diameter were 0.1 mm with a 95% CI of 2.09 mm and -1.89 mm. Nodule diameter measurements for the MR sequence showed excellent interrater agreement.
**Conclusion:** Pulmonary MRI with an accelerated 3D gradient echo sequence showed comparable quantitative measurements to low-dose CT. The average examination time of 2.5 minutes is a relevant factor for applying MRI in lung cancer screening as an alternative screening method.
**Limitations:** Our study included only 38 subjects with a consequently limited range of pathologies.
**Ethics committee approval:** This study was approved by the ethics committee of Technical University of Munich School of Medicine.
**Funding for this study:** No funding was received for this study.
**Author Disclosures:**
Andreas Sauter: Nothing to disclose
Markus Graf: Nothing to disclose
Alexander Wolfgang Marka: Nothing to disclose
Sebastian Ziegelmayer: Nothing to disclose
Kilian Weiss: Investigator: Philips GmbH, Hamburg, Germany
Marcus Makowski: Nothing to disclose
Dimitrios C. Karampinos: Nothing to disclose
Joshua Gawlitza: Nothing to disclose
Manuel Steinhardt: Nothing to disclose

### RPS 304-7

**Conjugate gradient reconstruction for pulmonary thin-section MRI with ultra-short TE: capability for acquisition time reduction while maintaining image quality and nodule detection**

*Y. Ohno*[1], K. Yamamoto[2], M. Ikedo[2], M. Yui[2], M. Shinohara[2], Y. Sano[2], H. Nagata[1], T. Ueda[1], Y. Ozawa[1]; [1]Toyoake/JP, [2]Otawara/JP
*(yohno@fujita-hu.ac.jp)*

**Purpose:** The purpose of this study was to compare the capability of conjugate gradient reconstruction (CG-Recon) for acquisition time reduction, image quality and nodule detection performance with conventional grid reconstruction (Grid-Recon) on pulmonary thin-section MRI with ultra-short TE (UTE-MRI) at in vitro and in vivo studies.
**Methods or Backround:** Firstly, a commercially available NEMA phantom was scanned by UTE-MRI sequence under reduction of sampling spoke numbers as follows: original (n=1: UTEoriginal), 1/2 (UTE1/2), 1/4 (UTE1/4) and 1/6 (UTE1/6). Then, each piece UTE-MRI data was reconstructed using both methods. Secondly, 40 patients with lung nodule underwent thin-section CT and UTE-MRI by same sequences, and all UTE-MRI data were also reconstructed with each method. To determine the influence of spoke number reduction and reconstruction method on quantitative image quality at in vitro study, full width at half maximum (FWHM) of phantoms was assessed. At in vivo study, lung signal-to-noise ratio (SNR), overall image quality, artifact and probability for nodule presence were assessed by ROI measurement or 5-point scales. FWHM and SNR were compared among all UTE-MR data by Tukey's HSD test. All qualitative indexes were compared among all UTE-MR data by Wilcoxon's signed rank test.
**Results or Findings:** FWHM, SNR overall image quality and nodule detection probability of each UTEoriginal and UTE1/2 with CG-Recon were significantly better than those of others (P<0.05). Artifact of each UTEoriginal and UTE1/2 with CG-Recon were significantly lower than those of others (P<0.05).
**Conclusion:** Conjugate gradient reconstruction is useful for reducing acquisition time while maintaining image quality and nodule detection performance on pulmonary thin-section MRI with UTE.
**Limitations:** This was a single centre study with a limited study population.
**Ethics committee approval:** This study was approved by the IRB of Fujita Health University Hospital.
**Funding for this study:** A research grant was received from Canon Medical Systems Corporation.
**Author Disclosures:**
Maiko Shinohara: Employee: Canon Medical Systems Corporation
Yoshiyuki Ozawa: Nothing to disclose
Masato Ikedo: Employee: Canon Medical Systems Corporation
Kaori Yamamoto: Employee: Canon Medical Systems Corporation
Yuichiro Sano: Employee: Canon Medical Systems Corporation
Takahiro Ueda: Nothing to disclose
Hiroyuki Nagata: Grant Recipient: Canon Medical Systems Corporation
Masao Yui: Employee: Canon Medical Systems Corporation
Yoshiharu Ohno: Grant Recipient: Canon Medical Systems Corporation

### RPS 304-8

**Longitudinal morpho-functional MRI for the management of incidental pulmonary nodules in patients with COPD: a nationwide multicentre trial**

*L. Zhu*[1], L. Qian[2], H-U. Kauczor[2], M. O. Wielpütz[2]; [1]Shanghai/CN, [2]Heidelberg/DE
*(monica_zhul@163.com)*

**Purpose:** The purpose of this study was to evaluate the capability to accurately diagnose and predict the long-term outcome of incidental pulmonary nodules in heavy smokers by MRI in multicentre trial research.
**Methods or Backround:** Patients who participated in two rounds of same-day MRI and LDCT scans or who had histopathological proofs were included from 16 centres nationwide. The accuracy of MRI in predicting nodule growth and lung cancer was verified by LDCT and histopathological results, and the consistency of MRI and LDCT in judging the outcome of nodules was evaluated. MRI parsimonious and fuller multivariable logistic regression models were constructed.
**Results or Findings:** 248 patients were analysed in the present study out of 567 patients after their first round exam, with 11 patients having had histopathological proofs after the imaging examination. MRI has similar detection accuracy to LDCT for patients with malignant nodules and revealed a substantial intermethod agreement with LDCT on the long-term outcome judgment of incidental nodules based on two rounds of imaging assessment (κ=0.78-0.80). Our MRI full model showed significant improvement in the predictive accuracy of incidental nodule progression over the parsimonious model (AUC 0.91 vs 0.81, P＜0.05), and shows no difference when compared to the LDCT model (P=0.45). This suggests that the MRI full model, which associated sociodemographic, clinical and characteristics based on MRI imaging, has similar potential to LDCT in diagnosing incidental pulmonary nodules as well as in progression prediction in high risk patients.
**Conclusion:** MRI plays a promising role in evaluating and predicting the outcome of incidental nodules in the at-risk population, which may contribute to radiation management and public health improvement.
**Limitations:** Most of the included incidental nodules were small and without surgery or biopsy, so LDCT served as the standard of reference for this part.
**Ethics committee approval:** This research was approved by the ethics committee of the university of Heidelberg.
**Funding for this study:** This work was supported in part by grants from the German Federal Ministry of Education and Research BMBF to the German Center for Lung research (82DZL004A1, 82DZL009B1).
**Author Disclosures:**
Mark O. Wielpütz: Nothing to disclose
Hans-Ulrich Kauczor: Nothing to disclose
Li Qian: Nothing to disclose
Lin Zhu: Nothing to disclose

11:30-12:30 Research Stage 2

### Research Presentation Session: Head and Neck

### RPS 308

### Temporal bone and dental imaging

**Moderator**
B. Kovacsovics; Linköping/SE
*(bea.kovacsovics@gmail.com)*

### RPS 308-2

**Radiologic prevalence and distribution of otic capsule dehiscence in patients with third window syndrome symptoms and a new classification**

A. Yenigun, *Y. B. Polat*, E. Polat, S. Balsak, M. Basoz, S. Tugrul, O. Ozturan; Istanbul/TR
*(yagmurbasakkilinc@gmail.com)*

**Purpose:** The most well-known type of otic capsule dehiscence is Superior Semicircular Canal Dehiscence (SSCD). However, several other types of otic capsule dehiscence affect the semicircular canals, cochlea, and vestibule. Our research aimed to examine the frequency, distribution, and correlation between radiologic otic capsule dehiscence in patients exhibiting symptoms of third window syndrome. Additionally, we introduced a new classification system for semicircular canal dehiscence (SCD).
**Methods or Backround:** In this retrospective study, we included cases who applied to the ENT department of our university hospital between January 2015 and September 2023 and underwent standard reformations and Pöschl plane CT scans due to symptoms suggestive of third window syndrome. A head and neck radiologist and a general radiologist jointly assessed each CT and decided on measurements and classifications.
**Results or Findings:** In the study, 219 patients (438 temporal bones) were examined. Semicircular canal dehiscences (SCD) were categorised into four types: type 0 (no SCD), type 1 (unilateral single canal dehiscence), type 2 (bilateral single canal dehiscence), and type 3 (unilateral multiple localisation dehiscence), and type 4 (bilateral multiple localisation dehiscence). SCD was observed in 69/219 (31.5%) patients; 150 were type 0 (68.5%), 29 type 1 (13.2%), 21 type 2 (9.6%), 15 type 3 (6.9%) and 4 were type 4 (1.8%). Cochlear-fascial dehiscence (CFD) and Vestibular aquaduct-jugular bulbus dehiscence (VA-JBD) were seen in 56/219 (25.6%) and 22/219 (10%) patients, respectively. Type 2 and Type 4 were significantly more frequent in cases with CFD than other types. VA-JBD was seen significantly more in Type 0 cases than in other types.
**Conclusion:** When we examine the otic capsule, we see that the possibility of CFD increases in bilateral SCD cases. The radiologist should evaluate the otic capsule as a whole. Particular attention should be paid to multiple channels, bilateral localisation as well as cochlear and vestibular dehiscences.
**Limitations:** No limitations were identified.
**Ethics committee approval:** This study received institutional review board approval and the reference number is E-54022451-050.05.04-125457.
**Funding for this study:** No funding was received for this study.
**Author Disclosures:**
Yağmur Başak Polat: Nothing to disclose
Selahattin Tugrul: Nothing to disclose
Alper Yenigun: Nothing to disclose
Emre Polat: Nothing to disclose
Meliha Basoz: Nothing to disclose
Serdar Balsak: Nothing to disclose
Orhan Ozturan: Nothing to disclose

### RPS 308-3

**Diagnostic value of cochlear nerve characteristics for occupational noise-induced hearing loss**

*N. Wang*, L. Liu, L. Zhang, Y. Liu, L. Liu, J. Shi; Tianjin/CN

**Purpose:** The aim of this study was to explore the diagnostic value of cochlear nerve characteristics in the diagnosis of occupational noise-induced hearing loss (NIHL).
**Methods or Backround:** This prospectively observational study included patients with NIHL, patients with noise exposure, and healthy volunteers at our hospital between January 2021 and September 2022. Multiplanar reconstruction of bilateral cochlear nerves was performed to extract the cochlear nerves characteristics, including the long diameter, short diameter, average diameter, length-to-diameter ratio, cross-sectional area, and perimeter of the cochlear nerves at three different planes, and the diagnostic value for NIHL were evaluated by area under the receiver operating characteristic curve (AUC).
**Results or Findings:** The NIHL (n=27, 24 males, aged 47.9±6.2 years), noise-exposed (n=41, 34 males, aged 46.9±6.7 years), and healthy control (n=27, 23 males, aged 48.0±6.1 years) groups were included. In the bottom plane, the short diameter (0.55±0.15 vs 0.75±0.16 mm), long diameter (0.83±0.18 vs 1.07±0.23 mm), average diameter (0.69±0.15 vs 0.91±0.18 mm), area (0.39±0.15 vs 0.72±0.21 mm2), and perimeter (2.33±0.48 vs 3.08±0.58 mm) of the NIHL group were significantly lower than those of the noise-exposed group (all P<0.05). The bottom plane area showed the best diagnostic value, with AUC of 0.921 (95% CI: 0.855-0.987).
**Conclusion:** Cochlear nerve characteristics might be helpful in the diagnosis of NIHL, and the bottom plane area showed the best diagnostic value.
**Limitations:** The sample of NIHL patients was relatively small; more patients need to be included in the future to evaluate the diagnostic efficacy of quantitative parameters of the cochlear nerve. Some patients' nerves were too slender, and so were smaller than the resolution of MR 3D-SPACE T2WI sequences. Some volume effects resulted in some layers of the cochlear nerves not being displayed, so those results could not be included in this study.
**Ethics committee approval:** This study was approved by the clinical application project ethics committee of Tianjin occupational diseases precaution and therapeutic hospital.
**Funding for this study:** MR 3D-SPACE T2WI sequences can measure the quantitative parameters of the cochlear nerve in NIHL patients in non-invasive and radiation-free conditions. These parameters can help with clinical differential diagnosis, distinguishing between NIHL patients and noise-exposed patients. The bottom plane had the highest diagnostic efficiency.
**Author Disclosures:**
Ying Liu: Nothing to disclose
Nan Wang: Nothing to disclose
Lei Zhang: Nothing to disclose
Lei Liu: Nothing to disclose
Jun Shi: Nothing to disclose
Lan Liu: Nothing to disclose

### RPS 308-4

**Temporal bone fractures and related complications in patients with cranio-facial trauma: assessment with MDCT in the acute emergency setting**

*M. Pucci*, M. Becker, P. Senn, P. Scolozzi, P-A. A. Poletti, A. Platon; Geneva/CH

**Purpose:** The purpose of this study was to analyse the prevalence and complications of temporal bone fractures in adult and paediatric patients evaluated for cranio-facial trauma in the emergency setting.
**Methods or Backround:** A retrospective blinded analysis was performed based on the CT scans of a series of 294 consecutive adult and paediatric patients with cranio-facial trauma investigated in the emergency setting. Findings between the two populations were compared. The preliminary reports done by the on-call residents were compared with the retrospective analysis by an experienced reader, which served as a reference standard.
**Results or Findings:** CT revealed 126 fractures in 116/294 (39.5%) of patients although fractures were suspected clinically only in 70/294 (23.8%), P<0.05. Fractures were longitudinal, transverse and complex in 69.5%, 10.3 % and 19.8 % of cases, respectively. Involvement of the tympanic cavity, external auditory canal, ossicular chain, facial nerve canal, and otic capsule was present in 54%, 72.2%, 8.7%, 6.3% and 4.8%, respectively. Injuries of the venous sinuses and carotid canal were seen in 18.3% and 17.5% of temporal bone fractures. Vascular injuries were more common in children than in adults (31.8% vs 15.7%, P<0.05). 79.5% of patients with temporal bone fractures had both brain injuries and fractures of the facial bones and cranial vault. Compared to the experienced reader, the sensitivity of the on-call residents was 103/116 (88.7%).
**Conclusion:** Temporal bone fractures and related complications in particular vascular injuries are common in patients with cranio-facial trauma and need to be thoroughly looked for, especially in children.
**Limitations:** This is a retrospective study. Possible underestimation of the true prevalence of temporal bone trauma due to the selection of patients based on the PACS records.
There was a lack of analysis of the initial and long-term clinical assessment.
**Ethics committee approval:** The institutional Ethics Committee approved this study.
**Funding for this study:** No funding was received for this study.
**Author Disclosures:**
Alexandra Platon: Nothing to disclose
Paolo Scolozzi: Nothing to disclose
Minerva Becker: Nothing to disclose
Pierre-Alexandre Aloïs Poletti: Nothing to disclose
Pascal Senn: Nothing to disclose
Marcella Pucci: Nothing to disclose

### RPS 308-5

**Deep learning reconstruction algorithm with ultra-high resolution CT of the temporal bone: initial experience**

A. Beysang[1], N. Villani[1], F. Boubaker[1], *U. Puel*[1], A. Blum-Moyse[1], P. A. Teixeira[1], K. Haioun[2], C. Parietti[1], R. Gillet[1]; [1]Nancy/FR, [2]Otawara/JP

**Purpose:** The purpose of this study was to evaluate the image quality and clinical acceptance of a deep learning reconstruction (DLR) algorithm compared to traditional iterative reconstruction (IR) and simulated normal resolution (NR) reconstruction algorithms.
**Methods or Backround:** CT acquisitions were performed using an ultra-high resolution CT device with a Mercury 4 phantom at three dose levels (25, 40, 51 mGy) and on 13 temporal bones at our routine dose level (about 500 mGy.cm). Images were reconstructed with two IR algorithms (model-based (MBIR) and hybrid (HIR)), one DLR algorithm with 0.25 mm slice thickness, and one simulated NR algorithm. In phantom acquisition, detectability was compared. Three radiologists performed quantitative (stapes footplate and superstructure thicknesses and lengths) and qualitative (posterior canaliculus, tympanic chorda tympani, stapes footplate and superstructure depiction) measurements in human temporal bones. Mean noise values in the external auditory canal air and lateral semi-circular canal bony island were compared.
**Results or Findings:** Using DLR, detectability was superior to other reconstruction algorithms at the three dose levels. All thicknesses were significantly superior using NR compared to DLR and IR algorithms, whereas lengths did not differ. DLR and MBIR were superior to NR and HIR for posterior canaliculus and stapes superstructure and footplate depiction. DLR was superior to all other algorithms for tympanic chorda tympani depiction. Compared to NR, there was twice as much noise in the air using HIR, but DLR and MBIR yielded significantly lower noise values. Bony noise values were lower using DLR and MBIR than with NR and HIR, which provided equivalent values.
**Conclusion:** DLR yielded a gain in objective measurement, closer to anatomical data, and in noise with the best clinical acceptance among the evaluated reconstruction algorithms.
**Limitations:** There was no surgical confirmation and the number of patients was small.
**Ethics committee approval:** This study was approved by the ethics committee of CHRU de Nancy.
**Funding for this study:** No funding was received for this study.
**Author Disclosures:**
Achille Beysang: Nothing to disclose
Fatma Boubaker: Nothing to disclose
Pedro Augusto Teixeira: Nothing to disclose
Romain Gillet: Nothing to disclose
Karim Haioun: Nothing to disclose
Nicolas Villani: Nothing to disclose
Ulysse Puel: Nothing to disclose
Cécile Parietti: Nothing to disclose
Alain Blum-Moyse: Nothing to disclose

### RPS 308-6

**Synthetic CT images of temporal, sinonasal and facial bones from MRI**

*M. D. Van der Veen*[1], T. Van der Velden[2], P. R. Seevinck[2], J. Kuijer[1], B. Schulten[1], G. Adriaensen[1], T. Goderie[1], P. Merkus[1], B. Jasperse[1]; [1]Amsterdam/NL, [2]Utrecht/NL
*(m.d.vanderveen@amsterdamumc.nl)*

**Purpose:** Diagnostic evaluation and surgical planning of the head frequently require visualisation of both soft and bony tissues. Currently, this means a CT and MRI have to be obtained separately and then registered to combine the information from the two images. The objective of this study is to train and evaluate a machine learning algorithm to generate synthetic bone CT images from MRI of the head.
**Methods or Backround:** Paired 3T MRI (Philips, Siemens, GE) and CT scans of the head were used to train a vendor agnostic machine learning algorithm to generate synthetic CT images from the MRI data. MRI scans of patients not included in algorithm training were used to create synthetic CT images, which could be compared to their corresponding true CT images. A technical voxelwise comparison between synthetic CT and true CT was performed to quantify morphological and radiodensity accuracy. To clinically evaluate the algorithm, six surgeons and two radiologists will evaluate the visibility of clinically relevant landmarks for three areas (temporal, sinonasal and facial bones) on both synthetic CT and true CT on a 4-point Likert scale.
**Results or Findings:** Voxelwise comparison showed a surface distance error of 0.38 ± 0.37 mm for the skull and a mean radiodensity error of 4 ± 44 HU, with a correlation coefficient of 0.79 ± 0.08. In-depth clinical analysis of the images is currently ongoing and will be presented during the ECR 2024.
**Conclusion:** Generating synthetic CT images of the head from MRI is feasible, allowing inherently registered and efficient visualisation of soft and bony tissues using a single, radiation-free imaging modality.
**Limitations:** A limitation of the study is suboptimal blinding during evaluation of the scans, as synthetic CT and true CT can be visually distinguished by experienced physicians.
**Ethics committee approval:** The study was approved by the institutional review board of Amsterdam UMC, location VUMC (study number: 2022.0234). Written informed consent was obtained from all participants.
**Funding for this study:** Funding was provided by Amsterdam UMC, innovation impulse and by the research institute Amsterdam Public Health, within their programme Quality of Care.
**Author Disclosures:**
Gwijde Adriaensen: Nothing to disclose
Tijl Van der Velden: Employee: MRIguidance BV, Utrecht, The Netherlands
Paul Merkus: Nothing to disclose
Joost Kuijer: Nothing to disclose
Marlise Daniëlla Van der Veen: Nothing to disclose
Bert Schulten: Nothing to disclose
Thadé Goderie: Nothing to disclose
Peter Roland Seevinck: Founder: MRIguidance BV, Utrecht, The Netherlands
Bas Jasperse: Nothing to disclose

### RPS 308-7

**Dental imaging in clinical photon-counting CT at a quarter of DVT dose**

*S. Sawall*[1], J. Maier[1], S. Sen[2], H. Gehrig[1], T-S. Kim[1], H-P. Schlemmer[1], S. O. Schönberg[3], M. Kachelrieß[1], M. Rütters[1]; [1]Heidelberg/DE, [2]Kiel/DE, [3]Mannheim/DE
*(stefan.sawall@dkfz.de)*

**Purpose:** The main objective of this study was to investigate the image quality of a low-dose dental imaging acquisition protocol in the first clinical photon-counting computed tomography (PCCT) system in comparison to a normal-dose acquisition in a digital volume tomography (DVT) system.
**Methods or Backround:** Clinical PCCT systems offer an increased spatial resolution compared to previous generations of clinical systems. Its spatial resolution is in the order of dental DVT systems. Resolution-matched acquisitions of ten porcine jaws were performed in a PCCT (Naeotom Alpha, Siemens Healthineers) and in a DVT (Orthophos XL, Dentsply Sirona). PCCT images were acquired with 90 kV at a dose of 1 mGy CTDI16 cm. DVT used 85 kV at 4 mGy. Image reconstruction was performed using the standard algorithms of each system to a voxel size of 160×160×200 µm. The dose-normalised contrast-to-noise ratio (CNRD) was measured between dentine and enamel as well as dentine and bone. Two experienced readers qualitatively evaluated overall diagnostic quality of images and quality of relevant anatomical structures such as root channels and dentine. Reproducibility was assessed using the intraclass correlation coefficient (ICC).
**Results or Findings:** CNRD is significantly higher in all PCCT acquisitions. In particular, CNRD is 37% higher for the contrast dentine-enamel and 31% higher for the dentine-bone contrast ($p<0.05$). Overall diagnostic image quality was higher for PCCT over DVT ($p<0.02$ and $p<0.04$ for readers 1 and 2). Quality scores for considered anatomical structures were higher in PCCT compared to DVT (all $p<0.05$). Inter- and intrareader reproducibility were acceptable (all ICC>0.75).
**Conclusion:** PCCT provides an increased image quality over DVT even at far lower dose levels.
**Limitations:** The study is limited to porcine ex-vivo samples since multiple measurements are required. The resulting intersection lengths might not accurately reflect the clinical case of a human patient.
**Ethics committee approval:** No approval was required for this study since the used porcine jaws are not considered an animal experiment.
**Funding for this study:** Not applicable for this study.
**Author Disclosures:**
Ti-Sun Kim: Nothing to disclose
Stefan Oswald Schönberg: Nothing to disclose
Holger Gehrig: Nothing to disclose
Maurice Rütters: Nothing to disclose
Sinan Sen: Nothing to disclose
Heinz-Peter Schlemmer: Nothing to disclose
Marc Kachelrieß: Nothing to disclose
Stefan Sawall: Nothing to disclose
Joscha Maier: Nothing to disclose

### RPS 308-8

**Assessment of dental pulp by T2 mapping in vivo is influenced by age**

*K. Cankar*, A. Golez, A. Tenyi, K. Romaric, T. Robida, J. Vidmar; Ljubljana/SI
*(ksenija.cankar@mf.uni-lj.si)*

**Purpose:** The cone beam computed tomography (CBCT) provide only information on hard dental tissues. This in vivo study tested whether standard 3T clinical MRI system can be used to quantify the dental pulp state using the T2 mapping method.
**Methods or Backround:** Multi-echo-spin-echo (MESE) T2-weighted sequence in a single sagittal slice and with a field of view that covered the pulp chamber was employed. The teeth of the 15 subjects were divided in two groups according to their age. In the first group, children with the age between 11 and 15 were included (mean age 12.6±1.3 years) and in the second group were adults with the age between 24 and 48 (mean age 33.8±8.3 years). All T2 maps were calculated from the images of single-rooted premolar teeth. In the present study, only intact teeth without caries were included.
**Results or Findings:** In the group of adult's teeth, there were statistically significantly higher T2 relaxation times in dental pulp (125.6±19.1 ms) compared to T2 relaxation times obtained in children' teeth (111.9±11.2 ms) (p=0.022). In contrast, in the group of adult's teeth there was lower variability of dental pulp tissue relaxation times (35.0±4.9 ms) compared to the pulp tissue in children's teeth (44.6±8.4 ms) (p=0.0006).
**Conclusion:** An increase in the T2 relaxation times and a decrease in their variability in the adults could be attributed to lower cell to water ratio and increased homogeneity in the pulp tissue. The study confirmed that in vivo T2 mapping of dental pulp can be used to reliably quantify the dental pulp state. In healthy subjects, dental pulp assessment is strongly influenced by age.
**Limitations:** The teeth were not all from different subjects to maximize the number of teeth in the same field of view.
**Ethics committee approval:** The study has been independently reviewed and approved by the Ethical Committee of the National Ministry of Health (Approval number 0120-659/2016/6).
**Funding for this study:** This study was funded by the Ministry of Higher Education, Science and Technology, Slovenia: Grant No.P3-0019.
**Author Disclosures:**
Katja Romaric: Nothing to disclose
Ana Tenyi: Nothing to disclose
Ksenija Cankar: Nothing to disclose
Jernej Vidmar: Nothing to disclose
Tina Robida: Nothing to disclose
Aljaz Golez: Nothing to disclose

**11:30-12:30** **Research Stage 3**

### Research Presentation Session: Paediatric

### RPS 312

### Foetal and paediatric thoraco-abdominal imaging

**Moderator**
R. Carneiro; Lisbon/PT
*(rita.ccarneiro@gmail.com)*

### RPS 312-2

**Fractal analysis of perfusion in neuroblastoma using MRI predicts volumetric tumour response and outcome after induction chemotherapy**
*F. Michallek*, M. Dewey, A. Eggert, T. M. Thole-Kliesch; Berlin/DE
*(florian.michallek@charite.de)*

**Purpose:** Angiogenesis is a hallmark of cancer, however, evidence on the role of tumour perfusion assessment in paediatric patients with neuroblastoma is sparse, although contrast-enhanced magnetic resonance imaging (MRI) is routinely used in neuroblastoma patients. Fractal analysis allows to characterise perfusion patterns on routine contrast-enhanced MRI, which has potential as a non-invasive imaging biomarker for predicting treatment response and outcome by providing information on vascular changes during induction chemotherapy.
**Methods or Backround:** In a national multi-centre setting (30 centres), we retrospectively performed fractal analysis of contrast-enhanced, fat-saturated, T1-weighted MRI. Patients underwent MRI at the timepoint of diagnosis (TP1) and prior to surgical tumour resection (TP2). At both timepoints, fractal analysis was performed to quantify complexity of the tumour perfusion pattern by fractal dimension (FD). At TP1, we investigated, whether fractal analysis allows to predict local tumour response. At TP2, we evaluated prognostic implications of fractal analysis for predicting event-free-survival (EFS). Additionally, we applied fractal analysis to CD34-immunohistochemistry in a subset of patients to correlate imaging findings with underlying vascular structure. We adhered to the STARD guideline.
**Results or Findings:** We included n=73 patients in separate discovery (n=36; single-centre) and validation cohorts (n=37; 29 centres). At TP1, fractal analysis predicted relative volumetric tumour response with mean bias -0.05 (limits of agreement: -0.35–0.25) using Bland-Altman analysis. Areas with low FD at TP1 showed high tumour response, while high FD-areas tended to persist. At TP2, fractal analysis separated patients with and without favourable EFS in Kaplan-Meier-Analysis (p=0.0019) in tumours lacking MYCN-amplification. In CD34-immunohistochemistry, FD increased significantly after induction chemotherapy (FD-TP1: 1.23±0.09, FD-TP2: 1.44±0.07, p<0.001).
**Conclusion:** In a retrospective multi-centre setting, fractal analysis of perfusion predicted volumetric response and outcome after induction chemotherapy in patients with neuroblastoma.
**Limitations:** Prospective validation is lacking making this a limitation of the study.
**Ethics committee approval:** Neuroblastoma Registry 2016 German Clinical Trial Register with the DRKS-ID: DRKS00023442; Ethics committee number: 16-432.
**Funding for this study:** No funding was obtained for this study.
**Author Disclosures:**
Angelika Eggert: Nothing to disclose
Theresa M. Thole-Kliesch: Nothing to disclose
Marc Dewey: Research/Grant Support: German Research Foundation (DFG) in the Heisenberg Program (DE 1361/14-1, DFG project number: 213705389) Patent Holder: fractal analysis of perfusion imaging, jointly with Florian Michallek (EP3350773 and USPTO 2021 10,991,109; both granted); Other: M.D. is European Society of Radiology (ESR) Publications Chair (2022-2025); and the opinions expressed in this article are the author's own and do not represent the view of ESR. Per the guiding principles of ESR, the work as Publications Chair is on a voluntary basis and only remuneration of travel expenses occurs. He is also the editor of Cardiac CT, published by Springer Nature, and offers hands-on courses on CT imaging (www.ct-kurs.de). Research/Grant Support: EU-FP7 Framework Program (DISCHARGE EU FP EC-GA 603266); Research/Grant Support: graduate program on quantitative biomedical imaging (BIOQIC, GRK 2260/1, DFG project number: 289347353), for fractal analysis of myocardial perfusion (DE 1361/18-1, DFG project number: 392304398), the DFG Priority Programme Radiomics (DFG project number: 402688427) for the investigation of coronary plaque and coronary flow (DE 1361/19-1 [DFG project number: 428222922] and DE 1361/20-1 [DFG project number: 428223139] in SPP 2177/1); Research/Grant Support: Berlin University Alliance (GC_SC_PC 27) and from the Digital Health Accelerator of the Berlin Institute of Health Research/Grant Support: the GUIDE-IT project on data sharing of medical imaging trials (DE 1361/24-1 [DFG project number: 495697118]), the Quantitative Cardiovascular Imaging (QCI) meeting (DE 1361/22-1), and the Future of Medical Imaging meeting (DE 1361/28-1).
Florian Michallek: Research/Grant Support: German Research Foundation (DFG) for fractal analysis of myocardial perfusion (MI 2272/1-1, DFG project number: 392304398); Patent Holder: fractal analysis of perfusion imaging (EP3350773 and USPTO 2021 10,991,109; both granted; jointly with Marc Dewey)

### RPS 312-3

**Feasibility and diagnostic accuracy of early postoperative MRI after resection of neuroblastic tumours**
*M. Chaika*, M. Esser, P. Krumm, R. Guglielmi, S. Gassenmaier, C. Urla, S. Warmann, J. F. Schäfer; Tübingen/DE
*(maryanna.chaika@med.uni-tuebingen.de)*

**Purpose:** In standard MRI, it may be problematic to differentiate residual tumour (RT) from scar, reactive changes, or recurrence after three months. The aim of this study was to evaluate a short MRI protocol performed in the early postoperative period.
**Methods or Backround:** The inclusion criteria were: histologically confirmed neuroblastic tumour, resection by the reference surgery in our centre, early postoperative MRI and MRI preoperatively, and standardised MRI Protocol. The MRI protocol included the following sequences: T1w vibe Dixon before and after contrast, T2w with fat saturation and DWI with the calculation of the ADC map. The analysis was performed by 3 independent readers (resident/adults radiologist/board certified pediatric radiologist) using a 4-points Likert-scale . A multimodal reference standard was determined by: F/U imaging, consensus tumour-board, and consensus between senior radiologist and surgeon.
**Results or Findings:** Thirty-nine patients with a median of 46 age months (5-177). MRI was performed in the mean 8(+/- 5) days after surgery. RT was found in 13 patients by MRI and confirmed by the reference standard, with a typical location at the mesenteric root and retrocrural. 4 RTs were expected by the surgeons with a median volume of 19 ml (1-34 ml), and nine tumours were unexpected with a median volume of 1 ml (0.25-7). Sensitivity, specificity, and accuracy (reader) were 77,54, and 70 %(Resident), 81,85, and 82 %(Adult Radiologist), 92,92, and 92 %(Pediatric Radiologist). Reading the postoperative MRI alone, the diagnostic performance of the paediatric radiologist were 88,74, and 84%.
**Conclusion:** Early MRI protocol is feasible for determination of residual tumour. Experience in pediatric imaging is crucial to achieving high diagnostic precision. Reading the preoperative MRI improves diagnostic accuracy.
**Limitations:** Small sample size. The results are preliminary and need to be confirmed by follow-up studies.
**Ethics committee approval:** The local institutional review board (Ethics Committee at the Medical Faculty of the Eberhard Karls University, Tuebingen, Germany) approved this retrospective, monocentric study.
**Funding for this study:** No funding from any specific grant was received for this study.

**Author Disclosures:**
Patrick Krumm: Nothing to disclose
Maryanna Chaika: Nothing to disclose
Riccardo Guglielmi: Nothing to disclose
Michael Esser: Nothing to disclose
Cristian Urla: Nothing to disclose
Jürgen F. Schäfer: Nothing to disclose
Steven Warmann: Nothing to disclose
Sebastian Gassenmaier: Nothing to disclose

### RPS 312-4

**Neonatal pulmonary MRI at term-equivalent age for defining bronchopulmonary dysplasia severity and its relationship with short-term respiratory outcomes**

A. M. Munari[1], *G. Folco*[1], C. B. Monti[1], F. Rizzetto[1], N. A. R. Panarisi[2], S. Zirpoli[1]; [1]Milan/IT, [2]Assago/IT
*(gianluca.folco@unimi.it)*

**Purpose:** This prospective study aims to demonstrate that neonatal pulmonary magnetic resonance imaging (MRI), using commercially available sequences, can assess lung parenchymal injury associated with bronchopulmonary dysplasia (BPD) and may predict short-term respiratory outcomes.
**Methods or Backround:** Pulmonary MRI, using turbo spin echo and gradient echo sequences during natural sleep, was performed in premature infants who underwent routine brain MRI at term-equivalent age. Lung MRIs were scored using the modified Ochiai scoring system and a new proposed score, the Bronchopulmonary Dysplasia Magnetic Resonance Index (BPDMRI), based on four hyperaeration (hyperexpansion, emphysema, cysts, mosaic pattern) and four parenchymal variables (triangular subpleural opacities, fibrous stripes and bands, edema and atelectasis, distortion of bronchovascular bundles). Both scores were correlated with two short-term respiratory outcomes: the length of mechanical ventilation and the length of any respiratory support until discharge.
**Results or Findings:** The study included 25 premature infants (9 with BPD, 36%). Pulmonary MRI showed a wide variety in appearance of pulmonary parenchyma among BPD patients and revealed significant structural differences across the range of MRI scores. Both the modified Ochiai score and the BPDMRI resulted positively correlated to the length of mechanical ventilation (r=0.828, p<0.001; r=0.900, p<0.001) and the length of any respiratory support until discharge (r=0,897, p<0.001; r=0.953, p<0.001). In the severe disease group, the length of respiratory support showed a smaller IQR when related with the BPDMRI rather than when related with the clinical definition.
**Conclusion:** Pulmonary MRI identified lung parenchymal abnormalities associated with BPD, showing a strong correlation with the length of mechanical ventilation and the length of any respiratory support until discharge.
**Limitations:** Single centre, retrospective study is the limitation of this study.
**Ethics committee approval:** This study was ethically approved.
**Funding for this study:** No funding was received for this study.
**Author Disclosures:**
Francesco Rizzetto: Nothing to disclose
Salvatore Zirpoli: Nothing to disclose
Alice Marianna Munari: Nothing to disclose
Caterina Beatrice Monti: Nothing to disclose
Gianluca Folco: Nothing to disclose
Nicol Antonina Rita Panarisi: Nothing to disclose

### RPS 312-5

**Applicability of the O-RADS scoring system in children and adolescents: results of a preliminary retrospective monocentric study**

*E. Mercanzin*[1], P. Clauser[2], V. Rizzo[3], S. Sironi[1], A. Hojreh[2]; [1]Bergamo/IT, [2]Vienna/AT, [3]Maglie/IT

**Purpose:** The study aims to assess the accuracy of the Ovarian-Adnexal Reporting & Data System (O-RADS) classification on ultrasound (US) and magnetic resonance imaging (MRI) in paediatric patients.
**Methods or Backround:** In this single-centre retrospective study, all US and MRI studies performed between 2005 and 2022 for suspected ovarian lesions in patients < 18 years were included. Histopathology was considered as standard of reference. Two readers in consensus evaluated imaging characteristics as defined by the O-RADS (features, size, wall thickness, ascites). Each lesion was then assigned an O-RADS category. Chi-square test was used, sensitivity, specificity, and accuracy were calculated using a cutoff >O-RADS 3, and compared.
**Results or Findings:** Included in this study were 95 patients (mean age 12,6, range 0-18). Histology found 45 non-neoplastic cystic masses (47.4%), 47 benign neoplasms (49.5%) and 3 malignant neoplasms (3.2%). MRI was available in 80 cases and US in 72 cases. No significant difference in the US features was found between malignant and benign lesions (p>0.2). On MRI a significant difference was found between benign and malignant masses in terms of lesion diameter (p=0.02) and enhancement (p=0.025). MRI correctly classified all malignant cases (3/3, 100%), while US only 1/2 (50%). Specificity was comparable between modalities (respectively 89% vs 88%, p<0.0001). Accuracy was higher for MRI (87% vs 90%, p<0.0001).
**Conclusion:** Our results showed that the MRI O-RADS demonstrated a high diagnostic accuracy and can be applied in paediatric patients. In this population, a higher specificity would be desirable to reduce false positives and unnecessary follow up or surgery. A lower accuracy was found for US.
**Limitations:** This was a retrospective study, its limitations are the retrospective evaluation of the US images, low number of malignant lesions.
**Ethics committee approval:** Ethics committee: Medical University of Vienna 2057/2017 approved this study.
**Funding for this study:** No research funding was received for this study.
**Author Disclosures:**
Sandro Sironi: Nothing to disclose
Elisa Mercanzin: Nothing to disclose
Veronica Rizzo: Nothing to disclose
Azadeh Hojreh: Nothing to disclose
Paola Clauser: Speaker: Siemens

### RPS 312-6

**The distal colostogram for anorectal malformation: considerations in low- and middle-income countries**

*M. Leiderer*; Passau/DE

**Purpose:** The purpose of this study was to evaluate imaging challenges of anorectal malformations (ARM) in older male children and those with prior loop colostomy.
**Methods or Backround:** Treatment of ARM in low- and middle-income countries suffers from limited resources in paediatric surgery. Children undergo colostomy in the newborn period, and definitive treatment is delayed. Consequently, children are older than the recommended age for definitive reconstruction at time of surgery. A loop colostomy adds additional technical challenges. We reviewed all colostograms obtained for patients with ARM presenting for definitive repair at a specialised hospital in Uganda, between April and August 2023.
**Results or Findings:** Twenty-three patients were included, twelve male, of which seven with suspicion of rectourinary fistula. Three of these were up to 12 m (5 m, 9 m, 12 m), in all of which the fistula tract was visualised. No fistula tract was visualised in older patients (22 m, 3 y, 4y , 7 y), however, in three of these the fistula origin was seen as a small anterior tapering of the rectal pouch. In one patient with no sign of fistula the absence of a fistula was confirmed during surgery. Fourteen patients (61%) had a fecaloma in the distal pouch.
**Conclusion:** For all the patients with suspected genitourinary fistula older than 12 m the fistula did not opacify, an age group never expressly reported previously. Possible explanations are occlusion by accumulated feces and mucus or through colonic distention; and ability of older children to react to abdominal pressure by tensing the striated perirectal muscular complex. Fecaloma because of loop colostomy was common, leading to chronic rectal distension associated with a poorer functional outcome and increased infection risk.
**Limitations:** The small number of cases warrants further research with a larger case number. Also, no follow-up is available yet to assess functional outcome, making these the limitations of this study.
**Ethics committee approval:** The requirement for informed consent was waived for this retrospective study.
**Funding for this study:** No funding was received.
**Author Disclosures:**
Miriam Leiderer: Nothing to disclose

### RPS 312-7

**Evaluation of perfusion impairment in Kawasaki disease using fully quantitative cardiovascular magnetic resonance myocardial perfusion: correlation with left ventricular remodelling**

*Z. Zhou*, L-y. Wen, S. Azhe, L. Hu, Y-K. Guo; Chengdu/CN
*(zhouzhongqin29@163.com)*

**Purpose:** This study aims to assess perfusion impairment in Kawasaki disease (KD) using fully quantitative cardiovascular magnetic resonance (CMR) myocardial perfusion and correlates it with left ventricular (LV) remodeling.
**Methods or Backround:** Eight-seven children (60 males, 7.49±2.15 years) diagnosed with KD and 33 controls (22 males, 8.22±2.81 years) were enrolled to complete CMR. A dual-bolus protocol was used to acquire perfusion images. Myocardial blood flow (MBF) was acquired through post processing and corrected for the heart rate-blood pressure product (MBF corrected [MBFcor]). The diameter of coronary artery in patients with KD was standardized by Z score. Z score≥5 represented mid-giant coronary aneurysm.
**Results or Findings:** MBFcor was lower in patients with KD than that of controls (2.23±0.50 vs 2.48±0.67 mL/g/min, p=0.031), especially in patients at acute phase (2.08±0.54 vs 2.48±0.67 mL/g/min, p=0.026). The MBFcor in patients with Z score≥5 decreased when compared with the other patients with KD and controls (2.04±0.54 vs 2.34±0.44 vs 2.48±0.57 mL/g/min, p=0.005).

Wednesday

MBFcor was correlated with Z score in patients with KD (r=-0.305, p=0.004). Multivariate analysis revealed that age, acute phase and Z score was correlated with MBFcor (β=-0.238, p=0.019; β=-0.217, p=0.031; β=-0.290, p=0.005, respectively). Radial peak strain (PS), the absolute value of circumference PS, LV mass index and LV remodeling index was correlated with MBFcor (r=0.233, p=0.035; r=0.259, p=0.019; r=-0.268, p=0.012; r=-0.391, p<0.001, respectively). Multivariate analysis revealed that acute phase and MBFcor was associated with LV remodeling index (β=0.290, p=0.003; β=-0.235, p=0.025, respectively)
**Conclusion:** Fully quantitative CMR myocardial perfusion can evaluate myocardial perfusion impairment in children with KD. The perfusion impairment in KD is related to acute disease and coronary artery dilation and may cause abnormalities in LV function and LV remodeling.
**Limitations:** No limitations were identified.
**Ethics committee approval:** This study was approved by the Medical Ethics Committee of our hospital.
**Funding for this study:** No funding was received for this study.
**Author Disclosures:**
Zhongqin Zhou: Nothing to disclose
Ying-Kun Guo: Nothing to disclose
Lei Hu: Nothing to disclose
Ling-yi Wen: Nothing to disclose
Shiganmo Azhe: Nothing to disclose

**11:30-12:30** **Research Stage 4**

### Research Presentation Session: Physics in Medical Imaging

### RPS 313

### What lies in store for us in CT or MR imaging

**Moderator**
J. S. Andersson; Umea/SE
*(jonas.s.andersson@regionvasterbotten.se)*

### RPS 313-2

**Multi-spot source cone-beam CT toward quantitative image evaluations**
*A. Sarno*[1], P. Cardarelli[2], P. Mauriello[1], A. Minopoli[3], M. C. Mollo[1], S. Pardi[1], G. Paternò[2], M. Pugliese[1], R. de Asmundis[1]; [1]Naples/IT, [2]Ferrara/IT, [3]Pisa/IT
*(sarno@na.infn.it)*

**Purpose:** The purpose of this study was to investigate the potential of a cone beam CT (CBCT) scanner equipped with a multi-spot x-ray source for reduction of scatter and cone-beam artefacts toward quantitative evaluations.
**Methods or Backround:** The simulated study mimicked a CBCT scanner with a multi-spot x-ray source. This configuration consists in several focal spots arranged in a linear array parallel to the rotation axis and permits to acquire projections in an unconventional scanning trajectory (ExoCT). A specific beam collimation is employed for scatter reduction and a controller operates the several spots in order to have a non-conventional trajectory of the source during the gantry rotation for tightening the FOV sampling. In-silico studies were performed to evaluate the conspicuity improvements of selected details and the impact on accuracy of estimates of materials' attenuation coefficients/HU.
**Results or Findings:** In a test conducted using a Defrise phantom, which alternates PMMA slabs and 1-mm air gaps, ExoCT configuration demonstrated that the conspicuity of such gaps is kept constant across the reconstructed volume, differently from what happened in CBCT where it reduces moving along the axial direction. The use of an ExoCT configuration with 10 focal points parallel to the rotation axis permitted to reduce the scatter-to-primary ratio of 86%. On the other hand, three spots were sufficient to increase the estimation accuracy of the attenuation coefficients of a PMMA phantom up to 10%.
**Conclusion:** We investigated an innovative solution for updating the CBCT classical configuration toward quantitative evaluations of the images. Such a solution showed – unlike the results attainable with CBCT – to preserve image conspicuity over the entire reconstructed FOV and permitted to improve the accuracy in the estimates of the sample attenuation coefficients by 10%.
**Limitations:** The study was conducted in-silico making it a limitation.
**Ethics committee approval:** No ethical approval was required for this study.
**Funding for this study:** This work rose from qCT project funded by the Italian Ministry of University and Research (CUP E53D23012420006)
**Author Disclosures:**
Gianfranco Paternò: Nothing to disclose
Riccardo de Asmundis: Nothing to disclose
Maximilian Carlos Mollo: Nothing to disclose
Silvio Pardi: Nothing to disclose
Antonio Minopoli: Nothing to disclose
Mariagabriella Pugliese: Nothing to disclose
Paolo Mauriello: Nothing to disclose
Antonio Sarno: Nothing to disclose
Paolo Cardarelli: Nothing to disclose

### RPS 313-3

**Image domain deep-learning low-dose CT simulator**
*S. A. M. Tunissen*[1], N. Moriakov[2], M. Mikerov[1], E. J. Smit[1], I. Sechopoulos[1], J. Teuwen[2]; [1]Nijmegen/NL, [2]Amsterdam/NL

**Purpose:** The main goal of this study was to develop an image-domain method to generate low-dose CT (LDCT) scans from clinical-dose CT (CDCT) scans reconstructed with non-linear reconstruction methods, e.g., iterative reconstruction, without access to the reconstruction method.
**Methods or Backround:** The method consists of three convolutional neural networks (CNNs), to (i) denoise the CDCT to obtain a noiseless estimate, (ii) estimate the standard deviation (σ) in each voxel of the LDCT, and, (iii) estimate the local noise power spectra (NPS) in the LDCT. White noise is transformed into LDCT noise, using the σ and NPS estimates, and added to the noiseless estimate to obtain the simulated LDCT. For training, paired brain LDCTs and CDCTs were used, reconstructed with iterative reconstruction (AIDR3D, Canon Medical Systems), divided into training/validation/test sets (251/25/50 cases). Each CNN was evaluated on the test set by (i) determining the decrease in standard deviation in the cerebrospinal fluid (CSF), (ii) comparing the estimated and actual σ of the LDCT noise inside the skull, and, (iii) comparing the NPS of the generated and actual LDCT noise.
**Results or Findings:** The denoising network decreased the σ by a median (IQR) factor of 1.71 (1.61-1.95). The median (IQR) difference between estimated and actual σ was +0.1 (-0.2-+0.3) HU. The median (IQR) error of the radially-averaged 2D NPS of the simulated and actual LDCT noise inside the skull was 13.9% (11.9%-15.9%).
**Conclusion:** The proposed method allows for generation of LDCT from CDCT scans without access to the reconstruction algorithm and works fully in the image domain. Making LDCTs more available for testing new applications without patient radiation.
**Limitations:** The CNNs of the pipeline need to be retrained for different reconstruction methods. The method is tested only on brain scans, however it is not anatomy specific.
**Ethics committee approval:** Not applicable for this study.
**Funding for this study:** The funding for this study was funded by the Health Holland, Canon Medical Systems.
**Author Disclosures:**
Sjoerd A. M. Tunissen: Nothing to disclose
Nikita Moriakov: Nothing to disclose
Jonas Teuwen: Shareholder: shareholder Ellogon.AI.
Mikhail Mikerov: Nothing to disclose
Ioannis Sechopoulos: Research/Grant Support: Volpara Healthcare; Research/Grant Support: ScreenPoint Medical; Research/Grant Support: Lunit; Research/Grant Support: Siemens Healthcare; Speaker: Siemens Healthcare; Research/Grant Support: Sectra Benelux; Research/Grant Support: Canon Medical Systems; Research/Grant Support: iCAD
Ewoud J. Smit: Patent Holder: Radboud University Medical Center; Speaker: Canon Medical Systems

### RPS 313-4

**Anthropomophic 3D printed lung vessel phantoms combined with lung nodules for thorax CT imaging clinical performance evaluation**
*I. Hernandez-Giron*[1], P. McHale[1], A. Gaffney[1], B. Snow[1], J. Egan[1], C. D'Helft[1], R. Byrne[1], W. Veldkamp[2], J. den Harder[3]; [1]Dublin/IE, [2]Leiden/NL, [3]Amsterdam/NL
*(irene.debroglie@gmail.com)*

**Purpose:** This study aimed to design and manufacture 3D-printed low cost customised anthropomorphic phantoms for thorax CT imaging evaluation, including lung vessels and disease (lung parenchyma and nodules).
**Methods or Backround:** Two identical mirrored models of the lung vessel tree (vessels diameters between 1 cm and 1 mm) were generated inside elliptical inserts (10x15x6 cm) using in-house code (Matlab). By design, small phantom sections with vessels were closed to trap the 3D-printed powder in to mimic diseased parenchyma. Lung nodules with different geometries (spiculared, spherical, ovoids, rugous) were designed (Meshmixer), with 3-15 mm diameter range. A thorax elliptical model (30x20x6 cm) with holes to insert the lungs and the spine, was manufactured with PMMA using CNC. The lung inserts were 3D printed with TPU (HP, MJF technique), the spine with allumide (SLS) and the nodules with different 3D printers and materials (PA12-MJF, PA12-BlueSint-SLS, PRUSA Tough Resin). The thorax phantom was scanned in a Siemens

Wednesday

Somaton Edge Plus CT (thorax protocol) for a range of kV and the attenuation of the materials measured with selected ROIs and histograms.
**Results or Findings:** The attenuation of the phantom materials was, for 120 kV: thorax-PMMA (118+-4)HU, spine-allumide (785+-10)HU, lung vessels-TPU (80+-10HU), parenchyma-(raw-TPUpowder) (-680+-30)HU. For the lung nodules it was: PA12-Bluesint (-90+-20HU), PA12-MJM (-32+-10HU) and Prusa_ThoughResin(123+-10HU). The 3D printed materials showed in general a slight increase in HU with increasing kV. The total cost of this in-house anthropomorphic phantom was around 500 euros.
**Conclusion:** A low cost anthropomorphic thorax phantom, containing realistic lung vessel distributions and lung nodules was designed and manufactured with tissue-equivalent materials using 3D printing, to be used in task-based clinical assessment of image quality in CT.
**Limitations:** The accuracy and reproducibility of the 3D-printed lungs and nodules will be stablised in the future.
**Ethics committee approval:** This study did not use any patient data and did not require Ethics committee aproval.
**Funding for this study:** The lung models were created with in-house software created during the NWO funded CLUES project (Pr. Nr. 13592, 2015-2019, Wouter Veldkamp project leader). The in-house code was expanded on during the project Through the eyes of AI: safe and optimal integration of Artificial Intelligence in the Radiology Workflow (Pr.Nr 17392, 2019-2022), funded by NWO-Veni Personal grant programma awarded to I. Hernandez-Giron
**Author Disclosures:**
John Egan: Nothing to disclose
Barbara Snow: Nothing to disclose
Peter McHale: Nothing to disclose
Catherine D'Helft: Nothing to disclose
Wouter Veldkamp: Nothing to disclose
J.M. den Harder: Nothing to disclose
Irene Hernandez-Giron: Nothing to disclose
Richard Byrne: Nothing to disclose
Aoife Gaffney: Nothing to disclose

### RPS 313-5

**Development of a breast perfusion phantom including in-line optical spectroscopy measurements for the validation and testing of dynamic contrast-enhanced dedicated breast CT**

*L. Goris*[1], S. Manohar[2], I. Sechopoulos[3]; [1]Hengelo/NL, [2]Enschede/NL, [3]Nijmegen/NL

**Purpose:** To develop a dynamic perfusion breast phantom to validate the time-dependent quantitative iodine concentration estimates in dynamic contrast-enhanced dedicated breast CT (DCE-bCT).
**Methods or Backround:** Perfusion functionality is added to a 3D-printed anatomic breast phantom with tumor mimic, by incorporating a pumping system, including two programmable pumps for input of water and contrast agent, tubing, and an output reservoir. Potassium iodide is used as the contrast agent since its light absorbance was found to be concentration-dependent. The in-line spectroscopy setup includes a light source (400-600 nm), to illuminate the contrast inlet and outlet leading into and leaving the breast phantom respectively, and photodetectors to measure the transmitted light. To evaluate precision, nine different iodine concentrations (0.5-7.6 mg I/mL) were tested three times and the coefficient of variation (CoV) was calculated. For accuracy, measurements of six concentrations were used to fit a calibration curve, and the errors of the remaining three were calculated. The division of concentrations used for fitting and testing was repeated three times to determine the average error. A time-varying iodine profile, including wash-in and wash-out, was pumped through the phantom and simultaneously measured with the optical and DCE-bCT systems.
**Results or Findings:** The in-line spectroscopy measurements had a CoV of 0.29% and an average error of 0.13%. The optical system detected the time-varying iodine profiles, with the recorded timepoints matching those visible in the transmission curves.
**Conclusion:** A combination of a 3D-printed phantom, a pumping system, and real-time spectroscopy seems a feasible approach for physical simulations of DCE processes in a breast for validation of DCE-bCT.
**Limitations:** Iodine-concentration estimates through the tumor mimic are not yet possible.
**Ethics committee approval:** Not applicable
**Funding for this study:** ERC Grant No.864929
**Author Disclosures:**
Liselot Goris: Nothing to disclose
Srirang Manohar: Nothing to disclose
Ioannis Sechopoulos: Advisory Board: Koning Corp

### RPS 313-6

**Model-based noise correction of CT perfusion blood flow measurements based on digital perfusion phantoms**

*N. Vats*, P. Mayer, M. Klauß, H-U. Kauczor, W. Stiller, S. Skornitzke; Heidelberg/DE
*(nhvats29@gmail.com)*

**Purpose:** Image noise can negatively influence the accuracy of CT perfusion blood flow (BF) measurements, the aim of this study was to evaluate model-based noise correction using digital perfusion phantoms.
**Methods or Backround:** Digital perfusion phantoms were simulated by forward convolution of impulse response function with arterial input function averaged over 59 patients, considering ground-truth BF (GTBF) of 5-420ml/100 ml/min with a temporal sampling of 1.5s. To investigate the impact of noise on perfusion measurements, 2x576 random samples of Gaussian noise (standard deviation=25HU) were introduced. These noise-added phantoms were evaluated on a commercially available workstation (syngo.via; Siemens Healthineers) using deconvolution to measure noise-impacted BF. Phantoms were iteratively generated and evaluated to measure BF accurately by approximating GTBF from noise-impacted BF. To this end, noise correction was estimated from differences between measured BF and noise-impacted BF. The absolute difference between BF and GTBF were calculated, as well as the contrast-to-noise ratio (CNR). For validation, parenchyma and tumors of 14 pancreatic adenocarcinoma patients were evaluated.
**Results or Findings:** The measured noise-impacted BF and noise-corrected BF were 140.3±111.7 ml/100ml/min, and 131.9±125.9 ml/100ml/min, respectively, whereas GTBF was 131.3±127.7 ml/100ml/min. After correction, the average absolute difference in BF measurements decreased significantly from 18.8 to 3.6 ml/100ml/min and CNR improved from 2.52 to 2.66. For patients, BF converged from 148.3±50.8 ml/100ml/min to 155.0±91.5 ml/100ml/min for parenchyma, and 45.8±20.3 ml/100ml/min to 13.3±18.7 ml/100ml/min for tumour tissue following correction.
**Conclusion:** Convergence of BF in simulated and patient data signifies efficacy of developed algorithm in improving measurement accuracy by correcting negative influence of image noise. With further refinement, algorithm holds the potential to standardise perfusion measurements, enhancing comparability across patients, imaging centres, and equipment vendors, thus contributing to more accurate diagnosis and treatment planning.
**Limitations:** For patient dataset, ground-truth were unavailable, and BF in some tumours approached to zero with correction.
**Ethics committee approval:** The research protocol received approval from the ethics committee at University Hospital Heidelberg and was conducted in accordance with the ethical standards of the World Medical Association (Declaration of Helsinki).
**Funding for this study:** This study was funded by the BMBF: grant 031L0163.
**Author Disclosures:**
Neha Vats: Nothing to disclose
Wolfram Stiller: Nothing to disclose
Miriam Klauß: Nothing to disclose
Hans-Ulrich Kauczor: Nothing to disclose
Philipp Mayer: Nothing to disclose
Stephan Skornitzke: Employee: Philips Market DACH GmbH

### RPS 313-7

**An update on iMet-MRI: a European project aimed at improving metrology for quantitative MRI**

C. McGrath[1], C. Clarkson[2], A. McDowell[2], E. Cooke[2], M. Causevic[3], M. Cashmore[2], P. Tofts[4], A. Manzin[5], *M. G. Hall*[2]; [1]Belfast/UK, [2]London/UK, [3]Sarajevo/BA, [4]Brighton/UK, [5]Torino/IT
*(matt.hall@npl.co.uk)*

**Purpose:** Conventional clinical MRI is designed to produce single-use images for use by individual human radiologists. Because conventional MRI does not contain quantitative information, comparisons of images between different scanners and timepoints is extremely challenging. Quantitative MRI (qMRI), in contrast, aims to makes measurements of physical parameters. As such it offers huge potential for improved reproducibility and to detect diffusion changes in tissue which are difficult or impossible to detect in conventional images. Effective measurements require uncertainties to be understood and the performance of individual systems to be quantified. The iMet-MRI project aims to provide a solid foundation for qMRI to support clinical translation. This is an update on its progress.
**Methods or Backround:** Metrologically characterised materials suitable for quantitative T1 and T2 imaging, diffusion, fat fraction, and iron content. Samples with multiple target parameter values have been produced. We have developed two sets of scan protocols: one maximising measurement quality, another for clinically feasible timescales. Analysis codes have been developed for all measurands and analysis is in progress. We have also developed a detailed digital phantom which has allowed simulation-based investigation of sensitivity to different measurement inaccuracies.

**Results or Findings:** A modular phantom designed and manufactured, phantoms validated, and scan protocols developed. Data has been received from core MRI systems and is being analysed, with additional acquisition continuing.
**Conclusion:** The project is now approximately 75% complete, and we are on target to deliver all our objectives. Experience from protocol implementation is being collated ahead of the production of a good practice guide for MRI QA. The software tools will also be made available to allow open reproducibility of all our results.
**Limitations:** The project's focus is the performance of the scanner, not the interaction of scanner and patient.
**Ethics committee approval:** Not Applicable for this study.
**Funding for this study:** This project (iMet-MRI) has received funding from the EMPIR programme co-financed by the Participating States and from the European Union's Horizon 2020 research and innovation programme.
**Author Disclosures:**
Paul Tofts: Nothing to disclose
Matt G. Hall: Nothing to disclose
Matt Cashmore: Nothing to disclose
Alessandra Manzin: Nothing to disclose
Cailean Clarkson: Nothing to disclose
Amy McDowell: Nothing to disclose
Elizabeth Cooke: Nothing to disclose
Cormac McGrath: Nothing to disclose
Merima Causevic: Nothing to disclose

### RPS 313-8
**Magic angle Magnetic resonance imaging (maMRI): first in vivo study using a unique low field scanner: what can we see?**
*D. Amiras*, K. Chappell, J. McGinley, H. Lanz, C. Gupte, M. Ristic; London/UK
*(dimitri.amiras@nhs.net)*

**Purpose:** Conventional knee MRI (cMRI) imaging is sensitive and specific at identifying acute injuries in soft tissues due the presence of blood or fluid. However, due to the inherent low MRI signal in collagen, cMRI does not show a significant contrast between the scarred lax tissues with poor collagen alignment and normal tissues. It is observed that well aligned collagen fibres produce increased signal, at short TEs, when imaged at the Magic Angle (MA≈55°) to the main magnetic field (B0). Our aim is to exploit this effect. The aim of our study was to investigate whether a novel low-field magnet rotating about 2 axes could exploit the magic angle phenomenon to generate images of soft tissues in the knees of healthy volunteers (maMRI).
**Methods or Backround:** A bespoke low-field system was developed to exploit the magic angle effect. This comprises a 0.15T open magnet with B0 being parallel to the magnet poles. The magnet can be rotated about two orthogonal axis to produce arbitrary orientation of B0 to the stationary patient. The overall magnet assembly provides an accessible gap sufficient to comfortably accommodate extremity imaging. The scanner was also capable of performing cMRI to a standard comparable to other low-field MRI. A pre-clinical study with ethical approval for imaging healthy volunteers imaged subjects, maMRI sequences were performed on each subject in addition to cMRI.
**Results or Findings:** Our results highlight the potential benefit of maMRI - healthy ligaments and tendons could potentially be differentiated from those that are degenerative and those with chronic injuries. This is of considerable interest in informing clinical decision-making and can provide valuable information for the prevention of injury.
**Conclusion:** Our results highlight the potential benefit of magic maMRI - healthy ligaments and tendons could potentially be differentiated from those that are degenerative and those with chronic injuries. This is of considerable interest in informing clinical decision-making and can provide valuable information for prevention of injury.
**Limitations:** Not applicable for this study.
**Ethics committee approval:** Local ethics committee approved this study.
**Funding for this study:** This study was funded with the Wellcome Trust Innovator Award.
**Author Disclosures:**
John McGinley: Nothing to disclose
Dimitri Amiras: Nothing to disclose
Karyn Chappell: Nothing to disclose
Mihailo Ristic: Nothing to disclose
Chinmay Gupte: Nothing to disclose
Harry Lanz: Nothing to disclose

**11:30-12:30 Room G1**

### Research Presentation Session: Radiographers

### RPS 314

### Research evidence to inform and shape the future

**Moderators**
J. Gardling; Lund/SE
*(jenny.gardling@med.lu.se)*
D. Akata; Ankara/TR
*(dakata@hacettepe.edu.tr)*
**Author Disclosures:**
Deniz Akata: Consultant: General Electric

### RPS 314-3
**An investigation into the knowledge, attitudes, and practice of lead shielding utilisation in interventional radiology departments and cath-labs across Europe**
M. O'Rouke[1], N. Moore[1], S. Svetlic[2], H. L. Bucknall[3], M. F. McEntee[1], *A. England*[1]; [1]Cork/IE, [2]Milan/IT, [3]London/UK
*(aengland@ucc.ie)*

**Purpose:** According to current literature, there is a lack in the knowledge, attitudes and practices (KAP) of radiation protection (RP) among interventional radiology (IR) and Cath-lab staff. To the best of the authors' knowledge, there are no studies investigating the radiation protection KAP of radiology staff within IR departments and cath-labs across Europe. This study aims to determine the RP KAP of staff within IR and cath-labs across Europe and the associated influencing factors.
**Methods or Backround:** A cross-sectional study in the form of an online questionnaire was developed. Participation was advertised online via online platforms and through email. Inclusion criteria included qualified healthcare professionals currently working in Interventional Radiology and cath-labs across Europe. Section 1 of the questionnaire consisted of questions regarding demographic data. Section 2 comprised of questions regarding RP training and protocols. Section 3 involved questions regarding the use of different types of RP lead shields, both personal and co-worker use. Section 4 concluded the questionnaire with questions regarding other methods of minimising radiation dose within the departments.
**Results or Findings:** A total of 178 responses to the questionnaire were recorded. Most respondents were female (72%), radiographers (75%) and within the age bracket of 25-34 (46%). Only (53%) had ever received RP specific training and the majority (63%, 80) of respondents were currently practicing in Ireland.
**Conclusion:** The KAP of IR and Cath-Lab staff regarding RP within departments across Europe is low. The unavailability of basic radiation protection tools and RP specific training courses/modules were some of the reasons for sub-optimal self-protection against ionising radiation exposure among the respondents. To avoid unnecessary exposure to themselves, co-workers, and patients, it is suggested that medical professionals be equipped with appropriate training and RP tools.
**Limitations:** Self-reporting questionnaire limits this study.
**Ethics committee approval:** This study was ethically approved by the Medical School SREC - University College Cork.
**Funding for this study:** No funding was obtained for this study.
**Author Disclosures:**
Mark F. McEntee: Nothing to disclose
Niamh Moore: Nothing to disclose
Silvia Svetlic: Nothing to disclose
Helen Louise Bucknall: Nothing to disclose
Andrew England: Board Member: EFRS Advisory Board: RoClub
Megan O'Rouke: Nothing to disclose

### RPS 314-4
**Review of diagnostic reference levels (DRLs) in interventional neuroradiology (INR)**
*M. Grech*, F. Zarb, R. Grech, P. Bezzina; Msida/MT
*(marvin.grech@gov.mt)*

**Purpose:** The objective of this study was to evaluate radiation dose quantities and methodology for establishing diagnostic reference levels DRLs (3rd quartile/75th percentile) for interventional neuroradiology (INR).

**Methods or Backround:** Four different databases: Scopus, Web of Science, PubMed, and Pro Quest were utilized using four keywords: Air Kerma-Area Product (PKA), Fluoroscopy Time (FT), Reference Air Kerma (CAK) and DRLs and their synonyms to search literature in English within the last decade.
**Results or Findings:** DRLs were recorded from 38 studies for different Neurointerventional procedures extracted from the literature for PKA (Gy/cm2), FT (Minutes), and RAK (mGy). Procedure - Number of studies / PKA (Gy/cm2) Mean (min/max) / FT (minutes) Mean (min/max) / RAK (mGy) Mean (min/max), Cerebral Angiography - 29 / 102.64 (41/256.65) / 11.73 (6/28.4) / 657.74 (289/921.1), Stroke Thrombectomy - 8 / 163.57 (110/225.1) / 41.34 (30/44/7) / 1012.56 / (730/1590), Aneurysm Coiling - 27 / 254.06 (52.1/487.4) / 54.51 (16/90) / 3309.89 (505/4750) AVM/AVF Embolisation - 9 / 384.63 (206.4/550) / 86.37 (58.57/535) / 4130.20 (2350/6000). Statistically significant ($p<0.05$) variations are evident between procedures based on their complexity. Other variations based on equipment type, operator experience and methods of data collection were also noted. The authors also sought to identify potential reasons for such discrepancies within the included studies.
**Conclusion:** To date, few studies have published data regarding DRL's in INR procedures. The evident variation in DRL quantities, warrants the need for optimization strategies, and guidelines for quality control and to reduce procedural radiation doses as per the "ALARA" principle. Establishment and analysis of DRLs in INR procedures is essential for subsequent optimisation of radiation doses in INR procedures.
**Limitations:** This is a literature review and is based on the information available within the reviewed papers.
**Ethics committee approval:** Not applicable for this study.
**Funding for this study:** No funding was received for this study.
**Author Disclosures:**
Francis Zarb: Nothing to disclose
Paul Bezzina: Nothing to disclose
Reuben Grech: Nothing to disclose
Marvin Grech: Nothing to disclose

### RPS 314-5

**Public awareness and preferences for medical radiation risk information**

N. Mernagh, A. England, N. Moore, R. Young, M. F. McEntee, *G. A. Curran*; Cork/IE
*(grainne.curran@ucc.ie)*

**Purpose:** New legislation says that healthcare workers must inform patients of the radiation dose and risks associated with medical imaging. Literature suggests the public are being poorly informed. There has been little research carried out where comparisons of risk have been evaluated in terms of patient understanding. The aim of this study is to investigate radiation awareness and preferences the public may have on radiation risk communication prior to medical imaging examinations.
**Methods or Backround:** A cross-sectional study was used to obtain quantitative and qualitative data from the public. The online questionnaire was designed using Qualitrics XM. The target participants were members of the public over 18 years, irrespective of whether they have undergone previous imaging. Data was analysed using Microsoft Excel.
**Results or Findings:** A total of 413 participants completed the survey. Majority (n=364,88.1%) had previously had imaging. Most respondents rated their knowledge on radiation risks as poor (n=213/401, 53.1%). Participants were asked who should be communicating these risks with them, the person carrying out the examination (usually the radiographer) received the most responses (n=334, 34.7%), while the referrer was the second most common (n=224, 23.3%). For the risk communication methods, majority stated very helpful for the associated risk is negligible/minimal/very-low/low/moderate (34.2%), followed by the associated risk of cancer is 1 in 2000 (33.4%).
**Conclusion:** The public have a poor awareness of radiation risks from medical imaging. Multiple responses to who should communicate risk suggests a multidisciplinary approach is warranted. Simple language to convey risk is preferred along with phrases which directly address cancer risk.
**Limitations:** Self-reporting survey limits the scope of this study.
**Ethics committee approval:** Medical School SREC - University College Cork approved this study.
**Funding for this study:** No funding was received for this study.
**Author Disclosures:**
Niamh Mernagh: Nothing to disclose
Mark F. McEntee: Nothing to disclose
Niamh Moore: Nothing to disclose
Rena Young: Nothing to disclose
Gráinne Alison Curran: Nothing to disclose
Andrew England: Advisory Board: RoClub; Board Member: EFRS

### RPS 314-6

**Correlating breast lesion dimensions: imaging vs pathological results**

*L. M. S. d. Jesus*, A. F. C. L. Abrantes, S. I. Rodrigues, L. F. Carvalho, L. P. V. P. V. Ribeiro; Faro/PT
*(00laura.mj@gmail.com)*

**Purpose:** This study aims to establish the correlation between breast neoplasm dimensions as measured via Mammography (MGM), Ultrasonography (US), and Magnetic Resonance Imaging (MRI), and the real tumour dimensions obtained from anatomopathological reports of surgical specimens.
**Methods or Backround:** A retrospective study was carried out on 56 women diagnosed with breast cancer, between January 2021 and November 2022, in a Portuguese Public Hospital. The study assessed the size of the primary tumour using MGM, US, and breast MRI. Subsequently, we compared these measurements with those obtained from the anatomopathological examination.
**Results or Findings:** Our findings revealed that, in MGM, US, and MRI, there was an overestimation in 34/56 (60.7%), 18/56 (32%), and 31/56 (55%) cases, respectively, and an underestimation in 18/56 (32%), 34/56 (60.7%), and 19/56 (33%) cases, respectively. We observed an absolute agreement in measurements for 4/56 cases between MGM and US when compared to pathological anatomy, and 6/56 cases between MRI and pathological anatomy.
**Conclusion:** Our study concluded that there were no significant differences in tumour size measurements between MGM and MRI when compared to pathological anatomy. Additionally, we found that factors such as histological type or breast density did not significantly influence measurement accuracy. Notably, MRI exhibited the highest accuracy in evaluating lesion dimensions, demonstrating a superior correlation with tumour measurements obtained through anatomopathological examination.
**Limitations:** The main limitations of this study are the small sample size due to the number of patients who underwent neoadjuvant chemotherapy, the lack of measurements of all the lesions in all the available imaging modalities due to the external origin of the patients, and the issue of measurement error.
**Ethics committee approval:** This study was funded by the Uaif: 159-2022.
**Funding for this study:** Not applicable for this study.
**Author Disclosures:**
António Fernando Caldeira Lagem Abrantes: Nothing to disclose
Sónia Isabel Rodrigues: Nothing to disclose
Lénis Fátima Carvalho: Nothing to disclose
Laura Martins Segura de Jesus: Nothing to disclose
Luís Pedro Vieira Pedro Vieira Ribeiro: Nothing to disclose

### RPS 314-7

**Patients' experience to MRI examinations: a systematic qualitative review with metasynthesis**

*I. Nieto Alvarez*[1], J. Madl[1], L. Becker[1], O. Amft[2]; [1]Erlangen/DE, [2]Freiburg/DE
*(isabel.nieto@fau.de)*

**Purpose:** The review informs practitioners, patients, and policymakers of observations for future research, and describes the experience and characterising factors in MRI through the adult patients' voice for guidance of future efforts addressing patient needs.
**Methods or Backround:** Patients often mention distress, anxiety or claustrophobia related to MRI, resulting in no-shows, disturbances of the workflow, and lasting psychological effects. Patients' experience varies and is moderated by socio-demographic aspects alongside the clinical condition. While qualitative studies help understand individuals' experiences, to date a systematic review and aggregation of MRI individuals' experience is lacking. We conducted a systematic search in PubMed, Scopus, Web of Science, and PsycInfo databases according to the PRISMA guidelines to identify primary studies reporting patients' responses to MRI. For quality appraisal, the Joanna Briggs Institute (JBI) tools were used. Metasynthesis, a concept map, and meta-aggregation were used for data synthesis.
**Results or Findings:** We identified eight papers on patients' experience description for qualitative meta-summary (294 fulltexts, 46 studies with sufficient quality, 49 quantitative studies), 220 patients in total. Meta-aggregation of 144 patient quotes answered: (1) experiences before, at the scanning table, during, and after an MRI, (2) differences based on clinical condition, and (3) characterising factors, including coping strategies, look-and-feel of medical technology, interaction with professionals, and information. Noteworthy across studies was the difficulty with the confined space, fear of results, need for information and coping strategies.
**Conclusion:** Our findings provide a foundational description of adult patients' MRI experience, revealing themes and characterising factors at each stage of the procedure through the patients' voice.
**Limitations:** Most publications lack participants' health literacy level, occupation, developmental conditions, ethnicity, or country of origin. Studies were mostly conducted in university hospitals. Interviews' raw data unavailability impeded computer-aided analysis.
**Ethics committee approval:** The study was conducted according to a systmatic literature review with meta-summary and metasynthesis of published patient quotes.

**Funding for this study:** This research did not receive any specific grant from funding agencies in the public, commercial, or not-for-profit sectors.
**Author Disclosures:**
Isabel Nieto Alvarez: Employee: Siemens Healthineers
Linda Becker: Nothing to disclose
Oliver Amft: Other: Hahn-Schickard
Janika Madl: Employee: Siemens Healthineers

### RPS 314-8
**Reporting radiographers in Europe: an online survey**
*M. R. V. Pedersen*[1], J. Jensen[2], C. Senior[3], N. Gale[4], C. J. Heales[4], N. Woznitza[5]; [1]Vejle/DK, [2]Odesa/DK, [3]Axminster/UK, [4]Exeter/UK, [5]London/UK

**Purpose:** Reporting radiographers undertake an important role in health care and for the radiographer profession in general. First introduced in the UK, and now in several other European countries. The objective was to investigate the workforce of reporting radiographers across the European Federation of Radiographer Societies (EFRS) community.
**Methods or Backround:** A voluntary anonymous 34-item electronic survey was distributed online using social media accounts such as Twitter, Facebook, and LinkedIn cover a wide range of topics relating to professional roles, advanced practice, education, and seniority. The questionnaire was distributed during a 12-week period in 2022.
**Results or Findings:** A total of 345 individual responses were received from 15 countries with majorities of respondents from United Kingdom (n=245, 71%) and Denmark (n=66, 19%). The mean age was 41.9 (S.D 9.8), similar for females, 42.5 (S.D 9.0), and men 40.9 years (S.D 9.7). Most reporting radiographers worked in public hospitals (90%). The vast majority of the respondents (n=270, n=94%) authored and signed their own clinical reports while a minority (n=18, 6%) stated that their reports were checked by radiologists.
**Conclusion:** The survey highlights the scope of practice of reporting radiographers working in Europe. Reporting is becoming a career path for an increasing number of radiographers across Europe.
**Limitations:** A limitation is that the survey was published in English, so language may have formed a barrier to participation within non-English speaking countries.
**Ethics committee approval:** Research Ethics Committee of the University of Southern Denmark approved this study.
**Funding for this study:** No funding was received for this study.
**Author Disclosures:**
Nick Woznitza: Nothing to disclose
Malene Roland Vils Pedersen: Nothing to disclose
Janni Jensen: Nothing to disclose
Niamh Gale: Nothing to disclose
Claire Senior: Nothing to disclose
Christine J. Heales: Nothing to disclose

### RPS 314-9
**Density bone evaluation by ECHOS: a pilot study**
*R. Santos*, J. Nobrega; Coimbra/PT
*(rutemartinssantos@gmail.com)*

**Purpose:** The purpose of this study was to correlate the BMD values obtained through ECHOS with the individual characteristics of the participants and to compare the BMD values (T-Score and Z-Score) between DEXA and ECHOS.
**Methods or Backround:** Two hundred and nineteen participants were submitted to an ECHOS evaluation and 16 of 219 participants were evaluated by DEXA. All of the participants were evaluated on lumbar spine. All participants answered a sociodemographic questionnaire with factors influencing bone mineral density (eating habits, smoking, physical activity) and signed informed consent.
**Results or Findings:** Of the 219 participants, 68% were female, 54.8% practiced physical activity, 5.9% had hormonal pathologies, 53% were between 22 and 59 years and 11.4% smoked regularly in, however, after comparing the BMD there was a significant correlation with weight, consumption of tobacco and foods rich in calcium but not with the other variables this in the first study, however the second study revealed the existence of a significant correlation between the values of Z-Score but despite this in the values of T-Score was not verified.
**Conclusion:** In short, this study showed that some factors such as smoking, weight and calcium intake may influence the values obtained in ECHOS and also the existence of agreement between the two techniques used to assess BMD. ECHOS, having the advantages of ultrasound, can be an imaging method used to screen for osteoporosis or osteopenia. However future studies must be developed.
**Limitations:** Some questionnaires were not completed by participants, the equipments were not always available, not allowing the evaluation of same number of subjects.
**Ethics committee approval:** The study in question obtained authorisation from the ethics of the Polytechnic Institute of Coimbra (IPC) with number 161_CEIPC_2022.
**Funding for this study:** Not applicable for this study.
**Author Disclosures:**
Rute Santos: Nothing to disclose
João Nobrega: Nothing to disclose

### RPS 314-10
**Liver fibrosis and steatosis ultrasound screening: a FibroScan study**
*R. Santos*, B. Fonseca; Coimbra/PT
*(rutemartinssantos@gmail.com)*

**Purpose:** The aim of this study was to correlate the values of hepatic fibrosis and steatosis obtained through FibroScan with the individual characteristics of the participants, aiming to identify risk factors associated with the presence or absence of these conditions.
**Methods or Backround:** Data collection was conducted through a screening initiative. The exclusion criteria were used: people weighing more than 30 kg/m2, people under 18 years of age and participants who have already undergone kidney or liver transplants. The population was invited to participate in the evaluation, and the entire procedure was explained to them. Volunteers underwent a FibroScan examination and answered sociodemographic questions.
**Results or Findings:** Out of 169 participants, 29% were men, 31.5% were overweight and obese, 15.9% had high cholesterol, 2.9% had diabetes, and 13% had hypertension (HTN). After analysis, 92 participants were selected for fibrosis evaluation and 107 for hepatic steatosis evaluation. The majority of participants did not have fibrosis or hepatic steatosis (76.1% and 64.4%, respectively). 7.6% of participants had moderate to severe fibrosis, and 3.3% had cirrhosis. Regarding hepatic steatosis, 6.6% had the more severe grades. Hepatic fibrosis was not associated with diabetes, cholesterol, HTN, sex, or age. Hepatic steatosis was associated with diabetes, cholesterol, HTN, and age but was not related to the participants' sex.
**Conclusion:** Hepatic fibrosis and steatosis may be related to risk factors. It is important to screen the population so that an early diagnosis of liver disease can be made and possible worsening and complications can be avoided. Fibroscan is a technique that allows a rapid assessment of the liver and its changes, making it ideal for broad population screening.
**Limitations:** Some of the participants did not answer all the questions. The fibroscan examination was performed by more than two radiographers.
**Ethics committee approval:** It was a student initiative, where only the data was used afterwards, so no ethical approval was sought.
**Funding for this study:** Not applicable for this study.
**Author Disclosures:**
Rute Santos: Nothing to disclose
Beatriz Fonseca: Nothing to disclose

**13:00-14:30** **Research Stage 1**

### Research Presentation Session: Vascular

### RPS 415
### Examining portal venous thrombosis: diagnosis and management strategies

**Moderator**
A. Mazzaro; Verona/IT
*(andrea.mazzaro1558@gmail.com)*

### RPS 415-2
**Vascular complications of liver transplantation in children, early diagnosis and management**
*A. F. Mejía León*, L. M. Acosta, L. Acosta, J. E. Chaustre Soledad, S. Trujillo, J. M. Perez, G. Caviedes; Bogotá/CO
*(afmejia8@gmail.com)*

**Purpose:** The aims of this study were to: (1)Describe the most common early vascular complications of liver transplant in the paediatric population. (2) Show the main imaging findings through different case examples of our institution. (3) Compare the radiological findings found in our cohort of patients with those described in the literature.
**Methods or Backround:** The number of paediatric patients undergoing transplantation has grown exponentially in recent years, enhancing the importance of early detection of the different complications related to early and late postoperative stage in the clinical scenario. The radiologist and radiologist-in-training must be aware of these complications and early imaging manifestations.Understanding the findings in different imaging modalities, is essential to a proper approach, with Doppler ultrasound being one of the most important modalities for early detection of complications. Through a multimodality approach we intend to show the importance of each of these modalities for early diagnosis, and likewise, to describe the type of management according to the postoperative time. The management of these complications is not limited to surgical management. The role of interventional radiology is increasingly recognised, depending on the type of complication and the postoperative time being a fundamental pillar in the management of these patients.
**Results or Findings:** Regarding the main vascular complications in the postoperative period of liver transplants in paediatrics, we found greater prevalence of arterial thrombosis, follow by stenosis of the hepatic artery and hepatic veins. Some of these cases were managed by interventional radiology, obtaining favorable results.
**Conclusion:** Findings do not differ from those described in literature presenting a similar frequency of appearance. When complications are detected soon in the early postoperative period, this group of patients benefit from earlier interventions, which translates into better clinical outcomes.
**Limitations:** Only Latin American patients were included limiting the scope of this study.
**Ethics committee approval:** Low risk research hence no ethical approval was required.
**Funding for this study:** No funding was obtained for this study.
**Author Disclosures:**
Juan Manuel Perez: Nothing to disclose
Stephanie Trujillo: Nothing to disclose
Laura Acosta: Nothing to disclose
Gabriel Caviedes: Nothing to disclose
Andrés Felipe Mejía León: Nothing to disclose
Jose Eduardo Chaustre Soledad: Nothing to disclose
Lina Maria Acosta: Nothing to disclose

### RPS 415-3

**Reliability and accuracy of tomographic 3D ultrasound for grading vessel stenosis: a phantom study**

*A. A. Alzahrani*[1], M. Aslam[2], S. R. Sultan[3]; [1]Makkah/SA, [2]London/UK, [3]Jeddah/SA
*(pwp666@hotmail.com)*

**Purpose:** The aim of this phantom study was to assess the accuracy of 3-D tomographic ultrasound (t3DUS) for grading stenosis, using the manufacturer's measurements as the gold standard. The percentage of maximum stenosis was obtained using 2-D ultrasound (2DUS) and t3DUS imaging techniques on a peripheral vascular phantom, including channels with 50%, 75% and 90% stenosis.
**Methods or Backround:** A high-resolution premium Philips Elite ultrasound imaging system (Philips Healthcare, Bothell, WA, USA) was used to obtain maximum percentage of stenosis from a peripheral vascular phantom using 2DUS and t3DUS (PIUR Imaging GmbH, Vienna, Austria) imaging techniques. Inter-operator reproducibility and accuracy of DR and AR 2DUS and t3DUS in measuring maximum stenosis percentage were assessed.
**Results or Findings:** The inter-operator reproducibility of DR and AR 2DUS and t3DUS maximum stenosis measurements between operator A and B (n = 90 for each operator per technique) was excellent (DR 2DUS, ICC = 0.96, 95% confidence interval [CI]: 0.900.98, p < 0.001
**Conclusion:** Free-hand t3DUS is a reproducible and accurate imaging method for grading stenosis. The inter-operator reproducibility of t3DUS for grading stenosis was excellent with a low coefficient of variation. The mean difference in stenosis measurements from manufacturer reference values for all channels was lower in t3DUS than in 2DUS. There was significant under- and overestimation of 2-D DR and AR, respectively, compared with Tomographic 3-DUS for grading vessel stenosis.
**Limitations:** Not applicable for this study.
**Ethics committee approval:** Not applicable for this study.
**Funding for this study:** This study was funded by the King Abdullah Medical City KAMC.
**Author Disclosures:**
Mohammed Aslam: Nothing to disclose
Adel Ahmad Alzahrani: Nothing to disclose
Salahaden R. Sultan: Nothing to disclose

### RPS 415-4

**Reducing the radiation doses and contrast volume in CT portal venography for patients with sinusoidal obstruction syndrome on a second-generation dual-layer spectral CT**

*Y. Wang*; Zhengzhou/CN

**Purpose:** The aim of this study was to investigate the feasibility of 60keV virtual monoenergetic images (VMIs) of spectral CT imaging, combined with low contrast dose for patients with sinusoidal obstruction syndrome (SOS) by comparing with polyenergetic CT images.
**Methods or Backround:** In the prospective study, 28 patients who underwent dual-layer spectral CT scanning were divided into two group, group A (control group,120kVp) and group B (experimental group, 100 kVp). 95 mL of 350 mgI/mL iodixanol was administered to the control group, and personalised injection protocol (25 mgI/kg/s with a duration of 17 seconds) was used for group B, respectively. CT numbers of the portal veins (PVs), liver parenchyma, and subcutaneous fat tissue in the abdomen were measured by the region of interest (ROI). The standard deviation of the images was interpreted as the objective image noise (IN). The diagnostic acceptability (DA) and sharpness of PV margins were obtained using a 5-point score.
**Results or Findings:** Hepatic heterogeneity, puddle-like or micronodular appearance, peripheral distribution of heterogeneity, clover-like sign, splenomegaly, as well as the subjective impression of the observer were significantly associated with SOS diagnosis. The observers' confidence in the diagnosis of SOS increased significantly in group B. The CT value of liver in group B was higher than group A, conversely, SNR and CNR were lower ($P < 0.05$). Compared with group A, the effective radiation dose of group B decreased by 26.6%. The average iodine loads were 33.3 g and 28.3±3.6 g, respectively.
**Conclusion:** Compared to conventional polychromatic CT images, 60 keV VMIs of 100 kVp improved diagnostic performance of sinusoidal obstruction syndrome in portal venography imaging with reduced contrast media and lower radiation dose.
**Limitations:** Our sample is small in spite of a long period of study limiting the scope of this study.
**Ethics committee approval:** This study was approved by the First affiliated hospital of Zhengzhou university Ethics committee.
**Funding for this study:** No funding was received for this study.
**Author Disclosures:**
Yiran Wang: Nothing to disclose

### RPS 415-5

**Development and validation of a new score for selecting the best vascular access for liver cancer intra-arterial procedures (trans-femoral or trans-radial access score)**

*A. Maresca*, L. Tenore, A. Posa, A. Contegiacomo, M. Lippi, L. Natale, R. Iezzi; Rome/IT
*(alessandromaresca12@gmail.com)*

**Purpose:** Intra-arterial procedures play an important role in the treatment of liver neoplastic lesions. Trans-radial artery access has been considered for IR procedures; the choice of a femoral or radial approach is based on the operator’s experience or preference. This study aims to develop a CT-based scoring system for selecting the best candidates for trans-radial approach.
**Methods or Backround:** All the patients who underwent TACE using a trans-radial approach were included. Patients’ clinical data, pre-procedural CT-image and lesion location were registered and a CT scoring system was developed. Procedural complexity, based on angiographic and procedural variables, was evaluated. The relationship between procedural difficulty and the categorical variables were statistically tested.
**Results or Findings:** One hundred and eight-two trans-radial TACE procedures were analysed; only 93 patients were retrospectively included in our study due to the adequacy of the data. The parameters which resulted significant ($p<0.0001$) in determining the procedural difficulty were represented by the aortic arch diameter, the suprarenal aortic diameter, the celiac trunk takeoff angle and the anatomical variants of celiac trunk anatomy. Multivariate regression analysis identified four variables like predictors of procedural complexity: radial caliber < 2.6 mm, left subclavian artery angle < 65 degrees, suprarenal aorta diameter > 33 mm, celiac trunk takeoff angle > 81 degrees. Using these four variables, a 4-point risk score was developed (1-2: easy – 3-4: complex).
**Conclusion:** TAS (transradial access) score seems to be useful to predict complexity of endovascular interventions through a transradial approach.
**Limitations:** The most important limitation of the study is that it is focused on the learning curve of a single operator on a single procedure (lobar chemoembolisation). Data are not applicable to all operators and/or other procedures and do not take into account individual operator variabilities and differences in procedure complexity.
**Ethics committee approval:** The study was led under the approval of the local ethics committee and the institutional review board (IRB).
**Funding for this study:** No funding was obtained for this study
**Author Disclosures:**
Alessandro Maresca: Nothing to disclose
Roberto Iezzi: Nothing to disclose
Andrea Contegiacomo: Nothing to disclose
Luigi Natale: Nothing to disclose
Lorenzo Tenore: Nothing to disclose
Alessandro Posa: Nothing to disclose
Marcello Lippi: Nothing to disclose

### RPS 415-6

**Direct Percutaneous Thrombolysis (DPT): an effective method of salvaging thrombosed native arteriovenous fistula**

*V. Selvamurugan*[1], R. Prasad[2], R. R. Yadav[3], P. Hasani[4], A. Israr[3], H. Lal[3], S. Sharma[3]; [1]Madurai, Ut/IN, [2]Delhi/IN, [3]Lucknow/IN, [4]Vadodara/IN
*(vigneshmdrd@gmail.com)*

**Purpose:** Thrombosed arteriovenous fistulas (AVFs) are either treated by thrombectomy or pharmaco-mechanical thrombolysis with or without percutaneous balloon angioplasty. In this study, we have described an effective and economical technique of salvaging these fistulas using a 20–22-gauge spinal needle and urokinase - direct percutaneous thrombolysis (DPT).
**Methods or Backround:** This prospective study comprised of 148 patients out of which 120 patients presented with AVF thrombosis and were divided into two groups; those with no obvious stenosis on ultrasound (n=38) and second with venous stenosis (n=82). Remaining 28 patients developed thrombosis post angioplasty for venous stenosis. Percutaneous injection of urokinase into the thrombus was done under ultrasound guidance, followed by balloon angioplasty if there was associated stenosis.
**Results or Findings:** In 38 patients who didn't have any stenosis, 32 AVFs were successfully thrombolysed by DPT, with technical success of 84.2%. Remaining six patients required angioplasty because of chronic nature of clot. In 82 patients who had venous stenosis, 80 cases were treated successfully by DPT followed by angioplasty with technical success of 97.5%. In third group (n=28), who developed thrombosis post angioplasty, 100% success rate was noted. The mean length of thrombus was 31.4 ± 4.6 mm and mean diameter of thrombosed vein was 10.5 ± 1.2 mm. There were no major complications encountered during the procedure.
**Conclusion:** Ultrasound guided DPT with urokinase is a safe and economical option for salvaging thrombosed AVF without vascular stenosis that does not need angioplasty.
**Limitations:** The main limitation was inherent selection bias, as we did not enroll patients with very long thrombosed segment and grossly dilated thrombosed venous segment. There was also lack of comparison between the various groups enrolled.
**Ethics committee approval:** All procedures performed in the studies involving human participants were in accordance with the ethical standards of the institutional research committee. Retrospective analysis so ethical approval not taken.
**Funding for this study:** No funding was obtained for this study.
**Author Disclosures:**
Raghunandan Prasad: Nothing to disclose
Amrin Israr: Nothing to disclose
Hira Lal: Nothing to disclose
Srishti Sharma: Nothing to disclose
Vignesh Selvamurugan: Nothing to disclose
Prerna Hasani: Nothing to disclose
Rajanikant R. Yadav: Nothing to disclose

### RPS 415-7

**Relevance of preoperative and early postoperative USG vascular assessment in predicting haemodialysis AV fistula failure in chronic kidney disease patients**

*S. Gupta*, M. Ahmad; Aligarh/IN
*(shikhargu@gmail.com)*

**Purpose:** The increasing prevalence of chronic kidney disease (CKD), coupled with advancements in the diagnosis and treatment of renal diseases and improvements in life expectancy, has led to a greater number of patients requiring hemodialysis. The preferred method of vascular access for haemodialysis is AV fistula formation; however, it is associated with a high rate of failure.
**Methods or Backround:** In this prospective study, 40 CKD patients planned for initiation of maintainence hemodialysis were selected and preoperative ultrasound vessel mapping and early postoperative ultrasound assessment on day seven were utilised to establish criteria for predicting early fistula failure. Preoperative ultrasound mapping was employed to assess various factors such as cephalic vein diameter, compressibility, and colour flow, radial and brachial artery diameter, peak systolic velocity, and intimal wall calcification. Postoperatively, ultrasound examinations were conducted on day seven and at six weeks to evaluate fistula blood volume and detect any complications.
**Results or Findings:** A significant association between fistula failure and factors such as cephalic vein diameter, brachial artery diameter, intimal vessel wall calcification, and comorbid conditions like diabetes mellitus was observed. Furthermore, the blood flow at day seven was notably lower in the failure group compared to those with a functioning fistula and any fistula with blood flow <154 ml/min on day 7 may be predictive of early fistula failure.
**Conclusion:** Preoperative vessel mapping and early postoperative ultrasonography is indispensable for patients who require AV fistula formation for hemodialysis and provide valuable information for selecting suitable vessels in successful fistula creation and enable early intervention to salvage a failing fistula after the surgery. By utilising these, healthcare professionals can make informed decisions and take necessary steps to optimise the outcomes of AV fistula formation in patients undergoing haemodialysis.
**Limitations:** No limitations were identified in this study.
**Ethics committee approval:** Institutional Ethics Committee approved this study.
**Funding for this study:** No fundings were received for this study.
**Author Disclosures:**
Mehtab Ahmad: Nothing to disclose
Shikhar Gupta: Nothing to disclose

### RPS 415-8

**Imaging of abdominal arteries: intra-individual comparison of Relaxation-Enhanced Angiography without contrast and triggering (REACT) with 4D contrast-enhanced MR angiography at 3T**

*J. P. Janssen*[1], L. Goertz[1], K. Kaya[1], J-P. Grunz[2], T. Persigehl[1], K. Weiss[3], L. Pennig[1], C. H. Gietzen[1]; [1]Cologne/DE, [2]Würzburg/DE, [3]Hamburg/DE

**Purpose:** The main aim of this study was to compare a Novel Relaxation-Enhanced Angiography without contrast and Triggering (REACT) sequence with 4D contrast-enhanced magnetic resonance angiography (4D CE-MRA) for imaging of the abdominal arteries.
**Methods or Backround:** Thirty patients (35.7±16.8 years; 10 males) who received abdominal vessel imaging using a standardised protocol at 3T were included in this retrospective, single centre study. The protocol comprised both 4D CE-MRA and flow-independent REACT (Compressed SENSE factor 10, reconstructed voxel size 0.8x0.8x0.9 mm3) sequences. Two radiologists independently evaluated abdominal arteries for the presence of stenosis, variants, and other vascular findings (e.g., dissection). Subjective image quality of arteries was assessed using a 4-point Likert scale (1=non-diagnostic, 4=excellent). Vessels were classified based on size: (1) aorta (supra- and infrarenal segments), (2) large (celiac trunk, superior mesenteric artery, renal arteries), (3) medium (splenic artery, common and proper hepatic artery) and (4) small (gastric arteries, hepatic arteries, inferior mesenteric artery) arteries.
**Results or Findings:** REACT yielded a median acquisition time of 304 s. Considering 4D CE-MRA as the standard of reference, REACT achieved a sensitivity of 87.5% and a specificity of 100% for relevant (≥50%) stenosis while detecting 89.3% of vascular variants and 100% of other findings. Vessel quality was comparable between both sequences at the aorta (4D CE-MRA: 3.94±0.24, REACT: 3.88±0.44; p<0.044) as well as for medium (4D CE-MRA: 2.93±0.96, REACT: 2.77±0.90; p=0.028) and small arteries (4D CE-MRA: 2.15±0.85, REACT: 2.04±0.86; p=0.032). In contrast, 4D CE-MRA yielded slightly higher scores for large arteries (4D CE-MRA: 3.61±0.61, REACT: 3.35±0.72; p<0.001).
**Conclusion:** REACT provides a good diagnostic performance for the detection of relevant stenosis, variants, and other findings of abdominal arteries while yielding to 4D CE-MRA comparable image quality, underlining its use for non-contrast evaluation of the abdominal vasculature.
**Limitations:** No limitations were identified in this study.
**Ethics committee approval:** The study is retrospective so no ethical approval was sought.
**Funding for this study:** No funding was received for this study.
**Author Disclosures:**
Lenhard Pennig: Speaker: Philips Healthcare
Carsten H. Gietzen: Nothing to disclose
Kenan Kaya: Nothing to disclose
Kilian Weiss: Employee: Philips Healthcare
Jan-Peter Grunz: Speaker: Siemens Healthineers
Jan Paul Janssen: Nothing to disclose
Lukas Goertz: Nothing to disclose
Thorsten Persigehl: Nothing to disclose

### RPS 415-9

**Vascular access devices registry as a working tool to improve infection control and thromboembolism management in cancer patients**

*M. Cherkashin*, S. Alexandrov, F. Valieva, A. Nikolaev, T. Bolshakova, E. Ilyukhin, N. Berezina; Saint-Petersburg/RU
*(mikhail.a.cherkashin@gmail.com)*

**Purpose:** The aim of this study was to discuss permanent vascular access devices registry development and implementation.
**Methods or Backround:** Vascular access devices (port-systems, peripherally implanted central catheters etc) characterised by thrombotic and infectious complications. After device implantation oncology patient can receive different types of anti-tumour therapy in different hospitals and sometimes it's very hard to obtain clear information - which device was implanted, were there any complications etc. The applicable way to exchange clinically important information between hospitals is creation of electronic registers with access for healthcare providers, involved in patient management. We decided to use online platform "Russian Registry of Treatment of Venous Thromboembolism" (NCT03881345) with some customisation, related to central venous devices, concominante blood stream infections, device malfunctions etc.

**Results or Findings:** Registry was started with 3 hospitals operating with paediatric oncology in St Petersburg. System development includes relevant steps: level of access (in each hospital should be dedicated owner with total access and each healthcare provider, involved in device management should have rights to upload information); data fields customisation (type of device, place of insertion, technical features during implantation, complications, malfunctions, thrombosis/occlusion, infection, therapy etc). In first month since process was started, 24 cases were uploaded (14 port-a-caths, 8 PICCs, 2 midlines) based on retrospective data we estimate 400-600 cases per year.
**Conclusion:** Registry is the extremely helpful tool for patient tracking during all stages of cancer treatment. Based on our results, such types of initiatives should be spreaded on city level with all paediatric oncology departments involvement.
**Limitations:** No information was provided by the submitter.
**Ethics committee approval:** This study was funded approved by Local ethics committee.
**Funding for this study:** This study was funded by the hospital.
**Author Disclosures:**
Evgeny Ilyukhin: Nothing to disclose
Sergey Alexandrov: Nothing to disclose
Mikhail Cherkashin: Nothing to disclose
Fatima Valieva: Nothing to disclose
Tatyana Bolshakova: Nothing to disclose
Natalia Berezina: Nothing to disclose
Aleksey Nikolaev: Nothing to disclose

### RPS 415-10

**Diagnostic performance of angio-MRI in the evaluation of large vessel vasculitis: preliminary results**

*E. Duka*[1], L. Di Meglio[1], E. Tombetti[1], M. Papa[2], G. M. Roda'[1], G. Carrafiello[1]; [1]Milan/IT, [2]Gragnano Trebbiense/IT
*(ejonaduka@hotmail.com)*

**Purpose:** The study aims to evaluate diagnostic performance and reproducibility of angio-MRI in the evaluation of patients affected by large vessel vasculitis (Takayasu arteritis and Giant cell arteritis).
**Methods or Backround:** Retrospective analysis of 22 patients affected by large vessel vasculitis that performed angio-MRI in our centre being referred from the Immunology and Rheumatology colleagues. Two readers with five- and two-years' experience in angio-MRI retrospectively analysed the images. The evaluation regarded the involvement of aorta, supra-aortic trunks, splanchnic vessels and iliofemoral vessels. The inter-observer agreement and reliability was established with Cohen's kappa test. The diagnostic findings were stated in percentage. The diagnostic performance was expressed in terms of percentage with 95% of interval confidence in terms of sensitivity and specificity.
**Results or Findings:** Aortic involvement (wall thickening with enhancement) was the commonest finding in 63% of patients. The diagnostic performance was 100% specificity, 80% sensitivity and the inter-reader agreement showed moderate accuracy (κ 0.88).
**Conclusion:** Angio-MRI represents a valid and reliable tool in the evaluation of patients affected by large cell arteritis, in the diagnosis, management and during follow-up.
**Limitations:** There is a small number of patients presenting with the pathology since it is a rare disease. More patients could be enrolled in order to develop a more accurate description of MRI signs of this disease.
**Ethics committee approval:** No ethics committee approval necessary.
**Funding for this study:** No funding was received for this study.
**Author Disclosures:**
Maurizio Papa: Nothing to disclose
Enrico Tombetti: Nothing to disclose
Giovanni Maria Roda': Nothing to disclose
Letizia Di Meglio: Nothing to disclose
Ejona Duka: Nothing to disclose
Gianpaolo Carrafiello: Nothing to disclose

### RPS 415-11

**Usefulness of low-energy virtual monochromatic CT imaging with deep-learning image reconstruction in the delineation of endoleaks after endovascular stent-graft placement**

*T. Higashigawa*[1], Y. Ichikawa[1], K. Nakajima[2], T. Kobayashi[1], K. Domae[1], A. Yamazaki[1], N. Kato[1], H. Sakuma[1]; [1]Tsu/JP, [2]Ise/JP
*(higashikawat@clin.medic.mie-u.ac.jp)*

**Purpose:** The aim of this study was to investigate the usefulness of low-energy virtual monochromatic imaging combined with deep learning image reconstruction (DLIR) in improving the delineation of endoleak after endovascular stent-graft placement in contrast-enhanced dual-energy CT (DECT).
**Methods or Backround:** Sixty-one consecutive patients (median age, 79 years; 46 men) after endovascular stent-graft placement who underwent contrast-enhanced DECT between December 2021 and February 2023 were studied. Virtual monochromatic 40- and 70-keV images were reconstructed using DLIR (TrueFidelity-H) and conventional hybrid iterative reconstruction (IR) (ASIR-V50%). Contrast-to-noise ratio (CNR) of endoleak on the venous phase CT were calculated. Four different reconstructed image series (hybrid IR and DLIR at two energy levels, 40- and 70-keV) were displayed side-by-side and visually evaluated for endoleak conspicuity on a 5-point comparative scale from 0 (best) to -4 (significantly inferior).
**Results or Findings:** A total of 30 out of 61 patients had endoleak (type II, 27; type III, 2; type I, 1). CNR of the endoleak were significantly higher in DLIR than in hybrid IR on both 40- and 70-keV images (40-keV, 14.5 ± 7.3 vs 8.6 ± 4.2, $p < 0.001$; 70-keV, 8.7 ± 4.5 vs 5.5 ± 2.6, $p < 0.001$). The endoleak conspicuity score for 40-keV DLIR images (Reviewer 1, -0.2 ± 0.4; Reviewer 2, 0.0 ± 0.0) was significantly higher than 40-keV hybrid IR (Reviewer 1, -0.5 ± 0.5; Reviewer 2, -1.0 ± 0.0; $p < 0.05$), 70-keV DLIR (Reviewer 1, -1.8 ± 0.4; Reviewer 2, -2.0 ± 0.0; $p < 0.001$) and 70-keV hybrid IR images (Reviewer 1, -1.8 ± 0.4; Reviewer 2, -2.4 ± 0.5; $p < 0.001$), respectively.
**Conclusion:** The utilisation of low-energy virtual monochromatic imaging combined with DLIR method improves the delineation of endoleak after endovascular stent-graft placement.
**Limitations:** Retrospective study limits the scope of this study.
**Ethics committee approval:** The current study was conducted with the approval of our institutional review board, and written informed consent was waived because existing clinical CT data were used for this study. The opportunity to opt out of participating in this study was provided by a notice posted on the hospital's website. No patient indicated an intention to be excluded from this study.
**Funding for this study:** No funding was was received for this study.
**Author Disclosures:**
Kensuke Domae: Nothing to disclose
Takatoshi Higashigawa: Nothing to disclose
Hajime Sakuma: Nothing to disclose
Yasutaka Ichikawa: Nothing to disclose
Noriyuki Kato: Nothing to disclose
Tatsuhiro Kobayashi: Nothing to disclose
Akio Yamazaki: Nothing to disclose
Ken Nakajima: Nothing to disclose

**13:00-14:30** **Research Stage 2**

### Research Presentation Session: Oncologic Imaging

### RPS 416

### Radiomics and artificial intelligence

**Moderator**
D. Leithner; New York, NY/US
*(doris.leithner@gmail.com)*

### RPS 416-2

**Handcrafted radiomics, deep radiomics and transcriptomics data provide complementary and potentiating prognostic information in soft-tissue sarcoma patients**

*A. Crombé*, C. Lucchesi, F. Bertolo, M. Kind, A. Michot, R. Perret, F. Le Loarer, A. Bourdon, A. Italiano; Bordeaux/FR

**Purpose:** The purpose of this study was to identify subgroups of soft-tissue sarcoma (STS) patients using handcrafted and deep radiomics, to understand them, and investigate their impact on metastatic relapse-free survival (MFS).
**Methods or Backround:** We included all consecutive adults with newly diagnosed locally-advanced STS managed at our sarcoma centre between 2008 and 2020, with contrast-enhanced (CE) baseline MRI. After MRI post-processing, segmentation and reproducibility assessment, 175 handcrafted radiomics features (h-RFs) from T1-weighted imaging (WI), T2-WI and fat-suppressed CE-T1-WI were calculated. Convolutional autoencoder neural network (CAE) and half-supervised CAE (HSCAE) were trained in repeated cross-validation on CE-T1-WI from one training cohort (n=200 patients) and validated on a testing cohort (n=25 patients), to extract 1024 deep radiomics features (d-RFs) per model. Following RNAseq of 110 samples, gene expression levels were calculated. Unsupervised classifications based on h-RFs, CAE, HSCAE and RNAseq were built with hierarchical clustering and explained according to histological features, radiological features, gene expression, pathway and survival analyses.

Wednesday

**Results or Findings:** 225 patients were included (120 men [53.3%], median age: 62 years). Three radiomics classifications were obtained (h-RF, CAE and HSCAE groups), which were not associated with the transcriptomics groups, but with prognostic radiological features known to correlate with higher grade (all P-values<0.001), and Sarculator groups (all P-values<0.001). HSCAE and h-RF groups were also associated with MFS in multivariable Cox regressions (P =0.0146 and 0.0043, respectively). Combining these groups improved the prognostic performances of the transcriptomics groups alone (c-index=0.603, increasing to 0.666 with h-RF [P=0.0380] and 0.709 with HSCAE [P=0.0110]). Fifteen genes were dysregulated and two pathways were up-regulated in the h-RF groups, which were linked to tumorigenesis and immune response.
**Conclusion:** Radiophenotypes of STS on pre-treatment MRI obtained with handcrafted and deep radiomics were explainable by radiologists, independently associated with MFS and strengthened transcriptomics signature.
**Limitations:** This is a retrospective single-centre study.
**Ethics committee approval:** The study was approved by the Institutional Review Board of Bergonié Institute, comprehensive cancer centre of Bordeaux, France.
**Funding for this study:** No funding was received for this study.
**Author Disclosures:**
Aurélien Bourdon: Nothing to disclose
François Le Loarer: Nothing to disclose
Antoine Italiano: Nothing to disclose
Amandine Crombé: Nothing to disclose
Audrey Michot: Nothing to disclose
Frédéric Bertolo: Nothing to disclose
Raul Perret: Nothing to disclose
Michèle Kind: Nothing to disclose
Carlo Lucchesi: Nothing to disclose

### RPS 416-3

**CT lung cancer screening: pricing and cost-saving potential for deep learning computer-aided lung nodule detection software**
Y. Du[1], M. Greuter[2], *M. Prokop*[3], G. De Bock[2]; [1]Hangzhou/CN, [2]Groningen/NL, [3]Nijmegen/NL
*(mathias.prokop@radboudumc.nl)*

**Purpose:** This study aimed to explore appropriate pricing for commercial deep learning computer aided detection (DL-CAD) systems in different modes of use to maximise cost savings and identify the most cost-effective mode for lung cancer screening.
**Methods or Backround:** We evaluated DL-CAD as a concurrent, prescreening, and second reader in three representative countries. A scoping review was conducted to estimate the radiologist reading time with and without DL-CAD. The hourly cost of radiologist time was collected for the US, UK and Poland, and the monetary equivalent of saved time was calculated. The minimum number of screening CTs needed to reach break-even for a one-time investment for a DL-CAD was calculated.
**Results or Findings:** The mean reading time per case without DL-CAD was 2.5 minutes. It decreased by more than one minute when using DL-CAD as a concurrent and prescreening reader, respectively. It increased by about a half minute for DL-CAD as second reader. These reading times translated into costs of one to four euros per case for concurrent reading and one to six euro for prescreening reading. To reach break-even with a one-time investment for a DL-CAD, the minimum number of CT scans was about 12,000-54,000 for concurrent reader and 9,000-65,000 for prescreening reader.
**Conclusion:** Based on the current pricing, it is necessary for the per case cost to be significantly below €6 or for DL-CAD to be used in a high-workload setting to reach break-even in lung cancer screening. The use of DL-CAD as a prescreening reader has the greatest potential for cost savings.
**Limitations:** This study focused on the costs associated with DL-CAD, as it was beyond the study's scope to consider downstream costs related to diagnosis and treatment.
**Ethics committee approval:** Because this article does not contain any studies on human or animal subjects, ethics committee approval was not sought.
**Funding for this study:** This work is a part of NELCIN-B3 project. The NELCIN-B3 project is funded by The Royal Netherlands Academy of Arts and Sciences (Grant No. PSA_SA_BD_01) and Ministry of Science and Technology of the People's Republic of China, National Key R&D Program of China (Grant No. 2016YFE0103000).
**Author Disclosures:**
Yihui Du: Nothing to disclose
Geertruida De Bock: Nothing to disclose
Mathias Prokop: Nothing to disclose
Marcel Greuter: Nothing to disclose

### RPS 416-4

**A fully automated deep learning model based on multiparametric imaging for predicting tumour recurrence of locally advanced rectal cancer after neoadjuvant chemoradiotherapy: a multicentre study**
*Z. Liu*, Y. Sun, T. Tong; Shanghai/CN
*(21211230003@m.fudan.edu.cn)*

**Purpose:** The purpose of this study was to develop and validate a fully automated deep learning model based on multimodal MRI for DFS prediction of locally advanced rectal cancer (LARC) patients treated with neoadjuvant radiotherapy and chemotherapy (nCRT).
**Methods or Backround:** A total of 462 rectal cancer patients treated with nCRT from three centres were retrospectively enrolled, including clinical information, baseline multimodal MRI images (T2, ADC, Dapp, Kapp), and DFS data. The data from centres I and II were combined (373 cases) and randomly assigned to training, validation, and internal testing sets. The data from centre III were used as the external testing (89 cases). We developed a multitask joint survival model that simultaneously performed segmentation, risk classification, and survival prediction. These multitasks collaborated with each other, contributing to fully exploiting the key features of both imaging data and clinical data. Considering the multimodal input, an attention mechanism was introduced to efficiently capture useful information within and between modalities to minimise the impact of noise on experimental results. In addition, we experimented with conventional singletask models and compared the performance with our model.
**Results or Findings:** For risk classification, our model achieved significantly better performance than the singletask model, with an AUC of 0.960 vs 0.782 in the internal testing set, and remained at an AUC of 0.767 in the external testing set; for segmentation and survival prediction tasks, our model achieved a dice of 0.748 and a C-index of 0.731 in internal testing set, respectively.
**Conclusion:** Multitask deep learning model are expected to provide fully automated one-step prediction, contributing to optimising personalised treatment for LARC patients.
**Limitations:** Sample size were relatively small. Predictions were only made for DFS and not implemented for OS.
**Ethics committee approval:** No information provided by the submitter.
**Funding for this study:** Funding for this study was provided by the National Natural Science Foundation of China (81971687,82001776).
**Author Disclosures:**
Yiqun Sun: Nothing to disclose
Zonglin Liu: Nothing to disclose
Tong Tong: Nothing to disclose

### RPS 416-5

**Prognostic value of the consensus molecular subtype 4 (CMS4) predicted by multiparametric radiomics-based machine learning in colorectal cancer: a multicentre retrospective study**
*Z. Liu*, Y. Sun, T. Tong; Shanghai/CN
*(21211230003@m.fudan.edu.cn)*

**Purpose:** The consensus molecular subtype (CMS) is a novel classification system that reflects the genetic characteristics of the tumour. Among the four subtypes, CMS4 is associated with the worst prognosis. This study aimed to investigate whether a radiomics-based machine learning approach could predict CMS4 status in CRC patients.
**Methods or Backround:** A total of 228 CRC cases from three centres were retrospectively included. Cases from centre I were divided into training (138 cases) and validation sets (33 cases) in an 8:2 ratio; cases from centre II and III were combined as the external testing set (57 cases). Sequencing data and baseline MRI images, including T2-weighted (T2WI) and contrast-enhanced (CE) sequences, were available for each case. The sequencing data was input into the CMS classification system to generate CMS subtype outcomes. Radiomics features from the two sets were extracted with the same parameter settings. Several machine learning algorithms were applied in sample balance, feature normalisation, feature filters, and classifier construction to explore the best-performing and most robust model for CMS4 prediction. The rad-score for each patient was calculated by the T2WI and CE models separately. The combined model was established by applying logistic regression on the results of the above two models.
**Results or Findings:** We found that the CE model achieved better performance than the T2 model in both the test set (0.815 vs 0.790) and external validation set (0.741 vs 0.702). After merging the two models, the predictive performance of the Merged model was further improved, with the AUCs of 0.855 and 0.759 in the test set and external validation set.
**Conclusion:** Multiparametric radiomics-based machine learning shows promising potential in distinguishing CMS4 from other subtypes of CRC.
**Limitations:** The study's relatively small sample size as well as the manual delineation of the lesions' contours were identified as limitations.
**Ethics committee approval:** No information provided by the submitter.

**Funding for this study:** Funding for this study was provided by the National Natural Science Foundation of China (81971687,82001776).
**Author Disclosures:**
Yiqun Sun: Nothing to disclose
Zonglin Liu: Nothing to disclose
Tong Tong: Nothing to disclose

### RPS 416-6

**Interlesional morphological heterogeneity as a novel noninvasive prognostic biomarker**

*Z. Elkarghali*, M. Hattink, O. Maxouri, S. Rostami, D. I. Rodríguez Sánchez, S. Trebeschi, R. G. H. Beets-Tan; Amsterdam/NL
*(z.elkarghali@nki.nl)*

**Purpose:** Interlesional genetic heterogeneity is an established fact in tumour biology; however, performing a biopsy on every lesion is not feasible. For the first time in the literature, we explore the use of medical image analysis techniques to quantify morphological heterogeneity between lesions as a proxy for biological heterogeneity and analyse its value in stratifying patients based on prognosis.
**Methods or Backround:** We collected a diverse pancancer cohort of 1692 CE-CT scans with genetically proven diagnoses and performed complete 3D tumour segmentation. From each delineation (n=11,268 lesions), we derived radiomic features that fully characterise each lesion's morphology. We utilised seven similarity distance metrics (Euclidean, Chebyshev, City-Block, Minkowski, Correlation, Bray-Curtis, and Cosine) to measure the median morphological dissimilarity of lesions in a patient. Survival analysis (log-rank test) was performed to compare patients with high or low interlesional morphological heterogeneity (relative to the data set level median distance).
**Results or Findings:** We computed seven distance metrics for every combination of lesions within a patient and calculated the median as a patient level metric of morphological interlesional heterogeneity. Chebshev (X2=12.49, P =0.000408), City-Block (X2=12.08, P =0.000508), Euclidean (X2=11.64, P =0.000646), and Minkowski (X2=11.64, P=0.000646) distance measures could all strongly stratify patients into high- and low-risk groups. Cosine (X2=4.13, P=0.042) and correlation (X2=3.99, P=0.046) similarity metrics were also predictive to a lesser extent. Bray-Curtis distance measures could not significantly stratify patients (X2=3.35, P=0.067).
**Conclusion:** Interlesional morphological heterogeneity, as measured by radiomics and similarity distance metrics, strongly predicted overall survival.
**Limitations:** External validation has yet to be performed as a proof-of-concept study despite including over 1500 real-world cases.
**Ethics committee approval:** IRB approval was granted: IRBd19-147.
**Funding for this study:** No funding was provided for this study.
**Author Disclosures:**
Stefano Trebeschi: Nothing to disclose
Diana Ivonne Rodríguez Sánchez: Nothing to disclose
Zuhir Elkarghali: Nothing to disclose
Sajjad Rostami: Nothing to disclose
Regina G. H. Beets-Tan: Nothing to disclose
Olga Maxouri: Nothing to disclose
Merle Hattink: Nothing to disclose

### RPS 416-7

**Clinical assessment of deep learning reconstruction-based accelerated rectal MRI**

*W. Peng*, L. Wan, X. Tong, F. Yang, S. Wang, L. Li, H. Zhang; Beijing/CN
*(pengwenching@163.com)*

**Purpose:** The purpose of this study was to conduct a clinical assessment of deep learning reconstruction (DLR)-based rectal MRI in comparison to standard MRI.
**Methods or Backround:** Patients with biopsy-proven rectal adenocarcinoma from November/2022 to May/2023 were prospectively enrolled in the study to undertake rectal MRI, including protocols using standard fast spin-echo (FSEstandard) and DLR-based accelerated FSE (FSEDL). Imaging quality including signal-noise ratio (SNR), contrast-noise ratio (CNR), as well as subjective scoring based on Likert scale were assessed by two radiologists. Diagnostic performance including T-staging, N-staging, EMVI, and MRF was further evaluated by five radiologists. The time consumed in the application of each diagnostic metric was documented for reading efficiency analysis.
**Results or Findings:** In total, 117 patients (77 males; age range 21 – 77 years) were enrolled in the study; 60 patients undertook radical surgery. DLR enabled a reduction of 65% in acquisition time. Moderate to excellent intra- and interreader agreement was achieved for all assessment metrics. FSEDL exhibited higher SNR, CNR, and subjective scores in noise, tumour margin clarity, visualisation of bowel wall layering and rectal mesorectal fascia, overall image quality, and diagnostic confidence (P < 0.05). FSEDL was rated higher T-staging accuracy by junior readers (reader 1, 58% vs 70%, P = 0.016; reader 3, 60% vs 76%, P = 0.021), with comparable performance in evaluating N-staging, EMVI, and MRF. No difference was found concerning diagnostic performance by senior readers (P > 0.05). FSEDL exhibited shorter diagnostic time in T-staging and overall evaluation by all readers, as well as in EMVI and MRF by junior readers (P < 0.05).
**Conclusion:** FSEDL is clinically feasible for rectal MRI, which could facilitate improved image quality and reading efficiency than FSE standard, while reducing 65% acquisition time. Moreover, it has potential in helping junior radiologists improve the accuracy of T-staging.
**Limitations:** This was a single-centre study.
**Ethics committee approval:** This prospective study received approval from our institutional review board, and written informed consent was obtained from all participants.
**Funding for this study:** Funding was received from the CAMS Innovation Fund for Medical Sciences (CIFMS) [grant number 2021-I2M-C&T-A-017], Capital's Funds for Health Improvement and Research (CFH) [grant number 2022-2-4024], the National Natural Science Foundation of China [grant number 81971589], 2020 SKY Imaging Research Fund [grant number Z-2014-07-2003-01], and CAMS Innovation Fund for Medical Sciences (CIFMS) [grant number 2022-I2M-C&T-B-077].
**Author Disclosures:**
Lijuan Wan: Nothing to disclose
Fan Yang: Nothing to disclose
Sicong Wang: Employee: GE Healthcare China
Lin Li: Nothing to disclose
Xiaowan Tong: Nothing to disclose
Hongmei Zhang: Nothing to disclose
Wenjing Peng: Nothing to disclose

### RPS 416-8

**Radiomic analysis of PI-RADS 4 and 5 lesions detected on 3T mpMRI: role in the diagnosis of clinically significant prostate cancer**

*P. A. Bonaffini*[1], A. Corsi[2], R. Muglia[1], G. Perugini[3], M. Roscigno[3], L. F. Da Pozzo[3], P. Marra[1], S. Sironi[1]; [1]Monza/IT, [2]Liege/BE, [3]Bergamo/IT
*(pa.bonaffini@gmail.com)*

**Purpose:** The purpose of this study was to identify radiomic features potentially supporting the detection of clinically significant prostate cancer (csPC) in PI-RADS 4/5 lesions detected on 3T multiparametric MRI (mpMRI) studies.
**Methods or Backround:** We retrospectively enrolled patients who underwent a 3T mpMRI (June 2016-March 2021) and with at least one PI-RADS 4-5 lesion (PI-RADS v2.1). Final pathological findings from fusion MRI-targeted biopsies served as ground truth. Clinical (age, PSA, PSA density) and MRI conventional parameters (prostate volume, mean ADC in circular 2D ROI) were collected. Included lesions were manually contoured on itk-SNAP on ADC maps, and axial T2 images; volumes of interest were also obtained. Radiomic features were extracted using Pyradiomics. Clinical and radiomic features best correlating with final histological results were selected. All models were assessed through 100 repetitions using 5-fold cross-validation. Sensitivity and specificity were assessed on validation samples.
**Results or Findings:** Among 945 patients who had undergone prostate mpMRI within the study period, 99 patients (median age 69 years) with 111 PI-RADS 4-5 lesions met the inclusion criteria. At the end of the histopathological analysis, 79 lesions (71%) were found to be csPC (GS≥7). The best predicting clinical (PSA density) and radiomic (ADC-wavelet-LLL_glrlm_Long Run High Gray Level Emphasis/texture feature, T2-wavelett-HHH_glszm_Gray Level Variance and T2-wavelet-LLL_glszm_Gray Level Variance/heterogenicity feature) multivariate model for PI-RADS 4-5 lesions obtained 79% sensitivity, 80% specificity, 91% PPV, 63% NPV and 79% accuracy. When considering only peripheral zone lesions, a multivariate model with only the same radiomic features gained 86% sensitivity, 80% specificity, 93% PPV, 70% NPV and 84% accuracy.
**Conclusion:** Texture and heterogeneity features extracted from 3T mpMRI T2 and ADC sequences may improve the detection of csPC in PI-RAD 4 and 5 lesions, demonstrating a better performance when considering only peripheral zone lesions and correlating also with PSA density in the zone-ignorant model.
**Limitations:** This was a monocentric retrospective study. The sample size was limited.
**Ethics committee approval:** This study was conducted following the Declaration of Helsinki. Patients' anonymity was granted.
**Funding for this study:** No funding was received for this study.
**Author Disclosures:**
Sandro Sironi: Nothing to disclose
Marco Roscigno: Nothing to disclose
Paolo Marra: Nothing to disclose
Pietro Andrea Bonaffini: Nothing to disclose
Giovanna Perugini: Nothing to disclose
Riccardo Muglia: Nothing to disclose
Luigi Filippo Da Pozzo: Nothing to disclose
Andrea Corsi: Nothing to disclose

### RPS 416-9

**Imaging derived biomarkers integrated with clinical and laboratory values predict recurrence of hepatocellular carcinoma after liver transplantation**

*T. P. T. Hoang*[1], P. Schindler[2], N. Börner[1], M. Masthoff[2], M. Seidensticker[1], J. Ricke[1], M. Ingrisch[1], O. Öcal[1], M. Wildgruber[1]; [1]Munich/DE, [2]Münster/DE

**Purpose:** The purpose of this study was to investigate the prognostic value of computed tomography (CT) derived imaging biomarkers in hepatocellular carcinoma (HCC) recurrence after liver transplantation (LT) and develop a predictive nomogram model.
**Methods or Backround:** This retrospective study included 178 patients with histopathologically confirmed HCC who underwent liver transplantation between 2007 and 2021 at the two academic liver centres. We evaluated dedicated imaging features from baseline multiphase contrast-enhanced CT supplemented by several clinical findings and laboratory parameters. Time-to-recurrence (TTR) was estimated by Kaplan–Meier analysis. Univariable Cox proportional hazard regression and multivariable least absolute shrinkage and selection operator (LASSO) regression were used to identify independent prognostic factors for recurrence. A nomogram model was then built based on the independent factors selected through LASSO regression, to predict the probabilities of HCC recurrence at one, three, and five years.
**Results or Findings:** The rate of HCC recurrence after LT was 17.4% (31 of 178). The LASSO analysis revealed six independent predictors associated with an elevated risk of tumour recurrence. These predictors included the presence of peritumoural enhancement, the presence of over three tumour lesions, the largest tumour diameter exceeding 3 cm, serum alpha-fetoprotein (AFP) levels surpassing 400 ng/mL, and the presence of a tumour capsule. Conversely, a history of bridging therapies was found to be correlated with a reduced risk of HCC recurrence. In addition, Kaplan-Meier analysis with log-rank test showed patients with irregular margins, satellite nodules, or small lesions displayed significantly shorter time-to-recurrence. Our nomogram demonstrated good performance, yielding a C-index of 0.835 and AUC values of 0.86, 0.88, and 0.85 for the predictions of 1-year, 3-year, and 5-year TTR, respectively.
**Conclusion:** Imaging parameters derived from baseline contrast-enhanced CT showing malignant characteristics and aggressive growth patterns, along with serum AFP and a history of bridging therapies, can serve as biomarkers for predicting HCC recurrence after transplantation.
**Limitations:** First, this study has a limited sample size. Second, patients with various types of bridging therapies have been included. Although this situation causes inhomogeneity, it reflects the daily clinical routine of large transplantation centres.
**Ethics committee approval:** This study was approved by an ethics committee; the notification can be found under the number 22-0110.
**Funding for this study:** No funding was provided for this study.
**Author Disclosures:**
Osman Öcal: Nothing to disclose
Nikolaus Börner: Nothing to disclose
Max Seidensticker: Nothing to disclose
Philipp Schindler: Nothing to disclose
Thi Phuong Thao Hoang: Nothing to disclose
Michael Ingrisch: Nothing to disclose
Moritz Wildgruber: Nothing to disclose
Max Masthoff: Nothing to disclose
Jens Ricke: Nothing to disclose

### RPS 416-10

**CT texture analysis as a predictor for the genetic profile of mass-forming intrahepatic cholangiocarcinoma**

*A. Ammirabile*[1], L. Viganò[1], V. Zanuso[1], F. Fiz[2], M. E. Laino[1], M. Francone[1], M. Sollini[1], L. Rimassa[1]; [1]Milan/IT, [2]Genoa/IT
*(angela.ammirabile@humanitas.it)*

**Purpose:** Intrahepatic cholangiocarcinoma (ICC) is an aggressive disease with increasing incidence. Comprehensive molecular profiling has shown genetic alterations that could be the target of systemic therapies. Texture analysis of imaging has led to a reliable prediction of pathology data. This study investigates whether CT-based radiomics can non-invasively predict genetic alterations in ICC.
**Methods or Backround:** All consecutive patients eligible for systemic therapy for a mass-forming ICC (01/2016-06/2022) were considered. Inclusion criteria were: the availability of a contrast-enhanced CT at diagnosis before any treatment with an adequate quality of the portal phase for textural analyses, complete molecular profiling by NGS or FISH evaluation for FGFR2 gene fusion/rearrangement. Genetic analyses were performed on surgical specimen or biopsy. The tumour was manually segmented and radiomic features were automatically extracted using the LifeX software. Predictive models were built considering clinical and radiomic data.
**Results or Findings:** 90 patients were enrolled (58 NGS,32 FISH) with a median age of 65 years. The most common genetic alterations were FGFR2 (20/90), IDH1-2 (12/58), KRAS (9/58). The performances of the predictive models for FGFR2 and IDH1-2 improved by adding radiomic features to clinical data, reaching a C-index of 0.892 (vs 0.800 of the clinical model) and 0.811 (vs 0.670), respectively, at internal validation. The pure radiomic model for the prediction of KRAS mutations achieved a C-index of 0.862 at internal validation (vs 0.660 of the pure clinical model) without further improvements with the addition of clinical features.
**Conclusion:** The radiomic features extracted from CT at ICC diagnosis can potentially provide a reliable noninvasive prediction of its genetic status with a major impact on therapeutic strategies.
**Limitations:** The limitations of the study are its retrospective nature, lack of external validation as well as the commonest mutations being the subject of analysis.
**Ethics committee approval:** The local review board of each centre approved the study protocol (coordinating centre approval: protocol number 142/21, date of approval 17/03/2021). Because of the retrospective nature of the study, the need for specific informed consents was waived.
**Funding for this study:** Funding was provided by the AIRC grant #2019–23822.
**Author Disclosures:**
Lorenza Rimassa: Nothing to disclose
Angela Ammirabile: Nothing to disclose
Valentina Zanuso: Nothing to disclose
Martina Sollini: Nothing to disclose
Maria Elena Laino: Nothing to disclose
Francesco Fiz: Nothing to disclose
Marco Francone: Nothing to disclose
Luca Viganò: Nothing to disclose

### RPS 416-11

**CT-based radiomics of cholangiocarcinoma and peritumoural tissue improves survival prediction: development of a clinical-radiomic model**

*A. Ammirabile*[1], F. Fiz[2], S. Langella[3], M. Serenari[4], M. Sollini[1], A. Chiti[1], G. Torzilli[1], F. Leva[1], L. Viganò[1]; [1]Milan/IT, [2]Genoa/IT, [3]Turin/IT, [4]Verona/IT
*(angela.ammirabile@humanitas.it)*

**Purpose:** In patients affected by intrahepatic cholangiocarcinoma (ICC), the prediction of survival based on morphological and clinical parameters has limited reliability. The present study aims to elucidate if the textural features of ICC and its peritumoural tissue extracted from preoperative computed tomography (CT) improve the prediction of survival after resection.
**Methods or Backround:** All consecutive patients undergoing resection for ICC at six high-volume centres (2009-2019) were considered. The arterial and portal phases of CT performed <60 days before surgery were analysed. The tumour was manually segmented (tumour-VOI), a 5-mm automatic volume expansion was applied to encompass the peritumoural tissue (margin-VOI). The radiomic features were automatically extracted by the LifeX software. For overall and progression-free survival (OS/PFS), we considered pre- and post-operative predictive models, based on clinical data and radiomic features from portal and arterial phases.
**Results or Findings:** 215 patients were included (median age 67.5 years). The three-year OS/PFS rates were 57.0% and 34.9% (median follow-up 28 months). The predictive model of OS based on clinical variables had a C-index of 0.681. The performance progressively improved by adding the radiomic features: C-index =0.710 including portal tumor-VOI, C-index =0.752 including portal tumour-Margin-VOI; C-index=0.764 including all arterial and portal VOIs. The latter model retained clinical variables (CA19-9, tumour pattern), tumour features (density, homogeneity, GLRLM indices), and margin data (kurtosis, compacity, shape). The model had a performance equivalent to the post-operative clinical model including the pathology data (C-index =0.765). The same results were observed for PFS.
**Conclusion:** CT-based radiomics of ICC and peritumoural tissue improves prediction of survival, and, in combination with clinical data, leads to a preoperative estimation of outcome equivalent to the post-operative one.
**Limitations:** The limitations of the study are its retrospective nature, lack of external validation as well as the absence of radiomic features from CT late-phase.
**Ethics committee approval:** The local review board of each centre approved the study protocol (coordinating centre approval: protocol number 142/21, date of approval 17/03/2021). Because of the retrospective nature of the study, the need for specific informed consent was waived.
**Funding for this study:** Funding was provided by the AIRC grant #2019–23822.
**Author Disclosures:**
Matteo Serenari: Nothing to disclose
Francesca Leva: Nothing to disclose
Angela Ammirabile: Nothing to disclose
Serena Langella: Nothing to disclose
Guido Torzilli: Nothing to disclose
Martina Sollini: Nothing to disclose
Francesco Fiz: Nothing to disclose
Arturo Chiti: Nothing to disclose
Luca Viganò: Nothing to disclose

### RPS 416-12
**Radiomics in colon cancer: how to identify high-risk patients**
*M. Polici*, D. Valanzuolo, D. Pugliese, G. Tremamunno, F. Palmeri, M. Zerunian, D. De Santis, D. Caruso, A. Laghi; Rome/IT
*(policimichela28@gmail.com)*

**Purpose:** The study aimed to develop a radiomic model able to identify high-risk colon cancer by analysing properative CT scans.
**Methods or Backround:** The study population included 300 patients with nonmetastatic colon cancer were retrospectively enrolled from January 2015 to June 2020. The population was divided into two groups, high-risk and no-risk, following the presence of at least one high-risk clinical factor between staging T4, LVI, PNI, budding, and nodal metastases. All patients had baseline CT scans, and 3D cancer segmentation was performed on the portal phase by two expert radiologists using open-source software (3DSlicer v4.10.2). Among the 107 radiomic features extracted, stable features were selected to evaluate the interclass correlation (ICC) (cut-off ICC > 0.8). Stable features were compared between the two groups (T-test or Mann–Whitney), and the significant features were selected for univariate and multivariate logistic regression to build a predictive radiomic model. Furthermore, survival analyses were performed with Kaplan-Meier curves, with progression within 24 months considered as end-point.
**Results or Findings:** In total, 210/300 were classified as high-risk and 90/300 as no-risk. A total of 27 radiomic features were stable (0.80 ≤ ICC < 0.92). Among these, 15 features were significantly different between the two groups ($P < 0.05$), and only eight features were selected to build the radiomic model. The radiomic model yielded an AUC of 0.73. Three radiomic features demonstrated correlation with progressive disease in Kaplan-Meier curves.
**Conclusion:** In conclusion, the radiomic model could be seen as a performant, non-invasive imaging tool to properly stratify colon cancers with high-risk disease especially in preoperative setting.
**Limitations:** The retrospective nature of the study was identified as a limitation; the study populations were unbalanced.
**Ethics committee approval:** Written informed consent was acquired for all patients and Institutional Review Board approval was obtained.
**Funding for this study:** No funding was received for this study.
**Author Disclosures:**
Damiano Caruso: Nothing to disclose
Marta Zerunian: Nothing to disclose
Daniela Valanzuolo: Nothing to disclose
Giuseppe Tremamunno: Nothing to disclose
Domenico De Santis: Nothing to disclose
Federica Palmeri: Nothing to disclose
Dominga Pugliese: Nothing to disclose
Andrea Laghi: Nothing to disclose
Michela Polici: Nothing to disclose

**13:00-14:30** **Research Stage 3**

### Research Presentation Session: Genitourinary

### RPS 407
### Kidney and bladder imaging

**Moderator**
R. Girometti; Udine/IT
*(rgirometti@sirm.org)*
**Author Disclosures:**
Rossano Girometti: Board Member: European Radiology

### RPS 407-2
**Validation of the bladder cancer “MRI Pathway”: results from a multicentre prospective study**
*E. Messina*, A. Dehghanpour, A. Borrelli, S. Lucciola, C. Catalano, V. Panebianco; Rome/IT
*(emanuele.messina@uniroma1.it)*

**Purpose:** The validate a new bladder cancer (BCa) diagnostic pathway focusing on magnetic resonance imaging (MRI). Moreover, to identify clinical, pathological, and radiological characteristics, including the Vesical Imaging-Reporting and Data System (VI-RADS) score, individually demonstrating a significant correlation with muscle-invasive BCa (MIBC).
**Methods or Backround:** Prospective multicentres study (four centres). Patients with BCa suspicion underwent MRI before trans-urethral resection of bladder tumour (TURBT). Two experienced uro-radiologists in each centre independently analysed MRIs. According to VI-RADS, a cutoff of ≥ 3 or ≥ 4 was assumed to define MIBC. TURBT and/or cystectomy reports were compared with preoperative VI-RADS scores to assess MRI accuracy for discriminating between non-muscle-invasive versus MIBC. Performance was assessed by ROC curve analysis. Univariable and multivariable logistic regression models were implemented.
**Results or Findings:** A final cohort of 251 patients was enrolled (median age 73 [IQR: 64, 77.5]). MRI showed sensitivity, specificity, PPV, NPV, and accuracy for MIBC detection ranging from 83-93%, 80-92%, 67-81%, 93-96%, and 84-89% for the more experienced readers. The area under the curve (AUC) was 0.95 (0.91-0.99). In the multivariable logistic regression model, haematuria ($p = 0.02$), tumour size ($p=0.03$), the VI-RADS score, using both a cutoff of 3 and 4 ($p<0.0001$), and concomitant hydronephrosis ($p=0.02$) were the variables correlating with a bladder cancer staged as ≥ T2. The inter-reader agreement was substantial (k=0.85).
**Conclusion:** VI-RADS score has been validated as a reliable preoperative tool predicting BCa muscle-invasiveness. Moreover, if integrated as an MRI biomarker with clinical and imaging data, VI-RADS can be integrated into a customised diagnostic approach to select patients more precisely, with the goal of reducing the risk of diseases’ understaging and enhancing clinical outcomes.
**Limitations:** High-volume referral centres, reduced reproducibility limit this study.
**Ethics committee approval:** Institutional Ethics Committee approved this study.
**Funding for this study:** Not applicable for this study.
**Author Disclosures:**
Sara Lucciola: Nothing to disclose
Valeria Panebianco: Nothing to disclose
Antonella Borrelli: Nothing to disclose
Emanuele Messina: Nothing to disclose
Ailin Dehghanpour: Nothing to disclose
Carlo Catalano: Nothing to disclose

### RPS 407-3
**Contrast-enhanced CT radiomic analysis for the preoperative prediction of pathological T3a upstaging in renal cell carcinoma**
*E. Yuan*[1], Y. Chen[1], B. Song[1], J. Yao[1], H. Yin[2], H. Zhang[2]; [1]Chengdu/CN, [2]Beijing/CN
*(yuanenyu_radiology@163.com)*

**Purpose:** The study aims to develop and temporally validate the radiomic models for identifying pathological T3a upstaging in renal cell carcinoma.
**Methods or Backround:** A total of 1003 patients with renal cell carcinoma in our hospital were retrospectively enrolled. The patients were assigned to development dataset (n = 729) and temporal validation dataset (n = 274). The pathological T3a status of each patient was pathologically confirmed. Radiomic features of the lesion and the 5 mm peritumoural area were extracted from the manually labeled portal venous phase CT images. Tumour model, peritumour model, and combine model were developed using tumour, peritumour, and both features. The modeling pipelines were internally validated using 1000*100 nested cross validation, and the final models were temporally validated in an independent temporal validation set. The model in each experiment was evaluated by using metrics of discrimination, calibration, and clinical utility. The frequencies of features being selected were also analysed.
**Results or Findings:** The tumour shape based feature "sphericity" was the most frequently selected feature with the largest coefficient in the final models. In internal validation, the tumour pipeline and combine pipeline showed similar discrimination (AUC = 0.8362±0.0327 vs. 0.8347±0.0331), calibration, and clinical utility, while the peritumour pipeline showed slightly but signification poor discrimination (AUC = 0.7994±0.0369), calibration, and clinical utility than the other two. In temporal validation, the tumour model, peritumour model, and combine model showed similar discrimination (AUC = 0.8457 vs. 0.8489 vs. 0.8426), calibration, and clinical utility.
**Conclusion:** The radiomic models showed favorable performance in predicting pathological T3a upstaging preoperatively in renal cell carcinoma patients.
**Limitations:** This is a single centre study. The models needs further multicentre and prospective validation.
**Ethics committee approval:** The written consent was waived because of the retrospective design.
**Funding for this study:** No funding was received for this study.
**Author Disclosures:**
Jin Yao: Nothing to disclose
Huiling Zhang: Nothing to disclose
Hongkun Yin: Nothing to disclose
Enyu Yuan: Nothing to disclose
Bin Song: Nothing to disclose
Yuntian Chen: Nothing to disclose

### RPS 407-4

**Neoadjuvant chemotherapy VI-RADS (nacVI-RADS) for the assessment of tumour response to neoadjuvant immunotherapy in muscle-invasive bladder cancer**

G. Brembilla, *M. Cosenza*, T. Russo, G. Basile, D. Raggi, C. Mercinelli, F. Montorsi, A. Necchi, F. De Cobelli; Milan/IT
*(cosenza.michele@hsr.it)*

**Purpose:** The aim of this study was to investigate the diagnostic accuracy and reproducibility of the neoadjuvant chemotherapy VI-RADS (nacVI-RADS) in assessing MIBC response to neoadjuvant immunotherapy.
**Methods or Backround:** Two hundred and twenty bladder MRI scans from 110 patients who underwent pre- and post-immunotherapy MRI prior to radical cystectomy (RC) were retrospectively reviewed by two readers using nacVI-RADS scores. The diagnostic accuracy of nacVI-RADS was assessed using histopathology of RC specimens as the standard of reference, relative to two endpoints: complete pathologic response (ypT0) and downstaging (≤ypT1). The threshold for MRI positivity was considered nacVI-RADS ≥3; for downstaging, an additional threshold of nacVI-RADS ≥4 was also tested. Interobserver agreement was assessed with agreement coefficient 1 (AC1), Cohen's k coefficient and percentages of agreement.
**Results or Findings:** Complete pathologic response was observed in 42% of patients. Complete radiologic response (i.e.: nacVI-RADS 1-2) was reported in 35.5% and 49.9% by reader 1 and reader 2, respectively. nacVI-RADS ≥3 had a sensitivity and NPV for >ypT0 residual disease of 82% (95%CI: 74-88) and 74% (95%CI: 64-83), respectively; for >ypT1 residual disease, sensitivity and NPV were 97% (95%CI: 90-99) and 97% (95%CI: 91-99), respectively. For the assessment of residual >ypT1 disease, a potentially better tradeoff between sensitivity, specificity and NPV was obtained for higher positivity thresholds of MRI. nacVI-RADS had an AUC for ypT0 of 79.6%, for ≥ypT1 of 86.3%. Interreader agreement was substantial regardless the MRI positivity threshold (AC1 0.65-0.68; k 0.66-0.67).
**Conclusion:** nacVI-RADS criteria showed good accuracy and reproducibility in assessing MIBC response to neoadjuvant treatments.
**Limitations:** Single centre, experienced readers limit this study.
**Ethics committee approval:** This study was approved by the Ethics Committee of the Fondazione IRCCS Istituto Nazionale dei Tumori, Milan, Italy.
**Funding for this study:** Funding for this research was provided by Merck Sharp & Dohme LLC, a subsidiary of Merck & Co., Inc., Rahway, NJ, USA, and Associazione Italiana per la Ricerca sul Cancro (AIRC), Grant number: MFAG: 2017 Id.20617.
**Author Disclosures:**
Francesco Montorsi: Nothing to disclose
Chiara Mercinelli: Nothing to disclose
Daniele Raggi: Nothing to disclose
Giuseppe Basile: Nothing to disclose
Michele Cosenza: Nothing to disclose
Giorgio Brembilla: Nothing to disclose
Francesco De Cobelli: Nothing to disclose
Tommaso Russo: Nothing to disclose
Andrea Necchi: Advisory Board: MSD, Roche, Bayer, AstraZeneca, Clovis Oncology, Janssen, Incyte, Seattle Genetics/Astellas, Bristol-Myers Squibb, Rainier Therapeutics, Bycicle Therapeutics, GlaxoSmithKline, Basilea Pharmaceutica, Catalym; Research/Grant Support: MSD, AstraZeneca, Ipsen, Gilead

### RPS 407-5

**Development and validation of a diagnostic model based on contrast-enhanced CT to identify clear cell renal cell carcinoma in solid small renal masses: a multicentre study**

*J. Han*, Y. Tao, Y. Zhang; Zhuhai/CN
*(handaile@163.com)*

**Purpose:** The aim of this study was to develop and validate a diagnostic model based on contrast-enhanced CT for identifying clear cell renal cell carcinoma (ccRCC) in small renal masses (SRMs).
**Methods or Backround:** This retrospective multicentre study enrolled patients with pathologically confirmed SRMs. Data from three centres were used as training set (n=229), with data from one centre serving as an independent external validation set (n=81). Univariate and multivariate logistic regression analyses were used to screen independent risk factors for ccRCC and build the classification and regression tree (CART) model. Three radiologists were asked to diagnose the SRMs in each case independently based on professional experience and re-evaluated using the CART model.
**Results or Findings:** A total of 71% (220/310) of SRMs were ccRCC. Enhancement pattern, early dark cortical band (EDCB), the ratio of lesion to normal cortex attenuation (L/C) in the corticomedullary phase, non-enhancing phase L/C and sex were used to develop the CART model. In the testing cohort, the AUC and accuracy of the CART model were 0.903, 85.1%. The accuracy of radiologists was 67.9%,58.0%, and 56.8%. With the CART model support, the accuracy of radiologists improved to 86.4%, 84.0%, 79.0%. Interobserver agreement was significantly improved with the use of model aids (0.323 vs 0.654, P＜0.001).
**Conclusion:** The CART diagnostic model can identify ccRCC in SRMs and help radiologists make the diagnosis, potentially reducing the number of unnecessary biopsies.
**Limitations:** First, since it was a retrospective study, the existing selection bias may affect the results, and further prospective verification is needed in future work. Second, the data were collected from different institutions, and the scanning protocols were different. To minimise the impact of internal and external factors on the results, the quantitative data were standardised, and an independent external validation set was established.
**Ethics committee approval:** This study was approved by the institutional review boards of the Fifth Hospital of Sun Yat- sen University.
**Funding for this study:** This work was supported in part by the National Key Research and Development Program of China under Grant Nos. 2023YFE0204300, in part by the National Natural Science Foundation of China under Grant No: 81801809, 82371917, 81830052, 81971691, 12126610, 62371476; in part by the Guangzhou Technology Program of Agriculture and Social Development of Key Research and Development Scheme under Grant No: 2023B03J1237, in part by the Basic and Applied Basic Research Foundation of Guangdong Province under Grant No: 2020A1515010572, and in part by the Zhuhai Basic and Applied Basic Research Foundation under Grant No: ZH22017003200001PWC.
**Author Disclosures:**
Yaqin Zhang: Nothing to disclose
Jiayue Han: Nothing to disclose
Yuxi Tao: Nothing to disclose

### RPS 407-6

**Comparison of photon-counting and energy-integrating detector CT systems for the characterisation of cystic renal lesions on virtual non-contrast imaging**

*L. Lofino*, F. Schwartz, M. Al Tarhuni, A. Abadia, F. Ria, E. Samei, D. Marin; Durham, NC/US
*(ludovica.lofino@humanitas.it)*

**Purpose:** The study aims to compare the absolute CT attenuation errors of cystic renal lesions and abdominal organs on virtual non-contrast images (VNC) between photon-counting (PCCT) and energy-integrating detector CT (EID).
**Methods or Backround:** In this HIPAA compliant, IRB-approved retrospective study, multiphase CT scans from one PCCT and two EID dual-source dual-energy CTs were retrieved. A total of 56 BMI-matched patients (26 women, 30 men; mean age 58.5 ± 15.3 years; mean BMI 29.0 ± 6.8 kg/m2), were included: 16 for PCCT and 20 each per EID systems. Attenuation measurements of abdominal organs were recorded on VNC and True Noncontrast (TNC) datasets. Furthermore, attenuation measurements of 16 cystic renal lesions (eight for PCCT and eight for EID) were compared on VNC and TNC. Absolute CT attenuation errors were calculated and compared between PCCT and EID for the entire population and a subset of 20 obese patients using paired t-tests. Absolute CT attenuation errors were also compared for all cystic renal lesions and for renal lesions <1 cm, separately.
**Results or Findings:** PCCT yielded significantly lower absolute CT attenuation errors than EID on VNC for spleen (2.6 ± 6.2 vs 8.0 ± 10.3) and pancreas (4.4 ± 1.8 vs 7.7 ± 9.7) for all patients and for liver, spleen and pancreas in the obese patient cohort (P<0.05). Furthermore, PCCT yielded significantly lower absolute CT attenuation errors compared to EID for all cystic renal lesions (2.0 ± 1.3 vs. 12.0 ± 8.9) and for renal lesions <1 cm (1.4 ± 0.9 vs. 19.1 ± 6.8), P<0.01.
**Conclusion:** PCCT has significantly lower absolute CT attenuation errors for abdominal organs and cystic renal lesions in VNC images, compared to two dual-energy EID CTs.
**Limitations:** Single centre, retrospective study with limited number of cases.
**Ethics committee approval:** Ethics comittee approved the study.
**Funding for this study:** No funding was required for this study.
**Author Disclosures:**
Ludovica Lofino: Nothing to disclose
Andres Abadia: Employee: Siemens Healthineers, USA
Mohammed Al Tarhuni: Nothing to disclose
Fides Schwartz: Nothing to disclose
Daniele Marin: Research/Grant Support: Siemens Healthineers, USA
Ehsan Samei: Nothing to disclose
Francesco Ria: Nothing to disclose

### RPS 407-7

**Diagnostic accuracy and inter-reader agreement of nac-VIRADS scoring system: a prospective validation study**

*A. Dehghanpour*, E. Messina, A. Borrelli, S. Lucciola, C. Catalano, V. Panebianco; Rome/IT
*(ad48ad@yahoo.com)*

**Purpose:** Recently a novel scoring system named nac-VIRADS (neoadjuvant chemotherapy VI-RADS) was proposed to assess radiological response to chemotherapy in MIBC (muscle-invasive bladder cancer) patients using mpMRI. The primary aim of this study was to validate the performance of nac-VIRADS scoring system and assess its inter-reader agreement. Additionally, to investigate the radiological and clinicopathological features independently correlating with response to systemic therapy.
**Methods or Backround:** This prospective multicenter study included patients with non-metastatic MIBC who underwent trans-urethral resection of bladder tumour (TURBT) and/or repeated TUR, followed by neoadjuvant chemotherapy (NAC), radical cystectomy (RC), and extended pelvic lymph node dissection. Patients underwent pre- and post-treatment multiparametric MRI. Radiological response was evaluated by two experienced urogenital radiologists using nac-VIRADS scoring system. The reference standard was histopathologic reports and tumour regression grade (TRG) from RC. Nac-VIRADS performance was assessed by means of ROC analysis, deriving sensitivity, specificity, PPV, NPV, and accuracy. Interreader agreement was determined with Cohen's k statistics. Univariable and multivariable analyses were implemented.
**Results or Findings:** Fifty-five patients were included. n=13 were classified as nac-VIRADS 1-2, n=14 as nac-VIRADS 3, n=22 as nac-VIRADS 4, and n=6 as nac-VIRADS 5. Overall, nac-VIRADS score showed a high diagnostic performance in concordance with histological results expressed by TRG. Diagnostic performance showed a range of 86%-92% sensitivity, 71%-79% specificity, 80%-82% (PPV, and 79%-83% NPV. AUC was 0.92 (95% CI: 0.83–1) for the more experienced reader. Inter-reader agreement was almost perfect (K = 0.82).
**Conclusion:** nac-VIRADS scoring system offers a reliable and reproducible approach, employing a clear algorithm, to assess response to systemic therapy in MIBC patients. Its implementation in a standardised pathway has the potential to significantly influence therapeutic decision-making and enhance overall patient survival.
**Limitations:** Limited sample size limits the scope of this study.
**Ethics committee approval:** The study was approved by the Institutional Ethical Committee.
**Funding for this study:** No funding was received for this study.
**Author Disclosures:**
Sara Lucciola: Nothing to disclose
Valeria Panebianco: Nothing to disclose
Antonella Borrelli: Nothing to disclose
Emanuele Messina: Nothing to disclose
Ailin Dehghanpour: Nothing to disclose
Carlo Catalano: Nothing to disclose

### RPS 407-8

**Novel CT-derived biomarkers for prediction of renal split function in preoperative nephrectomy planning**

*C. O. Page*, D. Ap Dafydd, C. Kelly-Morland, W. Cazzaniga, D. Nicol, D. Levine, S. J. Withey; London/UK
*(christopher.page2@nhs.net)*

**Purpose:** The aim of this study was to investigate the feasibility of utilising preoperative contrast-enhanced CT to estimate renal split function, compared to standard-of-care nuclear medicine studies (DMSA and MAG3) in preoperative planning for nephrectomy.
**Methods or Backround:** Retrospective analysis was conducted on patients with either DMSA- or MAG3-derived estimates of split renal function (NM-SRF) and a contemporaneous contrast-enhanced CT scan. Patients with hydronephrosis were excluded. Using reconstruction software with density thresholding (Syngo.via, Siemens Healthineers), renal volumes and mean attenuation values were calculated. The product of these from each kidney was used to calculate a CT-derived estimated split renal function (CT-SRF). The estimated percentage renal function from the right kidney from NM-SRF and CT-SRF were compared using Pearson correlation coefficient. The estimated remaining renal function following nephrectomy using NM-SRF and CT-SRF was compared to actual GFR values obtained >3 months post-operative. The difference between estimated and true post-operative GFR values were compared using Student's T-test.
**Results or Findings:** n=49 patients (mean age 65.8 ± 9.3 years; 73.4% male) were included in the initial analysis. The Pearson correlation coefficient between CT-SRF and NM-SRF was 0.835, indicating a very strong correlation (p=<0.0001). For n=28 patients, post-operative renal function (>3 months following the date of nephrectomy) was available. CT-SRF and NM-SRF underestimated post-operative GFR by 12.1(±11.9) and 10.5(±12.2) ml/min/1.73 m2, respectively. There was no significant difference in performance between these tests (p=0.604). Time to calculate CT-SRF was 157±68 seconds.
**Conclusion:** CT-SRF was a time-efficient and feasible way of estimating split renal function with comparable performance to standard-of-care NM-SRF in this small retrospective cohort.
**Limitations:** Larger prospective studies are required to assess whether it is possible to use the available CT data to avoid the additional radiation exposure, hospital attendance, and cost associated with DMSA and MAG3 testing.
**Ethics committee approval:** Local institutional board review approved this study.
**Funding for this study:** No funding was obtained for this study.
**Author Disclosures:**
Samuel Joseph Withey: Nothing to disclose
Derfel Ap Dafydd: Nothing to disclose
David Nicol: Nothing to disclose
Walter Cazzaniga: Nothing to disclose
Christian Kelly-Morland: Nothing to disclose
Daniel Levine: Nothing to disclose
Christopher Owen Page: Nothing to disclose

### RPS 407-9

**Is the R.E.N.A.L. nephrometry score useful for predicting outcomes in percutaneous ablation of renal masses?**

*M. Poida*, M. Paniagua González, M. Camarena Gea, C. Gerlotti Slusnys, P. López Gómez, J. De Luis Yanes, C. Calles Blanco, E. De Miguel Campos, A. Garcia Perez; Madrid/ES

**Purpose:** The R.E.N.A.L. nephrometry score was developed to classify renal masses based on their complexity (low, intermediate, and high) and guide decision-making in cases that could benefit from partial nephrectomy. Although initially designed for this purpose it might also be useful for predicting outcomes in percutaneous ablation of renal masses-a treatment that has emerged as an alternative to surgery and has shown promising results in small renal masses. In this study, we aim to examine the cases of renal masses treated with ablation in our institution.
**Methods or Backround:** We reviewed cross-sectional studies prior to the intervention and retrospectively applied the R.E.N.A.L. score to a total of 81 renal masses treated at our institution between January 2020 and June 2023, obtaining two groups based on the complexity of the lesions: low (N=44) and intermediate-high (N=37). Additionally, we analysed all available follow-up studies to date to identify complications and signs of tumour recurrence.The Fisher's exact test was used to test for an association of R.E.N.A.L. score with complications and recurrences.
**Results or Findings:** The immediate complications (the majority being hematomas at the site of intervention) occurred in 3 cases of low complexity (6.8%) and in 9 cases of intermediate-high complexity (24.3%). There was a significant association between the complexity group and the immediate complication rate (p<0.033). Tumoural recurrences were seen in 8 cases of low complexity (18.1%) and in 3 cases of intermediate-high complexity (8.1%). No significant association has been found (p=0.331).
**Conclusion:** The R.E.N.A.L. scoring system could be a useful tool for predicting outcomes in percutaneous ablation for renal masses.
**Limitations:** Due to a lack of cases in the high complexity group, we had to fuse it with the intermediate complexity group for statistical analysis.
**Ethics committee approval:** No ethical approval was required for this study.
**Funding for this study:** No funding was obtained for this study.
**Author Disclosures:**
Claudia Gerlotti Slusnys: Nothing to disclose
Jorge De Luis Yanes: Nothing to disclose
Carlos Calles Blanco: Nothing to disclose
María Camarena Gea: Nothing to disclose
Mihail Poida: Nothing to disclose
Miguel Paniagua González: Nothing to disclose
Angela Garcia Perez: Nothing to disclose
Paola López Gómez: Nothing to disclose
Enrique De Miguel Campos: Nothing to disclose

### RPS 407-10

**Sensing Ultrasound Localisation Microscopy for the visualisation of glomeruli in living humans**

*S. Bodard*; Paris/FR
*(sylvainbod@hotmail.com)*

**Purpose:** Estimation of glomerular function is necessary to diagnose kidney diseases. However, the study of glomeruli in the clinic is currently done indirectly through urine and blood tests. A recent imaging technique called Ultrasound Localisation Microscopy (ULM) has appeared. It is based on the ability to record continuous movements of individual microbubbles in the bloodstream. The aim of this study was to use ULM for glomeruli visualisation. Although ULM improved the resolution of vascular imaging up to tenfold, the imaging of the smallest vessels had yet to be reported.

**Methods or Backround:** We acquired ultrasound sequences from living humans and then applied filters to divide the data set into slow-moving and fast-moving microbubbles. We performed a double tracking to highlight and characterise populations of microbubbles with singular behaviours. We decided to call this technique “sensing ULM” (sULM).
**Results or Findings:** In this study, we report the observation of microbubbles flowing in the glomeruli in living humans. We present a set of analysis tools to extract quantitative information from individual microbubbles, such as remanence time or normalised distance.
**Conclusion:** As glomeruli play a key role in kidney function, it would be possible that their observation yields a deeper understanding of the kidney. It could also be a tool to diagnose kidney diseases in patients. More generally, it will bring imaging capabilities closer to the functional units of organs, which is a key to understand most diseases, such as cancer, diabetes, or kidney failures.
**Limitations:** The spatial resolution of CEUS, i.e. between 70 and 150 µm, the localisation error of 43±4 µm - that increases with depth due to divergent ultrasound beam - and the presence of efferent and afferent arterioles in the same track as glomeruli leaded us to a rough PDW estimation with sULM
**Ethics committee approval:** The Ethics Committee of the French Society of Radiology approved this study (CERF, reference number CRM-2203-240).
**Funding for this study:** This study was funded by the European Research Council under the European Union Horizon H2020 program (ERC Consolidator grant agreement No 772786-ResolveStroke).
**Author Disclosures:**
Sylvain Bodard: Nothing to disclose

### RPS 407-11
**Multiparametric MRI to evaluate tumour response of locally advanced bladder cancer after radioimmunotherapy**
*S. Reischl*, M. Makowski, A. Sauter; Munich/DE

**Purpose:** Neoadjuvanttherapy, including radioimmunotherapy (RIT), is considered for locally advanced bladder cancer (BC) patients that are not candidates for primary surgery. Evaluating tumor response to neoadjuvant therapy is crucial for determining the feasibility of bladder-sparing strategies. This study aims to evaluate the utility of multiparametric magnetic resonance imaging (mpMRI) using the Vesical Imaging-Reporting And Data System (VI-RADS) criteria in evaluation of tumour response to RIT.
**Methods or Backround:** This study was designed as secondary analysis of a tertiary-center, prospective study including patients with locally advance BC treated by neoadjuvant RIT and followed by cystectomy. MpMRI was performed pre- and post-RIT and the imaging protocol followed the VI-RADS guidelines, including T2-weighted, diffusion-weighted and contrast-enhanced sequences. Semi-quantiative scoring image analysis was performed by two radiologists. Inter- and intrareader agreement were assessed using Kendall's τ. Histopathological examination served as ground truth for the evaluation of the diagnostic accuracy of mpMRI in post-RIT mpMRI.
**Results or Findings:** Fifteen patients underwent post-RIT mpMRI and subsequent cystectomy. Intrareader agreement ($\tau$=0.74; $p<0.001$ and $\tau$=0.53; $p<0.015$) was superior to interreader agreement ($\tau$=0.38; $p<0.081$) post-RIT. Of nine patients with residual tumour only one case of carcinoma in situ (pTis) was scored false-negative, while of five cases with muscle-invasive residual tumour (≥pT2) only one patient was scored false-negative (VI-RADS≤2) for muscle invasion, in each case by one reader.
**Conclusion:** This study suggests VI-RADS criteria in mpMRI can be valuable tools for evaluating tumour response in BC patients after RIT. These findings open the door to expanding bladder-sparing strategies for a broader patient population, pending validation in larger studies.
**Limitations:** This study was restricted by its limited cohort size.
**Ethics committee approval:** This study was approved by the ethical review board of our institution.
**Funding for this study:** This study was funded by inhouse ressources the Department of Radiology of the TUM.
**Author Disclosures:**
Andreas Sauter: Nothing to disclose
Stefan Reischl: Nothing to disclose
Marcus Makowski: Nothing to disclose

### RPS 407-12
**Utilisation of lesion heterogeneity on plain CT imaging using standard deviation and mean HU values in differentiating between solid/complex cystic and benign cystic renal lesions**
*B. K. Alabri*, I. Alsalmi; Muscat/OM
*(r20131@resident.omsb.org)*

**Purpose:** The main goal of this study is to evaluate the utility of standard deviation and mean Hounsfield Unit (HU) values in plain CT scans to distinguish between solid/complex cystic lesions and benign cystic lesions in the kidneys. The hypothesis is that there is a statistically significant difference in HU standard deviation between cystic and solid renal masses.
**Methods or Backround:** A retrospective analysis of 239 cases over the past 13 years was conducted, focusing on histologically proven renal masses for patients who underwent a plain CT scan of the abdomen prior to biopsy or resection of a renal lesion, with exclusion criteria applied for cases of angiomyolipomas and lesions smaller than one cm. Attenuation values were assessed through region of interest (ROI) measurements, ensuring comprehensive coverage while avoiding areas of calcifications and necrosis. Renal MRI data from the same period were used as a control group for simple cysts.
**Results or Findings:** The study included 239 cases, dividing renal masses into simple cystic (n=115, 48.1%) and solid (n=124, 51.9%) groups based on CT scans. Average Hounsfield Units (HU) in simple cysts were much lower (mean=7.10) than in solid lesions (Mean=37.35) with a p-value <0.001. Standard deviation (SD) values followed a similar pattern, being lower in simple cysts (Mean=12.85) compared to solid lesions (Mean=16.61) with a p-value <0.001. HU was more effective than SD in distinguishing them. A scoring system combining HU and SD effectively differentiated between cystic and solid renal lesions, showing potential clinical use.
**Conclusion:** Combining Hounsfield unit and standard deviation values improves renal lesion characterisation on CT scans, offering potential for more precise clinical decisions and patient management.
**Limitations:** Single-centre retrospective nature, raising possible issues with regard to generalisation of results and selection bias and sample size limit this study.
**Ethics committee approval:** The study is retrospective and hence, no ethical approval was sought.
**Funding for this study:** No funding was provided for this study.
**Author Disclosures:**
Balqees Khamees Alabri: Investigator: Investigator and speaker / presenter
Ishaq Alsalmi: Consultant: Supervisor Consultant: Radiology consultant, Body Imaging, Royal Hospital

**13:00-14:30 Research Stage 4**

### Research Presentation Session: Abdominal Viscera & GI Tract

### RPS 401
### Diagnosis and response assessment in colorectal cancer

**Moderator**
S. Gourtsoyianni; Athens/GR
*(sgty76@gmail.com)*

### RPS 401-2
**Value of Iodine uptake derived from DECT in differentiating malignant from benign colorectal wall thickening**
*L. Widmer*[1], F. Cherbanyk[1], H. Thoeny[1], S. Malekzadehlashkariani[2]; [1]Fribourg/CH, [2]Sion/CH

**Purpose:** The aim of this study was to assess the performance of iodine uptake derived from Dual-energy computed tomography (DECT) in differentiating malignant from benign colorectal wall thickening.
**Methods or Backround:** DECT is widely used to characterise and differentiate tumours. However, data regarding its diagnostic performance for the characterisation of colorectal tumours are limited. In this single-centre IRB-approved study, 50 consecutive patients with both histopathology-proven colorectal adenocarcinoma and benign colon wall pseudo-thickening were prospectively enrolled. Two radiologists quantitatively analysed the iodine uptake of the tumors and iodine ratios as well as density measurements in Hounsfield units (HU) from portal venous phase DECT. Paired t-test and predictive power score were used to compare variables of malignant and benign wall thickening. A priori statistical power analysis using G*Power estimated that 45 patients were necessary to reach a 95% statistical power for a one-sided paired t-test with a medium effect size and an alpha of 0.05.
**Results or Findings:** Significantly greater iodine uptakes were found in tumours than in benign wall pseudo-thickenings (medium to strong predictive power, delta = 0.13, $t(49) = 7.3$, $p < 0.001$, pps = 0.64). There were no equivalence between the tumours and the benign wall pseudo-thickenings (delta = 0.17, $t(49) = 6.4$, $p = 1$), but significantly greater HU values in tumours compared to benign wall pseudo-thickenings (strong predictive power, $t(49) = 15.8$, $p < 0.001$, pps = 0.79).
**Conclusion:** DECT demonstrated a significant difference in HU and iodine uptake in malignant colorectal tumours in comparison to benign colorectal wall pseudo-thickening. However, this study surprisingly shows that HU is a variable of better value than iodine uptake in discriminating a tumour from a non-tumour pseudo-thickening.

**Limitations:** Single-centre study and small sample size; adenocarcinoma as only histopathological subtype; manual delineation of ROIs limit the scope of this study.
**Ethics committee approval:** CER-VD, swissethics approved this study.
**Funding for this study:** No funding was obtained for this study.
**Author Disclosures:**
Sonaz Malekzadehlashkariani: Nothing to disclose
Harriet Thoeny: Nothing to disclose
Floryn Cherbanyk: Nothing to disclose
Lucien Widmer: Nothing to disclose

### RPS 401-3

**Performance of CT in the locoregional staging of non-rectal colon cancer: detailed radiology-pathology correlation with a special emphasis on tumour deposits and extramural venous invasion**

*D. Karahacıoğlu*, O. C. Taskin, A. Armutlu, B. Saka, R. Esmer, D. Bugra, E. Balik, V. Adsay, B. Gurses; Istanbul/TR
*(duygu.keles@gmail.com)*

**Purpose:** The purpose of this study was to investigate the performance of CT in detecting the diagnostic and prognostic characteristics (established in rectal adenocarcinomas) in non-rectal colon malignancies via close radiologic and pathologic correlation in order to improve patient care.
**Methods or Backround:** CT and pathology data from 137 resected colonic adenocarcinoma cases were reviewed for prognostic parameters by dedicated radiologists and pathologists whose primary practice is GI cancers. Discrepant cases were re-evaluated together with correlation of radiologic-gross photographs and microscopic findings.
**Results or Findings:** For T-stage, overall CT tended to understage (in 12.4% of cases), performed better in left versus right colon, with the lowest performance observed in transverse colon, and the highest rate of misstaging at sigmoid. The sensitivity, specificity, and accuracy of CT in the detection of EMVI were 58,5 %, 82,1 %, 73 % (which was slightly better for high-load EMVI compared to low-load). These figures for TDs were 57,9 %, 92,4 %, 87,6 and for LNs, 44.7%, 72.7%, and 63.5%. In 13 patients with discrepancies, on-site correlation resulted in modification of the initial radiology or pathology findings.
**Conclusion:** The criteria increasingly employed for rectal cancers and were elucidated by careful pathologic radiologic correlation in our studies demonstrate that CT can be more effectively used in prognostication of colon cancers. CT appears to be more applicable in left colon, although mis-stagings are most common in the sigmoid, for which more non-committal reporting may be necessary in uncertain cases. CT is highly effective in detection of TDs and EMVI (especially high-load), but less so for LNs. TD and EMVI should be considered for incorporation into routine reporting CT proformas for colon cancer.
**Limitations:** The major limitation is the absence of specimen radiology, which prevents the correlation of LN/TD on a case-by-case basis.
**Ethics committee approval:** This study was approved by the IRB number: 2022.092.IRB1.038
**Funding for this study:** No funding was recieved for this study.
**Author Disclosures:**
Duygu Karahacıoğlu: Nothing to disclose
Burcu Saka: Nothing to disclose
Ayse Armutlu: Nothing to disclose
Bengi Gurses: Nothing to disclose
Volkan Adsay: Nothing to disclose
Dursun Bugra: Nothing to disclose
Orhun Cıg Taskin: Nothing to disclose
Emre Balik: Nothing to disclose
Rohat Esmer: Nothing to disclose

### RPS 401-4

**Preoperative evaluating peritoneal cancer index in peritoneal metastatic colon cancer patients using CT-based deep-learning model**

*S. Zhang*[1], F. Shen[2], C. Shao[2]; [1]Jinan/CN, [2]Shanghai/CN

**Purpose:** This study aimed to build a convolution neural network to automatically segment the colon cancer lesion and evaluate peritoneal cancer index (PCI) in peritoneal metastasis (PM) cases based on preoperative CT scans.
**Methods or Backround:** A total of 177 consecutive colon cancer patients with preoperative abdominopelvic CT scans and PM administered exploratory laparotomy were retrospectively analysed. Surgical PCIs (sPCIs) were confirmed by the surgery team, and CT-PCI scores were assessed by radiologists. Totally 90 cases were assigned to training set. Then, 87 cases were enrolled as test set. Initially, we utilised nn-UNet to build an automatic segmentation model based on CT scans at portal venous phase. Secondly, we constructed deep learning model (DL-Model) to assess sPCIs. The performances in distinguishing between low-sPCI (≤ 20) and high-sPCI (> 20) cases were also assessed by receiver operating characteristic (ROC) curve analysis and decision curve analysis (DCA).
**Results or Findings:** The automatic segmentation model showed excellent performance. The DL-Model had higher classification performance than the subjective evaluation in all datasets (AUCs were 0.867 and 0.830 in training and test sets, vs 0.767 and 0.694, respectively). The usefulness of the proposed DL-Model was confirmed by DCA.
**Conclusion:** This study showed that the DL-Model based on abdominopelvic CT scans were useful for preoperative detecting PCI in colon cancer patients with PM, especially showed increased diagnostic performance than subjective CT-PCI.
**Limitations:** It is unclear whether our findings would apply to the lesion with neoadjuvant treatment, which needs further research.
**Ethics committee approval:** Committee on Medical Research Ethics of Changhai Hospital approved this study.
**Funding for this study:** No funding was received for this study.
**Author Disclosures:**
Fu Shen: Nothing to disclose
Chengwei Shao: Nothing to disclose
Shaoting Zhang: Nothing to disclose

### RPS 401-5

**Early regression index (ERI) on MR images predicts complete responders after neo-adjuvant chemo-radiotherapy**

C. Fiorino, D. Palumbo, *M. Mori*, G. Palazzo, A. Pellegrini, A. Damascelli, S. Steidler, A. Del Vecchio, F. De Cobelli; Milan/IT
*(mori.martina@hsr.it)*

**Purpose:** The early regression index (ERI) resulted to predict treatment response in rectal cancer patients. Aim of the current study was to prospectively assess tumour response to neo-adjuvant chemo-radiotherapy (nCRT) of locally advanced oesophageal cancer using ERI, based on MRI.
**Methods or Backround:** From January 2020 to May 2023, 30 patients with oesophageal cancer were enrolled in a single arm prospective study (ESCAPE). Patients were scanned with PET-MRI at three time points: (i) before nCRT (tpre); (ii) mid-radiotherapy, tmid; (iii) after nCRT, 8-12 weeks before surgery (tpost); nCRT delivered 41.4Gy/23fr with concurrent carboplatin and paclitaxel. For patients that skipped surgery, complete clinical response (cCR) was assessed if patients showed no local relapse after one year; patients with pathological complete response (pCR) or with cCR were considered as complete responders (pCR+cCR). Segmentations of GTV volumes were performed by two observers (Vpre, Vmid, Vpost) on T2w MRI: ERI and other volume regression parameters at tmid and tpost were tested as predictors of pCR+cCR.
**Results or Findings:** At the time of the analysis complete data of 25 patients were available: 3/25 with complete response at imaging refused surgery and 2/3 were cCR; in total, 10/25 patients showed pCR+cCR (pCR=8/22). Both ERImid and ERIpost identified pCR+cCR patients, with ERImid showing better performance (AUC:0.78, p=0.014). A logistic model combining ERImid and Vpre improved performances (AUC:0.93, p<0.0001). Inter-observer variability in contouring GTV did not affect the results.
**Conclusion:** Despite the limited numbers, interim analysis of ESCAPE study suggests ERI as a robust predictor of complete response after nCRT for esophageal cancer. Further validation on larger populations is warranted.
**Limitations:** Interruptions due to Covid-19 pandemia caused the downsizing of the sample size.
**Ethics committee approval:** The study received institutional review board approval and written informed consent was obtained from all participants.
**Funding for this study:** The study was supported by AIRC (Italian Association for Cancer Research) under Investigator Grant – IG 2019 – ID 23015 project.
**Author Disclosures:**
Claudio Fiorino: Nothing to disclose
Diego Palumbo: Nothing to disclose
Antonella Del Vecchio: Nothing to disclose
Alessandro Pellegrini: Nothing to disclose
Stephanie Steidler: Nothing to disclose
Francesco De Cobelli: Nothing to disclose
Martina Mori: Nothing to disclose
Gabriele Palazzo: Nothing to disclose
Anna Damascelli: Nothing to disclose

### RPS 401-6

**Role of IVIM MRI in response assessment in colorectal carcinoma**

C. J. Das, *S. Chandola*, A. Soni; New Delhi/IN

**Purpose:** The aim of this study was to. assess the usefulness and performance of Diffusion-weighted imaging (DWI) with IVIM- DKI for assessing post chemotherapy (CT)/Chemo and Radiotherapy (CTRT) response in colorectal carcinoma and compare with PET/CT parameters
**Methods or Backround:** Forty patients with rectosigmoid cancer underwent baseline staging multiparametric MRI and 18-FDG PET/CT and follow-up with both scans post chemo radiotherapy. Quantitative diffusion, IVIM and DKI parameters, viz. apparent (ADC) and molecular (D) diffusion coefficient, perfusion coefficient (f), and kurtosis (K) were measured from non-necrotic areas and semi-quantitative PET parameters including SUV max, SUV ratio, Metabolic tumour volume (MTV), and total lesion glycolysis (TLG) were derived from the PET/CT images; and correlated with the patient's response keeping RECIST 1.1 criteria as the gold standard.
**Results or Findings:** A statistically significant increase in D and ADC with a decline in K was noted after therapy in all patients. No significant difference was seen among the percentage change in the parameters observed post-therapy among the responders and non-responders. Both the responders as well as non-responders depicted a statistically significant increase in D and ADC, and a significant decline in K values post-therapy. Among 17 patients with follow-up PET/CT imaging, a significant decline in all parameters of the primary lesion was seen post-therapy. Responders (n=12) showed a significant decline in all parameters from baseline after therapy, whereas non-responders did not show any such decline. Post-therapy MTV, followed by TLG were found to have strongest correlation with a positive response, with AUCs of 0.933 and 0.900 on receiver operator curves.
**Conclusion:** 18-FDG PET/CT is the more accurate single modality for assessing both response as well as tumour burden post therapy, while ADC and D derived from DWI and IVIM respectively are useful adjuncts for assessment of response.
**Limitations:** No information provided by the submitted.
**Ethics committee approval:** The ethics committee notification can be found under the number ID - IEC PG-621/25.11.20, RT-06/23.12.2020.
**Funding for this study:** No funding was provided for this study.
**Author Disclosures:**
Abhishek Soni: Nothing to disclose
Chandan Jyoti Das: Nothing to disclose
Stuti Chandola: Nothing to disclose

### RPS 401-7

**Can MRI accurately assess the TN staging of rectal cancer patients with local regrowth during watchful waiting?**

*X. Ou*, B. M. Geubels, D. M. J. Lambregts, B. Grotenhuis, G. L. Beets, R. G. H. Beets-Tan, M. Maas; Amsterdam/NL
*(x.ou@nki.nl)*

**Purpose:** Local regrowth during watchful waiting occurs in +/-25% of patients. Salvage surgery is possible in the vast majority. MRI is used for regrowth staging, but its accuracy is unknown. The aim is to evaluate the accuracy of MRI for preoperative TN-staging of rectal cancer patients with local regrowth during organ preservation.
**Methods or Backround:** Rectal cancer patients identified with local regrowth during watchful waiting all underwent salvage surgery (TME or local excision). Patients who underwent local excision as salvage surgery were excluded from N-status analyses if their follow-up after local excision was < 24 months. An expert radiologist scored the ymrTN-stage on pre-surgery MRIs. The ymrT and ymrN-stages were compared with final ypT and ypN-stages at histopathology. For analysis, T0, T1 and T2 were combined as T0-2 and T3 and T4 combined as T3-4; N1 and N2 were combined as N+. Diagnostic performance (accuracy, sensitivity, specificity) were calculated.
**Results or Findings:** Among these patients, 26 had ypT-stage available (17 ypT0-2, 9 ypT3-4) and 22 had ypN-stage available (15 ypN0, 7ypN+). The MRI-to-surgery interval ranged from 2 to 19 weeks (median: 7 weeks). 20 had luminal regrowth, one had nodal regrowth, and six had both. Accuracy, sensitivity and specificity for ypT-staging were 81%, 76% and 89%. Overstaging occurred in 15%. Accuracy, sensitivity and specificity for ypN-staging were 82%, 80% and 82%. Understaging occurred in 14%.
**Conclusion:** MRI can accurately stage local regrowth during watchful waiting before surgery. In ypT-staging, overstaging is common, while ypN-staging is more prone to understaging.
**Limitations:** Multiple readers need to be included to assess the interobserver agreement and evaluate the impact of radiologists' experience on ymrTN-staging accuracy. Sample size is rather small. Selection bias needs to be considered for the highly selected group.
**Ethics committee approval:** Not applicable for this study.
**Funding for this study:** Not applicable for this study.
**Author Disclosures:**
Monique Maas: Nothing to disclose
Xinde Ou: Nothing to disclose
Doenja Marina Johanna Lambregts: Nothing to disclose
Brechtje Grotenhuis: Nothing to disclose
Regina G. H. Beets-Tan: Nothing to disclose
Geerard L. Beets: Nothing to disclose
Barbara Melissa Geubels: Nothing to disclose

### RPS 401-8

**The involvement of mesorectal fascia by tumour deposits and extramural venous invasion predicts poor overall survival in locally advanced rectal cancer**

*Y. Feng*, T. Tong; Shanghai/CN
*(fengyaru915@163.com)*

**Purpose:** Mesorectal fascia (MRF) involvement serves as an adverse prognostic indicator for locally advanced rectal cancer (LARC). However, prognoses among MRF+ patients vary. This study aims to further classify MRF involvement in MRF+ cases and investigate its prognostic implications.
**Methods or Backround:** We conducted a retrospective analysis of data from LARC patients who were evaluated MRF+ and underwent surgery after neoadjuvant chemoradiotherapy. Patients were categorised into four groups based on MRF involvement: MRF+ (Tumour) vs MRF+ (Non-tumour), MRF+ (Extramural venous invasion; EMVI) vs MRF+ (Non-EMVI), MRF+ (lymph node; LN) vs MRF+ (Non-LN), and MRF+ (Tumour deposits; TDs) vs MRF+ (Non-TDs). Survival curves were generated using the Kaplan-Meier (K-M) method, and differences in overall survival (OS) were assessed using the log-rank test. Prognostic factors were evaluated through multivariate Cox proportional hazards models.
**Results or Findings:** A total of 400 MRF+ LARC patients were included in the study. The occurrence of MRF+ (Tumour), MRF+ (TDs), and MRF+ (EMVI) was higher in patients aged ≥60 years. In K-M survival analysis, both MRF+ (TDs) and MRF+ (EMVI) cases were associated with significantly worse OS compared to MRF+(non-TDs) and MRF+ (non-EMVI). Conversely, there were no significant differences in OS between MRF+ (Tumour) and MRF+ (LN) cases and MRF+ (Non-tumour) and MRF+ (non-LN). In the multivariate COX regression analysis, MRF+ (TDs) demonstrated a significant association with OS (HR 1.744; 95% CI, 1.064-2.860). Similarly, MRF+ (EMVI) displayed a significant correlation OS (HR 1.886; 95% CI, 1.076-3.306). Conversely, there were no significant associations observed between MRF+ (Tumour) or MRF+ (LN) and OS.
**Conclusion:** MRF+ (TDs) and MRF+ (EMVI) independently indicated higher risks for adverse prognosis in MRF+ LARC patients, providing valuable insights for treatment and follow-up strategies.
**Limitations:** This was a retrospective and single centre study.
**Ethics committee approval:** This research was approved ethically.
**Funding for this study:** No funding was obtained for this study.
**Author Disclosures:**
Yaru Feng: Nothing to disclose
Tong Tong: Nothing to disclose

### RPS 401-9

**Performance of dual-layer spectrum CT virtual monoenergetic images to assess early rectal adenocarcinoma T-stage: comparison with MR**

*Z. Jia*, W. Yuan, X. M. Liu, H. Zhang, J. Dai, L. Guo, W. C. Chen, X. Liu; Guang Zhou/CN

**Purpose:** Dual-layer spectrum CT (DLSCT) can provide information about material decomposition and improve lesion visualization that may be useful to assess preoperative T-stage in early rectal carcinoma (ERC). This study aimed to investigate the image quality of virtual monoenergetic images (VMI) and conventional polyenergetic image (PEI) from DLSCT, and compare the performance with MR in assessing preoperative T-stage for early rectal adenocarcinoma (ERA).
**Methods or Backround:** This retrospective study included 67 ERA patients (mean age 62±11.1 years) who underwent DLSCT and MR examination. VMI 40-200 keV and poly energetic image (PEI) were reconstructed. The image noise, signal-to-noise ratio (SNR), contrast-to-noise ratio (CNR), and tumour contrast of different energy levels were calculated and compared, respectively. Two radiologists independently assess the image quality of the VMIs and PEI using five-point scales. The diagnostic accuracies of DLSCT and HR-MRI for ERA T-staging were evaluated and compared.
**Results or Findings:** The maximum noise was observed at VMI 40 keV, and noise at VMI 40-200keV in the arterial and venous phases showed no statistically significant difference (all p>0.05). The highest SNR and CNR were obtained at VMI 40 keV, significantly greater than other energy levels and PEI (all p<0.05). Tumour contrast was more evident than PEI at 40-100keV in the arterial phase and at 40keV in the venous phase (all p <0.05). When compared with PEI, VMI 40keV yielded the highest scores for overall image quality, tumour visibility, and tumor margin delineation, especially in the venous phase (p<0.05). The overall diagnostic accuracy of DLSCT and HR-MRI for T-stage was 65.67% and 71.74% and showed no statistically significant difference (p>0.05).
**Conclusion:** VMI 40keV provides the best image quality and improves the diagnostic confidence for ERA staging.

**Limitations:** Not applicable for this study.
**Ethics committee approval:** The study received institutional review board approval and written informed consent was obtained from all participants
**Funding for this study:** This work was supported by Guangzhou Basic and Applied Basic Research Foundation 2023A03J024, National Nature Science of Foundation of China [Grant No.82202259] and the Youth Talent Project of The Second Affiliated Hospital of Guangzhou University of Chinese Medicine(ZY2022YL05)
**Author Disclosures:**
JianHao Dai: Nothing to disclose
Xiao Min Liu: Nothing to disclose
Hanliang Zhang: Nothing to disclose
Ziqi Jia: Nothing to disclose
Wenjing Yuan: Nothing to disclose
Wei Cui Chen: Nothing to disclose
Xian Liu: Nothing to disclose
Lei Guo: Nothing to disclose

**15:00-16:00 Research Stage 1**

### Research Presentation Session: Cardiac

### RPS 503

### Trials and meta-analyses: cardiac CT and MRI

**Moderator**
F. Pontana; Lille/FR
*(francois.pontana@chru-lille.fr)*

### RPS 503-2

**Cardiovascular CT and MR imaging in Europe: insights from the ESCR registry**

*F. Catapano*[1], L. J. Moser[2], M. Francone[1], R. Vliegenthart[3], C. Catalano[4], M. Gutberlet[5], H. Alkadhi[2]; [1]Milan/IT, [2]Zürich/CH, [3]Groningen/NL, [4]Rome/IT, [5]Leipzig/DE
*(federica.catapano@humanitas.it)*

**Purpose:** The aim of this study was to provide an overview of advanced cardiovascular imaging practices in Europe using structured data from the European society of cardiovascular radiology (ESCR) registry.
**Methods or Backround:** Numbers on cardiovascular CT and MRI examinations were extracted from the ESCR-registry between 2009 and October 2023. Data collection included the total/annual numbers of examinations, indications, complications, and reporting habits.
**Results or Findings:** The ESCR registry demonstrates a 6.8-fold increase of annually submitted CT examinations from 2,244 to 15,267, and a 4.7-fold increase of MRI examinations from 2,803 to 13,183 between 2010 and 2022. Reporting of CT (76%) and MRI (71%) was mostly performed by radiologists, and, to a lesser degree, in consensus with non-radiologists (19% and 27%, respectively). Main indications for cardiac CT were suspected coronary artery disease (CAD) (59%), TAVI-planning (21%), valve disease (7%) and preablation (6%). Main MRI indications were myocarditis (26%), suspected CAD (including stress-imaging) (21%), and cardiomyopathy (19%). Adverse event rates were very low for CT (0.3%) and MRI (0.7%).
**Conclusion:** The largest available registry on Cardiovascular CT and MRI in Europe demonstrates a considerable increase in exam numbers, in particular for CAD and CT. These findings collectively contribute to our understanding of the current state of cardiovascular imaging in Europe.
**Limitations:** Our data, extracted from an ESCR registry, may inherently favour a radiological perspective in the representation of clinical practice in cardiac imaging. Nevertheless, they do represent the largest cases archive in Europe.
**Ethics committee approval:** The study is retrospective or educational.
**Funding for this study:** No funding was received for this study.
**Author Disclosures:**
Matthias Gutberlet: Nothing to disclose
Lukas Jakob Moser: Nothing to disclose
Rozemarijn Vliegenthart: Nothing to disclose
Federica Catapano: Nothing to disclose
Marco Francone: Nothing to disclose
Carlo Catalano: Nothing to disclose
Hatem Alkadhi: Nothing to disclose

### RPS 503-3

**Cost-effectiveness of computed tomography in patients with atypical chest pain clinically referred for invasive coronary angiography: randomised controlled trial**

M. Bosserdt[1], *M. Mohamed*[1], K. Neumann[1], N. Rieckmann[1], H. Dreger[1], V. Brodszky[2], T. Reinhold[1], A-M. Mielke[1], M. Dewey[1]; [1]Berlin/DE, [2]Budapest/HU
*(mahmoud.mohamed@charite.de)*

**Purpose:** Is coronary computed tomography (CT) cost-effective compared with invasive coronary angiography (ICA) in patients with atypical chest pain who are clinically referred for ICA?
**Methods or Backround:** A prespecified cost-effectiveness analysis of 329 patients with atypical angina or chest pain from a randomised pragmatic trial comparing CT and ICA conducted at a university hospital in Germany was performed. Cost-effectiveness was analysed for up to 3 years of follow-up from the health sector perspective using quality-adjusted life years (QALYs) derived from the EQ-5D-3L questionnaire. Costs were obtained from each individual's outpatient and inpatient billing data and included cardiovascular medications, hospitalisations, emergency department visits, cardiologist visits, and cardiac examinations. Data analysis included 500 multiple imputations followed by 1,000 bootstrapping iterations for each imputed data set, and the net monetary benefit was calculated.
**Results or Findings:** There was no statistically significant difference in mean QALYs at either one-year or three-year follow-up, while the mean cost per patient was significantly lower in the CT group compared with the ICA group, both at one year (difference in €: -1,647.8, -2,198.3 to -1,937.0) and at three years (difference in €: -1,543.3, -2,228.0 to -830.0). At a willingness-to-pay threshold of €20,000/QALY, the average incremental net monetary benefit of CT over ICA was €1,256.5 (164.8 to 2331.8) at one year and €1202.0 (-1,378.7 to 3,961) at three years. The incremental net monetary benefit of CT over ICA at three years was the highest in patients with a pretest probability of CAD above 30% (€ 1445.6, -1803.1 to 4637.0).
**Conclusion:** A CT-first strategy for the management of patients with atypical angina or chest pain was more cost-effective than a direct-to-ICA strategy.
**Limitations:** This analysis considered only cost from the health sector perspective.
**Ethics committee approval:** This trial was approved by the ethics committee of the Charité –Universitätsmedizin Berlin (EA1-1-080-08) and by the German Federal Office for Radiation Protection (Z5–22462/2–2008-048). Before the randomisation all participants gave written informed consent.
**Funding for this study:** This study was funded by a grant of the Heisenberg programme of the German Research Foundation to Marc Dewey.
**Author Disclosures:**
Henryk Dreger: Nothing to disclose
Mahmoud Mohamed: Nothing to disclose
Konrad Neumann: Nothing to disclose
Nina Rieckmann: Nothing to disclose
Thomas Reinhold: Nothing to disclose
Marc Dewey: Research/Grant Support: MD reported grants from the Heisenberg Programme of the German Research Foundation, grants for fractal analysis of myocardial perfusion from the German Research Foundation, and a grant from the Berlin University Alliance (GC_SC_PC 27) outside the submitted work; Research/Grant Support: In addition, MD holds a joint patent with Florian Michallek on dynamic perfusion analysis using fractal analysis (PCT/EP2016/071551 and USPTO 2021 10,991,109 approved); Other: MD is European Society of Radiology (ESR) Research Chair (2019–2022) and ESR Publications Chair (2022-2025); and the opinions expressed in this article are the author’s own and do not represent the view of ESR.
Valentin Brodszky: Nothing to disclose
Maria Bosserdt: Nothing to disclose
Anna-Maria Mielke: Nothing to disclose

### RPS 503-4

**Detection of calcified plaques on coronary CT angiography compared to thin-slice non-contrast CT; multicentre trial evaluation**

*K. Schulze*[1], B. Föllmer[1], F. Biavati[1], R. Bockelmann[1], M. Bosserdt[1], F. Michallek[1], J. D. Dodd[2], K. F. Kofoed[3], M. Dewey[1]; [1]Berlin/DE, [2]Dublin/IE, [3]Copenhagen/DK

**Purpose:** The aim of this study was to assess the feasibility of assessing calcified coronary artery plaques on CT angiography (CTA) compared to non-contrast CT (NCCT) in a multicentre study.
**Methods or Backround:** This study included 47 patients from the DISCHARGE trial subgroup (mean age 62.0 ± 11.0 years, 57.4% male) with available thin-slice (< 0.7 mm) NCCT and CTA. The diagnostic accuracy and detection of manually segmented coronary calcified plaques was automatically assessed for CTA and NCCT using a definition of a volume of at least 1 mm³. Plaques on CTA were defined as missed if there was no spatial overlap with a calcified plaque on NCCT after registration. Sensitivity and specificity were calculated using NCCT as reference standard. Lesion level statistics were analysed for plaque density and volume parameters, with plaques categorised into the groups 'all' and 'missed'.
**Results or Findings:** NCCT identified 314 coronary calcified plaques of which 213 (32% sensitivity) were missed in CTA alone. CTA was not associated with false positive calcified plaques. Missed coronary calcified plaques on CTA had higher density (537.3 HU ± 224.9 HU) compared to NCCT (467.9 HU ± 215.3 HU). These plaques were detectable on NCCT based on density, while CTA density was closer to aortic density (393.4 HU ± 101.0 HU). Additionally, missed plaques were smaller in volume (7.15 $mm^3$ ± 9.0 $mm^3$) compared to all plaques (21.0 $mm^3$ ± 57.3 $mm^3$). Overlooking calcified coronary plaques would have led to the omission of 11 out of 47 (23%) patients with CAD-RADS 1, incorrectly categorising them as CAD-RADS 0.
**Conclusion:** CTA can miss up to two thirds of coronary calcified plaques visible on NCCT, highlighting the important role of NCCT.
**Limitations:** This study involved a small subset of 47 patients from three of the 26 DISCHARGE trial centres.
**Ethics committee approval:** The DISCHARGE trial was approved by the ethics committee at Charité–Universitätsmedizin Berlin as the coordinating centre, by the German Federal Office for Radiation Protection, and by local or national ethics committees.
**Funding for this study:** This study was funded by grants from the EU-FP7 Framework Program (FP 2007-2013, EC-GA 603266) and funded by the Deutsche Forschungsgemeinschaft (DFG, German Research Foundation) in the graduate program on quantitative biomedical imaging (BIOQIC, DFG project number: 289347353, GRK 2260/1).
**Author Disclosures:**
Kenrick Schulze: Nothing to disclose
Klaus F. Kofoed: Grant Recipient: Received financial support from Rigshospitalet, University of Copenhagen, during the conduct of the study. Received grants from AP Møller og hustru Chastine McKinney Møllers Fond, Danish Heart Foundation, The Danish Agency for Science, Technology and Innovation by The Danish Council for Strategic Research, Health Insurance Company Denmark. Grant Recipient: Received funding from EU-FP7 Framework Program (DISCHARGE EU FP EC-GA 603266); Grant Recipient: He additionally received unrestricted research grant from Canon Medical Corporation and GE Healthcare.
Robin Bockelmann: Nothing to disclose
Marc Dewey: Grant Recipient: EU (EC-GA 603266 in HEALTH.2013.2.4.2-2) DFG (DE 1361/14-1, DE 1361/18-1, BIOQIC GRK 2260/1, Radiomics DE 1361/19-1 [428222922] and 20-1 [428223139] in SPP 2177/1), GUIDE-IT (DE 1361/24-1), Berlin University Alliance (GC_SC_PC 27), Berlin Institute of Health (Digital Health Accelerator); Author: Cardiac CT (Springer Nature); Other: Hands-on cardiac CT courses (www.ct-kurs.de) Institutional research agreements: Siemens, General Electric, Philips, Canon. Patent on fractal analysis of perfusion imaging (jointly with Florian Michallek, EPO 2022 EP3350773A1, and USPTO 2021 10,991,109, approved) M.D. is European Society of Radiology (ESR) Publications Chair (2022-2025); the opinions expressed in this presentation are the author's own and do not represent the view of ESR.
Florian Michallek: Nothing to disclose
Federico Biavati: Nothing to disclose
Maria Bosserdt: Grant Recipient: Received funding from EU-FP7 Framework Program (DISCHARGE EU FP EC-GA 603266). Department of Radiology, Charité – Universitätsmedizin Berlin, Freie Universität Berlin and Humboldt-Universität zu Berlin, Germany
Jonathan Dermot Dodd: Grant Recipient: Received funding from EU-FP7 Framework Program (DISCHARGE EU FP EC-GA 603266). Is an Associate Editor for Radiology, a member of the Editorial Board for Radiology Cardiothoracic Imaging, and an Associate Editor for the Quarterly Journal of Medicine. All non-paid. Author: Receives royalties as a co-author of book chapters in the Stat-Dx book Series Diagnostic Imaging – Cardiovascular and the textbook CT and MRI in Cardiology, Elsevier.
Bernhard Föllmer: Nothing to disclose

### RPS 503-5

**Dedicated cardiovascular screening in lung cancer screening: preliminary results from the European 4-IN-THE-LUNG-RUN trial**
*D. Han*[1], M. Vonder[1], C. Van Der Aalst[2], A. Schmitz[3], J. W. C. Gratama[4], M. Silva[5], H. J. De Koning[2], M. Oudkerk[1]; [1]Groningen/NL, [2]Rotterdam/NL, [3]Amsterdam/NL, [4]Apeldoorn/NL, [5]Parma/IT
*(han@i-dna.org)*

**Purpose:** The 4-IN-THE-LUNG-RUN (4ITLR) trial, which was recently started and aims to enroll 26,000 participants, offers the opportunity for prospective cardiovascular screening within a lung cancer screening program. Although current guidelines advise the assessment of coronary calcifications on chest CT scans of any kind, this requires specific imaging acquisition and reconstruction for accurate Agatston score evaluation. This study explores the potential benefits of cardiovascular screening within the initial participants of 4ITLR trial.
**Methods or Backround:** The inclusion criteria were: age 60-79 years, smoking history of ≥35 pack-years, and current smoking or quitting within the last 10 years. High-temporal-resolution low-dose chest CT scans using a third-generation dual-source CT scanner were performed on 443 participants between January 15th and March 29th, 2023, at a single centre. An automatic assessment of the Agatston score was conducted on dedicated cardiac reconstructions that utilised a slice thickness/increment of 3.0/1.5 mm, a medium-sharp kernel, and high-pitch acquisition, with an FBP algorithm and 120 kVp. This allowed for the reliable categorisation of the participants' risk based on their Agatston score, with categories being low risk (0 score), moderate risk (1-99), high risk (100-399), and very high risk (≥400).
**Results or Findings:** The mean age was 68.6 years (SD 4.9), with 56.9% male. Median Agatston scores were 242.1 (IQR 34.8-939.9) for men and 56.3 (IQR 2.5-365.0) for women. 16.0%, 30.7%, 19.6%, and 33.6% of participants were at low, moderate, high, and very high CHD risk, respectively. About 47% fell into low/moderate CHD risk categories.
**Conclusion:** The initial lung cancer screening identified one third of participants at significantly high CHD risk, while half were suitable for preventive CHD treatment. Notably, 16% had a low CHD risk, exempting them from CHD preventive medication as per existing guidelines.
**Limitations:** No limitations were identified.
**Ethics committee approval:** The minister of public health, well-being and sport has approved the execution of the 4-IN-THE-LUNG-RUN study.
**Funding for this study:** This project has received funding from the European Union's Horizon 2020 programme under grant agreement no 848294.
**Author Disclosures:**
Jan Willem C. Gratama: Nothing to disclose
Marleen Vonder: Nothing to disclose
Matthijs Oudkerk: Nothing to disclose
Alexander Schmitz: Nothing to disclose
Harry J. De Koning: Nothing to disclose
Daiwei Han: Nothing to disclose
Carlijn Van Der Aalst: Nothing to disclose
Mario Silva: Nothing to disclose

### RPS 503-6

**Prognostic value of CT-derived myocardium-related parameters in patients with aortic stenosis: a systematic review and meta-analysis**
X. He[1], Y. Li[1], Y. Wang[1], X. Lu[1], J. Nan[1], L. Cao[1], Y. Wang[2], G. Wang[1], *Z. Chen*[1]; [1]Lan Zhou city/CN, [2]Shanghai/CN

**Purpose:** The aim of this study was to investigate the prognostic value of CT-derived myocardium-related parameters in patients with severe aortic stenosis (AS) who have undergone aortic valve replacement (AVR).
**Methods or Backround:** Four databases (PubMed, Web of Science, Embase, and Cochrane) were searched to identify studies investigating the prognostic performance of CT-derived myocardium-related parameters in patients with AS. A random effects model for meta-analysis was conducted to calculate pooled hazard ratios (HR) and 95% confidence intervals (CI) in order to assess the prognostic value. The I2 statistic was used to assess heterogeneity.
**Results or Findings:** In this analysis, ten studies were identified, six of which involved 662 patients reporting CT-derived extracellular volume fraction (ECV), and four studies, including 1244 patients reporting CT-derived left ventricular global longitudinal strain (LVGLS). The meta-analysis revealed that ECV, whether considered as a dichotomous variable (pooled HR: 4.12, 95% CI: 2.76-6.15, I2 =0%, P< 0.001), or as a continuous variable (pooled HR: 1.15, 95% CI: 1.05-1.25, I2 =74%, P=0.002), and LVGLS, whether considered as a dichotomous variable (pooled HR: 1.70, 95% CI: 1.31-2.19, I2 =0%, P< 0.001) or a continuous variable (pooled HR: 1.07, 95% CI: 1.05-1.10, I2=0%, P< 0.001) were all significant predictors for all-cause mortality in patients with AS after AVR.
**Conclusion:** This study has demonstrated the significant prognostic value of pre-AVR CT-derived ECV and LVGLS, both as dichotomous and continuous variables, in predicting all-cause mortality in patients with AS. These findings enhance our understanding of the pathophysiology of AS and assist in optimizing the timing of AVR.
**Limitations:** Limited number of studies in meta-analysis.
**Ethics committee approval:** No information provided by the submitter.
**Funding for this study:** This study is supported by the Lanzhou Science and technology project Foundation (2020-2D-80) and First Hospital of Lanzhou University Hospital Foundation (ldyyyn2019-78).
**Author Disclosures:**
Xiande Lu: Nothing to disclose
Gang Wang: Nothing to disclose
Yu Wang: Nothing to disclose
ZiXian Chen: Nothing to disclose
Jiang Nan: Nothing to disclose
Yuxi Li: Nothing to disclose
Xiangui He: Nothing to disclose
Liang Cao: Nothing to disclose
Yong Wang: Nothing to disclose

### RPS 503-7

**Mitral annular disjunction: the boundary between normal and pathological. Results from a large multicentre National Register of the Section of Cardioradiology of the SIRM**

*E. Bruno*[1], A. Palmisano[1], S. Dell'Aversana[2], R. Faletti[3], N. Galea[4], M. Gatti[3], C. Liguori[2], S. Pradella[5], A. Esposito[1]; [1]Milan/IT, [2]Naples/IT, [3]Turin/IT, [4]Rome/IT, [5]Florence/IT
*(bruno.elisa@hsr.it)*

**Purpose:** Mitral annular disjunction (MAD) is an anatomic variant characterised by the atrialisation of the mitral valve junction; its prevalence and pathological role are still debated. The aim of the study is to evaluate the incidence of MAD in a vast cohort of patients who underwent cardiac magnetic resonance (CMR), defining the correlation with morpho-functional or myocardial tissue alterations and arrhythmias.
**Methods or Backround:** Multi-center observational study involving 13 Italian hospitals. CMR from January to June 2019 were evaluated, assessing the presence of MAD, structural (prolapse, curling, regurgitation) and tissue alterations (LGE, T1, T2, ECV), volumetric and functional features, clinical suspicion, diagnosis, presence and type of arrhythmias.
**Results or Findings:** From a total of 2611 patients (67% XX, 53 [IQR 39-65] years old), 5.4% (142 patients, 65% XX, 52 [IQR39-63] year-old) had MAD. Of them, 8% underwent CMR for the suspicion of valvopathy, 5% for arrhythmias and 87% for other causes. 47/142 (33%) patients had arrhythmias, associated with valve prolapse (p=0.004) and bigger MAD length (p< 0.001). 83/142 (58%) had MAD without other cardiomyopathies, with prolapse in 43% of cases, associated with increased incidence of curling (64% vs 17%; p-value<0.001) and higher ECV values (29% vs 25%; p=0.003). Bi-leaflet prolapse was associated to more severe MAD compared to patients with single-leaflet prolapse or without (6 vs 3.5 and 4 mm; p=0.083), bigger left atrial volume (40 mL/ m2 vs 2 mL/ m2 and 27 mL/ m2; p=0.011), left ventricle volume (170 mL vs 134 and 111 mL; p< 0.001), higher rate of moderate-severe regurgitation (45% vs 6% and 0%; p< 0.001) and arrhythmias (63% vs 36%; p=0.037), without significant differences in the presence of LGE, whereas more frequent (35% vs 25% and 27%; p=0.794).
**Conclusion:** MAD is frequent in the population even without valvopathies. Its severity, the association with structural alterations lead to a higher risk of myocardial remodelling and arrhythmias.
**Limitations:** Absence of follow-up data.
**Ethics committee approval:** Prot. MIAMI
**Funding for this study:** No funding was received for this study.
**Author Disclosures:**
Antonio Esposito: Nothing to disclose
Elisa Bruno: Nothing to disclose
Riccardo Faletti: Nothing to disclose
Anna Palmisano: Nothing to disclose
Silvia Pradella: Nothing to disclose
Carlo Liguori: Nothing to disclose
Marco Gatti: Nothing to disclose
Serena Dell'Aversana: Nothing to disclose
Nicola Galea: Nothing to disclose

**15:00-16:00** **Research Stage 2**

### Research Presentation Session: Artificial Intelligence & Machine Learning & Imaging Informatics

### RPS 505

### Optimising radiological excellence: the synergy of AI and quality in radiology

**Moderator**
N. Buls; Jette/BE
*(nico.buls@uzbrussel.be)*
**Author Disclosures:**
Nico Buls: Advisory Board: GE Healthcare; Speaker: GE Healthcare

### RPS 505-2

**Assessing visual quality variability in deep learning reconstructed MRI: a focus on lesions**

*Q. Y. van Lohuizen*[1], S. Fransen[1], C. Roest[1], T. Kwee[1], F. Simonis[2], D. Yakar[1], H. Huisman[3]; [1]Groningen/NL, [2]Enschede/NL, [3]Nijmegen/NL

**Purpose:** This study assessed the structural similarity index measure (SSIM), the leading metric for image quality, for its reliability in evaluating deep learning reconstructed (DLRecon) MRI scans. The study particularly focused on images reconstructed using a state-of-the-art DLRecon algorithm, targeting full transversal, prostate regions and lesion-specific fields of view (FOV) for SSIM assessment.
**Methods or Backround:** A retrospective analysis was conducted using two datasets. The recurrent inference machine (RIM), a k-space-based DLRecon algorithm, was trained on the public prostate NYU fastMRI k-space dataset (N=312) and externally validated using prostate MRI scans of eight patients from the University Medical Center Groningen. Clinically significant prostate cancer lesions (N=17) with PI-RADS scores from three to five were delineated by expert radiologists. Image quality was assessed using SSIM on three FOVs: the full transversal FOV, the prostate, and the lesion level. Assessments were conducted at varying acceleration factors from 2x to 8x.
**Results or Findings:** Significant differences in SSIM values were observed across FOVs in MRI scans reconstructed by the DLRecon algorithm. Specifically, lesion FOVs had lower SSIM values (0.482± 0.057 at 8-fold acceleration) compared to the full transversal FOV (0.905± 0.018) and the prostate FOV (0.870± 0.023). All differences were statistically significant (p< 0.001, Wilcoxon tests). Furthermore, linear mixed-effects models revealed a significantly steeper rate of SSIM degradation in lesion-specific FOVs, suggesting greater variability in image quality in these critical areas.
**Conclusion:** DLRecon algorithms like the RIM showed significantly lower SSIM values in lesion-specific FOVs compared to the full transversal and prostate FOVs. This discrepancy calls into question the adequacy of SSIM as a standalone quality metric, emphasising the need for more targeted quality assessments in future DLRecon algorithm development.
**Limitations:** No limitations were identified.
**Ethics committee approval:** The study is retrospective.
**Funding for this study:** Funding was provided by Health~Holland and Siemens Healthineers.
**Author Disclosures:**
Henkjan Huisman: Nothing to disclose
Stefan Fransen: Nothing to disclose
Quintin Yves van Lohuizen: Nothing to disclose
Thomas Kwee: Nothing to disclose
Derya Yakar: Nothing to disclose
Christian Roest: Nothing to disclose
Frank Simonis: Nothing to disclose

### RPS 505-3

**Image quality and sharpness improvement in coronary CT angiography using a deep-learning super-resolution reconstruction algorithm: a phantom study**

T. W. Holmes[1], S. Sharma[2], S. Ross[2], K. Schultz[2], P. Gleason[1], J. Schuzer[2], Z. Yu[2], R. Thompson[2], *A. Pourmorteza*[1]; [1]Atlanta, GA/US, [2]Vernon Hills, IL/US
*(amir.pourmorteza@gmail.com)*

**Purpose:** The purpose of this study was to investigate the performance of a super-resolution deep-learning-based reconstruction (DLR) algorithm named precision image quality engine (PIQE) developed for cardiac CT against two

Wednesday clinical reconstruction algorithms: adaptive iterative dose reduction (AIDR) and high-resolution DLR (AiCE).
**Methods or Backround:** We 3D-printed inserts with microfluidic channels (d= 0.25 - 3.5 mm) with stents and calcified plaques embedded and filled with dilutions of iodinated contrast agent. The inserts were placed inside a 12-cm diameter water tank and scanned on a clinical CT scanner with prospective ECG-gating: 120 kVp, exposure: 25, 50, 250, and 400 mAs. Images were reconstructed with matched parameters using AIDR, AiCE, and PIQE: 512 x 512 matrix, 0.312 x 0.312 x 0.5 mm3 voxel size. PIQE images were also reconstructed with a 1024 x 1024 matrix and 0.156 x 0.156 x 0.5 mm3. We evaluated CT number stability, contrast-to-noise ratio (CNR), and image sharpness as a function of radiation dose.
**Results or Findings:** CT number deviations from the 400 mAs baseline were measured in iodine, water, and fat inserts and were in the [-1.1 3.1], [-1.1, 3.4], and [-2.2 0.26] for AIDR, AiCE, and PIQE, respectively. CNRs between iodine, water, fat (soft plaque), and calcium (hard plaque), were between 36% to 97% higher for PIQE compared to AIDR, with maximum CNR improvement observed in the lowest dose (25 mAs) scans. AiCE images showed a 0% - 37% increase in CNR in low-dose scans (25,50 mAs), however, their CNR was between 11% to 27% lower for the higher-dose scans (400,250 mAs), compared to AIDR. MTF cutoff at 10% was 8.98, 10.68, 10.44, and 13.61 lp/cm for AIDR, AiCE, PIQE, and PIQE1024 respectively.
**Conclusion:** Overall, DLR algorithms improved CNR and image sharpness between 16%-18% at normal resolution voxel size. Furthermore, PIQE improved image sharpness by 51% when reconstructed at high-resolution voxel size.
**Limitations:** More experiments mimicking different patient sizes are warranted.
**Ethics committee approval:** No information provided by the submitter.
**Funding for this study:** This study was sponsored research agreement with Canon Medical Research USA.
**Author Disclosures:**
Zhou Yu: Employee: Canon Medical USA
Steven Ross: Employee: Canon Medical USA
Richard Thompson: Employee: Canon Medical USA
Amir Pourmorteza: Research/Grant Support: Cannon Medical USA; Advisory Board: CarioWise Inc; Employee: Emory University; Research/Grant Support: GE HealthCare; Research/Grant Support: Siemens Healthineers
Thomas Wesley Holmes: Research/Grant Support: Cannon Medical USA; Consultant: CarioWise Inc; Employee: Emory University; Research/Grant Support: GE HealthCare; Research/Grant Support: Siemens Healthineers
Patrick Gleason: Employee: Emory University
Kurt Schultz: Employee: Canon Medical USA
John Schuzer: Employee: Canon Medical USA
Shobhit Sharma: Employee: Canon Medical USA

### RPS 505-4

**Elevating TIPSS procedures: AI denoising's impact on cone-beam CT image quality and diagnostic confidence**

*R. Dehdab*, A. S. Brendlin, S. Afat, G. Grözinger, K. Nikolaou, J. Mück; Tuebingen/DE
*(reza.dehdab@med.uni-tuebingen.de)*

**Purpose:** This study aimed to assess the utility of artificial intelligence (AI)-based denoising techniques in enhancing cone-beam computed tomography (CBCT) images during transjugular intrahepatic portosystemic shunt (TIPSS) procedures, taking into account variations in body mass index (BMI).
**Methods or Backround:** A retrospective review of 60 patients undergoing TIPSS between 2016 and 2022 was approved. Patients were categorised and paired based on BMI and divided according to image acquisition durations of three seconds (3s) and six seconds (6s). CBCT images were processed with AI denoising (ClariCT.AI, ClariPI®). Image quality was quantitatively and qualitatively evaluated, considering image noise, radiation dose, and Contrast-to-noise ratio (CNR) and diagnostic confidence. Relationships between BMI, noise, CNR and radiation doses were also analysed.
**Results or Findings:** AI-based deep learning denoising (DLR) reduced image noise in 3s and 6s acquisition groups. Despite higher initial noise in the 3s group, Post-DLR, noise equalised (3s-Original vs. DLR: T-Statistic: 57.06, p<0.001; 6s-Original vs. DLR: T-Statistic: 41.11, p<0.001). Positive BMI-noise correlation occurred in 3s (r=0.564, P-value=0.006), not in 6s (r=0.3738, P=0.09). Both groups showed a strong BMI-radiation dose correlation (3s-Correlation Coefficient (r): 0.681, p<0.001; 6s- Correlation Coefficient (r): 0.681, p<0.001). Diagnostic confidence was higher in 6s DLR (Kruskal-Wallis H Test: Statistic: 152.63, p<0.001), with 3s DLR achieving comparable confidence to 6s originals (Mann-Whitney U Test: Statistic: 3943.5, p=0.83).
**Conclusion:** In conclusion, our study demonstrates that AI-enhanced denoising techniques in CBCT images during TIPSS procedures not only significantly improve image quality but also reduce radiation exposure, highlighting the potential of AI in medical imaging for optimising diagnostic accuracy while prioritising patient safety.
**Limitations:** The study's relatively small sample size and single-centre setting may limit the generalisability of the findings.
**Ethics committee approval:** This study was retrospectively approved by the Institutional Review Board with a waiver for the need for informed consent (#167/2022BO2).
**Funding for this study:** No funding was received for this research.
**Author Disclosures:**
Gerd Grözinger: Nothing to disclose
Konstantin Nikolaou: Nothing to disclose
Andreas Stefan Brendlin: Nothing to disclose
Saif Afat: Nothing to disclose
Reza Dehdab: Nothing to disclose
Jonas Mück: Nothing to disclose

### RPS 505-5

**3D human reconstruction algorithm: a novel technique for prospective image quality control in chest radiography**

*Y. Tan*, P. Wu, Z. Ye, Y. Hou, C. Xia, Z. Li; Chengdu/CN
*(1371584795@qq.com)*

**Purpose:** The purpose of this study was to propose a novel real-time 3D human reconstruction algorithm and investigate its performance of identifying incorrect body postures and radiographer's operations in chest radiography.
**Methods or Backround:** A total of 83 chest post-anterior (PA) images and 71 chest lateral (LA) images shot by different radiographers were included for this study. The 3D human reconstruction algorithm took a photo as input and output a 3D mesh containing body morphology information, which was mainly based on a series of deep neural networks, including SMPL-X and HybrIK-X. A camera fixed in front of the beam limiter was used to capture photos at the time of exposure. Automatic measurement tools were developed for 3D human evaluation. Indexes including shrug (PA), scapula position (PA), arms up (LA), postures, exposure field, and projection point were assessed in both subjective and 3D human evaluation. Subjective results were regarded as reference standard. Sensitivity, specificity, and Kappa consistency of each index were calculated.
**Results or Findings:** In the chest PA of 3D human evaluation, the accuracy of identifying exposure field was 100%. The sensitivity, specificity, and Kappa value of shrug, scapula position, postures, and projection point were 0.82, 0.92, 0.73; 0.86, 0.83, 0.69; 1, 0.93, 0.73; 0.54, 0.86, 0.42. In the chest LA of 3D human evaluation, the accuracy of identifying exposure field was also 100%. The sensitivity, specificity and Kappa value of arms up, postures and projection point were 0.83, 0.94, 0.63; 0.95, 0.80, 0.65; 0.57, 1, 0.68.
**Conclusion:** 3D human reconstruction algorithm showed good ability in identifying incorrect body postures and radiographer's operations in chest radiography. Further improvement of this algorithm is needed to enhance its accuracy.
**Limitations:** The sample size was small and the measurements of projection point in 3D human evaluation needed modification.
**Ethics committee approval:** This study was retrospective, so did not require an ethics review.
**Funding for this study:** No funding was received for this study.
**Author Disclosures:**
Chunchao Xia: Nothing to disclose
Peng Wu: Nothing to disclose
Zhenlin Li: Nothing to disclose
Yuqi Tan: Nothing to disclose
Zheng Ye: Nothing to disclose
Yuzhou Hou: Nothing to disclose

### RPS 505-6

**MRI-radiomics for MGMT promoter methylation prediction in glioma: methodological quality, systematic review, and meta-analysis**

*F. M. Doniselli*, R. Pascuzzo, M. Moscatelli, M. Grisoli, L. M. Sconfienza; Milan/IT

**Purpose:** This study aimed to evaluate the methodological quality and diagnostic accuracy of MRI-based radiomic studies predicting O6-methylguanine-DNA methyltransferase (MGMT) promoter methylation status in gliomas.
**Methods or Backround:** PubMed Medline, EMBASE, and Web of Science were searched to identify MRI-based radiomic studies on MGMT methylation in gliomas published until December 31, 2022. Three raters evaluated the study methodological quality with Radiomics Quality Score (RQS, 16 components) and Transparent Reporting of a Multivariable Prediction Model for Individual Prognosis Or Diagnosis (TRIPOD, 22 items) scales. Risk of bias and applicability concerns were assessed with QUADAS-2 tool. A meta-analysis was performed to estimate the pooled area under the curve (AUC) and to assess inter-study heterogeneity.
**Results or Findings:** We included 26 studies. The median RQS total score was 8 out of 36 (22%, range: 8%-44%). Thirteen studies performed external validation. All studies reported AUC or accuracy, but only 4 (15%) performed calibration and decision curve analysis. No studies performed phantom analysis, cost-effectiveness analysis, and prospective validation. The overall TRIPOD adherence score was between 50% and 70% in 16 studies and below

50% in 10 studies. The pooled AUC was 0.78 (95% CI: 0.73-0.83, I2=93.4%) with a high inter-study heterogeneity. Studies with external validation and including only WHO-grade IV gliomas had significantly lower AUC values (0.65, 95% CI: 0.57-0.73, p<0.01).
**Conclusion:** Study RQS and adherence to TRIPOD guidelines was generally low. Radiomic prediction of MGMT methylation status showed great heterogeneity of results and lower performances in grade IV gliomas, which hinders its current implementation in clinical practice.
**Limitations:** Some included studies did not report AUC values; grey literature was not included.
**Ethics committee approval:** This study was a meta-analysis.
**Funding for this study:** No funding was received for this study.
**Author Disclosures:**
Marina Grisoli: Nothing to disclose
Fabio Martino Doniselli: Nothing to disclose
Marco Moscatelli: Nothing to disclose
Luca Maria Sconfienza: Nothing to disclose
Riccardo Pascuzzo: Nothing to disclose

### RPS 505-7

**Automatic uncertainty-based quality controlled segmentation of multimodal cardiac MR images**

J. Michaud, T. W. Arega, *S. Bricq*; Dijon/FR
*(stephanie.bricq@u-bourgogne.fr)*

**Purpose:** Although deep learning-based segmentation methods have shown promise in automating the segmentation of cardiac MRI images, they are not widely used in clinical practice due to the lack of robustness and reliability of the models. We propose an uncertainty-based quality control (QC) framework to identify failed segmentations and to enhance the reliability of multimodal cardiac MRI segmentation models.
**Methods or Backround:** Automatic and accurate analysis of myocardial tissue characterisation is highly dependent on the quality of the segmentation result. We proposed here an automatic quality controlled T1 mapping and LGE segmentation. The cardiac structures were segmented from LGE, native and post-contrast T1 mapping images using a Bayesian Swin Transformer-based U-Net. The quality of the segmentation output is assessed using uncertainty-based QC metrics. These uncertainty features are used as inputs to a random forest-based classifier to evaluate the segmentation quality and reject bad segmentations. The proposed framework was tested on a private cardiac MR dataset with various diseases.
**Results or Findings:** The proposed uncertainty-based quality control framework is robust in detecting inaccurate segmentations. Proposed QC method achieves an area under the ROC curve (AUC) of 0.922 for native T1 images, 0.886 for post-contrast T1 images, and 0.918 for LGE images on binary classification (bad or good segmentation).
**Conclusion:** The proposed framework automatically segments cardiac structures on multimodal MR images and rejects inaccurate segmentation results. It can be applied to other segmentation methods to detect segmentation failures and to enhance the reliability of the segmentation models.
**Limitations:** In addition to identify segmentation failures, it could be interesting to understand the underlying causes or sources behind them. By gaining insights into why certain segmentation results are rejected, the model’s reliability could be enhanced.
**Ethics committee approval:** No information provided by the submitter.
**Funding for this study:** Funding was received from the French National Research Agency (ANR) with reference number: ANR-19-CE45-0001-01-ACCECIT.
**Author Disclosures:**
Stéphanie Bricq: Nothing to disclose
Joffrey Michaud: Nothing to disclose
Tewodros Weldebirhan Arega: Nothing to disclose

### RPS 505-8

**The performance of a commercial artificial intelligence algorithm in an external quality assurance scheme regularly used by humans in the NHS breast screening programme**

*Y. Chen*, A. Taib, I. Darker, J. James; Nottingham/UK
*(yan.chen@nottingham.ac.uk)*

**Purpose:** The purpose of this study was to compare the performance of human readers in the National Health Service Breast Screening Programme and a commercially available artificial intelligence (AI) algorithm when interpreting Personal Performance in Mammographic Screening (PERFORMS) external quality assurance test sets.
**Methods or Backround:** Two consecutive PERFORMS sets, each consisting of 60 challenging normal, benign, and biopsy proven malignant mammography cases, were assessed by humans between 2018 and 2021 and AI in 2022. Suspicion of malignancy scores were assigned to features detected. Each breast was considered separately, and the highest score was used to assess performance using a pre-defined recall threshold. Sensitivity, specificity and ROC analysis was used to compare the performance of AI and human readers retrospectively.
**Results or Findings:** 552 human readers interpreted both PERFORMS sets. There were 161 normal, 70 malignant, and 9 benign breasts. There was no difference in the area under the receiver operating curve for AI and human readers (0.93 and 0.88 respectively, p=0.15). At the developer suggested recall threshold, there was no difference in AI and human reader sensitivity (84% and 90% respectively, p=0.34), but the specificity of the AI was higher than the human readers (89% and 76% respectively, p=0.003). Using recall thresholds to match mean human reader performance (90% sensitivity and 76% specificity), the AI showed no difference in performance with a sensitivity of 91% (p=0.73) and specificity of 77% (p=0.85).
**Conclusion:** Diagnostic performance of AI was comparable to that of the average human reader when evaluating cases from two enriched test sets of the PERFORMS scheme.
**Limitations:** The PERFORMS sets consist of a small number of mammograms. These are not representative of typical screening populations as they are enriched with challenging malignant cases.
**Ethics committee approval:** Following discussion with the local research and development team, the requirement for ethical approval was waived due to the retrospective nature of the study.
**Funding for this study:** Funding for this study was received from Lunit Inc.
**Author Disclosures:**
Adnan Taib: Nothing to disclose
Jonathan James: Nothing to disclose
Iain Darker: Nothing to disclose
Yan Chen: Nothing to disclose

**15:00-16:00 Research Stage 3**

### Research Presentation Session: Musculoskeletal

### RPS 510

### Upper extremities and tumours

**Moderator**
M. Rupreht; Maribor/SI
*(mitja.rupreht@guest.arnes.si)*

### RPS 510-2

**Deep-learning accelerated 3T MRI of the shoulder after rotator cuff repair: a comparison to standard 3T MRI**

*R. P. Marcus*, G. C. Feuerriegel, A. A. Marth, D. Nanz, R. Sutter; Zurich/CH
*(roy.marcus@outlook.com)*

**Purpose:** This study aimed to assess the diagnostic performance of accelerated MRI acquisition with deep-learning reconstruction and edge sharpening in patients after rotator cuff repair.
**Methods or Backround:** Unenhanced MRI scans of the operated shoulder were performed on 116 patients using a 3 Tesla (T) scanner (Magnteom Prisma, Siemens Healthineers). Standard and accelerated protocols were used during the same scanning session acquiring three sequences in coronal plane (STIR, PD TSE and PD FS), two sequences in sagittal plane (STIR and T1 TSE), and one sequence in axial plane (PD FS) with a slice thickness of 3mm each. The acquisition times of the standard and the accelerated protocols were recorded. Two experienced radiologists, blinded to the acquisition protocol, evaluated postsurgical changes of the tendons as per Sugaya classification (1: sufficient thickness, 2: sufficient thickness with partial high intensity, 3: insufficient thickness, 4: minor discontinuity, 5: major discontinuity).
**Results or Findings:** Interrater and intrarater reliabilities of the evaluated 207 operated tendons were strong (Kappa=0.824-0.863). The accelerated protocol reduced the acquisition time by 80% (3.7mins (2.5-6.3) vs. 18.4mins (17-33.7) for accelerated vs. standard protocol, p<0.001). A total of four intrarater disagreements were observed (2%), mostly due to motion artifacts in the standard acquisition. No motion artifact was recorded during the accelerated protocol.
**Conclusion:** Accelerated MRI acquisition with deep-learning reconstruction and edge sharpening significantly shortened the acquisition time while an excellent depiction of evaluating the condition of the shoulder after rotator cuff repair.
**Limitations:** This study has a small sample size.
**Ethics committee approval:** This study was approved by a local Ethics Committee in the Canton of Zurich.

**Funding for this study:** No funding was received for this study.
**Author Disclosures:**
Adrian Alexander Marth: Nothing to disclose
Daniel Nanz: Nothing to disclose
Reto Sutter: Nothing to disclose
Georg Constantin Feuerriegel: Nothing to disclose
Roy P. Marcus: Nothing to disclose

### RPS 510-3

**Bone mineral density assessment from spectral localiser radiographs: proof-of-concept with clinical photon counting detector CT**

*L. J. Moser*[1], J. Pitteloud[1], V. Mergen[1], D. Frey[1], T. Nowak[2], O. Distler[1], M. Eberhard[3], H. Alkadhi[1]; [1]Zurich/CH, [2]Forchheim/DE, [3]Unterseen/CH

**Purpose:** This study aimed to evaluate the feasibility and accuracy of bone mineral density (BMD) quantification from spectrally acquired localiser radiographs (LRs) with photon-counting detector (PCD)-CT, in comparison with DXA.
**Methods or Backround:** For this experimental study, spectrally-resolved LRs on a clinical dual-source PCD-CT scanner (NEAOTOM Alpha, Siemens) acquired with 140kVp and with four different tube currents (10mA, 20mA, 30mA and 40mA) of the European Spine Phantom were obtained. Scans were repeated three times. We used two setups: (A) the phantom placed directly on the CT table and (B) the phantom submerged in water with an anterior-posterior diameter of 30cm simulating a slightly overweight patient. Corresponding DXA scans were acquired. Assessment of aBMD in LRs was enabled by leveraging the spectral information with material decomposition maps for hydroxyapatite and water. The spine phantom-supplied values were used as the reference standard.
**Results or Findings:** The mean absolute error (MAE) in setup A for LR-derived aBMD across all tube currents and vertebrae was 0.003g/cm2 with a relative difference of ΔaBMD ranging from -1.5% to 1.1%. Coefficients of variance (CV) were below 1% stratified for tube current in setup A. In setup B, the MAE was 0.018g/cm2, and the ΔaBMD ranged from +1.0% to +5.3%. CV was below 3% for all tube currents and below 2% excluding 10mA. The absolute error of DXA scans in both setups (-0.041g/cm2 for setup A, -0.06g/cm2 for setup B) was higher than corresponding MAEs from PCD-CT.
**Conclusion:** Our phantom study indicates that LRs obtained with PCD-CT enable the calculation of BMD with high accuracy.
**Limitations:** This was a phantom study and represents pre-clinical software to reconstruct raw data from LRs.
**Ethics committee approval:** This study was performed on a phantom.
**Funding for this study:** No funding was received for this study.
**Author Disclosures:**
Janique Pitteloud: Nothing to disclose
Victor Mergen: Nothing to disclose
Matthias Eberhard: Nothing to disclose
Oliver Distler: Nothing to disclose
Lukas Jakob Moser: Nothing to disclose
Tristan Nowak: Employee: Siemens
Diana Frey: Nothing to disclose
Hatem Alkadhi: Speaker: Siemens

### RPS 510-4

**Automatic motion analysis of the wrist using dynamic CT imaging for diagnosis of scapholunate ligament injuries**

*M. Haenen*, E. Teule, S. Hummelink, I. Sechopoulos, B. van der Heijden; Nijmegen/NL
*(maranda.haenen@radboudumc.nl)*

**Purpose:** This study aimed to analyse wrist kinematics in healthy and scapholunate interosseous ligament (SLIL) injured wrists using four-dimensional computed tomography (4DCT) and a fully automated motion analysis algorithm.
**Methods or Backround:** 4DCT scans of 41 healthy wrists and eight wrists with arthroscopically-confirmed Geissler 4 SLIL injuries were acquired. Each scan consisted of one conventional static CT image and two dynamic imaging sequences; wrist radial-ulnar deviation (RUD) and flexion-extension (FE). Scanner rotation time was 0.275s, and acquisition parameters include 80 kV and 40 mAs. A sampling rate of 10Hz resulted in 144 dynamic 4DCT frames per wrist. Bones were automatically segmented in each frame using an artificial intelligence-based algorithm, and the scapholunate distance (SLD) and sagittal scapholunate angle (SLA) were automatically estimated per dynamic frame and resampled to a uniform distribution of wrist positions for intersubject comparison. The median and maximum SLD and SLA values were calculated. A Mann-Whitney U test was employed to compare both groups.
**Results or Findings:** The median and maximum SLDs and SLAs were significantly larger in the injured group compared to the healthy group. For example, the median and interquartile range were as follows: RUD (SLD) 2.49mm [1.36-3.40mm] vs 0.86mm [0.64-1.06mm], p<.001; RUD (SLA) 93.0º [83.8-103.0º] vs 69.2º [62.3-74.1º], p<.001 and FE (SLD) 1.99mm [1.31-3.23mm] vs 0.92mm [0.71-1.17mm], p<.001; FE (SLA) 92.3º [66.6-102.3º] vs 71.9º [57.0-85.2º], p<.001).
**Conclusion:** 4DCT is an emerging modality that enables non-invasive analysis of wrist motion. SLDs and SLAs automatically estimated from 4DCT scans during wrist motion could be used to diagnose SLIL injuries non-invasively.
**Limitations:** The small sample size of injured wrists and the lack of independent validation of quantitative estimates limit this study.
**Ethics committee approval:** This study was approved with the ABR number: NL72518.091.19.
**Funding for this study:** This study was internally funded.
**Author Disclosures:**
Erin Teule: Nothing to disclose
Ioannis Sechopoulos: Speaker: Siemens healthcare; Research/Grant Support: Siemens Healthcare, Canon Medical Systems, ScreenPoint Medical, Sectra Benelux, Volpara Healthcarea and Lunit; Advisory Board: Koning Corp.
Brigitte van der Heijden: Nothing to disclose
Maranda Haenen: Nothing to disclose
Stefan Hummelink: Nothing to disclose

### RPS 510-5

**Initial findings of IVIM-DWI validation using DCE-MRI in soft tissue sarcomas**

*G. Timoçin Yığman*, A. Peker, Y. E. Senturk, H. Özen Atalay; Istanbul/TR
*(gizemtimocin1@gmail.com)*

**Purpose:** The aim of this study was to investigate the relationship between quantitative DCE-MRI and intravoxel incoherent motion diffusion-weighted imaging (IVIM-DWI) parameters in patients with soft tissue sarcomas (STS).
**Methods or Backround:** From March 2022 to August 2023, patients were included who were histopathologically diagnosed as STS in our centre and who had a DCE-MRI and IVIM-DWI. Patients with a history of chemotherapy or radiotherapy before MRI and patients with a diagnosis of liposarcoma were excluded. Two radiologists with specific musculoskeletal imaging experience evaluated all the exams (3 and 4 years). Patients' age, gender, and the longest diameter of the mass (LD) were noted. DCE-MRI measurements were made from the Ktrans maps, avoiding bleeding, necrosis, and cystic areas. ROIs were placed the area with the highest perfusion in both. Permeability parameters of the masses (Ktrans, AUC, Kep, Ve) were noted. IVIM-DWI was obtained using axial EPI with 11-b values. IVIM-DWI was processed semi-automatically using Syngo.via V8.5 Frontiers (Siemens, Germany) and FD, D, and DP (D*) were calculated.
**Results or Findings:** Ten patients, two females and eight males, were included. The mean age of the patients was 58±15.05 (23-70), and the mean LD was 124±66.44 mm (56-280). Interobserver agreement was excellent for DCE-MRI and good for IVIM-DWI parameters. There was a strong correlation between K trans and FP (r=0.76; p=0.011), Kep and DP (r=0.77; p=0.009), and AUC and FP (r=0.72; p=0.020). No significant correlation was observed in other parameters.
**Conclusion:** No study in the literature compares IVIM-DWI and DCE-MRI parameters in tumours. Our study demonstrated a strong correlation between parameters. Studies with larger patient groups are needed to correctly interpret IVIM-DWI parameters and replace DCE-MRI.
**Limitations:** The study is limited by the small number of patients from a single centre.
**Ethics committee approval:** This study was approved by the Koc University Biomedical Research Ethics Committee, Istanbul.
**Funding for this study:** No funding was received for this study.
**Author Disclosures:**
Hande Özen Atalay: Nothing to disclose
Yunus Emre Senturk: Nothing to disclose
Gizem Timoçin Yığman: Nothing to disclose
Ahmet Peker: Nothing to disclose

### RPS 510-6

**Ultrasound-guided five per cent dextrose and corticosteroid injections in patients with carpal tunnel syndrome: one-year follow-up**

V. Potočnik Tumpaj, G. Omejec, *Ž. Snoj*; Ljubljana/SI

**Purpose:** This study aimed to compare the long-term treatment effects of ultrasound (US)-guided five per cent dextrose and corticosteroid injections in patients with carpal tunnel syndrome (CTS).

**Methods or Backround:** We prospectively recruited 57 patients with mild to moderate bilateral CTS. Symptom severity and functional status were assessed with the Boston Carpal Tunnel Questionnaire (BCTQ) and visual analogue scale (VAS). Electrodiagnostic studies (EDx) and median nerve US were performed. For the left wrist, injectate was randomly selected (6ml of five per cent dextrose or 2ml of corticosteroid mixed with 4ml saline). Afterwards, the right wrist was injected with the remaining solution. Patients and examiners were blinded to the selection of the solution. Follow-up was performed at one, three, six, and 12 months.
**Results or Findings:** Both solutions demonstrated significant improvement for clinical outcome in VAS (at one, three and six months follow-up; p<0.05) and BCTQ score (at one, three, six, and 12 months follow-up; p<0.05) compared to baseline. No changes in median nerve cross-sectional area (CSA) were present, however, the flattening ratio significantly improved at three, six, and 12 months follow-up (p<0.05). No significant difference was present between solutions at each follow-up (p>0.05).
**Conclusion:** Ultrasound-guided carpal tunnel injections of corticosteroids and five per cent dextrose significantly improve clinical outcomes in patients with CTS up to one-year follow-up. No differences were present between solutions.
**Limitations:** No limitations were identified.
**Ethics committee approval:** This study was approved by the Slovenian National Ethics Committee.
**Funding for this study:** No funding was received for this study.
**Author Disclosures:**
Gregor Omejec: Nothing to disclose
Vesna Potočnik Tumpaj: Nothing to disclose
Žiga Snoj: Nothing to disclose

### RPS 510-7

**Bone lymphoma presenting with lower back pain: a case series**

*V. Di Martino*, N. Di Meglio, A. Perrella, G. Bagnacci, C. Sica, M. A. Mazzei; Siena, SI/IT
*(dimartino7@student.unisi.it)*

**Purpose:** We report six cases of bone lymphoma presenting with lower back pain which occurred over a two year period, from 2020 to 2022, in our centre. This study highlights relevant imaging features with a special focus on MR.
**Methods or Backround:** Bone lymphoma presenting with musculoskeletal pain is an often challenging diagnosis because its imaging features are nonspecific. It is classified either as primary bone lymphoma (PBL, no other extraosseous site involved), or secondary bone involvement in systemic lymphoma (SBL, secondary bone lymphoma). SBLs are much more frequent (30-50% of all non-Hodgkin Lymphomas, NHLs, and 5- 10% of all Hodgkin Lymphomas, HLs), while primary bone lymphomas are rare (<5% of primary bone tumours and 1% of all NHLs, more often a form of diffuse large B cell lymphoma, DLBCL).
**Results or Findings:** Four females and two males (aged 21 to 80 years) with persistent lower back pain underwent an MRI and subsequently a body contrast-enhanced CT and/or a PET/CT. The diagnosis was confirmed with a biopsy: four PMLs (DLBCL), two SBLs (one HL and one NHL). In all cases bone lesions were characterised by marked signal hypointensity on T1-weighted MRI corresponding to heterogeneous hyperintensity on T2-weighted images, asymmetric, not confined to the subchondral bone and, in two cases, also associated with soft tissue involvement but without significant cortical bone destruction. In four cases bone lesions were visible at CT with a permeative or lytic pattern. Both cases of SBL presented systemic symptoms as well (fever and night sweats).
**Conclusion:** Bone lymphoma should always be considered in the differential diagnosis of patients presenting with lower back pain, especially if the imaging features of bone lesions are atypical or associated with fever, night sweats, and weight loss.
**Limitations:** This study is limited by its case series methodology.
**Ethics committee approval:** No information provided by the submitter.
**Funding for this study:** No funding was received for this study.
**Author Disclosures:**
Maria Antonietta Mazzei: Nothing to disclose
Vito Di Martino: Nothing to disclose
Cristian Sica: Nothing to disclose
Armando Perrella: Nothing to disclose
Nunzia Di Meglio: Nothing to disclose
Giulio Bagnacci: Nothing to disclose

**15:00-16:00 Research Stage 4**

### Research Presentation Session: Oncologic Imaging

### RPS 516

### Whole-body MRI in oncology

**Moderator**
M. Zerunian; Rome/IT
*(marta.zerunian@gmail.com)*
**Author Disclosures:**
Marta Zerunian: Speaker: Ge Healthcare, Bayer, Bracco, Philips

### RPS 516-2

**Patients' selection for pelvic exenteration for gynaecologic neoplasm using whole-body MRI: preliminary results**

*S. Persiani*, G. Avesani, A. Perazzolo, L. D'Erme, C. Panico, V. Rufini, B. Gui, E. Sala; Rome/IT
*(salvatorepersiani@gmail.com)*

**Purpose:** Pelvic exenteration is used to treat recurrent pelvic localisation in gynaecological patients. Critical patient selection is crucial due to high postoperative morbidity. Typically, patients undergo preoperative MRI to evaluate local disease extent and FDG-PET-CT to detect lymph nodes and distant metastasis. This study aims to evaluate whole-body-MRI (WB-MRI) in detecting extrapelvic disease in gynaecologic cancer patients awaiting pelvic exenteration and to test WB-MRI's reproducibility among radiologists.
**Methods or Backround:** In this IRB-approved study from June 2021 to September 2023, patients with recurring gynaecological cancer prepped for pelvic exenteration underwent standard FDG-PET-CT, pelvic MRI, and additional sequences to assess extrapelvic disease, adding 20-25 minutes to the MRI exam time. Two radiologists reviewed WB-MRIs for extrapelvic involvement, comparing findings to FDG-PET-CT. Diagnostic accuracy and interradiologist agreement were evaluated.
**Results or Findings:** We included 36 patients. Seven patients had extra-pelvic localisations at PET-CT and were excluded from surgery. WB-MRI correctly identified six patients with extrapelvic involvement; the patient not identified had a PET-positive para-aortic lymph node with a short axis of 8mm. WB-MRI identified one patient with a suspicious peritoneal nodule, which was PET-negative and histologically defined as fibrotic area after surgery. The radiological PI showed a sensitivity of 0.85 (CI 0.42-0.99), specificity of 0.96 (CI 0.82-0.99), positive predictive value of 0.91 (CI 0.60-0.99) and negative predictive value of 0.94 (CI 0.72-0.97), with an overall accuracy of 0.93 (CI 0.80-0.99). The agreement between radiologists was 0.72.
**Conclusion:** WB-MRI has a good performance in detecting extrapelvic involvement in patients with gynaecologic malignancies and has good reproducibility; it may be considered a stand-alone technique for patient selection for pelvic exenteration for gynaecologic malignancies. However, these findings need to be confirmed in a larger sample.
**Limitations:** This was a monocentric study with a small sample size.
**Ethics committee approval:** This study was approved by the Fondazione Policlinico Gemelli Ethics Committee with reference number: 3813.
**Funding for this study:** No funding was received for this study.
**Author Disclosures:**
Vittoria Rufini: Nothing to disclose
Giacomo Avesani: Nothing to disclose
Benedetta Gui: Nothing to disclose
Alessio Perazzolo: Nothing to disclose
Evis Sala: Nothing to disclose
Salvatore Persiani: Nothing to disclose
Camilla Panico: Nothing to disclose
Luca D'Erme: Nothing to disclose

### RPS 516-3

**Performance comparison between whole body MRI and 18F-PSMA-1007 PET CT in the detection of tumour recurrence in prostate cancers**

*M. Garcia Fontes*, E. Otero, L. Valuntas, V. Gigirey, J. Mattos, G. Dos Santos, J. P. Gambini, P. Duarte, O. Alonso; Montevideo/UY
*(margarita.garciafontes@cudim.org)*

**Purpose:** The aim of this study was to determine whether Whole Body Magnetic Resonance (WBMRI) with 3 Tesla offers similar performance to 18F-PSMA-1007 PET CT for recurrent tumour detection in prostate cancers.
**Methods or Backround:** A WBMRI scan with a 3T GE equipment and a 18F-PSMA-1007 PET CT with a 64 GE PET -CT were performed in patients with prostate cancer and the suspicion of tumour recurrence. Thirty patients between 55 and 81 years old, with a PSA value between 0.4 and 41 ng/ml, were included. The number of bone lesions, suspicious nodes, local recurrences, and incidental findings with both methods were compared. The WBMRI study protocol was based on the METastasis Reporting and Data System for Prostate Cancer (MET-RADS-P). An AXIAL T2 high Resolution and AXIAL DWI FOCUS sequences of the prostate were added to better the assessment of local tumour recurrence. Statistics analysis was performed.
**Results or Findings:** Both WBMRI and 18F-PSMA 1007 PET CT detected the same number of bone and lymph node metastases and local tumour recurrence. WBMRI found more bone lesions in two cases. Incidental findings revealed that both detected a neuroendocrine tumour in the right iliac fossa and the WBMRI detected a kidney tumour.
**Conclusion:** WBMRI seems to be an adequate method for the evaluation of tumour recurrences of prostate cancer with the advantages of greater accessibility, lower cost, and repeatability compared to PET CT18F-PSMA-1007. Further advantages include its lack of radiation and lack of contrast media usage.
**Limitations:** WBMRI requires patient collaboration due to its longer duration compared to PET CT PSMA, and some of these patients may be uncomfortable during the study. Biosafety limitations must also be considered. In order to validate the use of WBMRI in control of patients with prostate cancer recurrence, a larger series is necessary.
**Ethics committee approval:** No information provided by the submitter.
**Funding for this study:** No funding was received for this study.
**Author Disclosures:**
Pablo Duarte: Nothing to disclose
Laura Valuntas: Nothing to disclose
Elena Otero: Nothing to disclose
Juan Pablo Gambini: Nothing to disclose
Javier Mattos: Nothing to disclose
Veronica Gigirey: Nothing to disclose
Margarita Garcia Fontes: Nothing to disclose
Gerardo Dos Santos: Nothing to disclose
Omar Alonso: Nothing to disclose

### RPS 516-4

**Applying ONCO-RADS to whole-body MRI cancer screening in a retrospective cohort of asymptomatic subjects**

*C-J. Lin*[1], Y-S. Hu[2], C-A. Wu[1], H-J. Chiou[1]; [1]Taipei/TW, [2]New Taipei/TW
*(bcjlin@gmail.com)*

**Purpose:** The aim of this study was to evaluate the frequencies of cancer in asymptomatic subjects undergoing whole-body magnetic resonance imaging (WB-MRI) within the Oncologically Relevant Findings Reporting and Data System (ONCO-RADS) categories.
**Methods or Backround:** We retrospectively included 2064 asymptomatic subjects participating in the WB-MRI cancer screening program with a 3-T scanner between 2017 and 2022. Results of further investigations, including additional imaging and histopathology exams performed at our institute, were used to confirm cancer. Two radiologists blinded to the clinical outcome categorised the WB-MRI findings by using the ONCO–RADS categories as follows: 1 (normal), 2 (benign finding highly likely), 3 (benign finding likely), 4 (malignant finding likely), and 5 (malignant finding highly likely). Firth logistic regression analysis was conducted to determine the associations between the subject characteristics and findings of ONCO-RADS category ≥4.
**Results or Findings:** Of the 2064 subjects, 43 (2.1%) individuals had findings of ONCO-RADS category ≥4 and 24 (1.2%) had cancer confirmed. The cancer frequencies per subject were 0.1%, 5.4%, 42.9%, and 75% for ONCO-RADS category 2, 3, 4, and 5, respectively. In the multivariable model, older age (odds ratio (OR): 1.035, p=0.029), history of hypertension (OR: 2.051, p=0.026), hepatitis B carrier (OR: 2.584, p=0.013), and prior surgery (OR: 3.787, p<0.001) were independently associated with ONCO-RADS-category-≥4 findings.
**Conclusion:** The ONCO-RADS designed for cancer risk stratification was validated that higher numbers indicate greater likelihood of cancer. The application of ONCO-RADS facilitates risk-based management after WB-MRI for cancer screening.
**Limitations:** This was a retrospective study with contrast usage in the general population.
**Ethics committee approval:** This study was approved by the Taipei Veterans General Hospital Internal Review Board.
**Funding for this study:** Funding for this study was received with the code: MOST 112-2314-B-A49-064.
**Author Disclosures:**
Hong-Jen Chiou: Nothing to disclose
Chung-Jung Lin: Nothing to disclose
Yong-Sin Hu: Nothing to disclose
Chia-An Wu: Nothing to disclose

### RPS 516-5

**Predictive role of sustained imaging MRD negativity assessed by diffusion-weighted whole-body MRI in multiple myeloma**

*B. Frittoli*, A. Belotti, T. Falcone, C. Saeli, S. Gambarini, R. Ambrosini, A. Tucci, L. Grazioli; Brescia/IT
*(bfrittoli@gmail.com)*

**Purpose:** Diffusion-weighted whole-body magnetic resonance imaging (DW-MRI) is used in the management of multiple myeloma (MM) as part of the criteria for Response Assessment Category (RAC), as stated by the Myeloma Response Assessment and Diagnosis System (MY-RADS). Its prognostic usefulness in the detection of minimal residual disease (MRD) after autologous stem cell transplantation (ASCT) has been previously established. The goal of our study is to examine the predictive role of sustained imaging MRD-neg assessed by DW-MRI.
**Methods or Backround:** DW-MRI and RAC criteria were performed in 70 MM patients, both after ASCT and at 1-year during maintenance therapy in order to evaluate imaging-residual-disease. We combined DW-MRI results with those of bone marrow samples, collected at day +100 after ASCT and at 1-year in MRD-neg patients.
**Results or Findings:** Multivariable analysis showed that progression free survival (PFS) and overall survival (OS) were predicted by DW-MRI persistent disease (RAC≥2): p=0.001, HR 0.12 (95% CI: 0.05–0.30) for PFS, p=0.032, HR 0.20 (95% CI: 0.05–0.87) for OS. Median PFS was significantly longer for patients with imaging MRD-neg at 1-year (RAC 1) compared to patients with residual disease on DW-MRI (RAC≥2) (median PFS: 55.4 vs. 28.4 months; 3-years PFS: 91% vs. 30%, respectively (HR 0.12; 95% CI: 0.04–0.35; p=0.0001)). OS of imaging MRD-neg patients at 1-year was significantly longer for patients with RAC1 vs. RAC≥2 (median OS: not reached (NR) vs. 63 months; 3-years OS: 100% vs. 82%, respectively (HR 0.13; 95% CI: 0.03–0.66; p=0.0007)).
**Conclusion:** Sustained imaging MRD negativity assessed by DW-MRI with RAC criteria has strong predictive relevance for survival in MM patients on maintenance therapy after ASCT. This work has also been published in a journal (well done) (doi: 10.1002/ajh.26995).
**Limitations:** The retrospective nature of observations and the relatively low number of patients represent major limitations of our study.
**Ethics committee approval:** This study has been done in accordance with the Declaration of Helsinki. The submitter provided no additional information.
**Funding for this study:** No funding was received for this study.
**Author Disclosures:**
Teresa Falcone: Nothing to disclose
Roberta Ambrosini: Nothing to disclose
Alessandra Tucci: Nothing to disclose
Angelo Belotti: Nothing to disclose
Luigi Grazioli: Nothing to disclose
Chiara Saeli: Nothing to disclose
Sebastiana Gambarini: Nothing to disclose
Barbara Frittoli: Nothing to disclose

### RPS 516-6

**Potential added value of whole-body DWI-MRI in the diagnostic workup of patients with adenocarcinoma of unknown primary (ACUP): a prospective pilot study**

*J. Willemse*, M. Lahaye, P. Snaebjornsson, S. Marchetti, M. Vollebergh, L. van Golen, W. Vogel, R. G. H. Beets-Tan, D. M. J. Lambregts; Amsterdam/NL
*(j.willemse@nki.nl)*

**Purpose:** The purpose of this prospective diagnostic pilot study was to investigate the potential added benefit of whole-body (chest-abdomen) MRI including DWI (further referred to as WB-MRI) in this diagnostic work-up of patients with adenocarcinoma of unknown primary (ACUP).
**Methods or Backround:** From January 2022 to August 2023, the option of WB-MRI was added as an adjunct diagnostic tool to the routine clinical workup, including CT, FDG-PET/CT, lab tests, and biopsies for ACUP patients in our institution. The choice of whether to perform an MRI was discussed by a multidisciplinary team and guided by all available clinical information (e.g. if the primary tumour could likely be suspected to be located within the abdomen/pelvis). We analysed the impact of WB-MRI in terms of primary tumour identification and detection of additional metastatic sites.
**Results or Findings:** WB-MRI was performed in 27 ACUP patients. In 6/27 (22%) of patients, WB-MRI suggested a possible primary tumour location undiagnosed on previous CT and/or FDG-PET/CT, including 2 bile duct cancers, 1 ovarian, 1 appendiceal, 1 duodenal and 1 pancreatic cancer. In 5 of these 6 cases, the WB-MRI diagnosis aligned with and supported the final diagnosis, established by integration of clinicopathological data with whole genome sequencing. In addition, WB-MRI discovered extra metastatic sites in 6/27 (22%) of patients, including peritoneal metastases (n=3), bone (n=1), kidney (n=1) and testicular metastases (n=1).

**Conclusion:** This study demonstrates the potential added value of WB-MRI in the search for the underlying primary tumour in the complex diagnostic work-up of patients with disseminated adenocarcinoma of unknown primary.
**Limitations:** This was a small pilot study with a correspondingly small cohort.
**Ethics committee approval:** This was a retrospective analysis of data acquired prospectively as part of routine clinical care, approved by the local Institutional Research Board.
**Funding for this study:** No funding was received for this study.
**Author Disclosures:**
Larissa van Golen: Nothing to disclose
Max Lahaye: Nothing to disclose
Marieke Vollebergh: Nothing to disclose
Petur Snaebjornsson: Nothing to disclose
Wouter Vogel: Nothing to disclose
Doenja Marina Johanna Lambregts: Nothing to disclose
Jeroen Willemse: Nothing to disclose
Regina G. H. Beets-Tan: Nothing to disclose
Serena Marchetti: Nothing to disclose

### RPS 516-7

**The impact of whole-body MRI for staging unfavourable intermediate- and high-risk prostate cancer prior to curative-intent surgery, radiotherapy, hormonal therapy: all-in-one MRI preliminary data**

*C. M. Girlando*[1], S. Alessi[1], Y. Zambanini[1], S. Luzzago[1], B. Frittoli[2], S. Gambarini[2], L. Grazioli[2], G. Petralia[1]; [1]Milan/IT, [2]Brescia/IT

**Purpose:** This is a multicentric, prospective interventional study, with the aim of comparing the accuracy of Whole-Body Magnetic Resonance Imaging (WB-MRI) with that of conventional imaging (Computed Tomography and Bone Scintigraphy) for the systemic staging of patients with unfavourable intermediate- or high-risk prostate cancer (PCa) and how WB-MRI staging results affect the treatment decision.
**Methods or Backround:** In two IRCCS centres, patients with unfavourable intermediate- or high-risk PCa were enrolled and underwent systemic staging with conventional imaging and WB-MRI. Staging examinations were reported blinded to each other. A first systemic staging and treatment decision was made by the multi-disciplinary team based on conventional imaging staging only and, after the outcome of the WB-MRI, was formulated the definitive therapeutic proposal based on all available test results. We compared the diagnostic accuracy of WB-MRI staging with that of conventional imaging staging and reported how WB-MRI staging resulted in changes in treatment decision.
**Results or Findings:** This is a preliminary evaluation based on the first 80 subjects enrolled. Of these, 2 were excluded due to drop-out. In the remaining 78 patients WB-MRI, compared to conventional imaging, led to an upstage in 19 patients (24.4%) and to a downstage in 9 patients (11.5%), leading to a change in the therapeutic proposal in 17 patients (21.8%).
**Conclusion:** WB-MRI has shown superior diagnostic accuracy compared to conventional imaging in the systemic staging of patients with unfavourable intermediate- or high-risk PCa with better risk stratification and more appropriate treatment proposals.
**Limitations:** No limitations were identified.
**Ethics committee approval:** This study was approved by the Institutional Ethics Committee (UID 2080), and all patients provided informed consent for their participation in the study in addition to that for the individual radiological procedures.
**Funding for this study:** Funding was received from the Italian Ministry of Health with Ricerca Corrente and 5x1000 funds.
**Author Disclosures:**
Sarah Alessi: Nothing to disclose
Stefano Luzzago: Nothing to disclose
Giuseppe Petralia: Nothing to disclose
Cristiano Michele Girlando: Nothing to disclose
Ylenia Zambanini: Nothing to disclose
Luigi Grazioli: Nothing to disclose
Sebastiana Gambarini: Nothing to disclose
Barbara Frittoli: Nothing to disclose

### RPS 516-8

**Whole-body MRI in staging of diffuse large B-cell lymphoma**

*M. Wagnerová*, P. Vodicka, K. Benesova, A. Bocan, D. Zogala, A. Burgetova, M. Trneny, L. Lambert; Prague/CZ
*(monika.wagnerova@gmail.com)*

**Purpose:** In this study, we evaluated the diagnostic performance of whole body magnetic resonance imaging (wbMRI) in the staging of diffuse large B-cell lymphoma and patients' preference for the staging modality.
**Methods or Backround:** In this single-centre prospective study, adult patients diagnosed with DLBCL underwent wbMRI and 18-F FDG PET/CT as the reference standard. Two radiologists evaluated wbMRI for nodal (12 nodal regions) and extranodal involvement. Patients received a questionnaire about wbMRI.
**Results or Findings:** 14 of 78 patients consented to participate in and complete the study. The sensitivity and specificity of wbMRI in the assessment of nodal involvement was 0.84 and 1.00. Extranodal involvement was apparent in all 14 instances. Staging was concordant with PET/CT in all patients. The most frequent concern was the fear of an enclosed environment and the duration of the examination.
**Conclusion:** Despite excellent sensitivity and specificity of wbMRI in staging of DLBCL, wbMRI is preferred less by the patients due to the fear of an enclosed environment and the duration of the examination.
**Limitations:** There was a low number of patients willing to participate, and no evaluation of treatment response.
**Ethics committee approval:** This study was approved by the Ethics Committee of the General University Hospital in Prague (12/20 Grant VFN IGP).
**Funding for this study:** Funding for this study was received from the Czech Ministry of Health (MH CZ-DRO, General University Hospital in Prague - VFN, 00064165; VES NU21-03-00411) and from institutional funding at the Charles University in Prague (Cooperatio, Medical Diagnostics and Basic Medical Sciences and Haematology-Oncology).
**Author Disclosures:**
Lukas Lambert: Nothing to disclose
Katerina Benesova: Nothing to disclose
David Zogala: Nothing to disclose
Andrea Burgetova: Nothing to disclose
Andrej Bocan: Nothing to disclose
Monika Wagnerová: Nothing to disclose
Prokop Vodicka: Nothing to disclose
Marek Trneny: Nothing to disclose

**16:30-17:30** **Research Stage 1**

### Research Presentation Session: Breast

### RPS 602

### The role of breast imaging in breast cancer treatment

**Moderator**
N. A. Healy; Cambridge/UK
*(nualahealy@gmail.com)*

### RPS 602-2

**Comparison of radiomics-based machine-learning classifiers for pretreatment prediction of pathologic complete response to neoadjuvant therapy in breast cancer**

*X. Li*, C. Li, L. Jiang, M. Chen; Beijing/CN
*(lixue_shea@outlook.com)*

**Purpose:** In recent years, machine learning (ML) classifiers have been used to establish high-performance predictive models for pathological complete response (pCR) in breast cancer after neoadjuvant therapy (NAT). However, few studies have compared the effectiveness of different ML classifiers. This study investigated the ability of radiomics models based on pre- and post-contrast first-phase T1WI to predict breast cancer pCR after NAT and compared the performance of different ML classifiers.
**Methods or Backround:** In this retrospective study, 300 patients from the Duke-Breast-Cancer-MRI dataset who underwent NAT were included, including pCR (n=76) and non-pCR (n=224) cases. These patients were randomly divided into training and validation groups at a ratio of 8:2. Radiomics features were extracted from pre- and post-contrast first-phase T1WI images of each patient. The radiomics model was built using features selected through the Spearman correlation analysis and the LASSO algorithm after normalisation. Seven ML classifiers were used to assess the predictive performance of the radiomics models.
**Results or Findings:** Out of the seven classifiers used, the LightGBM classifier performed best in predicting breast cancer pCR, with an AUC of 0.813 in the validation group (accuracy 78.3%, sensitivity 46.7%, specificity 100.0%). During subgroup analysis, RF achieved the highest AUC in pCR prediction in luminal breast cancers (0.859, accuracy 85.9%, sensitivity 68.8%, and specificity 83.3%), and DT yielded the highest AUC in pCR prediction in triple negative (TN) breast cancers (0.909, accuracy 88.2%, sensitivity 81.8%, and specificity 100%).
**Conclusion:** Overall, the LightGBM-based radiomics model demonstrated superior performance in predicting breast cancer pCR, while RF and DT displayed promising results in predicting pCR for luminal and TN breast cancers, respectively, during subgroup analysis.

**Limitations:** Our study included different NAT treatment regimens, and subgroup analysis based on treatment regimens was not performed.
**Ethics committee approval:** Data obtained from the Cancer Imaging Archive (TCIA, https://www.cancerimagingarchive.net/) did not require ethical approval; informed consent was waived since the TCIA dataset de-identified patient information.
**Funding for this study:** No funding was received for this study.
**Author Disclosures:**
Xue Li: Nothing to disclose
Min Chen: Nothing to disclose
Chunmei Li: Nothing to disclose
Lei Jiang: Nothing to disclose

### RPS 602-3

**Ultrasound guided vacuum-assisted biopsy to assess pathological complete response to neoadjuvant therapy: a paradigm shift in breast cancer treatment**

*E. D'Ascoli*[1], C. Depretto[1], G. Della Pepa[1], C. De Berardinis[1], G. Irmici[1], C. Cazzella[2], D. Ballerini[1], A. Bonanomi[1], G. P. Scaperrotta[1]; [1]Milan/IT, [2]Bergamo/IT

**Purpose:** The aim of this study was to evaluate the diagnostic performance of a pre-surgical mini-invasive ultrasound-guided biopsy to predict pathological complete response (pCR) in breast cancer (BC) patients after neoadjuvant therapy (NAT) in order to assess the possibility of omitting surgery in exceptional responders.
**Methods or Backround:** We enrolled patients with histologically confirmed TN, Her2+ and Luminal B cT1-cT2-cT3 cN0-cN1 monofocal BC who received NAT with complete radiological remission on imaging (ultrasound, mammography, MRI and CEM) or with breast residual tumor <1cm. Patients underwent ultrasound guided vacuum-assisted breast biopsy (VABB) and the results were compared with the final histological results obtained after surgery.
**Results or Findings:** We enrolled 27 patients; 15/27 lesions were classified as TN, 6/27 Her2+ and 6/27 Luminal B. 22/27 cases showed complete radiological response; 5/27 had residual tumor <1cm. In two cases, VABB identified residual disease and post-surgical histological examination was negative. Overall, among the remaining 25 cases, in 22 (88%) there was concordance between the VABB result and the final histological examination. Among TN and Her2+ concordance was observed in 17/19 (89.5%) cases. VABB did not identify the presence of residual invasive disease in one case of Luminal B (16.7%) and in one case of TN (6.7%); in one TN case (6.7%) VABB was negative but definitive histological examination showed the presence of carcinoma in situ.
**Conclusion:** pCR rates are rapidly improving, especially in TN and Her2+ BC patients. Reliably identifying patients with pCR could lead to deescalation of locoregional therapy after NAT, allowing surgery to be omitted in exceptional responders; this would result in a reduction of post-surgical complications and healthcare costs, and improvement in quality of life.
**Limitations:** The main limitations are the single-centre nature of the study and the limited number of patients.
**Ethics committee approval:** This study was approved by the Independent Ethics Committee at the IRCCS Istituto Nazionale dei Tumori, Milano.
**Funding for this study:** No funding was received for this study.
**Author Disclosures:**
Gianmarco Della Pepa: Nothing to disclose
Elisa D'Ascoli: Nothing to disclose
Alice Bonanomi: Nothing to disclose
Claudia De Berardinis: Nothing to disclose
Daniela Ballerini: Nothing to disclose
Gianfranco Paride Scaperrotta: Nothing to disclose
Catherine Depretto: Nothing to disclose
Caterina Cazzella: Nothing to disclose
Giovanni Irmici: Nothing to disclose

### RPS 602-4

**Unenhanced MRI for assessment of response to neoadjuvant therapy in patient with locally advanced breast cancer: diagnostic value of DWI/ADC**

*M. Pasculli*, F. Galati, V. Rizzo, G. Moffa, R. Maroncelli, F. Cicciarelli, F. Pediconi, C. Catalano; Rome/IT
*(marcella1.pasculli@gmail.com)*

**Purpose:** The aim of this study was to investigate the predictive value of DWI/ADC (DW-MRI) for the assessment of response to neoadjuvant therapy (NT) in patients with locally advanced breast cancer (LABC).
**Methods or Backround:** Patients with LABC candidate to NT, and who underwent pre-treatment breast MRI between March 2021 to March 2022, were retrospectively enrolled. MRI-based staging and DWI/ADC values (x10-3mm2/s) were analysed. According to post-surgical outcomes, patients were classified as complete responders (pCR) and non-complete responders (non-pCR). Pre-treatment ADC values were compared to the tumour's pathological outcome and post-treatment downstaging. The diagnostic accuracy of DWI-ADC in differentiating between pCR and non-pCR groups was calculated with receiver operating characteristic (ROC) analysis.
**Results or Findings:** 36 patients were evaluated (pCR, n=20; non-pCR, n=16). Pre-treatment lesion ADC values were significantly different between the two groups (p=0.034), while no association was found between pre-NT tumour size and pathological response. ADC values pre-teatment showed significant correlations with loco-regional downstaging after therapy (r=-0.537, p=0.022) and with tumour volume reduction (r=-0.480, p=0.044). ADC values could differentiate pCR from non-pCR patients, with a sensitivity of 75% and specificity of 70%.
**Conclusion:** ADC values on pre-treatment MRI were strongly associated with the outcome in patients with LABC, both in terms of pathological response and loco-regional downstaging after NT, suggesting the use of Unenhanced DW-MRI as a potential predictive tool of response to therapy.
**Limitations:** This was a single-centre study with a limited number of patients.
**Ethics committee approval:** Informed consent was waived due to the retrospective nature of this study, as approved by our local Ethical Committee.
**Funding for this study:** No funding was received.
**Author Disclosures:**
Roberto Maroncelli: Nothing to disclose
Giuliana Moffa: Nothing to disclose
Veronica Rizzo: Nothing to disclose
Francesca Galati: Nothing to disclose
Marcella Pasculli: Nothing to disclose
Federica Cicciarelli: Nothing to disclose
Federica Pediconi: Nothing to disclose
Carlo Catalano: Nothing to disclose

### RPS 602-5

**Performance of the node-RADS scoring system for standardised magnetic resonance imaging assessment of lymph nodes in breast cancer**

*R. Maroncelli*, F. Pediconi, M. Pasculli, A. Marra, F. Cicciarelli, V. Rizzo, G. Moffa, F. Galati, C. Catalano; Rome/IT

**Purpose:** The Node-RADS score was recently introduced to offer a standardised and comprehensive evaluation of lymph node invasion (LNI) based on a five-item Likert scale. We tested Node-RADS score diagnostic performance and assessed the applicability and feasibility of the score among readers.
**Methods or Backround:** A retrospective study was conducted on BC patients who underwent lymph node dissection between January 2020 and January 2023. All patients underwent breast contrast-enhanced magnetic resonance imaging. Sensitivity, specificity, positive predictive value (PPV), and negative predictive value (NPV) were calculated for different Node-RADS cut-off values (>1, >2, >3, >4) in predicting LNI. Pathologic results were considered the gold standard. Additionally, the overall diagnostic performance was evaluated using ROC curves and the Area Under the Curve (AUC). Finally, Cohen's K analysis was used to assess interreader agreement.
**Results or Findings:** The final population includes 192 patients. By increasing the Node-RADS cut-off values, specificity and PPV rose from 71.4% to 100% and 76.7% to 100%, respectively for Reader 1 and 69.4% to 100% and 74.6% to 100% for Reader 2. Node-RADS >2 could be considered the best cut-off value due to its balanced performance. Node-RADS exhibited an AUC of 0.97 for Reader 1 and 0.93 for Reader 2. Node-RADS assigned on CE-MRI images independently predict LNI after adjusting for other variables in a multivariable regression analysis (p<0.001 for both Readers). An excellent interreader agreement was found (K=0.834).
**Conclusion:** The current study establishes the groundwork for implementing Node-RADS as a method for assessing regional lymph nodes in BC patients. The Node-RADS score has demonstrated moderate-to-high overall accuracy in identifying LNI, providing the flexibility to establish different cut-off values based on specific clinical scenarios.
**Limitations:** This study was based on a relatively limited cohort.
**Ethics committee approval:** Informed consent was waived due to the retrospective nature of our study, and approved by the local Ethical Committee.
**Funding for this study:** No funding was received for this study.
**Author Disclosures:**
Roberto Maroncelli: Nothing to disclose
Giuliana Moffa: Nothing to disclose
Veronica Rizzo: Nothing to disclose
Francesca Galati: Nothing to disclose
Marcella Pasculli: Nothing to disclose
Federica Cicciarelli: Nothing to disclose
Federica Pediconi: Nothing to disclose
Andrea Marra: Nothing to disclose
Carlo Catalano: Nothing to disclose

### RPS 602-6

**Incorporating breast cancer molecular subtype rather than axillary disease extent on baseline 18F-FDG PET/CT in axillary treatment strategies**

F. v. Amstel[1], C. De Mooij[1], J. Simons[2], C. Mitea[1], C. Van Der Pol[3], E. Luiten[4], L. Koppert[2], M. Smidt[1], *T. van Nijnatten*[1]; [1]Maastricht/NL, [2]Rotterdam/NL, [3]Leiderdorp/NL, [4]Al Ain/AE
*(thiemovn@gmail.com)*

**Purpose:** In clinically node-positive (cN+) patients treated with neoadjuvant systemic therapy (NST), axillary disease extent on baseline 18F-FDG PET/CT combined with pathologic axillary response to NST has been proposed to guide axillary treatment de-escalation. This study aimed to assess how breast cancer molecular subtype would affect such a treatment strategy.
**Methods or Backround:** Patients with cN+ breast cancer treated with NST in the RISAS trial (NCT02800317) who underwent 18F-FDG PET/CT at baseline were included (period 2017-2019). Baseline 18F-FDG PET/CT exams were centrally reviewed to differentiate between limited (1-3) and advanced axillary disease (≥4 hypermetabolic axillary lymph nodes). After NST, all patients underwent the RISAS-procedure followed by completion axillary lymph node dissection. Axillary pathologic complete response (pCR) rates were stratified by axillary disease extent on baseline 18F-FDG PET/CT, and subsequently by hormone receptor (HR)+/HER2-, HR+/HER2+, HR-/HER2+ and triple negative (TN) molecular subtypes.
**Results or Findings:** A total of 185 patients were included, 62.7% with limited and 37.3% with advanced baseline axillary disease. Overall axillary pCR rate was 29.7% (7% for HR+/HER2-, 52.6% for HR+/HER2+, 75% for HR-/HER2+, and 34.1% for TN; p<0.001). Axillary pCR rates did not significantly differ between limited versus advanced baseline axillary disease within the molecular subtypes. Breast molecular subtype showed to be a significant predictor of axillary response.
**Conclusion:** Axillary pCR rates between limited and advanced axillary disease on baseline 18F-FDG PET/CT were not significantly different within each of the breast molecular subtypes. Breast molecular subtype is important here since it showed to be a significant predictor of axillary response. Therefore, baseline axillary disease extent should be given a less important status while breast molecular subtype should be considered important to guide axillary treatment strategies in cN+ patients treated with NST.
**Limitations:** No limitations were identified
**Ethics committee approval:** Due to the retrospective design of the study, the necessity to obtain written informed consent was waived by the local medical ethics committee.
**Funding for this study:** Funding was received from the University Fund Limburg (SWOL; project 20.048) and Dutch Cancer Society (KWF – REFINE-trial; project 14055).
**Author Disclosures:**
Marjolein Smidt: Nothing to disclose
Thiemo van Nijnatten: Nothing to disclose
Cornelis De Mooij: Nothing to disclose
Cristina Mitea: Nothing to disclose
Florien van Amstel: Nothing to disclose
Janine Simons: Nothing to disclose
Linetta Koppert: Nothing to disclose
Ernest Luiten: Nothing to disclose
Carmen Van Der Pol: Nothing to disclose

### RPS 602-7

**MRI characteristics predicting recurrence/metastases in breast cancer patients receiving neoadjuvant chemotherapy**

*A. Syed*, J. Bansal, M. Wallace; Cardiff/UK
*(dr_aishasyed@yahoo.co.uk)*

**Purpose:** The aim of this study was to evaluate MRI and tumour characteristics predicting recurrence or metastases in breast cancer patients post neoadjuvant chemotherapy ( NACT).
**Methods or Backround:** A retrospective evaluation of all breast MRIs for NACT monitoring between 2009 and 2018 was performed. All patients were followed up for at least five years. Factors including patients' age, tumour size, receptor status, number of lymph nodes, and MRI characteristics were evaluated. SPSS was used for statistical evaluation, and p<0.05 was considered a significant result. Binomial logistic regression was used to evaluate factors, controlling for other variables. The median age of patients was 45 years (range 25 to 73).
**Results or Findings:** Out of 135 patients, 114 had adequate data for evaluation in this study. Thirty-three (28.9%) patients showed local recurrence or metastases. The median time to an event from the date of diagnosis was 35 months (range 0-144 months). Compared to a non-mass enhancement, a mass-like enhancement was statistically associated with fewer events (p=0.011). The factors most significantly associated with an event were triple negative (TN) status, a higher number of lymph nodes on baseline MRI, and post-surgery (ypN). No significant association was found between T stage, tumour grade or MR response pattern (concentric versus crumbling).
**Conclusion:** Of all breast cancer patients receiving NACT, 28.9% showed an adverse event at a median of 35 months. Factors predicting an event in breast cancer patients receiving NACT were TN receptor status, non-mass enhancement on MRI, and higher lymph node status.
**Limitations:** This was a small retrospective study.
**Ethics committee approval:** This was a retrospective study of imaging, therefore no ethics approval was required.
**Funding for this study:** No funding was received for this study.
**Author Disclosures:**
Jyoti Bansal: Nothing to disclose
Aisha Syed: Nothing to disclose
Megan Wallace: Nothing to disclose

### RPS 602-8

**Baseline MRI BI-RADS and breast oedema score features as predictors for axillary lymph node response to neoadjuvant chemotherapy in breast cancer**

*C. Malhaire*[1], O. Umay[1], F. Frouin[2]; [1]Paris/FR, [2]Orsay/FR
*(caroline.malhaire@curie.fr)*

**Purpose:** The aim of this study was to assess the association between pre-treatment breast MRI features and axillary lymph node residual disease in women treated for node-positive breast cancer by neoadjuvant chemotherapy (NAC).
**Methods or Backround:** In this single-centre, retrospective study, women with node-positive breast cancer who underwent NAC and pre-treatment breast MRI between 2016 and 2021 were included. MRIs were evaluated using the standardised BI-RADS and T2-weighted Breast Oedema Score. Univariate analysis and multivariate logistic regression analysis were conducted to evaluate clinicopathological and MRI variables association with lymph node residual disease. A prediction model was developed from the logistic regression analysis and evaluated on a randomly split train and test set (7:3 ratio).
**Results or Findings:** Of 142 breast cancers, 59% achieved post-NAC nodal response, varying by subtype: luminal 24%, HER2+ 69%, and TN 75%. Factors associated with nodal response were TN and HER2+ subtypes, high Ki67, and tumour-infiltrating lymphocytes. Univariate analysis identified MRI features like anterior third location, indicating the depth of the tumour within the breast, and irregular shape as significant for residual axillary disease. In multivariate analysis, the anterior location and the absence of intratumoural T2 hyperintensity remained significant. Adding MRI features to anatomopathological variables enhanced nodal residual disease prediction models.
**Conclusion:** Luminal subtypes, low Ki67 levels, anterior third location, and lack of intratumoural T2 hyperintensity are independently associated with axillary residual disease and provide additional predictive value to predict lymph node residual disease after NAC.
**Limitations:** Study limitations include a single-centre design, a retrospective nature, and a limited sample size for histomolecular subtype subgroup analysis.
**Ethics committee approval:** This study was approved by the relevant Institutional Reveiw Board.
**Funding for this study:** No funding was received.
**Author Disclosures:**
Frédérique Frouin: Nothing to disclose
Caroline Malhaire: Nothing to disclose
Ozgun Umay: Nothing to disclose

16:30-17:30 **Research Stage 2**

### Research Presentation Session: Interventional Radiology

### RPS 609

### New insights in interventional radiology above the neck

**Moderator**
F. Wagner; Berne/CH
*(franca.wagner@insel.ch)*

### RPS 609-2

**Predictors of parenchymal haematoma after mechanical thrombectomy in patients with large ischeamic core due to a large vessel occlusion in the anterior circulation**

*D. De Leoni*, A. M. Alexandre, A. Broccolini, A. Pedicelli; Rome/IT
*(deleonidavide3@gmail.com)*

**Purpose:** This study aimed to evaluate the predictors of any type of parenchymal haematoma according to ECASS II classification and the predictors of type II parenchymal haematoma after MT in patients with an ASPECTS score 5 due to a LVO in the anterior circulation.
**Methods or Backround:** In this retrospective observational study, databases of 16 comprehensive stroke centres were screened for consecutive patients with AIS due to LVO diagnosed between January 2016 and December 2021. The ASPECT score was calculated from two standard axial CT slices. Brain bleeding was confirmed with CT or MRI scan (24 and 72h after MT). Heidelberg classification was used to classify haematoma. Presence of PH type 2 was selected as the primary outcome measure, due to its relevance in the overall clinical outcome of patients.
**Results or Findings:** 408 patients with LVO in the anterior circulation and an ASPECT score ≤5 that received MT were selected. Higher number of passages in the second technique (OR 4.176, 95% CI 1.444 – 12.003, p=0.008) was predictive of PH type 2, whereas procedure conducted under general anesthesia was associated with a lower risk of PH type 2 (OR 0.084, 95% CI 0.011 – 0.612, p=0.015). Higher age (OR 1.073, 95% CI 1.023 – 1.126, p=0.004) and number of passages during the second technique (OR 2.119, 95% CI 1.106 – 4.374, p=0.025) were an independent predictor of any PH.
**Conclusion:** Our study showed that an increased number of passages during MT in patients with large baseline brain infarct is a predictor of post-procedural PH of any type. The choice of general anesthesia for MT should be considered as this is associated with a lower risk of occurrence of PH type 2.
**Limitations:** This was a retrospective study.
**Ethics committee approval:** No information provided by the submitter.
**Funding for this study:** No funding was received for this study.
**Author Disclosures:**
Andrea Maria Alexandre: Nothing to disclose
Davide De Leoni: Nothing to disclose
Aldobrando Broccolini: Nothing to disclose
Alessandro Pedicelli: Nothing to disclose

### RPS 609-3

**Emergency carotid artery stenting in endovascular stroke treatment: a feasible and relatively safe procedure**

*R. Kaufmann*, L. C. Van Dijk, W. Stomp, K. De Laat, S. F. De Bruijn, J. Piet, S. C. Klink, H. van Overhagen; The Hague/NL
*(ruth.kaufmann@gmail.com)*

**Purpose:** Emergency carotid artery stenting (eCAS) during endovascular stroke treatment (EVT) is considered controversial in the literature. We performed a retrospective analysis in our institute to assess safety and efficacy.
**Methods or Backround:** During 2015-2023, 533 patients underwent EVT. Seventy patients underwent 71 eCAS procedures concurrently. Patient files were analysed on baseline characteristics, EVT procedure, and complication rates.
**Results or Findings:** There were 46 men and 24 women with a mean age of 73 years (range 53-99 years). Preprocedural NIHSS scores ranged from 3 to 25 (median 14). Indications were significant carotid artery stenosis (n=36), occlusion (n=28), and dissection (n=7; 3 of 7 occurred during EVT). Carotid revascularisation and stenting were performed prior to intracranial revascularisation in the vast majority of patients. Stenting was performed in the left carotid artery (n=39) and in the right carotid artery (n=32), using 53 Carotid Wallstents, 15 Cordis Precise stents, and 3 X-pert stents. Additional endovascular treatment was performed in 55 patients; carotid T (n=4), M1 (n=28), M1 and M2 (n=5), M2 (n=16), M3 (n=2), and A2 (n=1). All patients received a single intra-arterial dose of 5000 to 10000 IU heparin during the procedure. mTICI scores immediately postprocedural were either 2b (n=22) or 3 (n=49). Complications occurred in 10 out of 71 patients (14%): intracranial bleeding (n=5 (7%)), access bleeding (n=4 (6%)), and vessel occlusion (n=1) (1%)).
**Conclusion:** eCAS seems feasible and relatively safe with low intracranial bleeding rates in patients undergoing EVT. A single intra-arterial bolus of heparin does not seem to increase the occurrence of intracranial bleeding.
**Limitations:** Our study is a retrospective, single-centre cohort study.
**Ethics committee approval:** Since it is a retrospective study, we received a waiver from our ethical committee.
**Funding for this study:** No funding was received for this study.
**Author Disclosures:**
Karlijn De Laat: Nothing to disclose
S.J. Camiel Klink: Nothing to disclose
Hans van Overhagen: Nothing to disclose
Lukas C. Van Dijk: Nothing to disclose
Sebastiaan F.T.M. De Bruijn: Nothing to disclose
Ruth Kaufmann: Nothing to disclose
Wouter Stomp: Nothing to disclose
Jurgen Piet: Nothing to disclose

### RPS 609-4

**Endovascular treatment of basilar artery occlusion: experience in a tertiary hospital**

*F. E. Dianderas Gutiérrez*, A. Alboniga-Chindurza Barroeta, E. Zapata Arriaza, M. Aguilar Perez, R. F. Ocete Pérez, A. Zamora Piñero, A. González García; Sevilla/ES
*(stebandg93@hotmail.com)*

**Purpose:** This study is a description and analysis of the experience in endovascular treatment of basilar artery occlusion (BAO) in a tertiary hospital with a reference population of 2.5 million inhabitants, one of the largest in Europe.
**Methods or Backround:** Patients with BAO in the period 2017-2021 registered in the Andalusian thrombectomy database (ARTISTA) of the Huelva-Seville node were included. A statistical analysis of the demographic variables, recanalisation rate, and good functional prognosis (modified Ranking scale (mRs≤2y≤3) as well as complications of the procedure was performed.
**Results or Findings:** Of 148 patients (7.8% of the ARTISTA database), median 70.6 years (63-80), pre-procedure NIHSS of 14 (6-24.7), 31.1% received fibrinolysis, 21.6% were awakening strokes, and 12.8% were in-hospital. The symptom onset-recanalisation time was 358 minutes (256-631), with a median recanalisation puncture of 40 minutes (17.7-89). In 62.2% of cases there was BA occlusion; in 23.9% and 9.9% of cases this also involved the vertebral and posterior cerebral arteries, respectively. In 61.1% of the cases, the endovascular procedure was performed with local anesthesia (general: 34%, sedation: 4.9%). Distal aspiration was performed in 82%, stent retriever in 6%, Solumbra in 5% and ATP and /or stent in 7%. The recanalisation rate was TICI ≥2b in 85,7% of the cases. The MR at 90 days was 0-2 in 37.9% and 0-3 in 46.4%. 36.4% of the patients were Rank 6. In 9.3% of cases there were some complications of the procedure (dissection in 2.2%). Symptomatic intracranial haemorrhage occurred in 5% of cases (PH1: 2.1%, PH2: 2.9%). 36.4% of cases required admission to the ICU; the rest of the patients went to the Stroke Unit.
**Conclusion:** Our results in the treatment of patients with OAB are similar to those described in the ATTENTION and BAOCHE recent studies.
**Limitations:** No limitations were identified.
**Ethics committee approval:** This was a retrospective and educational study. All data was anonymised.
**Funding for this study:** No funding was received for this study.
**Author Disclosures:**
Elena Zapata Arriaza: Nothing to disclose
Aynara Zamora Piñero: Nothing to disclose
Alejandro González García: Nothing to disclose
Francisco Esteban Dianderas Gutiérrez: Nothing to disclose
Asier Alboniga-Chindurza Barroeta: Nothing to disclose
Rafael Felix Ocete Pérez: Nothing to disclose
Marta Aguilar Perez: Nothing to disclose

### RPS 609-5

**Chronic subdural haematoma: is endovascular treatment a safe and effective alternative?**

E. Fandiño Benito, *I. Bermudez-Coronel*, J. C. Mendez; Madrid/ES
*(isbermudezcoronel@gmail.com)*

**Purpose:** The purpose of this study was to determine the effectiveness and safety of middle meningeal artery embolisation in chronic subdural haematoma.

**Methods or Backround:** A total of 34 patients underwent middle meningeal artery embolisation, with 3 patients treated in 2021, 12 in 2022, and 19 in 2023. Out of these, 11 of the 34 patients (32.35%) underwent bilateral embolisation. Prior to embolisation, 18 patients (52.94%) had previously undergone surgical drainage of the haematoma, while embolisation served as the primary treatment for 12 patients (35.29%). In all cases, embolisation was carried out using embolic liquid agents.
**Results or Findings:** Patient follow-up was carried out and measurement and evolution of the haematoma was recorded over time. On average, the thickness of the haematoma decreased by 70% compared to the pre-treatment CT scans. During the follow up period, two patients died due to other underlying medical conditions. Additionally, one patient experienced sixth cranial nerve palsy, which spontaneously resolved within 48 hours following the procedure. Notably, while general anesthesia was chosen for only 56.6% of the total cases, it is increasingly becoming the preferred option over sedation anesthesia. Encouragingly, throughout the two-year follow-up, no patient required surgical or endovascular reintervention.
**Conclusion:** Middle meningeal artery embolisation appears to be an effective and safe treatment for chronic subdural haematoma, both, as a the primary option and as an adjuvant treatment to surgery, ultimately reducing the need for reinterventions.
**Limitations:** The primary limitation of this study lies in its observational nature, conducted at a single centre. To establish more robust conclusions, a multicentre randomised controlled trial should be carried out.
**Ethics committee approval:** No information provided by the submitter.
**Funding for this study:** No funding was received for this study.
**Author Disclosures:**
Jose C. Mendez: Nothing to disclose
Eduardo Fandiño Benito: Nothing to disclose
Isabel Bermudez-Coronel: Nothing to disclose

### RPS 609-6

**Endovascular management of post-irradiated carotid blowout syndrome in patients with lower neck cancers**

*F-C. Chang*; Taipei/TW
*(fcchang374@gmail.com)*

**Purpose:** Lower neck cancers (LNCs) include specific tumour types and have vascular collaterals from other head-and-neck cancers (HNCs). This prospective study evaluated the outcome of endovascular management of post-irradiated carotid blowout syndrome (PIRCBS) in LNC patients by comparing reconstructive management (RE) and deconstructive management (DE).
**Methods or Backround:** Between 2015 and 2021, LNC patients complicated with PIRCBS who underwent endovascular therapy were prospectively included. The patients underwent DE by permanent embolisation of both internal carotid artery (ICA) and external carotid artery (ECA) with coils (Nester, Cook; Concerto nylon detachable coils, ev3 Neurovascular) and/or adhesive agent (histoacryl, Braun Surgical). ICA was occluded if pathological lesion was located at ICA only. Cross embolisation including proximal and distal to the pathological lesion were done in all patients. Technical outcomes were evaluated by immediate haemostasis and neurological complications. The haemostatic outcome was evaluated by the haemostatic result and haemostatic period.
**Results or Findings:** 59 patients (mean age: 58.45±10.25, 56 men) were enrolled, including 28 RE patients and 31 DE patients. The results of RE versus DE were as follows: rebleeding events: 13/28 (46.4%) versus 10/31 (32.3%), p=0.265; haemostatic periods (month): 9.4±14.0 versus 14.2±27.8, p=0.589; neurological complications: 4/28 (14.3%) versus 5/31 (16.1%), p=0.844; survival time (months): 11.8±14.6 versus 15.1±27.5, p=0.605.
**Conclusion:** We observed that there was no difference of rebleeding risk and neurological complication between DE and RE groups. RE could be used as a potential routine treatment for PIRCBS in patients with LNC.
**Limitations:** Several limitations were noted in this study, including variable entities and staging of malignancy in the lower neck region. The treatment modalities of our patients with LNC were also heterogeneous, which might influence the outcome of CBS.
**Ethics committee approval:** This study was approved by the Taipei Veterans General Hospital with code: 2022-02-001CU.
**Funding for this study:** This study received funding from Taipei Veterans General Hospital, Taiwan [V110C-037, V111C-028, V112C-059, V112D67-002-MY3-1 (to FCC)], National Science and Technology Council, Taiwan [109-2314-B-075-036, 110-2314-B-075-032 and 112-2314-B-075-066 (to FCC)].
**Author Disclosures:**
Feng-Chi Chang: Nothing to disclose

### RPS 609-7

**Efficacy and safety of use of flow diverting silk stents in endovascular treatment of intracranial aneurysms**

E. Sahibli, *E. Kara*, E. H. Karlı, M. Barburoğlu; Istanbul/TR
*(krezg@hotmail.com)*

**Purpose:** The purpose of this study was to determine the feasibility, efficacy, and safety of flow converting silk stents in treating intracranial aneurysms.
**Methods or Backround:** The study included 75 patients (mean age 49.54 years), with at least six months of angiographic and long-term follow-up imaging, with 95 aneurysms treated in 78 different procedures. The patients' pre-treatment and follow-up clinical data, procedure results, demographic characteristics, aneurysm characteristics and localisations, occlusion rates, early and late mortality, and permanent morbidity rates were analysed retrospectively.
**Results or Findings:** According to the morphology of the aneurysms, 89 (93.7%) were saccular, 2 were fusiform (2.1%), 2 (2.1%) were blister, and 2 (2.1%) were dissected; according to size, 31 were (32.63%) small, 40 were (42.11%) medium, 11 were (11.58%) large, and 13 were (13.58%) giant; according to localisation 87 were (91.58%) anterior circulation (AC) and 8 were (8.42%) posterior circulation (PC); according to wall placement 91 (95.79%) were lateral and 4 (4.21%) were bifurcated; according to aspect ratio, 40 (42.11%) had a narrow neck and 52 (54.73%) had a wide neck. Two patients were treated for ruptured blister aneurysms. Aneurysm occlusion rates were found to be 87.2% at 12 months, and 93.68% at long-term follow-up. A total of 9 patients (11.53%) developed procedural complications. In angiographic long-term follow-ups, postprocedural late complications developed in 7 patients (8.9%). The overall mortality rate was 5.33%, with postprocedural early 2.66% and late 2.66%. The permanent morbidity rate was 1.33%. The mean follow-up period was 42.68±26.5 months, and the mean occlusion time was 8.76±8.3 months.
**Conclusion:** It was concluded that the use of flow-diverting silk stent was effective and safe in the endovascular treatment of intracranial aneurysms. Technical success was worse in PC aneurysms compared to anterior, mortality and morbidity rates were lower in anterior circulation.
**Limitations:** The study was retrospective.
**Ethics committee approval:** The study was approved by the Ethics Committee of the Medical School of Istanbul University Istanbul on the 24th September 2021 with code number 17.
**Funding for this study:** No funding was received.
**Author Disclosures:**
Mehmet Barburoğlu: Nothing to disclose
Elif Hazal Karlı: Nothing to disclose
Eshgin Sahibli: Nothing to disclose
Ezgi Kara: Nothing to disclose

### RPS 609-8

**Contour device implantation for treatment of intracranial aneurysms in the basilar tip**

*K. Mostafa*, F. Bueno Neves, F. Gärtner, T. Klintz, N. Larsen, S. Peters, J. Hensler, O. Jansen, F. Wodarg; Kiel/DE
*(mostafa.karim86@gmail.com)*

**Purpose:** The purpose of this study was to assess and evaluate the feasibility and the periinterventional aspects of embolisation of intracranial aneurysms located in the basilar tip using the Contour Neurovascular System (CNS).
**Methods or Backround:** Treatment of basilar apex aneurysms remains challenging regarding the nobility of the parent vessel and their often wide-necked configuration. Novel intrasaccular flow-disruption devices constitute an endovascular treatment alternative besides coiling and stent-assisted procedures. The presented study reports periinterventional and feasibility aspects of embolising basilar tip aneurysms with the CNS. Here, a retrospective analysis of eight patients after CNS implantation into a basilar apex aneurysm was performed. Data on intervention times, radiation dose, procedural success, and complications were gathered. All patients received follow-up digital subtraction angiography after six months.
**Results or Findings:** CNS implantation was successful in all patients. Mean device instrumentation time was 18.8±7.7 minutes with a mean full intervention time of 100±65.8 minutes. Mean full procedure radiation dose was 1917 (421-5107) cGy/cm2. Upon interventional angiographic follow-up after six months, six aneurysms were adequately occluded. One showed constant slight perfusion inside the device without the need for reintervention and one patient had undergone reintervention. No deaths were reported.
**Conclusion:** CNS implantation is an alternative endovascular method for embolising intracranial aneurysms located in the basilar tip with short intervention times and promising short- and medium-term follow-up data concerning aneurysm occlusion and reinterventions.
**Limitations:** The number of patients included in this study was small due to the single-centre design as well as the novelty of the CNS combined with the rarity of the specific aneurysm. Hence it can not be concluded that basilar apex aneurysm treatment using the CNS is equal to conventional procedures. Additionally, further research is needed to evaluate long-term stability.

**Ethics committee approval:** This study was approved by the Ethikkomission der Christian-Albrechts-Universität Kiel.
**Funding for this study:** No funding was received.
**Author Disclosures:**
Tristan Klintz: Nothing to disclose
Sönke Peters: Nothing to disclose
Fritz Wodarg: Nothing to disclose
Johannes Hensler: Nothing to disclose
Friederike Gärtner: Nothing to disclose
Karim Mostafa: Nothing to disclose
Fernando Bueno Neves: Nothing to disclose
Olav Jansen: Nothing to disclose
Naomi Larsen: Nothing to disclose

**16:30-17:30** **Research Stage 3**

### Research Presentation Session: Neuro

### RPS 611

### Brain tumour imaging: advanced techniques

**Moderator**
J. Boban; Novi Sad/RS
*(jasmina.konstantinovic@gmail.com)*

### RPS 611-2

**Amide proton transfer-weighted imaging (APT-wi) for molecular and survival prediction of diffuse adult gliomas: single-centre retrospective study**
*F. M. Doniselli*, M. Moscatelli, M. Verri, R. Pascuzzo, M. Grisoli; Milan/IT

**Purpose:** The aim of this study was to observe the correlation between APT signal characteristics of low- and high-grade glial neoplasms and the demographic characteristics, mutational status and grading, using first-order statistics.
**Methods or Backround:** We retrospectively included patients treated for brain tumor in our centre between October 2020 and October 2022 if they matched our set criteria, which were the presence of analysable pre-operative APT-weighted imaging (APT-wi), or a diagnosis of a glioma of any grade. Volumetric ROI on the whole lesion ("tum_ROI"), on the whole lesion excluding the necrotic component ("les_ROI") and on necrotic component alone - if present ("nec_ROI") - were placed; from each ROI, the mean, median, standard deviation, 10th percentile, 90th percentile, skewness, and kurtosis of APT values were extracted. One-year survival, IDH, grade, and MGMT promoter methylation were recorded.
**Results or Findings:** 52 patients (median age 56 years, IQR 25-76, 35 males; 5 astrocytoma grade 2, 8 grade 3 astrocytoma and 39 glioblastoma) were included. Median tumour volume was 40.7cm3 (0.38-167.6). Mean APT values in the les_ROI ranged approximately between 1 and 3 while mean APT values in tum_ROI ranged between 1 and 7. Mean and 90th percentile APT values in les_ROI was statistically different between grade 4 and LGG (p=0.042 and p=0.011 respectively) and for 1 year survival (p=0.011 and p=0.012). No statistical significance was found between IDH-wild type and mutated gliomas nor between methylated and unmethylated MGMT promoter tumours.
**Conclusion:** In our cohort of glioma patients, APT-wi provides useful information to discriminate between tumour gradings as well as one-year survival.
**Limitations:** The study was limited by the limited cohort of patients, which occurred as it was a single-centre study.
**Ethics committee approval:** This was a retrospective study.
**Funding for this study:** No funding was received.
**Author Disclosures:**
Marina Grisoli: Nothing to disclose
Fabio Martino Doniselli: Nothing to disclose
Marco Moscatelli: Nothing to disclose
Riccardo Pascuzzo: Nothing to disclose
Mattia Verri: Nothing to disclose

### RPS 611-3

**Decoding malignant glioma heterogeneity by PET-MRI habitat analysis of HYpoxia, PERfusion and DIffusion imaging: preliminary results of the HYPERDIrect study**
G. Nocera, N. Pecco, M. Bailo, M. Callea, P. V. Scifo, P. Della Rosa, F. Gagliardi, A. Falini, *A. Castellano*; Milan/IT
*(castellano.antonella@hsr.it)*

**Purpose:** Malignant gliomas are characterised by considerable intratumour heterogeneity, directly related to treatment failure. A novel method for cancer detection involves identifying regions or habitats within tumours by assessing shared imaging characteristics. This approach utilises quantitative analysis of conventional and advanced imaging data through mathematical models, which effectively partition the tumour into voxel-based subregions exhibiting similar radiological features. The integration of multiple images further refines the creation of distinct tumour habitats. In this study, habitat analysis has been applied on hybrid PET/MR images to map hypoxia, neoangiogenesis, cellularity, and tumour metabolism.
**Methods or Backround:** Twenty patients with suspected malignant glioma candidate for surgical resection or biopsy underwent preoperative hybrid 3T PET/MRI acquisitions for assessing HYpoxia (using quantitative blood oxygenation level-dependent – q-BOLD imaging), PErfusion (using Dynamic Contrast-Enhanced DCE-MRI), DIffusion (usng DTI), and methionine-PET for tumour metabolism. Data obtained were processed to generate HYPERDIrect habitat maps. In FLAIR-derived tumour volumes, an automatic clustering algorithm classified voxels of each quantitative map into two clusters (high and low intensity) . The combination of the clusters from all maps identified eight distinct habitats. Maps were imported into the neuronavigational system to perform imaging-guided sampling for histopathological correlation.
**Results or Findings:** Preliminary findings demonstrated high habitat imaging reproducibility and a reliable correlation between the expected microenvironment of the different habitats and the actual histopathological characteristics: samples from more aggressive habitats (with reduced diffusivity, high perfusion, and low hypoxia) histologically coincide with regions displaying elevated cell density and increased microvascular proliferation. Samples from less aggressive habitats (high diffusivity, low perfusion, and low hypoxia) correspond to glioma infiltrative areas.
**Conclusion:** Habitat imaging using the HYPERDIrect map approach might serve as a potential biomarker for non-invasively characterizing tumour heterogeneity in vivo.
**Limitations:** No limitations were identified.
**Ethics committee approval:** This study was approved by the Ethics Committee of Ospedale San Raffaele on March 9th, 2022 (code 19/INT/2022).
**Funding for this study:** Funding was received from the Italian Ministry of Health, grant number GR-2018-12365670.
**Author Disclosures:**
Andrea Falini: Nothing to disclose
Nicolò Pecco: Nothing to disclose
Michele Bailo: Nothing to disclose
Marcella Callea: Nothing to disclose
Gianluca Nocera: Nothing to disclose
Pasquale Della Rosa: Nothing to disclose
Antonella Castellano: Nothing to disclose
Paola Vittoria Scifo: Nothing to disclose
Filippo Gagliardi: Nothing to disclose

### RPS 611-4

**Vasari-based features nomogram to predict the tumour-infiltrating CD8+ T cell levels in glioblastoma**
*C. Xue*, J. Zhou; Lanzhou/CN
*(1102599617@qq.com)*

**Purpose:** Tumour-infiltrating CD8+ T cells play a key role in glioblastoma development, malignant progression, and recurrence. This study sought to establish nomograms based on the Visually AcceSAble Rembrandt Images (VASARI) features of multiparametric magnetic resonance imaging (MRI), to determine the expression levels of tumour-infiltrating CD8+ T cells in patients with glioblastoma.
**Methods or Backround:** Pathological and imaging data of 140 patients with glioblastoma confirmed by surgery and pathology were retrospectively analysed. The levels of tumour-infiltrating CD8+ T cells in tumour tissue samples obtained from patients were quantified using immunohistochemical staining. Patients were divided into high and low CD8 expression groups. The MRI images of patients with glioblastoma were analysed by two radiologists using the VASARI scoring system.

**Results or Findings:** A total of 25 MRI-based VASARI imaging features were evaluated by two neuroradiologists. The features with the greatest predictive power for CD8 expression levels were cystic (OR, 3.063; 95% CI: 1.387, 6.766; P=0.006), haemorrhage (OR, 2.980; 95% CI: 1.172, 7.575; P=0.022), and ependymal extension (OR, 0.257; 95% CI: 0.114 0.581; P=0.001). A logistic regression model based on these three features showed better sample predictive performance (AUC=0.745; 95% CI: 0.665, 0.825; Sensitivity=0.527; Specificity=0.857).
**Conclusion:** The VASARI feature-based nomogram model shows promise in predicting the level of infiltrative CD8 expression in GB tumours noninvasively for earlier tissue diagnosis and more aggressive treatment.
**Limitations:** This study was limited by the lack of data, all from one centre. Further studies were not conducted in combination with multimodal MRI metrics. The predictive performance of the parameters included in this study was not high and may be improved in future studies using artificial intelligence methods.
**Ethics committee approval:** This study was approved by the Lanzhou University Second Hospital (Approval No. 2020A-070).
**Funding for this study:** Funding was received from the National Natural Science Foundation of China (grant number 82071872).
**Author Disclosures:**
Junlin Zhou: Author: The authors of this manuscript declare no relationships with any companies whose products or services may be related to the subject matter of the article.
Caiqiang Xue: Author: The authors of this manuscript declare no relationships with any companies whose products or services may be related to the subject matter of the article.

### RPS 611-5

**Assessment of axonal fibre integrity using DWI MRI models in post-surgery low-grade glioma patients**

A. Van Der Hoorn, H. L. Van der Weide, M. Kramer, J. Kłos, R. Borra, E. De Vries, *C. W. Van der Weijden*; Groningen/NL
*(c.w.j.van.der.weijden@umcg.nl)*

**Purpose:** Diffusion-weighted imaging (DWI) using MRI is commonly used for characterising and monitoring patients with low-grade glioma (LGG). Advanced DWI models can potentially provide more detailed microstructural information than traditional DWI parameters. This study aims to establish the relationship between outcome parameters derived from advanced DWI models, and to assess the parameter reliability of these models in post-surgery LGG-patients.
**Methods or Backround:** The study involved 14 post-surgery LGG-patients and 6 healthy controls (HC) who underwent DWI and T2w-FLAIR-MRI scans. Several DWI modeling approaches (diffusion tensor imaging, DTI; diffusion kurtosis imaging, DKI; white matter tract integrity, WMTI; neurite orientation dispersion and density imaging, NODDI; fixel based analysis, FBA) were employed to extract parameters like fractional anisotropy (FA), intracellular diffusivity (ICD), axonal water fraction (AWF), and fibre density (FD). These parameters were chosen to assess axonal fibre integrity, which should be absent in the surgical cavity, affected in the perisurgical cavity (hyperintensity on T2w-FLAIR-MRI), and unaffected in normal appearing white matter (NAWM) of LGG-patients and WM of HC.
**Results or Findings:** All parameters were significantly different between different tissues, with a gradient of low (DTI-FA, DKI-FA, WMTI-ICD, WMTI-AWF) or no signal (NODDI-ICD, FBA-FD) in the surgical cavity, to medium signal in peri-surgical cavity, and high signal in NAWM/WM. Spearman correlations revealed that all parameters corresponded well with each other (R2=0.69-0.99).
**Conclusion:** The results of WMTI-ICD and WMTI-AWF suggest the presence of intracellular diffusivity in the surgical cavity, probably because the model does not account for cerebrospinal fluid. These WMTI-data and the high agreement between parameters derived from different models suggest that if only diffusivity direction is required, DTI-FA might suffice. Should one would like to measure intracellular water specifically, then NODDI-ICD would be the better alternative.
**Limitations:** No limitations were identified.
**Ethics committee approval:** This study was approved by the Medical Ethics Review Board (METc) of the University Medical Centre, Groningen.
**Funding for this study:** No funding was received for this study.
**Author Disclosures:**
Hiska L. Van der Weide: Nothing to disclose
Justyna Kłos: Nothing to disclose
Ronald Borra: Nothing to disclose
Erik De Vries: Nothing to disclose
Chris W.J. Van der Weijden: Nothing to disclose
Miranda Kramer: Nothing to disclose
Anouk Van Der Hoorn: Nothing to disclose

### RPS 611-6

**Predictive value of cellular metabolism parameters derived from perfusion MRI for the overall survival of patients with glioblastoma**

*C. W. Van der Weijden*, R. Borra, E. De Vries, A. Van Der Hoorn; Groningen/NL
*(c.w.j.van.der.weijden@umcg.nl)*

**Purpose:** Perfusion weighted imaging (PWI) with MRI is used to characterise and monitor patients with glioblastoma during and after treatment. Using advanced models, PWI MRI can provide information about metabolism. Metabolic parameters are altered upon tumour growth, making them potential indicators of early tumour progression. This study aims to determine the predictive value of advanced PWI parameters for the overall survival of glioblastoma patients.
**Methods or Backround:** Dynamic susceptibility contrast (DSC) PWI and T1w post-contrast MRI was acquired in 7 patients with post-surgery GBM. PWI was modelled to obtain the mean transit-time (MTT), relative cerebral blood volume (rCBV), relative cerebral blood flow (rCBF), capillary transit-time heterogeneity (CTH), oxygen extraction fraction (OEF), and cerebral metabolic rate of oxygen (CMRO2) for regions around the surgical cavity. Three perilesion regions were defined on the T1w post-contrast images, respectively at distances of 0-0.5cm, 0.5-1.0cm, and 1.0-1.5cm from the surgical cavity. The outcome parameters were associated with overall survival.
**Results or Findings:** Visual inspection of the data showed a gradient in the parameters from the surgical cavity outward, albeit not statistically significant. The MTT for perilesion of 0-0.5cm, 0.5-1.0cm, and 1.0-1.5cm distance from the surgical cavity, was 4.2±1.2, 3.6±1.3, 3.5±1.3, rCBV was 2.0±0.6, 2.0±0.5, 2.3±0.7, rCBF was 33.9±11.2, 45.0±15.0, 50.7±7.2, CTH was 4.7±1.2, 4.3±1.5, 4.3±1.4, OEF was 0.4±0.1, 0.4±0.1, 0.4±0.1, and CMRO2 was 12.4±2.8, 14.4±2.7, 16.3±3.4, respectively. None of the parameters were significantly correlated with overall survival.
**Conclusion:** Although no significant differences were observed yet, the PWI parameters seemed to normalise with greater distance from the surgical cavity. This was even stronger for the currently hardly used CMRO2, although the sample size will be increased to be able to draw firm conclusions.
**Limitations:** The study was limited by its sample size.
**Ethics committee approval:** This study was approved by the Medical Ethics Review Board (METc) at the University Medical Centre, Groningen.
**Funding for this study:** No funding was received for this study.
**Author Disclosures:**
Ronald Borra: Nothing to disclose
Erik De Vries: Nothing to disclose
Chris W.J. Van der Weijden: Nothing to disclose
Anouk Van Der Hoorn: Nothing to disclose

### RPS 611-7

**Selective vulnerability of cognitive networks in patients with glioma measured by resting-state fMRI**

*L. Pasquini*[1], A. Napolitano[2], M. Jenabi[1], K. Peck[1], M. Schmid[2], A. I. Holodny[1]; [1]New York City, NY/US, [2]Rome/IT
*(lucapasquini3@gmail.com)*

**Purpose:** Gliomas affect the whole brain through structural or functional disconnection. This study investigates the impact of gliomas on the cortical synchronisation of brain networks using resting-state fMRI (rs-fMRI). We hypothesised a different vulnerability of brain networks depending on underlying function, tumour location and grade.
**Methods or Backround:** We recruited 147 glioma patients (89M, 50.95±16.13y, 92 high-grade, 55 low-grade) and 200 healthy controls (HCs) with rs-fMRI. Glioma segmentation was performed using 3D-slicer on FLAIR. Group independent component analysis (ICA) was used to extract spatially independent components (IC), subsequently categorised into their respective networks using NeuroMark fMRI 1.0 atlas. We computed cosine similarity (CS) and ran a permutation test to compare, for each IC, the CS of each patient against the HCs distribution. A Chi-squared test was used to verify the significance of networks alterations, tumour location ,and genetics ($p<0.05$).
**Results or Findings:** Out of the 20 network components in HCs, 10.38±1.43 resulted altered in patients, including cognitive control network (CCN); default mode network (DMN); subcortical network (SCN); sensorimotor network; visual network (VN). CCN showed significant alterations with tumours in the temporal lobe (p=0.005), Broca's (p=0.01), and Wernicke's area (p=0.041). Tumours in Wernicke's area also altered the DMN (p=0.04), SCN (p=0.038), and VN (p=0.003). Network alterations persisted with increased distance from the tumour, and were more pronounced with higher WHO-grade ($p<0.001$).
**Conclusion:** These results indicate specific vulnerability of cognitive networks to tumour growth. Functional alterations extend beyond tumour boundaries, and increase with WHO-grade. Tumour location in known eloquent areas exerts widespread effects on brain networks.
**Limitations:** Limitations of this study include its retrospective design and lack of complete neuropsychological testing.
**Ethics committee approval:** This study was approved by the local Institutional Research Board's ethics committee with code: (16-360).

**Funding for this study:** Funding was received from the National Institutes of Health (NIH): NIH-NIBIB R01 EB022720, NIH-NCI R21 CA220144, NIH-NCI P30 CA008748; MSK Cancer Center Support Grant/Core Grant P30 CA008748.
**Author Disclosures:**
Andrei Igor Holodny: Owner: fMRI Consultants, LLC
Antonio Napolitano: Nothing to disclose
Kyung Peck: Nothing to disclose
Luca Pasquini: Nothing to disclose
Mehrnaz Jenabi: Nothing to disclose
Maurizio Schmid: Nothing to disclose

**16:30-17:30** **Research Stage 4**

### Research Presentation Session: Artificial Intelligence & Machine Learning & Imaging Informatics

### RPS 605

### Impact of AI on diagnosis of prostate cancer

**Moderator**
M.-F. Bellin; Le Kremlin-Bicêtre/FR

### RPS 605-2

**Artificial intelligence and radiologists at prostate cancer detection in MRI: outcomes of the PI-CAI challenge**

*A. Saha*, J. Bosma, J. J. Twilt, D. Yakar, M. Elschot, J. Veltman, J. Fütterer, M. De Rooij, H. Huisman; Nijmegen/NL
*(anindo@ieee.org)*

**Purpose:** Diagnostic performance of AI systems at detecting clinically significant prostate cancer in MRI, in comparison to radiologists using PI-RADS v2.1, has not been studied at scale. Autonomous AI systems can alleviate the increasing demand in medical imaging and reduce overdiagnosis in prostate cancer management.
**Methods or Backround:** We trained, tuned, and tested an independently developed AI system at detecting Gleason grade group ≥2 prostate cancer, using a retrospective cohort of 10,207 MRI examinations (9129 patients) from four European tertiary care centres. In parallel, we facilitated an observer study with 62 radiologists (45 centres, 20 countries) and 400 testing cases. Reference standard was histopathology and ≥3 years of follow-up. Our study design was established and preregistered with 16 international multidisciplinary experts.
**Results or Findings:** In the subset of 400 testing cases that was used to facilitate the observer study, on average, the AI system demonstrated superior diagnostic performance with an AUROC of 0.91 (95% CI, 0.87-0.94), than the pool of 62 radiologists at PI-RADS v2.1 with an AUROC of 0.86 (95% CI, 0.83-0.89). In all 1000 testing cases, the AI system showed marginally lower specificity of 68.9% (95% CI, 65.3-72.4%) than the standard of care during routine practice with a specificity of 69.0% (95% CI, 65.5-72.5%), when thresholded to match the same sensitivity of 96.1% (95% CI, 94.0-98.2%) as the PI-RADS ≥3 operating point.
**Conclusion:** An AI system, trained on thousands of cases, is superior in differentiating significant prostate cancer at MRI in comparison to radiologists at PI-RADS v2.1, but marginally less specific in comparison to the standard of care in routine practice.
**Limitations:** The study utilised a retrospective design and was based on histologic verification guided by routine practice. There was an absence of intercontinental, multi-ethnic patient data and MRI examinations from all major commercial vendors.
**Ethics committee approval:** This study was approved by institutional or regional review boards at each contributing center (identifiers: REK 2017/576; CMO 2016-3045; IRB 2018-597; ZGT23-37), and was conducted in accordance with the principles of the Declaration of Helsinki. Informed consent was exempted, given the retrospective scientific use of deidentified MRI scans and clinical data.
**Funding for this study:** This study received funding from the EU Horizon 2020: ProCAncer-I (grant number 952159), Health~Holland (grant number LSHM20103).
**Author Disclosures:**
Jurgen Fütterer: Nothing to disclose
Joeran Bosma: Nothing to disclose
Henkjan Huisman: Nothing to disclose
Anindo Saha: Nothing to disclose
Matthijs Elschot: Nothing to disclose
Derya Yakar: Nothing to disclose
Jeroen Veltman: Nothing to disclose
Maarten De Rooij: Nothing to disclose
Jasper Jonathan Twilt: Nothing to disclose

### RPS 605-3

**Data integration using AI, PI-RADS, and clinical data to reduce false positives in prostate MRI**

*A. W. Rix*[1], P. Burn[2], N. Vasdev[3], A. Bradley[4], A. Andreou[5], J. Aning[6], T. Barrett[1], A. R. Padhani[7], A. Shah[8]; [1]Cambridge/UK, [2]Taunton/UK, [3]Stevenage/UK, [4]Truro/UK, [5]Bath/UK, [6]Bristol/UK, [7]Northwood/UK, [8]Winchester/UK

**Purpose:** This study aimed to determine how multi-modal decision support models, integrating clinical data, PI-RADS, and AI, could help optimise patient selection for biopsy following MRI for suspected prostate cancer.
**Methods or Backround:** Clinical history, MRI, PI-RADS, and histopathology data were obtained retrospectively from a five-site, multi-vendor study of a diagnostic patient population. 352 patients were assigned for model training/ tuning, and 235 patients (Grade Group≥2 prevalence 34%) for held-out testing. GG≥2 cancer was verified by standard-of-care MRI-directed biopsy. Patients scored PI-RADS 1/2 without biopsy were considered negative. Automated AI-based software that identifies and scores patients/ lesions for risk of GG≥2 was separately trained using the same training data. Multi-modal machine learning models were trained for combinations of AI scores, clinical variables including PSA-density (PSAD), and the original reporting radiologists' PI-RADS scores. Sensitivity, specificity, and AUC were compared per-patient on the held-out test data with the PI-RADS assessments and AI scores alone.
**Results or Findings:** The original PI-RADS scores identified GG≥2 patients with sensitivity 1.00 (95% CI 1.00-1.00), specificity 0.67 (0.61-0.75) and AUC 0.94 (0.91-0.97). AI detected GG≥2 patients with sensitivity 0.97 (0.93-1.00), specificity 0.55 (0.47-0.62) and AUC 0.88 (0.84-0.92) using bpMRI data. Combining AI scores and PSAD based on TZ volume (TZ-PSAD) gave sensitivity 0.95 (0.90-0.99, p<0.001), specificity 0.70 (0.63-0.77, p<0.001) and AUC 0.90 (0.85-0.93, p=0.25). Combining PI-RADS, AI, and TZ-PSAD gave sensitivity 0.99 (0.96-1.00, p<0.001), specificity 0.83 (0.77-0.89, p<0.001), and AUC 0.96 (0.93-0.98, p=0.003). TZ-PSAD gave slightly better AUC than whole-prostate PSAD. Other clinical variables had no statistically significant benefit. Findings with bpMRI and mpMRI AI were similar.
**Conclusion:** Decision support models combining PI-RADS, AI scores, and PSAD could significantly reduce false positive biopsies while maintaining sensitivity, compared to AI or PI-RADS assessments alone.
**Limitations:** This study used standard-of-care limited biopsy for the ground truth.
**Ethics committee approval:** This study was approved with the UK HRA IRAS number: 278640.
**Funding for this study:** Funding was received from Lucida Medical.
**Author Disclosures:**
Aarti Shah: Nothing to disclose
Antony William Rix: Founder: Lucida Medical
Paul Burn: Nothing to disclose
Alison Bradley: Nothing to disclose
Tristan Barrett: Nothing to disclose
Anwar R. Padhani: Shareholder: Lucida Medical
Jon Aning: Shareholder: Lucida Medical
Nikhil Vasdev: Nothing to disclose
Adrian Andreou: Nothing to disclose

### RPS 605-4

**DL algorithm for MRI prostate volume can automatically tailor the threshold of PSA density in combination with other risk factors for the prediction of clinically significant PCa**

*A. Venturi*[1], A. Colarieti[2], D. Fazzini[1], M. Interlenghi[1], E. Schiavon[1], M. Alì[1], I. Castiglioni[1], S. Papa[1], F. Sardanelli[2]; [1]Milan/IT, [2]San Donato Milanese, Milan/IT
*(venturi@deeptracetech.com)*

**Purpose:** The aim of our study was to determine the optimal threshold for Prostate-Specific Antigen density (PSAd) when the prostate volume is automatically computed by a deep learning (DL) algorithm on T2-weighted MRI images as a contouring method. This standardised, repeatable, and reliable predictor was then combined with other predictors of clinically significant prostate cancer (csPCa).

Wednesday

**Methods or Backround:** We conducted a multicentric retrospective study, including patients assessed by mpMRI prior to prostate biopsy. csPCa was defined as a PCa with any ISUP grade group ≥2 (Gleason ≥3+4). We trained a U-Net based DL algorithm on T2-weighted images and tested by Dice Similarity Coefficient (DSC) in comparison with three board certified radiologists that segmented the prostate slice-by-slice blinded to each other. Twenty repetitions were performed.
**Results or Findings:** We included 279 patients, aged 65.5±8.0 years. The developed DL algorithm achieved a reliability (DSC) of 0.86. Repeatability was 100%. The computed PSAd ranged from 0.02–2.36ng/ml/cm3. A PSAd threshold of 0.10ng/ml/cm3 showed the best balanced sensitivity/ specificity of 0.66/ 0.64, respectively, on an external dataset of 86 patients. However, when combined with patient age, a PSAd threshold of 0.11ng/ml/cm3 and an age threshold of 67 improved sensitivity up to 0.84, without affecting the specificity.
**Conclusion:** Our results showed how PSAd threshold can be obtained by an automatic DL algorithm applied on T2-weighted images, considering the slice-by-slice prostate volume (i.e. not based on geometric approximations, such as ellipsoid diameters), and specifically optimised in combination with patient age. The inclusion of radiomics features from T2-weighted and DWI could allow a further specific optimisation of PSAd threshold.
**Limitations:** These patient cohorts were collected exclusively by Italian centres.
**Ethics committee approval:** This study was approved by the Ethics Committees of relevant centres.
**Funding for this study:** No funding was received for this study.
**Author Disclosures:**
Anna Colarieti: Nothing to disclose
Elia Schiavon: Employee: DeepTrace Technologies
Isabella Castiglioni: Founder: DeepTrace Technologies; Shareholder: DeepTrace Technologies
Sergio Papa: Nothing to disclose
Francesco Sardanelli: Grant Recipient: Bayer Grant Recipient: Bracco Grant Recipient: General Electric Healthcare; Advisory Board: DeepTrace Technologies
Deborah Fazzini: Nothing to disclose
Alessandro Venturi: Employee: DeepTrace Technologies
Marco Alì: Consultant: Bracco Imaging S.p.A.
Matteo Interlenghi: Shareholder: DeepTrace Technologies; Founder: DeepTrace Technologies

### RPS 605-5

### Impact of AI on the diagnosis of prostate cancer with mprMRI for novice radiologists: results of a single-centre study

*T. Russo*, G. Brembilla, E. Camisassa, M. Cosenza, R. Pennella, L. Quarta, G. Gandaglia, A. Briganti, F. De Cobelli; Milan/IT
*(t.russo.1994@gmail.com)*

**Purpose:** The purpose of this study was to assess the impact of a commercial AI software (Quantib Prostate) on the diagnostic accuracy and interreader agreement of novice radiologists in the interpretation of multiparametric MRI of the prostate (mpMRI).
**Methods or Backround:** Accurate interpretation of mpMRI of the prostate requires training on large case series and is affected by interreader variability. AI software have been developed to overcome these limitations and assist radiologists in evaluating mpMRI. This is a single-centre retrospective study on 110 patients who underwent mpMRI for clinical suspicion of PCa (+/- targeted biopsy) at a single center. All mpMRIs were reviewed by three novice readers (radiology residents; Reader 1, 2, 3 – R1, R2, R3) with four years (R1 and R2) and one year (R3) experience in body imaging. All MRI exams were interpreted and reported in a sequential fashion: first, radiologists interpreted the exam without AI assistance; then, they were unblinded to AI results and re-reported the MRI exam. Histopathological results from MRI-targeted and concomitant systematic biopsies were considered the standard of reference; clinically significant PCa (csPCa) was considered ISUP>1. The primary objective was to compare the diagnostic accuracy of the readers without and with AI assistance.
**Results or Findings:** 61% (67/110) of patients had any PCa (ISUP≥1), and 43% (48/110) had csPCa (ISUP≥2). The diagnostic performance of R1 and R2 remained similar with and without Quantib Prostate. R3's sensitivity and overall accuracy for csPCa improved from 81% and 55% to 91% and 60%, respectively. Percentage of interreader agreement was 74% (IC 0.684 to 0.807) without Quantib and 73% (IC 0.678 - 0.801) with Quantib.
**Conclusion:** AI-based software (Quantib Prostate) may improve the diagnostic accuracy of novice radiologists for identifying csPCa.
**Limitations:** This was a retrospective single-centre study.
**Ethics committee approval:** This study was approved by an ethics committee.
**Funding for this study:** No funding was received for this study.
**Author Disclosures:**
Michele Cosenza: Nothing to disclose
Renato Pennella: Nothing to disclose
Giorgio Brembilla: Nothing to disclose
Francesco De Cobelli: Nothing to disclose
Tommaso Russo: Nothing to disclose
Giorgio Gandaglia: Nothing to disclose
Alberto Briganti: Nothing to disclose
Enrico Camisassa: Nothing to disclose
Leonardo Quarta: Nothing to disclose

### RPS 605-6

### Multireader evaluation of a deep learning computer-aided system for prostate MRI in men with prostate cancer being considered for active surveillance

*L. I. Loebelenz*[1], A. Samani[2], A. Azam[2], D. Manea[3], D. Prezzi[2], A. Sharkey[2], O. Williams[2], S. J. Withey[2], V. Goh[2]; [1]Bern/CH, [2]London/UK, [3]Iasi/RO

**Purpose:** The Prostate Imaging-Reporting and Data System (PI-RADS) has standardised practice but variation in radiologist reporting performance remains an issue. Artificial intelligence (AI) may improve this. We aimed to evaluate the impact of a commercial deep learning (DL) software across readers of different experience in men with low-intermediate risk prostate cancer being considered for active surveillance.
**Methods or Backround:** This retrospective study included men with low-intermediate risk prostate cancer. Five readers with varying levels of experience (<1 year to ≥5 years of experience), trained in three different countries, evaluated the initial bi-parametric prostate MRI, with and without DL-assistance in a randomised design. PI-RADSv2.1 scores were recorded and compared between reads and between readers, and against ground truth using metrics including area under the receiver operating characteristics curve (AUC). Fleiss-Kappa analysis was performed for interreader agreement. Radiological ground-truth was independent expert scoring/annotation of focal lesions. Histological ground-truth was the International Society of Urological Pathologists grade group (GG) score.
**Results or Findings:** 100 men were included with mean age 61±7 years and mean PSA density 0.15±0.09 (SD). There were 23 ISUP GG ≥2 cancers on histology. At an individual-reader level, for PI-RADS scoring compared to the radiological ground truth, AUC ranged from 0.65 to 0.80. DL-assistance increased AUC, although the magnitude of benefit varied across the reader pool, AUC ranging from 0.69 to 0.82. Additionally, DL-assistance appeared to reduce interreader variability. Reader agreement (weighted kappa) ranged from 0.24 to 0.56 without DL-assistance, compared to 0.45-0.55 with DL-assistance.
**Conclusion:** AI can improve performance for PI-RADS scoring particularly for non-expert readers in this cohort, and may also reduce variability in reader performance.
**Limitations:** This was a single centre, retrospective study.
**Ethics committee approval:** This study was approved with the code: 18/NW/0297.
**Funding for this study:** This study received no direct funding.
**Author Disclosures:**
Samuel Joseph Withey: Nothing to disclose
Amy Sharkey: Nothing to disclose
Aishah Azam: Nothing to disclose
Vicky Goh: Other: Funding to institution from Siemens - PhD studentship in last three years
Omoremi Williams: Nothing to disclose
Amit Samani: Nothing to disclose
Davide Prezzi: Nothing to disclose
Laura Isabel Loebelenz: Nothing to disclose
Daniela Manea: Nothing to disclose

### RPS 605-7

### Assessing radiomic stability: impact of annotation variability on radiomics features consistency in different anatomical regions

*C. Prieto-de-la-Lastra*[1], A. Jimenez-Pastor[2], A. Picó Peris[2], D. Veiga Canuto[2], L. Marti-Bonmati[2]; [1]Madrid/ES, [2]Valencia/ES
*(carmenprieto@quibim.com)*

**Purpose:** Radiomic features are calculated from delineated regions of interest (ROIs), characterising the patient and anatomical region where they have been calculated. Therefore, the segmentation quality can substantially impact the power of radiomics. In this study, the discrepancies among different segmentations of the same ROIs are compared to analyse radiomics stability in different anatomical regions.

**Methods or Backround:** Two datasets were inspected, each with different annotations: 100 MRI studies with the prostate gland (central and peripheral) and seminal vesicles segmented; and 960 MRI scans with annotated neuroblastic lesions. The original segmentations were modified with dilations and erosions of structuring element (SE) equal to 1, 2 or 3 voxels, simulating the annotations from different radiologists. Therefore, 7 segmentations were generated for each case. 1015 radiomic features were calculated from each mask. The distributions of characteristics across annotations were compared through the Wilcoxon-test, paired and non-paired. The correlation among the different simulated annotators was analysed with the intraclass correlation index (ICC). Finally, the most stable variables maintained across all the experiments were inspected.
**Results or Findings:** In the central prostate gland, the number of stable variables from Wilcoxon analysis was 165, 8 from Wilcoxon-paired evaluation and 743 from ICC tests. According to the peripheral prostate gland, the results were 40, 0, and 406. In the seminal vesicles analysis, the results were 107, 3, and 514. Finally, the neuroblastoma dataset resulted in 34, 0, and 637 stable variables from each of the experiments, respectively. Furthermore, in both datasets, the number of stable features decreased as the size of the SE increased.
**Conclusion:** Radiomics is less stable when annotations highly differ from the original ROIs, being more susceptible to sharper and irregular shapes as the peripheral gland and cancer lesions.
**Limitations:** No limitations were identified.
**Ethics committee approval:** Thi study was approved by the Institutional Review Board and written informed consent was waived from all participants.
**Funding for this study:** Funding was received from PRIMAGE (PRedictive In-silico Multiscale Analytics to support cancer personalised diagnosis and prognosis, empowered by imaging biomarkers), a Horizon 2020|RIA project (Topic SC1-DTH-07-2018), grant agreement no: 826494.
**Author Disclosures:**
Diana Veiga Canuto: Nothing to disclose
Ana Jimenez-Pastor: Nothing to disclose
Alfonso Picó Peris: Nothing to disclose
Luis Marti-Bonmati: Nothing to disclose
Carmen Prieto-de-la-Lastra: Nothing to disclose

### RPS 605-8

**Deep learning-based risk estimation for personalised follow-up in low-risk prostate cancer surveillance**

*C. Roest*[1], T. Kwee[1], P. Van Leeuwen[2], H. Huisman[3], D. Yakar[1]; [1]Groningen/NL, [2]Amsterdam/NL, [3]Nijmegen/NL

**Purpose:** Timely follow-up in active surveillance of low-risk prostate cancer (PCa) is crucial for early detection of disease progression and to minimise overuse of diagnostics. MRI-based deep learning (DL) may optimise follow-up timing by estimating progression risk.
**Methods or Backround:** This multi-centre study included 1607 MRI scans of 1143 men undergoing MRI for suspicion of harbouring clinically significant (cs, defined as International Society of Urological Pathology>1) PCa, who were negative for csPCa at the time of the MRI scan. A novel DL model was developed, which used MRI and routine clinical parameters to predict the risk of PCa progression (defined as csPCa at follow-up). The model was internally cross-validated in 829 exams, and externally validated in 778 exams. Cox-regression assessed whether the model predicted risk of progression. Time-dependent receiver-operating characteristic curve analysis was used to compare our proposed model to established risk estimation tools (European Randomised study of Screening for Prostate Cancer [ERSPC], Prostate Cancer Prevention Trial risk calculators [PCPT]) and PI-RADS. The area-under-the-curve was calculated five years after MRI. Optimized follow-up intervals were derived from Kaplan-Meier curves.
**Results or Findings:** DL scores predicted progression (internal: hazard-ratio [HR] 14.01, CI 6.61-30.65; p<0.001; external: HR 16.21, CI 3.48-75.5; p<0.001). DL achieved the highest area-under-the-curve in internal (0.75, CI 0.66-0.85) and external cohorts (0.7, CI 0.64-0.76). Internally, DL outperformed ERSPC (p=0.002) and PI-RADS (p=0.006). Externally, DL outperformed ERSPC (p=0.02) and PCPT scores (p<0.001). On internal validation, DL identified a 20% stratum of very-low-risk PCa with <10% risk of missed progression after 3.5 year follow-up, and <15% risk on external validation.
**Conclusion:** Our proposed DL model provided more accurate risk estimations compared to established methods. DL risk scores may help to personalise follow-up protocols for low-risk PCa.
**Limitations:** No limitations were identified.
**Ethics committee approval:** This was a retrospective study.
**Funding for this study:** Funding was provided by a grant from Siemens-Healthineers.
**Author Disclosures:**
Henkjan Huisman: Nothing to disclose
Pim Van Leeuwen: Nothing to disclose
Thomas Kwee: Nothing to disclose
Derya Yakar: Nothing to disclose
Christian Roest: Research/Grant Support: Grant recipient, Siemens Healthineers

### RPS 605-9

**Development and external validation of PSMA-PET/MR based radiomics models to predict Gleason score in prostate cancer**

*T. Yang*[1], W. Tao[1], Y. Song[1], J. Zhang[2], X. Niu[1], G. Fu[1], G. Bai[1]; [1]Huai'an/CN, [2]Taizhou/CN

**Purpose:** This research aims to establish radiomics models based on PSMA PET/MR scans with external validation to predict GS of PCa.
**Methods or Backround:** A total of 192 PCa patients were enrolled in this study, including 160 patients in the internal validation set (Centre A) and 32 ones in the external validation set (Centre B and Centre C). PET/MR scans were performed prior to clinical treatment, and three kinds of radiopharmaceuticals ((18F)-PSMA-1007, (68Ga)Ga-PSMA-11 and (Al18F)-PSMA-BCH) were randomly applied in the PSMA-targeted PET examinations. The patients were divided into the low-risk group (GS≤7) and the high-risk group (GS>7). 1409 high-throughput features were extracted from each ROI and selected using the LASSO algorithm. Radiomics models were constructed based on the above selected features through machine learning algorithm of LR, NB, RF, SVM, and XGBoost through 30 times 4-fold repeated cross-validation, respectively. The performance of every model was evaluated through the ROC curve. The optimal algorithm and radiomics model were chosen according to the AUC value.
**Results or Findings:** 12-14 radiomics features and NB algorithm were selected to radiomics models' establishment. In the external validation set, the models based on PSMA-PET, T2WI, and ADC maps exhibited stable predictive performance with AUC values of 0.762, 0.698, and 0.668 (75.0%, 65.6%, and 71.9% accuracy).
**Conclusion:** Our study demonstrated that PSMA-targeted PET-based radiomics model occupied better performance in the GS prediction than those based on T2WI and ADC through the external cohort validation. Radiomics model of PSMA-targeted PET could be utilised to predict PCa prognosis noninvasively and help clinicians make individualised treatment plans for patients.
**Limitations:** The number of patients in our study was small, and further large-scale data research from multiple centres will be conducted in the future.
**Ethics committee approval:** This study was approved by the Ethics Committee of The Affiliated Huaian No.1 People's Hospital of Nanjing Medical University (Date 2021.12.22 / No YX-2021-113-01).
**Funding for this study:** This study received funding from the Huai'an Science and Technology Project (grant no. HAB202017 to WT).
**Author Disclosures:**
Genji Bai: Nothing to disclose
Tianshuo Yang: Nothing to disclose
Guangbo Fu: Nothing to disclose
Xiaobing Niu: Nothing to disclose
Jun Zhang: Nothing to disclose
Yaqi Song: Nothing to disclose
Weijing Tao: Nothing to disclose

### Thursday, February 29

**08:00-09:00** **Research Stage 1**

### Research Presentation Session: Artificial Intelligence & Machine Learning & Imaging Informatics

### RPS 705

### Radiomics applications in MRI

**Moderator**
A. Ammirabile; Milan/IT
*(angela.ammirabile@humanitas.it)*

### RPS 705-2

**Magnetic resonance radiomics-derived sphericity correlates with seizure in brain arteriovenous malformations**

*J-Y. Lin*[1], C. Lu[2], Y-S. Hu[3], J. K. Loo[1], K-L. Lee[1], C. Liao[4], C-J. Lin[1]; [1]Taipei/TW, [2]Taipei City/TW, [3]New Taipei/TW, [4]Texas, TX/US
*(zan40004@gmail.com)*

**Purpose:** Angioarchitectural analysis of brain arteriovenous malformations (BAVMs) is qualitative and subject to interpretation. This study quantified the morphology of and signal changes in the nidal and perinidal areas by using MR radiomics and compared the performance of MR radiomics and angioarchitectural analysis in detecting epileptic BAVMs.
**Methods or Backround:** From 2010 to 2020, a total of 111 patients with supratentorial BAVMs were retrospectively included and grouped in accordance with the initial presentation of seizure. Patients' angiograms and MR imaging results were analysed to determine the corresponding angioarchitecture. The BAVM nidus was contoured on time-of-flight MR angiography images. The perinidal brain parenchyma was contoured on T2-weighted images, followed by radiomic analysis. Logistic regression analysis was performed to determine the independent risk factors for seizure. ROC curve analysis, decision curve analysis (DCA), and calibration curve were performed to compare the performance of angioarchitecture-based and radiomics-based models in diagnosing epileptic BAVMs.
**Results or Findings:** In multivariate analyses, low sphericity (OR: 2012.07, p=0.04) and angiogenesis (OR: 5.30, p=0.01) were independently associated with a high risk of seizure after adjustment for age, sex, temporal location, and nidal volume. The AUC for the angioarchitecture-based, MR radiomics-based, and combined models was 0.672, 0.817, and 0.794, respectively. DCA confirmed the clinical utility of the MR radiomics-based and combined models.
**Conclusion:** Low nidal sphericity and angiogenesis were associated with high seizure risk in patients with BAVMs. MR radiomics-derived tools may be used for noninvasive and objective measurement for evaluating the risk of seizure due to BAVM.
**Limitations:** Although the dataset acquired from a single institution and machine may improve the homogeneity of image data quality, an external validation dataset should be considered in future studies to improve the generalisability of prediction models.
**Ethics committee approval:** This study was approved by the Third Institutional Review Board of Taipei Veterans General Hospital. The protocol was implemented after review and approval by the Human Research Protection Center of TPEVGH (IRB No.: 2020–06-005C).
**Funding for this study:** Funding was received from the Taipei Veterans General Hospital (grant number: V111C- 073) and Taiwan's Ministry of Science and Technology (grant number: MOST- 109–2628-B-0).
**Author Disclosures:**
Chien-Yi Liao: Nothing to disclose
Jing Kai Loo: Nothing to disclose
Chung-Jung Lin: Nothing to disclose
Kang-Lung Lee: Nothing to disclose
Yong-Sin Hu: Nothing to disclose
Jih-Yuan Lin: Nothing to disclose
Chia-Feng Lu: Nothing to disclose

### RPS 705-3

**MRI radiomics and machine learning: an innovative MRI radiomics and machine learning-based method to predict treatment response in MRI-guided HIFU ablation of bone metastasis**

*V. D'Agostino*[1], M. P. Aparisi Gomez[2], R. Sassi[1], A. G. Morganti[1], M. Buwenge[1], A. Bazzocchi[1]; [1]Bologna/IT, [2]Auckland/NZ
*(valerio.dagostino123@gmail.com)*

**Purpose:** Pain management of bone metastases is performed with systemic and local therapies. External Beam radiotherapy is currently the gold standard for treatment of painful metastases, however MRI-guided high-intensity focused ultrasound (MRgHIFU) has shown great results in pain relief. To date, a reliable imaging method to predict the success of the treatment is yet to be defined. This work aims to investigate the potential role of a radiomics-based machine-learning (ML) algorithm applied to pre- and post-treatment MR T1w and T2w-images for the prediction of a clinical success (reduction of ≥6 point in the numerical rating pain scale) of MRgHIFU treatment.
**Methods or Backround:** 188 patients (112 females, 76 males) with 200 bone metastases, who underwent MRgHIFU ablation were retrospectively selected and classified into two groups, on the basis of clinical success of the treatment. Two-dimensional segmentations were manually drawn by an MSK-expert radiologist on axial pre- and post-treatment T1w and T2w sequences. Radiomic feature extraction was performed using PyRadiomics. To reduce dimensionality, variance and intercorrelation analysis were used. Subsequently, a LogitBoost classifier was trained with stratified cross-validation, tested and validated within our population.
**Results or Findings:** Group A (Responders) reported 112 lesions; group B (Not-responders) reported 88 lesions. 3567 radiomics features were extracted, of which 2864 were discarded due to high intercorrelation (>0.8). The feature selection process identified ten features to build the ML classifier, which was able to correctly classify 94% of instances on the training set and 85% on the testing set. Weighted average precision and recall were 0.90 and 0.92 respectively, while the AUROC curve was 0.88. The performance was similar within the validation set.
**Conclusion:** An ML-classifier powered by MRI radiomics might be a feasible tool to predict bone metastases pain response to MRgHIFU.
**Limitations:** The study was limited by a lack of external validation, mild class imbalance, and its retrospective nature.
**Ethics committee approval:** No information provided by the submitter.
**Funding for this study:** No funding was received for this study.
**Author Disclosures:**
Alessio Giuseppe Morganti: Nothing to disclose
Milly Buwenge: Nothing to disclose
Alberto Bazzocchi: Nothing to disclose
Maria Pilar Aparisi Gomez: Nothing to disclose
Rebecca Sassi: Nothing to disclose
Valerio D'Agostino: Nothing to disclose

### RPS 705-4

**Reproducible radiomics features from multi-MRI-scanner test-retest-study: influence on performance and generalisability of radiomics models**

*M. Wennmann*, L. Rotkopf, F. Bauer, H. Goldschmidt, T. F. Weber, H-P. Schlemmer, S. Delorme, K. H. Maier-Hein, P. Neher; Heidelberg/DE
*(m.wennmann@dkfz.de)*

**Purpose:** The aim of this study was to evaluate the influence of using only a subset of reproducible radiomics features, defined in a prior in-vivo multi-MRI-scanner test-retest-study, on the generalisability and external performance of radiomics models.
**Methods or Backround:** This retrospective study used data acquired between 2015 and 2021. The task for the radiomics models was to predict bone marrow plasma cell infiltration from MRI in myeloma patients. Different machine learning (ML) models were trained on data from Centre 1, using either all radiomics features, or using only reproducible radiomics features defined by a prior in-vivo multi-MRI-scanner test-retest study. Models were tested on an internal and a multicentric external data set. Pearson correlation coefficient r and mean absolute error (MAE) between predicted and actual plasma cell infiltration were used to quantify the model performance. The difference between performance on the internal and external test set was calculated to measure generalisability.
**Results or Findings:** 302 MRIs from 300 patients from 8 centres were included. When using only reproducible features compared to all features, for all ML models the generalisability improved. However, for the best model, a random forest regressor, the model using all features still outperformed the model using only reproducible features on the external test set (r of 0.44 vs. 0.33 and MAE of 20.5 vs 21.9). When comparing the external performance across all combinations of ML models and feature selection methods, a random forest regressor using all features (r=0.44, MAE=20.5) showed the best external performance.
**Conclusion:** A radiomics feature selection based on in-vivo reproducibility experiments between different MRI scanners improves the generalisability of radiomics models, however, does not necessarily lead to an improvement of the external performance of the overall best radiomics model.
**Limitations:** The study is limited by its retrospective nature.
**Ethics committee approval:** This study was approved by the local Institutional Research Board with code: S-537/2020.
**Funding for this study:** No funding was received for this study.
**Author Disclosures:**
Hartmut Goldschmidt: Research/Grant Support: Amgen, Array Biopharma/Pfizer, BMS, Celgene, Chugai, Dietmar-Hopp-Foundation, GlycoMimetics Inc., GSK, Heidelberg Pharma, Hoffmann-La Roche, Karyopharm, Janssen, Johns Hopkins University, Incyte, Millenium

Pharmaceuticals Inc., Molecular Partners, Merck Sharp and Dohme, MorphoSys AG, Mundipharma GmbH, Pfizer, Sanofi, Takeda, and Novartis, Sanofi; Advisory Board: Adaptive Biotechnology, Amgen, BMS, Janssen, Sanofi; Other: Amgen, BMS, Chugai, GlaxoSmithKline, Janssen, Novartis, Sanofi and Pfizer
Fabian Bauer: Nothing to disclose
Stefan Delorme: Nothing to disclose
Peter Neher: Nothing to disclose
Lukas Rotkopf: Nothing to disclose
Tim Frederik Weber: Nothing to disclose
Klaus H. Maier-Hein: Nothing to disclose
Markus Wennmann: Nothing to disclose
Heinz-Peter Schlemmer: Consultant: Curagita and Bayer; Consultant: Siemens, Curagita, Profound, and Bayer; Speaker: Siemens, Curagita, Profound, and Bayer; Advisory Board: Curagita

### RPS 705-5

**MRI-based radiomics analysis for prediction of treatment response to neoadjuvant chemoradiotherapy in patients with locally advanced rectal cancer: a large multicentric study**

T. Hu[1], J. Gong[1], Y. Sun[1], M. Li[1], C. Cai[1], Y. Cui[2], X. Zhang[3], T. Tong[1], *Y. Feng*[1]; [1]Shanghai/CN, [2]Taiyuan/CN, [3]Beijing/CN
*(fengyaru915@163.com)*

**Purpose:** The aim of this study was to investigate the ability of the MRI-based radiomics models for pretreatment prediction of good response (GR) to neoadjuvant chemoradiotherapy (nCRT) in patients with locally advanced rectal cancer (LARC).
**Methods or Backround:** A total of 921 patients with LARC were retrospectively recruited from 3 hospitals, including a training dataset (TD) (n=508) and external validation datasets 1 (EVD1) (n=242) and 2 (EVD2) (n=171). Radiomics features were extracted from the T2WI and ADC images. Three classifications, including logistic regression (LR), random forest (RF), and support vector machine (SVM) were applied to construct radiomics models for predicting GR. The clinical-MRI model was constructed with significant clinical characteristics and MRI morphological features by using the logistic regression analysis. The prediction performance was evaluated using the area under the curve (AUC) and decision curve analysis (DCA).
**Results or Findings:** Two clinical-MRI features and ten radiomic features were selected for the GR prediction. Compared to models from other classifiers and the clinical-MRI model, the model obtained with SVM showed promising discrimination of GR to nCRT with AUCs of 0.798 (95% CI, 0.758-0.837), 0.790 (95% CI, 0.725-0.856) and 0.743 (95% CI, 0.666-0.821) in the training and two external validation datasets respectively. Decision curve analysis confirmed that the radiomics models were clinically useful.
**Conclusion:** The MRI-based radiomics model exhibited better performance for response prediction to nCRT in LARC patients than the clinical-MRI model, and also provided value for prognosis prediction.
**Limitations:** Selection bias may have been introduced by excluding patients with clinical complete response. The ROI did not include lymph nodes. Manual segmentation of ROIs is a time-consuming procedure and requires accurate identification of MRI lesions.
**Ethics committee approval:** The study was approved by the Institutional Review Boards of all participating centres, and the requirement for informed consent was waived due to the retrospective nature.
**Funding for this study:** Funding for this study was received from the National Natural Science Foundation of China (No.82001776, 81971687, 82271946),Shanghai Natural Science Foundation (No.20ZR1412700).
**Author Disclosures:**
Yaru Feng: Nothing to disclose
Chongpeng Cai: Nothing to disclose
Jing Gong: Nothing to disclose
Yiqun Sun: Nothing to disclose
Menglei Li: Nothing to disclose
Yanfen Cui: Nothing to disclose
Tingdan Hu: Nothing to disclose
Xiaoyan Zhang: Nothing to disclose
Tong Tong: Nothing to disclose

### RPS 705-6

**Prediction of the Ki-67 expression level for head and neck squamous cell carcinoma with machine learning-based multiparametric MRI radiomics**

*W. Chen*, G. Lin, J. Ji; Lishui/CN
*(lsszxyycwy@163.com)*

**Purpose:** The aim of this study was to develop and validate a machine learning-based fusion model to preoperatively predict Ki-67 expression levels in patients with head and neck squamous cell carcinoma (HNSCC) using multiparametric magnetic resonance imaging (MRI) images.
**Methods or Backround:** A total of 152 patients with pathologically proven HNSCC were retrospectively enrolled and divided into training (n=106) and validation (n=46) cohorts. Features were extracted from T2-weighted imaging fat suppression and contrast-enhanced T1-weighted images and screened using the least absolute shrinkage and selection operator (LASSO) regression. Seven machine learning classifiers, including k-nearest neighbors (KNN), support vector machine (SVM), logistic regression (LR), random forest (RF), linear discriminant analysis (LDA), naive Bayes (NB), and eXtreme Gradient Boosting (XGBoost) were trained. The best classifier was evaluated to calculate radiomics (Rad)-scores and combined clinical factors to build a fusion model, which was visualised as a nomogram. Performance was evaluated by the area under the receiver operating characteristic curve (AUC), calibration curve, and decision curve analysis (DCA).
**Results or Findings:** The SVM classifier showed the best performance, with an AUC of 0.862 in the validation cohort. The fusion model incorporating SVM-based Rad-scores, clinical T stage and MR-reported LN status was constructed and achieved AUCs of 0.915 (0.864–0.967) and 0.896 (0.770–0.966) in the training and validation cohorts, with accuracies of 91.98% and 84.78%, respectively. The calibration curves demonstrated a good model fit, and DCA showed the clinical benefits of the fusion model.
**Conclusion:** The machine learning-based fusion model based on multiparametric MRI can predict the expression of Ki-67 in HNSCC patients, which might be helpful for prognosis evaluation and clinical decision-making.
**Limitations:** This is a retrospective single-centre study with unavoidable bias and a limited sample size.
**Ethics committee approval:** All procedures performed in studies involving human participants were in accordance with the ethical standards of the institutional and/or national research committee and with the 1964 Helsinki declaration and its later amendments or comparable ethical standards. This study was approved by the Institutional Review Board and Human Ethics Committee of the Fifth Affiliated Hospital of Wenzhou Medical University (No. 2023-523), with the requirement for patient informed consent being waived due to its retrospective nature. All patients' information was anonymised prior to the analysis.
**Funding for this study:** Funding for this study was received from the National Natural Science Foundation of China (No.82072026).
**Author Disclosures:**
Jiansong Ji: Nothing to disclose
Weiyue Chen: Nothing to disclose
Guihan Lin: Nothing to disclose

### RPS 705-7

**Texture analysis and rectal cancer: correlation with histology and prognosis in patients with advanced rectal cancer**

*I. Mariani*, C. Maino, T. P. Giandola, C. Talei Franzesi, D. Ippolito; Monza/IT
*(i.mariani.dot@gmail.com)*

**Purpose:** The aim of this study was to retrospectively collect radiomic data from preoperative rectal MR and determine the possible relationships between texture analysis and response to neoadjuvant treatment.
**Methods or Backround:** 88 patients with biopsy-proven advanced rectal adenocarcinoma, staging MR and RAR after neoadjuvant treatmente were enrolled. Based on tumour regression grade, we considered TRG 1-2 patients as responders and TRG 3-5 patients as non-responders. Texture analysis was conducted by using LIFex software, where T2-weighted oblique axial MR sequences were uploaded; a region-of-interest (ROI) was manually drawn on a single slice. Features with a Spearman correlation index >0.5 have been discarded and a LASSO feature selection has been applied. Selected features were trained using bootstrapping.
**Results or Findings:** According to TRG classes 49 patients (55.8%) were considered responders while 39 (44.2%) as non-responders. Two features were associated with responders' classes: GLCM_Homogeneity and Discretised Histo Entropy log 2. Regarding GLCM_Homogeneity, the area under the receiver operating characteristic curve (AUC), sensitivity (sens), specificity (spec), positive predictive value (PPV), and negative predictive value (NPV) were: 0.779 (95% CIs=0.771-0.816), 86% (80-90), 67% (60-71%), 81% (76-84), and 88% (84-90), respectively. Regarding Discretised Histo Entropy log 2, diagnostic values were as follows: AUC=0.775 (0.700-0.801), sens=80% (74-83), spec=63% (58-69%), PPV=77% (70-81), and NPV=82% (80-85). By combing both radiomics features the radiomics signature diagnostic accuracy increased (AUC=0.844, p<0.05). Finally, the AUC of 1000 bootstraps was 0.810.
**Conclusion:** Texture analysis can be considered an advanced complementary diagnostic tool to determine a possible correlation between pre-surgical MR data and response to neoadjuvant therapy.
**Limitations:** Considering its low robustness, further studies with a larger cohort of patients should aim to validate these preliminary data.
**Ethics committee approval:** No information provided by the submitter.
**Funding for this study:** No funding was received for this study.
**Author Disclosures:**
Cesare Maino: Nothing to disclose
Teresa Paola Giandola: Nothing to disclose
Davide Ippolito: Nothing to disclose
Cammillo Talei Franzesi: Nothing to disclose
Ilaria Mariani: Nothing to disclose

### RPS 705-8

**Prediction of overall survival in paediatric neuroblastoma patients through machine learning in the large multi-institutional PRIMAGE cohort**

*J. Lozano*[1], A. Jimenez-Pastor[1], G. Weiss[2], D. Veiga Canuto[1], B. Martínez De Las Heras[1], A. Cañete Nieto[1], B. Hero[3], R. Ladenstein[4], L. Marti-Bonmati[1]; [1]Valencia/ES, [2]Boston, MA/US, [3]Cologne/DE, [4]Vienna/AT
*(joselozano@quibim.com)*

**Purpose:** Neuroblastoma (NB) is the most frequent and highly aggressive solid cancer in childhood, in which imaging plays a pivotal role at every step of the patient's journey. This study sought to develop a machine learning model using clinical, molecular, and magnetic resonance (MR) radiomics features to predict patient's overall survival (OS) and aid in their stratification.
**Methods or Backround:** A database of 513 patients was used for model training, validation, and testing. Furthermore, 22 additional patients from hospitals not originally in the database were utilised as an external test. Manual tumour segmentations of the NB were conducted on the corresponding T2-weighted MR images to segment the primary tumour by an experienced radiologist. In total, 107 radiomics features were extracted and subsequently harmonised across manufacturers and magnetic field strengths using the nested ComBat methodology. Finally, radiomic features were combined with the clinical and molecular data to serve as input for the models. A nested cross-validation approach was used as training methodology to select the best preprocessing and model configuration.
**Results or Findings:** A C-index of 0.788±0.049 was achieved in the test, being a random survival forest the model showing the best performance. For the additional 22 patients, a C-index of 0.934 was obtained. The model exhibited superior predictive performance and patient stratification compared to the standard risk group INRG. Interpretability analysis revealed the significance of clinical variables, with radiomics features related to lesion heterogeneity and size playing an important role in prediction.
**Conclusion:** The OS predictive model demonstrated high performance and alignment with established clinical variables, highlighting the importance of radiomics features. It presents new evidence for enhancing patient care and clinical decision-making.
**Limitations:** Greater sample sizes are required in the external test to confirm the results.
**Ethics committee approval:** This study was approved by an Institutional Review Board and written informed consent was obtained from all participant centres.
**Funding for this study:** Funding was received from PRIMAGE (PRedictive In-silico Multiscale Analytics to support cancer personalized diagnosis and prognosis, empowered by imaging biomarkers), a Horizon 2020|RIA project (Topic SC1-DTH-07-2018), grant agreement no: 826494.
**Author Disclosures:**
Diana Veiga Canuto: Nothing to disclose
Luis Marti-Bonmati: Nothing to disclose
Glen Weiss: Nothing to disclose
Ana Jimenez-Pastor: Nothing to disclose
Barbara Hero: Nothing to disclose
Ruth Ladenstein: Nothing to disclose
Adela Cañete Nieto: Nothing to disclose
Blanca Martínez De Las Heras: Nothing to disclose
Jose Lozano: Nothing to disclose

### RPS 705-9

**mp-MRI radiomic model predicts peri-tumour tertiary lymphoid structures in hepatocellular carcinoma: a multi-centre study**

*S. Long*, J. Chen, L. Zhong, W. Liu, Y. Pei, W. Li; Changsha/CN
*(lscmustupup@163.com)*

**Purpose:** The massive presence of peri-tumour tertiary lymphoid structure (mpTLS) questions whether HCC patients can benefit from immunotherapy. However, it has been identified only by pathological examination. This study aims to develop a noninvasive tool using preoperative multiple parameter MR imaging (mpMRI) radiomic for predicting mpTLS.
**Methods or Backround:** 584 consecutive HCC patients (mpTLS+:154; mpTLS-:430) were retrospectively recruited from four independent institutes and were divided into training (n=307) in one institute, and a validation cohort (n=277) in the other three institute. 76 HCC participants (mpTLS+:21; mpTLS-:55) were also enrolled as prospective cohort, including the relapse-free survival (RFS) and overall survival (OS). All subjects underwent preoperative mpMRI. Three different models (Model 1: peri-tumour model; Model 2: intra-tumour model; Model 3: combined models 1 and 2) were constructed to stratify mpTLS+. The optimal model was decided by the maximum area under of curve (AUC) in the training set and validated in both the validation and the prospective cohort, which was further used to predict the RFS and OS in the prospective data.
**Results or Findings:** For retrospective data, Model 3 (AUC:0.92) was the optimal model for diagnosing mpTLS+ than model 2 (AUC:0.87) and model 1 (AUC:0.85) in training cohorts (all P<0.001), which was validated in validation cohort (AUC: 0.91 vs 0.85 vs 0.84; all P<0.001), and prospective corhort (AUC:0.91 vs 0.84 vs 0.77; all P<0.001). For prospective data, Model 3 could predict the RFS (P < 0.001) and OS (P<0.001) based on mpTLS+.
**Conclusion:** Model 3 (the combined model) is a reliable and noninvasive tool for predicting mpTLS and further can forecast OS and RFS, which is helpful in deciding immunotherapy for HCC patients.
**Limitations:** No limitations were identified.
**Ethics committee approval:** This study was approved with the Ethics Committee number: 2018111101.
**Funding for this study:** Funding was received from the National Natural Science Foundation of China [82071895 and 82271984] to W.Z.L.; Hunan Provincial Science and Technology Department [2023JJ30903 and 2022JJ30950] to W.Z.L. and Y.G.P. National Geriatric Disease Clinical Medical Research Center Foundation [2022LNJJ08] to Y.G.P. Youth Project of Natural Science Foundation of Hunan Province to W.G.L.
**Author Disclosures:**
Wenguang Liu: Nothing to disclose
Wenzheng Li: Nothing to disclose
Shichao Long: Nothing to disclose
Yigang Pei: Nothing to disclose
Linhui Zhong: Nothing to disclose
Juan Chen: Nothing to disclose

**08:00-09:00 Research Stage 2**

### Research Presentation Session: Neuro

### RPS 711

### Stroke: treatment and outcome

**Moderator**
M. T. El-Diasty; Jeddah/SA
*(meldiasty@hotmail.com)*

### RPS 711-2

**Cost-effectiveness of mobile stroke units in Germany: a perspective on catchment areas, operating modes, and staffing**

*F. Tollens*, C. Hoyer, K. Szabo, J. S. Rink; Mannheim/DE

**Purpose:** Estimating the cost-effectiveness of future mobile stroke unit (MSU) services with respect to local idiosyncrasies is essential for optimising major program determinants and enabling large-scale implementation of MSU services. Therefore, the aim of this study was to assess the cost-effectiveness of MSU services for varying urban German settings.
**Methods or Backround:** Costs of different operating modes and hours including weekend and non-weekend coverage, including different personnel configurations, were simulated for the German healthcare system. Ischaemic stroke incidence, circadian distribution, rates of alternative diagnoses and stroke mimics, as well as missed cases, were incorporated to model case coverage and patient-level costs of acute stroke care. Based on internationally reported stroke outcomes, a five-year Markov Model was applied to analyse the cost-effectiveness outcomes for varying catchment zone populations.
**Results or Findings:** Compared to regular emergency medical services, stroke care by MSU achieved additional 0.06 QALYs on average over a five-year time horizon. Assuming a catchment zone of 400,000 inhabitants and 12 hour/ seven day coverage resulted in an incremental cost-effectiveness ratio (ICER) of €42,069 per QALY gained. Lower ICERs were possible when coverage was expanded to 16 hour-service on 7 days per week. Sensitivity analyses revealed that the population size within the catchment zones and the number of ischaemic strokes missed by MSU deteriorated economic performance of MSU significantly.
**Conclusion:** Major determinants of cost-effectiveness should be addressed when setting up novel MSU programs. For the German healthcare system, the catchment zone should cover at least 400,000 inhabitants and MSU service should cover 12-16 hours per day, including weekends.
**Limitations:** This study used a model-based economic evaluation of MSU program costs and stroke case estimates.
**Ethics committee approval:** There was no ethical approval necessary as no human data was analysed.
**Funding for this study:** No funding was received for this study.
**Author Disclosures:**
Carolin Hoyer: Nothing to disclose
Johann S Rink: Nothing to disclose
Fabian Tollens: Nothing to disclose
Kristina Szabo: Nothing to disclose

### RPS 711-3

**Set-up and planning of a MSU service in a medium-sized German urban area**

F. Tollens, *J. S. Rink*, C. Hoyer, K. Szabo; Mannheim/DE
*(johann.rink@medma.uni-heidelberg.de)*

**Purpose:** Mobile stroke units (MSU) equipped with mobile CT scanners have demonstrated major improvements in prehospital stroke care. Due to the geographical, social, and structural characteristics of the German city of Mannheim within the Rhine-Neckar Metropolitan Region, concepts of previously established MSU services cannot directly be transferred to Mannheim. The present analysis aimed to identify major determinants that need to be considered when setting up an MSU service in Mannheim.
**Methods or Backround:** Program costs consisting of hardware investments, CT scanner and contrast media injector, personnel, and project management were estimated based on hospital accounting and vendors. Local stroke statistics from 2015 to 2021 were analysed to calculate the circadian distribution of strokes and local incidence rates. Future MSU patient numbers and program costs were analysed for varying operating modes, daytime coverage models, and staffing configurations. Case coverage and economic determinants were assessed in sensitivity analyses.
**Results or Findings:** 54.3% of all stroke patients were admitted during a ten-hour time window on weekdays. When assuming that about half of all incident stroke patients did not call the emergency centre or were missed at dispatch, an average of 0.8 stroke patients could be expected in a 10-hour shift each day in Mannheim, which could potentially be increased by expanding the catchment area. Overall estimated MSU costs amounted to €815,087 per annum for this operation mode. Teleneurological assessment reduced overall costs by 11.7%.
**Conclusion:** This analysis provides a framework of determinants and considerations to address during the design process of a novel MSU program to balance stroke care improvements with the sustainable use of scarce resources.
**Limitations:** The study is limited by using a model-based economic evaluation of MSU program costs and stroke case estimates.
**Ethics committee approval:** There was no ethical approval necessary as no human data was analysed.
**Funding for this study:** No funding was received for this study.
**Author Disclosures:**
Carolin Hoyer: Nothing to disclose
Johann S. Rink: Nothing to disclose
Fabian Tollens: Nothing to disclose
Kristina Szabo: Nothing to disclose

### RPS 711-4

**Clinical scores and imaging-based thrombus biomarkers for predicting recanalisation after stent-retriever mechanical thrombectomy**

*K. J. Gordillo Zabaleta*[1], V. Cuba[1], O. Chirife[1], E. Ripoll[1], S. Aixut[1], L. Aja[1], A. Nuñez[1], M. Comas[2], J. Puig[2]; [1]Barcelona/ES, [2]Girona/ES
*(kjgordilloz408@gmail.com)*

**Purpose:** Stent-retrievers (SR) are safe and effective devices for acute ischaemic stroke due to large vessel occlusion (LVO). However, the efficacy of this technique in treating certain patients remains dubious. No imaging biomarkers are available to predict the efficacy of these devices. The prediction of a fast and complete recanalisation after a single device pass (first-pass effect, FPE) could improve and optimise decision-making in treating acute stroke. The objective of this study was to identify predictors of recanalisation after SR mechanical thrombectomy.
**Methods or Backround:** We performed a retrospective observational study with prospective inclusion to assess the efficacy and safety of the Catchview Maxi stent-retriever device (BALT, Montmorency, France). We included 94 stroke patients with LVO. Location, length, visual density, attenuation Hounsfield Unit (HU) coefficient, and permeability were the thrombus-related parameters analysed on non-contrast CT and CT angiography. Collaterals and intracranial vascular calcification were also evaluated. FPE was defined as the achievement of mTICI2c-3.
**Results or Findings:** FPE (26 patients, 28.3%) was associated with smoking (p=0.030), greater rates of baseline mRS 0-2 (p=0.008), lower NIHSS score at baseline (p=0.040), 24-NIHSS score (p<0.001), greater rates of 90-day mRS 0-2 (p=0.003), shorter thrombus length (p=0.05), and good collaterals (p=0.070). Patients with final mTICI 2c/3 (63 patients, 66%) were younger (median age, 74 vs 80 years; p<0.001), had lower 24-NIHSS score (16 vs 19; p=0.010), higher greater rates of good functional outcome at 90 days (mRS 0-2, 40.7% vs 7.1%; p=0.003), lower 24h-infarct volume (23.9 vs 98 mL; p=0.057), higher thrombus HU ratio (1.3 vs 1.2; p=0.034), and higher thrombus permeability (enhancement percentage difference, 38.7 vs 21.9; p=0.016).
**Conclusion:** Combining clinical scores and imaging-based thrombus biomarkers is useful for predicting recanalisation after SR mechanical thrombectomy.
**Limitations:** This was a monocentric retrospective study design.
**Ethics committee approval:** This study was approved by BITE-OUT (reference number of approval: PR237/23).
**Funding for this study:** No funding was received for this study.
**Author Disclosures:**
Sonia Aixut: Nothing to disclose
Josep Puig: Nothing to disclose
Victor Cuba: Nothing to disclose
Enric Ripoll: Nothing to disclose
Karina Janeth Gordillo Zabaleta: Nothing to disclose
Oscar Chirife: Nothing to disclose
Marc Comas: Nothing to disclose
Ana Nuñez: Nothing to disclose
Lucía Aja: Nothing to disclose

### RPS 711-5

**Haemodynamic consequences in endovascular treatment of symptomatic carotid stenosis: a comprehensive analysis**

*J. Rodríguez Castro*[1], E. Murias Quintana[1], S. Budiño Torres[1], J. M. Jiménez Pérez[1], M. García Ramos[1], E. Uceda Andrés[1], P. García Martínez[2], P. Vega Valdés[1]; [1]Oviedo/ES, [2]Salamanca/ES
*(jorocas03@gmail.com)*

**Purpose:** The purpose of this study was to assess the prevalence of bradycardia and hypotension during and immediately post-endovascular intervention in patients with symptomatic carotid stenosis. Additionally, the research explores personal variables, radiological stenosis characteristics, procedural techniques, and associated medical complications contributing to these symptoms.
**Methods or Backround:** We conducted a retrospective observational cohort study, analysing a registry of 54 patients who underwent angioplasty and stent placement for symptomatic carotid stenosis at our centre. A thorough collection of clinical and analytical variables was performed for subsequent statistical analysis.
**Results or Findings:** Of the sampled patients, 34% (17) experienced haemodynamic depression post-procedure, with only four presenting symptoms. Notably, three symptomatic patients were smokers, and two consumed over 40 standard drink units (SDUs) of alcohol daily. The four patients with hypotension had significant contralateral internal carotid artery (ICA) stenosis. Among those with bradycardia, 10 of 16 had stenosis near the carotid bifurcation. Interestingly, patients developing complications had shorter hospitalisation times compared to those without.
**Conclusion:** The incidence of haemodynamic depression in patients with symptomatic carotid stenosis undergoing angioplasty and stent placement is 34%. Elevated alcohol consumption (>40 SDUs/day) and significant contralateral ICA stenosis increase the risk of post-treatment hypotension in our series. Characteristics of the stent, angioplasty balloon, use of corticosteroids, and procedural duration showed no correlation with haemodynamic depression. Development of haemodynamic depression post-angioplasty does not necessitate prolonged hospitalisation or increase in serious complications.
**Limitations:** While our study sheds light on significant associations, the retrospective nature and limited sample size may warrant further investigation for a more comprehensive understanding.
**Ethics committee approval:** Written consent has been obtained from all patients to receive the described treatment. Patient data has been anonymised. Due to the nature of this study involving a routine interventional procedure, specific consent from the Hospital Ethics Committee was not required.
**Funding for this study:** No funding was received for this study.
**Author Disclosures:**
Mateo García Ramos: Nothing to disclose
Pedro Vega Valdés: Nothing to disclose
José María Jiménez Pérez: Nothing to disclose
Elena Uceda Andrés: Nothing to disclose
Paloma García Martínez: Nothing to disclose
José Rodríguez Castro: Nothing to disclose
Sara Budiño Torres: Nothing to disclose
Eduardo Murias Quintana: Nothing to disclose

### RPS 711-6

**Post-stroke cerebral blood flow derived from arterial spin labelling as a biomarker of clinical outcome following endovascular therapy**

M. R. Hernandez Petzsche[1], G. Hoffmann[1], C. Zimmer[1], C. Maegerlein[1], T. Boeckh-Behrens[1], S. Wunderlich[1], S. Kaczmarz[1], M. T. Berndt[1], *N. Sollmann*[2]; [1]Munich/DE, [2]Ulm/DE
*(nico.sollmann@tum.de)*

**Purpose:** Arterial spin labelling (ASL) enables measuring cerebral blood flow (CBF), and it has been proposed to be sensitive to detect perfusion changes following ischaemic stroke. Post-stroke infarct perfusion has been associated with higher risk of haemorrhagic transformation (HT) and better clinical outcome. This study aimed to evaluate ASL-based CBF in the infarct core as a prognostic biomarker for clinical outcome, and to investigate the relationship between CBF increase and HT risk.
**Methods or Backround:** 111 patients (median age: 74 years, 50 men) underwent cerebral MRI (median 4 days after mechanical thrombectomy for ischaemic stroke due to large vessel occlusion of the anterior circulation) including pseudo-continuous ASL, diffusion-weighted imaging (DWI), and susceptibility-weighted imaging (SWI). %CBF increase was calculated within the segmented DWI-positive infarct territory (in relationship to the unaffected contralateral side). Functional independence was defined as a modified Rankin Scale (mRS) 0-2 at 90 days post-stroke. National Institutes of Health Stroke Scale (NIHSS) scores were determined at admission.
**Results or Findings:** In univariate analysis, age (odds ratio (OR)=0.97, p=0.03), pre-stroke mRS (OR=0.36, p<0.001), NIHSS at admission (OR=0.93, p=0.04), Alberta Stroke Program Early Computed Tomography Score (ASPECTS) at admission (OR=1.26, p=0.03), complete recanalisation (OR=2.95, p=0.03), DWI-positive infarct volume on post-treatment MRI (OR=0.994, p=0.002), and %CBF increase (OR=1.009, p=0.04) were associated with functional independence. In multivariate regression, %CBF increase (OR=1.01, p=0.02), pre-stroke mRS (OR=0.30, p<0.001), and infarct volume (OR=0.99, p=0.001) were significantly associated with functional independence. Post-stroke infarct %CBF increase was comparable between patients with and without HT (median %CBF increase 20.6 in patients without HT and 9.8 in patients with HT, p=0.45).
**Conclusion:** ASL-derived %CBF increase from DWI-positive infarct territories may independently predict functional independence. Yet, infarct %CBF increase was not significantly associated with increased risk of HT.
**Limitations:** No limitations were identified.
**Ethics committee approval:** This study was approved by the Technical University of Munich Ethics Committee.
**Funding for this study:** No funding was received for this study.
**Author Disclosures:**
Stephan Kaczmarz: Employee: Philips GmbH Market DACH, Hamburg, Germany
Maria Teresa Berndt: Nothing to disclose
Silke Wunderlich: Nothing to disclose
Claus Zimmer: Nothing to disclose
Moritz R. Hernandez Petzsche: Nothing to disclose
Nico Sollmann: Nothing to disclose
Gabriel Hoffmann: Nothing to disclose
Christian Maegerlein: Nothing to disclose
Tobias Boeckh-Behrens: Nothing to disclose

### RPS 711-7

**Automated MRI-based basal ganglia and thalamus assessment at the acute-subacute phase after middle cerebral cortical stroke can help predict the 3-month mRS shift**

*S. Zenkhri*, M. Bénédicte, R. Corredor-Jerez, D. Strambo, P. Michel, V. Dunet; Lausanne/CH
*(s.zenkhri@gmail.com)*

**Purpose:** The purpose of this study was to evaluate the prognostic value of individual volumetric and ADC variations of the basal ganglia (BG) and thalamus during the acute-subacute phases following a cortical middle cerebral artery (MCA) stroke to predict the modified Rankin scale (mRS) at 3 months.
**Methods or Backround:** In this retrospective single-centre study, 153 patients (69.3±15.3 years, 85 males) admitted for MCA cortical stroke at the acute-subacute phase (<21 days) from January 2018 to October 2020 were included. MR performed on a 3T (MAGNETOM Vida, Siemens) with DWI and T1-MP-RAGE sequences were used for analysis. T1-MP-RAGE images were processed with the MorphoBox© research application to automatically segment BG and thalamus and compute Z-scores considering the confounding effects of age and sex. ADC values were extracted from the co-registration between ADC map and segmented T1-MP-RAGE. The differences between ipsilateral and contralateral values to stroke, and asymmetric index were computed. Stroke volume was manually segmented on DWI. Multivariate regression analysis was performed to search for predictors of 3-months mRS.
**Results or Findings:** The mean stroke volume was 8.3±20.5ml. The mean delay between stroke onset and MRI evaluation was 56.5±78.5 hours. The Z-score difference between thalami correlated with the core volume (β=0.29, p<0.001) and delay from stroke onset (β=0.18, p=0.038). The ADC mean value difference between thalami correlated with the delay from stroke onset (F=6.55, p=0.0003). Asymmetric Z-score putamen index (F=5.50, p=0.021) and ADC thalamus index (F=5.49, p=0.021) independently predicted mRS shift at 3 months.
**Conclusion:** At the acute-subacute phase of MCA cortical stroke, a reduced thalamic and BG volume and low ADC on the stroke side correlate with core volume and delay from onset. They could additionally help to predict mRS shift at 3 months.
**Limitations:** This is a retrospective monocentric analysis.
**Ethics committee approval:** All procedures performed in studies involving human participants were in accordance with the ethical standards of the institutional and/ or national research committee and with the 1964 Helsinki declaration and its later amendments or comparable ethical standards. All collected data were anonymised to comply with national ethical guidelines and laws. Therefore, patients' consent was waived.
**Funding for this study:** The authors of this research declare no conflict of interest, and no funding was received for this study.
**Author Disclosures:**
Vincent Dunet: Nothing to disclose
Marechal Bénédicte: Consultant: R&D Integrated Decision Support Team Manager | Advanced Clinical Imaging Technology at Siemens Healthineers. Equipment Support Recipient: MorphoBox© image processing
Ricardo Corredor-Jerez: Consultant: R&D Integrated Decision Support Team Manager | Advanced Clinical Imaging Technology at Siemens Healthineers. Equipment Support Recipient: MorphoBox© image processing
Patrik Michel: Nothing to disclose
Salim Zenkhri: Nothing to disclose
Davide Strambo: Nothing to disclose

### RPS 711-8

**Dual-energy computed tomography can detect subclinical abnormalities of high density in the gyrus in patients after neuro-endovascular treatment**

*S. Suh*, P. Byeongsu, J. H. Han; Seoul/KR
*(sangil.suh@gmail.com)*

**Purpose:** Abnormal high density in the gyrus (AHDG) frequently appears in non-contrast brain CT scans post-neuro-endovascular procedures utilising iodinated contrast. The exact nature of AHDG remains elusive. This study employs Dual-Energy CT (DECT) to explore whether AHDG could represent a 'minor' subarachnoid haemorrhage, confirming that it is iodine, not blood. We aimed to elucidate the nature, incidence, and risk factors of AHDG observed in DECT scans post-embolisation of unruptured intracranial aneurysms.
**Methods or Backround:** A retrospective review of clinical data from 230 patients who underwent endovascular embolisation for unruptured intracranial aneurysms at our hospital from October 2018 to March 2023 was conducted. Low- and high-energy images were analysed using a 3-material decomposition algorithm focused on brain parenchyma, haemorrhage, and iodine. The osmolality of the contrast agents used in the procedures was 290mOsm/kg, 515mOsm/kg, and 616mOsm/kg. Chi-squared tests and logistic regression analyses were utilised to identify risk factors associated with AHDG.
**Results or Findings:** DECT images of 230 patients were visually inspected, and three physicians independently analysed them, ensuring strong interobserver agreement. Consensus was reached through discussion in cases of discrepancy. Among the patients, 96 (41.7%) exhibited AHDG. Chi-squared tests revealed significant relationships between AHDG and variables like sex, aneurysm location, and usage of higher osmolarity contrast agents. Logistic regression analyses demonstrated significant correlations between AHDG and age (OR 1.03), procedure time (OR 1.02 per minute), aneurysm location (OR 10.15), and the usage of higher osmolarity contrast agents (OR 2.70).
**Conclusion:** DECT effectively detects AHDG in patients with unruptured intracranial aneurysms post-embolisation. The findings underscore the need for further research to unravel the complex relationships between the osmolarity of contrast agents and the incidence of AHDG.
**Limitations:** No limitations were identified.
**Ethics committee approval:** Ethics committee approval is in progress.
**Funding for this study:** No funding was received for this study.
**Author Disclosures:**
Park Byeongsu: Investigator: Data collection
Jung Hoon Han: Investigator: Statistic analysis
Sangil Suh: Investigator: Writing

08:00-09:00 **Research Stage 3**

### Research Presentation Session: Musculoskeletal

### RPS 710

### Sports imaging

**Moderator**
P. Robinson; Leeds/UK
*(philip.robinson10@nhs.net)*

### RPS 710-2

**Assessing cartilage regeneration in a minipig model: cutting-edge MR techniques challenge the gold standard**
K. L. Radke, B. Valentin, A. Müller-Lutz, *M. Frenken*; Düsseldorf/DE

**Purpose:** The purpose of this study was to examine the potential of differently composed MR sequences, including T1, T2, T2* and T1ρ, in assessing cartilage regeneration. These MR techniques were compared to dGEMRIC, providing valuable insight into non-invasive methods for monitoring and assessing cartilage repair after surgery.
**Methods or Backround:** In orthopaedics, assessing articular cartilage regeneration safely and effectively remains a challenge, despite advancements in compositional MRI. We aimed to investigate the efficacy of various compositional MR sequences in quantitatively assessing cartilage and compare them to the gold standard, delayed gadolinium-enhanced MR imaging of cartilage (dGEMRIC). In a preclinical minipig model, we induced standardised osteochondral defects in the proximal femur of 14 animals, divided into four groups: porcine collagen scaffolds with autologous adipose stromal cells (ASC), autologous bone marrow stromal cells (BMSC), unpopulated scaffolds (US), and an untreated defect group. After six months, we employed different MR composition methods, including dGEMRIC, T1, T2, T2*, and T1ρ, using a clinical 3T MR scanner.
**Results or Findings:** Results showed significant differences in the untreated defect group, with lower dGEMRIC values (404.86±64.2 ms, P=0.018) and higher T2 times (44.24±2.75 ms, P<0.001) in the defect region. Conversely, there were no significant differences in dGEMRIC values among the three treatment groups (ASC, BMSC, US), indicating successful cartilage reconstruction.
**Conclusion:** Notably, dGEMRIC proved effective for monitoring cartilage regeneration. Interestingly, T2 imaging emerged as a reliable alternative, offering non-contrast cartilage imaging for future in vivo studies exploring different treatment modalities' cartilage regeneration potential.
**Limitations:** These results have not yet been translated from animal to human.
**Ethics committee approval:** The study was approved by the Ethics Committee of the University of Düsseldorf.
**Funding for this study:** Funding was received from the University of Düsseldorf, Deutsche Arthrose-Hilfe.
**Author Disclosures:**
Birte Valentin: Nothing to disclose
Kar Ludger Radke: Nothing to disclose
Anja Müller-Lutz: Nothing to disclose
Miriam Frenken: Nothing to disclose

### RPS 710-3

**Limited magnetic resonance imaging in acute upper ankle injuries**
*A. Gökduman*; Frankfurt a. Main/DE

**Purpose:** The purpose of this study was to investigate the diagnostic accuracy of limited magnetic resonance imaging for acute ankle injuries in patients with negative plain radiographs.
**Methods or Backround:** Patients that had undergone a routinely performed 3-T MRI scan after acute trauma of the upper ankle with unremarkable plain radiographs (within a two-week interval) were eligible for study inclusion in this retrospective study. For reference, standard definition five radiologists blinded to clinical information evaluated the full MRI study (PD-weighted fat-saturated axial, coronal, and sagittal sequences, and T1-weighted coronal/sagittal sequences) in consensus reading sessions. After six weeks, the studies were independently reevaluated by utilising only PD-weighted fat-saturated coronal sequences. Sensitivity, specificity, and accuracy for injuries of the most relevant bone structures (tibia, fibula, and talus), ligaments (syndesmotic, medial, and lateral ligaments), and tendons (tibialis and peroneal tendons) assessed by application of a binary classification (0=no injury, 1=injury) were the primary metrics of diagnostic performance. Diagnostic confidence was rated by the usage of a 5-point Likert scale (1=low; 5=high).
**Results or Findings:** 137 patients were enrolled in this study. A total of 108 bone injuries, 126 ligament injuries, and 5 tendon injuries were defined by the consensus. Limited MRI showed overall high diagnostic accuracy for assessing bone (sensitivity: 97%, specificity: 93%, accuracy: 95%), ligament (sensitivity: 92%, specificity: 94%, accuracy: 93%), and tendon (sensitivity: 80%, specificity: 100%, accuracy: 90%) injuries in comparison to full MRI (p<0.01). Diagnostic confidence was comparable (scores: 4.37/5 vs 4.41/5; p=0.12) and interreader agreement for limited MRI was high (K=0.83).
**Conclusion:** Limited MRI provides high diagnostic accuracy and confidence for assessing acute trauma injuries of the upper ankle.
**Limitations:** No limitations were identified.
**Ethics committee approval:** This study was approved by vote through the relevant Ethics Board.
**Funding for this study:** No funding was received for this study.
**Author Disclosures:**
Aynur Gökduman: Nothing to disclose

### RPS 710-4

**Surgical correlation of peroneal compartment pathology in 2D and 3D ankle MRIs with interreader reliability**
*M. R. Akram*, N. Rajamohan, A. Chhabra, G. Liu, M. VanPelt, Y. Xi, F. Duarte Silva; Dallas, TX/US
*(muhammad.akram@utsouthwestern.edu)*

**Purpose:** Peroneal compartment pathology (PCP) is a common yet underdiagnosed source of lateral ankle pain, often requiring clinical examination and imaging for diagnosis. Current literature lacks research on 3D MRI accuracy for PCP. We investigated interreader reliability (IRR) for PCP using parallel 2D and 3D ankle MRIs, hypothesising higher IRR and accuracy with this combined analysis than with 2D alone.
**Methods or Backround:** We retrospectively screened 634 patients with 3-Tesla ankle MRIs, including multiplanar proton density DIXON and isotropic 3D fast-spin echo acquisitions (3DFSE), and excluded patients with unretrievable operative reports or prior peroneal compartment surgery. Our final sample comprised 27 scans from 26 patients who underwent peroneal surgery at least six months post-MRI. Two radiologists (14- and 1-year post-musculoskeletal fellowship) analysed scans for peroneus brevis (PB) and peroneus longus (PL) tenosynovitis, tendinopathy, and tears. We evaluated IRR using intraclass correlation coefficient (ICC) and assessed sensitivity (Se) and specificity (Sp) against surgical results.
**Results or Findings:** IRR was good for PB/PL tendons (ICC=0.735-0.724, respectively) and common peroneal tenosynovitis (ICC=0.630), and was fair for PL/PB tenosynovitis (ICC=0.573-0.541, respectively). Surgical reports identified PB partial/split tears in 51.9% (MRI Se=63.6-83.3% and Sp=66.7-83.3%) and complete tears in 7.4% (MRI Se=25-100% and Sp=88.5-100%) of cases. Regarding the PL tendon, surgical reports identified 7.4% partial/split tears (MRI Se=0-28.6% and Sp=80-92%) and 3.7% complete tears (MRI Se=33.3-100% and Sp=93.2-100%). The readers identified an average of 31.5% PL tenosynovitis, 27.8% PB tenosynovitis, and 46.3% common peroneal tenosynovitis cases.
**Conclusion:** MR identification of PCP had moderate IRR with higher specificity than sensitivity. Additional 3D sequencing did not improve accuracy compared to prior literature.
**Limitations:** Surgical reports prioritised tears over tendinopathy details, which led to the merger of normal and tendinopathy subgroups and prevented us from analysing accuracy for tendinopathy.
**Ethics committee approval:** The study was retrospective.
**Funding for this study:** No funding was received for this study.
**Author Disclosures:**
Flavio Duarte Silva: Nothing to disclose
Avneesh Chhabra: Research/Grant Support: Research grant from Imaging Biopsy Lab, Inc.; Consultant: ICON Medical and Treace Medical Concepts, Inc.; Advisory Board: Medical advisor for Imaging Biopsy Lab, Inc.; Speaker: Siemens; Other: Receives royalties from Jaypee and Wolters
Naveen Rajamohan: Nothing to disclose
Muhammad Rubeel Akram: Nothing to disclose
George Liu: Nothing to disclose
Michael VanPelt: Nothing to disclose
Yin Xi: Nothing to disclose

### RPS 710-5

**Shear-wave elastography for the evaluation of tendinopathies: a systematic review and meta-analysis**
*M. Basile*[1], S. Gitto[1], C. Messina[1], Ž. Snoj[2], S. Glanola[1], S. Bargeri[1], G. Castellini[1], L. M. Sconfienza[1], D. Albano[1]; [1]Milan/IT, [2]Ljubljana/SI
*(mariachiara.basile@unimi.it)*

**Purpose:** The purpose of this study was to compare pathologic and healthy tendons using shear-wave elastography (SWE).
**Methods or Backround:** A systematic review with meta-analysis was conducted by searching Pubmed and EMBASE up to September 2022. Prospective, retrospective, and cross-sectional studies that used SWE in the assessment of pathologic tendons versus control were included. Our primary outcomes were SWE velocity (m/s) and stiffness (kPa). Methodological quality was assessed by the methodological index for non-randomized studies (MINORS). We used the mean difference (MD) with corresponding 95% confidence intervals (CIs) to quantify effects between groups. We performed sensitivity analysis in case of high heterogeneity, after excluding poor quality studies according to MINORS assessment. We used Grades of Recommendation, Assessment, Development and Evaluation to evaluate the certainty of evidence (CoE).
**Results or Findings:** Overall, 16 studies with 676 pathologic tendons (188 Achilles, 142 patellar, 96 supraspinatus, 250 mixed) and 723 control tendons (484 healthy; 239 contralateral tendon) were included. Five studies (31.3%) were judged as having poor methodological quality. Shear-wave velocity and stiffness meta-analyses showed high heterogeneity. According to a sensitivity analysis, pathologic tendons had a lower shear wave velocity (MD −1.69m/□; 95% CI -1.85; -1.52; n=274; I2 50%) compared to healthy tendons with very low CoE. Sensitivity analysis on stiffness still showed high heterogeneity.
**Conclusion:** Pathological tendons may have reduced SWE velocity compared to controls. Future robust high-quality longitudinal studies and clear technical indications on the use of this tool are needed.
**Limitations:** We included different a study design in order to obtain more literature, however this choice has incorporated some methodological heterogeneity.
**Ethics committee approval:** Institutional Review Board approval was not required because no patient data was used for this study.
**Funding for this study:** No funding was received for this study.
**Author Disclosures:**
Silvia GIanola: Nothing to disclose
Silvia Bargeri: Nothing to disclose
Carmelo Messina: Nothing to disclose
Salvatore Gitto: Nothing to disclose
Luca Maria Sconfienza: Nothing to disclose
Mariachiara Basile: Nothing to disclose
Greta Castellini: Nothing to disclose
Žiga Snoj: Nothing to disclose
Domenico Albano: Nothing to disclose

### RPS 710-6

**Muscle mapping evaluation in athletes with myocardial inflammation**
A. Lupi, D. Creazzo, *M. Tordin*, S. Zinato, E. Quaia, A. Pepe; Padua/IT
*(tordinmattia.me@gmail.com)*

**Purpose:** In the context of magnetic resonance imaging (MRI), modern-day mapping techniques allow for precise and quantitative tissue characterisation and are increasingly widespread. In the musculoskeletal field, MRI finds extensive application thanks to its high soft tissue contrast. In specific clinical scenarios, T1/T2 mapping can be used as a valuable tool to provide skeletal muscle tissue characterisation. To the best of our knowledge, correlation between myocardial and skeletal muscle involvement has never been explored. The aim of our study is to evaluate muscle mapping in athletes with myocardial inflammation.
**Methods or Backround:** We included competitive athletes consecutively referred to our center for a Cardiovascular Magnetic Resonance (CMR) (1.5T Siemens), that showed myocardial inflammation. T1 and T2 mapping values were extracted from single Regions of Interest (ROIs) manually traced always in the same back muscles included in the Field of View (latissimus dorsi or serratus anterior), both on MOLLI and T2p-SSFP images, and compared with reference values obtained in our Institute from fifty healthy volunteers stratified by age and sex. Image analysis was performed using cvi42.
**Results or Findings:** Fifteen patients; twelve males, mean age 50.1±14.7 years, with myocardial inflammation assessed with parametric and/ or nonparametric techniques, were included. Normal T1 and T2 mapping muscle values were found in four (26.6%) patients (mean T1 852.3±10.1ms and T2 38.8±1.5ms). In one female patient (6.7%) with peri-myocarditis by CMR, T2 mapping values turned out to be pathological (44.2ms, n.v. 30-42ms) and in ten male patients (66.6%) T1 mapping values turned out to be pathological (n.v. 762-853ms).
**Conclusion:** These preliminary results show that muscle oedema could be found in subjects with myocardial inflammation. Further studies on larger population and prognostic data are needed to better understand increased T1 mapping values significance.
**Limitations:** No limitations were identified.
**Ethics committee approval:** This study was approved by the Azienda Ospedale Università Padova.
**Funding for this study:** No funding was received for this study.
**Author Disclosures:**
Stefania Zinato: Nothing to disclose
Amalia Lupi: Nothing to disclose
Alessia Pepe: Nothing to disclose
Dario Creazzo: Nothing to disclose
Emilio Quaia: Nothing to disclose
Mattia Tordin: Nothing to disclose

### RPS 710-7

**Rethinking the anatomical variants role in peroneal tendinopathy**
O. D. Rodriguez Martinez, *P. S. Perez*, M. E. Scherer, J. E. Prieto Terán, N. E. Alanis, I. Bidart, S. Centofante, E. Piedra, O. Montaña; Buenos Aires/AR
*(pamemeli14@gmail.com)*

**Purpose:** The study purpose was to assess the anatomical variants prevalence of peroneal tendon tract in patients with external retromalleolar pain and define their association with the development of tendinopathies.
**Methods or Backround:** A retrospective review of 1929 ankle MRI scans (Philips 1.5T) was performed between January and December 2022. We found 117 patients with peroneal tendon pathology in the context of external ankle pain. In these, the prevalence of convex/ flat retromalleolar sulcus (CFRS), hypertrophic peroneal tubercle of the calcaneus greater than 5mm (HPTC), peroneus quartus muscle (PQM), and os peroneum presence were evaluated as anatomical variants that could be predisposed to the development of pathology tendinous. Cases with fractures in the area of interest, people under 15 years old and over 80 years old, and studies with suboptimal imagen quality and/ or with acquisition artifacts such as magic angle were excluded from the study.
**Results or Findings:** In the 117 patients studied, it was found that 69% (n=81) had at least one anatomical variant in the peroneus tendons tract up to their distal insertions, with the loss of the concavity of the retromalleolar sulcus being the most frequent (65 cases). HPTC was the second most common variant, with eleven cases, and finally four cases of PQM were found. Only one case of os peroneum was found in analysed patients, probably due to the method used (MRI).
**Conclusion:** We found a high prevalence of SRCP and TPCH in association with peroneal tendinopathies. This demonstrates the importance of a systematic evaluation of these anatomical variants as possible causes of external retromalleolar pain, and its search and mention in routine ankle study is recommended.
**Limitations:** No limitations were identified.
**Ethics committee approval:** The study is retrospective, so approval is not needed.
**Funding for this study:** This study was funded by DIM.
**Author Disclosures:**
Ignacio Bidart: Nothing to disclose
Nahir Evangelina Alanis: Nothing to disclose
Matías Enrique Scherer: Nothing to disclose
Oscar Montaña: Nothing to disclose
Santiago Centofante: Nothing to disclose
Ezequiel Piedra: Nothing to disclose
Jimmy Eduardo Prieto Terán: Nothing to disclose
Oscar Danilo Rodriguez Martinez: Nothing to disclose
Pamela Soledad Perez: Nothing to disclose

### RPS 710-8

**Patients at risk of bone non-union in distal radius fractures: value of dual-energy CT-derived metrics**
*L. D. Grünewald*, V. Koch, C. Booz, S. Martin, S. Mahmoudi, T. Vogl; Frankfurt a. Main/DE

**Purpose:** Distal radius fractures (DRF) are among the most diagnosed fractures in emergency departments, and bone non-union can lead to persistent pain and functional limitations. Despite the clinical implications, there is currently no established approach to identify patients at risk of bone non-union. The purpose of this study was to evaluate various metrics derived from routine CT scans of the distal radius to identify patients at risk of bone non-union.
**Methods or Backround:** The distal radius of patients who underwent dual-energy CT (DECT) between 01/2016 and 08/2021 was retrospectively analysed. Cortical HU, trabecular HU, cortical thickness, and DECT-based bone mineral density (BMD) were obtained from all examinations. Patient files and follow-up images of patients were examined for the occurrence of bone non-union. Receiver-operating characteristic (ROC) analysis identified AUC values for BMD, HU values, and cortical thickness, and logistic regression models were used to evaluate their associations with the occurrence of bone non-union.
**Results or Findings:** 263 patients (median age 52 years; interquartile range 36–64) were included in this study. ROC curve analysis demonstrated a significantly higher AUC value for DECT-derived BMD compared to cortical HU, trabecular HU and cortical thickness (0.83 vs. 0.63, 0.60 and 0.56, respectively; p<0.01). Logistic regression models confirmed a significant association of lower DECT-derived BMD with the occurrence of bone non-union (Odds Ratio, 0.93; p<0.001), but not of cortical HU, trabecular HU or cortical thickness (p>0.05 for all values, respectively).
**Conclusion:** CT examinations of the distal radius obtained in clinical routine may serve as a useful tool in identifying patients at risk of developing bone non-union. Dual-energy CT-derived bone mineral density is a better predictor of bone non-union compared to cortical HU, trabecular HU, and cortical thickness.

**Limitations:** The study is limited by the possibility of preselection bias, as well as the limiting of research to dual-energy CT scans.
**Ethics committee approval:** Written consent was waived due to the retrospective nature of the study.
**Funding for this study:** No funding was received for this study.
**Author Disclosures:**
Simon Martin: Nothing to disclose
Christian Booz: Nothing to disclose
Thomas Vogl: Nothing to disclose
Vitali Koch: Nothing to disclose
Scherwin Mahmoudi: Nothing to disclose
Leon David Grünewald: Nothing to disclose

### RPS 710-9

**Diagnostic accuracy of a fast deep learning-based MRI protocol in diagnosing meniscal and ligament tears of the knee: comparison with standard MRI protocol**
*F. Lombardo*, C. Longo, E. Oliboni, S. Crosara, G. Sala, T. Mignolli, G. Foti; Negrar/IT
*(fabio.lombardo@me.com)*

**Purpose:** The purpose of this study was to evaluate the diagnostic accuracy of a deep learning- (DL) based protocol in identifying meniscal and ligament tears of the knee in comparison to standard magnetic resonance imaging (MRI) protocol.
**Methods or Backround:** We included 88 consecutive patients (46 males and 42 females, mean age of 55.2) between April and May 2023. All patients underwent standard MRI protocol, including multiplanar 3 mm T1W, STIR and DP fat-saturated sequences (acquisition time 18 minutes) and a fast protocol acquired with DL sequences (acquisition time 5 minutes) the same day. Four radiologists (21, 16, 13 and 5 years of experience, respectively), blinded to standard MRI protocol, evaluated the presence of meniscal and ligament tears on DL protocol images. Standard MRI images served as standard of reference (consensus reading of two additional experienced MSK radiologists). Diagnostic accuracy values of DL protocol (qualitative assessment) and inter-observer agreement were calculated.
**Results or Findings:** Standard MRI revealed the presence of meniscal tear in 45/88 patients (51.1%) and ligament tears in 37/88 cases (42.0%). Sensitivity, specificity and overall accuracy of DL protocol for meniscal and ligaments tear were 97.7% (44/45), 100% (43/43) and 98.9% (87/88), and 100% (37/37), 98.0% (50/51) and 98.9% (87/88), respectively. The diagnostic accuracy values of DL protocol were similar to those of standard protocol (p=0.43). Conversely, the acquisition time for DL protocol was significantly lower if compared to that of standard protocol (p=0.001). The inter-observer agreement was near perfect (k=0.96).
**Conclusion:** Despite a significant reduction of acquisition time, the DL MRI protocol of the knee showed similar diagnostic accuracy value compared to standard MRI protocol.
**Limitations:** The study is limited by its small sample size.
**Ethics committee approval:** This study was approved by the relevant Institutional Research Board.
**Funding for this study:** No funding was received for this study.
**Author Disclosures:**
Giuseppe Sala: Nothing to disclose
Thomas Mignolli: Nothing to disclose
Chiara Longo: Nothing to disclose
Fabio Lombardo: Nothing to disclose
Stefano Crosara: Nothing to disclose
Giovanni Foti: Nothing to disclose
Eugenio Oliboni: Nothing to disclose

**08:00-09:00 Research Stage 4**

### Research Presentation Session: Interventional Radiology

### RPS 709

### Novel treatment options for malformations and benign tumours

**Moderator**
R. Dežman; Ljubljana/SI
*(rok.dezman@gmail.com)*

### RPS 709-2

**Polidocanol sclerotherapy of venous malformations in children- efficacy, safety, and predictors of outcome and complications**
*N. Schmitt*, J. Lorenz, S. Hohenstatt, F. Ruping, P. Günther, M. Bendszus, M. Möhlenbruch, D. F. Vollherbst; Heidelberg/DE

**Purpose:** Venous malformations (VMs) represent the most common type of congenital vascular malformations and feature a wide range in size, location, appearance, and clinical symptoms. Percutaneous sclerotherapy (PS) using polido-canol is an established treatment, aiming at an improvement of the clinical symptoms. The influence of patient- and disease-specific factors on treatment success and complications in children has not been systematically addressed so far.
**Methods or Backround:** All children (<18 years) with VMs who were treated with PS using polidocanol between 01/2011 and 11/2021 in our institution were included. Demographics, clinical data and radiological features were analysed and the influence of patient- and VM-related factors on the outcome and complications according to the CIRSE classification were investigated.
**Results or Findings:** A total of 83 children were included with a symptom improvement in 54.0%, stable symptoms in 34.9%, and worsening in 11.1%. The overall complication rate was 13.3% with an absence of permanent complications. Complications were more frequently in children after the treatment of extremity-located VMs (p=0.01; complication rate 12.2% for VMs of the extremities and 1.6% for VMs in other locations). This suggests that VMs located along body parts with a more intense movement may increase the probability for periprocedural complications with a further impact by the increased venous pressure in the lower extremities. There was no significant factor affecting the clinical outcome with a tendency for a worse outcome in children suffering from VM-related swelling (p=0.11).
**Conclusion:** PS of VMs using polidocanol can be an effective treatment option in children with an acceptable safety profile. Complications can be more frequently expected for VMs of the extremities and should be considered for therapy planning.
**Limitations:** This was a retrospective single-centre analysis, which included a disproportionate incidence of head and neck VMs and assessed outcomes subjectively by the patients themselves.
**Ethics committee approval:** This study was approved by the Ethics Committee of the Medical Faculty at Heidelberg University.
**Funding for this study:** No funding was received for this study.
**Author Disclosures:**
Julia Lorenz: Nothing to disclose
Martin Bendszus: Nothing to disclose
Sophia Hohenstatt: Nothing to disclose
Dominik F. Vollherbst: Nothing to disclose
Markus Möhlenbruch: Nothing to disclose
Niclas Schmitt: Nothing to disclose
Fabian Ruping: Nothing to disclose
Patrick Günther: Nothing to disclose

### RPS 709-3

**Long-term outcomes of hand arteriovenous malformation management: single referral vascular anomalies centre over 25 years' experience**
*Y. D. Lee*, S. Y. Lee, K. B. Park, D-I. Kim, S. S. Yang, Y. S. Do; Seoul/KR
*(science0914@gmail.com)*

**Purpose:** Hand arteriovenous malformations (AVMs) are extremely difficult to manage for their functional importance and cosmetic problems. A single centre retrospective study was conducted to identify long-term outcomes of multidisciplinary team management of hand AVM.
**Methods or Backround:** Retrospective vascular anomalies centre data was reviewed from 1995 to 2022. Patient's demographics, Schobinger's AVM stage, sclerotherapy details, surgical history, and adverse events after sclerotherapy were reviewed.

**Results or Findings:** 150 hand AVM patients visited our hospital from 1995 to 2022. The mean age was 33 years (range, 1-75 years) and 91 of 150 were female. 44 were Schobinger stage II and 106 were stage III. 101 patients (67%) received a total of 319 sessions of percutaneous sclerotherapy. Angiographic devascularisation rates after sclerotherapy were as follows: 16 had 100% devascularisation, 30 had over 90% devascularisation, 34 showed 50-90% devascularisation, 15 showed 0-50% of devascularisation, and six showed aggravation. 123 of 319 of (39%) had sclerotherapy-related adverse events (112 were minor and 11 were major adverse events). 15 of 101 (15%) patients eventually received amputation surgery after mean 1618 days (range, 3-5444 days) after sclerotherapy (sclerotherapy-related necrosis (n=3) and delayed amputation (n=12)). 13 of 150 (9%) of patients received primary surgical amputation for ulcer or bleeding (all Schobinger stage 3). The remaining 36 patients (34%) followed without any procedure.
**Conclusion:** In our study, 67% of hand AVM patients (101/150) were initially treated with sclerotherapy and 9% of patients (13/150) by surgery. 80% of patients showed response to sclerotherapy (over 50% devascularisation rate) with a major complication rate of 7%. However, 15% of patients eventually received amputation surgery after sclerotherapy.
**Limitations:** The limitations of the study are its retrospective nature and single-centre experience.
**Ethics committee approval:** Ethical approval was not sought for the present study because this was a retrospective observational study conducted at the Samsung Medical Centre.
**Funding for this study:** No funding was received for this study.
**Author Disclosures:**
Dong-Ik Kim: Nothing to disclose
Shin Seok Yang: Nothing to disclose
Kwang Bo Park: Nothing to disclose
Sang Yub Lee: Nothing to disclose
Young Soo Do: Nothing to disclose
Yong Deok Lee: Nothing to disclose

### RPS 709-4

**Analysis of adverse events after peripheral arteriovenous malformation embolisation: single Korean referral vascular anomalies centre experience**
*J. Ban*[1], S. Y. Lee[1], K. B. Park[1], D-I. Kim[1], S. S. Yang[1], Y. S. Do[2]; [1]Seoul/KR, [2]Gyeonggi/KR
*(banjiyoon7536@gmail.com)*

**Purpose:** Peripheral arteriovenous malformations (AVMs) are extremely difficult to manage for their functional importance and cosmetic problems. This single-centre retrospective study was conducted to identify factors that contribute to adverse events (AEs) after embolisation of AVMs using percutaneous sclerotherapy.
**Methods or Backround:** A retrospective review of patients who underwent sclerotherapy of AVMs from 1997 to 2022 was performed. 1273 procedures in 444 patients (median age: 28.0 years; interquartile range: 18.0-38.0 years) were evaluated. The AVM characteristics and details of AEs were analysed. According to Society of Interventional Radiology guidelines, AEs were divided into minor and major. Univariate and multivariate analyses were performed to find factors associated with AEs.
**Results or Findings:** Ethanol (94.2% of procedures, mean 26.2ml per session) and coils (33.4% of procedures, mean 31.4 coils) were mainly used for sclerotherapy. Upper extremity AVMs were most common (39.6%), followed by lower extremity (33.9%), abdomen-pelvis (22.4%), and thorax and neck (4.1%). 297 of 1273 procedures (23.3%) had sclerotherapy-related adverse events (261 were minor and 36 were major adverse events). Skin damage and transient nerve injury were most frequent in AEs. Coil used OR, 0.3672; 95% CI, 0.2607-0.5172, P<0.0001), diffuse AVMs (OR, 0.6837; 95% CI, 0.5095-0.9174; P=0.0112), body AVMs (OR, 0.522; 95% CI, 0.3685-0.7393; P=0.0003), and skin non-involvement (OR, 0.5741; 95% CI, 0.4268-0.7721; P=0.0012) were negatively associated with AEs.
**Conclusion:** In our ethanol and coil based AVM sclerotherapy, 297 AEs were developed after 1273 sessions of AVM sclerotherapy (23.3%). Most AEs were minor. Use of coil, diffuse involvement, body AVM and skin non-involvement showed protective effect on AEs.
**Limitations:** Our study was limited to a single centre and based on retrospective review of previous procedures.
**Ethics committee approval:** Ethical approval was not sought for the present study because this was a retrospective study conducted at Samsung Medical Center with an observational nature.
**Funding for this study:** No funding was received for this study.
**Author Disclosures:**
Dong-Ik Kim: Nothing to disclose
Shin Seok Yang: Nothing to disclose
Kwang Bo Park: Nothing to disclose
Sang Yub Lee: Nothing to disclose
Jiyoon Ban: Nothing to disclose
Young Soo Do: Nothing to disclose

### RPS 709-5

**Ultrasound-guided ethanol ablation of medial and lateral neck cysts**
*K. M. Duvivier*, C. Peeters, S. Eerenstein, P. De Graaf; Amsterdam/NL
*(k.duvivier@amsterdamumc.nl)*

**Purpose:** The purpose of this study was to evaluate the treatment effect, cosmetic aspects, and the quality of life (QOL) of ethanol ablation (EA) as a minimally invasive treatment of TGDC and BCC neck cysts.
**Methods or Backround:** Between February 2022 and July 2022, 21 patients with either a TGDC or BCC neck cyst were prospectively included in this study. EA was performed in 21 patients. Treatment was performed following a standard protocol including ultrasound (US) guided cyst aspiration and rinsing with saline followed by injection of 96% ethanol. Patients received a questionnaire both before EA and after three months of follow-up. Therapeutic outcomes, including volume reduction ratio, therapeutic success rate, cosmetic results, and complications were evaluated.
**Results or Findings:** Twenty-one patients were included who underwent EA for TGDC (N=12, 57%) and BCC (N=9, 43%). The mean age was 41.1 years old (IQR:33.5-51.5) with 6 males (29%) and 15 females (71%). Of the 21 eligible patients, 20 completed the questionnaire pre-EA and post-EA. The mean volume of the cyst prior to treatment was 10.9cc (IQR: 17.01-2.79) and after EA- treatment was 1.20cc (IQR: 0.11-1.42), wich results in a mean volume reduction of 81.86% (IQR:83.50-98.00). Two patients required a second treatment. No significant complications occurred during follow-up. 89% (N=16/18) Of the patients show an overall improvement of the QOL. The VAS-score during the procedure had a mean of 1.4 (N=21) and after the procedure of 0.7 (N=21) on a 0-10 pain scale.
**Conclusion:** EA is an effective, safe, and minimally invasive method of treating TGDC and BCC neck cysts . The procedure is well tolerated and can be repeated, when necessary, without complications or hindering any surgery.
**Limitations:** The limitations of this current study are the small sample size and the short follow-up period.
**Ethics committee approval:** No information provided by the submitter.
**Funding for this study:** No funding was received for this study.
**Author Disclosures:**
S. Eerenstein: Nothing to disclose
Pim De Graaf: Nothing to disclose
Caroline Peeters: Nothing to disclose
Katya M. Duvivier: Nothing to disclose

### RPS 709-6

**Transarterial embolisation of renal angiomyolipomas: a prophylactic treatment for reducing myogenic components**
*L. Bracciscshi*[1], L. Bellini[2], F. Modestino[1], L. Bartalena[1], M. C. Galaverni[1], M. Renzulli[1], A. Cappelli[1], L. Bianchi[1], C. Mosconi[1]; [1]Bologna/IT, [2]Rome/IT
*(lorenzo.bracciscshi@studio.unibo.it)*

**Purpose:** Renal angiomyolipomas (AMLs) are benign hamartomatous tumors containing elements of smooth muscle, fat and vascular tissue. 70% of all AMLs are sporadic and tend to be solitary. The most concerning complication of AMLs is the risk of bleeding which is linked to dimensions and can lead to retroperitoneal haemorrhage. Selective embolisation of the renal artery has been approved as a prophylactic treatment option. Nevertheless, there is still a lack of literature. The aim of this study was to evaluate the short and long-term efficacy of AML embolisation. The percentage of fat and myogenic component was calculated in each AML and compared in volume before and after the embolisation of each component was evaluated.
**Methods or Backround:** A total of 26 AMLs were treated between May 2015 and February 2023. All lesions were completely embolised according to the angiographic criteria of vascular stasis and the absence of arterial feeders. Cyanoacrylate glue was the most commonly used embolic agent.
**Results or Findings:** The efficacy of embolisation was determined over a mean follow-up period of 30.5 months. Mean lesion size at diagnosis was 56.85mm with a pre-treatment mean percentage of myogenic component around 60%. Embolisation of the renal AMLs was technically successful for all 26 lesions. Long-term follow-ups showed a mean diametre reduction of 23.57mm with a 50% reduction in the volume of the myogenic component.

**Conclusion:** Transarterial embolisation is effective in reducing the volume of the myogenic component of AMLs, therefore lowering the chance of haemorrhagic events while preserving renal function. No major complications were observed demonstrating the highly safe profile of the procedure.
**Limitations:** This was a retrospective study.
**Ethics committee approval:** No information provided by the submitter.
**Funding for this study:** No funding was received for this study.
**Author Disclosures:**
Francesco Modestino: Nothing to disclose
Lorenzo Bianchi: Nothing to disclose
Matteo Renzulli: Nothing to disclose
Cristina Mosconi: Nothing to disclose
Luigi Bellini: Nothing to disclose
Laura Bartalena: Nothing to disclose
Alberta Cappelli: Nothing to disclose
Lorenzo Braccischi: Nothing to disclose
Maria Cristina Galaverni: Nothing to disclose

**09:30-11:00 Research Stage 1**

### Research Presentation Session: Abdominal Viscera & GI Tract

### RPS 801

### Liver steatosis and fibrosis quantification

**Moderator**
T. V. Bartolotta; Palermo/IT
*(tommasovincenzo.bartolotta@unipa.it)*
**Author Disclosures:**
Tommaso Vincenzo Bartolotta: Consultant: Samsung, GE; Research Grant/Support: Samsung, GE, Bracco, Philips; Speaker: Samsung, GE, Bracco

### RPS 801-2

**Gravitational 2D/3D MR-elastography vs pneumatic 2D MR-elastography: prospective evaluation of a novel system**
*V. Koch*[1], L. D. Grünewald[1], J. Gotta[1], S. Mahmoudi[1], C. Booz[1], R. Hammerstingl[1], O. Darwish[2], T. Vogl[1], R. Sinkus[3]; [1]Frankfurt a. Main/DE, [2]London/UK, [3]Paris/FR

**Purpose:** Magnetic resonance elastography (MRE) can quantify tissue biomechanics non-invasively and represents a promising technique for assessing fibrosis in nonalcoholic fatty liver disease (NAFLD). This research presents the preliminary results of a prospective study designed to investigate the value of 2D/3D-MRE utilising the gravitational concept compared to the current product solution (2D-MRE Resoundant).
**Methods or Backround:** Thirty-two participants with different stages of NAFLD were examined twice at 60Hz (Aera, 1.5T, Siemens Healthineers, Germany): firstly, using the Resoundant system (2D-MRE, SE-EPI sequence, 11secs BH) and secondly, using the gravitational transducer approach (2D-MRE and 3D-MRE, GRE sequence, TE=9.2ms (in-phase) and fractional motion encoding at 30mT/m, 14secs BH). While 2D-MRE provides solely the magnitude of the complex shear modulus |G*|, 3D-MRE allows for the additional quantification of both real and imaginary parts of G*. Data extraction and analysis was performed twice by two readers.
**Results or Findings:** We found a high level of correlation between the 2D-MRE acquisitions obtained using both the Resoundant MRE system and the gravitational transducer approach (r≥0.83 (95% CI, 0.69 to 0.92), p<0.001), although there was a clear bias with 2D-MRE overestimating stiffness values. For markers indicative of liver damage, GOT levels were below 30 for |G*|<4kPa, while for |G*|>4kPa, GOT levels were above 30 in a binary fashion. Interestingly, the 3D-MRE approach showed different correlations depending on the level of GOT expression: for GOT<30 wave attenuation showed a clear trend wrt GOT (r=-0.27, P=0.37), while for GOT>30 viscosity correlated very well to GOT (r=0.61, P=0.047). Viscosity for GOT<30 clustered at low values, similar to attenuation for GOT>30.
**Conclusion:** Gravitational MRE represents a novel method for the sensitive characterisation of patients with NAFLD, with 3D-MRE carrying the potential to provide imaging biomarkers for liver damage.
**Limitations:** This research is based on preliminary data.
**Ethics committee approval:** This study received approval from the relevant local Ethics Committee.
**Funding for this study:** No funding was received for this study.
**Author Disclosures:**
Christian Booz: Nothing to disclose
Omar Darwish: Nothing to disclose
Thomas Vogl: Nothing to disclose
Vitali Koch: Nothing to disclose
Scherwin Mahmoudi: Nothing to disclose
Renate Hammerstingl: Nothing to disclose
Ralph Sinkus: Nothing to disclose
Leon David Grünewald: Nothing to disclose
Jennifer Gotta: Nothing to disclose

### RPS 801-3

**Real-world risk-stratification of patients with chronic liver disease using quantitative magnetic resonance imaging**
N. Nakrour[1], E. Shumbayawonda[2], M. Pansini[3], M. Harisinghani[1], *S. M. Parekh*[2]; [1]Boston, MA/US, [2]Oxford/UK, [3]Lugano/CH

**Purpose:** The objective of this study was to assess the comparative diagnostic value of multiparametric MRI (mpMRI) and magnetic resonance elastography (MRE) in real-world clinical practice for managing suspected chronic liver disease.
**Methods or Backround:** A retrospective analysis of the prospective MR exams of 77 patients referred to tertiary chronic liver disease practices. Patients underwent MRE and mpMRI (LiverMultiScan) as a part of their routine clinical care. MRE measures liver fibrosis with liver stiffness (kPa). LiverMultiScan quantifies liver disease activity (iron-corrected T1, cT1), fat (proton density fat fraction, PDFF), and iron content (T2*). MRE≥3kPa indicates any level of fibrosis; cT1≥800ms and ≥875ms indicate active and high-risk disease, respectively.
**Results or Findings:** 55% (42) of patients were diagnosed with MASLD/MASH and 45% with mixed chronic liver diseases, including alcoholic liver disease, viral hepatitis, hemochromatosis, high ferritin, among others. The majority, 71% (55), had normal liver stiffness (≤3kPa); however, 29% (22) of these had active disease (cT1>800ms), with 14% (11) having elevated cT1 indicative of high-risk disease (cT1>875ms). There was a linear significant correlation between MRE and cT1 (r=0.411, p=0.0004), and those with elevated MRE (MRE>3kPa) had cT1 864±74ms. cT1 correlated with PDFF (r=0.5, p<0.001), but MRE was not (r=-0.055, p=0.65). cT1 was successful in 99% (76) of patients, while MRE was successful in 90% (69).
**Conclusion:** MRE and cT1 provide clinically complementary information on liver health state, with cT1 identifying patients with underlying liver disease activity who are at risk of worse outcomes but have normal liver stiffness. These patients would benefit from more intense clinical management or surveillance to prevent clinical outcomes. Caution should be taken in utilising MRE in patients with suspected high liver iron.
**Limitations:** MRE and cT1 had an unreliable result in one patient. MRE technical failures were in patients with elevated liver iron.
**Ethics committee approval:** This study was approved with code: Protocol #2020P000706.
**Funding for this study:** This study was partially funded.
**Author Disclosures:**
Sanjay M. Parekh: Shareholder: Perspectum Ltd.; Employee: Perspectum Ltd.
Nabih Nakrour: Nothing to disclose
Michele Pansini: Nothing to disclose
Mukesh Harisinghani: Nothing to disclose
Elizabeth Shumbayawonda: Shareholder: Perspectum Ltd.; Employee: Perspectum Ltd.

### RPS 801-4

**MRI hepatic steatosis and sarcopenia in metabolic patients: a correlation prospective study**
*B. Masci*, M. Zerunian, S. Nardacci, F. Pucciarelli, M. Polici, I. Nacci, D. Principessa, D. Caruso, A. Laghi; Rome/IT
*(benedetta.masci93@gmail.com)*

**Purpose:** The purpose of this study was to evaluate the possible correlation between hepatic steatosis and sarcopenia assessed with skeletal muscle index (SMI) at unenhanced MRI of the abdomen in patients with multiple metabolic risk factors.
**Methods or Backround:** 105 patients with metabolic syndrome were prospectively enrolled between October 2022 and June 2023. Each patient underwent 1.5T upper abdomen MRI examination, acquisition protocol included axial proton density fat fraction (PDFF), magnetic resonance elastography (MRE) and axial T1 weighted gradient-echo sequences targeted at the third lumbar vertebra (L3) level. A single radiologist performed quantitative image analysis on a dedicated workstation. Steatosis and myosteatosis estimation expressed as a percentage and liver stiffness (kPa) were collected and grading of steatosis and fibrosis was assigned. The presence of sarcopenia was assessed by segmenting the L3 dual-echo images with ImageJ pixel analysis software and calculating the SMI. Correlations among parameters were assessed using Spearman's rank coefficient using a dedicated software and p<0.05 was considered significant.

**Results or Findings:** PDFF values of hepatic steatosis were found in 65% of patients (6.7±1.8%); liver stiffness resulted in higher compatibility with inflammation values in 35% of patients (2.6kPa±0.2kPa). SMI average values were compatible with sarcopenia in 71% of patients (Males: 41.5 ± 6.8cm ^2/m^2; Females: 33.5 ± 3.2cm ^2/m^2).The statistical analysis showed significant correlation between hepatic steatosis and sarcopenia (P<0.03, rho 0.17). Moreover, by stratifying the cohort for sex, a significant correlation between the steatosis grade and sarcopenia in female patients (P<0.02, rho -0.38) emerges.
**Conclusion:** Patients with metabolic syndrome and hepatic steatosis are correlated with lower SMI values and sarcopenia, and the correlation is stronger in female patients. This method might be a non-invasive, radiation-free and repeatable method for a comprehensive metabolic assessment of the patients at diagnosis and at follow-up.
**Limitations:** We analysed a small population sample and performed a manual segmentation, which was often time consuming. A further limitation is the single vendor technology analysis approach taken.
**Ethics committee approval:** This study was approved by the Institutional Review Board, and written informed consent was obtained for all patients.
**Funding for this study:** No funding was received for this study.
**Author Disclosures:**
Damiano Caruso: Nothing to disclose
Benedetta Masci: Nothing to disclose
Francesco Pucciarelli: Nothing to disclose
Marta Zerunian: Nothing to disclose
Ilaria Nacci: Nothing to disclose
Andrea Laghi: Nothing to disclose
Daniele Principessa: Nothing to disclose
Michela Polici: Nothing to disclose
Stefano Nardacci: Nothing to disclose

### RPS 801-5

**Does meal or water intake affect ultrasound attenuation coefficient estimate of liver fat content?**
*R. G. Barr*[1], G. Ferraioli[2]; [1]Youngstown, OH/US, [2]Pavia/IT
*(rgbarr@zoominternet.net)*

**Purpose:** The purpose of this study was to assess whether meal or water intake may affect the measurement of the ultrasound attenuation coefficient imaging, a parameter that is directly related to liver fat content.
**Methods or Backround:** The study was performed in two centres (in Italy and in the USA). Attenuation coefficients were obtained using the ATI algorithm implemented in the Aplio i-series ultrasound systems (Canon Medical Systems, Japan) by one operator at each centre. Measurements were performed at baseline and 5, 15, 30, and 45 minutes after drinking 500ml of water (group1), or 30, 45, 60, 90, and 120 minutes after eating a meal of about 600 kilocalories (group2). Multilevel generalised estimating equations for repeated measures were used for the statistical analysis to consider the clustered nature of the data.
**Results or Findings:** Twenty-one individuals were enrolled: 11 (10 females; aged 43.7±12.5 years) in Italy and 10 (7 females; aged 59.7±7.1 years) in the USA. At the B-mode ultrasound, six (28.6%) had liver steatosis. The baseline attenuation coefficient values, in dB/cm/mHz were 0.62 (0.11) in group1 and 0.66 (0.12) in group2. There was not any significant difference in attenuation coefficient values at every timing either in group1 or group2. This result did not change even when controlling for sex, age, and skin-to-liver capsules.
**Conclusion:** The measurement of the attenuation coefficient, which is a biomarker of liver steatosis, does not require a fasting state and drinking water does not affect the result.
**Limitations:** Although the findings of this study were statistically significant, the number of participants was small.
**Ethics committee approval:** This study was approved by the Western Reserve Health IRB, Youngstown, Ohio, USA, and the Fondazione IRCCS Policlinico S. Matteo, Pavia, Italy.
**Funding for this study:** No funding was received for this study.
**Author Disclosures:**
Giovanna Ferraioli: Other: Elsevier Publisher - royalties; Speaker: Canon Medical Systems, Fujifilm Healthcare, Mindray Bio-Medical Electronics Co., Philips Healthcare, Siemens Healthineers. Consulting fees: Philips Healthcare (to her) and Siemens Healthineers
Richard G. Barr: Advisory Board: Lantheus MEdical, Hologic; Speaker: Philips Ultrasound, Siemens Healthineers, Canon Medical, Mindray, Hologic; Equipment Support Recipient: Siemens Healthiness, Mindray, Samsung, Philips Ultrasound, Canon Medical, Hologic, Fujifilm; Board Member: International Ultrasound Contrast Society

### RPS 801-6

**Non-invasive assessment of steatosis and fibrosis in patients with risk factors for NAFLD: agreement among quantitative US and multiparametric MRI**
*I. Nacci*, F. Pucciarelli, B. Masci, M. Zerunian, D. De Santis, D. Caruso, A. Laghi; Rome/IT
*(ilaria.nacci@uniroma1.it)*

**Purpose:** The purpose of this study was to assess ultrasound attenuation coefficient (AC) for quantifying liver fat deposit and to evaluate two-dimensional shear wave elastography (2D-SWE) for quantifying liver fibrosis using MRI-proton density fat fraction (MRI-PDFF) and magnetic resonance elastography (MRE) as the references, respectively, in patients with risk factors for NAFLD.
**Methods or Backround:** This prospective study included patients with risk factors for NAFLD (diabetes or metabolic syndrome). Patients with secondary causes of fat deposition were excluded. All participants underwent liver QUS and MRI on the same day, and laboratory tests within 30 days. Based on MRI-PDFF and MRE, we obtained normal liver, liver steatosis and liver fibrosis groups. We examined diagnostic performance of AC and 2D-SWE for detecting liver fat content and stiffness using area under receiver operating characteristic curve (AUC). We also analysed correlations of QUS biomarkers to MRI using Spearman correlation coefficient.
**Results or Findings:** A total of 54 participants were included. Of these participants, 21 (38.9%) had MRI-PDFF≥5%, and 10 (18.5%) had MRE≥2.9kPa. AUC of AC for determining greater than and equal mild steatotic livers was 0.80 (95% confidence interval [CI]: 0.67-0.94). AUC of 2D-SWE for determining greater than and equal F1 liver fibrosis was 0.59 (95% CI: 0.40-0.79). AC had a sensitivity of 71.4% and a specificity of 87.9%, 2D-SWE had a sensitivity of 60% and a specificity of 52.3%. AC did not correlate well with MRI-PDFF in assessing hepatic steatosis (r=0.43), and 2D-SWE did not correlate with MRE in evaluating liver fibrosis (r=0.39).
**Conclusion:** Our preliminary results show that QUS biomarkers are more accurate for screening mild hepatic steatosis than fibrosis in patients with risk factors for NAFLD, but do not accurately discriminate different degrees of steatosis and fibrosis.
**Limitations:** The sample size was small, and the study was single-centred.
**Ethics committee approval:** The study was approved by the relevant Institutional Review Board, and all patients gave informed consent.
**Funding for this study:** No funding was received for this study.
**Author Disclosures:**
Damiano Caruso: Nothing to disclose
Benedetta Masci: Nothing to disclose
Francesco Pucciarelli: Nothing to disclose
Marta Zerunian: Nothing to disclose
Domenico De Santis: Nothing to disclose
Ilaria Nacci: Nothing to disclose
Andrea Laghi: Nothing to disclose

### RPS 801-7

**Ultrasound-based steatosis grading system using 2D-attenuation imaging: an individual patient data meta-analysis with external validation**
C. Hobeika[1], M. Ronot[1], D. Valla[1], J. M. Correas[2], V. Vilgrain[1], *M. Dioguardi Burgio*[1]; [1]Clichy/FR, [2]Paris/FR
*(marco_dioguardi@hotmail.it)*

**Purpose:** Non-invasive tools assessing steatosis, such as ultrasonography-based 2D-attenuation imaging (ATI), are needed to tackle the worldwide burden of NAFLD. This one-stage individual patient data (IPD) meta-analysis aimed to create an ATI-based steatosis grading system.
**Methods or Backround:** A systematic review (EMBASE+MEDLINE, 2018-2022) identified studies, including patients with histologically or MRI-PDFF-verified ATI values for grading steatosis (S0 to S3). One-stage IPD meta-analyses were conducted using generalised mixed models with a random study-specific intercept. Created ATI-based steatosis grading system (aS0 to aS3) was externally validated on a prospective cohort of patients with type 2 diabetes and NAFLD (n=174, histologically and MRI-PDFF verified steatosis).
**Results or Findings:** Eleven enrolled studies included 1374 patients, classified into S0, S1, S2, and S3 in 45.4%, 35.0%, 9.3%, and 10.3% of the cases. ATI was correlated with histologically (r=0.60; 95%CI: 0.52,0.67; p<0.001), and MRI-PDFF (r=0.70; 95%CI: 0.66,0.73; p<0.001) quantified steatosis while uncorrelated with liver stiffness (r=0.03; 95%CI: -0.04,0.11, p=0.343). Steatosis grade (Coefficient: 0.27; 95%CI: 0.07,0.47; p=0.008) was the only independent factor associated with ATI, while age, sex, BMI, chronic hepatitis, and alcohol consumption were not. ATI marginal means within S0, S1, S2, and S3 subpopulations were 0.59 (95%CI: 0.56,0.61), 0.69 (95%CI: 0.65,0.72), 0.77 (95%CI: 0.73,0.81), and 0.84 (95%CI: 0.80, 0.89) dB/cm/MHz; all contrasts between grades were significant (p<0.0001). Three ATI thresholds were calibrated to create a new ATI-based steatosis grading system (aS0 to aS3, cut-offs: 0.66, 0.73, and 0.81dB/cm/MHz). Its external validation showed Obuchowski measures (to interpret as AUC values) of 0.84±0.01 and 0.82±0.02 with histologically- and MRI-PDFF-based references.

**Conclusion:** ATI is a reliable non-invasive marker of steatosis. This validated ATI-based steatosis grading system could be valuable in assessing NAFLD patients.
**Limitations:** There is a difference in steatosis prevalence among the included studies.
**Ethics committee approval:** This study was approved by the local Institutional Research Board.
**Funding for this study:** No funding was received for this study.
**Author Disclosures:**
Jean Michel Correas: Nothing to disclose
Marco Dioguardi Burgio: Nothing to disclose
Maxime Ronot: Nothing to disclose
Dominique Valla: Nothing to disclose
Valérie Vilgrain: Nothing to disclose
Christian Hobeika: Nothing to disclose

### RPS 801-8

**Depth dependence of the backscatter coefficient measurement for ultrasound fat quantification**

*G. Ferraioli*[1], A. De Silvestri[1], R. G. Barr[2]; [1]Pavia/IT, [2]Rootstown, OH/US
*(giovanna.ferraioli@unipv.it)*

**Purpose:** It has been reported that the estimate of the ultrasound attenuation coefficient (AC) for liver fat quantification is affected by the depth of measurements, with a linear decrease of values with depth. The purpose of this paper was to determine whether the backscatter coefficient (BSC) has the same behavior.
**Methods or Backround:** This retrospective study was performed with the Sequoia ultrasound system equipped with the ultrasound derived fat fraction (UDFF) algorithm (Siemens Healthineers, Germany) that combines BSC with AC. UDFF was obtained positioning the upper edge of the region of interest (3x3 cm) at 1.5, 2, 3, 4, 5 cm below liver capsule. BSC data were extracted from UDFF offline. The median value of five acquisitions was used for statistical analysis. A fractional polynomial regression, which selects the best model considering the polynomial development of the variables of interest, was used. The covariates included were age, sex, skin-to-liver-capsule distance, and stiffness. The distance was included as linear factor or with a power of -2;-1;-0.5;0;0.5;1;2;3 or each possible pair of them. Best fitting models was chosen according to partial F test. Body mass index (BMI) was not included because of collinearity with skin-to-liver capsule distance.
**Results or Findings:** Fifty-one individuals (25 females, mean age: 61±13 years; mean BMI: 27±6kg/m2; skin-to-liver-capsule distance: 2.0±0.5cm; liver stiffness: 8±6kiloPascal) were studied. Best fitting model included depth as square root (beta -40; 95% confidence intervals from -64 to -15) and linear factor (beta 11; 95% confidence intervals from 4 to 18). Skin-to-liver-capsule distance and stiffness also were independent predictors of BSC.
**Conclusion:** There is a depth dependence in liver BSC measurement that can substantially affect results. A standardised acquisition protocol is needed to compare results and to reliably assess changes in serial measurements.
**Limitations:** No limitations were identified.
**Ethics committee approval:** The study is retrospective.
**Funding for this study:** No funding was received for this study.
**Author Disclosures:**
Annalisa De Silvestri: Nothing to disclose
Giovanna Ferraioli: Speaker: Canon Medical Systems, Fujifilm Medical Systems, Mindray Ultrasound, Philips Ultrasound, Siemens Healthineers; Advisory Board: Philips Ultrasound
Richard G. Barr: Research/Grant Support: Siemens Healthiness, Philips Ultrasound, Mindray Ultrasound, Samsung Ultrasound, Canon Ultrasound, Hologic Ultrasound; Advisory Board: Lantheus; Medical Speaker: Siemens Healthiness, Philips Ultrasound, Mindray Ultrasound, Canon Medical Systems, Hologic Ultrasound

### RPS 801-9

**Artificial intelligence-calculated hepatorenal index for quantitative evaluation of hepatic steatosis in metabolic dysfunction-associated steatotic liver disease**

Z. Zsombor, A. D. Rónaszéki, B. Csongrády, R. Stollmayer, B. K. Budai, V. Bérczi, P. Maurovich-Horvat, K. Hagymási, *P. N. Kaposi-Novák*; Budapest/HU

**Purpose:** The goal of our study was to evaluate artificial intelligence-calculated hepatorenal index (AI-HRI) as a diagnostic method for hepatic steatosis.
**Methods or Backround:** We prospectively enrolled 103 patients with clinically suspected metabolic dysfunction-associated steatotic liver disease (MASLD). All patients had a quantitative ultrasound (QUS), including AI-HRI, ultrasound attenuation coefficient (AC), and ultrasound backscatter-distribution coefficient (SC) measurements. The ultrasonographic fatty liver indicator (US-FLI) score was also calculated. The magnetic resonance imaging fat fraction (MRI-PDFF) was the reference to classify patients into four grades of steatosis: none <5%, mild 5-10%, moderate 10-20%, and severe ≥20%. We determined the agreement between AI-HRI by two examiners using the intraclass correlation coefficient (ICC) of 68 cases.
**Results or Findings:** The AI-HRI was significantly different between groups without (1.480 ±0.607, p<0.003) and with mild steatosis (2.155 ±0.776), as well as between mild and moderate steatosis (2.777 ±0.923, p<0.018). AI-HRI showed a moderate correlation with AC (Spearman's r=0.597), SC (rs=0.473), US-FLI (r=0.5), and MRI-PDFF (r=0.528). The agreement in AI-HRI was good between the two examiners (ICC=0.635, 95% confidence interval (CI)=0.411–0.774, p<0.001). The AI-HRI could detect mild steatosis (AUC=0.758, 95% CI=0.621–0.894) with fair and moderate/severe steatosis (AUC=0.803, 95% CI=0.721–0.885) with good accuracy. However, the performance of AI-HRI was not significantly different (p<0.578) between the two diagnostic tasks.
**Conclusion:** AI-HRI is an efficient, reproducible, and accurate QUS method to diagnose mild and moderate hepatic steatosis.
**Limitations:** This is a single-centre study with a limited number of patients.
**Ethics committee approval:** This study was approved by the Regional and Institutional Science and Research Ethics Committee at Semmelweis University (Protocol number: SE RKEB 140/2020, 16-07-2020).
**Funding for this study:** No funding was received for this study.
**Author Disclosures:**
Pál N. Kaposi-Novák: Consultant: Samsung-Medison Ltd.; Investigator: Samsung-Medison Ltd.
Krisztina Hagymási: Investigator: Samsung-Medison
Barbara Csongrády: Nothing to disclose
Aladár David Rónaszéki: Nothing to disclose
Pál Maurovich-Horvat: Nothing to disclose
Róbert Stollmayer: Nothing to disclose
Viktor Bérczi: Nothing to disclose
Zita Zsombor: Nothing to disclose
Bettina Katalin Budai: Nothing to disclose

### RPS 801-10

**Fatty liver disease in the adult population: new multiparametric ultrasound compared to magnetic resonance**

C. Solito, C. Di Bella, P. Pacini, G. T. Lucarelli, *M. Renda*, V. Dolcetti, G. Del Gaudio, V. Cantisani; Rome/IT

**Purpose:** Metabolic dysfunction-associated fatty liver disease (MAFLD) is the most frequent chronic liver disease in the adult population and liver biopsy diagnosis is the gold standard. The purpose of this study is to evaluate the diagnostic accuracy of the new Hepato-Renal Index with Automated ROI Recommendation (EzHRI™), Tissue Attenuation Imaging (TAI™), and Tissue Scatter distribution Imaging (TSI™) ultrasound software for hepatic steatosis quantification, comparing with MRI.
**Methods or Backround:** 92 patients underwent multiparametric ultrasound evaluation and 3T MRI evaluation with two different techniques- 3D T2* with Dixon pulse multiple-echo sequence (IDEAL IQ; MR-PDFF- and MR spectroscopy). The best QUS cut-offs have been identified and the QUS results are compared with MRI proton density fat fraction for diagnosis and quantification of liver fat content. Inter-observational variability was also tested between two operators.
**Results or Findings:** The best cut-off values for MAFLD diagnosis are EzHRI>1.21; TAI>0.63 and TSI>92 with a sensitivity of 97.5%, 88.8% and 98.8% respectively and a specificity of 83.3%, 100% and 66.7% respectively. Comparing QUS with MRI in detection of liver steatosis, sensitivity and specificity were 88.8% and 100% for TAI, 98.8% and 66.7% for TSI and 97.5% and 83.3% for EzHRI. In the quantification of liver fat content, TAI sensitivity and specificity were 80%-100%, 100%-100% and 100%-100% for mild, moderate and severe steatosis respectively; TSI sensitivity and specificity were 97.8%-66.7%, 100%-66.7% and 100%-66.7% for mild, moderate and severe steatosis respectively; EzHRI sensitivity and specificity were 95.6%-83.3%, 100%-83.3% and 100%-83.3% for mild, moderate and severe steatosis respectively.
**Conclusion:** QUS is a useful tool for screening and monitoring patients with MAFLD and can assist in avoiding unnecessary biopsies.
**Limitations:** This study is limited by interoperator variability, which diminishes its reproducibility. Furthermore, there is reduced accuracy in patients with obesity or renal disease.
**Ethics committee approval:** This study was approved by Sapienza-Roma. No additional information was provided by the submitter.
**Funding for this study:** No funding was received for this study.
**Author Disclosures:**
Giovanni Del Gaudio: Nothing to disclose
Maurizio Renda: Nothing to disclose
Patrizia Pacini: Nothing to disclose
Chiara Di Bella: Nothing to disclose
Giuseppe Tiziano Lucarelli: Nothing to disclose
Vincenzo Dolcetti: Nothing to disclose
Carmen Solito: Nothing to disclose
Vito Cantisani: Nothing to disclose

### RPS 801-11

**Fully automated MRI-based convolutional neural network for noninvasive diagnosis of cirrhosis**

*T. Zheng*[1], Y. Zhu[2], Y. Chen[1], Y. Qu[1], Y. Chen[2], B. Song[1]; [1]Chengdu/CN, [2]Shanghai/CN
*(zhengtianying.su@foxmail.com)*

**Purpose:** This study aimed to develop a fully automated diagnostic convolutional neural network (CNN) model for cirrhosis based on liver MRI and serum biomarkers.
**Methods or Backround:** This single-centre retrospective study enrolled consecutive patients receiving pathological evaluation of liver fibrosis stage and contrast-enhanced liver MRI between March 2010 and May 2021. On the training set, an MRI-based CNN model was constructed for cirrhosis against pathology, and then a combined model was developed integrating the CNN model and serum biomarkers. On the testing set, the area under the receiver operating characteristic curve (AUC) was computed to compare the diagnostic performance of the combined model with that of aminotransferase-to-platelet ratio index (APRI), fibrosis-4 index (FIB-4), and radiologists. The influence of potential confounders on the diagnostic performance was evaluated by subgroup analyses.
**Results or Findings:** A total of 934 patients (median age, 53 years; 762 men; training, n=840) were enrolled, 601 (64%) with pathological cirrhosis. The CNN model was constructed on pre-contrast T1- and T2-weighted imaging, and the combined model was developed integrating the CNN model, age, and eight serum biomarkers. On the testing dataset, the combined model achieved an AUC of 0.89, which outperformed FIB-4, APRI and two radiologists (AUC: 0.71 to 0.78, all p<0.05). Subgroup analyses revealed comparable diagnostic performances of the combined model in patients with different sizes of focal liver lesions.
**Conclusion:** Based on pre-contrast T1- and T2-weighted imaging, age, and serum biomarkers, the combined model allowed accurate diagnosis of cirrhosis, independent of size of focal liver lesions.
**Limitations:** The limitations of the study are that it is a single-centre retrospective study, lack of a head-to-head comparison with elastography techniques, and unknown model performance compared with a combination of radiologists and serum biomarkers .
**Ethics committee approval:** This study was approved by the Ethics Committee on Biomedical Research, West China Hospital of Sichuan University (2021-1370).
**Funding for this study:** Funding was received from the National Natural Science Foundation of China (Grant No. U22A20343, 82101997), National Health Commission Capacity Building and Continuing Education Center (Grant No. YXFSC2022JJSJ007), the 1.3.5 project for disciplines of excellence, West China Hospital, Sichuan University (Grant No. ZYJC21012), Med-X Center for Informatics, Sichuan University (NO.YGJC007), and the Science and Technology Department of Sichuan Province (No. 2022YFS0071).
**Author Disclosures:**
Yali Qu: Nothing to disclose
Yidi Chen: Nothing to disclose
Yinan Chen: Nothing to disclose
Bin Song: Nothing to disclose
Yajing Zhu: Nothing to disclose
Tianying Zheng: Nothing to disclose

### RPS 801-12

**Noninvasive assessment of liver segmental volumes and its relationship with 5-year prognostication**

*D. Catucci*, J. Hrycyk, N. F. Lange, V. Obmann, A. Berzigotti, L. Ebner, A. Christe, J. T. Heverhagen, A. T. Huber; Berne/CH

**Purpose:** This study aimed to analyse the performance of caudate to right lobe ratio (CRL-R), liver segmental volume ratio (LSVR) and liver segmental volume and attenuation ratio (LSVAR) to screen for chronic liver disease (CLD) on routine abdominal CT scans and to predict the 5-year probability of transplant-free survival and first hepatic decompensation.
**Methods or Backround:** This retrospective study included 108 patients without CLD (noCLD-group; n=108), as well as 98 patients with CLD and liver biopsy. All patients underwent abdominal CT scans between 03/2015 and 08/2017. Patients with CLD were further divided into three groups based on their liver fibrosis degree: early CLD (F0-F2; eCLD-group; n=40), advanced CLD (F3-F4; aCLD-group; n=20), and aCLD with clinically significant portal hypertension according to the BAVENO VII consensus (aCLDPH-group; n=38). CRL-R, LSVR, and LSVAR were measured in all patients. The study analysed the 5-year outcomes of each patient, including death or liver transplantation and first hepatic decompensation. Statistical analysis included the Kruskal-Wallis test, ROC curve analysis, and the Kaplan-Meier curve.
**Results or Findings:** CRL-R, LSVR and LSVAR differed significantly between all groups (p<0.001). A CRL-R cutoff-value of > 0.93 proofed best to detect patients with CLD (sensitivity of 69%, specificity of 78%). Patients with both CRL-R >0.99 and LSVR >0.37 had the lowest probability of 5-year transplant-free survival (46%) and the lowest probability of a decompensation-free 5-year course (75%).
**Conclusion:** CRL-R, LSVR and LSVAR allow screening for CLD and prognostication of 5-year transplant-free survival and occurrence of first hepatic decompensation.
**Limitations:** The limitations of this study were its retrospective design and the lack of invasive measurement of the hepatovenous pressure gradient, which was not available due to ethical concerns.
**Ethics committee approval:** This retrospective study was approved by the Institutional Review Board.
**Funding for this study:** This study received funding by the Swiss National Science Foundation (SNF), Grant number 188591.
**Author Disclosures:**
Johannes T. Heverhagen: Nothing to disclose
Damiano Catucci: Nothing to disclose
Verena Obmann: Nothing to disclose
Joris Hrycyk: Nothing to disclose
Lukas Ebner: Nothing to disclose
Naomi Franziska Lange: Nothing to disclose
Andreas Christe: Nothing to disclose
Adrian Thomas Huber: Nothing to disclose
Annalisa Berzigotti: Nothing to disclose

**09:30-11:00 Research Stage 2**

### Research Presentation Session: Breast

### RPS 802

### Applications of contrast-enhanced mammography (CEM)

**Moderator**
E. M. Fallenberg; Munich/DE
*(eva.fallenberg@tum.de)*
**Author Disclosures:**
Eva M. Fallenberg: Advisory Board: Bayer Healthcare, GE Healthcare, BD, B-RayZ, Guerbet; Board Member: EUSOBI, AG-Breast Imaging German x-ray Society; Grant Recipient: German resaerch association; Speaker: GE Healthcare, Bayer Healthcare, Guerbet, Siemens, BD, Roche

### RPS 802-2

**A large-scale single-centre evaluation of enhanced mammography (CEM) in the pre-operative staging of breast cancer**

*F. Pugliese*, G. Migliaro, G. Bicchierai, C. Bellini, D. De Benedetto, F. Di Naro, L. A. Incardona, V. Miele, J. Nori; Florence/IT
*(francescapugliese28@gmail.com)*

**Purpose:** The purpose of this study was to evaluate the role and efficacy of contrast enhanced mammography in the pre-operative management of patients with biopsy-proven breast cancer.
**Methods or Backround:** We selected 524 patients with breast cancer who underwent CEM as preoperative staging and had breast cancer-related surgery at our institution, between 2017 and 2020. We analysed those cases in which CEM led to additional imaging or biopsy and those in which it changed the type of surgery. CEM sensitivity in identifying the index lesion and sensitivity, specificity, positive (PPV) and negative (NPV) predictive values, and accuracy in the correct preoperative staging of breast cancer of the whole population and in various subgroups were calculated.
**Results or Findings:** CEM changed the surgical plan in 22.48% of cases (118/525 patients) when the extension of the index lesion was different from what was previously detected by conventional imaging or when an additional lesion was detected. The sensitivity, specificity, PPV and NPV, and accuracy of CEM were 94.74%, 95.86%, 86.40%, 98.5%, diagnostic accuracy was 95.62% and AUC of ROC curve was 0.953. Conclusively, after using a chi-square test, the statistical analysis showed a higher diagnostic performance in patients with palpable lesions vs patients with non-palpable ones (p=0.0022).
**Conclusion:** CEM changed the surgical plan in 22.48% of our series, proving to be an accurate exam in the presurgical staging of breast cancer. CEM diagnostic performances support its reliability in the presurgical staging setting, for a lesion-tailored management, leading to the best surgical plan, avoiding over- or undertreatment.

**Limitations:** No limitations were identified.
**Ethics committee approval:** This study is retrospective.
**Funding for this study:** No funding was received for this study.
**Author Disclosures:**
Giulia Bicchierai: Nothing to disclose
Federica Di Naro: Nothing to disclose
Francesca Pugliese: Nothing to disclose
Diego De Benedetto: Nothing to disclose
Jacopo Nori: Nothing to disclose
Vittorio Miele: Nothing to disclose
Chiara Bellini: Nothing to disclose
Giuliano Migliaro: Nothing to disclose
Ludovica Anna Incardona: Nothing to disclose

### RPS 802-3

**Outcomes of surveillance using contrast-enhanced mammography in women with a personal history of breast cancer**

J. Matheson[1], A. Rose[1], C. Nickson[2], K. Elder[1], *B. Mann*[1]; [1]Parkville, VIC/AU, [2]Sydney, NSW/AU
*(bruce.mann@mh.org.au)*

**Purpose:** Annual mammography with selective ultrasound is standard for women with Personal History of Breast Cancer (PHBC). Rates of interval cancers of 3.6 per 1000 screens have been reported. Earlier diagnosis of recurrences and new cancers may allow more effective treatment. This study aimed to clarify the utility of contrast-enhanced mammography (CEM), a more sensitive scan than conventional mammography and comparable to MRI, for surveillance.
**Methods or Backround:** This was a retrospective study of 1,190 women with PHBC undergoing CEM surveillance between June 2016 and December 2022. Outcomes of initial CEM and subsequent imaging were collected, including incident surveillance-detected and interval cancers, recalls for assessment, and pathology and treatment details. Descriptive statistics and hazards modelling was used.
**Results or Findings:** There were 3,784 surveillance episodes: 1,190 first CEMs and 2,594 subsequent surveillance episodes. 79% of women had at least three annual rounds of surveillance. 186/3784 surveillance episodes were recalled for assessment (recall rate 4.9%). 72 (39%) recalled cases were true positive- 50 invasive and 22 DCIS. The cancer detection rate was 19/1000. 51% of recalls were due to contrast and 35% of these were true positive. Invasive cancers were predominantly stage 1 (64%) or stage 2 (32%) and most were grade 2 (44%) or grade 3 (47%). The median invasive cancer size was 16mm (IQR 9-25mm). The rate of symptomatic interval cancers was 0.8 per 1000 screens (program sensitivity 96.0%). Surveillance-detected cancer rates differed significantly by index cancer subtype ($\chi 2=11.9$, $p=0.0026$), with the highest rates for women with TNBC.
**Conclusion:** Routine CEM in surveillance of women with PHBC led to higher cancer detection and lower interval cancer rates than published series. CEM appears to increase the sensitivity of surveillance programs for women with PHBC. This may allow more effective treatment.
**Limitations:** This was a single institution study.
**Ethics committee approval:** The study was approved by the Melbourne Health Research and Ethics Committee, QA2019129.
**Funding for this study:** Funding was received by an unrestricted grant from Hologic.
**Author Disclosures:**
Allison Rose: Research/Grant Support: Hologic
Julia Matheson: Nothing to disclose
Carolyn Nickson: Nothing to disclose
Kenneth Elder: Nothing to disclose
Bruce Mann: Research/Grant Support: Hologic

### RPS 802-4

**Contrast-enhanced mammography in local staging of screen-detected breast cancer**

C. MacCallum[1], B. Mann[1], C. Nickson[2], *A. Rose*[1]; [1]Parkville, VIC/AU, [2]Carlton, VIC/AU

**Purpose:** BreastScreen Australia provides assessment of suspected breast cancer to the point of diagnosis using conventional imaging and percutaneous biopsy. Comprehensive local staging is not performed. The purpose of this study was to introduce contrast-enhanced mammography (CEM) for local staging of screen-detected cancer at our academic hospital. We report findings for otherwise occult disease and impact on treatment.
**Methods or Backround:** Women with screen-detected cancer undergoing staging CEM between November 2018 and April 2022 were identified retrospectively. Additional contrast-detected abnormalities were investigated with preoperative percutaneous biopsy, surgical biopsy, or problem-solving MRI. Findings were recorded. Invasive cancer or DCIS were considered as true positives (TP) and other findings as false positives (FP). Impact on surgical decisions was assessed.
**Results or Findings:** 204 patients underwent CEM, of whom 62/204 (30%) had 76 additional abnormalities. 36/76 (47%) were TP and 40/76 (53%) FP. CEM identified malignant occult lesions (mOLs) in 30/204 patients (15%). 75% moLs were invasive and 25% DCIS. 83% (30/36) of mOLs were ipsilateral. Most invasive mOLs were Grade 2 (20/27, 74.1%). Patients with higher background parenchymal enhancement (BPE) had a higher rate of mOLs (20% for moderate/marked vs 4% for minimal/mild, $p=0.0023$). No statistically significant differences were found by mammographic density or age. Surgical management was changed in 45/204 (22%) patients, 30 with mOLs and 15 with benign and atypical proliferative lesions. Altered surgery included wider resection (24/45), conversion to mastectomy (8/45), contralateral breast surgery (6/45), additional ipsilateral excision (5/45), and bracketing (2/45).
**Conclusion:** CEM for local staging of screen-detected cancers identified additional malignant lesions in 15% of patients (particularly those with higher BPE), with many clinically significant lesions. Findings led to surgical management change in 22% of patients. Larger studies with oncological outcomes are needed.
**Limitations:** This was a single arm study. Larger numbers and oncological outcomes are needed to overcome the limitations of this study.
**Ethics committee approval:** This study was approved by a Quality Assurance project by the Melbourne Health Research and Ethics Committee.
**Funding for this study:** Funding was received by a research grant supported by Hologic.
**Author Disclosures:**
Allison Rose: Nothing to disclose
Carolyn Nickson: Nothing to disclose
Caroline MacCallum: Nothing to disclose
Bruce Mann: Research/Grant Support: Hologic, unrestricted grant

### RPS 802-5

**Comparison of the diagnostic accuracy of contrast-enhanced spectral mammography with delayed image and digital mammography in the diagnosis of breast cancer in BI-RADS 4 mammographic findings**

*A. Ainakulova*, Z. J. Zholdybay, D. Kaidarova, Z. M. Amankulov, M. Gabdulina; Almaty/KZ
*(ar89@list.ru)*

**Purpose:** The purpose of this study is to compare the diagnostic accuracy of contrast-enhanced spectral mammography (CESM) with delayed image and digital mammography (DM) in breast cancer detection in BI-RADS 4 mammographic findings among mass lesions, suspicious microcalcifications, asymmetries, and architectural distortions.
**Methods or Backround:** Between September 2018 and May 2021, mammographic examinations of 1968 women were carried out according to clinical indicators. According to the results of mammographic examination, BI-RADS 4 mammographic findings were revealed in 374 (19%) patients, requiring differential and clarifying diagnostics. All 374 patients underwent CESM with delayed image. All lesions were histologically verified. Lesions were categorised based on ACR BI-RADS Fifth Edition. On CESM with delayed image, lesions were categorised by dynamic patterns of contrast enhancement.
**Results or Findings:** Of the 398 lesions identified, 208 (52.26%) were malignant and 190 (47.73%) were benign. Mass lesions were detected in 240 (64.2%) women, suspicious microcalcifications in 60 (16%) women, architectural distortions in 31 (8.3%) women, and asymmetry in 43 (11.5%) women. For DM and CESM with delayed image among mass lesions sensitivity was 82.1% and 95.5%; specificity was 51.4% and 89.2% respectively. For DM and CESM with delayed image among suspicious microcalcifications, asymmetries, and architectural distortions, sensitivity was 100%, whereas specificity was 82% and 97.4%; 65% and 96.3%; 57.1% and 94.1% respectively. The ROC curve of all BI-RADS 4 mammographic findings had an area under the curve of 0.965 for CESM with delayed image, and of 0.749 for DM ($p=0.000$).
**Conclusion:** CESM with delayed image showed high sensitivity and specificity in the diagnosis of breast cancer among mass lesions. CESM with delayed image significantly increased specificity in the diagnosis of breast cancer among microcalcifications, asymmetries, and architectural distortions, and may be helpful to avoid unnecessary biopsies in BI-RADS 4 mammographic findings.
**Limitations:** The study used a small number of patients with suspicious microcalcifications, asymmetries, and architectural distortions.
**Ethics committee approval:** No information provided by the submitter.
**Funding for this study:** No funding was received for this study.
**Author Disclosures:**
Madina Gabdulina: Nothing to disclose
Zhandos Mukhtaruly Amankulov: Nothing to disclose
Zhamilya J. Zholdybay: Nothing to disclose
Dilyara Kaidarova: Nothing to disclose
Akmaral Ainakulova: Nothing to disclose

### RPS 802-6

**Diagnostic performance of lesion conspicuity and contrast-to-noise ratio (CNR) values on contrast-enhanced-mammogram (CEM) recombined images in predicting malignancy**

*I. Allajbeu*, R. Manavaki, M. Nanaa, N. Healy, V. Papalouka, N. Payne, F. Gilbert; Cambridge/UK
*(ia359@cam.ac.uk)*

**Purpose:** The purpose of this study was to compare the diagnostic performance of lesion conspicuity and contrast-to-noise ratio (CNR) on CEM recombined images in differentiating benign and malignant lesions.
**Methods or Backround:** CEM examinations with BI-RADS scores of 3-5 and histopathologically confirmed findings were eligible for inclusion. Two breast radiologists assessed lesion conspicuity as low, moderate, or high, based on the BIRADS CEM lexicon. Two regions-of-interest (ROI) representing lesion and background, respectively, were delineated on the early (CC) and late (MLO) views to calculate CNR1 and CNR2, respectively, using CNR=(Sa-Sb)/Sb (Sa: maximum value in the lesion ROI, Sb: mean value in the background ROI). Associations between nominal, or continuous and nominal variables were assessed using chi-squared test or rank-biserial correlation coefficient (rrb) respectively. Receiver operating characteristic (ROC) curves and area-under-the curve (AUC) were used to assess diagnostic performance. p values < 0.05 were deemed statistically significant.
**Results or Findings:** Of 143 lesions, 85 were normal/ benign and 58 malignant confirmed by histopathology. All high conspicuity lesions were invasive cancers (9/9, 100%) with 82% (49/60) low conspicuity lesions being normal/ benign, and 78% (45/58) malignant lesions showing either moderate or high conspicuity ($\chi^2$=60.1, p<.001). Malignancy was associated with increased CNR1 (rrb=0.65, p<.001) and CNR2 (rrb=0.41, p<.001) values. CNR1 performed better in differentiating benign from malignant lesions (AUC 0.75, accuracy: 71%, sensitivity: 80%, specificity 63%) than CNR2 (AUC 0.71, accuracy: 68%, sensitivity: 71%, specificity 65%) or lesion conspicuity (AUC: 0.75%, accuracy: 71%, sensitivity: 80%, specificity: 64%).
**Conclusion:** Both conspicuity and CNR quantitative assessment of enhancing lesions on CEM can improve specificity in differentiating benign from malignant breast disease. Quantitative measurement of lesion enhancement performs better than conspicuity in predicting malignancy, with CNR calculated on the early view offering the best diagnostic performance.
**Limitations:** This was a single centre retrospective study with a limited dataset.
**Ethics committee approval:** The study was approved by the BRAID trial (IRAS project ID: 25137).
**Funding for this study:** Funding was received as part of the BRAID trial.
**Author Disclosures:**
Roido Manavaki: Nothing to disclose
Muzna Nanaa: Nothing to disclose
Iris Allajbeu: Research/Grant Support: Funding from GE healthcare
Fiona Gilbert: Research/Grant Support: GE healthcare
Vasiliki Papalouka: Nothing to disclose
Nicholas Payne: Research/Grant Support: GE healthcare
Nuala Healy: Nothing to disclose

### RPS 802-7

**Feasibility and performance of contrast-enhanced tomosynthesis compared to contrast-enhanced MRI**

*P. Clauser*[1], N. Pötsch[1], P. Kapetas[1], M. Hörnig[2], M. Weber[1], R-I. Milos[1], P. A. Baltzer[1], T. H. Helbich[1]; [1]Vienna/AT, [2]Forchheim/DE
*(clauser.p@hotmail.it)*

**Purpose:** The performance of contrast-enhanced mammography (CEM) is limited by the 2D image acquisition. We hypothesise that contrast-enhanced tomosynthesis (CE-DBT) could allow for improved lesion characterisation, truly comparable to contrast-enhanced magnetic resonance imaging (CE-MRI). The aim of our analysis was to compare the diagnostic performance of a CE-DBT prototype with CE-MRI.
**Methods or Backround:** Women presenting with suspicious findings on mammography, DBT, or ultrasound were invited to participate in the study. Participants underwent CE-DBT of the breast with suspicious findings using a dedicated prototype. The suspicious findings were biopsied and only histologically verified lesions were included in the analysis. Four readers (R1 and R2 non-experienced; R3 and R4 experienced) evaluated the images, blinded to patients' history, previous imaging, and histology. The readers evaluated CE-MRI and CE-DBT (including DBT and synthetic mammography) in separate sessions and gave a BI-RADS score for each finding. Sensitivity and specificity were calculated and compared.
**Results or Findings:** We included 84 patients (mean age 56 years, range 39-70) with 91 histologically verified breast lesions (27 benign, 64 malignant). Sensitivity was comparable between CE-DBT and CE-MRI for R3 (both 100%), while sensitivity was higher with CE-MRI than with CE-DBT for the other readers (CE-DBT raging from 89 to 94%, CE-MRI from 97 to 100%). Specificity was overall higher for experienced than for non-experienced readers. Specificity improved with CE-MRI for non-experienced readers, while no significant difference was detected between CE-DBT and CE-MRI for experienced readers.
**Conclusion:** Our study showed that CE-DBT has a good diagnostic performance for the characterisation of breast lesions, almost comparable to that of CE-MRI of the breast.
**Limitations:** This was a single centre, cancer enriched dataset.
**Ethics committee approval:** The study was approved by the Ethics Committee and patients gave written informed consent.
**Funding for this study:** Funding for this study was received from Siemens Healthcare GmBH.
**Author Disclosures:**
Pascal A.T. Baltzer: Nothing to disclose
Nina Pötsch: Nothing to disclose
Thomas H. Helbich: Nothing to disclose
Mathias Hörnig: Employee: Siemens Healthcare GmbH
Ruxandra-Iulia Milos: Nothing to disclose
Panagiotis Kapetas: Nothing to disclose
Michael Weber: Nothing to disclose
Paola Clauser: Speaker: Siemens Healthcare GmbH

### RPS 802-8

**Preoperative contrast mammography vs MRI: which one is better?**

*C. Mbongo*, M. B. Barrio Piqueras, P. Malmierca Ordoqui, M. Jiménez Vázquez, A. Elizalde, L. J. Pina Insausti; Pamplona/ES
*(cmbongoh@unav.es)*

**Purpose:** The aim of this study was to compare the sensitivity and accuracy of Contrast Mammography (CEDEM) vs MRI as preoperative imaging techniques.
**Methods or Backround:** This is a retrospective lesion-based study. From January 2018 to December 2019, 77 patients underwent CEDEM, MRI and percutaneous biopsy at our centre as a part of the breast lesion preoperative protocol. CEDEM and MRI images were then reviewed, and BI-RADS categorisation for each lesion was subsequently collected. The final histopathologic report was also assessed and considered the gold standard. BI-RADS categories 1 to 3 were considered negative, while 4 and 5 were classified as positive. A McNemar test was used to compare the sensitivity of both imaging techniques, and ROC curves were performed to assess the areas under the curve, both using SPSS statistical software.
**Results or Findings:** The 77 female patients (37-84, mean 55.5 y/o) showed 98 lesions with pathologic correlation (16 benign; 82 malignant: 77 invasive ca, of which 43 were LumA, 22 LumB, 6 pure Her2, 6 Triple negative, and finally 5 DCIS). The sensitivity of CEDEM vs MRI was 83.3% vs 97.6% (p=0.002). The areas under the curve were 0.87 vs 0.86 (p=0.9), respectively.
**Conclusion:** Although MRI is significantly more sensitive than CEDEM, the accuracy was similar for both modalities.
**Limitations:** We had a low number of cases; it was a monocentric study, and no intra/interobserver variability was studied.
**Ethics committee approval:** No information provided by the submitter.
**Funding for this study:** No funding was received for this study.
**Author Disclosures:**
Carmen Mbongo: Nothing to disclose
Patricia Malmierca Ordoqui: Nothing to disclose
Miguel Barrio Barrio Piqueras: Nothing to disclose
Arlette Elizalde: Nothing to disclose
Marcos Jiménez Vázquez: Nothing to disclose
Luis Javier Pina Insausti: Nothing to disclose

### RPS 802-9

**Tumour involvement of the nipple at preoperative contrast-enhanced mammography (CEM): preliminary results of a multicentre diagnostic accuracy study**

*T. van Nijnatten*[1], A. Cozzi[2], R. Alcantara[3], G. Della Pepa[4], J. James[5], C. Depretto[4], G. P. Scaperrotta[4], S. Schiaffino[2]; [1]Maastricht/NL, [2]Lugano/CH, [3]Barcelona/ES, [4]Milan/IT, [5]Nottingham/UK
*(thiemovn@gmail.com)*

**Purpose:** The aim of this study was to assess the diagnostic accuracy of contrast-enhanced mammography (CEM) in the prediction of pathologic nipple involvement, CEM diagnostic accuracy was also compared with contrast-enhanced breast MRI scans.
**Methods or Backround:** This retrospective multicentre study included patients with biopsy-proven breast cancer who underwent preoperative CEM in three referral centres (Italy, Spain, the Netherlands), between April 2013 and June 2023. Patients were included if they did not undergo neoadjuvant therapy and if they underwent either mastectomy or central lumpectomy. After local image review by board-certified breast radiologists (5-to-10 years of experience), the diagnostic performance of CEM in predicting nipple involvement was calculated, taking surgical pathology as the reference standard and compared with MRI (McNemar's test) in a subset of patients.
**Results or Findings:** A total 70 patients (median age 64 years, interquartile range 51–73 years) with breast cancer (50/70 invasive ductal carcinoma, 16/70 invasive lobular carcinoma, 4/70 pure DCIS) were included in the analysis,

Thursday

59/70 also having undergone MRI. Nipple involvement at surgical pathology was found in 25/70 cases (35.7%). Considering all 70 patients, CEM had a 72.0% sensitivity (95% CI 50.6–87.9%) and an 88.9% specificity (95% CI 76.0–96.3%) for the prediction of pathological nipple involvement. All seven false negative cases presented as non-mass enhancement lesions without peri-areolar skin thickening. Among the 59 patients with both CEM and MRI, CEM had a 65.0% sensitivity (95% CI 44.1–85.9%), significantly lower (p=0.014) than the 95.0% sensitivity of MRI (95% CI 85.4–100%), whereas no significant differences (p=0.179) were found between CEM specificity (97.4%, 95% CI 92.5–100%) and MRI specificity (89.7%, 95% CI 80.2–99.3%).
**Conclusion:** Preoperative CEM showed moderate sensitivity and high specificity in assessing nipple involvement. Compared to MRI, CEM had significantly lower sensitivity and non-significantly higher specificity.
**Limitations:** The study is limited by its reliance on preliminary data, as well as the exclusion of NAT patients.
**Ethics committee approval:** The study is a pooled analysis of data from other studies, which received approval from Ethics Committees in three different centres (Maastricht University Medical Centre +, the Netherlands; Hospital Del Mar, Barcelona, Spain; and the Fondazione IRCCS Istituto Nazionale dei Tumori, Milano, Italy).
**Funding for this study:** No funding was received for this study.
**Author Disclosures:**
Gianmarco Della Pepa: Nothing to disclose
Andrea Cozzi: Nothing to disclose
Rodrigo Alcantara: Nothing to disclose
Thiemo van Nijnatten: Speaker: Bayer Speaker: GE Healthcare
Simone Schiaffino: Speaker: GE Healthcare; Other: Bracco Imaging Other: Arterys Inc.
Gianfranco Paride Scaperrotta: Nothing to disclose
Caterine Depretto: Nothing to disclose
Jonathan James: Nothing to disclose

### RPS 802-10

### Nipple sparing mastectomy: can CEM predict the nipple–areola complex (NAC) involvement?

*G. Migliaro*, C. Bellini, G. Bicchierai, F. Di Naro, D. De Benedetto, F. Pugliese, L. A. Incardona, V. Miele, J. Nori; Florence/IT
*(giuliano.migliaro@gmail.com)*

**Purpose:** The purpose of this study was to assess the role of Contrast-Enhanced Mammography (CEM) as a predictor of invasion of the NAC in patients undergoing nipple-sparing mastectomy (NSM).
**Methods or Backround:** In our retrospective study, three radiologists reviewed 1479 presurgical CEMs of patients who underwent NSM for biopsy proven malignant lesions, conducted at our centre between 2018 and 2019, in consensus. We included 64 CEMs of 64 patients; we evaluated the TND (tumour-nipple distance) dividing patients according to the shortest TND seen either in CC or in MLO. The correct extension of the disease, the enhancement type, and the enhancing conspicuity were described for every lesion. Sensitivity, specificity, negative, and positive predictive values of CEM for nipple invasion (PPV and NPV) were calculated. Our gold standard was the final histology of the surgical specimen.
**Results or Findings:** The patients were beween 38 and 84 years old (mean age 56.9, median age 52). The shortest TND between the two projections varied from 0mm to 75mm. Among the lesions with TND≤10mm (25/64), 19/25 (76%) resulted as invading the NAC while 6/25 did not; three had a low enhancement conspicuity, and 22 had a moderate or high conspicuity. Amid the lesions with TND>10mm (39/64), only 7/39 (18%) invaded the NAC. Conversely, 32/39 did not; five had a low enhancement conspicuity and 34 had a moderate or high conspicuity. Comparing the two groups of lesions, satisfying NPV (82.1%) and specificity (84.2%) emerged and acceptable VPP and sensitivity (76% and 73%) were reported. No statistical significance emerged.
**Conclusion:** CEM is a useful tool that can help predict the NAC invasion in pre-surgical assessments, excluding when it is not infiltrated due to the high NPV and specificity of this series.
**Limitations:** The study is limited by the small number of patients included.
**Ethics committee approval:** This is a retrospective study.
**Funding for this study:** No funding was received for this study.
**Author Disclosures:**
Giulia Bicchierai: Nothing to disclose
Francesca Pugliese: Nothing to disclose
Diego De Benedetto: Nothing to disclose
Jacopo Nori: Nothing to disclose
Vittorio Miele: Nothing to disclose
Chiara Bellini: Nothing to disclose
Giuliano Migliaro: Nothing to disclose
Ludovica Anna Incardona: Nothing to disclose
Federica Di Naro: Nothing to disclose

### RPS 802-11

### Is late-phase contrast-enhanced mammography after neoadjuvant therapy useful?

*D. Oral*, C. R. Acar, A. B. Kale, O. Acar, I. Ş. S. Örgüç; Manisa/TR
*(dilsahoral@gmail.com)*

**Purpose:** This study aims to evaluate the need for late-phase contrast-enhanced mammography (CEM) in assessing post-treatment residual tumor in breast cancer patients undergoing neoadjuvant therapy (NAT).
**Methods or Backround:** In this prospective study, 61 patients were included. CEM examinations, comprising both early and late-phase mediolateral oblique images, were conducted before and after NAT. During the evaluation of CEM images, we separately noted the level of enhancement and the size of index malignant lesions for both phases. Findings were compared with post-surgical pathology reports.
**Results or Findings:** Residual lesions were detected in 40 (65.57%) patients in pathology. The sensitivity of both early and late-phase images in determining complete response was 90.47%. While the specificity was 55% in the early phase, it increased to 65% in the late phase. Area under curve was 0.727 and 0.777 for early and late-phase images, respectively in ROC analysis. In the correlation analysis for residual lesion size, a significant correlation (p<0.01) was observed between pathological results and both images. Notably, late-phase images exhibited a high degree of correlation, with an R-value of 0.887 compared to 0.867 for the early-phase images. Among 23 patients, when comparing late-phase images to early-phase images, higher contrast enhancement levels were observed in four (17%) lesions, an increase in size in twelve (52%) lesions, and both phenomena in seven (30%) lesions. Among the 19 lesions that exhibited larger residual lesion sizes in late-phase images, 18 of them were more closely aligned with pathological results and one was in complete response in pathological reports.
**Conclusion:** In our study, late-phase images provided better results in determining the residual tumor compared to the early phase in 29.50% of the patients.
**Limitations:** No limitations were identified.
**Ethics committee approval:** This study was approved by the Clinical Research Ethics Committee of Manisa Celal Bayar University (Decision Number : E-85252386-050.04.04-200753).
**Funding for this study:** No funding was received for this study.
**Author Disclosures:**
Ihsan Şebnem Sebnem Örgüç: Nothing to disclose
Omer Acar: Nothing to disclose
Ahmet Burak Kale: Nothing to disclose
Dilşah Oral: Nothing to disclose
Cagdas Riza Acar: Nothing to disclose

### RPS 802-12

### Inter-reader agreement between breast imagers using the first version of the BI-RADS CEM lexicon

*C. Zarcaro*[1], A. Santonocito[2], L. Zeitouni[3], F. Ferrara[4], P. Kapetas[5], R-I. Milos[5], T. H. Helbich[5], P. A. Baltzer[5], P. Clauser[5]; [1]Palermo/IT, [2]Turin/IT, [3]Riyadh/SA, [4]Rome/IT, [5]Vienna/AT

**Purpose:** The aim of this study was to evaluate the inter-reader agreement of the Contrast Enhanced Mammography (CEM) lexicon, a new addition to the 5th edition of the BI-RADS Mammography lexicon.
**Methods or Backround:** Three breast imaging fellows reviewed 462 lesions in 421 routine clinical breast CEM in a IRB-approved, single-center, retrospective study using the first version of the CEM BI-RADS lexicon and the fifth edition of the BI-RADS Mammography lexicon. Blinded to patient outcomes, the readers assessed breast and lesion features on low-energy (LE) images (ACR breast density, type of lesion, associated architectural distortion), lesion features in recombined (RC) images (type of enhancement, lesion conspicuity, mass shape, mass margin, mass internal pattern of enhancement, non-mass distribution, non-mass internal pattern of enhancement, enhancing asymmetry), and provided a final BI-RADS assessment. Inter-reader agreement was calculated for each feature using Fleiss' and Cohen's kappa coefficient. Sensitivity and specificity were calculated.
**Results or Findings:** On both LE and RC images, there was moderate to substantial inter-reader agreement for breast density and many lesion features, particularly for type of lesion ($\kappa$=0.654) and type of enhancement ($\kappa$=0.664). Agreement was moderate to substantial also on CEM mass enhancement descriptors, while it was fair to moderate for non-mass enhancement and enhancing asymmetry descriptors. Inter-reader agreement for final LE and CEM BI-RADS assessment was moderate ($\kappa$=0.421) and fair ($\kappa$=0.364). Sensitivity and specificity were high, although specificity was more variable.
**Conclusion:** On both LE and RC images, there was moderate to substantial inter-reader agreement for the assessment of breast density and many imaging findings. Agreement was moderate to substantial on CEM mass enhancement, while it was fair to moderate for non-mass enhancement and enhancing asymmetry descriptors.
**Limitations:** This was a single-centre study.

**Ethics committee approval:** This study was approved by the relevant Institutional Review Board (IRB).
**Funding for this study:** No funding was received for this study.
**Author Disclosures:**
Pascal A.T. Baltzer: Nothing to disclose
Calogero Zarcaro: Nothing to disclose
Francesca Ferrara: Nothing to disclose
Thomas H. Helbich: Nothing to disclose
Ruxandra-Iulia Milos: Nothing to disclose
Layla Zeitouni: Nothing to disclose
Ambra Santonocito: Nothing to disclose
Panagiotis Kapetas: Nothing to disclose
Paola Clauser: Nothing to disclose

### RPS 802-13

**Preoperative assessment of nipple-areolar complex infiltration: is CEM as reliable as breast MRI?**
*S. Sanità*, M. Lorenzon, M. Cirillo, L. Cereser, R. Girometti, C. Zuiani; Udine/IT

**Purpose:** The purpose of this study was to compare sensitivity and specificity of Contrast Enhanced Mammography (CEM) and breast Magnetic Resonance Imaging (MRI) in the assessment of the infiltration of nipple-areolar complex (NAC) in pre-operative assessment.
**Methods or Backround:** We retrospectively included all patients over the age of 18 with newly diagnosed, biopsy-proven breast cancer, who underwent either CEM or 1.5T MRI between January 2022 and December 2022 for preoperative assessment in our centre. Exclusion criteria were neoadjuvant chemotherapy, and when a pathological analysis of the surgical specimen was not available. Enhancing NAC were considered infiltrated with both techniques. We calculated and compared CEM and MRI sensitivity, specificity, PPV, and NPV for the infiltration of NAC, considering the surgical specimen as the standard of reference. The statistical significance level used was $P<0.05$.
**Results or Findings:** After assessment of 168 MRI and 146 CEM, we included 109 MRI and 108 CEM. 23 MRI-patients and 13 CEM-patients had NAC infiltration at pathological analysis. Contrast enhancement of NAC was detected in 28/109 MRI and 15/108 CEM. The sensitivity, specificity, PPV, and NPV of MRI versus CEM were 92% (C.I. 74-99%), 94.05% (C.I. 87-98%), 82.14% (C.I. 66-92%), 97.53% (C.I. 91-99%) versus 92.86% (C.I. 66-99%), 97.87% (C.I. 93-99%), 86.67% (C.I. 62-96%), and 98.92% (C.I. 93-99%) respectively. Differences among MRI and CEM were not statistically significant with sensitivity $\Delta=0.860\%$, $p=0.812$; specificity $\Delta=3.820\%$; $p=0.154$; PPV $\Delta=4.530\%$; $p=0.359$, and NPV $\Delta=1.390\%$; $p=0.439$.
**Conclusion:** CEM seems to be as reliable as MRI in the assessment of infiltration of NAC.
**Limitations:** The study was monocentric and retrospective, with a limited number of patients.
**Ethics committee approval:** No information provided by the submitter.
**Funding for this study:** No funding was received for this study.
**Author Disclosures:**
Chiara Zuiani: Nothing to disclose
Silvia Sanità: Nothing to disclose
Michele Lorenzon: Nothing to disclose
Rossano Girometti: Nothing to disclose
Mariachiara Cirillo: Nothing to disclose
Lorenzo Cereser: Nothing to disclose

**09:30-11:00** **Research Stage 3**

### Research Presentation Session: Neuro

### RPS 811

### Foetal and neonatal neuroimaging: epilepsy and the senses

**Moderator**
C. Deuschl; Essen/DE
*(cornelius.deuschl@uk-essen.de)*

### RPS 811-2

**One-minute full brain multi-contrast MRI vs brain CT in paediatric patients in the elective setting: a prospective feasibility study**
*F. De Luca*, A. Kits, D. Martin Muñoz, A. Aspelin, O. Kvist, Y. Osterman, S. Diaz Ruiz, S. Skare, A. Falk Delgado; Stockholm/SE

**Purpose:** The radiological examination of paediatric patients with suspected cerebral pathology is performed at first instance with brain CT when MRI resources and general anaesthesia are unavailable. The study aimed to evaluate the tolerance to a complete diagnostic fast brain MRI (EPIMix) by paediatric patients without general anaesthesia and to compare the diagnostic accuracy of EPIMix and brain CT.
**Methods or Backround:** Paediatric patients with a referral for elective brain CT between March 2019 and March 2020 were prospectively included and underwent EPIMix and brain CT without anaesthesia. Three readers evaluated EPIMix and CT image datasets separately, blinded from any clinical information. The study outcomes were to assess the tolerance to a complete diagnostic EPIMix sequence without general anaesthesia and the diagnostic performance in classifying scans as non-pathological or pathological. The two image modalities were compared using descriptive statistics and Fisher's exact test. Furthermore, a side-by-side evaluation of EPIMIx and CT image datasets was performed by two additional unblinded readers and one previously blinded reader.
**Results or Findings:** All included paediatric patients (n=15) tolerated EPIMix well. The EPIMIx and CT scans were reported as non-pathological in 13 and 12 cases, respectively, by the three readers, while two cases of EPIMix and three cases of CT were classified as pathological by one reader (Fisher's exact test, reader 1–3 p=1.00). Side-by-side evaluation reported all scans as non-pathological on both EPIMIx and CT image datasets.
**Conclusion:** Paediatric patients tolerated the diagnostic fast brain MRI EPIMix without the need for general anaesthesia. Further, EPIMix showed a comparable diagnostic performance to brain CT. An article from this study has been published in BMC Medical imaging. The Version of Record of this article is published in BMC Medical Imaging and is available online at https://doi.org/10.1186/s12880-024-01196-6
**Limitations:** The three blinded readers evaluating the images had varied experiences. However, there were no significant differences in scan classification between EPIMix and CT, which enhances the applicability of our findings.
**Ethics committee approval:** This study was approved by the Swedish Ethical Review Authority.
**Funding for this study:** Funding for the authors of this study was received from Stockholms Läns Landsting. Funding was also received from the Svenska Sällskapet för Medicinsk Forskning and Karolinska Institutet.
**Author Disclosures:**
Anna Falk Delgado: Nothing to disclose
Asa Aspelin: Nothing to disclose
Francesca De Luca: Nothing to disclose
Annika Kits: Nothing to disclose
Stefan Skare: Nothing to disclose
Ola Kvist: Nothing to disclose
Daniel Martin Muñoz: Nothing to disclose
Yords Osterman: Nothing to disclose
Sandra Diaz Ruiz: Nothing to disclose

### RPS 811-3

**Diffusion tensor magnetic resonance neuroimaging metrics of neonates with intraventricular haemorrhages predict neurodevelopmental outcome**
*S. Seeliger*, P. Kienast, V. Schmidbauer, K. Goeral, J. Elis, D. Prayer, G. Kasprian; Vienna/AT

**Purpose:** Intraventricular haemorrhages (IVH) are a major cause of neurodevelopmental impairment in premature neonates. Approximately 15-20% of neonates delivered prior to reaching 32 weeks of gestation (GW) suffer from IVH. This study aims to identify new noninvasive markers based on diffusion tensor magnetic resonance imaging (DTI) to accurately predict neurodevelopmental outcomes in preterm infants with IVH.
**Methods or Backround:** Neuroimaging was performed on 41 (n=15, 37% female) preterm neonates (mean GW 26,34 ± 2.6 weeks SD at the time of birth) with IVH using DTI. Regions of interest (ROIs) were placed in early myelinating structures. Fractional anisotropy (FA) and apparent diffusion coefficient (ADC) were measured in the set ROIs. Neonatal outcome scores were calculated using the Bayley Scales of Infant and Toddler Development conducted at 12 months and 24 months corrected age. Statistical analyses, including Pearson's correlations and ANOVA, were performed to establish correlations between DTI metrics and neurodevelopmental outcomes.
**Results or Findings:** FA of the medulla oblongata showed a highly significant correlation with the one-year outcome ($r=-0.559$, $p=0.004$). FA of the right internal capsule showed a significant correlation between one-year outcome ($r=-0.404$, $p=0.045$) and two-year language outcome ($r=-0.658$, $p=0.020$). FA of the left internal capsule showed highly significant correlations between cognitive ($r=-0.525$, $p=0.007$), motor ($r=-0.637$, $p<0.001$), and motor outcome ($r=-0.527$, $p=0.007$) after 12 months corrected age.
**Conclusion:** DTI-based MRI provides valuable prognostic information on future neurodevelopmental outcomes after IVH. DTI enhances the sensitivity of neonatal MR neuroimaging and could support clinical decision-making for these vulnerable patients.
**Limitations:** No limitations were identified.
**Ethics committee approval:** The study was approved with the approval code: EK 2455/2020.

**Funding for this study:** Funding for the authors of this study, Patric Kienast and Julia Elis, was provided by the Vienna Science and Technology Fund (WWTF; LS20-030, PIMIENTO).
**Author Disclosures:**
Victor Schmidbauer: Nothing to disclose
Julia Elis: Nothing to disclose
Daniela Prayer: Nothing to disclose
Patric Kienast: Nothing to disclose
Selina Seeliger: Nothing to disclose
Katharina Goeral: Nothing to disclose
Gregor Kasprian: Nothing to disclose

### RPS 811-4

**Quantitative synthetic MRI- vs MR-fingerprinting-based relaxometry of foetal brainstem myelination: a post-mortem imaging study**

V. Schmidbauer, *J. Malik*, I-V. A. Malla Houech, J. Binder, E. Gelpi, D. Prayer, G. Kasprian; Vienna/AT
*(malik.jakobs@gmail.com)*

**Purpose:** Novel quantitative magnetic resonance imaging (MRI) modalities provide relaxometric properties that are linked to myelinogenesis. The aim of this work was to investigate ongoing myelin development in foetuses based on a quantitative post-mortem imaging approach using Synthetic MRI- and MR-Fingerprinting (MRF). Furthermore, the results of both modalities were compared.
**Methods or Backround:** In 26 cases, quantitative post-mortem foetal MR data were available. Relaxometric measurements (T1-/T2-relexation times (T1R/T2R)) were determined in the medulla oblongata and the midbrain using Synthetic MRI-/MRF-specific post-processing procedures. Pearson's correlations were applied to detect relationships between T1R/T2R metrics and gestational age (GA) at MRI. Intra-class correlation coefficients were calculated to proof the consistency of the results provided by both modalities.
**Results or Findings:** Both modalities provided quantitative data that revealed negative correlations with GA at MRI: Synthetic MRI-derived T1R (medulla oblongata ($r=-0.459$; $p=0.021$); midbrain ($r=-0.413$; $p=0.040$)) and T2R (medulla oblongata ($r=-0.625$; $p<0.001$); midbrain ($r=-0.571$; $p=0.003$)) versus MRF-derived T1R (medulla oblongata ($r=-0.433$; $p=0.035$); midbrain ($r=-0.386$; $p=0.062$)) and T2R (medulla oblongata ($r=-0.883$; $p<0.001$); midbrain ($r=-0.890$; $p<0.001$)). Results consistency between both MR approaches ranged between 0.661; confidence interval (CI: 0.351 – 0.841) (T2R: medulla oblongata) and 0.920 (CI: 0.821 – 0.965) (T1R: midbrain).
**Conclusion:** Synthetic MRI and MRF provide consistent results. Both modalities hold high sensitivity to quantify ongoing myelinogenesis prenatally. Therefore, quantitative MRI holds promising potential for the assessment of biochemical aspects of brain maturation in the second half of pregnancy.
**Limitations:** The study was limited by its small sample size.
**Ethics committee approval:** This study received ethical approval with the code: 1585/2023.
**Funding for this study:** No funding was received for this study.
**Author Disclosures:**
Victor Schmidbauer: Nothing to disclose
Julia Binder: Nothing to disclose
Jakob Malik: Nothing to disclose
Intesar-Victoria Aiman Malla Houech: Nothing to disclose
Daniela Prayer: Nothing to disclose
Gregor Kasprian: Nothing to disclose
Ellen Gelpi: Nothing to disclose

### RPS 811-5

**The visibility of the indusium griseum on foetal MRI postmortem and in vivo**

*I. Pogledic*[1], M. Bobić-Rasonja[2], C. Mitter[1], A. Štajduhar[2], E. Schwartz[1], G. Kasprian[1], M. Judaš[2], D. Prayer[1], N. Jovanov-Milošević[2]; [1]Vienna/AT, [2]Zagreb/HR
*(ivana.pogledic@meduniwien.ac.at)*

**Purpose:** The study aimed to assess the visibility of the indusium griseum (IG) on MR scans of the foetal human brain and to evaluate its reliability as an imaging biomarker of the regularity of brain midline development.
**Methods or Backround:** The retrospective observational study encompassed T2-w 3T MR images postmortem from ninety foetal brains and immunohistochemical sections from 41 foetal brains with regular brain development. Three raters independently evaluated the visibility of IG, and weighted kappa statistics and regression analysis were used for visibility evaluation.
**Results or Findings:** The visibility of the IG was the highest between the 25 and 30 GW period (inter-rater kappa 0.623-0.709) and excellent intra-rater variability (kappa 0.81 – 0.93). The immunochemical analysis of the IG discloses the expression of highly hydrated extracellular molecules in IG as the substrate of higher signal intensity and the best visibility of IG during the mid-foetal period. The IG anatomic position above the callosal trunk could influence the measurements of CC thickness when imaging methods are used at its developmental peak. Super-resolution imaging modalities may improve the visibility of the IG in in vivo MRI in the near future, strengthening the value of IG as a biomarker.
**Conclusion:** The knowledge of developmental brain histology and foetal age allows us to predict the IG-visibility in MRI and use it as a biomarker to evaluate the morphogenesis of the brain midline. IG is particularly interesting as a biomarker in postmortem pathological examination by MRI.
**Limitations:** The number of encompassed cases due to the unavailability of MRI scans without brain pathologies is the main limitation of this study; therefore multicentric foetal MR studies, with more cases included, could elucidate the role of IG in health and disease and its prognostic value.
**Ethics committee approval:** Approval for the histological part of the study was given by the Ethics Committee of the University of Zagreb, School of Medicine, following the Declaration of Helsinki (No.: 380-59-10106-19-111/210 class:641-01/19-02/01). The MRI part of the study was approved by the Ethics Committee of the Medical School of the University of Vienna (EK 1211/2019).
**Funding for this study:** Funding was received from the Croatian Science funds: IP-2019-04-3182, DOK-01-3771, DOK-02-5988); University of Zagreb School of Medicine Funds: (10106-22-3116; 10106-23-2487), and by the European Regional Development Fund: "Experimental and clinical research of hypoxic-ischemic damage in perinatal and adult brain" (GA KK01.1.1.01.0007).
**Author Disclosures:**
Nataša Jovanov-Milošević: Nothing to disclose
Christian Mitter: Nothing to disclose
Mihaela Bobić-Rasonja: Nothing to disclose
Andrija Štajduhar: Nothing to disclose
Ernst Schwartz: Nothing to disclose
Miloš Judaš: Nothing to disclose
Daniela Prayer: Nothing to disclose
Ivana Pogledic: Nothing to disclose
Gregor Kasprian: Nothing to disclose

### RPS 811-6

**Regional cortical thinning and aberrant cortical folding in sleep-related hypermotor epilepsy**

*H. Pu*, X. Su, S. Zhang, Q. Yue, Q. Gong; Chengdu/CN
*(phxia0409@126.com)*

**Purpose:** Sleep-related hypermotor epilepsy (SHE) is a focal epilepsy whose neurobiological factors such as cortical surface structure alterations remain poorly understood. The objective of this study was to investigate the neuromorphometric abnormalities and their relationship with clinical characteristics in SHE patients using structural magnetic resonance imaging (MRI).
**Methods or Backround:** 69 patients with SHE and 69 healthy controls (HCs) were prospectively recruited and underwent high-resolution T1-weighted MRI scanning. The surface-based morphometry (SBM) analysis based on Computational Anatomy Toolbox (CAT12) was used to evaluate structural parameters such as cortical thickness, fractal dimension, gyrification, and sulcal depth. The two groups were compared with two-sample t-tests ($p<0.05$, family-wise error (FWE) corrected at cluster level). The association between the cortical morphological changes in local brain regions and the epilepsy duration or the seizure frequency was explored with correlation analysis.
**Results or Findings:** SHE patients exhibited significantly decreased cortical thickness in the left inferior-parietal, right superior-parietal, bilateral lateral-occipital cortices, left fusiform, and lingual gyri ($p=0.00001$ - $0.00004$), and decreased sulcal depth in the right precentral and postcentral gyri ($p\leq0.00001$) compared to HCs . However, there were no statistically significant differences in the fractal dimension or gyrification between SHE and HCs (all $P>0.05$). Correlation analysis showed a negative correlation between the left inferior-parietal cortical thickness alteration and the duration of SHE ($r=-0.325$, $p=0.006$).
**Conclusion:** Our study revealed that cortical thinning and aberrant folding of local cerebral cortices existed in SHE and may be associated with clinical hypermotor semiology. These findings might provide a foundation for understanding the epilepsy network disturbances and the underlying neuropathologic mechanism of SHE.
**Limitations:** This study used a cross-sectional design with a relatively small sample size. Some patients received anti-epileptic drugs, which may impact the results.
**Ethics committee approval:** This study was approved by the local Ethical Committee of West China Hospital, Sichuan University.
**Funding for this study:** Funding was received from the National Natural Science Foundation of China (Grant No. 82271961).
**Author Disclosures:**
Qiyong Gong: Nothing to disclose
Xiaorui Su: Nothing to disclose
Qiang Yue: Nothing to disclose
Simin Zhang: Nothing to disclose
Huaxia Pu: Nothing to disclose

### RPS 811-7

**Task-negative brain activity as an indicator of task engagement in preoperative temporal lobe epilepsy patients: a functional MRI study**

*R. Stepponat*, M. S. Yildirim, J. Leinkauf, F. Fischmeister, S. Bonelli, G. Kasprian; Vienna/AT
*(radheshyam.stepponat@meduniwien.ac.at)*

**Purpose:** This study aimed to analyse the relationship between task-negative and task-positive brain activity, focusing on the stability of the default mode network during verb-generation and sentence-comprehension and its relationship to language lateralisation and task-engagement.
**Methods or Backround:** In clinical practice, fMRI is mainly used for preoperative language lateralisation assessment, commonly employing verb-generation (VG) and sentence-comprehension (SC) tasks. Language lateralisation is determined by calculating the lateralisation index (LI) for task-positive brain activity, but variability in task performance can lead to uncertain results. Twenty-three patients (12 male, 11 female), averaging 28.6 years, with lesional (n=13) and MR-negative (n=10) temporal lobe epilepsy underwent preoperative fMRI. Standard preprocessing was conducted. The LI toolbox was used to assess temporal lobe lateralisation for the task-active contrast. Region of interest (ROI) analysis of the posterior cingulate cortex (PCC, Brodmann-area 23 and 31) at a threshold of $p \leq 0.01$ was conducted for the task-negative contrast.
**Results or Findings:** Sixteen patients displayed consistent temporal lobe lateralisation: 13 left and 3 bilateral lateralization, while seven patients exhibited varying lateralisation between tasks. In VG task-negative ROI analysis, four patients lacked PCC activation, while others showed left (n=3), right (n=3), or bilateral (n=13) activation. In SC all but one patient displayed bilateral PCC activation. Divergent lateralisation between tasks was significantly associated with PCC inactivity in VG task-negative ROI analysis using X2 (1, N=23)= 4.5, p=0.033.
**Conclusion:** The absence of PCC activation during VG may be indicative of incompliance, epilepsy-associated DMN changes, and cognitive impairment due to epilepsy, all of which may limit the clinical usability of the data. Further functional connectome subgroup analysis is underway.
**Limitations:** ROI analysis conducted in the study was limited to PCC. The study's sample size is a further limitation; a larger sample size may yield more significant correlations. Task-compliance was also only partially documented.
**Ethics committee approval:** This study was approved by the Medical University of Vienna, EK: 1141/2023.
**Funding for this study:** No funding was received for this study, which was conducted as part of a PhD-thesis.
**Author Disclosures:**
Mehmet Salih Yildirim: Nothing to disclose
Joel Leinkauf: Nothing to disclose
Florian Fischmeister: Nothing to disclose
Silvia Bonelli: Nothing to disclose
Radheshyam Stepponat: Nothing to disclose
Gregor Kasprian: Nothing to disclose

### RPS 811-8

**Altered neurovascular coupling in thyroid-associated ophthalmopathy: a combined resting-state fMRI and arterial spin labelling study**

*W. Chen*[1], H. Hu[2], X-Q. Xu[2], F-Y. Wu[2]; [1]Suzhou/CN, [2]Nanjing/CN
*(chenwen1994@foxmail.com)*

**Purpose:** Besides the well-documented ophthalmic manifestations, thyroid-associated ophthalmopathy (TAO) is believed to be related to emotional and psychological abnormalities. Given the previous neuroimaging evidence, we hypothesised that TAO patients would have altered neurovascular coupling associated with clinical-psychiatric disturbances. The purpose of this study was to investigate neurovascular coupling changes in TAO by combining resting-state functional magnetic resonance imaging (rs-fMRI) and arterial spin labelling (ASL) techniques.
**Methods or Backround:** Amplitude of low-frequency fluctuation (ALFF) was calculated from rs-fMRI (evaluating neuronal activity), and cerebral blood flow (CBF) was computed from ASL (evaluating vascular response) in 37 TAO patients and 21 healthy controls (HCs). Global neurovascular coupling was assessed by across-voxel CBF-ALFF correlation, and regional neurovascular coupling was evaluated by CBF/ALFF ratio. Auxiliary analyses were performed by using fractional amplitude of low-frequency fluctuation (fALFF) and regional homogeneity (ReHo) as rs-fMRI measures.
**Results or Findings:** TAO patients showed significantly reduced global CBF-ALFF coupling compared with HCs. Moreover, TAO patients exhibited decreased CBF/ALFF ratio in the left lingual gyrus (LG)/fusiform gyrus (FFG), and increased CBF/ALFF ratio in the bilateral precuneus (PCu). In TAOs, CBF/ALFF ratio in the left LG/FFG was positively correlated with visual acuity, whilst CBF/ALFF ratio in the bilateral PCu was negatively correlated with MoCA score. The auxiliary analyses showed trends of reduced global neurovascular coupling (i.e. CBF-fALFF correlation and CBF-ReHo correlation), as well as significant altered regional neurovascular coupling (i.e. CBF/fALFF ratio and CBF/ReHo ratio) in several brain regions.
**Conclusion:** TAO patients had altered neurovascular coupling in the visual and higher order cognitive cortices. The neurovascular decoupling might be a possible neuropathological mechanism of TAO.
**Limitations:** The sample size was relatively small, and the cross-sectional design may limit the evaluations of brain changes along with the development of the disease.
**Ethics committee approval:** This study was approved by the Ethics Committee of the First Affiliated Hospital of Nanjing Medical University (ref: 2021-SRFA-024).
**Funding for this study:** Funding was received from the National Natural Science Foundation of China (NSFC) (81801659 to Hao Hu) and Clinical Capability Promotion Project of Jiangsu Province Hospital (to Xiao-Quan Xu).
**Author Disclosures:**
Fei-Yun Wu: Nothing to disclose
Xiao-Quan Xu: Nothing to disclose
Wen Chen: Nothing to disclose
Hao Hu: Nothing to disclose

### RPS 811-9

**Structural and functional brain changes after glucocorticoid therapy in thyroid-associated ophthalmopathy**

*Q. Wu*, J. Zhou, W-H. Jiang, X-Y. Pu, X-Q. Xu, H. Hu, F-Y. Wu; Nanjing/CN
*(koalaw1994@163.com)*

**Purpose:** The purpose of this study was to investigate the brain structural and functional alterations in patients with thyroid-associated ophthalmopathy (TAO) before and after glucocorticoid therapy, using voxel-based morphometry (VBM) as well as resting-state functional magnetic resonance imaging (MRI) with amplitude of low-frequency fluctuation (ALFF) and regional homogeneity (ReHo).
**Methods or Backround:** Between 2019 and 2022, 32 patients with TAO and 23 healthy controls were recruited to undergo pre-therapy MRI scans. Intravenous glucocorticoid therapy was administered to all patients. Twenty-six of the patients were available for rescanned MRI three months after the end of therapy. The VBM, ALFF and ReHo methods were used to evaluate the brain structural and functional differences.
**Results or Findings:** Before therapy, TAO patients showed significantly decreased grey matter volume in the left orbital part of superior frontal gyrus (ORBsup) and the medial superior frontal gyrus (SFGmed) than healthy controls. Patients had higher ALFF values in bilateral gyrus rectus and olfactory cortex as well as lower values in bilateral cuneus. The patients also showed decreased ReHo values in bilateral lingual gyrus. After therapy, increased grey matter volume in left anterior cingulate gyrus and SFGmed, increased ALFF values in bilateral cuneus and superior occipital gyrus, as well as increased ReHo values in bilateral SFGmed were found in TAO patients compared to the pre-therapy cohort. Compared to controls, decreased grey matter volume in the left ORBsup was observed in post-therapy TAO patients.
**Conclusion:** Our results indicated that TAO might cause functional and structural deficits in the visual and emotional regions of the brain, with recovery in the former and partial restoration in the latter after effective glucocorticoid therapy. These findings may lead to a deeper understanding of the pathophysiological mechanism behind TAO.
**Limitations:** The sample size was relatively small.
**Ethics committee approval:** This study was approved by the Review Board of the First Affiliated Hospital, Nanjing Medical University.
**Funding for this study:** Funding was received from the National and Natural Science Foundation of China (NSFC) (81801659 to Hao Hu), Jiangsu Province Capability Improvement Project through Science, Technology and Education (JSDW202243 to Fei-Yun Wu), and Jiangsu Province Hospital (the First Affiliated Hospital, Nanjing Medical University) Clinical Capability Enhancement Project (JSPH-MC-2021-8 to Xiao-Quan Xu).
**Author Disclosures:**
Fei-Yun Wu: Nothing to disclose
Jiang Zhou: Nothing to disclose
Qian Wu: Nothing to disclose
Xiao-Quan Xu: Nothing to disclose
Wen-Hao Jiang: Nothing to disclose
Hao Hu: Nothing to disclose
Xiong-Ying Pu: Nothing to disclose

### RPS 811-10

**The neural correlates of hearing loss-related distress: an fMRI study**

*F. Aldhafeeri*; Hafar Albatin/SA
*(aldhafeeri8@hotmail.com)*

**Purpose:** Hearing loss (HL) is associated with a decline in the ability to hear high-frequency sounds, resulting in impaired speech comprehension, particularly under adverse listening conditions. The presence of hearing loss in older adults has been found to have a substantial influence on their overall quality of life and general well-being. Additionally, there is evidence suggesting a potential correlation between HL and the development of depressive symptoms as well as feelings of social isolation. The purpose of this study was to examine how hearing impairment affects the brain's emotional processing network.
**Methods or Backround:** A total of 16 subjects with hearing loss and 15 age and gender-matched healthy controls were recruited in this study. All participants underwent functional magnetic resonance imaging (fMRI) during which they were asked to listen to emotionally evocative sound clips. These were rated as pleasant unpleasant or neutral. The sounds were obtained from the International Affective Digitised Sounds (IADS). To examine the impact of hearing loss severity on neuronal activity, a correlation analysis between the mean fMRI blood oxygen level-dependent (BOLD) signal and the hearing thresholds in both groups were performed.
**Results or Findings:** Compared to the healthy control group, HL subjects demonstrated exaggerated brain activation in auditory, limbic and frontal regions. HL group exhibited a positive correlation between the mean fMRI BOLD signal and hearing threshold in the right superior temporal gyrus and anterior cingulate.
**Conclusion:** Current findings indicate that hearing loss leads to neuronal reorganisation, particularly in brain networks that are involved in the processing of emotions.
**Limitations:** This study is limited by a relatively small number of subjects. The contribution of the scanner's noise with the auditory stimuli is a further limitation.
**Ethics committee approval:** This study was approved by the University's Local Research Ethics Committee.
**Funding for this study:** No funding was received for this study.
**Author Disclosures:**
Faten Aldhafeeri: Nothing to disclose

### RPS 811-11
**Causal associations of genetically determined tinnitus with neuroimaging traits: evidence from a Mendelian randomisation study**
*J. Sun*, H. Lv, Z. Wang; Beijing/CN
*(samantha_sunjing@163.com)*

**Purpose:** Potential reverse causality and unmeasured confounding factors are common biases in most neuroimaging studies on tinnitus and central correlates. The causal association of tinnitus with neuroimaging features also remains unclear. This study aimed to investigate the causal relationship of tinnitus with neuroplastic alterations using Mendelian randomisation (MR).
**Methods or Backround:** Summary-level data from a genome-wide association study (GWAS) of tinnitus were derived from UK Biobank (n=117,882). The GWAS summary statistics for four global-brain tissues and 14 sub-brain gray matter volumetric traits were also obtained (n=up to 33,224). A bidirectional MR analysis was conducted to explore the causal relationship between tinnitus and neuroanatomical features at global-brain and sub-brain levels.
**Results or Findings:** Genetic susceptibility to tinnitus was causally associated with increased white matter volume (OR=2.361, 95% CI 1.033–5.393) and total brain volume (OR=2.391, 95% CI 1.047–5.463) but inversely associated with cerebrospinal fluid volume (OR=0.362, 95% CI 0.158–0.826). A smaller grey matter volume in the left Heschl's gyrus and right insular cortex and larger grey matter volume in the posterior division of the left parahippocampal gyrus may lead to an increased risk for tinnitus (OR=0.978, 95% CI 0.961–0.996; OR=0.987, 95% CI 0.976–0.998; and OR=1.015, 95% CI 1.001–1.028, respectively).
**Conclusion:** Genetic susceptibility to tinnitus was causally associated with increased white matter volume and total brain volume. Volume alteration in several cortical regions may indicate a higher tinnitus risk, and further research is recommended for causality inference at the level of sub-brain regions. Our findings provide genetic evidence for elucidating the underlying pathophysiological mechanisms of tinnitus-related neuroanatomical abnormalities.
**Limitations:** The difference in the genetic background between different ancestries limits the broader application of our findings.
**Ethics committee approval:** This study was based on publicly available summary data, and ethical approval of each GWAS study was obtained from the relevant institutional review board.
**Funding for this study:** Funding was received as part of Grant 62171297 (H.L.) from the National Natural Science Foundation of China, No. ZYLX202101 from Beijing Hospitals Authority Clinical Medicine Development of Special Funding Support, No. 2021-135 from Beijing Municipal Health Commission-Beijing Key Clinical Discipline Funding.
**Author Disclosures:**
ZhenChang Wang: Nothing to disclose
Han Lv: Nothing to disclose
Jing Sun: Nothing to disclose

### RPS 811-12
**Multimodal quantitative magnetic resonance imaging of the thalamus supports distinct outcomes in tinnitus patients treated with sound therapy**
*Q. Chen*; Beijing/CN
*(chenqian8319@163.com)*

**Purpose:** The objective of this study was to systematically investigate structural and functional alterations in the thalamus and its subregions using multimodal magnetic resonance (MR) imaging and examine its clinical relevance in tinnitus patients with different outcomes after sound therapy (narrowband noise).
**Methods or Backround:** In total, 60 patients with persistent tinnitus and 57 healthy controls (HCs) were recruited. Based on treatment efficacy, 28 patients were categorised into the effective group and 32 into the ineffective group. Five MR imaging measurements of the thalamus and its seven subregions, including gray matter volume, fractional anisotropy, fractional amplitude of low-frequency fluctuation, and functional connectivity (FC), were obtained for each participant and compared between the groups.
**Results or Findings:** Patients in both groups exhibited widespread functional and diffusion abnormalities in the whole thalamus and several subregions, with more obvious changes observed in the effective group. All tinnitus patients had abnormal FC compared with the HCs; FC differences between the two patient groups were only observed in the striatal network, auditory-related cortex, and the core area of the limbic system. We combined the multimodal quantitative thalamic alterations and used it as an imaging indicator to evaluate prognosis before sound therapy and achieved a sensitivity of 71.9% and specificity of 85.7%.
**Conclusion:** Similar patterns of thalamic alterations were identified in tinnitus patients with different outcomes, with more obvious changes observed in the effective group. Our findings support the tinnitus generation hypothesis of frontostriatal gating system dysfunction. A combination of multimodal quantitative thalamic properties may be used as indicators to predict tinnitus prognosis before sound therapy.
**Limitations:** This is not a longitude study.
**Ethics committee approval:** This study was approved by the Ethics Committee of Beijing Friendship Hospital, Capital Medical University (No. 2017-P2-134-01).
**Funding for this study:** Funding was received as part of Grant No. 82302284 from the National Natural Science Foundation of China.
**Author Disclosures:**
Qian Chen: Nothing to disclose

**09:30-11:00** **Research Stage 4**

### Research Presentation Session: Chest

### RPS 804
### Quantitative analysis of chest CT findings

**Moderator**
M. Occhipinti; Florence/IT
*(mariaelena.occhipinti@gmail.com)*
**Author Disclosures:**
Mariaelena Occhipinti: Consultant

### RPS 804-2
**Elexacaftor/ tezacaftor/ ivacaftor influences body tissue composition in adults with cystic fibrosis: a fully automated CT-based analysis**
*M. Opitz*, S. Zensen, J. Haubold, B. M. Schaarschmidt, M. Forsting, L. Umutlu, R. Hosch, F. Nensa, D. Westhölter; Essen/DE

**Purpose:** A poor nutritional status is associated with worse pulmonary function and survival in people with cystic fibrosis (pwCF). CF transmembrane conductance regulator (CFTR) modulators can improve both pulmonary function and body weight, but more data is needed to evaluate its effects on body composition. This study aims to investigate the body composition in pwCF receiving triple-combination elexacaftor/ tezacaftor/ ivacaftor (ETI) therapy.
**Methods or Backround:** A pre-trained, deep-learning network was used to perform a fully automated body composition analysis (BCA) on chest CTs from adult pwCF before and after receiving ETI therapy. Muscle and adipose tissues were quantified and divided by bone volume to obtain body size-adjusted ratios. Results from BCA were correlated with pulmonary function parameters.

**Results or Findings:** At baseline, chest CT-based BCA was conducted in 66 pwCF, with 33 (50%) receiving either mono or dual-combination CFTR modulator therapy. Mono/ dual-combination CFTR modulator therapy was associated with higher intra- and intermuscular adipose tissue and epicardial adipose tissue ratios, while other BCA markers remained unchanged. After receiving triple-combination ETI, marked increases were observed in all adipose tissue ratios among pwCF, including the total adipose tissue ratio (+46.21%, p<0.001). In contrast, only small, but statistically significant, increases of the muscle ratio were measured in the overall study population (+1.63%, p=0.008). There were weak associations between the rate of change of the muscle ratio and the rate of change of percent predicted FEV1 (r=0.360, p=0.004).
**Conclusion:** Our findings suggest that CFTR modulator therapies primarily affect adipose tissues, not muscle tissue, in adults with CF. These findings may have implications for the future nutritional management of pwCF.
**Limitations:** CT-based BCA was performed in chest CT scans. Abdominal CT scans or full-body CT scans were not available and body composition might differ in other parts of the body.
**Ethics committee approval:** The study was approved by the Institutional Review Board (no. 22-11073-BO).
**Funding for this study:** Funding was received from the German Research Foundation (DFG)-initiated clinician scientist program FU 356/12–2 (DW, JH).
**Author Disclosures:**
Michael Forsting: Nothing to disclose
Benedikt Michael Schaarschmidt: Nothing to disclose
Johannes Haubold: Nothing to disclose
Sebastian Zensen: Nothing to disclose
Lale Umutlu: Nothing to disclose
Dirk Westhölter: Nothing to disclose
René Hosch: Nothing to disclose
Marcel Opitz: Nothing to disclose
Felix Nensa: Nothing to disclose

### RPS 804-3

**Association between altered subcutaneous adipose tissue and survival in a high-risk population of heavy smokers participating in lung cancer screening**

*F. B. Pallasch*, J. Weiß, F. Bamberg, M. Jung, J. B. Fingerhut, M. Reisert; Freiburg im Breisgau/DE
*(fabian.pallasch@gmail.com)*

**Purpose:** There is increasing evidence that body composition is of prognostic relevance. While most studies focus on musculature, little is known about adipose tissue. The purpose of this study was to apply a deep-learning model for automatic 3D quantification of subcutaneous adipose tissue (SAT) on chest CT and investigate its association with mortality in a lung cancer screening population.
**Methods or Backround:** 3D SAT was automatically segmented on chest CT of 26,144 individuals participating in the National Lung Screening Trial at baseline and after one year follow-up (n=52,228 scans). SAT volume (SATvol) and density (SATHU) were quantified. The primary outcome was all-cause mortality. Additional outcomes were lung cancer and cardiovascular mortality. Cox regression was used to assess the association between SATvol and SATHU at baseline, and a decrease in SATvol and SATHU (≥10%) and mortality.
**Results or Findings:** In 26,144 individuals (age 61.4±5.0 years; 40.9% female) 1839 (7%) deaths occurred over a median follow-up of 6.5 years. At baseline, only SATHU was associated with all-cause mortality after multivariable adjustment for clinical risk factors (age, sex, race, smoking status, pack years, prevalent hypertension, diabetes, past stroke and myocardial infarction (HR: 1.07 95%CI (1.02-1.12); p=0.003). After one year, individuals with a decline in SATvol or SATHU ≥10% had a significantly worse outcome compared to stable SATvol or SATHU (HR adjusted for the same risk factors SATvol: HR 1.94, 95%CI (1.77-2.13), p<0.001; SATHU: HR 3.1, 95%CI (2.79-3.44), p<0.001). Similar associations were found for lung cancer and cardiovascular mortality.
**Conclusion:** Deep learning models can quantify SAT on chest CT scans. SATHU at baseline and a decrease in SATvol and SATHU within one year are associated with mortality beyond clinical risk factors, which may help to improve personalised risk assessment.
**Limitations:** The study is limited by its retrospective analysis.
**Ethics committee approval:** The study is based on a publicly available dataset.
**Funding for this study:** No funding was received for this study.
**Author Disclosures:**
Marco Reisert: Nothing to disclose
Jakob Weiß: Nothing to disclose
Matthias Jung: Nothing to disclose
Fabian Bamberg: Nothing to disclose
Johannes Beat Fingerhut: Nothing to disclose
Fabian Bernhard Pallasch: Nothing to disclose

### RPS 804-4

**Assessment of chest wall muscle atrophy by computed tomography and correlation with cardiopulmonary function in systemic sclerosis**

*M. Giannetti*, S. Paciulli, A. Gigante, A. Colalillo, L. Marchitelli, L. Conia, C. Catalano, N. Galea; Rome/IT
*(martina.giannetti@uniroma1.it)*

**Purpose:** Systemic sclerosis (SSc) is a multi-organ disease, which can affect the lung parenchyma resulting in an interstitial lung disease (ILD). Pulmonary function in those patients is influenced by lung disease and chest wall muscles (CWM) activity. High-resolution CT (HRCT) is a valuable tool to evaluate ILD progression and CWM atrophy. The purpose of this study was to investigate the potential contribution of CWMarea (CWMA) to the ventilatory efficiency and exercise capacity in patients with SSc.
**Methods or Backround:** Forty-four SSc patients undergone cardiopulmonary exercise testing (CPET), HRCT and transthoracic echocardiography (TTE) were retrospectively enrolled. The CWMA was manually traced at the level of the 9th thoracic vertebra on CT images. CWMA was correlated with SSc duration and CPET parameters.
**Results or Findings:** The patient median age was 53 (IQR 43.5-58) with a BMI of 22.10 kg/m2 (IQR 24.25-20.55). The median disease duration was 9.50 years (IQR 5.5-15) and the median CWMA was 43.9 cm^2 (IQR 36.8-56.5). We found a median %FVC of 100 (IQR 107-88), a %FEV1 of 95 (IQR 104.5-86), a %TLC of 93 (IQR 103.5-81.5), a %DLCO of 77 (IQR 86-68.5), and a DLCO/Va of 81.5% (IQR 93.5-69) for pulmonary functional parameters. TTE revealed a median EF of 60% (IQR 62.5-60), a RV and LV transverse diameter of 29mm (IQR 31-26 and 44mm (IQR 46-42) respectively), a TAPSE of 22 (IQR 25-21), and a PAPs of 28 (IQR 30-25). CPET parameters used were Watt-max 80% (IQR 105.5-62), VO2max 1211ml/min (IQR 1451-1026.5), VO2max 20.7ml/min/Kg (IQR23.82-17.9), VO2@AT 789.0ml/min (IQR 952-679), OUES 1364ml/min/L/min (IQR 1629-1229), VEmax 49.55L/min (IQR 60.5-44.4), VTmax 1.51L (IQR 1.88-1.21), VO2/HRmax 7.95mL/beat (IQR 9.35-6.90), and SpO2max 98% (IQR 99-97). A significant positive bivariate correlation (P<0.05) was demonstrated between CWM area, and BMI and CPET parameters.
**Conclusion:** Patients with SSc see a rediction in their accessory respiratory muscle volume and this correlates with a decline in cardiopulmonary function.
**Limitations:** The study is limited by its small sample size and single centre methodology.
**Ethics committee approval:** No information provided by the submitter.
**Funding for this study:** No funding was received for this study.
**Author Disclosures:**
Serena Paciulli: Nothing to disclose
Livia Marchitelli: Nothing to disclose
Amalia Colalillo: Nothing to disclose
Luca Conia: Nothing to disclose
Antonietta Gigante: Nothing to disclose
Martina Giannetti: Nothing to disclose
Nicola Galea: Nothing to disclose
Carlo Catalano: Nothing to disclose

### RPS 804-5

**Image registration enables quantitative regional correlation of structural and functional abnormalities assessed with CT and MRI in COPD**

*Y. Xu*[1], O. Weinheimer[1], S. Triphan[1], J. Grolig[1], O. von Stackelberg[1], J. Biederer[1], C. J. Galban[2], H-U. Kauczor[1], M. Wielpütz[1]; [1]Heidelberg/DE, [2]Ann Arbor, MI/US
*(yiling.xu@med.uni-heidelberg.de)*

**Purpose:** In the assessment of COPD, quantitative indices of emphysema, and functional small airways disease (fSAD), as well as perfusion were developed separately for CT and MRI, respectively. Their regional interdependencies, however, have not been assessed. The aim of this study was to utilise a machine-learning-based image registration pipeline to align pulmonary abnormalities regionally and examine voxel-wise correlations of emphysema and fSAD with quantitative perfusion MRI.
**Methods or Backround:** Sixty-three patients from the multi-centre COSYCONET cohort underwent same-day low-dose paired inspiratory-expiratory CT as well as morphofunctional MRI. Lungs were segmented CT and on T1-weighted VIBE MRI using an in-house pipeline, separately. The segmentation was forwarded to an iterative deformable registration model, where both CT and functional 4D perfusion MR are registered to the spatial layout of VIBE MR. The correlation between quantitative CT-based parametric response maps (PRMemphysema, PRMfSAD) and MRI-based pulmonary blood flow (PBF) and perfusion defects (QDP) was studied on a voxel level. For comparing interpatient PBF, we weighted each voxel according to its relative proportion to the patient’s lung.

**Results or Findings:** Lung voxels labeled as PRMEmphysema, PRMfSAD, and PRMNormal in CT were classified as perfusion defects in 74.59%, 57.63%, and 30.98%, of cases respectively. Dice coefficient equals 0.681 beween PRMNormal and well perfused regions. The normalised PBF was 45.8, 56.9, and 85.6 ml/100ml/min for PRMEmphysema, PRMfSAD, and PRMNormal voxels respectively.
**Conclusion:** We proposed a novel registration pipeline to spatially align CT, T1-weighted MRI and 4D perfusion MR. Results suggest CT-based PRMEmphysema and PRMfSAD labels are strongly correlated to low PBF, and had a higher tendency to be classified as perfusion defects. Perfusion information is supplementary to PRM, requiring further investigation.
**Limitations:** The method is yet to be evaluated on a larger subgroup of the cohort.
**Ethics committee approval:** The study was approved by the Ethik-Kommission der Medizinischen Fakultät der Universität Heidelberg, S-656/2012.
**Funding for this study:** No funding was received for this study.
**Author Disclosures:**
Oliver Weinheimer: Nothing to disclose
Jürgen Biederer: Nothing to disclose
Hans-Ulrich Kauczor: Nothing to disclose
Oyunbileg von Stackelberg: Nothing to disclose
Julian Grolig: Nothing to disclose
Mark Wielpütz: Nothing to disclose
Simon Triphan: Nothing to disclose
Craig J. Galban: Nothing to disclose
Yiling Xu: Nothing to disclose

### RPS 804-6

**Radiomic features changes of lung tissue in 1024 matrix CT images before the visualisation of pulmonary metastases: a preliminary study**
*H. Ren*[1], S. Dong[2], H. Yu[1]; [1]Shanghai/CN, [2]Beijing/CN
*(renhuadoc@163.com)*

**Purpose:** The purpose of this study was to investigate whether there are microscopical and invisible changes in lung CT images before the detection of pulmonary metastases.
**Methods or Backround:** Sixteen patients with pulmonary metastases were followed up for more than three months between January 2019 and June 2023. Radiomic features were extracted from high-resolution CT images before and after the visualisation of metastases. Elastic registration was applied to align images after metastasis with those before metastasis. Lung metastases were delineated using 3D-slicer, and contralateral lung tissue was outlined as contrast. Radiomic analysis was performed on the pre-metastasis images using features including first-order, grey-level co-occurrence matrix (glcm), grey-level dependence matrix (gldm), grey-level run length matrix (glrlm), grey-level size zone matrix (glszm), neighbourhood grey-tone difference matrix (ngtdm), high-order filter features, and wavelet-based features. The signatures of pre- and post-metastasis images were analysed.
**Results or Findings:** A total of 58 lesions met the criteria (29 metastases, 29 controls), yielding 1209 radiomic features. Wilcoxon tests identified 362 radiomic features with significant differences between pre- and post-metastasis images ($P<0.05$). These features were used to build a K-neighbours Classifier model. The results showed an accuracy of 0.775, sensitivity of 0.65, and specificity of 0.733 in predicting metastasis occurrence before lesion visualisation. Logistic regression analysis indicated an AUC value of 0.8.
**Conclusion:** Lung tissue may undergo subtle changes before the visualisation of pulmonary metastases. Radiomic features might reflect these changes earlier, but further validation is required with a larger dataset.
**Limitations:** Due to this study's retrospective single-centre nature, some selection bias may be unavoidable. Furthermore, all CT scans used in our study were obtained and provided by only one company.
**Ethics committee approval:** The study was approved by the Institutional Ethics Committee of Shanghai Chest Hospital, Shanghai Jiao Tong University School of Medicine. Individual consent for this retrospective analysis was waived.
**Funding for this study:** The study was funded by the National Natural Science Foundation of China (grant number 82071873), the Clinical Research Special Project of Shanghai Municipal Health Commission (grant number 20234Y0020), and the China International Medical Foundation (grant number z-2014-07-2301).
**Author Disclosures:**
Shushan Dong: Nothing to disclose
Hua Ren: Nothing to disclose
Hong Yu: Nothing to disclose

### RPS 804-7

**Associations of quantitative HRCT-derived scores of interstitial lung disease (ILD) extent with two-year transplant-free survival in patients with progressive fibrosing ILD in the ILD-PRO Registry**
*G. Kim*[1], A. Swaminathan[2], T. Whelan[2], M. Neely[2], J. Todd[2], S. Palmer[2], D. Wojdyla[2], C. Conoscent[3], J. Goldin[1]; [1]Los Angeles, CA/US, [2]Durham, NC/US, [3]Ridgefield, CT/US
*(gracekim@mednet.ucla.edu)*

**Purpose:** Associations between quantitative measures of fibrosis on HRCT and survival in patients with progressive fibrosing ILDs are not well established. The purpose of this study was to evaluate the prognostic value of HRCT-derived scores in the ILD-PRO Registry.
**Methods or Backround:** Patients had an ILD other than IPF, reticular abnormality and traction bronchiectasis, and met criteria for ILD progression within the prior 24 months. HRCT images taken closest to enrolment were analysed following lobar segmentation. A machine-learning algorithm derived the following quantitative scores: quantitative lung fibrosis (QLF); quantitative ground glass (QGG); quantitative honeycomb (QHC); quantitative ILD (QILD: sum of QLF, QGG and QHC). Cox proportional hazards models for time to death or lung transplant were fit and Kaplan-Meier event rates reported.
**Results or Findings:** Among 331 patients, median QLF, QGG, QHC and QILD scores were 14.8%, 22.7%, 0.07% and 42.3%, respectively. Event rates for death or lung transplant at 2 years in the highest vs lowest tertiles of QLF, QGG, QHC and QILD scores were 31.2% vs 19.9%, 30.4% vs 26.3%, 22.8% vs 19.4%, and 31.7% vs 17.3%, respectively. There were no significant differences in the risk of death or lung transplant across tertiles of any of the scores. The HRs (95% CI) for risk of death or lung transplant for the highest vs lowest tertiles of QLF, QGG, QHC and QILD scores were 2.20 (1.04, 4.66), 1.72 (0.88, 3.37), 0.92 (0.45, 1.90) and 2.38 (1.09, 5.20), respectively.
**Conclusion:** Patients with progressive fibrosing ILDs in the ILD-PRO Registry who had QLF or QILD scores in the highest tertiles showed trends towards an increased risk of death or lung transplant over 2 years compared with those with scores in the lowest tertiles.
**Limitations:** The study is limited by only evaluating associations between quantitative HRCT scores and short-term disease progression in patients with progressive pulmonary fibrosis.
**Ethics committee approval:** The study was approved by the Institutional Review Board of each participating centre: ClinicalTrials.gov Identifier: NCT01915511.
**Funding for this study:** The IPF-PRO/ILD-PRO Registry is supported by Boehringer Ingelheim Pharmaceuticals, Inc (BIPI) and run in collaboration with the Duke Clinical Research Institute (DCRI) and enrolling centers.
**Author Disclosures:**
Jamie Todd: Investigator: the Duke Clinical Research Institute, which receives funding from Boehringer Ingelheim to co-ordinate the IPF-PRO/ILD-PRO Registry.
Craig Conoscent: Employee: Boehringer Ingelheim, which provides funding for IPF-PRO/ILD-PRO Registry.
Jonathan Goldin: Founder: Voiant Clinical (formerly known as MedQIA).
Megan Neely: Investigator: the Duke Clinical Research Institute, which receives funding from Boehringer Ingelheim to co-ordinate the IPF-PRO/ILD-PRO Registry.
Timothy Whelan: Grant Recipient: Biogen, Celgene, Galapagos, Genentech, Gilead, Global Blood Therapeutics, Kadmon, Nitto, Pulmonary Fibrosis Foundation, Renovian, FibroGen, Toray; Consultant: from Breath Therapeutics, Genentech, United Therapeutics; investigator and Steering Committee member for IPF-PRO/ILD-PRO Registry funded by Boehringer Ingelheim.
Daniel Wojdyla: Investigator: the Duke Clinical Research Institute, which receives funding from Boehringer Ingelheim to co-ordinate the IPF-PRO/ILD-PRO Registry.
Scott Palmer: Investigator: the Duke Clinical Research Institute, which receives funding from Boehringer Ingelheim to co-ordinate the IPF-PRO/ILD-PRO Registry.
Aparna Swaminathan: Investigator: the Duke Clinical Research Institute, which receives funding from Boehringer Ingelheim to co-ordinate the IPF-PRO/ILD-PRO Registry.
Grace Kim: Grant Recipient: Boehringer Ingelheim and Genentech; Consultant: Voiant Clinical (formerly known as MedQIA).

### RPS 804-8

**Quantitative CT and computational fluid dynamics characterisation for subclinical structural-functional monitoring in humidifier disinfectant-associated lung injury**
J. Choi[1], K. J. Chae[2], H. Ko[2], W. Chung[2], G. Y. Jin[2], *C. Lee*[2]; [1]Kansas City, KS/US, [2]Seoul/KR

**Purpose:** The purpose of this study was to characterise subclinical lung structure-function alteration in humidifier disinfectant-associated lung injury (HDLI) using quantitative computed tomography (qCT) and computational fluid dynamics (CFD) analysis.

**Methods or Backround:** For 103 toxic HD-exposed patients, commercial and in-house software computed 186 multiscale lung structure-function features through quantitative inspiratory-expiratory CT image matching and one-dimensional CFD breathing simulations. We first characterised HD-associated structure-function abnormality from 72 adults with normal-appearing CTs and pulmonary function tests (PFTs), and then reviewed two-year changes in 31 patients in the national HDLI monitoring program of Korea.
**Results or Findings:** In HD-affected normal-appearing cases, airway narrowing resulted in a greater tracheobronchial air pressure drop particularly through the left lower lobe segmental airways (r=0.54, p<0.001). Elevated air trapping, upper and middle lobes motion, and reduced volume expansion reduced transpulmonary pressure (r=-0.69, 0.53, 0.50; p<0.001, all), while elevated high attenuation area reduced lower lobe motion (r=-0.50, p<0.001), implying no direct impact of basal lung inflammation and fibrosis on tidal breathing characteristics. Reduced whole lung motion, reduced right lower lobe ventilation, and elevated left upper lobe ventilation elevated airway resistance (r=-0.61, -0.62, 0.61; p<0.001, all). Two-year changes of these features characterised recovering, stable, and worsening subgroups of the 31 monitoring patients with no eminent changes in visual CT features and PFTs.
**Conclusion:** qCT-CFD analysis provided novel imaging-based regional lung structure-function characterisation of subclinical lung damage, recovery, and worsening in HDLI. This novel approach may help assess underlying mechanisms of complicated and worsening lung recovery.
**Limitations:** The relatively small sample size limits the applicability of the study.
**Ethics committee approval:** The study was approved with approval code: 2020-06-037, from the Jeonbuk National University Hospital.
**Funding for this study:** The study received funding from the Mid-Career Bridging Program through Seoul National University.
**Author Disclosures:**
Changhyun Lee: Nothing to disclose
Hongseok Ko: Nothing to disclose
Wonchul Chung: Nothing to disclose
Jiwoong Choi: Nothing to disclose
Kum Ju Chae: Nothing to disclose
Gong Yong Jin: Nothing to disclose

### RPS 804-9

**Multiparametric evaluation of radiomics features and dual-energy CT iodine maps for discrimination and outcome prediction of thymic masses**
*S. Martin*, S. Mahmoudi, I. Yel, C. Booz, L. D. Grünewald, J-E. Scholtz, K. Eichler, T. Vogl, V. Koch; Frankfurt a. Main/DE

**Purpose:** The objective of this study was to investigate the diagnostic value of radiomics features and dual-source dual-energy CT (DECT) based material decomposition in differentiating low-risk thymomas, high-risk thymomas, and thymic carcinomas.
**Methods or Backround:** This retrospective study included 32 patients (16 males, mean age 66±14 years) with pathologically confirmed thymic masses who underwent contrast-enhanced DECT between October 2014 and January 2023. Two experienced readers evaluated all patients regarding conventional radiomics features, as well as DECT-based texture features, including attenuation (HU), iodine density (mg/ml), and fat fraction (%). Data comparisons were performed using analysis of variance (ANOVA) and chi-square statistic tests. Receiver operating characteristic (ROC) curve analysis and Cox regression tests were used to discriminate between low-risk/ high-risk thymomas and thymic carcinomas.
**Results or Findings:** Of the 32 thymic tumors, 12 (38%) were low-risk thymomas, 11 (34%) were high-risk thymomas, and 9 (28%) were thymic carcinomas. Values differed significantly between low-risk thymoma, high-risk thymoma, and thymic carcinoma regarding DECT-based texture features (p≤0.023) and 30 radiomics features (p≤0.037). The area under the curve (AUC) to differentiate between low-risk/ high-risk thymomas and thymic cancer was 0.998 (95% CI, 0.915–1.000; p<0.001) for the combination of DECT imaging parameters and radiomics features, yielding a sensitivity of 100% and specificity of 96%. During a follow-up of 60 months (IQR, 35-60 months), the multiparametric approach, including radiomics features, DECT parameters, and clinical parameters, showed an excellent prognostic power to predict all-cause mortality (c-index = 0.978 (95% CI, 0.958–0.998), p=0.003).
**Conclusion:** A multiparametric approach including conventional radiomics features and DECT-based texture features facilitates accurate, non-invasive discrimination between low-risk/ high-risk thymomas and thymic carcinomas.
**Limitations:** No limitations were identified.
**Ethics committee approval:** This retrospective study was approved by the Ethical Review Board of the publishing institution, and written informed consent was waived.
**Funding for this study:** No funding was received for this study.
**Author Disclosures:**
Simon Martin: Nothing to disclose
Christian Booz: Speaker: Siemens
Ibrahim Yel: Speaker: Siemens
Thomas Vogl: Nothing to disclose
Jan-Erik Scholtz: Nothing to disclose
Vitali Koch: Nothing to disclose
Scherwin Mahmoudi: Nothing to disclose
Leon David Grünewald: Nothing to disclose
Katrin Eichler: Nothing to disclose

**12:15-13:45** **Research Stage 3**

### Research Presentation Session: Musculoskeletal

### RPS 910

### Musculoskeletal ultrasound and ultrasound-guided intervention

**Moderator**
M. Posadzy; Poznan/PL
*(magdalenaposadzy@gmail.com)*

### RPS 910-2

**High-resolution ultrasound of the postoperative flexor digitorum tendon of the fingers after zone-II reconstructive surgery**
*M. Pansecchi*, F. Zaottini, R. Picasso, F. Pistoia, M. Maccio, S. Rinaldi, D. Bianco, G. Marcenaro, C. Martinoli; Genoa/IT

**Purpose:** Despite advancements in tendon repair techniques, there are still several complications that can occur after tendon surgery. These include adhesion formation, tendon repair rupture, and stiffness of the joints. The aim of this study was to describe the potential role of high-resolution ultrasound (US) to detect postoperative flexor tendon complications after zone-II surgery.
**Methods or Backround:** Twenty-five consecutive patients were submitted to imaging examination for impaired finger flexion/extension after zone-II reconstructive surgery for a previous tear of the flexor digitorum profundus and/or superficialis tendons. Dynamic US examination was performed by means of long- and short axis planes using 18-5 and 22-8MHz linear array probes.
**Results or Findings:** High-resolution US was able to identify suture material and distinguish stitches of variable thickness and appearance. US imaging over the tendon repair was able to identify focal swelling and prominent knots obstructing tendon gliding. After FT repair, interphalangeal joint contraction and stiffness was found in eight cases without any tendon gliding abnormality. Obstructing gliding and adhesions were observed in twelve cases. In 4/12 cases, adhesions were subtle to recognize and could only be identified on dynamic scans based on traction movements of peritendinous tissues. Tendon repair rupture was observed in n=3 cases as a new tendon discontinuity at the repair site. In one case, bone shortening after P1 fracture and fixation with metallic plate was diagnosed by US. In another case, the FDP tendon was screwed.
**Conclusion:** Although difficult to perform and operator-dependent, high-resolution US should be regarded as the modality of choice to evaluate the postoperative flexor digitorum tendons in the fingers due to its dynamic capabilities and excellent detail resolution.
**Limitations:** Further studies based on larger series are needed to define the ultimate value of ultrasound in this field.
**Ethics committee approval:** The study was conducted in accordance with the Declaration of Helsinki.
**Funding for this study:** No funding was received for this study.
**Author Disclosures:**
Giovanni Marcenaro: Nothing to disclose
Deborah Bianco: Nothing to disclose
Simone Rinaldi: Nothing to disclose
Federico Zaottini: Nothing to disclose
Carlo Martinoli: Nothing to disclose
Michelle Pansecchi: Nothing to disclose
Riccardo Picasso: Nothing to disclose
Federico Pistoia: Nothing to disclose
Marta Maccio: Nothing to disclose

### RPS 910-3

**Ultrasound-guided thread-release of the first extensor compartment of the hand: initial experience in the anatomical model**

*L. Lechner*, S. A. Jengojan, P. Sorgo, G. Bodner, V. Moser, L. Hirtler; Vienna/AT

**Purpose:** The objective of this study was to develop and evaluate a minimally-invasive ultrasound-guided thread-release technique for safely and effectively decompressing the first extensor compartment in anatomical specimens.
**Methods or Backround:** De Quervain's tenosynovitis is a common disabling condition of the hand, arising due to the compression of the tendons in the first extensor compartment. If conservative treatments fail, surgical intervention is required. In recent years, minimally-invasive ultrasound-guided procedures, especially for carpal tunnel syndrome and trigger finger, are on the rise. However, thus far the thread technique was not studied in the release of the first extensor compartment for possible future treatment of De Quervain´s tenosynovitis in an anatomical or clinical setting. For this study, we developed a protocol for the minimally-invasive ultrasound-guided thread-release and performed it prospectively on ten fresh cadaveric hands. Subsequently, the specimens were dissected and assessed by an experienced anatomist regarding efficacy and safety of the procedure. Ultrasound visibility, degree of transection, and unnecessary damage to surrounding neurovascular structures were documented.
**Results or Findings:** Ultrasound visibility was sufficient in all specimens. Out of ten interventions, a complete release was achieved in nine cases. Slight, clinically irrelevant, lesions on the underlying extensor tendons were observed in one case. Macroscopically visible neurovascular structures were not harmed in any case.
**Conclusion:** Real-time ultrasound-guided thread-release is a reliable and safe minimally-invasive method for releasing the first extensor compartment in anatomical specimens while protecting the surrounding structures.
**Limitations:** This study was conducted on anatomical specimens. Thus, results may differ in the clinical setting. Due to cadaver availability, the initial sample size was limited. Therefore, further anatomical and clinical trials are warranted.
**Ethics committee approval:** This study was approved by the Ethics Committee at the Medical University of Vienna.
**Funding for this study:** No funding was received for this study.
**Author Disclosures:**
Lisa Lechner: Nothing to disclose
Gerd Bodner: Nothing to disclose
Veith Moser: Nothing to disclose
Philipp Sorgo: Nothing to disclose
Suren Armeni Jengojan: Nothing to disclose
Lena Hirtler: Nothing to disclose

### RPS 910-4

**Ultrasound-guided thread-transection of the arcade of frohse is an effective and minimally-invasive method for posterior interosseus nerve release in the anatomical model**

*L. Lechner*, L. Hirtler, P. Sorgo, G. Bodner, V. Moser, S. A. Jengojan; Vienna/AT

**Purpose:** The purpose of this study was to expand the area of application for effective and safe minimally-invasive ultrasound-guided thread-interventions to the transection of the Arcade of Frohse.
**Methods or Backround:** Transection of the Arcade of Frohse becomes clinically relevant in the treatment of radial tunnel syndrome, a rare and painful compressive neuropathy in the proximal forearm. Ultrasound-guided thread-release is a promising new technique for the decompression of peripheral nerves. This study aims to gain initial experience in performing thread-release on the posterior interosseus nerve by transecting the Arcade of Frohse in the anatomical model. Ten ultrasound-guided thread-interventions were performed in fresh cadaveric hands. Subsequent anatomical dissections evaluated outcome of the transection and possible damage to adjacent structures. Ultrasound visibility was also documented for every intervention.
**Results or Findings:** A complete transection of the Arcade of Frohse was achieved in eight out of ten specimens. Ultrasound visibility was sufficient in all cases. There was no macroscopically visible damage to surrounding neurovascular structures. However, small lesions to the supinator muscle and the superficial extensor muscle were unavoidable in three cases.
**Conclusion:** Ultrasound-guided thread-transection is a safe and effective minimally-invasive procedure for transecting the Arcade of Frohse in an anatomical model.
**Limitations:** This study was conducted on fresh anatomical specimens. Therefore, patient results may differ and further cadaveric and clinical studies are warranted. Furthermore, the study sample size is limited due to cadaver availability.
**Ethics committee approval:** This study was approved by the Ethics Committee at the Medical University of Vienna.
**Funding for this study:** No funding was received for this study.
**Author Disclosures:**
Lisa Lechner: Nothing to disclose
Gerd Bodner: Nothing to disclose
Veith Moser: Nothing to disclose
Philipp Sorgo: Nothing to disclose
Suren Armeni Jengojan: Nothing to disclose
Lena Hirtler: Nothing to disclose

### RPS 910-5

**Evaluation of tibial and median nerves in patients with diabetic peripheral neuropathy using shear wave elastography**

*A. Singh*; Amritsar/IN
*(dr.amancs@gmail.com)*

**Purpose:** Diabetic peripheral neuropathy (DPN) is a major complication of diabetes mellitus. Clinical examination and nerve conduction study (NCS) of both tibial and median nerves are done to diagnose DPN. The purpose of this study was to perform high-resolution ultrasound with shear wave elastography (SWE) to assess tibial and median nerves mean stiffness as an indicator for development of DPN.
**Methods or Backround:** The study was conducted on 90 subjects which included 30 healthy individuals, 30 diabetic patients without DPN and 30 diabetic patients with DPN. The tibial and median nerve stiffness was measured with SWE. The differences in stiffness values among patients with DPN, patients with clinically defined DPN, patients without DPN, and healthy volunteers based on clinical features and electrodiagnostic tests were evaluated.
**Results or Findings:** The tibial nerve stiffness based on mean (EMean), minimum (EMin), and maximum (EMax) shear elasticity indices was significantly higher in patients with DPN and clinically defined DPN than that in patients without DPN and control subjects ($p<0.05$). EMin cutoff value had a sensitivity, specificity, PLR, and NLR. The inter- and intraobserver agreements were excellent for the SWE measurements.
**Conclusion:** Tibial nerve stiffness is significantly higher in diabetic patients with DPN and clinically defined DPN. The EMean and EMin have a good accuracy for identifying DPN. SWE has a potential use for cases with clinically defined DPN.
**Limitations:** The study was limited by its small sample size.
**Ethics committee approval:** This study was approved by the Institutional Ethical and Research Committee.
**Funding for this study:** No funding was received for this study.
**Author Disclosures:**
Amandeep Singh: Nothing to disclose

### RPS 910-6

**Imaging features of hourglass constriction of the radial nerve with surgical correlation**

*J. Kim*, Y. Kim, C-H. Lee, S. Lee; Seoul/KR
*(masterk95@naver.com)*

**Purpose:** The objective of this study was to evaluate the imaging characteristics of hourglass constriction (HC) of the radial nerve using ultrasonography (USG) and MRI correlating with surgical observations.
**Methods or Backround:** From March 2020 to September 2023, the preoperative USG and MRI of six patients (5 men, 1 woman; mean age 30.5 years old) who were surgically confirmed were retrospectively evaluated. The location and number of HCs identified by both USG and/or MRI, as well as the signal intensity of the nerve in axial fat-suppressed T2 weighted images (FST2), were assessed and compared with surgical observations.
**Results or Findings:** A total of 37 HCs were identified across the six patients. All patients exhibited three or more multiple HCs. The most common locations for HCs were the intermuscular space between the brachialis and brachioradialis muscles (19/37, 51.4%), followed by the lateral epicondyle level (7/37, 18.9%) and the arcade of Frohse (4/37, 10.8%). On FST2, the peripheral high signal intensity and central low signal intensity (referred to as the "bull's eye sign") was most frequently observed proximal to the HC (20/37, 54.1%). Out of the 37 HC sites, 23 were found to have constriction bands around the perineurium of the fascicle of the deep radial nerve. This was most often located near the branching site from the radial nerve within the intermuscular space between the brachioradialis and brachialis muscles.
**Conclusion:** Hourglass constrictions of the radial nerve typically show multiple involvements in the perineurium of the deep radial nerve fascicle with bull's eye sign, especially around the branching site from the radial nerve between the brachialis and brachioradialis muscles.
**Limitations:** This study was a retrospective study of a single centre, with a small sample size and a lack of consensus in the accurate cutoff degree of significant hourglass constriction.
**Ethics committee approval:** This study was approved by the Hanyang University Research Committee.

**Funding for this study:** No funding was received for this study.
**Author Disclosures:**
Chang-Hun Lee: Nothing to disclose
Seunghun Lee: Nothing to disclose
Yeoju Kim: Nothing to disclose
Juin Kim: Nothing to disclose

### RPS 910-7

**High-frequency ultrasound of pulleys of the flexor tendons of the toes**

*F. Pistoia*, F. Zaottini, R. Picasso, M. Maccio, D. Bianco, S. Rinaldi, M. Pansecchi, C. Martinoli; Genoa/IT
*(federicopistoia1@gmail.com)*

**Purpose:** The purpose of this study was to determine the ability of high-frequency ultrasound (HRUS) to assess the pulleys of the flexor tendons of the toes.
**Methods or Backround:** The initial phase of the study involved examining the pulley system of flexor tendons of the toes in six cadaveric specimens using high-frequency ultrasonography. Coloured latex was then injected under US guidance at various pulley levels. In the subsequent part of the research, a 22-8 MHz US probe was used to evaluate the toe pulley system in 40 feet of 20 healthy volunteers. Then, five patients with hammer toes were evaluated using the same US probe.
**Results or Findings:** The coloured latex was placed correctly in all six specimens. The toe pulleys were consistently identified with HRUS on all forty healthy volunteers. All five patients demonstrated a mean thickness value of the AIII pulley of the affected toes superior to the respective toes in the healthy group.
**Conclusion:** High-frequency US allows excellent depiction of toe pulleys. However, further research is necessary to understand the potential impact of thickened pulleys on hammer toe deformity.
**Limitations:** This is a first explorative study. Further research in larger population is needed to understand the clinical value of this ultrasound application.
**Ethics committee approval:** This study was performed in line with the principles of the Declaration of Helsinki.
**Funding for this study:** No funding was received for this study.
**Author Disclosures:**
Deborah Bianco: Nothing to disclose
Simone Rinaldi: Nothing to disclose
Federico Zaottini: Nothing to disclose
Carlo Martinoli: Nothing to disclose
Michelle Pansecchi: Nothing to disclose
Riccardo Picasso: Nothing to disclose
Federico Pistoia: Nothing to disclose
Marta Maccio: Nothing to disclose

### RPS 910-8

**Ultrasound-guided percutaneous irrigation of extra-shoulder calcific tendinopathy**

*F. Serpi*[1], U. Viglino[2], C. Messina[1], S. Fusco[1], S. Gitto[1], F. Lacelli[3], L. M. Sconfienza[1], D. Albano[1]; [1]Milan/IT, [2]Genoa/IT, [3]Pietra Ligure/IT
*(francesca.serpi@unimi.it)*

**Purpose:** This study aimed to investigate the efficacy and safety of ultrasound-guided percutaneous irrigation of calcific tendinopathy (US-PICT) applied out of the shoulder, comparing its effectiveness to US-PICT of the rotator cuff.
**Methods or Backround:** Patients subjected to US-PICT for extra-shoulder calcific tendinitis (Case Group) were compared to those subjected to US-PICT of the rotator cuff (Control Group). We had pre-procedure VAS (visual analogue scale) pain score, 1-month and 3-month VAS of patients of the Case Group, pre-procedure, and 3-month VAS of patients of the Control Group.
**Results or Findings:** The Case Group consisted of 41 patients (27 women; mean age: 45±9years): 26 gluteus medius, 5 patellar tendon, 3 rectus femoris, 2 gluteus maximus, 2 common extensor tendon, 1 extensor carpi radialis longus, 1 pes anserinus, 1 peroneus longus. The Control Group included 41 patients (27 women; mean age: 47±11 years). The mean pre-procedure VAS of the Case Group was 8.8±0.7 with a significant (P<0.001) drop at 1 month (4.5±0.6) and 3 months (3.6±0.6). The mean pre-procedure VAS of the Control Group was 8±1.4 and dropped to 3.1±1.6 after 3 months (P<0.001). Post-treatment VAS at 3 months was not significantly different between two Groups (p=0.134). Similarly, the decrease of VAS from baseline to three months was not significantly different between the two Groups (p=0.264).
**Conclusion:** This study demonstrated the safety and effectiveness of US-PICT as a valuable therapeutic option for extra-shoulder calcific tendinitis, with similar clinical outcome to the same procedure performed in the rotator cuff.
**Limitations:** This study had a relatively small sample size with few cases in some localisations. A further limitation is the retrospective nature of the study, warranting prospective studies to compare the procedure with other conservative approaches and to understand how to manage these patients postoperatively.
**Ethics committee approval:** No information provided by the submitter.
**Funding for this study:** No funding was received for this study.
**Author Disclosures:**
Francesca Lacelli: Nothing to disclose
Stefano Fusco: Nothing to disclose
Carmelo Messina: Nothing to disclose
Salvatore Gitto: Nothing to disclose
Luca Maria Sconfienza: Nothing to disclose
Umberto Viglino: Nothing to disclose
Francesca Serpi: Nothing to disclose
Domenico Albano: Nothing to disclose

### RPS 910-9

**Shear wave of patellar and Achilles tendon on healthy patients: intervendor intraobserver and interobserver comparison**

*U. Viglino*[1], F. Esposito[2], D. Albano[3], S. Gitto[3], C. Messina[3], L. M. Sconfienza[3]; [1]Genoa/IT, [2]Napoli/IT, [3]Milan/IT

**Purpose:** Shear wave elastography is a renowned ultrasound technique that quantitatively evaluates the elasticity of tissues. The aim of this study is to validate the reproducibility of elasticity measurements of the Achilles and patellar tendons in healthy subjects using ultrasound machines from three different manufacturers and to compare intraobserver and interobserver variations between two examiners.
**Methods or Backround:** High-resolution linear probes were used on three ultrasound machines from different manufacturers. The study included 13 healthy volunteers with no prior history of tendon injuries. Data acquisition was performed in both supine and prone rest positions, with three measurements taken for each tendon on each side of the body, for a total of 26 tendons. Each joint was repositioned after the measurement. A comparison of intraobserver variations was made with a reevaluation after two weeks (T1).
**Results or Findings:** Intraobserver reproducibility of the three different acquisitions on each tendon was demonstrated for both observers on the three ultrasound machines (p<0.001). Intraobserver reproducibility T0-T1 was statistically significant (p<0.05) for only one observer across the three ultrasound machines for both tendons. Interobserver reproducibility was statistically significant (p<0.05) only for patellar tendon on two of the ultrasound machines [0.398 (CI 0.02-0.676); 0.421 (CI 0.048-0.691)]. Intervendor reproducibility was poor, not statistically significant [Reader 1 0.257 (CI -0.138-0.581) for patellar and 0.222 (CI -0.174-0.556) for Achilles] [Reader 2 0.075 (CI -0.315-0.443 for patellar and -0.118 (CI -0.477-0.275) for Achilles].
**Conclusion:** This study demonstrated that shear wave ultrasound could be a useful quantitative tool for the study of tendons but its reproducibility is acceptable only in specific conditions (such as with the same sonographer). Reproducibility has shown to be suboptimal in other applications, for example in reexamination, with a different sonographer, and poor with different ultrasound machines.
**Limitations:** This study was limited by the lack of supersonic shear imaging (SSI).
**Ethics committee approval:** No information provided by the submitter.
**Funding for this study:** No funding was received for this study.
**Author Disclosures:**
Carmelo Messina: Nothing to disclose
Salvatore Gitto: Nothing to disclose
Luca Maria Sconfienza: Nothing to disclose
Umberto Viglino: Nothing to disclose
Domenico Albano: Nothing to disclose
Francesco Esposito: Nothing to disclose

### RPS 910-10

**Establishing baseline enthesis stiffness range in a healthy adult population**

*M. K. J. Mahazer*; Terengganu/MY
*(khairijahidi@unisza.edu.my)*

**Purpose:** The purpose of this study is to determine the potential of shear wave elastography (SWE) in measuring the median stiffness value of enthesis in healthy subjects and to assess interobserver reliability.
**Methods or Backround:** Ninety-eight healthy male and female subjects aged 20-50 years underwent SWE of the bilateral enthesis of quadriceps, suprapatellar, infrapatellar, Achilles, and triceps performed by two experienced sonographers. Each enthesis was evaluated five consecutive times using the ElastQ software. Median shear wave velocity values were compared for each enthesis by each examiner. Interobserver agreement was evaluated.
**Results or Findings:** Kruskal Wallis and ANOVA tests showed significant differences in median shear wave velocity (SWV) of enthesis for quadriceps, suprapatellar, and left Achilles between the 40-50 age group vs the 20-29 age group and the 30-39 vs 40-49 age groups. However, insignificant difference of median SWV was found in the infrapatellar and right Achilles. The median (IQR) stiffness of quadriceps for age groups 20-39 and 40-50 were 2.96m/s (0.42) and 2.80m/s (0.36), suprapatellar for age groups 20-39 were 3.00m/s (0.27), and for ages 40-50 were 2.90m/s (0.32), and left Achilles 20-39 years old 33.12 m/s (0.55) and 40-50 years old (2.91m/s (0.29) respectively. Good interobserver agreement was found between quadriceps, suprapatellar, infrapatellar, and Achilles (95% CI; 0.84) (p<0.01).
**Conclusion:** The study suggests that shear wave elastography holds potential as a reliable technique for quantifying enthesis stiffness. It is noteworthy that age does not appear to have a significant influence on the median shear wave velocity (SWV) of entheses, except for the quadriceps, suprapatellar, and left Achilles in patients aged 40-50.
**Limitations:** The study was limited by its small sample size, and the lack of additional imaging such as MRI.
**Ethics committee approval:** This study was approved by the University of Malaya Medical Centre, MREC ID NO: 2021112-10731.
**Funding for this study:** No funding was received for this study.
**Author Disclosures:**
Mohammad Khairi Jahidi Mahazer: Investigator: Radiographer at Teaching Hospital University of Sultan Zainal Abidin

**RPS 910-11**
**High-resolution ultrasound of the anterior talofibular ligament: correlation between morphological and clinical features**
*G. Marcenaro*; Genoa/IT

**Purpose:** The anterior talofibular ligament (ATFL) is usually considered as a single fascicle but it is actually made of two different bands, the superior and the inferior bands. The main objective of this study is to determine the role that these two fascicles play in the ankle stability.
**Methods or Backround:** We recruited 86 patients who accessed the emergency department for ankle sprain. An x-ray exam has been used to rule out patients with fractures. After 14 days patients underwent an ultrasound examination to look for injuries of the superior and inferior fascicle of the ATFL. Each fascicle has been given a score (0 to 3) to describe the severity of the injury. An objective measure of the articular instability has been made measuring the minimum (a) and maximum (b) distance between the tip of the fibula and the body of the talus, positioning the foot in plantar flexion and inversion (a) and in dorsiflexion and eversion (b). Two months after the exam, patients were asked to fill in the CAIT questionnaire to provide objective data about ankle instability, which was correlated with the findings of the ultrasound exam.
**Results or Findings:** In a population of 86 patients, the multivariate analysis shows that there is a significant relation between injury of the calcaneofibular ligament CFL and ankle instability (p≤0.029) and between the two fascicles of the ATFL an injury of the superior is more likely to cause instability.
**Conclusion:** The CFL is the most important ligament for preserving ankle stability. There is a trend showing that the superior band of the ATFL is more responsible for ankle stability than the inferior, which is to be confirmed with a broader dataset.
**Limitations:** Recruitment for a follow-up study is in progress, as this study is based on the questionnaire responses of 100 patients.
**Ethics committee approval:** The study has been approved by the ethics committee of San Martino Hospital HRCCS of Genoa, Italy.
**Funding for this study:** No funding was received for this study.
**Author Disclosures:**
Giovanni Marcenaro: Nothing to disclose

**12:30-13:30 Research Stage 1**

### Research Presentation Session: Emergency Imaging

### RPS 917

### Optimising imaging in emergency radiology: current and future trends

**Moderator**
I. Gunes Tatar; Ankara/TR
*(idil.gunes.tatar@gmail.com)*

**RPS 917-2**
**An evaluation of multiple upper limb x-rays: is adjacent imaging necessary?**
*D. Abu Awwad*, E. U. Ekpo; Sydney, NSW/AU
*(dania.abuawwad@sydney.edu.au)*

**Purpose:** Medical imaging is a valuable diagnostic tool in emergency departments. However, the reliance on imaging leads to increased practices that may deviate from evidence-based guidelines. X-ray requests for extremities sometimes also simultaneously ask for X-ray projections for other adjacent regions. However, the usefulness of these additional projections is unclear. Our aim was to assess the diagnostic yield of X-rays performed on upper limb regions adjacent to the area of injury.
**Methods or Backround:** Data were collected retrospectively and included all patients who had X-rays of multiple upper limb regions. Clinical history notes and radiological reports were used to establish the diagnostic yield of X-rays performed on a region adjacent to the injured limb, and the association between patient symptoms and diagnostic outcomes were assessed using Chi-Square analysis.
**Results or Findings:** A total of 1455 upper limb X-ray imaging were performed on 591 patients. A third of these patients had three or more X-rays. About 95% of the abnormalities were detected on X-rays performed at the site of injury. The diagnostic yield of X-rays performed on the region adjacent to the injured limb was only 1.5%. Chi-Square analysis showed that X-ray examination of adjacent limb was less likely to detect new (previously undetected) abnormalities ($\chi2(2) = 498.981$; p = <0.001). New abnormalities were more common in the wrist or shoulder, and the majority were due to an injury and in women older than 65 years.
**Conclusion:** Adjacent upper limb images have very low diagnostic yield or impact. A change in the practice is needed to maximise the benefits of imaging and reduce the cost and risks associated with additional X-ray imaging of asymptomatic adjacent limbs.
**Limitations:** No limitations were identified.
**Ethics committee approval:** This study was approved by the Sydney Local Health District Human Research Ethics Committee (2022/ETH01291).
**Funding for this study:** No funding was provided for this study.
**Author Disclosures:**
Dania Abu Awwad: Nothing to disclose
Ernest Usang Ekpo: Nothing to disclose

**RPS 917-3**
**High-pitch head CT in uncooperative emergency department patients with acute head trauma: evaluation of image quality and detection of acute intracranial pathologies**
*J-E. Scholtz*, D. Leitner, L. D. Grünewald, S. Mahmoudi, V. Koch, C. Booz, T. Vogl; Frankfurt a. Main/DE
*(janerikscholtz@gmail.com)*

**Purpose:** The purpose of this study was to evaluate image quality and diagnostic accuracy of an unenhanced high-pitch head CT protocol used in uncooperative emergency department (ED) patients after acute trauma.
**Methods or Backround:** We retrospectively compared high-pitch (1.55) head CT protocol (CTDIVOL 34.4 mGy) used in uncooperative ED patients and 1:1 age-matched ED patients who underwent standard-pitch (0.55) head CT protocol (CTDIVOL 39.5 mGy) after acute trauma between January 2023 and August 2023. Gray matter (GM) signal-to-noise ratio (SNR), white matter (WM) SNR, GM-WM contrast-to-noise ratio (CNR), and posterior fossa artefact index (PFAI) were compared. Two radiologists performed qualitative image evaluation of intracranial and bone structures, artefacts, and diagnostic evaluation for acute intracranial pathologies and fractures.
**Results or Findings:** Overall, 87 patients [male, n=55(63.2%), 68±22 years] underwent high-pitch CT protocol. Quantitative image evaluation did not show significant differences between high-pitch and standard protocols: GM SNR, mean±SD, 18.3±3.4 vs. 17.6±2.6, WM SNR, 15.2±2.5 vs. 14.8±2.3, GM-WM CNR 2.3±0.7 vs 2.2±0.6, PFAI, 3.4±0.3, all p≥0.41. Grey-white differentiation [median (IQR); 5(4,5) vs. 5(4,5)], delineation of cerebrospinal fluid spaces, and bone structures were rated very good for both protocols without significant differences (p≥0.05). Artefacts in the supratentorial region were low in both protocols (p=0.09), while beam-hardening artefacts in the infratentorial region were slightly worse in high-pitch scans (p=0.001). Diagnostic confidence to evaluate for intracranial haemorrhage, brain herniation, and fracture was very good for both protocols without significant differences. Inter-rater agreement for the detection of intracranial haemorrhages (n=40), herniation (n=17), and fractures (n=14) was excellent for both protocols (κ,1.0).
**Conclusion:** High-pitch head CT protocol is a safe and robust alternative in uncooperative ED patients to evaluate for acute intracranial pathologies after trauma.
**Limitations:** The high-pitch head protocol has not been evaluated for ischaemic stroke imaging.
**Ethics committee approval:** This was a retrospective study with a waiver for informed consent.
**Funding for this study:** No funding was received for this study.
**Author Disclosures:**
Christian Booz: Nothing to disclose
Thomas Vogl: Nothing to disclose
Jan-Erik Scholtz: Nothing to disclose
Vitali Koch: Nothing to disclose
Scherwin Mahmoudi: Nothing to disclose
Leon David Grünewald: Nothing to disclose
Daniel Leitner: Nothing to disclose

### RPS 917-4

**A simple clinical algorithm for prediction of relevant CT findings could reduce the number of low-value cranial CT scans by more than 20%**

J. M. Plasencia Martinez, *M. S. Canales*, E. Otón González,
N. I. Casado Alarcón, E. C. Cotillo Ramos, H. Ortiz Mayoral, B. Molina-Lozano,
J. M. Garcia Santos; Murcia/ES
*(sanchezcanales.marta@gmail.com)*

**Purpose:** The number of cranial CT scans is exponentially increasing, without a clear benefit. Our aim is to determine the yield of urgent cranial CT depending on the clinical reason for consultation (RC), and to put forward a diagnostic algorithm.
**Methods or Backround:** This was a retrospective, randomised study in patients who underwent urgent cranial CT for non-traumatic reasons, between 2017 and 2021. Using binary logistic regression, the discriminatory capacity of the variables was analysed. The independent variables were RC and personal history, and the dependent variables are relevant radiological findings (RRF) described in urgent cranial CT: acute ischaemia and haemorrhage, mass, oedema, or unknown hydrocephalus, and relevant clinical-radiological findings (RCRF): patients with RRF and/or need for hospital admission.
**Results or Findings:** We recruited 702 patients, median age 62 [interquartile range 47-76] years, 339 (48.3%) males. Variables that increased the RCRF risk (Odds Radio -OR- >1, P<0.05) were motor deficit (OR_5.086; 95%CI_2.893-8.940), speech deficit (OR_5.042; 95%CI_2.871-8.854), seizure disorder (OR_4.688; 95%CI_2.106-10.436), cognitive impairment (OR_2.639; 95%CI_1.316-3.851), sensory deficit (OR_2.223, 95%CI_1.300-3.800), oncological history (OR_1.750; 95%CI_1.238- 2.475), and sudden altered mental status (OR_1.553; 95%CI_1.055-2.286). Variables that decreased the RCRF risk (OR<1, P<0.05) were dizziness (OR_0.652; 95%CI 0.45-0.943), nausea-vomiting (OR_0.594; 95%CI_0.385-0.917), syncope-presyncope (OR_0.580; 95%CI_0.357-0.941), acute headache (OR_0.528; 95%CI_0.368-0.756), and vertigo (OR_0.267; 95%CI_0.142-0.505). When the above RCs did not show discriminative ability, the ability of the remaining RCs was re-evaluated. An algorithm depicted is segregated, with sensitivity and negative predictive value above 92%, which would reduce 23.9% of CTs in the full sample and 35.2% in non-admitted patients, failing to diagnose 7% of RRF.
**Conclusion:** A clinical algorithm is proposed for relevant radiological findings in non-traumatic urgent cranial CT scans, potentially avoiding at least 23% of CT scans.
**Limitations:** The study is awaiting prospective external validation.
**Ethics committee approval:** This study was approved with code CETI: 02/22.
**Funding for this study:** No funding was received for this study.
**Author Disclosures:**
Estefania Corina Cotillo Ramos: Nothing to disclose
Elena Otón González: Nothing to disclose
Juana María Plasencia Martinez: Nothing to disclose
Jose Maria Garcia Santos: Nothing to disclose
Marta Sánchez Canales: Nothing to disclose
Nuria Isabel Casado Alarcón: Nothing to disclose
Belén Molina-Lozano: Nothing to disclose
Herminia Ortiz Mayoral: Nothing to disclose

### RPS 917-5

**Evaluation of low-dose whole brain CT perfusion-driven CT angiography utilising a deep learning image reconstruction algorithm**

*Y. Zhou*, L. Lei, Z. Wang, W. Cao, S. W. Yue; Zheng Zhou/CN
*(zyh921209zyh@163.com)*

**Purpose:** The purpose of this study is to investigate the image quality and diagnostic accuracy of low-dose whole brain CT perfusion-driven CT angiography with deep learning image reconstruction (DLIR) in patients with patients with acute ischeamic stroke (AIS) caused by large vessel occlusion (LVO).
**Methods or Backround:** This prospective study collected 62 patients with AIS caused by LVO, divided into standard-dose (80keV 150Ams) and low-dose (80keV 100Ams) CTP examination. The FBP, ASIR-V40% / 80% were using standard-dose group, and ASIR-V80% and DLIR (M, H) were using low-dose group. ROIs were drawn in the CTP arterial phase images of siphon portion of the internal carotid artery (ICA), middle cerebral artery M1 segment (MCA-M1), and temporal lobe for objective evaluation of image noise, SNR, and CNR. Subjective evaluation included edge sharpness of vascular lumen, display of small blood vessels, and overall image quality.
**Results or Findings:** The low-dose DLIR-H group showed lower image noise. CNR of low-dose DLIR-H group in the ICA and MCA-M1 were higher than standard-dose FBP, ASIR-V40% groups, and low-dose DLIR-M group (P<0.05). In subjective evaluation, the low-dose DLIR-H group was superior to the standard-dose FBP, ASIR-V40% groups, and low-dose DLIR-M group (P<0.05). The radiation dose in the low-dose group was significantly lower than that in the standard dose group (P<0.05).
**Conclusion:** Reconstruction of low-dose CTP arterial phase images through DLIR may aid in the detection of small responsible vessels. It provides a valuable low-dose scanning option for clinical evaluation without the need for additional scanning protocols and extra radiation exposure and shortens the examination time.
**Limitations:** The number of AIS patients included in our study was limited and more cases are expected for further studies.
**Ethics committee approval:** Our retrospective study has obtained approval from the Institutional Review Board.
**Funding for this study:** No funding was received for this study.
**Author Disclosures:**
Zhihao Wang: Nothing to disclose
Weimeng Cao: Nothing to disclose
Song Wei Yue: Nothing to disclose
Limin Lei: Nothing to disclose
Yuhan Zhou: Nothing to disclose

### RPS 917-6

**Evaluation of an optimised CT protocol for the assessment of suspected cauda equina syndrome and nerve root compression**

*P. J. Dempsey*, G. Lambe, D. T. Ryan, J. W. Power, A. Yates, G. Kenny,
P. J. Macmahon; Dublin/IE

**Purpose:** The evaluation of suspected cauda equina syndrome and nerve root compression relies on urgent MRI. However, access to MRI can be limited in many healthcare systems, especially in the out-of-hours setting. CT, by comparison, is more readily accessible. The purpose of this study was to evaluate the diagnostic accuracy of an optimised CT lumbar spine protocol in the assessment of suspected cord and nerve root compression using MRI as the reference standard.
**Methods or Backround:** Individuals presenting to the emergency department and referred for MRI for suspected nerve root compression were prospectively enrolled to have a CT spinal stenosis protocol in addition to MRI. Central canal and neural foraminal stenosis was qualitatively and quantitatively assessed at each lumbar level on CT by an expert radiologist blinded to the clinical information and outcome. Following a 4 week interval the process was repeated for each MRI.
**Results or Findings:** 59 individuals were included in the final analysis. In 22 (39%) cases no significant stenosis was identified. In a further 22 (37%) cases disc pathology was identified that was managed conservatively. 13 (22%) individuals proceeded to urgent surgical decompression. In one (2%) instance an alternative diagnosis was identified. The sensitivity, specificity, positive and negative predictive values for CT in detecting acute neural compression were 97% (CI: 82-99%), 97% (CI: 83-99%), 97% (CI: 92-99%) and 97% (CI 83-99%) respectively.
**Conclusion:** CT accurately detected nerve compression and emergent surgical lesions. This can be a useful tool in selecting appropriate patients for emergent MRI in the on-call setting to allow for better emergency resource allocation.
**Limitations:** The study was limited by its relatively small sample size, its reliance on external multicentre validation, and from the lack of a universally agreed stenosis assessment method. This was a single centre study.
**Ethics committee approval:** This study was approved by an ethics committee.
**Funding for this study:** No funding was received for this study.
**Author Disclosures:**
Peter Joseph Macmahon: Nothing to disclose
Philip Jude Dempsey: Nothing to disclose
David Thomas Ryan: Nothing to disclose
Andrew Yates: Nothing to disclose
Jack W. Power: Nothing to disclose
Grace Kenny: Nothing to disclose
Gerard Lambe: Nothing to disclose

### RPS 917-7

**Relevance of computed tomography for the detection of septic foci: a systematic review and meta-analysis**

*A-C. Stahl*, K. Rubarth, M-I. Opper Hernando, G. Muench, M. Dewey,
J. Pohlan; Berlin/DE
*(ann-christine.stahl@charite.de)*

**Purpose:** This systematic review and meta-analysis was conducted to investigate the diagnostic accuracy of computed tomography (CT) in patients with suspected or confirmed sepsis.
**Methods or Backround:** We searched Medline by using PubMed and Embase by using Ovid with a systematic search strategy to identify studies assessing the role of CT in patients with suspected sepsis or sepsis of unknown origin. Reviews, meta-analyses, editorials, comments, or case reports were not eligible for inclusion. In addition, we searched the references of the included studies to ensure no relevant studies were missed. PubMed was last searched on 01.01.2023 and Ovid on 22.01.2023. We extracted the sensitivity and specificity using 2-by-2 tables from each study. The methodological quality was assessed with the help of the QUADAS-2 tool. Pooled estimates of per-CT sensitivity, specificity, and diagnostic odds ratio were calculated by using random effects meta-analysis for proportions via logit transformation and presented as forest plots. This review was registered in PROSPERO (ID: CRD42023412009).
**Results or Findings:** Five studies which included an overall of 838 patients met our inclusion criteria. The different reference standards used were the final diagnosis from the discharge note, operation, local drainage, needle aspiration, and autopsy. The pooled estimate of sensitivity was 83.2% (95% CI: 69.5–91.4%) while the pooled estimate of specificity was 62.4% (95% CI: 49.9-73.5%). The pooled estimate of the diagnostic odds ratio was 9.0 (95% CI: 2.2-36.3). Both sensitivity (I2=67%, p=0.02) and odds ratio (I2=77%, p<0.01) showed significant variation between the studies.
**Conclusion:** CT has good sensitivity but limited specificity in patients with suspected or confirmed sepsis and may thus be considered an appropriate test.
**Limitations:** Due to the low number of included studies as well as their heterogeneity the generalizability of our results may be limited.
**Ethics committee approval:** As the presented study is a systematic review and meta-analysis no approval by an ethics committee is needed.
**Funding for this study:** No funding was received for this study.
**Author Disclosures:**
Julian Pohlan: Nothing to disclose
Marc Dewey: Other: Institutional research agreements: Siemens, General Electric, Philips, Canon Grant Recipient: EU (EC-GA 603266 in HEALTH.2013.2.4.2-2) DFG (DE 1361/14-1, DE 1361/18-1, BIOQIC GRK 2260/1, Radiomics DE 1361/19-1 [428222922] and 20-1 [428223139] in SPP 2177/1), GUIDE-IT (DE 1361/24-1), Berlin University Alliance (GC_SC_PC 27), Berlin Institute of Health (Digital Health Accelerator); Other: Editor: Cardiac CT (Springer Nature); Board Member: M.D. is European Society of Radiology (ESR) Publications Chair (2022-2025); the opinions expressed in this presentation are the author’s own and do not represent the view of ESR.; Other: Hands-on cardiac CT courses (www.ct-kurs.de); Patent Holder: Patent on fractal analysis of perfusion imaging (jointly with Florian Michallek, EPO 2022 EP3350773A1, and USPTO 2021 10,991,109, approved).
Ann-Christine Stahl: Nothing to disclose
Gloria Muench: Nothing to disclose
Maria-Isabel Opper Hernando: Nothing to disclose
Kerstin Rubarth: Nothing to disclose

### RPS 917-8

**The problem-solving role of the 'wait and repeat CT' approach in the diagnosis and treatment of acute abdomen**

*E. Akçiçek*, A. G. Erdemir, I. S. Idilman, M. R. Onur, E. Akpinar; Ankara/TR
*(esra.akcicek@hotmail.com)*

**Purpose:** Computed tomography (CT) findings in patients with acute abdominal symptoms are sometimes insufficient to make a definitive diagnosis. Patients may be offered repeat imaging and, to our knowledge, the results of this approach have not been studied. We aimed to investigate the impact of the 'wait and repeat CT' approach on the diagnostic and therapeutic processes of emergency patients with acute abdominal symptoms who could not be diagnosed with the initial CT but were diagnosed with a repeat CT.
**Methods or Backround:** Approval was obtained from the ethics committee. Patients admitted to the emergency department between January 2013 and July 2023 (≥18 years) were selected from those who had a repeat CT examination on the same admission. Trauma patients and initial examinations with artefacts were excluded. According to the findings of repeat CT, patients were divided into five subgroups: preliminary diagnosis did not change (group-A), suspicious preliminary diagnosis confirmed (group-B), progression after initial diagnosis (group-C), regression after initial diagnosis (group-D), and completely different diagnosis (group-E). The diagnoses in each group and the relationships between the groups were analysed statistically.
**Results or Findings:** 228 examinations were found, of which 72 were excluded due to artefacts and seven due to trauma. Of the 149 patients, 21 were in group-A (14.09%), 60 in group-B (40.26%), 32 in group-C (21.47%), 25 in group-D (16.77%), and 11 in group-D (7.38%). Overall, the most frequent cases were partial bowel obstruction (n: 41, 27.51%) and the majority of patients whose findings regressed to the second CT (group-D; n:18, 72.0%). The "wait and repeat" approach is statistically significant in the follow-up and discharge decision of patients with partial obstruction (p<0.01).
**Conclusion:** The 'wait and repeat' approach may be helpful in follow-up of acute abdomen, especially if it is a bowel obstruction.
**Limitations:** The number of patients was limited.
**Ethics committee approval:** This study was approved by the Hacettepe University Ankara, Ethics commitee No: GO 23/566.
**Funding for this study:** No funding was received for this study.
**Author Disclosures:**
Esra Akçiçek: Nothing to disclose
Ahmet Gürkan Erdemir: Nothing to disclose
Ilkay Sedakat Idilman: Nothing to disclose
Erhan Akpinar: Nothing to disclose
Mehmet Ruhi Onur: Nothing to disclose

### RPS 917-9

**The distribution of computed tomography findings for nonagenarians with acute abdominal conditions: a descriptive study for more than a decade of observation**

A. G. Erdemir, *A. N. Bolukcu*, E. Akçiçek, M. R. Onur, I. S. Idilman, E. Akpinar; Ankara/TR
*(bolukcuaysenur@gmail.com)*

**Purpose:** The objective of the study was to apply computed tomography (CT), which is useful to determine the causes of acute abdomen in nonagenarian (≥90 years) individuals, since they are less likely to present with the traditional symptoms. We aimed to describe the CT scan findings of acute abdominal etiologies in nonagenarian patients over a ten-year period, which to our knowledge has never been studied in this context.
**Methods or Backround:** Nonagenarians with acute abdomen admitted to the emergency department between January 2013 and June 2023 with CT (n:620) were studied. Of these, trauma patients (n:112) were excluded. For the remaining 508 patients, an assessment was made based on symptoms, demographics, etiology, surgical interventions, and mortality within the following day, week, and month.
**Results or Findings:** Positive findings for acute abdomen were detected in 258 patients (50.78%). There were 131 patients (50.77%) requiring surgical intervention and 54 of them (20.93%) were able to undergo surgical intervention. According to primary acute abdominal pathology, they were categorized as follows: bowel obstruction (n:36, 13.95%), acute cholecystitis (n:29, 11.24%), arterial occlusion (n:28, 10.85%), stercoral colitis (n:21, 8.13%), bowel infection (n:16, 6.20%), kidney stone (n:15, 5.81%), bowel perforation (n:8, 3.10%), venous occlusion (n:6, 2.32%), acute appendicitis (n:4, 1.55%). We categorised patients based on their mortality within the following day (22.72%), week (34.09%), and month (43.18%). Malignancy (n:114, 22.48%) was the most frequent chronic abdominal pathology accompanying them.
**Conclusion:** The spectrum of acute abdominal etiology in nonagenarians, studied for the first time on such a large scale, differs considerably from other segments of the population. Most importantly, admission to CT for acute abdomen in nonagenarians can be an indicator of the likelihood that more than a third (or even nearly half) may die within a month.
**Limitations:** It was a single-centered and retrospective study.
**Ethics committee approval:** This study was approved by Hacettepe University Noninterventional Clinical Research Ethics Committee.
**Funding for this study:** No funding was received for this study.
**Author Disclosures:**
Esra Akçiçek: Nothing to disclose
Ahmet Gürkan Erdemir: Nothing to disclose
Ilkay Sedakat Idilman: Nothing to disclose
Erhan Akpinar: Nothing to disclose
Ayse Nur Bolukcu: Nothing to disclose
Mehmet Ruhi Onur: Nothing to disclose

**12:30-13:30 Research Stage 2**

### Research Presentation Session: Physics in Medical Imaging

### RPS 913

### Spectral imaging: quantification and performance evaluation

**Moderator**
O. Rampado; Turin/IT
*(orampado@cittadellasalute.to.it)*

### RPS 913-2

**Comparison of image quality of abdominal CT examinations and virtual noncontrast images between photon-counting and energy-integrating detector CT**

*L. Lofino*, F. Schwartz, F. Ria, M. Z. Zarei, E. Samei, A. Abadia, D. Marin; Durham, NC/US
*(ludovica.lofino@humanitas.it)*

**Purpose:** The purpose of this study was to compare the image quality of portal venous phase (PVP) abdominal CT examinations and virtual non-contrast images (VNC) between photon-counting CT (PCCT) and energy-integrating Detector CT (EID).
**Methods or Backround:** Multi-phase CT scans from one PCCT and two EID CTs were retrieved. 45 BMI-matched patients were included: 15 for PCCT and

30 for EID. In vivo image quality parameters were measured and compared for PVP and VNC. CTDIvol values were also recorded for all examinations. Because scanner tube current modulation adapts to patient size, the radiation dose was compared among scanners accounting for BMI using a figure of merit: FOM=1/(BMI*lnCTDIvol). A five-point scale was used to assess the reader's perception of image quality.
**Results or Findings:** Compared to the two EID, PCCT yielded significantly improved resolution and noise magnitude for both PVP (MTFf10 = 0.55 ± 0.08 for PCCT vs. 0.50 ± 0.04 and 0.49 ± 0.03 for Flash and Force; noise = 9.76 ± 3.10 vs. 15.35 ± 4.14 and 10.70 ± 1.34) and VNC (MTFf10 = 0.56 ± 0.01 for PCCT vs. 0.51 ± 0.05 and 0.51 ± 0.03 for Flash and Force; noise = 9.59 ± 2.77 vs. 13.90 ± 3.57 and 10.83 ± 2.83), P<0.02. A similar trend was confirmed in a subset of overweight patients. Our FOM analysis suggests that, for equal radiation exposure levels and comparable patient size, PCCT yields 20% noise reduction compared to the two EID, with 18% reduction in overweight patients. Reader's perceived image noise was lower and overall image quality was higher for PCCT compared to EID.
**Conclusion:** PCCT yields a significantly lower radiation dose, with improved image quality in both the PVP and VNC of abdominal CT examinations.
**Limitations:** This was a single center retrospective study with a limited number of cases.
**Ethics committee approval:** An ethics committee approved the study.
**Funding for this study:** No funding was received for this study.
**Author Disclosures:**
Ludovica Lofino: Nothing to disclose
Andres Abadia: Employee: Siemens Healthineers, USA
Fides Schwartz: Nothing to disclose
Daniele Marin: Research/Grant Support: Siemens Healthineers, USA
Ehsan Samei: Nothing to disclose
Francesco Ria: Nothing to disclose
Mojtaba Zarei Zarei: Nothing to disclose

### RPS 913-3

### Assessment of artefacts, material identification, and quantification in the presence of metal objects using photon-counting spectral CT

B. Tariq[1], N. Werghi[1], E. I. Memisoglu[1], N. Maalej[1], O. Sikander[2], F. Naseer[2], *A. Y. Y. Raja*[1]; [1]Abu Dhabi/AE, [2]Islamabad/PK
*(aamir.raja@ku.ac.ae)*

**Purpose:** Metal artefacts in computed tomography (CT) obscure the visualisation and assessment of anatomical structures and lead to misinterpretation of patient diagnosis and treatment. This study aims to use photon-counting spectral CT and evaluate the identification and quantification of clinically relevant materials such as iodine (as a contrast agent) and hydroxyapatite (a mineral in bones and teeth) in the presence of metal objects.
**Methods or Backround:** A multi-material phantom was used with inserts of varied quantities of iodine (4.83, 9.66, and 14.56 mg/cm3) and hydroxyapatite (201.6 and 406.9 mg/cm^3). Three sets of scans were acquired: one without a metal insert, one with steel, and one with aluminium. Image acquisition used a Mars spectral scanner (Microlab 5x120) operated at 118 kVp, 80 µA, and 160 ms with 981 circular projections. Images were reconstructed in five energy bins: 7-40, 40-50, 50-60, 60-79, and 79-118 keV. Energy and material-density images were assessed by linear regression, sensitivity, specificity, area under the curve (AUC), and root-mean-square-error (RMSE). For demonstrative purposes, a biological sample (a sheep heart) with a steel insert was also scanned and evaluated.
**Results or Findings:** Results indicate reduced metal artefacts and an enhanced signal-to-noise ratio (up to 25%) in the higher energy bins. All energy bins revealed strong linearity (R^2>0.97) across the concentrations of material. Material identification and quantification were measured for iodine (without metal- Sensitivity 80%; Specificity 90%; AUC 0.80; RMSE 22%; with metal- Sensitivity >77%; Specificity >90%; AUC >0.74; RMSE 26%) and hydroxyapatite (without metal- Sensitivty 83%; Specificity 93%; AUC 0.8; RMSE,14%; with metal- Sensitivity >78%; Specificity >93%; AUC >0.77; RMSE >22%). Images of the biological sample showed comparable results to the multimaterial phantom.
**Conclusion:** SPCCT can accurately identify and quantify clinically relevant materials, such as iodine and hydroxyapatite, in the presence of metal objects.
**Limitations:** No limitations were identified.
**Ethics committee approval:** No information provided by the submitter.
**Funding for this study:** Funding was received through the Research and Innovation grant from the Khalifa University, UAE (Project#: 8474000563).
**Author Disclosures:**
Briya Tariq: Nothing to disclose
Aamir Younis Younis Raja: Nothing to disclose
Esat Izzet Memisoglu: Nothing to disclose
Osama Sikander: Nothing to disclose
Nabil Maalej: Nothing to disclose
Farhat Naseer: Nothing to disclose
Naoufel Werghi: Nothing to disclose

### RPS 913-4

### Iodine quantification accuracy kVp-switching dual-energy CT

S. Blazis[1], T. Ruytenberg[2], N. De Jong[2], *J. Heemskerk*[2]; [1]Goes/NL, [2]Leiden/NL
*(j.w.t.heemskerk@lumc.nl)*

**Purpose:** The purpose of this study was to determine (1) whether quantification of iodinated contrast medium of a kVp-switching dual-energy CT is accurate, and (2) what the limit of this quantification is.
**Methods or Backround:** Dual-energy CT has been proposed as a tool for semi-quantitative perfusion imaging, using iodine concentration maps. We have investigated the accuracy and limitations of iodine quantification of a fast kVp-switching DECT. Two phantoms were scanned: a Gammex multi-energy phantom, and a 180mm diameter cylindrical water-filled ('linearity') phantom containing twelve 16mm diameter tubes with iodinated contrast medium with concentrations ranging from 0 to 30 mgI/ml.
**Results or Findings:** In line with what has been documented elsewhere, measurements with the 'linearity' phantom indicate that lower iodine concentrations (<2mgI/ml) are underestimated with 60-20%. For higher concentrations, the accuracy of iodine quantification is in the order of a few percent. Nevertheless, depicted iodine concentrations increase monotonously with actual concentrations, indicating potential for scoring purposes or thresholding for clinical applications. Gammex phantom images show that distinction between iodine and calcium is challenging for the kVp-switching DECT. This is also apparent from VNC images, where iodine concentrations >3mgI/ml are no longer fully suppressed, and HU values for calcium inserts are reduced.
**Conclusion:** Dual-energy CT is increasingly proving itself as a useful expansion of CT imaging. Discrimination and quantification of iodinated contrast medium is a promising additional functionality for, for example, detection of intra-cranial hemorrhage or myocardial defect. Fast kVp-switching DECT switching facilitates this iodine quantification, but within limits, and allows creation of virtual non-contrast maps at no extra dose cost. Quantitative iodine imaging with CT remains challenging for clinically relevant concentrations <3mgI/ml, and further investigation of accuracy of iodine quantification and VNC-maps is warranted.
**Limitations:** This project included phantoms scans only.
**Ethics committee approval:** This project only included phantom scans.
**Funding for this study:** No funding was received for this study.
**Author Disclosures:**
Nick De Jong: Nothing to disclose
Thomas Ruytenberg: Nothing to disclose
Stephan Blazis: Nothing to disclose
Jan Heemskerk: Nothing to disclose

### RPS 913-5

### Performance of iodine quantification and monochromatic attenuation through high-pitch dual-source photon-counting CT: a phantom study

*P. Liu*, S. Zhou, Z. Xu, H. Dong, J. Li, S. Lin, W. Yang, F. Yan, L. Qin; Shanghai/CN
*(liuyynp@163.com)*

**Purpose:** The objective of this study was to investigate the feasibility and accuracy of iodine quantification and monoenergetic attenuation using PCD-CT in standard pitch and high-pitch scanning at different scan parameters in a phantom model.
**Methods or Backround:** Four inserts with known iodine concentrations (2, 5, 10, and 15 mg/mL) were placed in the removable CT phantom and scanned using high-pitch (3.2) and standard pitch (0.8) on PCD-CT. Two tube voltages (120 and 140 kVp) and four radiation doses (1, 3, 5, and 10 mGy) were alternated. Each scan setting was repeated three times. Mean iodine concentration and monoenergetic attenuation were recorded. Percentage absolute bias (PAB) was assessed for iodine quantification. Image noise and monoenergetic attenuation were evaluated at 40, 70, 100, and 140 keV.
**Results or Findings:** 96 acquisitions were conducted. In small phantom, the PAB was 2.96% (1.75%, 4.56%) and 1.67% (1.00%, 3.42%) for high-pitch and standard pitch, respectively. In large phantom, these numbers were 3.72% (1.75%, 5.97%) and 2.94% (1.75%, 4.70%) respectively. Linear regression analysis revealed that only phantom size significantly influenced (P < 0.001) the accuracy of iodine quantification. Background noise increased with a decrease in keV level and radiation doses. Attenuation errors at 70, 100, and 140 keV remained below 10 HU, with 37.5% cases surpassing 10 HU at 40 keV. Linear regression analysis revealed comparable accuracy of monoenergetic attenuation between high-pitch and standard pitch (P=0.332).
**Conclusion:** High-pitch scanning in PCD-CT can be used to quantify iodine density and monoenergetic CT values with high accuracy, thereby potentially benefiting multienergy-based tissue differentiation and material decomposition in clinical settings.
**Limitations:** The results of our study were focused on a phantom and have not been verified in patients, which must be conducted in the future.
**Ethics committee approval:** The study is a phantom experiment.
**Funding for this study:** No funding was received for this study.

**Author Disclosures:**
Shushen Lin: Nothing to disclose
Wenjie Yang: Nothing to disclose
Le Qin: Nothing to disclose
Zhihan Xu: Nothing to disclose
Fuhua Yan: Nothing to disclose
Haipeng Dong: Nothing to disclose
Peng Liu: Nothing to disclose
JiQiang Li: Nothing to disclose
Shanshui Zhou: Nothing to disclose

### RPS 913-6

**Multi-contrast ultra-high-resolution K-edge imaging with edge-on deep-silicon photon-counting detectors: phantom evaluations of a clinical prototype scanner**

T. W. Holmes[1], N. Nezami[2], A. Jobayer[3], R. Bujila[4], J. Maltz[3], Z. Yin[5], *A. Pourmorteza*[1]; [1]Atlanta, GA/US, [2]Baltimore, MD/US, [3]Waukesha, WI/US, [4]Stockholm/SE, [5]Niskayuna, NY/US
*(amir.pourmorteza@gmail.com)*

**Purpose:** Photon-counting detectors (PCD) have helped improve diagnostic value of CT by providing spectral information, increasing the spatial resolution, and reducing the radiation dose. The combination of these advantages could enable dose-efficient ultra-high-resolution (UHR) multi-contrast imaging by using the K-edges of materials. Properly timed injections of two contrast agents can generate co-registered maps of the anatomy in different perfusion phases and eliminate multi-phasic examinations. Moreover, use of solid k-edge materials such as gold and tungsten as fiducial markers in surgical tools could improve CT-guided interventions. Si-based PCDs with eight adjustable thresholds are a new development that promise better spatial and spectral resolution compared to the existing CdTe/CZT technologies. We investigated the utility of Si-PCD in multi-contrast UHR tasks.
**Methods or Backround:** We prepared a series of anthropomorphic phantoms mimicking coronary arteries with atherosclerotic plaques and stents, tumours embolised with radiopaque microspheres, and human head including various concentrations and mixtures of iodine- and gadolinium-based contrast agents. Another series were made including different components of a 1-mm micro-robot designed for neurosurgery containing solid neodymium and tungsten components. The phantoms were scanned on a whole-body prototype Si-PCD scanner and material-specific UHR images were generated. We compared iodine and gadolinium quantification, image resolution, and material separation of the system to a state-of-the-art dual-energy energy-integrating detector (EID) CT scanner.
**Results or Findings:** While the dual-energy EID system failed at separating the two materials, 8-energy Si-PCD could distinguish them. Iodine quantification accuracy was not significantly different between the two systems in the absence of gadolinium. Gadolinium accuracy was [-0.3 0.7] mgGd/mL for Si-PCD. Spatial resolution was significantly higher in Si-PCD as measured by in-stent and free lumen diameters.
**Conclusion:** Si-PCD has the potential to improve spatial resolution and provide multi-material quantification simultaneously.
**Limitations:** This study utilised a prototype scanner.
**Ethics committee approval:** No information provided by the submitter.
**Funding for this study:** This study was part of a sponsored research agreement with GE Healthcare.
**Author Disclosures:**
Jonathan Maltz: Employee: GE HealthCare
Robert Bujila: Employee: GE HealthCare
Amir Pourmorteza: Employee: Emory University; Research/Grant Support: Cannon Medical USA; Research/Grant Support: Siemens Healthineers; Research/Grant Support: GE HealthCare; Advisory Board: CarioWise Inc
Nariman Nezami: Employee: University of Maryland School of Medicine
Thomas Wesley Holmes: Research/Grant Support: Siemens Healthineers; Employee: Emory University; Consultant: CarioWise Inc; Research/Grant Support: GE HealthCare; Research/Grant Support: Cannon Medical USA
Zhye Yin: Employee: GE HealthCare
Ali Jobayer: Employee: GE HealthCare

### RPS 913-7

**Dual-energy tomosynthesis of the chest using a triple-layer x-ray detector**

*S. Tilley*, J. Potipcoe, K. S. Karim; Waterloo, ON/CA
*(stilley@kaimaging.com)*

**Purpose:** The purpose of this study was to investigate the feasibility of dual energy (DE) tomosynthesis using a triple-layer x-ray detector. Of particular interest is whether a set of DE images, acquired with a tomosynthesis acquisition protocol and processed using a traditional DE algorithm, can be reconstructed into material specific three-dimensional volumes. Adding the material separation capabilities of DE with the depth localisation of tomosynthesis may provide enough patient information to negate the costly use of computed tomography (CT) for many patients. Furthermore, using a multi-layer detector for DE as opposed to multiple exposures may permit portable, spectral tomosynthesis.
**Methods or Backround:** We acquired 61 projection images of a chest phantom using the Reveal 35C x-ray detector. The x-ray source was linearly translated parallel to the phantom’s longitudinal axis to produce an angular sweep of 30 degrees. A a standard digital radiograph (DR) and two DE images, bone and soft-tissue, were calculated from each triple-layer projection image. A variant of logarithmic subtraction was used for the DE images, using the same parameters for each projection. We reconstructed these three datasets using the same model-based conjugate gradient algorithm with quadratic regularisation and a 5mm slice thickness.
**Results or Findings:** Three sets of tomographic slice data were reconstructed: DR, bone, and soft-tissue. Structures in these images were successfully separated by slice (localisation) and DE image (material identification). The DE data used the same reconstruction algorithm as the DR data, indicating that tomosynthesis can be readily applied to DE images.
**Conclusion:** This proof-of-concept study demonstrates the feasibility of DE tomosynthesis using a triple-layer detector. Future work will explore dose requirements, sensitivity to various pathologies, improved reconstruction techniques, and application in a portable system.
**Limitations:** No limitations were identified.
**Ethics committee approval:** No information provided by the submitter.
**Funding for this study:** No funding was received for this study.
**Author Disclosures:**
Karim S. Karim: Founder: KA Imaging, Inc
Steven Tilley: Employee: KA Imaging, Inc
Jay Potipcoe: Employee: KA Imaging, Inc

**12:30-13:30** **Research Stage 4**

### Research Presentation Session: Abdominal Viscera & GI Tract

### RPS 901

### Inflammatory bowel disease (IBD): an ongoing challenge in imaging

**Moderator**
I. De Kock; Ghent/BE
*(isabelle.dekock@ugent.be)*
**Author Disclosures:**
Isabelle De Kock: Research Grant/Support: Research grant from the Ghent University Hospital Fund for Innovation and Clinical Research

### RPS 901-2

**Multiparametric MRI in the assessment of CD transmural activity: a cluster analysis**

*F. Maccioni*, L. Busato, A. Valenti, S. Cardaccio, A. Longhi, M. Barletta, S. Oliva, M. Aloi, C. Catalano; Rome/IT
*(francesca.maccioni@uniroma1.it)*

**Purpose:** Several MRI parameters have been validated as expressions of Crohn's disease activity, using colonoscopy as the gold standard (GS). However, the only reliable GS for Crohn's disease activity is the pathological specimen, which is rarely available. This study was conducted to select the MRI parameters most indicative of disease activity, compared with several clinical reference GSs, but also independently of a GS, through a cluster analysis.
**Methods or Backround:** We retrospectively analysed 60 paediatric patients with Crohn's disease at onset and at follow up, who underwent MREnterography , ileo-colonoscopy and clinical-laboratory examinations in a 30-day time interval. The sixteen different MRI parameters most indicative of intestinal inflammation were intracorrelated and correlated with the Simple Endoscopic Score for Crohn's Disease, with the pCDActivity score, foecal calprotectin and C-reactive protein. Statistical analysis performed included cluster analysis and Cramer's analysis.
**Results or Findings:** Significant results were obtained using cluster analysis, which identified two clusters with severe (Group 1) and mild-to-moderate (Group 2) disease. Eight out of sixteen MRI parameters varied significantly within the clusters: the length, DWI, T2 fat suppressed wall signal, T2 fat suppressed fat signal, arterial-phase contrast enhancement, venous-phase contrast enhancement, late-phase contrast enhancement, stratified wall enhancement, and comb sign ($p<0.001$). No significant association was found between MRI, clinical, laboratory or endoscopic parameters, except a mild association between foecal calprotectin and arterial, venous and late-phase contrast enhancement.

**Conclusion:** The cluster analysis identified eight MRI parameters that significantly increased in mild to severe Crohn's disease. By grading these nine parameters, an effective non-invasive activity score for disease monitoring can be developed and validated in paediatric patients.
**Limitations:** This is a single-centre retrospective analysis. A deeper analysis of the missed correlation between MRI, clinical, and endoscopic parameters is required.
**Ethics committee approval:** No information provided by the submitter.
**Funding for this study:** No funding was received for this study.
**Author Disclosures:**
Alessandro Longhi: Nothing to disclose
Marina Aloi: Nothing to disclose
Sara Cardaccio: Nothing to disclose
Alessandra Valenti: Nothing to disclose
Salvatore Oliva: Nothing to disclose
Martina Barletta: Nothing to disclose
Carlo Catalano: Nothing to disclose
Ludovica Busato: Nothing to disclose
Francesca Maccioni: Nothing to disclose

### RPS 901-3

### Evaluation of body composition and bone density in human adults with inflammatory bowel diseases

*J. Ritter*[1], M. Nickel[1], M. El Husseini[1], P. H. Gouder[1], F. Nensa[2], J. S. Kirschke[1], R. Braren[1]; [1]Munich/DE, [2]Essen/DE

**Purpose:** Chronic inflammatory bowel diseases such as ulcerative colitis (UC) and Crohn's disease (CD) are associated with malabsorption disorders. These carry the potential to alter body parameters such as bone density and fat-muscle composition. The aim of this study was to detect and quantify potential changes in the age- and gender-specific parameters of bone density and body composition.
**Methods or Backround:** A total of 433 patients (n=153 UC, n=280 CD) with chronic inflammatory bowel disease were investigated retrospectively. The patient groups studied were age-correlated against each other and a reference cohort (n=811). All patients received a CT scan within the clinical setting. Bone marrow density (BMD) values were extracted using an custom-made segmentation and classification algorithm. Body tissue composition was assessed in a subcohort of 116 patients (35 UC, 81 CD) using a deep learning–based volumetric tissue classification system.
**Results or Findings:** Significantly reduced BMD compared to a reference cohort was seen in almost all age and gender groups in both UC and CD (p-value range: 1.35E-08 to 0.03). Within the subcohort, the body composition showed significantly lower muscle mass in male UC patients and gender-independent in patients with CD. The proportion of adipose tissue, on the other hand, did not significantly change. In the subgroup of CD patients, significantly lower muscle mass was found with short-term cortisone use, and among continuously-medicating female patients. However, earlier cortisone use did not lead to significant changes in muscle mass.
**Conclusion:** Chronic inflammatory bowel diseases are associated with loss of BMD and muscle mass. The latter are mainly observed in patients with Crohn's disease on recent cortisone therapy.
**Limitations:** The limitation of the study is the limited availability of therapy data leading to a relatively small subcohort.
**Ethics committee approval:** The study was approved by the Ethics Committee of the University Hospital rechts der Isar, Technical University of Munich (Number 87/18S).
**Funding for this study:** No funding was received for this study.
**Author Disclosures:**
Jessica Ritter: Nothing to disclose
Malek El Husseini: Nothing to disclose
Jan S. Kirschke: Nothing to disclose
Rickmer Braren: Nothing to disclose
Manuel Nickel: Nothing to disclose
Philip Hermann Gouder: Nothing to disclose
Felix Nensa: Nothing to disclose

### RPS 901-4

### CT-based radiomics signature of visceral adipose tissue and bowel lesions for identifying patients with Crohn's disease resistant to infliximab

*Y. Wang*[1], Z. Luo[2], Z. Zhou[1], X. Shen[1], X. Wang[1], B. Huang[2], S-T. Feng[1], X. Li[1]; [1]Guangzhou/CN, [2]Shenzhen/CN
*(wangydi@mail2.sysu.edu.cn)*

**Purpose:** The purpose of this study was to develop a radiomics model combining VAT and bowel features to improve the predictive efficacy of infliximab (IFX) therapy on the basis of bowel model.
**Methods or Backround:** This retrospective study included 231 CD patients (training cohort, n=112; internal validation cohort, n=48; external validation cohort, n=71) recruited from two tertiary centres. A machine-learning VAT model and bowel model were developed separately to identify CD patients with primary nonresponse to IFX. A comprehensive model consisting of VAT and bowel radiomics features was further established to verify whether features extracted from VAT would improve the predictive efficacy. Area under the curve (AUC) and decision curve analysis were used to compare the prediction performance. Clinical utility was compared using integrated differentiation improvement (IDI).
**Results or Findings:** VAT model and bowel model exhibited comparable performance for identifying patients with primary nonresponse in internal [AUC of VAT model vs bowel model, 0.737 (95% CI, 0.590-0.854) vs 0.832 (95% CI, 0.750-0.896)] and external validation cohort [AUC of VAT model vs bowel model, 0.714 (95% CI, 0.595-0.815) vs 0.799 (95% CI, 0.687-0.885)], with relatively good net benefit. Comprehensive models adding VAT into bowel model yielded a satisfactory predictive efficacy in both internal [AUC, 0.840 (95% CI, 0.706-0.930)] and external validation cohort [AUC,0.833 (95% CI, 0.726-0.911)], with significantly improved predictive efficacy (IDI=4.2% and 3.7%; both P<0.05).
**Conclusion:** VAT has an effect on IFX treatment response and improves the performance for identification of CD patients at high risk of primary nonresponse to IFX therapy.
**Limitations:** An initial limitation was the use of CTE rather than MRE to develop radiomics models. In future studies, CT-based radiomics framework may facilitate artificial development in the field of MR through transfer learning. Besides, the radiomics signatures extracted from single-phase images underutilised all of the information. Lastly, sample size is limited.
**Ethics committee approval:** This study was approved by the Institutional Review Board of The First Affiliated Hospital of Sun Yat-sen University (No. [2020]351), and the need for informed patient consent was waived.
**Funding for this study:** Funding was received from the National Natural Science Foundation of China [grant numbers 82070680, 82072002, 82270693, 82271958].
**Author Disclosures:**
Shi-Ting Feng: Nothing to disclose
Zhengran Zhou: Nothing to disclose
Bingsheng Huang: Nothing to disclose
Xinyue Wang: Nothing to disclose
Xuehua Li: Nothing to disclose
Xiaodi Shen: Nothing to disclose
Zixin Luo: Nothing to disclose
Yangdi Wang: Nothing to disclose

### RPS 901-5

### Comparison of findings on transabdominal ultrasound and magnetic resonance enterography with macroscopic changes on colonoscopy in patients with Crohn's disease

*V. Válek*, T. Rohan, L. Prokopova, D. Bartusek, M. Smela, S. Konecny, J. Husty, V. Zboril, P. Kavrikova; Brno/CZ
*(atavalek@gmail.com)*

**Purpose:** This study sought to compare the sensitivity and specificity of ultrasound and magnetic resonance (MRI) enterography in the evaluation of inflammatory changes in Crohn's disease (CD) in patients with different macroscopic findings on colonoscopy.
**Methods or Backround:** This retrospective study analysed 47 consecutive patients with CD who underwent contrast enhanced MRI enterography, expert bowel ultrasound and colonoscopy within 1 month at the University Hospital between 2018 and 2022. Based on the endoscopic findings, patients were divided into four groups: 1 (ulcers), 2 (ulcers and aphthae below 5mm), 3 (aphthae below 5mm, oedema, erythema), and 4 (aphthae below 5mm). In all patients, four segments of the colon and terminal ileum were evaluated (together 215 segments). The sensitivity and specificity of ultrasound and MRI in the evaluation of inflammatory changes were compared in all analysed locations (Fisher's exaxt test). Macroscopic colonoscopic findings were the gold standard.
**Results or Findings:** Group 1 achieved significantly better sensitivity and specificity in the assessment of inflammatory activity in CD than the other groups (p<0.001), with sensitivity and specificity on MRI 90% and 91% and 88% and 87% on ultrasound. The combination of ultrasound and MRI findings increased sensitivity and specificity to 94% and 93% (p=0.02; 0.003). In the independent colon evaluation, ultrasound achieved higher sensitivity (89%, 81%, 58%, 60%) compared with MRI (83%, 63%, 33%, 20%) in all groups. The difference was significant in group 1 and 2 (p<0.001) and nonsignificant in groups 3 and 4 (p=0.622; 0.576).
**Conclusion:** A combination of MRI and bowel ultrasound is useful in the evaluation of bowel activity in patients with CD. Ultrasound can contribute to the assessment of disease activity, especially in the colon, compared to MRI.
**Limitations:** The study was limited by being retrospective, and by its small patient cohort.
**Ethics committee approval:** This study was approved by the Institutional Review Board of the University Hospital, Brno.

**Funding for this study:** No funding was received for this study.
**Author Disclosures:**
Vladimir Zboril: Nothing to disclose
Stefan Konecny: Nothing to disclose
Lucie Prokopova: Nothing to disclose
Jakub Husty: Nothing to disclose
Daniel Bartusek: Nothing to disclose
Pavla Kavrikova: Nothing to disclose
Tomas Rohan: Nothing to disclose
Vlastimil Válek: Nothing to disclose
Marketa Smela: Nothing to disclose

### RPS 901-6

**A rare course of fistula and location of abscess in patients with perianal fistula: a sign for Crohn’s disease?**
*S. N. Yılmazer Zorlu*, D. Kuru Öz, G. A. Erden; Ankara/TR
*(sezernilyilmazer@gmail.com)*

**Purpose:** The objective of this study is to compare the presence of intramural abscesses formed within the internal anal sphincter, which is the extension of the circular muscle layer of the rectum and a distinct craniocaudal course of the perianal fistula within external the anal sphincter (EAS) in patients with and without Crohn's disease (CD).
**Methods or Backround:** Magnetic resonance images (MRI) with possible presence of perianal fistulas obtained between 2011 and 2023 were retrospectively rewiewed. Patients with malignancy, ulcerative colitis, haematological disease, and anorectal surgery were excluded. We divided our cohort into two groups according to medical records; those with CD and those without any underlying aetiology. Images were evaluated for both number and type of fistula according to Park’s classification, localisation of internal orifice ,presence of course within EAS, and presence of intramural abscesses.
**Results or Findings:** Of the 578 patients with perianal fistula, 109 patients had CD while 469 patients did not have any known disease and have been considered idiopathic. There were 19 patients with concomitant intramural abscess (CD:14, idiopathic:5). There was a statistically significant difference between the groups in terms of the presence of intramural abscess($p<0.001$). In CD patients, it was statistically observed that the internal orifice was more frequently located in the distal rectum and at the 3 o'clock position relative to the anal clock, compared to the idiopathic group($p<0.001$; $p=0.04$ respectively). There were 60 fistulas (idiopathic:55, CD:5) that extended along the course of the external sphincter. 66.7% of extrasphincteric fistulas were found in CD patients, although other fistula types were primarily seen as idiopathic. Regarding the fistula localization and presence of course within EAS, there was significant difference between the groups ($p<0.001$).
**Conclusion:** Despite the rarity, intramural abscess may be indicative of CD.
**Limitations:** The findings of this paper must be confirmed with studies involving larger patient numbers. Furthermore, comparisons cannot be made with the intraoperative findings.
**Ethics committee approval:** This study was approved by the Ankara University Clinical Research Ethics Committee.
**Funding for this study:** No funding was received for this study.
**Author Disclosures:**
Sezer Nil Yılmazer Zorlu: Nothing to disclose
Diğdem Kuru Öz: Nothing to disclose
Gül Ayşe Erden: Nothing to disclose

### RPS 901-7

**Integrating gut microbiota and metabolites to decode variations in magnetic resonance enterography features of bowel damage in Crohn's disease: development of a multi-scale diagnostic model**
*L. Huang*, R. Zhang, X. Shen, Y. Wang, X. Wang, Z. Li, Z. Li, X. Li; Guangzhou/CN
*(huanglli27@mail2.sysu.edu.cn)*

**Purpose:** Bowel damage (BD) significantly impacts Crohn's disease (CD) patient's prognosis, showing varied magnetic resonance enterography (MRE) features. This study seeks to investigate the potential mechanisms underlying MRE features of BD using multi-scale data and develop an optimal diagnostic model for BD.
**Methods or Backround:** 230 CD patients were prospectively recruited and categorized into BD (n=103) and non-BD (n=127) using the Lémann index. All patients underwent MRE. Faecal 16S rRNA gene sequencing, and faecal and blood metabolomics were conducted. Relationships between MRE findings, gut microbiota, and faecal/blood metabolites were analysed using causal mediation analysis. Diagnostic models for BD were constructed using gut microbiota, faecal metabolites, blood metabolites, and MRE findings alone or in combination, with their performance assessed using ROC analysis.
**Results or Findings:** Seven MRE features, including penetration, bowel thickness, and perienteric T2WI signal, differed significantly between BD and non-BD patients. The components of gut microbiota and faecal/blood metabolites were distinct between the two groups. Causal mediation analysis revealed that Blautia may promote intestinal penetration accompanied by perienteric inflammation (i.e., perienteric T2WI signal) through faecal arachidonic acid and blood ceramide (d18:2/23:1). Individual component models, including intestinal microbiota, faecal metabolites, and blood metabolites, yielded AUCs of 0.588 (95% CI: 0.453 - 0.713), 0.671 (95% CI: 0.538 - 0.787), 0.640 (95% CI: 0.533 - 0.737), respectively. Incorporating MRE into these factors to construct a multi-scale model increased the AUC by 0.708 (95% CI: 0.582 - 0.823).
**Conclusion:** BD-induced MRE findings are partially attributable to microbial and metabolite factors, providing novel insights into the possible mechanisms driving these MRE alterations. The inclusion of MRE enhances diagnostic performance of the multi-scale model for BD, rendering it a promising tool for BD diagnosis.
**Limitations:** The diagnostic model lacks validation in an external dataset.
**Ethics committee approval:** This study was approved by the Ethics Committee of the First Affiliated Hospital, Sun Yat-sen University (No. IIT-2021[215], Guangzhou, China).
**Funding for this study:** No funding was received for this study.
**Author Disclosures:**
Zhoulei Li: Nothing to disclose
Lili Huang: Nothing to disclose
Xinyue Wang: Nothing to disclose
Ruonan Zhang: Nothing to disclose
Xuehua Li: Nothing to disclose
Yangdi Wang: Nothing to disclose
Xiaodi Shen: Nothing to disclose
Zhoulei Li: Nothing to disclose

### RPS 901-8

**Changes in 3D volumetry of perianal fistulas can predict the clinical outcome in perianal fistulising Crohn's disease**
*J. Rimola Gibert*[1], B. Caballol[1], C. Saavedra[2], S. Rodriguez Gomez[1], A. Fernàndez-Clotet[1], M. C. Masamunt[1], E. Ricart[1], I. Ordás[1]; [1]Barcelona/ES, [2]Ibague/CO
*(jrimola@clinic.cat)*

**Purpose:** Changes in the 3D volumetry of perianal fistulas measured by MRI could be an imaging biomarker of interest. The objective of the study is to determine the value of volumetric changes in perianal fistulas in patients with Crohn's disease (CD) after medical treatment to predict subsequent outcome.
**Methods or Backround:** This was a retrospective single-center pilot study. We have included CD patients with perianal fistulas who started biological therapy between 2012 and 2020 and have (1) both pre- and post-treatment (3-18 months) pelvic MRI and (2) follow-up after post-treatment MRI of at least 2 to 5 years. According to the last visit, patients were categorised as clinically active or in remission (absence of suppuration). Using specific software we calculated the 3D volumetry of the fistulas, the active component (hypersignal on T2) and the fibrotic component (hyposignal on T2). We compared MRI volumetric changes between both MRIs and calculated the value of MRI volumetric changes in predicting remission using a multivariate stepwise logistic regression analysis.
**Results or Findings:** Among the 24 patients included, 13 were in perianal remission in the last follow-up. We determined that the % of change of % of active component as predictor of clinical remission (OR 1.06 [1.02-1.11] $P=0.008$). The logistic regression analyses indicated that the AUC of the ROC curve was 85,3% ($p<0.0001$). Our results show that a reduction of ≥16% of the % active component of the fistula has a sensitivity of 84.6 and specificity of 81% to predict clinical remission.
**Conclusion:** Changes in the 3D volumetry of perianal fistulas on MRI have value in predicting clinical evolution. The decrease in the active component could be a therapeutic target.
**Limitations:** The low number of patients and the follow-up range are the main limitations of this study.
**Ethics committee approval:** This study was approved with the local code HCB/2021/0380.
**Funding for this study:** No funding was received for this study.
**Author Disclosures:**
Berta Caballol: Nothing to disclose
Maria Carme Masamunt: Nothing to disclose
Ingrid Ordás: Nothing to disclose
Agnès Fernàndez-Clotet: Nothing to disclose
Elena Ricart: Nothing to disclose
Jordi Rimola Gibert: Nothing to disclose
Carolina Saavedra: Nothing to disclose
Sonia Rodriguez Gomez: Nothing to disclose

**14:00-15:30** **Open Forum Radiographers**

### Research Presentation Session: Radiographers

### RPS 1014

### Optimisation of practice, care and safety: tips and tricks

**Moderators**
T. Stankovic; Zagreb/HR
*(tvstankovic@gmail.com)*
A. J. van der Molen; Leiden/NL
*(molen@lumc.nl)*

### RPS 1014-3

**A phantom image quality study comparing ultra-low and standard dose computed tomography protocols for the investigation of non-accidental injury**

L. Kingston, M. F. McEntee, *N. Moore*, R. Young, C. S. Lee, A. England; Cork/IE
*(niamh.moore@ucc.ie)*

**Purpose:** The purpose of this study was to determine if there is potential for the development and utilisation of a low dose computed tomography (CT) protocol for the diagnosis of suspected non-accidental injury (NAI) and to establish if the suggested ultra-low dose (ULD) CT protocol could deliver image quality (IQ) comparable to that of a standard dose (STD) CT protocol.
**Methods or Backround:** CT images were acquired on a Revolution Apex™ scanner, using a new-born whole body phantom, and reconstructed using deep learning software. Two protocols were compared in terms of IQ, an ULD protocol, with a dose-length product (DLP) of 1.49mGycm2 and a STD protocol, with a DLP of 22.92mGycm2. Participants graded IQ using a modified 4-point Likert scale. Mann-Whitney and chi-squared tests were carried out to determine if there was a significant difference in IQ between the protocols. A t-test was performed to determine if there was a significant difference in participants level confidence in the IQ between the protocols.
**Results or Findings:** 27 participants met the inclusion criteria and scored both image sets. Both the Mann-Whitney and chi-squared test demonstrated significant difference ($P \leq 0.05$) in the IQ between the protocols, for both the evaluation of cortical and trabecular bone. T-test results demonstrated a significant difference ($P \leq 0.05$) in the level of confidence amongst participants between the protocols.
**Conclusion:** This phantom-based study suggests that the IQ of the STD protocol was significantly better when compared to the ULD protocol, for the investigation of suspected NAI. Further work is required to fully optimise an ULD-CT protocol for the investigation of NAI.
**Limitations:** This was a phantom-based study using images without the presence of pathology.
**Ethics committee approval:** This study was approved by the Medical School SREC, University College Cork.
**Funding for this study:** No funding was received for this study.
**Author Disclosures:**
Conor Sean Lee: Nothing to disclose
Mark F. McEntee: Nothing to disclose
Niamh Moore: Nothing to disclose
Lisa Kingston: Nothing to disclose
Rena Young: Nothing to disclose
Andrew England: Board Member: EFRS Advisory Board: RoClub

### RPS 1014-4

**Evaluation of anatomical structures by pulmonary angio-CT in patients with PTE and COVID-19**

*A. F. Colucas*, S. I. Rodrigues, L. P. V. P. V. Ribeiro, A. F. C. L. Abrantes, O. Lesyuk; Faro/PT

**Purpose:** The aim of this study was to identify anatomical changes in the pulmonary angio-CT associated with the clinical worsening of COVID-19 and pulmonary thromboembolism. Results were compared between non-COVID-19 patients and COVID-19 patients, in order to understand whether the presence of this infection interfered with the workflow at the hospital level and whether it had an influence on changes in anatomical structures at the cardiac level.
**Methods or Backround:** This quantitative descriptive-correlational study with a sample of 134 angio-CT exams was conducted in a public hospital, on patients with pulmonary Thromboembolism (PTE), separated into two groups; 81 non-Covid-19 patients and 53 COVID-19 patients. Measurements were taken of the Right Ventricle (RV), Left Ventricle (LV) and Pulmonary Artery Trunk (PAT) to assess whether there was RV dilatation (when RV/LV >1).
**Results or Findings:** Pulmonary angio-CT scans have increased 88% since the COVID-19 pandemic and consequently, an increase in positive diagnoses for PTE. Regarding the results, 85.2% and 82.7% of the non-COVID-19 patients showed dilatation of the RV and dilatation of the PAT respectively. Considering the COVID-19 group, 92.5% and 96,2% of the patients showed dilatation of the RV and PAT, respectively. Considering the Spearman test, the correlation between "COVID-19" and the "RV dilatation" is verified.
**Conclusion:** Comparing the results for both groups, it was found that the group with COVID-19 presented a higher incidence of anatomical changes at the cardiac level, and male gender has been a predictive factor for the presence of both pathologies.
**Limitations:** It is difficult to identify if the non-covid patients had previous infections and a bigger sample is necessary to evaluate the changes in anatomical structures.
**Ethics committee approval:** This study was approved by the ethics committee; decision number 164/2022.
**Funding for this study:** No funding was received for this study.
**Author Disclosures:**
António Fernando Caldeira Lagem Abrantes: Nothing to disclose
Sónia Isabel Rodrigues: Nothing to disclose
Ana Filipa Colucas: Nothing to disclose
Oksana Lesyuk: Nothing to disclose
Luís Pedro Vieira Pedro Vieira Ribeiro: Nothing to disclose

### RPS 1014-5

**Ultra-low-dose CT protocol for left ventricular ejection fraction (LVEF) and combination with chest-abdomen-pelvis CT**

*M. W. Kusk*[1], S. Hess[2], O. Gerke[2], C. Stolzenburg Oxlund[1], L. Deibjerg Kristensen[1], T. E. J. Ormstrup[1], J. M. Christiansen[1], S. J. Foley[3]; [1]Esbjerg/DK, [2]Odense/DK, [3]Dublin/IE
*(martin.weber.kusk@rsyd.dk)*

**Purpose:** The aim of this study was to test the accuracy of ultra-low-dose CT for left ventricular ejection fraction (LVEF-CT). A secondary objective was to examine the feasibility of combination with routine Chest-Abdomen-Pelvis (CAP) CT in oncology patients as potential one-stop replacement for nuclear multigated (MUGA) scans to monitor chemotherapy cardiotoxicity.
**Methods or Backround:** Patients underwent LVEF-CT with cardiac MRI as reference. In oncology patients, MUGA scans were also performed and LVEF-CT was combined with CAP CT, with modified injection protocol. For each modality, two readers measured LVEF. We assessed bias using a Bland-Altman analysis and correlation via Pearson correlation. Interreader agreement was measured using ICC. ROC analysis was performed using MRI 50% LVEF cut-off for reduced LVEF and sensitivity/specificity calculated at maximum Youden Index. We compared CAP-scans to previous scans, using ROI mesasurements, visual grading characteristics and diagnostic acceptability scores.
**Results or Findings:** 77 datasets of 82 patients were suitable. Mean doses were 1.4 mSv for LVEF-CT, 5.7 mSv for MUGA and 7.4 mSv for CAP. CT-derived LVEF bias to MRI varied from 2-10 %, dependent on measurement modes. ROC AUCs exceeded 0.95 and sensitivity/specificity of 100% were achieved with cut-offs from 53-63% LVEF. CT-derived LVEF correlated better with MRI (R=0.87 vs 0.71) and had higher ICC than MUGA (0.98 vs 0.62). CAP image quality showed decreased renal medulla/cortex discrimination, but the number of diagnostic scans was not significantly different from previous scans. Mean examination times were 16, 28, and 32 minutes for CT, MRI and MUGA, respectively.
**Conclusion:** CT LVEF measurement is feasible at a quarter of MUGA dose and can be combined with chest-abdomen-pelvis CT, for a one-stop examination.
**Limitations:** Temporal changes in LVEF were not examined. MUGA subgroup had limited patients with reduced LVEF to measure MUGA classification accuracy.
**Ethics committee approval:** This study was approved by the regional committees on health research ethics for Southern Denmark (approvals. no. S-20210094 and S-20220031).
**Funding for this study:** The study was funded by the Esbjerg Fund (IC Møllers Fond), the research fund of the Danish Radiographers Association (Radiograf Rådet) and the Karola Jørgensen Fund for health research at Esbjerg University Hospital.
**Author Disclosures:**
Martin Weber Kusk: Speaker: Siemens Healthineers, 2022 Danish CT User Meeting
Janus Mølgaard Christiansen: Nothing to disclose
Christina Stolzenburg Oxlund: Nothing to disclose
Lone Deibjerg Kristensen: Nothing to disclose
Søren Hess: Nothing to disclose
Tina E. J. Ormstrup: Nothing to disclose
Oke Gerke: Nothing to disclose
Shane J. Foley: Nothing to disclose

### RPS 1014-6

**Radiomic features of kidney stones with use of virtual non-contrast on photon counting CT: a phantom study**

*S. Lysdahlgaard*[1], L. N. Hansen[1], I. Riebenholt Nielsen[1], B. R. Mussmann[2], J. Jensen[2], H. U. Jung[3], M. W. Kusk[1]; [1]Esbjerg/DK, [2]Odense/DK, [3]Vejle/DK
*(simon.f.l.89@gmail.com)*

**Purpose:** This study aimed to assess the accuracy of kidney stone radiomics and Hounsfield unit (HU) measurements using the Virtual Non-contrast (VNC) technique compared to the traditional True Non-contrast (TNC).
**Methods or Backround:** A Gammex RMI-461a head/body phantom incorporating 18 brushite and 18 uric acid kidney stones. All stones were scanned using low-dose non-contrast computed tomography (NCCT) and standard-dose abdominal protocols. Scans were conducted both without and with stones embedded in contrast media. Images were reconstructed using the VNC technique. Each stone was segmented using 3D slicer and 1.150 radiomics features were extracted. T-tests were used to test for statistically significant differences in mean feature values between stone classifications, with Bonferroni correction applied. A p-value of > 0.05 was considered significant.
**Results or Findings:** Radiomic features from brushite and uric acid for abdomen and NCCT protocols with contrast media yielded 286 and 285 significant values ($p < 0.05$), respectively. Following Bonferroni correction, these numbers were 35 and 31 values ($p < 0.000044$). Radiomic features for both stone types using abdomen and NCCT protocols without contrast media were 446 and 466 significant values ($p < 0.05$), with Bonferroni correction yielding 238 and 224 values ($p < 0.000044$), respectively. Comparative analysis showed that 17 radiomic features were significant in scans in TNC, while 196 features were significant in scans in VNC.
**Conclusion:** This study found fewer statistically significant radiomic features between brushite and uric acid stones in VNC compared to TNC, for both NCCT and abdomen protocols scanned on PCCT. Radiomic features extracted from different stone types further emphasise the potential of this method in enhancing diagnostic precision.
**Limitations:** This study was a phantom study, with only two types of kidney stones, and an operator-dependent segmentation tool.
**Ethics committee approval:** Consent was waived by the ethics committee.
**Funding for this study:** No funding was received for this study.
**Author Disclosures:**
Martin Weber Kusk: Nothing to disclose
Janni Jensen: Nothing to disclose
Simon Lysdahlgaard: Nothing to disclose
Helene Ulrik Jung: Nothing to disclose
Bo Redder Mussmann: Nothing to disclose
Idamarie Riebenholt Nielsen: Nothing to disclose
Louise Norman Hansen: Nothing to disclose

### RPS 1014-7

**An investigation into the impact of radiation dose information on the subjective evaluation of CT image quality**

C. S. Lee, L. Kingston, M. F. McEntee, *R. Young*, N. Moore, M. Maher, A. England; Cork/IE

**Purpose:** The use of medical imaging radiation exposure has dramatically increased in recent years and is continuing to do so. Ultra-low dose examinations may combat this, but switching to ultra-low dose protocols in CT is difficult due to the potential loss of image quality. This has been assessed within numerous studies which have found high interobserver variability. One possible reason for this high variability is the presence of contextual bias. This study aims to investigate the presence and magnitude of bias created through the presence of radiation dose information on images.
**Methods or Backround:** Two CT datasets of images were created using a paediatric phantom. The phantom was scanned using a 'standard' dose protocol and an 'ultra-low' dose protocol. Participant 'radiographers' were asked to score the image quality of each image using the 'PGMI' scoring system. Participants were requested to focus on scoring image quality for cortical and trabecular bone, across the full skeleton. Participants were given the images either with or without the dose information present. Data was analysed statistically.
**Results or Findings:** 53 participants were included. 25 completed the dataset with the dose information present, and 38 completed the dataset without the dose information. Overall, it was found that the dataset with dose information provided received a 23% and 22% higher score for both cortical and trabecular bone image quality, respectively for the standard dose images and 7% and 4% for those acquired using the ultra-low dose protocol.
**Conclusion:** The addition of radiation dose information created contextual bias in both datasets and it had a larger impact on the standard dose CT images.
**Limitations:** This study was based on CT images acquired using a paediatric phantom.
**Ethics committee approval:** This study was approved by the Medical School SREC, University College Cork.
**Funding for this study:** No funding was received for this study.
**Author Disclosures:**
Conor Sean Lee: Nothing to disclose
Mark F. McEntee: Nothing to disclose
Niamh Moore: Nothing to disclose
Lisa Kingston: Nothing to disclose
Rena Young: Nothing to disclose
Andrew England: Advisory Board: RoClub; Board Member: EFRS
Michael Maher: Nothing to disclose

### RPS 1014-8

**Implementation and standardisation of CT optimisation tools across Europe: results from the iViolin project survey**

S. J. Foley, J. McNulty, *N. J. Abualroos*; Dublin/IE
*(nadin.abualroos@ucd.ie)*

**Purpose:** To identify the current availability and implementation of CT optimisation tools during lung, colorectal, and stomach cancer routine scanning protocols across different centres in Europe.
**Methods or Backround:** An online survey consisting of 18 questions was disseminated to six different clinical sites across Europe via iViolin management team in January 2023. The survey was addressed to key imaging representatives (namely a radiologist, radiographer and medical physicist) from each of the six contributing university hospitals. The survey analysed the availability and frequency of use of the available optimisation tools during lung, colorectal, and stomach cancer routine scanning protocols.
**Results or Findings:** Responses were received from all six centres in five European countries with data reported for 22 CT scanners (Siemens: 64%, Philips: 18%, GE: 14%, Canon: 5%). Significant variations in the availability and utilisation of CT optimisation tools in different centres were identified, with automatic exposure control and iterative reconstruction algorithms widely available and in frequent use. The least available tools were additional filtration, high-pitch scanning, and deep-learning reconstruction. Respondents stated that barriers to implementation include subjective local preferences.
**Conclusion:** Results demonstrate varying availability and use of CT optimisation tools. While surveyed centres have access to many optimisation tools, some challenges remain in their full implementation and usage.
**Limitations:** Primary limitation is the limited sample size consisting of just six hospitals from five countries but convenience sampling was used to expedite data collection.
**Ethics committee approval:** As a low risk study which collected data on CT protocols in current use and information was not sensitive application for full ethical review was not necessary
**Funding for this study:** This project is co-funded under the EU4Health Programme 2021–2027 under grant agreement no. 101056832.
**Author Disclosures:**
Nadin Jamal Abualroos: Nothing to disclose
Jonathan McNulty: Nothing to disclose
Shane J. Foley: Nothing to disclose

### RPS 1014-9

**Evaluation of sexual dimorphism of paranasal sinus and sella turcica by computed tomography: a retrospective study of a Portuguese population**

*R. R. Gaspar*[1], M. Lopes[2], T. Ribeiro[2], B. M. Magalhães[1], P. M. Martins[2]; [1]Coimbra/PT, [2]Aveiro/PT
*(rosa.cristina.ramos@gmail.com)*

**Purpose:** A potential for sexual dimorphism has been pointed out regarding the study of paranasal sinuses (PS) and sella turcica (ST) in a forensic context, but results are conflicting in the literature. The aim of this study is to ascertain if PS: maxillary sinus (MS) sphenoidal sinus (SS) and ST measurements present sexual dimorphism in a Portuguese population.
**Methods or Backround:** This was a retrospective, observational study including 53 CT scans of the PS from Portuguese subjects (31 males/22 females. Exclusion criteria were age below 18 years, trauma, tumours, previous surgery, and sinus dysplasia. Measurements of length (AP), width (WI), height (HE), distance between MS (BIL), and volume of MS were obtained bilaterally; for the SS AP, WI, and HE were also obtained. ST measures included AP diameter, depth, and length. All linear measurements were done with RadiAnt DICOM Viewer. The volume of the MS was estimated from linear measurements. Data were analysed using SPSS-24. Normality and homogeneity were assessed. T-student and Mann-Whitney tests were applied. Statistical significance was set at .05.
**Results or Findings:** Males present mean values higher than females except for measures of the sella turcica, in all instances. Statistically significant differences between sexes were found for five of the measurements: left WI ($p=.042$), left HE ($p=.004$), left volume ($p=.033$) of the MS, HE of the SS ($p=.011$), and ST AP Diameter ($p=.016$). A discriminant equation involving the measures with normal distribution was done. The height of the left MS shows an accuracy of 66% in predicting sex.
**Conclusion:** The present study reveals that, at least, the height of the maxillary sinus presents sexual dimorphism. The methods hold promise in the field of forensic sciences to characterise the Portuguese population.

**Limitations:** A larger sample size should be considered.
**Ethics committee approval:** In this retrospective study, the data under analysis were subject to irreversible anonymisation and stored by the investigator in an encrypted digital format during this research.
**Funding for this study:** No funding was received for this study.
**Author Disclosures:**
Bruno M. Magalhães: Nothing to disclose
Telma Ribeiro: Nothing to disclose
Mário Lopes: Nothing to disclose
Paula Maria Martins: Nothing to disclose
Rosa Ramos Gaspar: Nothing to disclose

### RPS 1014-10

**The application of low-dose chest CT for the diagnosis and monitoring of pulmonary infections in neutropenic patients**

*E. M. Agadakos*, A. Zormpala, N. Zaios, C. Kapsiocha, M. Voulgarelis, N. Sypsas, L. A. Moulopoulos, V. Koutoulidis; Athens/GR
*(eagadakos@gmail.com)*

**Purpose:** The aim of our study was to investigate the image quality and the diagnostic performance of low-dose Chest CT (LDCCT) for the diagnosis and monitoring of pulmonary infections in neutropenic patients.
**Methods or Backround:** Data from 164 consecutive neutropenic patients of a total of 256 CT examinations [149 LDCCT and 107 Standard Dose Chest CT (SDCCT)] were obtained between May 2015 and June 2019 . Examiners analysed the image quality and evaluated the radiologic findings associated with pulmonary infections to determine differences in diagnostic performance between the two imaging protocols.
**Results or Findings:** The LDCCT protocol reduced radiation dose to patients by 47%. LDCCT images compared well in terms of image noise and image quality to SDCCT images. Detection of consolidation and ground glass opacity was significantly lower with the LDCCT protocol (LDCCT: 27.5% and 64.4%, respectively, SDCCT: 45.8% and 82.2%, respectively) with all the respective p-values, unadjusted and adjusted for sex, age and BMI analyses as well as the corresponding odds ratios. Similarly, LDCCT diagnostic sensitivity rated lower for nodules ≥3mm and ground glass halo in nodule(s) but was not affected by sex, age and BMI. There were no statistically significant differences in diagnostic performance between the two protocols for the detection of cavitation in nodules, diffuse interlobular septal thickening, pleural effusion, pericardial effusion and lymphadenopathy.
**Conclusion:** LDCCT achieved a remarkable dose reduction, image quality and noise levels rated well yet underestimated four important radiologic findings associated with pulmonary infections in neutropenic patients.
**Limitations:** The limitations encountered during the current study were the lack of follow-up of patient progress and the effect on the prognosis and survival rate. Another limitation was that both data sets were obtained using only a single IR algorithm strength setting.
**Ethics committee approval:** The study was compliant with the General Data Protection Regulation (679/2016 EE) and was approved by the hospital's scientific council and ethics committee.
**Funding for this study:** This study did not receive any specific grant from funding agencies in the public, commercial, or not-for-profit sectors.
**Author Disclosures:**
Efthimios M. Agadakos: Nothing to disclose
Vassilis Koutoulidis: Nothing to disclose
Michael Voulgarelis: Nothing to disclose
Nikolaos Zaios: Nothing to disclose
Chrysoula Kapsiocha: Nothing to disclose
Nikolaos Sypsas: Nothing to disclose
Lia Angela Moulopoulos: Nothing to disclose
Alexandra Zormpala: Nothing to disclose

### RPS 1014-11

**What radiographers need to know to identify optimised protocols for pulmonary embolism studies using computed tomography**

*L. Kozma*, L. Pittet, M. Champendal, L. Flaction, C. S. D. Reis; Lausanne/CH
*(lucas.kozma@gmail.com)*

**Purpose:** CTPA is the preferred method for assessing pulmonary embolism, but protocol variations result in huge radiation doses variations. This study synthesised optimised CTPA protocols to improve radiation protection, contrast media use, and image quality identified in the literature.
**Methods or Backround:** Optimised CTPA protocols for adult patients were used, including special groups (pregnant women; high-BMI individuals), with a focus on improving radiation protection; use of iodinated contrast agents; image quality, exam success rate were identified following JBI methods. "Embase" and "PubMed" databases were searched. Two independent reviewers performed a blinded assessment. Articles published after 2018, with over 100 participants, meeting the above criteria were included. Data was extracted and analysed, with categorised subsets and descriptive statistics being applied.
**Results or Findings:** Twenty-eight articles, involving 100 to 3998 participants, revealed various optimisation techniques falling into five categories: acquisition, injection, breathing, reconstruction parameters, adaptations for specific populations. Reducing scan length with a 37-48% dose reduction, high-pitch scans, dose modulation (auto-mA and ODOM), lower kV (low-kV), auto-kVp, and dual-energy methods were proposed to lower dose and enhance patient outcomes. For pregnant women, 100 kV beams were recommended, with free breathing and an increased injection rate.
**Conclusion:** Optimising CTPA protocols is a complex task due to the amount of parameters that is necessary to consider. It requires focused studies on specific protocol variables to gather reliable and applicable data. Regular protocol reviews are crucial for effective optimisation, to include all technical updates. Radiographers are central to the optimisation process combining their technical expertise with patient-centred care expertise.
**Limitations:** No experimental studies were conducted.
**Ethics committee approval:** No information was provided by the submitters.
**Funding for this study:** No funding was received for this study.
**Author Disclosures:**
Claudia Sa Dos Reis: Nothing to disclose
Laurence Flaction: Nothing to disclose
Léo Pittet: Nothing to disclose
Lucas Kozma: Nothing to disclose
Mélanie Champendal: Nothing to disclose

### RPS 1014-12

**Enhancing radiation safety for nuclear medicine radiographers: the impact of introducing the automated radiopharmaceutical preparation and administration system**

*S. Pichierri*, G. R. Bonfitto, A. Roletto, L. Bombelli, E. Scaramelli, S. V. Fasulo, A. Savi, M. Olivieri, D. Catania; Milan/IT
*(pichierri.serena@gmail.com)*

**Purpose:** In Nuclear Medicine (NM), radiopharmaceutical doses have traditionally been administered in unit doses, divided and administered manually by personnel. The recent shift in many hospitals towards automated preparation and injection systems aims to minimize staff exposure to radiation. However, studies on their efficacy and potential benefits to radiographers are still limited. The aim of this preliminary study is to investigate the radiation exposure of NM staff involved in automated preparation and injection of (18)F-fluorodeoxyglucose (FDG), compared to manual administration.
**Methods or Backround:** From April to July 2023, NM radiographers' radiation exposure was recorded with electronic dosimeters during (18)F-FDG administration with the automated preparation and injection system and compared with the exposure during manual preparation and administration.
**Results or Findings:** By analysing the radiation exposure recorded with the electronic dosimeter provided to the radiographers, it was found that the individual whole-body radiation exposure for radiographers for each patient averaged 4,03 µSv ± 1,2 during the use of the automated preparation and injection system and it averaged 6,93 µSv ± 1,5 during manual preparation and administration. Whole-body exposure was significantly reduced with automated administration by 41,85% compared to the value associated with manual administration ($p < 0,05$).
**Conclusion:** Based on preliminary results collected in our hospital, the use of an automated preparation and injection system could greatly reduce the radiation exposure of NM radiographers involved in the administration of (18)F-FDG. The results of this study will be used by our team to increase the use of the automated radiopharmaceutical preparation and administration system and more deeply evaluate its potential benefits in terms of reduction of radiation exposure on the whole NM staff.
**Limitations:** No limitations were identified.
**Ethics committee approval:** No information provided by the submitter.
**Funding for this study:** No funding was received for this study.
**Author Disclosures:**
Luca Bombelli: Nothing to disclose
Simone Vito Fasulo: Nothing to disclose
Diego Catania: Nothing to disclose
Andrea Roletto: Nothing to disclose
Elena Scaramelli: Nothing to disclose
Serena Pichierri: Nothing to disclose
Annarita Savi: Nothing to disclose
Michela Olivieri: Nothing to disclose
Giuseppe Roberto Bonfitto: Nothing to disclose

### RPS 1014-13

**An investigation into radiographers' ability to identify developmental dysplasia of the hip (DDH) on radiographs and factors related to observer performance**

N. G. Carr[1], A. England[1], J. D. Thompson[2], N. Moore[1], R. Young[1], *M. F. McEntee*[1]; [1]Cork/IE, [2]Barrow-in-Furness/UK
*(mark.mcentee@ucc.ie)*

**Purpose:** DDH refers to hip joint dislocation or predisposition in young children. Early detection and management are essential for effective treatment and the prevention of long-term effects. The aim of this research was to look into radiographers' abilities to detect DDH on x-ray images and identify factors that influence their performance.
**Methods or Backround:** At the European Congress of Radiology, radiographers were given a structured questionnaire which included a test bank of images. There were 25 paediatric cases in the questionnaire, and participants were asked to diagnose DDH and rate the image quality. Data were analysed to determine the sensitivity, specificity, accuracy, and area under the receiver operating characteristic (ROC) curve.
**Results or Findings:** The image set was reviewed by 30 radiographers. The average sensitivity for diagnosing DDH was 56.2%, with a specificity of 40.2%. With an area under the curve of 0.6522, the ROC curve study demonstrated slightly better performance than chance. Collimation, lower limb position, and the absence of patient rotation were identified as key factors influencing participants' diagnostic ability.
**Conclusion:** The study discovered that radiographers' ability to recognise developmental dysplasia of the hip (DDH) on radiographs varies and is impacted by characteristics such as training, experience, and technological consideration. Additional training and standard approaches are required to increase their diagnostic performance in DDH. DDH imaging training programmes for radiographers can improve their skills and may lead to better patient care.
**Limitations:** Given the nature of the study, participants would have expected a prevalence of DDH within the image bank.
**Ethics committee approval:** This study was approved by the Medical School SREC, University College Cork.
**Funding for this study:** No funding was received for this study.
**Author Disclosures:**
Mark F. McEntee: Nothing to disclose
Niamh Moore: Nothing to disclose
Niall Gerard Carr: Nothing to disclose
Rena Young: Nothing to disclose
Andrew England: Advisory Board: RoClub; Board Member: EFRS
John David Thompson: Nothing to disclose

### RPS 1014-14

**The impact and effect of imaging referral guidelines on patients and radiology services: a systematic review**

*Y. X. Tay*[1], S. J. Foley[2], R. P. Killeen[2], M. E. Ong[1], J. McNulty[2]; [1]Singapore/SG, [2]Dublin/IE
*(tayyixiang87@gmail.com)*

**Purpose:** The objective of this systematic review was to offer a comprehensive overview and explore the associated outcomes from imaging referral guidelines for various key stakeholders, such as patients and radiologists.
**Methods or Backround:** An electronic database search was conducted in Medline, Embase and Web of Science to retrieve citations published between 2013 and 2023. The search was constructed using medical subject headings and keywords. Full-text articles and reviews written in English were included. The quality of the included papers was assessed using the mixed methods appraisal tool. A narrative synthesis was undertaken for the articles selected.
**Results or Findings:** The search yielded 4,384 records. Following abstract, full-text screening, and removal of duplication, 31 studies of varying levels of quality were included in the final analysis. Before-and-after studies, retrospective data analysis, randomised controlled trials, interrupted time series, and cohort studies were included. Imaging referral guidelines from the American College of Radiology were most commonly used. Clinical decision support systems were the most evaluated mode of intervention, either integrated or standalone. Interventions showed reduced patient radiation doses and waiting times for imaging. There was a general reduction in radiology workload and utilisation of diagnostic imaging. Low-value imaging utilisation decreased with an increase in the appropriateness of imaging referrals and ratings and cost savings. Clinical effectiveness was maintained during the intervention period without notable adverse consequences.
**Conclusion:** The use of evidence-based imaging referral guidelines is important to improve the quality of healthcare and outcomes while reducing healthcare costs. Imaging referral guidelines could be an essential component of improving the value of radiology in the healthcare system.
**Limitations:** Studies included in this analysis reflect a significant degree of heterogeneity in terms of their research design, study population, interventions utilised, and outcomes evaluated.
**Ethics committee approval:** Ethical review was not required as this study constitutes a systematic review of the available literature.
**Funding for this study:** Funding was received from the SingHealth Talent Development Fund and via the Singapore General Hospital Scholarship.
**Author Disclosures:**
Yi Xiang Tay: Nothing to disclose
Jonathan McNulty: Nothing to disclose
Marcus E. Ong: Nothing to disclose
Ronan P. Killeen: Nothing to disclose
Shane J. Foley: Nothing to disclose

**14:00-15:30** **Research Stage 1**

### Research Presentation Session: Artificial Intelligence & Machine Learning & Imaging Informatics

### RPS 1005

### Harnessing AI for enhanced reporting and workflow

**Moderator**
E. Kotter; Freiburg/DE
*(elmar.kotter@uniklinik-freiburg.de)*

### RPS 1005-2

**Does patient education level impact comprehension of radiology reports: can AI level the playing field?**

*M. B. Aziz*[1], R. Husam Al-Deen[2], M. H. Chowdhury[3], M. I. K. Inayat[2], H. Ahmed[2], B. Syed[2], H. M. Khan[2], A. Pervez[2], S. Syed[2]; [1]Blackburn/UK, [2]London/UK, [3]Basildon/UK
*(mohammed.aziz@elht.nhs.uk)*

**Purpose:** This study investigates the impact of artificial intelligence in improving the comprehension of radiology reports for readers based on their educational background. Radiology reports serve as vital communication between healthcare professionals and patients, yet their comprehensibility can vary based on the patients' educational attainment.
**Methods or Backround:** 40 musculoskeletal MRI reports, 20 each written by consultant radiologists and generative AI (ChatGPT), were evaluated by 10 participants across multiple educational backgrounds, namely secondary education, university student, university graduates, medical students and qualified doctors. AI-generated reports were produced from radiologist-authored reports with a standardised prompt for comprehension for the layman without removing detail. Comprehension was evaluated using a standardised metric in a Likert scale, and a comparison between the reports for comprehension by educational stratification was performed.
**Results or Findings:** Our study showed, for radiologist-authored reports, a combined understandability and readability average of 2.38 out of 5 (higher values indicate better comprehension) for secondary school participants, 3.13 for non-medical university students, 2.79 for university graduates, 2.26 for medical students, and 3.00 for doctors: as the level of medically-related education increases, individuals achieved a higher mean for comprehension. When compared to AI-generated reports, the mean scores for comprehension were 4.225 for secondary school, 4.55 for non-medical university students, 3.58 for university graduates, 4.40 for medical students, and 3.89 for doctors, demonstrating no pattern between the mean scores of individuals of varying educational levels – a level playing field.
**Conclusion:** AI-generated reports demonstrated better comprehension among recipients across the educational spectrum, highlighting the potential of AI to remove inaccessibility to conversations surrounding a patient's health and allow patients to make informed medical decisions.
**Limitations:** The limitation of the study is the sample size consisted of 10 individuals, requiring further research into the applicability of AI in enhancing patient access to radiology reports.
**Ethics committee approval:** Not considered research as per MRC decision tool.
**Funding for this study:** No funding was received for this study.

**Author Disclosures:**
Bari Syed: Nothing to disclose
Rawaha Husam Al-Deen: Nothing to disclose
Hafsa Masroor Khan: Nothing to disclose
Mohammed Bilal Aziz: Nothing to disclose
Hassan Ahmed: Nothing to disclose
Anum Pervez: Nothing to disclose
Mohammed Ibrahim Kashif Inayat: Nothing to disclose
Mohammed Himaayat Chowdhury: Nothing to disclose
Sabeeh Syed: Nothing to disclose

### RPS 1005-3

**ChatGPT-4 makes cardiac MRI reports easy to understand: a feasibility study**

*B. Salam*, D. Kravchenko, L. Weinhold, S. Nowak, A. M. Sprinkart, U. I. Attenberger, D. Kütting, J. A. Luetkens, A. Isaak; Bonn/DE

**Purpose:** This study aimed to evaluate the ability of Chatbot Generative Pre-trained Transformer 4 (ChatGPT-4) to transform cardiac MRI reports into comprehensible text for medical laypersons.
**Methods or Backround:** ChatGPT-4 was used to generate three simplified versions of 20 various cardiac MRI reports using the same prompt (n=60). Two cardiovascular radiologists evaluated factual correctness, completeness of relevant findings, and serious misinformation with potential harm (total ratings, n=360), while medical laypersons evaluated the understandability of both versions (total ratings, n=200 and n=600, respectively) on a Likert scale (1 “strongly disagree”, 5 “strongly agree”). Readability grade level of reports were measured using the Automated Readability Index. Mann-Whitney U test and Intraclass Correlation Coefficient (ICC) were performed.
**Results or Findings:** ChatGPT-4 reports were generated on average in 52 sec (8–78 sec). The median reading grade levels of the ChatGPT-4 versions were significantly lower (10 [9-12] vs 5 [4-6]; p<.001) and easier to understand for laypersons than original reports (1 [1-1] vs 4 [4-5]; p<.001). Radiologists’ ratings of the ChatGPT-4 versions reached a median of 5 (5-5) for all three categories with “strong agreement” for factual correctness in 92% and completeness of relevant findings in 84% of the reports. Test-retest agreement for layperson understandability between the three simplified reports generated from the same original report was moderate (ICC: 0.54; p<.001). Interrater agreement between radiologists was high (ICC: 0.92; p<.001).
**Conclusion:** ChatGPT-4 can transform complex cardiac MRI reports into more understandable, layperson-friendly language without compromising factual correctness or completeness. That can help convey patient-relevant radiology information in an easy-to-understand manner.
**Limitations:** Exploratory study design. Relatively small sample size of medical laypersons. During the questionnaire completion process, participants may have experienced a learning effect as they read through the simplified reports, potentially influencing their subsequent assessment of understandability.
**Ethics committee approval:** Because of the use of fictitious, unidentifiable data, approval of an institutional review board was not required.
**Funding for this study:** No funding was received for this study.
**Author Disclosures:**
Julian Alexander Luetkens: Nothing to disclose
Alexander Isaak: Nothing to disclose
Alois M. Sprinkart: Nothing to disclose
Sebastian Nowak: Nothing to disclose
Leonie Weinhold: Nothing to disclose
Dmitrij Kravchenko: Nothing to disclose
Ulrike I. Attenberger: Nothing to disclose
Daniel Kütting: Nothing to disclose
Babak Salam: Nothing to disclose

### RPS 1005-4

**Cost-consequence analysis of artificial intelligence-assisted image reading in lung cancer screening**

*H. L. Lancaster*[1], K. Togka[1], X. Pan[1], M. Silva[2], D. Han[1], M. Oudkerk[1]; [1]Groningen/NL, [2]Parma/IT
*(h.l.lancaster@umcg.nl)*

**Purpose:** This modelling study aimed to estimate clinical and costs-consequences of a hypothetical AI-assisted image reading solution in LCS in the Netherlands, compared to image reading without AI. Lung cancer screening (LCS) with LDCT detects lung cancer earlier and leads to a reduction in lung cancer mortality by 20-24% (as shown in the NLST and NELSON RCTs). However, implementing LCS may exacerbate the workload of radiologists. Artificial intelligence(AI) exhibits promising outcomes in the detection, segmentation, and classification of lung nodules for LCS. Despite encouraging findings, AI assisted image reading is rarely used within clinical practice.
**Methods or Backround:** A cost-consequence analysis was conducted, capturing costs and effects of different LCS scenarios at baseline from a healthcare perspective. Essential model inputs included; eligible population, screening population, image reading time by radiologists, average weighted time, image reading time by AI, costs, screening effectiveness without AI, and discrepancies in image reading. Control scenario: LCS without AI-assisted image reading. Two radiologists independently read all CT scans. Scenario A: LCS with AI as a parallel-reader. AI read all CT-scans in parallel with a radiologist and the discrepant results assessed by a consensus radiologist. Scenario B: LCS with AI as a first-reader. AI read all CT-scans first, then a radiologist confirmed positive scans and identified false-positive classifications.
**Results or Findings:** LCS with AI-assisted image reading has the potential to reduce image reading costs by 37% and 73%, in Scenario A and B respectively (total reading costs [Control: €29,676,879; Scenario A: €18,704,843; Scenario B: €8,146,251]). Additionally, utilising AI as the first reader may reduce the radiologists’ workload.
**Conclusion:** The incorporation of AI-assisted image reading into LCS yields substantial reductions in costs associated with image reading. Our findings support AI utilisation in LCS to alleviate constraints on healthcare resources.
**Limitations:** No limitations were identified.
**Ethics committee approval:** Performed using published data and expert opinions.
**Funding for this study:** No funding was received for this study.
**Author Disclosures:**
Matthijs Oudkerk: Nothing to disclose
Harriet Louise Lancaster: Nothing to disclose
Mario Silva: Nothing to disclose
Katerina Togka: Nothing to disclose
Daiwei Han: Nothing to disclose
Xuanqi Pan: Nothing to disclose

### RPS 1005-5

**Deep learning assisted curation of the CANDID-III dataset with free-text reports**

*S. Feng*[1], Q. Liu[2], D. Ritchie[3], B. K. J. Wilson[3]; [1]Mosgiel/NZ, [2]Auckland/NZ, [3]Dunedin/NZ
*(sijingfeng@gmail.com)*

**Purpose:** This studay aimed to curate the CANDID-III dataset, which consists of adult chest radiographs with comprehensive labels derived from both manual and AI-assisted annotation.
**Methods or Backround:** The CANDID-II dataset is an in-development chest radiograph dataset containing 33,486 anonymised free-text radiological reports. CANDID-III inherited the same 45 radiological labels from the CANDID-II dataset, which were mapped to UMLS ontology for standardisation, forming the manually labelled portion of the CANDID-III dataset. An ensemble transformer-based label extraction model was trained and validated on the CANDID-II dataset in an 80:20 proportion. The model was then used to automatically label the remaining CANDID-III dataset. An evaluation set of 552 reports was assessed by selected annotation team members. Label-specific ‘mention’ F1 scores were calculated for the final ensemble model, with ‘not mentioned’ as negative and ‘indeterminate, absent, present’ as combined positive classifications.
**Results or Findings:** The completed CANDID-III dataset contains 322,473 images and 220,977 anonymised free-text radiological reports from 94,210 unique patients (1:1.04 M:F ratio). AI-assisted annotation was performed on 88% of the CANDID-III dataset. For the AI-assisted annotation portion of the CANDID-III dataset, the labelling model has a macro-F1 score of 0.88 and micro-F1 score of 0.94 across all findings. Seven labels are shared with CheXpert, with F1 scores ranging from 0.93 to 1.0. F1 scores for 30 CANDID-III labels are above 0.90, while 8 labels range between 0.80 and 0.90.
**Conclusion:** The CANDID-III dataset adds numerous new clinically significant radiological annotations that are labelled to a high accuracy. It contributes to the repertoire of publicly available chest radiograph datasets for AI development. Instructions to access the dataset can be accessed at DOI: 10.17608/k6.auckland.22726004.
**Limitations:** Single institution dataset, radiologists' opinion is used as label ground truth rather than objective quantitative measures.
**Ethics committee approval:** The study was approved by the University of Otago Human Ethics Committee.
**Funding for this study:** No funding was received for this study.
**Author Disclosures:**
Ben Kenneth Joseph Wilson: Author: nothing to disclose
Qixiu Liu: Author: nothing to disclose
Darren Ritchie: Author: nothing to disclose
Sijing Feng: Author: nothing to disclose

### RPS 1005-6

**Sixteen thousand and counting: performance of an artificial intelligence tool for identifying common pathologies on chest radiographs and report prioritisation**

*C. Horst*[1], N. Ruechuseth[1], C. Allwin[1], V. Naidu[1], Y. Zhu[2], R. O'Shea[1], C. Goncalves[1], M. Narbone[1], V. Goh[1]; [1]London/UK, [2]Warwick/UK
*(carolyn.horst@gmail.com)*

**Purpose:** Chest radiograph (CXR) artificial intelligence (AI) tools may streamline reporting times and improve patient outcomes through decision-support functionalities. However, clinical uptake has been limited and a better understanding of their accuracy at different probability thresholds for different use cases is required. The objective of this study is to better understand the accuracy at different probability thresholds for different AI use cases. Chest radiograph (CXR) artificial intelligence (AI) tools may streamline reporting times and improve patient outcomes through decision-support functionalities, however, clinical uptake has been limited.
**Methods or Backround:** 16996 CXRs were retrospectively scored from 0-100 probability by an AI tool for 8 common pathologies. Corresponding historical free text reports were processed by a natural language processing (NLP) model to establish ground truth. Sensitivities and specificities for the eight findings were calculated for four positive AI score thresholds (5, 15, 30 and 45). A composite label of 'normal' was created, where none of the individual labels were above the given probability threshold, and sensitivities and specificities calculated.
**Results or Findings:** Per-finding sensitivities ranged from 0.46-0.94 and specificities from 0.53-0.99, depending on pathology and positive threshold. For ‘normal’ CXRs, sensitivities ranged from 0.51-0.82, and specificities from 0.82-0.95.
**Conclusion:** Our analysis demonstrates the importance of acceptable thresholds for ‘positive’ findings for different pathologies. A very high sensitivity may be appropriate for emergency findings e.g. pneumothorax, with a sacrifice in the specificity. Conversely, a high specificity is preferable to triage low risk studies for reporting without missing actionable pathology. Sensitivities and specificities at the approved threshold of 15 provide a balance of sensitivity and specificity. Our study demonstrates a flexible approach to using AI for CXR analysis of common abnormalities, and the possibility of using the tool for identifying 'normal' radiographs for triaging purposes.
**Limitations:** There may be inaccuracies in the NLP outputs that have not been controlled for, and the AI definitions of certain pathologies may not align with radiologists’ local reporting practices. This effect was partially mitigated by the large number of analysed cases.
**Ethics committee approval:** Retrospective study.
**Funding for this study:** No funding was provided for this study.
**Author Disclosures:**
Mariapaola Narbone: Nothing to disclose
Nateewat Ruechuseth: Nothing to disclose
Vicky Goh: Nothing to disclose
Yuanyi Zhu: Nothing to disclose
Carolyn Horst: Nothing to disclose
Caitlin Allwin: Nothing to disclose
Robert O'Shea: Nothing to disclose
Carla Goncalves: Nothing to disclose
Vishvan Naidu: Nothing to disclose

### RPS 1005-7

**Glioblastoma patient monitoring using a large language model: accurate and effective summarisation of radiological reports with GPT-4**

*R. A. Terzis*, K. R. Laukamp, J-M. Werner, N. Galldiks, S. Lennartz, D. Maintz, M. Schlamann, M. Schoenfeld, J. Kottlors; Cologne/DE

**Purpose:** The purpose of this study was to evaluate this possibility, particularly focusing on the capacity of LLMs to extract meaningful information from complex textual input. Monitoring of glioblastoma patients involves multiple MRI scans, making the process complex and resource-heavy. The advent of large language models (LLMs) presents an opportunity to facilitate physician support by summarising radiological results and disease tracking data.
**Methods or Backround:** We retrospectively included 225 examinations from 45 patients with biopsy-confirmed glioblastoma who were treated at our institution. The large language model, GPT-4, was supplied with the five most recent MRI reports, including clinical information in text form. The model's task was to synthesize the disease course, present the current state, and produce the R-code for a suitable graphic representation. Summaries generated by GPT-4 were evaluated by two expert neuro-oncologists experts with >20 and >8 years of experience, respectively. The evaluation categories included: (1) accuracy and logical-semantic representation, determined by assessing four distinct items on a binomial scale of "yes" or "no"; (2) overall quality; and (3) utility in patient monitoring and therapeutic decision-making, assessed using a 5-point Likert scale, with higher scores indicating more favourable results.
**Results or Findings:** The summaries from GPT-4 matched the expert consensus on the disease progression 86.7% of the time. GPT-4's disease course summaries received a median score of 4 in terms of quality and were perceived to have a median utility score of 3.
**Conclusion:** GPT-4 effectively outlined the disease progression with significant precision, value, and relevance for clinicians. Our results underline the potential of large language models for radiological and medical workflow optimisation.
**Limitations:** Limitations are reliance on text-only data, the GPT-4 model's knowledge cutoff in 2021, the "black-box" problem and a single-center linguistic focus.
**Ethics committee approval:** This study received ethical approval, and informed consent was waived due to the retrospective design. No patient-identifying information was provided to the artificial intelligence.
**Funding for this study:** This study was funded in part by the German Federal Ministry of Education and Research Network of University Medicine 2.0 (Grant no. 01KX2121).
**Author Disclosures:**
Simon Lennartz: Nothing to disclose
Kai Roman Laukamp: Nothing to disclose
David Maintz: Nothing to disclose
Jonathan Kottlors: Nothing to disclose
Jan-Michael Werner: Nothing to disclose
Michael Schoenfeld: Nothing to disclose
Norbert Galldiks: Nothing to disclose
Robert Angelo Terzis: Nothing to disclose
Marc Schlamann: Nothing to disclose

### RPS 1005-8

**Initial experience of a fully digital workflow for radiological evaluation in clinical trials**

*M. Scott*[1], J. Burwick Nyberg[1], T. Sundin[1], M. Gelotte[1], P. Eckerbom[1], J. Wikström[1], P. Liss[1], T. Bjerner[2]; [1]Uppsala/SE, [2]Linköping/SE
*(martin.scott@regionuppsala.se)*

**Purpose:** The aim of this project was to use a research PACS and set up a fully digital workflow for radiologic evaluation in clinical trials. Radiological evaluation of tumour response during oncologic treatment is an important task for many radiology departments. Reporting of such evaluation has previously been and is still often documented on paper and not digitally in the PACS (Picture Archiving and Communication System).
**Methods or Backround:** This project was carried out at the Department of Radiology, Uppsala University Hospital. Studies to be radiologically evaluated in a research trial were pseudonymised using RSNA CTP and exported from the clinical Philips Vue PACS to an external server, the research PACS, consisting of another Philips Vue PACS with some adaptations to its configuration. A notification of a new study to evaluate was then sent by email to the reading radiologist by the CTP. A structured report template was set up in the research PACS where findings were marked and hyperlinks were included. The report can then be read, and the hyperlinks used by the oncologist in the Philips VueMotion web-interface of the research PACS. The report also includes graphs visualising lesion development over time.
**Results or Findings:** Since the start of the digital workflow in September 2023 it has rapidly been adopted by the research nurses and the radiologists involved and the workflow is now considered more efficient and consistent. For the oncologist involved in the study it is a great advantage to be able to easily see the measurements and the graphs in the web viewer. Time is saved by reduced paper handling.
**Conclusion:** A digital workflow significantly improves the handling of oncology studies that includes radiological evaluation of tumour response to treatment.
**Limitations:** No limitations were identified.
**Ethics committee approval:** This is not a clinical study.
**Funding for this study:** No funding was received for this study.
**Author Disclosures:**
Johan Burwick Nyberg: Nothing to disclose
Tomas Bjerner: Advisory Board: Tomas Bjerner was Medical Advisor for Carestream Health until Carestream Health was acquired by Philips 2019
Per Liss: Nothing to disclose
Johan Wikström: Nothing to disclose
Monika Gelotte: Nothing to disclose
Tora Sundin: Nothing to disclose
Per Eckerbom: Nothing to disclose
Martin Scott: Nothing to disclose

### RPS 1005-9

**Reshaping CT imaging workflow through intelligent AI orchestration using RAPID: radiologic automated processing for image distribution**

*R. Hosch*, V. Parmar, J. Kohnke, K. A. Borys, K. Arzideh, G. Baldini, J. Haubold, L. Umutlu, F. Nensa; Essen/DE

**Purpose:** The purpose of this study was to introduce RAPID, an algorithm for swiftly and automatically orchestrating images based on detected anatomical landmarks and body regions.In the rapidly evolving medical AI field, radiologists are incorporating AI models into clinical practice, aiming for enhanced efficiency and workflow optimisation. This necessitates the implementation of an "Orchestrator" capable of automatically directing images to appropriate AI models without manual intervention. Existing CT solutions predominantly rely on DICOM tags, which offer limited and often unreliable information like SeriesDescription.
**Methods or Backround:** 13,211 abdominal and 6,789 whole-body CT scans from 20,000 patients (42.75% female) were used. Topograms from these scans were employed for three tasks: classification (torso, head-neck, hands, legs), region detection (head, brain, pericardium, thorax, abdomen), and organ detection (lung, heart, spine, liver, kidneys, spleen, stomach, colon, pancreas, brain, hip). Series specific organ and body region segmentations generated using the Body and Organ Analysis algorithm (BOA) were mapped onto topograms using DICOM geometry as ground truth. YOLOv8 models were trained for classification and object detection and evaluated using F1-score and mean Average Precision (mAP0.5).
**Results or Findings:** Classification achieved a weighted F1-score of 0.92. Region detection reached 0.96 mAP, while organ detection scored 0.94 mAP. After topogram-based robust classification and detection, orchestration rules were established to automatically route series to suitable AI models if they met the model's prerequisites.
**Conclusion:** RAPID accurately and efficiently locates body regions and organs in CT scans using topograms. These landmarks facilitate series orchestration for AI applications. RAPID employs "deep content inspection" for precise routing decisions, prioritizing image data over manual entered meta data.
**Limitations:** The trained models should be evaluated on external datasets. In addition, the number of relevant landmarks for object detection should be extended.
**Ethics committee approval:** This study adhered to all guidelines defined by the approving institutional review board of the investigating hospital. The Institutional Review Board waived written informed consent due to the study's retrospective nature. Complete anonymisation of all data was performed before inclusion in the study.
**Funding for this study:** No funding was received for this study.
**Author Disclosures:**
Giulia Baldini: Nothing to disclose
Katarzyna Anna Borys: Nothing to disclose
Johannes Haubold: Nothing to disclose
Judith Kohnke: Nothing to disclose
Vicky Parmar: Nothing to disclose
Lale Umutlu: Nothing to disclose
Kamyar Arzideh: Nothing to disclose
René Hosch: Nothing to disclose
Felix Nensa: Nothing to disclose

### RPS 1005-10

**A novel radiology communication tool to reduce workflow interruptions: clinical evaluation of RadConnect**

M. Sevenster[1], K. Hergaarden[2], O. Hertgers[2], N. Kruithof[2], J. Roelofs[2], S. Romeijn[2], D. D. Nguyen[1], *S. Vosbergen*[1], H. J. Lamb[2]; [1]Amsterdam/NL, [2]Leiden/NL
*(sandra.vosbergen@philips.com)*

**Purpose:** The objective of this study was to test the hypothesis that a novel asynchronous communication tool (RadConnect) reduces radiologist workflow interruptions. Effective stakeholder communication across the imaging value chain is a crucial responsibility of radiologists. However, typically communication tools are used that were not created specifically for the unique needs of imaging. This contributes to frequent radiologist interruptions.
**Methods or Backround:** We conducted a difference-in-difference before-after study. Before adoption of RadConnect, technologists used three conventional communication methods to consult radiologists (in-person, telephone, general-purpose enterprise chat [GPEC]). After adoption, participants used RadConnect as a fourth. Technologists manually recorded every radiologist consult request related to neuro and thorax CT scans in the 40 days before and 40 days after adoption of RadConnect. Telephone traffic volume to section beepers was obtained from the hospital telephone system. The abdomen beeper was included as control group. The value and usability experiences were collected through an electronic survey and structured interviews.
**Results or Findings:** Adoption of RadConnect resulted in 53% reduction of synchronous (in-person, telephone) consult requests: from 6.1±4.2 per day to 2.9±2.9 ($P < 0.001$). There was a 77% decrease ($P < 0.001$) in telephone volume to the neuro and thorax beepers, while no significant volume change was noted to the abdomen beeper (control). The positive impact of RadConnect on workflow interruptions was not solely observed through statistical analysis, but was also confirmed through the survey (46% response rate) and interviews.
**Conclusion:** RadConnect significantly reduced workflow interruptions. RadConnect differentiates from a general chat application by role-based interaction and a prioritized worklist overview, which was valued by study participants. Future iterations of RadConnect can potentially contribute to a more focused work environment.
**Limitations:** This was a single-centre study with use limited to CT scans for select sections.
**Ethics committee approval:** The Internal Committee for Biomedical Experiments of Royal Philips (Amsterdam, The Netherlands) approved this study (ICBE-S-000556) and it was registered in clinicaltrials.gov (NCT05540444). Protocol review was waived by the Medical Ethics Committee of the Leiden University Medical Center (N22.056)
**Funding for this study:** Funding was received from Philips.
**Author Disclosures:**
Omar Hertgers: Employee: Leiden University Medical Centre; Research/Grant Support: Philips
Duy Duc Nguyen: Employee: Philips
Stephan Romeijn: Employee: Leiden University Medical Centre; Research/Grant Support: Philips
Merlijn Sevenster: Shareholder: Philips; Employee: Leiden University Medical Centre; Employee: Philips
K.F.M. Hergaarden: Research/Grant Support: Philips; Employee: Leiden University Medical Centre
Joost Roelofs: Employee: Leiden University Medical Centre
Hildo J. Lamb: Consultant: Philips; Employee: Leiden University Medical Centre
Natalie Kruithof: Employee: Leiden University Medical Centre
Sandra Vosbergen: Employee: Philips; Shareholder: Philips

### RPS 1005-11

**Enhancing radiology workflows through efficient x-ray image-based orchestration and classification**

*J. Kohnke*, R. Hosch, J. Haubold, V. Parmar, K. A. Borys, K. Arzideh, L. Umutlu, F. Nensa; Essen/DE
*(judith.kohnke@uk-essen.de)*

**Purpose:** The purpose of this study was to present a classification algorithm to classify up to 12 different X-ray procedures in milliseconds. In radiology, the X-ray modality consistently stands out as the predominant diagnostic procedure in terms of utilization. With the exponential growth in radiological examinations and the concurrent proliferation of artificial intelligence (AI) integrations, there is an emergent demand for an image-based routing system. This system should be adept at automated image orchestration while also being proficient in precise image classification, ensuring the optimisation of data quality and supporting automatic AI workflows.
**Methods or Backround:** An internal dataset of 15,502 x-rays encompassing various anatomical regions for this study was collected which contains the following classes: knee (n=1,676), pelvis (n=692), foot (n=1,686), ankle (n=1,664), wrist (n=1,620), thigh (n=1,183), hips (n=1,496), thorax (n=893), shoulder (n=1,633), thorax lying (n=639), lumbar spine (n=813), and hands (n=1,507). Each class was split using an 80/20 approach for the initial training process. For image classification, the YoloV8 algorithm was employed, and F1-score, sensitivity, and specificity metrics were used for evaluation.
**Results or Findings:** The algorithm demonstrated strong performance with an overall sensitivity of 0.997 and an overall specificity of 0.970. In addition, the model reached an overall F1-score of 0.970, highlighting a robust classifier performance.
**Conclusion:** The presented algorithm shows an accurate and reliable classification performance which could benefit X-ray orchestration and data quality in radiology.
**Limitations:** The trained algorithm should be expanded using more detailed classes for specific X-ray types and evaluated on external data.
**Ethics committee approval:** This study adhered to all guidelines defined by the approving institutional review board of the investigating hospital. The Institutional Review Board waived written informed consent due to the retrospective nature of the study. Complete anonymisation of all data was performed before inclusion in the study.
**Funding for this study:** No funding was received for this study.
**Author Disclosures:**
Katarzyna Anna Borys: Nothing to disclose
Johannes Haubold: Nothing to disclose
Judith Kohnke: Nothing to disclose
Vicky Parmar: Nothing to disclose
Lale Umutlu: Nothing to disclose
Kamyar Arzideh: Nothing to disclose
René Hosch: Nothing to disclose
Felix Nensa: Nothing to disclose

### RPS 1005-12

**Diagnostic accuracy and time efficiency of a novel deep learning algorithm for the assessment of intracranial haemorrhage: first results**

*C. Booz*[1], G. M. Bucolo[2], V. Koch[1], L. D. Gruenewald[1], L. S. Alizadeh[1], A. Gökduman[1], T. D'Angelo[3], T. J. Vogl[1], I. Yel[1]; [1]Frankfurt a. Main/DE, [2]Barcellona Pozzo di Gotto/IT, [3]Messina/IT
*(boozchristian@gmail.com)*

**Purpose:** The objective of the study was to evaluate the diagnostic accuracy and time efficiency of a deep learning-based pipeline using a Dense U-net architecture for the assessment of intracranial hemorrhage (ICH) in unenhanced head CT scans.
**Methods or Backround:** This retrospective study included 502 CT scans of 502 patients (mean age, 70 ± 13 years; 248 men and 254 women) who had undergone an unenhanced head CT scan for the assessment of ICH. All CT scans were analysed by the algorithm and a board-certified radiologist independently for the presence of ICH. In case of ICH presence, ICH had to be defined as intraparenchymal hemorrhage (IPH), intraventricular hemorrhage (IVH), subarachnoid hemorrhage (SAH), subdural hemorrhage (SDH) and epidural hemorrhage (EDH). Additionally, the time until first temporary diagnosis of ICH was measured. Three board-certified radiologists analysed the CT scans in consensus reading sessions to establish the standard of reference for hemorrhage presence and classification.
**Results or Findings:** The reference standard revealed a total of 554 different ICH presences (IPH, n=172; IVH, n=26; SAH, n=163; SDH, n=178; EDH, n=15). The algorithm showed a high diagnostic accuracy for the assessment of ICH with a sensitivity of 92%, specificity of 95% and an accuracy of 93%. Concerning the most frequently present different ICH types in this study, the sensitivity was 92%, 93% and 93% (IPH, SAH and SDH, respectively), and the specificity was 95%, 96% and 95% (IPH, SAH and SDH, respectively). Regarding analysis time, the algorithm was significantly faster compared to the temporary report of the assigned radiologist (15 ± 2 s vs 277 ± 14 s, $p < 0.001$).
**Conclusion:** A novel deep learning algorithm provides high diagnostic accuracy combined with time efficiency for the identification and classification of ICH in unenhanced CT scans.
**Limitations:** Single-centre retrospective study.
**Ethics committee approval:** This study was approved by the local IRB.
**Funding for this study:** No funding was received for this study.
**Author Disclosures:**
Christian Booz: Speaker: Siemens Healthineers
Leon D. Gruenewald: Nothing to disclose
Ibrahim Yel: Speaker: Siemens Healthineers
Vitali Koch: Nothing to disclose
Thomas J. Vogl: Nothing to disclose
Aynur Gökduman: Nothing to disclose
Tommaso D'Angelo: Speaker: Philips Speaker: Bracco
Leona Soraja Alizadeh: Nothing to disclose
Giuseppe Mauro Bucolo: Nothing to disclose

**14:00-15:30** **Research Stage 2**

### Research Presentation Session: Abdominal Viscera & GI Tract

### RPS 1001

### Characterisation of upper GI-tract malignancies and beyond

**Moderator**
D. Tamandl; Vienna/AT

### RPS 1001-2

**A proposal for a new fluoroscopy severity assessment in achalasia: the IVA score**

*G. Fontanella*[1], S. Borrelli[2], B. Brogna[3]; [1]Benevento/IT, [2]Mirabella Eclano/IT, [3]Avellino/IT
*(giovanni.fontanella@hotmail.com)*

**Purpose:** The aim of the study was to establish a quali-quantative fluoroscopic severity assessment for achalasia, comparable to the equivalent clinical Eckhard scoring system.
**Methods or Backround:** From September 2020 to August 2022, 69 patients already diagnosed with achalasia and scored with ESS, were recruited and evaluated with our fluoroscopy barium protocol. The AP sequence was used to divide the esophagus into nine segments, according to Brombart's classic description, plus the gastro-esophageal junction. Three scoring items were chosen, after a profiling study of achalasia, to depict the features, some mutually exclusive, of the three clinical subtypes: lumen dilation, stasis, spasm. Each esophageal segment was scored for the three items (1 point, item present; 0 points, no item), the IVA score was calculated by summing points up until a maximum of 20 points for each subtype was reached. IVA scores were then normalised on a 0-12 scale to be compared to ESS.
**Results or Findings:** IVA and ESS scores were not found to be statistically diverging in 60/69 patients (86.95%, p=0.05). IVA scores were diverging and superior to ESS in 6/69 patients (8.69%); in this group of patients, the ESS' 'chest pain'/'weight loss' items were found to be biasing factors. IVA scores were inferior to ESS in just 3/69 patients (4.34%). In all the patients with a diverging IVA score (9/9), ESS scores were found to be lower than 6/12.
**Conclusion:** IVA score was found to be consistent and compatibile with ESS scores, especially in patients with moderate to severe achalasia. The apparent superiority of imaging scores in a small proportion of patients might instead be used as a revealing tool to call out patients in which the ESS does not reflect the disease's severity, due to internal biases.
**Limitations:** This was a monocentric study and there was a limited number of patients.
**Ethics committee approval:** This study was approved by the FBF BN ethical committee.
**Funding for this study:** No funding was received for this study.
**Author Disclosures:**
Simona Borrelli: Nothing to disclose
Barbara Brogna: Nothing to disclose
Giovanni Fontanella: Nothing to disclose

### RPS 1001-3

**Early tumour shrinkage as a predictor of survival in patients with advanced esophageal squamous cell carcinoma treated with first-line checkpoint inhibitors**

*M. W. Ling*, Y. Zhou, J. Gao; Zhengzhou/CN
*(mumuwl718@163.com)*

**Purpose:** Early tumour shrinkage (ETS) is a promising parameter for assessing treatment responses. Our study hypothesised that an ETS with an optimal cut-off value was an imaging biomarker for advanced esophageal squamous cell carcinoma (ESCC) treated with first-line immunotherapy.
**Methods or Backround:** We retrospectively enrolled 129 patients with unresectable locally advanced ESCC treated with first-line immunotherapy between 2019 and 2021. ETS was defined as the relative change in the sum of the target lesions' longest diameters at the first evaluation compared with that at baseline. Multivariate analyses were conducted to identify the significant prognostic variables in progression-free survival (PFS) and overall survival (OS).
**Results or Findings:** The median value of ETS was 29.5%. An ETS with a 10% cut-off value was statistically significantly associated with PFS in the univariate analysis (hazard ratio [HR]: 2.26; 95% confidence interval [95% CI]: 1.21-4.24; $p = 0.009$). Besides, in the univariate analysis, the longest diameter, maximum short diameter, central necrosis on enhanced computed tomography, enhanced pattern, and ETS values were statistically significant predictive factors for OS. In the multivariate analysis, ETS with a 10% cut-off value was an independent predictive factor for OS (HR: 3.14; 95% CI: 1.45-6.83; $p = 0.004$).
**Conclusion:** ETS is associated with survival outcomes in patients with advanced ESCC treated with immunotherapy. Early tumour size shrinkage of at least 10% can be regarded as a promising biomarker predictor for PFS and OS.
**Limitations:** First, there might have been some bias. Second, the ETS was not sufficient to fully reflect the dynamic process of tumour response to treatment, including the combination of tumour response and treatment time points. The tumour size reduction pattern, morphological response based on imaging, and other factors might be related to the patient's prognosis.
**Ethics committee approval:** Ethics committee approval was provided by the first affiliated hospital of Zhengzhou University.
**Funding for this study:** Funding was provided by the Youth Project of the Henan Natural Science Foundation (no. 212300410271) and the Youth Project in Medical Science and Technology of Henan Province (no. SBGJ202003016).
**Author Disclosures:**
Yue Zhou: Nothing to disclose
Mu Wan Ling: Nothing to disclose
Jianbo Gao: Nothing to disclose

### RPS 1001-4

**Radiographic characterisation of enlarged lymph nodes in locally advanced esophageal squamous cell carcinoma (ESCC) patients treated with neoadjuvant immunotherapy**

*C. Xie*[1], Z. Ning[1], Y. Gao[2], J. Chen[1], Q. Zhang[1], Z. Liu[1], Y. Hu[1]; [1]Guangzhou/CN, [2]Jinan/CN
*(xiechenyi@gdph.org.cn)*

**Purpose:** Neoadjuvant immunotherapy has emerged as a promising therapeutic approach for locally advanced ESCC. This study aims to systematically evaluate suspicion concerning lymph node involvement by synthesising well established imaging observations based on routine workup and reassessment CT images.
**Methods or Backround:** In our retrospective study, we enrolled a total of 100 patients diagnosed with locally advanced ESCC. CT images were meticulously evaluated by experienced radiologists, and enlarged LN were further analysed for size measurement (long-axis diameters, short-axis diameters, and corresponding ratios) and morphological appearances (shape, enhancement pattern, the completeness of the extracapsular border, the presence of fatty hilum, necrosis, fusion, and conglomeration) for prediction of lymph node metastasis (LNM). Subsequently, we compared clinicopathological characteristics between enlarged LN groups presenting different radiological features for exploration of their potential biological significance.
**Results or Findings:** Neoadjuvant immunotherapy yielded a radiologically enlarged LN in 27/100 (27%) of patients. We observed a significant increase in the size of the long-axis diameter (63% vs 39%, p = 0.041) in pathologically negative enlarged LN than malignant LN. A cut-off value of >40% change in lymph node long-axis size was established as a statistically significant discriminator of LNM (AUC = 0.747). Reactive enlargements of LNs are more likely to occur in patients with favourable prognostic and predicative biomarkers (PD-L1 positivity, lower expression of ECD, EGFR, and CD44V6) .
**Conclusion:** Our study has contributed to our understanding of the correlation between CT-based morphological features and enlargements of LNs, potentially addressing a gap in current knowledge. Our findings indicate the observed radiological appearance of cancer progression in lymph nodes may actually be attributed to a special response pattern following neoadjuvant immunotherapy. This step is crucial for ensuring the development of an appropriate clinical treatment plan.
**Limitations:** The sample size was limited.
**Ethics committee approval:** No information provided by the submitter.
**Funding for this study:** No funding was received for this study.
**Author Disclosures:**
Zaiyi Liu: Nothing to disclose
Yingying Gao: Nothing to disclose
Jiahui Chen: Nothing to disclose
Chenyi Xie: Nothing to disclose
Ziyu Ning: Nothing to disclose
Yihuai Hu: Nothing to disclose
Qingling Zhang: Nothing to disclose

### RPS 1001-5

**The significance of small lymph nodes on CT for poorly cohesive advanced gastric cancer**

G. Cho, *E. S. Lee*; Seoul/KR
*(seraph377@gmail.com)*

**Purpose:** The purpose of this study was to compare the size of metastatic lymph nodes on preoperative CT in poorly cohesive advanced gastric cancer with other types of advanced gastric cancers.
**Methods or Backround:** Literature concerning poorly cohesive gastric cancer is scarce and lymph node metastasis is a well known prognostic factor in gastric cancer patients. So, herein, we evaluated poorly cohesive AGC with emphasis on the difference in size of metastatic LNs, compared with other types of AGC. We retrospectively included AGC patients, who underwent gastric cancer surgery at Chung-Ang University Hospital from February 2018 to May 2023. Two abdominal radiologists independently reviewed abdomen CT scans and evaluated the largest size of visible LNs on each gastric LN stations. Measurable LNs (Defined as >3 mm SD) were matched with full pathology report on electronic database records and the metastasis status was determined. We evaluated the size difference of metastatic LNs using independent t-test.
**Results or Findings:** A total of 140 patients (median age, 67 years [IQR, 58-77 years]; 92 men) were evaluated. Poorly cohesive cancer was present in 27 patients (19.7%). Total number of the measurable LNs was 425. 216 out of 425 LNs were matched as malignant based on pathology report. The size of metastatic LNs in poorly cohesive AGC was significantly smaller than metastatic LNs in other types of AGC (p<0.001, geometric mean size: 5.774 vs 7.613).
**Conclusion:** The size of metastatic LNs in poorly cohesive AGC is significantly smaller than metastatic LNs in other types of AGC. Therefore, lowering the size threshold to 5 mm could improve preoperative CT evaluation of metastatic LNs in poorly cohesive AGC.
**Limitations:** The study is a retrospective single-centre study. We evaluated only the largest metastatic LNs per station rather than each and every visible lymph node.
**Ethics committee approval:** This study was approved by the Chung Ang University Hostpital International Review Board.
**Funding for this study:** No funding was received for this study.
**Author Disclosures:**
Gyeongme Cho: Nothing to disclose
Eun Sun Lee: Nothing to disclose

### RPS 1001-6

**Quantitative DCE parameters combined with apparent diffusion coefficient to evaluate molecular typing of gastric cancer**

*Y. Liangliang*, J. Li, J. Qu; Zhengzhou/CN

**Purpose:** To explore the feasibility of quantitative DCE parameters derived from DCE-MRI combined with ADC values to predict the molecular typing of gastric cancer (GC).
**Methods or Backround:** 43 patients were enrolled in this retrospective study. Mean values, 10th, 25th, 50th, 75th, 90th percentile values of quantitative DCE parameters (Ktrans, Kep, Ve) and ADC values were manually extracted. The specimens were performed with five biomarkers, including EBER in situ hybridisation, MLH1, PMS2, E-Cadherin and P53. According to the different expression results, they were divided into five molecular types. The aberrant E-cadherin group and aberrant P53 group were combined into a high-grade malignant group, and the other three groups were combined into a low-grade malignant group. The quantitative DCE parameters or ADC values between two malignant groups were compared.
**Results or Findings:** There were significant differences in Ktrans mean, Ktrans 25%, Ktrans 50%, Ktrans 75%, Ktrans 90%, Kep mean, Kep 10%, Kep 25%, Kep 50%, Kep 75%, Kep 90%, Ve 10%, Ve 25% and ADC between two malignant groups of GC (p = 0.006, 0.044, 0.007, 0.007, 0.009, 0.004, 0.032, 0.024, 0.004, 0.005, 0.016, 0.021, 0.028, 0.018, respectively). Ktrans 90% and ADC were independent risk factors for predicting the two malignant groups. The AUC values, sensitivity, specificity, positive predictive value, and negative predictive value of Ktrans 90%, ADC, Ktrans 90% + ADC were 0.720, 0.688, 0.798; 44.4%, 88.9%, 88.9%; 88.0%, 52.0%, 60.0%; 72.7%, 57.1%, 61.5%; 68.7%, 86.7%, 88.2%, respectively.
**Conclusion:** Quantitative DCE parameters combined with ADC values can assess different malignant groups based on molecular types of GC, which may provide new directions for the evaluation of GC.
**Limitations:** Firstly, the sample size is too small. Secondly, this study did not classify molecularly type of GC according to TCGA.
**Ethics committee approval:** This study was approved by the Ethics Review Committee of Henan cancer hospital.
**Funding for this study:** No funding was received for this study.
**Author Disclosures:**
Jing Li: Nothing to disclose
Jinrong Qu: Nothing to disclose
Yan Liangliang: Nothing to disclose

### RPS 1001-7

**Differential analysis of apparent diffusion coefficient values based on primary tumour and perigastric lymph node for distinguishing N stages of gastric cancer**

*Y. Liangliang*, J. Li, J. Qu; Zhengzhou/CN

**Purpose:** The aim of this study was to explore the differences in ADC values based on the primary tumour and perigastric lymph nodes for distinguishing N stages of gastric cancer (GC).
**Methods or Backround:** 160 GC patients from April 2019 to April 2022 were enrolled in this retrospective study. ADC values and relative ADC values (ADCT, rADCT, ADCLN, rADCLN) based on primary tumours and perigastric first station lymph nodes were measured separately. The ANOVA or Kruskal Wallis test was used to compare differences in ADCT, rADCT, ADCLN, rADCLN values between different N stages. ROC curves were used to determine the optimal parameters and diagnostic efficacy for predicting N0 + 1 and N2 + 3 stages.
**Results or Findings:** There were significant differences in ADCT, rADCT, ADCLN, rADCLN values to distinguish different N stages of GC (p values of <0.001, 0.023, <0.001, <0.001, respectively). The AUC values, sensitivity, specificity, positive predictive value, and negative predictive value of ADCT, rADCT, ADCLN, and rADCLN values in predicting N0 + 1 and N2 + 3 stages of GC were 0.714, 0.632, 0.739, 0.743; 87.7%, 46.2%, 52.3%, 83.1%; 49.5%, 72.6%, 80.0%, 57.9%; 54.3%, 53.6%, 64.2%, 57.4%; 85.5%, 66.3%, 71.0%, 83.3%, respectively.
**Conclusion:** Both ADC values and rADC values can be used to distinguish different N stages of GC, and rADC values based on perigastric lymph nodes have the highest diagnostic efficacy in predicting N0 + 1 and N2 + 3 stages of GC.
**Limitations:** Firstly, this study is a single-centre retrospective study, which may bring some bias to the results. Secondly, as this study mainly evaluates whether there are differences between ADCT and ADCLN values in distinguishing N stages of GC, we did not evaluate T stage and clinical stage, which will be our future research direction.
**Ethics committee approval:** This study was approved by the Ethics Review Committee of Henan cancer hospital.
**Funding for this study:** No funding was received for this study.
**Author Disclosures:**
Jing Li: Nothing to disclose
Jinrong Qu: Nothing to disclose
Yan Liangliang: Nothing to disclose

### RPS 1001-8

**Predioction of the signet ring cells percentage in diffuse-type gastric carcinoma: comparison between morphological CT analysis and radiomics**

*I. Capitoni*, G. Bagnacci, L. Ferradini, N. Di Meglio, A. Perrella, L. Volterrani, M. A. Mazzei; Siena/IT
*(capitoni2@student.unisi.it)*

**Purpose:** Recent evidence has shown that in patients with diffuse-type gastric carcinoma (GC), a high percentage of signet ring cells (SRC) represents a positive prognostic factor (reduced 5-year mortality risk by four times). The aim of this study was therefore to predict the percentage of SRC in GC through radiomics and morphological criteria applied to staging CT scans.
**Methods or Backround:** 44 patients were selected based on the following inclusion criteria: (1) re-evaluation of the percentage of SRC through histopathological examination and (2) good quality of preoperative staging CT scans. The 10% cut-off of SRC was considered to classify patients into PC-NOS (pure poorly cohesive) and PC-SRC (with signet ring cell component). The CT images were assessed by two readers with different levels of experience, who evaluated 12 dichotomous criteria and post-contrast enhancement: the readers also segmented the tumours, and a radiomic analysis was performed using specific software (pyradiomics).
**Results or Findings:** Among the two groups (SRC<10% and SRC>10%), significant differences were found in the distribution of six dichotomous variables and in the post-contrast behavior (ΔHU late-portal 21.5±24.9 VS -26.1±27.2, p=0.001). From the radiomic analysis, 81 out of 106 variables were found to be reproducible between the two readers (ICC>0.75), and the developed model, including three variables, had an area under the curve of 0.726.
**Conclusion:** Patients with different percentages of ring-shaped cells can be accurately identified on CT scans. A larger patient cohort is desirable in the future, considering the prognostic impact of this information, which can only be extracted from a comprehensive analysis of the primary neoplasm.
**Limitations:** This was a monocentric study.
**Ethics committee approval:** Informed consent was obtained from all individual participants included in the study.
**Funding for this study:** No funding was received for this study.
**Author Disclosures:**
Maria Antonietta Mazzei: Nothing to disclose
Iacopo Capitoni: Nothing to disclose
Lorenzo Ferradini: Nothing to disclose
Armando Perrella: Nothing to disclose
Nunzia Di Meglio: Nothing to disclose
Luca Volterrani: Nothing to disclose
Giulio Bagnacci: Nothing to disclose

### RPS 1001-9

**Mystery of lymphoma in gastrointestinal tract**

*F. Mohsin*; Karachi/PK
*(farwadoc@gmail.com)*

**Purpose:** It is important from a prognostic point of view to distinguish primary GI lymphoma from secondary extra-nodal involvement by disseminated nodal disease. Although it has a vast variety of imaging features and exact diagnosis relies on histopathologic analysis, certain imaging appearances like a bulky mass with diffuse infiltration, fat planes preservation, no signs of obstruction, multiple site involvement, associated bulky lymphadenopathy can strongly suggest the diagnosis.
**Methods or Backround:** Primary gastrointestinal (GI) lymphoma is an uncommon disease but is the most frequently occurring extra-nodal lymphoma (10–30%) and is almost exclusively of non-Hodgkin type. The stomach, small bowel, large bowel, and oesophagus are involved in decreasing order of frequency. Risk factors for the development of gastrointestinal lymphoma include H/pylori, immunosuppression, Celiac disease, IBD and HIV.
**Results or Findings:** In the stomach, they typically demonstrate marked thickening of the wall with homogeneous enhancement and submucosal spread. In the small bowel, distal ileum is classically the most common site because of the greater amount of lymphoid tissue in this portion of the bowel. Typical presentation is a thick walled infiltrating mass with aneurysmal dilatation without obstruction which occurs due to tumour invasion into the muscularis propria causing destruction of its intramural autonomic nerve plexus. In the large bowel, they usually appear as bulky polypoid masses on CT, larger than the ones that can be encountered in colorectal adenocarcinomas and may extend beyond the bowel wall, thus presenting as enormous peritoneal masses, that can also be cavitated. Colonic lymphoma usually involves a longer segment, moreover, usually located near the ileocaecal valve and grows into the terminal ileum, not invading or obstructing neighbouring viscera.
**Conclusion:** It is important to decipher the mystery of primary GI lymphomas on the ground of radiology for better management.
**Limitations:** No limitations were identified.
**Ethics committee approval:** Not applicable
**Funding for this study:** No funding was received for this study.
**Author Disclosures:**
Farwa Mohsin: Nothing to disclose

### RPS 1001-10

**Response to neoadjuvat treatment in metastatic gastric cancer: proposal of new radiological criteria from a prospective Italian registry**

*G. Bagnacci*[1], A. Perrella[1], N. Di Meglio[1], L. Funicelli[2], F. Pittiani[3], A. Veltri[4], P. Morgagni[5], G. Mura[6], M. A. Mazzei[1]; [1]Siena/IT, [2]Milan/IT, [3]Brescia/IT, [4]Orbassano/IT, [5]Forli/IT, [6]Arezzo/IT
*(giuliobagnacci@gmail.com)*

**Purpose:** The introduction of neoadjuvant therapy (NAT) for gastric cancer (GC) has led to the need for radiologists to assess response to treatment using CT. The shortcomings of the RECIST 1.1 criteria in assesing NAT response for gastric cancer are well known. Currently, prognostic predictions rely heavily on TNM staging information obtained from staging CT.
**Methods or Backround:** A prospective Italian registry ("METAGASTRO"), focusing on patients with metastatic gastric cancer (GC), is currently ongoing. Our subanalysis included patients who had undergone at least one cycle of neoadjuvant chemotherapy and had both staging and restaging CT scans reviewed by experienced radiologists. We selected 124 patients from six centres affiliated with the GIRCG (Italian Research Group for Gastric Cancer). For each patient, detailed data were collected on approximately 90 variables related to T, N, and M parameters in both staging and restaging.
**Results or Findings:** As expected, patients without peritoneal involvement or with fewer than two hepatic metastases or isolated pathological paraortic lymph nodes demonstrated significantly better survival (p=0.001). RECIST 1.1 offered no prognostic insights (p = 0.233). New criteria, incorporating different lymph node thresholds and Peritoneal Cancer Index (PCI), resulted in effective stratification (p <0.001). The combination of new response criteria with oligometastatic status provided optimal prognostic stratification (p <0.001), with the most favourable group exhibiting a median survival of 41.3 months. Interestingly, non-oligometastatic patients displaying a partial response had a prognosis similar to stable oligometastatic patients.
**Conclusion:** The combination of initial staging and new criteria for response provided satisfactory prognostic stratification for patients affected by metastatic GC. The prognostic value of CT scans can be improved significantly with the expertise of skilled radiologists.
**Limitations:** The small sample size, limited interreader agreement as well as slight variation in time interval between staging and restaging CT were identified as limitations.
**Ethics committee approval:** The study received institutional review board approval and written informed consent was obtained from all participants. The prospective study was approved by the Ethical committee "Comitato Etico Regionale per la Sperimentazione. Clinica della Regione Toscana, Sezione AREA VASTA SUD EST", protocol number 13082_2018, 21/05/2018.
**Funding for this study:** No funding was received for this study.
**Author Disclosures:**
Maria Antonietta Mazzei: Nothing to disclose
Luigi Funicelli: Nothing to disclose
Andrea Veltri: Nothing to disclose
Paolo Morgagni: Nothing to disclose
Gianni Mura: Nothing to disclose
Armando Perrella: Nothing to disclose
Nunzia Di Meglio: Nothing to disclose
Frida Pittiani: Nothing to disclose
Giulio Bagnacci: Nothing to disclose

### RPS 1001-11

**The utility of computed tomography features and histogram texture analysis parameters as diagnostic tool in preoperative differentiation of high-risk gastrointestinal stromal tumours**

*M. Mitrovic*, J. Kovac, L. Lazic, A. Jankovic, D. Šaponjski, S. Milosevic, K. Ebrahimi, D. Mašulović, A. Djuric-Stefanovic; Belgrade/RS
*(dr_milica@yahoo.com)*

**Purpose:** The aim of the study is to determine the morphological characteristics of the tumour obtained by the analysis of the conventional computed tomography examination and texture analysis parameters, which may be useful as imaging biomarkers for the preoperative prediction of high-risk gastrointestinal stromal tumours.
**Methods or Backround:** This was a prospective cohort study that was carried out in the period from 2020 to 2023. The study included 79 patients who underwent CT examination and texture analysis, surgical resection of a lesion that was suspicious for GIST, as well as pathohistological and immunohistochemical analysis.
**Results or Findings:** Textural analysis pointed out Min Norm (p=0.032) as a histogram parameter of the first order statistically significant in the prediction of HR GIST, while Min Norm (p=0.007), Skewness (p=0.035) and Kurtosis (p=0.003) showed significance in predicting high grades of this tumour. Univariate regression analysis identified tumour diameter, margin appearance, growth pattern, lesion shape, structure, mucosal continuity, presence of enlarged feeding or draining vessel (EFDV) and Max Norm as significant predictive factors for HR GIST. Multivariate regression analysis extracted interrupted mucosa (p <0.001) and presence of EFDV (p <0.001) as independent predictive CT features for HR GIST with an AUC of 0.878 (CI: 0.797-0.959), sensitivity of 94%, specificity of 77% and accuracy of 88% in predicting HR GIST.
**Conclusion:** The morphological characteristics of the tumour detected by conventional CT examination still have the greatest value in the preoperative stratification of the metastatic risk of gastric GIST. The incorporation of texture analysis into the basic imaging protocol may further improve the preoperative assessment of risk stratification.
**Limitations:** Our study did not include a follow-up of the involved patients.
**Ethics committee approval:** Our research was permitted by an Ethical Committee of the School of Medicine, University of Belgrade, code 1322/II-6, and written informed consent was obtained from all patients.
**Funding for this study:** This research received no external funding.
**Author Disclosures:**
Dušan Šaponjski: Nothing to disclose
Stefan Milosevic: Nothing to disclose
Aleksandra Jankovic: Nothing to disclose
Jelena Kovac: Nothing to disclose
Ljubica Lazic: Nothing to disclose
Keramatollah Ebrahimi: Nothing to disclose
Milica Mitrovic: Nothing to disclose
Dragan Mašulović: Nothing to disclose
Aleksandra Djuric-Stefanovic: Nothing to disclose

### RPS 1001-12

**Percutaneous transhepatic balloon-assisted (PTB-A) embolisation with ethylene-vinyl alcohol copolymer (EVOH) of duodenal stump fistula after gastrectomy for benign and malignant disease**

*C. Sallemi*, F. Bodini, F. Rosella; Brescia/IT
*(claudiosallemi@hotmail.it)*

**Purpose:** Duodenal stump fistula (DSF) is one of the most serious complications following gastrectomy, with a high risk of morbidity and mortality and a long period of hospitalisation. When conservative management fails, percutaneous transhepatic biliary drainage is useful to reduce duodenal pressure and fistula output by aspirating bile. However, it needs a long healing time and often needs to be followed by other treatments, such as percutaneous or endoscopic injection of glue. The aim of this study was to explore the feasibility and efficacy of a novel technique of percutaneous DFS embolisation with ethylene vinyl alcohol copolymer (EVOH) combined with an occlusion balloon in the duodenal stump for the treatment of DSF after gastrectomy for malignant or benign disease.
**Methods or Backround:** From 2018 to 2023, 11 consecutive patients underwent PTB-A embolisation with EVOH for the treatment of DSF. Clinical and technical success, morbidity and mortality were analysed. Fistula recurrence was also evaluated. Technical success was defined as the absence of contrast media extravasation from the duodenal stump at fistolography after the embolisation. Clinical success was defined as no leakage from the percutaneous tract of the fistula 48 hours after the embolisation. Recurrence was defined as a fistula that recurred after clinically complete healing.
**Results or Findings:** Technical success was achieved in all cases. Clinical success was obtained in 8/10 patients in a single treatment. 2/10 patients were re-treated to achieve complete fistula healing. No procedure-related complications and mortality were recorded. No relapse of fistula occurred during follow-up.
**Conclusion:** PTB-A embolisation with EVOH of DSF after gastrectomy is a feasible and safe procedure and seems to be effective to achieve complete healing of the fistula.
**Limitations:** This was a retrospective study.
**Ethics committee approval:** IRB approval was not waived; written informed consent for PTB-A embolisation was obtained from patients who signed a specific institutional procedure-related consent valid for retrospective observational studies.
**Funding for this study:** No funding was received for this study
**Author Disclosures:**
Claudio Sallemi: Nothing to disclose
Flavio Bodini: Nothing to disclose
Francesco Rosella: Nothing to disclose

**14:00-15:30** **Research Stage 3**

### Research Presentation Session: Breast

### RPS 1002

### What is new in breast ultrasound?

**Moderator**
A. Evans; Dundee/UK
*(a.z.evans@dundee.ac.uk)*

### RPS 1002-2

**False negative and false positive cases after implementation of an automated breast ultrasound system for breast cancer screening: a retrospective study in 1219 women**

*E. Nikolova*, J. K. Weber, G. Zanetti, J. Wieler, T. Frauenfelder, M. Marcon; Zurich/CH

**Purpose:** To investigate the false positive (FP) and false negative (FN) rate over three years after implementation of an automated breast ultrasound (ABUS) system.
**Methods or Backround:** In this IRB-approved retrospective study all women undergoing ABUS examination in our department between October 2015 and October 2018 have been revised (1995 women in total, mean age±SD, 48.9±13 years). We included all women who have undergone a follow-up of at least 24 months after ABUS examination or with BI-RADS category 4 or 5, who have undergone histological evaluation. FP cases were defined as cases classified BI-RADS 3, 4 or 5 in ABUS who have shown to be benign lesions during follow-up or after biopsy. FN cases were defined as all cases of cancer diagnosed during follow-up and that were retrospectively already visible in the ABUS examination. Descriptive statistics were used.
**Results or Findings:** 1219 women (51.9±11.0 years) were included and classification after ABUS examination was: BI-RADS 1 n=136 (11.1%), BI-RADS 2 n=890 (73.0%), BI-RADS 3 n=161 (13.2%), BI-RADS 4 n=15 (1.2%) and BI-RADS 5 n=17 (1.4%). Patients had a mean follow-up±SD of 54.3±16.7 months. A woman was diagnosed with an invasive carcinoma 14 months and another 24 months after ABUS examination classified BI-RADS 2; both lesions were already visible in previous ABUS and mammography and the "retraction phenomenon sign" was visible in the ABUS-coronal reconstruction. A malignancy was also diagnosed in 3/161(1.9%) BI-RADS 3 lesions, 7/15 (46.6%) BI-RADS4 lesions and 17/17 lesions (100%) BI-RADS 5.The FN rate was 6.9% (2/29 cases). The FP rate was 13.9% (166/1190 cases). Among the 14 malignant lesions, in one case the lesion was only visible in ABUS and not in mammography (7.1%); in three cases lesion was visible in both but multifocality only in ABUS (21.4%).
**Conclusion:** We found a FN rate of 6.9% and a FP rate of 13.9%. To avoid false negative cases ABUS coronal reconstruction should be accurately evaluated.
**Limitations:** This was a retrospective study.
**Ethics committee approval:** Ethics committee approval was received; KEK 2016-00064.
**Funding for this study:** No funding was received for this study.
**Author Disclosures:**
Julia Katharina Weber: Nothing to disclose
Elizabet Nikolova: Nothing to disclose
Thomas Frauenfelder: Nothing to disclose
Giulia Zanetti: Nothing to disclose
Jann Wieler: Nothing to disclose
Magda Marcon: Nothing to disclose

### RPS 1002-3

**Learning curve over three years after implementation of an automated breast ultrasound system in an academic radiology department**

*E. Nikolova*, J. K. Weber, G. Zanetti, J. Wieler, T. Frauenfelder, M. Marcon; Zurich/CH
*(enikolova902@icloud.com)*

**Purpose:** The aim of this study was to investigate the learning curve after implementation of an automated breast ultrasound system (ABUS) over three years.

Thursday

**Methods or Backround:** In this IRB-approved retrospective study we included all women undergoing ABUS examination in our department between October 2015 and October 2018 who have undergone a follow-up of at least 24 months after ABUS examination or with BI-RADS category 4 or 5, who have undergone histological evaluation. The number of additional handheld ultrasound exams (AHHUS) for better definition of an ABUS finding and number of false positive (FP) cases were noted. FP were defined as cases classified BI-RADS 3, 4 or 5 in ABUS who have shown to be benign lesions during follow-up/after biopsy. AHHUSs and FPs were compared in the first 6 months versus second 6 months, yearly over the three years and considering presence/absence of previous ultrasound exam (UE). Chi-square test was applied.
**Results or Findings:** A total of 1223 women (mean age±SD, 51.9±11.0 years) were included: 346 (28.3%), 597 (48.8%) and 280 (22.9%) exams were performed respectively in the year 1, 2 and 3. 288/1223 (23.5%) had no previous UE. FP cases were 13/45 (28.9%) in the first 6 months and 70/301 (23.3%) in the second 6 months (p=.454). Cases with AHHUS were 16/45 (35.6%) in the first 6 months and 66/301 (21.9%) in the second 6 months (p=.038). Over three years FP were 83/346 (24.0%), 76/597 (12.7%) and 14/280 (5.0%) for year 1,2 and 3 respectively (p <.001). AHHUS were 82/346 (23.7%), 57/597 (9.5%) and 19/280 (6.8%) for year 1,2 and 3, respectively (p <.001). FP and AHHUS were more frequent in women without previous ultrasound exam (FP:21.2% versus 12.0%, p<.001 and AHHUS: 17.7% versus 11.4%, p <.001).
**Conclusion:** After ABUS implementation FP and AHHUS gradually reduced, especially after the first year; FP and AHHUS are more frequent in women without previous ultrasound exam.
**Limitations:** This was a retrospective study.
**Ethics committee approval:** Ethics committee approval was given; KEK 2016-00064.
**Funding for this study:** No funding was received for this study.
**Author Disclosures:**
Julia Katharina Weber: Nothing to disclose
Elizabet Nikolova: Nothing to disclose
Thomas Frauenfelder: Nothing to disclose
Giulia Zanetti: Nothing to disclose
Jann Wieler: Nothing to disclose
Magda Marcon: Nothing to disclose

### RPS 1002-4

**Added detection potential of ultrasound in the diagnostic assesment of breast cancer: results from personalised, integrated, network, knowledge (PINK) study: an Italian longitudinal multicentric study**

*L. A. Incardona*[1], M. Franchini[2], P. Belli[3], G. M. Giuseppetti[4], G. P. Scaperrotta[5], E. Cassano[3], C. Di Maggio[6], E. Montrucchio[7], J. Nori[1]; [1]Florence/IT, [2]Pisa/IT, [3]Rome/IT, [4]Ancona/IT, [5]Vimodrone/IT, [6]Padua/IT, [7]La Spezia/IT
*(incardonaludovica@gmail.com)*

**Purpose:** The P.I.N.K. study seeks to evaluate the enhanced diagnostic precision in breast cancer screening across Italy, achieved through various imaging technology combinations, with a particular emphasis on the added diagnostic value of Ultrasound (US) in detecting breast lesions, especially those undetected by Mammography (MX).
**Methods or Backround:** Initiated in 2018, the study engaged 30,023 patients aged 40 and above, undergoing MX and at least US and/or Tomosynthesis (TS). Inclusion criteria encompassed patients with histopathologically-confirmed B3-B5 lesions and a positive US assessment (U4-U6), utilizing a complete diagnostic pathway of MX+TS+US or MX+US+TS. The primary objective was to ascertain the additional detection rate of US compared to other imaging techniques, stratifying cases detected solely through US by the MX risk category and age at diagnosis/mammographic breast density.
**Results or Findings:** Of the recruited, 883 had proven breast B3-B5 lesions, with 408 and 27 undergoing MX+TS+US and MX+US+TS pathways respectively. US added detection rate in the MX+TS+US pathway was 14.7%, increasing to 21.5% in benign MX (R2) cases and decreasing to 12% in negative MX (R1) cases. US demonstrated an elevated diagnostic rate in the youngest (40-49 years) and oldest (over 70 years) subgroups, and a higher detection rate (26.1%) in women with dense breasts.
**Conclusion:** The findings suggest that US could be a significant addition to the current breast cancer screening approach, especially where MX may not detect lesions, potentially improving early detection and treatment, thereby enhancing patient outcomes.
**Limitations:** No limitations were identified.
**Ethics committee approval:** This was a retrospective study.
**Funding for this study:** No funding was provided for this study.
**Author Disclosures:**
Cosimo Di Maggio: Nothing to disclose
Jacopo Nori: Nothing to disclose
Edgardo Montrucchio: Nothing to disclose
Gianfranco Paride Scaperrotta: Nothing to disclose
Michela Franchini: Nothing to disclose
Ludovica Anna Incardona: Nothing to disclose
Enrico Cassano: Nothing to disclose
Gian Marco Giuseppetti: Nothing to disclose
Paolo Belli: Nothing to disclose

### RPS 1002-5

**Can the application of the Koios decision support system optimise diagnostic performance of hand-held breast ultrasound? A prospective study**

*G. Irmici*[1], C. Depretto[1], A. Cozzi[2], G. Della Pepa[1], E. D'Ascoli[1], C. De Berardinis[1], A. Bonanomi[1], S. Marziali[1], G. P. Scaperrotta[1]; [1]Milan/IT, [2]Lugano/CH
*(irmici.giovanni25@gmail.com)*

**Purpose:** The aim of this study was to investigate if an artificial intelligence-based decision-support system (Koios DS) influences diagnostic performance of hand-held breast US performed by radiologists of different experience and can reduce the number of unnecessary breast biopsies.
**Methods or Backround:** This prospective monocentric study enrolled consecutive patients referred for breast biopsy in a tertiary-level centre between May 2022 and January 2023. A junior (2 years of breast imaging experience) and a senior reader (20 years experience) assigned US BI-RADS categories before and after Koios application. Differences in diagnostic performance, taking biopsy as the reference standard, were assessed by comparing areas (AUC) under the receiver-operating characteristic curves (DeLong's test). The number of unnecessary biopsies (i.e. yielding benign results) before and after the application of Koios was compared with the McNemar's test.
**Results or Findings:** We enrolled 222 patients (median age 58 years, interquartile range 46–72) with 226 lesions (at biopsy: 137 malignant, 89 benign). The diagnostic performance of the junior reader (AUC 0.785, 95% confidence interval [CI] 0.736–0.836) was significantly improved (p <0.001) by Koios (AUC 0.864, 95% CI 0.819–0.908). Likewise, the diagnostic performance of the senior reader (AUC 0.823, 95% CI 0.777–0.869) was significantly improved (AUC 0.868, 95% CI 0.825–0.912, p <0.001). The diagnostic performance of the junior reader assisted by Koios (AUC 0.864, 95% CI 0.819–0.908) was higher than that of the senior reader alone (AUC 0.823, 95% CI 0.777–0.869), albeit non-significantly (p=0.053). The application of Koios was able to significantly reduce the number of unnecessary biopsies both for the junior (from 51.6% to 32.6%, p<0.001) and the senior reader (from 46.1% to 33.7%, p<0.001).
**Conclusion:** The Koios decision-support system is able to significantly improve the performance of junior and senior readers and reduce the number of unnecessary biopsies.
**Limitations:** This was a single-centre study within a high-experience setting.
**Ethics committee approval:** No information provided by the submitter.
**Funding for this study:** No funding was received for this study.
**Author Disclosures:**
Gianmarco Della Pepa: Nothing to disclose
Andrea Cozzi: Nothing to disclose
Sara Marziali: Nothing to disclose
Elisa D'Ascoli: Nothing to disclose
Alice Bonanomi: Nothing to disclose
Claudia De Berardinis: Nothing to disclose
Gianfranco Paride Scaperrotta: Nothing to disclose
Catherine Depretto: Nothing to disclose
Giovanni Irmici: Nothing to disclose

### RPS 1002-6

**Management of radiological and histopathological discrepancies of breast lesions after US-guided biopsy**

*V. Marisi*[1], M. C. Torrione[2], S. Hannan[2], F. Ricciardella[2], G. Piccolo[2], A. Di Credico[2], A. Figorilli[2], M. Muzi[2], M. Caulo[2]; [1]Vasto/IT, [2]Chieti/IT

**Purpose:** The aim of the study was to analyse the concordance between radiological and histopathological characteristics of breast lesions, demonstrate the high predictive value of the BIRADS (Breast Imaging-Reporting and Data System) classification and asses managment, surgical treatment and follow-up.
**Methods or Backround:** In this retrospective study, we analysed the discrepant findings between imaging and pathological findings in patients undergoing breast biopsy between January 2022 and April 2023. 857 patients with 957 lesions undergoing ultrasound-guided breast biopsy were included in this study. Exclusion criteria were BIRADS4 b having been performed on patients or biopsies with another first-instance method (stereotaxis or MR-guided biopsy).

**Results or Findings:** 52 discordant biopsies were evaluated by two experts in breast radiology. 32 classified as BIRADS3 or 4a were found to be B5 on histological examination (2 infiltrating lobular carcinoma, 8 ductal carcinoma in situ, 16 invasive carcinoma NST, 2 infiltrating ductal carcinoma, 1 papillomatosis, 1 infiltrating papillary carcinoma and 2 invasive G2 carcinoma); 20 biopsies classified BIRADS4c or 5 were B1 (4), B2 (10) and B3 (6). According to discordance radiological and histopathological findings, 2 patients with B1 results underwent a new biopsy or VAAB: the result was B5 (1) and B3 (1); 5 patients with B2 result underwent VABB or surgical excision: the result was B5 (4 patients) and B2 (1 patients).
**Conclusion:** The BIRADS classification is fundamental in breast cancer diagnosis. When there are discrepancies between imaging and histological results a discussion by an interdisciplinary team is mandatory: this is crucial in clarifying why they exist and to re-evaluate the most suitable methodology in continuing the diagnostic process. In our series, 52% of suspicious lesions with a benign result were found to be malignant after rebiopsy or surgery.
**Limitations:** No limitations were identified.
**Ethics committee approval:** No information provided by the submitter.
**Funding for this study:** No funding was received for this study.
**Author Disclosures:**
Sumi Hannan: Nothing to disclose
Gaetano Piccolo: Nothing to disclose
Vanessa Marisi: Nothing to disclose
Federica Ricciardella: Nothing to disclose
Maria Concetta Torrione: Nothing to disclose
Andrea Figorilli: Nothing to disclose
Massimo Caulo: Nothing to disclose
Angela Di Credico: Nothing to disclose
Marzia Muzi: Nothing to disclose

### RPS 1002-7

**Response prediction of neoadjuvant treatment in breast cancer patients at baseline ultrasound: comparison of B-mode and a deep-learning model**

*P. Kapetas*[1], C. Fürböck[1], R. Aggarwal[2], B. Altuwayjiri[3], P. Clauser[1], T. H. Helbich[1], G. Langs[1], P. A. Baltzer[1]; [1]Vienna/AT, [2]Southend-On-Sea/UK, [3]Riyadh/SA
*(panagiotis.kapetas@meduniwien.ac.at)*

**Purpose:** The study aimed to evaluate whether pre-therapeutic breast ultrasound (US) can serve in the prediction of breast cancer (BC) patients non-responding to neoadjuvant chemotherapy (NAC) and to compare two different models.
**Methods or Backround:** This retrospective, IRB-approved study included 245 patients with histologically confirmed BC undergoing NAC. The dataset was divided into a training (165 cases) and a validation set (80 cases). A representative B-mode US image of each tumour from the pre-treatment examination was selected. Two experienced breast fellows independently evaluated the lesions using standard BI-RADS descriptors. Logistic regression was used to identify independent predictors of NAC response in the training set and create a model. Additionally, a Resnet18-based neural network with Dropout layers to decrease the amount of overfitting was trained to predict the treatment outcome. The performance of both models was evaluated on the validation set using descriptive statistics. Postoperative histology was the standard of reference for treatment response.
**Results or Findings:** 145 patients (59.2%) did not achieve a pathological complete response. From the BI-RADS descriptors, oval or round shape, microlobulated or spiculated margin and the presence of calcifications or edema proved to be independent predictors of pCR. A model using these showed an accuracy, sensitivity, specificity, positive and negative predictive value of respectively 65%, 82%, 41%, 67% and 61% for the prediction of non-responders to NAC. Compared to that, the DL-based model achieved an accuracy, sensitivity, specificity, positive and negative predictive value of 76%, 88%, 59%, 76% and 78%.
**Conclusion:** Breast US can accurately predict lack of response to NAC for BC patients prior to its initiation. A DL model using images from the baseline US examination demonstrates an increased diagnostic performance as compared to standard B-mode BI-RADS descriptors.
**Limitations:** Monocentric study, small patient number.
**Ethics committee approval:** Ethics committee - additional information: This study was approved by the Medical University of Vienna (Ref. Number: 1793/2013)
**Funding for this study:** No funding was received for this study.
**Author Disclosures:**
Pascal A.T. Baltzer: Nothing to disclose
Christoph Fürböck: Nothing to disclose
Georg Langs: Nothing to disclose
Basmah Altuwayjiri: Nothing to disclose
Thomas H. Helbich: Nothing to disclose
Panagiotis Kapetas: Nothing to disclose
Paola Clauser: Nothing to disclose
Reena Aggarwal: Nothing to disclose

### RPS 1002-8

**Ultrasound Doppler twinkling-guided identification of a breast biopsy marker after neoadjuvant systemic therapy: preliminary data from a phase one clinical trial**

*C. Lee*, G. Hesley, M. Urban, M. Piltin; Rochester, MN/US
*(lee.christine@mayo.edu)*

**Purpose:** The purpose of this clinical trial is to evaluate the safety and twinkling signature of the marker after months of NST. Ultrasound detection of commercial breast biopsy markers is challenging, particularly in the axilla in patients with node-positive breast cancer responding favorably to neoadjuvant systemic therapy (NST). We have developed a biopsy marker that demonstrates robust ultrasound color Doppler twinkling.
**Methods or Backround:** In an ongoing, prospective, single-arm trial involving 10 patients with node-positive breast cancer, a twinkling marker was implanted near the conventional biopsy marker (control) in the positive axillary lymph node before the start of NST. Ultrasound was performed at baseline, during NST, and preoperatively to assess for twinkling and B-mode conspicuity of the twinkling and commercial markers. Based on the preoperative ultrasound, the surgeon determined whether I-125 seed localisation of the positive node was necessary or if intraoperative ultrasound guidance, as part of their surgical practice, would be used to retrieve the marked positive node.
**Results or Findings:** Eight of 10 patients with a mean age of 49.5 years (SD 14.1) have enrolled. Each patient has a different commercial biopsy marker in the positive node. Thus far, four of the eight patients had ultrasounds during NST, and twinkling of the twinkling marker was readily present on two different ultrasound scanners (GE Logiq and Fujifilm Arietta). No adverse events. Three patients have completed targeted axillary dissection, and the surgeon successfully used ultrasound twinkling intraoperatively to identify the positive node. None of the three patients required preoperative axillary radioactive seed localisation.
**Conclusion:** Early preliminary results are promising for identifying a marker by ultrasound Doppler twinkling after NST, possibly obviating preoperative localisation.
**Limitations:** The small study size and involvement of a single surgeon are limitations.
**Ethics committee approval:** The study received approval from the Mayo Clinic Institutional Review Board, and written consent was obtained from all participants. This study is registered under ClinicalTrials.gov NCT0554347.
**Funding for this study:** Funding was received from Mayo Clinic President's Discovery Translation Program.
**Author Disclosures:**
Gina Hesley: Nothing to disclose
Mara Piltin: Nothing to disclose
Matthew Urban: Nothing to disclose
Christine Lee: Nothing to disclose

### RPS 1002-9

**Tanner stage and ultrasonographic breast development in girls diagnosed with premature thelarche and central precocious puberty; a prospective follow-up in CPP patients receiving "blocking" therapy**

C. Boldrini, *R. Dattoli*, A. Marra, S. Amodeo, P. Belli, R. Manfredi; Rome/IT
*(roberta.dattoli@hotmail.it)*

**Purpose:** This study aims to evaluate, in a population of pediatric patients diagnosed with premature thelarche (PT) and central precocious puberty (CPP), the correlation existing between the clinical stage of breast development according to Tanner and the ultrasonographic stage of the mammary gland, both at the diagnosis and after pubertal "blocking" therapy, the latter evaluated in CPP girls. Under hormonal stimulation from birth until puberty, various changes in the mammary gland occur.
**Methods or Backround:** A population of 42 pediatric female patients with evidence of breast "bump" at an age below the threshold of eight years was selected. The girls clinically ascertained to have breast development (Tanner stage B2 and subsequent) underwent routine tests: measurement of basal levels of FSH, LH and E2, GnRH stimulation test, determination of bone age using hand-wrist X-ray, vitamin D dosage. The ultrasonographic stage was established by taking into consideration the dimensionally largest breast and assigned from one to five. Patients diagnosed with CPP were candidates for carrying out blocking therapy (Decapeptyl®); after six months of therapy they were recalled to evaluate again growth speed, bone age, and US breast development.
**Results or Findings:** It was observed that the average number of patients at the diagnosis of CPP was stage 3 at the US examination (versus Tanner stage B2), and that after six months of blocking therapy the average was stage 2 at US. Clinical evaluation according to Tanner stages tended to overestimate breast development.
**Conclusion:** The assessment of the ultrasonographic stage of breast development represents a novel method to help clinicians in the evaluation of little girls affected with PT e CPP, either in the diagnosis or follow-up, and might become routine very soon in the future.
**Limitations:** Limited number of patients

**Ethics committee approval:** No information provided by the submitter.
**Funding for this study:** The study did not receive any funding.
**Author Disclosures:**
Cristiana Boldrini: Nothing to disclose
Angelica Marra: Nothing to disclose
Roberta Dattoli: Nothing to disclose
Riccardo Manfredi: Nothing to disclose
Silvia Amodeo: Nothing to disclose
Paolo Belli: Nothing to disclose

### RPS 1002-10

**Invasive recurrence after breast conserving treatment of ductal carcinoma in situ of the breast between 1989 and 2021: time trends and the role of tumour grade**

*L. Duijm*[1], R. O'Leary[2], L. Boersma[2], M. van der Sangen[3], S. Siesling[4], R-J. Schipper[3], A. Voogd[2]; [1]Nijmegen/NL, [2]Maastricht/NL, [3]Eindhoven/NL, [4]Utrecht/NL
*(lemduijm@hotmail.com)*

**Purpose:** The aim of this study was to provide updated figures on the risk of invasive ipsilateral breast cancer (iIBC) after breast conserving surgery (BCS) of ductal carcinoma in situ (DCIS) with or without adjuvant radiotherapy (RT). A second aim was to analyse the association between DCIS grade and the risk of iIBC following BCS.
**Methods or Backround:** In this population-based, retrospective cohort study, the Netherlands Cancer Registry collected patient information on 25,719 women who received a new diagnosis of DCIS in the Netherlands from 1989-2021 and who underwent BCS, of which 19,034 (74%) received adjuvant RT. Kaplan-Meier analyses and Cox multivariable regression models were used in the analyses.
**Results or Findings:** A total of 1,135 patients experienced an iIBC. The 10-year cumulative iIBC incidence rates for patients diagnosed in the periods 1989-1998, 1999-2008 and 2009-2021 and undergoing BCS only, were 12.6%, 9% and 5% (P<0.001), respectively. For those undergoing BCS with RT these figures were 5.7%, 3.7% and 2.2%, respectively (P<0.001). In the multivariable analyses, DCIS grade was not significantly associated with the risk of iIBC.
**Conclusion:** Since 1989 the risk of iIBC has decreased substantially. No significant association of DCIS grade with the risk iIBC was found, stressing the need for more powerful prognostic factors to guide the loco-regional treatment of DCIS.
**Limitations:** The well known poor reproducibility among pathologists in grading DCIS lesions will haveresulted in misclassification of tumour grade and is a potential source of information bias.
**Ethics committee approval:** Informed consent was not necessary for this observational study. Patients are informed that their data is part of the Netherlands Cancer Registry, and there is an optional opt-out procedure available. No approval for this study was required from a Medical Ethics Committee.
**Funding for this study:** No funding was received for this study.
**Author Disclosures:**
Maurice van der Sangen: Nothing to disclose
Lucien Duijm: Nothing to disclose
Rebecca O'Leary: Nothing to disclose
Sabine Siesling: Nothing to disclose
Liesbeth Boersma: Nothing to disclose
Adri Voogd: Nothing to disclose
Robert-Jan Schipper: Nothing to disclose

**14:00-15:30 Research Stage 4**

### Research Presentation Session: Interventional Radiology

### RPS 1009

### New insights in percutaneous tumour ablation

**Moderator**
A. Gangi; Strasbourg/FR
**Author Disclosures:**
Afshin Gangi: Advisory Board: Boston Sc; Patent Holder: Aptiomed

### RPS 1009-2

**Correlation between thermal dose in real-time thermometry and postprocedural ablation zone in MRI-guided microwave ablation of liver tumours**

*O. Öcal*[1], S. Lentini[1], O. Dietrich[1], P. Bour[2], T. Faller[2], J. Ricke[1], M. Seidensticker[1]; [1]Munich/DE, [2]Pessac/FR
*(osman.oecal@med.uni-muenchen.de)*

**Purpose:** This study aims to evaluate the correlation of thermal dose volume with the ablation zone in postprocedural first-day images. Microwave ablation is an established treatment alternative to surgical resection with similar efficacy and better tolerability in patients with primary or secondary liver tumours with limited disease burden. Nevertheless, it is associated with around 10% local disease recurrence, possibly due to incomplete tumour ablation. MRI guidance offers better lesion targeting with higher soft-tissue contrast during the procedure, as well as the possibility of real-time thermometry. However, no in-vivo comparison of real-time thermometry with the ablation zone in follow-up images has been reported.
**Methods or Backround:** All procedures were performed in general anesthesia on a 1.5T MRI scanner. The real-time thermometry sequence was acquired using gradient-echo EPI sequences, and thermal dose (CEM43 of 240 minutes as a threshold) maps were created using dedicated software (Certis Solution, Certis Therapeutics, Pessac, France). Thermal dose volumes were compared with the ablation zone in postprocedural first-day images using Pearson correlation test.
**Results or Findings:** 24 patients with 27 lesions were included in this study. The volume of thermal dose and ablation zone showed a strong correlation (R=0.89, p<0.001).
**Conclusion:** The real-time thermal dose mapping shows a very good correlation with the ablation zone volume obtained one day after the procedure. Real-time visualisation of inadequate ablation margins could reduce the local recurrence rates with the possibility of reablating lesions within the same procedure.
**Limitations:** - Retrospective nature of the study - Relatively small sample size
**Ethics committee approval:** This study was approved by the Ludwig Maximilian University of Munich.
**Funding for this study:** No funding was received for this study.
**Author Disclosures:**
Olaf Dietrich: Nothing to disclose
Thibaut Faller: Employee: Certis Therapeutics
Max Seidensticker: Nothing to disclose
Osman Öcal: Nothing to disclose
Pierre Bour: Employee: Certis Therapeutics
Sergio Lentini: Nothing to disclose
Jens Ricke: Nothing to disclose

### RPS 1009-3

**Comparison of thermal ablation combined with synchronous TACE and TACE in liver metastasis of neuroendocrine tumours of different pathologic grades and different primary sites**

*H. Sun*; Shanghai/CN

**Purpose:** The objective of this study was to compare the thermal ablation combined with synchronous TACE and TACE in patients with liver metastasis of NETs of different pathologic grades and primary sites.
**Methods or Backround:** A retrospective study was performed on patients with liver metastases of NETs in the interventional therapy Department of Zhongshan Hospital of Fudan University from November 1, 2006 to July 31, 2022, who were divided into synchronous ablation group and TACE group and subgroups according to pathological grades and primary sites. The patients were followed up until July 31, 2023. The endpoint was progression-free survival (PFS) and overall survival (OS).

**Results or Findings:** A total of 86 patients were collected, including 34 patients in simultaneous ablation group and 52 patients in TACE group, 45 patients with G2 stage, 51 patients of pNETs. The median PFS was 18.0 months in the TACE group (95% CI, 6.0-30.0 months) and 29.0 months in the synchronous ablation group (95% CI, 10.0-48.0 months), with no statistical difference (P=0.22). In the 45 patients with G2 stage, the median OS and PFS are 47.0 and 12.0 months in the TACE group and 59.0 and 32.0 months in the synchronous ablation group (P=0.45 0.032),with the difference of PFS was statistically significant. Of the 51 pNETs patients, the median OS and PFS are 47.0 and 18.0 months in the TACE group and 59.0 and 34.0 months in the synchronous ablation group (P=0.22 0.005),with the difference of PFS was statistically significant.
**Conclusion:** Simultaneous ablation can delay disease progression of liver metastasis of neuroendocrine tumours, and has a good safety, especially for patients with liver metastases of neuroendocrine tumours of intermediate or low grade or pancreatic origin.
**Limitations:** Due to the small sample size and confounding factors, some differences are not reflected.
**Ethics committee approval:** This study was approved by Zhongshan Hospital of Fudan University.
**Funding for this study:** Funding was received from the National Health Commission Capacity Building And Continuing Education Center (GWJJ2022100303), and Fudan University Integrated Medical Engineering Program (yg2022-6).
**Author Disclosures:**
Huiyi Sun: Nothing to disclose

### RPS 1009-4

**3D fusion is superior to 2D point-to-point contrast-enhanced US to evaluate the ablative margin after RFA for hepatocellular carcinoma**
*H. Long*[1], X. Zhou[1], X. Zhang[1], J. Ye[2], T. Huang[1], L. Cong[3], G. Huang[1], X. Xie[1]; [1]Guangzhou/CN, [2]Foshan/CN, [3]Shenzhen/CN
*(longhy9@mail.sysu.edu.cn)*

**Purpose:** The purpose of the study was to compare the 3D and 2D contrast-enhanced ultrasound (CEUS)-derived techniques in evaluating the ablative margin (AM) after radiofrequency ablation (RFA) for hepatocellular carcinoma (HCC).
**Methods or Backround:** In total, 98 patients with 98 HCCs were enrolled. The 2D CEUS point-to-point imaging (2DCEUS-PI) and the 3D CEUS fusion imaging (3DCEUS-FI) were compared to distinguish an adequate AM ≥ 5 mm. Risk factors for local tumour progression (LTP) after RFA were analysed by the Kaplan-Meier method with the log-rank test.
**Results or Findings:** The mean registration time of 3DCEUS-FI and 2DCEUS-PI was 5.0 and 9.3 min, respectively ($p < 0.0001$). The kappa coefficient was 0.680 for agreement between 2DCEUS-PI and 3DCEUS-FI in the evaluation of AM ($p < 0.0001$). Tumours with AM < 5 mm by 2D CEUS-PI were all identified as AM < 5 mm by 3D CEUS-FI. Nonetheless, 16 (26%) tumours identified as AM ≥ 5 mm by 2DCEUS-PI were re-classified as AM < 5 mm by 3DCEUS-FI. During a median follow-up time of 31.2 months (range, 3.2-66.0 months), LTP was identified in 8 tumours. The estimated 1-/2-/3-year cumulative incidence of LTP was 4.4%, 8.1%, and 10.3%, respectively. Higher estimated cumulative incidence of LTP was identified in tumours with AM < 5 mm by 2DCEUS-PI (at 3-year, 27.2% vs 0%; $p < 0.001$), and by 3DCEUS-FI (at 3-year, 20.7% vs 0%; $p = 0.004$).
**Conclusion:** 3DCEUS-FI excelled in the evaluation of AM when compared with 2DCEUS-PI. With equivalent efficacy in the prediction of LTP, 3DCEUS-FI was superior to 2DCEUS-PI for its automatic and time-saving procedure.
**Limitations:** Further studies of these techniques in guiding intraoperative management for HCC are warranted to validate their value in achieving adequate AM and lowering the LTP rate.
**Ethics committee approval:** This prospective study was approved by the institutional review board of the First Affiliated Hospital of Sun Yat-sen University and informed consent was obtained from all patients.
**Funding for this study:** This research was supported by the National Natural Science Foundation of China (Grants No. 92059201, No. 81530055, and No 815014937.
**Author Disclosures:**
Jieyi Ye: Nothing to disclose
Haiyi Long: Nothing to disclose
Longfei Cong: Nothing to disclose
Xiaoyu Zhou: Nothing to disclose
Guangliang Huang: Nothing to disclose
Xiaoyan Xie: Nothing to disclose
Xiaoer Zhang: Nothing to disclose
Tongyi Huang: Nothing to disclose

### RPS 1009-5

**Gradual incremental high-power radiofrequency ablation with multi-electrodes for small hepatocellular carcinoma: a prospective study**
*S. Hwang*, J. M. Lee; Seoul/KR
*(readybox123@gmail.com)*

**Purpose:** This study prospectively assesses the local tumor progression (LTP) and intrahepatic remote recurrence (IRR) rates of gradual, stepwise, high power RFA in treating HCCs (≤4 cm).
**Methods or Backround:** Patients with single HCCs (≤4 cm) scheduled for treatment with gradual, stepwise, high energy RFA, utilising a separable clustered electrode and a two-channel generator, ranging from 60 W to 200 W for each generator, between January 2020 and July 2022, were prospectively enrolled. The ablation procedure targeted the index tumor, guided by real-time US-CT/MR fusion imaging, and alternately delivered monopolar energy to twp of the three clustered electrodes. Primary, secondary, and tertiary endpoints encompassed the 3-year LTP rate, IRR rate, and recurrence free survival (RFS) rate, respectively. Technical success, complications, and cumulative incidences of LTP and IRR, along with RFS, were assessed and estimated using the Kaplan–Meier method.
**Results or Findings:** Among 110 participants (83 men and 27 women, mean age: 66.4±7.6 years), 116 HCCs (mean size: 1.65±0.59 cm) were treated with no major complications. LTP and IRR were observed in four and 29 patients, respectively. At a median follow-up of 41.0 months (range: 35.4-46.6 months), the estimated 1-year, 2-year, and 3-year cumulative incidences were as follows: LTP (0.9%, 3.6%, 7.0%) and IRR (13.9%, 20.5%, 31.4%). The corresponding recurrence-free survival rates were: LTP (99.1%, 96.4%, 93.0%) and IRR (86.1%, 79.5%, 68.6%).
**Conclusion:** Gradual increment of dual switching monopolar RF applicators may help improve LTP and IRR compared to conventional methods.
**Limitations:** The study was conducted in a single centre with a single-arm, which might introduce some degree of bias in the results. Multi-center studies with a larger sample size and control groups are essential for further validating the results and making more generalised conclusions.
**Ethics committee approval:** This study was conducted ethically in accordance with the World Medical Association Declaration of Helsinki and approved by the Institutional Review Board of Seoul National University Hopsital. All enrolled participants provided written informed consent.
**Funding for this study:** This study received technical support and was supported by a research grant from STARmed Co.
**Author Disclosures:**
Jeong Min Lee: Nothing to disclose
Sungjun Hwang: Nothing to disclose

### RPS 1009-6

**Microwave ablation for colorectal liver metastases with ultrasound fusion imaging assistance: a stratified analysis study based on tumour size and location**
*E. Xu*, J. Lin, H. Liu, S. Liang, R. Yan; Shenzhen/CN
*(xuerjiao@126.com)*

**Purpose:** This study aimed to investigate the efficacy of microwave ablation (MWA) for colorectal liver metastases (CRLM) with ultrasound fusion imaging assistance, and conduct stratified analysis based on tumour size and location.
**Methods or Backround:** Patients with CRLM who underwent MWA with the assistance of ultrasound fusion imaging from February 2020 to February 2023 in our hospital were enrolled into this retrospective study. Ultrasound fusion imaging was used for detection, guidance, monitoring and immediate evaluation throughout the MWA procedures. The technical success, technical effectiveness, local tumour progression (LTP), intrahepatic progression and overall survival (OS) were recorded and analysed. The subgroup analysis of efficacy of MWA for CRLM was performed according to tumour size and location.
**Results or Findings:** A total of 51 patients with 122 nodules were enrolled. Both technical success and effectiveness were acquired in all nodules (122/122, 100%). In a median follow-up period of 19 months (range, 5-39 months), LTP was observed in 2.5% of the nodules (3/122). The cumulative intrahepatic progression rates were 38.7% and 52.1% at 1 year and 2 years, respectively. The cumulative OS rates were 90.8% and 77.5% at 1 year and 2 years, respectively. Patients were divided into subgroups according to tumour size (≥30 mm, n=13; <30 mm, n=38) and tumour location (perivascular, n=20; non-perivascular, n=31 and subcapsular, n=36; non-subcapsular, n=15). The cumulative intrahepatic progression rates were similar between the subgroups regarding tumour size and perivascular location, while significantly higher in the subcapsular group than in the non-subcapsular group (p=0.021).
**Conclusion:** Ultrasound fusion imaging assisted-MWA exhibited satisfactory local efficacy for CRLM, especially for non-subcapsular tumors.
**Limitations:** First, it was a retrospective single-arm study performed in a single centre. Second, this study merely focused on short-term local tumors and intrahepatic effectiveness.

**Ethics committee approval:** This study was approved by the Eighth Affiliated Hospital of Sun Yat-Sen University.
**Funding for this study:** Funding was received from the National Natural Science Foundation of China (No. 82272011) and Natural Science Foundation of Guangdong Province (No. 2022A1515012155).
**Author Disclosures:**
Ronghua Yan: Nothing to disclose
Huahui Liu: Nothing to disclose
Shuang Liang: Nothing to disclose
Erjiao Xu: Nothing to disclose
Jia Lin: Nothing to disclose

### RPS 1009-7

**Microwave ablation in malignant liver lesions: extracellular vesicle surface expression as prognostic parameter**

*T. J. Vogl*[1], J. Trebicka[2], E. Oppermann[1], L. Ysermann[1], W. Gu[1], A. Pascher[2], B. Strücker[2], W. Bechstein[1], M. Juratli[2]; [1]Frankfurt a. Main/DE, [2]Münster/DE
*(t.vogl@em.uni-frankfurt.de)*

**Purpose:** The study aimed to characterise extracellular vesicles (EV) by liquid biopsy in preinterventional patients with primary and secondary hepatic malignancies treated with microwave ablation.
**Methods or Backround:** Blood samples of 38 HCC patients and 15 patients with hepatic metastases were collected immediately pre- and post-interventional. Subsequently, the characterisation of 37 surface epitopes of EVs by magnetic bead-based particle sorting and fluorescence-associated cell scanning (FACS) was conducted.
**Results or Findings:** The HCC-group before intervention revealed an activation of T-cell-associated EV protein expressions with significantly increased CD40, CD86 and CD8, vs. the non-HCC group. Additionally, the pre- and post-interventional HCC group showed an increment of the B-cell associated marker CD20 vs. the non-HCC group, respectively. The tumour cell associated surface epitopes, CD44, CD133 and CD24 were significantly higher expressed in the pre-interventional HCC vs. non-HCC group (p=0.029, p=0.009, p=0.004). Regarding the clinical parameters, the HCC-group showed a significantly higher expression of CD9 in patients with recurrent HCC, nonalcoholic steatohepatitis (NASH)-related HCC and cirrhotic HCC. Furthermore, the subgroup analysis of HCC-patients showed a significantly lower expression of CD44 in NASH-related HCC and cirrhotic HCC. Pre-interventional cytokine levels of TH1 and Treg cells (IL2+IL17) correlated strongly with elevated CD44 levels in HCC patients. Additionally, pre-interventional IL6 levels correlated strongly with a high expression of CD19 and CD86. Regarding the post-interventional changes increased CD19, CD20 and CD44 levels correlated strongly with an elevated CD4/CD8 ratio and negatively correlated with CD4/CD8 ratio changes.
**Conclusion:** EV surface expressions correlated with cytokine levels in pre-interventional HCC-patients showed a CD4+ TH1 response, associated with CD44 expression.
**Limitations:** Small number of participants.
**Ethics committee approval:** This study was approved by the ethic committee of the Johann Wolfgang Goethe university, Frankfurt (19-443, 10.09.2020).
**Funding for this study:** No funding was received for this study.
**Author Disclosures:**
Jonel Trebicka: Nothing to disclose
Andreas Pascher: Nothing to disclose
Mazen Juratli: Nothing to disclose
Thomas J. Vogl: Nothing to disclose
Wenyi Gu: Nothing to disclose
Lisa Ysermann: Nothing to disclose
Wolf Bechstein: Nothing to disclose
Benjamin Strücker: Nothing to disclose
Elsie Oppermann: Nothing to disclose

### RPS 1009-8

**Interventional treatment of unresectable pancreatic cancer: single-centre evaluation over 12 years using microwave ablation (MWA) and transarterial chemoembolisation (TACE)**

*T. J. Vogl*, R. Cojocaru, H. Adwan; Frankfurt a. Main/DE
*(t.vogl@em.uni-frankfurt.de)*

**Purpose:** The aim of th e study was to retrospectively evaluate the safety and efficacy of microwave ablation (MWA) and transarterial chemoembolisation (TACE) in the treatment of unresectable pancreatic cancer (UPC) regarding local tumour progression and complication rates.
**Methods or Backround:** From 2010 to 2022, a total of 150 patients with UPC underwent either combined TACE and MWA treatment (n=67, 40 males/27 females) or TACE as monotherapy (n=83, 40 males/43 females). In the first group, 23/67 (34.3%) patients had metastatic pancreatic cancer and 44/67 (65.7%) locally advanced pancreatic cancer. In total, 222 TACEs and 71 MWAs were performed (average: 3.3 TACEs/patient prior to MWA. 4 patients received 2 MWAs, 63 patients 1 MWA). 18/67 (26.9%) patients were treated with curative intent, 49/67 (73.1%) with palliative or symptomatic intent. In the second group, 45/83 (54.2%) patients had metastatic pancreatic cancer and 38/83 (45.8%) patients locally advanced pancreatic cancer. In total, 250 TACEs were performed (average three TACEs/patient). 3/83 (3.6%) patients were treated with curative intent, 80/83 (96.4%) with palliative/symptomatic intent.
**Results or Findings:** No major complications occurred during the procedure. In 4/67 (6%) patients, mild hemorrhage was observed after MWA without further consequences. In the first group, a follow-up was available for 47/67 patients two to six months post MWA with PET-CT (n=1), abdominal CT (n=8) and abdominal MRI (n=38). Local tumour progression was observed in 5/47 patients (10.6%). In the second group, 1-month MRI follow-up was available for all 83 patients. Local tumour progression was observed in 9/83 (10.8%) patients.
**Conclusion:** TACE and MWA are both safe methods in the local treatment of unresectable pancreatic cancer. Combined TACE and MWA could be a promising treatment option for patients with UPC.
**Limitations:** Retrospective study design.
**Ethics committee approval:** This study was approved by the ethics committee of the Johann Wolfgang Goethe University Frankfurt.
**Funding for this study:** No funding was received for this study.
**Author Disclosures:**
Ruxandra Cojocaru: Nothing to disclose
Thomas J. Vogl: Nothing to disclose
Hamzah Adwan: Nothing to disclose

### RPS 1009-9

**CT-based deep-learning radiomics nomogram for the prediction of immediate response in colorectal cancer lung metastases treated by radiofrequency ablation**

*H. Huang*, W. Li; Shanghai/CN
*(hhzfudan2012@126.com)*

**Purpose:** The aim of this study was to construct deep learning radiomics nomogram to assess the instant response in lung metastases of colorectal cancer (CRC) after radiofrequency ablation (RFA).
**Methods or Backround:** We retrospectively included 515 lung metastases in 233 CRC patients who received RFA (412 in the training group and 103 in the test group). Multivariable analysis was performed to identify independent risk factors for developing the clinical model. Tumor and ablation regions of interest (ROI) were split into three spatial habitats through K-means clustering and dilated with 5 mm and 10 mm thicknesses. Deep learning (DL) features and radiomics features including intratumour, peritumour, and habitat were extracted from intraoperative CT data. Predictive models using common machine learning classifiers were constructed, and a nomogram was developed by combining DL and radiomics signature with clinical factors. The performance was primarily evaluated using the area under the receiver operating characteristics curve (AUC) via the DeLong test, calibration curves through the Hosmer-Lemeshow test, and decision curve analysis.
**Results or Findings:** A total of 412 out of 515 metastases (80%) achieved complete response. Four clinical variables (cancer antigen 19-9, simultaneous systemic treatment, site of lung metastases, and electrode type) were utilised to construct the clinical model. The Habitat signature combined with the Peri-5 signature achieved the best radiomics signature, with AUC values of 0.994 and 0.870 in the training cohort and test cohort, respectively. The nomogram performed well on both data cohorts (0.997 and 0.909, respectively) and outperformed the clinical model and DL signature.
**Conclusion:** The proposed CT-based DL radiomics nomogram can offer precise predictions and valuable assistance to physicians in developing personalised treatment strategies.
**Limitations:** It was a single-centre retrospective study with a limited sample size.
**Ethics committee approval:** This study was performed in line with the principles of the Declaration of Helsinki. Approval was granted by the Ethics Committee of Fudan University Shanghai Cancer Center (Approval Number: 1612167-18).
**Funding for this study:** This work was supported by the National Natural Science Foundation of China (No. 82272095).
**Author Disclosures:**
Wentao Li: Author: none
Haozhe Huang: Nothing to disclose

### RPS 1009-10

**High-power (150W) microwave ablation (MWA) is as safe and effective as 100W MWA for the treatment of lung lesions**

*L. V. Sciacqua*, A. M. Ierardi, S. Carriero, C. Lanza, G. V. D. Amato, G. Carrafiello; Milan/IT
*(lucilla.sciacqua@unimi.it)*

**Purpose:** The objective of this study was to compare the safety and efficacy of high-power (150W) vs. standard (100W) microwave ablation (MWA) of lung lesions.

**Methods or Backround:** We retrospectively evaluated 28 consecutive patients (age 43-84) treated with standard (100W, 16/28 patients) or high-power (150W, 12/28 patients) MWA at our Institution from January 2021 to July 2023. All histologic specimens were centrally revised at our institution, with 18/28 lesions being classified as primary lung malignancies and 10/28 as metastases (seven from colorectal adenocarcinoma, one from laryngeal cancer and two from hepatocellular carcinoma). Six patients received MWA after failure of first-line treatments, including surgery and radiotherapy. Tumour response was evaluated three months after treatment with computed tomography (CT).
**Results or Findings:** Mean lesions' size was 19 mm (range 7-40 mm) with an average ablation time of 3'42'' (range 1'30''-6'30''). Technical success was defined as the adequate positioning of the treatment antenna under Cone Beam CT (CBCT) guidance and was achieved in all patients. Complete ablation of the target lesion was confirmed in all patients at follow-up CT evaluation. Peri-procedural complications (i.e., pneumothorax flap) were reported in five patients, all belonging to the 100W arm, and were all resolved by day 6 post-treatment. Four patients, equally distributed into the two arms, demonstrated modest pleural effusion at follow-up imaging.
**Conclusion:** 150W MWA demonstrated overlapping safety and efficacy when compared to 100W MWA, but its shorter ablation time can allow for sparing more healthy tissue. Our results, if confirmed on larger cohorts and in prospective trials, might further support the role of high-power 150W MWA for the treatment of lung lesions.
**Limitations:** The main limitation of our study is represented by the small size of the treatment cohort and by its retrospective nature.
**Ethics committee approval:** The study is retrospective.
**Funding for this study:** No funding was received for this study.
**Author Disclosures:**
Lucilla Violetta Sciacqua: Nothing to disclose
Serena Carriero: Nothing to disclose
Gaetano Valerio Davide Amato: Nothing to disclose
Anna Maria Ierardi: Nothing to disclose
Carolina Lanza: Nothing to disclose
Gianpaolo Carrafiello: Nothing to disclose

### RPS 1009-11

### Electrochemotherapy for the treatment of metastatic bone disease

*N. Papalexis*, G. Peta, M. Carta, S. Quarchioni, E. Costantino, L. Campanacci, M. Miceli, G. Facchini; Bologna/IT
*(nicolaspapalexis@gmail.com)*

**Purpose:** The primary objective of this study was to assess the effectiveness and safety of electrochemotherapy (ECT) using bleomycin for the treatment of bone metastases, with a focus on evaluating radiological responses, pain reduction, and improvements in the patients' quality of life.
**Methods or Backround:** Between 2009 and 2022, we enrolled 106 cancer patients (mean age of 61 years) who had bone metastases. Among these patients, 64 had lesions in the upper limbs, 18 in the lower limbs, 29 in the pelvis, and 10 in the thorax and vertebrae. The treatment involved ECT, which utilises electric pulses to enhance the local delivery of bleomycin into the tumour cells. A bolus injection of bleomycin (15 mg/m2) was administered intravenously, followed by the application of 8 electric pulses at 1000V/cm between each pair of electrodes (performed eight minutes after the drug injection) using the Cliniporator VITAE (Igea S.p.A., Carpi, Italy) and specialised electrodes. The procedures were conducted with the guidance of CT or fluoroscopy.
**Results or Findings:** Of the patients, 94 received a single course of ECT, 11 underwent two courses, three received three, and two had four courses. All eligible patients reported a reduction in pain ranging from 30% to 100%, with an average pain relief of 57% following local ECT treatment. Furthermore, 74% of assessable patients experienced a pain reduction of over 50% after undergoing ECT treatment. No general complications related to the treatment were observed, although two cases of skin necrosis were documented as local complications.
**Conclusion:** Our data strongly support the feasibility and safety of employing ECT in patients with bone metastatic lesions. Patients exhibited positive radiological responses and reported enhancements in their daily activities and overall health.
**Limitations:** Retrospective study, lack of control group.
**Ethics committee approval:** This study was approved by the ethics committee and data protection authority by the Institutional Review Board for retrospective studies.
**Funding for this study:** No funding was received for this study.
**Author Disclosures:**
Marco Miceli: Nothing to disclose
Michela Carta: Nothing to disclose
Errani Costantino: Nothing to disclose
Laura Campanacci: Nothing to disclose
Giancarlo Facchini: Nothing to disclose
Giuliano Peta: Nothing to disclose
Simone Quarchioni: Nothing to disclose
Nicolas Papalexis: Nothing to disclose

### RPS 1009-12

### Initial experience with Bleomycin Electroporation in locally advanced and metastatic tumour spread

*I. Bayram*, R. Brill, E. Elolf, M. Guntau, M. Damm, W. A. A. Wohlgemuth, S. Schob, C. Loberg; Halle a.d. Saale/DE
*(dr.irembayram@gmail.com)*

**Purpose:** Malignant primary tumours or metastases in superficial localisation often present with a variety of local complications, for example pain, ulceration, necrosis with infections and bleeding. The management of a malignant wound is always complex and at some point may become futile. Bleomycin Electroporation (BEST) is a proven treatment approach for skin melanoma and superficial vascular malformations. Based on the efficacy in context of the aforementioned, we evaluated if local tumour complications, most importantly haemorrhage, can be controlled by BEST.
**Methods or Backround:** 5 patients (3 female, 2 male) with locally aggressive tumours were treated with BEST. Tumour entities included breast cancer, giant cell sarcoma, pleomorphic dermal sarcoma and a vertebral metastasis of a thoracic vertebral body from gastric cancer. All tumours showed significant bleeding accompanied by ulcerations, superinfection and necrosis. BEST was performed by systemic application of 25mg Bleomycin i.v. together with current applied via hexagonal electrodes, needle length 1.5 – 2.5cm in 14 – 58 applications.
**Results or Findings:** Bleeding was immediately stopped in all cases. Pre- and postinterventional thermography revealed significant downcooling after the procedure caused by the vascular lock effect.
**Conclusion:** Bleomycin electroporation is safe and effective in treatment of local tumour complications.
**Limitations:** No limitations were identified.
**Ethics committee approval:** The study received institutional review board approval and written informed consent was obtained from all participants.
**Funding for this study:** No funding was provided for this study.
**Author Disclosures:**
Irem Bayram: Nothing to disclose
Moritz Guntau: Nothing to disclose
Marko Damm: Nothing to disclose
Erck Elolf: Nothing to disclose
Richard Brill: Nothing to disclose
Stefan Schob: Nothing to disclose
Christina Loberg: Nothing to disclose
Walter A. A. Wohlgemuth: Nothing to disclose

### RPS 1009-13

### Evone® flow-controlled ventilation during percutaneous interventional radiology procedures: a clinical feasibility and safety assessment

*G. Xhepa*, A. Silvani, S. Cappio, G. Raia, A. Leoncini, F. Pedersoli, A. Saportio, S. Rizzo, F. Del Grande; Lugano/CH
*(genti_xh@hotmail.com)*

**Purpose:** Evone® is a flow-controlled ventilation (FCV) device that actively removes air from the lungs using a small-bore cuffed tube (Tritube). This constant flow ventilation enables protective patient ventilation with minimal diaphragm movement and smooth motion of abdominal organs. The objective of the study is to evaluate feasibility and safety of Evone® FCV in percutaneous interventional radiology procedures.
**Methods or Backround:** Patients who underwent percutaneous procedures between 01/01/2022 and 30/04/2023. Exclusion criteria were age below 18 as well as contraindications for percutaneous procedures. MDMT consensus and written consent was obtained before the procedure. The primary endpoints were safety, feasibility, and technical success, defined as the biopsy/ablation of the target lesion. Secondary endpoints were procedure time, patient dose and hospitalisation. Adverse Events (AEs) were classified according to the validated 2023 SIR Classification.
**Results or Findings:** During the period under review 40 percutaneous procedures were performed in 20 patients (13 males) under general anaesthesia with Evone® system in 22 treatment sessions. Median age was 66.2 years (range 47–83 years). Of the lesions found, 14 were in the kidney (9 left, 5 right), 7 were hepatic and 3 pulmonary. The median number of procedures per patient was 2 and there were 1.7 procedures per lesion (24 lesions). The procedures performed comprised 20 biopsies, 16 Microwave Ablations (MWA), 2 Radiofrequency Ablations (RFA) and 2 Cryoablations. The technical success rate was 100%. The median time expended per session was 83 minutes. The median DLP per session was 2294.1 mGy/cm. The AEs rate per procedure was 15%. The rate of mild AEs was 7.5% (1 local pain, 2 PNX not treated). The rate of moderate AEs was 7.5% (2 urinary tract infection, 1 acute urinary retention, both successfully treated). Three patients had day hospital procedures. The median duration of hospitalisation per session was 64.4 hours, with 1 patient being hospitalised for 360 hours.
**Conclusion:** The Evone® FCV system improves conditions in IR procedures while providing safe ventilation.
**Limitations:** The small number of patients as well as the heterogeneity of treated lesions and image guiding procedures were identified as limitations.

**Ethics committee approval:** Informed consent froms were signed by all patients.
**Funding for this study:** No funding was received for this study.
**Author Disclosures:**
Giorgio Raia: Nothing to disclose
Genti Xhepa: Nothing to disclose
Andrea Leoncini: Nothing to disclose
Andrea Saportio: Nothing to disclose
Stefania Rizzo: Nothing to disclose
Filippo Del Grande: Nothing to disclose
Federico Pedersoli: Nothing to disclose
Alessio Silvani: Nothing to disclose
Stefano Cappio: Nothing to disclose

**16:00-17:30 Research Stage 1**

### Research Presentation Session: Hybrid, Molecular and Translational Imaging

### RPS 1106

### Advances in hybrid, molecular and translational imaging

**Moderator**
A. Esposito; Milan/IT
*(esposito.antonio@hsr.it)*
**Author Disclosures:**
Antonio Esposito: Advisory Board: GE healthcare; Grant Recipient: Educational Grant by Siemens, Bayer and MS

### RPS 1106-2

**Assessment of hypoxia-induced neoangiogenesis in breast cancer xenografts using non-contrast-enhanced multiparametric MRI and fluorescent multiplexed immunohistochemistry**
*S. J. Bartsch*[1], K. Brožová[1], V. Ehret[1], J. Friske[1], L. Kenner[1], K. Kratochwill[1], D. Laimer-Gruber[1], T. H. Helbich[1], K. Pinker-Domenig[2]; [1]Vienna/AT, [2]New York, NY/US

**Purpose:** Tumour neoangiogenesis is an important hallmark of cancer progression, triggered by alternating selective pressures from the hypoxic tumour microenvironment. Multiparametric MRI combining blood oxygen level dependent (BOLD) MRI, depicting blood oxygen saturation, and intravoxel incoherent motion (IVIM) MRI, capturing intravascular and extravascular diffusion, allows insights into tumour progression, capturing tumour oxygenation and neovascularisation simultaneously. The combination of BOLD- and IVIM-MRI may provide non-invasive, non-contrast-enhanced imaging biomarkers of neoangiogenesis for the discrimination of breast cancer (BC) molecular subtypes. We compare our findings to fluorescent multiplexed immunohistochemistry (MP-IHC).
**Methods or Backround:** An in-vivo study on 36 female athymic nude mice, which were inoculated with luminal A, Her2+ and triple negative BC cells, was conducted. MRI was performed using a 9.4T Bruker BioSpec 94/30USR system. Following IVIM-MR imaging, tumour oxygenation was measured at 21% oxygen and 100% oxygen for BOLD measurements. Tumours were resected for MP-IHC staining of CD-31, PDGFR-beta and Hif1-alpha.
**Results or Findings:** Hyperoxic BOLD MRI discriminates luminal A from Her2+ and triple negative BCs, while the IVIM-derived parameter fIVIM allows the distinction of luminal A and Her2+ from triple negative BCs. A principal component analysis (PCA) of BOLD and IVIM-MRI derived parameters reveals differences between triple negative and other BC molecular subtypes. In a PCA of MP-IHC stains, luminal A BCs clustered separately from other BC molecular subtypes.
**Conclusion:** Our multivariate analysis of BOLD and IVIM parameters highlights how oxygen delivery is constricted and neoangiogenesis is increased in triple negative BCs. The combined interpretation with MP-IHC provides a holistic view on hypoxia-induced neoangiogenesis in BCs. We conclude that non-contrast-enhanced mpMRI using BOLD and IVIM imaging provides promising imaging biomarkers for an assessment of hypoxia-induced neoangiogenesis in three BC molecular subtypes.
**Limitations:** No limitations were identified.
**Ethics committee approval:** This preclinical study was approved by the Austrian Federal Ministry of Education, Science and Research (project number BMFWF-66.009/0284-WF/V/3b/2017).
**Funding for this study:** Funding was provided by the Vienna Science and Technology Fund (WWTF), grant no. LS19-018.
**Author Disclosures:**
Silvester Julian Bartsch: Nothing to disclose
Viktoria Ehret: Nothing to disclose
Klára Brožová: Nothing to disclose
Katja Pinker-Domenig: Nothing to disclose
Thomas H. Helbich: Nothing to disclose
Daniela Laimer-Gruber: Nothing to disclose
Lukas Kenner: Other: micorOne CBmed
Klaus Kratochwill: Nothing to disclose
Joachim Friske: Nothing to disclose

### RPS 1106-3

**Avoiding anaesthesia in oncologic paediatric patients using short acquisition times with FDG total-body PET/CT**
*C. Mingels*, B. A. Spencer, H. Nalbant, M. Rokni, Y. G. Abdelhafez, F. Sen, R. Badawi, L. Nardo; Sacramento, CA/US

**Purpose:** Given the increased signal collection efficiency, Total-Body (TB) PET/CT allows for protocol flexibility including acquisition time (AT). This study aimed to define the shortest AT with maintained diagnostic quality to avoid or reduce anaesthesia time in children.
**Methods or Backround:** Twelve oncologic paediatric patients were injected with 4.24 MBq/kg 18F-FDG and scanned in list mode for 20 minutes on uEXPLORER TB-PET/CT after 120 min uptake time. 300s, 150s, 75s, and 37s data sets were extracted and reconstructed with varying iterations (4-6) and filters (4-6mm FWHM Gaussian or Metz) and then compared to the full data set reconstructed with 4 iterations (20 subsets), 256x256 matrix, no smoothing. All reconstructions were simultaneously displayed and independently rated from 1 (best) to 4 (worst) for interpretation/diagnosis by three nuclear medicine physicians. Additionally, signal-to-noise ratio (SNR), tumour-to-background ratio (TBR) and contrast-to-noise ratio (CNR) were calculated using a 30mm diameter sphere in the right liver lobe and tumour lesions segmented using a 40%-iso-contour. Data sets were compared using Student's t-test to the reference data set for changes in semi-quantitative measurements.
**Results or Findings:** Readers selected the reference reconstruction parameters for the 300s and 150s data sets but preferred additional smoothing for shorter data sets. Liver, blood-pool and bone SNR decreased with shortened AT. TBR and CNR were not significantly different between 20 min, 300 s and 150 s. Compared to 20 min TBR and CNR reduced significantly in 75 s and 37 s reconstructions (TBR 20 min: 3.64; 75 s: 2.40, $p < 0.05$ and 37 s: 2.09, $p < 0.05$; CNR 20 min: 11.29; 75 s: 2.36, $p=0.01$ and 37 s: 1.77, $p=0.01$).
**Conclusion:** 150 s paediatric acquisitions on TB-PET/CT obtained after 120 minutes uptake time show no significant semi-quantitative image quality reduction compared to 20 min scans and may decrease or avoid the need of anaesthesia.
**Limitations:** The study featured a small cohort.
**Ethics committee approval:** The evaluation was approved by the hospital committee.
**Funding for this study:** Funding was received from the US National Institutes of Health R01-CA249422;R01-CA206187.
**Author Disclosures:**
Fatma Sen: Nothing to disclose
Mehrad Rokni: Nothing to disclose
Hande Nalbant: Nothing to disclose
Benjamin A. Spencer: Nothing to disclose
Clemens Mingels: Nothing to disclose
Ramsey Badawi: Nothing to disclose
Lorenzo Nardo: Nothing to disclose
Yasser Gaber Abdelhafez: Nothing to disclose

### RPS 1106-4

**Characterisation of international metabolic prognostic index (IMPI) and in its components in CAR T-cell treatment of lymphoma**
*M. Winkelmann*, V. Blumenberg, K. Rejeski, V. Bücklein, F. Dekorsy, J. Ricke, M. Subklewe, W. G. Kunz; Munich/DE
*(michael.winkelmann@med.uni-muenchen.de)*

**Purpose:** Chimeric antigen receptor T-cell therapy (CART) is approved for relapsed or refractory (r/r) non-Hodgkin lymphoma (NHL). Recently, the international prognostic metabolic index (IMPI), which is composed of metabolic tumour volume (MTV), age, and Ann Arbor stage, was introduced. As higher patient age is a positive prognostic factor and higher MTV is a negative prognostic factor in the context of CART, we compared the predictive value of each component of the IMPI and its prognostic value for progression-free survival (PFS) and overall survival (OS).
**Methods or Backround:** Consecutive r/r NHL patients and 18F-FDG PET/CT imaging at baseline were selected. Ann Arbor stage and patient age were calculated at the time of lymphodepletion. MTV was calculated with an absolute SUV cut-off of 4.

**Results or Findings:** 43 patients were included (37% female, 63% male) with a median age of 66 years and baseline MTV of 276 cm2. Ann Arbor stage was 1 in four patients, 2 in 11 patients, 3 in eight patients, and 4 in patients. Splitting patients by median IMPI showed minor differences in median PFS and OS, which were statistically not significant. Dichotomisation by median MTV alone revealed a larger, statistically significant difference in median PFS and a larger nonsignificant difference in OS. Patients with an older age demonstrated a slightly longer PFS and OS, whereas there was no relevant difference between the two Ann Arbor risk groups.
**Conclusion:** For r/r NHL patients undergoing CAR T-cell therapy, IMPI represents a promising tool for risk assessment. However, in our study MTV alone was superior in PFS and OS stratification. A larger cohort should be used to assess whether MTV alone or in combination with measurement of tumour dissemination can improve survival prediction.
**Limitations:** This was a single centre study with a limited patient population.
**Ethics committee approval:** All medical records and imaging studies were reviewed with the approval of the LMU Munich Institutional Review Board (LMU Ethics Committee, project number 19-817). Informed consent was obtained from all individual participants included in the study.
**Funding for this study:** Funding for this study was received from Bayrisches Zentrum für Krebsforschung (BZKF) as well as Förderung für Forschung und Lehre der LMU (FöFoLe).
**Author Disclosures:**
Marion Subklewe: Speaker: Amgen, Astra Zeneca, Gilead, Janssen, Pfizer, Roche, Takeda, GSK, BMS/Celgene, Novartis; Research/Grant Support: Amgen, BMS/Celgene, Gilead, Morphosys, Novartis, Roche, Seattle Genetics, Takeda; Consultant: Aven Cell, CDR-Life, Ichnos Sciences, Incyte Biosciences, Molecular Partners, Novartis, Pfizer, Takeda
Viktoria Blumenberg: Research/Grant Support: Amgen, Kite/Gilead, Celgene/BMS
Michael Winkelmann: Nothing to disclose
Kai Rejeski: Research/Grant Support: Kite/Gilead, Novartis
Wolfgang Gerhard Kunz: Advisory Board: Bristol Myers Squibb
Veit Bücklein: Research/Grant Support: Pfizer, Kite/Gilead, Novartis, Celgene/BMS
Franziska Dekorsy: Nothing to disclose
Jens Ricke: Nothing to disclose

### RPS 1106-5

**First report of 23Na-MRI in kidney cancer to detect differential sodium-accumulation in benign vs malignant tumours**

*I. Horvat Menih*, J. Birchall, M. McLean, M. Zamora-Morales, A. Bebb, J. Kaggie, A. Warren, G. Stewart, F. A. Gallagher; Cambridge/UK
*(ih357@cam.ac.uk)*

**Purpose:** Renal cell carcinoma (RCC) poses a significant clinical challenge. Accurate early detection is hindered by limitations in current diagnostic methods. This study investigates the potential of 23Na-MRI to noninvasively differentiate kidney tumour subtypes, shedding light on sodium regulation in renal cancer.
**Methods or Backround:** Eight patients with kidney tumours underwent 23Na-MRI. Total sodium concentration (TSC) was calculated based on an 80mM 23Na-phantom. Regions of interest were drawn on TSC maps. Histopathology was determined on renal mass biopsy or surgical specimen. Immunohistochemical markers (CD31, Ki67) were assessed in a separate patient cohort. RNAseq counts for NHE3 and Na+/K+-ATPase were obtained from online databases. Statistical analyses were performed in Graphpad Prism.
**Results or Findings:** 7 renal oncocytomas (RO), 2 chromophobe RCCs (chRCC) and 2 clear cell RCCs (ccRCC) were identified in the 8 participants. 4 participants are undergoing active surveillance, and 4 underwent nephrectomy. TSC was highest in ccRCC, and lowest in chRCC, while normal tissue and RO had comparable values. The main Na+ transporter in the kidney, NHE3, was highest in ccRCC and downregulated in chRCC. By contrast, ATPase was highest in chRCC, followed by ccRCC and RO. As expected, the ccRCC as the most aggressive kidney tumour subtype, exhibited highest vascularity (CD31) and highest proliferation rate (Ki67), which may have further contributed to the increased sodium signal detected on imaging.
**Conclusion:** We have shown feasibility for 23Na-MRI to noninvasively detect sodium concentration differences across kidney tumours, and associated findings with molecular markers of sodium handling. This prepares the ground for future research and shows potential to improve the clinical management of kidney cancer.
**Limitations:** It was not possible to quantify intracellular/extracellular sodium. There was no separation of cortex and medulla, and no correction for cystic/necrotic areas.
**Ethics committee approval:** The investigation of the differential biology of benign and malignant renal masses using advanced magnetic resonance imaging techniques (IBM-Renal) was approved by the Cambridge research ethics committee, no.: 22/EE/0136.
**Funding for this study:** This work is supported by the Cancer Research UK Cambridge Centre (RQAG/119) as well as the NIHR Cambridge Biomedical Research Centre.
**Author Disclosures:**
Grant Stewart: Nothing to disclose
Jonathan Birchall: Nothing to disclose
Ines Horvat Menih: Nothing to disclose
Joshua Kaggie: Nothing to disclose
Mary McLean: Nothing to disclose
Anne Warren: Nothing to disclose
Alice Bebb: Nothing to disclose
Ferdia Aidan Gallagher: Nothing to disclose
Maria Zamora-Morales: Nothing to disclose

### RPS 1106-6

**Validation of the standardisation framework SSTR-RADS 1.0 for neuroendocrine tumours using the novel SSTR-targeting peptide [18F]SiTATE**

*R. Ebner*, A-K. Lohse, M. P. Fabritius, J. Rübenthaler, P. Bartenstein, J. Ricke, F. Grawe; Munich/DE
*(ricarda.ebner@med.uni-muenchen.de)*

**Purpose:** Somatostatin receptor positron emission tomography/computed tomography (SSTR-PET/CT) using [68Ga]-labelled tracers is a widely used imaging modality for neuroendocrine tumours (NET). Recently, [18F]SiTATE has shown great potential due to its favourable clinical characteristics. We aimed to evaluate the reproducibility of SSTR-RADS 1.0 for structured interpretation and treatment planning of NETs using [18F]SiTATE.
**Methods or Backround:** Four readers assessed [18F]SiTATE-PET/CT of 95 patients according to the SSTR-RADS 1.0 criteria at two different time points. Each reader selected and evaluated up to 5 target lesions per scan. Overall scan score and the decision on peptide receptor radionuclide therapy (PRRT) were considered. Inter- and intrareader agreement was determined using the intraclass correlation coefficient (ICC).
**Results or Findings:** Interreader agreement for identical target lesions (ICC≥85%), overall scan score (ICC≥90%) and decision to recommend PRRT (ICC≥85%) showed excellent agreement. However, significant differences were observed in recommending PRRT within ERs (p =0.020) and IRs (p =0.004). Compartment-based analysis demonstrated good to excellent interreader agreement for most organs (ICC≥74%), except for lymph nodes (ICC≥52%).
**Conclusion:** SSTR-RADS 1.0 represents an accurate and reproducible framework system for stratifying [18F]SiTATE-PET/CTs as an alternative for [68Ga]-labelled PET/CTs in NET-imaging. However, excellent interreader agreement on the overall scan score and the decision for PRRT was observed, there were variations in PRRT recommendations, highlighting the complexity of such decisions, suggesting the need for multidisciplinary input. Compartment-based assessments demonstrated excellent interreader agreement for the liver, soft tissue, and skeleton, with varying agreement for lymph nodes, emphasising the importance of functional imaging for small lesions.
**Limitations:** The absence of histopathological confirmation for each target lesion and blinding of readers to patients' clinical status may have influenced inter- and intrareader agreement, indicating the need to integrate clinical information into the interpretation of [18F]SiTATE-PET/CT scans.
**Ethics committee approval:** The analysis of the data was approved by the institutional ethics board of LMU Munich (IRB 20-1077).
**Funding for this study:** No funding was received for this study.
**Author Disclosures:**
Matthias Philipp Fabritius: Nothing to disclose
Freba Grawe: Nothing to disclose
Ann-Kathrin Lohse: Nothing to disclose
Peter Bartenstein: Nothing to disclose
Ricarda Ebner: Nothing to disclose
Johannes Rübenthaler: Nothing to disclose
Jens Ricke: Nothing to disclose

### RPS 1106-7

**First application of novel human granzyme B imaging agent in a humanised melanoma mouse model treated with immune checkpoint inhibitor therapy**

*P. Summer*[1], N. Gallon[1], N. Bulmer[1], S. Prabhu[1], P. Heidari[2], U. Mahmood[2]; [1]Charlestown, MA/US, [2]Boston, MA/US
*(priska.summer@meduniwien.ac.at)*

**Purpose:** This study aims to evaluate whether a novel human granzyme B PET imaging agent CYT-200 (Cytosite) labelled with 68Ga can be used as a reliable, non-invasive biomarker for detecting intratumoural granzyme B levels, while evaluating the efficacy of immune checkpoint inhibitor therapy in vivo.
**Methods or Backround:** Melanoma-bearing humanised NSG mice were randomly assigned to receive either anti-PD1/CTLA4 combined, anti-PD1 alone or saline (controls) 9, 12 and 15 days after tumour implantation. PET imaging was performed prior to treatment initiation (baseline), such as on days 4, 7 and 14 after the first treatment dose. Tumour-bearing mice were injected

Thursday with 68Ga-CYT-200 labelled 1h prior to PET/CT imaging. Intra-tumoural T-cell activity was determined by tumour to blood ratio (TBR), calculated by the standard uptake values (SUVs) of the tumours normalised against the heart blood pool. Treatment response was assessed by tumour growth measurements over 35 days after the first treatment dose.
**Results or Findings:** PET imaging on day 4 after treatment initiation showed the highest TBR compared to the baseline (3.08±0.98 vs. 1.22±0.48) with a gradual decrease of the TBRs on day 7 (2.76±1.85), and 14 (1.91±0.23). Anti-PD1/CTLA4 decreased the tumour size on days 4 and 7 after treatment initiation (336±38 mm3 vs.154±31 mm3 and 131±129 mm3, respectively). However, tumour growth recurred up to the final time point, 12 days after treatment initiation (181±112 mm3), correlating with the T-cell activity measured through in vivo PET imaging using CYT-200.
**Conclusion:** CYT-200 labelled with 68Ga can detect intra-tumoural T-cell activity associated with tumour killing following immune checkpoint inhibitor therapy in a humanised mouse model for melanoma.
**Limitations:** The study may have a limited number of mice, which could affect the generalisability and statistical power of the findings.
**Ethics committee approval:** All animal studies were approved and conducted according to the IACUC guidelines.
**Funding for this study:** Funding for this study was received by the National Institutes of Health.
**Author Disclosures:**
Pedram Heidari: Nothing to disclose
Priska Summer: Nothing to disclose
Niklas Bulmer: Nothing to disclose
Umar Mahmood: Consultant: CytoSite Biopharma
Naomi Gallon: Nothing to disclose
Suma Prabhu: Nothing to disclose

### RPS 1106-8

### Assessment of CAR T-cell therapy response in mice with melanoma using Granzyme B PET imaging

*P. Summer*[1], N. Bulmer[1], N. Gallon[1], S. Prabhu[1], P. Heidari[2], U. Mahmood[2]; [1]Charlestown, MA/US, [2]Boston, MA/US
*(priska.summer@meduniwien.ac.at)*

**Purpose:** The purpose of this study is to determine whether granzyme B PET imaging can predict treatment response to CAR T-cell therapy while evaluating the efficacy of CAR T-cell therapy for melanoma.
**Methods or Backround:** Chimeric antigen receptor (CAR) T-cell therapy is a novel cell-based immunotherapy that urgently requires a reliable tool to determine patient response rapidly and accurately. Four days after melanoma cell implantation, tumour-bearing NSG mice received either 2x10^6 CAR T-cells (n=9) or vehicle (controls; n=8) intravenously. Treatment response was evaluated by tumour growth measurements up to 35 days post-treatment. A human granzyme-B-specific agent, CYT-200, labelled with 68Ga was used for PET imaging to assess intra-tumoural T-cell activity on days 2, 7 and 14 after treatment. Further, liver and colon uptake were compared between the groups to evaluate granzyme B expression within different organs.
**Results or Findings:** Tumoural 68Ga-hGZP uptake was significantly greater in the CAR T group 2 (3.1 ± 1.2 vs. 1.1 ± 0.4, P = 0.0017) and 7 (2.0 ± 1.1 vs. 1.1 ± 0.1, P = 0.0111) days after treatment, much earlier than when CAR T treated mice first presented with significantly lower tumour volumes eleven days after tumour implantation (61.8 mm3 ± 8.7 vs. 287.1 mm3 ± 157.6, P = 0.0455).
**Conclusion:** Quantitative in vivo imaging of intratumoural granzyme B provides early prediction of treatment response as early as 2 days after treatment. With the recent translation of granzyme B PET imaging to cancer patients treated with checkpoint-inhibitors, these findings may help inform CAR T response assessment in patients.
**Limitations:** The study may have a limited number of mice, which could affect the generalisability and statistical power of the findings.
**Ethics committee approval:** IACUC approval was obtained.
**Funding for this study:** Funding for this study was received from the National Insitutes of Health.
**Author Disclosures:**
Pedram Heidari: Nothing to disclose
Priska Summer: Nothing to disclose
Niklas Bulmer: Nothing to disclose
Umar Mahmood: Consultant: CytoSite Biopharma
Naomi Gallon: Nothing to disclose
Suma Prabhu: Nothing to disclose

### RPS 1106-9

### Are we ready for novel radionuclides in nuclear medicine in Europe: PRISMAP survey perspective

*M. Radzina*[1], L. Saule[1], E. Mamis[1], E. Pajuste[1], T. E. Coccolios[2], T. Stora[3]; [1]Riga/LV, [2]Brussels/BE, [3]Geneva/CH

**Purpose:** The study aimed to understand the current status and future perspectives of novel radionuclides in Europe, through a survey. In order to support the ongoing research across Europe and to facilitate access to novel radionuclides, the PRISMAP consortium (European medical radionuclides programme) was established.
**Methods or Backround:** A consortium questionnaire was disseminated among 30 countries. We received 114 respondents through the PRISMAP consortium and user community, professional associations and preclinical/clinical end users in Europe and the current status of clinical end users in nuclear medicine was identified.
**Results or Findings:** A total of 40 preclinical/clinical users institutions took part in the survey. Clinical end users currently use the following radionuclides in their studies: 177 Lu, 68 Ga, 111 In, 90 Y, other alpha emitters, 225 Ac, 64 Cu, and Terbium isotopes. Radionuclides that would be of interest for users within the next 2 to 5 years are 64 Cu, Terbium radionuclide “family” and alpha emitters, such as 225 Ac. Active industry involvement in joint outreach activities will provide access to new radionuclides and new purity grades for medical research. This will enhance clarity and regulatory procedures to foster research with radiopharmaceuticals and improve the delivered radionuclide data and regulation, along with biomedical research capacity.
**Conclusion:** The current perspective shows that nuclear medicine specialists/clinical end users from broad parts of Europe are not only interested in new radionuclides for diagnostics, but also in therapy and technology advancements that confirm their interest in development. This study was preliminary and should be extended outside the PRISMAP consortium.
**Limitations:** Not all European countries were covered - the majority of responses came from Western Europe, most notably the Benelux, France and Italy. More emphasis is needed to reach respondents from South-Eastern Europe.
**Ethics committee approval:** Not applicable.
**Funding for this study:** This project has received funding from the European Union’s Horizon 2020 research and innovation programme under grant agreement No 101008571 (PRISMAP). This document reflects only the view of the author(s). The funding agenc(y/ies) is/are not responsible for any use that may be made of the information it contains.
**Author Disclosures:**
Elina Pajuste: Nothing to disclose
Edgars Mamis: Nothing to disclose
Thierry Stora: Nothing to disclose
Maija Radzina: Nothing to disclose
Laura Saule: Nothing to disclose
Thomas Elias Coccolios: Nothing to disclose

### RPS 1106-10

### Micro-CT vs conventional CT: preliminary results of comparing radiomic features in vivo and ex vivo in lung cancer

*L. Brizzi*, L. Preda, C. Bortolotto, D. Gioacchino; Pavia/IT
*(md.brizzileonardo@gmail.com)*

**Purpose:** Radiomic analysis holds the potential to revolutionise lung cancer management by providing nuanced insights into tumour characteristics, ultimately aiding in early detection and personalised treatment, thus reducing mortality rates significantly. This study aims to investigate correlations between radiomic features in images obtained both from conventional spiral-CT and micro-CT scans of post-surgical anatomical specimens extracted from lung cancer patients.
**Methods or Backround:** Three patients with lung cancer (pT2-3N0-1M0) underwent conventional in vivo spiral-CT before surgery. After surgery, post-surgical specimens were scanned using both spiral-CT (slice 0.3 mm) and micro-CT (slice thickness 40 µm). Tumour lesions from in vivo CT and within the specimens were segmented manually and semi-automatically using ITK-SNAP. A comparative radiomic analysis using Pyradiomics software was conducted, comparing features (shape and II order) from both image modalities before and after surgery, using ICC for assessing agreement.
**Results or Findings:** Concordance statistics for the 107 extracted radiomic features between image modalities revealed the following percentages of agreement: 79% when comparing micro-CT scans and conventional CT scans of the anatomical specimens, 74% when comparing micro-CT scans of the anatomical specimens and conventional in vivo CT scans, and 95% when comparing conventional CT images of the anatomical specimens with conventional in vivo CT scans. Among all the extracted features the highest degree of concordance was found in GLCM (Gray Level Co-occurrence Matrix; variation between 0.06-7.97%) and shape-based features (0.01-0.92%).

**Conclusion:** These preliminary findings indicate good to excellent concordance of radiomic features extracted from micro-CT and conventional CT scans, both in vivo and ex vivo. These results establish micro-CT as a possible tool for investigating the biological basis of radiomic features. In clinical practice, the diagnostic potential of micro-CT could be expanded to the point of performing "virtual biopsies".
**Limitations:** The possible limitations are the small sample size, and the feasibility of the study.
**Ethics committee approval:** 1. Patients' consent was acquired by signing standard forms according to hospital protocol.
**Funding for this study:** Funding was received from Ricerca Corrente Founding.
**Author Disclosures:**
Leonardo Brizzi: Nothing to disclose
Lorenzo Preda: Nothing to disclose
Chandra Bortolotto: Nothing to disclose
D'Ambrosio Gioacchino: Nothing to disclose

### RPS 1106-11

**Preoperative FDG uptake is associated with histopathological response in patients with operable NSCLC patients receiving neo-adjuvant immuno-chemotherapy: a prospective single-centre study**
*D. Kifjak*, M. J. Hochmair, J. Klinger, K. Sinn, A. Hoda, R-I. Milos, A. Haug, H. Prosch, L. Beer; Vienna/AT

**Purpose:** The aim of this study was to evaluate the association between 18-FDG-PET CT quantitative imaging markers and histological responses from patients with operable non-small cell lung cancer (NSCLC) treated with neo-adjuvant immuno-chemotherapy.
**Methods or Backround:** This prospective, single-centre study included 22 patients (7 male, 15 female) with NSCLC who were treated with neo-adjuvant immuno-chemotherapy and underwent preoperative 18F-FDG-PET-CT. We semi-automatically extracted the standardised uptake value (SUVmax) and metabolic tumour volume (MTV). The histological results were retrieved from patients' records. Patients were assigned to either complete pathologic response (cPR) or non-cPR groups. A Mann-Whitney-U-Test was calculated to determine if there were differences between SUVmax, MTV and histologic response.
**Results or Findings:** Eleven patients had a cPR, while eleven had a non-cPR. The SUVmax was lower in patients with cPR compared to those without cPR (median: 4.69 (IQR 5.7) vs. 9.9 (IQR 19.3)), p=0.049. The MTV was not significantly different between the two groups (median: 16.9 (IQR) 71.4, 29.2 (IQR 110.7)) p=0.0622.
**Conclusion:** Presurgical SUVmax was associated with cPR in NSCLC patients receiving immuno-chemotherapy. However, cPR can also be observed in patients with high SUVmax values.
**Limitations:** This study is a single-centre study with a small sample size.
**Ethics committee approval:** EC number 1521/2015
**Funding for this study:** Funding was received from the Austrian Federal Ministry for Digital and Economic Affairs, the National Foundation for Research, Technology and Development and the Christian Doppler Research Association.
**Author Disclosures:**
Katharina Sinn: Nothing to disclose
Maximilian Johannes Hochmair: Nothing to disclose
Alexander Haug: Nothing to disclose
Helmut Prosch: Nothing to disclose
Julian Klinger: Nothing to disclose
Lucian Beer: Nothing to disclose
Daria Kifjak: Nothing to disclose
Ruxandra-Iulia Milos: Nothing to disclose
Alireza Hoda: Nothing to disclose

### RPS 1106-12

**Size matters when distinguishing ATTR and AL cardiac amyloidosis on PiB PET/CT**
*A. Chuprin*, J. Young, M. K. Jain, M. Mahowald, F. Kestel, Amyloid research group; Jacksonville, FL/US
*(anthonychuprin@gmail.com)*

**Purpose:** Pittsburgh B Compound (PiB) is one of the newer investigational radiotracers studied for use in evaluation of cardiac amyloid. Potential advantages include the ability to distinguish between types of cardiac amyloid (ATTR vs AL), additional prognostic information, and monitoring of treatment response. Most if not all studies have quantified cardiac PiB uptake using standardized uptake value (SUV) metrics. We investigate applications of volumetric analysis of PiB PET and compare it to SUV metrics.
**Methods or Backround:** Retrospective, blinded, case-cohort study on eight patients with biopsy proven ATTR (n=5) and AL (n=3) amyloidosis with cardiac involvement who had a PiB PET/CT.
**Results or Findings:** Compared with ATTR cardiac amyloidosis, AL amyloidosis showed higher mean PiB volumetric uptake (246 mL vs 29.5 mL, p = 0.0005), mean SUVmax (7.3 vs 5.5, p = 0.085) and mean SUVmean (4.3 vs 3.7, p = 0.191. There was no overlap between ranges of volumetric PiB uptake in cases of cardiac AL amyloidosis and cardiac ATTR amyloidosis (162.6 - 316.1 mL vs. 0 - 60.7 mL), unlike with SUVmax (6.6-8.1 vs. 2.9-8) and SUVmean (2-5.2 vs. 4.1-4.7).
**Conclusion:** Volumetric PiB PET analysis shows unique application for distinguishing AL from ATTR cardiac amyloidosis. Given increasingly sophisticated PACS paving the way for more feasible volumetric analysis, we expect volumetric PET analysis to be more readily available for clinical application. However, further analysis with a larger cohort is needed to confirm these findings and explore any association with volumetric PiB uptake and clinical outcomes.
**Limitations:** The limitation of this study is the sample size.
**Ethics committee approval:** This study was approved by the Institutional Research Ethics Board.
**Funding for this study:** Funding was received from the Mayo Internal Funding-Clinical Research Operations Group (CROG).
**Author Disclosures:**
Jason Young: Nothing to disclose
Manoj K. Jain: Nothing to disclose
Frances Kestel: Nothing to disclose
Anthony Chuprin: Nothing to disclose
Amyloid research group: Nothing to disclose
Madeline Mahowald: Nothing to disclose

**16:00-17:30** **Research Stage 2**

### Research Presentation Session: Artificial Intelligence & Machine Learning & Imaging Informatics

### RPS 1105

### Novel AI models redefining radiology diagnostics

**Moderator**
L. Cerdá-Alberich; Valencia/ES
*(leogibi230@gmail.com)*

### RPS 1105-2

**The use of large language models for first triage decisions for patients at risk for reaction during intravenous contrast administration: a proof of concept**
G. Avesani[1], M. Marin[2], *M. Dolciami*[1], L. D'Erme[1], A. Perazzolo[1], L. Russo[1], V. Celli[1], B. Gui[1], E. Sala[1]; [1]Rome/IT, [2]Gravedona/IT
*(miriam.dolciami@gmail.com)*

**Purpose:** The aim of this study was to determine whether a large language model (GPT 3.5) can provide accurate and valuable guidance on management for patients at risk for reaction during intravenous contrast administration.
**Methods or Backround:** Six guidelines from various scientific societies were collected, both in English and the local language. These documents were embedded using OpenAI embeddings within the LangChain framework, creating a database to provide information to a GPT-3.5 turbo model. We formulated 100 clinical scenarios describing different situations, combining allergic and renal problems (e.g., moderate to severe allergic reaction and different renal functions) and different types of contrast media (iodine and gadolinium). We asked the model to give a textual answer for each clinical scenario indicating the correct patient management following the previously given guidelines. The responses generated by the model were evaluated by a human expert in the field, considering formal correctness and clinical usefulness. A Likert 5-point scale for each task (correctness and usefulness) was used to judge the answers (from 5 = correct/safe or very useful to 1 = completely wrong or completely useless for clinical purposes). We dichotomised the responses with a cut-off of ≥4 to consider the answers acceptable.
**Results or Findings:** The model's answers were judged formally correct and safe for patients in 95% of scenarios and valuable in 84% of cases. Predominantly, answers deemed not valid were considered too vague to be used.
**Conclusion:** LLMs have the potential to aid in the clinical management of critical patients. Such models can be very useful for novice personnel or initial screenings. Better performance might be achieved with fine-tuning and the "tree of thought" techniques.

**Limitations:** There was limited prompt engineering and fine tuning.
**Ethics committee approval:** No patients data were used.
**Funding for this study:** No funding was received for this study.
**Author Disclosures:**
Giacomo Avesani: Nothing to disclose
Benedetta Gui: Nothing to disclose
Alessio Perazzolo: Nothing to disclose
Veronica Celli: Nothing to disclose
Evis Sala: Nothing to disclose
Matteo Marin: Nothing to disclose
Luca D'Erme: Nothing to disclose
Luca Russo: Nothing to disclose
Miriam Dolciami: Nothing to disclose

### RPS 1105-3

**Enhancing quick-acquired MRI scans with the DL-based Aikenist framework: a clinical assessment**

C. S. Arvind[1], S. S. Bhat[2], B. Dikendra[3], S. Z. T. Jordan[1], A. Amrapuram[2], *B. K. N. Prakash*[1]; [1]Singapore/SG, [2]Bangalore/IN, [3]Chennai/IN
*(bhanu_prakash@bii.a-star.edu.sg)*

**Purpose:** MRI remains a cornerstone for clinical diagnostics and research. High-resolution MRI, though detailed, necessitates extended acquisition, increasing patient discomfort and risk of motion artifacts. A frequently used alternative is the quick-acquisition technique, but it tends to compromise image quality due to noise and diminished contrast.
**Methods or Backround:** This study employed a DL-based Aikenist post-processing enhancement on QuickScan-acquired MR scans from 30 brain scans (5400 slices) and 32 abdomen subjects (1920 slices). The brain and abdomen data were acquired from different MRI scanners (GE, Siemens, Toshiba) at different locations using different acquisition protocols, introducing scanner variability.
**Results or Findings:** Our results highlighted significant improvements in image quality metrics, even accounting for scanner variability. For brain scans, the average SNR surged from 28.44 to 42.92 (p <0.001) and CNR from 11.88 to 18.03 (p <0.001). Meanwhile, abdominal scans experienced an SNR leap from 35.30 to 50.24 (p <0.001) and an impressive CNR ascent from 10,290.93 to 93,767.22 (p <0.001). Furthermore, in a double-blinded evaluation, clinicians emphasised the enhanced visibility of intricate anatomical structures, intrastructural changes, such as the IMAT, muscle boundaries, tissue-tissue interface, brain structural delineation, and improved bias field correction, which were previously not accentuated. Their feedback solidified the clinical importance of our enhancement, particularly in discerning smaller regions earlier concealed by noise and reduced contrast.
**Conclusion:** Aikenist enhancement doesn't merely elevate MRI image aesthetics but offers a robust solution to ensuring diagnostic accuracy without extending scan times. As MRI scans become more integral to healthcare, innovations like this pave the way for a more patient-centred and efficient imaging process.
**Limitations:** Results, though promising, stem from specific anatomies and scanner types. Efficacy may fluctuate with varied MRI parameters.
**Ethics committee approval:** The IRB approved this study.
**Funding for this study:** No funding was received for this study.
**Author Disclosures:**
Ashwin Amrapuram: Nothing to disclose
Channarayapatna Srinivasa Arvind: Nothing to disclose
Seema S. Bhat: Nothing to disclose
Baduwal Dikendra: Nothing to disclose
Sim Zheng Ting Jordan: Nothing to disclose
Bhanu K. N. Prakash: Nothing to disclose

### RPS 1105-4

**Frameworks for artificial intelligence research in medical image analyses: a systematic review**

*M. Kanabagatte Nanjundappa*[1], V. Kulkarni[2], A. Kulkarni[3], Y. M[4], C. Maram[5]; [1]Manipal/IN, [2]Leesburg, VA/US, [3]Bengaluru/IN, [4]Karlsruhe/DE, [5]Hyderabad/IN
*(knm_mit@yahoo.com)*

**Purpose:** Artificial intelligence (AI) has a strong footprint in radiology workflow, from image acquisition to reporting findings. This review attempts to overview such AI frameworks in medical image analyses (in diagnostics and therapeutics) from a biomedical engineering perspective.
**Methods or Backround:** Several AI, machine learning (ML), and deep learning (DL) frameworks have been developed by academic research institutes and healthcare companies that are available as open-source software frameworks. Commercially available and community-based DL frameworks are reviewed. The frameworks were compared according to various parameters such as the technology used, CPU/GPU-based implementation, feature learning time, performance evaluation, whether they are desktop installations or cloud-based applications to work with and deployment type (commercial grade with production code or research prototype) and clinical validation.
**Results or Findings:** More than a hundred open-source DL frameworks are available. A few have done exceptionally well in computer-aided diagnosis systems, such as Microsoft InnerEye, NVidia CLARA, pyRadiomics, and MONAI. Regulatory body approvals and clinical validations are pending in many reviewed products.
**Conclusion:** This review paper helps the researchers, radiology residents, and radiologists to gain insight into these frameworks and libraries and select the right one for fast prototype development for image analysis in radiology applications.
**Limitations:** We could not evaluate all the AI frameworks as they have vast applications in many imaging modalities for diagnosis and therapy, and also, most of them are clinically not fully validated to accept them as clinical solution.
**Ethics committee approval:** This is a review and hence ethical issues did not arise.
**Funding for this study:** No funding was received for this study.
**Author Disclosures:**
Anjali Kulkarni: Nothing to disclose
Manjunath Kanabagatte Nanjundappa: Nothing to disclose
Yogeesh M: Nothing to disclose
Vamshikrishna Kulkarni: Nothing to disclose
Charan Maram: Nothing to disclose

### RPS 1105-5

**Energy-optimised scheduling of CT examinations through mathematical modelling**

*M. Segeroth*[1], A. Nurkanović[2], J. Vosshenrich[1], M. Diehl[2], T. Heye[1]; [1]Basel/CH, [2]Freiburg/DE

**Purpose:** Radiology departments and medical imaging devices in particular are major energy consumers within a hospital. The aim of this study was to calculate possible energy savings by optimally scheduling CT examinations.
**Methods or Backround:** Data of all CT examinations performed in our tertiary care radiology department in 2015 on three CT scanners was retrospectively included. Data consisted of examination timestamps and power consumption in kilowatts. The optimal scheduling problem was formulated using linear constraints, a linear objective function and only binary decision variables as an integer linear programming (ILP) problem. This formulation allowed rigorous modelling of a nonconvex and nondifferentiable objective function and offers the possibility to compute the optimal solution even for very large models.
**Results or Findings:** In total 261 workdays were analysed, with 15'072 CT examinations scheduled on the three CT scanners. The duration to solve the ILP for every workday was 10.14 s (9.46-10.80 s). The model yielded a 34.9% reduction in the scanners' combined daily energy consumption through optimal examination scheduling. In absolute values, daily energy consumption could be decreased by 42.2 kWh from 121.0 kWh (120.6-121.4 kWh) to 78.8 kWh (77.8-79.8 kWh; P <.001). Energy savings are primarily attributed to examination shifting, allowing for increased system off times. Overall, 10,930.6 kWh in energy, $2,864 in cost, and 1,399.1 kgCO2eq in carbon emissions could theoretically be saved in our setting.
**Conclusion:** Optimised CT examination scheduling through automatic modelling has substantial sustainability and cost benefits for radiology departments. Feasibility of model implementation in clinical routine needs further investigation.
**Limitations:** Although it was ensured that the approach was optimised, this study requires extensive clinical testing.
**Ethics committee approval:** This study was approved by the ethics committee of northwest and central Switzerland, project ID 2022-01016.
**Funding for this study:** No funding was received for this study.
**Author Disclosures:**
Tobias Heye: Nothing to disclose
Armin Nurkanović: Nothing to disclose
Moritz Diehl: Nothing to disclose
Martin Segeroth: Nothing to disclose
Jan Vosshenrich: Nothing to disclose

### RPS 1105-6

**medBERT.de: a German BERT model tailored for the medical domain: insights into the results of radiological text classification and entity recognition**

*F. Busch*[1], J-M. Papaioannou[1], P. Grundmann[1], F. Borchert[2], L. C. Adams[3], L. Xu[1], M. Makowski[3], A. Löser[1], K. K. Bressem[1]; [1]Berlin/DE, [2]Potsdam/DE, [3]Munich/DE
*(felix.busch@charite.de)*

**Purpose:** We developed medBERT.de, a German BERT (Bidirectional Encoder Representations from Transformers) model for the medical domain trained on 4.7 million German medical documents. Here, we present the results of our custom pretrained BERT models for classification and entity recognition tasks from radiology reports.
**Methods or Backround:** medBERT.de is built on the standard BERT architecture, featuring 12 layers with 768 hidden units each, 8 attention heads, and a 512-token input limit. Three distinct radiological benchmarks based on

2,000 radiology reports, respectively, obtained from a level 1 hospital in Germany, were developed to span various report lengths and tasks: a short text classification from chest x-rays, a longer report classification from chest CT examinations, and a named entity recognition (NER) task from medium-sized CT/x-ray reports of the wrist. Reports were manually labelled by radiologists and medical students for various pathologies and therapeutic devices. The model and benchmarks were made publicly available (https://huggingface.co/GerMedBERT/medbert-512).
**Results or Findings:** medBERT.de displayed superior performance for the chest x-ray (AUROC: 84.65) and CT classification (AUROC: 96.69) tasks compared to previously published German BERT models. For the NER task, the model trained with deduplicated data achieved the highest AUROC of 83.28. Notably, medBERT.de's performance on longer texts from CT reports (258 ± 100 words) was especially pronounced compared to x-ray (98 ± 27 words) or NER (108 ± 41 words) tasks.
**Conclusion:** The study underscores the potential of domain-specific BERT models in efficiently processing radiology reports. Their ability to handle varying report lengths with remarkable accuracy makes them promising tools for radiological applications.
**Limitations:** medBERT.de is primarily based on data from radiology reports. The origin of the data from a single university hospital could lead to bias.
**Ethics committee approval:** This study was approved by an ethics committee; IRB-approval number: EA2/078/22.
**Funding for this study:** No funding was received for this study.
**Author Disclosures:**
Florian Borchert: Nothing to disclose
Keno K. Bressem: Nothing to disclose
Jens-Michalis Papaioannou: Nothing to disclose
Lisa C. Adams: Nothing to disclose
Lina Xu: Nothing to disclose
Felix Busch: Nothing to disclose
Alexander Löser: Nothing to disclose
Marcus Makowski: Nothing to disclose
Paul Grundmann: Nothing to disclose

### RPS 1105-7

**Image fusion using pixelwise gradient model for image fusion (PGMIF)**
*K-H. Cheng*, J. Cai; Hong Kong/HK
*(21040684r@connect.polyu.hk)*

**Purpose:** Magnetic resonance imaging (MRI) plays a pivotal role in the accurate delineation of tumours for radiotherapy. However, conventional MRI sequences often show inconsistencies in tumour contrast across patients. This study aimed to assess the potential of a novel multimodal image fusion method, the pixelwise gradient Model for Image Fusion (PGMIF), to improve MRI tumour contrast and its consistency across patients.
**Methods or Backround:** We utilised T1-w and T2-w MR images from a cohort of 80 patients. The proposed PGMIF was based on a pixelwise gradient to capture the shape of the input images and a Generative Adversarial Network (GAN) term for capturing image contrast. It was compared with other fusion algorithms: gradient model with maximum comparison among images (GMMCI), deep learning model with weighted loss (DLMWL), pixelwise weighted average (PWA), and maximum of images (MoI). Two metrics were used to test the fusion methods' performance: tumour contrast-to-noise ratio (CNR) and a refined Sobel operator analysis to measure the edge sharpness.
**Results or Findings:** PGMIF surpassed in both metrics, registering a CNR of 1.237 ± 0.100. This marked a significant enhancement compared to T1-w (0.976 ± 0.052) and T2-w MR images (1.077 ± 0.087). PGMIF also outperformed other models including GMMCI, DLMWL, PWA, and MoI. In the Sobel operator analysis, PGMIF again showed the highest Sigmoid of Sobel Metric values for T1-w and T2-w MR images comparisons, demonstrating the contrast amplification and edge acuity.
**Conclusion:** The novel PGMIF method shows its potential to enhance MRI tumour contrast while retaining the anatomical structures from the source images. Its implementation could be useful in NPC tumour delineation.
**Limitations:** The ripples of the input images may be amplified in the fused images.
**Ethics committee approval:** The data set used was approved by the Research Ethics Committee in Hong Kong (Kowloon Central/Kowloon East, reference number: KC/KE-19-0085/ER-1).
**Funding for this study:** This research was partly supported by research grants of Mainland-Hong Kong Joint Funding Scheme (MHKJFS) (MHP/005/20), Project of Strategic Importance Fund (P0035421) and Projects of RISA (P0043001) from The Hong Kong Polytechnic University of The Hong Kong Polytechnic University, Shenzhen Basic Research Program (JCYJ20210324130209023) of Shenzhen Science and Technology Innovation Committee, and Health and Medical Research Fund (HMRF 09200576), the Health Bureau, The Government of the Hong Kong Special Administrative Region.
**Author Disclosures:**
Ka-Hei Cheng: Nothing to disclose
Cai J. Jing Cai: Nothing to disclose

### RPS 1105-8

**Recurrence-free survival prediction in head and neck cancers using deep learning: a multicentre, multimodal approach harnessing uncertainty estimation and counterfactual explainability**
*Z. Salahuddin*, H. C. Woodruff, Y. Chen, X. Zhong, P. Lambin; Maastricht/NL
*(zohaib.salahuddin@live.com)*

**Purpose:** This study aims to develop an end-to-end trustworthy deep learning model for predicting recurrence-free survival (RFS) in head and neck cancers, utilising FDG-PET and CT images and automated delineations, with a focus on increasing confidence and explainability through uncertainty predictions and counterfactual image generation.
**Methods or Backround:** Given the prevalence and severity of head and neck cancers worldwide, an algorithm capable of accurately predicting RFS could significantly enhance therapeutic planning and patient management. The developed adaptive 3D resnet-50 deep learning model was trained on multimodal data (clinical data, FDG-PET, and CT images) using a multi-task logistic regression framework. Fivefold cross-validation was performed on 378 patients from 5 different centres, and 111 patients from 2 different centers were used as an external test set. Automated delineations of tumour and lymph nodes were obtained via a modified nnUNet. The model utilised a multi-head multi-loss function to estimate prediction uncertainty and employed a VAE-GAN for latent space traversal, generating counterfactual images to explore and visualise hypothetical scenarios and enhance explainability.
**Results or Findings:** The model demonstrated a competitive c-index of 0.681 [95% CI: 0.663 - 0.694] in fivefold cross-validation and 0.671 on two external test sets. Predictions with lower uncertainty are correlated with superior performance, evidenced by a c-index of 0.683. Kaplan-Meier curve demonstrated a significant split between low and high-risk groups. Counterfactuals revealed that both shape and texture features from FDG-PET and CT images are important for predicting survival.
**Conclusion:** The developed model exhibits promising potential in providing trustworthy and interpretable RFS predictions for H&N cancer patients, leveraging multicentre multimodal data, uncertainty estimates, and counterfactual explainability.
**Limitations:** The model necessitates prospective validation, and conducting an in-silico trial is imperative to assess the clinical efficacy of the counterfactuals and uncertainty predictions.
**Ethics committee approval:** Institutional Review Boards of all participating PROVIDER institutions permitted use of images and clinical data, either fully anonymised or coded, from all cases for research purposes only. Retrospective analyses were performed in accordance with the relevant guidelines and regulations as approved by the respective institutional ethical committees with protocol numbers: MM-JGH-CR15-50 (HGJ, CHUS, HMR, CHUM) and CER-VD 2018-01513 (CHUV). For CHUP, institutional review board approval was waived as all patients signed informed consent for use of their data for research purposes at diagnosis. For MDA, ethics approval was obtained from the University of Texas MD Anderson Cancer Center Institutional Review Board with protocol number: RCR03-0800. For USZ, ethics approval was related to the clinical trial NCT01435252 entitled "A phase II study in patients with advanced head and neck cancer of standard chemoradiation and add-on Cetuximab". For CHB, the fully anonymised data originates from patients who consent to the use of their data for research purposes. List of PROVIDERS: HGJ: Hôpital Général Juif, Montréal, CA; CHUS: Centre Hospitalier Universitaire de Sherbrooke, Sherbrooke, CA; HMR: Hôpital Maisonneuve-Rosemont, Montréal, CA; CHUM: Centre Hospitalier de l'Université de Montréal, Montréal, CA; CHUV: Centre Hospitalier Universitaire Vaudois, CH; CHUP: Centre Hospitalier Universitaire de Poitiers, FR; MDA: MD Anderson Cancer Center, Houston, Texas, USA; USZ: UniversitätsSpital Zürich, CH; CHB: Centre Henri Becquerel, Rouen, FR.
**Funding for this study:** Funding for thist study was received from EuCanImage n° 952103.
**Author Disclosures:**
Yi Chen: Nothing to disclose
Zohaib Salahuddin: Nothing to disclose
Henry Christian Woodruff: Shareholder: Minority shares in radiomics SA
Xian Zhong: Nothing to disclose
Philippe Lambin: Shareholder: Minority shares in the companies Radiomics SA, Convert pharmaceuticals, Comunicare, and LivingMed Biotech. Patent Holder: Co-inventor of two issued patents with royalties on radiomics (PCT/NL2014/050248, PCT/NL2014/050728) licensed to Radiomics SA and one issue patent on mtDNA (PCT/EP2014/059089) licensed to ptTheragnostic/DNAmito, three non-patented invention (softwares) licensed to ptTheragnostic/DNAmito, Radiomics SA and Health Innovation Ventures and three non-issues, non-licensed patents on Deep LearningRadiomics and LSRT (N2024482, N2024889, N2024889).

### RPS 1105-9

**A unified transformer-based model for characterisation and diagnosis of focal liver lesions on multiparametric MRI images**

*Z. Shen*, F. Yan; Shanghai/CN
*(zshen1224@163.com)*

**Purpose:** The aim of this study was to develop and evaluate an AI model that incorporates liver imaging reporting and data system (LI-RADS) criteria and other relevant radiological features for the diagnosis of FLLs using AMRI data. Apart from that, we aimed to compare the performance of the AI model and radiologists with different levels of experience in the internal and external test set.
**Methods or Backround:** We retrospectively collected MRI data from 1,024 patients with 1,147 FLLs who underwent contrast enhanced abdominal MRI. The FLLs were classified into six categories: hepatocellular carcinoma (HCC), intrahepatic cholangiocarcinoma (ICC), metastasis, cyst, haemangioma and focal nodular hyperplasia (FNH). We trained the AI model on MRI images of 560 FLLs from January 2020 to July 2022 in the training set. We evaluated the performance of the AI model using the internal test set (243 FLLs from July 2022 to August 2023) and the external test set (344 FLLs from public data set). We used the DeLong method and the McNemar test to compare the performance of the AI model and two radiologists with different levels of experience.
**Results or Findings:** The AI model achieved an overall accuracy of 0.87, a sensitivity of 0.85, a specificity of 0.91, and an area under the receiver operating characteristic curve (AUC) of 0.92 for FLL diagnosis on the internal test set. The AI model outperformed junior radiologists in terms of accuracy, sensitivity, specificity and AUC except one senior radiologist had similar accuracy and specificity but lower sensitivity. The AI model also showed good generalisation ability across different centres, with an AUC of 0.90 for external public data set.
**Conclusion:** The proposed AI model based on radiological characteristics can effectively diagnose FLLs using MRI data and can assist radiologists in improving their diagnostic performance and efficiency.
**Limitations:** No limitations were identified.
**Ethics committee approval:** No information provided by the submitter.
**Funding for this study:** No funding was received for this study.
**Author Disclosures:**
Fuhua Yan: Nothing to disclose
Zhehan Shen: Nothing to disclose

### RPS 1105-10

**Establishing robust ground truth labels to create machine learning response assessment models using an innovative fusion technique of rectal MRI and whole mount histopathologic specimens**

N. Horvat, J. M. Santos, *J. Ninčević*, C. Firat, J. Heiselman, J. Chakraborty, J. Shia, J. Garcia-Aguilar, M. J. Gollub; New York, NY/US
*(nincevij@mskcc.org)*

**Purpose:** MRI-based radiomics is a promising objective tool for predicting rectal cancer (RC) treatment response but lacks generalisability. Whole mount histology (WMH) is considered the gold standard reference method for point-by-point comparison, and radiomic models have yet to be trained using WMH to assess a rigid point-based registration method to evaluate the fusion of the rectum between WMH and MRI accuracy.
**Methods or Backround:** The study included 18 consecutive rectal cancer patients on neoadjuvant therapy and total mesorectal excision from 2018 to 2021. A multimodal radiology-pathology image registration workflow was developed. First, a radiologist and pathologist delineated the tumour bed, internal and external rectal borders, and eight corresponding MR and WMH image landmarks. Second, automated rescaling computed point-based registration of images via delineated landmarks was performed. Third, initial rigid alignment of MR and WMH images accounted for rectal distension differences using biomechanically constrained plane strain elastic deformable registration. Fourth, a combination of in-house rigid registration, active contours, and finite element software performed image registration. Fifth, a 3D slicer rendered outputs from the multimodal image fusion system to obtain accurate and precise visualisation.
**Results or Findings:** Dice overlap and modified Hausdorff distance of the delineated MR and pathology images showed a significantly good correlation between external and internal border segmentations (P-values <.05, comparing in each case mean values averaged across the mean values from each of the three levels per case).
**Conclusion:** Deformable registration significantly improves the internal and external contour agreement over rigid point-based registration. Establishing such a method will allow the generation of ground truth labels to predict complete response and improve patient care by safely avoiding surgery.
**Limitations:** The limitations of the study are its retrospective design and small sample.
**Ethics committee approval:** This study was approved by the institutional review board, with a waiver for written informed consent, and was compliant with the Health and Insurance Portability and Accountability Act.
**Funding for this study:** This project was partly supported by the National Cancer Institute Cancer Center Core Grant P30 CA008748 and the Society of MSK (PI: Natally Horvat). The RSNA Research & Education Foundation supported the project described through grant number RSD2302 (PI: Natally Horvat). The content is solely the authors' responsibility and does not necessarily represent the official views of the RSNA R&E Foundation.
**Author Disclosures:**
Julio Garcia-Aguilar: Nothing to disclose
Marc Jeffrey Gollub: Nothing to disclose
Joao Manoel Santos: Nothing to disclose
Jayasree Chakraborty: Nothing to disclose
Jinru Shia: Nothing to disclose
Jon Heiselman: Nothing to disclose
Natally Horvat: Consultant: Guerbet Speaker: Bayer
Canan Firat: Nothing to disclose
Josip Ninčević: Nothing to disclose

### RPS 1105-11

**External validation of Alzheimer's disease machine-learning models: generalisability and clinical features**

*H. R. Pereira*[1], V. Sá Diogo[1], D. Prata[2], H. Alexandre Ferreira[1]; [1]Lisbon/PT, [2]London/UK
*(hri.pereira@campus.fct.unl.pt)*

**Purpose:** Recent studies have shown the potential of machine-learning models, based on magnetic resonance imaging (MRI) features, in aiding the diagnosis of Alzheimer's disease (AD) and mild cognitive impairment (MCI). However, these models usually lack generalisability: most use data from the same public data sets, are trained with curated patient and healthy subject data and are not validated with independent "real-world" data.
**Methods or Backround:** We aimed to validate in clinical practice our previously developed models (derived also from public data sets): model 1 – cognitively normal (CN) vs AD, showing a balanced accuracy (BAC) of 90.6%, sensitivity of 91.5%, and specificity of 89.7%; and model 2 - CN vs MCI vs AD, showing a BAC of 62.1% in the multiclassification. Additionally, we explored the features of the misclassified cases. MRI T1-weighted MPRAGE morphometric data (computed with freesurfer 7.1.1) were used from Portuguese hospital patients, comprising 8 AD, 9 MCI and 21 CN (19 headache and 2 depression) patients.
**Results or Findings:** Our model 1 showed a BAC of 97.6%, sensitivity of 100.0%, and specificity of 95.2%, whilst misclassifying one CN as an AD patient. Model 2, on the other hand, showed a BAC of 65.8% (7 CN misclassified as 5 MCI and 2 AD; 5 MCI as 3 CN and 2 AD; 1 AD as MCI). Misclassified MCI patients showed volume changes in brain regions similar to those found in AD (amygdalar and temporal atrophy) or CN (hippocampal sparing), and the opposite was observed for the misclassified AD as MCI patient (entorhinal atrophy only).
**Conclusion:** The results suggest that our models may be of clinical use, provided that physicians frame the classification output within anamneses and clinical findings.
**Limitations:** The study's main limitation was the small data set tested.
**Ethics committee approval:** All the data used in this study was approved by the ethics committee of each Portuguese hospital.
**Funding for this study:** This work was financially supported by Fundação para a Ciência e Tecnologia (FCT) under the projects UIDB/00645/2020 (https://doi.org/10.54499/UIDB/00645/2020), UIDP/00645/2020 (https://doi.org/10.54499/UIDP/00645/2020) , SAICTPAC/0010/2015 and DSAIPA/DS/0065/2018. FCT has further supported HRP through the individual PhD grant 2021.08306.BD, and DP through 2022.00586.CEECIND.
**Author Disclosures:**
Hugo Alexandre Ferreira: Founder: NeuroPsyAI
Vasco Sá Diogo: Nothing to disclose
Diana Prata: Founder: NeuroPsyAI
Helena Rico Pereira: Nothing to disclose

### RPS 1105-12

**Towards safer imaging: a comparative study of deep learning-based denoising and iterative reconstruction in intraindividual low-dose CT scans using an in-vivo large animal model**

*J. Mück*, B. Stenzl, J. Hofmann, S. Afat, A. S. Brendlin; Tübingen/DE
*(jonas.mueck@med.uni-tuebingen.de)*

**Purpose:** Computed tomography (CT) scans are a significant source of medically induced radiation exposure. Novel deep learning-based denoising (DLD) algorithms have been shown to enable diagnostic image quality at lower radiation doses than iterative reconstruction (IR) methods. However, most comparative studies employ low-dose simulations due to ethical constraints. We used real intraindividual animal scans to investigate the dose reduction capabilities of a DLD algorithm in comparison to IR.
**Methods or Backround:** Fourteen sedated pigs underwent two 100% CT scans on the same third generation dual-source scanner, with a two-month interval between each scan. Both times, we additionally reduced the mAs to

50%, 25%, 10%, and 5%. All scans were reconstructed using ADMIRE level 2 (IR2) and the DLD algorithm, resulting in a total of 280 data sets. Objective image quality measures (CT number stability, noise, and contrast-to-noise ratio) were assessed. Three radiologists independently evaluated subjective image quality, and interrater agreement was analysed using Spearman's correlation coefficient. Adequately corrected mixed-effects modeling analysed objective and subjective image quality.
**Results or Findings:** Neither dose reduction nor reconstruction method negatively affected CT number stability (p>0.999). In terms of objective image quality, the DLD algorithm achieved a 25% radiation dose while maintaining noise and contrast-to-noise ratio comparable to 100% IR2. Interrater agreement for subjective image quality ratings was strong (r≥ 0.69, mean 0.93±0.05, 95% CI 0.92-0.94; each p <0.001). Subjective assessments indicated that DLD at 25% radiation dose was comparable to 100% IR2 in terms of image quality, sharpness, and contrast (p≥ 0.281).
**Conclusion:** The DLD algorithm can achieve image quality comparable to the standard IR method but with a significant dose reduction of up to 75%. This suggests a promising avenue for lowering patient radiation exposure without sacrificing diagnostic quality.
**Limitations:** This was a single centre study with a specific hardware and software set-up.
**Ethics committee approval:** This large animal study was approved by the Regional Council (C 01/19 G) and conducted following EU Directive No 2010/63/EU.
**Funding for this study:** No funding was received for this study.
**Author Disclosures:**
Andreas Stefan Brendlin: Nothing to disclose
Johannes Hofmann: Nothing to disclose
Saif Afat: Nothing to disclose
Benedikt Stenzl: Nothing to disclose
Jonas Mück: Nothing to disclose

**16:00-17:30** **Research Stage 3**

### Research Presentation Session: Cardiac

### RPS 1103

### Evolving cardiac MRI techniques: development and clinical applications

**Moderator**
D. Beitzke; Vienna/AT
**Author Disclosures:**
Dietrich Beitzke: Consultant: Cardialysis Cor Lab; Speaker: Siemens Healthineers

### RPS 1103-2

**Blood native T1 time for estimating synthetic haematocrit and extracellular volume: derivation of a conversion formula at 3T**
M. Puseljic, C. Reiter, M. Fuchsjäger, *J. Schmid*; Graz/AT

**Purpose:** Cardiac magnetic resonance imaging (CMR) has emerged as a vital tool for characterising cardiac disorders, with T1 mapping offering quantitative insight into myocardial tissue. The aim of the study was to correlate haematocrit (Hct) levels from blood sampling with native T1 times in the left ventricular (LV) blood pool to derive a formula for estimating synthetic Hct levels (Hctsyn) and for calculation of synthetic extracellular volume fraction (ECVsyn).
**Methods or Backround:** In this retrospective analysis, native T1 times in the LV blood pool (T1blood) were correlated with Hct levels from blood sampling within 24 hours (Hct24h) in 250 CMR scans (3T, MOLLI5(3)3), divided into a derivation and validation cohort. A linear regression equation was derived to calculate Hctsyn and ECVsyn, which was then externally validated.
**Results or Findings:** In the derivation cohort (n=167), Hct24h exhibited a strong association with T1blood (r = -0.711, p < 0.001). The resultant regression equation, Hctsyn = 1/T1blood * 1355.52 - 0.310, enabled calculation of Hctsyn. In the validation cohort (n=83), Hctsyn displayed a good correlation with Hct24h (r = 0.726, p < 0.001). Additionally, ECVsyn, calculated using Hctsyn, demonstrated an excellent correlation with ECV24h (r = 0.940, p < 0.001). Bland-Altman plots confirmed minimal bias in ECVsyn estimation (0.28%).
**Conclusion:** A formula for estimating Hctsyn from T1blood was derived, enabling the calculation of ECVsyn. This approach offers a non-invasive alternative to blood sampling and demonstrates good agreement with established methods, enhancing the clinical utility of CMR.
**Limitations:** Applicability of the results to different scanner setups may be limited by variations in scanner and sequence characteristics.
**Ethics committee approval:** This study was approved by an ethics committee; internal reference number: 35-324 ex: 22/23.
**Funding for this study:** No funding was received for this study.
**Author Disclosures:**
Michael Fuchsjäger: Nothing to disclose
Clemens Reiter: Nothing to disclose
Marijan Puseljic: Nothing to disclose
Johannes Schmid: Nothing to disclose

### RPS 1103-3

**A novel approach to measuring synthetic extracellular volume without invasive blood sampling: the sheva-3t cmr study**
*C. Mantini*, A. Sorella, V. Di Mascio, G. Bisaccia, M. Foglietta, D. Calvo Garcia, L. D'Angelo, S. Gallina, F. Ricci; Chieti/IT
*(cesare.mantini@gmail.com)*

**Purpose:** Existing CMR methods for extracellular volume (ECV) measurement necessitate haematocrit level measurements, which can be impractical in clinical settings. We aimed to derive and validate a multiparametric model for synthetic ECV assessment in clinical 3T CMR.
**Methods or Backround:** We recruited 505 consecutive patients undergoing clinical 3T CMR exams with 48-hour haematocrit sampling. Participants were randomly split into derivation (n=405) and validation (n=100) cohorts. Native T1 was measured in both left ventricular (LV) and right ventricular (RV) blood pools. We derived and validated a multiparametric model for synthetic haematocrit estimation, including covariates selected by multivariate linear regression analysis. The conventional ECV was calculated using a standard blood haematocrit value. Synthetic ECVs were obtained from LV and RV T1 values using Fent's equation and from the 4-factor synthetic haematocrit. We assessed the correlation, agreement, accuracy of classification, and trueness between synthetic and conventional ECVs.
**Results or Findings:** In the derivation cohort, sex, heart rate, and LV and RV native T1 values were selected as independent predictors of haematocrit and built into a 4-factor model. The 4-factor synthetic haematocrit showed better correlation with blood sampling than the LV and RV synthetic haematocrits (R2:0.380; R2:0.341; R2:0.316, respectively). The 4-factor ECV model showed good correlation with conventional ECV, similar to LV and RV ECVs (R2:0.834, R2:0.823, and R2:0.815, respectively), yet yielded the lowest bias (4-factor ECV:-0.024; RV-ECV:-0.162; LV-ECV:-1.067). These findings were confirmed in the validation cohort (4-factor ECV: R2:0.835; bias:-0.26; LV-ECV: R2:0.807; bias:-0.38; RV-ECV: R2:0.777; bias:-1.22).The 4-factor model exhibited substantial agreement (Cohen's kappa: 0.64) and trueness compared with conventional ECV.
**Conclusion:** The novel 4-factor synthetic model improves precision and trueness for haematocrit and ECV estimation. Our findings support broader utilisation of synthetic ECV in 3T settings, obviating the need for invasive blood sampling while ensuring clinical accuracy and reliability.
**Limitations:** This was a retrospective singlecentre study.
**Ethics committee approval:** No information was provdided by the submitter.
**Funding for this study:** No funding was received for this study.
**Author Disclosures:**
Melissa Foglietta: Nothing to disclose
Anna Sorella: Nothing to disclose
Cesare Mantini: Nothing to disclose
Sabina Gallina: Nothing to disclose
Luca D'Angelo: Nothing to disclose
Valeria Di Mascio: Nothing to disclose
Giandomenico Bisaccia: Nothing to disclose
Fabrizio Ricci: Nothing to disclose
Darien Calvo Garcia: Nothing to disclose

### RPS 1103-4

**T1 mapping and extracellular volume for predicting pre-heart failure in children with Duchenne Muscular Dystrophy**
*X. Zhang*, H. Xu, H. Fu, Y-K. Guo; Chengdu/CN
*(altman_0302@163.com)*

**Purpose:** Pre-heart failure (pre-HF) is being recognised as an essential stage known to progress to symptomatic HF. However, early detection of HF in Duchenne Muscular Dystrophy (DMD) is difficult due to progressive muscle necrosis, physical inactivity, and lack of awareness of their deteriorating cardiac function. Therefore, our study aimed to investigate the prognostic value of cardiac magnetic resonance (CMR) for pre-HF in children with DMD.
**Methods or Backround:** A total of 113 patients with DMD (age 8.0 [7.0, 9.0] years; 111 boys, 98.2%) were included from July 2018 to July 2022. The study outcome was pre-HF. According to the AHA/ACC/HFSA guideline for the management of HF, pre-HF was defined as patients without current or prior symptoms of HF but with evidence of abnormal cardiac biomarkers, functional or structural heart disease. Survival estimates were calculated by Kaplan-Meier curves with the log-rank test.
**Results or Findings:** During a mean follow-up of 24.2 ± 11.6 months, a total of 34 patients reached pre-HF. Univariate Cox regression analyses and

Multivariate stepwise analyses showed that native T1 (hazard ratio [HR]: 1.014, 95% CI: 1.005-1.022; p =0.002) in model 1 and ECV (HR: 1.366, 95% CI: 1.219-1.531; p <0.001) in model 2 had significant prognostic associations with pre-HF. The cut-off value of ECV was 30.1% (AUC: 0.782; sensitivity: 79.4%; specificity: 75.9%). A native T1 cut-off value of 1294.83 ms had a sensitivity of 55.9%, a specificity of 96.2, and an AUC of 0.790. The cut-off value of ECV and native T1 significantly differed between patients with and without pre-HF in both the LGE-positive and -negative groups (all P < 0.001).
**Conclusion:** T1 mapping and ECV had prognostic value for pre-HF in DMD patients, which provided optimal risk stratification for early cardiac involvement in DMD patients.
**Limitations:** This was a retrospective single centre analysis.
**Ethics committee approval:** The Institutional Review Boards of our hospital approved this retrospective study.
**Funding for this study:** No funding was received for this study.
**Author Disclosures:**
Huayan Xu: Nothing to disclose
Hang Fu: Nothing to disclose
Ying-Kun Guo: Nothing to disclose
Xinyuan Zhang: Nothing to disclose

### RPS 1103-5

**Joint bright- and black-blood late gadolinium enhancement and T1-rho mapping for robust myocardial scar imaging**

*V. De Villedon De Naide*[1], M. Stuber[2], J. H. Zhang[2], K. Narceau[1], P. Gut[2], V. Nogues[1], M. Villegas-Martinez[1], H. Cochet[1], A. Bustin[1]; [1]Pessac/FR, [2]Lausanne/CH
*(victor.de-villedon@ihu-liryc.fr)*

**Purpose:** Bright-blood sequences are used to retrieve heart anatomy information, while black-blood late gadolinium enhancement has shown potential for scar detection. Contrast-agent-free T1-rho mapping is a promising technology for quantifying cardiomyopathies. The aim of this study is to provide a single sequence that combines the strengths of black-blood scar detection, bright-blood scar localisation, and T1-rho scar quantification.
**Methods or Backround:** The proposed 2D whole-heart SPOT1-rho acquisition is a single-shot breath-held sequence gathering black- and bright-blood images that are averaged for optimal detection and localisation of scarred tissue. For the bright-blood shots, five shots with increasing T1-rho preparation times were acquired, to generate a T1-rho map, for scar quantification. A phantom (T1MES) experiment was first conducted with PSIR, SPOT, T1-rho mapping and the proposed SPOT1-rho acquisition. The obtained T1-rho values were compared, along with mean signal intensities from bright- and black-blood images. The same sequences were then prospectively tested in two patients with myocardial infarction and one healthy subject using a 1.5T Siemens Aera scanner. 3-slice short-axis images were acquired 15min post-injection of Gadolinium. T1-rho values were measured in both remote and injured myocardial regions.
**Results or Findings:** In phantom, an excellent correlation was observed between the reference T1-rho values and those obtained using the SPOT1-rho sequence, along with consistent signal intensities. In patients, elevated myocardial T1-rho values were measured in injured areas, closely matching values obtained from the reference T1-rho mapping sequence.
**Conclusion:** The proposed SPOT1-rho combines bright- and black-blood imaging and T1-rho mapping to enhance scar detection, localisation, and quantification, offering a promising and versatile tool for myocardial assessment in both research and clinical settings.
**Limitations:** Validation of the SPOT1-rho sequence requires a greater patient cohort, while clinical application of T1-rho mapping is still at an early stage.
**Ethics committee approval:** No information provided by the submitter.
**Funding for this study:** This project was supported by funding from the French National Research Agency under grant agreements Equipex MUSIC ANR-11-EQPX-0030, ANR-22-CPJ2-0009-01, ANR-21-CE17-0034-01, Programme d'Investissements d'Avenir ANR-10-IAHU04-LIRYC. This project has received funding from the European Research Council (ERC) under the European Union's Horizon 2020 research and innovation programme (grant agreement No101076351).
**Author Disclosures:**
Victor De Villedon De Naide: Nothing to disclose
Pauline Gut: Nothing to disclose
Aurelien Bustin: Nothing to disclose
Hubert Cochet: Nothing to disclose
Kalvin Narceau: Nothing to disclose
Jana Huiyue Zhang: Nothing to disclose
Manuel Villegas-Martinez: Nothing to disclose
Victor Nogues: Nothing to disclose
Matthias Stuber: Nothing to disclose

### RPS 1103-6

**Cardiac magnetic resonance in infarct-like myocarditis: transmural late gadolinium enhancement is associated with long-term outcome**

*A. Isaak*, J. Wirtz, D. Kravchenko, N. Mesropyan, U. I. Attenberger, C. Öztürk, S. Zimmer, D. Kütting, J. A. Luetkens; Bonn/DE
*(alexander.isaak@ukbonn.de)*

**Purpose:** The aim of this study was to evaluate the prognostic value of cardiac magnetic resonance (CMR) imaging parameters in terms of the occurrence of MACE in patients with infarct-like myocarditis.
**Methods or Backround:** Patients with acute myocarditis confirmed by CMR between 2007 and 2020 were retrospectively identified. Only patients with infarct-like presentation (chest pain and ST-segment elevation on electrocardiogram and/or troponin elevation) were included into analysis. Functional and morphological imaging analyses were performed. Late gadolinium enhancement (LGE) was qualitatively and quantitatively assessed. The association between different parameters and the occurrence of major adverse cardiac events (MACE; including cardiovascular death, new onset of acute symptoms or heart failure symptoms, implantation of pacemaker or defibrillators) within 5 years after discharge was tested using a univariable and multivariable Cox regression and Kaplan-Meier analysis.
**Results or Findings:** 130/345 patients (38%) had infarct-like presentation (mean age, 40±19 years; 97 men, 75%). LGE lesions involved mostly the subepicardium (111/130 patients [85%]; midwall: 45/130 patients [35%]; both subepicardium and midwall: 27/130 patients [21%]). Septal segments were involved in 42/130 patients (32%). Transmural LGE extension was present in 15/130 patients (12%). The median extent of LGE was 7% (IQR, 4-10). Median duration of follow-up was 19.3 months (IQR, 4.5-53), and MACE occurred in 18/130 patients (14%). Univariable Cox regression analyses revealed an association between MACE and both, LGE extent and transmural LGE extension. In multivariable Cox regression analysis, transmural extension of LGE was an independent predictor for MACE (Hazard ratio, 6.34; 95% CI: 2.29, 17.49; P <0.001). Patients with transmural extension of LGE had a shorter event-free time on Kaplan-Meier analysis (mean [95%CI], 136 weeks [70, 198] vs 236 weeks [221, 252]; log rank P <0.001).
**Conclusion:** Transmural LGE on CMR seems to be associated with long-term occurrence of MACE in infarct-like myocarditis.
**Limitations:** The retrospective nature of the study was identified as a limitation.
**Ethics committee approval:** This was a retrospective study.
**Funding for this study:** A.I. was funded by the BONFOR Research Commission of the Medical Faculty Bonn (BONFOR-Forschungskommission der Medizinischen Fakultät Bonn) and by the German Research Foundation (Deutsche Forschungsgemeinschaft, DFG) under Germany's Excellence Strategy—EXC2151—390873048.
**Author Disclosures:**
Julian Alexander Luetkens: Nothing to disclose
Sebastian Zimmer: Nothing to disclose
Narine Mesropyan: Nothing to disclose
Alexander Isaak: Nothing to disclose
Can Öztürk: Nothing to disclose
Dmitrij Kravchenko: Nothing to disclose
Ulrike I. Attenberger: Nothing to disclose
Johannes Wirtz: Nothing to disclose
Daniel Kütting: Nothing to disclose

### RPS 1103-7

**Left ventricular diastolic dysfunction grading from a single MR 4D flow measurement**

*C. Reiter*, U. Reiter, E. Kolesnik, C. Kräuter, A. Schmidt, D. Scherr, M. Fuchsjäger, G. Reiter; Graz/AT
*(clemens.reiter@medunigraz.at)*

**Purpose:** The aim of this study was to evaluate the feasibility of assessing left ventricular (LV) diastolic dysfunction from a single MR 4D flow measurement using the established echocardiographic algorithm.
**Methods or Backround:** 94 prospectively recruited patients underwent echocardiography and nearterm MR whole-heart 4D flow imaging. LV ejection fraction (EF), left atrial volume index (LAVI), early- (E) and late (A) diastolic transmitral velocities, early diastolic myocardial tissue velocity (e'), and tricuspid regurgitation velocity (TR) were determined from echocardiography and used for grading of diastolic dysfunction according to the 2016 ASE/EACVI algorithm. Comparable variables were derived from MR 4D flow: LVEF and LAVI were evaluated from multiplanar reformatted magnitude images. E, A and e' were analysed from 4D flow velocity fields, and mean pulmonary arterial pressure (mPAP) was assessed from duration of vortex in the main pulmonary artery. LV diastolic dysfunction was graded according to the same algorithm as in echocardiography. Relationships between echocardiographic and 4D flow parameters were analysed by correlation analysis, the agreement for grading between modalities for LV diastolic dysfunction was investigated by contingency table analysis.

Thursday

**Results or Findings:** LV diastolic dysfunction of grade 0, indeterminate, grade I, grade II and grade III were found in 51, 9, 13, 13 and 8 subjects by echocardiography. All volumetric and velocity parameters from MR and echocardiography correlated strongly (r=0.75-0.92). In cases where TR was assessable with echocardiography, a strong correlation to MR-derived mPAP was found (r=0.81). Using cut-offs of LAVI >50 ml/m2 and mPAP >25mmHg, there was excellent agreement between 4D flow and echocardiographic grading of diastolic dysfunction with a weighted kappa of 0.85.
**Conclusion:** The assessment of left ventricular diastolic function from a single 4D flow measurement is possible with excellent agreement to echocardiography.
**Limitations:** This was a single-centre study.
**Ethics committee approval:** This study was approved by an ethics committee; ClinicalTrials.gov, NCT01728597 & ClinicalTrials.gov, NCT03253835.
**Funding for this study:** Funding was received from the OeNB Anniversary Fund 17934.
**Author Disclosures:**
Ursula Reiter: Nothing to disclose
Gert Reiter: Employee: Gert Reiter is an employee of Siemens Healthineers.
Michael Fuchsjäger: Nothing to disclose
Ewald Kolesnik: Nothing to disclose
Daniel Scherr: Nothing to disclose
Clemens Reiter: Nothing to disclose
Albrecht Schmidt: Nothing to disclose
Corina Kräuter: Nothing to disclose

### RPS 1103-8

### Non-invasive diagnosis of pulmonary hypertension: a comparison between MR 4D flow and echocardiography

*G. Reiter*, C. Reiter, C. Kräuter, E. Kolesnik, D. Scherr, A. Schmidt, M. Fuchsjäger, U. Reiter; Graz/AT
*(gert.reiter@siemens-healthineers.com)*

**Purpose:** MR time-resolved 3-directional phase-contrast (4D flow) imaging allows estimation of mean pulmonary arterial pressure (mPAP) and diagnosis of pulmonary hypertension (PH) from the duration (t_vortex) of vortical blood flow in the main pulmonary artery. The purpose of the study was to investigate the relationship between 4D flow and standard echocardiographic measures for pressure estimation and diagnosis of PH.
**Methods or Backround:** 94 patients were prospectively investigated by transthoracic echocardiography and near-term 3T 4D flow imaging. Echocardiographic evaluation included the measurement of the tricuspid regurgitant jet velocity (TR) and calculation of the tricuspid regurgitant pressure gradient (TRPG). 4D flow data were employed to derive t_vortex and calculate mPAP. The relationship between TRPG and t_vortex was analysed by regression analysis, the agreement on the presence of PH by contingency table analysis.
**Results or Findings:** A tricuspid jet was identified in 69 patients (73%). For these subjects the relationship between t_vortex and TRPG was well described by a linear model of t_vortex on TRPG (R=0.88). The definitions of PH by mPAP≥25 mmHg and by TR>2.8 m/s for echocardiography were consistent with the linear model and optimised the agreement of 4D flow and echocardiography for diagnosis of PH. The resulting kappa-values were 0.94 for subjects with visible tricuspid jet and 0.90 for all subjects. Using the threshold of mPAP>20 mmHg for diagnosis of PH resulted in the cut-off TR≥2.5 m/s, yielding kappa-values of 0.79 for subjects with visible tricuspid jet and 0.67 for the entire population.
**Conclusion:** There is a strong relationship between 4D flow and echocardiographic measures for diagnosis of PH. While the old PH-cut-off mPAP≥25 mmHg shows high agreement between 4D flow-based and echocardiographic PH diagnosis, the recently introduced PH-cut-off mPAP>20 mmHg reduces this agreement substantially.
**Limitations:** There were no invasive measurements.
**Ethics committee approval:** This study was approved by the ethics committee of the Medical University of Graz, Austria; ClinicalTrials.gov: NCT01728597, NCT03253835.
**Funding for this study:** Funding for this study was received from the OeNB Anniversary Fund 17934.
**Author Disclosures:**
Ursula Reiter: Nothing to disclose
Gert Reiter: Employee: Research & Developement Siemens Healthineers
Michael Fuchsjäger: Nothing to disclose
Ewald Kolesnik: Nothing to disclose
Daniel Scherr: Nothing to disclose
Clemens Reiter: Nothing to disclose
Albrecht Schmidt: Nothing to disclose
Corina Kräuter: Nothing to disclose

### RPS 1103-9

### Quantitative perfusion CMR with adenosine for the assessment of coronary microvascular disease in heart failure with preserved ejection fraction patients

*A. Ezponda Casajus*[1], C. Mbongo[1], P. Kellman[2], A. González Miqueo[1], G. Bastarrika Alemañ[1]; [1]Pamplona/ES, [2]Bethseda, MD/US
*(aezponda@unav.es)*

**Purpose:** Coronary microvascular disease (CMD) is a main mechanism in the development of diastolic dysfunction, a condition frequently observed in heart failure with preserved ejection fraction (HFpEF) patients. Quantitative stress perfusion cardiovascular magnetic resonance (CMR) is a useful non-invasive technique for the assessment of CMD. The aim of our study was to compare myocardial perfusion reserve (MPR) and myocardial blood flow (MBF) values by using an adenosine stress/rest quantitative perfusion CMR protocol in stage-B (asymptomatic with known risk factors and diastolic dysfunction) and stage-C (with signs and symptoms) HFpEF patients.
**Methods or Backround:** Between December 2021 and November 2022, 39 HFpEF patients (23 in stage-B and 16 in C) underwent quantitative CMR with adenosine for the assessment of CMD. None of the patients had a prior history of severe CAD. Global MBF during rest, stress and MPR indices were calculated using automated pixelwise quantitative myocardial perfusion mapping. Demographics and cardiovascular disorders and risk factors were recorded for the different groups of the HFpEF cohort.
**Results or Findings:** Stage-B and stage-C patients do not present statistically significant differences in cardiovascular risk factors nor in prior medical history. Stage-C patients were significantly older than stage B patients (76.8n ±5.2 vs 67.1 ±8.4 years old, p <0.001). Stage-C HFpEF patients presented a significantly lower median of global MPR compared to stage B HFpEF patients (2.31, IQR 1.72-2.74 vs 3.20, IQR 2.80-3.55; p =0,004). Regarding stress perfusion values, global median endocardial BF was also significantly lower in patients at stage C (1.60, IQR 1.22-2.05 vs 2.23, IQR 1.66-2.55; p =0,044). There were no significant differences in rest MBF values between groups.
**Conclusion:** Stage-C HFpEF patients present significant lower values of non-invasive biomarkers of MCD evaluated with quantitative perfusion CMR, than asymptomatic HFpEF patients.
**Limitations:** No limitations were identified.
**Ethics committee approval:** This study was approved by the ethics committee of the Clínica Universidad de Navarra.
**Funding for this study:** CRUCIAL (H2020): this project has received funding from the SESAR Joint Undertaking under the European Union's Horizon 2020 research and innovation programme under grant agreement No 848109.
**Author Disclosures:**
Carmen Mbongo: Nothing to disclose
Peter Kellman: Nothing to disclose
Arantxa González Miqueo: Nothing to disclose
Ana Ezponda Casajus: Nothing to disclose
Gorka Bastarrika Alemañ: Nothing to disclose

### RPS 1103-10

### Residual myocardial hyperaemia in regadenoson stress/rest quantitative perfusion CMR

*A. Ezponda Casajus*, C. Mbongo, M. B. Barrio Piqueras, M. R. López de la Torre Carretero, P. Kellman, M. Vidorreta Díaz de Cerio, G. Bastarrika Alemañ; Pamplona/ES
*(aezponda@unav.es)*

**Purpose:** Regadenoson is a recently introduced vasodilator for stress CMR that possesses a relatively long half-life. As this fact may impact myocardial blood flow (MBF) and myocardial perfusion reserve (MPR) estimated when quantitative stress/rest CMR perfusion is performed, this study sought to investigate the presence of residual myocardial hyperaemia on the recovery phase in patients undergoing stress CMR.
**Methods or Backround:** Fifty patients with clinical indication for stress CMR underwent quantitative perfusion imaging in resting conditions, after regadenoson-induced hyperaemia (400 mcg, 5 mL), and 10 minutes after recovery with aminophylline. A total dose of 0.15 mmol/kg of Gadobutrol was administered. Studies showing late gadolinium enhancement (LGE) were excluded. Global myocardial blood flow during rest (MBF-rest), stress (MBF-stress) and recovery (MBF-recov) and MPR indices (MPR-rest and MPR-recov) were calculated using automated pixel-wise quantitative myocardial perfusion mapping.
**Results or Findings:** A total of 33 patients (25 male, mean age of 61.4 ±2.2 years) were included in the analysis. Seventeen studies showing LGE (15 transmural, 2 subendocardial) were excluded. Global MBF-rest and MBF-stress were 0.81 ±0.26 and 2.11 ±0.73, respectively. After recovery with aminophylline, myocardial perfusion did not return to the resting values (MBF-recov of 0.91 ±0.33) and statistically differed from MBF-rest (P <0.01), suggesting residual myocardial hyperaemia. This resulted in an abnormally low MPR-recov (2.51 ±0.85) with respect to MPR-rest (2.68 ±0.93) (P <0.01).

**Conclusion:** Despite the use of aminophylline to reverse the vasodilator effect, MBF does not return to resting values and MBF-recov cannot be used as a substitute for MBF-rest when regadenoson is used. Consequently, a rest/stress protocol is advised for quantitative CMR perfusion to obtain accurate MBF and MPR parameters.
**Limitations:** This study has included a reduced number of participants.
**Ethics committee approval:** No information provided by the submitter.
**Funding for this study:** No funding has been received for this study.
**Author Disclosures:**
Carmen Mbongo: Nothing to disclose
Peter Kellman: Nothing to disclose
Marta Vidorreta Díaz de Cerio: Nothing to disclose
Miguel Barrio Barrio Piqueras: Nothing to disclose
Manuel Rafael López de la Torre Carretero: Nothing to disclose
Ana Ezponda Casajus: Nothing to disclose
Gorka Bastarrika Alemañ: Nothing to disclose

### RPS 1103-11

**spCMR as a predictor validity for MACE**

*P. Palumbo*, D. Boccetti, S. Lamja, A. Barile, E. Di Cesare; L'Aquila/IT
*(palumbopierpaolo89@gmail.com)*

**Purpose:** The aim of our study is to analyse the impact of stress perfusion CMR on the management of patients with known or suspected angina CAD.
**Methods or Backround:** Coronary Artery disease (CAD) is a much-discussed topic because of its burden on healthcare systems and patients' quality of life. However, there is still debate about the best strategy to control angina symptoms and to reduce future events. This was a historical, prospective study. 164 patients with known or suspected CAD who underwent 3.0-T stress CMR were enrolled (22 F, 142 M; mean age 65.9 years CI 95% 64.67-67.11). We recorded all clinical information including angina symptoms and all major cardiac events (MACE) occurred during the follow-up, including cardiovascular death, arrhythmias and acute myocardial infarction. All therapeutic strategies adopted from clinicians were collected.
**Results or Findings:** Mean follow-up was 2.8 years (IQ range 0.9-3.9). During the follow up 52 patients reported MACE (32%), while 70 patients showed modification of angina symptoms (43 patients reported improvement of symtoms). In spite of the way angina symptoms developed, none of the therapeutic strategies analysed were advantageous in angina improvement. Ischaemia shows a high stratification validity for clinical evolution in IHD patients (OR 2.18, 95%CI 1.12-4.26; P-value 0.022). among therapeutic strategies, only revascularisation showed a significant impaction on MACE occurrence (OR 0.28 P-value 0.016).
**Conclusion:** IHD is a dynamic disease. CMR-derived ischaemia revealed a good prediction validity for MACE occurrence and adequate therapeutic strategies should be oriented on spCMR stratification.
**Limitations:** The number of patients, time of follow-up as well as the retrospective nature of the study were identified as limitations.
**Ethics committee approval:** This is a retrospective study.
**Funding for this study:** No funding was received for this study.
**Author Disclosures:**
Danilo Boccetti: Nothing to disclose
Pierpaolo Palumbo: Nothing to disclose
Ernesto Di Cesare: Nothing to disclose
Stefania Lamja: Nothing to disclose
Antonio Barile: Nothing to disclose

**16:00-17:30 Research Stage 4**

### Research Presentation Session: Genitourinary

### RPS 1107

### Female genitourinary imaging

**Moderator**
I. Blazic; Belgrade/RS
*(ivanablazic2015@gmail.com)*

### RPS 1107-2

**Early regression index (ERI) for the prediction of local control in locally advanced cervical cancer**

S. Bottazzi[1], *L. Russo*[1], D. Cusumano[2], R. Autorino[1], L. Boldrini[1], A. Amerighi[1], A. Rame[1], E. Sala[1], B. Gui[1]; [1]Rome/IT, [2]Olbia/IT

**Purpose:** Early Regression Index (ERI) is an image-based biomarker which combines gross tumour volume (GTV) measured at staging (Vpre) and at mid-therapy (Vmid) to quantify early tumour shrinkage. It showed promising results in predicting pathological complete response (pCR) in rectal and cervical cancer treated with neoadjuvant chemoradiotherapy. This study aims to evaluate the feasibility of ERI in predicting local control (LC) after cisplatinum-based chemotherapy and external beam radiotherapy (CRT) followed by brachytherapy in locally advanced cervical cancer (LACC), starting from T2-WI and apparent diffusion coefficient (ADC) maps.
**Methods or Backround:** 91 patients with LACC (FIGO IB3-IVA) underwent MRI for staging and after CRT. GTV was delineated on the axial oblique T2-WI and ADC map and ERI was calculated on both sequences. Response to CRT and brachytherapy was evaluated six months after the end of treatment using MRI and PET/CT exams. LC was considered in case of the absence of residual disease. The ERI performance was quantified by calculating the area (AUC) under the Receiver Operating Characteristic (ROC) curve and measuring sensitivity and specificity at the best threshold value.
**Results or Findings:** The performance of ERI-T2 (AUC=0.84; 95% CI 0.76-0.95) was superior to that reported by ERI-ADC (AUC=0.72; 95% CI 0.63-0.81). At the best cut-off threshold, ERI-T2 showed excellent specificity (100.0%) with limited sensitivity (67.4%), while ERI-ADC showed high specificity (87.2%) and low sensitivity (59.1%). At subgroup analysis, both ERI-T2 and ERI-ADC showed lower accuracy in adenocarcinoma (72.3% and 70.4%) compared to squamous subgroup (87.5% and 81.3%).
**Conclusion:** ERI is a promising biomarker in LACC treated with concurrent CRT. Combining ERI-T2 and ERI-ADC, it is possible to identify poor responders after CRT thus modulating the brachytherapy boost consequently.
**Limitations:** Retrospective single-center study
**Ethics committee approval:** Not applicable.
**Funding for this study:** No funding was received for this study.
**Author Disclosures:**
Andrea Amerighi: Nothing to disclose
Rosa Autorino: Nothing to disclose
Davide Cusumano: Nothing to disclose
Luca Boldrini: Nothing to disclose
Benedetta Gui: Nothing to disclose
Silvia Bottazzi: Nothing to disclose
Evis Sala: Nothing to disclose
Luca Russo: Nothing to disclose
Anna Rame: Nothing to disclose

### RPS 1107-3

**The performance of the node reporting and data system 1.0 (Node-RADS) in staging patients with cervical carcinoma according to the new FIGO classification (2018)**

*R. V. Ninkova*, A. Calabrese, F. Curti, S. Riccardi, M. Gennarini, V. Miceli, A. Cupertino, C. Catalano, L. Manganaro; Rome/IT
*(roberta.ninkova@gmail.com)*

**Purpose:** The aim of this study is to explore the diagnostic accuracy, specificity, sensitivity, positive and negative predictive value (PPV and NPV) of Node-RADS in assessing the risk of metastatic lymph node (LN) involvement of cervical cancer (CC) patients using magnetic resonance imaging (MRI). We also evaluated the applicability and feasibility of the score among three different readers with different years of experience.
**Methods or Backround:** From December 2014 to June 2023, 140 patients were treated at our department. 68 patients underwent staging MRI, neoadjuvant chemotherapy and radical surgery, and were included in our study. MRI images were retrospectively reviewed and Node-RADS scores were assigned for the main bilateral pelvic LN stations. LNs were evaluated in a scoring category of 1 to 5 to assess risk of metastatic involvement: 1 (very low), 2 (low), 3 (equivocal), 4 (high), 5 (very high). The adequacy of the score with respect to the actual neoplastic involvement was then evaluated by comparing it with the results of histological examination.
**Results or Findings:** The resulting sensitivities, specificities, PPVs, NPVs, and accuracies were 100%, 45%, 56%, 100%, 68% for >1, 92.8%, 72.5%, 70.3%, 93.5%, 80.9% for >2, 71.4%, 100%, 100%, 83.3%, 88.2% for >3, 42.9%, 100%, 100%, 71.4%, 76.4% for >4. The inter-observer agreement between the Node-RADS scores assigned by the senior reader compared with the scores assigned by junior reader 1 and the scores assigned by the senior reader compared with junior reader 2 was 0.888 and 0.738, respectively.
**Conclusion:** The Node-RADS score is a valid system for correct and standardised evaluation of LN stations, increasing diagnostic accuracy particularly for patients with CC. It represents an easily applicable standardisation system that can be useful in clinical practice, improving the proper management of patients with CC.
**Limitations:** The main limitation is the patient sample and the retrospective nature of the study.
**Ethics committee approval:** This study was conducted in accordance with the Declaration of Helsinki.
**Funding for this study:** No funding was received for this study.

**Author Disclosures:**
Marco Gennarini: Nothing to disclose
Alessandro Calabrese: Nothing to disclose
Valentina Miceli: Nothing to disclose
Sandrine Riccardi: Nothing to disclose
Angelica Cupertino: Nothing to disclose
Roberta Valerieva Ninkova: Nothing to disclose
Federica Curti: Nothing to disclose
Carlo Catalano: Nothing to disclose
Lucia Manganaro: Nothing to disclose

### RPS 1107-4

**CT-derived radiomics analysis helps to identify ovarian clear cell carcinoma subtype in epithelial ovarian cancer: a multicentre study**

*J. Ren*, Y-L. He, L. Mao, Y. Li, Z. Yuan, Z. Y. Jin, X. Li, H-D. Xue; Beijing/CN
*(renjing703@163.com)*

**Purpose:** The study aimed to assess the value of CT-derived radiomics in preoperatively identifying ovarian clear cell carcinoma (CCC) subtype in epithelial ovarian cancer (EOC) using multicenter datasets.
**Methods or Backround:** A total of 457 patients with EOCs were retrospectively recruited from three medical centres: Centre A (n=349), divided into training (n=280) and internal testing (n=69) sets based on surgery date, while Centre B (n=74) and Centre C (n=34) were external testing sets. Patients were grouped into CCC or non-CCC subtype based on postoperative pathology. We documented six clinical characteristics and assessed ten radiological characteristics of each lesion by two radiologists. Three models to identify CCC subtype in EOC were built: a radiomic signature using selected radiomic features, a traditional model with selected clinical and radiological characteristics, and an integrated model combining radiomic signature, clinical characteristics, and radiological characteristics. These models were constructed using JMIM feature selection and logistic regression in 10-fold cross-validation. Diagnostic performance was assessed in internal and external test sets.
**Results or Findings:** The mean age of the 457 patients was 53.7 ± 10.4 years. CCC accounted for 21.0% (96/457) of all EOC cases. The integrated model, which utilized the radiomic signature plus one clinical characteristic and three radiological characteristics, demonstrated better performance over the traditional model and the radiomic signature. The integrated model achieved AUCs of 0.890 in the internal testing set, 0.885 in external testing set 1, and 0.781 in external testing set 2, surpassing the traditional model with AUCs of 0.840, 0.884, and 0.735, and the radiomic signature with AUCs of 0.811, 0.836, and 0.778.
**Conclusion:** CT-derived radiomics analysis proved to be valuable in the identification of the CCC subtype in EOC, suggesting its potential to enhance subtype-specific therapeutic approaches.
**Limitations:** No limitations were identified.
**Ethics committee approval:** This retrospective study was approved by the local institutional review board, which waived the requirement for written informed consent.
**Funding for this study:** Funding was provided by Natural Science Foundation of China [grant No. 81901829]; and National High Level Hospital Clinical Research Funding [grant No. 2022-PUMCH-A-004 & 2022-PUMCH-A-109].
**Author Disclosures:**
Yong-Lan He: Nothing to disclose
Li Mao: Nothing to disclose
Zheng Yu Jin: Nothing to disclose
Hua-Dan Xue: Nothing to disclose
Jing Ren: Nothing to disclose
Xiuli Li: Nothing to disclose
Zhilin Yuan: Nothing to disclose
Yuan Li: Nothing to disclose

### RPS 1107-5

**Diagnostic performance and accuracy of the O-RADS MRI scoring system in adnexal masses**

*S. Bozer*, D. Kuru Öz, G. A. Erden; Ankara/TR
*(drsenabozer@yahoo.com)*

**Purpose:** The ovarian-adnexal reporting and data system-magnetic resonance imaging (O-RADS MRI) guideline determines the malignancy risk of adnexal lesions and provides a common language between radiologists and clinicians. The aim of this study was to determine the diagnostic performance of the O-RADS MRI scoring system.
**Methods or Backround:** Patients who underwent pelvic MRI with any clinical prediagnosis between January 2011-April 2023 were screened. Exclusion criteria were as fallows, history of malignancy or gynaecological surgery for non-malignant reasons, O-RADS 1 category lesions and contraindication for contrast administration. MR images were evaluated by two radiologists who were unaware of clinical and laboratory findings. Lesions with different scores were reviewed by consensus. Histopathologic results were accepted as the reference standard. Lesions that did not have a histopathologic diagnosis but regressed on follow-up imaging or remained stable for at least six months were considered benign. Lesions in the O-RADS 4 category and above were considered malignant. For statistical analysis, borderline tumours were included in the malignant category.
**Results or Findings:** A total of 209 adnexal lesions, of which 184 were benign, three were borderline, and 22 were malignant, were included in the study. The sensitivity, specificity, positive predictive value, negative predictive value and accuracy of the O-RADS MRI score were 100%, 97.2%, 83.3%, 100%, and 97.6%. Interobserver agreement was very high (k=0.95).
**Conclusion:** The sensitivity, specificity and overall accuracy of the O-RADS MRI score in this study were similar to those reported for the original score. Our findings support the use of the O-RADS MRI score in the evaluation of adnexal masses with high interobserver reliability without loss of diagnostic accuracy.
**Limitations:** The limitations of the study are small sample size, relatively low number of malignant lesions and retrospective nature.
**Ethics committee approval:** This study was approved by the Ethics committee.
**Funding for this study:** No funding was received for this study.
**Author Disclosures:**
Diğdem Kuru Öz: Nothing to disclose
Sena Bozer: Nothing to disclose
Gül Ayşe Erden: Nothing to disclose

### RPS 1107-6

**Thrombohaematoma-associated placentomegaly: opening the “black box” of pregnancy**

*E. A. Kirillova*[1], I. Mashchenko[1], P. Kozlova[1], L. Kashapova[2], T. Mamiseishvili[2], E. Shelepova[1], I. Zazerskaya[1], G. Trufanov[1]; [1]St. Petersburg/RU, [2]Moscow/RU
*(kirillovaelizaveta30@gmail.com)*

**Purpose:** Placentomegaly may be associated with severe foetal and maternal complications. The aim of this study was to provide useful diagnostic information on the differential diagnosis of placentomegaly and to describe the specific features of thrombohaematoma-associated cases.
**Methods or Backround:** Placental thrombohaematomas (PTHs) represent rare cases of placentomegaly that are associated with poor pregnancy outcomes and can be classified as subamniotic, subchorionic, retroplacental or marginal haemorrhages.
**Results or Findings:** We encountered six cases of PTHs in pregnant women aged 29.50 ± 5.96 years. The diagnosis was unclear on a routine ultrasound exam and required additional confirmation using MRI of the placenta performed at 23.33 ± 2.58 weeks. Unfavourable pregnancy outcome (foetal demise; n=3) included (1) acute massive retroplacental haematomas with a fluid-fluid level as a result of a sedimentation effect, (2) in two cases, all four types of PTHs were observed, (3) the mean gestational age at the time of delivery was 22 weeks. Unfavourable pregnancy outcome (live births; n=3) involved (1) all three patients having subamniotic PTHs (massive [n=2] and local [n=1] PTHs) and placental villous oedema, (2) two cases of preterm birth at 27 GW (in both cases of massive PTHs), and one case of full-term birth at 37 GW, (3) the mean gestational age at the time of delivery was 31 weeks. Some newborns demonstrated congenital anomalies (n=2), neurologic deficits and gastrointestinal complications (n=3 each). Respiratory support was required in three cases.
**Conclusion:** MRI of the placenta is a valuable tool for the differential diagnosis of placentomegaly. Early diagnosis is important for risk stratification and prenatal outcome prediction. Special caution should be exercised in case of thrombohaematoma-associated placentomegaly as this finding may be associated with a higher risk of preterm birth and poor neonatal outcomes.
**Limitations:** No limitations were identified.
**Ethics committee approval:** Not applicable.
**Funding for this study:** No funding was provided for this study
**Author Disclosures:**
Elizaveta Aleksandrovna Kirillova: Nothing to disclose
Irina Mashchenko: Nothing to disclose
Lilia Kashapova: Nothing to disclose
Ekaterina Shelepova: Nothing to disclose
Irina Zazerskaya: Nothing to disclose
Polina Kozlova: Nothing to disclose
Tamta Mamiseishvili: Nothing to disclose
Gennadiy Trufanov: Nothing to disclose

### RPS 1107-7

**Magnetic resonance protocol without contrast to characterise adnexal masses indeterminate at ultrasonography: a multicentre study**

*C. Panico*, G. Avesani, V. Simeon, M. Mangialardi, P. Sciaccotta, B. Gui, E. Sala; Rome/IT
*(camilla.panico@policlinicogemelli.it)*

**Purpose:** Appropriate management of patients with adnexal masses needs an accurate characterisation. Up to 20% of adnexal masses remains uncharacterised on US and MRI is needed for full characterisation. The O–RADS MRI score relies on intravenous gadolinium contrast. The purpose of this study was to show interim data of a multicentre prospective external evaluation of the non-contrast MRI adnex Score (NCMS) in characterising the adnexal masses.
**Methods or Backround:** 73 patients from six Italian centres were evaluated. NCMS was used to predict the malignancy of the ovarian lesions. The score is based on five categories (four and five indicate malignancy). One senior and one junior radiologist per centre read the images, unaware of the patients' clinical information except for age. Presence of malignant tumours was verified during histopathology after surgery or during the radiological follow-up after 12 months. Diagnostic accuracy in terms of the percentage of correctly classified diagnoses was measured. Agreement between senior and junior radiologist has been evaluated performing kappa statistics.
**Results or Findings:** 26 patients were classified as malignant. Malignant lesions were correctly classified in 78.08% of cases (Conf. Int. 68.38% - 86.92%). The agreement between senior and junior radiologist was 93.15% (under an expected agreement of 53.74%), with a kappa statistic of 0.79 (Conf. Int. 0.59-0.985).
**Conclusion:** The results on the accuracy and reproducibility of the diagnosis are in line with the potential use of the NCMS in clinical practice when contrast medium cannot be used for logistic or patient factors.
**Limitations:** The limitation of this study is the number of observations being low (only 20% of the number of observations needed according to the sample size calculation); the expansion of the number of cases in this multicentric study will overcome this problem.
**Ethics committee approval:** The proposal to the Ethical Committee of Fondazione Policlinico Gemelli (coordinating center) was presented on June 21st, 2021 and approved. Each centre had their participation approved by their own ethical committee.
**Funding for this study:** No funding was received for this study.
**Author Disclosures:**
Vittorio Simeon: Nothing to disclose
Giacomo Avesani: Nothing to disclose
Pietro Sciaccotta: Nothing to disclose
Benedetta Gui: Nothing to disclose
Evis Sala: Nothing to disclose
Camilla Panico: Nothing to disclose
Matteo Mangialardi: Nothing to disclose

### RPS 1107-8

**Diagnostic value of magnetic resonance imaging with diffusion-weighted imaging for differentiating low-grade endometrial stromal sarcoma from benign atypical leiomyoma**

*S. Park*, S. E. Rha, H. Kim, Y. R. Shin; Seoul/KR
*(abba118@naver.com)*

**Purpose:** The purpose of this study was to evaluate the diagnostic value of known qualitative magnetic resonance imaging (MRI) features and diffusion-weighted imaging (DWI)-based quantitative parameters in the differentiation of uterine low-grade endometrial stromal sarcoma (LGESS) from benign atypical leiomyoma (ALM).
**Methods or Backround:** This retrospective study includes 113 women (ALM= 93, LGESS= 20) imaged with pelvic MRI with DWI prior to surgery. Two readers independently evaluated each lesion for conventional qualitative features as well as quantitative parameters including the mean ADC value and the relative contrast ratio (RCR) on high b-value DWI. The diagnostic performance of both qualitative and quantitative parameters in discriminating LGESS and ALM was assessed.
**Results or Findings:** The qualitative MRI features that significantly differed between LGESS and ALM were ill-defined or infiltrative margin (p=0.018), irregular or nodular shape (p<0.001), intratumoral low-SI band on T2WI (p<0.001), intramyometrial nodular extension (p< 0.001), nodule-in-nodule appearance (p= 0.017), and cystic changes (p= 0.010). The mean ADC value of LGESS ($0.99 \pm 0.29 \times 10^{-3} mm^2/s$) was significantly lower than that of ALM ($1.25 \pm 0.31 \times 10^{-3} mm^2/s$) (p< 0.001) and the mean RCR of LGESS was 8.3 ± 5.5, which was significantly higher than that of ALM, 4.2 ± 2.7 (p< 0.001).
**Conclusion:** Although there are substantial overlaps in conventional imaging features between LGESS and ALM, applying quantitative parameters based on DWI and ADC map in addition to qualitative imaging features may help distinguish LGESS from ALM.
**Limitations:** The study is retrospective. There are heterogeneous MRI acquisition parameters, but no major differences were detected in the protocols among different equipment. In addition, the study includes a relatively small number of patients with low-grade ESS. Nonetheless, it's important to highlight that low-grade ESS is an exceedingly rare tumor, and our research encompasses a more substantial number of cases compared to previous reports.
**Ethics committee approval:** The study was approved by the catholic university of Korea, IRB
**Funding for this study:** No funding was received for this study.
**Author Disclosures:**
Soomin Park: Nothing to disclose
Hokun Kim: Nothing to disclose
Sung Eun Rha: Nothing to disclose
Yu Ri Shin: Nothing to disclose

### RPS 1107-9

**The peripheral cysts sign of ovarian fibroma and fibrothecoma: incidence and diagnostic value**

*C. Li*, M. Sun, R. Xu, J. Guan; Guangzhou/CN

**Purpose:** The purpose of this study was to recognise and analyse the cause of “cysts margination” sign in ovarian thecoma-fibroma groups (OTFG). And to investigate the value of this specific sign in the differential diagnosis between OTFG and other ovarian cystic and solid tumours.
**Methods or Backround:** A total of 112 patients with surgically proven OTFG were screened consecutively from January 2016 to December 2022. Preoperative CT/MR examinations were reviewed by one radiologist with 20 years of experience in genital/reproductive imaging. The “cysts margination” sign was clearly recognised in 25 patients (group 1), while absent in the remaining 87 patients (group 2). Clinical data were recorded and compared between the two groups. Preoperative CT/MR imaging of 25 OTFG patients with “cysts margination” sign and 50 matched patients with surgically proven other ovarian cystic and solid tumours were retrospectively reviewed by two independent radiologists (five and nine years of experience) before and after learning the sign. The diagnostic accuracy of OTFG and other ovarian tumours was calculated.
**Results or Findings:** 22.3% (25/112) of patients with OTFG presented with “cysts margination” sign. Histopathology confirmed these peripheral cysts to be ovarian cysts. The age (56±15 vs 43±16, p= 0.02), menopausal state (p< 0.001), and presence of endometrium thickening (p< 0.001) were significantly different between the two groups. With the addition of the sign into radiological evaluation, the diagnostic accuracy of OTFG by two independent radiologists improved from 10% and 35% to 90% and 90%, respectively.
**Conclusion:** “Cysts margination” sign can be seen in around 20% of OTFG patients, mostly in peri- or post-menopausal state, and may be associated with the elevated oestrogen production of the tumour. Familiarity with this specific sign can significantly increase the diagnostic efficacy of OTFG in menopausal women, thereby avoiding unnecessary further examination or treatment.
**Limitations:** No limitations were identified.
**Ethics committee approval:** The study was approved by the First Affiliated Hospital, Sun Yat-Sen University
**Funding for this study:** No funding was received for this study.
**Author Disclosures:**
Chang Li: Nothing to disclose
Rulin Xu: Nothing to disclose
Jian Guan: Nothing to disclose
Mengya Sun: Nothing to disclose

### RPS 1107-10

**Hybrıd F18-FDG PET/MRI in endometrial cancer: staging accuracy, quantitative analysis, and imaging characteristics of histological subtypes**

*D. Kuru Öz*, M. Araz, S. N. Yılmazer Zorlu, B. Varlı, C. Soydal, S. Taşkın, E. Ozkan, G. A. Erden, O. Kücük; Ankara/TR
*(digdem_k@hotmail.com)*

**Purpose:** The aim of this study is to evaluate the diagnostic performance of F18-FDG PET/MRI in endometrial cancer (EC) staging, determine the individual contributions of PET and MRI, compare PET/CT and PET/MRI findings, quantitatively assess the primary tumor (PT) and metastatic lymph nodes (LN), and investigate the differences in quantitative measurements among histological subtypes.
**Methods or Backround:** Between February 2020 and March 2022, a total of 44 patients who were diagnosed with EC via biopsy were included in the study. Following whole-body PET/CT, pelvic PET/MRI was performed. For quantitative analysis, the apparent diffusion coefficient (ADC) values for PT and LN were measured with MRI, while the maximum standard uptake value (SUVmax), metabolic tumor volume (MTV), and total lesion glycolysis (TLG) values were measured with PET/CT and PET/MRI. For statistical analysis Spearman correlation and Mann-Whitney U tests were performed.

Thursday

**Results or Findings:** Out of the 44 patients, 39 (88.6%) had concordance between PET/MRI staging and pathological staging, while 5 (11.3%) were discordant. There were nine patients (20.4%) with pelvic and/or para-aortic LN metastasis. In three patients (6.8%) with suspected LN involvement on MRI, PET accurately predicted LN involvement with pathological activity. In LN evaluation, the sensitivity, specificity, positive predictive value, negative predictive value, and accuracy for PET/CT were 66.6%, 93.9%, 66.6%, 91.1%, and 88%, respectively. For PET/MRI, the corresponding values were 88.8%, 88.2%, 66.6%, 96.7%, and 88.3%, respectively. For both PT and pathological LN, no strong correlation was found between ADC and SUVmax values (r> -0.009, p> 0.095,respectively).
**Conclusion:** PET/MRI, can be used in preoperative staging for the accurate assessment of both the primary tumor and nodal involvement. It offers high negative predictive value and has the potential to prevent unnecessary lymph node dissections while providing a high diagnostic performance as a hybrid imaging method.
**Limitations:** The limitation of this study is the small sample size.
**Ethics committee approval:** The Institutional Ethics Committee approved this retrospective study protocol and waived informed consent
**Funding for this study:** No funding was received for this study.
**Author Disclosures:**
Sezer Nil Yılmazer Zorlu: Nothing to disclose
Diğdem Kuru Öz: Nothing to disclose
Cigdem Soydal: Nothing to disclose
Elgin Ozkan: Nothing to disclose
Mine Araz: Nothing to disclose
Ozlem Kücük: Nothing to disclose
Salih Taşkın: Nothing to disclose
Gül Ayşe Erden: Nothing to disclose
Bulut Varlı: Nothing to disclose

### RPS 1107-11

**Imaging of peritoneal metastases in ovarian cancer using MDCT, MRI and FDG PET/CT: a systematic review and meta-analysis**

*M. Tzoumpa*[1], T. Siempis[2], G. Alexiou[1], M. Argyropoulou[1], A. C. Tsili[1]; [1]Ioannina/GR, [2]Belfast/UK
*(martz_me@hotmail.com)*

**Purpose:** The purpose of this study was to compare the diagnostic performance of multidetector CT (MDCT), MRI including diffusion-weighted imaging and FDG PET/CT in the detection of peritoneal metastases (PMs) in ovarian cancer (OC) patients.
**Methods or Backround:** A comprehensive search was performed for articles published from January 2000 to February 2023, in PubMed database. Inclusion criteria were the following: diagnosis/suspicion of PMs in patients with ovarian/fallopian/primary peritoneal cancer; initial staging or suspicion of recurrence (primary outcome); MDCT, MRI and/or FDG PET/CT performed for the detection of PMs; population of at least 10 patients; at least three data sets available for each imaging modality; surgical results, histopathologic analysis and/or radiologic follow-up, used as reference standard; per-patient and per-region data; and data for calculating sensitivity and specificity reported.
**Results or Findings:** Thirty-three studies were assessed, including 487 women with OC and PMs. On a per-patient basis, MRI ($P = 0.03$) and FDG PET/CT ($P < 0.01$) had highest sensitivity compared to MDCT. MRI and PET/CT had comparable sensitivities ($P = 0.84$). On a per-lesion analysis, no differences in sensitivity estimates were noted between MDCT and MRI ($P = 0.25$), MDCT and FDG PET/CT ($P = 0.68$), MRI and FDG PET/CT ($P = 0.35$).
**Conclusion:** Based on our results, FDG PET/CT and MRI had a higher diagnostic performance in the detection of PMs compared to MDCT, on a per-patient analysis. No differences were found on a per-lesion basis.
FDG PET/CT and MRI can be considered equivalent alternatives for the detection of PMs in OC. MDCT can be used as an alternative.
**Limitations:** The limitations of this study are due to selection bias, and the heterogeneity of the studies.
**Ethics committee approval:** No information provided by the submitter.
**Funding for this study:** No funding was provided for this study.
**Author Disclosures:**
George Alexiou: Nothing to disclose
Timoleon Siempis: Nothing to disclose
Martha Tzoumpa: Nothing to disclose
Maria Argyropoulou: Nothing to disclose
Athina C. Tsili: Nothing to disclose

### RPS 1107-12

**The pattern of radiological first relapse in ovarian cancer differs between different maintenance therapies: a retrospective analysis of a tertiary referral centre**

*G. Avesani*, L. D'Erme, A. Iacono, C. Panico, S. Bottazzi, F. Capomacchia, C. Marchetti, B. Gui, E. Sala; Rome/IT
*(giacomo.avesani@gmail.com)*

**Purpose:** The aim of this study was to evaluate the radiological distribution of disease at the first recurrence in patients with advanced ovarian cancer treated with different maintenance therapies (PARP-inhibitors versus Bevacizumab).
**Methods or Backround:** We retrospectively collected data on patients with recurrent advanced ovarian cancer from January 2017 to December 2022, who had III-IV FIGO stage at the diagnosis, a complete debulking (primary or interval surgery) followed by adjuvant chemotherapy and maintenance therapy. We included those with an available contrast-enhanced CT at the recurrence time (clinical, serological or radiological). We evaluated whether the presence of recurrence was visible (visible/non-visible solid tissue), the type (macronodular/micronodular) and the number of localisations (oligometastatic (<4 sites)/multi-metastatic) and if the relapse was only in lymph nodes.
**Results or Findings:** We evaluated 80 patients; 40 had Bevacizumab and 40 had PARP-i. The incidence of recurrence without any clearly visible soft tissue was significantly higher during Bevacizumab (20% versus 10%; $p= 0.04$). Micronodular diffuse relapse was more frequent in patients treated with Bevacizumab (58% versus 30%; $p= 0.03$). Oligometastatic recurrence was more frequent in PARP-i (45% versus 24%; $p= 0.03$). No difference was found in only nodal relapse between the two groups (18% versus 15%, $p> 0.05$).
**Conclusion:** The recurrence pattern in ovarian cancer differs based on the administered maintenance therapy. Secondary cytoreduction, which can be performed in oligometastatic disease, is more frequent during PARP-i maintenance. Radiologists should know this to make the correct diagnosis, especially avoiding delayed diagnosis of recurrence in patients treated with Bevacizumab.
**Limitations:** This is a retrospective study with a small sample size.
**Ethics committee approval:** This study was approved with the approval code: 6031
**Funding for this study:** No funding was received for this study.
**Author Disclosures:**
Giacomo Avesani: Nothing to disclose
Filippo Capomacchia: Nothing to disclose
Benedetta Gui: Nothing to disclose
Alessandra Iacono: Nothing to disclose
Silvia Bottazzi: Nothing to disclose
Evis Sala: Nothing to disclose
Camilla Panico: Nothing to disclose
Luca D'Erme: Nothing to disclose
Claudia Marchetti: Nothing to disclose

### Friday, March 1

**08:00-09:00** **Research Stage 1**

### Research Presentation Session: Breast

### RPS 1202
### Artificial intelligence (AI) in breast imaging

**Moderator**
H. W. Koch; Stavanger/NO

### RPS 1202-2
**Adding artificial intelligence (AI) case malignancy scoring in a breast screening programme to reduce screen-reading workload: a retrospective study**

*A. Nitrosi*, P. Giorgi Rossi, L. Verzellesi, N. Cucurachi, R. Vacondio, C. Campari, M. Bertolini, P. Pattacini, M. Iori; Reggio Emilia/IT
*(nitrosi.andrea@ausl.re.it)*

**Purpose:** The purpose of this study was to evaluate a strategy of integrating AI mammography case malignancy score (AI-CMS) to reduce breast screen-reading workload avoiding human second reading for mammograms with low AI-CMS.
**Methods or Backround:** We retrospectively analysed 31,747 consecutively collected screening exams from Reggio Emilia breast screening program (BSP), including 92 proven tumours and 5 pending diagnoses, to assess decision to recall (RD), recall rate (RR) and tumour detection of two simulated integrated AI and human reading protocols (ProFound AI 2D system iCAD Inc.). iCAD AI-CMS is a relative score representing the AI algorithm's confidence that a case is malignant in a 0% to 100% scale. To estimate the potential reduction in the numbers of human readings, iCAD acts as a reader C1 recalling women with AI-CMS greater than a predefined threshold (10%/15%/20%). If the radiologist – reader RH1 - disagrees with iCAD, the case undergoes to another radiologist RH2 and to a third radiologist arbitration RH3 in case of human disagreement (standard screening protocol).
**Results or Findings:** Assuming respectively 10%/ 15%/ 20% AI-CMS threshold, RD for C1 was 49.4%/ 37.7%/ 29.7%, for RH1 4.6%/ 4.6%/ 4.6%, for RH2 8.7%/ 10.6%/ 12.5% and for RH3 69.9%/ 69.7%/ 69.1%. The final RR was 3.70%/ 3.62%/ 3.58% versus actual RR of 3.86%. This corresponds to 48,975/ 45,180/ 42,785 versus 65,097 total human readings (corresponding to human workload reduction of 24.8%/ 30.6%/ 34.3%). There's no increase in false negative with 10% and 15% thresholds, whereas using the 20% threshold results in an additional false negative.
**Conclusion:** Adding AI-CMS support to a standard screening scenario could result in a substantial lower screen reading workload, a modest decrease in RR without any additional false negatives.
**Limitations:** The study only examines data from the Reggio Emilia BSP.
**Ethics committee approval:** Compliance with Ethical Standards Institutional Review Board approval was not required because it is a Clinical Audit about a technical development. This study was conducted in accordance with the routine quality assurance procedures established by the Local Health Authority for its screening programs. The Reggio Emilia Cancer Registry, which routinely collects the screening history of each case of breast cancer, has been approved by the Provincial Ethic Committee.
**Funding for this study:** This study was partially supported by the Italian Ministry of Health—Ricerca Corrente
**Author Disclosures:**
Laura Verzellesi: Nothing to disclose
Cinzia Campari: Nothing to disclose
Marco Bertolini: Nothing to disclose
Pierpaolo Pattacini: Nothing to disclose
Noemi Cucurachi: Nothing to disclose
Andrea Nitrosi: Nothing to disclose
Mauro Iori: Nothing to disclose
Rita Vacondio: Speaker: Invited speaker
Paolo Giorgi Rossi: Nothing to disclose

### RPS 1202-3
**An artificial intelligence tool to empower junior radiologists in breast cancer screening; AI as a second pair of eyes in mammography reading**

N. Ahmadinezhad, N. Sadighi, R. Ghavami Modegh, *M. Arab Ahmadi*, M. Rahmani, A. Arian, H. Dashti, H. R. Rabiee, M. Gity; Tehran/IR
*(mehran_arabahmadi@yahoo.com)*

**Purpose:** The aim of this study was to assess whether an artificial intelligence (AI) application can assist non-expert radiologists in improving their performance in detecting the possibility of cancer using digital mammography in an adjunctive workflow.
**Methods or Backround:** A retrospective study was conducted using 2060 digital mammography (DM) images of 515 women from 2018-2022, including 120 positive and 910 negative breasts, with four junior radiologists participating in the study. Radiologists independently reviewed and interpreted each case without AI assistance. Immediately after submitting their initial interpretations, they were provided with AI-generated interpretations to support their analysis and evaluation of the cases. Armed with this additional information, the radiologists had the opportunity to revise and resubmit their interpretations based on their expertise and insights from the AI system. Radiologists' performance before and after receiving AI assistance was compared using AUC, sensitivity, and specificity metrics.
**Results or Findings:** According to our findings, the integration of AI technology resulted in a notable enhancement in the performance of junior radiologists. The area under the curve (AUC) improved significantly from 0.812 to 0.837, sensitivity increased from 75.0% to 92.9%, and balanced accuracy enhanced by 3.63%. Additionally, AI proved to be highly beneficial for radiologists in identifying previously missed lesions across various types, including mass, calcification, distortion, and asymmetry.
**Conclusion:** AI can improve the diagnostic capabilities and detection rates of radiologists with less than five years of experience, enhancing their medical imaging performance.
**Limitations:** One of the limitation of this study is that we did not assess the effect of AI on final recall rates, which would require a live large-scale survey with a normal distribution to yield reliable results.
**Ethics committee approval:** The clinical study was conducted under the Research Ethic Certificate No. IR.TUMS. IKHC.REC.1399.490 issued by Tehran University of Medical Sciences on Feb. 24, 2021.
**Funding for this study:** No funding was received for this study.
**Author Disclosures:**
Nahid Sadighi: Nothing to disclose
Masoumeh Gity: Nothing to disclose
Nasrin Ahmadinezhad: Nothing to disclose
Hamid Reza Rabiee: Nothing to disclose
Maryam Rahmani: Nothing to disclose
Arvin Arian: Nothing to disclose
Rassa Ghavami Modegh: Nothing to disclose
Hamed Dashti: Nothing to disclose
Mehran Arab Ahmadi: Nothing to disclose

### RPS 1202-4
**Is it worth reading low-risk breast cancer screening mammograms as determined by an artificial intelligence (AI) system? A prospective, population-based study for DM and DBT (AITIC trial)**

*E. Elías Cabot*[1], S. Romero Martin[1], J. L. Raya Povedano[1], A. Rodriguez Ruiz[2], M. Álvarez Benito[1]; [1]Cordoba/ES, [2]Nijmegen/NL
*(eeliascabot@gmail.com)*

**Purpose:** The purpose of this study was to prospectively evaluate AI for safe workload reduction by excluding low risk cases for human reading and applying double reading to the rest in screening with digital mammography (DM) and digital breast tomosynthesis (DBT).
**Methods or Backround:** Participants in a breast cancer screening programme in Córdoba, Spain, (women, aged 50-71) are included in this prospective study and imaged with either DM or DBT. Two reading strategies are independently applied to each exam: Double blind and non-consensual reading of all exams (control arm) and an AI-based triaging (intervention arm), where an AI system (Transpara, ScreenPoint Medical) evaluates the cancer risk of all exams. Cases identified by AI as low risk (operating point pre-defined to yield approximately 70% of exams in this category) are automatically assessed as negative, while cases with intermediate and elevated risk are double read with AI-support. Readers are randomly assigned to each reading and blinded to other reading outcomes. We hypothesise that an AI-based screening workflow allows for substantial workload reduction and non-inferior cancer detection rate (CDR) and recall positive predictive value (PPV).
**Results or Findings:** Between March 2022 and June 2023, 19243 women participated. AI-based triaging, reading only 6583 exams (the 34% of the total scored by AI as intermediate and elevated risk), achieved superior CDR compared to double-reading of all cases (CDR 6.7/1000, 130 cancers vs. 5.9/1000 (114 cancers), p= 0.017), non-inferior recall PPV (12.6% [10.6-14.8%], 130/1032, versus 12.0% [10.0-14.3%], 114/947, p= 0.699), and increased RR (5.4% [5.0-5.7%], versus 4.9% [4.6-5.2%], p= 0.016).
**Conclusion:** AI-based triaging, excluding low risk mammograms from human reading, leads to a substantial reduction in reading workload in breast cancer screening without negatively affecting performance.
**Limitations:** The results include 70% of the target population (27000 women). We expect to complete the study in February 2024.
**Ethics committee approval:** Approval granted on 30.03.2021.
**Funding for this study:** Funding was received from the SEDIM foundation grant, to the value of 20.000 euros.

**Author Disclosures:**
Alejandro Rodriguez Ruiz: Employee: screenpoint medical employee
Sara Romero Martin: Nothing to disclose
Marina Álvarez Benito: Nothing to disclose
Esperanza Elías Cabot: Nothing to disclose
Jose Luis Raya Povedano: Nothing to disclose

### RPS 1202-5

**Correlating breast lesions in tomosynthesis CC and MLO views using artificial intelligence (AI)**

*S. M. Friedewald*[1], A. Dsouza[2], C. Parghi[3], A. Kshirsagar[2]; [1]Chicago, IL/US, [2]Marlborough, MA/US, [3]Houston, TX/US
*(sarah.friedewald@nm.org)*

**Purpose:** The purpose of this study was to evaluate a deep learning (DL) model for matching regions of interest (ROI) corresponding to the same lesion on tomosynthesis craniocaudal (CC) and mediolateral oblique (MLO) views
**Methods or Backround:** A CC-MLO lesion correlation system (CMCS) was developed to automatically match ROIs flagged by an AI breast lesion detection algorithm (Genius AI Detection, Hologic) in both CC and MLO views. The system combines geometric information with similarity between pairs of ROIs to assign a lesion correlation score. ROI pairs above a pre-defined threshold are presented to the reader for potential workflow enhancement. 864 consecutive subjects with biopsy proven malignant cancers were collected retrospectively under an IRB approved protocol from two large multi-centre breast imaging networks and one breast imaging facility at an independent cancer centre. Ground truth was determined by an expert using image available data. The pairs of ROIs flagged by the AI algorithm on malignant lesions were analysed by the CMCS system and compared with ground truth to estimate accuracy.
**Results or Findings:** Out of 864 patients with biopsy-proven malignancies, 614 lesion ROI pairs identified by experts were detected by the AI algorithm on both views. Analysis of these by CMSC resulted in 555 correctly matched pairs, resulting in an overall accuracy for all findings of 90.4% (95% CI: 88.1, 92.7) for biopsy-proven cancer cases.
**Conclusion:** The CC-MLO lesion correlation system was able to correctly match pairs of ROIs in CC and MLO views over 90% of the time for biopsy proven malignant lesions that were correctly flagged by the AI algorithm. This matching algorithm can be used to assist radiologists in triangulating one-view findings in the orthogonal view.
**Limitations:** This study is retrospective and not performed in a clinical setting.
**Ethics committee approval:** Not applicable.
**Funding for this study:** Funding was received from Hologic Inc.
**Author Disclosures:**
Ashwini Kshirsagar: Employee: Hologic, Inc.
Sarah Maier Friedewald: Research/Grant Support: Google; Research/Grant Support: Hologic, Inc.
Chirag Parghi: Nothing to disclose
Adora Dsouza: Employee: Hologic, Inc.

### RPS 1202-6

**Evaluating performance of an artificial intelligence (AI) detection system on prior screening tomosynthesis studies of breast cancer patients**

*S. M. Friedewald*[1], B. Shi[2], C. Parghi[3], A. Kshirsagar[2]; [1]Chicago, IL/US, [2]Marlborough, MA/US, [3]Houston, TX/US
*(sarah.friedewald@nm.org)*

**Purpose:** The purpose of this study was to evaluate an AI detection system in identifying breast cancer in up to two prior screening tomosynthesis studies in patients with biopsy-proven cancer detected during their most recent screening examinations.
**Methods or Backround:** Tomosynthesis screening and diagnostic studies with one or two associated prior screening examinations acquired between 2014 and 2021, were consecutively collected from 814 biopsy-proven cancer patients. At least one prior screening study (prior1) was available for 814 patients while two prior studies (prior1 and prior2) were available for 272. In the index exam where the cancer was mammographically detected, the cancer was annotated by an expert using all available data. The annotator also retrospectively reviewed prior1 and prior2 examinations and annotated corresponding lesions if visible irrespective of actionability. The AI algorithm (Genuis AI Detection 2.0, Hologic) independently analysed tomosynthesis examinations and marked potentially malignant findings with a score corresponding to the overall level of suspicion. Study-level sensitivity was calculated by comparing the location of any AI marks with ground truth.
**Results or Findings:** Sensitivity for cancer in 814 cases was 90.7% (738/814, 95% CI:88.6%-92.6%) in index studies. Sensitivity was 89.9% for studies with non-calcified malignancies and 92.6% for studies with malignant calcifications. Sensitivity for retrospectively visible findings amongst prior1 studies was 68.5% (341/498, 95%CI:63.1%-74.2%) and amongst the prior2 studies was 48.8% (79/162, 95%CI:39.9%-57.6%). For the 272 patients having 2 prior studies, average AI case score was 34.7 (SD:28.7) for prior2 studies, 40.9 (SD: 26.8) for prior1 study, and 63.8 (SD 24.7) for index studies diagnosed with biopsy-proven cancers.
**Conclusion:** This AI system can assist in identifying cancer on prior mammograms interpreted as normal. The temporal increase in case score for each study potentially correlates with cancer progression.
**Limitations:** This is a retrospective study.
**Ethics committee approval:** Not applicable.
**Funding for this study:** Funding was received from Hologic, Inc.
**Author Disclosures:**
Bibo Shi: Employee: Hologic, Inc.
Ashwini Kshirsagar: Employee: Hologic, Inc.
Sarah Maier Friedewald: Research/Grant Support: Google; Research/Grant Support: Hologic, Inc.
Chirag Parghi: Nothing to disclose

### RPS 1202-7

**AI detection of interval cancers: does size, grade, and time since screening affect sensitivity?**

*M. Nanaa*, T. van Nijnatten, N. Stranz, S. Carriero, N. Payne, I. Allajbeu, E. Giannotti, R. Manavaki, F. Gilbert; Cambridge/UK

**Purpose:** The objective of this study was to evaluate AI detection of interval cancers (IC) on screening mammograms by tumour size, grade, and time since screening.
**Methods or Backround:** Two radiologists (8 and 3–13 years' experience) classified 488 ICs (2011–2018) as visible or non-visible on screening mammography. Tumour volume doubling time (TVDT) was calculated for visible cancers [TVDT=ln (2). Δt/3. (ln d1-ln d2)], with the median TVDT for grade and receptor status of visible cancers used as a surrogate to estimate cancer size for non-visible cancers [T(SS)=T(SD)×e-(ln(2)/TVDT)×Δt], T(SS): tumour size screening, T(SD): tumour size diagnosis. The sensitivity of a commercial AI algorithm was analysed by tumour size, grade, receptor status, and time from screen to diagnosis at its default threshold for cancer detection (score 10).
**Results or Findings:** Median screening size was 12 mm (IQR 9–18) for visible cancers (280/488), with median estimated size 2.65 mm (IQR 1.26–5) for non-visible cancers (208/488). AI detected 58.2% (163/280) of visible and 30.7% (64/208) of non-visible cancers, p<0.001. AI localised 58.4% (31/53) of grade 1, 46.3% (103/222) of grade 2, 43.2% (87/201) of grade 3 cancers, p=0.14, 49.3% (195/395) of ER-positive cancers and 31.7% (27/85) of ER-negative cancers, p=0.003. The median time to interval was 666 days (IQR 405–895) for localised cancers and 708 days (IQR 480–929) for non-localised, p=0.057. The median size was 13 mm (IQR 9–19) and 12 mm (IQR 8–17) for localised and non-localised visible cancers, p=0.027, and 3.15 mm (IQR 1.93–5.51) and 2.27 mm (IQR 0.97–4.25) for non-visible cancers, p=0.002, respectively. Sensitivity for cancers <5mm, 5–9.9mm, and >=10mm was 33.3% (1/3), 49.4% (43/87), 62.6% (119/190) for visible; 27% (42/155), 32.2% (10/30), 54.5% (12/22) for non-visible.
**Conclusion:** AI is more likely to detect larger, ER-positive cancers, with a trend towards grade 1.
**Limitations:** The limitations of this study were the single site; tumour size was estimated in 42.6% of cases.
**Ethics committee approval:** This retrospective study used anonymised mammograms from two National Health Service Breast Screening Programme (NHSBSP) centres under ethical approval [Health Research Authority Research Ethics Committee (HRA REC) 20/LO/0104, HRA Confidentially Advisory Group (CAG) 20/CAG/0009, and Public Health England (PHE) Research Advisory Committee (RAC) BSPRAC_090].
**Funding for this study:** This research was supported by the NIHR Cambridge Biomedical Research Centre (BRC-1215-20014) and the CRUK early detection programme grant. The views expressed are those of the authors and not necessarily those of the NIHR or the Department of Health and Social Care. Council for At-Risk Academics (Cara) funded the research fellowship for M.N. (award no. 210211). We would like to thank the company for taking part in this study.
**Author Disclosures:**
Thiemo van Nijnatten: Nothing to disclose
Roido Manavaki: Nothing to disclose
Muzna Nanaa: Nothing to disclose
Iris Allajbeu: Nothing to disclose
Fiona Gilbert: Nothing to disclose
Elisabetta Giannotti: Nothing to disclose
Nicholas Payne: Nothing to disclose
Natalia Stranz: Nothing to disclose
Serena Carriero: Nothing to disclose

### RPS 1202-8

**How much has AI improved over the last five years? A benchmark evaluation of different versions of an AI mammography interpretation system**

*A. Rodriguez Ruiz*, A-K. Brehl, N. Karssemeijer, I. Sechopoulos, R. M. Mann; Nijmegen/NL

**Purpose:** The study aimed to retrospectively evaluate the breast cancer detection performance of different versions of the same mammography AI system developed since 2018.
**Methods or Backround:** Two enriched datasets (A: 60 exams, 24 cancers, read by 107 radiologists; B: 60 exams, 20 cancers, read by 73 radiologists) and one consecutively collected double-read screening dataset (22,961 exams with 370 cancers, including 163 screen-detected, 48 interval, and 159 next-round screen-detected) were gathered. All exams and radiologists are part of the Dutch breast cancer screening program. Each exam was processed by four versions of the same AI system (v1.3, v1.5, v1.7, and Beta-2023, Transpara, ScreenPoint Medical), developed between 2018-2023. All exams were independent from the AI development process. The sensitivity of AI was compared to that of the radiologists using the average radiologist specificity on each dataset, using parametric T-tests.
**Results or Findings:** In dataset A, the average radiologist specificity and sensitivity was 92% (CI: 91%-94%) and 83% (CI: 81%-85%). At this specificity, AI system versions v1.3-v1.7 achieved sensitivities ranging from 62% to 79%, while Beta-2023 achieved 88% sensitivity (CI: 68%-97%, P=0.92). In dataset B, the average radiologist specificity and sensitivity was 80% (CI: 78%-83%) and 85% (CI: 83%-88%). AI v1.3-v1.7 versions achieved 75%-85% sensitivity, while Beta-2023 achieved 95% sensitivity (CI: 75%-99%, P=0.73). In the screening dataset, the average radiologist specificity was 97.7% (CI: 97.4%-97.8%). Due to interval and next-round cancers, average radiologist sensitivity was only 40% (CI: 35%-45%). AI Beta-2023 achieved a sensitivity of 48% (CI: 43%-53%), statistically higher sensitivity than average single reading (P=0.002).
**Conclusion:** AI systems are continuously improving performance. In the evaluated system, the breast cancer detection performance has improved over time to surpass that of an average radiologist.
**Limitations:** An identified limitation was that the study includes data from a single country.
**Ethics committee approval:** Data was collected following IRB waiver at the institution.
**Funding for this study:** No funding was received for this study.
**Author Disclosures:**
Alejandro Rodriguez Ruiz: Employee: ScreenPoint Medical
Ritse Maarten Mann: Advisory Board: ScreenPoint Medical
Ioannis Sechopoulos: Nothing to disclose
Nico Karssemeijer: Founder: ScreenPoint Medical
Anne-Kathrin Brehl: Employee: ScreenPoint Medical

**08:00-09:00** | **Research Stage 2**

### Research Presentation Session: Artificial Intelligence & Machine Learning & Imaging Informatics

### RPS 1205

### Recent development in AI for lung nodule detection

**Moderator**
G. Cicchetti; Rome/IT
*(cicchetti.giuseppe88@gmail.com)*

### RPS 1205-2

**External validation of the Sybil risk model as a tool to identify low-risk individuals eligible for biennial lung cancer screening**

*F. Van der Graaf*[1], N. Antonissen[1], Z. Saghir[2], C. Jacobs[1], M. Prokop[1]; [1]Nijmegen/NL, [2]Hellerup/DK
*(fennie.vandergraaf@radboudumc.nl)*

**Purpose:** Lung cancer screening protocols for follow up intervals should minimise harm, maximise cost-effectiveness, and avoid diagnostic delays. ILST suggests biennial follow-up for low-risk participants. The study aimed to retrospectively evaluate Sybil, a deep learning algorithm predicting lung cancer risk for 6 years from one LDCT, comparing it to PanCan2b for identifying biennial screening eligibility.
**Methods or Backround:** DLCST baseline scans included 1870 non-cancer and 25 screen-detected cancer cases, diagnosed within 2 years. Sybil (scan level) and PanCan2b (per nodule) predicted risk of developing cancer within 2 years. For cases with no screen-annotated nodules, the PanCan2b risk score for participants was set as 0%. For both models, we used a nodule-risk cut-off of <1.5% to identify low-risk participants for biennial follow-up, based on ILST. For PanCan2b, the risk dominant nodule per scan was considered.
**Results or Findings:** The Sybil and PanCan2B models identified 1616 and 1697 individuals, respectively, meeting the criteria for biennial screening. This would result in a reduction of 87% and 94% of CT scans in the second screening round, respectively. The group referred for biennial screening included 8 and 9 cancers for Sybil and PanCan2B, respectively.
**Conclusion:** Both Sybil and PanCan2B selected a large group of low-risk participants for biennial screening when a <1.5% risk threshold was used at baseline CT. The difference between Sybil and the PanCan2b model is small. More research is needed to study the type of cancers with delayed diagnosis and whether such delay leads to diagnostic stage shift. In addition, more external validation of the Sybil model on other datasets is necessary to further assess its applicability in lung cancer screening, and to evaluate its performance on follow-up imaging.
**Limitations:** This study is a baseline, retrospective analysis on data from one screening trial.
**Ethics committee approval:** The study included data collected from the Danish Lung Cancer Screening Trial (DLCST). For DLCST, the Ethics Committee of Copenhagen county approved the study, and informed consent was obtained from all participants.
**Funding for this study:** Funding for this study is supplied by a research grant that is funded by the Dutch Science Foundation and Mevis Medical Solutions, Bremen, Germany
**Author Disclosures:**
Fennie Van der Graaf: Research/Grant Support: Dutch Science Foundation (NWO); Research/Grant Support: MeVis Medical Solutions, Bremen, Germany; Research/Grant Support: Dutch Ministry of Economic Affairs
Mathias Prokop: Other: Royalties to institution from MeVis Medical Solutions AG, Bremen, Germany; Research/Grant Support: MeVis Medical Solutions AG, Bremen, Germany, and Siemens Healthineers, Erlangen, Germany, and Canon Medical Systems
Zaigham Saghir: Nothing to disclose
Noa Antonissen: Nothing to disclose
Colin Jacobs: Research/Grant Support: MeVis Medical Solutions AG, Bremen, Germany, and Philips Medical Systems, Best, Netherlands; Other: Royalties to institution from MeVis Medical Solutions AG, Bremen, Germany

### RPS 1205-3

**Artificial intelligence: the key to significant reduction in baseline LDCT lung cancer screening associated workload when used as a first-read filter**

*H. L. Lancaster*[1], B. Jiang[1], M. Silva[2], J. W. Gratema[3], D. Han[1], J. Field[4], G. De Bock[1], M. A. Heuvelmans[1], M. Oudkerk[1]; [1]Groningen/NL, [2]Parma/IT, [3]Apeldoorn/NL, [4]Liverpool/UK
*(h.l.lancaster@umcg.nl)*

**Purpose:** Artificial intelligence (AI) is not a new concept in the field of lung cancer screening. To date, AI has predominantly been used to predict lung nodule malignancy risk (rule-in principle). However, to have an impact on reducing radiologist workload, a new rule-out approach is needed. This study aimed to evaluate if AI can be used as a first-read filter to rule-out negative cases (nodules <100 mm3), so that radiologists would only need to assess indeterminate-positive nodules, therefore significantly reducing workload.
**Methods or Backround:** External validation of AI (AVIEW_LCS, v1.1.39.14) was performed in a UKLS dataset containing 1254 LDCT-baseline scans. Scans were assessed independently by four manual readers and AI. Discrepancies between reads (manual/AI) were reviewed by a consensus panel of two experienced thoracic radiologists, blinded from the original results. Final classification was based on the consensus reference read. Cases were ultimately classified as correct, positive-misclassifications (PMs) (nodules classified by the reader/AI as ≥100 mm3, but at consensus <100 mm3) and negative-misclassifications (NMs) (nodules classified by the reader/AI as <100 mm3, but at consensus ≥100mm3).
**Results or Findings:** Based on consensus reference read, 816 (65%) cases were negative and 438 (35%) indeterminate-positive. AI had fewer NMs 68 (5%) than all manual readers [reader 1; NMs 205 (16%), reader 2; NMs 200 (16%), reader 3; NMs 236 (19%), reader 4; NMs 220 (18%)], which was reflected in an AI negative predictive value (NPV) of 91.7% (89.8-91.4%) [reader1; 79.0% (77.5-82.0%), reader 2; 80.1% (81.3-85.4%), reader 3; 77.5% (76.0-79.0%) and reader 4; 78.5% (77.0-80.0%). Workload reduction using AI was calculated at 65% [(total scans;1254-(correct positives; 370 + positive misclassifications; 59))/total scans;1254].
**Conclusion:** AI negative predictive performance is better than all manual readers. If used as a first-read filter, radiologists would only need to assess 35% of cases with indeterminate-positive nodules, meaning significant workload reduction.

**Limitations:** An identified limitation of this study was that true positives and negatives based on histological outcome were not reported, analyses will begin shortly.
**Ethics committee approval:** The UKLS study was approved by an ethics committee; this substudy was covered by previous approval as it only included de-identified data.
**Funding for this study:** No funding was received for this study.
**Author Disclosures:**
Matthijs Oudkerk: Nothing to disclose
Marjolein A. Heuvelmans: Nothing to disclose
Harriet Louise Lancaster: Nothing to disclose
Geertruida De Bock: Nothing to disclose
Mario Silva: Nothing to disclose
John Field: Nothing to disclose
Daiwei Han: Nothing to disclose
Jan Willem Gratema: Nothing to disclose
Beibei Jiang: Nothing to disclose

### RPS 1205-4

**Can an AI-driven decision aid reduce the time between chest x-rays and treatment for lung cancer patients?**
A. Keen, S. Wilkie, B. E. Morrissey, S. Prior, *L. Cameron*; Aberdeen/UK
*(lorna.cameron1@nhs.scot)*

**Purpose:** The primary aim of this study was to evaluate whether an AI product with the ability to identify chest x-ray (CXR) images of highest risk of lung cancer can reduce the time between imaging and treatment in those patients subsequently diagnosed with lung cancer.
**Methods or Backround:** The NHS Grampian Innovation, Radiology and Cancer Teams collaborated with the Centre for Sustainable Delivery and the Scottish Health Technology Group to design an evaluation of the real-world impact of using an AI product designed to risk stratify CXR images. Full pathway mapping was carried out and baseline time delays between all key points (CXR, CXR reporting, CT, CT reporting, MDT diagnosis and treatment) were established. CXR images flagged as highest risk of lung cancer were expedited for CXR reporting, CT and CT reporting. NHS Grampian radiologists collaborated with the company to calibrate the product in ways that maximised identification of lung cancer whilst not overwhelming CT capacity.
**Results or Findings:** Several months into the project the time between CXR and CT report has dropped from 22 to 10.3 days (N=132). Radiologists identified 28 images not flagged by the product about which they were concerned about cancer. Thus far, none of these patients have been diagnosed with cancer. Under the current calibration conditions, using radiologists' judgements, the product performs at 84.4 sensitivity and 90.5 specificity (N=24071).
**Conclusion:** Early results suggest AI risk stratification of CXR images may help healthcare organisations reduce the time taken to treat people diagnosed with lung cancer. This could be especially important for people who are diagnosed following CXR imaging for non-cancer reasons. In our region, this is about two thirds of people diagnosed with lung cancer.
**Limitations:** A limitation of this study is that these are early results from a 12 month evaluation.
**Ethics committee approval:** We were advised by our Research and Development Office that permissions were not necessary on this occasion. Local and national clinical governance and executive-level sponsorship is in place.
**Funding for this study:** Funding was received from the Scottish Government.
**Author Disclosures:**
Sasia Prior: Nothing to disclose
Struan Wilkie: Nothing to disclose
Andrew Keen: Nothing to disclose
Brian E Morrissey: Nothing to disclose
Lorna Cameron: Nothing to disclose

### RPS 1205-5

**Comparison of the impact of reconstruction kernels on pulmonary nodule volumetry in low-dose CT with iterative vs deep learning image reconstruction**
*L. D'hondt*[1], C. Franck[2], P-J. Kellens[1], F. Zanca[3], D. Buytaert[4], K. Bacher[1], A. Snoeckx[2]; [1]Ghent/BE, [2]Antwerp/BE, [3]Leuven/BE, [4]Aalst/BE
*(louisdho.dhondt@ugent.be)*

**Purpose:** The objective of the study was to investigate the impact of different reconstruction kernels and its interaction with other imaging parameters on nodule volumetry, since scan protocols, screening guidelines and vendor specifications typically define the soft kernel as the standard, thereby disregarding its potential influence.
**Methods or Backround:** We scanned the Lungman phantom containing 3D-printed lung nodules, encompassing six diameters (4 to 9 mm) and three morphology classes (lobular, spiculated, smooth), using a 256-slice CT scanner at various radiation doses (CTDIvol 6.04, 3.03, 1.54, 0.77, 0.41, 0.20 mGy) and reconstructed using different combinations of either soft or hard reconstruction kernels and iterative reconstruction (IR) or deep learning image reconstruction (DLIR) at varying strengths. The impact of these imaging parameters on semi-automatic volumetry measurements was analysed through multiple linear regression.
**Results or Findings:** We found that reconstruction kernel significantly impacts volumetric accuracy, both as primary factor and in interaction with the reconstruction algorithm and radiation dose. Overall, volumetric errors are lower with the soft kernel compared to the hard kernel. Additionally, we observed that the soft kernel exhibited reduced errors with increasing radiation dose, while this remained relatively constant across all doses for the lung kernel. Combination of the lung kernel with DLIR resulted in relative reduction in volumetric error up to 50% as opposed to IR, at all doses. Furthermore, this effect became more pronounced as the DLIR strength increased. Across all nodule morphologies and diameters using the lung kernel, DLIR consistently outperformed IR, with relative reductions between 20 and 90% in error.
**Conclusion:** Compared to other combinations of reconstruction algorithms and kernels, application of DLIR in combination with a hard kernel overall returns the highest volumetric accuracy for all pulmonary nodules, also at (ultra)low radiation doses.
**Limitations:** An identified limitation is that this is a phantom study.
**Ethics committee approval:** No ethics committee approval was needed, since this study used phantom images.
**Funding for this study:** Funding was provided by the FWO "Kom op tegen Kanker" project for lung cancer screening research in Belgium (Project number: G0B1922N).
**Author Disclosures:**
Caro Franck: Nothing to disclose
Klaus Bacher: Nothing to disclose
Louise D'hondt: Nothing to disclose
Annemiek Snoeckx: Nothing to disclose
Dimitri Buytaert: Nothing to disclose
Federica Zanca: Nothing to disclose
Pieter-Jan Kellens: Nothing to disclose

### RPS 1205-6

**Improving image quality of sparse-view lung cancer CT images using convolutional neural networks**
*T. Dorosti*[1], A. Ries[2], J. B. Thalhammer[1], A. Sauter[1], F. Meurer[1], T. Lasser[2], F. Pfeiffer[1], F. Schaff[2], D. Pfeiffer[1]; [1]Munich/DE, [2]Garching/DE

**Purpose:** This study aimed to improve the image quality of sparse-view computed tomography (CT) images with a U-Net for lung cancer detection and to determine the best trade-off between number of views, image quality, and diagnostic confidence.
**Methods or Backround:** CT images from 41 subjects (34 with lung cancer, seven healthy) were retrospectively selected (01.2016-12.2018) and forward projected onto 2048-view sinograms. Six corresponding sparse-view CT data subsets at varying levels of undersampling were reconstructed from sinograms using filtered back projection with 16, 32, 64, 128, 256, and 512 views, respectively. A dual-frame U-Net was trained and evaluated for each subsampling level on 8,658 images from 22 diseased subjects. A representative image per scan was selected from 19 subjects (12 diseased, seven healthy) for a single-blinded reader study. The selected slices, for all levels of subsampling, with and without post-processing by the U-Net model, were presented to three readers. Image quality and diagnostic confidence were ranked using pre-defined scales. Subjective nodule segmentation was evaluated utilising sensitivity (Se) and Dice Similarity Coefficient (DSC) with 95% confidence intervals (CI).
**Results or Findings:** The 64-projection sparse-view images resulted in Se=0.89 and DSC=0.81 [0.75, 0.86], while their counterparts, post-processed with the U-Net, had improved metrics (Se=0.94, DSC=0.85 [0.82, 0.87]). Fewer views lead to insufficient quality for diagnostic purposes. For increased views, no substantial discrepancies were noted between the sparse-view and post-processed images.
**Conclusion:** Projection views can be reduced from 2048 to 64 while maintaining image quality and the confidence of the radiologists on a satisfactory level.
**Limitations:** The sparse-view data generated for this study was obtained using simplified conditions not reflective of the complex reconstruction processes in clinical settings. Therefore, an exact measure of dose reduction is hence unachievable.
**Ethics committee approval:** The study was approved by the ethical review committee and was conducted in accordance with the regulations of our institution. All data was analysed retrospectively and anonymously.

**Funding for this study:** Funding was received from the Federal Ministry of Education and Research (BMBF) and the Free State of Bavaria under the Excellence Strategy of the Federal Government and the Länder, the German Research Foundation (GRK2274), as well as by the Technical University of Munich - Institute for Advanced Study.
**Author Disclosures:**
Andreas Sauter: Nothing to disclose
Tina Dorosti: Nothing to disclose
Johannes Benedikt Thalhammer: Nothing to disclose
Felix Meurer: Nothing to disclose
Annika Ries: Nothing to disclose
Franz Pfeiffer: Nothing to disclose
Florian Schaff: Nothing to disclose
Daniela Pfeiffer: Nothing to disclose
Tobias Lasser: Nothing to disclose

### RPS 1205-7

**Head-to-head validation of AI software for the detection of lung nodules in chest radiographs: Project AIR**

K. G. van Leeuwen[1], *S. S. Schalekamp*[1], M. J. Rutten[1], M. Huisman[1], C. M. Schaefer-Prokop[2], M. De Rooij[1], B. Van Ginneken[1]; [1]Nijmegen/NL, [2]Amersfoort/NL
*(steven.schalekamp@gmail.com)*

**Purpose:** Multiple commercial artificial intelligence (AI) products exist for the detection of lung nodules on chest radiographs, however, comparative performance data of the algorithms is limited. The purpose of the study was to perform independent stand-alone comparison of commercially available AI products for lung nodule detection on chest radiographs, benchmarked against human readers.
**Methods or Backround:** This retrospective, multicentre (n=7 Dutch hospitals) study was carried out as part of Project AIR, which is a Dutch initiative for independent, repeatable, multicentre validation of AI products in radiology. Seven out of 14 eligible AI products for the detection of lung nodules on chest radiographs were validated on a dataset of 386 chest radiographs. The reference was chest CT within 3 months of the chest radiograph. Performance was measured using area under the receiver operating characteristic curve (AUROC). Random subsets of chest radiographs (n=140) were read by 17 human readers, with varying levels of experience.
**Results or Findings:** Seven lung nodule detection products were validated on chest radiographs (January 2012 to May 2022) of 386 patients (mean age, 64 years ± 11 [SD]; 223 males). Compared to human readers (mean AUROC, 0.81 [95% CI: 0.77, 0.85]), four products performed better (AUROC range, 0.86-0.93 [95% CI: 0.82, 0.96]; P range, <.001-.04). No significant difference was found between the remaining three products and human readers (AUROC 0.79 [0.74,0.84] P=.33, 0.80 [0.75, 0.85] P=.60, 0.84 [0.80, 0.88] P=.26).
**Conclusion:** Compared to human readers, four AI products for detecting lung nodules on chest radiographs showed superior performance whereas three other products showed no evidence of difference in performance for the detection of lung nodules.
**Limitations:** The added value of these AI products in clinical practice has yet to be determined.
**Ethics committee approval:** The analysis was on anonymised retrospective data.
**Funding for this study:** No funding was received for this study.
**Author Disclosures:**
Cornelia M. Schaefer-Prokop: Nothing to disclose
Steven S. Schalekamp: Nothing to disclose
Merel Huisman: Nothing to disclose
Bram Van Ginneken: Nothing to disclose
Kicky Gerhilde van Leeuwen: Nothing to disclose
Matthieu J.C.M. Rutten: Nothing to disclose
Maarten De Rooij: Nothing to disclose

### RPS 1205-8

**Autonomous algorithmic monitoring of a deep-learning chest radiograph AI model using temporal divergences, in a clinical real-world setting**

*C. Liew*[1], A. Gupta[2], A. M. Surve[2], V. K. Venugopal[2]; [1]Singapore/SG, [2]New Delhi/IN
*(charlene.liew.j.y@singhealth.com.sg)*

**Purpose:** This study aimed to evaluate autonomous monitoring of AI models in a clinical environment by measuring temporal divergence of mathematical probability distributions.
**Methods or Backround:** Daily prediction scores and overall abnormality prediction score of a chest radiograph classification solution (Lunit Insight CXR) were used. There were a total of 11,572 chest radiograph studies analysed over 57 days continuously on an AI platform solution in a real-world clinical setting. Of these, 7,005 studies were classified as abnormal by the AI model. The probability distributions for the abnormal predictions and probability scores for 10 abnormal diagnoses were plotted on a daily basis. Jensen-Shannon divergence was used to measure the similarity between the probability distributions of current day and the previous day in a continuous moving fashion. Daily divergence between the probability distributions against the distribution of a fixed reference was also measured. A threshold of 0.2 for acceptable divergence was used. The studies in days where the threshold was breached were reviewed for any potential errors or misclassification.
**Results or Findings:** On Day 55, there was a system technical downtime, resulting in fewer cases being processed. This day's divergences were particularly prominent, with Pneumothorax recording 0.723. Excluding day 55, divergence ranged from 0.009-0.329 across findings. Divergence values for such findings were recalibrated against the moving averages of the previous three days.
**Conclusion:** We introduced an innovative algorithmic system to monitor deep learning AI solution performance using divergence scores. Divergences were detected on days where there were technical downtimes in the AI system. This emphasises the importance of continuous monitoring of AI in clinical applications, to detect various failures of AI models, which may be due to catastrophic algorithmic failure, data bias, model drift or population data drift.
**Limitations:** No limitations were identified.
**Ethics committee approval:** This study was approved by SingHealth Central Institutional Review Board: 2023/2280.
**Funding for this study:** No funding was received for this study.
**Author Disclosures:**
Abishek Gupta: Employee: Carpl.ai
Charlene Liew: Nothing to disclose
Vasantha Kumar Venugopal: Employee: Carpl.ai
Atharva Mangesh Surve: Employee: Carpl.ai

### RPS 1205-9

**A deep learning module for automated detection and reporting of clinically significant lung nodules on low-dose chest CT scans**

*V. Popov*[1], J. Afnan[1], U. Kalabic[2], Z. Li[3], D. Chen[3], D. Hassan[2], D. Radisic[2]; [1]Burlington, MA/US, [2]Wenham, MA/US, [3]East Lansing, MI/US
*(veljkopopov@gmail.com)*

**Purpose:** Lung cancer remains the leading cause of cancer death worldwide. Multicentre trials (NLST, NELSON) have proven the efficacy of lung cancer screening in high-risk patients using low-dose, non-contrast chest CT scans. A novel Artificial intelligence (AI) module for automated nodule detection and output to the structured report is proposed, to assist with increasing screening rates while maintaining high levels of diagnostic accuracy.
**Methods or Backround:** The nnDetection framework was applied to train a one-stage detector to segment nodules. Predictions from the nodule detector were fed through an efficient mechanism for reducing overlapping bounding boxes and a separate 3D deep convolutional neural network was trained for false positive reduction (FPR). The model was then trained on the LUNA16 database (800+ LDCT studies). The model was tested on a holdout subset of LUNA16 (89 studies) and the Cornell ELCAP database (40 studies), for nodules 6 mm or greater.
**Results or Findings:** LUNA16 dataset: The performance of the nnDetection framework results in a recall of 100%, a precision of 77%, and a false negative rate of 0%. Adding the FPR model, the recall remains at 100%, the precision increases to 84%, and the false negative rate is 0%. ELCAP dataset: For nodules 6 mm or larger, nnDetection with FPR results in a recall of 100%, a precision of 58%, and a false negative rate of 4%.
**Conclusion:** nnDetection + FPR performs very well in detecting clinically relevant nodules on the LUNA16 dataset. In addition, the model shows the ability to scale across LDCT datasets without fine tuning when applied to the ELCAP Cornell dataset, detecting all nodules 6 mm or greater.
**Limitations:** An identified limitation was the small datasets.
**Ethics committee approval:** Ethics committee approval was not needed as the study used publicly available datasets.
**Funding for this study:** Private funding was obtained for this study.
**Author Disclosures:**
Dong Chen: Nothing to disclose
Dusko Radisic: Board Member: BadApple
Uros Kalabic: Board Member: BadApple
Jalil Afnan: Board Member: BadApple
Zhaojian Li: Board Member: BadApple
Danial Hassan: Board Member: BadApple
Veljko Popov: Board Member: BadApple

08:00-09:00 **Research Stage 3**

### Research Presentation Session: Interventional Radiology

### RPS 1209

### New insights in musculoskeletal interventional radiology

**Moderator**
E. Petsatodis; Thessaloniki/GR
*(vpetsatodis@hotmail.com)*

### RPS 1209-2

**Usefulness of augmented reality with computed tomography fusion in chronic total occlusion percutaneous femoral interventions**

*I. Nieri*, J. M. Pernes, K. Najeh, S. Kalinger, C. Del Giudice; Paris/FR
*(irene.n@hotmail.it)*

**Purpose:** Femoral interventions could be challenging, particularly in the presence of CTO. The aim of the study was to evaluate the use of augmented reality superpositioning of a CT fusion to treat femoral lesions in patients with claudication and critical limb ischaemia in a retrospective registry.
**Methods or Backround:** From November 2022 to August 2023, a total of 47 patients (study group mean age 81 years old, 41 male), 32 of whom were suffering with claudication and 15 with critical limb ischaemia, with femoral CTO lesion were treated by endovascular recanalisation supported by augmented reality (Vessel Assist, GE Healthcare) to guide the procedure. A preprocedural CT scan was performed on all patients, that was used to fuse the 3D volume rendering with the fluoroscopy during the procedure in an augmented reality pattern. These patients were compared to a historical cohort of 120 matched patients (control group). The primary outcome of the study dose area product (DAP) and total cumulated air kerma (CAK) were evaluated. Technical success, fluoroscopy time and amount of used contrast agent were also evaluated.
**Results or Findings:** Technical success was obtained in all procedures. No difference in terms of risk factor and lesion length was observed between the study group and control group. DAP and CAK were significantly lower in the study group compared to the control group (3611.5 versus 15408.0 mGy*cm², p<0.001 – CAK 19.8 versus 68.5 mGy, p<0.001). Fluoroscopy time was significantly inferior compared to the study group (528 versus 1108s, p<0.001). Moreover, in the study group a significant inferior amount of contrast agent was used (67.9 versus 156.0 ml, p<0.001).
**Conclusion:** The use of augmented reality CT fusion during femoral recanalisation may reduce x-ray exposure and fluoroscopy time without any difference in technical success. A reduced amount of contrast media was used in the study group.
**Limitations:** The bias of a matched control study was an identified limitation.
**Ethics committee approval:** Ethics committee approval was not required for this retrospective study.
**Funding for this study:** No funding was received for this study.
**Author Disclosures:**
Kouloud Najeh: Nothing to disclose
Sophie Kalinger: Nothing to disclose
Costantino Del Giudice: Nothing to disclose
Jean Marc Pernes: Nothing to disclose
Irene Nieri: Nothing to disclose

### RPS 1209-3

**Study protocol: percutaneous treatment in patients with lumbar spinal stenosis. Rationale and design of a phase III, multicentre, randomised, controlled trial**

*A. Napoli*, G. Alfieri, M. Mattone, S. Perotti, C. Catalano; Rome/IT
*(alessandro.napoli@uniroma1.it)*

**Purpose:** The study aimed to compare the efficacy and safety of percutaneous therapy and surgical treatment for the management of lumbar spinal stenosis.
**Methods or Backround:** This represents the protocol for a multicentre, prospective, randomised, controlled, phase III trial comparing percutaneous therapy (PT) and surgical treatment (ST) for the management of lumbar spinal stenosis. Eligible patients are between 65 and 85 years with radiologically proven lumbar spinal stenosis causing neurogenic claudication. Patients will be randomly assigned to one of the two groups, PT and ST. Patients in the PT group will undergo treatment under local anaesthesia and mild sedation in prone position using a minimally invasive percutaneous approach under fluoroscopic guidance. Primary endpoints will be overall pain as measured by a numerical rating scale (range 0-10), functional disability as measured by the Zurich Claudication Questionnaire and adverse events. Outcomes will be assessed at 3-, 6- and 12-months following treatment. Statistical analysis will be performed on an intention-to-treat basis.
**Results or Findings:** We determined that a minimum sample size equal to 216 patients is needed to detect between-group differences of at least 2 NRS points at 3-month follow-up, allowing for a standard deviation of 2.5. Assumptions for the study design considered a two-tailed significance with alpha 5% and 90% statistical power.
**Conclusion:** A phase III trial will provide evidence on the actual efficacy of percutaneous treatment of patients with lumbar spinal stenosis. The intended size of the study population is sufficiently large to detect differences between the two groups.
**Limitations:** Unedified limitations were (1) that this was an open-label trial and (2) the absence of a sham or placebo group.
**Ethics committee approval:** No information provided by the submitter.
**Funding for this study:** No funding was received for this study.
**Author Disclosures:**
Giulia Alfieri: Nothing to disclose
Monica Mattone: Nothing to disclose
Stefano Perotti: Nothing to disclose
Carlo Catalano: Nothing to disclose
Alessandro Napoli: Nothing to disclose

### RPS 1209-4

**Percutaneous computer tomography (CT): guided treatment of lumbar joint synovial cysts**

*S. Triggiani*, G. Pellegrino, M. T. Contaldo, A. M. Ierardi, S. A. Angileri, P. Biondetti, P. Torcia, G. Carrafiello; Milan/IT
*(sonia.triggiani@unimi.it)*

**Purpose:** The aim of the study was to describe our experience of percutaneous treatment of eight patients with lumbar facet synovial cysts (LFCS).
**Methods or Backround:** Eight patients referred to our centre for neurological symptoms such as low back pain and radiculopathy, refracting to the oral unpainful therapy. At MRI scan all of them were diagnosed for LFCS: five patients on the right side, three on the left side. All but two cysts (L5-S1) were at L4-L5 level. The patients underwent CT-guided treatment in an aseptic environment. All of them were positioned prone and local anaesthesia was administered. Under CT- guidance, a 21-gauge needle was inserted into the facet joint with a posteroanterior approach homolateral to the cyst and 1-2 mL of non-ionic contrast agent was locally injected to localise the cyst. The rupture was performed through the injection of dilute contrast in the cyst with a high-pressure syringe. The treatment was considered successfully completed when an extravasation of contrast was observed outside the cyst's wall and into the epidural space. VAS-score was used to evaluate pain before and after the treatment.
**Results or Findings:** Before the treatment all patients referred a VAS-score of more than six, up to ten. 100% of procedures achieved the successful rate, radiologically intended as contrast extravasation in the site of the cyst. On telephone follow-up at six to ten months after the treatment, all patients referred a VAS-score lower than four. No complications occurred.
**Conclusion:** The benefits of CT-guided cyst rupture technique can justify the future prevalence of surgical choice, as all patients achieved an immediate improvement in pain and no complications were reported during the procedure and on telephone follow-up.
**Limitations:** Identified limitations were (1) that a small series was used and (2) that this was a single centre retrospective study.
**Ethics committee approval:** Not applicable as the study was retrospective.
**Funding for this study:** No funding was received for this study.
**Author Disclosures:**
Salvatore Alessio Angileri: Nothing to disclose
Sonia Triggiani: Nothing to disclose
Pierluca Torcia: Nothing to disclose
Pierpaolo Biondetti: Nothing to disclose
Maria Teresa Contaldo: Nothing to disclose
Giuseppe Pellegrino: Nothing to disclose
Anna Maria Ierardi: Nothing to disclose
Gianpaolo Carrafiello: Nothing to disclose

### RPS 1209-5

**Genicular artery embolisation for the treatment of symptomatic knee osteoarthritis**

*F. N. N. Fleckenstein*, B. Gebauer, B. Hamm, F. Collettini; Berlin/DE
*(florian.fleckenstein@charite.de)*

**Purpose:** Genicular artery embolisation (GAE) is an innovative minimally-invasive therapy for patients with symptomatic knee osteoarthritis (OA) refractory to conservative treatments, aiming to reduce synovial arterial hypervascularity. This study aimed to evaluate the safety and efficacy of GAE for the treatment of symptomatic knee OA.

**Methods or Backround:** A retrospective, single-centre study was conducted at our institution. Patients enrolled in the study were aged 40 to 90 years, had moderate to severe knee OA (Kellgren-Lawrence grade 2 to 4), and had previously experienced failure of conservative therapy. Baseline pain (assessed using the visual analog scale [VAS]) and symptom scores (Knee Injury and Osteoarthritis Outcome Score [KOOS]) were evaluated. After achieving femoral arterial access via a 4 Fr sheath, embolisation was performed using Imipenem/Cilastatin. Target vessels were determined using digital subtraction angiography in correlation with the patients' pain points. Adverse events and symptom scores were assessed at six weeks, three months, and six months after GAE.
**Results or Findings:** A total of 46 patients were enrolled, with a median age of 69 years (IQR, 61, 74). Knee OA severity was grade 2 in 12% of patients, grade 3 in 41%, and grade 4 in 47%. Technical success was achieved in 100% of procedures. Transient skin discoloration and transient mild knee pain after the procedure were noted in 18% of all cases, as expected. No complications were reported. The KOOS daily activity index and VAS improved by 87% and 71%, respectively, at six months from a median baseline of 57 (of 100) and 7 (of 10), respectively.
**Conclusion:** This retrospective study demonstrates that GAE is an effective and safe treatment for reducing OA-associated symptoms that are refractory to conservative therapy.
**Limitations:** This was a retrospective single-centre study, without a placebo or control arm.
**Ethics committee approval:** No information provided by the submitter.
**Funding for this study:** No funding was received for this study.
**Author Disclosures:**
Bernd Hamm: Nothing to disclose
Bernhard Gebauer: Nothing to disclose
Federico Collettini: Nothing to disclose
Florian Nima Nima Fleckenstein: Nothing to disclose

### RPS 1209-6

### Patellar tendinopathy: a promising treatment approach with stromal vascular fraction (SVF)

G. Martinese, *N. Papalexis*, G. Facchini, M. Di Carlo, M. Casavola, G. Vara, G. Filardo, S. Zaffagnini, M. Miceli; Bologna/IT
*(nicolaspapalexis@gmail.com)*

**Purpose:** Patellar tendinopathy is a common overload-related pathology in both athletes and the general population, which can hinder sports activities and limit daily life functions. The use of stromal vascular fraction (SVF) is an innovative approach in the treatment of cases resistant to conservative therapy. The aim of this study is to assess the effectiveness and safety of the experimental SVF treatment at 12 months post-infiltrative procedure, along with clinical and radiological correlations. A secondary objective is to evaluate interoperator agreement on certain radiological parameters to establish reproducibility.
**Methods or Backround:** Thirteen patients (11 males and 2 females) were included, treated with ultrasound-guided SVF infiltration obtained from subcutaneous abdominal adipose tissue. Each patient was clinically assessed at the baseline and 12-month follow-up using the following questionnaires: VISA-P, VAS scale, EQ-5D, EQ-VAS, and Tegner score. Simultaneously, B-mode ultrasound evaluation, colour and power Doppler, microvascular imaging (MVI), and compressive elastography were performed by two operators with varying levels of experience.
**Results or Findings:** No adverse events were reported following the infiltrative procedure. A significant clinical improvement was observed in all questionnaires (increase in VISA-P from 57.36±17.67 to 80.2±19.80; p=0.001), with a significant correlation with reduced vascular signal (VAS-colour Doppler C.I.=-0.747; p=0.024), thickness reduction (EQ-VAS-thickness C.I.=0.68; p=0.04), and increased elastographic modulus (Tegner score-E1 C.I.=0.65; p=0.04). Interoperator agreement proved to be excellent for various ultrasound parameters (colour Doppler ICI=0.91; 95% CI=0.08-0.94).
**Conclusion:** Ultrasound-guided SVF infiltration is a safe and promising procedure for treating patellar tendinopathy, with significant clinical and radiological improvements at a 1-year follow-up.
**Limitations:** Identified limitations were (1) that this was a non randomised study and (2) that there was a lack of control group.
**Ethics committee approval:** This study was approved by the Institutional Review Board and by the Ethics Committee.
**Funding for this study:** No funding was received for this study.
**Author Disclosures:**
Marco Miceli: Nothing to disclose
Giuseppe Filardo: Nothing to disclose
Mario Casavola: Nothing to disclose
Giulio Vara: Nothing to disclose
Giancarlo Facchini: Nothing to disclose
Maddalena Di Carlo: Nothing to disclose
Nicolas Papalexis: Nothing to disclose
Giuseppe Martinese: Nothing to disclose
Stefano Zaffagnini: Nothing to disclose

### RPS 1209-7

### The performance of non-contrast magnetic resonance angiography using cardiac-gated 3D fast spin-echo for 3D fusion guidance in transarterial embolisation for refractory shoulder pain

*Y-C. Hsu*, Y-C. Chen, C. Tung, L-H. Chiang; Taipei/TW
*(doc31578@gmail.com)*

**Purpose:** Transarterial embolisation (TAE) is a widely used the treatment for aggravating musculoskeletal pain in the extremities, that is refractory to conservative treatment. Three dimensional (3D) fusion guidance for fluoroscopic navigation has been used to overcome difficulties in identifying overlapping vessels during TAE. As a pre-fusion imaging for fluoroscopic navigation, the main advantage of using magnetic resonance angiography (MRA) is that it can be performed without the use of contrast media. However, a feasible sequence for non-contrast MRA (NC-MRA) suitable for the upper extremity has not yet been established. The aim of this study was to evaluate the performance of NC-MRA using cardiac-gated 3D fast spin-echo (FSE) in the detail of arterial morphology of the shoulder.
**Methods or Backround:** Seven consecutive patients who had refractory shoulder pain underwent NC-MRA and TAE. Two reviewers assessed image quality for visualisation of shoulder arteries by using a qualitative 4-point scale (1=not assessable to 4=excellent). The number and morphology of arterial branches of subclavian and axillary arteries that were adequate for TAE treatment of refractory shoulder pain were also evaluated. The results were compared with those from digital subtraction angiography during TAE. Interobserver agreement was evaluated with the κ statistic.
**Results or Findings:** NC-MRA using cardiac-gated 3D FSE is technically successful for arterial depiction of the shoulder. Image quality was considered excellent (median=4) and the κ coefficient was 0.85. Additionally, NC-MRA correctly represented arterial branches of subclavian and axillary arteries that were adequate for TAE treatment of refractory shoulder pain in all patients.
**Conclusion:** NC-MRA using cardiac-gated 3D FSE is technically and clinically feasible and represents a promising technique for 3D fusion guidance in TAE for refractory shoulder pain.
**Limitations:** The small number of patients was an identified limitation.
**Ethics committee approval:** This study was approved by the research institution of Tri-Service General Hospital.
**Funding for this study:** No funding was received for this study.
**Author Disclosures:**
Yi-Chih Hsu: Nothing to disclose
Lung-Hui Chiang: Nothing to disclose
Ya-Che Chen: Nothing to disclose
Ching Tung: Nothing to disclose

### RPS 1209-8

### Intra-articular hyaluronic acid and platelet-rich plasma as monotherapy or combination therapy, what is the evidence?

*S-F. Oon*, C. Nguyen; Melbourne/AU

**Purpose:** This study aimed to use a validated evidence-based practice tool to identify best current evidence for combined intra-articular hyaluronic acid (HA) and platelet-rich plasma (PRP) versus monotherapy in treating knee osteoarthritis.
**Methods or Backround:** Knee osteoarthritis is a degenerative joint disease characterised by progressive loss of articular cartilage from wear and tear. Combined HA and PRP intra-articular injections gained recent interest and may relieve pain and reduce progression of osteoarthritis through synergistic mechanisms. Current literature causes confusion due to mixed conclusions, even among meta-analyses. We used the McMaster University and National Health Service five-step systematic approach to conduct a bottom-up search through the primary and secondary literature. We limited our search to review and meta-analysis articles in the English language only, within the last three years.
**Results or Findings:** A total of 69 articles were retrieved and evaluated for validity and strength. Five final articles were selected for individual review and the results tabulated. For combined HA+PRP versus HA alone, HA+PRP demonstrated significant improvement in stiffness and physical function symptoms at 3, 6 and 12 months (p <0.001). There was no statistically significant difference between PRP+HA and HA alone in pain control (p=0.195). For HA+PRP versus PRP alone, pain reduction was seen at one month with PRP only. After this time, no statistically significant difference was found between the two groups with regards pain, stiffness or physical function.
**Conclusion:** Combination PRP with HA demonstrates superior symptom control when compared to HA monotherapy, but combination PRP and HA does not provide further long term statistically significant symptom control over PRP monotherapy.
**Limitations:** The limitation of articles to the English language and within the last 3 years could have reduced the robustness of search. Meta-analyses were of low quality due to extensive heterogeneity of the included studies.
**Ethics committee approval:** Ethical approval waived by Peter MacCallum Cancer Centre Ethics and Governance Committee: "Literature reviews of publically available information do not require ethics committee review."

**Funding for this study:** No funding was received for this study.
**Author Disclosures:**
Sheng-Fei Oon: Nothing to disclose
Chau Nguyen: Nothing to disclose

**08:00-09:00** **Research Stage 4**

### Research Presentation Session: Cardiac

### RPS 1203

### Spectral CT: new developments and clinical applications (part 1)

**Moderator**
R. N. Planken; Amsterdam/NL

### RPS 1203-2

**Iodine quantification with a CdZnTe clinical prototype photon-counting scanner at reduced radiation dose: initial cardiac phantom results and reproducibility analysis**

T. W. Holmes[1], S. Sharma[2], S. Ross[2], T. Labno[2], R. Zhang[2], S. Wu[2], Z. Yu[2], R. Thompson[2], *A. Pourmorteza*[1]; [1]Atlanta, GA/US, [2]Vernon Hills, IL/US
*(amir.pourmorteza@gmail.com)*

**Purpose:** This study aimed to assess effectiveness of a clinical prototype CT scanner with CdZnTe-based photon-counting detector (PCD) CT technology in spectral mode, specifically regarding the measurement of iodinated contrast agents at both standard and reduced radiation dosage levels.
**Methods or Backround:** We 3D-printed a series of phantoms mimicking coronary arteries with lumen diameter of 3.5 mm with stents, soft plaque, and hard plaques placed in them. The phantoms were placed inside two water tanks with 12 cm diameters. The water tanks were scanned on the prototype scanner at 120 kVp and 200, 100, and 50 mAs exposures in 6-bin spectral mode. The 50 mAs scan was repeated twice to assess reproducibility. We measured accuracy, stability with respect to radiation dose, and reproducibility of iodine through Bland-Altman analysis of values measured in circular regions of interest (ROIs) ranging from 2.2 mm to 10 mm in diameter.
**Results or Findings:** We measured very small bias of -0.15 and good 95% confidence interval (CI) [-1.52 1.21] for concentrations of iodine ranging from 0 to 35 compared to calibrated values (all values are reported as mg I/mL). At 50% radiation dose reduction we observed bias of 0.35 with CI=[-0.32 1.04] compared to the high-dose (200 mAs) measurements. At 75% dose reduction the bias and CI were 0.45 and [-0.49 1.44]. The 75% dose-reduced iodine maps showed good reproducibility with bias of 0.01 mg I/mL and confidence interval of [-0.66 0.69].
**Conclusion:** Accurate quantification of iodine is an important tool in characterisation of coronary artery plaques as well as for making detailed measurements of myocardial perfusion. PCD-CT shows potential for making accurate iodine measurements in dose-reduced settings.
**Limitations:** Limitations of this study were: this was a limited pilot study with 2 phantoms; more experiments mimicking different patient sizes are warranted.
**Ethics committee approval:** No information provided by the submitter.
**Funding for this study:** Funding was received through a sponsored research agreement with Canon Medical Research USA.
**Author Disclosures:**
Zhou Yu: Employee: Canon Medical USA
Steven Ross: Employee: Canon Medical USA
Richard Thompson: Employee: Canon Medical USA
Amir Pourmorteza: Research/Grant Support: Siemens Healthineers; Research/Grant Support: GE HealthCare; Research/Grant Support: Cannon Medical USA; Advisory Board: CarioWise Inc; Employee: Emory University
Thomas Wesley Holmes: Research/Grant Support: GE HealthCare; Research/Grant Support: Cannon Medical USA; Consultant: CarioWise Inc; Research/Grant Support: Siemens Healthineers; Employee: Emory University
Tom Labno: Employee: Canon Medical USA
Ruoqiao Zhang: Employee: Canon Medical USA
Shuoxing Wu: Employee: Canon Medical USA
Shobhit Sharma: Employee: Canon Medical USA

### RPS 1203-3

**Impact of photon-counting detector CT-based calcium removal algorithm on coronary stenosis grading and CT-FFR values**

*Z. Jokkel*[1], B. Vattay[1], M. Boussoussou[1], M. Vecsey-Nagy[1], M. Kolossvary[1], M. Kiss[2], B. Sipos[1], P. Maurovich-Horvat[1], B. Szilveszter[1]; [1]Budapest/HU, [2]Forcheim/DE

**Purpose:** CT-based fractional flow reserve (CT-FFR) provides non-invasive evaluation of lesion specific ischaemia. However, blooming artefacts from heavily calcified plaques could hinder the assessment of luminal stenosis and alter CT-FFR values. The objective of this study was to investigate the effect of a calcium removal algorithm on the grading of coronary stenoses and CT-FFR values in comparison to measurements based on the standard image reconstruction used for clinical evaluation.
**Methods or Backround:** We enrolled consecutive patients with calcified plaques and intermediate coronary artery stenosis (30-90%) who underwent a dual-source photon counting CT (NAEOTOM Alpha, Siemens Healthineers) for suspected coronary artery disease. A total of 54 lesions were analysed and two reconstructions were used (standard: 0.4 mm slice thickness, Bv40, 70keV; PureLumen calcium removal algorithm: 0.8mm slice thickness, Qr40, 70keV). Anatomical landmarks were used as fiducial markers to ensure that the same location was used for CT-FFR assessment. Area-stenosis and CT-FFR values distal to the plaques were measured using syngo.via Frontier CT-cFFR software (Siemens Healthineers). Comparison of stenosis and CT-FFR values was performed using Wilcoxon signed rank test.
**Results or Findings:** A total of 25 patients (mean age 64.5±8.1years, 24% female) were included in the study. Median calcium score and Q1-Q3: 258 (154.35-401.75). Mean area stenosis of the 54 lesions was 50.5±22.5% on standard reconstruction compared to 43.5±20.9% on PureLumen images ($p<0.001$). CT-FFR values were higher for PureLumen reconstructed lesions compared to standard reconstruction: 0.87±0.11 vs 0.82±0.15 ($p<0.001$).
**Conclusion:** PureLumen calcium removal technique might allow for more accurate assessment of the degree of stenosis by removing calcium blooming artefacts. In patients with intermediate stenosis, this resulted in lower stenosis grades and higher CT-FFR values, which may affect further therapeutic decisions.
**Limitations:** Correlation with invasive coronarography is warranted and will be available in a subpopulation.
**Ethics committee approval:** This study was approved by the Scientific and Research Ethics Committee of the Hungarian Medical Research Council (IV/665-3/2022/EKU) and was carried out in accordance with the tenets of the Declaration of Helsinki.
**Funding for this study:** Funding was provided by the János Bolyai Research Scholarship of the Hungarian Academy of Sciences. Project number RRF-2.3.1-21-2022-00003 has been implemented with the support provided by the European Union.
**Author Disclosures:**
Melinda Boussoussou: Nothing to disclose
Pál Maurovich-Horvat: Nothing to disclose
Milán Vecsey-Nagy: Nothing to disclose
Marton Kolossvary: Nothing to disclose
Bálint Szilveszter: Nothing to disclose
Borbála Vattay: Nothing to disclose
Zsofia Jokkel: Nothing to disclose
Barbara Sipos: Nothing to disclose
Máté Kiss: Employee: Siemens Healthcare GmbH, Forcheim/DE

### RPS 1203-4

**Stenosis quantification on virtual non-calcium images from photon-counting detector CT**

*V. Mergen*[1], S. Rusek[2], F. Civaia[2], P. Rossi[2], R. Rajagopal[3], R. Manka[1], A. Candreva[1], M. Eberhard[1], H. Alkadhi[1]; [1]Zurich/CH, [2]Monaco/MC, [3]Jodhpur/IN
*(victor.mergen@usz.ch)*

**Purpose:** This study aimed to evaluate, in patients with known or suspected coronary artery disease, the feasibility and accuracy of quantification of calcified coronary stenoses using virtual non-calcium (VNCa) images in CCTA with photon-counting detector (PCD) CT compared with quantitative coronary angiography (QCA).
**Methods or Backround:** This retrospective, institutional review board approved study included consecutive patients with calcified coronary artery plaques undergoing CCTA with PCD-CT and invasive coronary angiography between July and December 2022. Virtual monoenergetic images (VMI) and VNCa images were reconstructed. Diameter stenoses were quantified on VMI and VNCa images by two readers. Stenosis measurements from 3D-QCA served as the standard of reference. Measurements were compared using Bland-Altman analyses, Wilcoxon tests, intraclass correlation coefficients (ICC), and weighted-Kappa analysis.

**Results or Findings:** Thirty patients (mean age, 64 years ± 8 [standard deviation]; 26 men) with 81 coronary stenoses from calcified plaques were included. Ten of the 81 stenoses (12%) had to be excluded because of erroneous plaque subtraction on VNCa images. Median diameter stenosis determined on 3D-QCA was 22% (interquartile range, 11-35%; total range, 4-88%). As compared with 3D-QCA, VMI overestimated diameter stenoses (mean differences -7%, p<.001, ICC:.84 and -10%, p<.001, ICC:.87 for reader 1 and 2, respectively), whereas VNCa images showed similar diameter stenoses (mean differences 1%, p=.07, ICC:.93 and 0%, p=.68, ICC:.94 for reader 1 and 2, respectively).
**Conclusion:** This preliminary experience suggests that virtual calcium removal in CCTA with PCD-CT is feasible in a high proportion of calcified, minimal to moderate stenoses and has the potential to improve the quantification of coronary stenoses.
**Limitations:** First, this single centre retrospective study included only a limited number of patients and stenoses. Second, only a limited number of moderate and severe stenoses were present.
**Ethics committee approval:** No information provided by the submitter.
**Funding for this study:** No funding was received for this study.
**Author Disclosures:**
Rengarajan Rajagopal: Nothing to disclose
Victor Mergen: Nothing to disclose
Matthias Eberhard: Nothing to disclose
Filippo Civaia: Nothing to disclose
Philippe Rossi: Nothing to disclose
Alessandro Candreva: Nothing to disclose
Stéphane Rusek: Nothing to disclose
Robert Manka: Nothing to disclose
Hatem Alkadhi: Nothing to disclose

### RPS 1203-5

### Improvement of the visualisation of coronary stents by ultra-high-resolution photon-counting detector CT

*L. Qin*, S. Zhou, F. Yan, W. Yang; Shanghai/CN
*(tevez10@126.com)*

**Purpose:** This study aimed to investigate the quantitative and qualitative characteristics of coronary stents of ultra-high-resolution coronary CT angiography (CCTA) with different reconstruction kernels on photon counting detector-CT (PCD-CT) in patients after percutaneous coronary intervention (PCI).
**Methods or Backround:** From July 2023 to September 2023, 68 patients (132 stents) after PCI were enrolled to undergo CCTA on a dual-source PCD-CT system. Standard resolution images with thickness of 0.6 mm, increment of 0.4 mm and kernel of Bv48, and UHR images with thickness and increment of 0.2 mm and kernel of Bv48, Bv56, Bv60, Bv64, Bv72 and Bv76 were reconstructed. CT attenuation and noise were measured in the aorta root and within the stents. Stent and in-stent diameters were also assessed. Subjective image quality was evaluated by a Likert-5 point scale.
**Results or Findings:** Image noise significantly increased with the reduction of image thickness and elevation of kernels (2.6±5.7～136.3±18.5 HU, P<0.001). UHR images with Bv72 and Bv76 had the smallest differences between aorta and in-stent CT values (14.8±68.3 HU, 6.2±73.9 HU, P<0.001), and differences between stent and in-stent diameters (1.2±0.2 mm, 1.2±0.2 mm, P<0.001). UHR images with Bv72 had the largest in-stent lumen diameter (2.3±0.5 mm, P<0.001) and the smallest differences between stent diameter and nominal diameter (0.4±0.3 mm, P<0.001) compared to other images. Subjective analysis showed that images with Bv72 had the most superior effect of blooming artefact reduction (5 [5, 5], P<0.001), and in-stent lumen and stent demonstration (5 [5, 5], P<0.001). Bv72 also had the highest diagnostic confidence (5 [5, 5], P<0.001).
**Conclusion:** UHR CCTA on PCD-CT results in the significantly improved visualisation of coronary stents and Bv72 is the optimal reconstruction kernel to show the stent struts and in-stent lumen.
**Limitations:** An identified limitation was that the in-stent diameter and diagnostic accuracy for in-stent re-stenosis were not compared with the quantitative invasive coronary angiography.
**Ethics committee approval:** This study was approved by Ruijin hospital ethics committee, Shanghai Jiaotong University School of Medicine.
**Funding for this study:** No funding was received for this study.
**Author Disclosures:**
Wenjie Yang: Nothing to disclose
Le Qin: Nothing to disclose
Fuhua Yan: Nothing to disclose
Shanshui Zhou: Nothing to disclose

### RPS 1203-6

### Photon-counting CT angiography in pre-TAVR aortic annulus sizing: high-pitch spectral vs low-pitch ultra-high-resolution CT-angiography

*M. T. Hagar*[1], T. Jennerjahn[1], D. Westermann[1], S. Faby[2], C. Von Zur Mühlen[1], C. L. Schlett[1], F. Bamberg[1], T. Krauß[1], M. Soschynski[1]; [1]Freiburg im Breisgau/DE, [2]Forchheim/DE

**Purpose:** The study aimed to assess the diagnostic efficacy of low-pitch, retrospective ultra-high-resolution CT-angiography (UHR-CTA) and high-pitch, prospective spectral CTA (HPS-CTA) utilising a first-generation, dual-source photon-counting detector CT scanner in preprocedural planning for transcatheter aortic valve replacement (TAVR).
**Methods or Backround:** Consecutive patients with severe aortic valve stenosis were referred clinically, underwent UHR-CTA (collimation: 120 x 0.2 mm) and HPS-CTA (144 x 0.4 mm with full spectral information) for TAVR planning and were retrospectively enrolled. Radiation doses were extracted from CT reports. Two radiologists independently evaluated UHR-CTA and HPS-CTA datasets, analysing aortic annulus image quality (on a 4-point scale) and measuring the aortic annulus area (AAA) and perimeter (AAP). These measurements then informed hypothetical valve prosthesis sizing according to vendor recommendations.
**Results or Findings:** Among a total of 64 patients (mean age 81, ±7 SD; 28 women), HPS-CTA was associated with lower radiation exposure (4.1 mSv) than UHR-CTA (12.6 mSv, p<0.001, while UHR-CTA exhibited superior image quality (median score: 1, IQR: 1–2 vs. 2, IQR: 2–3; p<0.001). The AAA and AAP assessments from both CTA datasets were strongly correlated (Pearson r2 = 0.857), leading to consistent valve prosthesis sizing in 89% of patients. However, those with lower image quality on HPS-CTA (score ≥3) more frequently encountered varied sizing recommendations in hypothetical aortic valve prosthesis selection.
**Conclusion:** UHR-CTA and HPS-CTA by photon-counting CT technology provide reliable aortic annular evaluations for TAVR planning. While UHR-CTA provides enhanced image quality, HPS-CTA features lower radiation exposure. However, impaired image quality in HPS-CTA may necessitate additional UHR-CTA to avoid valve sizing discrepancies.
**Limitations:** Identified limitations were: (1) the study's generalisability may be limited by its focus on primarily older patients, (2) this was a single-centre study with a retrospective design, and (3) hypothetical valve prosthesis sizing based on aortic annular measurements might overlook other patient-specific factors in real-world decision-making.
**Ethics committee approval:** Informed written consent was obtained from all patients. This study was approved with the approval code: 21–2469; approval date: 21-09-2021.
**Funding for this study:** Funding was received from the Ministerium für Wirtschaft, Arbeit und Wohnungsbau Baden-Württemberg (35-4223.10/20).
**Author Disclosures:**
Martin Soschynski: Nothing to disclose
Tobias Krauß: Nothing to disclose
Dirk Westermann: Nothing to disclose
Muhammad Taha Hagar: Speaker: Siemens Healthcare
Christopher L. Schlett: Speaker: Siemens Healthcare
Theresa Jennerjahn: Nothing to disclose
Fabian Bamberg: Grant Recipient: Siemens Healthcare; Speaker: Siemens Healthcare
Constantin Von Zur Mühlen: Nothing to disclose
Sebastian Faby: Employee: Siemens Healthcare; Shareholder: Siemens Healthcare

### RPS 1203-7

### First clinical experience with a novel reconstruction algorithm to reduce stair-step artefacts in step-and-shoot cardiac photon-counting detector CT

*L. J. Moser*[1], V. Mergen[1], M. Eberhard[2], H. Alkadhi[1]; [1]Zurich/CH, [2]Unterseen/CH

**Purpose:** One of the most common cardiac CT scan modes is prospective ECG-triggering (or step-and-shoot (SAS)). Due to the stack-wise acquisition over usually several heart cycles, SAS may be associated with "stair-step" artefacts in the transitional areas, where stacks are misaligned or overlapping due to irregular heart rates. This study aimed to evaluate a novel cardiac imaging reconstruction algorithm designed for photon-counting detector CT to reduce the occurrence and severity of such artefacts.
**Methods or Backround:** In this clinical study, we included 50 consecutive patients who underwent cardiac CT on a clinical dual-source photon-counting detector CT scanner in the SAS mode. Each scan was reconstructed without and with the ZeeFree algorithm, which uses overlapping information to perform a non-rigid registration between the borders of two adjacent sub-volumes respectively, to minimise potentially occurring stair-step artefacts. The presence or absence and, if present, the extent of stair-step artefacts were rated on a visual analogue four-point scale.

**Results or Findings:** Forty of the total 800 coronary segments (5%) had stair-step artefacts, from which 12 (30%) led to a non-diagnostic image quality (1.5% of all segments). The novel reconstruction algorithm significantly reduced the number and extent of these artefacts compared to standard reconstructions (median score ZeeFree: 1; median score standard reconstruction: 3; p<0.001). From the initially rated 12 non-diagnostic segments in standard reconstruction, 9 (75%) improved to a diagnostic image quality using the algorithm.
**Conclusion:** Our results demonstrate the feasibility and effectiveness of a novel reconstruction algorithm, which significantly reduces stair-step artefacts in coronary CT angiography with dual-source photon-counting detector CT acquired in the SAS mode.
**Limitations:** Identified limitations were (1) that this was a single centre experience and (2) the effect on diagnostic accuracy was not evaluated.
**Ethics committee approval:** This was a retrospective study design and a waiver of written informed consent was obtained.
**Funding for this study:** No funding was received for this study.
**Author Disclosures:**
Victor Mergen: Nothing to disclose
Matthias Eberhard: Nothing to disclose
Lukas Jakob Moser: Nothing to disclose
Hatem Alkadhi: Speaker: Siemens

**09:30-11:00** **Research Stage 1**

### Research Presentation Session: Emergency Imaging

### RPS 1317

### Hot topics in emergency radiology

**Moderator**
M. A. Mazzei; Siena/IT
*(mmazzei@sirm.org)*

### RPS 1317-2

**Automated torso CT haemorrhage burden: a novel precision-medicine decision support tool for transfusion in trauma patients**
*N. Sarkar*, L. Zhang, M. Unberath, U. Bodanapally, J. Hu, G. Li, O. Turan, A. Corkum, D. Dreizin; Baltimore, MD/US

**Purpose:** Torso haemorrhage is a leading cause of preventable death in traumatic injury and can be precisely quantified on whole-body CT using deep learning segmentation methods. Prior studies have shown that quantitative assessment of haemorrhage burden in individual body cavities is associated with transfusion requirement. The purpose of this study is to examine total torso CT haemorrhage burden (tCTHB) in the thorax, abdomen, and pelvis as a predictor of massive transfusion (MT).
**Methods or Backround:** A dataset of 5060 trauma CT scans was used to select patients with hemothorax, haemoperitoneum, pelvic haematoma, or combinations thereof (n=593). A state-of-the-art deep learning method (nnU-net) was used to derive segmentation masks and volumes. tCTHB was calculated as the sum total of segmented torso haemorrhage in mL. MT, defined as at least 10 packed red blood cells (PRBCs) in 24 hours, served as the outcome. The area under curve (AUC) of shock index (SI) + tCTHB was compared to that of SI alone. Optimal tCTHB cutoff was determined by Youden J index. Spearman correlations between PRBCs and volumes were determined for tCTHB and individual intracavitary haemorrhage types.
**Results or Findings:** The AUC of SI + tCTHB (0.86 [95% CI: 0.75-0.97]) was higher (p=0.02) than the AUC of SI alone (0.67 [95% CI: 0.50-0.84]). 363 mL served as an optimal cut-off at peak Youden J index. The sensitivity and specificity of SI>1 and tCTHB>363 mL was 90% and 81%, whereas SI>1 alone had sensitivity and specificity of 50% and 91%. Spearman's r was moderate for tCTHB (0.42), low for haemorrhage volume in individual compartments (0.12-0.23), and low for SI (0.28).
**Conclusion:** tCTHB is a promising precision medicine marker for predicting massive transfusion, significantly improving accuracy over shock index alone.
**Limitations:** No limitations were identified.
**Ethics committee approval:** This study was reviewed and approved by the University of Maryland Baltimore Institutional Review Board (UMB IRB). It was determined by the UMB IRB to be exempt from the need for informed consent due to no more than minimal risk to patients.
**Funding for this study:** Funding was received from NIH K08 Grant EB027141-01A1.
**Author Disclosures:**
Abigail Corkum: Nothing to disclose
Jiazhen Hu: Nothing to disclose
Nathan Sarkar: Nothing to disclose
Guang Li: Nothing to disclose
Uttam Bodanapally: Nothing to disclose
Ozerk Turan: Nothing to disclose
David Dreizin: Nothing to disclose
Lei Zhang: Nothing to disclose
Mathias Unberath: Nothing to disclose

### RPS 1317-3

**Coronary calcification score in polytrauma CT of severely injured polytrauma patients as a prognostic factor for hospital mortality and intensive care unit treatment**
*H-J. Meyer*, T. Dermendzhiev, T. Denecke, M. Struck; Leipzig/DE

**Purpose:** Coronary artery calcifications defined by computed tomography (CT) provide prognostic relevance for vascular disorders and coronary heart disease, whereas their prognostic relevance in severely injured trauma patients remains unclear.
**Methods or Backround:** All consecutive trauma patients requiring emergency tracheal intubation before initial computed tomography at a level-1 trauma centre over a 12-year period (2008-2019) were reanalysed. The Weston score, a semiquantitative method to quantify the coronary calcifications, was evaluated as prognostic variable based upon whole-body trauma CT analysis.
**Results or Findings:** 458 patients (75% male) with a median age of 49 years, median injury severity score (ISS) of 26, and in-hospital mortality rate of 23.1% met the inclusion criteria and were analysed. Coronary artery calcification was present in 214 patients (46.7%). After adjustment with age, ASA classification ≥ III, ISS abbreviated injury scale head, and shock, the Cox proportional hazard model identified the Weston score as independent predictor for 24- hour-, 30-day-, and in-hospital mortality (HR 1.2, 95% CI 1.08 – 1.34, p= 0.001; HR 1.09, 95% CI 1.02 – 1.18, p= 0.017; and HR 1.1, 95% CI 1.02 – 1.18, p= 0.012, respectively).
**Conclusion:** CT-derived coronary calcification was significantly associated with hospital mortality in intubated polytrauma patients. In the subgroup of survivors, it was significantly associated with ICU LOS but not with mechanical ventilation duration.
**Limitations:** First, it is a single-centre retrospective study. Second, only patients requiring tracheal intubation and who underwent whole-body CT were included in this analysis. Third, although the Weston score is a semiquantitative imaging analysis, we cannot exclude investigator- related bias.
**Ethics committee approval:** The retrospective analysis was approved by the Ethical Committee at the Medical Faculty, Leipzig University, Leipzig, Germany, (IRB00001750, project ID 441/15ek, September 14, 2020).
**Funding for this study:** No funding was received for this study.
**Author Disclosures:**
Timm Denecke: Nothing to disclose
Manuel Struck: Nothing to disclose
Tihomir Dermendzhiev: Nothing to disclose
Hans-Jonas Meyer: Nothing to disclose

### RPS 1317-4

**Prevalence and extent of blunt cervical vascular injury (BCVI): a longitudinal single-centre study over 19 years**
*B. Mazini*, S. Sanson, V. Dunet, A. Denys, S. Schmidt; Lausanne/CH
*(bianca.mazini@chuv.ch)*

**Purpose:** To aim of this study was to assess the prevalence and extent of blunt cervical vascular injury (BCVI) in the context of high energy trauma, and to analyse the associated radiological findings, immediate clinical management and patients' outcome according to the extent of BCVI.
**Methods or Backround:** This single-center retrospective study included 89 consecutive patients with acute BCVI proven by computed tomography angiography-(CTA), that were selected from 8053 polytrauma patients addressed to our emergency department within 19 years. Two radiologists blinded to all clinical information jointly reviewed images regarding location and extent of BCVI, associated radiological findings and cerebral complications (stroke). Epidemiological and clinical data, and outcomes were retrieved from patients' medical records. Univariate and multivariate statistical analysis were performed. A p-value <0.05 was considered statistically significant.
**Results or Findings:** The prevalence of BCVI in our polytrauma population was 1.15% (26 females, mean age 46 years). In the 89 patients with BCVI 119 cervical arterial dissections were found. In 23 patients (25.8%) acute stroke was associated; overall mortality was 19% (n = 17). Patients' mortality was significantly predicted by clinical factors (Glasgow coma scale, and intubation at arrival), and radiological features, such as a higher grade of vascular injury and exact length of left vertebral artery dissection. In 61 (68.5%) patients with cervical fractures the vascular injury was adjacent. However, 28 (31.5%) arterial injuries were not associated with any bone fracture.

**Conclusion:** BCVI is a rare but serious complication of high energy cervical trauma, with high rate of morbidity and mortality. Systematic screening of the cervical arteries with angio-CT seems necessary in all polytrauma patients since >30% of cervical arterial lesions are not associated with nearby bone fractures.
**Limitations:** This is a retrospective study.
**Ethics committee approval:** Not applicable.
**Funding for this study:** No funding was received for this study.
**Author Disclosures:**
Alban Denys: Nothing to disclose
Servane Sanson: Nothing to disclose
Vincent Dunet: Nothing to disclose
Bianca Mazini: Nothing to disclose
Sabine Schmidt: Nothing to disclose

### RPS 1317-5

**Computed tomography imaging in earthquake trauma patients with crush syndrome and different levels of creatine kinase: is there a correlation with renal enhancement?**

*S. N. Yılmazer Zorlu*, M. Kul, A. Gürsoy Çoruh; Ankara/TR
*(sezernilyilmazer@gmail.com)*

**Purpose:** The objective of this study was to investigate whether varying serum creatine kinase (CK) levels in patients with crush syndrome have an impact on renal enhancement on computed tomography (CT) even in cases where renal injury is not clinically evident.
**Methods or Backround:** Portovenous phase abdominopelvic CT scans of earthquake (Turkey, February 2023) survivors and of patients without involvement in the earthquake and with preserved renal function (control group) were retrospectively analysed. Attenuation measurements were made from the cortex and medulla of the left kidney at the level of renal hilus, abdominal aorta (level of renal artery origin) and main portal vein. The medulla-to-cortex ratio was normalised to the contrast phase using the formula: nM/C-R= (Medulla Attenuation/Cortex Attenuation)/ (Portal Vein Attenuation/Aorta Attenuation). Renal cortex/aorta (C/A) attenuation ratio was also calculated. Earthquake survivors were categorised by serum CK levels into risk groups for acute kidney injury (AKI) (high-risk group:≥773 U/l, low-risk group:<773 U/l). CT findings were compared between groups and correlations with laboratory findings were made.
**Results or Findings:** In the earthquake survivor group 33 patients were in the low-risk and 12 in the high-risk category for AKI. The control group consisted of 50 patients. None of the 95 patients had impaired renal function.
Though no significant age, gender, nC/M-R and creatinine value differences were found (p> 0,05), the high-risk group had significantly lower renal C/A attenuation ratios than the low-risk and control groups (p= 0.043; p= 0.023, respectively). There was a negative correlation between CK and C/A attenuation ratios and a positive correlation between calcium levels and C/A attenuation ratios. The nC/M-R and serum levels for creatinine, potassium and CK did not significantly correlate.
**Conclusion:** Even in the absence of clinically evident AKI, cortical enhancement is diminished in patients with crush syndrome with higher CK levels.
**Limitations:** Laboratory follow up of patients could not be made.
**Ethics committee approval:** This study was approved by the Research Ethics Committee of Ankara University Faculty of Medicine.
**Funding for this study:** No funding was received for this study.
**Author Disclosures:**
Melahat Kul: Nothing to disclose
Sezer Nil Yılmazer Zorlu: Nothing to disclose
Ayşegül Gürsoy Çoruh: Nothing to disclose

### RPS 1317-6

**Portal venous contrast enhancement ratio of the adrenal glands and spleen as prognostic marker of mortality in patients with acute mesenteric ischaemia**

*F. A. Pfister*, M. Mehdorn, C. Schwartner, D. Seehofer, H-M. Tautenhahn, M. Struck, T. Denecke, H-J. Meyer; Leipzig/DE

**Purpose:** Contrast enhancement of the adrenal gland defined by computed tomography (CT) was previously analysed as a prognostic factor for critically ill patients due to various diseases. However, no study investigated this quantitative parameter in patients with acute mesenteric ischaemia, a potentially lethal disease. Therefore, the aim of this study was to evaluate the prognostic value of the contrast enhancement of the adrenal glands in patients with clinically suspected AMI.
**Methods or Backround:** All patients with clinically suspected AMI were retrospectively assessed between 2016 to 2020. All patients underwent surgical exploration. Overall, 134 patients (52 female patients, 38.8%) with a mean age of 69.2 ± 12.4 years were included into the present analysis. For all patients, the preoperative CT was used to calculate the contrast media enhancement of the adrenal glands and the spleen.
**Results or Findings:** Overall, 27 (20.1%) patients died within the 24-hour period, and 94 (70.1%) within 30 days. There were statistically significant differences regarding the mean values for adrenal-to-spleen ratio for 24h-mortality (p= 0.001) and 30-day mortality (p= 0.004), whereas the radiodensity of the inferior vena cava and the radiodensity of the spleen was statistically significant between survivors and non-survivors after 30-days (p= 0.037 and p= 0.028, respectively). In cox regression analysis mean adrenal radiodensity was associated with mortality after 24h with an HR of 1.09 (95% CI 1.02-1.16, p= 0.01).
**Conclusion:** The contrast media enhancement of the adrenal gland is associated with the 24h- and 30-day mortality in patients with AMI. However, the identified associations are worse compared to previous analyses regarding other critical diseases.
**Limitations:** First, it is a retrospective single-centre study with known inherent bias. Second, despite the quantitative nature of the measurements, there can still be some intrareader variability.
**Ethics committee approval:** Not applicable.
**Funding for this study:** No funding was received for this study.
**Author Disclosures:**
Timm Denecke: Nothing to disclose
Daniel Seehofer: Nothing to disclose
Manuel Struck: Nothing to disclose
Felix Alexander Pfister: Nothing to disclose
Matthias Mehdorn: Nothing to disclose
Christoph Schwartner: Nothing to disclose
Hans-Michael Tautenhahn: Nothing to disclose
Hans-Jonas Meyer: Nothing to disclose

### RPS 1317-7

**Comparison of brain injury in patients with and without facial fractures**

*I. T. Lupașcu*, S. Hostiuc, C. Adrian, B. Popa, C. A. Minoiu; Bucharest/RO
*(iulia_lupascu9e@yahoo.com)*

**Purpose:** This study aimed to examine the association between facial fractures and brain injury and to compare brain injuries in facial fracture patients with non-facial fracture patients.
**Methods or Backround:** Cerebral CT of 492 polytrauma patients, who were admitted to the hospital between January 2019 to July 2023, were retrospectively evaluated.
**Results or Findings:** From the total of 492 patients, 129 (26%) had facial fractures (102 men, 27 women, mean age 45 ±,17 years) and 363 (74%) were without facial fractures (253 men, 110 female, mean age 46 ±,17 years). Facial fractures were significantly correlated with brain injuries (p< .001), showing a greater incidence of brain lesions (54%, n= 68), compared to the non-facial fractures group (31%, n= 111). Subdural hematoma was the most frequent lesion (29%, n= 44) in the facial fracture group, while intracerebral haemorrhage was the most frequent lesion (33%, n= 73) in the non-facial fracture group. The zygomatic bone was the most frequently fractured (28%, n= 67) and it was significantly correlated to the presence of intracerebral haemorrhage, subdural and subarachnoid haemorrhage, but also with pneumocephalus and diffuse brain swelling (p< .05). Frontal sinus fracture (12%, n= 29) and its right or left side location were correlated with the presence and location of epidural haematoma and intracerebral haemorrhage (p< .05). Maxillary fractures were observed in 27% (n= 65) patients, nasal bones in 25% (n= 58) and mandible fractures in 8% (n= 19). In the facial fractures group, diffuse brain swelling was present in 18% (n= 23) patients, pneumocephalus in 10% (n= 12) and brain herniation in 6% (n= 8), while in the non-facial fractures group, diffuse brain swelling was observed in 7% (n= 27), pneumocephalus in 2% (n= 6) and brain herniation in 3% (n= 10) of patients.
**Conclusion:** There is a significant association between facial fractures and traumatic brain injury, with zygomatic bone being the most frequently fractured and subdural hematoma the most associated brain lesion.
**Limitations:** The limitation of the study was the retrospective design.
**Ethics committee approval:** Not applicable.
**Funding for this study:** No funding was received for this study.
**Author Disclosures:**
Costin Aurelian Minoiu: Nothing to disclose
Iulia Tatiana Lupașcu: Nothing to disclose
Bogdan Popa: Nothing to disclose
Sorin Hostiuc: Nothing to disclose
Cosmin Adrian: Nothing to disclose

### RPS 1317-8

**Assessment of Syngo.CT LVO: detection in photon counting CT and conventional CT**

*J. Boriesosdick*, N. Haag, S. Saeed, C. Mönninghoff, J. Borggrefe, J. R. Kroeger; Minden/DE

**Purpose:** Syngo.CT LVO detection (SIEMENS Healthineers) is a prototype for AI-assisted detection of large vessel occlusion in the anterior circulation area in patients with suspected ischaemic stroke. We investigated the sensitivity and specificity of the algorithm in photon counting CT (PCCT; NAEOTOM Alpha)

and conventional CT (SOMATOM GoTop/X.ceed/GoAll). Furthermore, we investigated whether there were differences in detection rate between proximal (internal carotid artery, M1 segment of middle cerebral artery) and distal (M2 segment) vessel occlusions.
**Methods or Backround:** We retrospectively analysed 443 cases with CT-Angiography of the head (Bv44-Kernel) with Syngo.CT LVO Detection. 176 cases showed vascular occlusion in the anterior circulation area. 150 cases were investigated with the PCCT with a total of 50 occluded vessels, 293 cases were investigated in conventional CT scanners with a total of 126 occluded vessels.
**Results or Findings:** The algorithm showed an overall sensitivity of 70.5% and a specificity of 98.5%. Thereby, we found significant differences (p= 0.013) in sensitivity between PCCT (84%) and conventional CT (65.1%). Proximal vessel occlusions were detected significantly better than M2- occlusions in all scanners with a sensitivity of 86.2% versus 26.1% (p< 0.001). While sensitivity regarding proximal vessel occlusions did not differ significantly between scanners (90.2% versus 84.3%; p= 0.359), sensitivity regarding M2- occlusions showed a significant difference in favor of PCCT (55.6% versus 18.9%; p= 0.039).
**Conclusion:** Syngo.CT LVO detection could assist radiologists in the rapid detection of large vessel occlusions as a triage tool in clinical routine. Thereby, the algorithm seems to work better with PCCT data, especially for M2- occlusions, probably due to a higher image quality compared to conventional detectors. Overall, the algorithm should be further trained specifically regarding the detection of M2- occlusions.
**Limitations:** The sign of the study was monocentric, and cases were prone to selection bias. Furthermore, there were only a few cases with M2- occlusions in PCCT.
**Ethics committee approval:** The study was conducted according to the guidelines of the Declaration of Helsinki and approved by the Institutional Ethics Committee of the Ruhr-University Bochum (19 July 2021, reference number 2021-827).
**Funding for this study:** No funding was received for this study.
**Author Disclosures:**
Christoph Mönninghoff: Nothing to disclose
Jan Boriesosdick: Nothing to disclose
Jan Robert Kroeger: Speaker: GE Healthcare; Advisory Board: Siemens Healthineers
Saher Saeed: Nothing to disclose
Nina Haag: Nothing to disclose
Jan Borggrefe: Speaker: Stryker, Philips Healtcare, Siemens Healthineers

### RPS 1317-9

### Incidence of contrast-induced acute kidney injury (CI-AKI) in trauma patients undergoing contrast-enhanced computed tomography using iso-osmolar contrast agent

*M. Gim*, H. S. Lee, K. Lee, J. K. Kim, J. Huh; Suwon/KR
*(minjigim1991@gmail.com)*

**Purpose:** In trauma patients, no report has been published on the preventive effect of iso-osmolar contrast agent for contrast-induced acute kidney injury (CI-AKI). We aimed to evaluate the incidence and severity of CI-AKI, and its predictive factors in trauma patients.
**Methods or Backround:** From the trauma registry in a regional trauma centre in Korea, patients who underwent CT scans with iodixanol and were followed up for at least 72 hours were consecutively included. Patient demographic details, co-morbidities, and laboratory test results were collected. CI-AKI was defined by the 2012 kidney disease improving global outcomes guideline. The severity of CI-AKI was classified by the RIFLE criteria. Trauma severity was assessed by the injury severity score (ISS) category. Predictive factors of CI-AKI were evaluated by univariate and multivariate logistic regression.
**Results or Findings:** Of 1115 patients who underwent CT with iodixanol, 799 were included in this study. The incidence of CI-AKI was 3.80% (30/799). Severe renal failure according to RIFLE criteria was 2.87% (23/799). Incidence of CI-AKI according to the ISS was 1.52% (9/592) in the minor group, 3.95% (7/177) in the moderate group, 42.30% (11/26) in the serious group, and 75.0% (3/4) in the severe group. In univariate analysis, significant predictive factors of CI-AKI based odds ratio (OR) included hypertension (2.87), heart disease (4.06), ISS serious category (47.50), and ISS severe category (194.33). In multivariate analysis, significant predictive factors were the ISS serious category [16.70 (95% CI, 16.70-138.96], and ISS severe category [245.22 (95% CI, 21.88-2748.38)].
**Conclusion:** In trauma patients who underwent CT with iodixanol, the overall incidence of CI-AKI of 3.8% (30/799) was considerably low. The main predictive factors of CI-AKI involved hypertension, heart diseases, and the ISS serious and severe categories, but its incidence and severity largely relied on the severity of trauma.
**Limitations:** This was a retrospective study.
**Ethics committee approval:** This study was approved by IRB of Ajou University Hospital.
**Funding for this study:** No funding was received for this study.
**Author Disclosures:**
Jai Keun Kim: Nothing to disclose
Minji Gim: Nothing to disclose
Jimi Huh: Grant Recipient: GE Healthcare
Kyungmin Lee: Nothing to disclose
Hyun Seung Lee: Nothing to disclose

### RPS 1317-10

### Utilising dual-layer detector spectral CT to predict clinical risk stratification in the diagnosis of acute pulmonary embolism

*Y. Zhou*, Z. Wang, L. Lei, W. Cao, S. Dong, S. W. Yue, Z. Zhou; Zheng Zhou/CN
*(zyh921209zyh@163.com)*

**Purpose:** The research utilised the iodine density map in conjunction with the effective atomic number map (ID-Z-eff map) to evaluate the rate of emboli detection in individuals diagnosed with acute pulmonary embolism (PE). Additionally, the study quantitatively assessed the burden of pulmonary thrombosis to predict the clinical risk stratification in these patients.
**Methods or Backround:** This prospective study comprised a cohort of 83 individuals diagnosed with PE who underwent CTPA utilising dual-layer detector spectral CT (DLCT). The spectral data were reconstructed to generate conventional CT images (CI) and ID-Z-eff maps. The number of identified emboli in both cohorts was quantified, and the diagnostic efficacy was assessed. The distribution of emboli was analysed using deep-learning lung segmentation. The severity of pulmonary embolism was evaluated using the Qanadli and Mastora scores. The receiver operating characteristic (ROC) curves were used to assess each score in distinguishing clinical risk stratification.
**Results or Findings:** 202 and 243 emboli were detected in conventional CT images (CI) and ID-Z-eff maps, respectively. The ID-Z-eff map demonstrated superior embolus detection capabilities compared to CI images (p< 0.05). 42/35 patients were categorized as low-/intermediate risk. There was a significant difference in pulmonary embolism index between the low-risk and medium-risk groups (Qanadli: 14.8%, 39.5%, p< 0.001; Mastora: 11.2%, 34.3%, p< 0.001). The pulmonary embolism index Qanadli and Mastora scores demonstrated promising discrimination in the low-/intermediate-risk groups (Qanadli: AUC=0.876, Mastora: AUC=0.875).
**Conclusion:** The ID-Z-eff map can enhance the sensitivity and accuracy of acute pulmonary embolism emboli detection. The pulmonary embolism index based on the ID-Z-eff map utilising DLCT can accurately predict the clinical risk stratification of acute pulmonary embolism.
**Limitations:** The sample size in this research is relatively limited.
**Ethics committee approval:** This study was approved by the Institutional Review Board
**Funding for this study:** No funding was received for this study.
**Author Disclosures:**
Shushan Dong: Nothing to disclose
Limin Lei: Nothing to disclose
Zhihao Wang: Nothing to disclose
Weimeng Cao: Nothing to disclose
Song Wei Yue: Nothing to disclose
Zhigang Zhou: Nothing to disclose
Yuhan Zhou: Nothing to disclose

### RPS 1317-11

### BRIXIA score, laboratory findings and vaccination status for prediction of mortality in severe COVID-19 pneumonia

*V. Opancina*[1], M. Muto[2], N. Zdravkovic[1], E. Ciceri[3]; [1]Kragujevac/RS, [2]Naples/IT, [3]Milan/IT

**Purpose:** The aim of our study was to investigate correlations between main laboratory parameters, vaccination status, and Brixia score, as well as to confirm if the Brixia score is a significant independent predictor of an unfavourable outcome (death) in hospitalised COVID-19 patients.
**Methods or Backround:** Chest X-ray has verified its role as a crucial tool in COVID-19 assessment due to its practicability, especially in emergency units. Likewise, the Brixia score has proven itself as a useful tool for COVID-19 pneumonia grading. The study was designed as a cross-sectional multicentric study. It included patients with a diagnosed COVID-19 infection who were hospitalised between September 2021 and February 2022 and had RT-PCR-confirmed COVID-19 and initial CXR and laboratory results.
**Results or Findings:** This study included a total of 279 patients with a median age of 62 years. The only significant predictor of an unfavourable outcome (death) was the Brixia score (adjusted odds ratio 1.148, p= 0.022). Also, the results of the multiple linear regression analysis ($R^2$= 0.334, F= 19.424, p< 0.001) have shown that male gender (B= 0.903, p= 0.046), severe COVID-19 (B=1.970, p< 0.001), and lactate dehydrogenase (B=0.002, p< 0.001) were significant positive predictors, while albumin level (B=–0.211, p< 0.001) was a significant negative predictor of the Brixia score.

**Conclusion:** Our results provide important information about factors influencing the Brixia score and its usefulness in predicting unfavourable outcomes in COVID-19 patients. These findings have clinical relevance, especially in epidemic and emergency circumstances. In order to monitor these patients effectively and to achieve efficient patient management, CXR is a great tool due to its wide availability. Also, the application of the Brixia score is clear and simple and benefits clinicians in daily praxis.
**Limitations:** The limitations of the study are its small sample size and the lack of a control group.
**Ethics committee approval:** This study was approved by the Ethics Committee of University Clinical Center Kragujevac, 62/2022.
**Funding for this study:** No funding was received for this study.
**Author Disclosures:**
Nebojsa Zdravkovic: Nothing to disclose
Valentina Opancina: Nothing to disclose
Mario Muto: Nothing to disclose
Elisa Ciceri: Nothing to disclose

**09:30-11:00** **Research Stage 2**

### Research Presentation Session: Paediatrics

### RPS 1312

### Neuro- and skeletal-paediatric radiology

**Moderator**
I. Barber; Esplugues de Llobregat/ES
*(ibarber@sjdhospitalbarcelona.org)*

### RPS 1312-2

**Evaluating AI and human-derived tumour volume estimations in paediatric osteosarcoma: association with histological response and survival predictions**

*L. C. Adams*[1], K. K. Bressem[1], W. Morakote[2], V. Suryadevara[2], A. Pribnow[2], S. Spunt[2], L. Baratto[2], J. Rosenberg[2], H. E. Daldrup-Link[2]; [1]Berlin/DE, [2]Palo Alto, CA/US

**Purpose:** The study aimed to assess the potential of using human- and AI-assessed tumour volume changes as markers for treatment response in paediatric osteosarcoma, in comparison to histological necrosis, and to evaluate their association with patient survival.
**Methods or Backround:** In paediatric osteosarcoma, the extent of tumour necrosis post-surgery is used to estimate treatment response. This single-centre retrospective study involved examining pre- and post-chemotherapy MRI scans of 57 paediatric and young adult patients with histologically confirmed osteosarcoma. A subset of these patients also underwent 18F-FDG PET. We correlated human- and AI-assessed tumour volume changes with overall (OS) and recurrence-free survival (RFS) metrics using concordance correlation (CC). Additionally, quantitative measures such as tumour volume/size changes and histological necrosis (%) were assessed for their impact on OS/RFS through logrank tests and Cox regression. We further compared tumour ADC and SUV between responders (≥90% histological necrosis) and non-responders.
**Results or Findings:** AI-assessed tumour volume change measurements were a stronger predictor of OS than histological necrosis (Harrell's C=0.88 versus C=0.70). Human assessments did not outpace necrosis in predicting survival outcomes. When examining histological necrosis ≥90% and AI-driven volume change of <25% post-chemotherapy, both were linked to improved OS (p=0.021 and p<0.0001 respectively). There was high agreement between human and AI measurements (CC coefficient >0.95). Furthermore, patients with a histological necrosis of ≥90% had higher ADC values both pre- and post-chemotherapy (both p=0.004), and displayed lower pre-therapy SUV values when compared to non-responders (p=0.001).
**Conclusion:** Both human and AI-derived tumour volume changes showed a correlation with histological necrosis, indicating their potential as markers for treatment response and OS in paediatric osteosarcoma. AI-driven volume change measurements demonstrated stronger predictive capabilities for OS than histological necrosis.
**Limitations:** Our research was based in a single centre, potentially limiting wider applicability.
**Ethics committee approval:** This study was approved by the IRB, with approval number 48854.
**Funding for this study:** Funding was received from the National Cancer Institute (grant R01CA269231).
**Author Disclosures:**
Sheri Spunt: Nothing to disclose
Keno K. Bressem: Nothing to disclose
Allison Pribnow: Nothing to disclose
Vidyani Suryadevara: Nothing to disclose
Heike Elisabeth Daldrup-Link: Nothing to disclose
Lisa C. Adams: Nothing to disclose
Lucia Baratto: Nothing to disclose
Wipawee Morakote: Nothing to disclose
Jarrett Rosenberg: Nothing to disclose

### RPS 1312-3

**Intracranial haemorrhage detected with prenatal MRI in foetuses with Chiari II malformation: indication or contraindication for foetal surgery**

S. Hui[1], D. Prayer[2], P. Kienast[2], J. Binder[2], K. Goeral[2], C. Mitter[2], T. Dorittke[2], G. Kasprian[2], *M. Stuempflen*[2]; [1]Guangzhou/CN, [2]Vienna/AT

**Purpose:** Intracranial haemorrhages (ICH) have emerged as a notable association in Chiari II malformation (CM II), yet their origins and clinical implications remain elusive. This study aims to validate the prevalence of ICH in CM II, investigate contributing factors to ICH, and delineate the phenotypic attributes associated with CM II and ICH.
**Methods or Backround:** A retrospective review of foetal MRI scans obtained in foetuses with CM II presenting (January 2007 to December 2022) at Vienna General Hospital was performed for ICH utilising EPI-T2* blood-sensitive sequence. Foetuses with aqueduct stenosis (AS) were also included as a control group. The incidence of ICH and corresponding gestational ages were compared between CM II and AS cases, and morphometric measurements (inner/outer CSF spaces, posterior fossa, venous structure) were compared among the three 1:1 age-matched groups: CM II+ICH, CM II-ICH, and AS+ICH. Additionally, a co-occurrence network was constructed to visualise associations between diagnostic features in ICH cases.
**Results or Findings:** A total of 101 foetuses with CM II and 90 controls with AS were included. The prevalence of ICH in foetuses with CM II was higher compared to the AS cases (28.7% vs 18.9%), accompanied by congested veins (deep vein congestion mainly in young foetuses, and cortical veins also affected in older foetuses). ICH was correlated with more distal vermis ectopia, reduced outer CSF spaces, and clivus-supraocciput angle. The co-occurrence network analysis underscored venous congestion and venous sinus stenosis as pivotal components within the network.
**Conclusion:** The prevalence of ICH among foetuses with CM II accentuates the interplay of venous congestion, ICH, and vasogenic oedema, hinting at potential anatomical attributes underlying the vicious cycle. Prenatal repair surgery should be applied to these cases as soon as possible.
**Limitations:** The retrospective nature of this study was an identified limitation.
**Ethics committee approval:** This study was approved by the Ethics Committee with approval code: 1716/2017.
**Funding for this study:** No funding was received for this study.
**Author Disclosures:**
Tim Dorittke: Nothing to disclose
Christian Mitter: Nothing to disclose
Marlene Stuempflen: Nothing to disclose
Shi Hui: Nothing to disclose
Julia Binder: Nothing to disclose
Daniela Prayer: Nothing to disclose
Patric Kienast: Nothing to disclose
Katharina Goeral: Nothing to disclose
Gregor Kasprian: Nothing to disclose

### RPS 1312-4

**Using 'fast' MRI head scan to improve paediatric patient experience for investigation of headaches**

*L. C. Walker*, A. Quigley, S. Choi, L. Armstrong, D. Evans, R. Kirkbride; Edinburgh/UK
*(lauz1212@hotmail.com)*

**Purpose:** There are numerous requests, per year, for Magnetic Resonance Imaging (MRI) head scans for paediatric patients who present with headaches, usually returning a normal resultant MRI scan. A fast head (FH) imaging protocol (IP) was developed, reducing scan length from 12 minutes 51 seconds (Standard Head (SH) IP) to four minutes 22 seconds, a 66% time reduction in scan-time, potentially improving patient experience.
**Methods or Backround:** 50 retrospective paediatric patients (mean age, 10 years ± 3.215 Standard Deviation (SD), referred for headaches, who had both FH and SH IP, at the same appointment, were included. Imaging was split on a picture archiving and communication system, randomised, then each folder blindly reported twice, separately, by two consultant neuro-radiologists. The original report was used as gold standard and compared with all imaging. Scores on a pre-determined ranking system were recorded by the radiologists using a four-point Likert scale (LS) for all scans. Intra-observer agreement was reviewed and SD was used as the statistical evaluation.

**Results or Findings:** The FH IP showed adequate overall image quality and, on three sequences, less motion artefact with a score of two or more points on the LS. In two cases on the T1 and fluid attenuation inversion recovery sequences the average score was less than two (1.95; 1.98) when visualising the basal ganglia. Although these scores were less than the SH imaging, the overall image quality was deemed to be sufficiently diagnostic across all points with a sensitivity of 82.7%, specificity of 76.5% and accuracy of 79.4%.
**Conclusion:** FH imaging shows pathology can confidently be identified despite a reduction in image quality and offers sufficient diagnostic clinical information. FH IP imaging can become the new SH IP when investigating headaches in paediatric patients.
**Limitations:** No limitations were identified.
**Ethics committee approval:** The study is retrospective.
**Funding for this study:** No funding was received for this study.
**Author Disclosures:**
Louisa Armstrong: Nothing to disclose
Samantha Choi: Nothing to disclose
Laura Christine Walker: Nothing to disclose
Alan Quigley: Nothing to disclose
Rachael Kirkbride: Nothing to disclose
David Evans: Nothing to disclose

### RPS 1312-5

**Reference values for knee ultrasound in children and adolescents: thickness of intercondylar cartilage, and length of ossified patella**

*S. C. Dias*[1], D. C. Carvalho[1], M. Castro[1], C. C. Dias[1], I. M. Ramos[1], I. Brito[1], K. Rosendahl[2]; [1]Porto/PT, [2]Tromsø/NO
*(silviacostadias@hotmail.com)*

**Purpose:** The study aimed to establish ultrasound-based reference values for intercondylar cartilage thickness and patellar length by age and sex.
**Methods or Backround:** This was a prospective, cross-sectional study on healthy volunteers aged 3–17 years. Exclusion criteria were those of chronic medicated diseases that affect the skeletal system or a recent trauma. Bilateral knee ultrasound was performed by one of two experienced examiners, with the child supine, knee 90° flexed for measurement of intercondylar cartilage thickness and extended for the patellar length. Interobserver variation on 27 participants was assessed.
**Results or Findings:** A total of 127 volunteers (67 females) with a median age of 10.9 years were included. Median thickness of the intercondylar cartilage was 3.1 mm (percentiles 2.5th – 97.5th: 2.1- 4.2 mm) for females and 3.4 mm (percentiles 2.5th – 97.5th: 2.3-4.7 mm) for males (p=0.005). There were no differences according to right or left side (p=0.757). The cartilage thickness decreased with age, with a median of 3.6 mm for 3–6-year-olds and 2.9 mm in 14-17 year-olds (p < 0.001). Median length of the ossified patella was 33.6 mm (percentiles 2.5th – 97.5th: 0.0 – 44.2 mm) on the right side versus 32.4 mm (percentiles 2.5th – 97.5th: 0.8 – 42.2 mm) on the left side (p < 0.001). No differences were seen according to sex (p=0.259 for the right and p=0.233 for the left side). The reproducibility between the two readers was high (intraclass correlation coefficient values > 0.9).
**Conclusion:** The intercondylar cartilage thickness decreased with increasing age, with females having thinner cartilage than males. The length of the ossified right patella was significantly longer than the left. The presented reference values can help discriminate between normality and pathology.
**Limitations:** No limitations were identified.
**Ethics committee approval:** The study registration number is: CE 191/22.
**Funding for this study:** No funding was received for this study.
**Author Disclosures:**
Iva Brito: Nothing to disclose
Miguel Castro: Nothing to disclose
Cláudia Camila Dias: Nothing to disclose
Sílvia Costa Dias: Nothing to disclose
Karen Rosendahl: Nothing to disclose
Diogo Costa Carvalho: Nothing to disclose
Isabel M. Ramos: Nothing to disclose

### RPS 1312-6

**The diagnostic performance of magnetic resonance imaging in the categorisation of paediatric neck lymph nodes: radiological and pathological correlations**

*N. Paytoncu*, E. Çalışkan, H. G. Düzkalır, M. Arifoğlu, N. Fıstıkçıoğlu, H. P. Günbey; Istanbul/TR
*(nazpaytoncu1@gmail.com)*

**Purpose:** In literature, there isn't a compact magnetic resonance imaging (MRI) study of the neck in the paediatric population covering both numerical data and morphological criteria. Therefore, the aim of this study was to present certain MRI features of neck lymph nodes in benign and malignant conditions in children.
**Methods or Backround:** Contrast-enhanced MRI of the neck of 51 paediatric patients aged 1-18 years (40 boys, 11 girls [10.08±4.73]) who underwent neck lymph node biopsy were retrospectively analysed. They were grouped as benign, including reactive (27 [52.9%]), lymphadenitis (11 [21.6%]), and malignant (13 [25.5%]). The groups were evaluated multiparametrically in terms of quantitative and qualitative variables.
**Results or Findings:** Medians and ranges (25-75th percentile) of long axis, short axis, area and apparent diffusion coefficient (ADC) values of the largest lymph node were 21 (17-24) mm, 14 (12-18) mm, 228.60 (144.79-351.82) mm2, 2531 (2457-2714) mm2/s for reactive, 24 (19-27) mm, 15 (11-20) mm, 271.80 (231.43-412.20) mm2, 2534 (2425-2594) mm2/s for lymphadenitis, 27 (23.50-31.50) mm, 20 (15-22) mm, 377.08 (260.47-530.94) mm2, 2337 (2254-2466) mm2/s for malignant, respectively. The lymph nodes with a long axis greater than 22 mm, a short axis greater than 16 mm, an area greater than 319 cm2, and an ADC value less than 2367 mm2/s may be malignant. Those with an atypical location (especially supraclavicular) have a higher rate of malignancy (p=0.003). Perinodal signal changes, nodal heterogeneity with cystic/necrotic areas and posterior cervical triangle location are common in lymphadenitis (p<0.001). Reactive lymph nodes are distributed symmetrically in both neck halves (p<0.001).
**Conclusion:** In an MRI-based approach to reactive, lymphadenitis and malignant lymph nodes for the paediatric era, not only numerical data such as long axis, short axis, surface area and ADC, but also morphological criteria such as location, distribution, internal structure and perinodal heterogeneity should be used.
**Limitations:** The study cohort could be expanded to include a greater number of participants. ADC measurements result from the solid millimetric section of mostly necrotic lymph nodes, which might not be optimal.
**Ethics committee approval:** The ethics review board at our institution approved this study (decision number: 2023/514/244/16) which is performed in accordance with the principles of the Declaration of Helsinki.
**Funding for this study:** No funding was received for this study.
**Author Disclosures:**
Hediye Pinar Günbey: Nothing to disclose
Naz Paytoncu: Nothing to disclose
Meral Arifoğlu: Nothing to disclose
Neriman Fıstıkçıoğlu: Nothing to disclose
Emine Çalışkan: Nothing to disclose
Hanife Gülden Düzkalır: Nothing to disclose

### RPS 1312-7

**Glymphatic system dysfunction and white matter connectivity alterations for diagnosing autism spectrum disorder in children**

*M. Wang*[1], K. He[2], D. Xu[1], L. Wang[1], G. Zhang[3], B. Peng[4], Y. Dai[4], L. Zhang[1], H. Jiang[1]; [1]Wuxi/CN, [2]Changchun/CN, [3]Shenzhen/CN, [4]Suzhou/CN
*(wyflipped@gmail.com)*

**Purpose:** Multiparametric MRI analysis can help elucidate the pathogenesis of autism spectrum disorder (ASD). This study aimed to analyse the glymphatic system and alterations of white matter connectivity for diagnosing ASD.
**Methods or Backround:** In this retrospective case-control study, the data of children aged 3–6 years with ASD, and typically developing (TD) children from two tertiary medical centres were collected. Patients with ASD were assigned to the mild-moderate, and severe groups using the Childhood Autism Rating Scale. Automated diffusion tensor imaging along the perivascular space (aDTI-ALPS) index and fractional anisotropy values were obtained on DTI. Node efficiency values were derived from the construction of structural brain networks. A general linear model was used to evaluate the group differences in aDTI-ALPS index, fractional anisotropy values, and brain network properties. Multiple MRI parameter features were evaluated for diagnostic efficacy using a machine learning framework.
**Results or Findings:** Seventy children with ASD (mean age, 4.25 years; 51 male patients) and 45 TD children (mean age, 4.54 years; 25 male patients) were included in the study. The aDTI-ALPS index was lesser in the mild-moderate ASD group than that in the control group (1.43 versus 1.58, p<.001). A further reduction in the aDTI-ALPS index was observed in the severe ASD group compared with that in the mild-moderate ASD group (1.43 versus 1.33, p<.001). The accuracies of diagnosing ASD and distinguishing severe ASD using aDTI-ALPS combined with extra-axial cerebrospinal fluid volume, white matter fractional anisotropy, and node efficiency were 82.50% and 85.00% respectively, and area under the curve were 0.88 and 0.89, respectively.
**Conclusion:** The multiparametric predictive model based on glymphatic system dysfunction and alterations in white matter connectivity enables the effective diagnosis and stratification of severity levels in autism spectrum disorder.
**Limitations:** No limitations were identified.
**Ethics committee approval:** The study received institutional review board approval and written informed consent was obtained from all participants.

**Funding for this study:** Funding was provided by the Precision Medicine Key Project of Wuxi Health Commission (grant number: J202107); Sanming Project of Medicine in Shenzhen (SZSM202011005); and Wuxi Science and Technology Development Project (CN) (grant number: N20192005).
**Author Disclosures:**
Yakang Dai: Nothing to disclose
Lei Wang: Nothing to disclose
Gongwei Zhang: Nothing to disclose
Miaoyan Wang: Nothing to disclose
Haoxiang Jiang: Nothing to disclose
Lili Zhang: Nothing to disclose
Dandan Xu: Nothing to disclose
Keyi He: Nothing to disclose
Bo Peng: Nothing to disclose

### RPS 1312-8

**Microstructural white matter change, emotional dysfunction and visual working memory in adolescents with borderline personality disorder**

*X. Yi*[1], B. T. Chen[2]; [1]Changsha/CN, [2]Duarte, CA/US
*(yixiaoping@csu.edu.cn)*

**Purpose:** Emotional dysfunction is one of the core symptoms in adolescent borderline personality disorder (BPD), with the underlying mechanisms remaining unclear. The aim of this study was to assess the alteration of microstructural white matter fibres and its association with visual working memory and emotional dysfunction in adolescent BPD.
**Methods or Backround:** A total of 53 adolescents with BPD aged 12–17 years and 39 age- and gender-matched healthy controls (HCs) were enrolled into this study. Based on diffusion tensor imaging (DTI) data, radial diffusivity (RD) and axial diffusivity (AD) were generated using Tract-Based Spatial Statistics (TBSS) method. Correlative analysis of microstructural alterations with visual working memory, non-suicidal self-injurious behaviours (NSSI) and childhood trauma were performed.
**Results or Findings:** Compared with HCs, adolescents with BPD showed lower AD values in the splenium of the corpus callosum, left anterior corona radiata, and left external capsule. In adolescents with BPD, higher RD values were observed in the genu of the corpus callosum, body of the corpus callosum, right anterior corona radiata, and right uncinate fasciculus. There were significant correlations between increased RD of genu and body of corpus callosum was negatively correlated with visual working memory (visual reproduction), NSSI (Ottawa Self-Injury Inventory-4C), and childhood trauma (Childhood Trauma Questionnaire-E) ($P<0.05$).
**Conclusion:** There were brain microstructural alterations within the cortical-limbic system in adolescents with BPD, and these changes were found to be associated with visual working memory, NSSI and childhood trauma in BPD. These results implicate that the microstructural alterations may serve as a potential neuroimaging biomarker for underlying pathological mechanisms in adolescents with BPD.
**Limitations:** The sample size was relatively small, and this was a cross-sectional study.
**Ethics committee approval:** The present study was approved by the ethics committee of our institute (IRB: 2022020227), and all participants and their legal guardians provided written informed consent.
**Funding for this study:** No funding was received for this study.
**Author Disclosures:**
Bihong T. Chen: Nothing to disclose
Xiaoping Yi: Nothing to disclose

### RPS 1312-9

**Lesion volume and spike frequency impact perfusion in focal cortical dysplasia: a paediatric arterial spin labelling study**

*A. G. Gennari*, G. Bicciato, S. Lo Biundo, R. Kottke, I. Yakoub, D. Cserpan, R. Tuura O'Gorman, G. Ramantani; Zurich/CH

**Purpose:** Arterial spin labelling (ASL) has yielded promising results in the presurgical workup of children with FCD-related epilepsy. Despite the increased utilisation of ASL, the interpretation of perfusion patterns and their correlations with other patient characteristics remains unclear. Our study evaluated the perfusion changes captured by ASL in this vulnerable subgroup and investigated their clinical, EEG, and MRI determinants.
**Methods or Backround:** We included children with an MRI-detectable FCD, who underwent ASL. We assessed ASL perfusion changes qualitatively by visual inspection and quantitatively by estimating the asymmetry index (AI). We correlated perfusion patterns and their extent, as well as the AI values, with clinical, EEG, and MRI features.
**Results or Findings:** We considered 18 scans from 15 children with FCD-related epilepsy; seven underwent resective epilepsy surgery. A total of 16 of 18 (89%) scans showed FCD-related perfusion changes: 10 FCDs were hypoperfused, whereas six were hyperperfused. Nine scans had perfusion changes larger than, and seven equal to, the FCD extent on anatomical images. Hyperperfusion in ASL was determined by frequent EEG spikes ($p=0.047$, Chi-square test). Perfusion changes in ASL larger than the FCD corresponded to larger lesion volumes ($p=0.017$, Wilcoxon-Mann-Whitney test). Higher AI values were determined by frequent EEG spikes ($p=0.004$, Welch t-test) and smaller lesion volumes (after controlling for age at MRI) in univariate analysis, but only frequent EEG spikes retained their significance in multivariate analysis.
**Conclusion:** ASL showed FCD-related perfusion changes in most cases, including smaller volume lesions, which may escape detection in anatomical MRI. Higher spike frequency may increase ASL yield in affected children. These observations may facilitate the interpretation of ASL findings, improving treatment management, counselling, and prognostication in children with FCD-related epilepsy.
**Limitations:** The main limitations are the retrospective design and the small sample size.
**Ethics committee approval:** The study was approved by and performed according to the guidelines and regulations of the local ethics committee (KEK-ZH 2019-01854). All parents gave written informed general consent to reuse clinical data for research.
**Funding for this study:** We thank the Anna Mueller Grocholski Foundation and the Swiss National Science Foundation (SNSF: 208184) (to G.R.) and the Swiss Government Excellence Scholarship (to A.G.G.) for funding. The funders had no role in the design or analysis of the study. None of the authors has any conflict of interest to disclose.
**Author Disclosures:**
Ruth Tuura O'Gorman: Nothing to disclose
Antonio Giulio Gennari: Nothing to disclose
Dorottya Cserpan: Nothing to disclose
Georgia Ramantani: Nothing to disclose
Ilona Yakoub: Nothing to disclose
Raimund Kottke: Nothing to disclose
Santo Lo Biundo: Nothing to disclose
Giulio Bicciato: Nothing to disclose

### RPS 1312-10

**Assessing the validity of MR imaging severity score for predicting the clinical outcome in acute necrotising encephalopathy of childhood**

*K. Khandwala*, K. Hilal, S. Kaleem, M. Mufarrih; Karachi/PK
*(kumail.khandwala@gmail.com)*

**Purpose:** Acute necrotising encephalopathy of childhood (ANEC) is a unique entity with bilateral grey and white matter involvement. The purpose of this study is to determine whether severity of MR imaging findings can predict the prognosis and the clinical outcome of patients with ANEC.
**Methods or Backround:** A retrospective cross-sectional study was conducted on 42 patients diagnosed with ANEC. An MR imaging severity score was devised for each patient according to a point system derived from the presence of haemorrhage, cavitation, enhancement, diffusion restriction and location of lesions. The scoring was categorised into mild, moderate, and severe. Clinical outcomes were determined at the time of discharge and at one-yearly follow-ups as mild disability, moderate disability, severe disability, and death.
**Results or Findings:** The study included 21 boys and 21 girls with a mean age of 71.5 months. No statistically positive correlation (r 0.1198) was found between the MR grading and the clinical outcome. A statistically significant correlation of diffusion restriction (p 0.03) and cerebellar involvement (p 0.05) with worse clinical outcome was seen. Those with presence of shock also correlated with worse outcomes (p 0.01).
**Conclusion:** Cerebellar involvement and presence of diffusion restriction on imaging, and presence of shock on presentation were associated with a worse clinical outcome in our study. The MR imaging severity score overall, however, did not correlate significantly with clinical outcome. Therefore, our results suggest the importance of combined clinical, laboratory, and neuroimaging findings in determining the prognostic outcome of patients with ANEC rather than imaging severity alone.
**Limitations:** This was a single centre study with limited resources to conduct extensive virological studies, follow-up MRI or assess RANBP2 target mutation for determining a genetic analysis. Our follow-ups were also limited because not all patients could be clinically examined.
**Ethics committee approval:** The study was approved with approval number: 2020-5529-14796.
**Funding for this study:** No funding was received for this study.
**Author Disclosures:**
Kiran Hilal: Nothing to disclose
Kumail Khandwala: Nothing to disclose
Musa Mufarrih: Nothing to disclose
Sidra Kaleem: Nothing to disclose

**RPS 1312-11**

**Bone involvement in children with lymphoma: results of a PET/MR study**

*L. Pilati*[1], G. Fichera[1], S. Bertin[1], M. Pillon[1], D. Cecchin[1], P. Zucchetta[2], C. Giraudo[1]; [1]Padua/IT, [2]Treviso/IT
*(lucia.pilati@studenti.unipd.it)*

**Purpose:** The objective of this study was to assess the diagnostic value of 18F-FDG-PET/MR for bone involvement in pediatric patients with lymphoma
**Methods or Backround:** Children and adolescents (i.e., <21 years-old) with lymphoma who underwent a 18F-FDG-PET/MR at diagnosis, from January 2017 to December 2022 were included. For each PET/MR site (skull, upper and lower extremities, and axial skeleton) and number of skeletal lesions (up to five) were recorded with a separate evaluation of T1w, TIRM and PET datasets. PET/MR was considered positive when a lesion was visible on T1W and/or TIRM and had high metabolic activity (higher than the mediastinal blood pool). Biopsy or a combined reference standard, including other radiological techniques and follow-up were used. The diagnostic value of T1W, TIRM, PET, and PET/MR was assessed by computing per-patient sensitivity (Se) and specificity (Sp).
**Results or Findings:** 59 patients (mean age 13.8±3.1years old; 32 females) matched the inclusion criteria. Overall, 11 (18.6%) patients, eight with Hodgkin-lymphoma, had bone involvement. Overall, 60 lesions were detected with the lower extremities being mostly affected (30 lesions), followed by the axial skeleton (16 lesions). The average size of the largest lesions was 26.5±16 mm. On T1w images were identified five false positive and four false negative patients (Se=63.6%, Sp=89.6%) while with TIRM six patients turned out to be false positive (Se=100%, Sp=87.5%). With PET and PET/MR occurred only one false negative (Se=90.91%, Sp=100%).
**Conclusion:** PET/MR including TIRM allows an accurate characterization of bone involvement in children and adolescents with lymphoma.
**Limitations:** No limitations were identified.
**Ethics committee approval:** Not applicable.
**Funding for this study:** No funding was received for this study.
**Author Disclosures:**
Giulia Fichera: Nothing to disclose
Diego Cecchin: Nothing to disclose
Pietro Zucchetta: Nothing to disclose
Lucia Pilati: Nothing to disclose
Marta Pillon: Nothing to disclose
Chiara Giraudo: Nothing to disclose
Sofia Bertin: Nothing to disclose

**RPS 1312-12**

**Pre- and postoperative foetal MRI for the evaluation of foetoscopic hybrid surgery in foetuses with spina bifida aperta**

*M. Schulze*[1], C. Keil[1], I. Bedei[2], V. Hohmann[1], B. Saß[1], S. Köhler[1], R. Axt-Fliedner[2], A. Kemmling[1]; [1]Marburg/DE, [2]Giessen/DE
*(maximilian_schulze@gmx.de)*

**Purpose:** Foetal MRI is important for the preoperative evaluation of spina bifida aperta (SBA) and its intracranial findings and is therefore relevant for treatment decisions. Postoperative MRI enables intrauterine evaluation of the surgical result, also with regard to the regression of ACM and possible further postpartum therapy requirements.
**Methods or Backround:** SBA is a congenital malformation of multifactorial aetiology. The sensorimotor impairment of function depends on the level of the spinal lesion. In addition, intracranial malformations such as Arnold Chiari malformation (ACM), hydrocephalus, callosal disorders, and heterotopias are found. The MOMS trial demonstrated the superiority of intrauterine therapy over postpartum therapy of SBA. The aim of the study is to evaluate the prenatal hybrid surgery (laparotomy with foetoscopic closure of the SBA) performed at the centre. From 2021- 2023, fifteen foetuses were operated on. Preoperative MRIs were performed in the 19th- 26th periods. SSW and postoperative MRIs were performed in the 30th-32nd week of pregnancy according to standard protocol, T2 HASTE, DWI.
**Results or Findings:** The ratio of boys to girls was 6:9. 10 foetuses (66%) showed a meningomyelocele, 5 (33%) had rachischisis, and lesion length was median of five segments (range 48). Motor levels were 4/15 foetuses (27%) at L5 and 11/15 foetuses (73%) at S1. 100% had an ACM; 4 showed heterotopias, 3 had CC dysgenesis and 2 had syringomyelia. Postop MRI showed complete closure of the SBA and regression of the ACM in all foetuses. 4/15 foetuses (27%) had a hydrocephalus, which was treated postnatally by means of a VP shunt. The postop foetal MRI findings were confirmed by postpartum clinical examination and postpartum MRI control.
**Conclusion:** Foetal MRI provides important morphological and functional information for the pre- and postoperative evaluation of intrauterine hybrid surgery.
**Limitations:** The study is retrospective.
**Ethics committee approval:** Not applicable.
**Funding for this study:** No funding was received for this study.
**Author Disclosures:**
Siegmund Köhler: Nothing to disclose
Roland Axt-Fliedner: Nothing to disclose
Maximilian Schulze: Nothing to disclose
Verena Hohmann: Nothing to disclose
Andre Kemmling: Nothing to disclose
Corinna Keil: Nothing to disclose
Benjamin Saß: Nothing to disclose
Ivonne Bedei: Nothing to disclose

**09:30-11:00** **Research Stage 3**

### Research Presentation Session: Artificial Intelligence & Machine Learning & Imaging Informatics

### RPS 1305

### Strategic deployment of AI

**Moderator**
D. Baditescu; Bucharest/RO

**RPS 1305-2**

**Early platform release of the federated European cancer imaging infrastructure**

A. S. Alic[1], D. Arce Grilo[1], M. Birhanu[2], E. Bron[2], V. Kalokyri[3], T. Kussel[4], K. Lang[5], K. Majcen[5], *I. Blanquer*[1]; [1]Valencia/ES, [2]Rotterdam/NL, [3]Heraklion/GR, [4]Heidelberg/DE, [5]Graz/AT
*(iblanque@dsic.upv.es)*

**Purpose:** EUCAIM (https://cancerimage.eu/) is a pan-European federated infrastructure for cancer images, fueling AI innovations.
**Methods or Backround:** This federated infrastructure is built upon a set of core services that comprise a public metadata catalogue, a federated search service following a common hyperontology, an access negotiation system, a coherent AAI and a distributed processing service. EUCAIM has recently released an early prototype with 40 image datasets from nine cancer types (breast, colon, lung, prostate, rectum, liver, diffuse intrinsic pontine glioma, neuroblastoma, glioblastoma) registered, related to the five projects in the AI4HI network (EUCANIMAGE, ProCAncer-I, INCISIVE, CHAIMELEON and PRIMAGE - https://future-ai.eu/), for a total of more than 200,000 image series from approximately 20,000 individuals. These collections follow a common metadata model defined in the EUCAIM project.
**Results or Findings:** This early prototype comprises a dashboard with guiding instructions, a public catalogue, a federated search engine and an access negotiation system in beta version.
**Conclusion:** This platform will permit users to discover, search, request, access and process medical imaging and associated clinical data in a flexible manner, supporting federated providers with different access levels and a future centralised repository. EUCAIM is based on cloud and container technologies, and it will be linked to intensive computing infrastructures such as EGI and supercomputing centres.
**Limitations:** The access negotiation service is currently in beta version and access requests will be forwarded to the providers.
**Ethics committee approval:** The project acts as a broker for accessing data and relies on the ethical approvals of the providers and requesters.
**Funding for this study:** This project is co-funded by the European Union under grant agreement 101100633
**Author Disclosures:**
Valia Kalokyri: Nothing to disclose
Ignacio Blanquer: Nothing to disclose
Tobias Kussel: Nothing to disclose
David Arce Grilo: Nothing to disclose
Andrei Stefan Alic: Nothing to disclose
Konrad Lang: Nothing to disclose
Mahlet Birhanu: Nothing to disclose
Esther Bron: Nothing to disclose
Kurt Majcen: Nothing to disclose

### RPS 1305-3

**Radiology AI deployment and assessment rubric (RADAR) for value-based AI in radiology**

B-J. Boverhof[1], K. Redekop[1], D. Bos[1], M. P. A. Starmans[1], J. Birch[2], A. G. Rockall[3], *J. J. Visser*[1]; [1]Rotterdam/NL, [2]Poole/UK, [3]Godalming/UK
*(j.j.visser@erasmusmc.nl)*

**Purpose:** The aim is to provide a comprehensive framework for value assessment of AI for radiology.
**Methods or Backround:** This paper presents the RADAR framework, which has been adapted from Fryback and Thornbury's imaging efficacy framework and facilitates valuation of radiology artificial intelligence (AI) from conception to local implementation. Special attention is placed on local efficacy to underscore the importance of appraising an AI system in its local environment. The RADAR framework is illustrated through a myriad of study designs that help conduct adequate valuation.
**Results or Findings:** The RADAR approach constitutes a seven-levelled-hierarchy, providing radiologists, researchers, and decision-makers with a conceptual framework for comprehensive AI valuation in radiology. RADAR is dynamic, catering to varying valuation throughout the AI's developmental cycle. Technical and diagnostic efficacy (RADAR-1 and RADAR-2) is assessed before clinical implementation and can be addressed by in-silico clinical trials and cross-sectional studies. The next phases, encompassing diagnostic thinking to patient outcome efficacy (RADAR-3 to RADAR-5) necessitate clinical integration and can be addressed through randomised controlled trials and cohort studies. Societal efficacy (RADAR-6) delves into broader societal implications, assessed through health-economic evaluations. Concluding the hierarchy, the extent to which previous assessments generalise locally (RADAR-7) are gauged with budget impact analysis and multi-criteria decision analysis.
**Conclusion:** The RADAR framework stands as a comprehensive solution for valuing radiology. With its progressive and hierarchical approach, as well as an emphasis on local efficacy, RADAR provides a comprehensive framework to illustrate radiology AI's value conform to the notion of value-based radiology.
**Limitations:** No limitations were identified.
**Ethics committee approval:** No information provided by the submitter.
**Funding for this study:** No funding was received for this study.
**Author Disclosures:**
Ken Redekop: Nothing to disclose
Judy Birch: Nothing to disclose
Bart-Jan Boverhof: Nothing to disclose
Martijn Pieter Anton Starmans: Nothing to disclose
Daniel Bos: Nothing to disclose
Andrea Grace Rockall: Nothing to disclose
Jacob Johannes Visser: Advisory Board: travel grant from Qure.ai; participation on a data safety monitoring board or advisory board from Contextflow, Noaber Foundation, and NLC Ventures; leadership or fiduciary role on the steering committee of the PINPOINT Project (payment to institution from AstraZeneca) and RSNA Common Data Elements Steering Committee (unpaid); phantom shares in Contextflow and Quibim Advisory Board: J.J. Visser: Grant to institution from Qure.ai, Enlitic; consulting fees from Tegus; payment to institution for lectures from Roche.

### RPS 1305-4

**Knowledge of AI governance, perceived challenges, opportunities, and suggestions for AI implementation by UK radiographers**

*N. Stogiannos*[1], T. J. T. O'Regan[1], M. Pogose[1], H. Harvey[1], A. Kumar[1], R. Malik[2], A. Barnes[1], M. F. McEntee[3], C. Malamateniou[1]; [1]London/UK, [2]Farnworth/UK, [3]Cork/IE
*(nstogiannos@yahoo.com)*

**Purpose:** Radiographers are key stakeholders in AI use for clinical imaging and radiation therapy. AI Implementation is key to harness the potential benefits of AI innovation. Knowledge of AI governance by all healthcare professionals is vital for AI implementation in clinical practice. This study aims to explore UK radiographers' knowledge and perceptions on AI governance.
**Methods or Backround:** An online survey on Qualtrics was distributed to UK-practicing radiographers via social media. Eligible respondents needed to have theoretical knowledge and/or practical expertise in the use of AI in medical imaging and/or radiation therapy. Descriptive and inferential statistics was used to analyse quantitative data and content analysis for open-ended questions.
**Results or Findings:** There were 88 valid responses. Lack of training, guidance, and funding are the most important challenges to AI implementation, as perceived by radiographers. Many radiographers (36.9%) were unaware of evaluation methods for AI tools, whilst 56.6% hadn't received any AI-specific training. Robust governance frameworks (30.7%), customised training (27.3%), and patient and public involvement (21.6%) were noted as strategic priorities by respondents.
**Conclusion:** Effective leadership, allocated time, and tailored training will contribute to successful AI implementation. Further research is needed to ensure radiographers can harness the benefits and minimise risks of AI.
**Limitations:** Selection bias might have occurred in this study, since data was collected online. Also, the skewed geographical distribution of the respondents may further limit the generalisability of the results.
**Ethics committee approval:** This study was approved by the City, University of London School of Health and Psychological Sciences Research Ethics Committee (reference: ETH2122-1015).
**Funding for this study:** This study received funding from the College of Radiographers CORIPS grant scheme (grant number: 209) and the City Radiography Research Fund.
**Author Disclosures:**
Mark F. McEntee: Nothing to disclose
Amrita Kumar: Nothing to disclose
Michael Pogose: Nothing to disclose
Hugh Harvey: Nothing to disclose
Anna Barnes: Nothing to disclose
Nikolaos Stogiannos: Nothing to disclose
Tracy Jane Tracy O'Regan: Nothing to disclose
Rizwan Malik: Nothing to disclose
Christina Malamateniou: Nothing to disclose

### RPS 1305-5

**Black box no more: a survey to explore AI adoption and governance in medical imaging and radiation therapy in the UK**

*N. Stogiannos*[1], T. J. T. O'Regan[1], A. Barnes[1], A. Kumar[1], R. Malik[2], M. Pogose[1], H. Harvey[3], M. F. McEntee[4], C. Malamateniou[1]; [1]London/UK, [2]Farnworth/UK, [3]Banstead/UK, [4]Cork/IE
*(nstogiannos@yahoo.com)*

**Purpose:** The clinical use of AI tools in medical imaging and radiation therapy (MIRT) has highlighted challenges to AI adoption and governance for healthcare professionals. This study aims to map the perceived challenges around clinical adoption of AI. Opportunities associated with AI and suggestions for future implementation are explored.
**Methods or Backround:** A multidisciplinary online survey on Qualtrics® was designed using expert focus groups and published literature and piloted (n=9) before distribution. It was shared via social media and professional networks to all MIRT professionals in the UK. Data was analysed using descriptive and inferential statistics on the SPSS software, whilst content analysis was employed for the open-ended questions.
**Results or Findings:** A total of 245 valid responses were received from different MIRT professionals. Lack of knowledge of AI governance frameworks was noted (42.1%). Prior AI training was significantly correlated with understanding of AI governance concepts (p=0.007 for MHRA and 0.001 for ISO standards). Respondents indicated that clear governance frameworks (11.4%), AI training (9%) and effective leadership (8.5%) are vital for successful AI adoption.
**Conclusion:** Knowledge of, and confidence in AI technologies correlate with prior AI-related training. Different professionals were familiar with frameworks related to their practice. Tailored AI training is needed to address knowledge gaps for a safe and successful AI adoption in medical imaging and radiation therapy in the UK.
**Limitations:** The small sample size of this study means results cannot be generalised to the broader UK medical imaging and radiation therapy AI ecosystem.
**Ethics committee approval:** This study was approved by the City, University of London School of Health and Psychological Sciences Research Ethics Committee (reference: ETH2122-1015).
**Funding for this study:** This study received funding from the College of Radiographers CORIPS grant scheme (grant number: 209) and the City Radiography Research Fund.
**Author Disclosures:**
Mark F. McEntee: Nothing to disclose
Amrita Kumar: Nothing to disclose
Michael Pogose: Nothing to disclose
Hugh Harvey: Nothing to disclose
Anna Barnes: Nothing to disclose
Nikolaos Stogiannos: Nothing to disclose
Tracy Jane Tracy O'Regan: Nothing to disclose
Rizwan Malik: Nothing to disclose
Christina Malamateniou: Nothing to disclose

### RPS 1305-6

**Radiographer education and learning in artificial intelligence (REAL-AI)**

*G. Doherty*[1], L. McLaughlin[1], R. Bond[1], J. McConnell[2], C. Hughes[1], S. L. McFadden[1]; [1]Belfast/UK, [2]Leeds/UK
*(doherty-g49@ulster.ac.uk)*

**Purpose:** Artificial intelligence (AI) is widespread in medical imaging, yet there is a paucity of information on education and training available for staff. Further research is required to identify what training is available, and what preparations are required to bring AI knowledge to levels that will enable radiographers to work competently alongside AI. This study aimed to: a) investigate current provision of AI education at UK higher education institutes (HEIs); b) explore the attitudes and opinions of educators.
**Methods or Backround:** Data were collected through two online surveys: 1) UK HEIs; 2) medical imaging educators. The surveys were distributed in the UK by the heads of radiography education (HRE), The Society of Radiographers and as part of the Research Hub at ECR 2023. The study was promoted on LinkedIn and Twitter (X), and through university channels.
**Results or Findings:** Responses were received from 22 HEIs in the UK and 33 educators from across Europe. Data analysis is ongoing, but preliminary findings show that 68.2% (n=15) of responding HEIs claim to have introduced AI into the curriculum already. 84.8% (n=28) of educators claim they themselves have received no training on AI despite having to embed it into the curriculum. The main reason for this, as cited by HEIs, is limited resources. 69.7% (n=23) of educators believe that AI concepts should be taught by an AI expert.
**Conclusion:** By surveying educators and HEIs separately, this study captured two different perspectives regarding the provision of AI education. This unique insight highlighted disharmony between HEIs and educators. Preliminary insights highlight that educators feel unprepared to deliver AI content, and HEIs are under pressure to add AI concepts to an already full curriculum.
**Limitations:** An identified limitation was that surveys, focus groups and interviews were conducted in the English language only.
**Ethics committee approval:** This study was approved by the Ulster University Filter Committee. Reference numbers: FCNUR-23-051 / FCNUR-23-006-A.
**Funding for this study:** This project has been part-funded by a College of Radiographers Industry Partnership Scheme, grant number 229 (AI).
**Author Disclosures:**
Jonathan McConnell: Nothing to disclose
Laura McLaughlin: Nothing to disclose
Ciara Hughes: Nothing to disclose
Raymond Bond: Nothing to disclose
Sonyia Lorraine McFadden: Nothing to disclose
Geraldine Doherty: Nothing to disclose

### RPS 1305-7

**International medical students' perceptions towards artificial intelligence in medicine: a multicentre, cross-sectional survey among 192 universities**

*F. Busch*[1], L. Hoffmann[1], D. Truhn[2], M. Makowski[3], K. K. Bressem[1], L. C. Adams[3]; [1]Berlin/DE, [2]Aachen/DE, [3]Munich/DE
*(felix.busch@charite.de)*

**Purpose:** Artificial intelligence (AI) is set to fundamentally change the educational and professional landscape for the next generation of physicians worldwide. This study aimed to explore the current international attitude of medical students towards AI in the medical curriculum and profession on a large, global scale and identify factors that shape their attitudes.
**Methods or Backround:** This multicentre, multinational cross-sectional study developed and validated an anonymous online survey of 15 multiple-choice items to assess medical, dentistry, and veterinary students' preferences for AI events in the medical curriculum, the current state of AI education, and students' AI knowledge and attitudes towards using AI in the medical profession. Subgroup analyses were performed considering gender, age, study year, tech-savviness, prior AI knowledge and AI events in the curriculum, and university location.
**Results or Findings:** Between April and October 2023, a total of 4,313 medical, 205 dentistry, and 78 veterinary students from 192 faculties and 48 countries responded to the survey. Most participants came from European countries (n=2,350), followed by North/South America (n=1,070) and Asia (n=944). Students showed predominantly positive attitudes towards AI in medicine (67.6%, n=3,091) and expressed a strong desire for more AI education (76.1%, n=3,474). However, they reported limited general knowledge of AI (75.3%, n=3,451) and felt inadequately prepared to use AI in their future careers (57.9%, n=2,652). Subgroup analyses revealed differences in attitudes between students from the Global South and North and on the continental level, among others.
**Conclusion:** This large-scale international study underlines the generally positive attitude of medical students towards the application of medical AI and explores variables that influence such attitudes. Our study highlights the necessity for a greater emphasis on AI education within medical curricula.
**Limitations:** The unequal regional representation and selection bias were identified as limitations.
**Ethics committee approval:** This study was approved by the IRB, with approval code: EA4/213/22.
**Funding for this study:** The authors report the results on behalf of the COMFORT consortium, an initiative of the Horizon Europe-funded COMFORT project (101079894).
**Author Disclosures:**
Lena Hoffmann: Nothing to disclose
Keno K. Bressem: Nothing to disclose
Lisa C. Adams: Nothing to disclose
Felix Busch: Nothing to disclose
Daniel Truhn: Nothing to disclose
Marcus Makowski: Nothing to disclose

### RPS 1305-8

**AI in routine teleradiology use: results of a large-scale test across Germany and Austria**

*T. B. T. Moeller*, P. F. W. Sögner; Dillingen/DE
*(moeller@reif-moeller.de)*

**Purpose:** The objective of this study was to answer the question of whether the use of AI is already having a quality-improving effect in routine teleradiological reporting throughout Germany and Austria.
**Methods or Backround:** We performed a study of 2,707 native CCT scans from the CT departments of 140 hospitals in Germany and Austria between March and April 2022, which were analysed using AI with haemorrhage analysis. The results were compared with the findings of more than 70 teleradiologists who did not have the AI results at that time. Possible discrepant findings were evaluated by two radiologists with specific neuroradiological CCT experience. Additionally, the in-house error statistics from 2021 and 2022-23 were reviewed.
**Results or Findings:** Of the 2,707 CCT examined by both radiologists and AI, 189 cases (approximately seven percent) were found to have intracranial haemorrhage described by both radiologists and AI. In 30 patients there was a discrepancy: the AI had seen a haemorrhage that had not been described by the radiologist. These cases were subsequently re-evaluated. Twelve (approximately 40%) of the 30 unclear examinations were classified as false positives by the AI, eight cases as questionable positives, and 10 cases as true positives. Thus, there were 199 cases with ICB in the studied patient group, of which > 5% were primarily missed by radiologists without AI support. A review of in-house error statistics also revealed a significant decrease in reported false findings for intracranial haemorrhage (from 16 in 2021 to one between 08/31/2022 and 08/31/2023).
**Conclusion:** The positive effects of AI on the quality of radiological reporting postulated in several studies can also be confirmed in practice and especially in the teleradiological context.
**Limitations:** This assumption should be substantiated by further studies.
**Ethics committee approval:** No information provided by the submitter.
**Funding for this study:** No funding was received for this study.
**Author Disclosures:**
Peter Franz Walter Sögner: Nothing to disclose
Torsten Bert Thomas Moeller: Nothing to disclose

### RPS 1305-9

**Artificial intelligence should only read a mammogram when it is certain: a hybrid breast cancer screening reading strategy**

*S. D. Verboom*, J. Kroes, S. Pires, M. Broeders, I. Sechopoulos; Nijmegen/NL
*(sarah.verboom@radboudumc.nl)*

**Purpose:** The aim of this study was to incorporate and evaluate uncertainty quantification metrics in an artificial intelligence (AI) breast cancer detection model and test their ability to guide a novel hybrid reading strategy in breast cancer screening in which recall decisions are only made by standalone AI when it exhibits high certainty.
**Methods or Backround:** Uncertainty quantification metrics were obtained from a modified version of a commercial AI breast cancer detection model by structured Monte Carlo dropout. The metrics were defined as the variance or entropy of one or all suspicious regions and used to estimate the certainty of the AI malignancy-present decision. With the proposed hybrid reading strategy, the recall decision is based on AI only when the predictions are classified as certain, and by standard radiologist-double reading otherwise. The new reading strategy was retrospectively tested on a previously-unseen subset of all digital mammographic screening examinations acquired between 2003-2018 from a unit of the Dutch National Breast Cancer Screening (n=41,469) with minimal 2-year follow-up.
**Results or Findings:** The best-performing uncertainty metric was the entropy of the mean output for the most suspicious region per case. The hybrid reading strategy using this uncertainty metric and a recall rate equal to the standard radiologist-double-reading strategy (27 per 1000) resulted in 46% of cases read by AI only and a cancer detection rate of 8.1 per 1000, which does not differ from the standard strategy (8.0 per 1000, p=0.217). The mean AUC of the AI model increased from 0.957 (95% CI 0.944-0.969) for all cases to 0.984 (95% CI 0.970-0.995) for the 46% of cases classified as certain (p<0.001).
**Conclusion:** Leveraging AI uncertainty to guide a hybrid AI-radiologist screening reading strategy can potentially reduce workload by ~46% without decreasing performance.

**Limitations:** Identified limitations were that this was a retrospective study with single-site data.
**Ethics committee approval:** According to the Dutch Central Committee on Research involving Human Subjects, ethical
approval was not necessary.
**Funding for this study:** aiREAD was financed by NWO, KWF, HH.
**Author Disclosures:**
Jaap Kroes: Employee: with Screenpoint Medical
Mireille Broeders: Speaker: Hologic, Siemens Healthcare; Research/Grant Support: Screenpoint Medical, Sectra Benelux, Hologic, Volpara Solutions, Lunit inc., iCAD
Santiago Pires; Employee: with Screenpoint Medical
Sarah Delaja Verboom: Nothing to disclose
Ioannis Sechopoulos: Speaker: Siemens Healthcare; Research/Grant Support: Siemens Healthcare, Canon Medical, Screenpoint Medical, Sectra Benelux, Hologic, Volpara Solutions, Lunit Inc., iCAD; Advisory Board: Koning Corp.

### RPS 1305-10

**Setting up a complaint data registry for research on the human: a Swiss experience**
*B. Dufour*[1], B. Rizk[1], C. Thouly[1], H. Brat[1], N. Heracleous[1], D. Goyard[2], P. Petetin[3], F. Zanca[4]; [1]Sion/CH, [2]Paris/FR, [3]Berre l'Etang/FR, [4]Leuven/BE
*(benoit.dufour@groupe3r.ch)*

**Purpose:** Since 2014, the Law on Human Research (LRH) in Switzerland protects individuals participating in human research projects, while ensuring quality and transparency. We detail the establishment of a Complaint Data Registry (CDR) within a private radiology network in Switzerland.
**Methods or Backround:** Data in the registry encompass DICOM images, examination reports, and clinical/demographic information. Key elements in creating the registry included defining its purpose and objectives, establishing governance (legal structure, general informed consent, access rights), and outlining operational procedures (data storage duration, pseudonymisation, encryption key access). For governance, we structured the organisational framework and designated responsible individuals. A workflow for informed consent, including consent for AI-based image analysis, was implemented. Patients receive an SMS before appointments, granting access to information about the data registry and consent process. Patients can opt in or out for research by digitally signing the consent form on their smartphone or at the centre on the day of the exam. Signed consents are stored in our RIS, allowing radiologists to identify approved research and AI-analysed data. For the operational processes, data are collected on a gateway, pseudoanonymised and sent to a cloud platform for storage, while ensuring segregation based on the data's source sites and projects.
**Results or Findings:** Results showed that 780,000 research consents were automatically stored in the RIS database between 18.01.2023-03.10.2023, with 678,235 consenting research data reuse (87%). Since implementing the registry, patient consent for AI-based data analysis increased from 56% to 92%.
**Conclusion:** Our experience in setting up a CDR could serve as a promising model for other institutions seeking to improve healthcare outcomes by leveraging complaint data.
**Limitations:** The Swiss context might be different in other countries and other RIS systems might not guarantee the same level of integration.
**Ethics committee approval:** The study is about setting up a data registry.
**Funding for this study:** No funding was received for this study.
**Author Disclosures:**
Natalie Heracleous: Nothing to disclose
Cyril Thouly: Nothing to disclose
Benoît Dufour: Nothing to disclose
Benoît Rizk: Nothing to disclose
David Goyard: Nothing to disclose
Hugues Brat: Nothing to disclose
Pierre Petetin: Nothing to disclose
Federica Zanca: Nothing to disclose

### RPS 1305-11

**Advancements in generative AI for radiological imaging**
E. Hofmeijer, X. Zu, *C. O. Tan*; Enschede/NL

**Purpose:** Generative artificial intelligence (AI) has emerged as a transformative force in the field of radiology. It can empower radiologists with tools to enhance image quality, reconstruct degraded data, and synthesize realistic images, improving diagnostic accuracy and efficiency. In particular, generative AI enables creation of synthetic datasets that facilitate training algorithms, as well as residents and fellows, when real-world data is scarce or difficult to obtain due to privacy concerns.
**Methods or Backround:** We have recently developed a pipeline for creating artificial 2D radiologic images. Publicly available standard and low-dose chest CT images (805 scans; 39,803 2D images, 17% containing lung nodules) were used to generate synthetic image. Five radiologists with experience in chest and thoracic imaging were asked to assess synthetic image quality compared to the real ones.
**Results or Findings:** Radiologists rated artificial images as 3.13 ± 0.46 (1 [unrealistic] to 4 [indistinguishable to the original image]), close to their rating of the original images (3.73 ± 0.31). An extended diffusion-based model was then used to identify features of the lung nodules that distinguish malignant versus benign ones and to generate further synthetic images that should reflect these features. The accuracy of malignant/benign classification based on synthetic images reached an accuracy of 85.5%.
**Conclusion:** Our results show that synthetic radiologic images are realistic and reliably adhere to the key radiographic features that are reflective of pathological changes. These results, when shown to be reliable across imaging modalities, organs, and pathologies, can enable tailored synthetic images on individual, personalised, patient profiles ("digital twins").
**Limitations:** The ethical considerations surrounding the use of generative AI in radiology need to be addressed.
**Ethics committee approval:** The data used for this work is based on publicly available sources: Lung Image Database Consortium (LIDC) and Image Database Resource initiative (IDRI).
**Funding for this study:** This study was funded by a ZonMw Innovative Medical Devices Initiative (IMDI) subsidy for the B3CARE project (dossier number: 10-10400-98-008).
**Author Disclosures:**
Elfi Hofmeijer: Nothing to disclose
Can Ozan Tan: Nothing to disclose
Xinrui Zu: Nothing to disclose

### RPS 1305-12

**Improving CT justification practices with machine learning and deep learning: a multi-site study**
*J. Potočnik*, E. M. Thomas, A. Lawlor, D. Kearney, R. P. Killeen, E. J. Heffernan, S. J. Foley; Dublin/IE
*(jaka.potocnik@ucd.ie)*

**Purpose:** The aim of this study was to compare human experts with machine learning (ML) and deep learning (DL) models for assessing justification of CT brain referrals. Multiclass classification of the anonymised referrals with ML and DL determined if prediction models could generalise and automate this process.
**Methods or Backround:** Anonymised adult brain CT referrals performed in 2020 and 2021 were sourced from three Irish CT centres. A total of 3,000 referrals were randomly selected. Two radiologists and radiographers retrospectively categorised the referrals using iGuide as: justified, unjustified, or potentially justified. The final justification label for each referral was determined by majority vote or consensus. Prior to the feature extraction with bag-of-words (BoW), term frequency-inverse document frequency, and Word2vec models, word tokenisation, stop words removal, and Enchant spell correction of unstructured clinical indications was performed. The dataset was randomly split into stratified training and test sets (80/20). Downsampling to the minority class ensured class balance. Support vector machines, logistic regression, gradient boosting classifier (GBC), multi-layer perceptron, and bidirectional long-short term memory neural network were evaluated. Their hyperparameters were tuned on the training set.
**Results or Findings:** A total of 11,090 referrals were collected and a random sample of 3,000 were reviewed. 238 (8.1%) were categorised as unjustified, 811 (27.4%) potentially justified, and 1,909 (64.5%) justified by raters. The best-performing classifier (BoW+GBC) achieved 94.4% accuracy and macro precision, recall, and F1 scores of 0.94.
**Conclusion:** ML and DL-based approaches can generalise and accurately predict the justification of radiology referrals in accordance with the iGuide categorisation. This may help in addressing poor European justification practices.
**Limitations:** Downsampling resulted in a smaller dataset for multiclass classification, which, in turn, led to suboptimal performance in DL. A larger, more representative dataset, along with a validation set, may provide better insights into performance.
**Ethics committee approval:** The University ethics committee has approved an ethics exemption (LS-E-21-216-Potocnik-Foley) based on the outcomes of local DPIAs.
**Funding for this study:** No funding was received for this study.
**Author Disclosures:**
Eric John Heffernan: Nothing to disclose
Jaka Potočnik: Nothing to disclose
Aonghus Lawlor: Nothing to disclose
Edel Mary Thomas: Nothing to disclose
Dearbhla Kearney: Nothing to disclose
Ronan P. Killeen: Consultant: xWave Technologies
Shane J. Foley: Nothing to disclose

### RPS 1305-13
**Artificial Intelligence in automated protocolling for Finnish brain MRI referrals**
*H. Huhtanen*, M. J. Nyman, A. Karlsson, J. Hirvonen; Turku/FI
*(hejohuh@utu.fi)*

**Purpose:** Advancements in AI-driven models for natural language processing has offered opportunities to automate many menial tasks that require understanding written text. Automating the protocolling of incoming MRI referrals could reduce interruptions in radiologists' workflow. The purpose of this study is to test different AI models in assigning a suitable protocol and the need for contrast medium for emergency brain MRI referrals.
**Methods or Backround:** For training and testing the models, we collected 1,563 and 390 Finnish emergency brain MRI referral texts, respectively. Data was labelled according to suitable imaging protocol and the need for contrast medium. We trained baseline machine learning (ML) models (three different algorithms) and newer deep learning (DL) models (BERT and GPT3) for classification. We also tested whether using less training data (50% of the training set) or using less data but upsampling it with augmentation affected model performance.
**Results or Findings:** In protocol and contrast medium prediction, GPT3 outperformed other models with accuracies of 84% and 91%, respectively. BERT models had accuracies of 78% and 89%, and the best ML models 77% and 86%, respectively. For DL models, using less training data affected performance negatively. Upsampling the data with augmentation boosted BERT's accuracy in the protocol task but not in the contrast medium task. For ML models, neither dataset size nor augmentation seemed to affect performance.
**Conclusion:** Our results show that there is potential in using AI in automatic protocolling. Although GPT3 outperformed other algorithms, BERT and ML models also performed well. However, the DL models seem to have more potential to improve performance with increasing dataset size, than the ML models.
**Limitations:** The limitations of this study are the high imbalance between MRI protocol classes and using data from only one institute.
**Ethics committee approval:** Review by the ethics committee was waived due to the retrospective nature of this study in accordance with national legislation.
**Funding for this study:** Funding was provided by the Emil Aaltonen Foundation (grant number: 230049), and the Radiological Society of Finland.
**Author Disclosures:**
Jussi Hirvonen: Nothing to disclose
Mikko Juhani Nyman: Nothing to disclose
Antti Karlsson: Nothing to disclose
Heidi Huhtanen: Nothing to disclose

**09:30-11:00 Research Stage 4**

### Research Presentation Session: Chest

### RPS 1304

### Advanced imaging of thoracic oncologic patients

**Moderator**
R. Cesar; Golnik/SI
*(rok.cesar@klinika-golnik.si)*

### RPS 1304-2
**DEB-BACE followed by systemic chemotherapy vs systemic chemotherapy alone for advanced lung adenocarcinoma: a propensity score match study**
J. Tu, *L. Lai*; Lishui/CN
*(1040121084@qq.com)*

**Purpose:** This retrospective study aimed to investigate the effectiveness and safety of sequential bronchial arterial chemoembolisation with drug-eluting beads (DEB-BACE) and chemotherapy versus systemic chemotherapy alone for advanced lung adenocarcinoma progressing on targeted therapy.
**Methods or Backround:** Stage III or IV lung adenocarcinoma patients in the chemotherapy group received an intravenous injection of pemetrexed 500 mg/m2 plus cisplatin 75 mg/m2 on day 1 for 4 cycles, with each cycle lasting for 21 days. Patients in the DEB-BACE plus chemotherapy group underwent DEB-BACE using CalliSpheres drug-eluting beads loaded with gemcitabine (400 mg) and received via the microcatheter cisplatin 75 mg/m2 and gemcitabine 600 mg/m2 followed by intravenous chemotherapy 3 weeks post DEB-BACE. The primary outcome was overall survival.
**Results or Findings:** Sixty-two patients who received chemotherapy and 69 who received DEB-BACE plus chemotherapy were included, with 36 patients in each group after PSM. After PSM, the median OS was 18.3 months in the chemotherapy group and 33.1 months in the DEB-BACE plus chemotherapy group ($P < 0.001$). Multivariate Cox regression analysis showed that DEB-BACE plus chemotherapy was associated with an 82% reduction in the risk of death versus chemotherapy only ($P < 0.001$). After PSM, treatment-emergent adverse events of grade 3 or worse occurred in 2 of 36 patients in the DEB-BACE plus chemotherapy group and 16 of 36 patients in the chemotherapy alone group.
**Conclusion:** DEB-BACE plus chemotherapy improves the response rate and extends the survival of III-IV lung adenocarcinoma patients progressing on targeted therapy. This offers this patient population, who otherwise have a rather dismal clinical outcome, an effective and safe treatment option.
**Limitations:** DEB-BACE has a learning curve in terms of target tumour feeding vessel selection, the number of embolised vessels, levels of embolisation and size of embolisation beads.
**Ethics committee approval:** The study was approved by the Research Ethics Group of the Ethics Committee at Lishui Central Hospital: ethical review of research (2022) number 373.
**Funding for this study:** Funding was received from the Medical and Health Science and Technology Plan of Zhejiang Province (grant number WKJ-ZJ-1932).
**Author Disclosures:**
Linqiang Lai: Nothing to disclose
Jianfei Tu: Nothing to disclose

### RPS 1304-3
**Tree-based models for predicting clinically significant pneumothorax in patients undergoing percutaneous coaxial core lung biopsy: a retrospective cohort study**
*M. E. Chevasco Hanze*, D. Castellon Plaza, S. A. Bolivar, B. Del Rio Carrero, H. Jofre; Barcelona/ES
*(miguelemilioch27@gmail.com)*

**Purpose:** This study aimed to create a prediction model for the development of significant pneumothorax following a CT-guided coaxial core lung biopsy (CT-CCLB) by employing machine learning tree-based models.
**Methods or Backround:** A total of 469 patients who underwent CT-CCLB were retrospectively included. A list of 22 patient, procedure and lesion characteristics were retrieved. Boruta analysis was used for selection of feature predictors. Afterwards, four tree models, namely CART, AdaBoost, GB and XGBoost, were applied. The final model was chosen based on PPR, PLR and AUC values. Final model and predictors behaviour were further evaluated by tree plot and SHAP analysis.
**Results or Findings:** Significant pneumothorax rate was 12.79%. GB classifier was found to have the best discriminating power (AUC = 76.82%; PPR = 3.80; PLR = 5.08 43.48%). The top five predictors were lesion size/depth, DLCO, BMI and involvement of fissures/bullae/emphysema during biopsy. Scenarios for encountering the highest significant pneumothorax occur when: 1) the mentioned surfaces are compromised and the procedure is done with the patient in a supine or lateral body position; 2) lesion size is lower than 22.37 mm and BMI is lower than 26.5; 3) lesion size is higher than 22.37 mm and depth is higher than 36.86 mm.
**Conclusion:** Significant pneumothorax after CT-CCLB was more likely to develop among patients with small lesions, lower BMI, higher depth, and biopsies done in prone/lateral position and with fissures/bullae/emphysema. Machine-learning models demonstrated a high predictive performance, with results being easy to visualise and read.
**Limitations:** Outweighing outcome was variable. Biopsies were made by radiology residents and attending radiologists
**Ethics committee approval:** No ethics committee approval was required because the present study was retrospective, with no intervention throughout the research.
**Funding for this study:** No funding was received for this study.
**Author Disclosures:**
Daniel Castellon Plaza: Nothing to disclose
Belén Del Rio Carrero: Nothing to disclose
Miguel Emilio Chevasco Hanze: Nothing to disclose
Hector Jofre: Nothing to disclose
Santiago A. Bolivar: Nothing to disclose

### RPS 1304-4
**CT-guided core needle biopsy is safe and accurate for the assessment of pulmonary lesions associated with cystic airspaces**
*M. Balbi*[1], N. C. Culasso[1], M. Barba[1], R. Senkeev[1], S. Capelli[2], A. Caroli[2], F. Filipello[3], L. Righi[1], A. Veltri[1]; [1]Orbassano/IT, [2]Bergamo/IT, [3]Verduno/IT
*(balbi.m@libero.it)*

**Purpose:** This study aimed to evaluate the safety and diagnostic capability of CT-guided core needle biopsy (CNB) in pulmonary lesions associated with cystic airspaces (PLACAs).

**Methods or Backround:** Consecutive pulmonary biopsies performed at the San Luigi Gonzaga Hospital (Orbassano, Italy) from February 2010 to January 2022 (n=3069) were retrospectively reviewed to identify patients who underwent CNB for PLACAs (n=90, case group; median age, 69.5 years, 95% confidence interval [CI], 62.0-75.0; 28 females). A group of CNB patients with non-cystic lesions matched for age, sex, emphysema, and lesion depth and dimensions (n=180, control group) was selected to compare the diagnostic yield and complication rate. The diagnostic performance for the final diagnosis was calculated. Univariate and multivariate logistic regressions were performed in case patients to identify risk factors for complications and a non-diagnostic specimen (i.e., nonspecific benignity, atypical cells, insufficient specimen). PLACAs' specimens were reviewed to assess histopathology.
**Results or Findings:** There were no significant differences between cases and controls in complication rate (overall: 40% versus 38%; major: 4% versus 6%, respectively) and non-diagnostic specimens (12% versus 9%). The diagnostic performance was similar in both groups (accuracy: 97.78% vs. 97.78%, sensitivity: 97.53% vs. 97.63%, specificity: 100% vs. 100%). Among the patient, procedural, and lesion-related data, the length of the needle pathway through the lung (odds ratio, [OR], 2.86; [95% CI, 1.08-7.80]; p=0.036) and the procedure time (OR, 10.93; 95% CI, 3.77-35.85; p < 0.001) were significant risk factors for complications. No variables predicted a non-diagnostic specimen. In most cases, PLACAs were adenocarcinoma (54%), and the cystic airspaces corresponded to tumour cystification (22 out of 31 resected specimens, 71%).
**Conclusion:** CT-guided CNB was safe and effective for assessing PLACAs. A long needle pathway and procedure time increased the complication risk.
**Limitations:** Identified limitations were (1) that this was a single-centre study and (2) the limited number of cases.
**Ethics committee approval:** The ethics committee approved the present study and waived the need for written informed consent.
**Funding for this study:** The authors state that this work has not received any funding.
**Author Disclosures:**
Serena Capelli: Nothing to disclose
Luisella Righi: Nothing to disclose
Andrea Veltri: Nothing to disclose
Rouslan Senkeev: Nothing to disclose
Noemi Cristina Culasso: Nothing to disclose
Federica Filipello: Nothing to disclose
Anna Caroli: Nothing to disclose
Maurizio Balbi: Nothing to disclose
Matteo Barba: Nothing to disclose

### RPS 1304-5

**Haemorrhage risk prediction after computed tomography-guided lung biopsy: combining clinical parameters and quantitative pulmonary vascular analysis**
*K-C. Lin*, Y-S. Huang, Y-C. Chang; Taipei/TW
*(kerrapple@gmail.com)*

**Purpose:** This study aimed to evaluate the utility of combining quantitative pulmonary vasculature measures with clinical factors for predicting pulmonary haemorrhage after computed tomography (CT)-guided lung biopsy.
**Methods or Backround:** Patients who underwent CT-guided lung biopsy were retrospectively included in this study. Clinical and radiographic variables were evaluated as predictors of pulmonary haemorrhage. The radiographic pulmonary vascular analysis included vessel count, vessel density, vessel diameter, vessel area, blood volume in small vessels with a cross-sectional area ≤ 5 mm2 (BV5), and total blood vessel volume (TBV) in the lungs. Univariate and multivariate logistic regressions were used to identify the independent risk factors of higher-grade pulmonary haemorrhage and establish the prediction model, which was presented in the form of a nomogram.
**Results or Findings:** A total of 126 patients was included (discovery cohort n=103, validation cohort n=23). Any pulmonary haemorrhage, higher-grade (grade ≥2) pulmonary haemorrhage, and hemoptysis occurred in 42.9%, 15.9%, and 3.2% of patients who underwent CT-guided lung biopsies. In the discovery cohort, patients with greater lesion depth (p=0.013), higher vessel density (p=0.033), and higher BV5 (p=0.039) were more likely to experience higher-grade haemorrhage. The nomogram prediction model for higher-grade haemorrhage built by the discovery cohort showed similar performance in the validation cohort.
**Conclusion:** Higher-grade pulmonary haemorrhage may occur after CT-guided lung biopsy. Lesion depth, vessel density, and BV5 are independent risk factors for higher-grade pulmonary haemorrhage. Nomograms that integrate both clinical parameters and radiographic pulmonary vasculature measures offer enhanced capability for the assessment of haemorrhage risk following CT-guided lung biopsy, thereby facilitating improved clinical care for patients.
**Limitations:** No limitations were identified.
**Ethics committee approval:** This study was approved by the Institutional Research Ethics Committee: approval number 202306051RIN.
**Funding for this study:** No funding was received for this study.
**Author Disclosures:**
Yeun-Chung Chang: Nothing to disclose
Yu-Sen Huang: Nothing to disclose
Keng-Chian Lin: Nothing to disclose

### RPS 1304-6

**De novo low-dose CT-guided lung biopsy technique: minimising radiation with maintained safety and diagnostic yield rates**
S. Vijayakumar[1], *A. Banerjee*[2], G. Tsaknis[2]; [1]Leicester/UK, [2]Kettering/UK
*(dravik11@gmail.com)*

**Purpose:** The purpose of this study is to present our innovative approach in developing a de novo low-dose CT-guided lung biopsy technique, aiming to significantly reduce radiation exposure (DLP less than 100 mGy-cm, only slightly more than CT fluoroscopy) without compromising histopathological accuracy or increasing complication rates.
**Methods or Backround:** We conducted a retrospective analysis of lung biopsy procedures performed using our novel low-dose CT-guided biopsy technique. A total of 100 patients with suspected lung lesions were included. The procedure involved precise planning and real-time image guidance, striking a balance between kVp and mAs to ensure that diagnostic image quality is maintained without unnecessary radiation exposure to the patient. Patient demographics, lesion characteristics, procedural details, radiation dose, histopathological findings, and post-procedural complications were meticulously recorded and analysed.
**Results or Findings:** The detailed findings are still in process, but some of the highlights include: 1) over 90-95% of cases had DLP below 100 mGy·cm, with almost all cases under 150 mGy·cm; and 2) varied kVp and mAs permutations were explored. Our protocol utilised 100kVp and 50mA, dropped to 30mA during biopsy, utilising precise planning and positions for challenging lung lesions, effectively reducing radiation doses.
**Conclusion:** Our study introduces a novel low-dose CT-guided lung biopsy technique, reducing radiation exposure to DLP <100mGy·cm while preserving high histopathological accuracy and patient safety. This approach is especially significant for patients needing recurrent scans and biopsies. Its effectiveness positions it as a potential standard, enhancing healthcare quality.
**Limitations:** For patients with very high BMI, increased radiation dose was necessary to target the lesion, usually higher than our target of 100kVp.
**Ethics committee approval:** No information provided by the submitter.
**Funding for this study:** No funding was received for this study.
**Author Disclosures:**
Georgios Tsaknis: Nothing to disclose
Siddharth Vijayakumar: Nothing to disclose
Avik Banerjee: Nothing to disclose

### RPS 1304-7

**Adherence to CT surveillance guidelines in early stage post-treatment lung cancer recurrence**
*S. Kneafsey*, D. P. Garrahy, D. Byrne, P. Beddy; Dublin/IE
*(siankneafsey@gmail.com)*

**Purpose:** Detection of lung cancer recurrence in patients post treatment can result in survival benefit, and enable early detection of new primary lung cancer. However, adherence to post-treatment surveillance protocols is highly variable. We assessed the adherence to CT surveillance guidelines in all patients treated with curative resection for non-small cell lung cancer (NSCLC) at our tertiary referral lung cancer centre.
**Methods or Backround:** All patients treated with curative intent surgery are followed up in a nurse led outpatient clinic. Patient demographics, postoperative imaging studies, histopathological results and follow-up appointment schedule were recorded. We defined the minimal standard of surveillance imaging studies (MSSIS) as ≥5 CT studies in the first 5 years as specified in our follow-up guidelines, based on the ESMO guidelines.
**Results or Findings:** A total of 1243 patients were included. The mean age was 66 ± 9.7. Of these, 579 (46.6%) were male, 665 (53.4%) were female. In total, 787 (63.3%) achieved the minimal standard of surveillance imaging studies directly within our centre, and are undergoing ongoing active surveillance. A further 170 (13.6%) patients achieved the minimal standard of surveillance imaging studies and were discharged from the surveillance service at five years post-surgery. A further 127 (10.2%) patients were discharged to surveillance services in satellite referral hospitals. A total of 149 (12%) patients achieved MSSIS but have since died. Five (0.4%) patients achieved MSSIS but are no longer under surveillance due to palliation. Five (0.4%) patients are no longer under active surveillance due to unknown reasons.
**Conclusion:** A total of 1111 (89.3%) patients achieved MSSIS at our institution during this surveillance period. Over two-thirds (63.6%) of patients are undergoing active surveillance. Existence of a dedicated nurse-lead lung cancer surveillance outpatient clinic had led to higher surveillance adherence rates than international literature reports.
**Limitations:** No limitations were identified.
**Ethics committee approval:** This study was approved by St. James Hospital Research and Innovation Office: project 8410.

**Funding for this study:** No funding was received for this study.
**Author Disclosures:**
Peter Beddy: Nothing to disclose
Danielle Byrne: Nothing to disclose
Sian Kneafsey: Nothing to disclose
Darragh Patrick Garrahy: Nothing to disclose

### RPS 1304-8

**Coronary calcification and interstitial lung disease are both independently associated with increased mortality in patients undergoing radiotherapy for stage 3 non-small cell lung cancer**

C. P. McKeag[1], *E. Hughes*[1], E. McGarry[1], S. Ghatorae[2], G. Cowell[1], J. Maclay[1]; [1]Glasgow/UK, [2]Larbert/UK
*(emily.hughes@nhs.scot)*

**Purpose:** Patients diagnosed with lung cancer are often multi-morbid. Assessment of comorbidities is possible on diagnostic CT and may influence survival.
**Methods or Backround:** We looked at overall survival outcomes in patients diagnosed with stage 3 non-small cell lung cancer undergoing radical radiotherapy (+/- systemic treatment) in the West of Scotland between 2017-2020. Mortality was right censored at two years, and the cohort consisted of 431 individuals. This was an update to a previous review of outcomes 2017-2019. We reviewed the diagnostic CT to identify common comorbidities including emphysema, coronary artery calcification, interstitial lung disease, and pleural effusion. Each of these was individually assessed for any impact on survival, and Kaplan-Meier curves were generated, with the aim of identifying potential markers of increased mortality.
**Results or Findings:** We showed a significant increase in overall mortality for patients with interstitial lung disease (ILD) ($p<0.005$), and for patients with severe coronary artery calcification compared to those with mild or less calcification ($p<0.005$). There was no statistically significant difference in mortality between moderate and mild or less coronary calcification ($p=0.13$), nor between moderate and severe ($p=0.23$). There was no statistically significant difference in mortality in patients with or without pleural effusion ($p=0.37$), nor between severe emphysema and mild or no emphysema ($p=0.49$).
**Conclusion:** Severe coronary artery disease and ILD are associated with reduced overall survival in patients undergoing radical radiotherapy for NSCLC. Further studies investigating cardiovascular complications of radiotherapy are required.
**Limitations:** No limitations were identified.
**Ethics committee approval:** This was a retrospective study.
**Funding for this study:** No funding was received for this study
**Author Disclosures:**
Gordon Cowell: Nothing to disclose
Simrit Ghatorae: Nothing to disclose
Emily Hughes: Nothing to disclose
John Maclay: Nothing to disclose
Erin McGarry: Nothing to disclose
Conor P. McKeag: Nothing to disclose

### RPS 1304-9

**Radiogenomics relationship of non-small cell lung cancer: preliminary results**

*M. P. Belfiore*, M. Sansone, V. Patanè, R. Monti, F. Grassi, G. Ciani, R. Grassi, S. Cappabianca; Naples/IT
*(mariapaola.belfiore@unicampania.it)*

**Purpose:** The aim of this study was to correlate the radiomics features with the genetic results obtained from liquid biopsy in patients with lung tumours.
**Methods or Backround:** We included 53 patients suffering from NSCLC who underwent pre-surgery CT (GE Revolution 128 MDCT) at the Radiology Department of the Campania University. Every patient performed liquid biopsy subject to informed consent for the genetic analysis. For the radiomic analysis, image processing CT volumes were manually delineated using ITK-snap 3.8.0. Radiomics features (first order, GLCM, GLRLM, GLSZM, GLDM, NGTDM) were computed using Pyradiomics in Python 3.7 environment. For the statistical analysis, association between radiomic features and gene mutations were assessed using feature importance based on ROC analysis; moreover, a machine learning approach based on SVM was used to evaluate the ability of radiomic features to predict gene mutations. Computations have been performed in the R environment using the CARET package.
**Results or Findings:** From the genetic analysis it turns out that the accuracy, i.e. the number of correct predictions and the total number of patients, obtainable using the selected group of features is of the order of 0.67. Some correlations between gene and features were found to be the case: ROS.miss.6.43.Arg167Gln with a feature group that included first-order glcm and glszm, ROS-miss.42.43.Asp2213 Asn with a broader set of features and ALK.miss.29.29.Asp1529 Glu with the same previous group but less intense.
**Conclusion:** Radiomics could better determine the accuracy of malignancy of pulmonary nodules, which have been detected by CT scan in order to treat curatively, select patients with early-stage lung cancer who are appropriate for post-surgical treatment, and determine patients with stage III NSCLC who can tolerate immunotherapy as consolidation therapy after concurrent treatment with chemotherapy-radiation therapy.
**Limitations:** These results are preliminary and require a greater number of observations.
**Ethics committee approval:** No information provided by the submitter.
**Funding for this study:** No funding was received for this study.
**Author Disclosures:**
Maria Paola Belfiore: Nothing to disclose
Francesca Grassi: Nothing to disclose
Roberta Grassi: Nothing to disclose
Salvatore Cappabianca: Nothing to disclose
Mario Sansone: Nothing to disclose
Giovanni Ciani: Nothing to disclose
Vittorio Patanè: Nothing to disclose
Riccardo Monti: Nothing to disclose

### RPS 1304-10

**A decade of image-guided pleural biopsies: a multicentre retrospective study examining diagnostic yield and complications**

*L. Peng*, S. Tsim, K. Blyth, G. Cowell; Glasgow/UK

**Purpose:** Pleural disease is common, with diagnostic options including pleural effusion aspiration, local anaesthetic thoracoscopy (LAT) and image guided biopsy. Despite diagnostic and therapeutic applications, LAT availability varies and is unfeasible with a complex pleural space. Ultrasound (US-) and Computed Tomography (CT-) guided biopsy remain commonly performed, yet post-procedural data is relatively sparse. A high prevalence of malignant pleural mesothelioma (MPM) in the West of Scotland offers an excellent opportunity to assess complication rate and diagnostic yield associated with image guided biopsy relative to other investigation strategies, to shape the consent process, develop care models and aid decision making around timely MPM diagnosis.
**Methods or Backround:** US- and CT-guided pleural biopsy procedures performed in six hospitals between 01.01.13 and 31.03.23 were identified by searching study codes and a key word search. Patient demographics, biopsy pathology, final diagnosis (either by repeat biopsy or consensus diagnosis via regional/national multidisciplinary team meeting) and procedural complications were recorded.
**Results or Findings:** 194 CT-guided pleural biopsies were performed using a coaxial technique. Seven (3.6%) had a peri-procedural pneumothorax on CT, with two (1.0%) evident on post-procedural chest radiograph. None required intervention. Two cases (1.0%) had haemoptysis. For diagnosis of pleural malignancy, sensitivity of CT-guided biopsy was 93.9% (95% CI 88.9-97.0%), specificity 100% (89.1-100%), positive predictive value (PPV) 100% (97.6-100%) and negative predictive value (NPV) 76.2 (63.7-85.4%). A total of 79 US-guided pleural biopsies were performed, without post-procedural pneumothorax. For diagnosis of pleural malignancy, sensitivity of US-guided biopsy was 93.1% (95% CI 84.5-97.7%), specificity 100% (59.0-100%), PPV 100% (94.6-100%) and NPV 58.3% (37.5-76.5%).
**Conclusion:** CT- and US-guided pleural biopsy is safe, offering excellent sensitivity and PPV in the diagnosis of pleural malignancy if LAT is not available or feasible.
**Limitations:** No limitations were identified.
**Ethics committee approval:** This study received Caldicott approval granted by the NHS board.
**Funding for this study:** No funding was received for this study.
**Author Disclosures:**
Selina Tsim: Nothing to disclose
Gordon Cowell: Nothing to disclose
Liam Peng: Nothing to disclose
Kevin Blyth: Nothing to disclose

### RPS 1304-11

**Structured reporting quality of chest CT for lung cancer staging: a double cohort study involving radiology residents**

*V. Peruzzi*, L. Cereser, C. Vecchia, F. Cortiula, M. Bortolot, G. Como, R. Girometti, C. Zuiani; Udine/IT

**Purpose:** To compare radiology residents' (RRs) report quality using the structured radiological model (SRM) from the Royal College of Radiologists with narrative reporting (NR) for chest CT staging of lung cancer. To assess reporting times for NR and SRM.
**Methods or Backround:** A study coordinator preliminarily selected 30 non-small cell lung cancer patients who underwent a baseline staging contrast-enhanced chest CT examination between 2014 and 2022 at our University Hospital. After attending a dedicated training lesson, four third-year RRs (RR1-4) independently reported all the CT examinations in two 2-month-apart separate reading sessions. In the first reading, all the RRs used the NR, while

Friday in the second reading, RR1-2 used the NR, and RR3-4 used SRM. Two chest-devoted radiologists, in consensus, rated the completeness and accuracy of all the NRs and SRMs. Two thoracic oncologists, in consensus, expressed the perceived clarity for the reports from the second reading session. All the quality indicators were expressed on a 100-point scale. The Wilcoxon test was used for statistical analysis.
**Results or Findings:** Comparing reading sessions, RR3-4 report completeness was significantly higher when using SRM vs. NR (90.7 vs. 74.0, $p<0.001$), while RR3-4 accuracy and RR1-2 completeness and accuracy values were not significantly different. In the second reading, report completeness, accuracy, and clarity of RR3-4 were significantly higher than RR1-2, with median values of 90.7 vs. 72.8 ($p<0.001$), 63.1 vs. 58.7 ($p=0.04$), and 87.3 vs. 68.3 ($p<0.001$), respectively. Median RR3-4 reporting time was significantly longer than RR1-2 (13.5 min vs. 10.6 min, $p<0.001$).
**Conclusion:** The completeness, accuracy, and clarity of SRM were superior to NR at the price of a longer reporting time.
**Limitations:** Retrospective, monocentric study, with a limited number of patients.
**Ethics committee approval:** Not applicable
**Funding for this study:** None
**Author Disclosures:**
Francesco Cortiula: Nothing to disclose
Martina Bortolot: Nothing to disclose
Chiara Zuiani: Nothing to disclose
Rossano Girometti: Nothing to disclose
Lorenzo Cereser: Nothing to disclose
Cecilia Vecchia: Nothing to disclose
Giuseppe Como: Nothing to disclose
Valeria Peruzzi: Nothing to disclose

**12:30-13:30 Research Stage 1**

### Research Presentation Session: Neuro

### RPS 1411

### Neuroimaging in white matter disease

**Moderator**
R. Zawadzki; Bialystok/PL

### RPS 1411-2

**A novel 7T MRI approach to detect posterior fossa demyelinating lesions in patients with multiple sclerosis**

E. Middlebrooks, V. Patel, X. Zhou, S. Straub, *L. Okromelidze*, V. Gupta, S. J. Singh Sandhu, S. Tao; Jacksonville, FL/US
*(lela.okromelidze@gmail.com)*

**Purpose:** Detection of infratentorial demyelinating lesions in multiple sclerosis (MS) presents a challenge in MRI. The goal of this study is to assess the efficacy of a novel MRI approach, lesion-attenuated MPRAGE acquisition (LAMA) in detection of demyelinating lesion within the posterior fossa and upper cervical spine in patients with MS on 7T MRI.
**Methods or Backround:** A retrospective cross-sectional study of 42 patients with MS who had 7T brain imaging with LAMA, 3D DIR and 2D T2-weighted turbo spin echo sequences was performed. Three neuroradiologists assessed lesion count in the brainstem, cerebellum, and upper cervical spinal cord using both DIR and T2-weighted images in one session, and LAMA in a separate one. Additionally, contrast-to-noise ratio (CNR) between LAMA and the conventional sequences was compared.
**Results or Findings:** LAMA identified more lesions than DIR+T2 (6.4 versus 3.0; $p<0.001$), exhibited better interrater agreement (ICC = 0.75 [95% CI = 0.41 – 0.88] versus 0.61 [95% CI = 0.35 – 0.78]), and higher contrast-to-noise ratio (3.7 ± 0.9) versus that of DIR (1.94 ± 0.7) and T2 (1.2 ± 0.7) (all $p < 0.001$). In cases with no lesions detected using DIR+T2, at least one lesion was identified in 83.3% with LAMA. Across all analysed brain regions, LAMA consistently detected more lesions than DIR+T2.
**Conclusion:** LAMA has demonstrated improved detection of infratentorial demyelinating lesions in patients with MS compared to traditional methods at 7T through high spatial resolution, robust lesion contrast, and resilience against prevalent 7T MRI artefacts such as B1+transmit heterogeneity. Integrating LAMA with standard MP2RAGE provides an additional tool for accurate characterisation of the extent of MS.
**Limitations:** AMA is limited to 7T. Further investigation is needed to assess its comparative advantage over other sequences at the reduced field strength.
**Ethics committee approval:** This retrospective study was approved by Mayo Clinic Institutional Review Board.
**Funding for this study:** No funding was received for this study.
**Author Disclosures:**
Vishal Patel: Nothing to disclose
Sina Straub: Nothing to disclose
Vivek Gupta: Nothing to disclose
Lela Okromelidze: Nothing to disclose
Sukhwinder Johnny Singh Sandhu: Nothing to disclose
Erik Middlebrooks: Nothing to disclose
Shengzhen Tao: Nothing to disclose
Xiangzhi Zhou: Nothing to disclose

### RPS 1411-3

**Comparative analysis of three commercially available artificial intelligence software programmes for brain volumetry and lesion segmentation in multiple sclerosis and dementia**

*A. Di Gioia*, F. Colarieti, P. Badini, C. Santobuono, A. Innocenzi, S. Martino, F. Bruno, A. Splendiani, E. Di Cesare; L'Aquila/IT
*(antonio.digioia.med@gmail.com)*

**Purpose:** The aim of this study was to compare the characteristics, results and experience by using three commercially available software programmes for segmentation and counting of multiple sclerosis lesions and measurement of dementia brain volume assessment.
**Methods or Backround:** Brain MRI with standard imaging protocol and including volumetric FLAIR and T1 sequences were performed in 28 patients (11 famales and 17 males) affected by multiple sclerosis (13 patients) and dementia (15 patients). MRI scans were segmented twice in each software package by two investigators. Intra-rater, inter-rater and between-software agreement was compared.
**Results or Findings:** The main differences were found in the segmentation of white matter lesions, in particular the recognition of hypointense lesions in T1 that were evidenced from each of the three software used. Furthermore differences were found in both total lesion volume and number of lesions, with a median difference mostly shown in the evaluation and recognition of periventricular lesions (-0.39ml; 95% CI from -1.745 to 1.0). Concerning volume calculation, the inter-software and interobserver analysis shows the main difference being in the evaluation of the frontal (-49.3ml; 95% CI from -60.9 to -37.8) and parietal region volume (-46.8; 95% CI from -51.2 to 42.4).
**Conclusion:** The use of artificial intelligence software is becoming an essential part of clinical evaluation in medicine, and in our specific case in the evaluation of multiple sclerosis lesions and measurement of dementia brain volume assessment. Despite technological progress, according to our clinical study, there doesn't yet seem to be a high level of agreement within and between the software in terms of intra-rater, inter-rater and intra-software programme differences. Therefore, users should be aware of the lack of interchangeability between these software programmes when they are applied in clinical practice.
**Limitations:** The low study sample and the fact this this was a retrospective analysis were identified as limitations.
**Ethics committee approval:** All subjects gave their informed consent for inclusion before they participated in the study. The study was conducted in accordance with the Declaration of Helsinki.
**Funding for this study:** No funding was received for this study.
**Author Disclosures:**
Antonio Di Gioia: Nothing to disclose
Alessandra Splendiani: Nothing to disclose
Chiara Santobuono: Nothing to disclose e
Pierfrancesco Badini: Nothing to disclose
Filippo Colarieti: Nothing to disclose
Antonio Innocenzi: Nothing to disclose
Ernesto Di Cesare: Nothing to disclose
Saverio Martino: Nothing to disclose
Federico Bruno: Nothing to disclose

### RPS 1411-4

**Effect of AI supported lesion detection in MS patients on reporting times**

*S. Peters*[1], G. Kellermann[2], J. Watkinson[1], M. Huhndorf[1], K. Stürner[1], O. Jansen[1], N. Larsen[1]; [1]Kiel/DE, [2]Hamburg/DE

**Purpose:** Evaluation of MR images of patients with multiple sclerosis (MS) is part of daily clinical routine. Consequently, a growing number of companies offer software for automated lesion detection. The aim of this study was to evaluate the effect of such software on the radiological reporting.
**Methods or Backround:** Four radiologists retrospectively evaluated MRI examinations of 50 MS patients and counted the lesions in FLAIR images separated by the locations periventricular, cortical/juxtacortical, infratentorial and unspecific. After six weeks they repeated the evaluation, this time additionally using the AI based software mdbrain. In both settings the required time for lesion detection was documented. Furthermore, the four radiologists evaluated follow-up MRIs of 50 MS patients concerning new and enlarging lesions in the same manner.

**Results or Findings:** To determine the lesion-load the average reporting time decreased from 286.85 seconds to 196.34 seconds (p>0.001). For the evaluation of the follow-up images the average reporting time dropped from 196.17 seconds to 120.87 seconds (p<0.001). The interrater reliabilities showed no remarkable change. The interclass correlation coefficient (ICC) was 0.83 for the determination of the lesion-load without software support and 0.84 with software support. For the follow-up evaluation, the ICC was 0.92 without software support and 0.82 with software support.
**Conclusion:** Increasing workload is a growing problem in daily clinical routine. Usage of AI based support for image-interpretation can be helpful in clinical routine and significantly lowers reporting times in a system with already limited resources. The interrater reliability was not remarkably affected be the usage of the software, probably because of the already high agreement without software support.
**Limitations:** With these results, a direct conclusion regarding the effect on the reporting quality cannot be made.
**Ethics committee approval:** The study was approved by the ethics committee of the medical faculty of the Christian-Albrechts-University (CAU) Kiel.
**Funding for this study:** No funding was received for this study.
**Author Disclosures:**
Klarissa Stürner: Nothing to disclose
Sönke Peters: Nothing to disclose
Joe Watkinson: Nothing to disclose
Olav Jansen: Nothing to disclose
Naomi Larsen: Nothing to disclose
Monika Huhndorf: Nothing to disclose
Gesa Kellermann: Nothing to disclose

### RPS 1411-5

**Brain white matter microstructural alterations in Behcet's patients correlate with cognitive impairment: a diffusion tensor imaging (DTI) study**
*O. A. Kargin*, S. Arslan, U. Uygunoglu, B. C. Poyraz, B. Korkmazer, E. Seyahi, O. Kizilkilic; Istanbul/TR
*(aykankargin@gmail.com)*

**Purpose:** Behcet's syndrome (BS) is a chronic, recurrent, multisystemic vasculitis of unknown aetiology. Neuroaxonal damage in its neurological involvement, known as Neuro-Behcet's syndrome (NBS), can lead to declines in neurocognitive functions, contributing to disease-related morbidity. Our study aims to evaluate the microstructural integrity of cerebral white matter tracts in patients with NBS and BS without neurological involvement via DTI, correlate DTI data with neurocognitive functions, and explore clinical-anatomical relationships to identify potential neuroimaging biomarkers.
**Methods or Backround:** The study comprised 34 NBS patients and 32 BS patients without neurological involvement, identified based on the Behcet's Syndrome International Study Group (ISG) and the International Consensus Recommendation (ICR) criteria. Additionally, 33 age- and educational status-matched healthy individuals were included as controls. All participants underwent DTI, as well as standardised neuropsychological tests assessing various cognitive domains, including attention, memory, verbal fluency, abstraction, executive control, visuospatial skills, and sensorimotor performance. DTI data were analysed using tract-based spatial statistics (TBSS) and automated probabilistic tractography to investigate intergroup differences. Subsequently, the correlations between DTI parameters of white matter tracts and neuropsychological test scores were examined.
**Results or Findings:** DTI revealed decreased fractional anisotropy and increased radial diffusivity, mean diffusivity, and axial diffusivity in both supratentorial and infratentorial white matter in NBS patients, indicating widespread loss of microstructural integrity. Moreover, this loss of integrity was also observed in BS patients without overt neurological involvement, albeit to a more limited extent. In NBS patients, specific white matter tracts, including superior thalamic radiation, cingulum, and fornix, were associated with poor cognitive performance across multiple domains.
**Conclusion:** Our results suggest that DTI findings can potentially serve as biomarkers to assess neurocognitive impairment associated with central nervous system involvement in BS.
**Limitations:** This is a cross-sectional study without longitudinal follow-up.
**Ethics committee approval:** The study was approved by Istanbul University-Cerrahpasa Clinical Research Ethics Committee (reference number 162).
**Funding for this study:** Funding was provided by the Scientific Research Projects Coordination Unit of Istanbul University-Cerrahpasa. Project number TTU-2022-36692.
**Author Disclosures:**
Emire Seyahi: Nothing to disclose
Serdar Arslan: Nothing to disclose
Ugur Uygunoglu: Nothing to disclose
Osman Kizilkilic: Nothing to disclose
Bora Korkmazer: Nothing to disclose
Burc Cagri Poyraz: Nothing to disclose
Osman Aykan Kargin: Nothing to disclose

### RPS 1411-6

**Usefulness of automatic fully convolutional neural network algorithm (TractSeg) in DTI determining cerebral white matter integrity in patients with primary Sjögren's syndrome**
*M. Sobański*[1], M. Gajowczyk[1], P. Rygiel[2], A. Korbecki[1], J. Korbecka[1], M. Sobańska[1], A. Sebastian[1], J. Bladowska[1]; [1]Wroclaw/PL, [2]Enschede/NL
*(michalsobanski11@wp.pl)*

**Purpose:** Primary Sjögren's syndrome (pSS) is an autoimmune disease in which central nervous system (CNS) involvement may occur. Incidence and pathogenesis of changes related to CNS in pSS is not well understood. Diffusion tensor imaging (DTI) can be used to demonstrate impaired integrity of cerebral white matter (CWM), especially by quantitative values such as fractional anisotropy (FA).
**Methods or Backround:** A study group containing 33 patients with primary Sjögren's syndrome and a control group containing 26 healthy patients were studied by performing DTI on a 3T MRI scanner. Data was reprocessed by automatic TractSeg algorithm, which is a fully convolutional neural network (FCNN), resulting in a segmentation of 72 white matter (WM) tracts per patient, as well as a value of FA for each tract.
**Results or Findings:** Reduction in FA in the study group relative to the control group was found in multiple CWM areas. Previously unreported involvement of the cerebellar WM tracts and optic radiations were found. There were also numerous correlations between reduced FA values in white matter tracts and rheumatological factors.
**Conclusion:** DTI examination is a sensitive advanced MRI technique for detecting impaired integrity of cerebral WM tracts in patients with pSS. The greatest reduction in FA in the study group was found in the cerebellar peduncles, which is also a novel observation. Other WM tracts also showed reduction in FA. Numerous associations between neuroradiological findings and rheumatological factors have been demonstrated. The utility of automated methods of collecting measurements in the DTI, such as TractSeg's FCNN, allows for a rapid and reproducible collection of qualitative and quantitative data.
**Limitations:** Identified limitations were the (1) small study group, and (2) lack of DTI study at the moment of diagnosis of pSS.
**Ethics committee approval:** The study received institutional review board approval and written informed consent was obtained from all participants. Opinion number of the Bioethics Committee at the Medical University of Wroclaw - KB - 578/2020.
**Funding for this study:** No funding was received for this study.
**Author Disclosures:**
Miłosz Gajowczyk: Nothing to disclose
Adrian Korbecki: Nothing to disclose
Justyna Korbecka: Nothing to disclose
Michał Sobański: Nothing to disclose
Martyna Sobańska: Nothing to disclose
Patryk Rygiel: Nothing to disclose
Agata Sebastian: Nothing to disclose
Joanna Bladowska: Nothing to disclose

### RPS 1411-7

**Quantitative assessment of lesion-cerebral cortex signal ratio in FLAIR sequence for discriminating white matter lesions from non-specific T2 hyperintensities in systemic lupus erythematosus**
R. Akdoğan[1], *A. Tezcan*[2], Z. Tüzün[1], S. A. Atalay[1], K. Kaşali[1], G. Polat[1], M. Alkan Melikoğlu[1]; [1]Erzurum/TR, [2]Malatya/TR

**Purpose:** In our study, we aimed to determine the diagnostic value of measuring the lesion-cortex signal ratio (LCSR) in the FLAIR sequence for distinguishing T2 hyperintense white matter lesions related to SLE from non-specific T2 hyperintensities, with the intention of providing guidance to clinicians in the diagnosis and treatment process of the disease.
**Methods or Backround:** A total of 30 SLE patients with white matter hyperintensities and without additional conditions were included in the study. A control group of 30 patients with non-specific hyperintensities in the white matter in the FLAIR sequence was selected. LCSR measurements were performed twice at different times by two radiologists. The data are presented as mean and standard deviation, and the Mann-Whitney U test was used to compare LCSR means between SLE patients and the control group. ROC analysis was conducted to assess whether LCSR measurement can be used to differentiate SLE white matter lesions from non-specific hyperintensities.
**Results or Findings:** The Cronbach's alpha value between the measurements of the two observers at two different time points was found to be 0.971 and 0.946, respectively, with a highly significant agreement value of 0.959 between them. The LCSR value for SLE patients was measured as 1.30±0.12, while it was significantly higher at 1.13±0.10 for the control group (p<0.001). In the ROC analysis conducted to determine whether LCSR measurement can be used to differentiate SLE white matter lesions from non-specific hyperintensities, the area under the curve was calculated as 0.872±0.043, and it was found to be statistically significant (p<0.001). Consequently, the LCSR

cut-off value was determined as 1.1939, with a sensitivity of 0.836 and a specificity of 0.833.
**Conclusion:** Measurement of LCSR in the FLAIR sequence allows for a high-precision differentiation between white matter lesions associated with SLE and non-specific T2 hyperintense lesions. Furthermore, the excellent interobserver agreement in LCSR measurements attests to its reproducibility.
**Limitations:** The retrospective design of this study was an identified limitation.
**Ethics committee approval:** This study received ethics committee approval.
**Funding for this study:** No funding was received for this study.
**Author Disclosures:**
Recai Akdoğan: Nothing to disclose
Gokhan Polat: Nothing to disclose
Kamber Kaşali: Nothing to disclose
Said Ali Atalay: Nothing to disclose
Alperen Tezcan: Nothing to disclose
Meltem Alkan Melikoğlu: Nothing to disclose
Zeynep Tüzün: Nothing to disclose

### RPS 1411-8

**Relationship between nutritional status and white matter integrity in the geriatric population: a retrospective DTI Study**
B. Atasoy Badur, *N. Atila*, P. Soysal, O. Ipar, A. Kaya, S. Balsak, A. Alkan; Istanbul/TR
*(nazatila@gmail.com)*

**Purpose:** Malnutrition is described as the clinical condition of decreased nutrient intake and/or inadequate nutrient absorption. It may result in adverse effects in geriatrics. The aim of this study is to investigate whether the nutritional status in the geriatric population is associated with the integrity of white matter tracts by using DTI.
**Methods or Backround:** This is a retrospective cross-sectional study with a total of 224 geriatric patients. Mini-nutritional assessment (MNA) was utilised to detect malnutrition risk. Routine brain MRI protocol and DTI were performed for all patients. 18 different white matter tracts in both cerebral hemispheres were evaluated by ROI-based approach. The MD, FA, AD and RD values were recorded.
**Results or Findings:** According to the MNA: 86 patients have normal nutritional status, 107 patients are with malnutrition risk and 31 patients are diagnosed with malnutrition. There is a significant difference between these three groups in terms of age and lab values of folate. There is no statistically significant difference between the groups in terms of gender, co-morbid disease and laboratory values of vitamin B12, vitamin D or thyroid function tests. Regarding the DTI values, the most affected white matter tracts are cingulum (CG), genu of corpus callosum, forceps minor, anterior limb of internal capsul, superior (SCP) and middle cerebellar peduncle. After adjusting for the folate and age on the effect of DTI parameters, CG and SCP are still affected.
**Conclusion:** The findings indicate that malnutrition in geriatric patients may be associated with deteriorated DTI values of white matter tracts, especially in cingulum and superior cerebellar peduncle, which might be considered as an indicator of impaired white matter integrity.
**Limitations:** The ROI-based approach is operator-dependent.
**Ethics committee approval:** This study was approved by the local ethics committee.
**Funding for this study:** No funding was received for this study.
**Author Disclosures:**
Ozlem Ipar: Nothing to disclose
Ahmet Kaya: Nothing to disclose
Bahar Atasoy Badur: Nothing to disclose
Pinar Soysal: Nothing to disclose
Serdar Balsak: Nothing to disclose
Naz Atila: Nothing to disclose
Alpay Alkan: Nothing to disclose

### RPS 1411-9

**Cerebral microstructural white matter changes measured by diffusion tensor imaging in asymptomatic carotid endarterectomy patients: a prospective study into predictors and correlation with vascular anatomy**
*C. Péter*, B. Szilveszter, M. Vecsey-Nagy, S. Borzsák, G. Gyebnár, Z. Mihály, Z. Czinege, P. Sótonyi, A. Varga; Budapest/HU

**Purpose:** Diffusion tensor imaging (DTI) metric changes were assessed in a prospective cohort of asymptomatic carotid endarterectomy (CEA) patients in correlation with patient characteristics, and vascular anatomy (4-axis; circle of Willis [CW] scores).
**Methods or Backround:** Prospectively enrolled patients (54, males 54%, age 69.7±7 years) had DTI pre-CEA (mean 2, range 0-31 days) and post-CEA (mean 2, range 0-5 days). DTI metrics (FA–fractional anisotropy, axial, mean, radial diffusivities – AD, MD, RD) were calculated for 16 white matter (WM) regions (8-8 regions of index and contralateral hemispheres) pre-CEA and post-CEA and compared using the repeated measure Wilcoxon signed rank test ($p<0.0125$, Bonferroni corrected). Preoperative supraaortic plus intracranial CTA was performed to create the 4-axis score (range 0-15,mthe lower the worse) and CW score (0–5, 0 if both the anterior plus ispilateral posterior semicircles towards the operated internal carotid artery are incomplete). Univariate and multivariate linear regression analyses were used to correlate main demographic, comorbidity data, vascular scores ($p<0.05$).
**Results or Findings:** Significantly decreased FA in 1/8 index WM region ($p=0.007$), singificantly increased AD in 7/8 index, 4/8 contralateral WM regions ($p<0.0001$-$0.0120$), significantly increased MD in 6/8 index, 4/8 contralateral WM regions ($p<0.0001$-$0.005$), significantly increased RD in 5/8 index, 3/8 contralateral WM regions ($p<0.0001$-$0.011$) was found. In 19/26 regions with significant DTI metric changes from pre-to-post-CEA, the 4-axis score was a significant predictor using univariate regression analysis ($p<0.001$-$0.038$), remaining significant with multivariate regression analysis in the index temporal, contralateral frontal, parietal WM regions ($p=0.006$-$0.012$) for MD, and in the index insular ($p= 0.042$) and temporal WM regions ($p=0.039$) for RD.
**Conclusion:** DTI metric changes suggesting microstructural deterioration were found from pre-CEA to post-CEA, more pronounced on the index side, with the weighted 4-axis score as a significant predictor.
**Limitations:** Identified limitations were (1) the small cohort and (2) the lack of correlation with surgical/anaesthesiology data.
**Ethics committee approval:** This study was approved by the institutional ethical committee with approval code: IV/667-1/2022/EKU.
**Funding for this study:** Funding was received from the National Research, Development and Innovation Office – NKFIH, K 129277.
**Author Disclosures:**
Milán Vecsey-Nagy: Nothing to disclose
Andrea Varga: Nothing to disclose
Zsófia Czinege: Nothing to disclose
Sarolta Borzsák: Nothing to disclose
Gyula Gyebnár: Nothing to disclose
Bálint Szilveszter: Nothing to disclose
Csongor Péter: Nothing to disclose
Péter Sótonyi: Nothing to disclose
Zsuzsanna Mihály: Nothing to disclose

**12:30-13:30 Research Stage 2**

### Research Presentation Session: Oncologic Imaging

### RPS 1416

### Latest CT technology in oncologic imaging

**Moderator**
D. Caruso; Rome/IT
*(dcaruso85@gmail.com)*
**Author Disclosures:**
Damiano Caruso: Speaker: Bayer, Bracco

### RPS 1416-2

**Rho/Z maps derived from dual-energy CT for differentiation of hypodense liver lesions in patients with colorectal carcinoma**
*M. Dimitrova*, A. Gökduman, V. Koch, L. D. Gruenewald, L. S. Alizadeh, S. Martin, T. J. Vogl, I. Yel, C. Booz; Frankfurt/DE

**Purpose:** This study aims to evaluate the diagnostic accuracy of dual-energy computed tomography (DECT)-based Rho/Z-maps for differentiation of hypodense liver lesions in patients with diagnosed colorectal carcinoma compared with conventional CT value measurements.
**Methods or Backround:** This retrospective study included 364 patients (mean age, 70 ± 13 years; 183 men and 181 women) suffering from colorectal carcinoma who had undergone third-generation dual-source DECT as part of tumour staging between January 2016 and January 2023. For this study, we measured Rho (electron density) and Z (effective atomic number) values as well as Hounsfield units (HU) in hypodense liver lesions. Values were compared, and diagnostic accuracy for differentiation was computed using receiver operating characteristic (ROC) curve analyses. MRI or biopsy served as a standard of reference for lesion characterisation.
**Results or Findings:** A total of 822 lesions (351 metastases and 471 cysts) in contrast-enhanced DECT images were evaluated. Mean Rho, Z and HU120kV values showed significant differences between both liver lesion types ($p<.0001$). The greatest difference between measured values for liver metastases and benign cysts was observed for Rho (MD, -35.31 ± 3.217, 92%; HU120kV MD, -21.46 ± 3.017, 60%). Rho and Z measurements showed a higher AUC value (Rho, 0.962; 95% CI, 0.936-0.989; Z, 0.918; 95% CI, 0.845-0.954) compared with HU120kV (0.802; 95% CI, 0.741-0.862) for lesion differentiation.

**Conclusion:** Rho and Z measurements derived from DECT allow for improved differentiation of liver metastases and cysts in patients with colorectal carcinoma compared with conventional CT value measurements.
**Limitations:** The single-centre retrospective study design was an identified limitation.
**Ethics committee approval:** This study was approved by the local IRB.
**Funding for this study:** No funding was received for this study.
**Author Disclosures:**
Simon Martin: Nothing to disclose
Christian Booz: Speaker: Siemens Healthineers
Leon D. Gruenewald: Nothing to disclose
Ibrahim Yel: Speaker: Siemens Healthineers
Mirela Dimitrova: Nothing to disclose
Vitali Koch: Nothing to disclose
Thomas J. Vogl: Nothing to disclose
Aynur Gökduman: Nothing to disclose
Leona Soraja Alizadeh: Nothing to disclose

### RPS 1416-3

**Photon-counting CT in depicting pancreatic ductal adenocarcinoma (PhDAC): a pilot study**

H. Blomstrand[1], W. C. Bartholomä[1], E. Tesselaar[1], B. Björnsson[1], T. Bjerner[1], M. Sandborg[1], N. Elander[2], *M. Woisetschlager*[1]; [1]Linköping/SE, [2]Liverpool/UK

**Purpose:** Pancreatic ductal adenocarcinoma (PDAC) is a challenging condition with low survival rates. Accurate imaging is vital for treatment decisions. Photon-counting CT (PCCT) is an emerging technology with potential benefits over standard CT. This pilot study assessed PCCT's usefulness in pancreatic and PDAC imaging compared to energy-integrating detector (EID) CT.
**Methods or Backround:** Eight PDAC patients received equivalent radiation doses of EID and PCCT scans, with five experienced evaluators rating image quality using a Likert scale. Quantitative measures including contrast-to-noise ratio (CNR), signal-to-noise ratio (SNR), noise levels, and tissue sharpness (line density profiles) were also recorded.
**Results or Findings:** PCCT images matched EID CT quality overall, with a slight preference for EID CT in arterial phase images for three parameters (p=0.015). Venous phase image quality showed no significant difference (p>0.05). Quantitatively, PCCT displayed a trend toward higher CNR and SNR, indicating potential advantages in tissue differentiation. Noise levels were lower in PCCT images, especially in pancreatic tissue (p=0.064). Line density profiles hinted at sharper tissue edges in PCCT, although not statistically significant.
**Conclusion:** In conclusion, this pilot study suggests that PCCT is a promising imaging tool for pancreatic cancer patients. Despite slight preferences for EID CT in certain criteria, PCCT's quantitative advantages and noise reduction trends indicate its potential for enhancing soft tissue discrimination and clinical outcomes, pending further advancements in PCCT technology. These findings suggest PCCT's promise in pancreatic imaging, offering similar image quality to EID CT. Quantitative assessments hint at potential benefits in tissue differentiation and noise reduction. While EID CT was preferred in some criteria, PCCT's advantages may improve with evolving technology.
**Limitations:** This is a pilot study with a low number of patients. Comparison was made only to one scanner (Somatom Force).
**Ethics committee approval:** This study was approved by the Swedish Ethical Review Authority.
**Funding for this study:** No funding was received for this study.
**Author Disclosures:**
Wolf Claus Bartholomä: Nothing to disclose
Erik Tesselaar: Nothing to disclose
Tomas Bjerner: Nothing to disclose
Michael Sandborg: Nothing to disclose
Nils Elander: Nothing to disclose
Mischa Woisetschlager: Nothing to disclose
Hakon Blomstrand: Nothing to disclose
Bergthor Björnsson: Nothing to disclose

### RPS 1416-4

**Correlation between iodine concentration in lung cancer on dual-energy CT and non-mucinous adenocarcinoma grading**

*H. Watanabe*, Y. Sato, K. Oikado, T. Terauchi; Tokyo/JP
*(pipitan4631@gmail.com)*

**Purpose:** The fifth edition of the WHO classification introduced a grading system for invasive non-mucinous adenocarcinoma. Lesions are categorised into Grade 1 (well-differentiated), Grade 2 (moderately differentiated), and Grade 3 (poorly differentiated) based on the percentage of histological subtypes with a high-risk pattern for poor prognosis. This study investigates the correlation between the iodine concentration (IC) of lung adenocarcinoma measured by dual-energy CT (DECT) and this WHO grading.
**Methods or Backround:** We examined preoperative contrast DECTs from 111 patients (46 men, 65 women; median age: 70 years; 37 Grade 1, 25 Grade 2, 49 Grade 3) diagnosed with primary lung adenocarcinoma between January and December 2022. Cases with a solid component diameter under 5 mm on CT or those with invasive mucinous adenocarcinoma were excluded. IC measurements were taken during the equilibrium phase, 3 minutes after contrast administration. The corrected IC was determined by dividing the IC in the solid component of the lesion by the IC in the descending aorta at the tracheal bifurcation level. An ordinal logistic regression model, accounting for corrected IC, smoking status, nodule type (solid or part-solid), gender, and age, was employed for the multiple regression analysis.
**Results or Findings:** The multivariable analysis identified that significant independent predictors for a higher grade of invasive non-mucinous adenocarcinoma were the corrected IC (OR, 2.74 [95% CI, 1.46–5.98]; p = .005) and the presence of a pure solid nodule (OR, 5.02 [95% CI, 2.21-11.8]; p<.001).
**Conclusion:** IC measurements using DECT may predict the grade of pulmonary invasive non-mucinous adenocarcinoma.
**Limitations:** This study's limitations include its single-centre focus and retrospective design.
**Ethics committee approval:** The institutional review board approved this study, and there was a waiver for the need for written informed consent.
**Funding for this study:** This study was financially supported by Bayer Pharma Japan.
**Author Disclosures:**
Hikaru Watanabe: Nothing to disclose
Takashi Terauchi: Nothing to disclose
Yoshinao Sato: Grant Recipient: Research/Grant Support: Bayer Japan Pharma
Katsunori Oikado: Nothing to disclose

### RPS 1416-5

**Deep learning-based iodine contrast-augmenting algorithm for low-contrast-dose liver CT to assess hypovascular hepatic metastasis**

*T. Lee*[1], J-H. Yoon[1], J. Park[2], J. Lee[1], J. W. Choi[1], C. Ahn[1], J-M. Lee[1]; [1]Seoul/KR, [2]Busan/KR
*(lee8720@gmail.com)*

**Purpose:** The study aimed to investigate the diagnostic value of low-contrast-dose liver CT using a deep learning-based iodine contrast-augmenting algorithm (DLICA) to assess hypovascular hepatic metastases.
**Methods or Backround:** This retrospective study included 128 patients who underwent contrast-enhanced DECT for hepatic metastasis surveillance between July 2019 and June 2022 using a 30% reduced iodine contrast dose in the portal phase. Three image types were reconstructed: 50-keV virtual monoenergetic images (50-keV VMI); linearly blended images simulating 120-kVp images (120-kVp); and post-processed 120-kVp images using DLICA (DLICA 120-kVp). Three reviewers evaluated lesion conspicuity, and contrast-to-noise ratios (CNRs) were measured from the regions of interest in the metastatic lesions and liver parenchyma. The detection performance for hepatic metastases was evaluated using a jackknife alternative free-response receiver operating characteristic method, with the consensus of two independent radiologists as the reference standard.
**Results or Findings:** DLICA 120-kVp demonstrated significantly higher CNR of lesions to liver (5.7±3.1 vs. 3.8±2.1 vs. 3.8±2.1) and superior lesion conspicuity (4.0[3.3–4.3] vs. 3.7[3.0–4.0] vs. 3.7[3.0–4.0]) compared with 50-keV VMI and 120-kVp (P<0.001 for all). Although there was no significant difference of merit in the figure for lesion detection among the three methods (P=0.105), DLICA 120-kVp had a significantly higher figure of merit for lesions with a diameter <20 mm than 50-keV VMI (0.677 vs. 0.648, P=0.007). DLICA 120-kVp also demonstrated higher sensitivity on a per-lesion basis than the 50-keV VMI (81.2% vs. 72.9%, P<0.001).
**Conclusion:** DLICA 120-kVp provided higher lesion conspicuity and similar diagnostic performance to detect hypovascular hepatic metastases compared with 50 keV VMI. DLICA 120-kVp can serve as an alternative to 50keV VMI for improved detection of liver metastases, particularly in the case of smaller lesions, circumventing the need for high-end DECT equipment.
**Limitations:** The retrospective study design and the small sample size were identified limitations.
**Ethics committee approval:** This retrospective study was approved by the Institutional Review Board of Seou National University Hospital and the requirement for written informed consent was waived.
**Funding for this study:** This study was supported by a research grant from ClariPi (No. 06-2022-4530).
**Author Disclosures:**
Taehee Lee: Nothing to disclose
Jihyuk Lee: Nothing to disclose
Jinyoung Park: Nothing to disclose
Jeong-Min Lee: Nothing to disclose
Jae Won Choi: Nothing to disclose
Chulkyun Ahn: Employee: Chulkyun Ahn is an employee of ClariPi. His role was confined to providing the DLICA image sets, and he did not participate in data analysis
Jeong-Hee Yoon: Nothing to disclose

**RPS 1416-6**

**The value of dual-layer spectral-detector CT in detecting neuroendocrine tumour liver metastases: comparison with polyenergetic CT and Gd-EOB-DTPA-enhanced MR**

*T. Xie*, W. Liu, W. Deng, Y. Wang, W. Tang, Z. Zhou; Shanghai/CN
*(xts1095349998@163.com)*

**Purpose:** The study aimed to investigate the value of dual-layer spectral-detector CT (DLCT) in detecting neuroendocrine tumour liver metastases (NETLM) and to compare the diagnostic performance of polyenergetic CT images (PEI), DLCT, and Gd-EOB-DTPA-enhanced MR.
**Methods or Backround:** Seventy-two patients with suspected NETLM who underwent both DLCT and Gd-EOB-DTPA-enhanced MR within three weeks were retrospectively enrolled, and they were divided into dataset 1 and dataset 2. In dataset 1, virtual monoenergetic images (VMI) at 40-140 keV (VMI40-140, 10-keV interval) were reconstructed. Signal-to-noise ratio (SNR) and contrast-to-noise ratio (CNR) of NETLM were measured and compared between PEI and VMIs. In dataset 2, two readers independently evaluated the diagnostic performance of PEI (Set 1), VMI + PEI (Set 2), and Gd-EOB-DTPA-enhanced MR (Set 3) for detecting NETLM. Subgroup analysis stratified by lesion size was performed.
**Results or Findings:** In dataset 1, VMI40 exhibited highest SNR and CNR across all energy levels, which was significantly higher than that of PEI ($P<0.01$). In dataset 2, a total of 477 lesions were identified (396 metastases, 81 benign lesions). Adding VMI40 into PEI resulted in a significantly improved per-lesion AUC and sensitivity (AUC, Set 1: 0.85; Set 2: 0.90, $P < 0.01$; Sensitivity, Set 1: 0.76; Set 2: 0.86, $P < 0.01$). Set 3 achieved the highest per-lesion AUC of 0.97 and per-lesion sensitivity of 0.95. Subgroup analysis demonstrated that the difference in diagnostic performance was concentrated on lesions <10 mm.
**Conclusion:** Gd-EOB-DTPA-enhanced MR yielded the highest diagnostic accuracy for NETLM detection. DLCT should be an alternative choice, given the superior image quality and incremental diagnostic value of VMI40.
**Limitations:** The retrospective study design introduces the potential risk of selection bias.
**Ethics committee approval:** This retrospective study was approved by the Institutional Review Board of the Fudan University Shanghai Cancer Center (1612167–18). Informed consent was waived owing to the retrospective nature of the study.
**Funding for this study:** Funding was received from the Hong Kong, Macao, and Taiwan Cooperation Project of Scientific and Innovative Plan of Shanghai (22490760800).
**Author Disclosures:**
Yu Wang: Employee: Employee at Philips Clinical Medical Research Division, Shanghai
Wei Tang: Nothing to disclose
Tiansong Xie: Nothing to disclose
Wei Liu: Nothing to disclose
Zhengrong Zhou: Nothing to disclose
Weiwei Deng: Employee: Employee at Philips Clinical Medical Research Division, Shanghai

**RPS 1416-7**

**Photon-counting computed tomography derived spectral reconstructions: a promising approach to diminish beam-hardening artefacts in thoracic imaging**

*F. Haag*[1], N. Große Hokamp[2], D. P. Overhoff[1], G. Dasegowda[3], A. Hertel[1], D. Nörenberg[1], S. O. Schönberg[1], M. K. Kalra[3], M. Frölich[1]; [1]Mannheim/DE, [2]Cologne/DE, [3]Boston, MA/US
*(florian.haag@umm.de)*

**Purpose:** The use of port implants in cancer patients during follow-up CT scans can result in beam hardening artefacts, which can significantly impact the quality and accuracy of the acquired images. The aim of this study is to improve image quality and accuracy in thoracic imaging using photon-counting computed tomography (PCCT) derived spectral reconstructions.
**Methods or Backround:** This retrospective single-centre study enrolled 49 patients with port systems undergoing CT for staging of oncologic disease. 8 ROIs for 19 spectral reconstructions (polyenergetic imaging, monoenergetic reconstructions from 40 to 190 keV [in steps of 10] as well as iodine maps and virtual non contrast [VNC]) were measured, resulting in a total of 7,448 mean and SD Hounsfield unit measurements of port-chamber associated hypo- and hyperdense artefacts, bilateral muscles and vessels. Additionally, a subjective analysis of the degree of artefacts was performed in consensus reading by two radiologists with more than 25 years and more than 5 years of experience in thoracic imaging.
**Results or Findings:** In quantitative analysis, a significant association of keV and iodine contrast as well as artefact intensity was noted ($p<0.001$). In qualitative assessment, utilisation of 120 keV monoenergetic reconstructions could reduce severe and pronounced artefacts completely, when compared to lower keV reconstructions ($p<0.001$). For imaging findings, no significant difference between monoenergetic reconstructions could be noted (all $p>0.05$).
**Conclusion:** PCCT-derived, monoenergetic CT reconstructions are capable of reducing beam-hardening artefacts in chest imaging. They may prove helpful to improve diagnostic confidence in these cases.
**Limitations:** The present study deals mainly with the potential of spectral reconstructions for artefact suppression. Other algorithms for artefact reduction were not investigated and could be the subject of further research.
**Ethics committee approval:** No information provided by the submitter.
**Funding for this study:** This research was funded by the Photon-counting Consortium Baden-Württemberg (PC3), BMBF-Research Campus M2OLIE and Hector Foundation, as well as a travel grant from ICON (DFG).
**Author Disclosures:**
Nils Große Hokamp: Speaker: Philips Healthcare
Florian Haag: Nothing to disclose
Stefan Oswald Schönberg: Nothing to disclose
Alexander Hertel: Nothing to disclose
Mannudeep K. Kalra: Grant Recipient: Siemens Healthineers
Giridhar Dasegowda: Nothing to disclose
Matthias Frölich: Nothing to disclose
Daniel Pasqual Overhoff: Nothing to disclose
Dominik Nörenberg: Nothing to disclose

**12:30-13:30** **Research Stage 3**

### Research Presentation Session: Vascular

### RPS 1415

### Exploring imaging and interventional treatment of atherosclerotic lesions: fresh perspectives

**Moderator**
I. Bargellini; Pisa/IT
*(irenebargellini@hotmail.com)*

**RPS 1415-2**

**Semi-automatic measurement of carotid intima-media thickness in atherosclerosis**

L. Zhang, *J. Xu*, M. Chen; Shanghai/CN
*(xujiatong17@163.com)*

**Purpose:** This study aims to demonstrate if semi-automatic measurement can improve the accuracy of carotid intima-media thickness (CIMT) .
**Methods or Backround:** This study analysed the results of carotid ultrasound examinations of 1637 patients. During the complete cardiac cycle, three sonographers used semi-automatic measurement of the systolic and diastolic and manual measurement to measure CIMT. The relevant data of the patients was also collected, including age, gender, diabetes status, and other cardiovascular risk factors.
**Results or Findings:** For semi-automatic measurement of CIMT, the intraclass correlation coefficient (ICC) of the three-operator agreement analysis is 0.99 (95% confidence interval: 0.984-0.994. There is a statistical difference between the semi-automatic and manual measurement. There is no statistical difference between the left CIMT and the right CIMT measured by semi-automatic and manual measurement. Compared with the cerebrovascular event group, the bilateral semi-automatic CIMT values between the normal group and the cerebrovascular event group were statistically different.
**Conclusion:** Using the semi-automatic measurement of CIMT, the consistency between operators is better, and the repeatability is higher. It can replace the manual measurement of CIMT, provide a more accurate and stable inspection method, and better evaluate the degree of atherosclerosis.
**Limitations:** The study also has certain limitations. In addition to being subject to the inherent limitations of retrospective research, due to technical problems, it needs to use specific machine measurements. Since the samples of this study come from the routine examination of the hospital, a larger community study is needed at a later stage, to further prove the conclusion of this study.
**Ethics committee approval:** Ethics approval was obtained from the Ethics Committee of Shanghai Tongren Hospital.
**Funding for this study:** This research received no specific grant from any funding agency in the public, commercial or not-for-profit sectors.
**Author Disclosures:**
Jiatong Xu: Nothing to disclose
Man Chen: Nothing to disclose
Lixia Zhang: Nothing to disclose

### RPS 1415-3

**Accuracy of an artificial intelligence-based algorithm for fully automated detection of aortic wall calcifications in chest CT**

*C. Schuppert*[1], R. Saffar[1], J. Sperl[2], J. Oravcová[3], M. Kreibich[1], J. Weiß[1], F. Bamberg[1], M. Czerny[1], C. L. Schlett[1]; [1]Freiburg im Breisgau/DE, [2]Erlangen/DE, [3]Bratislava/SK

**Purpose:** This study aimed to assess the accuracy of an artificial intelligence-based algorithm for fully automated detection of aortic wall calcifications in chest CT with a special focus on the surgically relevant clamping zone in the upper ascending aorta.
**Methods or Backround:** We retrospectively included 100 chest CT scans from 92 patients who were examined on a third-generation dual-source scanner. Subsamples comprised 47 scans with an aortic angiography using iodinated intravenous contrast media and ECG-gating and 53 unenhanced scans. A previously validated deep learning algorithm performed aortic landmark detection and aorta segmentation. The segmentation mask was divided into eight anatomic segments and aortic wall calcifications were detected using a mean-based HU thresholding, yielding a binary score for their presence. Algorithm parameters (calcium cluster size threshold, aortic mask dilatation) were varied to determine optimal performance. A visual rating served as a reference. Standard estimates of diagnostic accuracy and interrater agreement using Cohen's kappa were calculated.
**Results or Findings:** Aortic wall calcifications were observed in 74% of the examinations with a prevalence of 27% to 70% by aorta segment. Using different parameter combinations, the algorithm provided results for 97% to 100% of the examinations and 95% to 99% of the combined aortic segments. The best-performing parameter combination for the presence of calcifications in the aortic clamping zone yielded a sensitivity of 93% and a specificity of 82%, with an area under the receiver operating characteristic curve of 0.874. Using these parameters, the interrater agreement ranged from κ 0.66 to 0.92 per segment.
**Conclusion:** Fully automated detection of aortic wall calcifications in chest CT performs with good accuracy. This includes the critical preoperative assessment of the aortic clamping zone.
**Limitations:** Intravenous contrast media and ECG-gating were not individually assessed as factors influencing algorithm performance, which is a limitation.
**Ethics committee approval:** This retrospective single-centre study was approved by the local institutional review board.
**Funding for this study:** No funding was received for this study.
**Author Disclosures:**
Martin Czerny: Nothing to disclose
Christopher L. Schlett: Speaker: Siemens Healthineers; Speaker: Bayer Healthcare
Jakob Weiß: Research/Grant Support: Bayer Healthcare; Research/Grant Support: Siemens Healthineers
Jana Oravcová: Employee: Siemens Healthineers
Ruben Saffar: Nothing to disclose
Fabian Bamberg: Consultant: Bayer Healthcare; Speaker: Bayer Healthcare; Research/Grant Support: Bayer Healthcare; Speaker: Siemens Healthineers; Research/Grant Support: Siemens Healthineers
Jonathan Sperl: Employee: Siemens Healthineers
Christopher Schuppert: Nothing to disclose
Maximilian Kreibich: Nothing to disclose

### RPS 1415-4

**Quantitative parameters from dual-layer detector spectral CT (DLS-CT) in distinguishing non-calcified plaques and chronic total occlusion (CTO) components in lower limb arteries**

*L. Zhou*, N. Ding, Y. Cui, Y. Han, J. Yang, X. Zhang, P. Cao, X. Huang; Xi'an/CN
*(zhouli222729@126.com)*

**Purpose:** This study aimed to explore the application value of quantitative parameters from dual-layer detector spectral CT (DLS-CT) in distinguishing non-calcified plaques and chronic total occlusion (CTO) components in lower limb arteries
**Methods or Backround:** Peripheral arterial disease (PAD) patients with DLS-CT examinations were prospectively enrolled from October 2022 to February 2023. Non-calcified plaques with a stenosis degree > 50% and CTO lesions in the above-knee arteries were analysed. Quantitative parameters of conventional images, 40keV, 70keV, virtual non-contrast, iodine density, Z-effective (Zeff), electron density (ED), and slope of the energy spectrum curve (λHU, λHU = (CT40keV -CT70 keV)/-30) of the non-calcified plaques and CTO lesions were evaluated for statistical analysis.
**Results or Findings:** Ninety-three lesions in 24 patients were evaluated, consisting of 72 non-calcified plaques and 21 CTO lesions. Analysis of the results indicated that CT40keV, iodine density and Z-effective of CTO tended to decrease and λHU tended to increase compared with non-calcified plaques, with significant difference of all (P＜0.05). The area under the curve of ROC (AUC) for CT40keV, iodine density, Z-effective, and λHU in distinguishing non-calcified plaques from CTO were 0.71 (95% CI 0.60-0.79), 0.74 (95% CI 0.52-0.725), 0.70 (95% CI 0.59-0.79), and 0.63 (95% CI 0.53-0.73), respectively. The AUC for the combination of these parameters was 0.87 (95% CI 0.727-0.929) for distinguishing non-calcified plaques from CTO.
**Conclusion:** DLS-CT quantitative parameters have the potential to identify the composition characteristics of non-calcified plaques and CTO, providing new imaging indicators for understanding the pathological changes and progression mechanism of PAD.
**Limitations:** No limitations were identified.
**Ethics committee approval:** The study was approved by the Ethics Committee of the First Affiliated Hospital (ethics approval number: XJTUIAF2021LSY-223).
**Funding for this study:** No funding was received for this study.
**Author Disclosures:**
Xin Huang: Nothing to disclose
Pan Cao: Nothing to disclose
Yaqing Han: Nothing to disclose
Xiaoyue Zhang: Nothing to disclose
Yuling Cui: Nothing to disclose
Li Zhou: Nothing to disclose
Ningning Ding: Nothing to disclose
Jian Yang: Nothing to disclose

### RPS 1415-5

**Voxel-based calcified plaque analysis: a comparison between a novel silicon-based photon-counting CT prototype, a conventional energy-integrating detector CT and µCT**

*E. Verelst*[1], G. Van Gompel[1], B. Keelson[1], J. De Mey[1], D. Crotty[2], P. D. Deak[3], N. Buls[1]; [1]Brussels/BE, [2]Cork/IE, [3]Ittigen/CH

**Purpose:** The study aimed to investigate the accuracy of a second-generation silicon-based photon-counting CT (Si-PCCT) prototype in a voxel-wise evaluation of calcified plaques, compared to conventional energy-integrating detector CT (EIDCT) with high-resolution micro-CT as a reference.
**Methods or Backround:** Three human-resected arteries were embedded in a 2% agar-water phantom (d=20 cm). Helical scans were acquired using a second-generation Si-PCCT prototype (GE Healthcare, Milwaukee, USA) and a conventional EIDCT system (GE Revolution, Milwaukee, USA) at similar scan parameters (120 kV, 40 mm collimation, 0.9 pitch, 1s rotation and 17 mGy CTDIvol). Images were reconstructed using a bone kernel, 1024 matrix, 150 mm field-of-view and slice thickness of 0.42 mm (Si-PCCT) and 0.63 mm (EIDCT). Micro-CT images were acquired with a benchtop system (X-cube, Molecubes) using a spiral high-resolution acquisition protocol at 0.075 mm xy-pixel size and 0.2 mm slice thickness. Plaques (n=7) were analysed by a voxel-wise computation of plaque volume (mm3), plaque burden (%) and calcified plaque volume (%), i.e. voxels with CT-values >1000 HU. Micro-CT images were used as a reference. The differences between the two systems were tested with a paired sample t-test.
**Results or Findings:** Overall, Si-PCCT allowed for more accurate volume measurements, as the mean error towards the reference volume (12.2 mm3) was significantly reduced when compared to EIDCT (27 mm3), p=0.03. The mean error towards the reference plaque burden was also found to be significantly lower for Si-PCCT (6.6%) when compared to EIDCT (11.4%), p<0.001. Si-PCCT also allowed for a significant increase in detection of heavily calcified regions (60.5%), when compared to EIDCT (38.6%), p=0.01.
**Conclusion:** This study demonstrates an improved spatial resolution of a second-generation Si-PCCT prototype, allowing for an improved voxel-wise analysis on plaque volume, burden and composition, when compared to conventional EIDCT.
**Limitations:** The study was an ex vivo phantom study.
**Ethics committee approval:** Ethical approval was obtained for the use of three human arteries (carotid, femoral and iliac).
**Funding for this study:** This study was funded by the Radiology Department of the University Hospital of Brussels (UZ Brussel).
**Author Disclosures:**
Johan De Mey: Nothing to disclose
Emma Verelst: Nothing to disclose
Gert Van Gompel: Nothing to disclose
Paul D. Deak: Employee: GE Healthcare
Nico Buls: Nothing to disclose
Benyameen Keelson: Nothing to disclose
Dominic Crotty: Employee: GE Healthcare

### RPS 1415-6

**Role of dual-energy computed tomography (DECT) in detection of carotid artery monosodium urate deposition in patients with gout**

*M. D. Sarfraz*, L. Treanor, S. Masood, N. Murray, A. Sheikh, S. Nicolaou; Vancouver, BC/CA
*(danimughals1@gmail.com)*

**Purpose:** The study aimed to find out if a dual-energy CT can detect monosodium urate deposition in carotid arteries and whether the presence of monosodium urate crystals has any effect on atherosclerotic disease in terms of plaque volume.
**Methods or Backround:** This is a retrospective study. All patients who underwent any dual-energy neck imaging (like carotid angiogram, neck soft tissues or cervical spine) from January 2015 to December 2022 were included. All patients' charts were rigorously reviewed to find gout patients and avoid confounding variables. The same number of healthy controls (without a clinical history of gout or other rheumatic diseases) were included during the same study period. These healthy controls were then age (within 5 years), sex and confounders-matched to gout patients. DECT datasets were post-processed using gout application and a volumetric analysis of atherosclerotic plaques was performed using a calcium scoring application in matched gout patients with carotid monosodium deposition, gout patients without carotid monosodium deposition and control/non-gout patients.
**Results or Findings:** Out of a total 2157 patients who underwent dual-energy neck imaging during the study period, 85 were confirmed gout cases. From the 85 gout cases, two were excluded due to presence of streak artefacts from dentures, hence n=83. Out of the 83, monosodium urate deposition was detected in the carotid arteries of 10 patients (12%). None of the matched control patients demonstrated monosodium crystal deposition. Volumetric analysis of atherosclerotic plaques demonstrated larger plaque volumes in gout patients with monosodium urate deposition (n=10) than matched gout patients without monosodium urate deposition (n=10) and matched control/non-gout patients (n=10) (p value of 0.03).
**Conclusion:** Carotid arteries are not a common site for monosodium urate deposition, but if present can lead to increased atherosclerosis in the involved vessel, resulting in increased incidence of TIA/strokes.
**Limitations:** No limitations were identified.
**Ethics committee approval:** Ethics approval was obtained before starting the project.
**Funding for this study:** No funding was received for this study.
**Author Disclosures:**
Lee Treanor: Nothing to disclose
Savvas Nicolaou: Nothing to disclose
Nicolas Murray: Nothing to disclose
Salman Masood: Nothing to disclose
Muhammad Danish Sarfraz: Nothing to disclose
Adnan Sheikh: Nothing to disclose

### RPS 1415-7

**Correlation between carotid NASCET-stenosis and plaque quality as characterised by the new Plaque-RADS score**

*G. L. Chabert*[1], R. Cau[1], A. Schindler[2], G. Grassi[1], M. Porcu[1], T. Saam[3], L. Saba[1]; [1]Cagliari/IT, [2]Munich/DE, [3]Rosenheim/DE

**Purpose:** The study aimed to investigate the correlation between the degree of carotid plaque stenosis, as measured by the NASCET method, and plaque composition as categorised by the new Plaque-RADS grading system.
**Methods or Backround:** One hundred and twenty subsequent patients (61 males, 59 females; mean age 67.9±18.61 SD) who were admitted for suspected cerebral ischaemic disease (CVD) and underwent CT-angiography of the carotid arteries were included in the study. Both carotid arteries were evaluated for the presence and quality of atherosclerotic plaque via the recently introduced Plaque-RADS score, and the degree of carotid stenosis was quantified according to NASCET.
**Results or Findings:** In carotid vessels of the right side the average degree of stenosis increased with increasing lesion severity from 0±SD0% for Plaque-RADS 1 (n=41), 8.6±9.8SD% for Plaque-RADS 2 (n=36), 27.0±10.4SD% for Plaque-RADS 3 (n=33), to 46.4±23.8SD% for Plaque-RADS 4 (n=10). The same increase was present for the left side from 0±SD0% for Plaque-RADS 1 (n=41), 9.6±10.3SD% for Plaque-RADS 2 (n=25), 25.7±13.2SD% for Plaque-RADS 3 (n=45), to 62.4±30.2SD% for Plaque-RADS 4 (n=9). A t-test was conducted with a significant difference result of <0.005. Pearson correlation was also used (r=.807 for right stenosis with right plaque-RADS values; r=.763 for the left side), when considering only plaque-RADS values of 3 and 4 (r=.505 for right side; r=.622 for left side).
**Conclusion:** Plaque-RADS scores positively correlate with degree of carotid stenosis, especially at lower lesion grades. On the contrary, our results also show that already in vessels with low grade stenosis, features of high risk plaques may be encountered and easily described in a standardised fashion using the novel Plaque-RADS score.
**Limitations:** The study's data collection was confined to a single institution and evaluated with a new, yet to be completely confirmed, scoring method.
**Ethics committee approval:** The study is retrospective.
**Funding for this study:** No funding was received for this study.
**Author Disclosures:**
Andreas Schindler: Nothing to disclose
Riccardo Cau: Nothing to disclose
Gian Luca Chabert: Nothing to disclose
Tobias Saam: Nothing to disclose
Michele Porcu: Nothing to disclose
Luca Saba: Nothing to disclose
Giovanni Grassi: Nothing to disclose

### RPS 1415-8

**Imaging of the aorta in dual-source photon-counting CT: impact of low energy virtual monoenergetic imaging on image quality, vascular contrast and diagnostic assessability**

*I. Yel*[1], C. Booz[1], V. Koch[1], L. D. Gruenewald[1], L. S. Alizadeh[1], S. Martin[1], T. J. Vogl[1], S. Waldeck[2], D. P. Overhoff[2]; [1]Frankfurt/DE, [2]Koblenz/DE
*(dr.ibrahimyel@gmail.com)*

**Purpose:** To evaluate the impact of low energy VMI+ reconstructions on quantitative and qualitative image quality, vascular contrast and diagnostic assessability of the aorta in patients undergoing photon-counting CTA.
**Methods or Backround:** A total of 125 patients (69 male) who had undergone dual-source photon-counting CTA scans of the aorta were retrospectively analysed in this study. Standard 120 kV CT images and low keV VMI+ series from 40 to 100 keV with an interval of 15 keV were reconstructed. Quantitative analyses included evaluation of vascular CT numbers, SNR and CNR. CT number measurements were performed in the ascending aorta, the aortic arch, the thoracic and infrarenal descending aorta. Qualitative analyses were performed by three board-certified radiologists independently using five-point scales to evaluate image quality, vascular contrast and diagnostic assessability.
**Results or Findings:** Mean attenuation, CNR and SNR values were highest in 40 keV VMI+ reconstructions (HU, 1312 ± 13; CNR, 32 ± 8; SNR, 34 ± 10) followed by 55-keV VMI+ reconstructions (HU, 731 ± 9; CNR, 24 ± 6; SNR, 27 ± 9); all three mean values at these keV levels were significantly higher compared with the remaining VMI+ series and standard 120 kV CT series (HU, 160 ± 8; CNR, 18 ± 5; SNR, 26 ± 6) (p<.0001). The qualitative analysis showed the highest rating scores for 55 keV VMI+ reconstructions followed by 40 keV and 70 keV VMI+ series with a significant difference compared to standard 120 kV CT images regarding image quality, vascular contrast and diagnostic assessability of the aorta (p<.0001).
**Conclusion:** Low keV VMI+ reconstructions at a level of 40-55 keV significantly improve image quality, vascular contrast and diagnostic assessability of the aorta compared with standard CT series in photon-counting CTA.
**Limitations:** The single-centre retrospective study design was an identified limitation.
**Ethics committee approval:** This study was approved by the local IRB.
**Funding for this study:** No funding was received for this study.
**Author Disclosures:**
Simon Martin: Nothing to disclose
Christian Booz: Speaker: Siemens Healthineers
Leon D. Gruenewald: Nothing to disclose
Ibrahim Yel: Speaker: Siemens Healthineers
Vitali Koch: Nothing to disclose
Thomas J. Vogl: Nothing to disclose
Daniel Pasqual Overhoff: Nothing to disclose
Stephan Waldeck: Nothing to disclose
Leona Soraja Alizadeh: Nothing to disclose

12:30-13:30 Research Stage 4

### Research Presentation Session: Chest

### RPS 1404

### From diagnosis to prognosis: chest imaging

**Moderator**
G. Aviram; Tel Aviv/IL
*(aviramgalit@hotmail.com)*

### RPS 1404-2

**AI vs senior radiologists in detecting thoracic abnormalities on chest radiographs compared to CT**

S. Bennani[1], J. Ventre[1], V. Marty[1], E. Lacave[1], D. Hayashi[2], A. Kompel[3], A. Gupta[3], A. Guermazi[4], *N-E. Regnard*[1]; [1]Paris/FR, [2]Stony Brook, NY/US, [3]Boston, MA/US, [4]West Roxbury, MA/US
*(noreddine.regnard@gleamer.ai)*

**Purpose:** The study aimed to assess the diagnostic performances of an artificial intelligence (AI) software in the detection of thoracic abnormalities on chest radiographs compared to senior radiologists.
**Methods or Backround:** We collected 319 chest radiographs of patients above 22 years old who underwent thoracic CT within 72 hours. A senior chest radiologist annotated the radiographs for four abnormality types (pleural abnormality, consolidation, mediastinal-hilar abnormality, nodule) using CT findings as the ground truth. Three senior radiologists independently analysed the dataset, knowing clinical indications without CT access. Discrepancies were resolved by consensus. The AI (ChestView, Gleamer), a deep learning algorithm that detects the four abnormalities, was compared against the radiologists and their consensual analysis for sensitivity and specificity.
**Results or Findings:** The dataset included 168 radiographs (age: 64±16 years, 91 women): 129 with at least one abnormality, 39 without any abnormality. For consolidation, the sensitivities were 72% for AI, 54%, 80%, and 66% for the individual readers, and 71% for consensus, with specificities of 92% for AI, 80%, 77%, 85% for the readers, and 92% consensus. The sensitivities for mediastinal-hilar abnormalities, were 54% (AI), 43%, 27%, 48% (readers), and 54% (consensus); specificities were 95%, 88%, 96%, 93%, and 94%, respectively. For nodules, the sensitivities were reported as 67% for AI, 57%, 55%, 55% for the readers, and 62% consensus, with specificities of 89%, 52%, 88%, 81%, and 86%, respectively. Lastly, for pleural abnormalities, the sensitivities were 84%, 89%, 87%, 73%, and 83%, and the specificities were 93% for AI, 88%, 92%, 95% for the readers, and 94% for consensus.
**Conclusion:** The AI consistently matched or exceeded senior radiologists in detecting thoracic abnormalities.
**Limitations:** The dataset had few radiographs with high pathology prevalence.
**Ethics committee approval:** The study was approved by WCG (number IRB00000533).
**Funding for this study:** Funding for this study was received from Gleamer.
**Author Disclosures:**
Andrew Kompel: Nothing to disclose
Vincent Marty: Employee: Gleamer
Daichi Hayashi: Nothing to disclose
Avneesh Gupta: Nothing to disclose
Souhail Bennani: Employee: Gleamer
Nor-Eddine Regnard: Employee: Gleamer
Jeanne Ventre: Employee: Gleamer
Elise Lacave: Employee: Gleamer
Ali Guermazi: Nothing to disclose

### RPS 1404-3

**Impact of the recent global iodinated contrast agent shortage on positivity rate for pulmonary embolism in CT pulmonary angiograms and chest CT with contrast at two major US health care systems**

*A. Wismueller*[1], J. P. Kanne[2], L. Stockmaster[1], E. Weinberg[1], D. Shrier[1], A. Vosoughi[1], A. Kasturi[1], N. Hadjiyski[1]; [1]Rochester, NY/US, [2]Madison, WI/US

**Purpose:** The objective of this study was to quantitatively track the impact of the recent global shortage of iodinated contrast agents on pulmonary embolism (PE) positivity rate in CT pulmonary angiograms (CTPA) using AI-based image analysis at two major US healthcare systems.
**Methods or Backround:** Using commercial AI-based image analysis (Aidoc Medical), we analysed daily volumes, PE and incidental PE (iPE) positivity rates for 7,633 computed tomography pulmonary angiogram (CTPA) and 11,164 contrast-enhanced chest CT (CT+C) exams before and during the contrast agent shortage (both comprising 01.04.2022 through 01.07.2022). For comparison, we analysed daily volumes and positivity rates for intracranial haemorrhage (ICH) on 30,803 non-contrast head CT exams during the same period. We compared two observational periods: a pre-shortage control period (10.04.2022 through 40.04.2022), and a contrast shortage period (20.05.2022 through 10.06.2022). Percentage change metrics of case volumes and positivity rates for PE, iPE and ICH were calculated.
**Results or Findings:** Case volumes of CTPA exams dropped from baseline during the shortage period by 38.9% while PE positivity rates significantly increased by 31.1% (p< 10^-4, Welch's unequal variances t-test). Similarly, case volume of CT+C exams dropped during the contrast agent shortage period by 49.9% while iPE positivity rates significantly increased by 20.2%, (p< 10^-4). For comparison, non-contrast head CT volumes dropped by only 6.5%, and ICH positivity rates increased by only 15.9%, with no significant difference (p> 0.05).
**Conclusion:** Our results suggest a significant increase of PE/iPE positivity rates in significantly decreased CTPA/CT+C total exam volumes during the observed global contrast agent shortage period, while non-contrast head CT exam volumes and ICH positivity rates remained essentially stable. Our observations can be explained by more restrictive ordering patterns for contrast-enhanced studies during the shortage period.
**Limitations:** Ground truth was based on AI image analysis rather than NLP on radiology reports.
**Ethics committee approval:** This study was approved by URMC and UWHealth institutional IRB approvals.
**Funding for this study:** Funding was received from the ACR Innovation Award for AW; Aidoc Medical.
**Author Disclosures:**
Nathan Hadjiyski: Nothing to disclose
Jeffrey Paul Kanne: Nothing to disclose
Akhil Kasturi: Nothing to disclose
Larry Stockmaster: Nothing to disclose
David Shrier: Nothing to disclose
Eric Weinberg: Nothing to disclose
Axel Wismueller: Shareholder: Aidoc Medical, Tel Aviv; Advisory Board: Aidoc Medical, Tel Aviv; Grant Recipient: Aidoc Medical, Tel Aviv
Ali Vosoughi: Nothing to disclose

### RPS 1404-4

**Correlation between postoperative pulmonary venous congestion on chest radiograph and outcome after cardiac transplantation**

*C. S. Böttger*, C. B. Fink, V. Hettlich, F. S. Jenkins, D. Scheiber, H. Aubin, A. Lichtenberg, S. Reinartz, U. Boeken; Düsseldorf/DE
*(charlotte.boettger@med.uni-duesseldorf.de)*

**Purpose:** After heart transplantation (HTX), chest radiography plays an important role in the simple, non-invasive detection of pathological changes. Given the significant relevance of fluid balance following this procedure, particularly for hemodynamic regulation, the assessment of potential pulmonary venous congestion (p.v.-congestion) in chest radiographs is crucial. The objective of this analysis was to determine a potential correlation between early postoperative p.v.- congestion after cardiac transplantation and subsequent outcomes.
**Methods or Backround:** 302 patients underwent HTX between 09.2010 and 09.2023 in our department. Recipients were retrospectively divided into 3 groups based on the severity of signs of pulmonary venous congestion on chest x-ray on the first postoperative day: Group 1 had no signs of p.v.- congestion (n= 115), Group 2 showed signs of mild p.v.- congestion (n= 145) and Group 3 showed signs of moderate to higher-grade p.v.- congestion (n= 41). One patient was excluded due to intraoperative death.
**Results or Findings:** The 30-day mortality was 4.5% in group 1, 6.2% in group 2, and 22% in group 3 (p< 0.05). Combined donor heart/lung organ harvesting was performed significantly more often in group 2 compared to group 3. Intraoperative bypass time and duration of operation were shorter in groups 1 and 2 compared to group 3. There were no significant differences between groups regarding graft rejection or infection. Patients in group 3 developed significantly more neurological complications postoperatively. 1-year and 3-year survival was reduced in patients with signs of moderate to higher grade congestion on X-ray (p> 0.05).
**Conclusion:** In this analysis, it was demonstrated that the presence of signs of p.v.-congestion and the severity on the first postoperative day after HTX have an influence on survival. Therefore, a conservative estimate of survival can be made by assessing the first postoperative chest radiograph.

**Limitations:** This was a single centre study.
**Ethics committee approval:** Not applicable.
**Funding for this study:** No funding was received for this study.
**Author Disclosures:**
Charlotte Sonja Böttger: Nothing to disclose
Sebastian Reinartz: Nothing to disclose
Udo Boeken: Nothing to disclose
Vincent Hettlich: Nothing to disclose
Artur Lichtenberg: Nothing to disclose
Hug Aubin: Nothing to disclose
Freya Sophie Jenkins: Nothing to disclose
Christian Bijan Fink: Nothing to disclose
Daniel Scheiber: Nothing to disclose

### RPS 1404-5

**Comparison of two CT-derived methods to assess alveolar collapse as a potential prognostic imaging marker in pulmonary fibrosis**

S. Scharm[1], C. M. Schaefer-Prokop[2], A. Schreuder[3], J. Fuge[1], F. Wacker[1], B. Seeliger[1], A. Prasse[1], *H-o. Shin*[1]; [1]Hannover/DE, [2]Amersfoort/NL, [3]Nijmegen/NL
*(shin.hoen-oh@mh-hannover.de)*

**Purpose:** The objective of this study was to evaluate two different CT-derived approaches for the assessment of alveolar collapse, a potential precursor of pulmonary fibrosis.
**Methods or Backround:** For this single-centre retrospective longitudinal study, 66 consecutive patients with idiopathic pulmonary fibrosis underwent CT in inspiration and expiration and pulmonary function testing at baseline. The patient population was divided into two subgroups according to their status at 3 years (death or transplantation versus clinical surveillance). Parametric response maps were generated as scatterplots of voxel-wise attenuation values of paired inspiration and expiration scans after non-linear registration. Inspiratory and expiratory attenuation histograms were also generated and analysed. Voxels with an abnormally high increase in attenuation during expiration were interpreted as "collapsed" lung tissue. A Mann-Whitney U test was performed to assess the difference in CT-derived measures between the two subgroups and logistic regression was performed to test the predictive power of FVC and both CT-derived measures (PRM and histogram analyses).
**Results or Findings:** All CT-derived PRM and histogram measures were significantly different ($p < 0.005$) between the two patient subgroups. With a discriminatory performance of AUC = 0.788, 95% CI 0.679-0.898 for PRM and AUC = 0.791, 95% CI = 0.684- 0.899 these two CT-derived measures had a superior predictive performance compared to FVC alone with AUC = 0.708, CI 95% 0.581- 0.836. The advantage of the PRM analysis is a voxel-wise matching between inspiration and expiration due to the required registration. On the other hand, the histogram parameter approach does not require advanced postprocessing but lacks the direct spatial correlation of the attenuation change between inspiration and expiration.
**Conclusion:** Both methods perform similarly well in predicting patient outcome by directly or indirectly quantifying alveolar collapse, and both outperform FVC.
**Limitations:** No limitations were identified.
**Ethics committee approval:** This study was approved by IRB, with the approval code: No. 10726_BO_K_2023
**Funding for this study:** No funding was received for this study.
**Author Disclosures:**
Cornelia M. Schaefer-Prokop: Nothing to disclose
Anton Schreuder: Nothing to disclose
Hoen-oh Shin: Nothing to disclose
Benjamin Seeliger: Nothing to disclose
Sarah Scharm: Nothing to disclose
Jan Fuge: Nothing to disclose
Antje Prasse: Nothing to disclose
Frank Wacker: Nothing to disclose

### RPS 1404-6

**Effect of inspiratory lung volume on bronchial and arterial dimensions on chest CT**

*Y. Chen*[1], R. Latisenko[2], D. Lynch[3], P. Ciet[1], J-P. Charbonnier[2], H. A. W. M. Tiddens[2]; [1]Rotterdam/NL, [2]Nijmegen/NL, [3]Denver, CO/US
*(y.chen.1@erasmusmc.nl)*

**Purpose:** Bronchus-artery (BA) dimensions on chest CT are influenced by inspiratory lung volume. We aimed to assess the effect of inspiratory lung volume on BA-ratios in patients with COPD.
**Methods or Backround:** A selected group participating in the COPDGene study received a full dose (FD, 120kVp, 200mAs) and a reduced dose (RD, 120kVp 35mAs) CTs in the same imaging session. CTs were analysed using LungQ. For segmental (G0) and distal generations, the following diameters were measured: bronchial outer (Bout), inner (Bin), wall thickness (Bwt), and artery (A), and the following BA-ratios were computed: Bout/A, Blumen/A, Bwt/A, and bronchial wall area/bronchial outer area (Bwa/Boa). Total lung capacity of the CT (TLC-CT) was computed. Differences between the volumes (ΔTLC-CT%) between the two CTs were expressed as % of the highest TLC-CT. Mixed-effect models were used to investigate the influence of TLC-CT on BA-ratios adjusted for dose protocol. AUC was used to define a cut-off value for BA-ratios in relation to ΔTLC-CT%.
**Results or Findings:** 1319 patients with a mean (SD) age of 64.4 (8.7) years were included. 329 (124) BA-pairs could be analyzed per CT. No significant difference was found for TLC-CT between FD and RD scans. A ΔTLC-CT% of 5% significantly lead to 0.015 and 0.025 decreases in Bout/A and Bin/A and 0.004 and 0.015 decreases in Bwt/A and Bwa/Boa. The cut-off values to determine when BA-ratios were influenced by lung volume were for Bout/A 4.9%, Bin/A 3.7%, Bwt/A 4.4%, and Bwa/Boa 5.4%.
**Conclusion:** The BA biomarkers provide a robust quantification of bronchial changes on CT when differences in ΔTLC-CT% are kept below 5%. Standardising volumes for clinical follow-up and trials is recommended to optimise assessment for tracking airway disease changes over time.
**Limitations:** The study examined a selected group of an elderly population,
**Ethics committee approval:** The COPDGene study was approved by the institutional review boards at each of the 21 participating clinical sites.
**Funding for this study:** This study was funded by the Erasmus MC LungAnalysis supporting grant.
**Author Disclosures:**
Jean-Paul Charbonnier: Employee: Thirona
David Lynch: Nothing to disclose
Rudolfs Latisenko: Employee: Thirona
Pierluigi Ciet: Nothing to disclose
Harm A. W. M. Tiddens: Employee: Thirona
Yuxin Chen: Nothing to disclose

### RPS 1404-7

**Quantitative CT characterises baseline regional structure and function in idiopathic pulmonary fibrosis patients with one-year diffusing capacity decline**

*H. Ko*[1], W. C. Chung[2], J. Choi[3], S. M. Choi[2], C-H. Lee[2], K. J. Chae[4], C. H. Lee[2]; [1]Chuncheon/KR, [2]Seoul/KR, [3]Kansas City, KS/US, [4]Jeonju/KR
*(muldoo@gmail.com)*

**Purpose:** We characterised regional lung structure and function in idiopathic pulmonary fibrosis (IPF) patients with a one-year diffusing capacity of carbon monoxide (DLCO) decline using quantitative inspiratory and expiratory chest computed tomography (CT) analysis.
**Methods or Backround:** Baseline and one-year pulmonary function tests (PFTs) and baseline inspiration-expiration CT scans were collected from 51 IPF patients (71.5±5.9 years). Commercial and in-house quantitative CT (qCT) software were used for the segmentation and quantification of 113 regional lung structural-functional features. Baseline qCT features were compared between patients with 5% or more percent-predicted (%pred) DLCO decline (IPF-A) and the rest (IPF-B), using Wilcoxon rank-sum test.
**Results or Findings:** Compared to IPF-B (n= 25, 1 female), IPF-A (n= 26, 4 females) had less baseline high attenuation area percent (HAA%) in the right upper (RUL, -32.0%, p= 0.011) and lower (RLL, -29.3%, p= 0.027) lobes and greater anisotropic deformation index (31.2%, p= 0.030) and relative displacement (26.2%, p= 0.047) in the left lower lobe (LLL). IPF-A also had moderately greater normalised hydraulic diameter (Dh*) at the RLL segmental airways (sRLL) (9.3%, p= 0.054). Early traction bronchiectasis was observed in the sRLL of IPF-A. IPF-A showed CT features of greater extent and severity of fibrosis in IPF. Demographics and PFTs were not significantly different at baseline.
**Conclusion:** qCT analysis characterised baseline regional lung structure and function in IPF with DLCO decline and supported visual CT interpretation. Findings suggest that advanced fibrotic changes and RLL segmental airway traction bronchiectasis may be precursors of DLCO decline presumably with progressive pulmonary fibrosis.
**Limitations:** This study has a limited number of cases.
**Ethics committee approval:** The study protocol was approved by the institutional review boards (IRB) of Seoul National University Hospital (IRB: 1810-036-977)
**Funding for this study:** This study was supported by Korea Environmental Industry & Technology Institute (KEITI) grant 2018001360001 funded by the Ministry of Environment (MOE), Republic of Korea.
**Author Disclosures:**
Hongseok Ko: Nothing to disclose
Jiwoong Choi: Nothing to disclose
Kum Ju Chae: Nothing to disclose
Chang-Hoon Lee: Nothing to disclose
Won Chul Chung: Nothing to disclose
Sun Mi Choi: Nothing to disclose
Chang Hyun Lee: Nothing to disclose

### RPS 1404-8

**Default vs selective application of artificial intelligence to classify viral pneumonia on CT imaging: impact on human performance**

*F. Rizzetto*, L. Berta, L. A. Carbonaro, D. Artioli, F. Travaglini, G. Zorzi, A. Torresin, P. E. Colombo, A. Vanzulli; Milan/IT
*(francesco.rizzetto@unimi.it)*

**Purpose:** This study aimed to assess the impact of different artificial intelligence (AI)-powered approaches on human performance in distinguishing COVID-19 from other viral pneumonia on CT imaging.
**Methods or Backround:** Three experienced radiologists blindly evaluated 220 chest CT examinations of patients with viral pneumonia (n=151 COVID-19; n=69 other viruses), assigning a CO-RADS score before (S1) and after (S2) receiving results from a validated AI classifier. Inter-reader agreement with Gwet's agreement coefficient type-2 (AC2) and performance metrics were calculated for S1 and S2. Two different S2 scenarios were considered: one where the AI output was available for all cases (default approach), and another where the AI prediction was applied only to CO-RADS=3 cases from S1 (selective approach).
**Results or Findings:** The readers showed good-to-excellent agreement in assigning CO-RADS for all scenarios (range AC2=0.77-0.81). On average, CO-RADS changes between S1 and S2 occurred in 18% of cases, with 29% involving patients initially assigned CO-RADS=3. In these cases, the use of AI output correctly classified 85% of patients. Conversely, when the radiologists were confident in S1 diagnosis (CO-RADS≠3), class changes in S2 occurred in 7% of cases. This prevented incorrect diagnosis in 45% of patients but led to a missed correct classification in the remaining 55%. In S1, the readers achieved 78% accuracy, with 15% of patients classified as CO-RADS=3. In S2, accuracy was 81% with 16% of CO-RADS=3 for the default approach, and 79% with 10% of CO-RADS=3 for the selective approach. Significant differences were only observed in the proportion of CO-RADS=3 cases in S2 with the selective approach (p<0.009).
**Conclusion:** AI helps distinguish COVID-19 from other viral pneumonias in equivocal cases but its reliability diminishes when the reader is already confident on diagnosis.
**Limitations:** The main limitation is the retrospective single centre study design.
**Ethics committee approval:** This study received approval from the Local Ethics Committee (decision number: 188-22042020)
**Funding for this study:** No funding was received for this study.
**Author Disclosures:**
Francesco Rizzetto: Other: Bracco Imaging
Luca Berta: Nothing to disclose
Luca Alessandro Carbonaro: Nothing to disclose
Giulia Zorzi: Nothing to disclose
Diana Artioli: Nothing to disclose
Angelo Vanzulli: Nothing to disclose
Alberto Torresin: Nothing to disclose
Francesca Travaglini: Nothing to disclose
Paola Enrica Colombo: Nothing to disclose

**14:00-15:30** **Research Stage 1**

### Research Presentation Session: Abdominal Viscera & GI Tract

### RPS 1501

### Insights in pancreatic imaging with current and novel techniques

**Moderator**
N. Kartalis; Stockholm/SE
*(nikolaos.kartalis@me.com)*

### RPS 1501-2

**T1 and T2 mapping quantitative MRI in the characterisation of pancreatic cystic lesions: preliminary results**

*A. Beleù*[1], R. Napoli[2], E. Grosso[1], C. Nistri[1], M. Piccino[1], M. Massani[1], G. Zanus[1], G. Morana[1]; [1]Treviso/IT, [2]Padua/IT
*(alessandro.beleu@aulss2.veneto.it)*

**Purpose:** MRI characterisation of pancreatic cystic lesions is mainly based on qualitative and morphological criteria. Quantitative imaging techniques, such as T1 and T2 relaxation times mapping, could help in their characterisation of these lesions.
**Methods or Backround:** Patients undergoing abdominal MRI were prospectively enrolled. The T1- and T2-values of the content of the pancreatic cyst were sampled by a single radiologist by evaluating mean, minimum, maximum and range of T1 and T2. Dimensional and clinical-laboratory parameters were collected. Relaxation times were compared in different lesion groups using ANOVA and Kruskall-Wallis test.
**Results or Findings:** Of the 350 patients enrolled, 126 were finally included (age 68±11 years, 55% male). 106 branch-duct intraductal papillary mucinous neoplasms (IPMN-BD), two main duct (IPMN-MD), six serous cystadenomas (SCA), two mucinous cystadenomas (MCA), two cystic neuroendocrine tumours (cNET), two pseudocysts, two walled-off necrosis (WON), one lymphoepithelial cyst (LEC) and one cystic chronic pancreatitis were included. Mean T1 of all cystic lesions was 1985±826 ms, while median T2 was 96 [82-126] ms. T1 values were significantly different among all lesions (p=.02). IPMN-BD demonstrated mean (p=.02), minimum (p=.01), and maximum (p=.02) T2 values significantly different from SCA; ROC curve test showed that mean (AUC 0.78; p=.02), minimum (AUC 0.8; p=01) and maximum (AUC 0.78; p=.02) T2 values are sensitive in the differential diagnosis of IPMN-BD vs. SCA. A significant difference in mean T1 was observed (p=.03) compared to IPMN-BD and MCA. IPMN-BD showed significant difference in mean (p=.01), minimum (p=.01), maximum (p=.02) T1 and T2 range (p=.01) compared to WON. SCA mean (p=.01) and minimum T1 (p=.05) were significantly different to MCA.
**Conclusion:** The T1 and T2 relaxation times of pancreatic cystic lesions are different in many different lesion groups.
**Limitations:** The relatively low number of lesions, with different distribution in the groups, was an identified limitation.
**Ethics committee approval:** No information provided by the submitter.
**Funding for this study:** No funding was received for this study.
**Author Disclosures:**
Marco Piccino: Nothing to disclose
Giacomo Zanus: Nothing to disclose
Eva Grosso: Nothing to disclose
Alessandro Beleù: Nothing to disclose
Marco Massani: Nothing to disclose
Raffaele Napoli: Nothing to disclose
Giovanni Morana: Nothing to disclose
Cristina Nistri: Nothing to disclose

### RPS 1501-3

**Development and validation of an imaging-based model to predict the malignant potential of intraductal papillary mucinous neoplasm of the pancreas: comparison with international consensus guidelines**

*J. Park*[1], J. H. Kim[1], J. S. Bae[1], H-J. Kang[1], S-Y. Choi[2]; [1]Seoul/KR, [2]Bucheon/KR

**Purpose:** The study aimed to develop and validate imaging-based models for predicting the malignant risk of intraductal papillary mucinous neoplasm (IPMN).
**Methods or Backround:** We retrospectively analysed data from 245 surgically-confirmed IPMN patients who had preoperative CT and MRI scans for model development. Two radiologists assessed cyst size, presence of enhancing mural nodule (EMN), EMN size, main pancreatic duct (MPD) diameter, thickened/enhancing cyst wall, abrupt MPD calibre change with distal pancreatic atrophy, and lymphadenopathy. Multiple logistic regression models predicting malignancy risk were created using either continuous (model C) or dichotomised variables (model D) from the significant imaging features on univariable analysis. Validation included internal (n=55) and external (n=43) datasets with pathologically-confirmed IPMNs. Model performance was assessed using the area under the receiver-operating characteristic curve (AUC) and compared with the Fukuoka guideline-based model (model F).
**Results or Findings:** Model C identified age, EMN size, MPD diameter, and lymphadenopathy as independent predictors on CT, and age, presence of EMN, and EMN size on MRI. In model D, independent predictors were age ≥ 68, size ≥ 31 mm, EMN ≥ 6 mm, MPD ≥ 7 mm, and lymphadenopathy on CT, and age ≥ 68, size ≥ 33 mm, EMN ≥ 4.5 mm, MPD ≥ 7.5 mm, and lymphadenopathy on MRI. Model C (AUCs for external datasets, 0.785-0.899) performed slightly better than model D (AUCs, 0.736-0.912) without statistical significance. No significant difference was observed between models C and F (AUCs, 0.827-0.952). Combining model C with the presence of obstructive jaundice improved diagnostic performance (AUCs, 0.883-0.941) without statistical significance.
**Conclusion:** Our imaging-based models effectively predicted the malignant risk of IPMN, and its combination with clinical findings enhanced diagnostic accuracy.
**Limitations:** Identified limitations were the retrospective nature of this study and the limited sample size.
**Ethics committee approval:** This study was approved by the institutional review board of Seoul National University Hospital, and the requirement for informed consent was waived because of the retrospective nature of this study.

**Funding for this study:** No funding was received for this study.
**Author Disclosures:**
Jung Hoon Kim: Nothing to disclose
Junghoan Park: Nothing to disclose
Seo-Youn Choi: Nothing to disclose
Jae Seok Bae: Nothing to disclose
Hyo-Jin Kang: Nothing to disclose

### RPS 1501-4

**Common imaging findings and clinical effects of high-grade pancreatic intraepithelial neoplasia in the remnant pancreas in patients with intraductal papillary mucinous neoplasms of the pancreas**

*J. H. Kim*, M. C. Kim, S. K. Jeon, H-J. Kang; Seoul/KR
*(jhkim2008@gmail.com)*

**Purpose:** The study aimed to investigate the common computed tomography (CT) findings and clinical effects of HG PanIN in the remnant pancreas in patients with intraductal papillary mucinous neoplasm (IPMN) of the pancreas.
**Methods or Backround:** Two hundred and fifty one patients with surgically confirmed IPMNs (118 malignant [invasive carcinoma/high-grade dysplasia] and 133 benign [low-grade dysplasia]) were retrospectively enrolled. The grade of PanIN (233 absent/low-grade and 18 high-grade) was recorded, and all patients underwent serial CT follow-up before and after surgery. Two radiologists analysed the CT findings of high-risk stigmata or worrisome features according to 2017 international consensus guidelines. They also analysed tumour recurrence on serial follow-up CT after surgery. Statistical analyses were performed to identify significant predictors and clinical impact on postoperative outcomes of HG PanIN.
**Results or Findings:** PanIN grade showed a significant association with IPMN grade (p=0.012). Enhancing mural nodules ≥5 mm, abrupt main pancreatic duct (MPD) changes with distal pancreatic atrophy, increased mural nodule size and MPD diameter were common findings in HG PanIN (P<0.05). In multivariate analysis, abrupt MPD change with distal pancreatic atrophy (odds ratio [OR] 6.59, 95% CI: 2.32-18.72, <0.001) and mural nodule size (OR 1.05, 95% CI: 1.02-1.08, 0.004) were important predictors for HG PanIN. During postoperative follow-up, HG PanIN (OR 4.98, 95% CI: 1.22-20.33, 0.025) was significantly associated with cancer recurrence in the remnant pancreas.
**Conclusion:** Although PanIN is a microscopic, non-invasive precursor of invasive carcinoma, CT can be useful for predicting HG PanIN using common features, such as abrupt MPD changes and mural nodules. In HG PanIN, extra caution is needed to monitor postoperative recurrence during follow-up.
**Limitations:** Identified limitations were the retrospective nature of the study and its limited sample size.
**Ethics committee approval:** The study was approved by Seoul National University Institutional Review Board with approval code: 2007-183-1143.
**Funding for this study:** No funding was received for this study.
**Author Disclosures:**
Sun Kyung Jeon: Nothing to disclose
Jung Hoon Kim: Nothing to disclose
Hyo-Jin Kang: Nothing to disclose
Min Cheol Kim: Nothing to disclose

### RPS 1501-5

**Prediction of tumour cellularity in resectable PDAC from preoperative computed tomography imaging**

*F. Jungmann*, G. Kaissis, S. Ziegelmayer, F. N. Harder, K. Steiger, M. Makowski, R. Braren, F. Lohöfer; Munich/DE

**Purpose:** Pancreatic ductal adenocarcinoma (PDAC) remains a tumour entity with a poor prognosis and a 5-year survival rate below 10%. Until today, non-invasive prediction of individual patient outcomes remains an unresolved task. We previously showed a strong association between magnetic resonance imaging-based tumour cell estimates and patient survival. In this study, we aimed to transfer this finding to more broadly applied computed tomography (CT) imaging for non-invasive risk stratification.
**Methods or Backround:** Discrete cellularity regions of the PDAC resection specimen were analysed by a routine histopathological workup. Regional tumour cellularity and CT-derived Hounsfield Units (HU), as well as iodine concentrations, were regionally matched. One-way ANOVA and pairwise t-tests were performed to assess the relationship between different cellularity levels in conventional, virtual monoenergetic 40 keV (monoE 40 keV) and iodine map reconstructions.
**Results or Findings:** A statistically significant negative correlation between regional tumour cellularity in histopathology and CT-derived HU from corresponding image regions was identified. Radiological differentiation was best possible in monoE 40 keV CT images. However, HU values differed significantly in conventional reconstructions as well, indicating the possibility of a broad clinical application of this finding.
**Conclusion:** In this study, we establish a novel method for CT-based prediction of tumour cellularity for in-vivo tumour characterisation in PDAC patients.
**Limitations:** Identified limitations were: (1) the retrospective nature of the study; (2) the limited cohort size; and (3) that this was a single institution study.
**Ethics committee approval:** The study was approved by the local institutional review board of the Technical University of Munich.
**Funding for this study:** No funding was received for this study.
**Author Disclosures:**
Rickmer Braren: Nothing to disclose
Sebastian Ziegelmayer: Nothing to disclose
Friederike Jungmann: Nothing to disclose
Katja Steiger: Nothing to disclose
Marcus Makowski: Nothing to disclose
Felix N. Harder: Nothing to disclose
Georgios Kaissis: Nothing to disclose
Fabian Lohöfer: Nothing to disclose

### RPS 1501-6

**A nomogram based on contrast-enhanced CT radiomics to preoperatively predict perineural invasion for patients with pancreatic adenocarcinoma**

*Y. Deng*[1], H. Yu[1], Z. Huang[1], B. Song[2]; [1]Chengdu/CN, [2]Sanya/CN
*(1157871240@qq.com)*

**Purpose:** The study aimed to develop and validate a nomogram based on contrast-enhanced CT (CECT) radiomics features and clinical characteristics for preoperative assessment of perineural invasion (PNI) in pancreatic adenocarcinoma (PAC).
**Methods or Backround:** The retrospective study recruited 217 PAC patients with histopathology and randomly divided them into a training and testing cohort, at a ratio of 7:3. Radiomics features were extracted from the artery and portal venous phase of CECT. Univariate analysis and least absolute shrinkage and selection operator (LASSO) method were applied using the 10-fold cross-validation for feature selection in the training cohort. The selected features were integrated into the radiomics score (Rad-score). Two experienced radiologists evaluated the status of PNI based on CECT (CTPNI). A nomogram was constructed by Rad-score and characteristics of statistically significant differences. Calibration and classification metrics were used to evaluate the nomogram performance.
**Results or Findings:** The lymph node status determined on CT (CTLN) and CTPNI were statistically different between the PNI positive and negative groups. The AUC for the Rad-score in the training cohort was close to that in the testing cohort (AUC 0.720 and 0.640). The AUC of CTPNI was 0.610 in the training cohort and 0.675 in the testing cohort. The Rad-score, CTLN and CTPNI were used to construct a nomogram, and it achieves favourable discrimination of PNI status with an AUC of 0.846 and 0.741 in the training and testing cohort, respectively. The nomogram achieved best performance both in the training cohort and testing cohort compared with Rad-score and CTPNI (P<0.05).
**Conclusion:** The development of a nomogram incorporating Rad-score, CTLN, and CTPNI holds promise as a valuable non-invasive tool for the preoperative evaluation of PNI status in PAC patients.
**Limitations:** It is a retrospective, single-centre study with limited sample size, and no external validation group.
**Ethics committee approval:** The retrospective study was approved by the institutional review board of West China Hospital of Sichuan University. Each patient waived informed consent.
**Funding for this study:** No funding was received for this study.
**Author Disclosures:**
Haopeng Yu: Nothing to disclose
Zixing Huang: Nothing to disclose
Bin Song: Nothing to disclose
Yan Deng: Nothing to disclose

### RPS 1501-7

**Evaluation of the efficacy of neoadjuvant chemotherapy in pancreatic ductal adenocarcinoma by three-dimensional quantitative analysis of spectral CT**

*D. Zhang*[1], X. Zhang[2], Y. Zou[1]; [1]Chongqing/CN, [2]Chengdu/CN

**Purpose:** To explore the value of three-dimensional quantitative analysis of spectral CT in the efficacy evaluation of neoadjuvant chemotherapy for pancreatic ductal adenocarcinoma (PDAC).
**Methods or Backround:** This retrospective study enrolled 49 patients with PDAC who underwent contrast-enhanced spectral CT before one month and after two months of neoadjuvant chemotherapy from October 2021 to June 2023. Conventional polyenergetic images (PEIs), iodine-concentration (IC) images and Z-effective (Z-eff) images were generated at portal venous phase (PVP). The whole tumour volume, IC-total and Z-eff-total were measured using a semiautomatic segment software. The changes of the longest diameter and whole tumour volume, IC-mean and IC-total, Z-eff-mean and Z-eff-total were compared using Wilcoxon signed rank test. The cut-off value of changes for curative effect was obtained through a receiver operating characteristic (ROC) curve analysis.

**Results or Findings:** The changes of the longest diameter and whole tumour volume (P＜0.001), IC-mean and IC-total (P＜0.001), Z-eff-mean and Z-eff-total (P＜0.001) showed a significant difference, respectively. The change of the longest diameter's cut-off value was 0.193 (sensitivity 57.1%, specificity 93.3%, area under the ROC curve [AUC] 0.725). The change of the whole tumour volume's cut-off value was 0.222 (sensitivity 85.7%, specificity 73.3%, AUC 0.746). The change of the IC-total's cut-off value was 0.191 (sensitivity 92.9%, specificity 60%, AUC 0.795). The change of the Z-eff-total's cut-off value was 0.167 (sensitivity 85.7%, specificity 66.7%, AUC 0.800).
**Conclusion:** Three-dimensional quantitative parameters of spectral CT can effectively evaluate the efficacy of neoadjuvant chemotherapy in PDAC, and the diagnostic efficacy is higher than that of lesion's size.
**Limitations:** An identified limitation was the small number of patients in the study.
**Ethics committee approval:** This study was approved by the local institutional review board and informed consent was obtained from all patients.
**Funding for this study:** No funding was received for this study.
**Author Disclosures:**
Xiaodi Zhang: Nothing to disclose
Ya Zou: Nothing to disclose
Dan Zhang: Nothing to disclose

### RPS 1501-8

**Utility of conventional CT and MRI parameters in grading the severity of chronic pancreatitis: can delayed enhancement ratio be the guiding light?**

*M. Narang*, S. Sharma, K. S. Madhusudhan; New Delhi/IN
*(narangmohak@gmail.com)*

**Purpose:** The study aimed to evaluate the role of conventional CT and MRI parameters used in routine clinical practice for grading the severity of chronic pancreatitis (CP).
**Methods or Backround:** This prospective study conducted from December 2019 to December 2021 included 72 patients (mean age: 30 years; 59 men) with suspected or confirmed CP graded as equivocal, mild, and moderate-marked using composite imaging (Cambridge classification) and endoscopic ultrasound (Rosemont's criteria) who underwent multiphasic CT and MRI of the abdomen. Routine parameters such as antero-posterior pancreatic thickness (APPT) and delayed enhancement ratio (DER) were evaluated on CT. APPT, signal intensity ratio (SIR) on T1-weighted sequence, pancreatic duct contour (PDC), apparent diffusion coefficient (ADC) and DER were evaluated on MRI. Multiphasic CT of 20 renal donors and abdominal MRI of 20 patients with renal mass served as controls. One way ANOVA with post-hoc Dunn-Bonferroni correction was used for analysing APPT, ADC, SIR and DER. PDC were compared using chi-square test.
**Results or Findings:** The study group included patients with equivocal CP (n=20), mild CP (n=18) and moderate-marked CP (n=34). All individual parameters were significantly different between controls and moderate-marked CP (p<0.05). Additionally, DER (on CT and MRI) was significantly different between controls and mild CP (p<0.02, cut-off = 1.77 on CT, sensitivity = 88.89% and specificity = 75% and 3.56 on MRI, sensitivity = 83.33% and specificity = 95%). Area under curve (AUC) for DER on CT and MRI for differentiating controls and mild CP were 0.87 and 0.91 respectively.
**Conclusion:** DER on CT and MRI is a simple and accurate imaging marker for grading of CP and may be useful in the diagnosis of mild CP before the appearance of ductal changes.
**Limitations:** The small sample size was an identified limitation of this study.
**Ethics committee approval:** The study was approved by the Institutional Ethics Committee.
**Funding for this study:** No funding was received for this study.
**Author Disclosures:**
Sanjay Sharma: Nothing to disclose
Mohak Narang: Nothing to disclose
K. S. Madhusudhan: Nothing to disclose

### RPS 1501-9

**Intra- and interpatient variability of iodine concentration in pancreatic parenchyma for individualised contrast agent application protocols in dual-layer spectral CT**

*M. F. G. Konrad*, L. M. Wünschmann, T. Mokry, H-U. Kauczor, T. F. Weber, W. Stiller; Heidelberg/DE
*(mathis.konrad@med.uni-heidelberg.de)*

**Purpose:** Verifying the quantifiability of intra- and interpatient variability of iodine concentration (IC) in pancreatic parenchyma measured using dual-layer spectral CT for distinct individualised contrast agent (CA) application protocols in relation to liver parenchyma.
**Methods or Backround:** To compare the variability of IC in pancreatic parenchyma with that known for liver parenchyma, the mean IC of healthy pancreatic parenchyma was measured for 150 oncologic patients, who were retrospectively allocated to three groups (G1-G3, n=50 each) in a previous study. Imaging data were acquired during portal-venous contrast-enhanced thoraco-abdominal spectral CT staging examinations at two subsequent time points. The applied CA volume was either chosen by a radiographer based on body-mass-index resulting in identical (G1) or different (G2) volume for both examinations, or individually calculated (G3) using a predefined function. Intra- and interpatient coefficients of variation (CV) were calculated for the measured IC and attenuation in virtual monoenergetic image data (VMI, 40 keV) without and with normalisation to the reference structures portal vein, aorta, and spleen, respectively.
**Results or Findings:** Intra- and interpatient CV for IC were low in G3 (13.8% and 21.6%), higher in G2 (23.2% and 23.6%) and mixed in G1 (10.6% and 27.0%). Variability of IC was lower compared to liver parenchyma in G3 (-6.0% and -3.6%). Values with the highest variability (interpatient CV for G1) profit the most from normalisation (mean CV reduction of -5.0%). Normalisation to portal vein and aorta leads to examination-wise differences in IC no longer being significant (p>0.05). For all patient groups all CVs are lower for VMI compared to IC (-7.6% to -0.1%).
**Conclusion:** Variability of IC in pancreatic and liver parenchyma is comparable. Individualised calculation of CA volume leads to lower variability of IC. Normalisation can stabilise high-variability measurements.
**Limitations:** An identified limitation was the retrospective, single-centre nature of this study.
**Ethics committee approval:** This study was approved by the ethics committee of the Medical Faculty of the University of Heidelberg, approval number S-348/2019
**Funding for this study:** This study received research grant support from Philips Healthcare.
**Author Disclosures:**
Theresa Mokry: Speaker: Bayer; Speaker: DRG; Speaker: Bracco; Speaker: AKD Congress+Events
Wolfram Stiller: Research/Grant Support: Philips Healthcare
Hans-Ulrich Kauczor: Speaker: Philips Healthcare; Research/Grant Support: Philips Healthcare
Tim Frederik Weber: Research/Grant Support: Philips Healthcare
Mathis Franz Georg Konrad: Research/Grant Support: Philips Healthcare
Linda Marisa Wünschmann: Nothing to disclose

### RPS 1501-10

**Quantitative image analysis in pancreatic cancer: might contrast agents become redundant?**

*J. Gotta*, V. Koch, L. D. Grünewald, S. Mahmoudi, S. Martin, T. Vogl; Frankfurt/DE

**Purpose:** The evolution of computed tomography (CT) technology alongside the emerging field of radiomics has opened promising avenues in diagnostic assessment of cancer. This study seeks to explore the potential of a machine learning classifier rooted in dual-energy computed tomography (DECT) radiomics, specifically in its ability to differentiate between malignant and benign pancreatic lesions in non-contrast scans.
**Methods or Backround:** In this study, a total of 100 patients who underwent third-generation DECT between November 2018 and October 2022 were included. Of these, 60 patients had pancreatic cancer, while 40 had normal pancreatic tissue. 107 radiomics features per patient were extracted from non-contrast and arterial-enhanced DECT scans. To develop and validate our models, the dataset was divided into distinct training and test subsets. Stepwise feature reduction was conducted to identify the most important features. Subsequently, two gradient-boosted tree models were trained.
**Results or Findings:** The trained machine learning classifiers achieved a diagnostic accuracy of 0.97 in the arterial-enhanced model and 0.88 in non-contrast scans with areas under the curve of 0.97 (95% CI, 0.9178-1, p<0.001) and 0.96 (95% CI, 0.9013-1, p <0.001), respectively. No significant differences were observed between both models (p=0.52). Meanwhile, both CT-based radiomics models exhibited similar results in distinguishing between pancreatic tumours and normal pancreatic tissue.
**Conclusion:** Our study demonstrates the potential for employing radiomics on non-contrast DECT imaging to differentiate pancreatic cancer from healthy tissue. This innovative method holds promise in enhancing the early detection of pancreatic cancer, reducing radiation exposure and ultimately improving patient prognosis.
**Limitations:** Firstly, this study was conducted at a single centre. Secondly, due to the retrospective study design, inherent limitations may affect the reliability and generalisability of the findings. Finally, clinical variables were not integrated into our radiomics methodology, even though this might have led to a further increase in diagnostic performance.
**Ethics committee approval:** This study was approved by the ethics committee of the University Hospital Frankfurt.

**Funding for this study:** No funding was received for this study.
**Author Disclosures:**
Simon Martin: Nothing to disclose
Thomas Vogl: Nothing to disclose
Vitali Koch: Nothing to disclose
Scherwin Mahmoudi: Nothing to disclose
Leon David Grünewald: Nothing to disclose
Jennifer Gotta: Nothing to disclose

**14:00-15:30** **Research Stage 2**

### Research Presentation Session: Neuro

### RPS 1511

### Parkinson's, movement disorders, spine and nerve imaging

### RPS 1511-1

**Assessment of nerve root compression and dorsal root ganglia in patients with radiculopathy using 7 Tesla MR imaging**

*G. C. Feuerriegel*, A. A. Marth, C. Germann, F. Wanivenhaus, D. Nanz, R. Sutter; Zurich/CH

**Purpose:** This study aimed to evaluate the diagnostic value of 3D dual-echo steady-state (DESS) MR imaging of the cervical spine at 7 T compared to 3 T in patients with cervical radiculopathy.
**Methods or Backround:** Between March 2020 and January 2023, patients diagnosed with cervical radiculopathy were prospectively recruited prior to surgical decompression and underwent 3D DESS imaging on 3 T and 7 T MRI. Two radiologists independently assessed cervical nerve root compression and the dimensions of the dorsal root ganglion (DRG). Likert scales were used to assess signal intensity, visibility of nerve anatomy, diagnostic confidence and image artefacts. The degree of neuroforaminal stenosis was assessed on standard clinical 3 T scans. Statistical analysis included diagnostic accuracy and inter-reader reliability. The Wilcoxon signed-rank test was used to assess differences between groups.
**Results or Findings:** 48 patients (mean age 57±12 years, 22women) were included in the study with the highest prevalence of severe neuroforaminal stenosis observed at C6 (n=68) followed by C7 (n=43). Direct evaluation of nerve root compression showed significantly higher diagnostic confidence and visibility of cervical nerve rootlets, roots and DRG on 7 T DESS than on 3 T DESS (diagnostic confidence P=0.01, visibility: P<0.01). Assessment of nerve root compression using 7 T DESS allowed more sensitive grading than standard clinical MRI (P<0.01) and improved the performance in predicting sensory or motor dysfunction (AUC combined: 0.87).
**Conclusion:** 7T DESS imaging provides direct assessment of cervical nerve root compression in patients with radiculopathy, predicting sensory or motor dysfunction better than standard clinical MRI. The diagnostic confidence and image quality of 7T DESS were found to be superior to that of 3 T DESS.
**Limitations:** 7T MRI is not widely available and only patients with radiculopathy due to degenerative changes were included.
**Ethics committee approval:** This study was approved by the Cantonal Ethics Committee Zurich.
**Funding for this study:** No funding was received for this study.
**Author Disclosures:**
Adrian Alexander Marth: Nothing to disclose
Florian Wanivenhaus: Nothing to disclose
Daniel Nanz: Nothing to disclose
Christoph Germann: Nothing to disclose
Reto Sutter: Nothing to disclose
Georg Constantin Feuerriegel: Nothing to disclose

### RPS 1511-2

**Accuracy of automated AI-driven susceptiblity map-weighted MRI analyses for neurodegenerative Parkinsonism**

*E. Wallert*[1], E. Van De Giessen[1], M. Beudel[1], D. H. Shin[2], T. Van Mierlo[3], J. Blankevoort[4], H. Berendse[1], R. De Bie[1], J. Booij[1]; [1]Amsterdam/NL, [2]Seoul/KR, [3]Haarlem/NL, [4]Almere/NL
*(e.d.wallert@amsterdamumc.nl)*

**Purpose:** This study aimed to determine the accuracy of AI-driven automated diagnostic susceptibility map-weighted imaging (SMWI) software in patients who presented with a clinically uncertain Parkinsonian syndrome (CUPS).
**Methods or Backround:** Parkinson’s disease (PD) is characterized by degeneration of the substantia nigra (SN), particularly in the nigrosome-1 area, and is associated with locally increased iron deposition. The accuracy of a clinical diagnosis is estimated at 80%. SMWI is a novel MRI sequence that combines magnitude images with quantitative susceptibility mapping to enhance the contrast of nigrosome-1 and loss of its signal in neurodegenerative Parkinsonian patients due to iron deposition, which may aid diagnostics. A diagnostic accuracy study was performed in patients who had a dopamine transporter (DAT) SPECT because of CUPS between January 2019 and July 2023. The index test was the result of AI-driven diagnostic software (Heuron IPD, formerly mPDia) analysis of the SMWI sequence between May 2022 and September 2023. The reference standard was the result of the DAT SPECT.
**Results or Findings:** 132 patients were enrolled in this study. Twelve were excluded from the analysis; four because of uncertain DAT-SPECT results and eight because of severe artefacts on MRI. DAT-SPECT was abnormal in 53 and normal in 67 patients. In this preliminary analysis, the accuracy of the diagnostic software compared with the DAT-SPECT results was 88% with a sensitivity of 87% and specificity of 89%.
**Conclusion:** The diagnostic accuracy of the diagnostic software is promising in a clinically relevant population of patients who presented with CUPS.
**Limitations:** A limitation of this study is the delay between the index test and the reference test of 1.6 years. DAT-SPECT is considered to be a golden standard in the diagnostics of patients with CUPS. However, the follow-up time is limited to 2.3 years.
**Ethics committee approval:** This study was approved by the Amsterdam UMC METC.
**Funding for this study:** Funding for this study was received from Heuron Co.
**Author Disclosures:**
Elsmarieke Van De Giessen: Nothing to disclose
Jeroen Blankevoort: Nothing to disclose
Dong Hoon Shin: CEO: Heuron
Elon Wallert: Nothing to disclose
Martijn Beudel: Nothing to disclose
R.M.A. De Bie: Nothing to disclose
Henk Berendse: Nothing to disclose
Jan Booij: Nothing to disclose
Tom Van Mierlo: Nothing to disclose

### RPS 1511-3

**Functional brain connectivity in patients with de novo Parkinson's disease based on rs-fMRI**

*N. Pucci*, S. Minosse, E. Picchi, V. Ferrazzoli, F. Garaci, T. Schirinzi, F. Di Giuliano; Rome/IT

**Purpose:** This study aimed to evaluate a putative reorganisation of brain networks between de novo Parkinson's disease patients and healthy controls by measures based on graph theory.
**Methods or Backround:** 31 de novo PD patients and 30 healthy controls underwent MRI examination to obtain rsf-MRI and 3D-T1-MPRAGE data. The rs-fMRI data were pre-processed in FLS. The time series was parcelled into 116 regions using the Automated Anatomical Labelling Atlas (AAL) per subject. Pearson correlation between all-time series was used to generate subject-based adjacency matrices. Local nodal measures (degree and centrality), functional integration measures (global efficiency), functional segregation measures (local efficiency, clustering coefficient, transitivity and modularity), and resilience measures (assortment coefficient) were calculated using Brain Connectivity. The local measures were analysed by means of disruption index k. We calculated the hub regions in the two groups using the BC of each node. All local and global variables were compared using the Mann-Whitney U Test.
**Results or Findings:** We found no statistically significant differences in measures of global variables between de novo PD and healthy controls. We did find statistically significant differences due to disaggregation index k in all local metrics. For all statistically significant comparisons, the disruption index k was lower in the de novo PD group than in the healthy controls.
**Conclusion:** The alterations found by local network measurements highlight a reorganisation of brain networks in de novo PD patients, supporting the hypothesis that the analysis of functional connectivity may facilitate a better understanding of the complexity of PD pathophysiology from the earliest stages of the disease.
**Limitations:** Our study was performed in a relatively small sample size and didn't include neurocognitive tests, so we cannot examine putative associations between neurocognitive status and MRI parameters.
**Ethics committee approval:** The study protocol was approved by the local Institutional Review Board and adhered to the tenets of the Declaration of Helsinki. All subjects provided written informed consent.
**Funding for this study:** Funding for this study was supported by #NEXTGENERATIONEU (NGEU) and funded by the Ministry of University and Research (MUR), National Recovery and Resilience Plan (NRRP), project MNESYS (PE0000006) - A Multiscale integrated approach to the study of the nervous system in health and disease (DN. 1553 11.10.2022).

**Author Disclosures:**
Silvia Minosse: Nothing to disclose
Francesco Garaci: Nothing to disclose
Eliseo Picchi: Nothing to disclose
Tommaso Schirinzi: Nothing to disclose
Valentina Ferrazzoli: Nothing to disclose
Noemi Pucci: Nothing to disclose
Francesca Di Giuliano: Nothing to disclose

### RPS 1511-4

**Diagnostic value of quantitative susceptibility mapping in assessing iron deposition in the basal ganglia of patients with Parkinson's disease**

*V. Shahmaei*, F. Sodaei, F. Faeghi, F. Ashrafi; Tehran/IR
*(vahidshahmaei.sbmu@gmail.com)*

**Purpose:** This study aimed to assess iron deposition in the basal ganglia using QSM, determine the diagnostic value of this method, and explore the association between disease stage and QSM values in individual nuclei. Iron deposition in the brain is commonly associated with Parkinson's disease. Quantitative Susceptibility Mapping (QSM) has proven to be a more sensitive imaging technique compared to T2-weighted imaging, T2*, and R2. However, there have been limited studies utilising QSM to evaluate iron deposition in the basal ganglia of Parkinson's disease patients.
**Methods or Backround:** A total of 25 patients were assessed using the Hoehn and Yahr test, which classified them into three different stages. 15 healthy subjects were included as a control group. Routine MRI sequences and QSM were performed using a Siemens 3. Tesla scanner. Signal processing and image analysis were conducted using STI Suite software. Susceptibility measures of all basal ganglia nuclei were extracted individually.
**Results or Findings:** Significant differences in susceptibility measures were observed in the Substantia Nigra, Red Nucleus, Thalamic Nucleus, and Globus Pallidus nuclei between the patients and control groups (P-value < 0.001). There was a significant relationship between disease stage and QSM in the Substantia Nigra, Red Nucleus, and Globus Pallidus nuclei (P-value < 0.05, R= 0.51-0.78). QSM values demonstrated a significant association with disease stage across all nuclei (P-value < 0.001). Furthermore, QSM exhibited higher accuracy in the Substantia Nigra, Globus Pallidus, Red Nucleus, and Thalamic Nucleus, respectively (Acc= 88%-98%).
**Conclusion:** Utilising QSM to assess iron deposition in the Red Nucleus, Substantia Nigra, and Globus Pallidus nuclei can aid in the diagnosis and staging of patients with Parkinson's disease. Future studies focusing on the disease stage could provide further information.
**Limitations:** No limitations were identified.
**Ethics committee approval:** The informed consent was obtained from participants included in the study following the supervision of the ethics committee of Shahid Beheshti University of Medical Sciences, Tehran, Iran.
**Funding for this study:** No funding was received for this study.
**Author Disclosures:**
Forough Sodaei: Nothing to disclose
Farzad Ashrafi: Nothing to disclose
Fariborz Faeghi: Nothing to disclose
Vahid Shahmaei: Nothing to disclose

### RPS 1511-5

**Human visual nigrosome analysis improves AI-based diagnoses in neurodegenerative disease**

*E. Sayilir*[1], E. Piot[1], C. Bonardel[1], F. Renard[2], S. Grand[1], A. Attye[2], A. Krainik[1]; [1]Grenoble/FR, [2]La Tronche/FR

**Purpose:** This study aimed to evaluate the diagnostic performance of BrainGML a manifold-learning AI software (Geodaisics.com) in neurodegenerative diseases combined with the visual analysis of nigrosome imaging. Neurodegenerative diseases are associated with regional cerebral atrophy patterns, such as temporohippocampal and parietal atrophy in Alzheimer's disease (AD), and frontotemporal atrophy in frontotemporal dementia (FTD). Parkinson's (PD) and dementia with Lewy bodies (DLB) have subtle structural abnormalities such as insular atrophy and nigrosome loss. Artificial intelligence software shows potential for accurate diagnosis of atrophy patterns, while nigrosome analysis is still unavailable.
**Methods or Backround:** A retrospective study was conducted on patients with AD, FTD, PD, and DLB. BrainGML analysed cerebral atrophy using 3DT1 images, assigning the highest probability for Normal, AD, FTD, or PD. Nigrosome visual analysis was performed on susceptibility-weighted images by 4 radiologists who determined whether nigrosomes were normal or abnormal. The primary outcome was the accuracy of the radiological diagnosis, defined as 'Right', 'Wrong', or 'Undefined' (when nigrosome imaging was normal in PD and DLB, or when nigrosome imaging was abnormal in AD or FTD).
**Results or Findings:** The cohort included 79 patients (29 AD, 11 FTD, 26 PD, 13 DLB). Nigrosomes were normal in AD (100%) and FTD (100%), and were abnormal in PD (92%) and DLB (62%). BrainGML provided 59% Right diagnoses on trained diagnoses (all but DLB), and 49% on all patients including DLB. Combining nigrosome analysis with BrainGML decreased the ratio of 'Wrong' diagnoses from 51% to 15%, which was replaced by the increase of 'Undefined' diagnoses from 0 to 36%.
**Conclusion:** In conclusion, adding nigrosome visual analysis to BrainGML highest diagnosis probability turned most 'wrong' diagnoses into 'undefined' diagnoses.
**Limitations:** The main limitations are small population samples, unknown DLB diagnosis by BrainGML, and no pathological diagnoses.
**Ethics committee approval:** This study was approved by the French South-East Ethics committee.
**Funding for this study:** No funding was received for this study.
**Author Disclosures:**
Felix Renard: Founder: GEODAISICS
Claire Bonardel: Nothing to disclose
Alexandre Krainik: Advisory Board: GEODAISICS
Elodie Piot: Research/Grant Support: GEODAISICS
Ebru Sayilir: Nothing to disclose
Sylvie Grand: Nothing to disclose
Arnaud Attye: Founder: GEODAISICS

### RPS 1511-6

**Is pure akinesia with gait freezing associated with more ventriculomegaly in brain MRI than other subtypes of progressive supranuclear palsy?**

*M. Jiménez Vázquez*, P. Del Nido Recio, M. R. López de la Torre Carretero, C. Mbongo, M. Calvo Imirizaldu, R. Garcia de Eulate, P. Dominguez Echavarri, C. Espinoza-Vinces, M. R. I. Luquin; Pamplona/ES
*(mjimenezvaz@unav.es)*

**Purpose:** This study aimed to evaluate if patients with PAGF have more ventriculomegaly in MR imaging in comparison with other subtypes of PSP. Freezing of gait can be seen in progressive supranuclear palsy (PSP), specifically in the pure akinesia with gait freezing (PAGF) subtype, mimicking Normal Pressure Hydrocephalus (NPH).
**Methods or Backround:** We retrospectively analysed a cohort of 48 patients who underwent brain MRI between 2018 and 2023 due to freezing of gait as the main clinical feature. 40 patients were posteriorly diagnosed with PSP, four with NPH and four with Alzheimer's disease. After excluding the last patients, we divided the PSP patients into a group posteriorly diagnosed with PAGF (27) and another diagnosed with other PSP subtypes (13). Evans Index (EI) and Callosal Angle (CA) were measured in the two groups and cut-off values of EI >0.3 and CA <100º were selected for defining ventriculomegaly. Midbrain-to-pons ratio and frontal and midbrain atrophy were also evaluated. Fisher's Exact Test and T-test were applied to studying proportions and means.
**Results or Findings:** The mean EI was significantly higher in the PSP-PAGF group compared to the other subtypes group (0.32 vs 0.29, p=0.041), while the mean CA was significantly lower (103.69º vs 118.35º, p=0.008). Moreover, CA cut-off <100º showed a significant difference between both groups (p=0.015) with a specificity of 92.31%. EI cut-off >0.3 showed a non-significant sensibility of 74.07% (p=0.155). No significant differences were found in the midbrain-to-pons ratio and frontal and midbrain atrophy between the two groups.
**Conclusion:** Our results illustrate that patients with freezing of gait and ventricular enlargement in imaging will probably have a diagnosis of PSP-PAGF, after excluding NPH. PSP-PAGF is associated with more significant ventriculomegaly than other subtypes of PSP.
**Limitations:** No limitations were identified.
**Ethics committee approval:** The study is retrospective.
**Funding for this study:** No funding was received for this study.
**Author Disclosures:**
Pablo Dominguez Echavarri: Nothing to disclose
Carmen Mbongo: Nothing to disclose
Pablo Del Nido Recio: Nothing to disclose
Manuel Rafael López de la Torre Carretero: Nothing to disclose
Marta Calvo Imirizaldu: Nothing to disclose
Reyes Garcia de Eulate: Nothing to disclose
Marcos Jiménez Vázquez: Nothing to disclose
María Rosario Isabel Luquin: Nothing to disclose
Christian Espinoza-Vinces: Nothing to disclose

### RPS 1511-7

**Early re-emerging tremor after MRgFUS thalamotomy: case-control analysis of procedural and imaging features**

*P. Badini*, C. Santobuono, F. Colarieti, A. Di Gioia, A. Innocenzi, F. Bruno, A. Splendiani, E. Di Cesare, A. Catalucci; L'Aquila/IT
*(pierfrancescobadini@yahoo.it)*

**Purpose:** This study aimed to identify possible prognostic factors determining early tremor relapse after MRgFUS thalamotomy in patients with essential tremor (ET) and Parkinson's disease (PD).

**Methods or Backround:** 9 patients (6 ET, 3PD) who underwent Vim MRgFUS thalamotomy in a single institution and developed early re-emergent tremor were analysed. Demographic and clinical characteristics of patients were compared to a pairwise matched control group of patients with stable tremor relief at the same follow-up period. In both groups, procedural parameters of target and sonications and MR imaging findings (including lesion shape and volume in multiparametric sequences and dentatorubrothalamic tract DTI analysis) were compared.
**Results or Findings:** We did not find statistically significant differences in gender and age between the two groups. Concerning MRI analysis, we found larger thalamotomy lesions in the control group with stable outcomes, compared to patients with tremor relapse. In the tractography evaluation, we found a more frequent eccentric position of the DRTt in patients with tremor relapse.
**Conclusion:** The most relevant determining factors for tremor relapse after MRgFUS thalamotomy appear to be the size of the thalamotomy lesion and inaccurate thalamic targeting.
**Limitations:** Limited study sample and retrospective analysis.
**Ethics committee approval:** All subjects gave their informed consent for inclusion before they participated in the study. The study was conducted in accordance with the Declaration of Helsinki.
**Funding for this study:** No funding was received for this study.
**Author Disclosures:**
Antonio Di Gioia: Nothing to disclose
Alessandra Splendiani: Nothing to disclose
Chiara Santobuono: Nothing to disclose
Filippo Colarieti: Nothing to disclose
Antonio Innocenzi: Nothing to disclose
Ernesto Di Cesare: Nothing to disclose
Alessia Catalucci: Nothing to disclose
Federico Bruno: Nothing to disclose
Pierfrancesco Badini: Nothing to disclose

### RPS 1511-8

**Resting-state brain connectivity in patients with essential tremor undergoing vim ablation with MRgFUS: preliminary results**
*F. Colarieti*, C. Santobuono, P. Badini, A. Di Gioia, A. Innocenzi, A. Catalucci, F. Bruno, E. Di Cesare, A. Splendiani; L'Aquila/IT
*(filippo.colarieti@gmail.com)*

**Purpose:** This study aimed to verify any changes in brain connectivity in patients with essential tremors undergoing MRgFUs thalamotomy. Essential tremor is the result of abnormal communication between certain areas of the brain, including the thalamus, cerebellum, and brainstem. Vim MRgFUS thalamotomy is a new minimally invasive procedure used for the treatment of disabling tremors.
**Methods or Backround:** 15 patients with essential tremor (TE) (mean age= 64.1, SD= 13.1), without cognitive impairments (mean IQ= 109.0, SD= 9.9), were included in the study. These patients' resting-state brain connectivity was recorded before treatment and after six months with 3T MRI. The MRgFUS thalamotomy treatment was effective in all patients, with a mean FTM (Fahn-Tolosa-Marin tremor scale) reduction of the treated upper limb from 5.1 to 0.8. An analysis of ROI-to-ROI connectivity was then carried out with Coon Toolbox studying the mains brain networks to assess the difference in pre- and post-treatment brain connectivity.
**Results or Findings:** The results indicated significantly greater post-treatment connectivity between the anterior cerebellum and the left parietal lobe (default mode network) and between the left occipital cortex (visual network) and the cerebellum.
**Conclusion:** Although preliminary, our results indicate greater connectivity of the cerebellum following MRgFUS treatment, in accordance with better fine motor control reported by patients undergoing surgery.
**Limitations:** This was a low study sample.
**Ethics committee approval:** All subjects gave informed consent for inclusion before participating in the study. The study was conducted in accordance with the Declaration of Helsinki.
**Funding for this study:** No funding was received for this study.
**Author Disclosures:**
Antonio Di Gioia: Nothing to disclose
Alessandra Splendiani: Nothing to disclose
Chiara Santobuono: Nothing to disclose
Filippo Colarieti: Nothing to disclose
Antonio Innocenzi: Nothing to disclose
Ernesto Di Cesare: Nothing to disclose
Alessia Catalucci: Nothing to disclose
Federico Bruno: Nothing to disclose
Pierfrancesco Badini: Nothing to disclose

### RPS 1511-9

**Changes to the peripheral nervous system in chronic spinal cord injury: a study on 3T MR neurography**
*J. M. E. Jende*, L. Heutehaus, F. Preisner, C. Mooshage, R. Rupp, M. Bendzus, N. Weidner, F. Kurz, S. Franz; Heidelberg/DE
*(johann.jende@med.uni-heidelberg.de)*

**Purpose:** This study aimed to combine MRN and clinical assessments in individuals with chronic SCI and non-disabled controls. It is known that structural and functional changes to the peripheral nervous system (PNS) can occur in disorders of the central nervous system (CNS) such as multiple sclerosis. To date, it is unknown to what extent changes at the PNS level occur after spinal cord injury (SCI) and whether such changes are relevant for functional recovery or the development of neuropathic painful symptoms below the level of injury. 3 Tesla Magnetic Resonance Neurography (MRN) allows the detection and quantification of structural and functional damage to peripheral nerves.
**Methods or Backround:** 20 participants with chronic SCI and 20 controls matched for sex, age, and body-mass index underwent t2-weighted and diffusion-weighted MRN of the sciatic nerve. The sciatic nerve's mean cross-sectional area (CSA), fascicular lesion load, and fractional anisotropy (FA) were calculated. Results were correlated with clinical and electrophysiologic assessments.
**Results or Findings:** Sciatic nerve CSA and lesion load were larger (21.29 mm 2±5.82 vs 14.08 mm 2±4.62; $p<0.001$, and 8.70% ±7.47 vs 3.60% ±2.45; $p<0.001$) in individuals with SCI compared to controls, whereas FA was lower (0.55±0.11 vs 0.63±0.08; $p=0.022$). MRN parameters correlated with electrophysiological results but did not correlate with the extent of myelopathy or clinical severity of SCI.
**Conclusion:** Individuals with chronic SCI are subject to a decline of structural peripheral nerve integrity that may occur independently from the clinical severity of SCI. This may have an impact on functional recovery following SCI. The underlying cause of this phenomenon remains yet to be determined since no association with the extent of myelopathy or the severity of SCI-related disability could be found.
**Limitations:** This study is limited by its cross-sectional design and the small number of participants.
**Ethics committee approval:** This study was approved by the ethics committee of Heidelberg University Hospital.
**Funding for this study:** Funding for this study was received by the International Foundation for Research in Paraplegia, The Else Kröner-Fresenius Stiftung and the German Research Foundation.
**Author Disclosures:**
Rüdiger Rupp: Nothing to disclose
Fabian Preisner: Nothing to disclose
Norbert Weidner: Nothing to disclose
Johann Malte Enno Jende: Nothing to disclose
Steffen Franz: Nothing to disclose
Laura Heutehaus: Nothing to disclose
Felix Kurz: Nothing to disclose
Christoph Mooshage: Nothing to disclose
Martin Bendzus: Nothing to disclose

### RPS 1511-10

**AI-based three-dimensional analysis of lumbar vertebrae micromovements in spondylolisthesis patients with weight-bearing MRI**
R. Levi[1], *L. A. Cappellini*[1], M. Battaglia[1], F. Garoli[1], M. De robertis[1], G. Savini[1], M. Fornari[2], M. Grimaldi[2], L. S. S. Politi[2]; [1]Pieve Emanuele/IT, [2]Rozzano/IT
*(lucaalessandro.cappellini@st.hunimed.eu)*

**Purpose:** This study aimed to automatically evaluate the micro-instability of lumbar vertebrae through 3D deep-learning analysis of MRI in supine and standing positions in healthy subjects and patients with spondylolisthesis.
**Methods or Backround:** The current assessment of spondylolisthesis relies on the Meyerding grading system from morphodynamical lateral X-rays and supine MRI. These approaches measure instability only on the sagittal plane providing a partial representation of the disease. We retrospectively included 28 subjects (10 patients with spondylolisthesis and 18 controls) who underwent weight-bearing MRI (wbMRI) with 3D-HYCE acquisitions in supine and upright positions. Deep-learning algorithms were employed to segment vertebrae and dural sac and to quantify advanced micro-instability parameters: centre of mass (COM), variation of the angle passing through the COM, and roto-translation matrix (RTM). These parameters were correlated to pain numerical rating scale (PNRS).

**Results or Findings:** Automatic quantification of vertebral posterior wall listhesis resulted in the upgrading of Meyerding classification from grade I to II in 3 patients (30%). Patients with spondylolisthesis demonstrated significantly reduced lordotic curvature in the upright position compared to controls showing reduced anterior movement of COM (p=0.0105) and narrower anterior variation of the angle (p<0.001). The analysis of RTM reported higher rotation in controls for anteroposterior direction at L2-L3 level (p=0.038) and cranio-caudal direction at L3-L4 level (p=0.006). In patients, pre-operative PNRS evaluation of back pain was of 8.50 (std. 0.93) with a mean post-operative PNRS of 4.00 (std. 2.51). Translation in the anteroposterior movement was positively associated with pre-operative PNRS (R=0.701, p=0.042). Post-operative PNRS was positively associated with the latero-lateral movement (R=0.699, p=0.045).
**Conclusion:** This preliminary study highlights the clinical potentials of wbMRI and deep-learning 3D analysis for spondylolisthesis micro-movements assessment, offering a comprehensive insight into vertebral instability, peri-procedural pain, and indication to neurosurgical treatment.
**Limitations:** This study had a limited population.
**Ethics committee approval:** No information provided by the submitter.
**Funding for this study:** No funding was received for this study.
**Author Disclosures:**
Mario De robertis: Nothing to disclose
Letterio Salvatore Salvatore Politi: Nothing to disclose
Luca Alessandro Cappellini: Nothing to disclose
Giovanni Savini: Nothing to disclose
Federico Garoli: Nothing to disclose
Marco Grimaldi: Nothing to disclose
Riccardo Levi: Research/Grant Support: R.L. received funding for PhD scholarship by Esaote
Maurizio Fornari: Nothing to disclose
Massimiliano Battaglia: Nothing to disclose

### RPS 1511-11

**Neurite orientation dispersion and density imaging quantifies microstructural impairment in the thalamus and its connectivity in amyotrophic lateral sclerosis**
*Y-b. Cao*, H-J. Chen; Fuzhou/CN
*(caoyb77@163.com)*

**Purpose:** This study aimed to evaluate microstructural impairment in the thalamus and thalamocortical connectivity using neurite orientation dispersion and density imaging (NODDI) in amyotrophic lateral sclerosis (ALS).
**Methods or Backround:** This study included 47 healthy controls and 43 ALS patients, whose structural and diffusion-weighted data were collected. We used state-of-the-art parallel transport tractography to identify thalamocortical pathways in individual spaces. The thalamus was then parcellated into six subregions based on its connectivity pattern with the priori defined cortical (i.e., prefrontal/motor/somatosensory/temporal/posterior-parietal/occipital) regions. For each of the thalamic and cortical subregions and thalamocortical tracts, we compared the following NODDI metrics between groups: orientation dispersion index (ODI), neurite density index (NDI), and isotropic volume fraction (ISO). We also used these metrics to conduct receiver operating characteristic curve (ROC) analyses and Spearman correlation.
**Results or Findings:** In ALS patients, we found decreased ODI and increased ISO in the thalamic subregion connecting the left motor cortex and other extramotor (e.g., somatosensory and occipital) cortex (Bonferroni-corrected P <0.05). NDI decreased in bilateral thalamo-motor and thalamo-somatosensory tracts and in the right thalamo-posterior-parietal and thalamo-occipital tracts (Bonferroni-corrected P <0.05). NDI reduction in the bilateral thalamo-motor tract (P= 0.017 and 0.009) and left thalamo-somatosensory tract (P= 0.029) was correlated with disease severity. In thalamocortical tracts, NDI yielded a higher effect size during between-group comparisons and a greater area under ROC (P <0.05) compared with conventional diffusion tensor imaging metrics.
**Conclusion:** Microstructural impairment in the thalamus and thalamocortical connectivity is the hallmark of ALS. NODDI improved the detection of disrupted thalamocortical connectivity in ALS.
**Limitations:** Firstly, the study had a relatively small sample size. Secondly, the study design was cross-sectional. Thirdly, the pattern of connectivity between other subcortical areas and the cortex should also be evaluated in future ALS studies.
**Ethics committee approval:** This study was approved by the Research Ethics Committee of Fujian Medical University Union Hospital
**Funding for this study:** This study was supported by grants from the Graduate Student Academic Exchange Fund of Fujian Medical University.
**Author Disclosures:**
Hua-Jun Chen: Nothing to disclose
Yun-bin Cao: Nothing to disclose

**14:00-15:30** **Research Stage 3**

### Research Presentation Session: Genitourinary

### RPS 1507

### Prostate imaging and cancer management

**Moderator**
V. Logager; Copenhagen/DK
*(vibeke.loegager@regionh.dk)*

### RPS 1507-2

**Clinically significant prostate cancer detection of MRI in-bore biopsy after negative targeted plus systematic MRI/US fusions-guided biopsy**
*M. Boschheidgen*, M. Quentin, J. P. Radtke, T. Ullrich, L. R. Drewes, B. Valentin, P. Albers, G. Antoch, L. Schimmöller; Düsseldorf/DE
*(matthias.boschheidgen@med.uni-duesseldorf.de)*

**Purpose:** This study aimed to discuss the challenges of patients with suspicion of clinically significant prostate cancer (csPC) on multiparametric prostate MRI (mpMRI), but negative or inconclusive MRI/US fusion-guided biopsy (FB) in clinical practice; and to evaluate the utility of MRI in-bore biopsy (IB) in patients with discordant imaging and histopathologic results following FB.
**Methods or Backround:** Consecutive patients who underwent IB after FB between 01/2014 and 05/2022 with Prostate Imaging Reporting and Data System (PI-RADS) category 4 or 5 lesions on mpMRI at 3T, without histologically confirmed csPC, were included. The primary objective was the detection rate of csPC. Secondary objectives were to analyze these cases regarding clinical parameters, MRI parameters, and lesion localization.
**Results or Findings:** In the final cohort of 51 patients, the overall PC detection rate with IB was 71% and 47% for csPC. IB resulted in a Gleason score upgrade in 55% of cases with initial low-grade PC. The detected csPC was most commonly located apical and/or anterior. The detection rate of PC in PI-RADS category 4 was 58% and 94% in PI-RADS 5 (csPC 39% and 61%, respectively). Patients with csPC showed significantly smaller prostate volumes, a higher PI-RADS category, a higher prostate-specific antigen density (PSAD), and older age.
**Conclusion:** In a relevant proportion of patients with PI-RADS 4 or 5 but negative or inconclusive findings on previous FB and persistent suspicion of csPC, a csPC could be verified by subsequent IB. Therefore, IB might serve as a backup solution in cases of uncertainty.
**Limitations:** The limitations of this study were that it was a single-centre and retrospective design.
**Ethics committee approval:** This study was approved by the Ethikkommission Universität Düsseldorf.
**Funding for this study:** No funding was received for this study.
**Author Disclosures:**
Lukas Richard Drewes: Nothing to disclose
Birte Valentin: Nothing to disclose
Tim Ullrich: Nothing to disclose
Jan Philipp Radtke: Nothing to disclose
Peter Albers: Nothing to disclose
Matthias Boschheidgen: Nothing to disclose
Lars Schimmöller: Nothing to disclose
Michael Quentin: Nothing to disclose
Gerald Antoch: Nothing to disclose

### RPS 1507-3

**Magnetic resonance imaging characteristics and oncological follow-up of patients with high grade prostate cancer**
*M. Boschheidgen*, L. R. Drewes, B. Valentin, J. P. Radtke, P. Albers, G. Antoch, L. Schimmöller; Düsseldorf/DE
*(matthias.boschheidgen@med.uni-duesseldorf.de)*

**Purpose:** This study aimed to analyse multiparametric MRI characteristics of patients with ISUP 4 or 5 prostate cancer (PC) on biopsy and to investigate the correlation with biochemical recurrence (BCR) after radical prostatectomy (RPE).
**Methods or Backround:** In this single-centre cohort study, consecutive patients with mpMRI and ISUP 4 or 5 PC at the time of biopsy and/or RPE were retrospectively analysed. Clinical, MR-guided biopsy and mpMRI parameters were assessed. A subcohort of patients with RPE and follow-up was analysed separately. A univariate and multivariate analysis was performed to determine parameters which enable the identification of patients facing an elevated risk of BCR after RPE.

**Results or Findings:** 145 patients (mean age 70 years, median PSA 10.9 ng/ml) were analysed. 99% had PI-RADS score of 4 or 5, 48% revealed MRI T3 stage and median tumour diameter was 15mm. Cancerous areas showed a median ADC value of 668 ×10−3 mm2/s and exhibited contrast enhancement in 94% of the cases. For patients with and without BCR after RPE (n=82), MRI parameters were different for contact length to pseudocapsule, MRI cT3 stage, and localization of the index lesion (p<0.05). PSAD and MRI cT3 stage were independent parameters for the risk of BCR when incorporating clinical, biopsy, and MRI parameters.
**Conclusion:** ISUP 4 or 5 PC had distinctive MRI characteristics and were detected on MRI in all included cases. PSAD and cT3 stage on MRI are significant predictors for BCR after RPE. These results may help clinicians, to identify patients at higher risk for recurrence at follow-up. MRI has the ability to identify high-risk prostate cancer and may play a role in clinical risk stratification and prediction of biochemical recurrence after radical prostatectomy.
**Limitations:** The limitations of this study were that it was a single-centre, retrospective design.
**Ethics committee approval:** This study was approved by the Ethikkommission der Universität Düsseldorf.
**Funding for this study:** No funding was received for this study.
**Author Disclosures:**
Lukas Richard Drewes: Nothing to disclose
Birte Valentin: Nothing to disclose
Jan Philipp Radtke: Nothing to disclose
Peter Albers: Nothing to disclose
Matthias Boschheidgen: Nothing to disclose
Lars Schimmöller: Nothing to disclose
Gerald Antoch: Nothing to disclose

### RPS 1507-4

### Five-year outcomes after transurethral ultrasound ablation (TULSA) of localised prostate cancer

*J. Fütterer*[1], D. Bonekamp[2], T. Persigehl[3], S. Arora[4], S. Raman[5], K. J. Macura[6], A. Oto[7], T. Tirkes[8], D. Costa[9]; [1]Nijmegen/NL, [2]Heidelberg/DE, [3]Cologne/DE, [4]New Haven, CT/US, [5]Los Angeles, CA/US, [6]Baltimore, MD/US, [7]Chicago, IL/US, [8]Indianapolis, IN/US, [9]Dallas, TX/US
*(jurgen.futterer@radboudumc.nl)*

**Purpose:** This study aimed to discuss the magnetic resonance imaging-guided transurethral ultrasound ablation (TULSA) of the prostate using ultrasound to thermally coagulate tissue in-bore. Closed-loop control matches real-time feedback from MRI thermometry to the ablative energy administered to the prescribed volume. We report the 5-year follow-up from the TACT pivotal study.
**Methods or Backround:** 115 men with localised prostate cancer (PCa) were enrolled across 13 sites in 5 countries. Eligibility included Grade Group (GG) 1-2, stage≤T2b, and PSA≤15 ng/mL. Men received a single, whole-gland TULSA treatment sparing the prostatic urethra and urinary sphincter. Primary endpoints at 1 year were PSA reduction and adverse events. Secondary endpoints included 10-core biopsy and mpMRI at 1 year and adverse events, quality of life, PSA, and salvage treatment to 5 years.
**Results or Findings:** At baseline, (median [IQR]) age was 65 (59-69) years. By 5 years, PSA decreased from 6.3 (4.6-7.9) ng/mL pre-treatment to 0.63 (0.18-1.9) ng/mL (n=68), and 25 men (21.7%) received salvage treatment, without unexpected complications (10 prostatectomy, 11 radiotherapy, 3 ADT, 1 surgery and radiation). Median prostate volume at baseline and 1 year was 37.3 and 2.8 cc (92% decrease). At baseline and 1 year, 72/115 (63%) and 17/111 (15%) had ≥GG2 disease. By 5 years, 61/66 (92%) recovered pad-free continence; 80/92 (87%) preserved IIEFQ2≥2 erections. 12 men incurred Grade 3 adverse events (10%), with no Grade≥4 event or rectal injury. Intraprocedural imaging parameters, positive MRI and rising PSA at 1 year predicted the risk of salvage therapy by 5 years.
**Conclusion:** Effective disease control was achieved in 78% of men at 5 years after a single TULSA procedure. Favourable safety and functional outcomes were durable to 5 years.
**Limitations:** Modern protocols may translate into improved rates of local control in contemporary cases. A randomized controlled trial comparing TULSA with radical prostatectomy is underway (NCT05027477).
**Ethics committee approval:** No information was provided by the submitter.
**Funding for this study:** The funding for this study was received from Profound Medical.
**Author Disclosures:**
Jurgen Fütterer: Investigator: Profound Medical
David Bonekamp: Investigator: Profound Medical
Steven Raman: Investigator: Profound Medical
Aytekin Oto: Advisory Board: Profound Medical; Investigator: Profound Medical
Sandeep Arora: Investigator: Profound Medical
Katarzyna J. Macura: Investigator: Profound Medical
Daniel Costa: Speaker: Profound Medical; Investigator: Profound Medical
Thorsten Persigehl: Investigator: Profound Medical
Temel Tirkes: Investigator: Profound Medical

### RPS 1507-5

### Expect the unexpected: unveiling discrepancies in prostate cancer diagnosis with an MRI-pathology discordance analysis

*A. Stanzione*[1], K-L. Lee[2], N. Sanmugalingam[2], I. Rajendran[2], N. Sushentsev[2], I. Caglič[2], T. Barrett[2]; [1]Naples/IT, [2]Cambridge/UK
*(arnaldostanzione@yahoo.it)*

**Purpose:** This study aimed to investigate the underlying explanations for inconsistent results between prostate MRI and biopsy outcomes in biopsy-naïve patients. Additionally, it seeks to shed light on diagnostic errors and potential enhancements in the prostate cancer (PCa) diagnostic process.
**Methods or Backround:** A retrospective analysis was conducted on 2780 biopsy-naïve patients undergoing prostate MRI at a tertiary referral centre from October 2015 to June 2022. Patients exhibiting discordance between MRI and biopsy results were categorized into two groups: MRI-negative/Biopsy-positive and MRI-positive/Biopsy-negative (biopsy-positive defined as Gleason score ≥ 3+4). An expert uroradiologist reviewed cases with discrepancies, reassessing PI-RADS scores retrospectively, identifying previously unreported MRI targets, and evaluating MRI scan quality (MRI-negative/Biopsy-positive group). Categories for discrepancies included MRI overcalls (including known pitfalls), benign pathology findings, and errors in biopsy targeting.
**Results or Findings:** Patients who did not undergo biopsy (n=1258), with indeterminate MRI findings (PI-RADS 3, n=204), or with clinically insignificant PCa (Gleason score 3+3, n=216) were excluded. Of the remaining patients, 32/1102 (3%) were classified as MRI-negative/Biopsy-positive and 117/1102 (11%) as MRI-positive/Biopsy-negative. In the MRI-negative/Biopsy-positive group, 44% of the scans were deemed non-diagnostic quality. Upon retrospective image review, target lesions were identified in 28% of cases. In the MRI-positive/Biopsy-negative group, 42% were attributed to MRI overcalls, while 32% had benign pathology findings explaining the observed abnormalities on MRI. Biopsy targeting errors accounted for 11% of cases.
**Conclusion:** Prostate MRI demonstrated a high diagnostic accuracy, with low occurrences of discrepant findings. To further minimize these, it is essential to ensure optimal MRI quality and expert image assessment. Common reasons for MRI-positive/Biopsy-negative cases included benign pathology findings and MRI overcalls.
**Limitations:** The limitations of this study were that it was of retrospective design and single-centre.
**Ethics committee approval:** This study was approved by the Local IRB.
**Funding for this study:** No funding was received for this study.
**Author Disclosures:**
Nimalan Sanmugalingam: Nothing to disclose
Iztok Caglič: Nothing to disclose
Nikita Sushentsev: Nothing to disclose
Kang-Lung Lee: Nothing to disclose
Arnaldo Stanzione: Board Member: European Radiology
Tristan Barrett: Nothing to disclose
Ishwariya Rajendran: Nothing to disclose

### RPS 1507-6

### Integrated diagnostics for the detection of prostate cancer through the integration of data derived from a breath analysis device, MRI, and molecular analysis: a proof-of-concept study

*M. Pecoraro*, S. Novelli, E. Messina, L. Laschena, C. Catalano, V. Panebianco; Rome/IT
*(pecoraro.martina1@gmail.com)*

**Purpose:** This study aimed to deliver novel, cost-effective, evidence-based, non-invasive predictive risk-adapted diagnostic pathways for faster, earlier, more precise, accessible, and affordable detection and screening of prostate cancer.
**Methods or Backround:** A prospective single-centre cohort study, enrolling patients with PCa suspicion who underwent MRI, MRI-directed fusion biopsy (MRDB), was conducted. Patients also underwent breath analysis using a non-invasive device and molecular analysis (mir-302a-5p and 367). A network-based analysis was used to identify biomarkers drivers of clinically significant PCa.
**Results or Findings:** Overall, 46 men were enrolled and underwent MR for clinical suspicion of prostate cancer; during the day of the scan or the day of the biopsy procedure, patients also underwent breath analysis and plasma sampling for microRNA expression analysis. A total of 38 patients represented the entire cohort: 12 (31.5%) were negative for PCa, 8 (21.0%) had grade group (GG) 1 PCa, and 18 (47.3%) had GG >1 PCa. The proposed additional non-invasive analysis, including clinical data, MRI biomarkers, breath sensing and microRNAs, provided a higher net benefit with a biopsy avoidance rate of about 23% at a low disease probability.
**Conclusion:** The integration of multimodal data, including results from medical devices, imaging and omics can further improve the diagnostic pathway of patients with clinical suspicion of prostate cancer.
**Limitations:** The main limitation is the small sample cohort.
**Ethics committee approval:** No information was provided by submitter.

**Funding for this study:** This study was supported by the Italian National Complementary Plan - D3-4-HEALTH project - funded by the Next Generation EU.
**Author Disclosures:**
Valeria Panebianco: Nothing to disclose
Emanuele Messina: Nothing to disclose
Simone Novelli: Nothing to disclose
Martina Pecoraro: Nothing to disclose
Ludovica Laschena: Nothing to disclose
Carlo Catalano: Nothing to disclose

### RPS 1507-7

**Surveillance of one-year post-focal cryotherapy for clinically significant prostate cancer using mpMRI and PIRADS v2.1: an initial experience from a prospective phase II mandatory biopsy study**

*J. Velaga*, K. J. Tay, G. Hang, Y. G. Tan, N. Lath, J. S. Yuen, M. Chua, N. T. Ngo, Y. M. Law; Singapore/SG

**Purpose:** This study aimed to discuss the challenges of multiparametric magnetic resonance imaging (mpMRI) surveillance post-focal cryotherapy (FT) of prostate cancer as post-treatment artefacts alter mpMRI findings. In this initial experience, we assessed the diagnostic performance of mpMRI in detecting clinically significant prostate cancer (csPCa) after FT.
**Methods or Backround:** This single-centre phase II prospective clinical trial recruited 28 men with localized csPCa for FT between October 2019 and April 2021. 12-month post-FT mpMRI was performed prior to biopsy and sensitivity, specificity, positive predictive value (PPV) and negative predictive value (NPV) of all mpMRI-positive subjects were analysed. Chi square goodness of fit test correlated biopsy-positive PIRADS3 (P3) and PIRADS4/5 lesions with histology grade group. One-way ANOVA test assessed the performance of ADC values in differentiating csPCa, non-csPCa and benign lesions.
**Results or Findings:** Sensitivity, specificity, PPV and NPV of mpMRI were 100%, 14.28%, 53.84% and 100% for subjects with histologically proven cancer. Correlation of PIRADS v2.1 scores with histologically proven prostate cancer was statistically significant (p<0.5). Correlation of P3 lesions with non-csPCa was statistically significant (p<0.02535). Higher ADC value was associated with benign histology (adjusted odds ratio OR 0.97, 95% confidence interval: 0.94, 0.99) (p=0.008). Among the malignant lesions, higher ADC value was associated with non-csPCa (adjusted OR: 0.97; 95% CI: 0.95, 0.99) (p=0.032).
**Conclusion:** mpMRI is highly sensitive in detecting residual cancer. ADC values and PIRADS scores may be of value in differentiating csPCa from non-csPCa with a potential for risk stratification of men requiring re-biopsy versus non-invasive surveillance of remnant prostate.
**Limitations:** The limitation of the study was the small sample size.
**Ethics committee approval:** This study received IRB approval (SingHealth 2018/2482)
**Funding for this study:** Funding was received from the National Medical Research Council, Singapore.
**Author Disclosures:**
Kae Jack Tay: Grant Recipient: Dr KJ Tay received funding support from National Medical Research Council, Singapore. Grant number - NCT04138914
Narayan Lath: Nothing to disclose
John S.P. Yuen: Nothing to disclose
Jyothirmayi Velaga: Nothing to disclose
Yu Guang Tan: Nothing to disclose
Guanqi Hang: Nothing to disclose
Nye Thane Ngo: Nothing to disclose
Melvin Chua: Nothing to disclose
Yan Mee Law: Nothing to disclose

### RPS 1507-8

**Diagnostic performance of PSMA-PET/CT and multiparametric MRI assessed using the PI-RR scoring system for the detection of prostate cancer local recurrence and lymph node metastases**

*L. Laschena*, A. Dehghanpour, E. Messina, M. Bicchetti, C. Catalano, V. Panebianco; Rome/IT
*(ludovica.laschena@uniroma1.it)*

**Purpose:** This study aimed to discuss MRI and PSMA-PET/CT recommendations for patients at risk of developing prostate cancer (PCa) recurrence. Recently the Prostate Imaging for Recurrence Reporting (PI-RR) system has been proposed to standardize MRI acquisition, interpretation, and reporting in case of PCa local recurrence suspicion after therapies with curative intent. The study aimed (1) to determine the diagnostic yield and clinical value of PET/CT and MRI using PI-RR, and (2) to stratify results according to patient's pre/post-operative risk.
**Methods or Backround:** Retrospective multi-center study; 119 patients with clinical suspicion of PCa recurrence underwent MRI and PET/CT in close succession. Exclusion criteria included lack of sufficient follow-up data, pelvic radiation therapy (previous 6 months), and history of hormone therapy. PI-RR score was assigned independently by two experienced uroradiologists. PET/CT images were reviewed by an expert nuclear medicine physician. Reference-standard was defined using histopathologic findings, follow-up imaging or clinical response to treatment. Sensitivity, specificity, PPV, NPV, and accuracy were calculated. The intraclass-correlation coefficient was used to determine inter-reader agreement.
**Results or Findings:** MRI resulted positive (PI-RR ≥3) in 69 cases, PET/CT in 48 cases. We found a statistically significant difference in terms of rate of PCa recurrence between MRI and PET/CT (p=0.006). MRI (PI-RR ≥3) and PET-CT reveal respectively: sensitivity of 86% and 71%; specificity of 78% and 52%, PPV of 74% and 51%; NPV of 89% and 73%; accuracy of 81% and 60% for PCa recurrence detection. At multivariate analysis, only age (0.002) and PI-RR ≥ 3 (0.001) independently correlate with recurrence.
**Conclusion:** MRI proved its strong diagnostic performance in detecting PCa recurrence. Therefore, low-risk patients should be directed to MRI to rule out local recurrence; high-risk patients should be investigated using PET/CT to rule in local and distant recurrence.
**Limitations:** The limitations of this study were the high-volume tertiary referral centre and limited reproducibility.
**Ethics committee approval:** This study was approved by the Institutional Ethics Committee.
**Funding for this study:** No funding was received for this study.
**Author Disclosures:**
Marco Bicchetti: Nothing to disclose
Valeria Panebianco: Nothing to disclose
Emanuele Messina: Nothing to disclose
Ailin Dehghanpour: Nothing to disclose
Ludovica Laschena: Nothing to disclose
Carlo Catalano: Nothing to disclose

### RPS 1507-9

**Effect of preoperative PI-RADS assessment on pathological outcomes in patients who underwent radical prostatectomy**

Q. Peng, L. Xu, G. Zhang, J. Zhang, X. Zhang, X. Bai, *L. Chen*, Z. Jin, H. Sun; Beijing/CN
*(li.cherish@outlook.com)*

**Purpose:** This study aimed to assess the effect of preoperative MRI with standardized Prostate Imaging–Reporting and Data System (PI-RADS) assessment on pathological outcomes in prostate cancer (PCa) patients who underwent radical prostatectomy (RP).
**Methods or Backround:** This cohort study included patients who had undergone prostate MRI and subsequent RP for PCa between 2017 and 2022. The patients were divided into a PI-RADS group and a non-PI-RADS group. The pathological outcomes included pathological T stage (pT2 vs. pT3–4) and positive surgical margins (PSMs). Patients were further stratified according to statistically significant preoperative variables to assess the difference in pathological outcomes. A propensity score matching based on the above preoperative characteristics was additionally performed.
**Results or Findings:** A total of 380 patients were included, with 201 patients in the PI-RADS group and 179 in the non-PI-RADS group. The two groups had similar preoperative characteristics, except for clinical T stage (cT). As for pathological outcomes, the PI-RADS group showed a significantly lower percentage of pT3–4 (21.4% vs. 48.0%, p < 0.001), a lower percentage of PSMs (31.3% vs. 40.9%, p = 0.055), and a higher concordance between the cT and pT (79.1% vs. 64.8%, p = 0.003). The PI-RADS group also showed a lower proportion of pT3–4 (p < 0.001) in the cT1–2 subgroup and the cohort after propensity score matching. The PSM rate of cT3 patients was reduced by 39.2% in the PI-RADS group but without statistical significance (p = 0.089).
**Conclusion:** Preoperative MRI with standardized PI-RADS assessment could reduce the proportion of patients with non-organ-confined PCa undergoing PR and slightly reduce the PSM rate compared with non-PI-RADS assessment.
**Limitations:** This is a single-centre and retrospective cohort study, and our analysis lacks complete insight into the surgical protocol decisions of patients.
**Ethics committee approval:** This study was approved by the Institutional Review Board (IRB number I-22PJ1031).
**Funding for this study:** This work is supported by grants from the National High-Level Hospital Clinical Research Funding (Grant No. 2022-PUMCH-A-033, 2022-PUMCH-A-035, and 2022-PUMCH-B-069), the CAMS Innovation Fund for Medical Sciences (CIFMS) (Grant No. 2022-I2M-C&T-B-019), and the 2021 Key Clinical Specialty Program of Beijing.
**Author Disclosures:**
Jiahui Zhang: Nothing to disclose
Gumuyang Zhang: Nothing to disclose
Hao Sun: Nothing to disclose
Lili Xu: Nothing to disclose
Xin Bai: Nothing to disclose
Li Chen: Nothing to disclose
Qianyu Peng: Nothing to disclose
Zhengyu Jin: Nothing to disclose
Xiaoxiao Zhang: Nothing to disclose

### RPS 1507-10

**ProstaPilot: prostate cancer screening by magnetic resonance imaging with a biparametric protocol, preliminary cohort analysis after the first screening round**

*M. Standara*, M. Stanik, J. Kristek, O. Majek, M. Uher; Brno/CZ
*(standara@mou.cz)*

**Purpose:** This study aimed to validate the accuracy of biparametric magnetic resonance (bpMRI) prostate protocol in a screening population and its potential benefits in the screening of clinically significant prostate cancer (csPC). While PSA screening can reduce prostate cancer mortality, the risk of overdiagnosis and overtreatment of non-significant prostate cancer is a concern.
**Methods or Backround:** The ProstaPilot prospective cohort study enrolled 422 men aged 50-69 years between May 2022 and May 2023 who had not undergone PSA or prostate MRI in the past two years. If a patient had a PSA elevation ≥ 3 ng/ml, they were advised to undergo a systematic biopsy. If a patient had abnormal findings on bpMRI (PI-RADS 4-5 lesion), they underwent systematic and MRI/US targeted biopsy. The bpMRI was performed with a 15-minute PI-RADS compliant non-contrast protocol, and csPC was defined as ISUP grade ≥ 2 prostate cancer. The data underwent statistical analyses.
**Results or Findings:** In the first screening round, a positive MRI result was recorded in 17 (4.0%) men, and a positive PSA test result was found in 25 (5.9%) men, of whom seven (1.7%) had positive both MRI and PSA tests. Prostate biopsy was recommended to 35 study participants (8.3%) based on one or both positive tests. Of these, three men refused biopsy, and in the MRI-positive group, eight (1.9%) csPC and seven (1.7%) ISUP1 carcinomas were detected, while in the PSA-positive group, four (0.9%) and four (0.9%) carcinomas detected, respectively.
**Conclusion:** More clinically significant cancers were detected in the MRI-positive group than in the PSA-positive group. Due to limited capacity, MRI cannot be performed on all screened men. Further studies are needed to determine the optimal PSA level at which men should undergo bpMRI.
**Limitations:** No limitations were identified.
**Ethics committee approval:** The study was approved by Masaryk Memorial Cancer Institute Ethics committee: Ref. Nr. 2021/1715/MOU.
**Funding for this study:** Funding for this study was provided by the Ministry of Health of the Czech Republic: grant nr. NU22-09-00539 and Ministry of Health, Czech Republic – conceptual development of research organization (MOÚ, 00209805).
**Author Disclosures:**
Michal Stanik: Nothing to disclose
Michal Uher: Nothing to disclose
Michal Standara: Nothing to disclose
Jan Kristek: Nothing to disclose
Ondrej Majek: Nothing to disclose

### RPS 1507-11

**Reduction of total perfusion of prostatic adenoma following prostate artery embolisation for benign prostatic hyperplasia (BPH)**

*A. Dehghanpour*, S. Cipollari, E. Messina, L. Laschena, C. Catalano, V. Panebianco; Rome/IT
*(ad48ad@yahoo.com)*

**Purpose:** This study aimed to assess changes in total prostate and central gland volumes and changes in perfusion following prostate artery embolisation in patients with symptomatic benign prostatic hyperplasia (BPH).
**Methods or Backround:** In this prospective study, we included patients with symptomatic BPH refractory to medical therapy with no evidence or previous history of prostate cancer. Patients underwent multiparametric prostate MRI (mpMRI) prior to endovascular prostate artery embolisation; a follow-up mpMRI was performed 4 weeks after the procedure. Total prostate and central gland volumes were measured on both the baseline and follow-up mpMRI. A quantitative map of the Area Under the Curve (AUC), which represents the integral of the contrast agent concentration over time, was generated using the OleaSphere software from the perfusion-weighted images. The mean AUC value for all voxels within the central gland was then calculated. Embolisation procedures were performed through a femoral trans-arterial access, a microcatheter was used to catheterize prostate arteries bilaterally, and embolisation was achieved using non-reabsorbable microparticles (300-500 um), following contrast-enhanced cone-beam CT to confirm the correct positioning of the microcatheter.
**Results or Findings:** 27 patients were included in the study. Baseline total gland volume was 62 cc, central gland volume was 37, mean AUC of central gland perfusion was 31819.28. Technical procedural success was obtained in 25/27 patients (92%). Non-major or minor complications were reported following the procedures. A significant reduction of BPH symptoms was achieved in 22/27 patients (81%). There was a significant reduction in total prostate and central gland volumes (16.2%, p=0.021) and mean AUC perfusion values (14%, p=0.008).
**Conclusion:** Prostate artery embolisation is a safe and effective procedure for the treatment of symptomatic BPH. Central gland volume and perfusion were significantly reduced at follow-up mpMRI compared to baseline.
**Limitations:** The limitation of this study was the limited sample size.
**Ethics committee approval:** The study was approved by the Institutional Ethical Committee.
**Funding for this study:** No funding was received for this study.
**Author Disclosures:**
Valeria Panebianco: Nothing to disclose
Stefano Cipollari: Nothing to disclose
Emanuele Messina: Nothing to disclose
Ailin Dehghanpour: Nothing to disclose
Ludovica Laschena: Nothing to disclose
Carlo Catalano: Nothing to disclose

### RPS 1507-12

**Oncological and functional outcomes following MicroUS-guided focal laser ablation of localised prostate cancer: comparison of single and multi-laser fibers settings**

*F. Cornud*, A. Lefevre, M. Galiano; Paris/FR

**Purpose:** This study aimed to evaluate the safety, feasibility, and short-term functional and oncological outcomes of focal laser ablation (FLA) of tumours at low risk of progression, under micro-ultrasound guidance.
**Methods or Backround:** 58 PI-RADS>2 lesions were treated in 55 patients between July 2020 and June 2023 with a follow-up of 12 months for 29 patients. The mean age was 69±7.4 years. The mean PSA level was 7.9±3.5 ng/ml. The large tumour axis was ≤20mm. Gleason score was 6 in 10 lesions (10/58,17.2%) and 3+4 in 43 lesions (43/58,74.5%). The first 21 (21/58,36%) and the last 37 lesions (37/58,64%) were treated with a single and multifiber mode, respectively, using the Echolaser® system operating at 1064nm. Image fusion was used to cover the MRI-delineated target volume. A prostato-rectal hydrodissection was performed for posteromedial tumours. DCE-MRI was performed 4 days after FLA to evaluate the ablated volume.
**Results or Findings:** MicroUS visualised 53 tumours (53/58,91.4%). The multifiber mode achieved larger ablation volumes (19.1±11.8 vs 5.9±3.5cc, p=0.0001). A prostate-rectal fistula occurred postoperatively in a patient with a posteromedial lesion, which was treated surgically. Urinary and erectile functions were not affected by FLA. At 12 months follow-up of 34 tumours in 29 patients, in-field targeted biopsies (TB) diagnosed 13 recurrences (13/34,38.2%, Gleason pattern 4 in 8/13 tumours, 61.5%). Ten (10/13,77%) occurred with a single fibre setting. Four out-field recurrences (4/34,11.7%) were diagnosed. After retreatment of 6 recurrences, TB showed, at 12 months, an 80% negative TB-biopsy rate.
**Conclusion:** Multifiber microUS-guided FLA is a safe and feasible modality with promising short-term oncological and functional outcomes. Caution is required in posteromedial lesions.
**Limitations:** Long-term results are required to validate our findings
**Ethics committee approval:** This study was approved after written informed consent was obtained from the patients. This single-centre study was approved by our Institutional Review Board.
**Funding for this study:** The authors or the institution did not receive any funding to conduct this study
**Author Disclosures:**
Arnaud Lefevre: Nothing to disclose
François Cornud: Nothing to disclose
Marc Galiano: Nothing to disclose

### RPS 1507-13

**The accuracy of the MRI pathway in the detection of index prostate cancer lesion: a single-centre experience**

*S. Durmaz*, B. Coşkun, M. Kılıc, S. Madendere, T. Esen, M. Vural; Istanbul/TR
*(drselahattindurmaz@gmail.com)*

**Purpose:** This study aimed to examine the accuracy of the MRI pathway in detecting index prostate cancer (PCa) lesions in biopsy-naive men.
**Methods or Backround:** A retrospective review of 379 biopsy-naive men who underwent multiparametric MRI and subsequent in-bore prostate biopsy for the high-likelihood target (PI-RADS 4-5) was conducted. We identified 110 men with PCa who underwent radical prostatectomy as a definitive treatment and included them in this study. An analysis of the results from multiparametric MRI, in-bore needle biopsy, and whole-mount step-section specimens was conducted to determine the accuracy of the MRI pathway in detecting index lesions, which are defined as lesions with a higher GG and a larger volume. We also evaluated the results of in-bore prostate biopsy and final pathology for GG group compliance.
**Results or Findings:** In total, 122 high-likelihood targets (PI-RADS 4: 85 targets, PI-RADS 5: 37 targets) were biopsied. Whole-mount specimen analysis revealed multifocal involvement in 75 (68,2%) patients. A total of 83 non-biopsied tumour foci were identified at final pathology and more than half (44/83) of these foci were harbouring ISUP GG 1 tumour. Except for one patient, the index PCa focus was successfully detected and sampled by the MRI pathway approach. At final pathology, upgrade and downgrade rates were 27,3% (30/110), and 10% (11/110) respectively. The remaining 69 (62,7%)

patients had concordant ISUP GG with in-bore biopsy pathology. The majority of the upgraded group was composed of ISUP GG1 patients (20/30).
**Conclusion:** According to our findings, the MRI pathway is a safe and accurate approach in biopsy-naive men with high-likelihood multiparametric MRI findings.
**Limitations:** The limitations of this study included: It's retrospective design. Being a single-institution study with an experienced uroradiologist and uropathologist, a factor, which may limit its generalizability. Selection bias due to patient recruitment over 11 years.
**Ethics committee approval:** This study was approved by our Institutional Review Board and the requirement for informed written consent was waived.
**Funding for this study:** No funding was received for this study.
**Author Disclosures:**
Tarik Esen: Nothing to disclose
Metin Vural: Nothing to disclose
Bilgen Coşkun: Nothing to disclose
Selahattin Durmaz: Nothing to disclose
Mert Kılıc: Nothing to disclose
Serdar Madendere: Nothing to disclose

**14:00-15:30** **Research Stage 4**

### Research Presentation Session: Interventional Radiology

### RPS 1509

### Advanced imaging and challenging interventions in the liver

**Moderator**
A. Gjoreski; Skopje/MK
*(girizmelel@gmail.com)*
**Author Disclosures:**
Aleksandar Gjoreski: Consultant: Terumo Europe, Boston Scientific; Investigator: CIEMAR

### RPS 1509-2

**Trans-splenic antegrade coil-assisted transvenous occlusion (TACATO) of gastric varices associated with gastro-renal shunt in cirrhosis: a single-centre prospective preliminary study**
*S. Groff*, G. Cicognini, G. Barbiero, M. Battistel, S. Shalaby, M. Senzolo, G. De Conti; Padova/IT
*(stefano.groff@hotmail.com)*

**Purpose:** This study aimed to report the technical success and clinical efficacy of the sole antegrade trans-splenic approach for occlusion of gastric varices (GVs) associated with gastro-renal shunt (GRS) in cirrhotics.
**Methods or Backround:** All patients who bled from GVs associated with GRS without severe ascites or large oesophagal varices were selected for evaluation. From February 2020 to July 2023, 29 patients were evaluated and 19 were included for treatment as secondary prophylaxis and prospectively followed-up (mean time of 15.3 months; range 3-43 months). Splenoportography from trans-splenic access classified venous afferents to the GVs (Saad-Caldwell classification), which were selectively catheterized and embolized with coils+/-N-butyl-2-cyanoacrylate+methacryloxysulfolane and ethiodized oil (NBCA+LUF). Final splenoportography assessed occlusion of GVs (technical success). The trans-splenic tract was sealed with NBCA+LUF. Access size, number-size of microcoils, use of NBCA+LUF and fluoroscopy time were recorded. Postprocedural ultrasound and haemoglobin levels evaluated bleeding complications. Follow-up included clinical evaluation, contrast-enhanced CT (CECT) and esophagogastroduodenoscopy (EGD).
**Results or Findings:** Splenoportography identified Saad-Caldwell type 1b GVs in two cases, type 2b in ten cases and type 3b in seven cases. Different introducer sheath and microcoils sizes were used, with a mean number of 8.4 microcoils per patient (range 1-20). Microcoils alone were used in four patients and with NBCA+LUF in 15 patients. Mean fluoroscopy time was 20.8 min (range 7.1-43.1 min). Final splenoportography documented total occlusion of GVs in 15 patients (79%) and partial occlusion in four patients (21%). No bleeding complications. Clinical follow-up documented stable liver function, no re-bleeding from GVs nor development/worsening of ascites. CECT showed three cases of splenic/portal vein thrombosis. Follow-up EGD revealed the disappearance/reduction of GVs in all patients.
**Conclusion:** TACATO seems safe and effective for treating GVs associated with GRS and should be further evaluated in the algorithm of treatment.
**Limitations:** Preliminary study: larger number of patients required.
**Ethics committee approval:** This study was approved by the Padua University Hospital (HIC protocol #0034435)
**Funding for this study:** No funding was received for this study.
**Author Disclosures:**
Giorgio De Conti: Nothing to disclose
Stefano Groff: Nothing to disclose
Michele Battistel: Nothing to disclose
Giulio Barbiero: Nothing to disclose
Marco Senzolo: Nothing to disclose
Giancarlo Cicognini: Nothing to disclose
Sarah Shalaby: Nothing to disclose

### RPS 1509-3

**Factors impacting survival after transarterial radioembolisation in patients with intrahepatic cholangiocarcinoma: a combined analysis of the prospective CIRT and CIRT-FR studies**
*P. Reimer*[1], V. Vilgrain[2], D. Arnold[3], R. Loffroy[4], B. Sangro[5], M. Urdaniz[6], H. Pereira[7], N. de Jong[6], T. K. Helmberger[8]; [1]Karlsruhe/DE, [2]Clichy/FR, [3]Hambrug/DE, [4]Dijon/FR, [5]Pamplona/ES, [6]Vienna/AT, [7]Paris/FR, [8]Munich/DE
*(peter.reimer@partner.kit.edu)*

**Purpose:** This study aimed to discuss the treatment option of Transarterial radioembolization (TARE) with Yttrium-90 resin microspheres for patients with intrahepatic cholangiocarcinoma (ICC). Optimizing the timing of TARE in relation to systemic therapies and patient selection remains challenging. We report here on the effectiveness, safety, and prognostic factors associated with TARE for ICC in an analysis of the prospective observational CIRT (CIRSE Registry for SIR-Spheres Therapy) studies (NCT02305459 and NCT03256994).
**Methods or Backround:** A separate analysis of all 174 ICC patients within CIRT studies was performed. Patient characteristics and treatment-related data were collected at baseline; time-to-event data (overall survival [OS], progression-free survival [PFS] and hepatic PFS) were collected at every follow-up visit. Log-rank tests and a multivariable Cox proportional hazard model were used to identify prognostic factors.
**Results or Findings:** Patients receiving a first-line strategy of TARE, in addition to any systemic treatment, had a median OS and PFS of 32.5 months and 11.3 months. Patients selected for first-line TARE alone showed a median OS and PFS of 16.2 months and 7.4 months, whereas TARE as 2nd or further treatment-line resulted in a median OS and PFS of 12 and 9.3 months (p=0.0028), and 5.1 and 3.5 months (p=0.0012), respectively. Partition model dosimetry was an independent predictor for better OS (HR 0.59 [95% CI 0.37-0.94], p=0.0259). No extrahepatic disease, no ascites, and <6.1 months from diagnosis to treatment were independent predictors for longer PFS.
**Conclusion:** This combined analysis indicates that in unresectable ICC, TARE in combination with any systemic treatment is a promising treatment option in the first line. Partition model dosimetry improved the effectiveness of TARE.
**Limitations:** Patients and physicians were not blinded within the prospective CIRSE Registry for SIR-Spheres therapy reflecting the real-world treatment situation.
**Ethics committee approval:** This study was approved by applicable regulatory bodies and/or ethics committees in Germany, Switzerland, Turkey, Italy, Spain, Israel, France, and Belgium as applicable for observational studies.
**Funding for this study:** This study was funded by an unconditional research grant from Sirtex Medical Europe, GmbH.
**Author Disclosures:**
Romaric Loffroy: Nothing to disclose
Helena Pereira: Nothing to disclose
Dirk Arnold: Nothing to disclose
Bruno Sangro: Nothing to disclose
Maria Urdaniz: Nothing to disclose
Peter Reimer: Nothing to disclose
Niels de Jong: Nothing to disclose
Thomas Karl Helmberger: Nothing to disclose
Valérie Vilgrain: Nothing to disclose

### RPS 1509-4

**Simultaneous portal and hepatic vein embolisation in inducing future liver remnant hypertrophy: the impact of steatosis in hepatic regeneration**
*D. Santangelo*, D. Palumbo, M. Platì, C. Canevari, F. Ratti, L. Aldrighetti, F. De Cobelli; Milan/IT
*(santangelo.domenico@hsr.it)*

**Purpose:** This study aimed to assess the impact of preoperative LS on complications after major liver surgery and to identify any eventual liver steatosis modification pattern after liver venous deprivation (LVD). Liver steatosis (LS) has been widely associated with post-surgical complications in different surgery fields.

**Methods or Backround:** Patients who underwent, between 01/2019 and 12/2022, LVD were identified (n = 40). Those who ultimately underwent surgery (n = 27, 67.5%) were enrolled. LS was defined as mean liver density lower than 50 HU. Pre/post-procedural CT and scintigraphic data were collected. Data regarding post-surgical complications, in particular, post-hepatectomy liver failure (PHLF) were collected.
**Results or Findings:** Of the 27 patients undergoing surgery, 12 presented with LS at a preoperative CT scan. This subgroup demonstrated a higher chance of developing post-surgical complications ($p < 0.05$). Non-steatotic patients showed, after LVD, a rapid decrease of residual liver HU values (-5.60 HU, +/- 4.91). On the contrary, patients with baseline steatosis did not experience significant changes ($p < 0.001$). Those who developed post-LVD steatosis (n = 7) did not show an increased risk of developing post-surgical complications when compared to those who remained in their steatosis group. Patients who developed post-LVD steatosis demonstrate a lower functional degree of hypertrophy ($p < 0.05$).
**Conclusion:** This proof-of-concept study suggests two conclusions: (1) Pre-LVD LS is a risk factor for the development of post-surgical complications, and (2) LVD seems to function as a trigger for a modification of residual liver density; such “induced” post-LVD steatosis differs from the metabolic one both in terms of lower post-surgical complications rate and impaired function of the liver remnant. We are possibly looking at two different histological entities brought together by a similar radiological appearance.
**Limitations:** The limitations of this study are that it was retrospective and it was a small population.
**Ethics committee approval:** This study was approved by Leatum 64/INT/2021.
**Funding for this study:** No funding was received for this study.
**Author Disclosures:**
Luca Aldrighetti: Nothing to disclose
Diego Palumbo: Nothing to disclose
Marianna Platì: Nothing to disclose
Francesco De Cobelli: Nothing to disclose
Carla Canevari: Nothing to disclose
Francesca Ratti: Nothing to disclose
Domenico Santangelo: Nothing to disclose

### RPS 1509-5

### CT-guided high-dose-rate brachytherapy induces systemic proteins of proliferation and angiogenesis predicting outcome in HCC

*M. M. R. Stechele*[1], S. N. Goldberg[2], M. Wildgruber[1], P. M. Kazmierczak[1], L. Salvermoser[1], M. Alunni-Fabbroni[1], J. Ricke[1]; [1]Munich/DE, [2]Jerusalem/IL

**Purpose:** This study aimed to assess the potential prognostic value of proliferation and angiogenesis markers following CT-guided high-dose-rate brachytherapy (HDR-BT) of hepatocellular carcinoma (HCC).
**Methods or Backround:** For this prospective study, HDR-BT (1x15Gy) was performed in 24 HCC patients. Plasma was obtained at baseline and 48 hours post-HDR-BT and analysed using an Olink proteomics Target-96 immuno-oncology-panel, including multiple markers of angiogenesis and proliferation. Protein fold-change (FC) ratios were calculated. Patients without progression within 6m or systemic progression within two years were classified as responders (R; n=12). Patients with recurrence within 6m and/or tumour progression or extrahepatic disease within two years were classified as non-responders (NR; n=12).
**Results or Findings:** Angiopoietin-1 (median R: 0.86; NR: 1.29; p= 0.008) and PDGF-B (R: 0.89; NR: 1.25; p= 0.033) were significantly elevated in non-responders compared to responders. FC between responders and non-responders did not differ significantly for Angiopoietin-2, VEGF-A, and VEGFR-2. FC of EGF (R: 0.67; NR: 1.51; p= 0.028) was significantly elevated in non-responders compared to responders with no significant difference for FGF-2, HGF, and PGF. Kaplan-Meier analyses demonstrated a significantly shorter time to systemic progression for increased Angiopoietin-1 and EGF (p= 0.011 and 0.019 respectively), but not for remaining proteins (all p >0.1). Pooled analysis for all 9 proteins showed significantly shorter systemic progression for FC ≥ 1.3 in ≥ 3 proteins (p= 0.022).
**Conclusion:** Increased plasma levels of Angiopoietin-1 and EGF after HDR-BT for HCC are associated with poor response and may therefore function as predictive biomarkers of outcome.
**Limitations:** Given a small cohort, further expansion of protein panels and evaluation of potential dynamic changes of target proteins over time are needed to achieve optimal biomarker potential.
**Ethics committee approval:** In this monocentric clinical study, we analysed prospectively from acquired data of 24 patients from the “ESTIMATE” patient cohort (Studiennummer: DRKS00010587, Deutsches Register Klinischer Studien). Ethical approval was provided by the ethics committee Ethikkommission bei der LMU München: (reference number “17-346”) on June 20, 2017, and August 26, 2020.
**Funding for this study:** No funding was received for this study.
**Author Disclosures:**
Marianna Alunni-Fabbroni: Nothing to disclose
S. Nahum Goldberg: Nothing to disclose
Philipp M. Kazmierczak: Nothing to disclose
Moritz Wildgruber: Nothing to disclose
Lukas Salvermoser: Nothing to disclose
Matthias Max Rudolf Stechele: Nothing to disclose
Jens Ricke: Nothing to disclose

### RPS 1509-6

### Transjugular intrahepatic porto-systemic shunt in paediatric patients: comparison between standard approach and alternative techniques

*P. Marra*[1], R. Muglia[1], N. Liggeri[1], C. Sallemi[2], L. Dulcetta[1], F. S. Carbone[1], S. Sironi[1]; [1]Bergamo/IT, [2]Brescia/IT
*(pmarra@asst-pg23.it)*

**Purpose:** This study aimed to compare technical success, safety and clinical outcomes of TIPS performed with conventional technique or assisted by percutaneous approach in paediatric patients and difficult anatomies.
**Methods or Backround:** From January 2019, paediatric patients or young adults with native liver or split liver grafts undergoing TIPS were retrospectively reviewed. Group A underwent TIPS through a standard transjugular approach. Group B underwent standard DIPS or "gun-sight" TIPS assisted by transhepatic and transsplenic approaches. Technical success in terms of correct TIPS placement, safety in terms of complications and clinical outcomes in terms of bleeding and ascites control were assessed and compared between groups.
**Results or Findings:** 16 patients underwent TIPS placement due to portal hypertension and variceal bleeding (n=7), portal vein thrombosis (n=6), Budd-Chiari syndrome (n=1) or refractory ascites (n=1). Out of six patients with portal vein thrombosis, four were affected by chronic portal vein thrombosis and cavernous transformation, and failed percutaneous portal vein recanalization or surgical meso-rex shunt. In two cases, TIPS was a bridge to liver transplant. In group A, eight patients (n=3 with regular anatomy; n=3 with cavernoma; n=2 with Budd-Chiari) successfully underwent TIPS with septic shock in one case. In group B, eight patients (n=4 with regular anatomy; n=4 with cavernoma; none with Budd-Chiari) successfully received TIPS with severe bleeding and precipitating liver failure in one case. No technical failures were recorded. Clinical outcome was good in all patients.
**Conclusion:** Alternative techniques for TIPS placement have a great success rate with good clinical outcomes. In paediatric patients and difficult anatomies DIPS and "gun-sight" TIPS might be considered.
**Limitations:** Small sample and potential selection bias.
**Ethics committee approval:** The study Portal_01 was approved by the Ethical Committee of Bergamo (reg.92-21).
**Funding for this study:** No funding was received for this study.
**Author Disclosures:**
Sandro Sironi: Nothing to disclose
Paolo Marra: Nothing to disclose
Francesco Saverio Carbone: Nothing to disclose
Noemi Liggeri: Nothing to disclose
Claudio Sallemi: Nothing to disclose
Riccardo Muglia: Nothing to disclose
Ludovico Dulcetta: Nothing to disclose

### RPS 1509-7

### Feasibility, safety and mid-term outcomes of non-expandable biodegradable biliary stents in paediatric liver transplants

*P. Marra*, R. Muglia, F. S. Carbone, L. Dulcetta, S. Sironi; Bergamo/IT
*(pmarra@asst-pg23.it)*

**Purpose:** This study aimed to report the mid-term outcomes of percutaneous biliary drainage with double biodegradable stent placement for the treatment of benign anastomotic biliary strictures in paediatric liver transplants (PLT).
**Methods or Backround:** From March 2022, consecutive patients with PLT who underwent percutaneous transhepatic cholangiography (PTC), bilioplasty and placement of two side-by-side 10F helical-shaped (non-expandable) biodegradable (slow degradation profile of 11 weeks) biliary stents were prospectively enrolled. Feasibility of double stent placement, safety in terms of procedure-related complications, freedom from stricture recurrence at imaging and absence of symptoms/signs of cholestasis were assessed.
**Results or Findings:** Percutaneous transhepatic stent placement was performed in 19 patients (10 females; median age 7 years, range 3-11 years) for the treatment of perianastomotic strictures (18 hepatico-jejunostomy; 1 duct-to-duct anastomosis). Stenting was performed after indwelling of trans-anastomotic biliary drainage for a median time of 28 days (range 11.5-53 days). Double stent placement in a side-by-side fashion was technically successful in 15/19 (79%) patients; in four patients a single trans-anastomotic stent was implanted. Stent fragmentation/migration before the expected degradation time was observed in 4/19 (21%) patients. One patient was readmitted with cholangitis and conservatively treated; no other complications occurred. Stricture recurrence was observed during a median follow-up of 233.5 days (range 182.5-263 days) in 6/19 (32%) patients who required new PTC procedures.

**Conclusion:** Preliminary data suggests that the placement of biodegradable biliary stents may mitigate concerns related to the maintenance of external biliary drainage in PLT patients affected by benign perianastomotic biliary strictures. However, the long-term efficacy of non-expandable stents is limited and needs to be compared with that of expandable devices in the PLT population.
**Limitations:** This study had a limited sample and lacked case-control analysis.
**Ethics committee approval:** This study was approved by Reg. Sperim. N. 061/23
**Funding for this study:** No funding was received for this study.
**Author Disclosures:**
Sandro Sironi: Nothing to disclose
Paolo Marra: Nothing to disclose
Francesco Saverio Carbone: Nothing to disclose
Riccardo Muglia: Nothing to disclose
Ludovico Dulcetta: Nothing to disclose

### RPS 1509-8

**Role of different stent types in endoluminal IRE for recanalization of an occluded metal stent: mathematical model of temperature distribution**
*P. Matkulcik*, M. Hemzal, T. Andrasina, T. Rohan, M. Straka; Brno/CZ
*(p.matkulcik@gmail.com)*

**Purpose:** This study aimed to analyse the temperature distribution of endoluminal IRE for stent recanalization using a mathematical model in exact stent models.
**Methods or Backround:** Endoluminal IRE for recanalization of different uncovered metal stents (Ella-CS, Boston Scientific, S&G Biotech and Micro-Tech medical) using a 3-electrode IRE catheter was simulated in a mathematical model of perfused liver tissue. The obstruction of the metal stent was simulated by 1, 2 and 3 mm layers of interposed tissue. 2D and 3D calculations by finite element software (COMSOL Multiphysics) defined the extent of thermal distribution zones in different IRE protocols (100 100 µs pulses, 1 Hz at 300 V, 650 V, 1000 V,1300 V). Differences in temperatures at the electrode, on the stent and 1 mm outside of the stent depending on stent type, voltage and width of interposed tissue were investigated.
**Results or Findings:** The biggest differences - up to 21% - were observed with 1 mm interposed tissue at voltages set to 1000 V at the electrode and at the stent (52.606-64.433 °C and 52.643-63.687 °C respectively). The highest temperatures were observed in simulations with Ella-CS stent, on the other hand, the lowest temperatures were observed in simulations with Microtech stent across all simulations. At voltages set to 1300 V, temperatures at the electrode and at the stent with 1 mm interposed tissue were 72.146-83.073 °C and 72,556-81,921 °C respectively, thus IRE in those scenarios was producing also thermal effects of ablation.
**Conclusion:** In the mathematical model, substantial differences in temperatures were observed between the specific stents analysed. In the highest voltages with 1 mm interposed tissue, thermal effects of ablation were present.
**Limitations:** This was a computer simulation.
**Ethics committee approval:** Ethics committee approval was not necessary in this study.
**Funding for this study:** This study was funded by the Ministry of Health, Czech Republic: grant No.: NU21-08-00561.
**Author Disclosures:**
Martin Hemzal: Nothing to disclose
Tomas Andrasina: Nothing to disclose
Tomas Rohan: Nothing to disclose
Matej Straka: Nothing to disclose
Peter Matkulcik: Nothing to disclose

### RPS 1509-9

**Multiparametric MRI-based radiomics nomogram predicts the recurrence of hepatocellular carcinoma after postoperative adjuvant transarterial arterial chemoembolisation (PA-TACE)**
*X. Guo*, L. Zheng, J. Ji; Lishui/CN
*(1848382471@qq.com)*

**Purpose:** This study aimed to develop and validate a multiparametric MRI-based radiomics model for predicting the recurrence in hepatocellular carcinoma (HCC) patients after postoperative adjuvant transcatheter arterial chemoembolization (PA-TACE).
**Methods or Backround:** In this retrospective study, 117 HCC patients (81 for training and 36 for validation) treated with PA-TACE were included. Multiparametric radiomics features were extracted from T2-weighted imaging (T2WI), diffusion-weighted imaging (DWI) (b=800), and hepatic arterial phase (AP). Least absolute shrinkage and selection operator (LASSO)-COX regression was utilised to select radiomics features and calculate the radiomics score (Rad-score). Optimal clinical characteristics selected by univariate and multivariate Cox analysis were integrated with Rad-score to develop a recurrence-free survival (RFS) prediction model.
**Results or Findings:** 15 radiomics features were selected from three sequences of MRI. The significant difference in RFS between the high-risk and low-risk signature groups based on the Rad-score median value of 0.434 was confirmed in two cohorts ($P < 0.05$). The clinical independent predictors were neutrophil to lymphocyte ratio (NLR) (HR= 1.49, 95% CI: 1.1-2.1, P= 0.022) and tumour size (HR= 1.28, 95% CI: 1.1-1.5, P= 0.001). The clinical-radiomics model developed by Rad-score, NLR, and tumour size demonstrated favourable performance for predicting recurrence. The AUCs at 1-, 3- and 5-year time-dependent ROC curves based on the combined model were 0.82-0.91, and 0.75-0.91 in the two cohorts respectively.
**Conclusion:** The multiparametric MRI-based radiomics nomogram can well predict recurrence in HCC patients treated with PA-TACE and can serve as a valuable tool for clinical prognosis.
**Limitations:** Firstly, the study was retrospective. Secondly, all participants of this study were diagnosed with hepatitis B virus (HBV)-related HCC in China. Thirdly, our study did not explore tissue-related biomarkers such as specific gene mutations and signatures.
**Ethics committee approval:** This study was approved by the Institutional Review Board and Human Ethics Committee of Lishui Hospital of Zhejiang University. The requirement for informed consent was waived.
**Funding for this study:** Funding for this study was received from the National Natural Science Foundation of China (82102162 and 82072026), Natural Science Foundation of Zhejiang Province (LGF21H180002) and the Zhejiang Medical and Health Science Project (2022RC087).
**Author Disclosures:**
Jiansong Ji: Nothing to disclose
Liyun Zheng: Nothing to disclose
Xinyu Guo: Nothing to disclose

### RPS 1509-10

**The challenge of percutaneous recanalisation of chronic portal vein thrombosis: from the lack of evidence to clinical experience**
L. Dulcetta, *P. Marra*, F. S. Carbone, R. Muglia, S. Sironi; Bergamo/IT
*(pmarra@asst-pg23.it)*

**Purpose:** This study aimed to report the experience of a tertiary referral centre for pediatric and adult liver disease and transplantation in the IR management of chronic complete PVT. Complete chronic portal vein thrombosis (PVT) is a condition often requiring treatment to avoid potentially serious complications.
**Methods or Backround:** A retrospective search was performed to identify pediatric and adult patients with an imaging diagnosis of chronic complete PVT and with clinical signs of severe portal hypertension who underwent successful percutaneous portal vein recanalization (PVR) from January 2020 to October 2023. Clinical and imaging follow-up was analysed.
**Results or Findings:** 12 patients (median age=18,5 years, IQ=41 years; 9 males) with chronic complete PVT who underwent at least one attempt of IR treatment from January 2020 to October 2023 were retrieved. Six patients were paediatrics and 7 patients (58%) were liver transplant recipients. All patients had chronic occlusion of the main portal vein, either with (n=2) or without (n=10) occlusion of the superior mesenteric and/or the splenic veins. Cavernomatous transformation was noted in 11/12 (92%) patients. At the time of treatment, all patients presented clinical signs of portal hypertension. A total of 20 percutaneous transhepatic, transsplenic and/or transjugular procedures were performed. Overall technical success, defined as patency of the portal vein after at least one procedure, was achieved in all patients. After a median follow-up of 12 months, clinical success in terms of the absence of clinical signs of severe portal hypertension was achieved in all patients. No major complications occurred.
**Conclusion:** PVR is a feasible, safe, effective and minimally invasive procedure with excellent technical and clinical success for the management of chronic complete PVT in both pediatric and adult patients.
**Limitations:** No limitations were identified.
**Ethics committee approval:** No information provided by submitter.
**Funding for this study:** No funding was received for this study.
**Author Disclosures:**
Sandro Sironi: Nothing to disclose
Paolo Marra: Nothing to disclose
Francesco Saverio Carbone: Nothing to disclose
Riccardo Muglia: Nothing to disclose
Ludovico Dulcetta: Nothing to disclose

### RPS 1509-11

**Improved identification of good candidates for the treatment of intermediate/advanced hepatocellular carcinoma by Yttrium-90 transarterial radioembolisation**
*I. Oreistein*, C. Vitellius, C. Aubé, F. Oberti, J. Boursier, A. Bouvier, F. Lacoeuille, L. Vervueren, S. Poulard; Angers/FR
*(ioreistein@wanadoo.fr)*

**Purpose:** This study aimed to validate the prognostic score for HCC treated with TARE recently proposed by Spreafico and to improve prediction by considering also pre-operative dosimetry. Yttrium-90 transarterial radioembolisation is a treatment for intermediate/advanced hepatocellular carcinoma, but its position in the therapeutic arsenal remains poorly defined.
**Methods or Backround:** 86 patients with HCC treated by SIRT in our centre were included. The provisional tumoral dose of 90Yttrium was calculated during the work-up. The Spreafico prognostic score was calculated as previously described and delineated three patient groups with "favourable", "intermediate", and "dismal" prognosis. The main study outcome was overall survival and the secondary outcome was progression-free survival.
**Results or Findings:** Fifty-three patients (62%) were treated with Therasphere®, and 33 patients (38%) were treated with Sirsphere®. Median OS was 12.0 months (95% CI: 9.0-15.0), and median PFS was 5.0 months (95% CI: 3.5-6.5). OS was 15, 10 and 4 months in the three prognostic groups defined by the Spreafico score ($p < 0.001$). Independent predictors of OS were the presence of cirrhosis, an optimal provisional tumoral dose, and the ALBI grade. The CODAG score developed as the sum of points attributed to these three independent predictors, identified three patient groups: good (0-1 point), intermediate (2 points) and poor (3-4 points) candidates. The CODAG score better discriminated the prognosis with median OS in the three groups being respectively 32, 11, and 4 months ($p < 0.001$). Median PFS was respectively 8, 5 and 3 months in the three CODAG groups ($p < 0.001$). The provisional and the received tumoral doses were very well correlated (Rs=0.814, $p < 0.001$).
**Conclusion:** The CODAG score improves the identification of good candidates for the treatment of intermediate/advanced hepatocellular carcinoma with transarterial radioembolisation.
**Limitations:** This study needs to be validated with an external cohort. The CODAG score better discriminated the prognosis than the Spreafico score.
**Ethics committee approval:** The data collection is in agreement with the ethics committee.
Retrospective collection data in ANGERS.
**Funding for this study:** No information was provided by the submitter.
**Author Disclosures:**
Carole Vitellius: Nothing to disclose
Laurent Vervueren: Nothing to disclose
Frederic Oberti: Nothing to disclose
Jerome Boursier: Nothing to disclose
Inès Oreistein: Nothing to disclose
Christophe Aubé: Nothing to disclose
Séverine Poulard: Nothing to disclose
Antoine Bouvier: Nothing to disclose
Franck Lacoeuille: Nothing to disclose

### RPS 1509-12

**AI-derived body composition parameters as prognostic factors in patients with HCC undergoing TACE: results from a multicentre study**
*L. Müller*[1], T. A. Auer[2], J. Haubold[3], F. Nensa[3], M. Eisenblaetter[4], V. Steinle[5], D. Pinto Dos Santos[6], R. Klöckner[7]; [1]Mainz/DE, [2]Berlin/DE, [3]Essen/DE, [4]Freiburg/DE, [5]Heidelberg/DE, [6]Frankfurt/Köln/DE, [7]Lübeck/DE

**Purpose:** This study aimed to investigate the role of BCA parameters for prognosis prediction in patients with HCC undergoing transarterial chemoembolization (TACE). Body composition assessment (BCA) parameters have recently been identified as relevant prognostic factors for patients with hepatocellular carcinoma (HCC).
**Methods or Backround:** This retrospective multicentre study included a total of 754 treatment-naïve patients with HCC who underwent TACE at six tertiary care centres between 2010–2020. Fully automated artificial intelligence-based quantitative 3D volumetry of abdominal cavity tissue composition was performed to assess skeletal muscle volume (SM), total adipose tissue (TAT), intra- and intermuscular adipose tissue (IMAT), visceral adipose tissue (VAT), and subcutaneous adipose tissue (SAT) on pre-intervention computed tomography scans. BCA parameters were normalized to the slice number of the abdominal cavity. We assessed the influence of BCA parameters on median overall survival (OS) and performed multivariate analysis including established estimates of survival.
**Results or Findings:** Univariate survival analysis revealed that impaired median OS was predicted by low SM volume ($p < 0.001$), high TAT volume ($p = 0.013$), and high SAT volume ($p = 0.006$). In multivariate survival analysis, SM remained an independent prognostic factor ($p = 0.039$), while TAT and SAT volumes no longer showed predictive ability.
**Conclusion:** Skeletal muscle volume is an independent prognostic factor for survival prediction. Thus, the integration of SM into novel scoring systems could potentially improve survival prediction and clinical decision-making. Fully automated approaches are needed to foster the implementation of this imaging biomarker into daily routine.
**Limitations:** This study used retrospective data analysis and images were acquired using different scanner types.
**Ethics committee approval:** This study protocol was approved by the Ethics Committee of the Medical Association of Rhineland-Palatinate, Germany (permit number 15913).
**Funding for this study:** No funding was received for this study.
**Author Disclosures:**
Daniel Pinto Dos Santos: Nothing to disclose
Roman Klöckner: Nothing to disclose
Verena Steinle: Nothing to disclose
Johannes Haubold: Nothing to disclose
Michel Eisenblaetter: Nothing to disclose
Timo Alexander Auer: Nothing to disclose
Lukas Müller: Nothing to disclose
Felix Nensa: Nothing to disclose

### RPS 1509-13

**TARE of "multipedicular" malignant liver tumours: can we redistribute the blood flow?**
*R. Fruzza*[1], E. Bozzi[1], G. Lorenzoni[1], R. Cervelli[1], I. Bargellini[2], L. Crocetti[1], R. Cioni[1], E. Neri[1]; [1]Pisa/IT, [2]Candiolo/IT
*(rachelefruzza@gmail.com)*

**Purpose:** This study aimed to demonstrate that "multipedicular" liver tumours (MPLT, i.e. tumours with more than one feeding artery) can be treated by reducing the number of injection points through redistribution of flow, achieving complete coverage and response in the target lesion.
**Methods or Backround:** We retrospectively analysed our database of patients submitted to TARE between 2018 to 2022 and selected patients with MPLT. Diagnostic work-up before radioembolisation was evaluated, and all tumours with an embolised intra-hepatic feeding artery were identified. Careful evaluation of perfusion patterns was performed by SPECT-CT imaging 1 h after 99mTc-MAA injection and a PET-CT scan was performed after Y90 treatment. Tumour response of MPLT was evaluated after 40 days and after 3 months by CT using mRECIST criteria for primary tumours and RECIST 1.1 for liver metastases.
**Results or Findings:** We performed 230 cases of TARE. In 25 patients, accessory branch supplies were embolised using microcoils. Cross-perfusion in the embolised territory was evidenced by SPECT-CT in 22 cases and by PET-CT after Y90 injection in the other 3 cases. At 40 days follow-up we observed: 18 PR, 2 SD, 3 PD, and 2 patients were lost at follow-up. At 3 months follow-up: 10 PR, 1 CR, 4 PD, and 10 patients were lost to follow-up. No complications were reported after embolisation and 90Y administration.
**Conclusion:** Flow redistribution after embolisation of the accessory branches in MPLT was visible and achieved in all cases. Our results confirmed that intratumoral flow redistribution after embolisation, in terms of toxicity, median administered dose and radiological response, is safe and effective.
**Limitations:** No limitations were identified.
**Ethics committee approval:** No information provided by the submitter.
**Funding for this study:** No funding was provided for this study.
**Author Disclosures:**
Elena Bozzi: Nothing to disclose
Irene Bargellini: Nothing to disclose
Emanuele Neri: Nothing to disclose
Rachele Fruzza: Nothing to disclose
Rosa Cervelli: Nothing to disclose
Roberto Cioni: Nothing to disclose
Giulia Lorenzoni: Nothing to disclose
Laura Crocetti: Nothing to disclose

**16:00-17:30 Open Forum Radiographers**

### Research Presentation Session: Radiographers

### RPS 1614

### Current insights and future horizons

**Moderators**
L. Popovic; Belgrade/RS
*(ljubomirpopovic1991@gmail.com)*
G. Ligabue; Sassuolo/IT
*(gligabue@sirm.org)*

### RPS 1614-3

**Green radiology: a pilot study for a sustainable radiology project**
*A. Masperi*[1], F. Pala[2]; [1]Milan/IT, [2]Legnano/IT
*(andrea.masperi1992@gmail.com)*

**Purpose:** The purpose of this study was to estimate the energy impact of the radiology department, implement sustainable solutions in a testing period and evaluate the effects.

**Methods or Backround:** The project followed the energy diagnosis phases proposed by the UNI CEI/TR 11428 certification in a period between January and June 2023. A retrospective analysis mapped the activities and energy consumption of the functional categories involved imaging functional categories (IFC) and complementay functional categories (CFC) and 5 IFCs and 4 CFCs were defined as inefficient respectively. Four interventions were proposed such as optimisation of consumption, reallocation of diagnostic activities, awareness-raising in a green imaging protocol and staff training and a energy scorecard was drawn up. The proposed actions were deployed and the activities, energy consumption of critical IFCs and CFCs, and implementation of the green imaging protocol were monitored.
**Results or Findings:** A reduction in total energy consumption of 8% was achieved for IFCs, and 31.2% for CFCs against an increase in average diagnostic activity of 11.8%. 41/156 patients (26.2%) were retrospectively evaluated with the green imaging protocol, recording a potential reduction in energy consumption of 378.1 kW/patient (77.3%).
**Conclusion:** It is possible to identify with a fair degree of accuracy inefficient processes and functional devices that do not comply with the new guidelines proposed by the European Community for reducing energy consumption. Including radiographers in the drafting of energy dossiers offers the possibility of studying sustainable solutions that lead to concrete results, not only in terms of energy and money savings but also in terms of quality assistance.
**Limitations:** We only considered electricity consumption and not energy consumption from other sources. The main limitations in terms of green protocol are attributable to the evaluation of simulated patients, within a limited period, limited to the abdominal study.
**Ethics committee approval:** No information provided by the submitter.
**Funding for this study:** No funding was received for this study.
**Author Disclosures:**
Fabio Pala: Nothing to disclose
Andrea Masperi: Nothing to disclose

### RPS 1614-4

**Caring for tomorrow: sustainable practices in medical imaging departments implemented by radiographers and policy makers**
*S. S. Ghotra*, M. Champendal, L. Flaction, C. S. D. Reis; Lausanne/CH
*(switindersingh.ghotra@hesav.ch)*

**Purpose:** Global warming is one of the main public health concerns of our time. The purpose of this study was to identify salient approaches that can reduce the environmental impact of medical imaging departments/(MID).
**Methods or Backround:** A review was conducted following JBI methodology on PubMed, Embase and CINAHL to include studies published after 2013 (French, English). Combinations of keywords and MeSH terms related to environmental sustainability, recycling, medical waste and greening were applied. Three independent reviewers screened abstracts, titles and full text. Disagreements were solved through consensus.
**Results or Findings:** 4630 studies were identified; 38 articles met all criteria. Most of the studies were related to developed countries (32/38) and 6/38 were from non-developed countries. A third of the studies included were published after 2022. Articles focused on computed tomography (9/38), magnetic resonance imaging (6/38), interventional radiology (4/38), conventional radiography (4/38), ultrasound (2/38), mixed modalities (9/38). Seven main categories to reduce environmental impact were identified: 1) examination justification, 2) energy consumption, 3) waste production, 4) recycling opportunities, 5) local resources usage, 6) environmental pollution and 7) education. The study indicates the salience of sustainability analysis within quality assurance programmes.
**Conclusion:** To reduce the environmental impact of MID it is important to educate healthcare professionals and to justify adequately examinations, to control energy consumption and to improve health outcomes. Further studies need to be conducted to identify strategies that are most effective, supporting the decision making of managers and MI professionals.
**Limitations:** No experimental studies were conducted to identify the most cost-effective strategies.
**Ethics committee approval:** No information provided by the submitter.
**Funding for this study:** No funding was received for this study.
**Author Disclosures:**
Claudia Sa Dos Reis: Nothing to disclose
Laurence Flaction: Nothing to disclose
Switinder Singh Ghotra: Nothing to disclose
Mélanie Champendal: Nothing to disclose

### RPS 1614-5

**Burnout and job satisfaction among radiographers internationally**
E. Kennedy, A. England, N. Moore, R. Young, *M. F. McEntee*; Cork/IE
*(mark.mcentee@ucc.ie)*

**Purpose:** Burnout and low job satisfaction in healthcare can impact patient safety and staff retention. This study aims to gain information on the factors influencing the levels of burnout and job satisfaction among radiographers internationally, which can inform strategies for improving workforce supply and demand imbalance.
**Methods or Backround:** An online questionnaire was developed that included demographic questions and two validated instruments, The Maslach Burnout Inventory (MBI) and the Job Satisfaction Survey (JSS). Statistical analysis was performed using IBM SPSS. The questionnaire was distributed to state registered diagnostic radiographers through the EFRS Research Hub in 2023 and online through twitter, facebook and e-mail over a six-week period.
**Results or Findings:** 247 participants completed the questionnaire and 207 (84.5%) were female. The questionnaire had participants from 21 countries, with 66.5% being from Ireland. The mean values for EE (20), DP (11) and PA (36) indicate moderate levels of burnout among responding radiographers. 44.2% of radiographers were dissatisfied, 43.7% were ambivalent and only 12.1% had overall job satisfaction. Workload, work being underappreciated, and time pressures were ranked as the top three factors contributing to burnout. Staff numbers, workload and poor management, were ranked as the top three factors reducing job satisfaction.
**Conclusion:** Burnout levels were moderate and overall job satisfaction was very low in participating radiographers. Workload and being underappreciated were identified among many factors that impact job satisfaction and burnout.
**Limitations:** Despite the international intent of the study, two thirds of participants were from the Republic of Ireland.
**Ethics committee approval:** This study was approved by the Medical School SREC, University College Cork.
**Funding for this study:** No funding was received for this study.
**Author Disclosures:**
Eadaoin Kennedy: Nothing to disclose
Mark F. McEntee: Nothing to disclose
Niamh Moore: Nothing to disclose
Rena Young: Nothing to disclose
Andrew England: Board Member: EFRS; Advisory Board: RoClub

### RPS 1614-6

**R-AI-diographers: exploring the changing professional role and identity of radiographers in Europe in the era of artificial intelligence (AI)**
*G. Walsh*[1], M. F. McEntee[2], Y. Kyratsis[3], C. A. Beardmore[1], C. Malamateniou[1]; [1]London/UK, [2]Cork/IE, [3]Amsterdam/NL
*(gemma.walsh@outlook.com)*

**Purpose:** This study aims to gain insights into the changing roles and identities of diagnostic and therapeutic radiographers in the era of AI. The objective is to propose ways to better support the workforce in the face of fast technological changes.
**Methods or Backround:** Ethics approval and written informed consent was gained prior to data collection. A Europe-wide, cross-sectional study utilising a mixed methods online survey was designed with key stakeholder feedback and translated from English into eight languages. Snowball sampling was used for distribution via social media. All European radiographers (including students) were eligible to participate. The survey collected data on the following areas: a) demographics, b) the perceived short-term impact of AI on radiographer roles, c) the potential medium-to-long-term impact of AI, d) perceived opportunities and threats of AI implementation for radiographers' roles and careers, e) the preparedness of radiographers to work with AI and f) the potential for future AI leadership roles for radiographers.
**Results or Findings:** A total of 2,258 valid responses from 38 European countries were received. Country of practice, gender, modality expertise, and years of experience impacted the responses. Training quality and quantity influence the perceptions of AI. Despite some concerns around job security, survey responses were collectively projecting a feeling of optimism for the future of radiographer careers and professional identity. Knowledge, additional training, job satisfaction, better patient care, financial compensation and career advancement were presented as key motivators to professional engagement with AI.
**Conclusion:** This study provides insights into radiographers' attitudes towards AI implementation on the future of their professional identity. It proposes ways to better support the workforce in harnessing the benefits of AI.
**Limitations:** Survey translators were not professional translators but radiographers with expert knowledge of subject terminology.
**Ethics committee approval:** This study was approved by the ethics committee of City, University of London.
**Funding for this study:** CoRIPS from the College of Radiographers and City Radiography Research Fund.
**Author Disclosures:**
Gemma Walsh: Nothing to disclose
Mark F. McEntee: Nothing to disclose
Yiannis Kyratsis Yiannis Kyratsis: Nothing to disclose
Charlotte A. Beardmore: Nothing to disclose
Christina Malamateniou: Nothing to disclose

### RPS 1614-7

**What radiographers need to know about explainable artificial intelligence in medical imaging?**

*M. Champendal*[1], H. Müller[2], J. O. Prior[1], C. S. D. Reis[1]; [1]Lausanne/CH, [2]Sierre/CH
*(melanie.champendal@hesav.ch)*

**Purpose:** Artificial Intelligence/(AI) is seen as a "black box" and health professionals tend to not trust it, at least not fully. This study aimed to present to radiographers the main eXplainable Artificial Intelligence/(XAI) methods available for medical imaging/(MI) allowing them to understand it.
**Methods or Backround:** A review was conducted following JBI methodology, searching PubMed, Embase, Cinhal, Web of Science, BioRxiv, MedRxiv, Google Scholar for French and English studies post 2017 on explainability and MI modalities. Two reviewers screened abstracts, titles, full texts, resolving disagreements through consensus.
**Results or Findings:** 1258 results were identified; 228 studies meet all criteria. The terminology used across the articles varied: explainable (n=207), interpretable (n=187), understandable (n=112), transparent (n=61), reliable (n=31), intelligible (n=3) being used interchangeably. XAI tools applied to MI are mainly intended for MRI, CT and x-ray imaging to explain lung/(Covid) (n=82) and brain/(Alzheimer's; tumours) (n=74) pathologies. The main formats used to explain the AI tools were visual (n=186), numerical (n=67), rule-based (n=11), textual (n=11). The classification (n=90), prediction (n=47), diagnosis (n=39), detection (n=29), segmentation (n=13) and image quality improvement (n=6) were the main tasks explained. The most frequently provided explanations were local (78.1%); 5.7% were global, and 16.2% combined both local and global approaches.
**Conclusion:** The number of XAI publications in MI is increasing, in aid of the classification and prediction of lung and brain pathologies. Visual and numerical output formats are predominantly used. Terminology standardisation remains a challenge, as terms like "explainable" and "interpretable" are used as equivalent. Future work is required to integrate all stakeholders in the construction of XAI.
**Limitations:** The focus was solely on recent XAI developments, leading to the exclusion of studies published before 2018, which may have caused other tools that were explored during that period to be excluded.
**Ethics committee approval:** No information provided by the submitter.
**Funding for this study:** No funding was received for this study.
**Author Disclosures:**
Claudia Sa Dos Reis: Nothing to disclose
Henning Müller: Nothing to disclose
John O. Prior: Nothing to disclose
Mélanie Champendal: Nothing to disclose

### RPS 1614-8

**Identifying the needs of radiographers working in nuclear medicine departments for the co-construction of XAI tools for enhanced PET image reconstruction**

*M. Champendal*[1], H. Müller[2], J. O. Prior[1], C. S. D. Reis[1]; [1]Lausanne/CH, [2]Sierre/CH
*(melanie.champendal@hesav.ch)*

**Purpose:** AI is often viewed as a 'black box,' causing some distrust among healthcare professionals. This study aimed to pinpoint the needs of radiographers working in a nuclear medicine department to understand better the AI algorithm used for enhancing PET/CT images.
**Methods or Backround:** Two Focus Groups/(FG) were conducted to identify background knowledge and perspectives about AI. An introduction about XAI was performed, and specific needs and preferences were explored through the presentation of scenarios, giving results corresponding or not corresponding to the ground truth. The questions that XAI should explain to support radiographers in their practice were collected, as well as main characteristics in terms of output format, confidence levels, barriers and facilitators for AI use. Thematic analysis was carried out following the Braun & Clark Framework.
**Results or Findings:** Ten radiographers (aged 31-60) from various hospital settings discussed their needs for XAI. While three currently use AI tools in PET/CT with limited training, their main requirements for XAI tools include interactivity, adaptability, user-friendliness, and minimal workflow disruption. They found a 'trust index,' visual comparisons, example-based, and chatbots as valuable output formats. Facilitators for XAI use included training, support, early integration, and radiographers specialists, while barriers included lack of understanding, organisational challenges, and system capabilities.
**Conclusion:** Needs for XAI tools were identified, but it is important to improve radiographers' knowledge and prepare the system to implement it without negatively impacting the workflow and patient outcomes.
**Limitations:** Limitations were the focus group sample size impacting generalisation and XAI knowledge.
**Ethics committee approval:** This study did not require ethics committee approval since no personal data were collected.
**Funding for this study:** No funding was received for this study.
**Author Disclosures:**
Claudia Sa Dos Reis: Nothing to disclose
Henning Müller: Nothing to disclose
John O. Prior: Nothing to disclose
Mélanie Champendal: Nothing to disclose

### RPS 1614-9

**The role of virtual reality allowing dialogue between users and virtual patients for the preclinical education of radiographers: a pilot study**

*K. Kato*[1], D. Kon[2], K. Ueda[2], C. S. D. Reis[3], N. Ienaga[1], Y. Kuroda[1]; [1]Tsukuba/JP, [2]Narita/JP, [3]Lausanne/CH

**Purpose:** Head-mounted display-based immersive virtual reality (VR) coaching systems (HMD-VRC) in radiographers' training often lack adequate interaction through spoken dialogue between users and virtual patients (VP). To enable comprehensive training, including the confirmation process to prevent risks, the study aimed to develop an HMD-VRC that allows interaction with VP to enhance preclinical learning.
**Methods or Backround:** HMD-VRC replicates 3D models of an x-ray room and the VP, incorporating two main features: 1) a voice bot based on Dialogflow, Google LLC, for the VP to respond to user dialogue and 2) an on-screen hint display system for learning support. As a pilot study, first-year students (n=10) from a Radiography/Medical Imaging (MI) school without specialised education were given a 20-minute explanation using text and physical equipment. Subsequently, they received radiography education using HMD-VRC for approximately one hour. Objective assessments via role-playing with actual equipment were conducted before and after HMD-VRC education. A survey regarding HMD-VRC was administered, asking about the experience and learning process.
**Results or Findings:** HMD-VRC enabled all students to complete radiography training from patient entry to exit. Through HMD-VRC training, the average score of the objective assessments with actual equipment increased from 59.2% to 88.3%, nearly 1.5 times higher. Survey results suggested a deeper understanding of the process through HMD-VRC, highlighting the importance of communicating/discussing with the VP in training. Additionally, HMD-VRC training was found to alleviate anxiety in objective assessments via role-playing.
**Conclusion:** This pilot study suggests that education using HMD-VRC with responsive VP through spoken dialogue is effective for preclinical education, promoting practical learning for radiographers' training. This innovative approach holds promise for improving Radiography/MI training programs, facilitating the transition between theory and practice.
**Limitations:** Only chest radiography was considered, other body parts will be validated.
**Ethics committee approval:** This study was approved by the ethics committee of the International University of Health and Welfare (Approval No. 23-Im-030).
**Funding for this study:** This study was partly supported by the JSPS KAKENHI (JP23K11369).
**Author Disclosures:**
Katsuhiko Ueda: Nothing to disclose
Claudia Sa Dos Reis: Nothing to disclose
Daisuke Kon: Nothing to disclose
Kengo Kato: Nothing to disclose
Naoto Ienaga: Nothing to disclose
Yoshihiro Kuroda: Nothing to disclose

### RPS 1614-10

**The inclusion of cross-sectional imaging within preregistration radiography training curricula**

M. Friel, *R. Young*, N. Moore, M. F. McEntee, A. England; Cork/IE

**Purpose:** Clinical practice is a critical component of all radiography curricula. It provides students with essential skills and training, enabling them to develop professional characteristics for competent radiography practice. Educational institutions are challenged with regularly reviewing and developing strategies to ensure that curricula satisfy evolving service needs. This study aimed to gain insight into the inclusion of cross-sectional imaging (CT/MRI) within preregistration radiography training curricula.
**Methods or Backround:** An online questionnaire, based on previous EFRS surveys, included open and closed-ended questions, and ascertained on level of qualification, cross-sectional imaging incorporation, and tasks and assessments within programmes. The questionnaire was distributed through the EFRS Research Hub at the European Congress of Radiology (ECR2023), and online via social media. Closed questions were summarised using descriptive statistics, while open-ended questions were thematically analysed.

**Results or Findings:** Responses were received from 64 radiography educational institutions within 29 different countries. The inclusion of cross-sectional (CT/MRI) training was reported by 57 respondents (92%) as part of their institution's radiography programme. An increase in the amount of time dedicated to clinical training in cross-sectional imaging was reported by 24 respondents (38%). Overall, 32 individuals (53%) stated that CT should be reserved as a specialised modality for radiographers once they attain their basic qualification, while 36 respondents (60%) agreed that MRI should also be reserved as a specialised modality.
**Conclusion:** The findings indicate a lack of uniformity among preregistration radiography programmes, regarding cross-sectional imaging incorporation within their curricula. However, there appear to be divided views on the issue.
**Limitations:** Predominantly, responses to the survey came from countries where English is the first/dominant language.
**Ethics committee approval:** This study was approved by the Medical School SREC, University College Cork.
**Funding for this study:** No funding was received for this study.
**Author Disclosures:**
Mark F. McEntee: Nothing to disclose
Niamh Moore: Nothing to disclose
Rena Young: Nothing to disclose
Andrew England: Board Member: EFRS; Advisory Board: RoClub
Mariosa Friel: Nothing to disclose

### RPS 1614-11

**Radiography students' viewpoints of the clinical learning environment: a cross-sectional study**

*M. O'Connor*, J. McNulty; Dublin/IE
*(michelle.oconnor@ucd.ie)*

**Purpose:** Clinical practice is fundamental to the development of students' competence and professionalism. Objective instruments have been developed to monitor the quality of clinical learning environments (CLEs) in medicine and nursing. This study is the first to investigate radiography students' perspectives of their CLEs using the validated Undergraduate Clinical Education Environment Measure (UCEEM).
**Methods or Backround:** Undergraduate (UG) (n=365) and graduate-entry (GE) radiography students (n=45) from an Irish university were invited to complete the online, self-administered, questionnaire regarding their recent clinical placement. CLEs consisted of 25 public and 10 private hospitals nationally. The UCEEM contains 25 items congregated into two overarching dimensions, experiential learning and social participation, with four subscales: opportunities to learn in and through work and quality of supervision; preparedness for student entry; workplace interaction patterns and student inclusion; and equal treatment.
**Results or Findings:** 215 students participated (response rate 52.4%) (n=185 UG, n=30 GE), most of whom were based in public hospitals (87.4%). The mean total UCEEM score was 107.54±17.67 (optimal range: 90–125). Mean scores for 'experiential learning and social participation' were 74.2±12.5 (optimal range: 60–85) and 33.37±6.29 (optimal range: 30-40), respectively. Private hospitals ranked slightly higher than public hospitals for 'opportunities to learn in and through work and the quality of supervision' ($p < 0.05$). The highest ranked items related to 'equal treatment' and the 'opportunity to put theory into practice'. Few statistically significant differences existed between cohorts except for clinical supervision-related items which were scored highest by first year students.
**Conclusion:** Students across both programmes reported high levels of satisfaction with their CLEs in terms of experiential learning and social participation. This study provides valuable baseline data for comparison of Radiography CLEs.
**Limitations:** The study was limited to a single Irish university.
**Ethics committee approval:** The study was approved by University College Dublin (LS-C-22-153-Oconnor).
**Funding for this study:** No funding was received for this study.
**Author Disclosures:**
Michelle O'Connor: Nothing to disclose
Jonathan McNulty: Nothing to disclose

**16:00-17:30 Research Stage 1**

### Research Presentation Session: Artificial Intelligence & Machine Learning & Imaging Informatics

### RPS 1605

### Exploring the frontiers of AI-enhanced radiology reporting

**Moderator**
L. Donoso; Barcelona/ES
*(ldonoso@clinic.cat)*
**Author Disclosures:**
Luis Donoso: Advisory Board; Share Holder

### RPS 1605-2

**Utilising Chat-GPT4 for conversion of free-text head and neck cancer CT reports into structured reports**

*A. Gupta*, K. Rangarajan, A. Garg; New Delhi/IN
*(amit.aiims2014@gmail.com)*

**Purpose:** The purpose of this study was to assess the performance of generative pre-trained transformer 4 (GPT-4) for conversion of free-text computed tomography (CT) reports of head and neck cancer (HNCa) patients into structured reports using a predefined template.
**Methods or Backround:** We retrieved 50 CT reports of HNCa patients from our department. A structured CT report template for HNCa was prepared enumerating various anatomical sites and their respective subsites. Other key imaging findings were also included - status of cervical lymph nodes, airway compromise and involvement of other neck structures and vessels. In the chat portal of GPT-4, the prompt with best results for structured report generation was selected after prompt engineering. Generated structured reports were evaluated by a radiologist by recording the number of places featuring missing information, misinterpreted information and any additional information not present in the actual report. The reporting template was then modified to explicitly incorporate the areas of mistakes and new GPT-4 responses were recorded.
**Results or Findings:** GPT-4 successfully converted all 50 free-text reports into structured reports. There were ten places with missing information: tracheostomy tube (n=3), non-inclusion of sternocleidomastoid in strap muscles (n=2), extranodal tumour extension (n=3) and contiguous involvement of neck structures by nodal mass rather than the primary tumour (n=2). Four pieces of information were misinterpreted: abbreviations (n=2) and non-suspicious lung nodules regarded as distant metastases (n=2). GPT-4 did not indicate any additional findings. Upon the appropriate incorporation of missing areas in the reporting template and repeating the prompts, GPT-4 rectified all the reports with no repeated or additional mistakes.
**Conclusion:** The GPT-4 model can be used to structure free-text radiology reports using plain language prompts and a simple yet comprehensive reporting template.
**Limitations:** Fine-tuning using the GPT-4 application programming interface (API) was not done in our study.
**Ethics committee approval:** The Institutional Ethics Committee approved this study.
**Funding for this study:** No funding was received for this study.
**Author Disclosures:**
Krithika Rangarajan: Nothing to disclose
Amit Garg: Nothing to disclose
Amit Gupta: Nothing to disclose

### RPS 1605-3

**Enhancing radiology reporting efficiency through structured reports: a quantitative analysis**

P. Bombinski[1], *P. P. Paczuski*[1], K. Paczuski[1], B. Duranc[1], A. Kusak[2]; [1]Warsaw/PL, [2]Lodz/PL
*(pawel.paczuski@upmedic.io)*

**Purpose:** This study explores the impact of structured reporting on radiologists' efficiency, standardisation, and clinician comprehension. We propose and analyse key metrics to quantify the acceleration of report creation using predefined templates and trigger mechanisms.
**Methods or Backround:** Structured reports apply checklist-driven templates for standardised radiological reporting. These templates comprise a checklist of observations and predefined triggers, ensuring systematic reporting. Radiologists can click on checklist items, or trigger larger report segments, such as "norm" for healthy examinations, thereby reducing the need for free text input. Structured reports can be generated using a keyboard, mouse, or voice dictation and commands.
**Results or Findings:** Our results were based on 10,000 reports of various radiological examinations performed by 20 radiologists. Our proposed metrics for evaluating the efficacy of structured reporting include: number of keystrokes (each use of computer keyboard), number of checklist clicks (each interaction with the checklist), checklist accepted suggestions (number of checklist suggestions included in the final document), contextual accepted suggestions (number of contextual suggestions included in the final document), keystrokes saved, time saved, and total time spent producing the document. Our findings demonstrate that structured reporting significantly reduces keystrokes and accelerates report generation, with an average time saving of 30% compared to conventional keyboard use. Furthermore, 84% of the checklist suggestions were accepted, improving report standardisation and reducing errors.
**Conclusion:** Structured reporting offers a promising approach to enhance radiologists' reporting efficiency. By utilising predefined templates and triggers, radiologists can create reports more rapidly while ensuring a higher level of standardisation. Clinicians benefit from clearer, more consistent reports, which can lead to better patient care. This study underscores the potential for structured reporting to bring significant advancements in radiology practices, establishing a new benchmark for efficiency and standardisation.
**Limitations:** No limitations were identified.
**Ethics committee approval:** No information provided by the submitter.
**Funding for this study:** No funding was received for this study.
**Author Disclosures:**
Bartłomiej Duranc: Employee: upmedic
Przemyslaw Bombinski: Consultant: upmedic
Krzysztof Paczuski: Founder: upmedic
Paweł Pawel Paczuski: CEO: upmedic
Artur Kusak: Nothing to disclose

### RPS 1605-4

### Leveraging GPT-4 for structured radiology reporting: a multilingual proof-of-concept study

*F. Busch*[1], L. C. Adams[2], D. Truhn[3], A. Kader[1], S. Niehues[1], M. Makowski[2], K. K. Bressem[1]; [1]Berlin/DE, [2]Munich/DE, [3]Aachen/DE
*(felix.busch@charite.de)*

**Purpose:** The purpose of this study was to examine the feasibility of automated post-hoc transformation of free-text radiology reports into structured templates using Generative Pre-trained Transformer 4 (GPT-4), a natural language processing model by OpenAI, to standardise reporting language across institutions and enhance data extraction.
**Methods or Backround:** 170 fictional English CT and MRI free-text radiology reports of various body regions and examinations (e.g. MRI of the brain, spine, joints, heart, whole body, and prostate, and CT of the head, chest, spine, thorax, abdomen, and pelvis) were generated by two board-certified radiologists. 23 structured templates were created based on previously published templates and the RadReport Template Library. GPT-4's performance was evaluated based on the accuracy and consistency of the generated structured reports. In addition, GPT-4's performance in chest radiography classification was tested against the medBERT.de German medical language benchmark on 583 German chest radiography reports. All code, JSON report templates, and CT and MRI report texts were made openly available at: https://github.com/kbressem/gpt4-structured-reporting. The web demo application can be accessed at: kbressem.pythonanywhere.com.
**Results or Findings:** GPT-4 converted all 170 free-text reports into valid JSON files for automatic reading. The model identified all radiology report key findings without any errors or omissions and consistently chose the correct report template based on the free-text report content. In the medBERT.de chest radiography benchmark, GPT-4 surpassed the existing leading model by detecting three pathological findings (congestion, opacity, pneumothorax) and one therapeutic device category (venous catheter).
**Conclusion:** This proof-of-concept study demonstrates the potential of GPT-4 in post-hoc structured radiology report text transformation, offering a cost-effective and scalable solution for medical database organisation.
**Limitations:** Restricted access to GPT-4 requires potentially sensitive data to be shared with third parties. GPT-4 is not freely available but is comparatively inexpensive at about $0.10 per report.
**Ethics committee approval:** Ethics approval was not required as the study did not involve patient data.
**Funding for this study:** No funding was was received for this study.
**Author Disclosures:**
Keno K. Bressem: Nothing to disclose
Lisa C. Adams: Nothing to disclose
Stefan Niehues: Nothing to disclose
Avan Kader: Nothing to disclose
Felix Busch: Nothing to disclose
Daniel Truhn: Nothing to disclose
Marcus Makowski: Nothing to disclose

### RPS 1605-5

### Automated anonymisation of radiology reports: comparison of publicly available natural language processing and large language models for HIPAA-compliant data use

*M. C. Langenbach*[1], B. Foldyna[2], I. L. Langenbach[2], V. Raghu[2], T. Neilan[2], I. Hadzic[2], M. T. Lu[2], J. Heemelaar[2]; [1]Cologne/DE, [2]Boston, MA/US
*(marcel.langenbach@me.com)*

**Purpose:** The purpose of this study was to leverage publicly available offline natural language processing (NLP) methods and a large language model (LLM) to automatically remove PHI from free-text radiology reports to allow for secondary data use compliant with HIPAA regulations.
**Methods or Backround:** We compared two publicly available rule-based NLP models (Google's spaCy; NLPac, accuracy-optimised; NLPsp, speed-optimised; iteratively improved on a test set of 400 randomly selected free-text radiology chest CT reports) and one offline LLM-model (Llama-2, Meta-AI) for PHI-anonymisation. The three models were evaluated on a test set of 100 new randomly selected chest CT reports. Precision, recall, and F1-scores were calculated. Two investigators adjudicated anonymisation performance based on three PHI entities (dates, medical record number (MRN), and accession numbers (ACC)) and whether relevant data was deleted.
**Results or Findings:** NLPac and NLPsp successfully removed all instances of highly sensitive PHIs (dates (n=333), MRNs (n=6), ACCs (n=92)) from the test set. The LLM-model removed all MRNs, 96% of ACCs, and only 32% of dates. NLPac was the most consistent model, with a perfect F1-score of 1.00 for MRN, ACC, and dates, followed by NLPsp, which had lower precision (0.86) and F1-score (0.92) for dates with non-dates classified as dates in 54 instances (28 cases). The LLM-model had perfect precision for all PHIs but the lowest recall of 0.96 for ACC (missed 4 instances in 4 cases) and 0.52 for dates (missed 134/333 instances in 69 cases) (F1 scores 0.98 and 0.68, respectively). Importantly, NLPac and NLPsp did not remove relevant medical information, while the LLM-model removed relevant information in 10% (n=10).
**Conclusion:** Pre-trained publicly available NLP models can effectively anonymise free-text radiology reports, while anonymisation with an LLM is more prone to remove non-PHI data.
**Limitations:** This was a pilot study involving only chest CTs.
**Ethics committee approval:** The study was approved by the institutional review board (IRB no. 2023P002169) with a waiver of written informed consent.
**Funding for this study:** No funding was received for this study.
**Author Disclosures:**
Isabel Luisa Langenbach: Nothing to disclose
Ibrahim Hadzic: Nothing to disclose
Julius Heemelaar: Nothing to disclose
Borek Foldyna: Research/Grant Support: AstraZeneca, MedImmune, and MedTrace
Vineet Raghu: Nothing to disclose
Marcel Christian Langenbach: Nothing to disclose
Michael T. Lu: Research/Grant Support: AstraZeneca, Ionis, Johnson & Johnson Innovation, and Kowa
Tomas Neilan: Other: A. Curt Greer and Pamela Kohlberg and from Christina and Paul Kazilionis, the Michael and Kathryn Park Endowed Chair in Cardiology, a Hassenfeld Scholar Award Grant Recipient: National Institutes of Health/National Heart, Lung, and Blood Institute (R01HL137562, K24HL150238)

### RPS 1605-6

### A RAdiology Data EXtraction (RADEX) tool for fast and accurate information curation from free-text reports: case study on thyroid ultrasound examinations

*L. J. Howell*, A. Zarei, T. M. Wah, S. Karthik, H. H. L. Ng, J. McLaughlan; Leeds/UK
*(scljh@leeds.ac.uk)*

**Purpose:** Extracting information from 'free-text' radiology reports is important for service evaluation, audit, unbiased cohort selection, case retrieval, and translational research including labelling medical datasets for artificial intelligence analysis. While machine learning methods have potential for automating this task, reliance on large labelled datasets and specific computing requirements limits their usefulness. Methods using human-defined rules offer a practical alternative, enabling better utilisation of information-rich radiology reports.
**Methods or Backround:** Our tool, RAdiology Data EXtraction (RADEX), leverages clinicians' domain expertise for information extraction. It uses regular expressions (regex) for efficient and flexible text pattern-matching, including wildcard and proximity searches, Boolean logic, and negation handling. This rule-based approach enables clinical users to define complex queries without specialised software knowledge, giving an easy-to-understand method which allows predictions to be reviewed and rules updated in response to changing requirements and terminology. This transparency is vital for building trust and ensuring regulatory compliance.

**Results or Findings:** RADEX was applied to neck and thyroid ultrasound reports performed between 2015-2019 across five different hospitals. Nineteen sonographic observations were classified, including presence and multiplicity of thyroid nodules, British Thyroid Association thyroid nodule grading(s), altered thyroid echotexture, thyroiditis, thyroidectomy, nodal abnormality, and parathyroid adenomata. On an expert-labelled dataset of 400 reports, RADEX achieved >90% accuracy in all classes. Processing >10,000 reports took less than 60 seconds on a standard laptop.
**Conclusion:** This free open-source tool provides a scalable approach to extracting structured data from free-text reports, prioritising usability and explainability. It leverages regex's powerful pattern-matching without requiring knowledge of its complex syntax, suiting research and audit tasks where free-text information is key to understanding, but manual review is time-consuming and expensive.
**Limitations:** The main limitation of the study is that generalisability to other datasets/languages was not evaluated.
**Ethics committee approval:** REC review is not required
**Funding for this study:** Funding was provided by the UK Research and Innovation (UKRI) Engineering and Physical Sciences Research Council (EPSRC).
**Author Disclosures:**
Shishir Karthik: Nothing to disclose
Lewis James Howell: Nothing to disclose
Amir Zarei: Nothing to disclose
Tze Min Wah: Nothing to disclose
Helen Hoi Lam Ng: Nothing to disclose
James McLaughlan: Nothing to disclose

### RPS 1605-7

**Large language models for structured reporting with speech recognition: a comparative feasibility study**
*B. Kämpgen*[1], F. Jungmann[2], D. Feiler[3], I. Schmittel[1], J. Stöckmann[3], G. Arnhold[2], P. Mildenberger[2], C. Düber[2], T. Jorg[2]; [1]Würzburg/DE, [2]Mainz/DE, [3]Munich/DE
*(benedikt.kaempgen@empolis.com)*

**Purpose:** Conventional structured reporting (SR) using a mouse and keyboard is too time-consuming for broad user acceptance. In 2023, a dialogue system which allows radiologists to use speech recognition to fill SR-templates instead was introduced (T Jorg et al., Insights Imaging DOI: https://doi.org/10.1186/s13244-023-01392-y). However, the effort of training this NLP-based system for additional SR-templates is high, e.g., modelling of concepts, synonyms, and implicit knowledge.
**Methods or Backround:** We extended the dialogue system with a state-of-the-art causal Large Language Model (LLM), OpenAI GPT-4, with a suitable prompt asking to translate from text to an SR-template in JSON, and compared the performance of the original system with the extended one.
**Results or Findings:** The extended LLM dialogue system showed slightly lower F1 score / precision / recall compared with (Jorg et al. 2023) on the same evaluation dataset comprising 82 fictional (-0.18 / -0.29 / -0.21) and 50 real examples (-0.09 / -0.19 / +0.03) of urolithiasis CT reports, with LLM-based fictional (0.80 / 0.70 / 0.75) and real (0.81 / 0.77 / 0.86) versus original fictional (0.98 / 0.99 / 0.96) and real (0.90 / 0.96 / 0.83).
The LLM had difficulties with implicit information; therefore, a inconspicuous kidney did not automatically lead to the negation of pathologies such as obstructive uropathy. Also, the LLM would hallucinate "round" calculi, or assume "no calculi" in abnormal kidneys.
**Conclusion:** The LLM-based dialogue system requires substantially less effort of training for new templates by only requiring a suitable prompt and JSON representation, without substantial loss of quality. A challenge for its application is to control implicit knowledge and hallucinations.
**Limitations:** The study included only one closed-source LLM; beyond speech-to-structure, the LLM's generative capabilities to interact with users were not evaluated.
**Ethics committee approval:** The study did not require professional legal advice from the Institutional Review Board, or informed consent of patients, according to the state hospital law. All patient data were fully de-identified and retrospectively analysed.
**Funding for this study:** Funding was provided by the Bundesministerium für Bildung und Forschung (BMBF), 2022-2025, grant agreement number: 16SV9045, project KIPA.
**Author Disclosures:**
Gordon Arnhold: Nothing to disclose
Benedikt Kämpgen: Employee: Empolis Information Management GmbH
Jan Stöckmann: Employee: DFC-SYSTEMS GmbH
Christoph Düber: Nothing to disclose
Tobias Jorg: Nothing to disclose
Florian Jungmann: Nothing to disclose
Dennis Feiler: CEO: DFC-SYSTEMS GmbH
Peter Mildenberger: Nothing to disclose
Isabel Schmittel: Employee: Empolis Information Management GmbH

### RPS 1605-8

**Using large language models to improve quality and actionability of radiology reports**
N. Kumarasami, P. N., *K. Sivasailam*, B. Subramanian; Bangalore/IN
*(kalyan@5cnetwork.com)*

**Purpose:** The objective of this study was to provide radiologists with fine-tuned large language models (LLMs) to enhance the quality, clarity, and actionability of radiology reports, with specific focus on CT Abdomen reports. Current radiology reporting methods can lead to ambiguities or misdiagnoses, especially in a remote diagnostics/teleradiology set-up. The physician/surgeon is looking for a detailed qualitative and quantitative description of a finding based on his/her suspicions and the patient's symptoms in order to arrive at a narrower set of differential diagnoses, as well as the appropriate procedure(s) he/she may follow in case of surgical intervention. Our focus was on understanding the mechanics and technical architecture behind the integration of LLMs into radiology workflows to transform the findings of a pathology into a very detailed and actionable description that is useful and relevant for the referring physician/surgeon.
**Methods or Backround:** The authors fine-tuned a foundational model and built a radiology-specific large language Model, focused on CT Abdomen, using real-life reports and templates. Initially, the LLM was fine-tuned with a data set comprising 4,500 question-answer pairs curated by the authors using instruction fine-tuning methodology. Subsequently, a retrieval-augmented generation method was employed, refining the models with 120,000 real-world reports. In the practical set-up, radiologist interact with a chatbot-like interface and input the pathologies. Using patient history, an initial draft report materialises using the LLM. Radiologist continue responding to the chatbot culminating in a comprehensive report encompassing differential diagnoses.
**Results or Findings:** The LLMs were deployed in a remote diagnostics setup at 5C Network, India. Productivity went up by 270%. Queries from referring physicians dropped by 76%.
**Conclusion:** Incorporating LLMs into radiology workflows significantly enhances report clarity and accuracy, offering a promising avenue for optimised patient care and streamlined diagnostic processes.
**Limitations:** The set-up relies on radiologists identifying the primary pathologies correctly.
**Ethics committee approval:** No information provided by the submitter.
**Funding for this study:** Funding was received from 5C Network Private Limited, India.
**Author Disclosures:**
Kalyan Sivasailam: Nothing to disclose
Bargava Subramanian: Nothing to disclose
Naveen Kumarasami: Nothing to disclose
Praveen N.: Nothing to disclose

### RPS 1605-9

**A natural language processing pipeline to extract relevant information from mammography reports**
*N. Cihoric*, D. Reichenpfader, K. Nairz, R. Gaio, P. Rösslhuemer, G. Cereghetti, H. Bonel, H. Von Tengg-Kobligk, K. Denecke; Bern/CH

**Purpose:** Although mammography reporting is highly standardised, it results in mostly unstructured reports that are difficult to process automatically. Our aim is to extract relevant information from mammography reports and make it available in a structured format.
**Methods or Backround:** We established a framework for definition and extraction of facts from the unstructured radiology reports adopting rules and specifications from the German version of BIRADS Atlas. We defined an annotation schema that ensures identification of relevant phrases in a report and subsequent information extraction at a high quality through an iterative and counter check approach. This manual annotation is supported by an automated pre-annotation to simplify handling of common phrases. The identified phrases were mapped to a standard terminology based on common data elements (CDEs) to fill a structured form with extracted information.
**Results or Findings:** BERT-based large language models were then pre-trained and fine-tuned with annotations from 210 mammography reports. Thereby we also generated a LLM based on 100,000 reports in German retrieved from our hospital. An in-depth analysis will be presented.
**Conclusion:** Our annotation approach separates extraction of information from the template filling, which reduces model complexity and permits independent improvement of both tasks. The implemented pipeline is generalisable and will allow us to structure other types of radiology reports as well. The structured information can be used for follow-up tasks such as decision support, quality assessment or outcome prediction.
**Limitations:** Our large language model is almost exclusively based on German language and it is trained on texts originating from a single hospital.
**Ethics committee approval:** Kantonale Ethikkommission Bern approved this study.
**Funding for this study:** Funding was received from the Innosuisse Project "Smaragd", 59228.1.

**Author Disclosures:**
Daniel Reichenpfader: Nothing to disclose
Harald Bonel: Nothing to disclose
Nikola Cihoric: Nothing to disclose
Grazia Cereghetti: Nothing to disclose
Knud Nairz: Nothing to disclose
Roberto Gaio: Nothing to disclose
Hendrik Von Tengg-Kobligk: Nothing to disclose
Kerstin Denecke: Nothing to disclose
Philipp Rösslhuemer: Nothing to disclose

### RPS 1605-10

**Automatic structuring of radiology reports with on-premise open-source large language models**

*P. Woznicki*[1], C. Laqua[1], I. Fiku[1], A. Hekalo[1], T. Akıncı D'Antonoli[2], D. Pinto Dos Santos[3], B. Baeßler[1], F. C. Laqua[1]; [1]Würzburg/DE, [2]Basel/CH, [3]Cologne/DE
*(piotrekwoznicki@gmail.com)*

**Purpose:** Large language models (LLMs) have successfully been used to extract structured elements from plain text. However, data protection regulations restrict the use of commercial LLMs on patient data. This study evaluated state-of-the-art, on-premise LLMs for automatically structuring free-text radiology reports.
**Methods or Backround:** We applied a novel approach to controlling the LLM output, ensuring the validity of nested structured reports produced by a locally hosted Llama-2 model. We compiled a data set of chest radiographs (CXR) including 200 English reports from a publicly available MIMIC-CXR data set and 200 de-identified German reports from a university hospital. A detailed, nested reporting template, containing 61 fields, was prepared. Ground-truth reports were annotated by a consensus of radiologists. LLM was compared to two human readers (a junior resident in cardiology and a radiographer). Bayesian inference (Markov Chain Monte Carlo sampling) was used to calculate Matthew's correlation coefficient (MCC) from contingency tables, setting (-0.05;0.05) as the region of practical equivalence (ROPE).
**Results or Findings:** The average MCC of the LLM was 0.87 (94% HDI: 0.83; 0.90) for English and 0.67 (0.60; 0.73) for German reports. MCC differences were all overlapping ROPE for English: LLM-Human1 0.012 (-0.037; 0.061), LLM-Human2 -0.002 (-0.05; 0.05), Human1-Human2 -0.01 (-0.07; 0.04), and German reports: LLM-Human1 0.001 (-0.08; 0.08), LLM-Human2 -0.065 (-0.157; 0.027), Human1-Human2 -0.066 (-0.157; 0.026).
**Conclusion:** Post-hoc structuring of English CXR reports using local, open-source LLMs is feasible and on par with human readers. However, German reports were more challenging for the model. The understanding of semantics showed variability across specialties and languages.
**Limitations:** The study's small sample size as well as the fact that some reports lacked information on certain findings and were inconclusive or ambiguous were identified as limitations.
**Ethics committee approval:** This study was approved by an ethics committee (nr: 20221004 02). The need for individual informed consent was waived.
**Funding for this study:** This work was funded by the German Federal Ministry of Education and Research (Project: SWAG, 01KD2215A).
**Author Disclosures:**
Caroline Laqua: Nothing to disclose
Fabian Christopher Laqua: Nothing to disclose
Ina Fiku: Nothing to disclose
Daniel Pinto Dos Santos: Advisory Board: cook medical
Bettina Baeßler: CEO: LernRad GmbH
Amar Hekalo: Nothing to disclose
Tuğba Akıncı D'Antonoli: Nothing to disclose
Piotr Woznicki: Consultant: Smart Reporting

### RPS 1605-11

**Integrating AI results into standardised structured radiology reports: feasibility and implementation**

*C. Thouly*[1], B. Dufour[1], B. Rizk[2], D. Goyard[3], P. Petetin[4], H. Brat[1], F. Zanca[1]; [1]Sion/CH, [2]Villars-sur-Glane/CH, [3]Paris/FR, [4]Berre l'Etang/FR
*(cyril.thouly@groupe3r.ch)*

**Purpose:** One of the main challenges the industry of radiology currently faces is the integration of AI results into clinical workflow. Healthcare professionals navigate multiple systems and interfaces (PACS, RIS, AI report), with frequently inefficient workflows. We aimed at demonstrating the feasibility and effectiveness of integrating AI-derived results into standardised structured reports (SSR) for radiology, enhancing clinical workflow and reporting accuracy.
**Methods or Backround:** A collaboration was initiated among a RIS provider, an AI platform provider, and our R&D department within a multicentric radiology network. The structured AI results were sent to the RIS via HL7 ORU messages (TCP protocol) and one message was generated per analysis. Each element of the AI structured result was placed in an OBX segment of the HL7 message. We use PatientID and AccessionNumber to link images on the PACS and radiology report in the RIS. Segments were subsequently incorporated into SSR using a beacon in the RIS, undergoing multiple iterations for layout, wording, and punctuation accuracy. The percentage of AI pre-populated fields of SSR was estimated.
**Results or Findings:** AI results were promptly transmitted to the RIS as HL7 messages. On accessing the report in the RIS, radiologists encountered prepopulated SSR subsections. Currently 40 bone age and 140 knee MRI SSR templates were successfully integrated into clinical workflows. For bone age as well as for knee MRI, the percent of pre-populated report was 60%.
**Conclusion:** Seamless integration of AI results into SSRs is achievable during routine clinical workflows. The active involvement of radiologists ensures that resultant prepopulated reports align with their requirements.
**Limitations:** The success of this integration hinges on AI vendors delivering structured and standardised results. Inaccurate AI results present potential liability concerns for radiologists due to the risk of transmitting unchecked erroneous reports.
**Ethics committee approval:** No information provided by the submitter.
**Funding for this study:** No funding was received for this study.
**Author Disclosures:**
Cyril Thouly: Shareholder: SwissMed Holding
Benoît Dufour: Shareholder: SwissMed Holding
Benoît Rizk: Shareholder: SwissMed Holding; Shareholder: Holding G
David Goyard: Employee: Incepto Medical
Hugues Brat: Speaker: GE HealthCare; Shareholder: SwissMed Holding
Pierre Petetin: Employee: EDL
Federica Zanca: Nothing to disclose

### RPS 1605-12

**Structured reporting for efficient epidemiological and in-hospital prevalence analyses of pulmonary embolism**

*T. Jorg*, M. C. Halfmann, D. Graafen, C. Düber, P. Mildenberger, L. Müller; Mainz/DE
*(tobias.jorg@unimedizin-mainz.de)*

**Purpose:** Structured Reporting (SR) not only offers advantages regarding the report quality but, as an IT-based method, also the opportunity to aggregate and analyse large, highly structured data sets (data-mining). In this study, a data-mining algorithm was used to calculate epidemiological data and in-hospital prevalence statistics of pulmonary embolism (PE) by analysing structured CT reports.
**Methods or Backround:** All structured reports for PE CT scans from the last 5 years (n = 2790) were extracted from the SR database and analysed. The prevalence of PE was calculated for the entire cohort and stratified by referral type and clinical referrer. Distributions of the localisations of PEs (central, lobar, segmental, subsegmental, left-sided, right-sided, bilateral) were calculated, and the occurrence of right heart strain was correlated with the localisations.
**Results or Findings:** The prevalence of PE in the entire cohort was 24% (n = 678). The median age of PE patients was 71 years (IQR 58 – 80). The sex distribution was 1.2/1 (M/F). Outpatients showed a lower prevalence of 23% compared to patients from regular wards (27%) and intensive care unit (30%). Surgically referred patients had a higher prevalence than patients from internal medicine (34% vs 22%). Patients with central and bilateral PEs had a significantly higher occurrence of right heart strain compared to patients with peripheral and unilateral embolisms.
**Conclusion:** Data-mining of structured reports is a simple method by which to obtain prevalence statistics, epidemiological data, and the distribution of disease characteristics, as demonstrated for the use case of PE. The generated data can be helpful for multiple purposes, such as for internal clinical quality assurance or scientific analyses. To benefit from these, consistent use of SR is required and therefore recommended.
**Limitations:** The study is limited by its single-centre design.
**Ethics committee approval:** This study did not require professional legal advice from the Institutional Review Board, or the informed consent of patients, according to state hospital law. All patient data were fully de-identified.
**Funding for this study:** This study received no outside funding
**Author Disclosures:**
Dirk Graafen: Nothing to disclose
Christoph Düber: Nothing to disclose
Moritz Christian Halfmann: Nothing to disclose
Tobias Jorg: Nothing to disclose
Peter Mildenberger: Nothing to disclose
Lukas Müller: Nothing to disclose

**RPS 1605-13**

**Extracting information from unstructured MRI reports with a local open-source GPT model**

*B. Le Guellec*, A. Lefevre, C. Bruge, L. Hacein-Bey, J-P. Pruvo, G. Kuchcinski; Lille/FR
*(bastien.le.guellec@gmail.com)*

**Purpose:** We set out to use a local open-source GPT model to automate information extraction tasks from unstructured MRI reports. We calculated its performance on reports from emergency brain MRIs performed for patients with headaches.
**Methods or Backround:** All consecutive radiological reports from a French quaternary centre in 2022 were retrospectively reviewed. Two radiologists identified MRIs that were done for headaches. Four radiologists scored reports' conclusions as normal or abnormal. Abnormalities were labelled as either headache-generating or incidental. In parallel, Vicuna, an open-source GPT large language model, performed the same tasks. Vicuna's performances were evaluated using the radiologists' consensus as the gold standard.
**Results or Findings:** A total of 2398 reports were identified, of which 595 included headache in their indication. Median patient age was 35; 68% were female. The overall rate of causal findings in outpatients with headache was 23% (135/595). Our GPT-based method had an accuracy of >95% for simple information extraction tasks such as indication of the exam, patient sex and age, use of contrast medium injection and study categorisation as normal or abnormal. Vicuna's accuracy was 82% for the most complex task of causality inference between an abnormal MRI finding and symptoms.
**Conclusion:** We found that an open-source GPT model can extract information from radiological reports with excellent accuracy without further training. We hypothesise that this method could also be applied to any information extraction task relying on unstructured medical records.
**Limitations:** Due to the monocentric design of our study, we could not test for variability in reporting styles or language. Further studies will be needed to explore the adaptability of the proposed framework, even though it is expected to be high based on ability of generative language models to handle various languages seamlessly.
**Ethics committee approval:** This study was approved by the IRB of Lille University Hospital.
**Funding for this study:** No specific funding was received for this study.
**Author Disclosures:**
Grégory Kuchcinski: Nothing to disclose
Cyril Bruge: Nothing to disclose
Lotfi Hacein-Bey: Nothing to disclose
Jean-Pierre Pruvo: Nothing to disclose
Alexandre Lefevre: Nothing to disclose
Bastien Le Guellec: Nothing to disclose

**16:00-17:30** **Research Stage 2**

### Research Presentation Session: Breast

### RPS 1602

### Functional breast imaging modalities

**Moderator**
S. Perez Rodrigo; Madrid/ES
**Author Disclosures:**
Silvia Perez Rodrigo: Consultant: Hologic, BD, GE, Motiva; Speaker: Motiva, BD, GE

**RPS 1602-2**

**DCE MRI radiomic analysis in triple negative ductal invasive breast cancer: comparison between BRCA and non-BRCA mutated patients**

*C. Bozzola*, L. Crasti, E. Balboni, C. Beretta, L. Nocetti, G. Besutti, A. Toss, G. Ligabue, A. Pecchi; Modena/IT
*(179216@studenti.unimore.it)*

**Purpose:** This study aimed to determine whether and which radiomic features from breast dynamic contrast-enhanced (DCE) MRI predict the presence of BRCA1/BRCA2 mutations in patients with triple-negative ductal invasive (TNDI) breast cancer.
**Methods or Backround:** This retrospective study included consecutive patients histologically diagnosed with TNDI breast cancer who underwent breast DCE-MRI in 2010-2021. Baseline DCE-MRIs were retrospectively reviewed; two percentage maps of wash-in and wash-out were computed and breast lesions were manually segmented, drawing a 5 mm-Region of Interest (ROI) inside the tumour and another 5 mm-ROI inside the contralateral normal glandular tissue. Features for each map and each ROI were extracted with the Pyradiomics extension of 3D Slicer and considered first separately (tumour and contralateral gland) and then together. In each analysis, eight more important features for BRCA1/2 status classification were selected with Maximum Relevance Minimum Redundancy algorithm and used to fit four different classifiers.
**Results or Findings:** The population included 69 patients for a total of 88 lesions (21 in BRCA1-mutated, 2 in BRCA2-mutated, and 65 in non-BRCA-carriers). The best classifier for BRCA mutation was Logistic Regression fitted with both tumour and contralateral gland features, reaching an AUC of 0.78, a sensitivity of 0.9 (95%CI: 0.86-0.94) and a specificity of 0.78 (95%CI: 0.74-0.82). Two features were higher in BRCA-mutated compared to non-BRCA-mutated (correlation from grey level co-occurrence matrix and root mean square), both measured in the contralateral gland in wash-out maps. They represented the coarseness of the pattern inside the ROI and the variability of voxel intensities, respectively.
**Conclusion:** This study shows the feasibility of a radiomic study with DCE maps of the breast and the potential of radiomics in predicting BRCA mutational status.
**Limitations:** The limitations of the study were that it was a retrospective design and a limited sample size.
**Ethics committee approval:** This study was approved by the AVEN Ethical Committee.
**Funding for this study:** This research has received funding from the European Union-NextGenerationEUthrough the Italian Ministry of University and Research under PNRR -M4C2-I1.3 Project PE_00000019 "HEAL ITALIA" to Giulia Besutti, E93C22001860006.
**Author Disclosures:**
Giulia Besutti: Nothing to disclose
Angela Toss: Nothing to disclose
Luca Nocetti: Nothing to disclose
Annarita Pecchi: Nothing to disclose
Guido Ligabue: Nothing to disclose
Chiara Bozzola: Nothing to disclose
Cecilia Beretta: Nothing to disclose
Erica Balboni: Nothing to disclose
Laura Crasti: Nothing to disclose

**RPS 1602-3**

**Feasibility and added value of contrast-enhanced tomosynthesis**

*P. Clauser*[1], N. Pötsch[1], P. Kapetas[1], M. Hörnig[2], M. Weber[1], R-I. Milos[1], P. A. Baltzer[1], T. H. Helbich[1]; [1]Vienna/AT, [2]Forchheim/DE
*(clauser.p@hotmail.it)*

**Purpose:** This study aimed to compare the diagnostic performance of a CE-DBT prototype with CEM. Contrast-enhanced mammography (CEM) is increasingly used in clinical practice, but its value is limited by the 2D nature of the examination. Contrast-enhanced tomosynthesis (CE-DBT) allows a quasi-3D evaluation of contrast-enhanced images and could improve lesion characterisation.
**Methods or Backround:** This prospective study was approved by the ethics committee and all patients gave written informed consent. Women presenting with suspicious findings on mammography, DBT or ultrasound were invited to participate in the study. Participants underwent CE-DBT of the breast with suspicious findings using a dedicated prototype in addition to CEM. The suspicious findings were biopsied and only histologically verified lesions were included in the analysis. Four readers (R1 and R2 non-experienced; R3 and R4 experienced) evaluated the images, blinded to patients' history, previous imaging, and histology. The readers evaluated CEM (including mammography and recombined images) and CE-DBT (including DBT and synthetic mammography) in separate sessions and gave a BI-RADS score for each finding. Sensitivity and specificity were calculated and compared.
**Results or Findings:** We included 84 patients (mean age 56 years, range 39-70) with 91 histologically verified breast lesions (27 benign, 64 malignant). Sensitivity was comparable between CEM and CE-DBT for non-experienced readers, ranging from 90% to 95%. Sensitivity improved using CE-DBT for both experience readers (from 90% and 87% with CEM to 100% and 94% with CE-DBT). Specificity was lower for non-experienced than for experienced readers with both imaging modalities, with no significant differences between modalities.
**Conclusion:** Our study showed that CE-DBT allowed an improvement in sensitivity, particularly for experienced readers, with no significant decrease in specificity.
**Limitations:** This study was a single-centre and cancer-enriched dataset.
**Ethics committee approval:** This prospective study was approved by the local ethics committee.
**Funding for this study:** Funding was received from Siemens Healthcare GmBH.

**Author Disclosures:**
Pascal A.T. Baltzer: Nothing to disclose
Nina Pötsch: Nothing to disclose
Thomas H. Helbich: Nothing to disclose
Mathias Hörnig: Employee: Siemens Healthcare GmbH
Ruxandra-Iulia Milos: Nothing to disclose
Panagiotis Kapetas: Nothing to disclose
Michael Weber: Nothing to disclose
Paola Clauser: Speaker: Siemens Healthcare GmbH

### RPS 1602-4

**Quantitative and qualitative evaluation of reduced dose of high-concentration of contrast media in CESM (contrast enhancement spectral mammography)**

*D. Pugliese*, C. Bernardi, D. Caruso, M. Zerunian, G. Maccagno, R. Gallo, A. Laghi, A. Speranza; Rome/IT
*(dominga.pugliese@uniroma1.it)*

**Purpose:** This study aimed to investigate whether a reduced dose of iodinated contrast agent, at a higher concentration, maintains adequate image quality compared with a standard dose. Contrast-enhanced spectral mammography (CESM) produces dual-energy subtracted images that demonstrate iodine uptake in infiltrating breast tumours.
**Methods or Backround:** One hundred patients with BI-RADS 6 who underwent CESM study were enrolled: 50 women had received a dose of 1.5 ml/kg of Iomeprole 350mg/ml iodinated contrast agent and 50 women a dose of 1.0 ml/kg Iomeprole 400 mg/ml. Quantitative evaluation of the images was obtained by measuring two ROIs (regions of interest): the first placed on the contrast-enhancing lesion and the second on the background of the image. The percentage difference in signal between the enhancing lesion and the background (%RS) and the signal-to-noise ratio (SNR) was calculated. The qualitative assessment was independently analysed by two experienced radiologists using EUREF criteria.
**Results or Findings:** The quantitative evaluation showed a slightly higher average lesion-enhancing value in the group with Iomeprole 350 mg/ml vs 400 mg/ml (mean ROI: 2104.95±37.95 vs 2072.89±35.35, P=0.12). The difference between %RS and SNR calculated by the Wilcoxon test showed no statistically significant difference between the two groups (respectively, p-value >0.9 and p-value >0.4). Qualitative assessment scored equally for lesions' enhancement in both the first and second groups.
**Conclusion:** Our preliminary results showed that it is possible to obtain comparable image quality in CESM studies by using a reduced dose of high-concentration iodinated contrast agent, compared to the standard dose.
**Limitations:** This study was a single-centre study with a small sample size.
**Ethics committee approval:** Written informed consent was acquired for all patients and approved by the Institutional Review Board.
**Funding for this study:** No funding was received for this study.
**Author Disclosures:**
Damiano Caruso: Nothing to disclose
Marta Zerunian: Nothing to disclose
Annarita Speranza: Nothing to disclose
Dominga Pugliese: Nothing to disclose
Claudia Bernardi: Nothing to disclose
Andrea Laghi: Nothing to disclose
Rossella Gallo: Nothing to disclose
Giacomo Maccagno: Nothing to disclose

### RPS 1602-5

**Breast lesion analysis in contrast-enhanced photon-counting CT (PC-CT) reconstructions with special breast positioning: a prospective study on iodine quantification and lesion conspicuity**

*C. Wilpert*, O. Gebler, T. Stein, F. Bamberg, M. Windfuhr-Blum, C. Neubauer, J. Neubauer; Freiburg im Breisgau/DE

**Purpose:** The objective of this study was to describe the enhancement, iodine values and contrast of breast cancers, DCIS and benign breast lesions in contrast-enhanced (CE) PC-CT.
Photon-counting CT (PC-CT) has the advantage of high spatial resolution, reduced noise and improved iodine contrast compared to conventional CT. The prone breast positioning is promising for intermodal comparisons.
**Methods or Backround:** A prospective study included 70 women (55 years ±14SD) with biopsy-confirmed breast cancers and breast MRI for lesion comparisons (BI-RADS). A PC-CT (NAEOTOMAlpha, Siemens, Erlangen) of the thorax/abdomen was performed in a prone position with special breast bearing after bolus injection of iodinated contrast-medium (fixed delay: 85 seconds) with multiplanar reconstructions of both breasts (FOV: 34 cm, matrix: 1024 x 1024, slice-thickness: 2 mm) including iodine maps and 65 keV monoenergetic images. A ROI-based analysis of HU-values and spectral information was performed referenced to the ascending aorta for quantification of iodine amount.
**Results or Findings:** Analysis of 70 biopsy-proven breast cancers, 26 DCIS, six fibroadenoma, eight intramammary lymph nodes and eight cystic lesions. Eight additional cystic lesions could not be detected at all. Mean enhancement, mean iodine concentrations and % of enhancement to the aorta reference were highest in breast cancers (112 HU, 2.7 mg/ml, 52%) and differed significantly from all other lesion types including DCIS (91 HU,1.7 mg/ml, 33%), fibroadenoma (61 HU, 0.3 mg/ml, 5%), intramammary lymph nodes (79 HU,1.8 mg/ml, 38%) and cystic lesions (33 HU, 0 mg/ml, -1%); each p<0.001. The contrast was highest in breast cancers. Cystic lesions presented with low contrast.
**Conclusion:** Compared to all other types of lesions, breast cancers displayed the highest enhancement and iodine concentrations; these characteristics indicate that breast CT reconstruction might be suited for pre-therapeutic local breast cancer staging. Additionally, DCIS can be discriminated against with CE PC-CT. In contrast, cystic lesions cannot be displayed efficiently.
**Limitations:** Only a small number of biopsy-proven benign lesions were analysed. Morphologic features were not compared to MRI. PC-CT is not sufficient for the presentation of micro-calcifications.
**Ethics committee approval:** This study was approved by the Ethics Number: 21-1717, German clinical trials register: DRKS00028997
**Funding for this study:** This study was partially funded by the Young Researchers Grant awarded by the EUSOBI.
**Author Disclosures:**
Caroline Wilpert: Nothing to disclose
Jakob Neubauer: Nothing to disclose
Claudia Neubauer: Nothing to disclose
Oliver Gebler: Nothing to disclose
Fabian Bamberg: Nothing to disclose
Marisa Windfuhr-Blum: Nothing to disclose
Thomas Stein: Nothing to disclose

### RPS 1602-6

**Discrimination of axillary lymph nodes and lymph node metastasis in contrast-enhanced photon-counting CT (PC-CT) breast reconstructions**

*C. Wilpert*, M. Molina, T. Stein, F. Bamberg, M. Windfuhr-Blum, C. Neubauer, J. Neubauer; Freiburg im Breisgau/DE

**Purpose:** The objective of this study was to investigate whether lymph node metastases can be distinguished from benign lymph nodes and breast cancers by their enhancement in contrast-enhanced (CE) PC-CT. Imaging of axillary lymph nodes continues to be a challenge, where up to this date sonography remains the imaging reference. Photon-counting computed tomography (PC-CT) allows for high spatial resolution and iodine quantification.
**Methods or Backround:** Inclusion of 75 women (56 years ±14 SD) with biopsy-confirmed breast cancers and MRI available to confirm diagnostic criteria of benign lymph nodes (BI-RADS). A PC-CT (thorax/abdomen) was performed with special prone breast positioning (NAEOTOMAlpha, Siemens, Erlangen) after bolus injection of iodinated contrast medium (delay: 85 seconds) and multiplanar reconstructions of both breasts (FOV: 34 cm, matrix: 1024 x 1024, slice-thickness: 2 mm) including iodine maps and 65 keV monoenergetic images. ROI-based analysis was performed using the iodine concentration of the aorta ascendens as a reference.
**Results or Findings:** Analysis of 70 biopsy-proven breast cancers, 18 lymph node metastasis and 57 benign lymph nodes. Enhancement was 121 HU in metastasis, 112 HU in breast cancers and 105 HU in benign lymph nodes. Iodine concentrations and referenced enhancement (%) was highest in breast cancers (2.7 mg/ml, 52%). Lymphnode metastasis differed significantly from benign lymph nodes (2.5 mg/ml, 49% versus 1.9 mg/ml, 37%; enhancement: p=0.004; iodine: p=0.008) but not from breast cancers (enhancement: p=0.251; iodine: p=0.153). In contrast, benign lymph nodes differed significantly from breast cancers (p <0.001).
**Conclusion:** Enhancement of lymph node metastasis was similar to breast cancers and differing from benign lymph nodes, which might allow further differentiation. However, as ranges were rather wide we do not believe cut-off values can be drawn from the data for lymph node evaluation with PC-CT; instead, as known from MRI, ultrasound with it's ability to depict morphology and perfusion is expected to outperform PC-CT providing less morphologic information.
**Limitations:** Morphologic features of lymph nodes were not evaluated. PC-CT was not compared to other imaging modalities.
**Ethics committee approval:** This study was approved by the Ethics Number: 21-1717, German clinical trials register: DRKS00028997
**Funding for this study:** This research was partially funded by the Young Researchers Grant awarded by the European Society of Breast Imaging (EUSOBI).

**Author Disclosures:**
Caroline Wilpert: Nothing to disclose
Jakob Neubauer: Nothing to disclose
Moises Molina: Nothing to disclose
Claudia Neubauer: Nothing to disclose
Fabian Bamberg: Nothing to disclose
Marisa Windfuhr-Blum: Nothing to disclose
Thomas Stein: Nothing to disclose

### RPS 1602-7

**Quantitative DCE-MRI in breast cancer: different HER2 expression cohorts**

*Y. Hu*, M. Li, N. Shao, Z. Xiaoling; Guangzhou/CN
*(huyling5@mail.sysu.edu.cn)*

**Purpose:** This study aimed to quantitatively assess the differences in parameters of dynamic contrast-enhanced MRI (DCE-MRI) in HER2-zero, HER2-low, or HER2-positive tumours, and to build optimal models for early prediction of HER2-low breast cancer (BC).
**Methods or Backround:** Clinical and DCE-MRI data from 220 BC patients receiving neoadjuvant chemotherapy (NACT) were retrospectively analysed. Quantitative and semi-quantitative DCE-MRI parameters were compared in the HER2-zero, HER2-low, or HER2-positive groups before and after early NACT. Empirical models were developed to predict HER2-low BC using logistic regression analysis and receiver operating characteristic (ROC) analysis.
**Results or Findings:** Patients of HER2: low BC have a lower pCR rate compared with HER2-zero and HER2-positive (17.9% vs. 10.4% vs. 29.5%, p<0.001), predominantly in the HR (hormone receptor) negative group (22.2% vs. 7.7% vs. 40.5%, p<0.001). Before NACT, HER2-low BC exhibited higher Kep, Ktrans, Washin, and lower TME intratumoral perfusion characteristics, and higher Kep and lower TME in peritumoral than the other group. Notably, after early NACT, changes in Ktrans, Kep, and Washin in intratumoral and peritumoral perfusion were more pronounced in the HER2-low group compared to other groups. The ROC curves (AUC) for the pre-NACT intratumoral, peritumoral, and combined perfusion models were 0.675 (95% CI: 0.600-0.750), 0.661 (95% CI: 0.585-0.738), 0.731 (95% CI: 0.660-0.802). The combined pre-and-post-NACT perfusion model further improved predictive performance accordingly, with AUCs of 0.764 (95% CI: 0.637-0.865), 0.795 (95% CI: 0.711-0.878), 0.850 (95% CI: 0.774-0.926).
**Conclusion:** The study revealed perfusion heterogeneity between different HER2 statuses and identified the best imaging model as a non-invasive tool to predict HER2-low BC, which can help pre-treatment clinical decision-making.
**Limitations:** Further multi-centre validation is required.
**Ethics committee approval:** This study was approved by the Ethics Committee of the First Affiliated Hospital of Sun Yat-Sen University (No.2020-536).
**Funding for this study:** This study has received funding from the Beijing Science and Technology Innovation Medical Development Foundation (KC2021-JX-0044-2).
**Author Disclosures:**
Zhang Xiaoling: Nothing to disclose
Nan Shao: Nothing to disclose
Meizhi Li: Nothing to disclose
Yangling Hu: Nothing to disclose

### RPS 1602-8

**Proportion of suboptimal baseline 18F-FDG PET/CT exams in oestrogen positive breast cancer patients according to 18F-FDG uptake in the primary tumour: a single-centre retrospective analysis**

*M. Lenaerts*[1], L. Van der Voort[1], M. Smidt[1], C. Van De Weijer[1], V. Tjan-Heijnen[1], F. A. Gallagher[2], L. Aloj[2], T. van Nijnatten[1]; [1]Maastricht/NL, [2]Cambridge/UK
*(melissa.lenaerts@maastrichtuniversity.nl)*

**Purpose:** This study aimed to assess the proportion of ER-positive tumours with suboptimal 18F-FDG uptake. The extent of breast cancer is an important prognostic factor in locally advanced breast cancer (LABC). Therefore, staging using 18F-FDG PET/CT is recommended. However, previous studies have confirmed a significantly lower degree of 18F-FDG uptake in primary breast tumours of the oestrogen receptor (ER)-positive subtype compared to other subtypes. Consequently, suboptimal 18F-FDG uptake in ER+ LABC might lead to suboptimal staging.
**Methods or Backround:** Baseline 18F-FDG PET/CT scans of female patients diagnosed with ER+ LABC in the Maastricht University Medical Centre between 2011-2022 were retrospectively collected. The maximum standardised uptake value (SUVmax) of the primary tumour was measured. Different SUVmax cut-off values were applied to determine the proportion of suboptimal 18F-FDG PET/CT exams. Multivariable logistic regression was performed to determine the possible correlation between clinicopathological predictors and the SUVmax of the primary tumour.
**Results or Findings:** 74 patients were included. SUVmax cut-off values of 2.0, 2.5, 3.0, 3.5, 4.0, 4.5, and 5.0 correspond with a proportion of women with a primary tumour SUVmax below the cut-off value of 6.8%, 16.2%, 24.3%, 31.1%, 39.2%, 40.5%, 45.9%, respectively. When considering 3.0 as an arbitrary cut-off value for SUVmax of the primary tumour, multivariable logistic regression of both ER-percentage (10-100%) and tumour grade (1-2 vs. 3) showed a lower tumour grade to be significantly correlated with a lower SUVmax (0.07 [0.008–0.562]; p=0.013).
**Conclusion:** A considerable proportion of ER+ LABC patients have a relatively low SUVmax value of the primary tumour, indicating a potential suboptimal staging on the baseline 18F-FDG PET/CT exam. When considering SUVmax 3.0 as an arbitrary cut-off value of the primary tumour, 24.3% of the patients might have a suboptimal baseline 18F-FDG PET/CT exam.
**Limitations:** No limitations were identified.
**Ethics committee approval:** Due to the retrospective design of the study, approval was waived by the local ethics committee
**Funding for this study:** No funding was received for this study.
**Author Disclosures:**
Marjolein Smidt: Nothing to disclose
Christina Van De Weijer: Nothing to disclose
Thiemo van Nijnatten: Nothing to disclose
Luigi Aloj: Nothing to disclose
Vivianne Tjan-Heijnen: Nothing to disclose
Melissa Lenaerts: Nothing to disclose
Lois Van der Voort: Nothing to disclose
Ferdia Aidan Gallagher: Nothing to disclose

### RPS 1602-9

**Breast imaging reporting and data system in contrast-enhanced mammography (CEM): "Lesion Conspicuity" correlation with malignancy and tumour receptor pattern**

*D. Pupo*[1], L. Nicosia[2], A. C. Bozzini[2], L. Mariano[3], G. Signorelli[2], F. Abbate[2], F. Priolo[2], E. Cassano[2]; [1]Naples/IT, [2]Milan/IT, [3]Turin/IT
*(dave.dp93@gmail.com)*

**Purpose:** This study aimed to evaluate the diagnostic performance and relationship with the receptor profile of this new descriptor. The new version of the Breast Imaging Reporting And Data System (BIRADS) related to contrast-enhanced mammography (CEM) encourages investigations on a new enhancement descriptor: "Lesion Conspicuity" (LC): defined as the intensity of enhancement of a lesion in relation to the background parenchymal enhancement (BPE).
**Methods or Backround:** 325 patients with 381 breast lesions undergoing CEM before histological evaluation were selected. Four radiologists, in a blind study, classified LC into the following levels: absent, low, moderate and high. Considering "moderate" and "high" as predictive of malignancy, the diagnostic performance of CEM was calculated using histological biopsy results as the gold standard. The association between LC values and receptor profile of neoplasms was also evaluated.
**Results or Findings:** The mean age at CEM examination was 50 years (range: 45-59). Considering the LC value of the most experienced radiologist in Low Energy (LE) image interpretation, we obtained a sensitivity (SE) of 91.9% (95% CI: 88.6%-95.2%) and specificity (SP) of 67.2% (95% CI: 58.9%-75.5%). An association was observed between "high" lesion conspicuity with unexpressed ER/PgR (p=0.025), with Ki-67 > 20% (p=0.033) and with G3 grading (p=0.020).
**Conclusion:** The new descriptor, "Lesion Conspicuity," demonstrated more than satisfactory performance in predicting lesion malignancy and significant correlation with the receptor profile of malignant breast neoplasms.
**Limitations:** This was a monocentric study.
**Ethics committee approval:** The retrospective study was approved by The Institutional Review Board.
**Funding for this study:** No funding was received for this study.
**Author Disclosures:**
Luca Nicosia: Nothing to disclose
Anna Carla Bozzini: Nothing to disclose
Giulia Signorelli: Nothing to disclose
Francesca Abbate: Nothing to disclose
Francesca Priolo: Nothing to disclose
Enrico Cassano: Nothing to disclose
Davide Pupo: Nothing to disclose
Luciano Mariano: Nothing to disclose

16:00-17:30 **Research Stage 3**

### Research Presentation Session: Cardiac

### RPS 1603

### Biomarkers in cardiac imaging

**Moderator**
D. Suchá; Utrecht/NL

### RPS 1603-2

**A radiomic model to correlate the quality of pericoronary adipose tissue with significant coronary artery disease**

*M. De Giorgi*[1], S. Dell'Aversana[2], A. Ponsiglione[1], A. Paludi[1], L. Pinto[1], A. Annunziata[1], E. Cavaglià[2], R. Cuocolo[1], M. Imbriaco[1]; [1]Naples/IT, [2]Pozzuoli/IT
*(marcodeg9@gmail.com)*

**Purpose:** The study aimed to evaluate the correlation between pericoronary adipose tissue (PCAT) quality, assessed with radiomics features, and significant coronary artery stenoses (≥70%).
**Methods or Backround:** Four operators retrospectively analysed the images of 100 coronary CT exams (50 patients with significant coronary artery disease [CAD] and 50 healthy controls). The course of the coronary artery on axial CT images was semi-automatically segmented, followed by automated expansion of the VOI. A threshold was then applied to include exclusively the PCAT. Feature extraction was done with the open source PyRadiomics package, and an Intraclass Correlation Coefficient Analysis (ICC) was performed based on segmentations by four different readers, to assess feature stability. To explore the potential clinical value of these stable radiomics features an exploratory supervised machine learning analysis was performed. The training set patients were balanced using synthetic oversampling, followed by information gain ranking feature selection. Finally, using the WEKA software platform, a simple logistic model's performance was assessed.
**Results or Findings:** A total of 1,183 radiomics features from each VOI and other clinical values (age, gender hypertension, diabetes, dyslipidaemia, familiarity) were extracted. Of these, 1,068 were found to be stable at the ICC analysis (ICC lbound ≥0.75). After merging radiomics and clinical features, those with low variance were 108, while 777 proved to be highly intercorrelated. The model trained on the top five features based on information gain ranking achieved an AUC of 0.88 in the training data and 0.83 in the test set to identify significant stenoses.
**Conclusion:** PCAT radiomics paired with clinical data showed good discrimination between healthy and significantly stenotic coronary vessels, with a high degree of inter-reader reproducibility. Future studies are needed to create models predictive of prognosis or treatment response.
**Limitations:** This was a single-centre, retrospective study.
**Ethics committee approval:** This study was approved by local IRB, with a waiver for informed consent due to the retrospective, observational nature of the study.
**Funding for this study:** No funding was received for this study.
**Author Disclosures:**
Alessandra Annunziata: Nothing to disclose
Lorenzo Pinto: Nothing to disclose
Adriana Paludi: Nothing to disclose
Andrea Ponsiglione: Nothing to disclose
Enrico Cavaglià: Nothing to disclose
Massimo Imbriaco: Nothing to disclose
Serena Dell'Aversana: Nothing to disclose
Marco De Giorgi: Nothing to disclose
Renato Cuocolo: Nothing to disclose

### RPS 1603-3

**Advancing pericoronary inflammation evaluation: dual-energy CT-derived fat fraction as a promising imaging biomarker for cardiovascular risk stratification**

*S. Marziali*, C. B. Monti, D. Capra, F. Rizzetto, G. Folco, F. Sardanelli, F. Secchi; Milan/IT
*(sara.marziali@unimi.it)*

**Purpose:** The objective of this study is to assess the role of pericoronary fat fraction (FF) obtained via dual-energy CT as an innovative imaging biomarker to detect coronary inflammation, comparing it to conventional biomarkers such as pericoronary fat attenuation index (FAI) and the degree of coronary stenosis.
**Methods or Backround:** In our retrospective study, we reviewed patients who had undergone cardiac CT scans at our institution using a dual-energy calcium scoring scan. We collected both demographic and clinical data for each patient, including factors like family history of CAD, smoking habits, type 2 diabetes, hypertension, and hypercholesterolaemia. Our main objective was to evaluate fat fraction (FF), defined as the ratio of adipose tissue within a designated area: Each patient's FF maps were processed using the Syngo.via software, and regions of interest (ROIs) were delineated around the coronary arteries, specifically the left anterior descending artery (LAD), circumflex artery (CX), and right coronary artery (RCA). Concurrently, fat tissue attenuation (FAI) values were recorded. Information regarding coronary stenoses was extracted from CT reports for comprehensive analysis.
**Results or Findings:** The study encompassed a sample of 99 patients, comprising 32% females with a median age of 66 years (IQR: 58-74 years). A significant negative correlation emerged between FF and FAI across coronary arteries: LAD at ρ=-0.617 (p<0.001), LCX at ρ=-0.493 (p<0.001), and RCA at ρ=−0.506 (p<0.001). Pericoronary FF displayed a weak negative correlation with coronary stenosis at the LAD (ρ=-0.220, p=0.035), correlations were not statistically significant for the LCX and the RCA (p=0.572).
**Conclusion:** Fat fraction, evaluated using dual-energy CT, presents promise as an additional imaging biomarker for coronary inflammation. It could yield potential for cardiovascular risk stratification, underscoring the need for further, comprehensive research.
**Limitations:** This was single centre, retrospective study.
**Ethics committee approval:** The local ethics committee (Ethics Committee of IRCCS Ospedale San Raffaele) approved this retrospective study (protocol code "CardioRetro", number 122/int/2017; approved on 14th September 2017, and amended on 19th July 2022). Informed consent was waived due to the retrospective nature of the study.
**Funding for this study:** No funding was received for this study.
**Author Disclosures:**
Francesco Rizzetto: Nothing to disclose
Sara Marziali: Nothing to disclose
Francesco Secchi: Nothing to disclose
Francesco Sardanelli: Nothing to disclose
Caterina Beatrice Monti: Nothing to disclose
Gianluca Folco: Nothing to disclose
Davide Capra: Nothing to disclose

### RPS 1603-4

**Prognostic value of changes in epicardial adipose tissue volume and density on lung cancer screening CT**

*I. L. Langenbach*[1], I. Hadzic[1], R. Zeleznik[1], M. C. Langenbach[1], D. Maintz[2], T. Mayrhofer[1], M. T. Lu[1], H. Aerts[1], B. Foldyna[1]; [1]Boston/US, [2]Münster/DE
*(il.langenbach@gmail.com)*

**Purpose:** To investigate how longitudinal changes in epicardial adipose tissue (EAT) volume and density relate to all-cause, cardiovascular (CV), and lung cancer mortality in participants undergoing low-dose computed tomography (LDCT) for lung cancer screening.
**Methods or Backround:** EAT volume and density, known as potential risk factors for CV disease, were measured using a validated automated deep-learning algorithm on serial, non-ECG-synchronised, non-contrast chest LDCT in heavy smokers enrolled in the National Lung Screening Trial (NLST). EAT volumes were indexed to body surface area (cm3/m2). EAT volume and density changes over two years (EAT; t2-years–tbaseline) were categorised into increase, no-change, and decrease categories (no-change: volume: -7 to +11%; density: -3 to +2%). EAT was associated with all-cause, CV, and lung cancer mortality (10-year follow-up) in multivariable Cox regression models, adjusted for baseline EAT values, traditional CV risk factors, BMI, history of CV disease, and coronary artery calcium (CAC) score.
**Results or Findings:** Of 20,661 people (59% men; age: 61±5 years), 3,483/20,661 (16.9%) died over a median follow-up of 10.4 (9.9–10.8) years (CV death: 816/3,483 [23.4%]; lung cancer death: 705/3,483 [20.2%]). In general, EAT volume increased, while density slightly decreased over time (+2.5±11.0 cm3/m2 and -0.5±3.0 HU). In fully adjusted analysis, EAT volume decrease was related to all-cause and CV mortality (HR=1.34; 95%CI: 1.23–1.46; p<0.001, HR=1.27; 95%CI: 1.06–1.51; p=0.009, respectively), while EAT volume increase was associated with higher hazard of all-cause mortality (HR=1.15; 95%CI: 1.06–1.25; p<0.001). EAT density increase was associated with a higher risk for all-cause (HR=1.32; 95%CI: 1.22–1.44; p<0.001), CV (HR=1.30; 95%CI: 1.09–1.55; p=0.004), and lung cancer mortality (HR=1.34; 95%CI: 1.11–1.62; p=0.002).
**Conclusion:** Patients eligible for lung cancer screening with changes in EAT volume and density have higher mortality risks and a need for better risk stratification.
**Limitations:** Identified limitations were (1) that this was a retrospective study, and (2) that a high-risk population of heavy smokers was used.
**Ethics committee approval:** No information provided by the submitter.

**Funding for this study:** No funding was received for this study.
**Author Disclosures:**
Isabel Luisa Langenbach: Nothing to disclose
David Maintz: Nothing to disclose
Hugo Aerts: Nothing to disclose
Ibrahim Hadzic: Nothing to disclose
Thomas Mayrhofer: Nothing to disclose
Borek Foldyna: Research/Grant Support: AstraZeneca, MedImmune, and MedTrace
Roman Zeleznik: Nothing to disclose
Marcel Christian Langenbach: Nothing to disclose
Michael T. Lu: Research/Grant Support: AstraZeneca, Ionis, Johnson & Johnson Innovation, and Kowa

### RPS 1603-5

**Foetal epicardial fat thickness: can it serve as a marker for gestational diabetic mellitus?**

*A. Singh*; Amritsar/IN
*(dr.amancs@gmail.com)*

**Purpose:** To evaluate the role of foetal epicardial fat thickness as a marker to screen for GDM.
**Methods or Backround:** The study included pregnant patients at 24 + 0/6 to 28 + 0/6 weeks of gestation scheduled for a 75 g oral glucose tolerance test from December 2020 to March 2022. Antenatal ultrasound was performed on a Voluson E8 Expert BT12 (Wipro GE) ultrasound machine. Out of 180 patients, 60 patients were selected, that is, 30 patients with raised 75 g OGTT results (cases of GDM) and 30 patients with normal 75 g OGTT results. The collected data were transformed into variables, coded, and entered into Microsoft Excel. Data were analysed using the Shapiro–Wilk normality test, student's t-test or Mann–Whitney U test, chi-square test, or Fisher's exact test and statistically evaluated using the SPSS-PC-25 version.
**Results or Findings:** Foetal EFT was found to be significantly more in the GDM group in comparison to controls without GDM, and the increased foetal EFT was positively associated with 2-hour OGTT serum glucose values. The mean foetal epicardial fat thickness (EFT) in mothers with GDM was significantly larger, i.e., 0.17 ± 0.02 cm than in mothers without GDM, i.e., 0.12 ± 0.01 cm ($p < 0.001$). The receiver operating characteristic (ROC) curve plotted from values calculated from our results shows high sensitivity (i.e., 96.67%) and specificity (i.e., 90%) of foetal EFT as a predictor for GDM with an AUROC value of 0.96 and 95% confidence interval of 0.92 to 1.0.
**Conclusion:** EFT was significantly higher in foetuses of diabetic versus nondiabetic mothers, therefore concluding that epicardial fat thickness measurement in foetuses can serve as a novel marker in GDM.
**Limitations:** Study can be extended to a larger sample population.
**Ethics committee approval:** Due clearance was obtained from the institutional research and ethics committee.
**Funding for this study:** No funding was received for this study.
**Author Disclosures:**
Amandeep Singh: Nothing to disclose

### RPS 1603-6

**Is familial combined hypolipidemia (FHBL2) due to loss of function mutation in the Angiopoietin-like3 (ANGPTL3) gene protective for CAD?"**

*S. Paciulli*[1], G. C. Pambianchi[1], L. Marchitelli[1], M. Giannetti[1], M. Francone[2], C. Catalano[1], N. Galea[1]; [1]Rome/IT, [2]Milan/IT
*(serena.paciulli@gmail.com)*

**Purpose:** The purpose of this study was to compare the prevalence and severity of coronary atherosclerotic disease (CAD) and the density and volume of epicardial fat (EpF) between a group of patients with FHBL2 and an age- and gender-matched control group and to analyse the correlation between EpF and stenosis degree.
**Methods or Backround:** A group of 111 FHBL2 subjects were prospectively recruited and underwent coronary CT (CCTA). 46 normolipidemic asymptomatic age- and gender-matched subjects, who underwent CCTA according to current guidelines, were enrolled as a control group. CAD-RADS were used to determine stenosis severity and to divide patients into four classes (N= 0-1; L= 2; M= 3; S= 4-5). The density and volume of epicardial fat (EpF) were evaluated on CCTA images.
**Results or Findings:** According to CCTA images, FHLBL2 patients were classified as follows: 74 subjects were classed N (67%), 22 L (20%), 7 M (6%) and 8 S (7%); among the controls 17 subjects were classed N (37%), 16 L (35%), 8 M (17%) and 5 S (11%). In FHLBL2 subjects there was a higher prevalence of minimal or no stenosis (N: 67% vs 37%; $P = 0.001$), and a lower prevalence of mild stenosis (L: 20% vs 35%; $p < 0.05$), while there were no significant differences in the development of severe coronary atheromasia (S: 7% vs 11%; $P = 0.553$). There were no significant differences in terms of EpF density or volume between the two groups ($P > 0.060$ for both) but a correlation was observed between EpF density and volume and the degree of stenosis (Rho: - 0.563; $P = 0.023$ and Rho: 0.565; $P = 0.019$).
**Conclusion:** FHBL2 is associated with a lower prevalence and severity of CAD. An association between EpF density and volume and the severity of stenosis was also found.
**Limitations:** This was a single centre study.
**Ethics committee approval:** This study was approved by an ethics committee.
**Funding for this study:** No funding was received for this study.
**Author Disclosures:**
Giacomo Carlo Pambianchi: Nothing to disclose
Serena Paciulli: Nothing to disclose
Livia Marchitelli: Nothing to disclose
Marco Francone: Nothing to disclose
Martina Giannetti: Nothing to disclose
Nicola Galea: Nothing to disclose
Carlo Catalano: Nothing to disclose

### RPS 1603-7

**Combined use of MRI and PET imaging enhances diagnostic accuracy in cardiac sarcoidosis**

*K. H. Stensaeth*, H. Johansen, A. C. Dale; Trondheim/NO
*(knutsten@gmail.com)*

**Purpose:** Diagnosing cardiac sarcoidosis (CS) often presents challenges. This study sought to assess the efficacy of integrating cardiac MR (CMR) with positron emission tomography (PET)-MR for diagnosing CS and guiding patient management.
**Methods or Backround:** We conducted a retrospective review of 46 patients at St Olav's Hospital, Trondheim, Norway, between February 2017 and October 2021. Patients underwent CS evaluation using both CMR and PET-MR. Post-CMR, an experienced radiologist identified all patients as possibly having CS. A seasoned nuclear medicine physician then classified PET results as normal, unspecific, or positive. The final diagnosis combined imaging and clinical findings.
**Results or Findings:** Participants averaged 58±11 years of age; 24% were female. 28% had a previous biopsy confirming sarcoidosis. Predominant symptoms included dyspnea (26%), angina (15%), and syncope (13%), with 26% reporting arrhythmia history. Late gadolinium enhancement (LGE) prominently appeared in basal segments 2-6. Abnormal F18-FDG uptake occurred in 12 (26%) patients, with 10 patients deemed positive and two unspecific. During the inclusion period, mortality stood at 9%, exclusively among patients with positive FDG uptake. 43% exhibited compromised left ventricle (LV) function, 39% had hypertrophic cardiomyopathy, and 17% had a dilated LV. Alternative diagnoses comprised amyloidosis, giant cell myocarditis, systemic lupus erythematosus, Fabry's disease, arrhythmic right ventricle cardiomyopathy, and endocarditis. Additionally, 26% received an ICD or pacemaker.
**Conclusion:** In patients with potential CS, the combined use of CMR and PET imaging significantly aids diagnosis and management decisions. Integrating these modalities with clinical data can notably enhance diagnostic accuracy, especially when CMR results are ambiguous.
**Limitations:** This was a retrospective single centre study.
**Ethics committee approval:** This study was approved by an ethics committee; REK 6701 South-East C Norway.
**Funding for this study:** No funding was received for this study.
**Author Disclosures:**
Knut Haakon Stensaeth: Nothing to disclose
Haakon Johansen: Nothing to disclose
Ane Cecilie Dale: Nothing to disclose

**16:00-17:30** **Research Stage 4**

### Research Presentation Session: Musculoskeletal

### RPS 1610

### Pelvis, hip, and bone lesions

**Moderator**
M. C. De Jonge; Utrecht/NL
*(milkodejonge@gmail.com)*

### RPS 1610-2

**Can we differentiate acute osteomyelitis and vaso-occlusive crises in sickle cell disease patients?**

*F. Nabet*; Paris/FR
*(floriannabet@gmail.com)*

**Purpose:** Vaso-occlusive crisis (VOC) is the most common complication in individuals with sickle cell disease (SCD), but distinguishing it from acute osteomyelitis, which is also frequent, poses a daily clinical challenge. Some rare forms of VOC, localised in the periosteum (pVOC), are also difficult to differentiate from osteomyelitis with clinico-biological features. The aim of this study was to better characterise these two entities with imaging to improve the management of these fragile patients, particularly to avoid overly frequent antibiotic use.
**Methods or Backround:** From 2008 to 2023, 32 SCD patients with painful periosteal crisis and available MRI data were included (15 women), ranging in age from 17 to 39, with a baseline haemoglobin level of 8.25 (8-8.8), 27 (85%) with SS genotype and 16 (50%) with Bantu/Bantu haplotype. The formal diagnosis (osteomyelitis vs pVOC) was confirmed by the clinician team. In imaging, two independent radiologists blindly reviewed the MRIs and recorded simple descriptive criteria, including the presence or absence of subperiosteal collections, their size, cortical abnormalities, soft tissue oedema, circumferential character, T1 signal with fat saturation, T2 signal, type of enhancement, and associated bone marrow abnormality.
**Results or Findings:** T1 hyperintensity with fat signal saturation was exclusively found in cases of periosteal pVOC (8 cases, 42,9 % of pVOC cases). Furthermore, circumferential oedema was never observed in cases of pVOC (3 cases, 57,1% of osteomyelitis cases). Interobserver reproducibility was very good (Cohen's kappa = 0.82).
**Conclusion:** MRI appears to be a valuable tool to differentiate VOC and osteomyelitis in SCD patients with painful periosteal crises.
**Limitations:** This is a retrospective monocentric study with some missing data. Within our data set, there seems to be a higher proportion of SCD patients with vaso-occlusive phenotype than in the general SCD population.
**Ethics committee approval:** This study was approved by the HEGP ethics committee.
**Funding for this study:** No funding was received for this study.
**Author Disclosures:**
Florian Nabet: Nothing to disclose

### RPS 1610-3

**Hip muscle size and density are associated with trochanteric fractures of elderly women**

*P. Huang*, Y. Ge, A. Yu, L. Wang, X. Cheng; Beijing/CN
*(1911210542@bjmu.edu.cn)*

**Purpose:** We aimed to investigate the differences in hip muscle area and density between older patients with femoral neck (FNF) and trochanteric fractures (TRF)
**Methods or Backround:** A total of 554 older women patients were enrolled, including 314 FNF (77.02 ± 7.15 years) and 240 TRF (79.70 ± 6.91 years) for the comparisons. The area and density of the gluteus medius and minimus muscle (G.Med/MinM) and the gluteus maximus muscle (G.MaxM) were measured by CT. Total hip (TH) areal bone mineral density (aBMD) and femoral neck aBMD (FNaBMD) were measured by quantitative CT. A cut-off of 80 years was used to stratify the cohort and to further explore the age-specific relationship.
**Results or Findings:** For the total subjects, all these muscle parameters were higher in the FNF group than in the TRF group (P <0.001). The muscle parameters except for the G.Med/MinM density were significantly correlated with hip fracture typing after adjustment for age, BMI, and THaBMD. In the age ≧ 80 group, no statistically significant correlation was found between all hip muscle parameters and fracture types. In contrast, in the age < 80 group, interestingly, after adjustment for age, BMI, and THaBMD, the associations between G.MaxM density, G.MaxM area, G.Med/MinM density, and G.Med/MinM area and fracture type were all statistically significant.
**Conclusion:** Our results indicate that in older women, especially under 80 years of age, gluteus muscle parameters are related to trochanteric fractures.
**Limitations:** Firstly, this study features a cross-sectional design, and subsequent longitudinal cohort studies are warranted to further investigate the relationship between gluteal muscles and fracture types. Secondly, in the measurement, we chose to measure the healthy side to replace the data on the fractured side, which may be biased.
**Ethics committee approval:** This study was approved by the ethics committee of Beijing Jishuitan Hospital.
**Funding for this study:** This work is supported in part by the National Key Research and Development Program of China (No. 2020YFC2004902), National Key R&D Program of China (2021YFC2501700), National Natural Science Foundation of China (grant no. 81971617), Beijing Hospitals Authority Youth Programme (code: 20200402), and Beijing Hospitals Authority Clinical Medicine Development of Special Funding Support (code: ZYLX202107).
**Author Disclosures:**
Aihong Yu: Nothing to disclose
Pengju Huang: Nothing to disclose
Yufeng Ge: Nothing to disclose
Xiaoguang Cheng: Nothing to disclose
Ling Wang: Nothing to disclose

### RPS 1610-4

**Long-term active MRI-surveillance of low-grade central cartilage tumours of the long bones: towards an optimal assessment of imaging characteristics**

*J. De Rooy*, C. Deckers, D. Koopmanschap, E. Dierselhuis, B. Schreuder, I. Van der Geest, M. Prokop; Nijmegen/NL
*(jacky.derooy@radboudumc.nl)*

**Purpose:** Determining MRI characteristics that contribute to the understanding of the natural course of low-grade central cartilage tumours (CCTs) of the long bones during long-term active MRI-surveillance.
**Methods or Backround:** 92 patients (31 male, 61 female) with 92 low-grade CCTs with a minimum MR imaging follow-up of 48 months (range: 48-174, mean: 74.7) and at least one baseline and one follow-up MRI were retrospectively included. Patient age at diagnosis was 20 to 76 years (mean: 50.4 years); no aggressive MR features (cortical destruction, soft tissue mass) were present and no biopsy had been performed. MRI characteristics (size, cortical scalloping < or ≥ 10% of circumference, intralesional fat entrapment (FE); fatty replacement (FR) only on last MRI) were scored and CCTs were labelled as in regression (R; increasing FE and/or FR, decreasing size (>3mm), in progression (P; loss of FE and/or increasing size (>3mm), increasing scalloping) or stable (S).
**Results or Findings:** The majority of low-grade CCTs were labelled S or R (81/92, 88%); 68 out of 92 patients showed initial FE (74%) and 29/68 also showed FR. Out of 24 patients without initial FE, 13 (54%) developed FE over time and 8/13 also developed FR. Out of 11 patients (mean 33.4 years, 20-56 years) with growth (P, 12%), 6 showed initial FE (2/6 developing FR) and 4 developed FE (3/4 also developing FR). 1 CCT with developing FE showed new scalloping. Only one CCT showed growth without FE. None developed aggressive MRI characteristics.
**Conclusion:** Active MRI-surveillance can be safely recommended for low-grade CCTs of the long bones as none developed aggressive MRI characteristics during long-term follow-up. MRI characteristics FE and FR might predict benign behaviour of CCTs and should be assessed on follow-up MRI.
**Limitations:** This is a retrospective study.
**Ethics committee approval:** This study was approved by the institutional Review Board of Radboud University Medical Center.
**Funding for this study:** No funding was received for this study.
**Author Disclosures:**
Desirée Koopmanschap: Nothing to disclose
Ingrid Van der Geest: Nothing to disclose
Claudia Deckers: Nothing to disclose
Mathias Prokop: Nothing to disclose
Edwin Dierselhuis: Nothing to disclose
Jacoba De Rooy: Nothing to disclose
Bart Schreuder: Nothing to disclose

### RPS 1610-5

**Metal artifact reduction strategies for clinical photon counting computed tomography of total hip arthroplasty: a volumetric quantitative and qualitative phantom study**

*R. P. Marcus*, G. C. Feuerriegel, A. A. Marth, D. Nanz, R. Sutter; Zurich/CH
*(roy.marcus@outlook.com)*

**Purpose:** The purpose of this study was to systematically evaluate the impact of various scanning and reconstruction modes on both metal artifact volume and overall image quality in the context of a hip prosthesis phantom, acquired on a first generation clinical photon-counting detector computed tomography (PCCT).

**Methods or Backround:** A total hip prosthesis phantom was scanned on a PCCT (Naeotom Alpha, Siemens Healthineers) employing four distinct modes at 140 kV and constant dose of 7 mGy: Quantumplus (Q+), UHR-Quantumplus (UHR-Q+), QuantumSn (Q-Sn) and UHR-QuantumSn (UHR-Q-Sn); Sn = tin filter. Polychromatic and virtual monoenergetic images (VMI) were reconstructed with and without iterative metal artifact reduction (iMAR, Siemens Healthineers). Artifacts were quantified using a 3D printing software and image quality was evaluated by two radiologists.
**Results or Findings:** Tin filter reduced artifact volume in polychromatic reconstructions by 14% (298 ml (Q-Sn) vs 347 ml (Q+) and 310 ml (UHR-Q-Sn) vs 360 ml (UHR-Q+)). iMAR reduced the metal artifact volume by 46 – 57% with UHR-Q+ images achieving the lowest artifact volume at 150 ml. In VMI, the smallest total artifact volume was quantified at 130 keV with Q+ (150 ml) and UHR-Q+ (164 ml), at 120 keV with Q-Sn (169 ml) and UHR-Q-Sn (172 ml). iMAR reduced artifact volume in VMI: 130 ml in Q+ (150 keV), 140 ml in UHR-Q+ (160 keV), 134 ml in Q-Sn (150 keV) and 140 ml (UHR Q-Sn at 190 keV). Best subjective image quality was achieved for VMI Q+ with iMAR (65 keV), polychromatic UHR-Q+ with iMAR, VMI Q-Sn with iMAR (100 keV), polychromatic Q-Sn with iMAR, VMI UHR-Q-Sn (100 keV) and polychromatic UHR Q-Sn.
**Conclusion:** VMI or polychromatic images using tin filter, UHR and iMAR achieve the strongest artifact reduction and best image quality.
**Limitations:** This was a single phantom size study.
**Ethics committee approval:** This was a phantom study, hence no ethics committee approval was needed.
**Funding for this study:** This study received in house funding.
**Author Disclosures:**
Adrian Alexander Marth: Nothing to disclose
Daniel Nanz: Nothing to disclose
Reto Sutter: Nothing to disclose
Georg Constantin Feuerriegel: Nothing to disclose
Roy P. Marcus: Nothing to disclose

### RPS 1610-6

**Evaluation of lower extremity skeletal muscle fibrosis in peripheral arterial disease (PAD) using extracellular volume fraction (ECV) from dual-layer spectral CT**

*N. Ding*, L. Zhou, X. Zhang, Y. Han, Y. Cui, X. Huang, P. Cao, X. Zhang, J. Yang; Xi'an/CN

**Purpose:** Objectives Peripheral arterial disease (PAD) is characterised by arterial occlusion and fibrosis in the lower extremities. While extracellular volume fraction (ECV) obtained from dual-layer spectral CT has been established as a biomarker for myocardial fibrosis, its application in assessing lower extremities affected by PAD remains unexplored. This study aimed to assess the clinical feasibility of employing ECV for quantifying calf muscle fibrosis and comparing it between normal controls (NC) and PAD patients.
**Methods or Backround:** From October 2022 to February 2023, we recruited patients with PAD as well as sex and age-matched diabetics without peripheral arterial disease (ankle/brachial index ＞0.9, and＜1.3 ) as a control group. All participants underwent late iodine enhancement on lower extremity dual-layer spectral CT to determine the ECV of their calf muscles and the differences between the NC and PAD groups.
**Results or Findings:** A total of 53 patients (20 in the NC group and 33 in the PAD group) were recruited. The NC group exhibited significantly lower mean ECV compared to the PAD group (19.05% vs 28.89%, respectively, P < 0.001). Among PAD patients, those with collateral vessels had a slightly lower mean ECV than those without (26.89% vs 30.82%, respectively, P = 0.042). Additionally, patients with intermittent claudication in the PAD group had lower mean ECV values compared to patients with critical limb ischaemia (26.98% vs 33.52%, respectively, P = 0.028).
**Conclusion:** The evaluation of skeletal muscle fibrosis in PAD using ECV obtained from dual-layer spectral CT is both feasible and informative. ECV serves as a valuable tool in identifying the severity of PAD and assessing collateral vessel formation in affected patients.
**Limitations:** No limitations were identified.
**Ethics committee approval:** The study was approved by the ethics committee of the First Affiliated Hospital (ethics approval number: XJTUIAF2021LSY-223).
**Funding for this study:** No funding was received for this study.
**Author Disclosures:**
Xin Huang: Nothing to disclose
Pan Cao: Nothing to disclose
Yaqing Han: Nothing to disclose
Xiaoyue Zhang: Nothing to disclose
Xiaodi Zhang: Nothing to disclose
Yuling Cui: Nothing to disclose
Li Zhou: Nothing to disclose
Ningning Ding: Nothing to disclose
Jian Yang: Nothing to disclose

### RPS 1610-7

**Monostotic metachronous osteoid osteoma**

*S. Battisti*, G. Bisinella; Monselice/IT
*(dott.sarabattisti@gmail.com)*

**Purpose:** Osteoid osteoma (OO) is a benign bone forming tumour, which very infrequently has multifocal or multicentric presentation. Multiple nidi may be present close to each other in a single bone or in adjacent bones, or may be present in separate bones in multicentric lesions. We report five unusual cases of double localisation of osteoid osteoma in the same bone after a mean of three years interval (1-5 years). Further studies may establish a systemic etiology and associated morbidities.
**Methods or Backround:** From 2008, we treated 83 cases of osteoid osteoma with CT-guided radiofrequency thermal ablation. We used a 17 gauge needle; the RF was activated for 8 minutes using a temperature of 90 degrees C°. From 2016 to 2023 we retrospectively evaluated all cases with pain relapse after treatment. We found 8 patients (9.6%) with pain relapse out of a total of 83.
**Results or Findings:** The analysis of CT images showed that in 5 cases (62%) there was a second nidus close to the first one.
**Conclusion:** In conclusion, this is the first series of both monostotic and metachronous OO, also associated with pain relapse. Our case highlights the importance of considering a diagnosis of double nidus.
**Limitations:** The limitation of our retrospective study is the small sample size. Further studies with a larger sample size are needed to confirm our results.
**Ethics committee approval:** This case report was approved by our Institutional review board and patients' informed consent was obtained.
**Funding for this study:** This research did not receive any specific grant from funding agencies in the public, commercial, or not-for-profit sectors.
**Author Disclosures:**
Sara Battisti: Nothing to disclose
Gianluca Bisinella: Nothing to disclose

### RPS 1610-8

**Hip MRI in flexion abduction external rotation (FABER) position for assessment of the ischiofemoral interval in patients with hip pain: a feasibility study**

A. Heimann[1], T. D. Lerch[2], M. Tannast[1], S. Steppacher[2], M. Wagner[3], E. Schmaranzer[3], *F. Schmaranzer*[4]; [1]Fribourg/CH, [2]Bern/CH, [3]Sankt Johann in Tirol/AT, [4]Zurich/CH
*(florian.schmaranzer@insel.ch)*

**Purpose:** The purpose of this study was to assess the feasibility of flexion-abduction-external rotation (FABER) magnetic resonance imaging (MRI) of the hip to visualise changes in the ischiofemoral interval.
**Methods or Backround:** This is an IRB-approved retrospective single-centre study. Patients underwent non-contrast 1.5 T hip MRI in the neutral and FABER position. Two readers measured the ischiofemoral interval at three levels: proximal-/distal intertrochanteric distance and ischiofemoral space. Subgroup analysis was performed for hips with (>30°) /without high femoral torsion (<30°), or quadratus femoris muscle oedema (QFME), respectively. A receiver operating curve with calculation of the area under the curve (AUC) for prediction of QFME was calculated.
**Results or Findings:** 110 patients (121 hips, mean age 34 ± 11 years, 67 females) were evaluated. FABER-MRI led to narrowing (both P < 0.001) of the ischiofemoral interval, which decreased more at the proximal (mean decrease by 26 ± 7 mm) than at the distal (6 ± 7 mm) intertrochanteric ridge. With high femoral torsion/QFME, the ischiofemoral interval was significantly narrower at all three measurement locations compared to normal torsion/no QFME (P <0.05). Accuracy for predicting QFME was high with an AUC of 0.89 (95% CI 0.82 – 0.94) using a threshold of ≤ 7 mm for the proximal intertrochanteric distance.
**Conclusion:** Hip MRI in the FABER position is feasible, visualises narrowing of the ischiofemoral interval and may be helpful in diagnosing ischiofemoral impingement.
**Limitations:** This is a feasibility study; the value of MRI in FABER position needs to be further assessed in a large group of patients with ischiofemoral impingement.
**Ethics committee approval:** This study was approved by the medical university of Innsbruck.
**Funding for this study:** This study was partially funded by the Swiss national science foundation.
**Author Disclosures:**
Till Dominic Lerch: Nothing to disclose
Florian Schmaranzer: Research/Grant Support: Siwss national science foundation
Ehrenfried Schmaranzer: Nothing to disclose
Simon Steppacher: Research/Grant Support: Swiss national science foundation
Alexander Heimann: Nothing to disclose
Moritz Wagner: Nothing to disclose
Moritz Tannast: Nothing to disclose

### RPS 1610-9

**Are degenerative findings detected on traction MR arthrography of the hip associated with failure of arthroscopic femoroacetabular impingement surgery?**

T. D. Lerch[1], A. Heimann[2], M. Wagner[3], P. Vavron[3], S. Steppacher[1], M. Tannast[2], E. Schmaranzer[3], *F. Schmaranzer*[4]; [1]Bern/CH, [2]Fribourg/CH, [3]Sankt Johann in Tirol/AT, [4]Zurich/CH
*(florian.schmaranzer@insel.ch)*

**Purpose:** The purpose of this study was to identify preoperative degenerative features on traction MR arthrography associated with failure after arthroscopic femoroacetabular impingement (FAI) surgery.
**Methods or Backround:** This was a retrospective study including 102 patients (107 hips) undergoing traction magnetic resonance arthrography (MRA) of the hip at 1.5T and subsequent hip arthroscopic FAI surgery performed from January 2016 to February 2020, with complete follow-up. Clinical outcomes were assessed using the International Hip Outcome Tool (iHOT-12) score. Clinical endpoint for failure was defined as an iHOT-12 of <60 points or conversion to total hip arthroplasty. MR images were assessed by two radiologists for presence of 9 degenerative lesions including osseous, chondro-labral/ ligamentum teres lesions. Uni- and multivariate Cox regression analysis was performed to assess the association between MRI findings and failure of FAI surgery.
**Results or Findings:** Of the 107 hips, 27 hips (25%) met at least one endpoint at mean 3.7 ± 0.9 years follow-up. Osteophytic changes of femur or acetabulum (hazard ratio [HR] 2.5 - 5.0), acetabular cysts (HR 3.4) and extensive cartilage (HR 5.1) - and labral damage (HR 5.5) >2 hours on clockface were univariate risk factors (all P <0.05) for surgery failure. Three risk factors for failure were identified in the multivariate analysis: Acetabular cartilage damage > 2 hours on the clockface (HR 3.2, P = 0.01), central femoral osteophyte (HR 3.1, P = 0.02), and femoral cartilage damage with ligamentum teres damage (HR 3.0, P = 0.04).
**Conclusion:** Evaluation of negative predictors on preoperative traction MR arthrography holds the potential to improve risk stratification based on the already present joint degeneration ahead of FAI surgery.
**Limitations:** No comparison was possible to non-contrast MRI of the hip or direct MR arthrography without traction.
**Ethics committee approval:** The institutional review board of the medical university of Innsbruck approved this study.
**Funding for this study:** This study was partially funded by the Swiss national science foundation.
**Author Disclosures:**
Till Dominic Lerch: Nothing to disclose
Florian Schmaranzer: Research/Grant Support: Institutional funding from the Swiss national science foundation
Ehrenfried Schmaranzer: Nothing to disclose
Simon Steppacher: Research/Grant Support: Institutional fuding from the Swiss national science foundation
Alexander Heimann: Nothing to disclose
Moritz Wagner: Nothing to disclose
Moritz Tannast: Nothing to disclose
Peter Vavron: Nothing to disclose

### RPS 1610-10

**Decentration of the femoral head: is it associated with osseous deformities predisposing to hip instability?**

*F. Schmaranzer*[1], A. Heimann[2], S. Steppacher[3], M. Tannast[2], T. D. Lerch[3]; [1]Zurich/CH, [2]Fribourg/CH, [3]Bern/CH
*(florian.schmaranzer@insel.ch)*

**Purpose:** To assess the prevalence of "decentration of the femoral head" on different imaging planes and compare it between hip deformities.
**Methods or Backround:** IRB-approved retrospective diagnostic study in 351 patients (48% men, mean age 31 ± 12 years) with hip pain undergoing radiography and direct hip MR arthrography at 1.5T (sagittal-, axial-, and radial- PD-w TSE images). On radiographs, lateral centre edge angle and neck-shaft angle were measured. On MRI, measurements of femoral torsion were performed at the level of the lesser trochanter. The presence of decentration was defined as a layer of contrast agent between the posterior femoral and acetabular cartilage layer. The prevalence of decentration sign was compared between sagittal, axial, and radial images and between hips with/without hip dysplasia (lateral centre edge angle, LCE <25°), coxa antetorta (>39°) and valgus hips (neck-shaft angle >139°).
**Results or Findings:** Decentration was detected with the highest (P <0.001) frequency on radial (28%), followed by axial (13%) and sagittal (5%) images. Hips with decentration sign had a lower LCE angle (24 ± 9° vs 32 ± 7°; P <0.001), higher neck-shaft angle (134 ± 7° vs 131 ± 6°; P <0.001) and higher femoral torsion (24 ± 15° vs 16 ± 12°; P <0.001) than hips without decentration sign. Hips with decentration had a higher prevalence and were associated with hip dysplasia (56% vs 17%, OR of 6.4; P <0.001), coxa antetorta (18% vs 5%, OR of 4.2; P <0.001) and valgus deformity (24% vs 8%, OR of 3.8; P <0.001) compared to hips without decentration sign on MRI.
**Conclusion:** Decentration sign of the femoral head was most frequently detected on radial MR arthrography and was associated with osseous deformities predisposing to hip instability.
**Limitations:** The prevalence of decentration signs is unknown in asymptomatic volunteers.
**Ethics committee approval:** Institutional review board approval was granted by the University of Bern.
**Funding for this study:** This study was partially funded by the Swiss National Science Foundation.
**Author Disclosures:**
Till Dominic Lerch: Nothing to disclose
Florian Schmaranzer: Research/Grant Support: Institutional funding from the Swiss national science foundation
Simon Steppache: Research/Grant Support: Instituinal funding from the Swiss national science foundation
Alexander Heimann: Nothing to disclose
Moritz Tannast: Nothing to disclose

### RPS 1610-11

**The overlooked symptom coccydynia: evaluation of sacrococcygeal morphologic and morphometric findings**

*A. Şükün*[1], H. S. Öztoprak Çubuk[2], T. Cankurtaran[2], B. Yavuz[2], B. Yağdıran[2], C. Incekas[2]; [1]Antalya/TR, [2]Ankara/TR
*(abdullah.sukun@gmail.com)*

**Purpose:** Coccydynia is one of the most overlooked symptoms in daily clinical practice. Definitions for radiologic evaluation are controversial.
We aim to compare the morphology and morphometric measurements of the sacrococcygeal region with those of a healthy population to support radiologic decision making.
**Methods or Backround:** 26 traumatic and 50 idiopathic patients and 74 healthy controls were compared. The morphologic type of the coccyx, the presence of fusion, and the number of coccygeal segments were evaluated in both groups. Morphometric parameters such as sacrococcygeal angle (SCA), sacrococcygeal joint angle (SCJA), intercoccygeal angle (ICA), sacral slope (SS), coccyx curved length (CCL), sacrum curved length (SCL), coccyx length (CL), sacrum length (SL), and sacrococcygeal total length (SCTL) were investigated.
**Results or Findings:** Significant differences were found between the Coccydynia group and the healthy control group in morphologic parameters such as female gender, coccyx segment, coccyx morphology, presence of sacrococcygeal joint, and segment of sacrococcygeal joint fusion (p <0.05). In morphologic measurements, sacrococcygeal joint angle, sacrum curved length, sacrum length, coccyx, and sacrum curvature indexes were significantly increased (P <0.05). No significant difference was found in the morphologic and morphometric parameters evaluated when compared with the duration of coccydynia (P >0.05).
**Conclusion:** An increase in sacrococcygeal joint angle (SCJA), sacrum curved length (SCL), sacrum length (SL), sacrum curvature index (SCI), and coccyx curvature index (CCI) measurements predisposes to coccydynia. It would be more accurate to perform a radiologic evaluation by becoming familiar with these morphologic and morphometric parameters.
**Limitations:** The limitations of our study are that it is single-centre and retrospective. In our study, CT and MR images of healthy populations were compared and certain static parameters were evaluated. However, coccydynia is a dynamic pathology and dynamic radiological radiographs are needed for evaluation.
**Ethics committee approval:** This study was approved by the Institutional Review Board of Baskent University (Project no: KA23/07).
**Funding for this study:** No funding was received for this study.
**Author Disclosures:**
Burak Yağdıran: Nothing to disclose
Busra Yavuz: Nothing to disclose
Caner Incekas: Nothing to disclose
Tijen Cankurtaran: Nothing to disclose
Hazal Selvi Öztoprak Çubuk: Nothing to disclose
Abdullah Şükün: Nothing to disclose

### Saturday, March 2

08:00-09:00 **Research Stage 1**

### Research Presentation Session: Artificial Intelligence & Machine Learning & Imaging Informatics

### RPS 1705

### Radiomics applications in MDCT

**Moderator**
R. Mirón Mombiela; Herlev/DK
*(mirona@ufm.edu)*

### RPS 1705-2

**A reference framework for standardisation and harmonisation of CT radiomics features: the "CadAIver" analysis**

*R. Levi*[1], M. Mollura[2], G. Savini[1], F. Garoli[1], M. Battaglia[1], A. Ammirabile[1], L. A. Cappellini[1], R. Barbieri[2], L. S. Politi[1]; [1]Pieve Emanuele/IT, [2]Milan/IT
*(riccardo.levi@st.hunimed.eu)*

**Purpose:** The purpose of this study was to quantify the effects of different dose protocols, reconstruction algorithms, fields of view (FOVs) and CT scanners on radiomics features (RFs) of the lumbar vertebrae in a cadaveric trunk and to develop a normalisation algorithm to harmonise radiomics analyses.
**Methods or Backround:** We performed a total of 112 CT acquisitions of a cadaveric trunk on 3 different CT scanners from 2 different vendors, using varying kV (80-140) and mA (250-400). Each acquisition was performed using 2 FOVs (Abdomen/Spine) and two reconstruction kernels (Standard/Bone). Lumbar vertebrae were segmented using a convolutional neural network and RFs were extracted using pyradiomics. Intra/Inter-scanner analyses were assessed and each RF was tested using a generalised linear model (GLM) to assess the effects of all the above-mentioned CT acquisition parameters. GLM model was employed to standardise RFs across different acquisitions, and was compared to the ComBat algorithm, using 10-folds cross-validation (CV) R2.
**Results or Findings:** KV variation showed the highest feature modification in intra/inter-scanner analyses, with the FirstOrder features showing high variability (94.4%, Scanner 1). Little effects were evident upon mA variation. 100% of Shape features on all scanners were found significantly dependent on FOV, and 83% of GLSZM were statistically different between reconstruction kernels. The proposed GLM algorithm obtained a mean R2 across CV higher than 0.90 in 21 Radiomics features (19.6%), whereas ComBat obtained an high R2 value in 1 Radiomics feature (0.90%). GLM was statistically superior in 39 Radiomics features in respect to ComBat.
**Conclusion:** This study is the first attempt in describing the effects of CT acquisition parameters on RFs from cadaveric donor. Current and Voltage affect the RFs in different ways. GLM was superior to ComBat in normalising RFs across all different CT acquisitions.
**Limitations:** This study only featured vertebral analysis.
**Ethics committee approval:** Ethics committee approval is not required for the use of cadaveric body parts. The cadaver was donated willingly to science and obtained from medcure.org (Medcure, Orlando, FL, USA).
**Funding for this study:** No funding was received for this study.
**Author Disclosures:**
Riccardo Barbieri: Nothing to disclose
Luca Alessandro Cappellini: Nothing to disclose
Giovanni Savini: Nothing to disclose
Angela Ammirabile: Nothing to disclose
Letterio Salvatore Politi: Nothing to disclose
Federico Garoli: Nothing to disclose
Massimiliano Battaglia: Nothing to disclose
Maximiliano Mollura: Nothing to disclose
Riccardo Levi: Nothing to disclose

### RPS 1705-3

**Development of a radiomic model to predict the risk of hepatocellular carcinoma in cirrhotic patients**

*L. Leo*, S. Schirò, O. Bazzini, F. Cazzato, D. Stefanelli, M. V. Bazzocchi, G. Milanese, G. Missale, N. Sverzellati; Parma/IT
*(ludovica.leo@unipr.it)*

**Purpose:** Patients with cirrhosis are at increased risk of hepatocellular carcinoma (HCC); clinical and radiological scores for predicting the risk of developing HCC are currently unknown. The aim of the study is to assess if radiomics can identify cirrhotic patients at risk of HCC.
**Methods or Backround:** 98 subjects (M:F = 64:34; mean age 67 years ±9.81) were included in this retrospective monocentric study. Two groups were identified: group (a) with 49 patients (M:F=31:18; mean age 67.4 years ±9.12) who had a baseline CT with radiological signs of cirrhosis and follow-up CTs without evidence of HCC (LI-RADS 1-3); group (b) with 49 patients (M:F = 33:16; mean age 67.5 years ±10.5) who had a baseline CT with radiological signs of cirrhosis and evidence of HCC in one follow-up CT scan (LI-RADS 4/5). Four radiologists (three years of experience) provided complete liver segmentations by manually drawing volumes of interest (VOI) on non-enhanced baseline CT scans, extracting 851 radiomic features (RF). 100 train:test (0.7:0.3) splits were created and recursive feature elimination with a 5-fold cross-validation was performed on train partitions using the random forest classifier (RFC). Subsequently, RFC was trained by selecting iteratively an increasing number of features sorted by their occurrences to evaluate the minimum number of informative features. Finally, means and 95% confidence intervals of accuracy, sensitivity, specificity, precision, area under the receiver operating characteristic curve (ROC-AUC) were calculated on the test partitions.
**Results or Findings:** The best model exploited two features (wavelet-LLH_glcm_DifferenceAverage, wavelet-HLH_gldm_DependenceVariance), reaching accuracy, sensitivity, specificity, precision and ROC-AUC of 0.72 [0.70-0.73], 0.76 [0.74-0.79], 0.68 [0.65-0.70], 0.71 [0.69-0.73] and 0.78 [0.76-0.79], respectively.
**Conclusion:** Our radiomic model, evaluated in cross-validation, predicted the development of HCC with satisfactory performances and was more sensitive than specific.
**Limitations:** There was a monocentric retrospective cohort and no external validation.
**Ethics committee approval:** This study was approved by an ethics committee; 930/2022/OS/UNIPR (Protocol 5853 07/02/2023).
**Funding for this study:** No funding was received for this study.
**Author Disclosures:**
Francesca Cazzato: Nothing to disclose
Ludovica Leo: Nothing to disclose
Daniela Stefanelli: Nothing to disclose
Nicola Sverzellati: Nothing to disclose
Gabriele Missale: Nothing to disclose
Maria Vittoria Bazzocchi: Nothing to disclose
Silvia Schirò: Nothing to disclose
Gianluca Milanese: Nothing to disclose
Olimpia Bazzini: Nothing to disclose

### RPS 1705-4

**Influence of CT scanners on radiomics features in abdominal CT: a multicentre phantom study**

C. Aberle[1], M. Bach[1], O. Jimenez-del-Toro[2], R. Schaer[2], K. Flouris[3], E. Konukoglu[3], H. Müller[2], A. Depeursinge[2], *M. Obmann*[1]; [1]Basel/CH, [2]Sierre/CH, [3]Zurich/CH

**Purpose:** The aim of this study was to investigate the influence of different CT scanners on the stability and discriminative power of radiomics features using an anatomically accurate 3D-printed abdominal phantom.
**Methods or Backround:** Based on a patient's CT scan with multiple hepatic lesions, an anatomically and texturally realistic phantom was commercially 3D-printed using potassium-iodide ink on paper. The phantom was scanned on 13 CT scanners by 4 different manufacturers at 8 institutions with 10 scan repetitions each. A harmonised clinical oncologic CT acquisition protocol was used on all scanners. Images were reconstructed using iterative reconstruction algorithms. 86 radiomics features were assessed for six different ROIs (metastasis, haemangioma, 2 cysts, 2 normal liver parenchyma regions) using principal component analyses (PCA) and Kruskal-Wallis tests.
**Results or Findings:** For all ROIs, PCA analyses clearly showed clustering by scanners and manufacturers, with the same scanner models overlapping. Kruskal-Wallis tests for each ROI and radiomics feature showed significant differences between scanners in 511 of 516 tests ($P < 0.05$). Pairwise ROI comparison in the PCA showed both separation of the 13 different CT scanners and of the ROIs, while the separation between ROIs was stronger than between scanners.
**Conclusion:** In this multicentre study, radiomics features are impacted by CT scanner models in varying degrees, despite the use of matched acquisition and reconstruction parameters. When performing multicentre studies, an a priori phantom analysis and feature harmonisation techniques may be used to account for these influences and select more stable radiomics features.
**Limitations:** As the phantom includes 1-2 ROIs per tissue type, variability of the same tissue type was not studied and results of ROI separation may not be fully generalisable to tissue type classification. Patient motion cannot be assessed with this phantom and may aggravate interscanner variations.
**Ethics committee approval:** This was a phantom study.
**Funding for this study:** This work was partly supported by the Swiss Personalised Health Network with the QA4IQI Quality assessment for interoperable quantitative computed tomography imaging project DMS2445

and the IMAGINE project. It was also partially supported by the Swiss National Science Foundation (grant 205320_179069).
**Author Disclosures:**
Roger Schaer: Nothing to disclose
Markus Obmann: Nothing to disclose
Ender Konukoglu: Nothing to disclose
Michael Bach: Nothing to disclose
Adrien Depeursinge: Nothing to disclose
Kyriakos Flouris: Nothing to disclose
Christoph Aberle: Nothing to disclose
Henning Müller: Nothing to disclose
Oscar Jimenez-del-Toro: Nothing to disclose

### RPS 1705-5

**Radiomics and machine learning for the assessment of renal tumour histological subtypes on multiphase computed tomography: a multicentre trial with independent testing**

*S. Bachanek*[1], A. Leha[1], P. Zeuschner[2], A. Massmann[3], L. Trojan[1], J. Uhlig[1], J. Lotz[1], A. Uhlig[1]; [1]Göttingen/DE, [2]Homburg/DE, [3]Stuttgart/DE
*(sophie.bachanek@med.uni-goettingen.de)*

**Purpose:** The purpose of this study was to distinguish histological subtypes of renal tumours identified on multiphase computed tomography (CT) using radiomic features and machine learning in a multicentre setting.
**Methods or Backround:** Patients undergoing surgical resection and histopathological assessment of renal tumours at two tertiary urological centres between 2012 and 2022 were retrospectively included. Preoperative arterial and venous phase CTs from multiple referring imaging centres were segmented and standardised radiomic features extracted. After preprocessing and class imbalance, an extreme gradient boosting tree-based (XGB) machine learning (ML) algorithm was used to predict renal tumour subtypes using 10-fold cross-validation, assessed as multiclass AUC. ML algorithms were trained on data from one centre, and independently tested on data from the other centre.
**Results or Findings:** The training cohort comprised n=297 patients (n=191 ccRCC, n=40 pRCC, n=22 chRCC, n=28 oncocytomas, and n=16 AML), and the testing cohort n=121 patients (n=68/ n=20/ n=4/ n=26/ n=3). The XGB algorithm demonstrated a diagnostic performance of AUC=0.81/ 0.64/ 0.8 for venous / arterial / combined contrast phase CT in the training cohort, and AUC=0.75/ 0.67/ 0.75 in the independent validation cohort. In pairwise comparisons, the lowest diagnostic accuracy was evident for identification of oncocytomas (AUC=0.57-0.69), and the highest for identification of AMLs (AUC=0.9-0.94).
**Conclusion:** Radiomic feature analyses acquired from clinical routine CT yield robust results for renal tumour assessment. For renal tumour subtype discrimination, venous phase CT yields the most pertinent imaging information, without evident diagnostic benefit of an added arterial contrast phase. Among all renal tumours, oncocytomas are hardest to differentiate using CT.
**Limitations:** Limitations of the study mainly include the heterogeneity of renal tumour subtypes in the training and testing cohort with occasional resulting low case numbers in the subgroups.
**Ethics committee approval:** The study received ethical approval by the ethics committee at Goettingen University (No. 2/4/17) and at Saarland University (No. 67/19).
**Funding for this study:** No funding was received for this study.
**Author Disclosures:**
Annemarie Uhlig: Nothing to disclose
Lutz Trojan: Nothing to disclose
Andreas Leha: Nothing to disclose
Sophie Bachanek: Nothing to disclose
Johannes Uhlig: Nothing to disclose
Joachim Lotz: Nothing to disclose
Alexander Massmann: Nothing to disclose
Philip Zeuschner: Nothing to disclose

### RPS 1705-6

**Overcoming data scarcity in radiomics/radiogenomics using synthetic radiomic features**

*M. Ahmadian*, Z. Bodalal, H. J. van der Hulst, C. Vens, L. Karssemakers, R. G. H. Beets-Tan, M. van den Brekel, J. A. Castelijns; Amsterdam/NL
*(m.ahmadian@nki.nl)*

**Purpose:** This study evaluates the potential of synthetic radiomic data generation to augment the performance of radiomics/radiogenomics prediction models.
**Methods or Backround:** This study was conducted on a retrospectively collected cohort of 386 colorectal cancer patients for whom matched contrast-enhanced CT images and TP53 mutational status were available. Five different tabular synthetic data generation models were used to generate synthetic radiomic data based on real-world radiomics extracted from our cohort. The quality and reproducibility of the generated synthetic radiomic data were assessed. Synthetic radiomics were then combined with real-world radiomic training data to evaluate their impact on the predictive model's performance.
**Results or Findings:** Using only real-world radiomic data, increasing training samples (n=200, 400, 1000, 2055 lesions) improved the model's predictive performance on the unseen test set (average AUC=0.52, 0.53, 0.56, and 0.64). Synthetic tabular data generation models created reproducible synthetic radiomic data with properties highly similar to real-world data (for n =1000 lesions, average Chi-square =0.932, average basic statistical correlation =0.844). Augmenting predictive models with synthetic radiomics enhanced their performance by 9.61%, 11.32%, 16.07%, and 3.22% for the outlined training sets, respectively. Synthetic radiomics derived from randomised/noisy radiomic data failed to enhance predictive performance, while true signal data was effectively amplified.
**Conclusion:** Synthetic radiomic data, when combined with real radiomics, can enhance the performance of predictive models. Tabular synthetic data generation might help overcome limitations in medical AI stemming from data scarcity.
**Limitations:** Our study was limited to a retrospective monocentric cohort. It would be beneficial to validate our findings on external cohorts, helping to ensure that the conclusions were not specific to the centre where the data was gathered.
**Ethics committee approval:** This study was approved by the Institutional Review Board (IRB): IRBd19-147.
**Funding for this study:** This work was made possible via a grant from the Hanarth Foundation. The computational infrastructure for the analysis was funded via the Maurits en Anna de Kock Stichting and the NVIDIA Academic GPU programme.
**Author Disclosures:**
Zuhir Bodalal: Nothing to disclose
Conchita Vens: Nothing to disclose
Milad Ahmadian: Nothing to disclose
Luc Karssemakers: Nothing to disclose
Jonas A. Castelijns: Nothing to disclose
Regina G. H. Beets-Tan: Nothing to disclose
Hedda Joanne van der Hulst: Nothing to disclose
Michiel van den Brekel: Nothing to disclose

### RPS 1705-7

**Impact of population size and validation method on the performance of radiomics models: application to COVID lung lesions**

*A. Decoux*, L. Duron, A. Arnoux, L. S. Fournier; Paris/FR

**Purpose:** The purpose of this study was to explore the impact of population size and validation strategy on the estimated performance and reproducibility of radiomics studies.
**Methods or Backround:** Radiomics parameters were extracted from lung lesions segmented by experts on CT in 3,737 COVID-19 patients (STOIC cohort). 1121 (33%) patients were set aside to simulate an external validation population ("generalisation set"). Among the remaining patients, subpopulations of varying sizes were generated to simulate the training/test population for the radiomics study. Prediction models were trained on 100 bootstrapped samples to estimate variance of the AUC, i.e. model stability. Three validation strategies were tested: one time split, cross-validation and nested cross-validation. The mean and variance of AUCs of each model was calculated on the subpopulation as well as on the "generalisation" set, and the difference was the generalisation gap.
**Results or Findings:** Increasing the size of the training data sets improved model performance on both internal validation and generalisation sets, decreased the variance of performance on the validation set and decreased the generalisation bias, thereby increasing overall confidence in the model, with a plateau at 400 patients. Cross-validation helped reduce variance and generalisation bias compared to one time split. Nested cross-validation reduced variance but at the expense of increased generalisation bias.
**Conclusion:** As expected, population size has a strong impact on model performance, particularly on the estimated performance variance (stability) of models. This study is the first to estimate the minimum population size needed to improve generalisability of radiomics studies. However, as it is applied to a single data set, results are expected to vary according to predictive power of imaging for a given clinical question.
**Limitations:** Our generalisation set serves as a surrogate for an external validation set, it doesn't constitute a true external validation set.
**Ethics committee approval:** This is a retrospective study.
**Funding for this study:** This work was funded by the French government under management of the Agence Nationale de la Recherche as part of the "Investissements d'avenir" programme, reference ANR19-P3IA-0001 (PRAIRIE 3IA Institute).
**Author Disclosures:**
Laure S. Fournier: Nothing to disclose
Loïc Duron: Nothing to disclose
Armelle Arnoux: Nothing to disclose
Antoine Decoux: Nothing to disclose

**08:00-09:00** **Research Stage 2**

### Research Presentation Session: Neuro

### RPS 1711

### CSF, glymphatic system and MR contrast agents

**Moderator**
M. A. Lucic; Sremska Kamenica/RS
*(milos.a.lucic@gmail.com)*

### RPS 1711-2

**In vivo CSF mobility mapping at ultra-high field MRI in patients with cerebral amyloid angiopathy**

*K. Deike*, A. Radbruch, G. Petzold; Bonn/DE

**Purpose:** Impaired perivascular clearance is a critical step in the pathogenesis of cerebral amyloid angiopathy (CAA), which is characterized by amyloid-beta accumulation in cerebral vessels. However, only few brain clearance imaging techniques are available that can be applied non-invasively in humans and the majority of assessed parameters is of indirect and/or semi-quantitative nature. Therefore, this study aimed to quantifiy the cerebrospinal fluid (CSF) mobility in the PVS of CAA patients and healthy controls (HC).
**Methods or Backround:** To assess perivascular CSF mobility, we applied a specifically for this task developed 7-Tesla MRI sequence for the first time in patients with a diagnosed brain clearance disorder. The CSF mobility maps of 8 CAA patients (according to Boston criteria 2.0) and 9 HCs were used to semi-automatically segment the PVS in the centrum semiovale (CSO) and to calculate the CSF flow within the PVS.
**Results or Findings:** The CAA and HC group did not differ significantly in terms of age and gender. However, CAA patients depicted a significantly higher PVS volume in the CSO compared to HC ($p < 0.01$). CSF mobility correlated negatively with PVS size in both groups ($p < 0.01$), which is in accordance with expectations from fluid dynamics. While absolute CSF mobility did not differ significantly between both groups, CAA patients revealed a significantly lower decrease in CSF mobility with increasing PVS size compared to HC ($p < 0.05$).
**Conclusion:** This study is the first providing proof of concept for clinical usage of brain clearance imaging with non-invasive CSF mobility mapping in humans and revealed altered brain clearance in enlarged PVS of CAA patients.
**Limitations:** CSF mobility measurements do not allow to draw conclusions on net CSF flow, flow direction or overall clearance capacity.
**Ethics committee approval:** Number 437/21
**Funding for this study:** This work was supported by the EU Joint Programme for Neurodegenerative Disease Research (JPND) and the Fondation Leducq (Transatlantic Network of Excellence 23CVD03).
**Author Disclosures:**
Katerina Deike: Nothing to disclose
Gabor Petzold: Nothing to disclose
Alexander Radbruch: Nothing to disclose

### RPS 1711-3

**DTI-ALPS sequences for the study of the glymphatic system in idiopathic normal-pressure hydrocephalus**

*F. M. Doniselli*, A. Gans, M. A. Broggi, V. Redaelli, M. Moscatelli, M. Verri, D. Aquino, R. Pascuzzo, M. Grisoli; Milan/IT
*(fabio.doniselli@gmail.com)*

**Purpose:** Idiopathic normal-pressure hydrocephalus (iNPH) is a neurodegenerative disorder present in more than 5% of the population over 80 years of age, and the treatment of choice is ventriculoperitoneal (VP) shunt surgery. Assessment of high-volume lumbar tap test (LTT) has sufficiently high positive predictive values to predict VP response. Radiologic signs of iNPH (Evans index, transcallosal angle, and disproportionately dilated subarachnoid space (DESH)) provide diagnostic morphologic features but are not predictive of response to surgery. More recent etiologic hypotheses of iNPH include a dysfunction of the glymphatic system (GS). DTI imaging targeting perivascular spaces (DTI-ALPS) has shown the presence of reduced perivascular diffusivity in iNPH patients, an indirect index of GS dysfunction. The purpose of our work is to evaluate DTI-ALPS as a predictor of VP response.
**Methods or Backround:** Between 2021 and 2023, 89 patients diagnosed with probable iNPH underwent LTT testing. Of these, 22 were enrolled in the study with pre- and post-LTT DTI-ALPS sequences. Fifteen patients responded positively to LTT test and 14 of them underwent VP. Among the 14 patients who underwent VP, 9 underwent 3-month MR follow-up. 7 healthy patients were included as a control group.
**Results or Findings:** 68% (15/22) of patients responded positively to LTT; of these 93% (14/15) underwent VP surgery with clinical benefit in 86% (12/14). The ALPS index in controls was higher than in iNPH patients ($P < 0.01$). The ALPS index pre-LTT (T0) was significantly higher in LTT-responsive patients ($P < 0.01$). The ALPS index (T0) correlated with clinical indices ($P < 0.01$), and these were significantly improved at T3 ($P < 0.05$).
**Conclusion:** ALPS index provides a noninvasive, reproducible, and reliable diagnostic and prognostic tool in the evaluation of the iNPH patient that can significantly improve surgical selection.
**Limitations:** There was only a small cohort of patients and this was a single-centre study.
**Ethics committee approval:** This study was approved by the internal ethics committee.
**Funding for this study:** This study received no funding.
**Author Disclosures:**
Marina Grisoli: Nothing to disclose
Alessandro Gans: Nothing to disclose
Fabio Martino Doniselli: Nothing to disclose
Domenico Aquino: Nothing to disclose
Marco Moscatelli: Nothing to disclose
Veronica Redaelli: Nothing to disclose
Morgan Aldo Broggi: Nothing to disclose
Riccardo Pascuzzo: Nothing to disclose
Mattia Verri: Nothing to disclose

### RPS 1711-4

**Intraindividual comparison between gadopiclenol (0.05 mmol/kg)- and gadobutrol (0.1 mmol/kg)-enhanced MRI in terms of brain metastases visualisation**

A. Pichiecchio[1], L. Loevner[2], G. Hutóczki[3], K. Dziadziuszko[4], C. Groden[5], C. Oppenheim[6], *L. M. Farina*[7]; [1]Borgarello/IT, [2]Philadelphia, PA/US, [3]Debrecen/HU, [4]Gdańsk/PL, [5]Mannheim/DE, [6]Paris/FR, [7]Pavia/IT
*(lisa.farina@mondino.it)*

**Purpose:** Gadopiclenol (Elucirem™, Guerbet) is a high relaxivity macrocyclic gadolinium-based contrast agent (GBCA), approved by the FDA and currently under review by the EMA. This study aimed to compare contrast-enhanced MRI with gadopiclenol at 0.05 mmol/kg and gadobutrol at 0.1 mmol/kg in terms of visualisation of brain metastases.
**Methods or Backround:** This is a post hoc analysis of the phase III PICTURE (gadoPIClenol for cenTral nervoUs system magnetic REsonance) study. A subpopulation of patients with brain metastases (N=46) who underwent two separate MRIs with gadopiclenol and gadobutrol was analysed. Lesion visualisation parameters (border delineation, internal morphology and contrast enhancement) were assessed by three off-site blinded readers. Percentage of enhancement (E%), lesion to background ratio (LBR) and contrast to noise ratio (CNR) were measured. Overall diagnostic preference was assessed in a global matched pairs fashion by three additional blinded readers.
**Results or Findings:** For all readers, and all visualisation parameters, the difference in mean of scores showed the non-inferiority of gadopiclenol to gadobutrol (lower limit of 95% CI between -0.04 and -0.30 depending on the reader, above the non-inferiority margin [-0.35]). There was no significant difference between the two GBCAs in terms of CNR, while a higher E% was observed with gadopiclenol for two of three readers ($P \leq 0.0097$), and a higher LBR for the three readers ($P \leq 0.0036$). Readers preferred images with gadopiclenol in 54% to 59% of evaluations, reported no preference for 13% to 24% of evaluations, and preferred images with gadobutrol in 22% to 30% of evaluations.
**Conclusion:** MRI with gadopiclenol at 0.05 mmol/kg is non-inferior to gadobutrol at 0.1 mmol/kg for brain metastases visualisation.
**Limitations:** This is a post-hoc analysis with a limited number of patients.
**Ethics committee approval:** As a post-hoc analysis, this study did not require ethics committee approval.
**Funding for this study:** This study was sponsored by Guerbet.
**Author Disclosures:**
Katarzyna Dziadziuszko: Investigator: Investigator in the phase III PICTURE study
Laurie Loevner: Investigator: Investigator in the phase III PICTURE study
Gábor Hutóczki: Investigator: Investigator in the phase III PICTURE study
Lisa Maria Farina: Nothing to disclose
Christoph Groden: Investigator: Investigator in the phase III PICTURE study
Anna Pichiecchio: Investigator: Investigator in the phase III PICTURE study
Catherine Oppenheim: Investigator: Investigator in the phase III PICTURE study

**RPS 1711-5**

**Quantification and assessment of the chemical form of residual gadolinium in the skin after repeated administration of gadolinium-based contrast agents in rats**

*A. Treu*[1], J. Boyken[2], J. Lohrke[2], G. Jost[2], U. Thuss[1], H. Pietsch[2]; [1]Wuppertal/DE, [2]Berlin/DE

**Purpose:** Gadolinium presence in the body has triggered intense research in recent years. Most preclinical studies determined the overall Gd concentrations, this study investigated the distribution and chemical form of the applied GBCAs in the skin using matrix-assisted laser desorption/ionisation mass spectrometry imaging (MALDI MSI).
**Methods or Backround:** Rats received either a single (1x0.6 mmol/kg) or multiple intravenous injections within 2 weeks (8x0.6 mmol/kg) of gadobutrol or saline. The Gd distribution and concentration in skin after multiple injections were measured after 5 days and 5 weeks by inductively coupled plasma-mass spectrometry (ICP-MS) and laser ablation-ICP-MS (LA-ICP-MS). The intact gadobutrol in the skin (MW 604.72 g/mol) was determined with MALDI MSI 5 days (multiple dose) and 1 week post injection (single dose). Neodymium- or holmium-butrol was used as internal standard for quantification in MALDI MSI.
**Results or Findings:** Total gadolinium skin concentration measured by ICP-MS at 5 days after 8x0.6 mmol/kg for gadobutrol was 2.7±1.1 nmol/g. Importantly, gadobutrol was detected within the dermis as intact Gd-chelate using MALDI MSI. There were no indications for dechelation, as the Gd quantification and distribution by LA-ICP-MS was very similar with the intact gadobutrol detected by MALDI MSI. Intact gadobutrol was predominantly detected in the sweat glands with an average of 4.1±1.1 compared to 5.0±1.2 nmol/g total gadolinium measured by LA-ICP-MS.
**Conclusion:** MALDI MSI showed the chemical form of residual gadolinium being intact Gd-chelate. No indications for dechelation of free gadolinium were observed. Gadobutrol levels in the skin measured as total gadolinium via LA-ICP-MS were low and almost completely eliminated within 5 weeks. After 5 days, intact Gd-chelate was localised in sweat glands, suggesting a potential elimination route via sweat.
**Limitations:** There are species differences in terms of skin physiology between rats and humans.
**Ethics committee approval:** LaGeSo Berlin approved this study (approval for in vivo studies).
**Funding for this study:** All authors were Bayer AG employees.
**Author Disclosures:**
Axel Treu: Employee: Employee of Bayer AG
Gregor Jost: Employee: Employee of Bayer AG
Uwe Thuss: Employee: Employee of Bayer AG
Jessica Lohrke: Employee: Employee of Bayer AG
Janina Boyken: Employee: Employee of Bayer AG
Hubertus Pietsch: Employee: Employee of Bayer AG

**RPS 1711-6**

**Potential of heavily T2-weighted fluid-attenuated inversion recovery sequence for detection of contrast agent**

*L-P. Schmill*, S. Seehafer, S. Aludin, N. Larsen, O. Jansen; Kiel/DE
*(lars-patrick.schmill@uksh.de)*

**Purpose:** After angiographic thrombectomy in acute stroke with partial cerebral infarction, gadolinium-based contrast agents (GBCA) are rarely detected in the cerebrospinal fluid (CSF) on T1-weighted sequences. Such disruptions of the blood-CSF and brain-CSF barriers would in principle also be expected in inflammatory or tumour diseases, although they have not yet been observed and further studied. In order to make the detection of gadolinium as sensitive as possible and to be able to detect a deviation from the appropriate inversion time of the CSF with only small amounts of gadolinium, we have modified the fluid-attenuated inversion recovery (FLAIR) technique.
**Methods or Backround:** A MRI phantom was prepared using a series of dilutions of GBCA in isotonic saline (1:250; 1:500; 1:1,000; 1:2,000; 1:4,000; 1:8,000; 1:16,000; 1:32,000; 1:64,000; 1:128,000; 1:256,000) and native controls of pure isotonic saline. Around the test tubes, the phantom was filled with agarose gel with the addition of $MnCl_2$ (0.08 mmol/l) to match MRI tissue properties of the brain parenchyma. A 3T MRI (Magnetom, Vida, Germany) was subsequently used to test the FLAIR sequence with different parameters of TR, TE and TI in order to obtain the most complete suppression of isotonic saline and agarose gel while allowing high sensitivity for the contrast agent contained.
**Results or Findings:** Optimal detection of gadolinium at a dilution of 1:256,000 was possible using a FLAIR with a TR of 9,000 ms, a TI of 1,800 ms and a TE of 650 ms. The signal intensity ratio of sample (44 SI) to isotonic saline (17 SI) was 2.59.
**Conclusion:** The highly T2-weighted FLAIR sequence is excellent for the detection of subtle amounts of contrast agent in the CSF.
**Limitations:** This study lacked translation to clinical examinations.
**Ethics committee approval:** The study was approved by the institutional review board and written informed consent was obtained from all participants.
**Funding for this study:** No funding was received for this study.
**Author Disclosures:**
Lars-Patrick Schmill: Nothing to disclose
Schekeb Aludin: Nothing to disclose
Svea Seehafer: Nothing to disclose
Olav Jansen: Nothing to disclose
Naomi Larsen: Nothing to disclose

**RPS 1711-7**

**Assessment of pharmacokinetics and safety of gadoquatrane in renally impaired patients and its dialysability using clinical trial data, modeling and simulation approaches and in-vitro data**

*B. M. Hofmann*[1], G. Sutter[1], T. Fadini[1], S. Klein[1], E. Vendel[2], P. Vis[2], S. Heitmeier[3], T. Frenzel[1], W. Ebert[1]; [1]Berlin/DE, [2]Leiden/NL, [3]Wuppertal/DE
*(birte.hofmann@bayer.com)*

**Purpose:** The aim of this study was to evaluate the pharmacokinetics (PK) and safety of the novel, tetrameric macrocyclic gadolinium-based contrast agent (GBCA) gadoquatrane, using clinical trial data with modeling & simulation (M&S) approaches and in vitro testing for dialysability assessment, leveraging the broad knowledge of the class of GBCAs avoiding exposure of vulnerable patient populations, i.e. with severe renal impairment or ESRD, in clinical trials.
**Methods or Backround:** An open-label, single-dose phase 1 study investigated PK (plasma PK, excretion until 7 days p.i. and up to 6 months p.i.) and safety in adult participants (8 per group, dose: 0.025mmol/kg, i.e. 0.1mmol Gd/kg): 1) Mildly impaired renal function (RF) (eGFR: 60-89mL/min/1.73m2), 2) Moderately impaired RF (eGFR: 30-59mL/min/1.73m2) 3) matched controls (normal RF: eGFR: ≥90mL/min/1.73m2). PK in patients with severely impaired RF (eGFR<30mL/min/1.73m2) was simulated using an established popPK model. Dialysability of gadoquatrane was investigated in human blood using a clinical dialysis system versus gadobutrol.
**Results or Findings:** Similar Cmax and volume of distribution (Vss/BW) independent of renal function were observed. Systemic exposure (AUC) increased and CL/BW decreased with increasing renal impairment. Within the 7 days collection interval, gadoquatrane excretion was essentially complete in all groups. Late measurements with a highly sensitive LLOQ confirmed continuous excretion of trace amounts. Gadoquatrane was safe and well tolerated in all groups. Simulated plasma PK in severely impaired patients showed expected further increase of AUC and decrease of CL/BW according to renal function. In vitro investigations confirmed dialysability of gadoquatrane as known for gadobutrol.
**Conclusion:** Gadoquatrane displays expected PK and safety in mild and moderate renal impairment versus matched controls. Simulation of PK in severe renal impairment and in vitro testing of dialysability allowed avoiding exposure of vulnerable patient populations in clinical trials.
**Limitations:** The number of participants with RF in the clinical study was limited.
**Ethics committee approval:** The study protocol was reviewed and approved by each of the two study site's IEC/IRB before the start of the study.
**Funding for this study:** This study received funding from the Bayer AG.
**Author Disclosures:**
Stefan Klein: Employee: Bayer AG
Tommaso Fadini: Employee: Bayer AG
Peter Vis: Consultant: Bayer AG
Stefan Heitmeier: Employee: Bayer AG
Thomas Frenzel: Employee: Bayer AG
Gabriele Sutter: Employee: Bayer AG
Esmée Vendel: Consultant: Bayer AG
Birte Maria Hofmann: Shareholder: Bayer AG Employee: Bayer AG
Wolfgang Ebert: Employee: Bayer AG

08:00-09:00 Research Stage 3

### Research Presentation Session: Physics in Medical Imaging

### RPS 1713
### Optimisation and its tools

**Moderator**
A. Kuchcinska; Warsaw/PL
*(akuchcinska@wp.pl)*

### RPS 1713-2
**CT acquisition parameter selection in the real world: impacts on paediatric radiation dose and variation amongst 155 institutions**
*Y. Wang*, P. Chu, C. Stewart, R. Smith-Bindman; San Francisco, CA/US
*(yifei.wang@ucsf.edu)*

**Purpose:** Computed tomography (CT) exams have expanded rapidly since introduction, such that theoretical understandings of best dose optimisation practices and real-world applications are incongruent. This remains the case even in children, where the limitation of dose is even more important. We seek to quantify how often parameters are adjusted in real-world practice and their degree of contribution to real-world dose distribution. We identify discrepancies between parameters that are impactful in theory and impactful in practice.
**Methods or Backround:** This study analyses 25,000 consecutive children routine abdomen exams performed between November 2015 and Jan 2021 in the UCSF International CT Dose Registry of 155 institutions. We calculated geometric standard deviation (gSD) for five parameters (kV, mAs, spiral pitch, number of phases, scan length) to assess variation in practice. A Gaussian mixed regression model was performed to predict the radiation dose-length product (DLP) using the parameters. To reflect the theoretical impact, we predict the increase in DLP per 10% increase in the parameter. To reflect the real-world practical impact, we predict the increase in DLP per gSD increase in the parameter.
**Results or Findings:** Among studied examinations, mAs, number of phases, and scan length were frequently manipulated (gSD 1.47–1.79); kV was rarely manipulated (gSD 1.12). Theoretically, kV is the most impactful parameter (31% increase in DLP per 10% increase in kV, 8–11% for other parameters). In real-world practice, kV is less impactful; for each gSD increase in kV, the DLP increases by 40%. This value is highly surpassed by the analogous number in mAs (68%), while being comparable to other technical parameters.
**Conclusion:** Despite the potential impact of kV on radiation dose, this parameter is rarely manipulated in common practice and its potential remains untapped.
**Limitations:** This study uses mainly US Data.
**Ethics committee approval:** The institutional review board of the primary responsible university approved this research, and collaborating institutions obtained local approval or relied on the primary site's approval.
**Funding for this study:** The registry was funded by NIH and PCORI.
**Author Disclosures:**
Philip Chu: Nothing to disclose
Rebecca Smith-Bindman: Nothing to disclose
Carly Stewart: Nothing to disclose
Yifei Wang: Nothing to disclose

### RPS 1713-3
**Establishing indication-based diagnostic reference levels for paediatric computed tomography and international comparison: data from an international registry**
*D. Bos*[1], Y. Wang[2], C. Stewart[2], S. Zensen[1], J. Luong[2], P. Chu[2], R. Smith-Bindman[2]; [1]Essen/DE, [2]San Francisco, CA/US

**Purpose:** Computed tomography (CT) scans are essential for diagnosing paediatric patients, especially in emergencies. However, concerns arise about the potential carcinogenic risk associated with radiation exposure. CT radiation doses can vary widely depending on medical indications, protocols, and local practice. Our aim is to establish diagnostic reference levels (DRLs) based on clinical indications in paediatric patients.
**Methods or Backround:** A comprehensive analysis was performed using CT data from an international dose registry that includes CT scans performed in children under 18 years at more than 150 sites between January 2016 and January 2021. DRLs, defined as the 75th percentile, were calculated for volume-weighted CT dose index (CTDIvol) and dose-length product (DLP) across 15 broad CT categories, which reflect both the anatomic areas and radiation doses (low-dose [LD], routine-dose [RD], high-dose [HD]) required by the underlying imaging indications. We compared RD categories for head, chest, and abdomen/pelvis scans between facilities in the United States (US) and Europe.
**Results or Findings:** A total of 95,047 CT scans (54% male, 45% female) from 41 different indications were included in the analysis. The DRLs of DLP and CTDIvol mainly increased significantly with increasing age group ($p<0.05$). For head scans in 10- to 14-year-old children, the DRLs for DLP varied from 362 mGy·cm (LD) to 734 mGy·cm (RD) to 2,058 mGy·cm (HD). US DRLs were significantly higher than European DRLs for RD chest and abdomen/pelvis. Differences were inconsistent for RD head.
**Conclusion:** Optimising radiation dose for paediatric patients presents significant challenges due to variations based on age, size, clinical indication and protocol. Therefore, we have established indication-based DRLs for different CT categories and age groups to minimise excessive radiation doses and standardise practice.
**Limitations:** An identified limitation is that the registry contains mainly US data.
**Ethics committee approval:** The institutional review board of the primary responsible university approved this research, and collaborating institutions obtained local approval or relied on the primary site's approval.
**Funding for this study:** The registry was funded by NIH and PCORI.
**Author Disclosures:**
Philip Chu: Nothing to disclose
Denise Bos: Nothing to disclose
Rebecca Smith-Bindman: Nothing to disclose
Carly Stewart: Nothing to disclose
Sebastian Zensen: Nothing to disclose
Yifei Wang: Nothing to disclose
Jason Luong: Nothing to disclose

### RPS 1713-4
**Automated dose and noise assessment as a guide for harmonisation of abdominal CT protocols across multiple scanners**
*J. Vignero*[1], N. Fitousi[1], B. Miseur[1], J. Binst[1], K. Lemmens[1], V. Nuttens[2], H. Bosmans[1]; [1]Leuven/BE, [2]Aalst/BE
*(janne.vignero@uzleuven.be)*

**Purpose:** In this study a systematic approach is proposed for performance evaluation and, if necessary, harmonisation of abdominal computed tomography (CT) protocols across vendors using dose and noise metrics.
**Methods or Backround:** CTDIvol, water equivalent diameter (WED), global noise level (GNL) and metadata of 4,933 abdominal CT exams from six scanners were calculated by DOSE (Qaelum). Paediatric patients (<15 years), hard kernel reconstructions and scans with substantial image truncation were excluded. Patients were categorised into five size groups based on WED. Subgroups were defined based on protocol, scanner, kVp and kernel. Local working points (LWP) for CTDIvol and GNL were calculated from the median values for every subgroup. Reference working points (RWPs) were determined for each size group based on the median CTDIvol and GNL across all subgroups. The performance of each subgroup was assessed by comparing LWP to RWP.
**Results or Findings:** Patient size groups ranged between 200 mm and 422 mm, with corresponding increases in dose and noise RWP from 5 to 16 mGy and 10.9 to 12.5 HU, respectively. Variations in LWP among subgroups were large. The GNL with iterative reconstruction was significantly ($p=0.0$) lower than using filtered back projection. Some subgroups appeared to be overdosing or requiring higher image quality, while others might benefit from higher radiation doses. One scanner with substantially higher dose and noise LWP than RWP was identified as underperforming. Suggested actions were confirmed in a team comprising of radiologists, a radiographer and a physicist.
**Conclusion:** Combined evaluation of automated image noise evaluation, radiation dose and patient size points to clinically relevant adjustments of the scan protocols and was successfully applied in a multi-vendor CT scanner scenario.
**Limitations:** No limitations were identified.
**Ethics committee approval:** No information provided by the submitter.
**Funding for this study:** No funding was provided for this study.
**Author Disclosures:**
Victor Nuttens: Nothing to disclose
Hilde Bosmans: Shareholder: DOSE, QAELUM, Belgium
Kim Lemmens: Nothing to disclose
Janne Vignero: Nothing to disclose
Niki Fitousi: Employee: DOSE, QAELUM, Belgium
Joke Binst: Nothing to disclose
Bram Miseur: Nothing to disclose

### RPS 1713-5

**Clinical CT protocol comparison: an Italian multicentre study**

*R. Villa*[1], M. Daniotti[1], C. Ingraito[1], AIFM Italian Digital Radiology Working Group[2], N. Paruccini[1]; [1]Monza/IT, [2]Milan/IT
*(raffaele.vill@gmail.com)*

**Purpose:** In CT imaging, differences in manufacturers, models, scanning parameters, reconstruction algorithms, clinical tasks, and user's experience, introduce variability in the trade-off between diagnostic information and patient's radiation exposure. The extent to which each of these sources of variabilities impacts the overall procedure performance is still unknown. This study aimed to compare image quality and radiation dose in five acquisition protocols to ascertain how different technical features can affect CT performance, to inform protocol optimisation and design.
**Methods or Backround:** Five different acquisition protocols to investigate five different clinical tasks were investigated: intracranial haemorrhage, pulmonary embolism, acute abdomen, paediatric chest and paediatric acute abdomen. The Catphan 600 (The Phantom Laboratory, USA), was imaged in 34 CT scanners from 20 hospitals across Italy, for a total of 251 datasets, using the locally optimised clinical protocols. The Catphan body external-annulus was used to assess the effects of tube current modulation for the different manufacturers. Image quality was quantified in terms of the detectability index using an object contrast ranging 25-700 HU and lesion size ranging 5-25 mm, representative of clinical scenarios related to the examined protocols.
**Results or Findings:** Each investigated protocol showed differences between manufacturers in terms of dose levels and image quality values. The reconstruction algorithm's generation proved another driving element for image quality. Nevertheless, variability between CTs was found, even from the same vendor and technology.
**Conclusion:** This study suggested that manufacturer and reconstruction algorithms play a main role in the optimal trade-off between diagnostic information and radiation dose levels. Furthermore, the other examined CT procedure technical features, have a non-negligible impact. All the potential sources of variability should be included when designing optimization actions in clinical CT.
**Limitations:** Data numerosity should be increased to improve the study strength, especially for paediatric exams.
**Ethics committee approval:** No information provided by the submitter.
**Funding for this study:** No funding was received for this study.
**Author Disclosures:**
Raffaele Villa: Nothing to disclose
Nicoletta Paruccini: Nothing to disclose
Chiara Ingraito: Nothing to disclose
Martina Daniotti: Nothing to disclose
AIFM Italian Digital Radiology Working Group: Nothing to disclose

### RPS 1713-6

**Implementation of the German lung cancer screening protocol and image quality evaluation over three generations of CT systems**

*M. Ursani*[1], T. D. Do[1], H-U. Kauczor[1], H-P. Schlemmer[1], T. Lasser[2], S. O. Schönberg[3], S. Sawall[1], M. Kachelrieß[1]; [1]Heidelberg/DE, [2]Garching/DE, [3]Mannheim/DE

**Purpose:** To develop methods for the implementation of lung cancer screening using low-dose CT according to the German "Bundesamt für Strahlenschutz" (BfS) and to evaluate the resulting image quality in three generations of CT systems.
**Methods or Backround:** Experiments were performed using a thorax phantom (QRM, Germany) in three generations of CT systems (Somatom Flash/Force, Naeotom Alpha, Siemens Healthineers, Germany). The phantom and imaging protocols were tailored to meet BfS requirements for lung cancer screening. The phantom featured 1 cm sized spherical lung nodules with a contrast of 150 HU and fat extension rings mimicking different patient sizes, namely S (20×30 cm), M (25×35 cm), and L (30×40 cm). Acquisitions were performed at 1.3 mGy as defined as the maximum dose for a standard patient (BMI 26 kg/m²), employing patient-specific prefilters, if available. Images were reconstructed iteratively using the smoothest kernel ensuring a spatial resolution with a FWHM≤1 mm in both axial and longitudinal direction. Hence, resolution is only approximately matched among systems. Contrast-to-noise ratio (CNR) was used to assess the visibility of lung nodules in all images.
**Results or Findings:** A dose of 1.3 mGy was achieved using tube settings of 20 mAs/120 kV, 306 mAs/100 kV+Sn, and 150 mAs/100 kV+Sn for Flash, Force and Alpha. Image noise varied between 115 HU to 330 HU over all systems and phantom sizes. CNR (S to L) ranged from 1.30 to 0.36, 1.03 to 0.65, and 1.76 to 0.71 for Flash, Force, and Alpha.
**Conclusion:** A wide range of CT systems are capable of providing an image quality sufficient for lung cancer screening examinations. However, larger patients might require an increase in dose or slice thickness to account for the high image noise.
**Limitations:** The study is limited to phantom acquisitions.
**Ethics committee approval:** No information was provided by the submitter.
**Funding for this study:** No funding was received for this study.
**Author Disclosures:**
Stefan Oswald Schönberg: Nothing to disclose
Thuy Duong Do: Nothing to disclose
Hans-Ulrich Kauczor: Nothing to disclose
Mishal Ursani: Nothing to disclose
Heinz-Peter Schlemmer: Nothing to disclose
Marc Kachelrieß: Nothing to disclose
Stefan Sawall: Nothing to disclose
Tobias Lasser: Nothing to disclose

### RPS 1713-7

**Physics evaluation of a super resolution deep learning reconstruction algorithm in an anatomical background**

*K. Boedeker*, D. Shin, N. Akino, D. Mather; Otawara-shi, Tochigi/JP
*(kboedeker@mru.medical.canon)*

**Purpose:** Deep learning reconstruction algorithms for computed tomography (CT) are generally trained on anatomical data. Therefore, the purpose of this work is to characterise a super resolution deep learning reconstruction algorithm (DLR) compared to traditional hybrid iterative reconstruction (HIR) and filtered back projection (FBP) in terms of standard quantitative noise and spatial resolution measures, with the test objects inserted in both an anatomical background as well as in uniform phantom background.
**Methods or Backround:** A commercial super resolution DLR was assessed for cardiac mode. The modulation transfer function (MTF) was first measured using a standard edge-based approach on images acquired from the Catphan 600 with uniform background and reconstructed with DLR, HIR, and FBP. The cylindrical test objects used for assessment were next simulated via forward projection using a system model and inserted into raw patient data. The raw patient data was then reconstructed with DLR, HIR, and FBP. The MTF and standard deviation of noise were then measured. A 100 mm FOV was used for measurement. The Pearson correlation coefficient was determined for each reconstruction algorithm's MTF in patient versus phantom.
**Results or Findings:** The FBP MTF values in the anatomical background versus uniform phantom were within 10%. In both phantom and the anatomical background, noise reduction for DLR versus HIR was approximately 45-50%. The 10% of MTF was 11.6p/cm with DLR versus 8.4lp/cm with HIR in anatomical background. The Pearson correlation in anatomical background versus phantom was greater than 0.99 for all three reconstruction algorithms.
**Conclusion:** Traditional in-plane MTF applied in an anatomical background demonstrates significant improvement over HIR and correlates well with phantom measurements.
**Limitations:** An identified limitation is that a limited dose range was analysed.
**Ethics committee approval:** No information provided by the submitter.
**Funding for this study:** Funding was received from Canon Medical System Corporation.
**Author Disclosures:**
Dylan Mather: Employee: Canon Medical Systems Corporation
Kirsten Boedeker: Employee: Canon Medical Systems Corporation
Daniel Shin: Employee: Canon Medical Systems
Naruomi Akino: Employee: Canon Medical Systems Corporation

### RPS 1713-8

**Influence of foreign bodies on image quality in dark-field chest radiography**

*L. Kaster*[1], S. Karl[1], T. Urban[1], F. Gassert[1], R. C. Schick[1], T. Koehler[2], A. Sauter[1], F. Pfeiffer[1], D. Pfeiffer[1]; [1]Munich/DE, [2]Hamburg/DE
*(lennard.kaster@tum.de)*

**Purpose:** Dark-field radiography is a novel x-ray method that visualises microstructural properties of the examined object. Our pioneering studies, the first conducted on humans worldwide, have demonstrated that this method outperforms conventional radiographs in diagnosing and staging pulmonary diseases, like COPD and Covid-19. It is known from conventional x-ray radiographs and CT that foreign bodies, such as metal implants, can lead to artefacts and thus impair image quality. Therefore, this research evaluates the influence of foreign bodies on x-ray dark-field chest radiographs.
**Methods or Backround:** Experiments were conducted using a clinical dark-field chest radiography prototype, including investigating patient images. The "LUNGMAN" thorax phantom was used and measurements were performed with and without foreign bodies like breast implants, medical compresses and a pacemaker unit. The impact of these foreign bodies on image quality was analysed.
**Results or Findings:** The results demonstrated that the impact of a foreign body on image quality is contingent upon its material composition. Homogeneous materials, such as breast implants, do not contribute additional dark-field signals. Conversely, materials possessing a microstructure, exemplified by medical compresses, generate their own dark-field signals.

Notably, these signals do not influence the surrounding areas. In the case of a pacemaker unit, despite the absence of a microstructure, a dark-field signal can be observed. Furthermore, areas adjacent to the foreign body are also affected. For both medical compresses and the pacemaker unit, the dark-field signal emanating from tissue within the same radiation path cannot be reliably evaluated.
**Conclusion:** Foreign bodies have the potential to generate a substantial true or artificial dark-field signal, which can significantly impact the image quality and interpretability of dark-field radiographs. Therefore, it is crucial to consider these potential artefacts to ensure an accurate diagnosis
**Limitations:** No limitations were identified.
**Ethics committee approval:** The study was approved by the Ethics Commission of the Medical Faculty, Technical University of Munich, Germany (reference number: 166/20S).
**Funding for this study:** Funding was received from the Federal Ministry of Education and Research (BMBF) and the Free State of Bavaria under the Excellence Strategy of the Federal Government and the Länder, as well as by the Technical University of Munich – Institute for Advanced Study.
**Author Disclosures:**
Andreas Sauter: Nothing to disclose
Thomas Koehler: Employee: Philips
Florian Gassert: Nothing to disclose
Sandra Karl: Nothing to disclose
Theresa Urban: Nothing to disclose
Lennard Kaster: Nothing to disclose
Rafael Christian Schick: Nothing to disclose
Franz Pfeiffer: Nothing to disclose
Daniela Pfeiffer: Nothing to disclose

**08:00-09:00 Research Stage 4**

### Research Presentation Session: Chest

### RPS 1704

### Novel technological developments in chest imaging: from research to practice

**Moderator**
A. Christe; Berne/CH
*(andreas.christe@insel.ch)*

### RPS 1704-2

**Assessment of lung perfusion using dynamic digital radiography and comparison with nuclear medicine lung scintigraphy**
*S. Singh*, R. Sai, P. Manapragada, D. Benson; Birmingham, AL/US
*(ssingh@uabmc.edu)*

**Purpose:** Assessment of lung perfusion is required in the preoperative evaluation of patients being considered for lung transplant or resection. Dynamic digital radiography (DDR) is a newer radiographic technique that acquires rapid sequential diagnostic radiographs of the chest throughout multiple respiratory cycles. The regional lung perfusion can be estimated using DDR by assessing pixel signal changes throughout the cardiac cycle. This study will compare the results of the differential lung perfusion estimated using DDR with the same results obtained from lung scintigraphy, a conventional imaging modality.
**Methods or Backround:** A retrospective review of patients evaluated with both nuclear medicine lung scintigraphy and DDR was performed. The DDR examinations were performed between January 14, 2022, and July 27, 2023. Each patient had a lung scintigraphy perfusion study within six months of the DDR examination. The percent differential of perfusion between the lungs was calculated using both modalities and the results were analyzed for statistical correlation.
**Results or Findings:** Results for 75 patients were reviewed (mean age - 56 years, 32 Females). The mean absolute percent differential in perfusion between the right and left lungs was (14.7 ± 23.1%) using DDR and (15.08 ± 24.76%) using lung scintigraphy. There were five patients in which there was a discrepancy between the two modalities in determining which lung was better perfused. The Pearson coefficient of correlation was (0.915) indicating a strong correlation between the perfusion results obtained by the two modalities.
**Conclusion:** Differential lung perfusion estimated by DDR is strongly correlated with the same result obtained using lung scintigraphy. The speed and cost-effectiveness of DDR make it an attractive option for clinicians, potentially reducing wait times and healthcare costs for patients.
**Limitations:** Small numbers. Need more refined computer analysis.
**Ethics committee approval:** The study is IRB approved.
**Funding for this study:** No funding was received for this study.
**Author Disclosures:**
Rasagna Sai: Nothing to disclose
Satinder Singh: Nothing to disclose
Donald Benson: Nothing to disclose
Padma Manapragada: Nothing to disclose

### RPS 1704-3

**Silver x-ray spectrum modulation filter for lung cancer screening: comparison of capability for radiation dose reduction and detection performance with copper filter**
*Y. Ohno*[1], H. KImata[2], Y. Ito[2], K. Fujii[2], N. Akino[2], H. Nagata[1], M. Nomura[1], T. Ueda[1], Y. Ozawa[1]; [1]Toyoake/JP, [2]Otawara/JP
*(yohno@fujita-hu.ac.jp)*

**Purpose:** The purpose of this study was to compare the capability of low-dose lung cancer CT screening between silver (Ag) and copper (Cu) x-ray spectral modulation filters at in vitro and in vivo studies.
**Methods or Backround:** A chest CT phantom including simulated ground-glass and part-solid nodules was scanned with a 320-detector row CT with Ag and Cu filters at 0.6, 1.6 and 2.5 mGy at in vitro study. Then, 95 patients underwent low-dose CT lung cancer screening with Ag and Cu filters at the same radiation dose level (i.e. 1.6 mGy) as in the in vivo study. At in vitro study, SNR at each nodule were determined by region of interest (ROI) measurements at all protocols. At in vivo study, SNR of normal lung parenchyma was also determined by ROI measurement at each CT. Then, the probability of a nodule was assessed with 5-point visual score by two board-certified chest radiologists at both studies. Student's t-test was performed to compare SNR between Ag and Cu filters at each study. ROC and JAFROC analyses were performed to compare nodule detection capability between Ag and Cu filters.
**Results or Findings:** At in vitro study, SNR and AUC of Ag filter were significantly better than those of Cu filter at each dose level ($p < 0.05$). At in vivo study, SNR of Ag filter was significantly higher than that of Cu filter ($p < 0.05$), although no significant differences of averaged figure of merit were determined.
**Conclusion:** Low-dose CT screening with Ag filter can significantly improve image quality compared to screening using a Cu filter.
**Limitations:** This was a single institution study.
**Ethics committee approval:** IRB approval was received (Fujita Health University Hospital).
**Funding for this study:** This study received funding via a research grant from Canon Medical Systems Corporation.
**Author Disclosures:**
Yoshiyuki Ozawa: Nothing to disclose
Masahiko Nomura: Nothing to disclose
Kenji Fujii: Employee: Canon Medical Systems Corporation
Hirona KImata: Employee: Canon Medical Systems Corporation
Yuya Ito: Employee: Canon Medical Systems Corporation
Takahiro Ueda: Nothing to disclose
Hiroyuki Nagata: Grant Recipient: Canon Medical Systems Corporation
Yoshiharu Ohno: Grant Recipient: Canon Medical Systems Corporation
Naruomi Akino: Employee: Canon Medical Systems Corporation

### RPS 1704-4

**Ultra-low-dose photon-counting detector CT for imaging follow-up after lung transplantation**
L. Lechner, *R-I. Milos*, A. Korajac, D. Kifjak, D. Tamandl, M. Stuempflen, M. Watzenböck, A. E. Strassl, H. Prosch; Vienna/AT

**Purpose:** CT surveillance is performed in patients after lung transplantation for the detection of subclinical abnormalities. We assessed the potential of ultra-low-dose (ULD) photon-counting detector computed tomography (PCD-CT) when compared to routinely performed low-dose (LD) PCD-CT for the follow-up in lung transplanted patients.
**Methods or Backround:** For this prospective study adult patients underwent same-day ULD and LD PCD-CT (with inspiration and expiration scans for each radiation dose protocol) between March and May 2023. The 1 mm reconstructions ULD and LD scans were reviewed by three readers who independently assessed the subjective image quality of anatomical structures (using visual grading analysis, VGA, scores) and the presence of lung abnormalities (evaluated as present or absent). Interrater agreement was calculated using Fleiss' kappa.
**Results or Findings:** 83 patients (median age 62 years, 36 women) were included. The radiation dose of the ULD protocol comprised on average 15% of the one for LD protocol. For anatomical structures all readers rated ULD scans adequate to excellent in the majority of patients (92.8 - 97.6% for bronchial wall, 91.6 - 94% for fissures, and 80.7 - 100% for peripheral vessels). Interrater agreement was better on the LD protocol for the detection of ground glass

Saturday opacities, consolidations, and atherosclerosis (0.61 vs 0.50; 0.68 vs 0.6; 0.94 vs 0.72), and better for the ULD protocol for the detection of air trapping, tree-in-bud, bronchial wall thickening and nodules greater than 5 mm (0.66 vs 0.57; 0.69 vs 0.56; 0.44 vs 0.31; 0.45 vs 0.19). Both LD and ULD protocols showed comparable agreement for the detection of reticulations, linear atelectasis and distal bronchiectasis (between 0.18 and 0.37).
**Conclusion:** ULD PCD-CT offers sufficient image quality and is a feasible option for post-lung transplantation patient surveillance.
**Limitations:** No limitations were identified.
**Ethics committee approval:** The ethics committee notification can be found under the number EK Nr: 1891/2022.
**Funding for this study:** No funding was provided for this study.
**Author Disclosures:**
Lisa Lechner: Nothing to disclose
Helmut Prosch: Nothing to disclose
Marlene Stuempflen: Nothing to disclose
Andreas Eduard Strassl: Nothing to disclose
Aida Korajac: Nothing to disclose
Daria Kifjak: Nothing to disclose
Dietmar Tamandl: Nothing to disclose
Ruxandra-Iulia Milos: Nothing to disclose
Martin Watzenböck: Nothing to disclose

### RPS 1704-5

**Low-dose high-resolution photon-counting CT in patients with cycstic fibrosis: radiation dose and image quality in the clinical routine**
*M. Frings*, M. Welsner, S. Zensen, J. Haubold, D. Bos, L. Umutlu, B. M. Schaarschmidt, M. Forsting, M. Opitz; Essen/DE

**Purpose:** Patients with cystic fibrosis routinely undergo low-dose high-resolution (LD-HR) CT scans as part of their clinical management, primarily for monitoring chronic exacerbations or assessing treatment response with the triple-combination CFTR modulator therapy elexacaftor/tezacaftor/ivacaftor. The aim of this study is to compare the radiation dose and image quality of LD-HR protocols between photon-counting CT (PCCT) and CT scanner with an energy-integrating detector system (EID-CT) in this patient cohort.
**Methods or Backround:** Thirty-five patients who underwent a LD-HR chest CT scan with PCCT and had previously undergone a LD-HR chest CT with a standard EID-CT scanner were retrospectively included in this study. The dose-length product (DLP), volumetric CT-dose index (CTDIvol) and singal-to-noise ratio (SNR) were determined and explorative data analysis was performed. The overall image quality, image sharpness, and image noise of both full lung scans and enlarged sections highlighting specific findings were assessed by three radiologists using a 5-point Likert scale.
**Results or Findings:** The PCCT used significantly less radiation dose compared to the EID-CT (P <0.001), approximately 36%. PCCT consistently received significantly better ratings for overall image quality and image sharpness compared to EID CT (P <0.003). Additionally, PCCT exhibited significantly lower image noise (P <0.004) and a lower average signal-to-noise ratio (SNR) compared to EID CT (P <0.005).
**Conclusion:** Lung PCCT scans in patients with CF offer superior image quality while utilising substantially lower radiation doses in comparison to EID-CT scans.
**Limitations:** The limitations of this study are its retrospective and single-centre study design.
**Ethics committee approval:** This study was approved by an ethics committee.
**Funding for this study:** No funding was received for this study.
**Author Disclosures:**
Michael Forsting: Nothing to disclose
Denise Bos: Nothing to disclose
Benedikt Michael Schaarschmidt: Nothing to disclose
Johannes Haubold: Nothing to disclose
Sebastian Zensen: Nothing to disclose
Lale Umutlu: Nothing to disclose
Marko Frings: Nothing to disclose
Marcel Opitz: Nothing to disclose
Matthias Welsner: Nothing to disclose

### RPS 1704-6

**Optimal slice thickness and quantum iterative reconstruction level in low-dose ultra-high-resolution photon-counting detector CT of the lungs**
*D. Graafen*[1], M. C. Halfmann[1], T. S. Emrich[1], Y. Yang[1], C. Düber[1], R. Klöckner[2], L. Müller[1], T. Jorg[1]; [1]Mainz/DE, [2]Lübeck/DE

**Purpose:** Using photon-counting detector computed tomography (PCD-CT) alongside an innovative iterative reconstruction technique called quantum iterative reconstruction (QIR) holds the promise of significantly improving the quality of lung images. This research aimed to investigate how various QIR levels and different slice thicknesses affect the image quality of low-dose ultra-high-resolution (UHR) PCD-CT in lung examinations.
**Methods or Backround:** The study involved 51 patients who underwent unenhanced UHR-PCD-CT scans. The images were reconstructed using a 1024 matrix. Three slice thicknesses (1.0 mm, 0.4 mm, and 0.2 mm) were employed, along with three different QIR levels (2 to 4). Noise levels were calculated for all reconstructions. To evaluate the image quality in comparison to the clinical reference reconstruction (1.0 mm QIR-3), three radiologists rated the sharpness of different lung structures and the conspicuity of various lung abnormalities in the images using a 5-point Likert scale.
**Results or Findings:** The highest QIR level (QIR-4) reduced image noise effectively and received the best image quality ratings. Decreasing the slice thickness to 0.4 mm enhanced the sharpness of pulmonary structures and the conspicuity of different pathologies. However, when the thickness was reduced to 0.2 mm, the images were significantly affected by noise, resulting in a notable decline in quality scores.
**Conclusion:** The ideal reconstruction approach for low-dose UHR-PCD-CT lung imaging is to use a slice thickness of 0.4 mm together with the highest QIR level. The clinical implementation of this optimised UHR-PCD-CT protocol could potentially enhance the accuracy of diagnoses and boost confidence in lung imaging.
**Limitations:** Limitations of the study are the single-centre design and the small sample size of some lung pathologies.
**Ethics committee approval:** The study was approved by the Institutional Ethics Committee of Rhineland-Palatinate (Reg. Nr. 2022-16359).
**Funding for this study:** This research received no external funding.
**Author Disclosures:**
Roman Klöckner: Speaker: Siemens Healthineers
Dirk Graafen: Speaker: Siemens Healthineers
Christoph Düber: Nothing to disclose
Tilman Stephan Emrich: Advisory Board: Siemens Healthineers; Speaker: Siemens Healthineers
Moritz Christian Halfmann: Nothing to disclose
Tobias Jorg: Nothing to disclose
Lukas Müller: Nothing to disclose
Yang Yang: Nothing to disclose

### RPS 1704-7

**The feasibility and accuracy of using low- and ultra-low-dose photon counting detector CT to detect metastatic lung nodules in paediatric and young adults with malignant bone tumours**
*S. Zhou*, L. Qin, Z. Xu, F. Yan; Shanghai/CN
*(zhouss1202@hotmail.com)*

**Purpose:** We aimed to investigate the feasibility of low- and ultra-low-dose photon counting detector CT (PCD-CT) for chest CT follow-ups in paediatric and young adults with pulmonary metastasis of bone malignant tumours, and compare the accuracy of PCD-CT with intra-patient previous energy-integrating detector CT (EID-CT).
**Methods or Backround:** 83 subjects (male/female, 52/31) aged 19 [15; 21] years with prior chest EID-CT scans participated in low-dose (IQ level=16, n=43) and ultra-low-dose (IQ level=10, n=40) chest PCD-CT scanning with a tube voltage of Sn100. The contrast-noise-ratio (CNR), figure of merit for CNR (FOMCNR, normalising the CNR to effective dose), and edge sharpness of maximum nodule were calculated. Moreover, a 5-point Likert scale was used to subjectively evaluate the image quality.
**Results or Findings:** The low-dose and ultra-low-dose groups with effective doses of 0.40 [0.35; 0.44] and 0.22 [0.16; 0.27] mSv, which were 7.67 and 14.42 times lower than EID-CT, achieved a mean detection rate of 93.56% and 84.48% for preexisting lung nodules, respectively. PCD-CT with higher FOMCNR and sharpness but lower CNR than EID-CT (all P <0.05). In the low-dose group, in detail, the CNR, FOMCNR, and sharpness were 18.7 [14.8; 24.0] vs 19.7 [14.1; 26.2], 915 [632; 1578] vs 128 [63.2; 268], and 543 [446; 704] vs 422 [344; 545] Hounsfield Unit (HU)/pixel for PCD-CT and EID-CT, respectively. In the ultra-low-dose group, these values were 17.1 [15.4; 20.7] vs 22.4 [16.6; 26.6], 1490 [955; 2441] vs 150 [96; 207], and 594 [464; 752] vs 445 [362; 617] HU/pixel, respectively. PCD-CT showed statistically significant superiority in the image quality, motion artifacts, and display of lung nodules and skeleton, but inferior in the presentation of mediastinal lymph nodes and lung markings compared to EID-CT (P <0.05).
**Conclusion:** PCD-CT allowed reliable detection of metastatic lung nodules with significant radiation dose reduction.
**Limitations:** This technique has relatively poor visualisation of soft tissues, but it does not compromise the detection of crucial structures like lymph nodes.
**Ethics committee approval:** The study received institutional review board approval and written informed consent was waived.
**Funding for this study:** No funding was received for this study.
**Author Disclosures:**
Le Qin: Nothing to disclose
Zhihan Xu: Nothing to disclose
Fuhua Yan: Nothing to disclose
Shanshui Zhou: Nothing to disclose

**08:00-09:00** **Room G1**

### Research Presentation Session: Radiographers

### RPS 1714

### Enhancing patient experience and safety in medical imaging and radiotherapy

**Moderators**
A. Sarchosoglou; Athens/GR
*(anastasia.sarch@outlook.com)*
A. Blanco Barrio; Murcia/ES
*(anablancowhite@gmail.com)*

### RPS 1714-3

**Radiographers' current experiences interacting with patients with dementia and cognitive impairment during magnetic resonance imaging**

S. Hanley, P. C. Murphy, R. Young, N. Moore, A. England, *M. F. McEntee*; Cork/IE
*(mark.mcentee@ucc.ie)*

**Purpose:** Dementia is the umbrella term used to describe several conditions, causing damage to the brain. This study aims to investigate radiographers' current experiences interacting with patients with dementia and cognitive impairment during MRI.
**Methods or Backround:** This is a prospective quantitative study consisting of a questionnaire completed by MRI radiographers. The study location was in a single public hospital in the Republic of Ireland, performed over a six week period. The inclusion criteria required "dementia", "cognitive impairment", or "confusion" to be documented in the request form, detailed by the ward staff, or if any one of these were discovered by the MRI radiographers when the patient arrived.
**Results or Findings:** There were twenty questionnaires completed. The point when it was discovered the patient had dementia, cognitive impairment or confusion was predominantly through contact from the ward, accounting for 50%. Not one out of the twenty patients required someone being present during the scan. 30% of patients were agitated or distressed during the MRI examination. 15% of examinations needed to be aborted and rebooked and 25% were incomplete.
**Conclusion:** There were issues in discovering the patient had dementia, cognitive impairment, or confusion. There were implications regarding examinations needing to be aborted and rebooked. This is a concern as MRI is a very sought after modality. This is an area which needs to be addressed as these examination slots are vital for assisting patients along their clinical pathway.
**Limitations:** Identified limitation were (1) the single-centre nature of the study and (2) the relatively small sample size.
**Ethics committee approval:** This study was approved by the Medical School SREC, University College Cork.
**Funding for this study:** No funding was received for this study.
**Author Disclosures:**
Mark F. McEntee: Nothing to disclose
Niamh Moore: Nothing to disclose
Rena Young: Nothing to disclose
Sarah Hanley: Nothing to disclose
Andrew England: Board Member: EFRS Advisory Board: RoClub
Peter C. Murphy: Nothing to disclose

### RPS 1714-4

**Radiographers' experience during medical imaging of patients with dementia in Norway**

C. C. Chilanga[1], *C. Eikefet*[2], E. Kjelle[2]; [1]Svelvik/NO, [2]Drammen/NO
*(christine.eikefet@usn.no)*

**Purpose:** This study aimed to explore radiographers' experiences during imaging examinations of people living with dementia in Norway.
**Methods or Backround:** Semi-structured qualitative interviews were conducted with eight radiographers, four working in MRI or general radiography and four working in nuclear medicine, at three different hospital trusts in Norway. The interview guide included the following topics: radiographers' experience of working with patients living with dementia, challenges faced when conducting examinations for these patients, knowledge about dementia and initiatives in the department. All interviews were verbatim transcribed and inductive content analysis as described by Elo and Kyngäs was used to analyse the data.
**Results or Findings:** The analysis resulted in three main categories, each with two to five subcategories. The main categories were "radiographers experience", "measures taken to accommodate for the patient" and "competencies". The radiographers frequently encountered patients with dementia in the department. The challenges they faced included a lack of information before receiving patients with dementia, communicating with the patients, and ensuring their stability during the procedure. MRI safety was of particular concern when communication and information sharing were problematic. None of the departments had any overarching procedures or training related to patients with dementia. Creating a calm environment, collaborating with carers, scheduling adequate time for examinations, and possessing good communication skills were viewed as facilitators for conducting examinations successfully.
**Conclusion:** Radiographers experienced managing and imaging patients living with dementia to generally be uncomplicated. The knowledge on examining patients with dementia was mostly acquired through clinical practice. However, in some cases the department's environment, and communication problems caused stress and restlessness in patients.
**Limitations:** The limitation of this study is that findings from interviews with a few radiographers in Norway may not be easily generalised to other settings.
**Ethics committee approval:** The Norwegian Agency for Shared Services in Education and Research approved the treatment of personal information (project reference: 155338)..
**Funding for this study:** No funding was received for this study.
**Author Disclosures:**
Elin Kjelle: Nothing to disclose
Christine Eikefet: Nothing to disclose
Catherine Chilute Chilanga: Nothing to disclose

### RPS 1714-5

**An investigation into the perceived challenges and facilitators Parkinson's disease patients undergo when accessing radiology examinations**

B. Treanor, *M. F. McEntee*, N. Moore, R. Young, R. O'Regan, A. England; Cork/IE
*(mark.mcentee@ucc.ie)*

**Purpose:** Parkinson's disease (PD) is a progressive neurological disorder characterised by a wide range of symptoms. Understanding the facilitators and barriers for patients with PD accessing radiology is paramount, as this patient group would require frequent access to imaging and the nature of their condition can pose additional challenges. The aim of this study is to investigate the perceived barriers or facilitators to radiology examinations that improve or degrade the experiences for patients with PD by conducting an experience-based focus group.
**Methods or Backround:** This was a qualitative study in which the experiences of patients with PD were studied by conducting an audio-recorded experience-based focus group. Recruitment and data collection took place from March 2023 until April 2023. The primary researcher then personally validated the transcription. Following this, the primary reviewer and secondary reviewer each performed a thematic analysis of the transcription with no discrepancies found between both findings.
**Results or Findings:** There were eight participants in this study. There were three key themes identified for the facilitators which included "supportive hospital staff", "communication" and "knowledge". There were also three main themes identified for the barriers which included "anxiety", "knowledge", and "communication".
**Conclusion:** By addressing these facilitators and barriers, we can work towards creating a more supportive and patient-centred radiology environment for individuals living with PD. Further research is warranted to explore the experiences of patients with PD in radiology departments across different healthcare settings and geographical regions.
**Limitations:** Responses were received from a single service user group in Ireland. Involving participants from more groups or undertaking an international study would be advantageous.
**Ethics committee approval:** This study was approved by Medical School SREC, University College Cork.
**Funding for this study:** No funding was received for this study.
**Author Disclosures:**
Mark F. McEntee: Nothing to disclose
Niamh Moore: Nothing to disclose
Ben Treanor: Nothing to disclose
Rena Young: Nothing to disclose
Andrew England: Board Member: EFRS; Advisory Board: RoClub
Roisin O'Regan: Nothing to disclose

### RPS 1714-6

**The effect of inhalation aromatherapy on patient anxiety in nuclear medicine**

J. Sciberras, *K. Borg Grima*; Msida/MT
*(karen.borg-grima@um.edu.mt)*

**Purpose:** The aim of this study was to evaluate if chamomile intervention has an impact on the anxiety levels of patients undergoing a bone or thyroid scan in nuclear medicine.
**Methods or Backround:** A quantitative, experimental, prospective and cross-sectional design was employed for this study. Fifty participants who complied with this study's inclusion and exclusion criteria were enlisted. Using convenient sampling, the participants were equally distributed between the experimental and control group. In both groups, routine protocol was followed when scanning the patients, apart from those in the experimental group, who were additionally introduced to the chamomile smell. Following this, the State-Trait Anxiety Inventory Questionnaire was used to measure the participants' anxiety level before and after the scan, in both groups.
**Results or Findings:** Participants in both the experimental and control groups experienced higher pre-scan state anxiety (37.84, 40.20) scores when compared to post-scan state anxiety scores (26.24, 37.12). Furthermore, the post-scan state anxiety score of the experimental group was significantly lower compared to the control group's post-scan state anxiety score (P<0.001). In addition, when analysing the difference in anxiety levels between genders a statistical significance was noted. The results indicated that females were more vulnerable to anxiety reactions.
**Conclusion:** From this study's findings, there was statistical significance which indicated that chamomile intervention was effective at lowering the patients' anxiety levels in nuclear medicine. Additionally, the results of this study suggested that chamomile intervention should be used in clinical practice to reduce patients' stress levels since it is non-invasive and cheap.
**Limitations:** The small sample size used in this study could have influenced the accuracy of the results. For future research, a larger sample size should be applied and data should be collected over a longer period of time.
**Ethics committee approval:** This study was approved by the University of Malta Research Ethics Committee (UREC), and by the Data Protection officer of a state general hospital where this research was conducted.
**Funding for this study:** No funding was received for this study.
**Author Disclosures:**
Karen Borg Grima: Nothing to disclose
Jacob Sciberras: Nothing to disclose

### RPS 1714-7

**Evaluation of the influence of music in women undergoing radiation therapy**

*M. C. Ramos*[1], A. Rosa[2], F. Serra[1], A. F. Abrantes[2], L. P. V. P. V. Ribeiro[2], S. I. Rodrigues[2]; [1]Olhao/PT, [2]Faro/PT
*(magdacruzrt@gmail.com)*

**Purpose:** Breast cancer is the most commonly diagnosed cancer among women worldwide. Patients often experience anxiety, particularly during radiotherapy, due to the disruptions in their daily routines and the associated side effects. Music therapy has emerged as a promising psychosocial intervention, offering potential benefits such as reduced anxiety, pain relief, and an improved quality of life for cancer patients. The primary aim of this research was to conduct a comprehensive examination of how these factors collectively impact anxiety levels in breast cancer patients undergoing radiotherapy.
**Methods or Backround:** This study assume a comparative analysis between two groups of breast cancer patients. The assessment tools utilised consisted of a 'yes' system, a concise interview, the STAI (State-Trait Anxiety Inventory) questionnaire, and a visual analog comfort scale. To assess anxiety levels, the STAI questionnaire was administered twice: at the initiation of the treatment regimen and upon its completion.
**Results or Findings:** This study assessed anxiety levels, heart rate, education status, and comfort levels, along with examining the influence of music on anxiety during radiotherapy. The findings indicated no noteworthy age-related differences, but they did reveal a moderate correlation between anxiety and heart rate in the experimental group. No significant correlations were identified between anxiety and education status or comfort levels. Regarding the impact of music on anxiety between the groups, lower mean values of final anxiety were observed in the experimental group (64.00 ± 2.94) compared to the control group (68.65 ± 3.69).
**Conclusion:** While this study did not yield statistically significant differences in anxiety reduction through music during radiotherapy, its findings remain valuable and instructive. The feedback transmitted by the patients of the experimental group was shown to be favourable for the use of music, regardless of whether the results were significant or not.
**Limitations:** No limitations were identified.
**Ethics committee approval:** This study received authorisation from the institutional board.
**Funding for this study:** No funding was received for this study.
**Author Disclosures:**
Magda Cruz Ramos: Nothing to disclose
Sónia Isabel Rodrigues: Nothing to disclose
Fábio Serra: Nothing to disclose
Ana Rosa: Nothing to disclose
António Fernando Abrantes: Nothing to disclose
Luís Pedro Vieira Pedro Vieira Ribeiro: Nothing to disclose

### RPS 1714-8

**Using the e-Delphi method to develop informative nutritional leaflets for patients undergoing radiotherapy to the head and neck**

*D. Munro*, G. V. Dijk, J. G. Couto; Msida/MT

**Purpose:** Patients diagnosed with head and neck (HN) cancer undergoing radiotherapy (RT) frequently encounter distressing side effects that significantly impede their ability to consume food. This often results in deteriorating nutritional status and subsequent weight loss. The primary objective of this study was to evaluate the efficacy of a modified e-Delphi approach in creating informative dietary advice pamphlets aimed at addressing these side effects, while garnering consensus among healthcare professionals.
**Methods or Backround:** An e-Delphi methodology was employed, involving six participants representing various healthcare professions specialising in HN patient care (radiographers, nurses, oncologists). These participants were tasked with providing feedback on four dietary leaflets tailored to specific symptoms, all meticulously crafted based on an extensive prior literature review. Following each round of feedback, the participants' recommended modifications were incorporated. Significant alterations to the leaflets were subjected to participant voting before implementation.
**Results or Findings:** After three rounds of deliberation, unanimous consensus was achieved, as all participants expressed a "highly likely" inclination to incorporate the leaflets into their clinical practice. The majority of the participants' suggestions were consistent with existing literature. The only change that deviated from the literature, and was accepted through voting, pertained to sugar consumption.
**Conclusion:** The participants successfully attained consensus and developed leaflets aligned with literature recommendations, which they deemed suitable for clinical application. This variation of the e-Delphi method demonstrated its efficiency in establishing consensus among healthcare professionals concerning patient information resources.
**Limitations:** The participant pool could have been larger. However, all the radiotherapy professions were represented in the sample. Including a dentist could have been beneficial.
**Ethics committee approval:** The researchers submitted the research for records to the University of Malta's University Research Ethics Committee (reference number: FHS-2022-00039) following a self-assessment that showed that the research was low risk and did not require formal ethical approval.
**Funding for this study:** No funding was received for this study.
**Author Disclosures:**
Duncan Munro: Nothing to disclose
Jose Guilherme Couto: Nothing to disclose
Gerbert Van Dijk: Nothing to disclose

### RPS 1714-9

**Radiographers' knowledge, clinical expertise and application of pain management strategies in the radiology department**

R. O'Regan, N. Moore, B. Treanor, R. Young, M. F. McEntee, *A. England*; Cork/IE
*(aengland@ucc.ie)*

**Purpose:** Pain is a widely encountered medical concern that necessitates effective pain management interventions. Pain management challenges are reported within the literature as having a significant impact on medical practice, healthcare professionals, radiology departments, and the healthcare system. Radiographers frequently image patients who are in pain and thus require a heightened awareness and focus on this crucial issue. This study aims to evaluate radiographers' knowledge, clinical expertise, and challenges associated with pain management in the radiology department.
**Methods or Backround:** A qualitative research study consisting of a single focus group was conducted with six participants. The research questions focused on pain management challenges in the radiology department, with a particular emphasis on identifying radiographers' current practices and strategies to address these obstacles. The focus group study was moderated and video-recorded to facilitate comprehensive analysis. The data obtained from the study was transcribed and thematically analysed.
**Results or Findings:** Data analysed from the focus group identified four main themes: consequences of pain management, communication, professional experience, and barriers. In addition, the study identified there are no current protocols, policies, and guidelines in practice for effectively managing the challenges associated with imaging patients in pain within the radiology department.

**Conclusion:** The focus group study revealed an array of concerns related to the challenges associated with imaging patients in pain within the radiology department. The primary consequences identified were stress experienced by both staff and patients, the potential for suboptimal images, patient safety concerns, and negative effects on image quality. The results of this research study demonstrate how patient discomfort negatively affects examinations and identifies the potential consequences for radiographers aspiring to achieve optimal gold standard imaging.
**Limitations:** Identified limitations were: (1) the small sample size and (2) that participants were from a single hospital group in Ireland.
**Ethics committee approval:** This study was approved by Medical School SREC, University College Cork.
**Funding for this study:** No funding was received for this study.
**Author Disclosures:**
Mark F. McEntee: Nothing to disclose
Niamh Moore: Nothing to disclose
Ben Treanor: Nothing to disclose
Rena Young: Nothing to disclose
Andrew England: Board Member: EFRS; Advisory Board: RoClub
Roisin O'Regan: Nothing to disclose

### RPS 1714-10
**Deaf-friendly MRI: radiographers' communication toolkit**
*E. Stefani*, M. Centenaro, G. Cappellina; Treviso/IT
*(eleonora-stefani@libero.it)*

**Purpose:** Equality is vital in healthcare and communication is part of It. Most systems are adequately prepared to communicate with certain type of patients but they don't represent the majority of them. It is necessary to adjust communication to all types of patients. To tailor radiographers' communication to all types of patients is essential to ensure patient safety, compliance and image quality. The study aims to identify tools to improve radiographers' effective communication with deaf patients who undergo MRI examination.
**Methods or Backround:** This was a qualitative study. One focus group has been created including radiographers from three radiology departments in a hub and spoke organisation. A total of 25 radiographers participated in the data collection. Data was analysed using ATLAS.ti software.
**Results or Findings:** 85% of radiographers feel a sense of discomfort when dealing with deaf patients and only 20% of them know about policies supporting deaf patients in the radiological divisions they work in. Communication failures may occur due to: lack of knowledge about how to interact with deaf patients (90% of radiographers); difficulty in calling deaf patients in the waiting room (35%); and scheduling LIS interpreters (60%). Three tools have been identified in support of radiographers: (1) a visual system to call patients; (2) guidelines with essential points for interacting with the deaf patient; (3)
a video in LIS concerning safety in MRI has been realised.
**Conclusion:** Communication is a big part of the success of MRI radiological examination. Radiographers are the main interface to patients and it is important to tailor communication approaches to their needs and to get feedback from them. Tools supporting inclusion for deaf patients optimise image quality and enhance patient experience.
**Limitations:** It would be worth assessing radiographers both within the public and private sectors.
**Ethics committee approval:** No information provided by the submitter.
**Funding for this study:** No funding was received for this study.
**Author Disclosures:**
Michela Centenaro: Nothing to disclose
Giulia Cappellina: Nothing to disclose
Eleonora Stefani: Nothing to disclose

**09:30-11:00** **Research Stage 1**

### Research Presentation Session: Abdominal Viscera & GI Tract

### RPS 1801
### From guidelines to new approaches in liver tumour imaging

**Moderator**
C. Ayuso; Barcelona/ES
*(cayuso@clinic.cat)*

### RPS 1801-2
**Alternative surveillance using CT/MR improves clinical outcomes in high-risk patients with chronic hepatitis B**
*D. H. Lee*; Seoul/KR

**Purpose:** This study aimed to evaluate the outcome of alternative hepatocellular carcinoma (HCC) surveillance using CT/MR compared to US only in chronic hepatitis B (CHB) patients.
**Methods or Backround:** We enrolled consecutive CHB patients undergoing regular HCC surveillance, classifying into two groups: US only group and alternative surveillance group. The risk estimation for HCC in CHB (REACH-B) score was calculated to categorise high and low risk. Outcomes included 10-year overall survival (OS), size and Barcelona Clinic Liver Cancer (BCLC) stage of HCC, and OS after HCC diagnosis.
**Results or Findings:** A total of 2,024 patients were enrolled with 1,012 patients in each group. There was no significant difference in OS (96.0% in US only versus 96.8% in alternative surveillance; P=0.379). In both groups, HCC occurred in 66 patients. Medium size of HCC in the alternative surveillance was significantly smaller than US only (1.6 cm versus 2.1 cm; P<0.001). The rate of BCLC 0 stage HCC was also significantly higher in alternative surveillance than US only (71.2% [47/66] versus 42.4% [28/66]; P=0.003). OS after HCC diagnosis in the alternative surveillance group was significantly better than that in the US only group (83.0% versus 67.0%; P=0.025). In the high-risk group including 970 patients, alternative surveillance provided significantly better OS (97.3% versus 93.6%; P=0.029) and OS after HCC diagnosis (83.0% versus 60.6%; P=0.010) than US only. However, there was no significant difference in both OS (P=0.202) and OS after HCC diagnosis (P=0.937) in 1,054 patients with low risk.
**Conclusion:** Alternative surveillance using CT/MR enabled the detection of HCC at an earlier stage, with smaller size, than US only and had potential to improve OS after HCC diagnosis, especially for patients with high risk.
**Limitations:** The retrospective study design, prone to selection bias, was an identified limitation.
**Ethics committee approval:** This study was designed as a single-centre retrospective cohort study and approved by the institutional review board of Seoul National University Hospital (spproval number: H-2208-023-1346) with the waiver of requirement of informed consent.
**Funding for this study:** No funding was received for this study.
**Author Disclosures:**
Dong Ho Lee: Research/Grant Support: Research grants from Canon Medical System

### RPS 1801-3
**A comprehensive study on the feasibility and diagnostic potential of fluctuation imaging (FLI) in liver tumour assessment**
*D. H. Lee*, J. Y. Lee; Seoul/KR

**Purpose:** B-mode ultrasound (US) is commonly used for liver disease imaging, although it has limitations in characterising liver masses. This study aims to assess the clinical potential of a newly developed US technique called fluctuation imaging (FLI).
**Methods or Backround:** In this prospective exploratory study, we enrolled 65 patients diagnosed with liver tumours based on histopathology or typical imaging findings. All participants underwent US examination, which included FLI. FLI involved capturing approximately 100 frames of images during a 10-second breath-holding period to generate a color-coded FLI map. The presence of fluctuation signals within liver tumours on the FLI map was noted: the presence of increased fluctuation signal was represented as a yellow to red colour within the tumour. We compared the incidence of increased fluctuation signals using the chi-square test.

**Results or Findings:** FLI map creation was successful in 95.4% of cases (62/65), with three cases failing due to respiratory motion. Among the 62 analysed patients, 38 had 42 haemangiomas (median size: 1.9 cm), 13 had hepatocellular carcinoma (median size: 2.6 cm), two had cholangiocarcinoma (2.6 and 4.5 cm), and one each had cortical adenoma from hepato-adrenal fusion (3.0 cm), malignant mesothelioma (4.5 cm), and angiomyolipoma (6.1 cm). The remaining six participants had seven metastases from colon cancer (median size: 2.3 cm). Among the 42 haemangiomas, 23 (54.8%) exhibited increased fluctuation signals on the FLI map. In contrast, only three out of 25 non-haemangioma liver tumours (12.0%) showed increased fluctuation signals (54.8% for haemangioma's versus 12.0% for non-haemangiomas, P=0.001).
**Conclusion:** The acquisition of FLI maps during liver US examinations proved to be feasible, and the identification of increased fluctuation signals on the FLI map may assist in detecting haemangiomas.
**Limitations:** The small number of participants was an identified limitation.
**Ethics committee approval:** The institutional review board of Seoul National University Hospital approved this prospective study.
**Funding for this study:** Canon Medical Systems supported this study.
**Author Disclosures:**
Jae Young Lee: Nothing to disclose
Dong Ho Lee: Grant Recipient: Research grants from Canon Medical Systems

### RPS 1801-4

**Comparison of diagnostic guidelines for hepatocellular carcinoma on gadoxetic acid-enhanced liver magnetic resonance imaging**

*J-H. Yoon*, Y. K. Kim, W. Chang, J-I. Choi, B. J. Park, J-Y. Choi, H. S. Park, C. H. Lee, J. M. Lee; Seoul/KR
*(cinamon1@snu.ac.kr)*

**Purpose:** Non-invasive diagnostic guidelines for HCC vary, especially regarding hepatobiliary agent-enhanced magnetic resonance imaging (HBA-MRI). We evaluated the diagnostic performance of four guidelines and readers' judgment in diagnosing HCC using HBA-MRI in high-risk patients.
**Methods or Backround:** This retrospective study included treatment-naive patients at risk of HCC who underwent HBA-MRI from January 2015 to June 2018. Four radiologists, using a pre-programmed algorithm on a web-based platform, independently reviewed focal liver lesions (FLLs) according to four guidelines: American Association for the Study of Liver Diseases/Liver Imaging Reporting and Data System (AASLD/LI-RADS), Korean Liver Cancer Association-National Cancer Center (KLCA-NCC), European Association for the Study of the Liver (EASL), Asian Pacific Association for the Study of the Liver (APASL). Readers' judgment was also recorded. The guidelines' diagnostic performance was compared in all patients and subgroups. Additionally, the diagnostic odds ratio (DOR) was assessed.
**Results or Findings:** We analysed 2,445 FLLs in 2,237 patients; 69.3% (1,694/2,445) were HCC. KLCA-NCC demonstrated the highest accuracy (80.0%), followed by APASL, AASLD/LI-RADS (77.8%, 76.4%), and EASL (75.1%). APASL exhibited the highest sensitivity (89.1%), followed by KLCA-NCC (78.2%), while AASLD/LI-RADS displayed the highest specificity (89.6%), followed by EASL (88.1%). The DORs were 20.7 for AASLD/LI-RADS, 18.9 for KLCA-NCC, 16.8 for EASL, and 8.9 for APASL. The readers' judgment demonstrated higher accuracy than the guidelines (86.0%, P<0.001). In small (<20 mm) FLLs, Eastern guidelines showed higher accuracy than Western guidelines (P<0.001). Diagnostic accuracy of guidelines was 71.8%–79.5% in cirrhosis and 75.2%–82.3% in chronic hepatitis B without cirrhosis.
**Conclusion:** Eastern guidelines demonstrated high sensitivity, while Western guidelines displayed high specificity. KLCA-NCC achieved the highest accuracy, and AASLD/LI-RADS exhibited the highest DOR.
**Limitations:** The retrospective design inevitably introduced bias which we attempted to minimise by including a large population.
**Ethics committee approval:** This study was approved by the Institutional Review Board of Seoul National University Hospital and participating institutions.
**Funding for this study:** This work is financially supported by Bayer.
**Author Disclosures:**
Beom Jin Park: Nothing to disclose
Chang Hee Lee: Nothing to disclose
Jin-Young Choi: Nothing to disclose
Jeong Min Lee: Grant Recipient: Bayer
Young Kon Kim: Nothing to disclose
Won Chang: Nothing to disclose
Hee Sun Park: Nothing to disclose
Joon-Il Choi: Nothing to disclose
Jeong-Hee Yoon: Nothing to disclose

### RPS 1801-5

**Diagnostic performance of CT/MRI LI-RADS v2018 in non-cirrhotic steatotic liver disease**

J. Cao, A. Shon, L. S. Yoon, *J. R. Tse*; Stanford, CA/US

**Purpose:** A substantial proportion of patients with steatotic liver disease (SLD) develop hepatocellular carcinoma (HCC) without cirrhosis. Our purpose was to assess the diagnostic performance of CT/MRI LI-RADS v2018 (LI-RADS) among patients with non-cirrhotic SLD.
**Methods or Backround:** This IRB-approved, retrospective, single-centre study included 103 observations with histopathologic correlation from 77 adult patients who underwent liver CT/MRI from 2010-2023. All patients had histopathologic evidence of SLD without cirrhosis. Three board-certified abdominal radiologists (R1-R3) blinded to the tissue diagnoses assessed each observation by LI-RADS criteria and assigned a final category. Inter-reader agreements were assessed with Cohen's kappa. The positive predictive value (PPV), sensitivity, specificity, and accuracy of identifying HCC and overall malignancy were calculated.
**Results or Findings:** Of 103 observations, 59 (57%) were benign and 44 (43%) were malignant. PPV for HCC was 0-0% for LR-1, 0-0% for LR-2, 0-11% for LR-3, 13-22% for LR-4, 75-88% for LR-5, 0-8% for LR-M, and 50-75% for LR-TIV. For malignancy (including HCC), PPV was 0-0% for LR-1, 0-12% for LR-2, 5-12% for LR-3, 19-33% for LR-4, 78-88% for LR-5, 65-91% for LR-M, and 100-100% for LR-TIV. For LR-5 in detecting HCC, sensitivity was 83%, 79%, 83% (R1-3, respectively), specificity was 89%, 96%, 92%, and accuracy was 87%, 91%, and 89%. For composite categories of LR-5, LR-M, or LR-TIV in detecting overall malignancy, sensitivity was 89%, 89%, and 86%, specificity was 86%, 95%, and 81%, and accuracy was 87%, 92%, and 84% respectively. Most common false positives for LR-5 were due to hepatocellular adenomas. Inter-reader agreements for major features ranged from 0.660-0.833 and was 0.751 for the final category.
**Conclusion:** CT/MRI LI-RADS v2018 can be applied to non-cirrhotic SLD patients with high but imperfect specificity.
**Limitations:** This was a single-centre, retrospective study with selection bias of histologically-proven observations.
**Ethics committee approval:** The study was approved by the university Institutional Review Board
**Funding for this study:** No funding was received for this study.
**Author Disclosures:**
Jennie Cao: Nothing to disclose
Luke S. Yoon: Consultant: Intuitive Surgical, Inc
Andrew Shon: Nothing to disclose
Justin Ruey Tse: Consultant: Intuitive Surgical, Inc; Consultant: Absolutions, Inc

### RPS 1801-6

**Value of preoperative magnetic resonance imaging for diagnosis of LR-3 and LR-4 lesions coexisting with main hepatocellular carcinoma**

*Y. Xiao*, C. Yang, M. Zeng; Shanghai/CN
*(xiaoyuyao98@163.com)*

**Purpose:** The diagnostic performance of magnetic resonance imaging (MRI) features favouring hepatocellular carcinoma (HCC) in LR-3 and LR-4 observations in high-risk patients have been evaluated in previous studies. Our objective was to explore which preoperative clinical data and conventional MRI findings may indicate the presence of HCC in LR-3 and LR-4 lesions of HCC patients.
**Methods or Backround:** In this study, we enrolled 102 patients with histopathologically confirmed HCC coexisting with 110 LR-3 and LR-4 lesions (HCCs group [n = 66], non-HCCs group [n = 44]). Two radiologists retrospectively assessed the preoperative MRI features, and each lesion was assigned according to LI-RADS. Preoperative clinical data were also evaluated. Logistic regression analyses were used to assess the relative value of these parameters as potential predictors of HCC.
**Results or Findings:** On multivariate analysis, the presence of restricted diffusion (OR: 18.590, p<0.001), delayed enhancement (OR: 0.113, p<0.001), and T2WI mild-moderate hyperintensity (OR: 3.084, p=0.048) were found to be independent predictors for HCC diagnosis. The sensitivity and specificity of the above independent variables and their combination for the diagnosis of HCC ranged from 47.0–80.3% and 56.8–97.7%, respectively. Combining these three findings for the prediction of HCC resulted in a specificity greater than 97%, and the AUC further increased to 0.874.
**Conclusion:** The presence of restricted diffusion, delayed enhancement, and T2WI mild-moderate hyperintensity can be useful features in risk stratifying coexisting LR-3 and LR-4 lesions in HCC patients.
**Limitations:** Firstly, this was a single-centre retrospective study lacking longitudinal data. Secondly, the number of LR-4 lesions was also relatively small in our study, so our results might not represent the true spectrum of LR-3 and LR-4 observations in HCC patients.

**Ethics committee approval:** This retrospective study was approved by the local Ethics Committee, and written informed consent was waived due to its retrospective design.
**Funding for this study:** This study received funding from the National Natural Science Foundation of China (grant number: 82171897, 82272078).
**Author Disclosures:**
Mengsu Zeng: Nothing to disclose
Chun Yang: Nothing to disclose
Yuyao Xiao: Nothing to disclose

### RPS 1801-7

**A new criterion for diagnosis of subcentimetre hepatocellular carcinoma in treatment-naive patients using tumour biomarker and gadoxetic acid-enhanced MRI features**
*P. Huang*, F. Wu, C. Wang, Y. Xiao, G. Miao, C. Yang, M. Zeng; Shanghai/CN
*(chaffee_hp@163.com)*

**Purpose:** Based on imaging findings, current diagnostic criteria for subcentimetre hepatocellular carcinoma (HCC) have suboptimal diagnostic performances. The aim of the present study is to develop a diagnostic nomogram including gadoxetic acid-enhanced MRI features and tumour biomarkers.
**Methods or Backround:** This study enrolled treatment-naive patients with chronic hepatitis B who had a solitary subcentimetre observation from April 2016 to March 2023. The final diagnosis was confirmed by pathology for HCC and pathology or follow-up for non-HCC controls. Logistic regression analysis for imaging features and tumour biomarkers was used to identify independent predictors associated with HCC that were then incorporated into the nomogram. Diagnostic performances of the new criterion were compared to wash-in and -out.
**Results or Findings:** A total of 224 patients (116 with HCC and 108 with non-HCC) were divided into a training cohort (including 155 patients) and temporal validation cohort (including 69 patients). The factors associated with HCC diagnosis were alpha-fetoprotein >20 ng/mL or des-gamma-carboxy prothrombin >40 IU/mL, T2WI mild-moderate hyperintensity, arterial-phase hyperenhancement, portal-phase washout, and transitional-phase hypointensity. Incorporating these 5 factors, the nomogram achieved good concordance indexes of 0.92 and 0.95 in diagnosing subcentimetre HCC in the training and temporal validation cohorts, respectively, and had well-fitted calibration curves. Using a nomogram score of 296 as a cut-off, the new criterion yielded higher sensitivity than wash-in and -out (training cohort: 72.0% versus 51.2%, P<0.001; validation cohort, 79.4% versus 47.1%, P<0.001) without a significant decrease in specificity (training cohort: 90.4% versus 91.8%, P>0.999; validation cohort, 91.4% versus 91.4%, P>0.999).
**Conclusion:** Including tumour biomarkers in the diagnostic algorithm may improve the diagnostic performance for subcentimetre HCC.
**Limitations:** Our results may be limited in their generalisability, especially in regions other than HBV-endemic areas.
**Ethics committee approval:** The institutional review board of Zhongshan Hospital, Fudan University (number: B2020-372R) approved this study.
**Funding for this study:** Funding was received from The Clinical Research Plan of SHDC (number: SHDC2020CR1029B), National Natural Science Foundation of China (number: 82171897), and National Natural Science Foundation of China (number: 82371923).
**Author Disclosures:**
Mengsu Zeng: Nothing to disclose
Gengyun Miao: Nothing to disclose
Cheng Wang: Nothing to disclose
Peng Huang: Nothing to disclose
Yuyao Xiao: Nothing to disclose
Chun Yang: Nothing to disclose
Fei Wu: Nothing to disclose

### RPS 1801-8

**Quantitative CT analysis of hepatocellular carcinoma nodules in liver transplant candidates: impact of volume of interest and contrast phase on recurrence prediction**
*F. Rizzetto*, R. Manzini, L. Centonze, C. B. Monti, S. Garziano, J. Di Napoli, L. A. Carbonaro, A. Vanzulli; Milan/IT
*(francesco.rizzetto@unimi.it)*

**Purpose:** To evaluate how different lesion volumes-of-interest (VOIs) and contrast phases affect quantitative CT analysis of hepatocellular carcinoma (HCC) in liver transplant candidates.
**Methods or Backround:** Liver transplant candidates from 2010 to 2019, with waitlist placement CT showing suspicious HCC nodules (LI-RADS=4-5) and no treatment, were retrospectively included. HCC nodules, with or without a peritumoural region of 5, 10, and 15 mm, and "background" liver parenchyma were contoured across arterial, venous, and delayed phases. Radiomic features (RFs), both directly extracted from segmentations and normalised for "background" parenchyma, were computed and compared for inter-VOIs and inter-phase relative change (RC), and autocorrelation using Spearman's rho. RFs with RC<10% and rho>0.9 were considered redundant. Non-redundant RFs with different distribution in patients with and without post-transplant recurrence were selected to build multiple logistic regression models. Predictive capability for post-transplant recurrence was assessed comparing areas under the curve (AUC) from receiver operating characteristic analysis.
**Results or Findings:** The CT scans of 53 patients were selected, for a total of 1,260 segmentations from 105 nodules, with eight (15%) recurrence cases. Changing VOIs and phases, the number of redundant RFs was 14-26% and 16-34%, respectively, while 4% of RFs were autocorrelated. Selected non-redundant RFs allowed the building of eight predictive models (pseudo-r2=0.13-0.46), each comprising 5-12 RFs, with AUC ranging from 0.77 to 90 (p<0.02). Best performance was achieved when segmenting HCC nodules with or without 5 mm of peritumoural region in arterial phase. Venous and delayed phases showed predictive capability only for segmentations including 10-15 mm of peritumoural region after normalisation for background parenchyma.
**Conclusion:** Selection of optimal VOI and contrast phase combination is critical to maximize post liver transplant HCC recurrence prediction using quantitative CT imaging.
**Limitations:** The retrospective single-centre design and the limited sample size are the main limitations of the study.
**Ethics committee approval:** The local ethics committee approved the retrospective data collection in an anonymous, aggregated form.
**Funding for this study:** No funding was received for this study.
**Author Disclosures:**
Leonardo Centonze: Nothing to disclose
Francesco Rizzetto: Other: Bracco Imaging
Luca Alessandro Carbonaro: Nothing to disclose
Jacopo Di Napoli: Nothing to disclose
Caterina Beatrice Monti: Other: Bracco Imaging
Sergio Garziano: Nothing to disclose
Angelo Vanzulli: Nothing to disclose
Rebecca Manzini: Nothing to disclose

### RPS 1801-9

**Development of the MIEM score to predict survival after recurrence in patients with recurrent HCC following curative resection**
*H. Wei*, B. Song; Chengdu/CN

**Purpose:** This study aimed to create a predictive score for survival after recurrence (SAR) based on MRI features and clinical-pathological characteristics in patients with recurrent HCC following initial curative resection.
**Methods or Backround:** This single-centre, retrospective study included consecutive patients who underwent contrast-enhanced MRI within one month before curative resection for primary HCC and developed recurrence during follow-up from December 2011 to June 2021. Clinical (i.e. age, sex, aetiology, BCLC stage, and AFP level), pathological (i.e. tumour differentiation, microvascular invasion, liver capsule invasion, and cirrhosis), and recurrence-related data (i.e. adjuvant therapy, early versus late recurrence, and intrahepatic versus extrahepatic recurrence) were collected. MRI images were independently reviewed by two radiologists in terms of all LI-RADS version 2018 imaging features and several non-LI-RADS imaging features. Uni- and multivariable Cox regression analyses were used to identify variables associated with SAR. A regression-based predictive model was constructed with five-fold cross-validation.
**Results or Findings:** A total of 151 patients with recurrent HCC were included (median age, 53 years; 135 men). During a median follow-up period of 55 months, 31% (47/151) of patients experienced death. Predictors for SAR included non-smooth tumour margin (hazard ratio [HR] =4.6), rim arterial phase hyperenhancement (HR=1.9), extrahepatic recurrence (HR=1.9), and male sex (HR=0.5). By incorporating the above predictors, the MIEM score demonstrated a C-index of 0.71 (95% confidence interval: 0.64, 0.79). Using 11 as the threshold of the MIEM score, all patients were categorised into prognostically distinct low- and high-risk groups (P<0.001).
**Conclusion:** The MIEM score may serve as a useful tool to help predict SAR for patients with recurrent HCC following hepatectomy.
**Limitations:** The single-centre nature of the study, the retrospective study design, and lack of external validation were identified as limitations.
**Ethics committee approval:** This study was approved by the Biomedical Ethics Review Committee of West China Hospital, Sichuan University (approval number: 2022-651). The requirement for informed consent was waived (due to the retrospective design) by the Biomedical Ethics Review Committee of West China Hospital, Sichuan University.
**Funding for this study:** This work was supported by the National Natural Science Foundation of China (grant number: U22A20343).
**Author Disclosures:**
Hong Wei: Nothing to disclose
Bin Song: Grant Recipient: Prof. Bin Song is the grant recipient of the National Natural Science Foundation of China (Grant No. U22A20343).

### RPS 1801-10

**Prognostic role of CT and MRI of resectable intrahepatic mass forming cholangiocarcinoma**

*S. De Vizio*, E. Genco, F. M. Sessa, M. G. Brizi, E. Panettieri, F. Giuliante, E. Sala; Rome/IT
*(silvia.devizio91@gmail.com)*

**Purpose:** Imaging is crucial for diagnosis as well as for prognosis of intrahepatic mass forming cholangiocarcinoma. In the last decade some studies focused on diffusion weighted imaging (MRI) and arterial enhancement's (CT and MRI) role in prognosis stratification of resectable intrahepatic cholangiocarcinoma. The aim of this retrospective study was to evaluate the best prognostic imaging biomarkers for resectable intrahepatic mass forming cholangiocarcinomas.
**Methods or Backround:** We retrospectively evaluated 123 patients with histologically proven intrahepatic cholangiocarcinoma, surgically treated in our university hospital from 2010 to 2022. Patients were divided in two groups: a multiphasic contrast-enhanced CT group and a multiphasic hepato-specific contrast-enhanced MR-DWI group. Each group was further divided into resectable lesion and unresectable lesion sub-groups. The percentage of arterial enhancement at CT and MR was categorised as hypervascular (>50% of the lesion), peripherally enhancing (10-50%), or hypovascular (<10%); DWI was qualitatively evaluated on ADC maps and lesions graded as diffusion restriction (<1/3 of the lesion) and diffusion restriction (>1/3).
Analysis was performed by two expert readers.
Overall survival at 1-year and 3-years was assessed by Kaplan-Meyer curves whose differences were analysed by Cox analysis.
**Results or Findings:** A total of 26 patients were included in the MRI group (6 hypervascular, 14 peripheral, 6 hypovascular), and 35 in the CT group (7 hypervascular, 11 peripheral, 17 hypovascular); 62 patients were excluded because of poor quality of arterial phase and/or DWI images.
In both groups overall survival at 1-year was higher for the hypervascular group compared to the hypovascular group (87.3% versus 26.2%).
In the MRI group, 1-year survival was higher in the subgroup with diffusion restriction of <1/3 of the lesion.
At Cox proportional hazards model analysis, hypervascularity was associated with a better overall survival.
**Conclusion:** CT/MR hypervascularity and lower extent of diffusion restriction are both positive prognostic biomarkers.
**Limitations:** The retrospective study design was an identified limitation.
**Ethics committee approval:** No information provided by the submitter.
**Funding for this study:** No funding was received for this study.
**Author Disclosures:**
Filippo Maria Sessa: Nothing to disclose
Maria Gabriella Brizi: Nothing to disclose
Elena Panettieri: Nothing to disclose
Felice Giuliante: Nothing to disclose
Silvia De Vizio: Nothing to disclose
Evis Sala: Nothing to disclose
Enza Genco: Nothing to disclose

### RPS 1801-11

**Photon-counting detector CT for liver lesion detection: optimising virtual monoenergetic images across different sizes and doses**

D. Racine[1], V. Mergen[2], A. Viry[1], T. Frauenfelder[2], H. Alkadhi[2], V. Vitzthum[1], *A. Euler*[3]; [1]Lausanne/CH, [2]Zurich/CH, [3]Baden/CH

**Purpose:** To evaluate the optimal energy level of virtual monoenergetic images (VMI) from photon-counting detector CT (PCD-CT) for the detection of liver lesions as a function of phantom size and radiation dose.
**Methods or Backround:** An anthropomorphic abdominal phantom with liver parenchyma and lesions was imaged on a dual-source PCD-CT at 120 kVp. The lesions were hypo- or hyperattenuating and had diameters of 5-10 mm. Rings of fat-equivalent material were added to emulate medium- or large-sized patients. The medium size was imaged at 5, 2.5, and 1.25 mGy and the large size at 5 and 2.5 mGy, respectively. Each setup was imaged ten times. For each setup, VMIs from 40 to 80 keV at 5 keV increments were reconstructed with quantum iterative reconstruction at a strength level of 4 (QIR-4). Lesion detectability was measured as area under the receiver operating curve (AUC) using a channelised hotelling model observer with ten dense differences of Gaussian channels.
**Results or Findings:** Overall, highest detectability was found at 65 and 70 keV for both hypo- and hyperattenuating lesions in the medium and large phantom, independent of radiation dose (AUC range: 0.910-0.999 for the medium and 0.935-0.987 for the large phantom, respectively). Lowest detectability was found at 40 keV irrespective of the radiation dose and phantom size (AUC range: 0.784-0.993). Reducing radiation dose decreased lesion detectability more strongly at 40-50 keV as compared with 65-75 keV. At equal radiation dose, detection as a function of VMI energy differed more strongly for the large size as compared with the medium size phantom (12 % versus 6 %).
**Conclusion:** Detectability of hypo- and hyperattenuating liver lesions differed between VMI energies for different phantom sizes and radiation doses. VMI at 65 and 70 keV yielded highest detectability, independent of phantom size and radiation dose.
**Limitations:** The phantom study design was an identified limitation.
**Ethics committee approval:** Ethics committee approval was not needed because of the design as a phantom study.
**Funding for this study:** No funding was received for this study.
**Author Disclosures:**
Thomas Frauenfelder: Research/Grant Support: The institute receives financial support from Bayer and Siemens Healthineers unrelated to this work
Victor Mergen: Nothing to disclose
Veronika Vitzthum: Nothing to disclose
Anaïs Viry: Nothing to disclose
Damien Racine: Nothing to disclose
Andre Euler: Speaker: Siemens Healthineers
Hatem Alkadhi: Speaker: Siemens Healthineers; Research/Grant Support: The institute receives financial support from Bayer and Siemens Healthineers unrelated to this work

### RPS 1801-12

**Nomogram development and validation based on Gd-EOB-DTPA-enhanced MRI to predict IDH1 mutation for intrahepatic cholangiocarcinoma**

*X. Zhou*, M. Chen, D. Xu, S-T. Feng; Guangzhou/CN
*(zhouxq5@mail2.sysu.edu.cn)*

**Purpose:** Isocitrate dehydrogenase 1 (IDH1) mutation inhibitors show promise for targeted therapy in intrahepatic cholangiocarcinoma (ICC), but only for IDH1 mutated tumours. The purpose of this study is to establish and validate a model to predict IDH1 gene mutation in ICC based on preoperative Gd-EOB-DTPA-enhanced magnetic resonance imaging (MRI) images.
**Methods or Backround:** A total of 85 ICCs were randomly assigned to the training set (n=59) and the test set (n=26). Next generation sequencing and immunohistochemical analysis were performed for IDH1 mutation status. Preoperative imaging features of Gd-EOB-DTPA-enhanced MRI were qualitatively and quantitatively reviewed by two radiologists. Nomograms were developed based on the significant variables for differentiating the IDH1-mutated and IDH1-unmutated ICCs in the training sets.
**Results or Findings:** The IDH1 mutation rate was 20% (17/85). After filtering the factors (p<0.1) based on univariate analysis, the serum AFP value (p=0.17), intratumoural vessel (p=0.01), location of liver lobe (p=0.14), T1 change ratio (p=0.004), T2 central brightness (p=0.01) and ADC value (p=0.008) were incorporated into the nomogram for predicting IDH1-mutated ICCs. Combing all six significant variables provided a diagnostic accuracy of 88%, sensitivity of 75%, and a specificity of 90% for identifying IDH1 mutation status in the test set. The area under the receiver operating characteristic curve value of the logistical regression coefficient-based nomogram was 0.927 (95% confidence intervals, 0.838-1.000) and 0.881 (95% confidence intervals, 0.690-1.000) for the training and test sets. The nomogram exhibited good calibration and clinical usefulness.
**Conclusion:** Gd-EOB-DTPA-enhanced MRI-based nomogram effectively predicts IDH1 mutation status in ICCs and may assist clinicians in pretreatment decisions.
**Limitations:** Firstly, this is a retrospective study with potential patient selection bias. Secondly, this was a single-centre study with a small sample size.
**Ethics committee approval:** The study was approved by the institutional review board and ethical committee of Sun Yat-sen University.
**Funding for this study:** Funding was received from the National Natural Science Foundation of China (82271958, 81971684).
**Author Disclosures:**
Shi-Ting Feng: Nothing to disclose
Danyang Xu: Nothing to disclose
Xiaoqi Zhou: Nothing to disclose
Meicheng Chen: Nothing to disclose

### RPS 1801-13

**Detection of spoke-wheel pattern in focal nodular hyperplasia with microvascular flow imaging in a preliminary study**

B. Zsély, *A. D. Rónaszéki*, B. K. Budai, R. Surendranath, R. Stollmayer, Z. Zsombor, G. Győri, P. Maurovich-Horvat, P. N. Kaposi; Budapest/HU
*(ronaszeki.aladar@gmail.com)*

**Purpose:** Microvascular flow imaging (MVFI) is a novel Doppler ultrasound (US) technique to detect slow flow in capillary vessels. The spoke-wheel vascularity pattern is characteristic of focal nodular hyperplasia (FNH), and it is consistently detectable with MVFI. The identification of the typical patterns of vascularity, including the spoke-wheel pattern with MVFI can expedite diagnosis, spare patients from unnecessary procedures, and reduce costs.

**Methods or Backround:** This study retrospectively collected MVFI US images of 50 patients followed for known focal liver lesions (FLL). The lesions were diagnosed by contrast-enhanced ultrasound (CEUS), biopsy, or magnetic resonance imaging (MRI) with a liver-specific contrast agent. Patients were examined using a Samsung RS85 Prestige scanner. The vascularity of the lesions was evaluated with either conventional colour Doppler US or directional power Doppler imaging using the S-FlowTM application. We used the MV-FlowTM application for microvascular flow imaging by recording a 5-10 second-long video in a breath-hold. The Wilk test was used to check the normality of the data. Continuous variables were analysed with the Mann-Whitney U-test, categorical variables with Fisher's exact test, and chi-square test. A logistic regression analysis was performed with the individual morphological characteristics, the variables were characterised by odds ratio (OR) value, sensitivity, and specificity.
**Results or Findings:** The study patients included 21 FNHs, 7 haemangiomas, 9 hepatocellular carcinomas (HCC), and 13 liver metastases. The spoke-wheel pattern detectable with MVFI was detected in a higher number in cases of FNHs (OR>100 [95% CI: 45.365 - Inf]; p<0.001), which proved to be specific (1.0) and showed high sensitivity (0.955).
**Conclusion:** Our preliminary results show the spoke-wheel pattern can be easily detected with MVFI, even in the case of small FNHs, whose vascularisation pattern can help distinguish them from other FLLs.
**Limitations:** The retrospective study design was an identified limitation.
**Ethics committee approval:** The study has been approved by the institutional ethics committee of our university (SE-RKEB 140/2020).
**Funding for this study:** The authors received no funding for this study.
**Author Disclosures:**
Aladár David Rónaszéki: Nothing to disclose
Pál Maurovich-Horvat: Nothing to disclose
Boglárka Zsély: Nothing to disclose
Zita Zsombor: Nothing to disclose
Gabriella Györi: Nothing to disclose
Reddy Surendranath: Nothing to disclose
Bettina Katalin Budai: Nothing to disclose
Pál Novák Kaposi: Nothing to disclose
Robert Stollmayer: Nothing to disclose

**09:30-11:00** **Research Stage 2**

### Research Presentation Session: Breast

### RPS 1802

### Advances in breast MRI techniques

**Moderator**
N. Pötsch; Vienna/AT

### RPS 1802-2

**Abbreviated breast magnetic resonance imaging (MRI) protocols in a very high-risk population: friend or foe?**

L. Metaxa, V. Papalouka, N. Vidyaprakash, S. Lee, S. Dani, *T. Suaris*; London/UK

**Purpose:** AbbMRI aims to provide a cost-effective study without compromising diagnostic accuracy. We aim to assess the diagnostic performance of AbbMRI in high-risk breast cancer screening.
**Methods or Backround:** Four experienced breast radiologists retrospectively reviewed the AbbMRI protocol in 236 MRI breast studies performed for very high-risk between January 2018 – 2021. Readers were blinded to prior imaging, history, and patient outcomes. The protocol consisted of six sequences: pre-contrast T1W, T2W axial, two dynamic postcontrast, subtracted axial sequences and MIP reconstruction. Results were compared with existing reports of the full protocol (19 sequences).
**Results or Findings:** From the 236 MRI studies, four invasive malignancies and one B3 lesion (radial scar) were identified. The average time to read the AbbMRI was 69 seconds (range: 28 to 240 seconds; 95% CI: 63, 74 seconds). The inter-rater rate (IRR) between the abbreviated and full protocol was: 91.5%. The recall rate for AbbMRI was 9.5%, and 6.1% for full protocol. Sensitivity achieved by AbbMRI (100%) was equal to the full protocol (no cancers were missed). NPV were high for both reading sessions (100%). Specificity and PPV of the abbreviated MRI was lower compared to the full protocol (91.2 versus 94.7, and 8.9 versus 13.8%, respectively) (p value .01, a=0.5).
**Conclusion:** Abbreviated MRI is feasible in screening MRI of high risk patients, with sensitivity/CDR comparable to the full MRI protocol, but with a small increase in the recall rates. Abbreviated breast MRI can be considered as a safe alternative to conventional MR studies in UK very high-risk screening, reducing MR waiting times/backlog.
**Limitations:** Identified limitations were: (1) this was a retrospective study, with data for the full protocol collected from existing reports, where the radiologists were not blinded to the patients' personal history; (2) the abbreviated MRI was not double read.
**Ethics committee approval:** This study was approved by the Director of Research.
**Funding for this study:** No funding was received for this study.
**Author Disclosures:**
Tamara Suaris: Nothing to disclose
SookYeen Lee: Nothing to disclose
Nithya Vidyaprakash: Nothing to disclose
Linda Metaxa: Nothing to disclose
Vasiliki Papalouka: Nothing to disclose
Shefali Dani: Nothing to disclose

### RPS 1802-3

**Evaluation of pure DCIS with abbreviated breast MRI**

A. Arslan, *N. Basdogan*, C. S. Topal, F. Ezberci; Istanbul/TR
*(nurbanubasdogan@gmail.com)*

**Purpose:** The most reliable imaging method for detecting breast cancer is magnetic resonance imaging (MRI) of the breast. 'Abbreviated MRI' imaging protocol was developed to shorten scanning and evaluation time to screen large populations. The aim of our study is to determine the lesion detection rate with the abbreviated MRI protocol in patients with a histopathological diagnosis of pure DCIS (ductal carcinoma in situ without any invasive components) in the breast
**Methods or Backround:** Patients who underwent breast MRI between May 2021 and October 2023 in our institution were reviewed retrospectively by two radiologists (a radiologist with nine years of breast MRI experience, and a radiology resident with two years of MR experience). All the MRIs were carried out using a 1.5T MRI machine (GE Optima MR450w). Patients diagnosed with DCIS by biopsy were included in the study. Cases who had a history of breast cancer, had invasive tumour foci in addition to DCIS, and cases who received neoadjuvant chemotherapy, were excluded from the study. Abbreviated MRI protocol includes T1WI without contrast, T1WI with first minute contrast, and one minute subtraction image.
**Results or Findings:** A total of 36 DCIS diagnosed by biopsy were evaluated in our centre. Eleven cases were excluded from the study because they had invasive tumour foci in addition to DCIS. Except for five of the 25 pure DCIS, the others showed contrast enhancement in the first minute of contrast and subtraction images (sensitivity 80.0%). Of the five cases, one case had low-grade DCIS, two cases had intermediate-type DCIS, and two cases had high-grade DCIS. The mean age of the patients included in the study was 51.72 (39-80) years.
**Conclusion:** Pure DCIS could be detected with the high sensitivity abbreviated MRI protocol.
**Limitations:** The limited number of patients and the retrospective nature of the research are limitations of this study.
**Ethics committee approval:** The study was approved by Umraniye Reserch and Training Hospital.
**Funding for this study:** No funding was received for this study.
**Author Disclosures:**
Nurbanu Basdogan: Nothing to disclose
Fikret Ezberci: Nothing to disclose
Aydan Arslan: Nothing to disclose
Cumhur Selcuk Topal: Nothing to disclose

### RPS 1802-4

**Abbreviated vs full protocol in breast MRI: a meta-analysis comparing their diagnostic performances**

*O. Battaglia*, F. Pesapane, A. Rotili, O. D'Ecclesiis, S. Penco, G. Signorelli, L. Nicosia, S. Gandini, E. Cassano; Milan/IT
*(ottavia.battaglia@gmail.com)*

**Purpose:** In the last decade, the interest in magnetic resonance imaging abbreviated protocols (AB-MRI) has grown as shorter scan times translate into cost savings, enhanced patient tolerance and improved accessibility.
The aim of this meta-analysis is to compare the diagnostic performances of AB-MRI and full protocol MRI (FP-MRI) in breast cancer assessment.
**Methods or Backround:** We conducted a systematic review and meta-analysis from 01/11/2019 up to 12/12/2022. A biavariate model was used to calculate the summary estimates of sensitivity and specificity. Random effect models were used to calculate summary AUC and 95% CIs. Probability distributions for negative and positive predictive values were obtained.

**Results or Findings:** Out of 367 studies, a total of 11 studies were included. The summary sensitivity estimated from the AB-MRI compared to the FP-MRI is slightly lower (86.2%, 95% CI: 82.1-89.6 and 93.7%, 95% CI: 88.7-96.6, respectively). Summary estimates (SEs) of specificity are similar (79.7%, 95% CI: 73.2-84.8 for AB-MRI and 78.0%, 95% CI: 70.6-84.0 for FP-MRI). A better accuracy has emerged for FP-MRI (84.4% for AB-MRI and 87.6% for FP-MRI). The SEs of positive predictive value is similar (22.3% for AB-MRI and 20.4% for FP-MRI).
**Conclusion:** AB-MRI protocols have emerged as a potential alternative to overcame FP-MRI limits, offering increased efficiency and cost-effectiveness by reducing acquisition and reading times, improving accessibility, and enhancing the patient experience with similar diagnostic performances. Further research is needed to optimise the AB-MRI and to better define its indications. This includes investigating the role of machine learning algorithms to enhance lesion detection and characterisation within the AB-MRI. Long-term outcome studies are needed to evaluate AB-MRI's impact on patient outcomes, including detection rates and interval cancers.
**Limitations:** An identified limitation was the heterogeneity of the proposed AB-MRI protocols in the included studies.
**Ethics committee approval:** No information provided by the submitter.
**Funding for this study:** No funding was received for this study.
**Author Disclosures:**
Ottavia Battaglia: Nothing to disclose
Anna Rotili: Nothing to disclose
Sara Gandini: Nothing to disclose
Luca Nicosia: Nothing to disclose
Oriana D'Ecclesiis: Nothing to disclose
Silvia Penco: Nothing to disclose
Giulia Signorelli: Nothing to disclose
Filippo Pesapane: Nothing to disclose
Enrico Cassano: Nothing to disclose

### RPS 1802-5

**Enhancing the Kaiser score for unenhanced breast MRI**
*N. Pötsch*, P. Clauser, P. Kapetas, T. H. Helbich, P. A. Baltzer; Vienna/AT

**Purpose:** Unenhanced abbreviated breast MRI protocols aim to reduce scanning time and costs while enhancing access to breast MRI. However, in order to further exploit the advantages of the Kaiser score, a well-established clinical decision rule for breast MRI, it must be adapted to unenhanced protocols.
**Methods or Backround:** We studied 120 patients who underwent breast MRI between 2021 and 2023 for further evaluation of suspicious or equivocal findings in conventional imaging (BI-RADS 0, 3-5 in mammography and ultrasound) or for staging purposes (BI-RADS 6). All patients underwent biopsy for histologic verification or were followed for a minimum of 12 months to confirm lesion stability. MRI scans were conducted using 1.5T or 3T scanners using dedicated breast coils and a protocol in line with international recommendations additionally including DWI and ADC. Lesion characterisation relied solely on T2w and DWI/ADC-derived features (such as lesion type, margins, shape, internal signal, surrounding tissue findings, ADC value). Statistical analysis was done using decision tree analysis with benign (B2, B3) and malignant (B5a/b) as dependent variables.
**Results or Findings:** We analysed a total of 161 lesions, including 81 non-mass lesions, with a malignancy rate of 40%. The lesion margins (spiculated, irregular, or circumscribed) were identified as the most critical criterion within the decision tree. Further subclassification considered the ADC value as the second most important criterion. The resulting score demonstrated a strong diagnostic performance with an AUC of 0.840, providing clear rule-in and rule-out criteria.
**Conclusion:** Lesion characterisation in unenhanced MRI is feasible, with lesion margins and ADC value as the most important criteria.
**Limitations:** An identified limitation was that our analysis did not address lesion detection.
**Ethics committee approval:** Informed consent was waived.
**Funding for this study:** This study received no funding.
**Author Disclosures:**
Pascal A.T. Baltzer: Nothing to disclose
Nina Pötsch: Nothing to disclose
Thomas H. Helbich: Nothing to disclose
Panagiotis Kapetas: Nothing to disclose
Paola Clauser: Nothing to disclose

### RPS 1802-6

**Role of ultra-fast breast MRI in characterisation of breast masses: a step toward a faster MRI technique**
*R. Karam Mahmoud Mohammed*, D. M. Bayoumi, F. A. Shokeir; Mansoura/EG
*(rasha90karam@gmail.com)*

**Purpose:** To evaluate the efficacy of parameters derived from ultra-fast breast MRI, maximum slope (MS), initial enhancement phase (IE phase) and time to enhancement (TTE), in differentiation of benign and malignant breast lesions.
**Methods or Backround:** This is a prospective study that included 264 patients with 273 breast lesions, from which 120 were pathologically proven to be benign and 153 were pathologically proven to be malignant. An ultra-fast study was performed between the pre-contrast study and the first postcontrast sequence in the dynamic protocol using the TWIST sequence without fat suppression. The TWIST sequence was composed of a pre-contrast phase followed by 15 postcontrast TWIST series with a temporal resolution of 4.32 seconds each. We put a ROI at the hot spot of the lesion on subtracted images and a wash-in curve was automatically generated. From the ultra-fast wash in curve parameters were manually calculated (TTE and MS).
**Results or Findings:** TTE was significantly lower in malignant lesions compared to benign ones (median=8 seconds [0-20] versus 20 seconds [8-40], respectively. P value<0.001). However, MS was higher in malignant lesions compared to benign ones (median=15%/s [2-140] versus 5%/s [1-17] respectively. P value=<0.001). TTE cut-off value of 15.5 seconds showed a sensitivity of 78.4%, specificity of 80%, AUC of 0.876, and accuracy of 79%. However, MS, a cut off value of 7.25 %/s showed sensitivity of 82.4%, specificity of 70%, AUC of 0.86 and accuracy of 76.9%.
**Conclusion:** Ultra-fast breast MRI can be used as a faster and accurate MRI technique for differentiation between benign and malignant breast lesions.
**Limitations:** Identified limitations were: (1) the relatively small number of cases and (2) the fact that the ultra-fast technique was not compared to the conventional dynamic MRI technique.
**Ethics committee approval:** The study was approved by our institution's ethics committee (Mansoura Faculty of Medicine Institutional Research Board, reference number: R.23.05.2174). Written informed consent was obtained from all patients included in the study.
**Funding for this study:** No funding was received for this study.
**Author Disclosures:**
Rasha Karam Mahmoud Mohammed: Nothing to disclose
Dalia Mohammed Bayoumi: Nothing to disclose
Farah Ahmed Shokeir: Nothing to disclose

### RPS 1802-7

**Application of the Kaiser score in the assessment of suspicious contrast-enhancing lesions on breast MRI in patients with equivocal conventional imaging findings**
*K. Obradovic*, I. Petkovic, D. Pavlovic Stankovic, V. Urban, Z. C. Milosevic; Belgrade/RS

**Purpose:** To assess whether applying the Kaiser score might downgrade breast MRI BI-RADS categories in patients with equivocal conventional imaging findings (mammography and/or US) and avoid unnecessary biopsies.
**Methods or Backround:** Equivocal conventional imaging findings are one of the indications for problem-solving breast MRI. Generally, a combination of high sensitivity (98–100%) and suboptimal specificity (up to 88%) is inherent to breast MRI, which frequently results in false-positive diagnoses. Our single-centre study enrolled 61 consecutive patients from January 2017 to December 2018, with equivocal conventional imaging findings and 69 breast lesions on MRI (62 lesions in BI-RADS category 4, seven in BI-RADS category 5), undergoing US-CNB and/or surgery. All MRI examinations were re-evaluated after 4 years of the patients' follow-up, using the Kaiser score by two breast imaging radiologists and one radiology resident, individually and blinded to the pathological diagnosis and previous reading results, with the consensus about different opinions through consultation.
**Results or Findings:** Histopathology revealed 18 malignant and 51 benign lesions, resulting in a predominance of benignancy of 73.9%. The mean size of lesions on MRI was 15±10 mm; 50 lesions presented as masses (72.5%), 19 as a non-mass enhancement (27.5%), and there was no difference between malignant and benign lesions. The Kaiser score was as follows: 1–4 (BI-RADS 2/3) in 48 lesions (69.6%), 5–7 (BI-RADS 4) in 11 (15.9%), and 8–11 (BI-RADS 5) in ten (14.5%) (Figs. sensitivity and specificity were 100% and 94.1%, respectively, with three false-positive findings and no false-negative findings. The AUC was 0.971 (95% CI: 0.932–1.000).
**Conclusion:** Applying the Kaiser score to breast MRI, BI-RADS categories are reclassified (particularly BI-RADS 4 to BI-RADS 2/3), which can obviate unnecessary biopsies in breast lesions with equivocal conventional imaging findings.
**Limitations:** No limitations were identified.

**Ethics committee approval:** The study was approved by the Institute of Oncology and Radiology of Serbia: 1140-01
**Funding for this study:** No funding was received for this study.
**Author Disclosures:**
Vladimir Urban: Nothing to disclose
Katarina Obradovic: Nothing to disclose
Dragana Pavlovic Stankovic: Nothing to disclose
Zorica Cedomir Milosevic: Nothing to disclose
Ivana Petkovic: Nothing to disclose

### RPS 1802-8

**Breast multiparametric MRI (MP-MRI) to discriminate between pure ductal carcinoma in situ (DCIS) and microinvasive carcinoma (MIC): the importance of DWI**

*F. Cicciarelli*[1], V. Rizzo[2], F. Galati[3], M. Pasculli[3], R. Maroncelli[4], F. Pediconi[3], C. Catalano[3]; [1]Latina/IT, [2]Maglie/IT, [3]Rome/IT, [4]Guidonia/IT

**Purpose:** DCIS is a common subtype of breast cancer that is often upgraded to invasive cancer on the final pathology report of the surgical specimen. DCIS has an upgrade rate ranging in MIC from less than 10% to more than 40%. In these cases, histologic upgrade should be considered before surgical planning, to include sentinel lymph node biopsy in view of possible axillary lymph node metastasis. The aim of our study is to investigate whether breast multiparametric magnetic resonance imaging (MP-MRI) can distinguish between pure DCIS and MIC or invasive ductal cancer (IDC).
**Methods or Backround:** Between January 2018 and November 2022, patients with biopsy-proven DCIS who performed preoperative breast MP-MRI on 3T magnet were enrolled in this retrospective study. MP-MRI features (size, morphology, margins, extent of disease, presence of peritumoural oedema and curve I/T value, type and distribution of enhancement, restriction of diffusion and ADC value) were evaluated. For categorical variables chi-square test was assessed to investigate the association with the pathological outcome. Logistic regression analysis was performed to evaluate their prognostic value.
**Results or Findings:** Out of 129 patients with biopsy-proven DCIS, 36 presented foci of micro-infiltration on surgical specimens and eight was IDC. The presence of micro-infiltration foci was significantly associated with several MP-MRI features, such as tumour size ≥2 cm ($p=0.02$), clustered ring enhancement ($p<0.001$), and segmental distribution ($p<0.01$). Interestingly, a mean ADC value <1.3 x 10-3 mm2/s revealed to be a prognostic factor for the presence of micro-infiltration foci ($p< 0.05$).
**Conclusion:** Breast MP-MRI can predict the presence of micro-infiltration foci in biopsy-proven DCIS, and could be considered a valid tool for therapeutic planning in patients with DCIS.
**Limitations:** The relativity small number of patients was an identified limitation.
**Ethics committee approval:** No information provided by the submitter.
**Funding for this study:** No funding was received for this study.
**Author Disclosures:**
Roberto Maroncelli: Nothing to disclose
Veronica Rizzo: Nothing to disclose
Francesca Galati: Nothing to disclose
Marcella Pasculli: Nothing to disclose
Federica Cicciarelli: Nothing to disclose
Federica Pediconi: Nothing to disclose
Carlo Catalano: Nothing to disclose

### RPS 1802-9

**Screening without mammography using abbreviated breast MRI alone**

*S. Morscheid*, M. Bode, L. C. Huck, K. Strobel, C. K. Kuhl; Aachen/DE
*(smorscheid@ukaachen.de)*

**Purpose:** Breast MRI is recommended for supplemental screening for women with dense breasts. However, in women undergoing screening MRI, the additional cancer detection rate attributable to mammography has been shown to be limited. Based on recently published cost-effectiveness analyses, using MRI alone for screening would represent the most cost-effective screening method. The purpose of this study was to report first results on the outcome of women undergoing breast MRI as a stand-alone screening test (without mammography).
**Methods or Backround:** This is an ongoing study which has so far enrolled 1,085 women (aged 40-85 years, median 57 years) without personal history of breast cancer who underwent screening using abbreviated MRI alone. Breast MRI was performed on a 1.5T system with multichannel-coil, according to a standardised protocol. Validation of MRI diagnoses was achieved by follow-up (977/1,085) or biopsy (108/1,085). A further 54 biopsies were performed within the screening period.
**Results or Findings:** Women underwent a total 2,771 screening MRIs (mean 2.6 studies/participant), covering a total of 3,220 women-years and a subsequent follow-up period of 2,425 women-years. MRI was positive in 392/2,771 examinations (14.1%). Breast cancer was confirmed in 67, yielding a PPV3 (of biopsy) of 41.4%. The overall cancer detection rate was 20.8 per 1000 women-years. The median age of women diagnosed with cancer was 59 years. Cancers were DCIS in 18/67 and invasive cancers in 49/67. Of the invasive cancers, cancers were staged pT1a in 7/49, pT1b in 21/49, pT1c in 16/49, pT2 in 5/49, and as N0 in 42/49 (85.7%); N1 and N1mi in the remaining cases; all were staged M0. 60/67 patients with MRI-detected cancer underwent bilateral preoperative mammography. The MRI-known cancer was occult on mammography in 40/60 (67%).
**Conclusion:** Abbreviated breast MRI alone appears sufficient for breast cancer screening, ensuring early cancer detection.
**Limitations:** The pseudo-prospective study design was an identified limitation.
**Ethics committee approval:** This study was conducted in accordance with the Declaration of Helsinki and was approved by the Institutional Review Board of RWTH University Aachen.
**Funding for this study:** No external funding was received for this study.
**Author Disclosures:**
Stephanie Morscheid: Nothing to disclose
Kevin Strobel: Nothing to disclose
Christiane K. Kuhl: Nothing to disclose
Maike Bode: Nothing to disclose
Luisa Charlotte Huck: Nothing to disclose

### RPS 1802-10

**Looking beyond standard dosage in dynamic contrast enhanced breast MRI using a pre-trained deep-learning model**

*S. Pasumarthi Venkata*[1], P. Gulaka[1], G. Zaharchuk[2], R. Chamberlain[1]; [1]Menlo Park, CA/US, [2]Stanford, CA/US
*(srivathsa@subtlemedical.com)*

**Purpose:** Gadolinium-based contrast agents (GBCAs) are widely used in breast MRI. Increasing GBCA dosage improves lesion visualisation but safety concerns have been raised regarding long-term GBCA retention. In this work, we repurposed a dose reduction deep-learning (DL) model to synthesise contrast boosted (CB) breast MRI images that have better lesion visualisation without increasing dosage.
**Methods or Backround:** A DL model for dose reduction in brain MRI was previously trained to enhance 10% to 100% dose. This model was repurposed for boosting contrast in breast MRI by inputting pre-contrast and standard postcontrast (SC) sequences to synthesise contrast boosted (CB) sequences. Dynamic contrast enhanced (DCE) breast MRI data from 60 patients (30 internal, 30 Duke Breast MRI public dataset) were used to evaluate CB algorithm. CE images were normalised and co-registered to pre-contrast sequences, and the first time point post-injection was used as the postcontrast. Axial images were manually cropped to remove signals posterior to the pectoral muscles. Left and right sides were processed individually by the model. Lesion-to-background ratio (LBR) and contrast enhancement percentage (CEP) metrics were calculated on SC and CB images. ROIs were manually drawn for calculating quantitative metrics. Enhancement kinetic curves (time post-injection versus signal-intensity [SI]) were drawn with pre-contrast, SC and other CE time points along with the average SI of CB images.
**Results or Findings:** The mean LBR and CEP of CB (9.63±0.53 and 182.3%) were significantly better ($p<0.001$) than those of SC images (6.73 ± 0.34 and 93.4%). It was also observed that the DL model improves the lesion enhancement and visibility.
**Conclusion:** Quantitative and qualitative assessment has shown the feasibility of DL based contrast boosting in breast MRI to improve lesion visualisation.
**Limitations:** Detailed clinical evaluation required.
**Ethics committee approval:** No information provided by the submitter.
**Funding for this study:** No funding was received for this study.
**Author Disclosures:**
Srivathsa Pasumarthi Venkata: Employee: Subtle Medical Inc; Shareholder: Subtle Medical Inc
Greg Zaharchuk: Investigator: Stanford University; Founder: Subtle Medical Inc
Praveen Gulaka: Shareholder: Subtle Medical Inc; Employee: Subtle Medical Inc
Ryan Chamberlain: Shareholder: Subtle Medical Inc; Employee: Subtle Medical Inc

09:30-11:00 **Research Stage 3**

### Research Presentation Session: Oncologic Imaging

### RPS 1816

### Liver, biliary tract and pancreas

**Moderator**
C. Rio Bartulos; Braunschweig/DE
*(c.rio@skbs.de)*

### RPS 1816-2

**An image-based comparison of histological subtypes in intraductal papillary mucinous neoplasm of the pancreas**
*J. Ritter*, I. Pergolini, I. E. Demir, F. Lohöfer, R. Braren; Munich/DE

**Purpose:** Intraductal papillary mucinous neoplasms (IPMN) are precursor lesions of pancreatic ductal adenocarcinoma (PDAC). IPMN can be distinguished by histomorphological and immunohistochemical characteristics as intestinal, oncocytic, pancreatobiliary and gastric subtypes. The Fukuoka consensus guideline is a classification system for risk assessment in IPMN. The aim of the study was to compare these subtypes according to the Fukuoka criteria.
**Methods or Backround:** MRI scans of 104 patients undergoing pancreatic surgery for IPMN were re-examined for the Fukuoka criteria. Statistics on the distribution of Fukuoka criteria and the incidence of malignancy were performed.
**Results or Findings:** In IPMN with pancreatobiliary subtype (n=16), 75% had carcinoma compared to 44% with intestinal subtype (n=41) and only 25% with gastric subtype (n=47). Further analysis using the Fukuoka criteria compared all malignant versus benign IPMNs, regardless of the histological subtype. Here significantly more worrisome features (P-value 0.017) and high-risk stigmata (P-value 0.0077) were detected in the presence of malignancy. For the individual histological subtypes, no significant difference in the number of worrisome features and high-risk stigmata was found between the benign and malignant cases. A cross-subgroup comparison within the malignant IPMN showed that patients with PDAC based on pancreatobiliary or intestinal subtype had significantly more worrisome features than the gastric cohort (2.08/2.22 vs 1.25; P-value 0.040/0.026). Regarding the high-risk stigmata all three subtypes presented similarly.
**Conclusion:** According to our findings pancreatobiliary and intestinal subtype are more susceptible to malignant progression than gastric subtype. At the same time, our results show that the Fukuoka criteria are only of limited use for preoperative subtyping in IPMN.
**Limitations:** A limitation of this study is the relatively small number of patients of different subtypes. At least similarly sized patient groups could be formed in the case of malignancy.
**Ethics committee approval:** The study was approved by the ethics committee of the university hospital rechts der Isar, technical university of Munich (number 87/18S)
**Funding for this study:** No funding was received for this study.
**Author Disclosures:**
Jessica Ritter: Nothing to disclose
Rickmer Braren: Nothing to disclose
Ilaria Pergolini: Nothing to disclose
Ihsan Ekin Demir: Nothing to disclose
Fabian Lohöfer: Nothing to disclose

### RPS 1816-3

**Dual-layer detector spectral CT: a non-invasive preoperative tool for predicting histopathological differentiation in pancreatic ductal adenocarcinoma**
*W. Liu*, Z. Zhou, T. Xie, Y. Wang; Shanghai/CN
*(lw02017@163.com)*

**Purpose:** The study aimed to predict histopathological differentiation grades in patients with pancreatic ductal adenocarcinoma (PDAC) before surgery, with quantitative and qualitative variables obtained from dual-layer spectral detector CT (DLCT).
**Methods or Backround:** A total of 128 patients with histopathologically confirmed PDAC and preoperative DLCT were retrospectively enrolled and categorised into the low-grade (LG) (well and moderately differentiated, n=82) and high-grade (HG) (poorly differentiated, n=46) subgroups. The regions of interest were placed on tumours, and both conventional and spectral variables for PDAC were determined, including CT attenuation, contrast enhancement fraction (CEF), iodine concentration (IC), effective atomic number (Zeff), iodine enhancement fraction (IEF), and the slope of the spectral curve (K-slope). Necrosis was visually assessed on both conventional CT images (necrosis_con) and virtual mono-energetic images (VMIs) at 40 keV (necrosis_40keV). Receiver operating characteristic (ROC) curves were used to evaluate the efficiencies of variables in predicting tumour grade.
**Results or Findings:** Necrosis_con (odds ratio [OR]=2.84, 95% confidence interval [CI]: 1.13–7.13; p<0.001), necrosis_40keV (OR=5.82, 95% CI: 1.98–17.11; p=0.001) and IEF in the arterial (AP) and venous (VP) phases (OR=1.12, 95% CI:1.07–1.17; p<0.001) were significantly different between the LG and HG PDAC groups by univariable and multivariable analyses. IEF_AP/VP (AUC=0.754, p=0.016) and the combination model (AUC=0.812, p<0.001) had better predictive performances than necrosis_con (AUC=0.580). The combination model yielded the highest sensitivity (72%) and accuracy (79%), while IEF_AP/VP exhibited the highest specificity (89%).
**Conclusion:** Variables derived from DLCT have the potential to preoperatively evaluate PDAC tumour grade. Furthermore, spectral variables and their combination exhibited superior predictive performances than conventional CT variables.
**Limitations:** The small sample size, and at single institution, was an identified limitation.
**Ethics committee approval:** The study was approved by the Ethics Committee of Fudan University Shanghai Cancer Center.
**Funding for this study:** Funding was received from: Shanghai Science and Technology Innovation Action Plan, Hong Kong, Macao and Taiwan Science and Technology Cooperation Project (22490760800) and Shanghai Minhang District Medical Characteristic Specialty Development Project (2020MWFC05).
**Author Disclosures:**
Yu Wang: Nothing to disclose
Tiansong Xie: Nothing to disclose
Wei Liu: Nothing to disclose
Zhengrong Zhou: Nothing to disclose

### RPS 1816-4

**Preoperative scoring model for identifying proliferative hepatocellular carcinoma on multiphase liver MRI and its implication for surgical resection**
*J. Pan*, Y. Zhu, F. Chen; Hangzhou/CN

**Purpose:** The study aimed to develop and validate a scoring model for preoperative identification of proliferative hepatocellular carcinomas (HCCs) using multiphase liver MRI, and to compare the early recurrence rates between patients with model-predicted proliferative and nonproliferative HCCs after surgical resection.
**Methods or Backround:** Between September 2019 and August 2021, 476 consecutive patients with surgically proven HCC who underwent preoperative multiphase liver MRI were retrospectively enrolled. Logistic regression analyses were conducted to determine predictors associated with proliferative HCC. We established a scoring model based on the identified predictors (training cohort n=332) and verified it in a time-independent validation cohort (n=144). Early recurrence rates were evaluated by the Kaplan–Meier method with log-rank test.
**Results or Findings:** Serum alpha-fetoprotein level >100 ng/mL, irregular tumour margin, rim arterial phase hyperenhancement, marked diffusion restriction, and tumour-to-liver ratio at arterial phase ≤1.4 were independent predictors of proliferative HCCs. The optimal threshold of the scoring model for diagnosing proliferative HCCs was >18 points, with an area under the curve of 0.83 and 0.80 in the training and validation cohort, respectively. In both two cohorts, patients with model-predicted proliferative HCCs exhibited significantly higher early recurrence rates compared to those with model-predicted nonproliferative HCCs (all P<0.05). Moreover, patients with BCLC stage B-C and predicted nonproliferative HCCs exhibited similar early recurrence rates to those with BCLC stage 0-A and predicted proliferative HCCs in both cohorts (all P>0.05).
**Conclusion:** The developed scoring model, incorporating four MRI features and serum alpha-fetoprotein level, showed promising potential for predicting proliferative HCCs and identifying suitable surgical candidates for patients with HCC.
**Limitations:** This was a single-centre retrospective study with inherent selection bias.
**Ethics committee approval:** This study was approved by the Institutional Review Board of the First Affiliated Hospital, College of Medicine, Zhejiang University.
**Funding for this study:** This study received funding from the National Natural Science Foundation of China (12090020 and 12090025) and Zhejiang Provincial Natural Science Foundation Committee-Zhejiang Society for Mathematical Medicine Joint Fund Major Project (LSD19H180003).
**Author Disclosures:**
Junhan Pan: Nothing to disclose
Yanyan Zhu: Nothing to disclose
Feng Chen: Nothing to disclose

### RPS 1816-5

**Intraindividual comparison of CT and MRI for predicting vessels encapsulating tumour clusters in hepatocellular carcinoma: which imaging modality is better?**

*J. Pan*, Y. Zhu, F. Chen; Hangzhou/CN

**Purpose:** The study aimed to establish and validate scoring models for identifying vessels encapsulating tumour clusters (VETC) pattern in hepatocellular carcinoma (HCC) using CT and MRI, and to compare the predictive performance across the modalities.
**Methods or Backround:** Between June 2019 and August 2020, 324 consecutive patients with surgically confirmed HCC who underwent preoperative multiphase CT and MRI were retrospectively enrolled. Logistic regression analyses were conducted to determine predictors associated with the VETC positive HCC. We established two separate scoring models based on the identified predictors (training cohort n=227) and verified it in an independent validation cohort (n=97). Generalised estimating equations were used to compare the diagnostic performance of the two models.
**Results or Findings:** Tumour size >5.0 cm, intra-tumoural artery, three or four types of enhancement pattern, tumour-to-liver ratio at arterial phase >1.57, and serum AFP > 400 ng/ml were identified as independent predictors for VETC positive HCC on CT. Conversely, independent predictors on MRI included incomplete "capsule", necrosis or serve ischaemia, marked diffusion restriction, tumour-to-liver ratio at ADC map ≤1.03, and serum AFP >400 ng/mL. The CT-based and MRI-based scoring models were constructed using these predictors, with cut-off values of >17 points and >21 points, respectively. Compared to the MRI-based model, CT-based model exhibited lower sensitivity but higher specificity. However, the CT-based and MRI models demonstrated similar AUC values in both training (0.83 versus 0.85, P=0.468) and validation cohorts (0.81 versus 0.80, P=0.966).
**Conclusion:** Preoperative CT and MRI may show comparable predictive performances for identifying the VETC pattern in HCC. The application of CT or MRI should be determined by the preferred trade-off between sensitivity and specificity in clinical practice.
**Limitations:** This was a single-centre retrospective study with inherent selection bias.
**Ethics committee approval:** This study was approved by the Institutional Review Board of the First Affiliated Hospital, College of Medicine, Zhejiang University.
**Funding for this study:** This study received funding from the National Natural Science Foundation of China (12090020 and 12090025) and Zhejiang Provincial Natural Science Foundation Committee-Zhejiang Society for Mathematical Medicine Joint Fund Major Project (LSD19H180003).
**Author Disclosures:**
Junhan Pan: Nothing to disclose
Yanyan Zhu: Nothing to disclose
Feng Chen: Nothing to disclose

### RPS 1816-6

**CT evaluation of hepatic steatosis as a predictive factor for the development of surgical complications in patients with pancreatic adenocarcinoma resected after neoadjuvant chemotherapy**

*E. Boffa*, G. Zamboni, M. Antolini, A. Spezia, G. Malleo, G. Mansueto; Verona/IT
*(elisaboffa@yahoo.it)*

**Purpose:** The study aimed to assess the impact of neoadjuvant chemotherapy (NAT) on the onset of moderate-to-severe liver steatosis and postoperative complications in patients with pancreatic adenocarcinoma (PDAC).
**Methods or Backround:** This retrospective study included patients with PDAC who received NAT, underwent CT before and after NAT and underwent major pancreatic resections. All patients were included in a prospectively maintained registry; 71 patients were also enrolled in an IRB-approved prospective study and underwent intraoperative liver biopsy. Two readers in consensus drew multiple ROIs on liver and spleen parenchyma on the venous phase: the difference between the two mean densities was calculated and used to quantify steatosis. Imaging assessment of steatosis was compared with biopsy results in 71 patients. The population was divided into two groups based on the steatosis degree (group 1: <30%; group 2: >30%). Post-surgical complications and Clavien-Dindo Index (CDI) were compared between groups applying the chi-squared test.
**Results or Findings:** A total of 234 patients were included (113 males, 121 females; mean age 62 years). Liver steatosis at CT was significantly correlated with biopsy results (p=0.0002). After NAT, 82 patients (35%) developed steatosis or worsened their degree of steatosis. 109 patients (47%) developed post-surgical complications (POPF, liver failure, bile leak, PPAP). Moderate-severe steatosis before NAT was significantly correlated with a CDI ≥3 (p=0.0453) and with 90-days postoperative mortality (p=0.0262). A positive trend of correlation was observed between moderate-severe steatosis after NAT and CDI ≥3 and PPAP.
**Conclusion:** In patients affected by PDAC undergoing neoadjuvant treatment, liver steatosis evaluated on routine CT is associated with a higher risk of developing complications after major pancreatic resections.
**Limitations:** No limitations were identified.
**Ethics committee approval:** Informed consent was provided by all patients (PAD-R registry, n1101CESC). For this retrospective study, IRB approval was not required. IRB approval was obtained for the prospective LIMBO study.
**Funding for this study:** No funding was received for this study.
**Author Disclosures:**
Alessandro Spezia: Nothing to disclose
Giulia Zamboni: Nothing to disclose
Giancarlo Mansueto: Nothing to disclose
Elisa Boffa: Nothing to disclose
Monica Antolini: Nothing to disclose
Giuseppe Malleo: Nothing to disclose

### RPS 1816-7

**Apparent diffusion coefficient and tissue stiffness are associated with different tumour microenvironment features in hepatocellular carcinoma**

*J. Chen*, T. Chen, Z. Zhang, W. Zhenru, B. Song; Chengdu/CN

**Purpose:** The study aimed to investigate associations between tissue diffusion, stiffness, and cell density, nuclear volume fraction, tumour-stroma ratio (TSR), tumour stemness, and CD8+ T cell infiltration in resected hepatocellular carcinoma (HCC).
**Methods or Backround:** We included 72 HCC patients with preoperative magnetic resonance (MR) diffusion-weighted imaging and MR elastography examination. Free hands region of interests was placed on the central four slices of the apparent diffusion coefficient (ADC) map and the stiffness map, and the mean value of the tumour and peri-tumour parenchyma was estimated. Cell density, nuclear volume fraction, TSR, CK19 expression, and CD8+ T cell infiltration was estimated in the whole slide histopathological image of the resected tumour sample. Spearman's rank correlation coefficients and intraclass correlation coefficients were calculated.
**Results or Findings:** Inter-reader agreement was excellent regarding ADC and stiffness measurement. Tumour ADC correlated with cell density (r=-0.39, p=0.001) and nuclear volume fraction (r=-0.39, p=0.001). Tumour stiffness (TS) correlated with nuclear volume fraction (r=-0.28, p=0.02) and TSR (r=-0.33, p=0.005). Peri-tumour ADC correlated with CK19 expression (r=0.40, p=0.001) in HCC. Peri-tumour stiffness correlated with CD8+ T cell infiltration (r=0.27, p=0.02) in HCC.
**Conclusion:** In HCC, tumour ADC reflects tumour cellularity and nuclear volume fraction, tumour stiffness reflects nuclear volume fraction and TSR. Peri-tumour ADC is associated with CK19 expression in tumour, and the peri-tumour stiffness is associated with CD8+ T cell infiltration in tumour.
**Limitations:** The limitations of the study are its retrospective nature and small sample size.
**Ethics committee approval:** The study was approved by the Ethics Committee on Biomedical Research, West China Hospital of Sichuan University
**Funding for this study:** Funding was provided by the Science and Technology Support Program of Sichuan Province (grant number: 2022YFS0072) and the 1.3.5 Project for Disciplines of Excellence, West China Hospital, Sichuan University (grant number: ZYJC21012).
**Author Disclosures:**
Wu Zhenru: Nothing to disclose
Zhen Zhang: Nothing to disclose
Jie Chen: Nothing to disclose
Bin Song: Nothing to disclose
Tingyu Chen: Nothing to disclose

### RPS 1816-8

**Pancreatic cancer in photon-counting CT: low keV virtual monoenergetic images improve tumour conspicuity**

*M. M. Wöltjen*, J. R. Kroeger; Minden/DE

**Purpose:** The aim of the study was to identify differences in the tumour conspicuity of pancreatic adenocarcinomas in different monoenergetic or polyenergetic (T3D) series and contrast phases in photon-counting CT (PCCT).
**Methods or Backround:** A total of 34 patients were retrospectively enrolled in this study. Quantitative image analysis was performed with ROI measurements in different monoenergetic levels ranging from 40 up to 70 keV (5-point steps) and polyenergetic series. Tumour-parenchyma attenuation differences and CNR were calculated. A qualitative image analysis was accomplished by four radiologists. Differences between groups were evaluated for statistical significance using the Friedman test and in case of significant differences pair-wise post-hoc testing with Bonferroni correction was applied.

**Results or Findings:** Tumour-parenchyma attenuation difference was significantly different between the different image reconstructions for both arterial- and portal-venous-phase-images (p<0.001). Tumour-parenchyma attenuation difference was significantly higher on arterial-phase images at mono 40 keV compared to T3D images (p<0.001) and mono 55 keV images or higher (p<0.001). For portal-venous-phase images tumour-parenchyma attenuation difference was significantly higher on mono 40 keV images compared to T3D images (p<0.001) and mono 50 keV images (p=0.03) or higher (p<0.001). The same trend was seen for CNR. Tumour conspicuity was rated best on mono 40 keV images with 4.3±0.9 for arterial-phase images and 4.3±1.1 for portal-venous-phase images. In contrast, overall image quality was rated best on T3D images with 4.8±0.5 for arterial-phase images and 4.7±0.6 for portal-venous-phase images.
**Conclusion:** Low keV virtual monoenergetic images significantly improve the tumour conspicuity of pancreatic adenocarcinomas in PCCT based on quantitative and qualitative results. On the other hand, readers prefer polyenergetic images for overall image quality.
**Limitations:** Limitations were the monocentric study design and the limited number of patients.
**Ethics committee approval:** The study was conducted according to the guidelines of the Declaration of Helsinki and approved by the institutional review board (AZ: 2021-805).
**Funding for this study:** No funding was received for this study.
**Author Disclosures:**
Jan Robert Kroeger: Nothing to disclose
Matthias Michael Wöltjen: Nothing to disclose

### RPS 1816-9

**Is computed tomography-based body composition a reliable predictor of chemotherapy-related toxicity in pancreatic cancer patients?**
*V. Chianca*[1], M. Cefalì[2], C. Reiner[3], S. Stocker[3], M. Kissling[4], F. Del Grande[1], S. De Dosso[2], S. Rizzo[1]; [1]Lugano/CH, [2]Bellinzona/CH, [3]Zurich/CH, [4]Basle/CH
*(vitochianca@gmail.com)*

**Purpose:** Malnutrition, loss of weight and of skeletal muscle mass are frequent in pancreatic cancer patients, a majority of whom will undergo chemotherapy over the course of their disease. Available data suggest a negative prognostic role of these changes in body composition on disease outcomes; however, it is unclear whether tolerance to chemotherapeutic treatment is similarly and/or negatively affected. We aimed to explore this association by retrospectively assessing changes in body composition and chemotherapy-related toxicity in a cohort of advanced pancreatic cancer patients.
**Methods or Backround:** 131 patients (mean age 69.7 ± 9.0 years, 45% women and 55% men), most of whom (81.5%) had metastatic disease at diagnosis, were enrolled in this study. Body composition was evaluated through clinical parameters and through radiological assessment of muscle mass, skeletal muscle area, skeletal muscle index and skeletal muscle density; an assessment of fat distribution by subcutaneous adipose tissue and visceral adipose tissue was performed. We performed descriptive statistics, pre/post chemotherapy comparisons and univariate and multivariate analyses to assess the relation between changes in body composition and toxicity.
**Results or Findings:** Toxicity risk increased with an increase of skeletal muscle index (OR: 1.03) and body mass index (OR: 1.07), whereas it decreased with an increase in skeletal muscle density (OR: 0.96). Multivariate analyses confirmed a reduction in the risk of toxicity only with an increase in skeletal muscle density (OR: 0.96).
**Conclusion:** This study suggests that the retrospective analysis of changes in body composition is unlikely to be useful in predicting toxicity to gemcitabine-nab-paclitaxel.
**Limitations:** The small sample size was an identified limitation.
**Ethics committee approval:** The Ethics Committee approved this retrospective study with a waiver for informed consent (2020-01085).
**Funding for this study:** No funding was received for this study.
**Author Disclosures:**
Marc Kissling: Nothing to disclose
Vito Chianca: Nothing to disclose
Stefania Rizzo: Nothing to disclose
Filippo Del Grande: Nothing to disclose
Sara De Dosso: Nothing to disclose
Soleen Stocker: Nothing to disclose
Marco Cefalì: Nothing to disclose
Cäcilia Reiner: Nothing to disclose

### RPS 1816-10

**Intrahepatic mass-forming cholangiocarcinoma: is there additional prognostic value in using Gd-EOB enhanced MRI?**
*S. Halskov*, F. Krenzien, L. K. Segger, D. Geisel, B. Hamm, J. Ihlow, W. Schöning, T. A. Auer, U. Fehrenbach; Berlin/DE

**Purpose:** The study aimed to investigate the prognostic value of enhancement patterns of intrahepatic mass-forming cholangiocarcinomas (IMCCs) during the hepatobiliary phase (HBP) in gadoxetic acid (Gd-EOB) enhanced MRI.
**Methods or Backround:** We retrospectively identified 66 consecutive patients with histopathologically proven IMCCs (reference standard: resection) and preoperative Gd-EOB-enhanced MRI. Gd-EOB retention area was subjectively rated based on areas of intermediate signal intensity. Lesions were classified as either hypointense (0-25% retention area) or significantly-retaining (>25% retention area). Clinical, radiological and prognostic features were compared between these groups. The primary endpoint was recurrence-free-survival (RFS) after primary surgical resection.
**Results or Findings:** In total, 73% (48/66) of lesions were rated as hypointense and 29% (19/66) as significantly-retaining. While the hypointense subgroup more frequently featured local and distant intrahepatic metastases (p=0.039 and p=0.022) and an infiltrative growth pattern (p=0.005), RFS and clinical features did not differ significantly with Gd-EOB retention area or quantitatively measured HBP enhancement ratios. Lymph node metastasis was an independent predictor of poor RFS (p=0.001).
**Conclusion:** Gd-EOB-enhanced MRI reveals two subtypes of IMCC in the HBP: hypointense and signal-retaining. The hypointense subtype is associated with more frequent intrahepatic metastases and an infiltrative growth pattern, indicating potential tumour aggressiveness. However, this did not result in a significant difference in survival after primary resection of IMCC.
**Limitations:** This was a retrospective study of a surgical cohort, which could lead to selection bias: the prognostic value of imaging features in unresectable HCC remains unclear. Histopathological analysis to identify small-duct and large-duct IMCCs could have been of added value for identifying prognostic imaging subtypes. Quantitative analysis of HBP enhancement evaluated the whole tumour without taking intra-lesion heterogeneity in enhancement into account.
**Ethics committee approval:** Our institutional review board approved this retrospective study (internal registration number: EA2/016/14) and waived informed consent due to the retrospective nature. The study protocol conforms to the ethical guidelines of the 2002 Declaration of Helsinki.
**Funding for this study:** No funding was provided for this study.
**Author Disclosures:**
Laura Katharina Segger: Nothing to disclose
Bernd Hamm: Other: Bayer Schering Pharma, Berlin, Germany
Felix Krenzien: Grant Recipient: Berlin Institute of Health
Wenzel Schöning: Nothing to disclose
Uli Fehrenbach: Other: Bayer Schering Pharma, Berlin, Germany
Dominik Geisel: Other: Bayer Schering Pharma, Berlin, Germany
Timo Alexander Auer: Grant Recipient: Berlin Institute of Health; Other: Bayer Schering Pharma, Berlin, Germany
Sebastian Halskov: Nothing to disclose
Jana Ihlow: Grant Recipient: Berlin Institute of Health

### RPS 1816-11

**Efficacy and evaluation of response in hepatocellular carcinoma (HCC) treated with stereotactic body radiotherapy (SBRT)**
*F. Castagnoli*[1], A. Villanacci[2], M. Bertuletti[2], B. Frittoli[2], L. Grazioli[2]; [1]London/UK, [2]Brescia/IT

**Purpose:** The study aimed to evaluate changes in ADC values, size, wash-in, wash-out, T2 signal intensity, necrosis and capsule of HCCs treated with SBRT.
**Methods or Backround:** A total of 62 patients with HCC (65 lesions), treated with SBRT, who underwent baseline gadoxetate-enhanced MRI within four weeks before treatment and follow-up MRI within three months after SBRT were prospectively enrolled; 44 patients underwent a further MRI follow-up 12 months after SBRT. Three radiologists evaluated tumour size ($\partial$), ADC, wash-in, wash-out, capsule, necrosis and T2 signal of all lesions. Local control (LC) was evaluated after a median follow-up (FU) of 2.1 years. Delta($\Delta$)$\partial$ and ADC were correlated using Spearman correlation coefficient. $\Delta\partial$ and $\Delta$ADC were compared (Mann-Whitney test) between patients with and without LC. A logistic regression model was trained to estimate the probability of response of patients using categorical imaging variables.
**Results or Findings:** The median HCC ADC ($10^{-3}mm^2/s$) and dimension were 0.94 and 22.5 mm at baseline; 1.326 and 16.4 mm at 3-month follow-up; 1.352 and 10.6 mm at 12-month follow-up. At 12-months, the ADC values increased by 41.1% (p<0.001), whereas tumour size decreased by 52.7% (p<0.001). At 3-month follow-up there was no correlation between the increase in ADC values and decrease in lesion dimension (p=0.41). At 12-month follow-up, there was correlation between the increase in ADC values and decrease in lesion dimension (p=0.04). At 3- and 12-month follow-up, there was significant difference in $\Delta(\partial)$ and presence of wash-out between patients with and without LC (p=0.05, p=0.03; p=0.04, p=0.02). No significant correlation were found for $\Delta$(ADC), wash-in, T2w signal, necrosis and capsule between patients with and without LC.
**Conclusion:** SBRT showed excellent LC for HCC, which was associated with the degree of size change after treatment and absence of wash-out at follow-up.
**Limitations:** Identified limitations were the small sample size and the limited cases of local recurrence (n=7).

**Ethics committee approval:** The current study was carried out according to the Declaration of Helsinki (1964) and was approved by the Ethics Committee of the ASST Spedali Civili, Brescia.
**Funding for this study:** No funding was received for this study.
**Author Disclosures:**
Martina Bertuletti: Nothing to disclose
Francesca Castagnoli: Nothing to disclose
Luigi Grazioli: Nothing to disclose
Barbara Frittoli: Nothing to disclose
Alberta Villanacci: Nothing to disclose

**09:30-11:00** **Research Stage 4**

### Research Presentation Session: Head and Neck

### RPS 1808

### Thyroid and salivary gland imaging

**Moderator**
E. Vassallo; Msida/MT
*(edithvassallo@gmail.com)*

### RPS 1808-2

**Role of superb microvascular imaging for differentiation between benign and malignant neck lymph nodes**
*H. R. Cho*, M. Sunwoo, Y. Nam; Changwon-si, Gyeongsangnam-do/KR
*(gpffh1452@naver.com)*

**Purpose:** Ultrasound examination reveals different types of disease that cause lumps in the cervical lymph nodes. However, different types of disease can produce similar ultrasound findings, and despite the criteria that have been proposed to distinguish between them, greyscale ultrasound is still limited. Therefore, methods are being used to analyse vascular patterns using colour or power Doppler ultrasound. However, benign and malignant vascular patterns still overlap, and the use of superficial microvascular imaging techniques that detect microvasculature may help distinguish them.
**Methods or Backround:** From January to August 2023, 163 patients who received FNAB or biopsy for enlarged neck lymph nodes were included. All patients underwent US examinations of LNs consisting of greyscale US, PDUS and SMI. After sonography, each lymph node was evaluated histologically by ultrasound-guided FNAB or biopsy.
**Results or Findings:** Out of 163 patients, 18 patients were excluded due to non-diagnostic results. Of the 145 total lymph nodes, 49 were identified as malignant. SMI evaluation of nodal vascular pattern was of high sensitivity (69.4%), specificity (90.6%), and accuracy (83.4%) for differentiating metastatic and benign nodes, however SMI alone does not have a significant advantage in differentiating metastatic nodules compared to greyscale or PDUS. There were 38 indeterminate nodes on the greyscale Doppler image, of which five were histologically confirmed to be malignant. Of 38 indeterminate nodes, 23 were reclassified as benign by SMI, all of which were confirmed as benign.
**Conclusion:** Using a nodal classification based on a combination of sonographic features and SMI is effective in detecting and diagnosing metastatic lymphadenopathy, leading to improved treatment and prognosis for patients.
**Limitations:** Identified limitation were: (1) this was a short-term prospective study with a small number of cases; (2) inter-observer agreement could not be assessed due to on-site pattern analysis.
**Ethics committee approval:** This study was approved by an ethics committee.
**Funding for this study:** No funding was received for this study.
**Author Disclosures:**
Yoojin Nam: Nothing to disclose
Miok Sunwoo: Nothing to disclose
Hye Ree Cho: Nothing to disclose

### RPS 1808-3

**Blood leukocyte DNA methylation biomarker for distinguishing malignant from benign thyroid nodules**
*F. Wang*, D. Zhao, L. Liu; Shanghai/CN

**Purpose:** Discriminating between malignant thyroid nodules (MTNs) and benign thyroid nodules (BTNs) presents challenges in clinical practice. Our objective was to develop a blood leukocyte-based classifier to distinguish MTNs from BTNs, and to assess its potential for enhancing diagnostic performance.
**Methods or Backround:** This study included 91 MTNs and 114 BTNs. Differential methylation haplotype blocks (MHBs) in blood leukocytes between MTNs and BTNs were detected using reduced representation bisulfite sequencing (RRBS). Subsequently, a blood leukocyte DNA methylation (BLDM) classifier was developed.
**Results or Findings:** A 60-marker BLDM classifier achieved an area under the curve (AUC) of 0.86 in the validation cohort. The specificity of it was 90.91%, outperforming the 43.64% specificity of ultrasonography, albeit with a slightly lower sensitivity compared to ultrasonography (83.33% versus 97.62%). The BLDM classifier correctly identified 93.55% of patients whose nodules were suspected to be malignant by ultrasonography, but which were finally histologically benign. In micronodules, a higher specificity of 93.33% and accuracy of 88.24% for diagnosis of MTNs was displayed by this classifier, in comparison to the 6.67% specificity and 72.55% accuracy of ultrasonography.
**Conclusion:** We identified a novel BLDM classifier for distinguishing MTNs from BTNs. The clinical application of this classifier leads to enhanced diagnostic specificity compared to ultrasonography.
**Limitations:** The main limitation of this study is that all participants were recruited from a single centre, and this classifier will require validation in larger external cohorts in the future.
**Ethics committee approval:** This study was approved by the Institutional Review Committee of Zhongshan Hospital, Fudan University (B2022-390R).
**Funding for this study:** Funding was provided by the National Health Commission Capacity Building And Continuing Education Center (GWJJ2022100303) and Fudan University Integrated Medical Engineering Program (yg2022-6).
**Author Disclosures:**
Feihang Wang: Nothing to disclose
Lingxiao Liu: Nothing to disclose
Danyang Zhao: Nothing to disclose

### RPS 1808-4

**CEUS as an additional tool in differentiating hyperthyroid diffuse disease: destructive vs hyperproduction conditions**
*A. Borlea*, D. I. Stoian; Timisoara/RO
*(andreea.brl3@gmail.com)*

**Purpose:** This study aims to evaluate the efficacy and added value of contrast-enhanced ultrasound (CEUS) in differentiating between destructive thyroiditis and hyperproduction states in patients presenting with diffuse thyroid disease.
**Methods or Backround:** Hyperthyroid diffuse diseases present as multifaceted pathological entities, often categorised into destructive thyroiditis and hyperproduction-induced hyperthyroidism. A prospective cohort of 56 patients diagnosed with hyperthyroid diffuse disease underwent CEUS, alongside conventional ultrasound and other standard diagnostic investigations. Qualitative CEUS parameters, including perfusion patterns, enhancement intensity, and washout timing, were analysed and compared between the destructive thyroiditis group and the hyperproduction group.
**Results or Findings:** Preliminary results indicated distinctive CEUS perfusion patterns between destructive thyroiditis and hyperproduction-induced hyperthyroidism. Destructive thyroiditis demonstrated rapid enhancement (12 seconds, 10-14.5) followed by a swift washout (20 seconds, 18-26), reflective of increased vascular permeability and inflammation. In contrast, hyperproduction states typically exhibited prolonged enhancement, suggestive of increased vascularity due to important thyroid activity. CEUS exhibited very good diagnostic accuracy (AUC 0.905, sensitive 91%, specificity 88.3%), with an appreciable sensitivity and specificity when integrated with existing diagnostic paradigms. Furthermore, CEUS findings correlated with biochemical markers, strengthening its diagnostic usefulness.
**Conclusion:** By presenting distinct perfusion patterns and enhancement characteristics, CEUS enables a more precise imaging-based understanding of the underlying pathophysiological processes, thereby facilitating informed diagnostic and therapeutic decision-making.
**Limitations:** One primary limitation of this study resides in the relatively small sample size of 56 patients, which potentially constrains the generalisability of the findings. While the preliminary results provide insightful correlations and trends within the examined parameters, a more expansive, multicentric study with a larger patient cohort is crucial to validate and consolidate these findings
**Ethics committee approval:** The study was approved by the UMF Victor Babes Timisoara Ethics Committee.
**Funding for this study:** The study received no external funding.
**Author Disclosures:**
Dana I. Stoian: Nothing to disclose
Andreea Borlea: Nothing to disclose

**RPS 1808-5**

**Radiomics analysis of thyroid ultrasound in relation to the radioactive iodine therapy-related sialadenitis**

*H. Kim*, D. H. Lee; Suwon/KR

**Purpose:** Radioactive iodine therapy (RAIT) is a widely employed therapy after total thyroidectomy but is often associated with the development of RAIT-sialadenitis. This study aims to investigate whether preoperative thyroid ultrasound can identify the occurrence of sialadenitis in patients who have undergone total thyroidectomy followed by RAIT.
**Methods or Backround:** This retrospective single-centre study was conducted at Ajou University Hospital, a tertiary referral centre in Suwon, Korea. The study included patients who underwent total thyroidectomy and subsequent RAIT between January 2010 and October 2022. Regions of interest were drawn as 2D square regions, each with a minimum size of 100×100 point, encompassing only non-cancerous thyroid parenchyma. Radiomics features were extracted using a commercial program (AVIEW Research, Coreline Soft, Seoul, Korea). Radiomics features were selected through univariable logistic regression (p-value<.05). The performance of the diagnostic model in distinguishing the occurrence of RAIT-sialadenitis was evaluated by the area under the curve (AUC) of the receiver operating characteristic curve.
**Results or Findings:** The study included 62 normal patients and 20 patients with RAIT-sialadenitis. RAIT-sialadenitis was predicted by the excess kurtosis (p-value=.003) from the first order and histogram feature, as well as contrast (p-value=.043) from the neighbouring grey-tone difference matrix (NGTDM). The combination of the excessive kurtosis and contrast of NGTDM demonstrated good diagnostic ability to differentiate the occurrence of RAIT-sialadenitis (AUC 0.75, 95% confidence interval: 0.64–0.87).
**Conclusion:** Radiomics features from thyroid ultrasound, excess kurtosis, and contrast of NGTDM, may help the prediction of RAIT-sialadenitis and enhance patient care before RAIT.
**Limitations:** A relatively small number of patients were included. In addition, there was data heterogeneity derived from the ultrasound scanner and practitioners. Furthermore, there was no pathologic confirmation of the salivary gland.
**Ethics committee approval:** This retrospective study was approved by the Institutional Review Board of Ajou University Hospital (approval number: AJOUIRB-MDB-2022-226). The requirement for written informed consent was waived by the Clinical Research Review Committee of Ajou University Hospital. All procedures performed in this study complied with both the US Health Insurance Portability and Accountability Act regulations and the Declaration of Helsinki.
**Funding for this study:** This study was supported by the Ajou University (M-2023-C0460-00050).
**Author Disclosures:**
Da Hyun Lee: Nothing to disclose
Hyein Kim: Nothing to disclose

**RPS 1808-6**

**Arterial spin labelling MR technique in assessing blood perfusion of thyroid nodules and differentiating the malignancy by comparing with CT enhancement**

*Y. Xu*; Hangzhou/CN

**Purpose:** To investigate the feasibility of arterial spin labelling (ASL) MR technique to evaluate thyroid and nodule blood perfusion by comparing with enhanced thyroid CT examination, and to differentiate between benign and malignant nodules. .
**Methods or Backround:** A retrospective analysis was conducted on data of patients with thyroid nodules who underwent routine MRI and ASL examinations with thyroid CT enhancement from August 2022 to August 2023 in our hospital. Two radiologists independently rated the ASL image quality, measured the TBF (thyroidal blood flow) of thyroid and nodules, as well as the CT values of thyroid nodules in plain, arterial, and venous phases. The consistency of image quality between the two radiologists was analysed using Kappa analysis. The consistency of TBF and CT values measured by the two radiologists for thyroid and nodules was analysed using ICC analysis. The correlation between CT values and thyroid blood flow (TBF) in the thyroid and nodules was evaluated using Pearson correlation.
**Results or Findings:** A total of 70 patients with thyroid nodules underwent thyroid CT enhancement and ASL examination. A total of 11 cases were excluded. Finally, 59 patients with a total of 64 thyroid nodules were included. The consistency of image quality scores between the two radiologists was good (Kappa=0.745, P<0.001). The consistency of TBF measurements was ICC=0.780, 0.856, respectively (P<0.001). The correlation between TBF of thyroid and arterial CT enhancement value was mild (r=0.283, p=0.023), while the correlation between TBF of thyroid nodules and CT enhancement value was significant (r=0.754, P<0.01). TBF of thyroid nodule between benign and malignant was significant (p=0.034).
**Conclusion:** ASL can non-invasively quantitatively evaluate the blood perfusion of the thyroid and nodules with good repeatability, and can help differentiate between benign and malignant nodules.
**Limitations:** The small sample size was an identified limitation of this study.
**Ethics committee approval:** This study was conducted in accordance with the requirements of the institutional medical ethics committee (Hangzhou Medical College, Zhejiang Provincial People's Hospital Research Ethics Committee, document number: 202101291419000446756).
**Funding for this study:** This study was supported by the Zhejiang Medicine and Health Project of Science and Technology (2022KY581, 2022KY525).
**Author Disclosures:**
Yuyun Xu: Nothing to disclose

**RPS 1808-7**

**Risk stratification of incidental 18F-Fluorodeoxyglucose-avid thyroid nodules based on six societies' ultrasound risk stratification systems**

*C. Y. Shin*[1], D. G. Na[1], H. S. Lee[1], H. S. Ahn[2]; [1]Gangneung-si/KR, [2]Seoul/KR

**Purpose:** This study was aimed to determine the risk of malignancy in thyroid incidentalomas (TIs) detected on 18F-FDG PET/CT using the six societies' ultrasound (US)-risk stratification systems (RSSs) and to evaluate the diagnostic value of the SUVmax in PET-TIs.
**Methods or Backround:** This study included 215 consecutive patients with 236 TIs detected by focal thyroid uptake on 18F-FDG PET/CT. The final diagnoses were obtained by US-guided biopsy or thyroidectomy. The malignancy risk of all TIs were assessed according to the six US-RSSs including ACR-, Chinese (C)-, European (EU)-, and Korean-TIRADS.
**Results or Findings:** The overall malignancy rate of TIs was 47% and the malignancy risk of TIs was stratified by all US-RSSs (all, P<0.001). The observed malignancy risks of high-suspicion TIs were higher than the estimated malignancy risks only in ACR-, C-, and EU-TIRADSs (P≤0.035). The malignancy risks of intermediate-suspicion TIs were higher than the estimated malignancy risks in all US-RSSs (24.8-48.2%, P≤0.002). The malignancy risks of low-suspicion TIs were higher than the estimated malignancy risk only in the C- and EU-TIRADS (35% and 10%, respectively, P≤0.01). The SUVmax of malignant tumours was higher than that of benign nodules (4.7 versus 2.8, P<0.001) and the AUC for malignancy was 0.708. The SUVmax of high-grade malignancy was higher than that of low-grade malignancy (7.4 versus 4.0, P=0.002).
**Conclusion:** The malignancy risk of intermediate-suspicion TIs was increased in all US-RSSs, whereas the malignancy risks of high- and low-suspicion TIs was increased in only some RSSs. The biopsy size thresholds for intermediate suspicion TIs should be lowered in all US-RSSs. The SUVmax showed a fair diagnostic performance for malignancy in PET-TIs and the SUVmax needs to be considered in biopsies.
**Limitations:** No limitation were identified.
**Ethics committee approval:** This study is under the review of the ethics committee (number: 2023-09-007).
**Funding for this study:** No funding was received for this study.
**Author Disclosures:**
Dong Gyu Na: Nothing to disclose
Hyo Sang Lee: Nothing to disclose
Chae Young Shin: Nothing to disclose
Hye Shin Ahn: Nothing to disclose

**RPS 1808-8**

**Quantitative evaluation of thyroid gland vascularisation with microvascular ultrasonography and contribution of vascular index for differential diagnosis of Graves' disease and subacute thyroiditis**

*Z. S. Erdal*, M. N. Cerit, H. N. Şendur, S. Özhan Oktar, M. Yalçın, Ş. E. Doğan, M. Coşkun; Ankara/TR

**Purpose:** Microvascular ultrasonography (MVUS) is a new Doppler technique that provides higher sensitivity in differentiating slow flow from motion artifacts. The vascular index obtained by MVUS measures the blood flow rate in the tissue by determining the ratio of coloured pixels (blood flow) to all pixels in the ROI (region of interest) and gives a quantitative real time evaluation. The aim of the present study is to test the ability of the MVUS technique in differentiating cases with Graves' disease and subacute thyroiditis.
**Methods or Backround:** 86 cases (28 Graves' disease, 28 subacute thyroiditis, and 30 control group) were selected. Age, gender, BMI, fT3, fT4, TSH, TRAb, ESR, and CRP values were recorded. During the thyroid gland ultrasonography, vascular indexes were measured in the transverse and longitudinal axes in both lobes after measuring the thyroid gland dimensions. The correlations between mean vascular index value, age, BMI, and laboratory results were assessed. ROC (Receiver operating characteristic) curve analysis was performed to investigate the diagnostic performance of the vascular index in differentiating Graves' disease from subacute thyroiditis.
**Results or Findings:** In conclusion, mean thyroid gland volume and vascular index values were significantly higher in both disease groups than in the control group. The mean vascular index value was higher in the group with Graves' disease than in subacute thyroiditis. Remarkably, 88% sensitivity and 92% specificity were achieved with an optimum cut-off value of 26.31% for vascular index in differentiating Graves' disease from subacute thyroiditis.

**Conclusion:** Our results highlight the potential benefit of microvascular ultrasonography in the differential diagnosis of Graves' disease and subacute thyroiditis.
**Limitations:** The limitation of the study is the limited number of cases. In addition, we didn't compare the MVUS with other Doppler techniques.
**Ethics committee approval:** The study was approved by the Gazi University School of Medicine ethics committee (Reference number 782, 24.10.2022).
**Funding for this study:** No funding was provided for this study.
**Author Disclosures:**
Suna Özhan Oktar: Nothing to disclose
Meriç Coşkun: Nothing to disclose
Mahi Nur Cerit: Nothing to disclose
Şerife Ezgi Doğan: Nothing to disclose
Zeynep Sezgi Erdal: Nothing to disclose
Halit Nahit Şendur: Nothing to disclose
Muhittin Yalçın: Nothing to disclose

### RPS 1808-9

**Possibility to predict BRAFV600E mutations of papillary thyroid carcinoma via nomogram based on dual-layer detector spectral CT and clinical characteristics**
*D. Zhang*; Chongqing/CN

**Purpose:** The aim of this study was to investigate the effectiveness of a nomogram based on dual-layer detector spectral computed tomography (DLCT) for predicting BRAFV600E mutation in PTC.
**Methods or Backround:** The BRAFV600E is the most common mutant oncogene in thyroid cancer and is closely related to the aggressiveness of papillary thyroid carcinoma (PTC). The medical records of 253 patients with PTCs who underwent DLCT and BRAFV600E mutation detection (mutant group: n=203; wild group: n=50) were reviewed. DLCT quantitative parameters of arterial phase, typical radiological features and clinical information were compared by univariate and multivariate analysis between the mutant and wild-type BRAFV600E groups. A nomogram was developed based on the significantly different variables using multivariate logistic regression analysis. The nomogram performance was evaluated by the area under the receiver operating characteristic curve (AUC), calibration curve and decision curve analysis (DCA).
**Results or Findings:** The normalised iodine concentration (NIC), calcification and Hashimoto's thyroiditis (HT) were identified as independent risk factors of BRAFV600E mutation in PTC. The AUC value of the nomogram based on the three parameters was 0.75. The calibration curve of the nomogram revealed that the prediction result was in good agreement with actual observation. The decision curve demonstrated that the nomogram can provide a more satisfactory net benefit than the simple all or none-intervention standard strategy within a large range of threshold probabilities.
**Conclusion:** The DLCT-based nomogram with NIC, calcification, and HT has good effectiveness for predicting the BRAFV600E mutation in PTC. Keywords: dual-layer detector spectral CT, papillary thyroid carcinoma, BRAFV600E mutation, nomogram
**Limitations:** The two samples were somewhat imbalanced.
**Ethics committee approval:** The study is retrospective.
**Funding for this study:** No funding was received for this work.
**Author Disclosures:**
Dan Zhang: Nothing to disclose

### RPS 1808-10

**Magnetic Resonance (MR) sialographic assessment of masseter muscle and ductal kinking in patients with recurrent parotitis**
M. C. Firetto, *S. Triggiani*, G. Conte, L. Pignataro, G. M. M. Nicolino, G. Carrafiello; Milan/IT
*(sonia.triggiani@unimi.it)*

**Purpose:** The aim of this study was to assess the anatomical relationship between Stensen's duct and masseter muscle and analyse their implications in etiopathogenesis of recurrent parotitis secondary to masseter muscle dysfunction.
**Methods or Backround:** We enrolled 50 patients with suspect of masseter dysfunction due to bruxism: 41 unilateral recurrent parotitis, 9 bilateral parotitis. 33 patients made up the control group. All underwent power doppler ultrasonography, dynamic MR sialography, sialendoscopy. Basal and dynamic images of salivary glands were acquired at US by a small-parts transducer. MRI were acquired by 1.5T with head bobina. The before lemon sequences were STIR coronal and trasversal; T1 tse tra; T2 spc tra; T2 haste tra fs; T2 spc tra rst iso. 3-5 cc of lemon are administered through the mouth. Post lemon sequences were T2 haste tra fs; T2 spc tra; T2 spc rst tra. The scan lasts 40 minutes. At sialendoscopies, Stensen's duct ostium was dilated, then angled sialendoscope was inserted; exams end when all viable branches have been explored, pervious, without mucous plugs.
**Results or Findings:** Ultrasonography did not reveal Stensen's duct dilatation or obstruction. MRI sialography showed no statistically significant difference in imaging metrics. A subgroup analysis by gender, showed both parotids of male subjects with sialadenitis had a longer duct length compared to control subjects. In unilateral sialadenitis, comparison showed that parotids with sialadenitis had a wider duct. Sialendoscopy revealed acute duct angle in middle third of main duct; some mucous plugs were detected. Complete concordance between evidence of acute duct angle during sialendoscopy and wider duct in patients with parotitis was observed although not statistically significant.
**Conclusion:** This is one of the first studies in the literature analysing anatomical features relevant for the etiopathogenesis of recurrent parotitis secondary to the masseter muscle; we aim to increase the cohort population.
**Limitations:** There was a small number of patients.
**Ethics committee approval:** No information provided by the submitter.
**Funding for this study:** No funding was received for this study.
**Author Disclosures:**
Sonia Triggiani: Nothing to disclose
Maria Cristina Firetto: Nothing to disclose
Lorenzo Pignataro: Nothing to disclose
Giorgio Conte: Nothing to disclose
Gabriele Maria Maria Nicolino: Nothing to disclose
Gianpaolo Carrafiello: Nothing to disclose

### RPS 1808-11

**The role of magnetic resonance elastography in the evaluation of benign parotid tumours**
*V. Atamaniuk*, L. Hanczyk, M. Obrzut, I. Palasz-Krasowska, W. Domka, M. Cholewa, B. Obrzut; Rzeszow/PL
*(vitaliyacera500@gmail.com)*

**Purpose:** Magnetic resonance elastography (MRE) is the method of choice for the non-invasive evaluation of the biomechanical properties of numerous organs. MRE can be regarded as an advanced version of palpation, where the tissue is evaluated both qualitatively and quantitatively. We hypothesise that MRE can be a valuable tool for evaluating parotid tumours, which are often first diagnosed as palpable masses inside the parotid gland. This study aims to determine the feasibility of the parotid tumour MRE and assess the added value of tissue stiffness in tumour diagnosis and treatment. We believe that this new method can potentially enable us to circumvent the need for aspiration biopsy before surgery.
**Methods or Backround:** Seven patients with benign parotid tumours (two men and five women), aged between 45 and 70, participated in this study. A 1.5 T whole-body MRI scanner and a motion-sensitised 2D GRE MRE sequence were utilised for data acquisition. Shear waves in the parotid gland were induced using a custom passive driver fixed against the patient's face. Excitation frequencies of 40, 60, 70, 80, and 100 Hz were tested. Mean tumour stiffness was calculated using manually drawn masks delineating tumour areas.
**Results or Findings:** Among the tested frequencies, 60 Hz vibrations provided optimal shear wave illumination in the tumour area, allowing for the assessment of tumour stiffness in all patients. The estimated mean ± SD stiffness of parotid tumours was 1.44 ± 0.43 kPa. Tumour stiffness exhibited high heterogeneity both between and within patients.
**Conclusion:** This study demonstrates the feasibility of MRE for parotid tumours using a specific passive driver and appropriate excitation frequency. However, reliable reference data from healthy parotid glands are required for results comparison.
**Limitations:** The primary limitation of this study is its small sample size.
**Ethics committee approval:** This study received approval from the ethics committee of the Regional Medical Chamber (Resolution No 60/2022/B) and each volunteer provided written informed consent before participating in this study.
**Funding for this study:** No external funding was obtained for this study.
**Author Disclosures:**
Izabela Palasz-Krasowska: Nothing to disclose
Marzanna Obrzut: Nothing to disclose
Marian Cholewa: Nothing to disclose
Lukasz Hanczyk: Nothing to disclose
Wojciech Domka: Nothing to disclose
Vitaliy Atamaniuk: Nothing to disclose
Bogdan Obrzut: Nothing to disclose

**RPS 1808-12**

**Ultrasound-assisted identification of the cricothyroid ligament: use in head and neck ultrasound education**

*A. Weimer*[1], J. Weimer[2], L. Müller[2], C. Chrissostomou[3], R. Klöckner[4], M. Rink[3], J. Künzel[3]; [1]Heidelberg/DE, [2]Mainz/DE, [3]Regensburg/DE, [4]Lübeck/DE
*(andiweimer@gmx.net)*

**Purpose:** When other attempts to secure the airway are unsuccessful, a cricothyrotomy may be indicated. Sonographic guidance can support the identification of the cricothyroid ligament. The aim of this prospective study was to establish a structured training as part of head and neck ultrasound courses.
**Methods or Backround:** The training consisted of a 10-min lecture and a worksheet with an additional 10-min video tutorial followed by hands-on (45 min in groups of 4). To measure previous skills and satisfaction with the training as well as subjective and objective level of competence, evaluations were completed before (T1) and after (T2) the training. Furthermore, a practical test using a pocket device at T2 was performed. The answers of the evaluations were recorded using a Likert scale.
**Results or Findings:** Most participants had neither seen a cricothyrotomy (64.6%) nor had they performed it (79.6%) and they had no previous experience in using pocket devices (76.1%). Regarding the subjective competence, a significant improvement (T1 to T2) was measured in all competency areas (P <0.001), with the "Sonographic identification of Lig. conicum" (Δ=3.1 ± 1.6 scale points) reaching a particularly high increase. In the practical test, 89.2% of the possible points were achieved and an average of 101 seconds was needed for identification. Participants who already performed a cricothyrotomy, a tracheostomy or at least 30 head and neck sonographies tended to achieve higher scores and required less time.
**Conclusion:** Integration of a training concept for sonographic identification of the cricothyroid ligament was well accepted. Significant improvement in subjective competence as well as a high objective level of competence was achieved. Therefore, such training concepts should be standardised in head and neck ultrasound training.
**Limitations:** No evaluation of objective competence level at T1 was conducted; there was no control group without training.
**Ethics committee approval:** This study was approved by the ethics committee of the University of Regensburg.
**Funding for this study:** No funding was received for this study.
**Author Disclosures:**
Julian Künzel: Speaker: GE Healthcare Investigator: GE Healthcare
Roman Klöckner: Nothing to disclose
Christopher Chrissostomou: Nothing to disclose
Maximilian Rink: Nothing to disclose
Johannes Weimer: Nothing to disclose
Andreas Weimer: Nothing to disclose
Lukas Müller: Nothing to disclose

**12:15-13:45** **Research Stage 3**

### Research Presentation Session: Head and Neck

### RPS 1908

### Head and neck: advances in tumour imaging

**Moderator**
B. Ozgen Mocan; Chicago, IL/US
*(burcem@gmail.com)*

**RPS 1908-2**

**The prediction of biological features using MR imaging in head and neck squamous cell carcinoma: a systematic review and meta-analysis**

*H. J. van der Hulst*, R. W. Jansen, C. Vens, P. Bos, R. Martens, Z. Bodalal, R. G. H. Beets-Tan, M. van den Brekel, J. A. Castelijns; Amsterdam/NL

**Purpose:** Magnetic resonance imaging (MRI) is an indispensable, routine technique, providing morphological and functional imaging sequences. MRI can potentially capture tumour biology, allowing for longitudinal evaluations of such biology. This systematic review and meta-analysis evaluates the ability of MRI to predict tumour biology in primary head and neck squamous cell carcinoma (HNSCC).
**Methods or Backround:** Studies were screened, selected, and assessed for quality using appropriate tools according to the PRISMA criteria. Fifty-eight articles were analysed, examining the relationship between (functional) MRI parameters and biological features and genetics.
**Results or Findings:** Most studies focused on associations between individual MRI features and HPV status, revealing a consistent pattern wherein HPV-positive tumours exhibit significantly lower ADCmean (SMD: 0.82; p<0.001) and ADCminimum (SMD: 0.56; p<0.001) values. Lower ADCmean values are also associated with high Ki-67 levels, consistent with a diffusion restriction caused by high cellularity. Several perfusion parameters of the vascular compartment were found to be significantly associated with HIF-1α. Analysis of other biological factors (vascular endothelial growth factor [VEGF], epidermal growth factor receptor [EGFR], tumour cell count, p53, and micro vessel density [MVD]) yielded inconclusive results. This review showed that larger datasets with homogenous acquisition are required to develop and test radiomic-based prediction models capable of capturing different aspects of the underlying tumour biology.
**Conclusion:** Overall, this meta-analysis demonstrates that rapid and non-invasive characterisation of tumour biology features by MR-imaging is feasible and could enhance clinical outcome predictions and personalised patient management for HNSCC.
**Limitations:** While comprehensive, the review of MRI parameter associations with biological features is limited by the large variety of methods used in the included articles for calculating biological and MRI parameters.
**Ethics committee approval:** This was a review article.
**Funding for this study:** No funding was received for this study.
**Author Disclosures:**
Jonas A. Castelijns: Nothing to disclose
Zuhir Bodalal: Nothing to disclose
Conchita Vens: Nothing to disclose
Robin W. Jansen: Nothing to disclose
Roland Martens: Nothing to disclose
Regina G. H. Beets-Tan: Nothing to disclose
Hedda Joanne van der Hulst: Nothing to disclose
Michiel van den Brekel: Nothing to disclose
Paula Bos: Nothing to disclose

**RPS 1908-3**

**Comparison of diffusion-weighted MRI using imaging split acquisition of fast spin echo signal (SPLICE) and single-shot echo-planar imaging (SS-EPI) in tumours of the head and neck**

*H. J. van der Hulst*, R. Martens, B. Westerink, L. Braun, L. C. ter Beek, R. Tissier, R. G. H. Beets-Tan, M. van den Brekel, J. A. Castelijns; Amsterdam/NL

**Purpose:** Traditionally, diffusion-weighted imaging (DWI) uses single-shot echo planar imaging (SS-EPI) for its fast read-out but struggles with distortion, predominantly near bone-air interfaces. Turbo Spin Echo (TSE) DWI, like split acquisition of fast spin echo signal for diffusion imaging (SPLICE), may reduce this issue, especially in the head and neck area. This study compares DW-SPLICE with DW-SS-EPI for head and neck lesions (HN-lesions).
**Methods or Backround:** Between August 2020 and January 2022, 57 potential MRI HN-lesion exams were conducted using both SS-EPI and SPLICE techniques on a 3.0T MRI system. Lesions >0.5cm³ were delineated on both DWI-MRIs. The study assessed differences in whole lesion ADC-values through interclass correlation (ICC), Bland-Altman plots, and mixed-effect model regression. Two blinded radiologists evaluated image quality and distortion levels using a standardised questionnaire.
**Results or Findings:** Preliminary findings:
The eligible 41 MRI exams of 41 patients included 40 benign and 20 malignant HN-lesions, visible on both DWI techniques. Strong interclass correlation coefficients were found for mean ADC values (ICC agreement 0.864, 95% CI 0.782-0.917), consistency 0.862 (95% CI 0.780-0.915). The Bland-Altman regression showed discrepancies between SPLICE and EPI for higher average ADC values (ß=0.16, p=0.02), but not when assessing benign and malignant lesions separately. Radiologist evaluations of visual distortion showed a trend towards less distortion and better image quality for SPLICE-DWI.
**Conclusion:** High agreement and consistency in mean ADC values were seen for HN-lesions measured with both techniques. Qualitative analyses of radiologists resulted in a trend towards better distortion and image quality scores for SPLICE-DWI. While a potential ADC value discrepancy was observed in the Bland-Altman analyses, its clinical impact may be minimal if DWI-SPLICE can effectively replace SS-EPI.
**Limitations:** Identified limitations were: (1) the cohort consists of a mixed group of HN-lesions; (2) data is currently still preliminary.
**Ethics committee approval:** The study was approved by the IRBd22-270, registered at 28.10.2022.
**Funding for this study:** No funding was received for this study.

**Author Disclosures:**
Renaud Tissier: Nothing to disclose
Bram Westerink: Nothing to disclose
Jonas A. Castelijns: Nothing to disclose
Roland Martens: Nothing to disclose
Regina G. H. Beets-Tan: Nothing to disclose
Loes Braun: Nothing to disclose
Hedda Joanne van der Hulst: Nothing to disclose
Michiel van den Brekel: Nothing to disclose
Leon C. ter Beek: Nothing to disclose

### RPS 1908-4

**Does radiotherapy influence the diagnostic performance of ultrasound-guided fine-needle aspiration cytology of neck nodes in head and neck squamous cell carcinoma?**

*S. Neveu*, A. Escriva, M. Becker; Geneva/CH
*(sophie.neveu@hcuge.ch)*

**Purpose:** The study aims to evaluate and compare the diagnostic performance of ultrasound-guided fine-needle aspiration cytology (US-FNAC) in neck nodes of head and neck squamous cell carcinoma (HNSCC) patients with and without previous radiotherapy (RT+ versus RT-).
**Methods or Backround:** Retrospective study comprising a series of 99 consecutive HNSCC patients (66 RT- and 33 RT+, 25 females, 74 males, mean age = 67±12 years). A total of 136 nodal aspirations (92 RT- nodes, 44 RT+ nodes) were analysed. US-FNAC results were compared with the standard of reference (neck dissection and follow-up).
**Results or Findings:** The minimum/maximum axial diameter of biopsied RT- versus the RT+ nodes was similar (10.97±7.99 mm/15.9±10.9 mm versus 9.07± 5.9 mm/13.6±6.7 mm), t(134)=1.42/1.266, p<0.05. Likewise, nodal level distribution and US imaging characteristics of benign and malignant nodes were similar in both the groups (p>0.05). For diagnostic US-FNAC probes, the sensitivity, specificity, accuracy, positive and negative predictive values (PPV and NPV) of the US-FNAC in the RT- versus the RT+ group were 91.3%, 95.5%, 93.3%, 95.5%, 91.3% versus 94.7%, 88.9%, 91.8%, 89.5%, 94.4%, respectively (p>0.05). However, the proportion of non-diagnostic cytology results was significantly higher (7/44 = 15.9%) in the RT+ versus the RT- group (2/92 = 2.2%), p=0.005.
**Conclusion:** Although, irradiation significantly increases the proportion of non-diagnostic samples, it has no influence on the diagnostic performance of US-FNAC in neck nodes of HNSCC patients.
**Limitations:** Limitations of the current study include its retrospective design and a potential selection bias as only patients with US-FNAC were included in the current study.
**Ethics committee approval:** This retrospective study was approved by the local Ethics Committee and was done in accordance to the Helsinki declaration (CER 11-092R). Informed consent was waived.
**Funding for this study:** Funding was received from the institution.
**Author Disclosures:**
Minerva Becker: Nothing to disclose
Sophie Neveu: Nothing to disclose
Ananda Escriva: Nothing to disclose

### RPS 1908-5

**Detection of recurrent nodal disease in head and neck squamous cell carcinoma with multiparametric contrast-enhanced FDG-PET/DWIMRI**

*S. Neveu*[1], A. Varoquaux[2], O. Rager[1], C. De Vito[1], N. Dulguerov[1], C. Combescure[1], H. Zaidi[1], M. Becker[1]; [1]Geneva/CH, [2]Marseille/FR
*(sophie.neveu@hcuge.ch)*

**Purpose:** The purpose of this study was to determine the diagnostic performance of contrast-enhanced FDG-PET/DWIMRI to detect recurrent nodal disease in head and neck squamous cell carcinoma (HNSCC) treated with radio(chemo)therapy (RCT).
**Methods or Backround:** This prospective study included 47 patients treated with RCT ± surgery for advanced HNSCC who underwent 67 contrast-enhanced FDG-PET/DWIMRI examinations as part of their surveillance protocol. Lymph nodes (LNs) were evaluated prospectively using a 5-point Likert scale by experienced readers. Lymph node size, ADCmean/ADCmin and SUVmean/SUVmax were measured for all detected LNs. The standard of reference was histology of neck dissection specimens in 34% and follow-up ≥ 5years in 66% of patients. The diagnostic performance was assessed both in terms of sensitivity, specificity and accuracy and using Receiver Operating Analysis (ROC) in 67 examinations, 134 neck sides, 938 LN levels and 220 LNs.
**Results or Findings:** The prevalence of LN metastases depended on the type of analysis performed (50% per neck side, 5% per LN level and 44% per LN). Mean minimum axial diameter was 6.5±4.0 mm in benign and 7.0±5.7mm in malignant LNs (p>0.05) and only 11% of all nodes had a diameter >10 mm. ADC mean/ADC min values were 1.14±0.31|0.62±0.29x10-3 mm2/s and 1.17±0.29|0.78±0.29x10-3 mm2/s in benign and malignant LNs, respectively (p>0.05) while SUV mean/SUV max values were 1.87±0.84|2.38±1.15 in benign and 3.40±2.38|4.79±3.45 in malignant LNs (p<0.05). FDG-PET/DWI-MRI had a sensitivity/specificity/accuracy of 90%/64%/72% per examination, 88%/82%/83% per neck side, 74.5%/97%/96% per LN level, and 49.5%/76.4%/64.5% per LN. The areas under the curve (AUC) were 0.926 per examination, 0.933 per neck side, 0.874 per LN group and 0.661 per LN.
**Conclusion:** Analysis type heavily influences results. As most recurrent metastatic LNs are <1 cm in size, the sensitivity of contrast-enhanced FDG-PET/DWIMRI is limited in the per level and per LN analysis.
**Limitations:** The limitation was that it was a single study center.
**Ethics committee approval:** This prospective clinical study was approved by the Swissethics committee (Project ID 2017-00748) and performed in accordance with the guidelines of the Helsinki II declaration. Written informed consent was obtained from all subjects.
**Funding for this study:** Funding was received from the Swiss National Science Foundation (SNSF) (grant number: N0 320030_173091/1).
**Author Disclosures:**
Christophe Combescure: Nothing to disclose
Minerva Becker: Nothing to disclose
Claudio De Vito: Nothing to disclose
Habib Zaidi: Nothing to disclose
Olivier Rager: Nothing to disclose
Nicolas Dulguerov: Nothing to disclose
Arthur Varoquaux: Nothing to disclose
Sophie Neveu: Nothing to disclose

### RPS 1908-6

**Predicting response to exclusive combined radio-chemotherapy in naso-oropharyngeal cancer: the role of texture analysis**

*E. Bicci*, L. Calamandrei, M. Pietragalla, S. Busoni, S. Paolucci, F. Mungai, C. Nardi, L. Bonasera, V. Miele; Florence/IT

**Purpose:** The aim of the study was to identifying MRI texture features able to predict response to radiochemotherapy (RT-CHT) in patients with naso-oropharyngeal carcinoma before treatment to help clinical decision making.
**Methods or Backround:** 41 patients with naso-oropharyngeal carcinoma (NPC-OPC) were enrolled in the study and were divided into 2 groups (responders/non-responders) according to MRI, 18F-FDG-PET/CT performed 3-4 and 12 months after RT-CHT, and biopsy. Pre-RT-CHT lesions were segmented and radiomic features were extracted from ADC-maps and post-gadolinium T1 MRI images acquired on a single MRI scanner. A radiomic model differentiating responders from non-responders was built using the LASSO algorithm on all the features extracted, which allows at the same time to make feature reduction and selection. Receiver Operating Characteristic (ROC) curves and Area Under the Curve (AUC) values were generated a 95% confidence interval (CI) was reported.
**Results or Findings:** LASSO model selected five statistically significant features: Maximum 2D Diameter Slice, Dependence Non-uniformity, Gray Level Non-Uniformity, Small Area high gray level emphasis from post-gadolinium T1 MRI images and 10 Percentile from ADC maps AUC associated with the ROC was: 0.978 with 95% CI: 0.937-1.
**Conclusion:** Texture analysis on post-gadolinium T1-images and ADC-maps could potentially predict response to therapy in patients with NPC-OPC who will undergo exclusive treatment with RT-CHT, being, therefore, a useful tool in clinical decision-making.
**Limitations:** The limitations of this study were that (1)The use of an MRI-unit only is an advantage to make the sample as homogeneous as possible, but currently a disadvantage for the lack of generalizability of results. (2) The relative low sample size. Nevertheless, most studies on texture analysis of OPC and NPC are based on Computed Tomoghrapy (CT) and not MRI examinations.(3) The small number of patients with residual cancer for the well-known excellent response to RT-CHT of OPC (especially HPV+) and NPC.
**Ethics committee approval:** The study was approved by the Ethics Committee of our Institution (study protocol n: 21800)
**Funding for this study:** No funding was received for the study.
**Author Disclosures:**
Michele Pietragalla: Nothing to disclose
Vittorio Miele: Nothing to disclose
Cosimo Nardi: Nothing to disclose
Leonardo Calamandrei: Nothing to disclose
Eleonora Bicci: Nothing to disclose
Sebastiano Paolucci: Nothing to disclose
Luigi Bonasera: Nothing to disclose
Simone Busoni: Nothing to disclose
Francesco Mungai: Nothing to disclose

### RPS 1908-7

**Early intra-treatment T1 rho imaging predicts treatment response to induction chemotherapy in patients with advanced nasopharyngeal carcinoma**

*Q. Y. AI*, Y. M. Tsang, Z. Yu, B. B. Ma, E. P. Hui, W. Chen, A. D. King; Hong Kong/HK
*(aqy0621@gmail.com)*

**Purpose:** The aim of the study was early prediction of treatment response to induction chemotherapy (IC) in patients with advanced nasopharyngeal carcinoma (NPC) that could help clinicians to adjust treatment plans. This study evaluates whether quantitative T1 rho imaging, a pulse sequence that is newly applied to research in cancer, can predict treatment response to IC at early intra-treatment stage in patients with advanced NPC.
**Methods or Backround:** Twenty-five eligible patients who had advanced NPC planned for treatment with 3-cycle of IC + concurrent chemoradiation therapy were prospectively recruited. T1rho MRI scans of the primary tumour were performed pre-treatment and early intra-treatment (2-week after IC started) and the T1rho mean value was obtained. Treatment response to IC was evaluated at the third cycle of IC based on the RECIST 1.1. T1rho value of pre-treatment (T1rhopre) and percentage change in T1rho between pre- and intra-treatment scans (%Δ) =[(T1rhointra-T1rhopre)/T1rhopre *100%] were compared between responders (complete response and partial response) and non-responders (stable disease and progression disease) using Mann-Whitney U test. Median (interquartile) values were reported.
**Results or Findings:** Comparing responders (n= 15) and non-responders ( n=10), responders showed higher pre-treatment T1 rhopre [66.6 (66.2, 74.3) x msec vs. 63.8 (61.7, 69.5) x msec, p=0.03] and greater reduction in T1 rho [ %Δ decrease of -9.0% (-12.1%, 3.9%) vs. 3.2% (-4.9%, 6.6%), (p=0.03)].
**Conclusion:** Pre-treatment and early intra-treatment change in the T1 rho mean values show potential for predicting treatment response to IC in patients with advanced NPC.
**Limitations:** Preliminary results were reported. Further evaluation is needed.
**Ethics committee approval:** This study was approved by the Joint Chinese University of Hong Kong and New Territories East Cluster Clinical Research Ethics Committee.
**Funding for this study:** This study was funded by Research Grant Committee of The Hong Kong S.A.R. Government (Ref. 14100419)
**Author Disclosures:**
Brigette B.Y. Ma: Nothing to disclose
Ann Dorothy King: Nothing to disclose
Edwin P. Hui: Nothing to disclose
Weitian Chen: Nothing to disclose
Ziqiang Yu: Nothing to disclose
Qi Yong AI: Nothing to disclose
Yip Man Tsang: Nothing to disclose

### RPS 1908-8

**Radiologic predictors of response to neoadjuvant chemotherapy in patients with advanced oral cavity tumours**

*I. Burck*, A. Gleich, E. Herrmann, J-E. Scholtz, J. Gruen, P. Thönissen, M. Fleischmann, R. Winkelmann, D. Pinto Dos Santos; Frankfurt a. Main/DE

**Purpose:** The aim of this study was to explore whether radiological imaging features could predict response to neoadjuvant radiochemotherapy in patients with locally advanced oral-cavity tumour.
**Methods or Backround:** We included 28 Patients (14 women, median age 59+-9.5) with stage IVa oral cavity carcinoma who underwent neoadjuvant radiochemotherapy at our institution between February 2012 and August 2022. All patients received MRI scans before treatment, after 15 days of treatment and preoperatively. MRI images were judged qualitatively based on a Lickert scale with respect to their overall intensity and change in intensity and tumour extent between the scans. Additionally, scans were analysed quantitatively by measuring the absolute signal intensity (SI) of the tumour in ADC, DWI and T2 and normalising the values to the signal intensity of the spinal cord. Differences and quotients of normalised ADC-, DWI- and T2-SIs were calculated. Patients with pathological T1 or T0 stage were classified as responders, while all others were classified as non-responders.
**Results or Findings:** We found significant differences between responders and non-responders for normalized ADC-SI at both follow-up timepoints (p = 0.014 and p = 0.024, respectively), as well as for the quotient between normalized DWI-SI at baseline and preoperatively (p = 0.011) and the difference between normalized ADC-SI at baseline and preoperatively (p = 0.035).
**Conclusion:** Diffusion weighted imaging parameters (normalized ACD-SI and normalized DWI-SI) could predict response to neoadjuvant radiochemotherapy in jaw carcinoma and could potentially be used to guide treatment or extent of surgery in these patients.
**Limitations:** Limitations of this study include small sample size, single-centre and retrospective study design.
**Ethics committee approval:** This study was approved by the Institutional Review Board (approvals number 208/12 )
**Funding for this study:** No funding was obtained for this study.
**Author Disclosures:**
Ria Winkelmann: Nothing to disclose
Jan-Erik Scholtz: Nothing to disclose
Daniel Pinto Dos Santos: Nothing to disclose
Philipp Thönissen: Nothing to disclose
Alexander Gleich: Nothing to disclose
Eva Herrmann: Nothing to disclose
Jens Gruen: Nothing to disclose
Maximilian Fleischmann: Nothing to disclose
Iris Burck: Nothing to disclose

### RPS 1908-9

**Image-guided intraoperative assessment of surgical margins in oral cavity squamous cell cancer: a diagnostic test accuracy review**

*G. Carnicelli*, S. Lusi, G. M. Frigerio, L. Disconzi, G. Mercante, L. Balzarini, M. Francone, C. Giannitto; Milan/IT
*(carnicelligiorgia@gmail.com)*

**Purpose:** The objective of this study was to assess the imaging guidance of oral cavity squamous cell cancer. Incomplete primary resection of oral cavity squamous cell cancer has a dramatic impact on prognosis and re-treatment. Imaging guidance may improve the rate of negative margin resection (currently around 45%). A diagnostic test accuracy review was designed to assess the performance of intraoperative ultrasound and magnetic resonance imaging (MRI) in determining free margin status.
**Methods or Backround:** Two independent reviewers systematically screened articles from EMBASE, Cochrane and MEDLINE databases. The study protocol was designed according to standards of the Cochrane handbook for diagnostic test accuracy (DTA) reviews. Among inclusion criteria were MRI and ultrasound-based studies (2016-2023), a free-margin resection cut-off of 5 mm, frequency of US probes ≥ 15 MHz. Negative predictive value (NPV), sensitivity and specificity of intraoperative imaging were calculated for selected studies. The software used for the review process was Review Manager 5.4 .
**Results or Findings:** Ten articles were included in the final analysis, four used for data extraction (Figure 1). Overall NPV of both techniques for margins < 5 mm ranged from 0.50 to 0.91, with higher diagnostic yield of ultrasound compared to MRI. Sensitivity of imaging ranged from 0.07 to 0.75; specificity ranged from 0.81 to 1.00 (Figure 2). Overall, the mean rate of free margin resection was 55.8% ± 28 . In all studies with control group, imaging guidance always provided a benefit, with increase in free-margin resection of 35% ± 13; the accuracy decreased for stages ≥ T1-2 and with increasing depth of invasion (DOI) values .
**Conclusion:** NPV of intraoperative ultrasound in guiding oral cavity cancer resection remains suboptimal with 40-50% margins being missed, and varies with DOI and T stage. Nevertheless, it provides a mean 35% improvement in free margin resection rate.
**Limitations:** Heterogeneity of the included studies limits the scope of this study.
**Ethics committee approval:** There was no ethical committee approval for reasons related to the nature of the study.
**Funding for this study:** No funding was obtained for this study.
**Author Disclosures:**
Luca Disconzi: Nothing to disclose
Caterina Giannitto: Nothing to disclose
Giorgia Carnicelli: Nothing to disclose
Giuseppe Mercante: Nothing to disclose
Marco Francone: Nothing to disclose
Luca Balzarini: Nothing to disclose
Stefano Lusi: Nothing to disclose
Gian Marco Frigerio: Nothing to disclose

### RPS 1908-10

**Reproducibility and prognostic value of NI-RADS score in early post-operative CT for oral cavity cancer**

M. Panfili, *G. Mazzon*, S. Longo, S. Settimi, F. Pastore, D. A. Mele, M. Massaccesi, R. Calandrelli, S. Gaudino; Rome/IT

**Purpose:** This retrospective study aims to assess the inter-observer concordance of the NI-RADS score applied both for primary site and regional lymph-node levels in early post-operative CT scans in patients with surgically treated oral cavity cancer and its correlation with outcomes. The Neck Imaging reporting and Data System (NI-RADS) is a valuable tool for post-treatment imaging in head and neck cancers with good capability to predict disease recurrence. Yet, it remains underexplored its specific utility and prognostic value in early post-operative computed tomography (CT) scans in surgically treated patients with oral cavity cancer (OC) and eligible for adjuvant therapy.

**Methods or Backround:** Two experienced radiologists independently reviewed CT scans performed less than 90 days from surgery and assigned NI-RADS scores both for T site and N site. NI-RADS scores ranged from 1 to 3. The Cohen's kappa was used to test the interobserver agreement and the Kaplan-Meier survival analysis was used to evaluate correlation between NI-RADS scores and patients overall survival.
**Results or Findings:** Forty-two CT scans were reviewed with a median post-surgery time of 40 days. The Cohen's kappa statistic indicated substantial inter-observer agreement for the primary tumour (kappa = 0.91, 95% CI 0.79-1.00) and regional lymph nodes (kappa = 0.92, 95% CI 0.77-1.00). Patients with a NI-RADS score of 3 at the primary tumour level had a shorter median OS of 7 months compared to those with NI-RADS scores of 1, 2a, and 2b, who had a median OS of 68 months. This difference was statistically significant with a p-value of 0.006.
**Conclusion:** The study demonstrates that NI-RADS score in early post-operative CT has good reproducibility and could have prognostic implications.
**Limitations:** Retrospective nature and small number of patients.
**Ethics committee approval:** No information provided by the submitter.
**Funding for this study:** No funding was obtained for the study.
**Author Disclosures:**
Francesco Pastore: Nothing to disclose
Simona Gaudino: Nothing to disclose
Mariangela Massaccesi: Nothing to disclose
Rosalinda Calandrelli: Nothing to disclose
Marco Panfili: Nothing to disclose
Dario Antonio Mele: Nothing to disclose
Giovanni Mazzon: Nothing to disclose
Stefano Settimi: Nothing to disclose
Silvia Longo: Nothing to disclose

### RPS 1908-11

**Diagnostic accuracy of MRI in detecting perineural spread of head and neck tumours: a systematic review and meta-analysis**

*U. B. Abdullaeva*[1], B. Pape[2], J. Hirvonen[3]; [1]Tashkent/UZ, [2]Vaasa/FI, [3]Tampere/FI
*(umidasamira2@gmail.com)*

**Purpose:** The aim of this study was to review the diagnostic accuracy of MRI in detecting perineural spread (PNS) of head and neck tumours using histopathological or surgical evidence from the afflicted nerve as the reference standard.
**Methods or Backround:** Previous studies in the English language published in the last 30 years were searched from PubMed and Embase databases. We included studies that used MRI (with and without contrast enhancement) to detect PNS, histological or surgical confirmation of PNS, and reported exact numbers of patients required for assessing diagnostic accuracy. The outcome measures were sensitivity, specificity, positive predictive value (PPV), and negative predictive value (NPV). Heterogeneity was assessed with the Higgins inconsistency test (I2). P values smaller than 0.05 were considered statistically significant.
**Results or Findings:** A total of 11 retrospective studies were found reporting 319 nerve samples from 245 patients. Sensitivity ranged from 0.46 to 1.00, and specificity from 0.83 to 1.00, with median values of 0.96 and 0.88, respectively. Meta-analytic estimates and their 95% confidence intervals were: 0.85 (0.70-0.95), specificity 0.85 (0.80-0.89), PPV 0.86 (0.70-0.94), and NPV 0.85 (0.71-0.93). We found statistically significant heterogeneity for sensitivity (I2=72%, p=0.003) and PPV (I2=70%, p=0.038), but not for NPV (I2=65%, p=0.119) or specificity (I2=12%, p=0.842). The most frequent MRI features of PNS were nerve enlargement and enhancement. Squamous cell carcinoma and adenoid cystic carcinoma were the most common tumour types, and the facial and trigeminal nerves were the most commonly affected nerves in PNS. Only a few studies provided examples of false MRI diagnoses.
**Conclusion:** MRI has high diagnostic accuracy in depicting PNS of cranial nerves, yet this statement is based on scarce and heterogeneous evidence.
**Limitations:** The study is based on a limited number of retrospective studies with some studies subject to sampling bias.
**Ethics committee approval:** Since this is a systematic review, Institutional Review Board approval was not necessary.
**Funding for this study:** This study was financially supported by the Sigrid Jusélius Foundation.
**Author Disclosures:**
Bernd Pape: Nothing to disclose
Jussi Hirvonen: Nothing to disclose
Umida Bafoevna Abdullaeva: Nothing to disclose

**12:30-13:30** **Research Stage 1**

### Research Presentation Session: Interventional Radiology

### RPS 1909

### IR in the management of benign and malignant disease in male patients

**Moderator**
A. Torregrosa Andrés; Valencia/ES

### RPS 1909-2

**A single-centre retrospective study of 300 men with localised primary prostate cancer treated using MRI-guided transurethral ultrasound ablation (TULSA)**

*L. Engelage*[1], N. Behnel[1], P. Doerwald[2], L. Steinmeister[1], R. Muschter[1], A. Lumiani[1]; [1]Berlin/DE, [2]Hamburg/DE
*(lucas.engelage@altaklinik.de)*

**Purpose:** The objective of this study was to report a retrospective clinical service evaluation of TULSA for patients with organ-confined primary prostate cancer treated at a single centre.
**Methods or Backround:** 300 men with primary localised PCa visible on MRI and confirmed by biopsy were treated under clinical routine. Based on patients' preferences and disease characteristics, 163 men received whole-gland treatments and 137 focal TULSA. Adverse events (AEs), prostate-specific antigen (PSA), and functional and oncological outcomes were assessed using mp-MRI and biopsy, if necessary.
**Results or Findings:** Baseline characteristics include median [IQR] age 66 [60-73], PSA 6.85 ng/ml [4.64-9.5], overall cancer length 7.6 mm [4.35-10], prostate volume 49.2 cc [min 11–max 180], baseline risk stratifications include ISUP 1 (52/300), 2 (191/300), 3 (46/300), 4 (9/300), and 5 (2/300). 57 patients experienced Grade 1 and 2 complications, mostly resolving within 4 weeks through prolonged catheterisation and antibiotics. Grade 3 adverse events occurred in seven patients, resolving within 3 months. No grade 4 or higher AEs and no bowel-related complications were observed. Median [IQR] IIEF score of 24 [14-29] at baseline remained stable at the 48-mo follow-up with a value of 25 [13-30]. IPSS score immediately post-treatment increased from its baseline value of 8 [4-15] to 12 [5-19] and improved to 8 [2.5-10.5] by 12 months and further improved to 48-mo with 6.5 [3.5-8]. To the last follow-up, 26 men experienced biochemical failure (Phoenix≥2); MRI findings showed suspicion of residual cancer in 26 men, 54% (14/26) of which were confirmed by positive biopsy. 15 patients received salvage therapy (3 surgery, 12 other), 18 patients remained under active surveillance, and 13 patients underwent a single repeat TULSA.
**Conclusion:** TULSA offers promising safety, functional, and oncological results for the treatment of organ-confined prostate cancer.
**Limitations:** No systematic follow-up protocol limits this study.
**Ethics committee approval:** No ethical approval was sought for this study.
**Funding for this study:** Study is a clinical service evaluation and so no funding was obtained for this study.
**Author Disclosures:**
Pouya Doerwald: Author: Pouya Doerwald is an employee of Profound Medical.
Niklas Behnel: Nothing to disclose
Lucas Engelage: Nothing to disclose
Leonhard Steinmeister: Nothing to disclose
Rolf Muschter: Nothing to disclose
Agron Lumiani: Nothing to disclose

### RPS 1909-3

**Twelve-month outcomes of salvage transurethral ultrasound ablation (TULSA) for the treatment of localised radio-recurrent prostate cancer**

M. Anttinen[1], P. Mäkelä[1], *P. Doerwald*[2], P. Nurminen[1], T. Sainio[1], H. E. Pärssinen[1], R. Blanco Sequeiros[1], P. J. Boström[1]; [1]Turku/FI, [2]Hamburg/DE

**Purpose:** The purpose of this study was to report the 12-month safety and oncological outcomes of salvage TULSA (sTULSA) for men with localised radio-recurrent PCa.
**Methods or Backround:** Men with biopsy-proven localised PCa recurrence after RT were enrolled. mp-MRI and 18-F-PSMA-PET-CT were used to confirm organ-confined disease. Patients were followed every three months for adverse events (AEs, Clavien-Dindo), functional status, and PSA. Disease control was evaluated at 12-month by mpMRI, PSMA PET-CT, and prostate biopsy targeting the treatment area plus areas suspicious in imaging.

**Results or Findings:** Forty-one men underwent sTULSA (25 whole-gland and 16 focal ablation); one patient withdrew from the study due to frequent follow-up protocol three months after his treatment with undetectable PSA (<0.006 ng/ml). Baseline characteristics included median [IQR] age 73 [69-77], PSA 3.3 ng/ml [2.3-7], and an interval of 11 years [8-13] between RT and sTULSA. 12-month follow-ups are available for 31 men. AEs included two grade 3 events and 17 grade 2 events. One patient with castration-resistant PCa experienced a pubo-prostatic fistula and osteitis after sTULSA, treated with prolonged suprapubic catheterization and oral antibiotics. Median PSA at 12-month was 0.19 ng/ml (IQR 0.07–0.57) and was undetectable (<0.1) in 13 patients. Two patients were diagnosed with biochemical recurrence (PSA≥nadir+2) at 12-month follow-up, correlating with the extraprostatic disease on imaging. At 12-month MRI and PSMA PET-CT, 28/31 (90%) men had no visible cancer in the prostate, and four patients had seminal vesicle invasion. Biopsy outcomes at one year revealed that 26/30 (87%) were free of any PCa in the treatment region, while four patients had positive out-of-field biopsies.
**Conclusion:** Salvage therapy options for men with radio-recurrent PCa are limited. Twelve-month clinical outcomes of sTULSA show remarkable oncological outcomes with a favorable safety profile for treating localised radio-recurrent PCa.
**Limitations:** Small population
**Ethics committee approval:** Ethics committee approval was granted and informed consent was obtained from all study participants.
**Funding for this study:** This study was investigator-initiated so funding was received for it.
**Author Disclosures:**
Heikki Elias Pärssinen: Nothing to disclose
Peter J. Boström: Nothing to disclose
Pouya Doerwald: Author: Pouya Doerwald is an employee of Profound Medical.
Pertti Nurminen: Nothing to disclose
Teija Sainio: Nothing to disclose
Pietari Mäkelä: Nothing to disclose
Mikael Anttinen: Nothing to disclose
Roberto Blanco Sequeiros: Nothing to disclose

### RPS 1909-4

### CTA and angiographic prostatic artery anatomy in a Korean population

*S. H. Lee*, D. Shim, J. Yoo, B. Park, C. S. Park, S. K. Kim, J. W. Lee; Incheon/KR
*(shlee5205@naver.com)*

**Purpose:** This study aims to analyse the origins of prostatic arteries (PAs) in Korean population with LUTS or malignant hematuria and to compare with literatures. Prostatic artery embolisation (PAE) is a safe and effective treatment for lower urinary tract symptoms (LUTS) and hematuria from malignancy. Nevertheless, PAE remains challenging due to numerous anatomic variations.
**Methods or Backround:** From April 2018 to August 2023, 73 patients (mean age=68.9 ± 9.6) with LUTS (n=69) or malignant hematuria (n=4) underwent PAE. CTA and transcatheter angiography images were retrospectively reviewed for arterial anatomy evaluation. The branching pattern of internal iliac artery (IIA) and the origin of PA were categorised, and the incidence of each type was recorded.
**Results or Findings:** PAE was successfully implemented in 143 of 146 pelvic sidewalls. PA cannulation failed in three sidewalls due to total IIA occlusion. Most common IIA was A type (dividing into superior gluteal and gluteal-pudendal trunk, 72%). Eleven of 143 sidewalls exhibited dual PAs. A total of 154 PA origin was investigated. Internal pudendal artery origin (IPA, type IV) was most common, 40%, followed by superior vesical artery origin (type I, 25%) and obturator origin (type III) in 19%. Less commonly, anterior division of IIA origin (type II) was 7%. Type V (uncommon origins) was 8% including three distal IPA, two inferior gluteal, two medial femoral circumflex, and two rectal arteries and two quadfurcation and one trifurcation patterns.
**Conclusion:** In Korean population, the most common IIA pattern and PA origin was type A and IV, respectively, which aligns with previous reports.
**Limitations:** The limitations of the study include its retrospective nature, small sample size, and single centre study.
**Ethics committee approval:** This study is a retrospective study hence no ethical approval was required.
**Funding for this study:** No funding was received for this study.
**Author Disclosures:**
Borim Park: Nothing to disclose
Jung Whee Lee: Nothing to disclose
Seung Hyun Lee: Author: Collection of data and write manuscript
Chang Suk Park: Nothing to disclose
Jimin Yoo: Nothing to disclose
Sun Ki Kim: Nothing to disclose
Dongjae Shim: Author: Collection of data and write manuscript

### RPS 1909-5

### Six years of prostatic artery embolisation (PAE): a comprehensive and critical single-centre, single-operator retrospective analysis of 551 cases

*L. S. Alizadeh*, C. Booz, I. Yel, D. Radek, T. Vogl; Frankfurt a. Main/DE

**Purpose:** The objective of this study was to evaluate the efficacy, safety, and outcomes of prostatic artery embolisation (PAE) in patients with benign prostatic hyperplasia (BPH) over a six-year period at a single centre and to discern its impact on clinical symptoms, prostate volume, and procedural specifics.
**Methods or Backround:** A single-centre retrospective study was conducted, analysing 551 PAE interventions performed by a single interventional radiologist from January 2017 to July 2023. The study reviewed technical specifics, changes in prostate volume, PSA levels, International Prostate Symptom Score (IPSS), Quality of Life (QoL) scores, International Index of Erectile Function (IIEF), and procedural radiological metrics.
**Results or Findings:** The average age of patients was 68.81 years. A significant majority underwent bilateral embolisations in a single session (435 out of 551) with particles predominantly sized 100-300 µm (441 out of 551). Post-intervention, there was a notable reduction in prostatic volume (mean decrease of 9.66 ml) and PSA levels (mean decrease of 0.32 ng/ml). Symptomatic relief was evident with a substantial drop in IPSS (mean decrease of 9.17) and improved QoL scores (mean decrease of 1.74). IIEF scores showed a slight increase post-procedure. The average fluoroscopy time was 25.23 minutes, with an average dose area product (DAP) of 25452.08 µGym².
**Conclusion:** PAE emerges as a potent therapeutic intervention for BPH patients, offering symptomatic relief, reduction in prostate volume, and a favorable safety profile. We reported increasing patient numbers since the implementation of the technique. This single-center experience provides a comprehensive insight into the real-world applicability and efficacy of PAE.
**Limitations:** Single centre retrospective trial limits the scope of this study.
**Ethics committee approval:** The ethical approval was received from the Institutional ethics committee with a waiver for informed consent.
**Funding for this study:** No funding was obtained for this study.
**Author Disclosures:**
Christian Booz: Nothing to disclose
Ibrahim Yel: Nothing to disclose
Thomas Vogl: Nothing to disclose
Leona Soraja Alizadeh: Nothing to disclose
David Radek: Nothing to disclose

### RPS 1909-6

### 12-month outcomes of MRI-guided transurethral ultrasound ablation (TULSA) for the treatment of benign prostatic hyperplasia

A. Viitala[1], M. Anttinen[1], P. Mäkelä[1], *P. Doerwald*[2], P. Nurminen[1], H. E. Pärssinen[1], T. Sainio[1], R. Blanco Sequeiros[1], P. J. Boström[1]; [1]Turku/FI, [2]Hamburg/DE

**Purpose:** The aim of this study was to assess clinical outcomes of MRI-guided transurethral ultrasound ablation (TULSA) in treating benign prostatic hyperplasia (BPH).
**Methods or Backround:** Men with BPH scheduled for TURP were enrolled. EPIC-26, IPSS, IIEF-5, uroflowmetry, PSA, MRI, and complications in Clavien-Dindo were recorded.
**Results or Findings:** Thirty patients received TULSA, with a median follow-up of 16-mo (max 48-mo). At baseline, median [IQR] age was 67 years [64–72], PSA 3.1 ug/l [2.2–6.9], prostate volume 51.5 ml [min 29-max 107], average flow rate 4.1 ml/s [3.4–7], maximum flow rate 11 ml/s [8–15], voided volume 211 ml [139–336], and post-void residual(PVR) 71 ml [40–247]. Median sonication, hospitalization, and catheterization times were 39 min, 24 h, and 16 d, respectively. Complications included one grade 1, 8 grade 2, and one grade 3 events, all resolving within three-months. 24/30 patients have completed their 12-month follow-up with two men dropping off due to cancer diagnosis. Between baseline and 12 months, median values for measured parameters changed as follows: prostate volume, PSA, PVR reduced from 51.5 to 31 ml, 3.1 to 1.5 ug/l, and 71 to 41.5 ml, respectively. Average flow rate, Qmax, and voided volume increased from 4.1 to 8.7 ml/s, 11 to 18 ml/s, 211 to 301 ml, respectively. IPSS, IPSS QoL, IIEF-5 scores improved from 16.5 to 4, 4 to 1, and 15 to 19, respectively. EPIC-26 urinary incontinence, irritative/obstructive, bowel, sexual, and hormonal domains improved from 85.5 to 100, 65.6 to 94, 87.5 to 100, 54 to 67, and 95 to 100, respectively. Results of uroflowmetry, functional, and QOL questionnaires all improved at 12-month despite discontinuation of LUTS medication in 26/30 patients.
**Conclusion:** 12-mo clinical-outcomes demonstrate safety and efficacy of TULSA for treating BPH.
**Limitations:** Small population
**Ethics committee approval:** Ethics committee approval was granted and informed consent was obtained from all study participants.

**Funding for this study:** Study was funded by Profound Medical.
**Author Disclosures:**
Heikki Elias Pärssinen: Nothing to disclose
Antti Viitala: Nothing to disclose
Peter J. Boström: Nothing to disclose
Pouya Doerwald: Author: Pouya Doerwald is an employee of Profound Medical.
Pertti Nurminen: Nothing to disclose
Teija Sainio: Nothing to disclose
Pietari Mäkelä: Nothing to disclose
Mikael Anttinen: Nothing to disclose
Roberto Blanco Sequeiros: Nothing to disclose

### RPS 1909-7

**Recurrence rates of male varicocele after endovascular treatment using NBCA-MS (Glubran2): a retrospective study**

*A. Mininni*, L. Tomasino, E. Lanza, G. Ferrillo, D. Poretti, M. Francone, V. Pedicini; Milan/IT

**Purpose:** Male varicocele is characterised by high prevalence in young individuals, diminished fertility rates and adverse impact on the quality of life. The aim of this study is to evaluate the recurrence rate of varicocele in patients who had undergone endovascular treatment using N-butyl 2 cyanoacrylate plus methacryloxy sulfolane (NBCA-MS, Glubran2) glue.
**Methods or Backround:** A retrospective analysis of varicocele embolisation procedures completed between January 2016 and December 2022 was performed. A total of 102 patients were recruited (mean age 27.7 years) through phone interview. Before the procedure 10.8% patients had stage two disease severity; 61.8% had stage three and 27.5% had stage four. All patients manifested typical varicocele symptoms such as testicular heaviness, pain or spermiogram abnormalities. Improvement in spermiogram, recurrence rate of disease and need for new treatment were evaluated.
**Results or Findings:** Spermiogram improvement was observed in 81.3% of patients (49 patients underwent semen analysis 6-8 months after the procedure). Recurrence rate of disease was 10.8% (11 patients) and among those, patients requiring new treatment were 45.5% (three patients needed new endovascular embolisation treatment using NBCA-MS, and two patients needed classic surgery). No significant post-procedural complications were reported.
**Conclusion:** Endovascular embolisation using NBCA-MS glue is an effective treatment for male varicocele, offering very low recurrence rates and negligible complications.
**Limitations:** The single-centre and retrospective nature of the study were identified limitations.
**Ethics committee approval:** IRB approval was waived considering the retrospective non-interventional study design.
**Funding for this study:** No funding was received for this study.
**Author Disclosures:**
Vittorio Pedicini: Nothing to disclose
Alessandra Mininni: Nothing to disclose
Giuseppe Ferrillo: Nothing to disclose
Luigi Tomasino: Nothing to disclose
Ezio Lanza: Nothing to disclose
Marco Francone: Nothing to disclose
Dario Poretti: Nothing to disclose

### RPS 1909-8

**Endovascular embolisation for high-flow priapism in paediatric patients**

*E. Karapinar*, C-C. Ercan, M. S. Çakır, A. B. Aydin; Istanbul/TR
*(edanurkarapinarr@gmail.com)*

**Purpose:** High-flow priapism is a persistent penile tumescence, which is relatively uncommon compared to low-flow priapism. It is typically associated with a history of trauma leading to lacerations in the cavernosal arteries within the corpora cavernosa. Super-selective arterial embolisation is a well-established therapeutic technique; however, conservative management is usually the initial approach in paediatric population. As a tertiary medical centre, we are compelled to provide treatment to those who do not respond to conservative measures.
**Methods or Backround:** We observed a total of four paediatric patients with high-flow priapism between 2020-2022. Three of these patients had documented trauma histories, while the cause in one patient remained uncertain due to their mental status. All patients underwent conservative treatment for up to two weeks, with no observed response. Initially, we conducted Doppler ultrasound examinations to assess arterial waveforms and identify fistula tracts in the corpora cavernosa. Subsequently, we performed conventional angiography and proceeded with an embolisation plan.
**Results or Findings:** Among the patients, three exhibited angiographic evidence of arteriocavernosal fistulas. Super-selective embolisation with gel-foam was successfully completed in all patients, and post-procedural angiography revealed the absence of residual pathological flow. In long-term follow-up, all of these patients exhibited recovery with no reported complications overtime. Early postoperative and subsequent intermittent follow-up Doppler studies have consistently demonstrated normal flow. The fourth patient, who had an uncertain history of trauma, exhibited normal angiographic features and was deemed ineligible for embolisation.
**Conclusion:** Super-selective embolisation emerges as a promising technique for managing high-flow priapism not only in adults but also in paediatric patients.
**Limitations:** Given the rarity of this condition, our study was limited by the small number of patients.
**Ethics committee approval:** We adapted a treatment modality with proven successful outcomes in the literature to our own patients under the most suitable conditions. We did not employ any additional medications or medical interventions beyond the methods documented in the literature.
**Funding for this study:** No funding was received for this study.
**Author Disclosures:**
Ahmet Baris Aydin: Nothing to disclose
Edanur Karapinar: Nothing to disclose
Mehmet Semih Çakır: Nothing to disclose
Celal-Caner Ercan: Nothing to disclose

### RPS 1909-9

**MRI-guided transrectal focal laser ablation for localised low and intermediate risk prostate cancer: initial experience using an integrated laser ablation system**

Y. Wimper, L. te Molder, M. Sedelaar, J. Bomers, C. G. Overduin, *J. Fütterer*; Nijmegen/NL
*(jurgen.futterer@radboudumc.nl)*

**Purpose:** The aim of this study was to investigate the feasibility and safety of an integrated system for MRI-guided FLA in localized PCa. Current active treatment of localised prostate cancer (PCa) is directed towards whole-gland radical treatment. MRI-guided focal laser ablation (FLA) presents a minimally invasive treatment alternative for selected patients offering targeted tumour treatment at reduced morbidity. It relies on a laser applicator combined with real-time MR thermometry feedback for accurate ablation control. However, in current systems these are often non-integrated. Our study will enable a more precise tissue ablation without damaging vital adjacent structures.
**Methods or Backround:** Between April 2022 and May 2023, 10 patients (mean age: 66±7.2 years) with low- and intermediate risk PCa were prospectively included and treated with MRI-guided FLA using an integrated FLA system (Tranberg,CLS). Primary endpoints were technical success, procedure-related adverse events (AEs) and tumour response on MRI at 6 months. Secondary endpoints were prostate-specific antigen (PSA) levels, sexual and urinary function response at 6 months.
**Results or Findings:** Technical success was achieved in 10/10 (100%) patients. Two AEs were observed, acute urinary retention (classification D, n=1) and urinary tract infection (classification B, n=1). Six-month follow-up was available in 6/10 patients. In all six patients, follow-up MRI indicated no evidence of residual tumor. At six months, mean PSA level was reduced (5.9 vs. 3.4 ng/mL; p=0.59), urinary function scores (10.0 vs. 7.8; p=0.37) and sexual function scores improved (10.3 vs. 15.4; p=0.47) compared to baseline, all being non-statistically significant.
**Conclusion:** Our study demonstrates feasibility and safety of an integrated MRI-guided FLA system in patients with low-to-intermediate risk PCa. Initial experience indicates good oncological response and potential improvement in urinary and sexual function.
**Limitations:** This feasibility study is limited by a small, single-institution cohort and short-term follow-up.
**Ethics committee approval:** The ethical approval was obtained by the Institutional Review Board.
**Funding for this study:** Clinical Laserthermia Systems has provided funding for this study.
**Author Disclosures:**
Jurgen Fütterer: Nothing to disclose
Christiaan G. Overduin: Nothing to disclose
Michiel Sedelaar: Nothing to disclose
Lauren te Molder: Nothing to disclose
Yvonne Wimper: Nothing to disclose
Joyce Bomers: Nothing to disclose

**12:30-13:30** **Research Stage 2**

### Research Presentation Session: Musculoskeletal

### RPS 1910

### CT applications in musculoskeletal imaging

**Moderator**
P. Omoumi; Lausanne/CH
*(patrick.omoumi@chuv.ch)*

### RPS 1910-2

**Value-added opportunistic chest CT screening for osteoporosis using bone mineral density estimation computed by multi-view semi-supervised learning**

*J. Yang*[1], H. Guo[2], L. Le[2], M. Xu[2], H. Shuishi[1], F. Yang[1]; [1]Wuhan/CN, [2]Hangzhou/CN
*(yangjr931@163.com)*

**Purpose:** The aim of the study was to evaluate the value of bone mineral density estimation computed by multi-view semi-supervised learning on chest CT for opportunistic osteoporosis screening.
**Methods or Backround:** A total of 1295 patients who underwent chest CT for lung cancer screening and received dual-energy X-ray absorptiometry (DXA) during the same period were collected. They were divided into three groups: normal group, osteopenia group and osteoporosis group based on bone mineral density measured by DXA. And a new proposed indicator—bone mineral density estimation of all thoracic vertebrae and the first lumbar vertebra were automatically computed by multi-view semi-supervised learning on chest CT images. The differences of bone mineral density estimation were compared among the three groups. And the diagnostic efficacy for distinguishing osteopenia and osteoporosis from normal group were also evaluated.
**Results or Findings:** The bone mineral density estimation of thoracic and first lumbar vertebrae was significantly different among three groups and between any two groups ($P<0.001$), and decreased successively in normal group, osteopenia group and osteoporosis group. They were positively correlated with t-values of the first lumbar vertebrae ($R = 0.58$-$0.77$, $P<0.01$). And they had high diagnostic efficacy for distinguishing osteopenia and osteoporosis from normal group (AUC = 0.777~0.824).
**Conclusion:** The bone mineral density estimation of thoracic and first lumbar vertebrae, which computed by multi-view semi-supervised learning on chest CT, is of great value in the opportunistic screening of osteopenia and osteoporosis.
**Limitations:** The new marker—bone mineral density estimation can help patients realize the value-added value of chest CT, that is, during a chest CT examination, not only lung cancer can be screened, but also osteoporosis opportunistic screening can be realized. However, it was a single-center, retrospective study.
**Ethics committee approval:** The ethical approval was obtained by the Ethics Committee of Wuhan Union Hospital ([2021]0853).
**Funding for this study:** No funding was obtained for this study.
**Author Disclosures:**
Fan Yang: Nothing to disclose
Minfeng Xu: Nothing to disclose
Jinrong Yang: Nothing to disclose
Lu Le: Nothing to disclose
He Shuishi: Nothing to disclose
Heng Guo: Nothing to disclose

### RPS 1910-3

**Multiparametric contrast-enhanced dual-energy CT as a one-stop shop approach in arthritis imaging-first results of the HAI-DECT study**

*S. T. Ulas*, K. Ziegeler, J. Mews, U. Schneider, S. Ohrndorf, R. Biesen, D. Poddubnyy, F. N. Proft, T. Diekhoff; Berlin/DE

**Purpose:** This study aimed to evaluate the efficacy and diagnostic utility of contrast-enhanced dual-energy computed tomography (CE-DECT) in detecting and differentiating rheumatic joint diseases.
**Methods or Backround:** Patients with suspected hand arthritis were prospectively enrolled. All underwent CE-DECT imaging alongside the standard clinical evaluation. CE-DECT (80/135 kVp) was captured pre and three-minutes post weight-adapted contrast agent application. Reconstructions included two-material decomposition for tophus imaging, virtual non-calcium for bone marrow edema, and CT-subtraction for soft-tissue inflammation. Diagnoses using CE-DECT were juxtaposed with the initial and final evaluations by referring rheumatologists. A subsequent survey assessed CE-DECT's diagnostic utility and impact on patient management, rated on a 1-10 scale. Descriptive statistics were employed.
**Results or Findings:** Out of 67 patients, diagnoses included rheumatoid arthritis (29), psoriatic arthritis (8), crystal disease (13) and others, for example osteoarthritis and peripheral spondyloarthritis (17). In 57 cases (85%), CE-DECT diagnoses aligned with final clinical evaluations. CE-DECT altered clinical diagnoses for 21 patients (31%). Rheumatologists rated CE-DECT's diagnostic utility at an average of 8.31 (SD 2.49) and its contribution to patient management at 8.61 (SD 2.19).
**Conclusion:** CE-DECT offers standardized arthritis imaging. Its diverse diagnostic capabilities make it a potent tool for early diagnosis in rheumatic joint diseases.
**Limitations:** No limitations were identified.
**Ethics committee approval:** The study was approved by the local ethics committee (EA1/183/21) and the Federal Office for Radiation Protection (ZD 3-22464/2021-285-G).
**Funding for this study:** No funding was received for this study.
**Author Disclosures:**
Robert Biesen: Nothing to disclose
Denis Poddubnyy: Nothing to disclose
Torsten Diekhoff: Nothing to disclose
Jürgen Mews: Nothing to disclose
Katharina Ziegeler: Nothing to disclose
Sarah Ohrndorf: Nothing to disclose
Fabian N. Proft: Nothing to disclose
Udo Schneider: Nothing to disclose
Sevtap Tugce Ulas: Nothing to disclose

### RPS 1910-4

**Risk factors for compression fractures in patients treated with gastrectomy: emphasis on bone mineral density measured on opportunistic CT**

*S. Lee*, K. W. Kim; Seoul/KR
*(lsz95@naver.com)*

**Purpose:** The aim of this study was to assess apply osteosarcopenia measurement methods in CT patients. Osteosarcopenia is the coexistence of osteoporosis and sarcopenia, which has a significant impact on the survival outcomes of critically ill patients. Many studies tried to diagnose osteosarcopenia using opportunistic CT. However, in case of patients treated with gastrectomy, the loss of bone mineral density and muscle mass is not routinely evaluated, even though these patients often experience bone and muscle shrinkage and subsequent compression fractures after surgery. Therefore, we assessed bone mineral density (BMD) and skeletal muscle area (SMA) in these patients by applying osteosarcopenia measurement methods in opportunistic abdominal CT.
**Methods or Backround:** Among 2457 patients who had undergone gastrectomy between January 2003 and December 2010, we included 100 patients (mean age 58 years). Preoperative and 1-year postoperative abdominal CT scans were evaluated for BMD and SMA. Region of interest was situated in L1 vertebral body to estimate trabecular attenuation value to analyze BMD and SMA. To determine predictive risk factors for compression fractures, we implemented univariate logistic regression analysis.
**Results or Findings:** Between preoperative and postoperative CT, all patients showed a decline in the SMA ($116 \pm 27$ cm2 vs. $110 \pm 25$ cm2, $p<0.0001$) and the BMD ($177 \pm 50$ HU vs. $166 \pm 50$ HU, $p<0.0001$). Body weight ($60 \pm 9$ kg vs. $54 \pm 9$ kg, $p<0.0001$) and BMI ($24 \pm 3$ vs. $21 \pm 3$, $p<0.0001$) were also decreased. During the five-year follow-up record, five patients experienced osteoporotic vertebral compression fractures. Univariate analysis showed preoperative and postoperative BMD were significant risk factors for compression fractures, apart from age, sex, preoperative and postoperative SMA.
**Conclusion:** Preoperative and postoperative BMD measured on opportunistic CT are significant risk factors for compression fractures in patients treated with gastrectomy.
**Limitations:** Single center study and retrospective design.
**Ethics committee approval:** The study was approved by the IRB with the approval code: 2023-0976.

**Funding for this study:** No funding was obtained for this study.
**Author Disclosures:**
Suiji Lee: Nothing to disclose
Kyung Won Kim: Nothing to disclose

### RPS 1910-5

**Photon-counting detector computed tomography (PCD-CT) for quantitative microstructural imaging of bone**

A. Burghardt[1], A. Korajac[2], A. E. Strassl[2], S. Sadoughi[1], G. Kazakia[1], *J. M. Patsch*[2]; [1]San Francisco, CA/US, [2]Vienna/AT
*(janina.patsch@meduniwien.ac.at)*

**Purpose:** The goal of this study was to evaluate feasibility, performance and accuracy of PCD-CT for bone structure quantification. The quantification of bone quality by high-resolution research CT scanners (built for the peripheral skeleton) has been shown to improve fracture risk prediction. Now, photon-counting detector CT (PCD-CT) has been introduced as a clinical imaging tool with a generational advance in imaging performance.
**Methods or Backround:** Cadaveric phantoms (n= 20 1-cm metaphyseal sections of distal radius and tibia) were imaged by µCT, 1st- and 2nd-generation HR-pQCT, and PCD-CT. PCD-CT scans were acquired at 120kVp in Ultra-High Resolution (UHR) mode (200mAs, 16.1mGy). A 15mm FOV was reconstructed across 1024x1024 matrix using the Br92 kernel (146µm pixel size, 150µm slice thickness). Spatial resolution (10% MTF) and noise performance (SNR) were measured in idealized density and wire phantoms. Standard density, geometry, and structure measures were quantified and evaluated for accuracy by linear regression & Bland-Altman analysis.
**Results or Findings:** PCD-CT provided an exquisite depiction of cortical and trabecular microstructure. PCD-CT noise performance (SNR=20.5) was superior to 1st- and 2nd-gen HR-pQCT research scanners (SNR=15.4 and 7.1). The spatial resolution of PCD-CT (10% MTF=132 µm) was comparable to 1st-gen HR-pQCT (10% MTF=138 µm), and inferior to 2nd-gen HR-pQCT (10% MTF=95 µm). Accuracy of PCD-CT was high (R2>0.9) for trabecular bone volume and trabecular number, and comparable to 2nd-gen HR-pQCT.
**Conclusion:** PCD-CT provides significant advantages for measuring human bone structure on a clinical device, including superior noise performance and scan time, while spatial resolution is comparable to 1st-gen HR-pQCT and accuracy was comparable to 2nd-gen HR-pQCT. PCD-CT represents an exciting opportunity to translate quantitative microstructural skeletal imaging to a clinical platform.
**Limitations:** Actual in vivo-translation (= in-vivo scanning) is work in progress.
**Ethics committee approval:** Approval has been obtained for specimen donation and use.
**Funding for this study:** No funding was received for this study.
**Author Disclosures:**
Janina Maria Patsch: Nothing to disclose
Galateia Kazakia: Nothing to disclose
Andreas Eduard Strassl: Nothing to disclose
Aida Korajac: Nothing to disclose
Andrew Burghardt: Nothing to disclose
Saghi Sadoughi: Nothing to disclose

### RPS 1910-6

**Prediction of osteoporosis associated fractures: evaluation of dual-energy CT-derived metrics of the lumbar spine**

*L. D. Grünewald*, V. Koch, S. Mahmoudi, P. Reschke, S. Martin, C. Booz, I. Yel, T. Vogl; Frankfurt a. Main/DE
*(leonosddc@gmail.com)*

**Purpose:** To assess the association of dual-energy CT (DECT)-derived bone mineral density (BMD) assessment with the occurrence of acute insufficiency fractures of the spine and follow-up fractures in a 2-year period.
**Methods or Backround:** L1 of 160 patients (77 men, 83 women; mean age, 64.1 years, range, 19-94 years) who underwent dual-source DECT between 01/2016 and 12/2020 was retrospectively analyzed. For phantomless BMD assessment, a dedicated DECT postprocessing software based on material decomposition was manually applied. All depicted vertebrae were examined for signs of recent insufficiency fractures, and electronic health records were examined to obtain the incidence of osteoporotic fractures for a follow-up of 2 years after DECT. Receiver-operating characteristic (ROC) analysis was used to calculate AUC values, and logistic regression models were used to determine the associations of BMD, sex, and age with the occurrence of insufficiency fractures and follow-up fractures.
**Results or Findings:** A DECT-derived BMD threshold of 120.40 mg/cm³ yielded an AUC of 0.82 (p < .0001) to identify patients with one or more insufficiency fractures of the spine from patients without fracture, and a DECT-derived BMD cut-off of 93.70 mg/cm³ yielded an AUC of 0.9373 (CI, 0.867-0.977, p < .001) for the prediction of follow-up fractures within 2 two years after DECT. A lower DECT-derived BMD was associated with an increased risk to sustain insufficiency (Odds ratio of 0.93, 95% CI, 0.91-0.96, p < .001), and follow-up fractures (Odds ratio of 0.8710, 95% CI, 0.091-0.9375, p < .001), indicating a protective effect of increased DECT-derived BMD.
**Conclusion:** Dual-energy CT-derived bone mineral density assessment from routine examinations can be used to stratify the risk of sustaining osteoporosis-associated fractures following acute trauma and during a follow-up period of 2 years.
**Limitations:** Preselection Bias, Limited to Dual-Energy-CT, Retrospective Study
**Ethics committee approval:** Informed consent waived due to retrospective nature.
**Funding for this study:** No funding was obtained for this study.
**Author Disclosures:**
Simon Martin: Nothing to disclose
Christian Booz: Nothing to disclose
Ibrahim Yel: Nothing to disclose
Thomas Vogl: Nothing to disclose
Vitali Koch: Nothing to disclose
Scherwin Mahmoudi: Nothing to disclose
Philipp Reschke: Nothing to disclose
Leon David Grünewald: Nothing to disclose

### RPS 1910-7

**Cone beam CT in the emergency department: revising incidence rates for carpal bone fractures**

*S. Murphy*, E. McDermott, B. Gibney, P. J. Macmahon; Dublin/IE
*(sophieacmurphy@gmail.com)*

**Purpose:** The aim of this study was to evaluate and quantify the incidence of acute radio-carpal fractures when routinely employing CBCT in a major trauma centre.
**Methods or Backround:** In our institution Cone beam computed tomography (CBCT) is routinely utilised in the Emergency Department (ED) to investigate patients who have suspected acute radio-carpal fractures but negative x-rays. We have noted that the frequency of wrist fractures as published in the literature does not match the frequency encountered in clinical practice when using CBCT to investigate these patients. A retrospective analysis was conducted on all CBCT scans performed for wrist trauma in the ED between January 2018 and August 2023. Fracture incidence, type, and specific carpal bone locations were characterized.
**Results or Findings:** A total of 690 wrist CTCBs were performed on ED patients across the study period. 52% were female with a mean age of 44.5 years. Forty four percent (306/690) were positive for a fracture even though x-rays were negative, and 24% (72/306) of positive studies had more than one fracture identified. A total of 386 discrete fractures were identified and characterised like this: 62% Carpal bones (240), 24% Distal radius (93), 10%Metacarpal bone (38), 4% Distal Ulna (15). Of the 240 carpal bone fractures: 29%Trapezium (70), 29% Scaphoid (69), 22% Triquetral/triquetrum (53), 8% Hamate (18), 4% Pisiform (9), 3% Trapezoid (8), 3% Capitate (7), 3% Lunate (6).
**Conclusion:** Our data indicates that a revision in the conventional carpal bone fracture incidence estimates is needed, suggesting a much higher frequency of certain carpal bone fractures (esp trapezium) when CBCT is used as the imaging modality in wrist trauma assessment.
**Limitations:** Single centre study.
**Ethics committee approval:** Not applicable
**Funding for this study:** No funding was obtained for this study.
**Author Disclosures:**
Peter Joseph Macmahon: Nothing to disclose
Edward McDermott: Nothing to disclose
Brian Gibney: Nothing to disclose
Sophie Murphy: Nothing to disclose

### RPS 1910-8

**Potential of dual-energy CT-based collagen maps for the assessment of thoracic disc degeneration**

S. Mahmoudi, *L. D. Grünewald*, V. Koch, J-E. Scholtz, S. Martin, D. Pinto Dos Santos, C. Booz, T. Vogl, I. Yel; Frankfurt a. Main/DE
*(leonosddc@gmail.com)*

**Purpose:** The objective of this study was to assess the potential of Dual-Energy CT (DECT)-derived collagen maps for the assessment of disc degeneration in the thoracic spine.
**Methods or Backround:** We retrospectively included 51 patients who received both dual-source DECT (third generation dual-source DECT; Somatom Force; Siemens Healthineers) and MRI of the thoracic spine within two weeks between July 2020 and October 2022. Disc degeneration was independently evaluated by two blinded readers in DECT-derived collagen maps. Based on the Pfirmann grading system, thoracic disc generation was classified into no/mild (Pfirmann Grade 1&2), moderate (Pfirmann Grade 3&4), and severe (Pfirmann Grade 5). To assess the diagnostic value of DECT-derived color-coded collagen maps for the evaluation of thoracic disc degeneration, we calculated diagnostic accuracy, sensitivity and specificity using MRI as reference standard. 5-point Likert scales were used to evaluate diagnostic confidence (1=insufficient,2=low,3=moderate,4=high,5=excellent) and image

Saturday quality (1=non-diagnostic,2=weak,3=moderate,4=good,5=excellent) subjectively.
**Results or Findings:** In total, 612 intervertebral discs from 51 patients were evaluated (mean age, 68±16 years, 23 female). MRI depicted 135 non/mildly degenerated discs (22.1%), 470 moderately degenerated discs (76.8%), and seven severely degenerated discs (1.1%). Collagen maps achieved a high level of sensitivity (792 of 954 [83.0 %], specificity (221 of 270 [81.9 %]), and diagnostic accuracy (1013 of 1215 [83.4 %]) to discriminate between non/mild and moderate/severe thoracic disc degeneration. Inter-reader reliability was substantial (κ=0.82) for DECT collagen maps (p<.001). The subjective evaluation of disc degeneration using DECT-derived collagen maps showed moderate to high diagnostic confidence (median 3.5, interquartile range (IQR) 3-4) and moderate to good image quality (median 3.5, IQR 3.5-4).
**Conclusion:** The ability of DECT-derived collagen maps to evaluate the degree of disc degeneration may facilitate assessment of thoracic disc degeneration in patients with disc-related pathologies when MRI is not available.
**Limitations:** Not applicable for this study.
**Ethics committee approval:** The ethical review board of our institution approved this retrospective study and waived written informed consent.
**Funding for this study:** No funding was provided for this study.
**Author Disclosures:**
Simon Martin: Nothing to disclose
Christian Booz: Speaker: Siemens
Ibrahim Yel: Speaker: Siemens
Thomas Vogl: Nothing to disclose
Jan-Erik Scholtz: Nothing to disclose
Vitali Koch: Nothing to disclose
Scherwin Mahmoudi: Nothing to disclose
Daniel Pinto Dos Santos: Nothing to disclose
Leon David Grünewald: Nothing to disclose

**12:30-13:30** **Research Stage 4**

### Research Presentation Session: Paediatric

### RPS 1912

### The newest techniques in paediatric radiology: faster imaging, less radiation and contrast agents

**Moderator**
E. Vázquez Méndez; Barcelona/ES
*(elida.vazquez@vallhebron.cat)*

### RPS 1912-2

**Factors associated with CT Scan repetition among paediatrics and its association with cancer risk: a systematic review and meta-analysis study**

*T. Alshangeeti*[1], R. Ahmad[1], P. D. M. Abdullah Alshawsh[1], M. Elzaki[2]; [1]Kuala Lumpur/MY, [2]Madinah/SA
*(drshangeeti@gmail.com)*

**Purpose:** The study aimed to evaluate and quantify the risk of cancer in paediatric patients due to exposure to CT scans, including single and repeated exposure. To identify the types of cancer associated with radiation from CT and investigate the repeat rate, cumulative radiation doses, and reasons behind multiple exposures in paediatric populations.
**Methods or Backround:** A review of pediatric patients' CT scan repetition found factors contributing to it and its link to cancer risk. The study, which included 33 studies through Web of Science, Scopus, and PubMed, had fivestudies with over 7 million participants in the meta-analysis. The Newcastle-Ottawa Scale was used to assess potential bias. The study offers a comprehensive evaluation of the evidence regarding exposure and outcome.
**Results or Findings:** A meta-analysis found an increased risk of overall cancer and brain tumours for children who underwent CT scans. The risk of brain tumours increased by 53% and the evidence for an increased risk of leukaemia was less conclusive. A dose-response effect was observed, with patients receiving two or more repeat CT scans showing a markedly elevated risk compared to those who had only one scan. Many patients undergo repeat scans for injury reassessment rather than a change in their clinical condition, particularly in head injuries. A study found a disparity in radiation exposure levels and the lack of implementation of CT dose optimization strategies in hospitals, resulting in unnecessarily high radiation doses.
**Conclusion:** CT scan exposure poses a significant risk of cancer, especially brain tumours. Repeat scans may increase the risk of leukaemia.
**Limitations:** The heterogeneity in the selection of variables used to assess cancer risk and the lack of clear explanations for why CT scans were repeated in the articles were identified.
**Ethics committee approval:** We registered the protocol of this systematic review in the International Prospective Register of Systematic Reviews (PROSPERO) database, which can be accessed at http://www.crd.york.ac.uk/PROSPERO, and it is identified with the record number CRD42022342579
**Funding for this study:** No funding was obtained for this study.
**Author Disclosures:**
Mohammed Abdullah Alshawsh: Nothing to disclose
Tahani Alshangeeti: Nothing to disclose
Maisa Elzaki: Nothing to disclose
Rozilawati Ahmad: Nothing to disclose

### RPS 1912-3

**Myocardial strain assessment in the human foetus by cardiac MRI using Doppler ultrasound gating and feature tracking**

*B. Schönnagel*, M. Tavares de Sousa, P. Bannas, G. Adam, R. Fischer, L. Huber, M. Kaul, J. Herrmann, M. Dargahpour Barough; Hamburg/DE
*(b.schoennagel@uke.de)*

**Purpose:** The aim of this study was the assessment of myocardial strain by feature tracking magnetic resonance imaging (FT-MRI) in human fetuses with and without congenital heart disease (CHD) using cardiac Doppler ultrasound (DUS) gating.
**Methods or Backround:** Forty-three human foetuses (gestational age: 28-41 weeks) underwent dynamic cardiac MRI at 3T. Cine balanced steady-state free-precession imaging was performed using foetal cardiac DUS gating. FT-MRI was analysed using dedicated post-processing software. Endo- and epicardial contours were manually delineated from foetal cardiac 4-chamber views, followed by automated propagation to calculate global longitudinal strain (GLS) of the left (LV) and right ventricle (RV), LV radial strain, and LV strain rate.
**Results or Findings:** Strain assessment was successful in 38/43 fetuses (88%), 25 of them had postnatally confirmed diagnosis of CHD (e.g. coarctation, transposition of great arteries) and 13 were heart healthy. Five foetuses were excluded due to reduced image quality. In foetuses with CHD compared to healthy controls median LV-GLS (-13.2% vs. -18.9%; P<0.007), RV-GLS (-7.9% vs. -16.2%; P<0.006), and LV strain rate (1.4s-1 vs. 1.6s-1; P<0.003) were significantly higher (i.e., less negative). LV radial strain was without a statistically significant difference (20.7% vs. 22.6%; P=0.1). Bivariate discriminant analysis for LV-GLS and RV-GLS revealed sensitivity of 67% and specificity of 93% to differentiate between foetuses with CHD and healthy foetuses.
**Conclusion:** Myocardial strain was successfully assessed in the human foetus performing dynamic fetal cardiac MRI with DUS gating. Our study indicates that strain parameters may allow for differentiation between foetuses with and without CHD.
**Limitations:** No direct comparison with echocardiography. Myocardial strain was assessed from four chamber views only.
**Ethics committee approval:** The ethical approval was obtained from the local ethics committee.
**Funding for this study:** This study received a grant by the Deutsche Forschungsgemeinschaft.
**Author Disclosures:**
Michael Kaul: Nothing to disclose
Gerhard Adam: Nothing to disclose
Manuela Tavares de Sousa: Founder: Northh medical
Roland Fischer: Nothing to disclose
Lukas Huber: Nothing to disclose
Peter Bannas: Nothing to disclose
Björn Schönnagel: Founder: Northh medical GmbH
Maryam Dargahpour Barough: Nothing to disclose
Jochen Herrmann: Nothing to disclose

### RPS 1912-4

**Location comparison of radiation doses in paediatric Hickman line insertion**

*S. Hosseinzadeh*; Oxford/UK

**Purpose:** Percutaneous Hickman line insertion is a common and necessary procedure for the delivery of chemotherapeutics, long term medications, and patients with difficult vascular access. In our institution, paediatric Hickman line insertion occurs in the radiology department fluoroscopy room or in surgical theatres. Ultrasound-guided puncture of internal jugular vein is followed by guide-wire insertion and subsequently Hickman line tunnelled in the anterior chest wall. Position of the wire and line tip are confirmed fluoroscopically. We assessed differences in radiation exposure between fluoroscopy room and surgical theatres.

Saturday

**Methods or Backround:** Radiation dose and screening time were recorded from all paediatric Hickman line insertions over a three year period. Inter-room and inter-operator data was analysed. Mann-Whitney U Test was used for statistical analysis.
**Results or Findings:** Three hundred and thirty seven Hickman lines were inserted between January 2020 and May 2023. Radiation doses were significantly lower in the fluoroscopy room than in theatre (p<0.0001), and this effect remained when data was analysed in age subgroups and controlling for operators. Screening times were significantly reduced in the fluoroscopy room compared to theatre (p<0.05). Lower radiation dose (p<0.001) and screening time (p<0.01) was also observed in radiologist-performed insertions compared with surgeon-performed insertions in theatre.
**Conclusion:** The location and operator of Hickman line insertions significantly impacts the radiation dose and screening time in the procedure. Even controlling for operator, patients were exposed to lower radiation doses in our fluoroscopy room than in theatres.
**Limitations:** Theatre cases may be more complex than patients suitable for fluoroscopy room. Of note, however, this study spans the COVID-19 pandemic, during which many ‘routine’ procedures ordinarily carried out in fluoroscopy were performed in theatre, which may somewhat mitigate this effect.
**Ethics committee approval:** This study was performed as internal service evaluation using pooled anonymised data, and hence no ethics approval was required.
**Funding for this study:** This was a student project. No specific funding was acquired.
**Author Disclosures:**
Sara Hosseinzadeh: Nothing to disclose

### RPS 1912-5

**Reducing radiation dose and contrast medium volume in paediatric spectral CT imaging: how is the second generation dual-layer CT?**
L. Ren[1], N. Guo[2], Y. Zhang[1], *Y. Wang*[1]; [1]Zhenzhou/CN, [2]Beijing/CN
*(13298363780@163.com)*

**Purpose:** The objective of this study was to explore the feasibility of reduced radiation dose and contrast medium using dual-layer spectral CT in pediatric patients.
**Methods or Backround:** This study has been approved by the ethics committee. One hundred and twelve peadiatric patients were enrolled in this study and randomly separated into two groups. The study group underwent scanning with a DRI index of 13. The contrast agent volume was reduced with 0.2 ml/kg compared to the control group. Fifty keV monoenergetic images were reconstructed. The control group underwent scanning with a DRI index of 16, and the contrast agent volume were 1.2 to 1.8 ml/kg based on patient’s weight. Hundred kVp polyenergetic images were reconstructed. CT values and standard deviations (SD) of the abdominal aorta, abdominal aorta branches, hepatic veins, portal vein main trunk, liver parenchyma and paraspinal muscles were measured. Subjective evaluations of overall image quality were performed. Statistical analysis was conducted with student t-tests and rank sum tests.
**Results or Findings:** There were no statistically significant differences in age and weight between two groups (P>0.05). The CTDIvol, SSDE, and DLP of the study group were lower than those of the control group (each P<0.05). The contrast medium volume of the study group was reduced by approximately 24.0% compared to the control group. CT values for vessels and organs in the study group were higher than those in the control group (P<0.001). There was no statistically significant difference in subjective image quality scores between two groups (P>0.05). Both groups had image quality sufficient for diagnostic requirements.
**Conclusion:** In paediatric patients, dual-layer spectral CT can significantly reduce radiation dose and iodine contrast medium volume of abdominal and pelvic spectral imaging without compromising image quality.
**Limitations:** This study is a single-center study.
**Ethics committee approval:** Not applicable
**Funding for this study:** No funding was obtained for this study.
**Author Disclosures:**
Ning Guo: Nothing to disclose
Yonggao Zhang: Nothing to disclose
Lichen Ren: Nothing to disclose
Yiran Wang: Nothing to disclose

### RPS 1912-6

**The effect of arm positioning on radiation dose and image quality during paediatric CT chest examinations**
*C. E. Kelly*, O. Arthurs, I. C. Simcock; London/UK
*(charliek86@hotmail.com)*

**Purpose:** The aim of this study was to identify the optimal arm position for paediatric CT chest scanning if patients cannot raise both arms alongside their head, considering image quality and radiation dose.
**Methods or Backround:** Positioning the arms outside of the field of view, (alongside the head) during CT chest scans reduces radiation dose and artifacts. However, some children are unable to achieve this optimal positioning due to physical disability or an inability to co-operate. In this study we measured the effect of sub-optimal arm positioning on absorbed patient dose and image quality. We evaluated 154 chest CT scans performed with arms in the suboptimal position between January 2022 and July 2023 (age range 4 days and 16 years old), compared to mean age-weighted CTDIvol for patients with arms up.
**Results or Findings:** The highest doses were seen when one or both arms were positioned perpendicular to the torso and this was commoner in younger patients. Overall, mean radiation dose was 125% higher with both arms perpendicular, 50% higher with one arm perpendicular, 88% higher with both arms down, and 63% higher with one arm down. All scans were of diagnostic quality, but streaking was present in 47% of scans when both arms were perpendicular, 24% of scans with one arm perpendicular and 14% when both arms were down alongside the body.
**Conclusion:** Sub-optimal positioning of the arms during paediatric CT chest scans increases patient dose and reduces image quality. Arms should be placed alongside the body if they cannot be raised, to reduce radiation dose and improve image quality.
**Limitations:** The range of weights meant absolute CTDIvol values could not be compared but instead were compared as a proportion of the optimum CTDIvol value.
**Ethics committee approval:** The study utilised retrospective anonymous data analysis.
**Funding for this study:** No funding was received for this study.
**Author Disclosures:**
Charlotte Emma Kelly: Nothing to disclose
Ian C. Simcock: Nothing to disclose
Owen Arthurs: Nothing to disclose

### RPS 1912-7

**Ushering into another dimension: diagnostic utility of low-dose four dimensional (4D) dynamic airway CT in paediatric stridor**
*S. Saini*, K. S. Sodhi, A. Bhatia, A. K. Saxena, J. Mathew; Chandigarh/IN
*(shubhsaini99@gmail.com)*

**Purpose:** Dynamic airway disorders are an important cause of stridor in children. Fiberoptic bronchoscopy (FOB) is the gold standard and an invasive method of diagnosis of these disorders. There is lack of objective criteria for diagnosis of paediatric dynamic airway disorders. The study aims to assess the diagnostic utility of low-dose 4D dynamic airway CT in paediatric stridor on comparison with FOB.
**Methods or Backround:** We conducted a prospective study in 24 children (age:13 days-1.5 years, boys: 18) with stridor. Low-dose 4D dynamic airway CT was performed with 70kVp and 15 mAs. The number of acquisition cycles varied from five to ten. Stridor during CT acquisition was also noted. Image analysis was done using volume rendered reconstruction and axial CT images and degree of collapse was noted. FOB was performed within 72 hours of CT with blinding of both the respective observers. The findings were compared with FOB as the gold standard.
**Results or Findings:** The diagnostic accuracy of 4D dynamic airway CT was 91.6%, 95.7%, and 95.5% for the diagnosis of laryngomalacia, tracheomalacia, and bronchomalacia respectively. The sensitivity and specificity of 4D dynamic airway CT for the diagnosis of laryngomalacia was 94.7% and 80.0% respectively with substantial agreement between the two modalities (k=0.777). Sensitivity for detection of tracheomalacia and bronchomalacia was 90.9% and 75% respectively with 100% specificity. There was near perfect agreement between the two modalities with kappa coefficient of 0.913 for tracheomalacia and 0.831 for bronchomalacia. Mean radiation dose was 0.827 mSv. The presence of stridor during CT had a positive association with prediction of abnormality detection on CT.
**Conclusion:** 4D dynamic airway CT can be used as a robust and non-invasive objective tool in assessing dynamic airway diseases in children.
**Limitations:** Not applicable for this study.
**Ethics committee approval:** This study was approved by institutional ethics committee.
**Funding for this study:** No funding was received for this study.
**Author Disclosures:**
Kushaljit Singh Sodhi: Nothing to disclose
Shubham Saini: Nothing to disclose
Joseph Mathew: Nothing to disclose
Anmol Bhatia: Nothing to disclose
Akshay Kumar Saxena: Nothing to disclose

**14:00-15:30** **Research Stage 1**

### Research Presentation Session: Artificial Intelligence & Machine Learning & Imaging Informatics

### RPS 2005

### Recent AI advancements in MRI for precision imaging

**Moderator**
M. El-Koussy; Berne/CH

### RPS 2005-2

**T2-weighted fat-saturated and PD-weighted contrast synthesis from knee MRF maps via deep-learning: clinical feasibility study**

*M. T. Nevalainen*[1], O. Nykänen[2], J. Järvinen[1], V. Casula[1], L. Räsänen[1], M. Nissi[2], M. T. Nieminen[1]; [1]Oulu/FI, [2]Kuopio/FI
*(mika.nevalainen@oulu.fi)*

**Purpose:** To compare the diagnostic performance of the magnetic resonance fingerprinting (MRF)-derived synthetic MR images of the knee against conventional MR images.
**Methods or Backround:** MRF is an emerging technique to rapidly produce quantitative MRI maps, but its clinical feasibility remains low. However, through deep-learning (DL) synthetic conventional contrasts can be now derived. In this single-center prospective study 78 subjects with knee osteoarthritis were scanned on 3T scanners with isotropic T2-weighted fat-saturated (T2w fs) and PD-weighted (PDw) sequences, and with sagittal MRF sequence. U-net's were trained to synthesize contrasts from the MRF raw data. Thirty nine cases (1014 images) were used for DL training/validation and 39 cases (1008 images) for diagnostic comparison. Two experienced fellowship-trained musculoskeletal radiologists performed the MOAKS grading and Likert scale assessment of image quality. The inter-rater and inter-method reliability were evaluated using Cohen's kappa and percentages of exact matches.
**Results or Findings:** The inter-rater reliability was 0.720 (CI 0.625-0.814) for synthetic images and 0.755 (CI 0.678-0.832) for conventional images. Varying inter-method reliability was observed between the synthetic and conventional images: for cartilage grades $\kappa$-values varied between 0.418-0.862 (mean 0.747; exact matches 85.9%), for bone marrow lesions between 0.196-0.945 (mean 0.637; exact matches 91.0%), for osteophytes between 0.701-0.885 (mean 0.729; exact matches 60.3%), for meniscus pathology between 0.505-0.782 (mean 0.621; exact matches 78.2%). For effusion and Baker's cyst $\kappa$-values were 0.868 and 0.846, and exact matches 71.8% and 92.3%, respectively. The average Likert scores were better for the conventional than synthetic images (4.6 vs 3.2 for T2w fs) and (4.8 vs. 3.9 for PDw).
**Conclusion:** The MRF-derived DL-based synthetic clinical contrasts provide good interchangeability with state-of-the-art conventional MR sequences; however, further development is needed to enhance the image quality.
**Limitations:** Small number of patients, only sagittal images.
**Ethics committee approval:** This study was approved by the regional medical research ethics committee of the Wellbeing services county of North Ostrobothnia
**Funding for this study:** The funding of this study was received from The Finnish Medical Foundation, The Finnish Cultural Foundation, The Terttu Foundation.
**Author Disclosures:**
Victor Casula: Nothing to disclose
Mika Tapani Nevalainen: Nothing to disclose
Jyri Järvinen: Nothing to disclose
Olli Nykänen: Nothing to disclose
Lasse Räsänen: Nothing to disclose
Mikko Nissi: Nothing to disclose
Miika Tapio Nieminen: Nothing to disclose

### RPS 2005-3

**Leveraging brain MRI and wearable sensor data for early detection of neurodegenerative diseases**

*J. Lian*, V. Vardhanabhuti; Hong Kong/HK
*(jlian@connect.hku.hk)*

**Purpose:** Neurodegenerative diseases, including Parkinson's disease (PD) and Alzheimer's disease (AD), pose a significant healthcare burden to the aging population. Timely detection of these diseases, even before symptom onset, is crucial for early intervention. Although prior studies have shown that wearable movement-tracking data holds promise as a PD biomarker, the potential of combining it with brain MRI for improved prediction remains unexplored. This study aimed to investigate the role of brain MRI as a biomarker for predicting neurodegenerative diseases, alongside accelerometry data, using the UK Biobank dataset.
**Methods or Backround:** This study comprises 19,793 participants with brain MRI scans (T1 and T2), accelerometry data, polygenic risk scores (PRS), and questionnaire-derived lifestyle information. In total, 56 participants were classified as positive cases if diagnosed with any of AD, other forms of dementia, or PD at least one year after the initial assessment. We preprocessed the brain MRI data by segmenting it into 144 segments (normalized by intracranial volume). Disease incidence prediction was employed using XGBoost models, with results reported using the Area Under the Receiver Operator Characteristic Curve (AUROC).
**Results or Findings:** The comprehensive model, which incorporated all modalities, achieved an AUROC of 0.760 on the test dataset. Among the top 20 predictive features, 17 were related to MRI data, pertaining to different regions in the basal ganglia (e.g., ventral striatum, amygdala), hippocampus, and white matter hyperintensities. In contrast, an ablation study with a model excluding MRI data only achieved an AUROC of 0.645. Other important features include average activity acceleration between 5-7 p.m., and duration of sleep.
**Conclusion:** Our findings suggest the potential of brain MRI in conjunction with activity tracking data as predictive biomarkers for early neurodegenerative disease detection.
**Limitations:** Positive cases were not large enough, causing imbalanced dataset.
**Ethics committee approval:** The study was approved by the author's institution's local ethics board (UW-20814) at the University of Hong Kong. The population cohort in this study was from the UK Biobank 20 [Application Number 78730] which received ethical approval from the North West Multicentre Research Ethics Committee (REC reference: 11/NW/03820). All participants gave written informed consent before enrollment.
**Funding for this study:** No funding was obtained for this study.
**Author Disclosures:**
Varut Vardhanabhuti: Nothing to disclose
Jie Lian: Nothing to disclose

### RPS 2005-4

**Synthesis of artificial T1w full-dose images using low doses of gadolinium-based contrast agents: a new deep neural network approach of contrast signal mapping**

*R. Haase*, T. Pinetz, E. Kobler, Z. Bendella, C. Gronemann, D. Paech, A. Effland, K. Deike, A. Radbruch; Bonn/DE

**Purpose:** The main objective of this study was to test a new DNN approach to synthesize artificial T1w-full-dose images from corresponding non-contrast and low-dose images and compare its performance with two reimplemented state-of-the-art approaches (referred to as setting-A and B).
**Methods or Backround:** Two hundred and thirteen participants received an MRI brain scan with an adapted imaging protocol including a T1w-low-dose after 20% of the standard dose of a gadolinium-based contrast agent. Fifty participants were randomly chosen as test set. The new approach is referred to as setting-C. Synthesized artificial T1w-full-dose images were assessed using a reader-based study. Two readers scored the overall image quality, the interchangeability with the true T1w-full-dose in regard to the clinical conclusion, the contrast enhancement of lesions and their conformity to the respective true reference lesions.
**Results or Findings:** The overall image quality was rated lower in setting-A than in the two remaining settings, whose image quality did not differ from each other and the true full-dose images. The average counts of false positives per case were 0.33±0.93, 0.07±0.33, and 0.05±0.22 for the settings A-C, respectively. The proportion of scans scored as fully or mostly interchangeable was significantly higher in setting-C (70%) than in settings A (40%) and B (57%). The contrast enhancement was significantly reduced in all settings compared to the original T1w-full-dose. Using a five-point Likert-scale, there was no significant difference between the contrast signal reduction of setting-A (-1.10±0.98) and setting-B (-0.91±0.67), but between both settings and setting-C (-0.50±0.55). The average scores of conformity were 1.75±1.07, 2.19±1.04, and 2.48±0.91 for settings A-C, respectively.
**Conclusion:** The new approach showed a significantly better qualitative performance than published alternatives. Nevertheless, a relevant proportion of cases with inadequate synthesis of the contrast signal remains using a low-dose of 20% of the standard dose.
**Limitations:** Monocentric study.
**Ethics committee approval:** This study was approved by the Ethics Committee for Clinical Trials on Humans and Epidemiological Research with Personal Data of the Faculty of Medicine of the Rheinische Friedrich-Wilhelms University Bonn: reference no. 450/20.
**Funding for this study:** R.H. is funded by a research grant (BONFOR; O-194.0002.1) of the Medical Faculty of the University of Bonn. A.E. and T.P. are funded by the German Research Foundation under Germany's Excellence Strategy (EXC-2047/1, 390685813 and EXC-2151, 390873048).

**Author Disclosures:**
Katerina Deike: Shareholder: relios.vision GmbH
Alexander Effland: Shareholder: relios.vision GmbH
Daniel Paech: Shareholder: relios.vision GmbH
Erich Kobler: Shareholder: relios.vision GmbH
Robert Haase: Shareholder: relios.vision GmbH
Zeynep Bendella: Nothing to disclose
Thomas Pinetz: Shareholder: relios.vision GmbH
Alexander Radbruch: Shareholder: relios.vision GmbH
Christian Gronemann: Nothing to disclose

### RPS 2005-5

**Brain age fingerprinting from MR image using multi-level information fusion networks and its application in cognitive impairment patient screening**

N. Zhao, Y. Pan, Z. Xue, F. Gao, *F. Shi*, D. Shen; Shanghai/CN
*(feng.shi@uii-ai.com)*

**Purpose:** The aim of this study was to develop a model for estimating brain age from MR image on a large-scale normal aging population covering entire lifespan, and to assess its potential for early screening of cognitive impairment patients.
**Methods or Backround:** We proposed a novel approach to build a brain age prediction model in lifespan datasets using T1-weighted MR images. This approach consists of extracting three-level hierarchical information through neural networks and fusing them with the cross-attention mechanism, to capture inherent brain age fingerprinting in MR images. Specifically, the hierarchical information included: (1) brain volumes and ratios of 106 parcellations derived from a pre-trained segmentation model, (2) 2D image slices selected from specific brain regions, which potentially contain brain lesion information such as white matter hypointensities, lacunes, and perivascular spaces, (3) 3D CNN features with input of MR image.
**Results or Findings:** This study included 3,711 subjects aged 6-96 years from in-house datasets, with 3,372 cognitively normal (CN), 207 late MCI (LMCI), and 132 AD. Based on the proposed model, CN subjects achieved a mean absolute error of 2.72 years. Furthermore, when applying this model to cognitively impaired subjects, AD group had higher brain age gap (BAG) compared to both LMCI and CN groups (4.43 vs. 2.47 vs. -0.5 years; P < .001). Finally, combing BAG with learned age-related features as inputs of multi-layer perceptron for differentiating between CN, LMCI, and AD yielded predictive accuracy of 91% for CN vs. LMCI, 91% for CN vs. AD, and 96% for LMCI vs. AD.
**Conclusion:** The BAG from prediction model appears to be highly correlated with cognitive impairment and could be used for screening of cognitive impairment patients.
**Limitations:** The model utilises 3D high-resolution images while the extension to clinically low-resolution MRI scans should be studied.
**Ethics committee approval:** This study was approved by Autism Brain Imaging Data Exchange (ABIDE), Attention Deficit Hyperactivity Disorder (ADHD-200), Alzheimer’s Disease Neuroimaging Initiative, Open Access Series of Imaging Studies (OASIS), Consortium for reliability and reproducibility (CoRR), Consortium of Chinese Brain Molecular and Functional Mapping, HUASHAN Hospital, and RENJI Hospital, China, approved this study.
**Funding for this study:** This work was supported in part by National Natural Science Foundation of China (62131015), Science and Technology Commission of Shanghai Municipality (STCSM) (21010502600), Key R&D Program of Guangdong Province, China (2021B0101420006), STI2030-Major Projects (2022ZD021 3100), The China Postdoctoral Science Foundation (Nos. BX2021333, 2021M703340), and National Key Research and Development Program of China (2022YFE02 05700). Data collection and sharing for this project was funded by Shanghai Zhangjiang National Innovation Demonstration Zone Special Funds for Major Projects (ZJ2018-ZD-012), Shanghai Pilot Program for Basic Research (JCYJ- SHFY-2022-014), and Shanghai Pujiang Program (21PJ1421400).
**Author Disclosures:**
Feng Shi: Nothing to disclose
Dinggang Shen: Nothing to disclose
Zhong Xue: Nothing to disclose
Fei Gao: Nothing to disclose
Nan Zhao: Nothing to disclose
Yongsheng Pan: Nothing to disclose

### RPS 2005-6

**A fully automated approach for contrast-agent-free myocardial tissue characterisation using T1-rho mapping**

*V. De Villedon De Naide*[1], K. Narceau[1], N. Brillet[1], V. Nogues[1], J. H. Zhang[2], M. Villegas-Martinez[1], M. Stuber[2], H. Cochet[1], A. Bustin[1]; [1]Pessac/FR, [2]Lausanne/CH
*(victor.de-villedon@ihu-liryc.fr)*

**Purpose:** Myocardial T1-rho mapping is a promising biomarker, allowing for contrast-agent-free myocardial injury detection and quantification. However, its operator-dependant processing induces operator variability and radiologist workload rise. The aim of this study was to explore the feasibility of artificial intelligence-driven analysis for efficient and automated myocardial T1-rho mapping.
**Methods or Backround:** A cohort of 573 patients presenting various cardiomyopathies was divided into a training set (n=500) and a distinct test set (n=73). CMR imaging was conducted using a 1.5T Siemens Aera scanner. For each patient, pre-contrast breath-held 3-slice T1-rho mapping was performed, and contrast-enhanced LGE images were acquired in short-axis 12min post-injection of gadolinium. For each patient, a transformer-based model automatically segmented the left ventricular wall on T1-rho images. Then, the right-ventricle insertion points were detected using a U-Net. A 16-segment AHA model was then created for segmental T1-rho values analysis. Segmentation quality was assessed. T1-rho values were quantitatively retrieved for both manual and automated processing across patient, slice and segment levels. The concordance between methods was gauged. Processing times were measured.
**Results or Findings:** Automated processing of the T1-rho slices revealed a reduced processing time (~3 s vs. 1 min 51 s±22 s) in comparison to manual processing. Automated segmentation quality yielded favourable results (global DICE of 81.9±9.0%). Analysis of T1-rho values indicated no difference between manual and automated processing (54.9±4.6 ms vs. 55.4±5.1 ms, P=0.098). Strong correlations (ICC>0.8) were found with minimal biases at patient and slice levels, while agreement was lower at the segment level. All AHA segments did not differ significantly between manual and automated T1-rho measurements.
**Conclusion:** Automated processing of myocardial T1-rho maps shows strong agreement with manual processing and comparable segmentation quality with enhanced time efficiency, highlighting its promising clinical application.
**Limitations:** Further clinical studies in various cardiomyopathies are warranted.
**Ethics committee approval:** Not applicable
**Funding for this study:** This project was supported by funding from the French National Research Agency under grant agreements Equipex MUSIC ANR-11-EQPX-0030, ANR-22-CPJ2-0009-01, ANR-21-CE17-0034-01, Programme d’Investissements d’Avenir ANR-10-IAHU04-LIRYC. This project has received funding from the European Research Council (ERC) under the European Union’s Horizon 2020 research and innovation programme (grant agreement No101076351).
**Author Disclosures:**
Victor De Villedon De Naide: Nothing to disclose
Aurelien Bustin: Nothing to disclose
Hubert Cochet: Nothing to disclose
Nina Brillet: Nothing to disclose
Kalvin Narceau: Nothing to disclose
Jana Huiyue Zhang: Nothing to disclose
Manuel Villegas-Martinez: Nothing to disclose
Victor Nogues: Nothing to disclose
Matthias Stuber: Nothing to disclose

### RPS 2005-7

**Predicting Alzheimer’s progression from mild cognitive impairment: a generalised approach integrating automated MRI segmentation and longitudinal atrophy analysis**

J. Steiglechner, B. Bender, G. Lohmann, K. Scheffler, U. Ernemann, *T. Lindig*; Tübingen/DE
*(tobias.lindig@airamed.de)*

**Purpose:** This study sought to formulate a generalised classifier for differentiating progressive mild cognitive impairment (pMCI) from stable MCI (sMCI) by integrating brain MRI segmentation and longitudinal atrophy analysis, essential for early Alzheimer's disease (AD) intervention.
**Methods or Backround:** Utilising a segmentation model [AIRAmed] on 3D T1w high-field MRI, which labels 30 anatomical regions, we generated z-statistics through age- and sex-adjusted comparisons with a reference cohort concerning total intracranial volume (TIV) measures. Atrophy rates were quantified using a time-scaled approach. Z-statistics and atrophy rates were inputted into a classifier trained via an 80:20 train-test partition of the Alzheimer’s disease national initiative (ADNI) using logistic regression to distinguish pMCI from sMCI.

**Results or Findings:** Model generalisation was evaluated on four cohorts (retained test set of ADNI (53 pMCI/93 sMCI), Australian Imaging Biomarkers and Lifestyle Flagship Study of Ageing [AIBL] (15/42), Minimal Interval Resonance Imaging in Alzheimer's Disease [MIRIAD] (4/8), and Open Access Series of Imaging Studies [OASIS] (27/80)) achieving ROC AUCs of 0.86, 0.78, 0.90, and 0.85 respectively. Including atrophy rates improved overall performance from AUC 0.826 to 0.847, enhancing specificity from 70.4% to 75.8%.
**Conclusion:** This research underscores the efficacy of leveraging both static (volumetric measures) and dynamic (atrophy rate) features to predict MCI to AD progression. The novel biomarker introduced, combining MRI segmentation and longitudinal atrophy evaluation, not only presents high sensitivity and specificity, as validated by AUC ROC across various datasets but also emerges as a robust tool applicable in future research and diagnostic assistance.
**Limitations:** Limitations are not applicable for this study.
**Ethics committee approval:** This study used anonymised data.
**Funding for this study:** No funding was provided for this study.
**Author Disclosures:**
Tobias Lindig: Founder: AIRAmed; CEO: AIRAmed; Shareholder: AIRAmed
Julius Steiglechner: Employee: AIRAmed
Benjamin Bender: Founder: AIRAmed; Shareholder: AIRAmed
Klaus Scheffler: Nothing to disclose
Gabriele Lohmann: Nothing to disclose
Ulrike Ernemann: Founder: AIRAmed; Shareholder: AIRAmed

### RPS 2005-8

**Towards precision diagnosis: machine learning in identifying malignant orbital tumours with multiparametric 3 Tesla MRI**
*E. O'Shaughnessy*; Paris/FR

**Purpose:** Orbital tumours pose a diagnostic challenge due to their diverse locations and histopathological variations. In recent years, advancements in imaging have enhanced diagnosis, but classification remains challenging. The application of artificial intelligence in radiology and ophthalmology has shown promising results. The primary aim of this study was to develop and assess the performance of machine learning in accurately identifying malignant orbital tumors based on multiparametric 3 Tesla MRI data.
**Methods or Backround:** This single-center prospective study enrolled patients with orbital masses who underwent pre-surgery 3 Tesla MRI scans between December 2015 and May 2021. We employed a Random Forest model with nested stratified cross-validation, considering various combinations of explanatory features. SHAP (SHapley Additive exPlanations) values have been used to evaluate feature contributions, and multiple metrics assessed model performance.
**Results or Findings:** We analysed 113 patients (50.4% female, 49.6% male) with an average age of 51.5 [19-88]. Among the eight machine learning models evaluated, the one that incorporated all 46 explanatory features (morphology, DWI, DCE, and IVIM) achieved an AUC of 0.9 [0.73-0.99], while the "10-features signature" model attained an AUC of 0.88 [0.71-0.99]. The ten most influential features for the Random Forest model included three quantitative IVIM features, four quantitative DCE features, one quantitative DWI feature, one qualitative DWI feature, and age.
**Conclusion:** This study suggests that machine learning, when combining multiparametric MRI data, including DCE, DWI, and IVIM, can yield highly effective models for classifying orbital tumors. The "10-features signature" model may be preferred due to its strong performance, simplicity, and adherence to the parsimony principle.
**Limitations:** The study's limitations encompass its single-center design and the relatively small number of subjects.
**Ethics committee approval:** This study was approved by an Institutional Research Ethics Board and adhering to Declaration of Helsinki.
**Funding for this study:** This study was publicly funded.
**Author Disclosures:**
Emma O'Shaughnessy: Nothing to disclose

### RPS 2005-9

**Improving whole body MRI in multiple myeloma: reduced acquisition time with a deep learning reconstruction for diffusion-weighted imaging at 3 Tesla-preliminary results**
*J. Herrmann*, S. Gassenmaier, S. Ursprung, H. Almansour, S. Werner, S. Afat; Tübingen/DE

**Purpose:** The evaluation of bone disease in multiple myeloma (MM) is an important topic in oncologic imaging. The objective was to determine the impact of a Deep Learning (DL)-reconstruction for whole body (WB) diffusion-weighted-imaging (DWI) for staging MM patients at 3 Tesla compared to standard DWI on reducing acquisition time and improving image quality.
**Methods or Backround:** Thirty patients (mean age, 61±11 years; range, 35–82; 16 men, 14 women) were consecutively included in this retrospective, monocentric study between February and August 2023. Inclusion criteria were standard DWI (DWI_S) in clinically indicated MRI at 3 Tesla, and DL-reconstructed WB-DWI (DWI_DL). All patients were examined using the institution's standard MRI protocol for staging MM including DWI with two different b-values (0 s/mm² and 800 s/mm²) and calculation of apparent diffusion coefficient (ADC) maps. Image quality was qualitatively assessed by two radiologists using a visual 5-point Likert scale (5=best).
**Results or Findings:** The overall image quality was evaluated to be significantly superior in DWI_DL as compared to DWI_S for b=0 s/mm², b=800 s/mm², and ADC maps by all readers (p<0.05). The extent of noise was evaluated to be significantly less in DWI_DL as compared to DWI_S for b=0 s/mm², b=800 s/mm², and ADC maps by all readers (p<0.001). No significant differences were found regarding artifacts, lesion detectability, sharpness of organs, and diagnostic confidence (p>0.05). Acquisition time for DWI_S was 7:45 min and simulated acquisition time for DWI_DL was 5:03 min.
**Conclusion:** DWI_DL enhances image quality for WB-MRI in staging of multiple myeloma patients at 3 Tesla. Simulation results suggest a potential reduction in acquisition time of 35 %, highlighting the promise of DL in advancing clinical efficiency.
**Limitations:** Priliminary results of this study with a small sample size and retrospective design.
**Ethics committee approval:** This study was approved by the University of Tuebingen
**Funding for this study:** No funding was obtained for this study.
**Author Disclosures:**
Judith Herrmann: Nothing to disclose
Saif Afat: Nothing to disclose
Haidara Almansour: Nothing to disclose
Stephan Ursprung: Nothing to disclose
Sebastian Gassenmaier: Nothing to disclose
Sebastian Werner: Nothing to disclose

### RPS 2005-10

**Prediction of therapy response of breast cancer patients with machine-learning based on clinical data and imaging data derived from breast [18F]FDG-PET/MRI**
*K. Jannusch*[1], H. A. Peters[1], N-M. Bruckmann[1], J. Morawitz[1], F. Dietzel[1], L. Umutlu[2], G. Antoch[1], J. Kirchner[1], J. Caspers[1]; [1]Düsseldorf/DE, [2]Essen/DE
*(kai.jannusch@med.uni-duesseldorf.de)*

**Purpose:** The objective of this study was to evaluate if a machine-learning prediction model based on clinical- and imaging features derived from baseline breast [18F]FDG-PET/MRI staging can predict pathologic complete response (pCR) in patients with newly diagnosed breast cancer prior to neoadjuvant systemic therapy (NAST).
**Methods or Backround:** This study retrospectively enrolled 143 women with newly diagnosed breast cancer. All women underwent a breast [18F]FDG-PET/MRI, histopathological workup of their breast cancer lesions, and evaluation of clinical data. Fifty-six features derived from PET, MRI, sociodemographic/anthropometric, histopathologic, and clinical data were generated and used for input into an extreme-Gradient-Boosting model (XGBoost) to predict pCR. The model was evaluated in a five-fold nested-cross-validation and reduced the risk of overoptimistic estimations. Diagnostic model-performance was assessed by determining ROC-AUC, sensitivity, specificity, PPV, and NPV. Feature importances of XGboost were evaluated to assess which features contributed most to distinguish between pCR and non-pCR.
**Results or Findings:** Nested-cross-validation yielded a mean ROC-AUC of 80.4±6.0% for prediction of pCR. Mean sensitivity, -specificity, -PPV and -NPV of 54.5±21.3%, 83.6±4.2%, 63.6±8.5% and 77.6±8.1% could be achieved. Histopathological data were the most important features for classification of the XGBoost-model followed by PET-, MRI-, and sociodemographic/anthropometric features.
**Conclusion:** The evaluated multi-source XGBoost model shows promising results for reliably defining pCR in breast cancer patients prior to NAST. However, the yielded performance is yet insufficient for the algorithm to be implemented in the clinical decision-making process.
**Limitations:** Inhomogeneity, especially with regard to the histopathological characteristics or NAST therapy regimes might be one limitation. Nonetheless, the risk of inhomogeneity is consistent with the clinical reality of breast cancer patients and underlines the need for a large number of patients to be included in a machine-learning based approach. Thus, as many combinations as possible can be learned by the model.
**Ethics committee approval:** All procedures performed were in accordance with the ethical standards of the institutional research committee and with the principles of the 1964 Declaration of Helsinki and its later amendments. University Duisburg-Essen (study number 17-7396-B0) and University Düsseldorf (study number 6040R).

**Funding for this study:** The study has been funded by Deutsche Forschungsgemeinschaft (DFG), the German Research Foundation (DFG (BU3075/2-1 and KI2434/1-2).
**Author Disclosures:**
Helena Anne Peters: Nothing to disclose
Julian Caspers: Nothing to disclose
Frederic Dietzel: Nothing to disclose
Lale Umutlu: Nothing to disclose
Julian Kirchner: Nothing to disclose
Gerald Antoch: Nothing to disclose
Janna Morawitz: Nothing to disclose
Kai Jannusch: Nothing to disclose
Nils-Martin Bruckmann: Nothing to disclose

### RPS 2005-11

**Trading speed for certainty: artefacts and non-inferiority in AI-accelerated FLAIR imaging of the brain**

*M. A. Mutke*, T. Rusche, A. Lonak, K. Blackham, M-N. Psychogios, J. M. Lieb; Basle/CH
*(matthias.mutke@usb.ch)*

**Purpose:** AI-enhanced MRI sequences promise improved image resolution at reduced acquisition times but are often proprietary products, with limited evaluation. The goal of this study was to investigate whether an AI-enhanced FLAIR sequence of the brain is non-inferior to a standard sequence, as assessed qualitatively by experienced human radiologists at both general and individual levels.
**Methods or Backround:** Patients underwent both standard FLAIR-sequence and an AI-enhanced FLAIR product sequence with reduced acquisition time of 40% (deep-resolve-boost, Siemens Healthineers), post contrast injection on 1.5T and 3T MRIs. Two experienced neuroradiologists conducted a side-by-side comparison. Using a five-point Likert scale (ranging from non-diagnostic to excellent), images were assessed for signal-to-noise ratio, anatomic clarity, overall quality, imaging artefacts, and diagnostic confidence. Potential lesions (pseudolesions) unique to one sequence and not readily identifiable as artifacts were noted and compared (McNemar's test). A mixed-model analysis determined the sequence type's effect on ratings, adjusting for rater and patient variances. Non-inferiority was tested (lower bound of the confidence interval not exceeding -0.5 points).
**Results or Findings:** Thirty patients were assessed for cerebral metastases (n=11), glioma (n=9), meningioma (n=5) and other pathologies (n=5). In the mixed model, AI sequences significantly outperformed by 0.4 points (Likert scale) in anatomic clarity but underperformed by -0.5 points for artifacts, failing to meet non-inferiority. In all other categories, the AI sequence matched the standard. Both readers identified pseudolesions on a total of 5/30 patients.
**Conclusion:** The faster, AI-enhanced FLAIR sequence is non-inferior to the standard except for an increase in imaging artifacts. These artefacts can result in pseudolesions, potentially causing unnecessary follow-up imaging. We emphasize the need for critical and rigorous evaluation of new sequences and suggest a training phase for radiologists.
**Limitations:** Small sample size, single-centre with qualitative, subjective evaluation limit this study.
**Ethics committee approval:** The imaging was part of a quality control program, informed consent was waived by the local ethics committee.
**Funding for this study:** No funding was received for this study.
**Author Disclosures:**
Annika Lonak: Nothing to disclose
Matthias Anthony Mutke: Nothing to disclose
Marios-Nikos Psychogios: Nothing to disclose
Thilo Rusche: Nothing to disclose
Johanna Maria Lieb: Nothing to disclose
Kristine Blackham: Nothing to disclose

| **14:00-15:30** | **Research Stage 2** |
|---|---|

### Research Presentation Session: Breast

### RPS 2002

### Advances in breast intervention

**Moderator**
T. Sella; Jerusalem/IL
*(tamarse@hadassah.org.il)*

### RPS 2002-2

**Clinical pilot study using polygenetic risk score for personalised risk-based breast cancer screening**

*T. Hovda*[1], K. K. Sahlberg[1], S. Sõber[2], P. Padrik[2]; [1]Drammen/NO, [2]Tartu/EE
*(thovda@hotmail.com)*

**Purpose:** This study aimed to explore the potential for polygenetic risk score (PRS) as a tool for risk-based mammographic screening.
**Methods or Backround:** Population-based screening for breast cancer is mainly age-based. This pilot study included 30 women aged 40-50 without prior history of breast cancer, premalignant breast disease or prior genetic testing/counselling. We used a CE-marked clinical test (AnteBC, Antegenes, Estonia) to assess participants' PRS, and calculate absolute and relative 10-year breast cancer risk. Women were recommended mammographic screening based on the results; standard biennial screening age 50-69 if below average or medium level risk, or earlier start of biennial and/or annual screening if moderate (up to 2x increased) or high (≥2x increased) risk. We also collected information on breast density and family cancer history, and referred the women for further genetic testing for monogenetic pathogenic variants if indicated by family history.
**Results or Findings:** Mean age was 44.9 years (SD 3.0). Mean absolute 10-year-risk for breast cancer was 1.65% (95% confidence interval [CI] 1.35, 1.91), compared to 1.74% (95% CI 1.63, 1.86) for an average population with the same age distribution. Relative risk ≤1 was observed in 70% (21/30), relative risk 1-2 was observed in 27% (8/30) and relative risk ≥2 was observed in 3% (1/30). We observed no statistically significant difference in relative risk by breast density. Five participants (17%) were recommended further genetic testing due to positive family history.
**Conclusion:** Polygenetic risk score may be a useful tool for risk-based mammographic screening. Further studies with larger study populations, exploring cost-effectiveness and women's perspective are needed.
**Limitations:** The small sample size is a limitation, as well as exclusion of women with prior malignant/premalignant breast disease and prior genetic testing, as the results might be biased towards less risk.
**Ethics committee approval:** The study was approved by the regional ethical committee with approval number: REK 494936.
**Funding for this study:** The AnteNOR consortium is sponsoring the study through a grant from the Norwegian Grants Green ICT program.
**Author Disclosures:**
Peeter Padrik: Shareholder: Antegenes
Tone Hovda: Nothing to disclose
Siim Sõber: Shareholder: Antegenes
Kristine Kleivi Sahlberg: Nothing to disclose

### RPS 2002-3

**Contrast-enhanced spectral mammography (CESM) vs MRI-guided vacuum-assisted breast biopsy: towards a reduced number of unnecessary biopsies**

*M. M. Nadrljanski*, I. B. Krušac, D. Dimitrijevic, L. Raspopovic, A. Djajic, M. Mihajlović; Belgrade/RS
*(dr.m.nadrljanski@gmail.com)*

**Purpose:** The study aimed to assess contrast-enhanced spectral mammography (CESM) guided breast vacuum-assisted biopsy (VAB), compared to MRI-guided VAB in benign lesion biopsy reduction.
**Methods or Backround:** Consecutive 23 patients with suspicious findings on mammography or digital breast tomosynthesis (n=23; MX-BI-RADS 4), were included prospectively (October 2022 – October 2023), for CESM-guided or MRI-guided VAB. All patients were assessed with CESM. All enhancing lesions were subsequently biopsied with CESM-guided VAB. Non-enhancing lesions were assessed with DCE-MRI (1.5 T/3 T) and the non-mass enhancement (NME) lesions were biopsied with MR-VAB. Non-enhancing lesions on CESM and MRI were downgraded to MRI-BI-RADS 3. Histopathologic correlation was performed for all lesions.

**Results or Findings:** There were 11 CESM-enhancing lesions (n1=11, 47.83%) and 12 CESM-non-enhancing lesions assessed with DCE-MRI (n2=12, 52.17%). In n1, there were eight malignant lesions (B5, 72.73%: 3 IDC, 1 ILC, 4 DCIS) and three benign lesions (B2, 27.27%). In n2, there were nine NME and three non-enhancing lesions. All nine lesions in n2 were benign (B2, 44.44%: 4 FCC and B3, 55.56%: 2 ADH, 3 FEA). CESM-guided VAB achieved Se=100% (63.1-100.0%); Sp=80% (51.9-95.7%) with NPV 100% and accuracy of 86.9%. MRI-guided VAB in CESM-non-enhancing MRI-NME lesions achieved Se=100% (47.8-100%); Sp=42.9% (9.9-81.6%) and accuracy of 66.7%.
**Conclusion:** CESM-guided VAB identified all lesions subsequently histopathologically confirmed as malignant. All non-enhancing lesions on CESM (and MRI) were benign. All CESM non-enhancing lesions detectable as NME lesions on MRI, were benign. The absence of enhancement on CESM favours benign lesions. Should larger trials confirm the preliminary findings, CESM-guided VAB may considerably reduce the number of unnecessary biopsies.
**Limitations:** The limited number of patients (due to the recently introduced method) was an identified limitation.
**Ethics committee approval:** The study was approved with approval code: 500-01-1335 CESM 03.
Based on the above stated decision, the authorisation was granted for the realisation of the procedure(s).
**Funding for this study:** No funding was received for this study.
**Author Disclosures:**
Iva B. Krušac: Nothing to disclose
Dejan Dimitrijevic: Nothing to disclose
Marko Mihajlović: Nothing to disclose
Andjela Djajic: Nothing to disclose
Mirjan M. Nadrljanski: Nothing to disclose
Luka Raspopovic: Nothing to disclose

### RPS 2002-4

### Diagnostic performance of Kaiser score in BI-RADS 3 lesions: a promising tool to reduce unnecessary biopsies

E. Ostillio, *P. Costantini*, D. Razzini, L. Groenhoff, A. Tambasco, M. Brambilla, A. Gambaro, A. Carriero; Novara/IT
*(pietro.costantini.md@gmail.com)*

**Purpose:** To determine whether the Kaiser score can be a valuable tool for radiologists in assessing whether to recommend a biopsy for a BI-RADS 3 lesion.
**Methods or Backround:** Notably, BI-RADS 3 denotes considerable uncertainty in breast nodule diagnoses, situated between benign (BI-RADS 2) and malignant (BI-RADS 4) classifications. The Kaiser score, a magnetic resonance imaging (MRI)-based algorithm developed by Bazar et al., has proven effective for BI-RADS 4 and 5, but its suitability for BI-RADS 3 remains unexplored. Between January 2016 and August 2023, 100 BI-RADS 3 lesions were studied, excluding 21 patients who did not meet the criteria. These 79 remaining lesions, observed in adult patients with no prior biopsies or treatments, underwent MRI-guided biopsies. Two expert radiologists (with 30 and 25 years of experience) applied the Kaiser score, and the results were compared with histopathological reports.
**Results or Findings:** Among the biopsied lesions (n=79), 14 were positive, and 65 were negative. Comparing Kaiser score results (Kaiser score: 1-4 = negative; 5-11 = positive) with biopsy findings, the Kaiser score exhibited a sensitivity of 100% (95% CI: 73%-100%) and specificity of 100% (95% CI: 93%-100%).
**Conclusion:** The strong concordance between the Kaiser score and biopsy outcomes for BI-RADS 3 lesions suggests its potential value in clinical practice for guiding patients towards follow-up or biopsy decisions.
**Limitations:** This was a single-centre study and, even if it is of a modest number, the population sample could be bigger. Moreover, two expert radiologists analysed the breast lesions through the KS together, after a common agreement, thus impeding us to estimate the KS interobserver agreement.
**Ethics committee approval:** As it is a retrospective study, no ethics committee approval was required at our institution.
**Funding for this study:** Funding was received from the Fondo di Ateneo per la Ricerca - University of Eastern Piedmont.
**Author Disclosures:**
Marco Brambilla: Author: No financial relationships with any pharmaceutical companies, biomedical device manufacturers or other corporations
Léon Groenhoff: Author: No financial relationships with any pharmaceutical companies, biomedical device manufacturers or other corporations
Pietro Costantini: Author: No financial relationships with any pharmaceutical companies, biomedical device manufacturers or other corporations
Anna Gambaro: Author: No financial relationships with any pharmaceutical companies, biomedical device manufacturers or other corporations
Alessandro Carriero: Author: No financial relationships with any pharmaceutical companies, biomedical device manufacturers or other corporations
Davide Razzini: Author: No financial relationships with any pharmaceutical companies, biomedical device manufacturers or other corporations
Anna Tambasco: Author: No financial relationships with any pharmaceutical companies, biomedical device manufacturers or other corporations
Eleonora Ostillio: Author: No financial relationships with any pharmaceutical companies, biomedical device manufacturers or other corporations

### RPS 2002-5

### Optimising assessment and reducing benign biopsy rate in screen-recalled women

*E. U. Ekpo*[1], I. Hadadi[2], M. F. McEntee[3]; [1]Sydney/AU, [2]Abha/SA, [3]Cork/IE
*(ernest.ekpo@sydney.edu.au)*

**Purpose:** The study aimed to optimise the assessment of women recalled at screening and reduce benign biopsy rate.
**Methods or Backround:** We examined the assessment data of 538 women recalled at screening whose mammograms showed features suggestive of breast cancer. Using histopathology results as a reference standard, we examined the diagnostic performance of digital breast tomosynthesis (DBT) and ultrasound as assessment tools. Relative risk analysis and McNemar tests were used to assess and compare the potential for these assessment tools to reduce benign biopsy rates.
**Results or Findings:** The recall rate was higher in dense breasts. DBT demonstrated higher sensitivity than ultrasound in dense breasts (98.2% versus 80%; p<0.001), but lower specificity (15.4% versus 55%; p<0.001), positive predictive value (PPV) (61.3% versus 71%; p=0.04), and area under the receiver operating characteristic curve (AUC) (0.57 versus 0.67; p=0.001). DBT showed higher sensitivity than ultrasound (99.2% versus 84%; p<0.001) in non-dense breasts, but no differences were observed in specificity, PPV, and AUC. Calcifications were easily detected but overestimated on DBT relative to ultrasound. Ultrasound reduced the benign biopsy rate compared to DBT: BI-RADS A (21% versus 5%; p=0.04); BI-RADS B (23% versus 10%; p=0.003); BI-RADS C (34% versus 7%; p<0.001) and BI-RADS D (39% versus 9%; p<0.001). The number needed to assess to prevent one benign biopsy was significantly lower with ultrasound than DBT in dense breasts: BI-RADS C (1.8 versus 7; p<0.001) and BI-RADS D (1.9 versus 5.1; p=0.03).
**Conclusion:** In women with dense breasts, DBT has higher sensitivity, but lower specificity and PPV than ultrasound. Compared to DBT, ultrasound reduces the benign biopsy rate for all women.
**Limitations:** Data were from one facility and 60% of the sample was dense breasts. Also, DBT and ultrasound images were interpreted with knowledge of mammography findings.
**Ethics committee approval:** This study was approved by: Sydney Local Health District; Royal Prince Alfred Hospital Human Research Ethics Committee.
**Funding for this study:** This study was supported by Tour De Cure mid-career funding; University of Sydney, Faculty of Medicine and Health EMCR Emerging Star funding.
**Author Disclosures:**
Mark F. McEntee: Nothing to disclose
Ibrahim Hadadi: Nothing to disclose
Ernest Usang Ekpo: Nothing to disclose

### RPS 2002-6

### The ICE Study: to detect cryoimmunologic response induced by ultrasound-guided cryoablation on early breast cancer: preliminary results

*F. Galati*, M. Pasculli, V. Rizzo, G. Moffa, C. Napoletano, B. Cerbelli, C. Catalano, F. Pediconi; Rome/IT
*(francesca.galati@uniroma1.it)*

**Purpose:** The ICE Study is a pilot, prospective, case-control study with the primary aim of characterising the inflammatory response induced by ultrasound-guided tumour cryoablation, in blood samples from breast cancer (BC) patients. Secondary endpoints are the evaluation of cryoablation efficacy and safety, and imaging prediction of cryoablation effectiveness.
**Methods or Backround:** We enrolled patients with an early-stage BC, scheduled for breast surgery, not eligible for neo-adjuvant therapy and with a cryo-feasible cancer location. Patients enrolled in the cryo-group and in the control-group followed the same therapeutic pathway for the treatment of BC, in terms of blood sampling and surgery. However, the control-group did not undergo cryoablation.
**Results or Findings:** From July 2022 we enrolled 10 women in the cryo-group and 10 women in the control-group. On the surgical samples of the cryo-group, ultrasound-guided cryoablation caused a steatonecrotic area in all the patients and the treatment was complete in 9 out of 10 patients.

Cryoablation success was evaluated with magnetic resonance imaging (MRI) in 5 patients, with contrast enhanced mammography (CEM) in 4 patients and with breast ultrasound in 1 patient who refused contrast-enhanced imaging. MRI and CEM predicted cryoablation efficacy in 9 out of 9 cases. Regarding circulating markers of cryo-immunological response, the analysis of blood samples revealed a release of HGBM1, which acts as a key mediator of the immune system signalling cellular stress, in both control and cryo-groups. Furthermore, in the cryo-group increased levels of HGBM1 were associated with a significant rise in CD3+ T cells.
**Conclusion:** Cryoablation is safe, effective and has a role in immune system modulation. Enhanced imaging (MRI and CEM) can predict procedure success.
**Limitations:** The limited patient population was an identified limitation.
**Ethics committee approval:** The study obtained the approval of the Institutional Review Board of Sapienza University of Rome (reference: 6528, approved 24.11.2021).
**Funding for this study:** "The ICE Study" received funding from the Seed Grant funding programme of the European Society of Radiology (ESR) in collaboration with the European Institute for Biomedical Imaging Research (EIBIR), kindly supported by an unrestricted, non-exclusive grant from GE Healthcare.
**Author Disclosures:**
Giuliana Moffa: Nothing to disclose
Veronica Rizzo: Nothing to disclose
Francesca Galati: Nothing to disclose
Marcella Pasculli: Nothing to disclose
Bruna Cerbelli: Nothing to disclose
Federica Pediconi: Nothing to disclose
Carlo Catalano: Nothing to disclose
Chiara Napoletano: Nothing to disclose

### RPS 2002-7

### Role of vacuum assisted excision in treatment of ductal carcinoma in situ: preliminary results of a monocentric prospective pilot study

*S. Carriero*, L. Nicosia, A. C. Bozzini, F. Pesapane, A. Latronico, M. Pizzamiglio, E. Cassano; Milan/IT
*(serena.carriero@gmail.com)*

**Purpose:** The study aimed to evaluate effectiveness, safety and underestimation of vacuum assisted excision (VAE) compared to surgery in patients with low- and intermediate-grade ductal carcinoma in situ(DCIS).
**Methods or Backround:** From April 2023 to October 2023, all patients with a single cluster of microcalcifications (BI-RADS >3) <15 mm were enrolled. The goal in these patients was to perform VAE of the cluster. The procedure was done using an 8-gauge needle in two stages: first, eight samples were taken with macroscopic excision of the focus, then 4 "cleaning" samples were taken. The samples were categorised into three groups: micro-positive sample (MPS), micro-negative sample (MNS), and cleaning sample (CS). In all cases, the presence of any residual disease within the cleaning area and the rate of post-procedural complications were assessed. A reference clip was always placed at the end of the procedure. All lesions underwent surgery. After surgery, the presence of any residual disease in the surgical specimen was evaluated.
**Results or Findings:** A total of 98 patients with microcalcifications were prospectively enrolled, resulting in 49 DCI. Seven patients underwent VAE. In 100% of cases (7/7), the underestimation rate was 0%. In 57% (4/7) of cases, no residual disease was found within the cleaning area, and no in-situ pathology was detected in the subsequent surgery. In 43% (3/7), disease was found within the cleaning area, and foci of DCIS were there at surgery. No major post-procedural complications were observed.
**Conclusion:** Preliminary data have shown that VAE is a safe procedure with no diagnostic underestimation, which could be used in the excision of DCIS as an alternative to surgery, especially in cases where the "cleaning" area is free of DCIS.
**Limitations:** The major limitation of our study is the limited sample size of the patients
**Ethics committee approval:** The approval for this monocentric prospective study was obtained by the Ethics Committee of IRCCS IEO, Milano.
**Funding for this study:** This research received no external funding.
**Author Disclosures:**
Luca Nicosia: Nothing to disclose
Antuono Latronico: Nothing to disclose
Anna Carla Bozzini: Nothing to disclose
Maria Pizzamiglio: Nothing to disclose
Serena Carriero: Nothing to disclose
Filippo Pesapane: Nothing to disclose
Enrico Cassano: Nothing to disclose

### RPS 2002-8

### Cancer detection in relation to type and stage in the randomised Mammography Screening with Artificial Intelligence trial (MASAI)

V. Hernström, V. Josefsson, H. Sartor, D. Schmidt, A-M. Larsson, I. Andersson, A. Rosso, O. Hagberg, *K. Lang*; Malmö/SE

**Purpose:** To analyze cancer-detection rates and types of detected cancers in the full MASAI-trial study population.
**Methods or Backround:** 105,934 screening participants were randomised (1:1) to AI-supported screening or double-reading without AI (Standard of Care, SoC). In the intervention arm, screening examinations were triaged to low- and high-risk groups based on AI-derived risk scores (ScreenPoint, Transpara v 1.7 score 1–9 and 10, respectively). Low-risk examinations were single read and high-risk examinations double read. Computer-aided detection marks were available for examinations with risk score 8–10. A per protocol analysis on cancer detection in relation to subtype and stage was performed and cancer-detection rates were compared using the Fisher's exact test with 95% confidence intervals (CI). ClinicalTrials.gov number NCT04838756.
**Results or Findings:** 53,043 women were allocated to AI-supported screening and 52,872 to SoC. Mean age was 55.1 (SD 10.2) in both arms. 338 cancers were detected with AI-support and 263 with SoC, a cancer-detection rate of 6.4 (95% CI 5.7–7.1) vs. 5.0 (4.4–5.6) per 1000, a ratio of 1.28 (1.09–1.50, p=0.002), an increased detection of 51 invasive and 24 in situ cancers. AI-supported screening detected 204 cancers with non-specific histologic type and 122 cancers with T1c-stage, compared to 155 and 79 with SoC. Notably, 20 more non-luminal A invasive cancers, and 12 more DCIS grade 3 were detected with AI-support.
**Conclusion:** An AI-supported screen reading protocol resulted in a 28% increase in cancer detection compared to double reading without AI. Subtype and stage of detected cancers suggest that AI can aid in early detection of clinically relevant cancers.
**Limitations:** It is a single-institution trial.
**Ethics committee approval:** The study was approved by the Swedish Ethical Review Authority (2020-04936).
**Funding for this study:** Funding was received from: The Swedish Cancer Society; Regional Cancer Centres; Lund University ALF-funds.
**Author Disclosures:**
Ingvar Andersson: Nothing to disclose
Aldana Rosso: Nothing to disclose
Viktoria Josefsson: Nothing to disclose
Veronica Hernström: Nothing to disclose
Anna-Maria Larsson: Nothing to disclose
Kristina Lang: Nothing to disclose
Hanna Sartor: Nothing to disclose
Oskar Hagberg: Nothing to disclose
David Schmidt: Nothing to disclose

### RPS 2002-9

### Performance of vacuum-assisted excision (VAE) with "cavity margins shaving" technique in small clusters of suspicious microcalcifications

*F. Morciano*[1], R. Rella[1], M. Conti[1], P. Belli[1], O. Tommasini[1], F. Fornasa[2], E. Gori[1], G. Romanucci[2]; [1]Rome/IT, [2]Verona/IT

**Purpose:** The study aimed to assess the diagnostic performance of vacuum-assisted excision (VAE) under stereotactic guidance with "cavity margins shaving" to ensure total lesion removal in patients with ≤1 cm clusters of microcalcifications.
**Methods or Backround:** Patients with ≤1 cm clusters of microcalcifications were eligible. All VAE procedures were performed under stereotactic guidance with a 9G-needle. The first 12 specimens were retrieved into the first sample container (SC); then SC was changed without removing the biopsy needle and cavity margins were shaved with 12 additional specimens into a second SC. Inclusion criteria: complete removal of microcalcifications; B5 or B3 lesion; surgical histopathological examination or imaging follow-up (FUP) ≥24 months. Histopathologic assessment of specimens of the first and second containers was performed separately. Presence of residual lesion in the second SC was compared with surgical excision histology or imaging-FUP evolution, evaluating negative predictive value (NPV) and false-negative rate (FNR).
**Results or Findings:** A total of 60 lesions were included: 15 B5 and 45 B3. Surgery was performed on 33/60 lesions (all B5 and 18 B3) while the remaining 27/60 B3 lesions were sent to FUP, with one interval change with subsequent surgical excision revealing a ductal carcinoma in situ (DCIS). NPV of the absence of residual lesion in the second container was 81.5% (95% CI: 61.2%-93.0%). FNR was 18.5% (95% CI: 7.0%-38.7%). None of the atypical ductal hyperplasia (ADH) (n=14) or DCIS (n=11) cases were false-negative.
**Conclusion:** The absence of residual lesion in the "cavity margins shaving" of VAE (using a second SC) can predict complete lesion removal in small clusters of microcalcifications. VAE with "cavity margins shaving" technique is effective in confirming complete lesion removal in small clusters of microcalcifications and seems to be a promising decision-support tool to reduce subsequent surgical excision in selected lesions, such as ADH or DCIS.

**Limitations:** No limitations were identified.
**Ethics committee approval:** The study is retrospective.
**Funding for this study:** No funding was received for this study.
**Author Disclosures:**
Marco Conti: Nothing to disclose
Rossella Rella: Nothing to disclose
Elisabetta Gori: Nothing to disclose
Oscar Tommasini: Nothing to disclose
Francesca Morciano: Nothing to disclose
Francesca Fornasa: Nothing to disclose
Giovanna Romanucci: Nothing to disclose
Paolo Belli: Nothing to disclose

### RPS 2002-10

**Influence of complete lesion removal at VAB on the upgrade of B3 lesions presenting as microcalcifications: five years of experience from a referral centre**

*G. Irmici*[1], C. Depretto[1], L. Rabiolo[2], G. Della Pepa[1], E. D'Ascoli[1], C. De Berardinis[1], S. Schiaffino[3], A. Cozzi[3], G. P. Scaperrotta[1]; [1]Milan/IT, [2]Palermo/IT, [3]Lugano/CH
*(irmici.giovanni25@gmail.com)*

**Purpose:** B3 breast lesions have uncertain malignant potential, with different risks of upgrade to malignancy at surgery and/or follow-up. This study on B3 lesions presenting as microcalcifications, for which vacuum-assisted biopsy (VAB) represents the standard approach, aimed to investigate the influence of complete or partial lesion removal at VAB on the subsequent upgrade rate (UR).
**Methods or Backround:** For this retrospective monocentric study conducted in a referral centre, we retrieved 165 lesions presenting solely as microcalcifications and being categorised as BI-RADS 4/5 at mammography, then subsequently diagnosed as B3 at VAB (40 ADH, 53 FEA, 40 ALH, 18 PL, and 14 RS) between January 2016 and December 2020. Surgical pathology or at least 3-years follow-up were obtained to determine eventual lesion upgrade to malignancy. The $\chi^2$, Fisher's, and Mantel-Haenszel tests were performed to assess if complete lesion removal influenced URs, overall and among different B3 subtypes.
**Results or Findings:** Complete lesion removal was achieved in 99/165 (60.0%) cases. The rate of complete removal did not significantly differ among B3 subtypes (p=0.092, Bonferroni-adjusted multiple comparisons p≥0.101), ranging from 33.3% of PL (6/18) to 78.6% (11/14) of RS. The overall UR was 8.5% (95% CI 5.1–13.7%, 14/165), not significantly differing among B3 subtypes (p=0.562). Conversely, the UR of completely removed lesions (4.0%, 95% CI 1.6–9.9%) was significantly lower than that of partially removed lesions (15.2%, 95% CI 8.4–25.7%, p=0.020). At stratified analysis according to B3 subtypes, the risk ratio of upgrade among completely and partially removed FEA (0.15, 95% CI 0.01–1.26) was significantly lower (Mantel-Haenszel test p=0.034) than those of ADH (0.36, 95% CI 0.07–1.90) and of ALH (0.74, 95% CI 0.05–10.99).
**Conclusion:** The UR of B3 lesions is significantly influenced by complete lesion removal, both overall and among different B3 subtypes.
**Limitations:** The relatively small sample size was an identified limitation.
**Ethics committee approval:** No additional information provided by the submitter.
**Funding for this study:** No funding was received for this study.
**Author Disclosures:**
Gianmarco Della Pepa: Nothing to disclose
Andrea Cozzi: Nothing to disclose
Elisa D'Ascoli: Nothing to disclose
Claudia De Berardinis: Nothing to disclose
Simone Schiaffino: Nothing to disclose
Gianfranco Paride Scaperrotta: Nothing to disclose
Catherine Depretto: Nothing to disclose
Giovanni Irmici: Nothing to disclose
Lidia Rabiolo: Nothing to disclose

### RPS 2002-11

**High-risk breast lesions treated with vacuum-assisted excision (VAE): a systematic review and meta-analysis of 3,975 cases**

*A. Cozzi*[1], M. Cao[2], F. Del Grande[1], N. Sharma[3], S. Schiaffino[1]; [1]Lugano/CH, [2]Milan/IT, [3]Leeds/UK

**Purpose:** The objective of this study was to perform a systematic review and meta-analysis of the upgrade rates to malignancy and the surgical excision rates of B3 lesions treated with vacuum-assisted excision (VAE) after core-needle biopsy (CNB), while also analysing the procedural complication rate of VAE.
**Methods or Backround:** After protocol registration on PROSPERO (CRD42023396663), PubMed and EMBASE were searched for articles published up to 15/04/2023 reporting the use of VAE on B3 lesions diagnosed at CNB, taking surgical pathology and/or follow-up as reference standard. Three readers independently performed article selection and extracted data for the following endpoints: immediate upgrade rate, rate of complications, rates of immediate and long-term surgical excision, upgrade rates after immediate surgical excision and during follow-up. Random-effects meta-analyses of single proportions were performed for each endpoint.
**Results or Findings:** Sixteen studies (3,941 patients, 3,975 VAE procedures) from six countries, published between 2008 and 2023, were included in quantitative synthesis. The summary immediate upgrade rate at VAE was 4.0% (95% CI 1.4-7.6%). A total of 81 complications were found among 854 patients, the overall summary complication rate being 6.6% (95% CI 1.3-14.6%): 79 (97.5%) were minor complications whereas only two (2.5%) were major complications. Immediate surgical excision was performed in 214 cases, with a 1.5% immediate surgical excision summary rate (95% CI 0.1%-3.6%) and a corresponding 25.4% summary upgrade rate (95% CI 11.0-42.4%). Among 1,375 patients in follow-up, surgical excision was performed in 39 cases, with a 1.4% summary rate of surgical excision during follow-up (95% CI 0.1%-3.4%) and a summary upgrade rate during follow-up of 0.01% (95% CI 0.0-0.6%).
**Conclusion:** VAE of B3 lesions has low rates of procedural complications, immediate upgrade to malignancy, and subsequent surgical excision, highlighting its promising role as a first-line treatment for high-risk lesions.
**Limitations:** Substantial-to-high heterogeneity ($I^2$>74.1%) was an identified limitation.
**Ethics committee approval:** This systematic review and meta-analysis did not need any specific ethics committee approval.
**Funding for this study:** No funding was received for this study.
**Author Disclosures:**
Andrea Cozzi: Nothing to disclose
Nisha Sharma: Nothing to disclose
Mengmeng Cao: Nothing to disclose
Simone Schiaffino: Speaker: GE Healthcare; Other: Bracco Imaging; Other: Arterys Inc.
Filippo Del Grande: Nothing to disclose

### RPS 2002-12

**Intertumoral heterogeneity in primary breast tumours and synchronous axillary lymph node metastases: comparing immunohistochemical expression for concurrent core needle biopsy consideration**

*M. Ş. Aydın*, S. Doğan, H. Akgün; Kayseri/TR
*(msaydin16@gmail.com)*

**Purpose:** Breast cancer is a heterogeneous disease. Receptor expression can change throughout the progression of the disease. Clinically significant discordance has been detected between the primary breast tumour (PT) and metastases other than metastatic synchronous axillary lymph nodes (ALN). Therefore, routine biopsy is recommended from metastatic lesion. We aimed to evaluate the discordance between PT and ALN and address the need to concurrently biopsy ALN.
**Methods or Backround:** We prospectively performed core needle biopsies on 95 patients from September 2018 to September 2023 and performed immunohistochemistry (IHC) staining for oestrogen receptor (ER), progesterone receptor (PR), human epidermal growth factor receptor-2 (HER2), and Ki67 in both PTs and ALNs. Cutoff values were >1% for ER/PR and %15 for ki67. HER2 status was determined following ASCO/CAP guidelines. We categorized PTs and ALNs into intrinsic subtypes based on the St Gallen classification. We assessed the consistency using the Cohen's Kappa test.
**Results or Findings:** Discordance rates between PT and ALN were %6 for ER, %15 for PR, %7 for HER2 and %8 for Ki67. Very good agreement was observed for ER and HER2 (k-values 0.845, 0.812 respectively) and good agreement was observed for PR and Ki67 (k-value 0.666, 0.619). Discordance rates for intrinsic subtypes was found to be %21, and good agreement (k-value 0.724) was observed. There were 10 patients in whom PT was hormone receptor and/or HER2 negative but were positive for these markers in ALN.
**Conclusion:** There was good statistical consistency between PT and ALN receptor expression. But there were clinically significant discordances that may affect the treatment selection in some patients. Given the lack of complete concordance between PT and ALN, concurrent ALN core needle biopsy may still be considered. Larger studies will be necessary to further define the need of concurrent biopsy.
**Limitations:** The study included a small sample size.
**Ethics committee approval:** This study was approved by the Ethics Committee of Erciyes University Faculty of Medicine under reference number 2018/588.
**Funding for this study:** No funding was provided for this study.
**Author Disclosures:**
Serap Doğan: Nothing to disclose
Hülya Akgün: Nothing to disclose
Muhammed Şamil Aydın: Nothing to disclose

**14:00-15:30** **Research Stage 3**

### Research Presentation Session: Cardiac

### RPS 2003

### Clinical applications of AI and ML in cardiovascular imaging

**Moderator**
S. Boccalini; Lyon/FR
*(sara.boccalini@yahoo.com)*
**Author Disclosures:**
Sara Boccalini: Speaker: Philips Healthcare, Nothing related to the content of this moderation

### RPS 2003-2

**Deep learning denoising in cardiac CT imaging: improved image quality and workflow efficiency**

*A. S. Brendlin*, D. Wessling, J. Hofmann, J. Mück, B. Stenzl, S. Afat; Tübingen/DE

**Purpose:** To evaluate the impact of a deep learning denoising (DLD) algorithm on image quality, diagnostic confidence, and radiological workflows in the context of cardiac computed tomography angiography (CCTA).
**Methods or Backround:** One-hundred patients (mean age 60±11 years) with a CCTA were included. Images were reconstructed using iterative reconstruction strength 2 (IR2) and the DLD algorithm. Place-consistent noise measurements were used to compare objective image quality. In addition, two blinded readers independently assessed subjective image quality, diagnostic confidence, sharpness, and contrast in a forced-choice setup. The results of these assessments were summarised for a semiquantitative overall quality score. Agatston-score and cardiac age were analysed semi-automatically for both datasets using proprietary software, before and after manually correcting the initial software output. The time required for manual corrections was measured for each reader to compare possible workflow benefits. Properly corrected mixed-effects analysis with post hoc subgroup tests was used. Spearman's correlation coefficient measured inter-reader agreement for the image quality analysis.
**Results or Findings:** Noise in IR2 was significantly higher than for DLD (22.00±2.32 versus 13.33±2.87 HU; p<0.001). DLD reconstructions had a significantly higher mean overall quality score than IR2 (3.5±1.0 versus 0.48±1.0, p<0.001) with a good inter-rater agreement (r≥0.790; p≤0.001). There were no significant differences between cardiac age results (p=0.517) and Agatson score values (p=0.486) of IR2 and DLD. However, the time required for manual corrections was significantly shorter for DLD than for IR2 (54±44 versus 35±31 seconds, p<0.001).
**Conclusion:** DLD significantly improves image quality in cardiac CT and substantially reduces the time required for manual corrections in cardiac age assessment.
**Limitations:** Identified limitations were: (1) the single-centre design; (2) the retrospective design; (3) the specific hardware and software setup.
**Ethics committee approval:** The institutional review board approved retrospective eligibility analysis and data collection for this single-centre study's purpose with a waiver for the need for informed consent (reference number: 167/2022BO2).
**Funding for this study:** No funding was received for this study.
**Author Disclosures:**
Daniel Wessling: Nothing to disclose
Andreas Stefan Brendlin: Nothing to disclose
Johannes Hofmann: Nothing to disclose
Saif Afat: Nothing to disclose
Benedikt Stenzl: Nothing to disclose
Jonas Mück: Nothing to disclose

### RPS 2003-3

**Impact of AI-enabled motion compensation algorithm on coronary computed tomography angiography image quality**

*G. Stancanelli*, L. Dominici, L. Conia, G. C. Pambianchi, C. Catalano, N. Galea; Rome/IT
*(giuseppe.stancanelli@uniroma1.it)*

**Purpose:** Motion artefacts remain a major limitation of coronary computed tomography angiography (CCTA), especially in patients with high cardiac frequency or rhythm variability and in coronary segments subject to motion, even in relatively quiescent phases of the cardiac cycle. The aim of our study is to evaluate the impact on image quality of an AI-enabled interaction-free motion compensation reconstruction algorithm (MCR) compared to a standard filtered back projection reconstruction (FBP).
**Methods or Backround:** Fifty patients underwent CCTA on a 128-slice scanner (Incisive CT, Philips) with an ECG modulated retrospective acquisition protocol. Raw datasets were reconstructed during the telediastolic phase of the cardiac cycle using a standard FBP algorithm and processed on an offline workstation to generate interaction-free MCR images (Precise Cardiac Suite, Philips). The two image quality datasets were evaluated side by side by a reader with three years of experience blinded to the reconstruction technique. Image quality was graded per-segment and per-patient on a 1-4 scale based on the severity of the motion artefacts ("blurring", "winging" or "stairstep").
**Results or Findings:** Five hundred coronary artery segments were evaluated in both FBP and MCR reconstruction datasets; per segment coronary segmental image quality scores are reported in Fig 1. We observed a global statistically significant increase of mean scores after the application of the MCR algorithm (FBP: 2.75±1.04; MCR: 2.82±1.02 [p<0.01]. Overall, 15 out of 75 non-diagnostic segments were reclassified as diagnostic on MCR images, most of which (8/11) were on midRCA (p=0.02).
**Conclusion:** The application of MCR algorithm resulted in a global reduction of motion artefacts and an increase in image quality, with reclassification of non-diagnostic segments most evident on midRCA. This determined a better diagnostic performance of CCTA on segments most prone to motion artefacts.
**Limitations:** No limitations were identified.
**Ethics committee approval:** No additional information provided by the submitter.
**Funding for this study:** This study received no funding.
**Author Disclosures:**
Giacomo Carlo Pambianchi: Nothing to disclose
Giuseppe Stancanelli: Nothing to disclose
Luca Conia: Nothing to disclose
Nicola Galea: Nothing to disclose
Lorenzo Dominici: Nothing to disclose
Carlo Catalano: Nothing to disclose

### RPS 2003-4

**Automatic AI-based calcium scoring in cardiac and chest computed tomography: a validation study**

*I. Hamelink*, Z. Nie, T. Kwee, M. Dorrius, P. M. A. Van Ooijen, R. Vliegenthart; Groningen/NL
*(irishamelink@hotmail.com)*

**Purpose:** The extent of coronary artery calcium (CAC), assessed on computed tomography (CT), is a strong predictor of cardiovascular disease. The aim of this study is to validate the performance of an automatic AI system for quantifying CAC.
**Methods or Backround:** 687 participants (59±4.8 years; 48.8% men) of the population-based ImaLife cohort were analysed for CAC. The Agatston score (AS) on cardiac and chest CT scans were quantified manually by a radiologist and automatically by an AI system (AI-Rad Companion Chest prototype, Siemens Healthineers). Agreement of manual and AI measurements was assessed by sensitivity and accuracy, Bland-Altman analysis and Cohen’s kappa for classification in AS strata (0; 1-99; 100-299; ≥300).
**Results or Findings:** Three participants were excluded due to incorrect manual measurement or a history of coronary stenting, resulting in 684 participants for evaluation. In cardiac CT, 200 (29%) participants showed no CAC when evaluated manually. 331 (48.4%) participants showed AS between 1 and 99, 92 (13.5%) participants between 100 and 299 and 61 (8.9%) participants ≥300. AI software showed a high sensitivity for CAC: 98.1% in cardiac CT (accuracy 97.2%) and 95.4% in chest CT (accuracy 92.1%). Bland-Altman analysis showed systematic bias of 2.3 and repeatability coefficient of 23.0 for AS on cardiac CT; and -0.3 and 38.0 for AS on chest CT. Cohen’s kappa for agreement in AS categorisation was 0.94 for cardiac CT and 0.87 for chest CT, with concordance in 96.0 and 91.4% of cases, respectively.
**Conclusion:** AI-based CAC scoring shows a high detection rate compared to manual evaluation, with excellent performance for risk classification. Performance is slightly better in cardiac CT than in chest CT.
**Limitations:** For both scan protocols, a low-dose, high-pitch scan protocol was used; it is unclear how generalisable results are for other scan protocols.
**Ethics committee approval:** This study was approved by the Medical Ethics Committee of the University Medical Center Groningen
**Funding for this study:** The ImaLife study is supported by an institutional research grant from Siemens Healthineers and by the Ministry of Economic Affairs and Climate Policy by means of the PPP Allowance, made available by the Top Sector Life Sciences & Health to stimulate public-private partnerships.
**Author Disclosures:**
Monique Dorrius: Nothing to disclose
Iris Hamelink: Research/Grant Support: Siemens Healthineers
Peter M. A. Van Ooijen: Nothing to disclose
Rozemarijn Vliegenthart: Grant Recipient: Siemens Healthineers
Thomas Kwee: Nothing to disclose
Zhenhui Nie: Nothing to disclose

### RPS 2003-5

**AI-precision in cardiovascular risk assessment: non-gated chest CT coronary artery calcium scoring**

*D. Mu*[1], K. Yin[1], W. Chen[1], X. Chen[2], B. Zhang[1]; [1]Nanjing/CN, [2]Shanghai/CN

**Purpose:** This study aimed to assess the performance of an artificial intelligence-based coronary artery calcium score (AI-CACS) algorithm on non-gated chest computed tomography (CT) images in differentiating risk categories for cardiovascular diseases.
**Methods or Backround:** A prospective study enrolled 112 patients who underwent both chest CT and electrocardiogram (ECG)-gated non-contrast enhanced cardiac CT using the same equipment simultaneously. Chest CT images were reconstructed at three different thicknesses (1 mm, 3 mm, and 5 mm). The Agatston score, obtained semi-automatically from ECG-gated cardiac CT scans using a dedicated post-processing workstation, served as the reference. The AI-CACS software automatically derived the Agatston score from chest CT data. Correlations between AI-CACS and the reference Agatston score were calculated. The AI-CACS's performance in classifying risk categories, dichotomised at thresholds of 0, 100, and 400, was assessed.
**Results or Findings:** The AI-CACS showed strong correlations with the reference Agatston score for the three different slice thicknesses (1 mm: 0.973, 3 mm: 0.941, 5 mm: 0.834; all $p < 0.001$). Agreement in risk categories, assessed using kappa (κ) statistics, was substantial ($\kappa = 0.868$, $p < 0.001$), moderate ($\kappa = 0.772$, $p < 0.001$), and fair ($\kappa = 0.412$, $p < 0.001$) for 1 mm, 3 mm, and 5 mm slice thicknesses, respectively, with concordance rates of 91%, 84.8%, and 62.5%. When dichotomised at thresholds of 0, 100, and 400, the area under the curve for AI-CACS at the three slice thicknesses ranged from 0.785 to 0.996, 0.975 to 0.995, and 0.981 to 1.000, respectively.
**Conclusion:** The AI-CACS algorithm applied to chest CT images demonstrates promising performance in assessing cardiovascular disease risk. Using a 1 mm slice thickness for image reconstruction may yield the best results.
**Limitations:** A larger multi-centred, multi-vendor cohort study shall be conducted.
**Ethics committee approval:** This study was ethically approved by the IRB number: 2022-547-01.
**Funding for this study:** No funding was obtained for this study.
**Author Disclosures:**
Bing Zhang: Nothing to disclose
Kejie Yin: Nothing to disclose
Xingbiao Chen: Employee: Philips Healthcare
Wenping Chen: Nothing to disclose
Dan Mu: Nothing to disclose

### RPS 2003-6

**AI-based quantification of total and vessel-specific coronary artery calcifications in calcium scoring CT**

*L. Henriksson*, M. Sandstedt, A. Persson; Linköping/SE
*(lilianhenriksson@yahoo.se)*

**Purpose:** The objective of this study was to assess the correlation and agreement between fully automatic AI and standard semi-automatic evaluations by radiologists in calcium scoring CT (CSCT) examinations within the Swedish CardioPulmonary bio-image study (SCAPIS).
**Methods or Backround:** A retrospective observational study involving 4567 CSCT exams carried out on a dual-source 128x128 slice CT scanner (Somatom Definition Flash, Siemens Healthineers, Forchheim, Germany) was conducted. Automatic AI measurements and semi-automatic measurements included Agatston score (AS), volume score (VS), mass score (MS), and number of lesions in the coronary arteries (LM+LAD, CX, RCA).
**Results or Findings:** Pearson correlation coefficients (r) for total AS, VS, and MS were r=0.989, 0.988, and 0.988 respectively. Intra-class correlation coefficients (ICCs) showed high levels of agreement: 0.994 for total AS, 0.998 for VS, 0.998 for MS, and 0.960 for the number of lesions. Bland-Altman analysis indicated minimal bias across all metrics. Weighted kappa for risk category classifications was $\kappa$= 0.919, and the overall accuracy was 91.2%.
**Conclusion:** The study demonstrates excellent correlation and agreement between fully automated AI and radiologists' semi-automatic evaluations in CSCT examinations. The findings suggest that AI could be a reliable tool for calcium scoring, with implications for improving efficiency and standardisation in radiological assessments.
**Limitations:** Even though this is the largest study made so far regarding the accuracy of AI CSCT evaluations the study is limited by only including CSCT examinations performed on one type of CT scanner.
**Ethics committee approval:** This study was ethically approved by the Swedish ethical review authority, regional ethical review board in Göteborg: DNR 2021-00441.
**Funding for this study:** No funding was received for this study.
**Author Disclosures:**
Anders Persson: Nothing to disclose
Lilian Henriksson: Nothing to disclose
Mårten Sandstedt: Nothing to disclose

### RPS 2003-7

**A deep learning algorithm for fully-automated myocardial infarct scar segmentation**

*M. Schwab*, M. Pamminger, C. Kremser, M. Haltmeier, A. Mayr; Innsbruck/AT
*(matthias.schwab@i-med.ac.at)*

**Purpose:** Late gadolinium enhancement (LGE) cardiac magnetic resonance (CMR) imaging is considered the in vivo reference standard for assessing infarct size and microvascular obstruction (MVO) in ST-elevation myocardial infarction patients. As LGE distribution patterns can be quite complex and hard to delineate from the blood pool or epicardial fat, automatic segmentation of LGE CMR images is challenging. The aim of this work is to develop and evaluate a deep learning-based method that allows to perform LGE segmentation in a fully-automated way.
**Methods or Backround:** A cascaded framework of two-dimensional and three-dimensional convolutional neural networks (CNNs), specialized on identifying myocardial scars, was trained on a training data set consisting of 224 patients. On a test data set including LGE CMR images from 152 examinations AI-based segmentations were compared to manual segmentations, which were performed according to the +5-SD method. Further, on a big data set of 1012 patients automatically calculated infarct volumes were correlated with maximum levels of creatine kinase and cardiac troponin obtained after acute myocardial infarction and successful primary percutaneous coronary interventions.
**Results or Findings:** Mean Dice coefficients between manual and CNN segmentations were 64.1% for LGE and 85.3% for MVO, respectively. Further, linear correlation between manually and automatically calculated infarct sizes was very strong ($R=0.95$, $p<0.001$). Good correlation between AI measured LGE volumes and biochemical measurements could also be found (creatine kinase: $R=0.72$, $p<0.001$; cardiac troponin: $R=0.67$ $p<0.001$).
**Conclusion:** Our fully-automated framework for LGE segmentation provides measurements that can compete with the very time-consuming manual segmentations.
**Limitations:** The limitations of the study are that for evaluation of the method, data from only one hospital (University Hospital Innsbruck) was used.
**Ethics committee approval:** The study utilised retrospective data analysis.
**Funding for this study:** Funding was provided by the Austrian Science Fund (FWF): DOC 110.
**Author Disclosures:**
Markus Haltmeier: Nothing to disclose
Mathias Pamminger: Nothing to disclose
Agnes Mayr: Nothing to disclose
Christian Kremser: Nothing to disclose
Matthias Schwab: Nothing to disclose

### RPS 2003-8

**Artificial intelligence-based CT-derived FFR for the detection of haemodynamically significant coronary artery disease: a comparative study with dynamic stress CT myocardial perfusion imaging**

*A. Andary*, A. Rodriguez Musso, C. V. Gkizas, P. Carpentier, N. Abassebay, C. Lardemelle, B. Longere, F. A. Pontana; Lille/FR
*(antoine7andary@gmail.com)*

**Purpose:** CT myocardial perfusion imaging (CT-MPI) combined with coronary CTA integrates coronary artery anatomy with inducible ischaemia at the cost of a higher radiation and contrast reinjection. The aim of this study was to evaluate the diagnostic performance of a deep-learning model of CT-derived fractional flow reserve (FFR-AI) for the detection of haemodynamically significant coronary artery disease (CAD) compared to CT-MPI.
**Methods or Backround:** This retrospective study included 36 patients who underwent coronary CTA and dynamic stress CT-MPI on a third-generation dual-source CT system (SOMATOM Force, Siemens Healthineers). CT-MPI was performed when the maximal coronary stenosis was ≥50% (CAD-RADS≥3) or in the presence of stent, according to our centre's practice after injection of regadenoson. Perfusion maps were interpreted by two radiologists by consensus. A perfusion defect was defined as a visually significant anomaly on the myocardial blood flow (MBF) map, in a coronary territory (windowing at 100 mL/100 mL/min by default). Curvilinear images of the main coronary arteries were then exported to CorEx model (version 1.0; Spimed-AI), which classified each of these arteries into two categories: FFR ≤0.8 or FFR >0.8.
**Results or Findings:** CT-MPI detected perfusion defects in 16 of 36 patients (44%). FFR-AI demonstrated a per-patient sensitivity, specificity, PPV, NPV and accuracy for the detection of hemodynamically significant stenosis of 100% (95% CI: 79%-100%), 50% (95% CI: 27%-73%), 61.5% (95% CI: 51%-71%), 100% and 72% (95% CI: 55%-86%), respectively. The areas under the ROC curve of FFR-AI were 0.75 (95% CI: 0.78-0.88).
**Conclusion:** FFR-AI provides high sensitivity and NPV for identifying haemodynamically significant CAD among patients with coronary stenosis ≥50%. FFR-AI could be used as a filter to avoid a subsequent CT-MPI and reduce radiation exposure and contrast reinjection.
**Limitations:** No limitations were identified.

**Ethics committee approval:** The study received institutional review board approval and written informed consent was obtained from all participants.
**Funding for this study:** No funding was received for this study.
**Author Disclosures:**
Claire Lardemelle: Nothing to disclose
Benjamin Longere: Nothing to disclose
Antoine Andary: Nothing to disclose
Christos Vasileiou Gkizas: Nothing to disclose
Paul Carpentier: Nothing to disclose
Aimée Rodriguez Musso: Nothing to disclose
François Ascagne Pontana: Nothing to disclose
Neelem Abassebay: Nothing to disclose

### RPS 2003-9

**Machine learning explainable analysis for prediction of atrial fibrillation recurrence after catheter ablation using clinical and radiological variables**

*A. P. Ruiz De Temiño*, J. M. Castro, M. J. Garfias, H. Trigueros Buil, A. Adarve Castro, D. F. Ferrández, B. Martínez-López; Alicante/ES
*(a.palazon.rtm@gmail.com)*

**Purpose:** Atrial fibrillation (AF) is a common arrhythmia with increasing prevalence and significant clinical impact. Catheter ablation has emerged as a treatment option for drug-resistant AF, with variable success rates. This study aimed to develop a machine learning-based model to predict AF recurrence after pulmonary vein ablation.
**Methods or Backround:** A retrospective case-control study included patients who underwent first radiofrequency or cryoablation between 2017 and 2022. CT scans were used to measure left atrial volume (LAV), periatrial adipose tissue (PAT), interatrial adipose tissue (IAT), and (EAT) epicardial adipose tissue volume. Demographic, clinical, and recurrence data were collected. Feature selection and data preprocessing were conducted, followed by model training using three machine learning techniques. Model evaluation included accuracy, precision, recall, F1-score, and ROC/AUC. SHAP analysis was performed to interpret feature importance.
**Results or Findings:** Sixty nine patients were included. Recurrence occurred in 29% of patients. Persistent AF exhibited a higher risk of recurrence (OR 1.99, $p<0.05$). Radiological variables like left atrial, PAT and IAT volumes were significantly higher in recurrence cases. The logistic regression model including clinical and radiological variables (model A) achieved the highest average precision, accuracy, f1-score, and recall during cross-validation. Model A's accuracy in the testing group was 0.86, 0.66, and 0.86 and the AUC were 0.91, 0.87, and 0.92 using NN, NB, and LR respectively. SHAP analysis revealed varying feature importance across techniques in model A emphasizing the LAV, PAT and AF type.
**Conclusion:** This study presents two models incorporating adipose tissue measurements for predicting AF recurrence after pulmonary vein ablation with the potential of utilizing multimodal data in predicting post-ablation outcomes for AF patients.
**Limitations:** Sample size is limited, which might lead to overfitting. However, undersampling, scaling and cross-validation were employed as methods to mitigate this.
**Ethics committee approval:** Patient data was handled confidentially and informed consent was waived by the ethics committee due to the retrospective nature and reliance on medical record review. Protocol registration number was PI2023-045.
**Funding for this study:** No funding was received for this study.
**Author Disclosures:**
Beatriz Martínez-López: Nothing to disclose
Helena Trigueros Buil: Nothing to disclose
Alvaro Palazón Ruiz De Temiño: Nothing to disclose
José Miguel Castro: Nothing to disclose
Maria Javiera Garfias: Nothing to disclose
Antonio Adarve Castro: Nothing to disclose
David Ferrández Ferrández: Nothing to disclose

### RPS 2003-10

**Impact of super resolution deep learning reconstruction with 1024 matrix in inter and intra reader reproducibility of pre-TAVR CT measurements**

A. Walch[1], F. Tatsugami[2], W. Fukumoto[2], D. Touitou-Gottenberg[1], A. Taniguchi[3], K. Haioun[3], K. Awai[2], C. Roy[1], *M. Ohana*[1]; [1]Strasbourg/FR, [2]Hiroshima/JP, [3]Otawara/JP
*(mickael.ohana@chru-strasbourg.fr)*

**Purpose:** Reproducibility of aortic annulus sizing and aortic valve opening area planimetry on pre-TAVR cardiac CT is essential. Whether the use of a Super Resolution Deep Learning Reconstruction (SR-DLR) algorithm with increased matrix size could modify the inter and intra reader correlation of these measurements, particularly in case of heavily calcified aortic cusps, is unknown. Our primary objective is therefore to compare inter and intra reader reproducibility of aortic annulus and aortic valve area planimetry measurements between DLR and SR-DLR.
**Methods or Backround:** Forty pre-TAVR CT with excellent image quality were retrospectively selected from two tertiary centers. Systolic phase was reconstructed with DLR in 512 and SR-DLR in 1024 matrixes. Four radiologists with different levels of expertise independently and randomly reviewed all 80 datasets to assess aortic annulus area and aortic valve planimetry. Two readers redid all measurements following a four week delay. Statistical analysis was performed using Bland-Altman plots and intraclass correlation coefficient (ICC).
**Results or Findings:** Interobserver agreement for aortic annulus area were excellent and similar between DLR (ICC 0.85, 95% CI 0.82-0.88) and SR-DLR (ICC 0.87, 95% CI 0.85-0.90). Interobserver agreement for aortic valve planimetry was higher with SR-DLR (ICC 0.90, 95% CI 0.86-0.92) than with DLR (ICC 0.83, 95% CI 0.80-0.85). This difference was more pronounced in the subgroup of patients with a heavily calcified aortic valve (calcium score >2000, n=24).
Intra-observer agreement for both measurements were slightly higher with SR-DLR.
**Conclusion:** SR-DLR with 1024 matrix could increase the reproducibility of aortic valve area planimetry, especially in heavily calcified aortic valve.
**Limitations:** Potential clinical implications of SR-DLR on device selection were not analyzed in this study.
**Ethics committee approval:** This study was ethically approved by the IRB from Strasbourg University Hospital.
**Funding for this study:** No funding was received for this study.
**Author Disclosures:**
Diane Touitou-Gottenberg: Nothing to disclose
Wataru Fukumoto: Nothing to disclose
Fuminari Tatsugami: Nothing to disclose
Karim Haioun: Employee: Canon Medical Systems Japan
Catherine Roy: Nothing to disclose
Adrien Walch: Nothing to disclose
Kazuo Awai: Nothing to disclose
Akira Taniguchi: Employee: Canon Medical Systems Japan
Mickaël Ohana: Research/Grant Support: Canon Medical Systems Europe; Speaker: Boehringer Ingelheim; Speaker: Canon Medical Systems Europe

### RPS 2003-11

**Enhancing preoperative risk assessment in noncardiac surgery (NCS): comparative evaluation of coronary CT angiography (CCTA), CT perfusion (CTP), and CT-derived fractional flow reserve (CT-FFR)**

*F. Brilli*, F. Catapano, C. Lisi, A. Caracciolo, M. Francone; Milan/IT
*(federicabrilli23@gmail.com)*

**Purpose:** Perioperative cardiovascular complications occur in approx. Three% of NCS hospitalisations. ESC guidelines recommended use of CCTA in low-to-intermediate likelihood of CAD, or in patients unsuitable for non-invasive functional testing undergoing non-urgent, intermediate/high-risk NCS. Our study sought to evaluate added value of CCTA-derived functional testing to predict revascularisation prior to NCS as compared to an anatomical-based strategy.
**Methods or Backround:** Single-cohort prospective observational study including 55 symptomatic patients with stable angina who underwent CCTA prior to NCS; besides general CCTA and CTP contraindications, the presence of a planned invasive coronary angiography (ICA) for preoperative investigation before surgery was a major exclusion criterion. CT-FFR was performed using a ML-based algorithm for FFR simulation in all moderate to severe lesions. A ROC curve analysis was used to assess diagnostic performances of CCTA vs CTP vs CT-FFR in patients undergoing ICA after non-invasive testing.
**Results or Findings:** Significant stenoses were found in 20 participants and confirmed with ICA and FFR-ICA in moderate lesions. At ROC analysis, CTP had the largest AUC on a per-patient level (AUC = 0,84) compared with CT-FFR (0,41). The diagnostic accuracy of CTP and CT-FFR at patient-based analysis were 91% and 79%, respectively. The patient-based sensitivity, specificity, PPV, and NPV of CTP were 100%, 80%, 87% and 100%, whereas these values for CT-FFR (when using ≤0.80 as cutoff value) were 60%, 72%, 60%, and 88%. CCTA underperformed CTP for the diagnosis of flow-limiting coronary stenosis (accuracy at patient-based analysis: 77% vs 91%).
**Conclusion:** CTP offers a one-stop solution for assessing ischemic heart disease in NCS patients.
**Limitations:** Small sample size (55 patients) with stable angina, single-cohort observational design introducing potential selection bias and lacked long-term follow-up data. Larger cohort studies are needed to confirm CTP role in these patients.
**Ethics committee approval:** Not applicable
**Funding for this study:** No funding was obtained for this study.
**Author Disclosures:**
Costanza Lisi: Author: author
Alessandra Caracciolo: Author: author
Federica Catapano: Author: author
Marco Francone: Author: author
Federica Brilli: Author: author

**RPS 2003-12**

**Artificial intelligence for automated classification of coronary lesions from computed tomography coronary angiography scans (ALERT)**

*V. Verpalen*[1], C. Coerkamp[1], J. Henriques[1], J-F. Paul[2], N. R. Planken[1]; [1]Amsterdam/NL, [2]Paris/FR
*(v.a.verpalen@amsterdamumc.nl)*

**Purpose:** The aim of this study was to evaluate the diagnostic performance of a deep-learning model (DLM) for quantifying coronary stenosis on computed tomography coronary angiography (CTCA) using the Coronary Artery Disease-Reporting and Data System (CAD-RADS).
**Methods or Backround:** This single centre retrospective study included 50 patients suspected of coronary artery disease (CAD). All CTCA examinations were obtained in routine clinical practice. Two expert readers and the DLM independently reassessed the CAD-RADS score per patient (n=50) and per vessel (n=150). Binary classification (CAD-RADS 0-2 or 3-5) and six group classification (CAD-RADS 0-5) were used for comparison among the human readers and between the readers and the DLM.
**Results or Findings:** Interhuman sensitivity, specificity, positive predictive value (PPV), negative predictive value (NPV), accuracy, and Cohen's kappa for detecting ≥50% stenosis (binary classification) were 86.4, 85.2, 82.6, 88.5, 85.7%, and 0.71 at patient level. Sensitivity, specificity, PPV, NPV, accuracy, and Cohen's kappa of the DLM for detecting ≥50% stenosis were 100, 69.6, 75.0, 100, 84.1%, and 0.69 at the patient level for reader 1 and 100, 66.7, 71.4, 100, 81.8%, and 0.65 for reader 2 as reference, respectively. For the six group classification at patient level, interhuman agreement was 65.3% and weighted kappa 0.78. For the DLM vs reader 1 and reader 2 this agreement was 54.5 and 56.8%, the weighted kappa was 0.70 and 0.61, respectively.
**Conclusion:** Ruling out obstructive CAD (≥50% stenosis) by the DLM is safe, considering the 100% sensitivity. The DLM yielded promising results in CAD-RADS classification (0-5). This DLM has potential to support and alert CTCA-readers in clinical practice.
**Limitations:** The main limitation of the study is that the CAD-RADS distribution present in the study population does not necessarily reflect local clinical practice, which might influence the local performance of the DLM.
**Ethics committee approval:** The study was approved by Ethics committee Amsterdam UMC: 2023.0484,
**Funding for this study:** No funding was received for this study.
**Author Disclosures:**
Nils Robrecht Planken: Nothing to disclose
Casper Coerkamp: Nothing to disclose
Victor Verpalen: Nothing to disclose
Jean-Francois Paul: CEO: Spimed-AI, 92340 Bourg-La-Reine, France; Founder: Spimed-AI, 92340 Bourg-La-Reine, France
Jose Henriques: Nothing to disclose

**RPS 2003-13**

**Super-resolution deep learning reconstruction for improved image quality of myocardial late enhancement CT**

*M. Takafuji*[1], K. Kitagawa[1], S. Mizutani[2], A. Hamaguchi[2], R. Kiso[2], K. Sasaki[2], Y. Funaki[2], H. Sakuma[1]; [1]Tsu Mie/JP, [2]Matsusaka/JP
*(masa-tkfj@clin.medic.mie-u.ac.jp)*

**Purpose:** Myocardial late enhancement CT (LE-CT) allows assessment of myocardial scar. Super-resolution deep learning image reconstruction (SR-DLR), which is trained on data acquired from ultra-high-resolution CT may improve image quality of LE-CT. The purpose of this study was to investigate image noise and image quality with SR-DLR compared with conventional DLR (C-DLR) and hybrid iterative reconstruction (hybrid-IR).
**Methods or Backround:** We retrospectively analyzed 30 consecutive patients who underwent LE-CT using 320-row CT. The CT protocol consisted of stress dynamic perfusion CT, coronary CT angiography and LE-CT. All images were reconstructed using three different algorithms: SR-DLR, C-DLR, and hybrid-IR. Image noise, signal-to-noise ratio (SNR), contrast-to-noise ratio (CNR), and overall image quality were compared. Overall image quality was assessed by five independent observers. Each observer had 30 points in each case and the points were allocated to LE-CT images reconstructed with the three different algorisms according to image quality. The scores were averaged across all observers.
**Results or Findings:** SR-DLR significantly decreased image noise by 33% compared to C-DLR (6.5±1.4 HU vs 9.7±1.7 HU, P<0.0001) and by 37% compared to hybrid IR (vs 10.4±2.8 HU, P<0.0001). SNR and CNR of LE-CT reconstructed using SR-DLR (SNR, 17.5±4.4; CNR, 4.6±0.8) were significantly higher than C-DLR (SNR, 11.4±2.8 p<0.0001; CNR, 3.1±0.6, p<0.0001) and hybrid-IR (SNR, 11.0±3.2, p<0.0001; CNR, 3.3±0.6, p<0.0001). SR-DLR significantly improved overall image quality of LE-CT compared to C-DLR (13.6±1.3 vs 8.6±0.7, p<0.0001) and hybrid-IR(vs 7.8±0.6, p<0.0001).
**Conclusion:** SR-DLR improved image noise, and image quality of myocardial LE-CT compared with C-DLR and hybrid-IR techniques. The SR-DLR approach has the potential to improve the assessment of myocardial scar by LE-CT and to lower the tube voltage and/or current of LE-CT, thus reducing the radiation dose of LE-CT.
**Limitations:** Not applicable.
**Ethics committee approval:** This study was approved by the institutional review board in our institution, and written informed consent was obtained from each individual before enrolling in the study (reference number: 210604-5-2).
**Funding for this study:** No funding was provided for this study.
**Author Disclosures:**
Ryosuke Kiso: Nothing to disclose
Akane Hamaguchi: Nothing to disclose
Kakuya Kitagawa: Nothing to disclose
Hajime Sakuma: Nothing to disclose
Kenji Sasaki: Nothing to disclose
Yuto Funaki: Nothing to disclose
Masafumi Takafuji: Nothing to disclose
Sachio Mizutani: Nothing to disclose

**14:00-15:30** **Research Stage 4**

### Research Presentation Session: Genitourinary

### RPS 2007

### Innovations in prostate imaging

**Moderator**
M. de Rooij; Nijmegen/NL
*(maarten.derooij@radboudumc.nl)*

**RPS 2007-2**

**Performance of an ultra-fast deep-learning accelerated MRI screening protocol for prostate cancer compared to a standard multiparametric protocol**

*B. Oerther*[1], H. Engel[1], R. Strecker[2], T. Benkert[2], E. Weiland[2], F. Bamberg[1], M. Benndorf[1], J. Weiß[1], C. Wilpert[1]; [1]Freiburg/DE, [2]Erlangen/DE
*(oerther@gmx.de)*

**Purpose:** The aim of this study was to establish and evaluate image quality of an ultra-fast MRI screening protocol for prostate cancer in biopsy-naïve men regarding PI-RADSv2.1 classification in comparison to the standard multiparametric protocol.
**Methods or Backround:** This prospective mono-institutional study included consecutive patients with suspected prostate cancer without prior biopsy. A PI-RADSv2.1 conform multiparametric MRI protocol was acquired in a 3 T MRI scanner (scan time: 25min 45sec). Additionally, two deep-learning accelerated sequences were acquired (scan time: 3 min 28 sec). Two readers evaluated image quality and the presence of prostate cancer. In a first reading session only the screening protocol (DL accelerated axial T2w and ZOOMit DWI) was available. Subsequently, the full conventional mpMRI protocol was assessed and served as a reference standard. Diagnostic performance was analyzed with mpMRI serving as the gold standard. Inter- and intra-reader agreement was assessed using weighted kappa statistics.
**Results or Findings:** The final cohort consisted of 77 patients with 97 lesions. Diagnostic performance of the screening protocol was excellent with a sensitivity and specificity of 100%/97% and 98%/83% (cut-off ≥ PI-RADS 3) vs. 100%/100% and 89%/98% (cut-off ≥ PI-RADS 4) for reader 1 and reader 2, respectively. Mean image quality (Likert-scaling) was 3.96 (R1) and 4.35 (R2) for the standard protocol vs. 4.74 and 4.57 for screening protocol (p < 0.05). Inter-reader agreement was moderate (κ: o.55) for the screening protocol and substantial (κ: o.61) for the multiparametric protocol. Intra- reader agreement was excellent (κ: o.98) for R1 and substantial (κ: o.79) for R2.
**Conclusion:** A DL accelerated screening protocol for prostate cancer in biopsy-naïve men proved similar diagnostic performance and better imaging quality compared to the conventional mpMRI protocol, requiring less than 15% of scan time.
**Limitations:** Monocentric study, limited number of patients; no histopathological ground truth
**Ethics committee approval:** This study was ethically approved the Local ethics committee of Freiburg.
**Funding for this study:** No funding was obtained for this study.
**Author Disclosures:**
Elisabeth Weiland: Employee: Siemens
Benedict Oerther: Nothing to disclose
Caroline Wilpert: Nothing to disclose
Ralph Strecker: Employee: Siemens
Hannes Engel: Nothing to disclose
Matthias Benndorf: Nothing to disclose
Fabian Bamberg: Nothing to disclose
Jakob Weiß: Nothing to disclose
Thomas Benkert: Employee: Siemens

**RPS 2007-3**

**Developing a dynamic predictive model for baseline detection and follow-up re-evaluation of the risk of prostate cancer progression on active surveillance (PROGRESS Prostate)**

N. Sushentsev[1], L. A. Abrego Rangel[2], N. Sanmugalingam[3], I. Caglič[1], V. Gnanapragasam[1], A. Warren[1], A. Zaikin[2], T. Barrett[1], *O. Blyuss*[2]; [1]Cambridge/UK, [2]London/UK, [3]Nowich/UK

**Purpose:** The aim of this study was to develop a dynamic predictive model for baseline detection and follow-up re-evaluation of the risk of prostate cancer (PCa) progression on active surveillance (AS).
**Methods or Backround:** Four hundred and twenty two AS patients were included in this study, of whom 82 (19.4%) experienced either histological PCa progression or radiological stage progression (PRECISE 5) over a median follow-up of 4.5 years. The baseline model included initial serum prostate-specific antigen (PSA) and PSA density (PSAD), MRI-derived Likert score, tumour diameter, and tumour grade group. The follow-up model included baseline Likert score along with longitudinal PRECISE scores, PSAD measurements, and repeat biopsy results. Model training and testing were performed in the 50/50 data split using several neural networks, with three-year progression as the outcome.
**Results or Findings:** The best-performing baseline model was a generalised additive model (GAM) including baseline PSAD and Likert score. With an overall test AUC of 0.65, the model achieved a 21% specificity at 95% sensitivity in the test set, which may be used to avoid repeat biopsies in a substantial proportion of patients with minimal risk of missing disease progression. The follow-up model, comprised of a long short-term memory recurrent neural network, included baseline Likert score together with longitudinal PRECISE and PSAD measurements, with its test AUC of 0.75 being significantly higher compared to that of PRECISE alone (AUC=0.61, P<0.01).
**Conclusion:** The development of MRI-driven risk-adapted AS predictive models is a high research priority in the field; this study shows the promise of the proposed approach to objectively stratify patients at baseline and significantly improve the performance of current standard-of-care PRECISE assessment for detecting disease progression in the follow-up.
**Limitations:** Lack of external validation to be addressed in future studies.
**Ethics committee approval:** This study was ethically approved by the HRA and Health and Care Research Wales (HCRW), IRAS Project ID: 288185.
**Funding for this study:** This study was funded by the Cancer Research UK ACED (A095792/EICEDAAP\100009).
**Author Disclosures:**
Nimalan Sanmugalingam: Nothing to disclose
Iztok Caglič: Nothing to disclose
Vincent Gnanapragasam: Nothing to disclose
Luis Alberto Abrego Rangel: Nothing to disclose
Nikita Sushentsev: Nothing to disclose
Oleg Blyuss: Nothing to disclose
Tristan Barrett: Nothing to disclose
Anne Warren: Nothing to disclose
Alexey Zaikin: Nothing to disclose

**RPS 2007-4**

**DWI²-improvement of diffusion-weighted imaging for the detection of clinical significant prostate cancer**

*B. Valentin*, M. Boschheidgen, T. Ullrich, J. P. Radtke, P. Albers, G. Antoch, H-J. Wittsack, L. Schimmöller; Düsseldorf/DE

**Purpose:** This study aimed to improve the contrast between prostate cancer (PC) and healthy tissue by diffusion-weighted imaging (DWI) post-processing by using a square method.
**Methods or Backround:** DWI post-processing was retrospectively applied on 40 patients with PC (median 68y and PSA 8.0 ng/ml) and multiparametric MRI (mpMRI) performed at 3 Tesla (Magnetom PRISMA® Siemens, Erlangen, Germany). In 20 patients a multi-shot readout segmentation (rs-EPI) plus zoomed single-shot imaging (z-EPI) sequence (Group 1) and in 20 patients a single-shot echo-planar imaging (ss-EPI) plus rs-EPI sequence (Group 2) was applied. All sequences (b1000 and b1800/2000) were squared and afterwards evaluated objectively using the SyngoVia Software (Siemens Healthineers, Erlangen) and subjectively by applying a 5-point scale from unacceptable, poor, moderate, good, to excellent.
**Results or Findings:** The squared processed DWI sequences showed significantly higher contrast-ratio (CR) for ss-EPI b1800 (p<0.001), rs-EPI b1000 (p<0.001), rs-EPI b1800 (p<0.001), z-EPI b1000 (p=0.002) and for z-EPI b2000 (p<0.001). Following post-processing of these sequences, a significant improvement in the subjective assessment of image quality was noticeable for ss-EPI b1000 (p=0.043), ss-EPI b1800 (p=0.030), rs-EPI b1000 (p<0.0001), rs-EPI b1800 (p<0.001) and z-EPI b1000 (p<0.001).
**Conclusion:** The application of the square post-processing for DWI results in a significant improvement in the CR between PC and healthy tissue, especially for high b values of ss-EPI or re-EPI. This method can be instrumental in enhancing the detection and differentiation of PC lesions.
**Limitations:** High b-values can cause overexposure of the lesion and highlighting of non-specific DWI restrictions. This could result in overdiagnosis as well as misinterpretation of the tumour lesion margins.
**Ethics committee approval:** The local ethics committee has positively reviewed this retrospective study.
**Funding for this study:** This study did not receive any funding.
**Author Disclosures:**
Birte Valentin: Nothing to disclose
Tim Ullrich: Nothing to disclose
Jan Philipp Radtke: Nothing to disclose
Peter Albers: Nothing to disclose
Matthias Boschheidgen: Nothing to disclose
Lars Schimmöller: Nothing to disclose
Hans-Jörg Wittsack: Nothing to disclose
Gerald Antoch: Nothing to disclose

**RPS 2007-5**

**Spectral diffusion analysis to improve the detection and classification of clinical significant prostate cancer**

*B. Valentin*, T. Thiel, T. Ullrich, M. Boschheidgen, J. P. Radtke, P. Albers, G. Antoch, H-J. Wittsack, L. Schimmöller; Düsseldorf/DE

**Purpose:** In this study, we applied the model-free nonnegative least squares (NNLS) method. The aim of this study was to assess the number of distinguishable diffusion components (spectral diffusion analysis) within the prostate and to differentiate between healthy and pathological prostate tissue.
**Methods or Backround:** NNLS imaging was performed at 3 Tesla (Magnetom PRISMA® Siemens, Erlangen, Germany) in 10 patients with prostate cancer (PC) (PI-RADS 5 and subsequent biopsy). The 16 b values used were 0, 50, 100, 150, 200, 300, 400, 500, 600, 700, 800, 1000, 1200, 1400, 1600, 1800 s/mm 2. Relative signal fractions and mean diffusivities of the diffusion components in the peripheral zone, central zone and PI-RADS 5 lesion were obtained using the regularized NNLS fitting of the intravoxel incoherent motion data.
**Results or Findings:** Three different diffusion components (10–4, 10–3, and 10–2 mm2/s) were detected in prostate tissue. In comparison, the three peaks were significantly different between healthy and diseased tissue. The fraction of the slow component was significant higher in PC (maximum amplitude of 0.2) compared with the unaffected prostate tissue (maximum amplitude of 0.05).
**Conclusion:** This pilot study demonstrated the feasibility of spectral diffusion weighted imaging for the differentiation of PC. The three distinguishable components may be related to slow tissue diffusion caused by higher tissue density of PC lesions, intermediate fluid flow caused by glandular tissue, and fast blood flow in prostatic vessels. A larger cohort study with a ISUP range is needed to further evaluate this technique.
**Limitations:** This study exclusively focused on PI-RADS 5 lesions. Consequently, we were unable to differentiate between lesion sizes and did not account for variations in high-, intermediate-, and low-risk prostate cancer cases.
**Ethics committee approval:** The local ethics committee has positively reviewed this prospective study.
**Funding for this study:** This study was funded by DFG.
**Author Disclosures:**
Birte Valentin: Nothing to disclose
Thomas Thiel: Nothing to disclose
Tim Ullrich: Nothing to disclose
Jan Philipp Radtke: Nothing to disclose
Peter Albers: Nothing to disclose
Matthias Boschheidgen: Nothing to disclose
Lars Schimmöller: Nothing to disclose
Hans-Jörg Wittsack: Nothing to disclose
Gerald Antoch: Nothing to disclose

**RPS 2007-6**

**Assessing the performance of generative pre-trained transformers against radiologists for PI-RADS classification based on prostate mpMRI text reports**

*K-L. Lee*, D. Kessler, T. Barrett; Cambridge/UK

**Purpose:** Large language models, such as ChatGPT and Bard, have sparked a wave of enthusiasm for their potential applications in clinical radiology, including formulating clinical interpretation of reports. This study aims to compare the classification abilities of ChatGPT, Bard, and two uroradiologists in assigning PI-RADS categories based on clinical text reports.

**Methods or Backround:** Clinical prostate MRI text reports from 100 consecutive treatment-naïve patients undergoing mpMRI between 06.11.2022 to 28.12.2022 were analysed. Clinical history and concluding remarks were removed from the text reports. Two uroradiologists with 14 and 3 years of prostate MRI reporting experience, retrospectively independently classified PI-RADS 2.1 categories on the edited text reports. The same reports were inputted manually into online ChatGPT-3.5 and Bard platforms to generate PI-RADS classifications (without prior training). Original report classifications were considered definitive, and comparisons were made to compare the original reports, the two radiologists, ChatGPT, and Bard. Agreement rates and Kappa scores were analysed.
**Results or Findings:** In the original reports, 52/100 MRIs were classified as PI-RADS 2, 9/100 as PI-RADS 3, 19/100 as PI-RADS 4, and 20/100 as PI-RADS 5, respectively. Compared to the original classifications, the senior and junior radiologists concurred on 95% and 90% of the reports, respectively, while ChatGPT and Bard aligned both on 67 reports. Notably, Bard assigned a non-existent PI-RADS 6 classification to two patients (2%). The interreader agreement (K) between the original reports and the senior radiologist, the junior radiologist, ChatGPT, and BARD were 0.92, 0.85, 0.55, and 0.49, respectively.
**Conclusion:** Concordance on PI-RADS scoring was high among radiologists, however, ChatGPT and Bard demonstrate poor performance for the text-based classification task.
**Limitations:** The limitation of the study is that this a relatively small sample of 100 reports.
**Ethics committee approval:** The study was approved by Cambridge University NHS Foundation Trust (reference number: 288185).
**Funding for this study:** No funding was provided for this study.
**Author Disclosures:**
Kang-Lung Lee: Nothing to disclose
Dimitri Kessler: Nothing to disclose
Tristan Barrett: Nothing to disclose

### RPS 2007-7

**Sodium MRI quantification of prostate tissue and therapy-naïve primary prostate cancer with internal references**

*F. Tollens*, F. G. Zöllner, A. Adlung, S. O. Schönberg, D. Nörenberg; Mannheim/DE

**Purpose:** The aim of this study was to quantify tissue sodium concentration (TSC) in healthy prostate tissue and prostate cancer regions based on internal references in order to evaluate TSC as a potential quantitative imaging biomarker.
**Methods or Backround:** Thirty-six patients with clinically suspected prostate cancer were included into this prospective study and underwent clinical multiparametric magnetic resonance imaging (MRI) and additional sodium MRI of the prostate. Imaging was performed at 3T using a dual-tuned 1H/23Na body-coil to acquire a 3D radial density-adapted 23Na sequence. For the quantification of tissue sodium concentration, femoral blood vessels were chosen as an internal reference and reported sodium levels of ~81 mM were assumed. Peripheral zone (PZ), transition zone (TZ) and tumor regions of interest were defined and TSC was extracted for each segmentation.
**Results or Findings:** Mean TSC differences between right and left femoral blood vessels was 3.3 ± 2.2 mM. TSC was significantly higher in the PZ (40.7 ± 6.0 mM) than in the TZ (37.5 ± 5.7 mM). Nine suspicious lesions (PI-RADS 4 and 5) were detected in eight men that were confirmed with Gleason scores of ≥ 3+3 by biopsy. TSC in prostate cancer (32.2 ± 5.5 mM) was significantly lower than in contralateral healthy regions (36.1 ± 3.9 mM, p=0.018).
**Conclusion:** Femoral blood vessels as an internal reference for TSC quantification are less prone to inaccuracies caused by B1 inhomogeneities as opposed to external sodium probes, which enables a robust quantification. TSC was significantly decreased within prostate cancer compared to healthy prostate tissue. Reduced TSC could represent a quantitative imaging biomarker that could improve prostate cancer risk stratification.
**Limitations:** Patients' blood sodium concentration assumed based on literature; only PI-RADS 4 and 5 lesions with histopathologic confirmation considered as confirmed prostate cancer lesions.
**Ethics committee approval:** The study was approved by the local ethics committee.
**Funding for this study:** This research project is part of the Research Campus M2OLIE and funded by the German Federal Ministry of Education and Research (BMBF) within the Framework "Forschungscampus: public-private partnership for Innovations" under the funding code 13GW0388A and 13GW0092D.
**Author Disclosures:**
Stefan Oswald Schönberg: Nothing to disclose
Anne Adlung: Nothing to disclose
Frank G. Zöllner: Nothing to disclose
Fabian Tollens: Nothing to disclose
Dominik Nörenberg: Nothing to disclose

### RPS 2007-8

**Qualitative comparison of conventional and deep-learning reconstructed diffusion-weighted magnetic resonance imaging sequence for prostate cancer imaging**

*Y. Lefebvre*[1], E. Venetis[1], T. Metens[1], T. Benkert[2], E. Weiland[2], M. A. Bali[1], N. Coquelet[1]; [1]Brussels/BE, [2]Erlangen/DE
*(yolene@compaqnet.be)*

**Purpose:** The goal of this study was to compares a conventional diffusion-weighted imaging (DWI-STD) to a faster DWI using deep-learning reconstruction strategies (DWI-DLR) for prostate cancer (PCa) imaging in terms of image quality (IQ) and diagnostic interpretation.
**Methods or Backround:** From February 2023 to July 2023, we query a retrospective monocentric database of patients with prostatic lesions who underwent a 3T magnetic resonance exam. Each patient successively underwent the DWI-DLR (acquisition time: 3 min38 s; research application) and the DWI-STD (4 min58s). Image analysis was performed by an expert radiologist in prostate imaging. Using high acquired b-value DWI images (800 s/mm2) and apparent diffusion coefficient (ADC), each sequence was rated using a 5-point Likert scale for overall IQ, noise, sharpness, contrast, artifacts and distortion. Lesion characteristics were assessed based on calculated b-value DWI (1400 s/mm2) and ADC maps, and rated for diagnostic confidence and detectability. Rating comparisons between the DWI sequences were performed using receiving operating characteristic curves and associated areas under the curve (AUC). P-values testing the null hypothesis that AUC equal 0.5 were computed and p-values below 0.05 Bonferroni-corrected for multiple comparisons were deemed significant.
**Results or Findings:** Twenty patients were included (mean age: 66.9 years, range: 58–80 years). We found that overall IQ (b800 and ADC); noise, sharpness and distortion (b800) were better for DWI-STD. Noise, sharpness and distortion (ADC); contrast and artifacts (b800 and ADC) were better for DWI-DLR. For lesions, diagnostic confidence and lesion detectability were better for DWI-DLR compared to DWI-STD. Significance was reached for contrast (b800 and ADC), noise and sharpness (ADC), and lesion detectability (b1400).
**Conclusion:** Our preliminary results show that PCa DWI can be acquired more rapidly using deep-learning reconstruction strategies without loss of IQ and diagnostic interpretation.
**Limitations:** No limitations were identified.
**Ethics committee approval:** The study is retrospective.
**Funding for this study:** No funding was received for this study.
**Author Disclosures:**
Thierry Metens: Nothing to disclose
Elisabeth Weiland: Nothing to disclose
Evangelis Venetis: Nothing to disclose
Maria Antonietta Bali: Nothing to disclose
Yolène Lefebvre: Nothing to disclose
Nicolas Coquelet: Nothing to disclose
Thomas Benkert: Nothing to disclose

### RPS 2007-9

**MRI radiomic model to predict prostate cancer of the anterior zone**

*S. Schirò*, L. Leo, M. Russomando, P. Canale, C. Zilioli, V. Casale, C. Roberti, C. Manna, N. Sverzellati; Parma/IT
*(silvia.schiro@unipr.it)*

**Purpose:** The purpose of this study was to assess the diagnostic performance of MRI radiomic model in the prediction of anterior zone prostate cancer.
**Methods or Backround:** This study included a retrospective monocenter dataset of subjects with prostate 1.5 T mpMRI showing an anterior zone lesion (PIRADS 3-5) and fusion target biopsy within six months. The histopathology results were the standard of reference. Two radiologists ( four years of experience) independently, reviewed and manually segmented the lesion with an open-source software at T2-weighted and ADC maps. Overall, 851 radiomics features (FR) were extracted for both T2-weighted and ADC maps. 100 train:test (0.7:0.3) splits were created and recursive feature elimination with a 5-fold cross-validation was performed on train partitions using the Random Forest Classifier (RFC). Subsequently, RFC was trained by selecting iteratively an increasing number of features sorted by their occurrences to evaluate the minimum number of informative features. Finally, means and 95% confidence intervals of accuracy, sensitivity, specificity, precision, area under the receiver operating characteristic curve (ROC-AUC) were calculated on the test partitions.
**Results or Findings:** Overall, 89 males (mean age 68 years; ±SD 8) were included. The anterior zone lesions were scored as follows: PI-RADS 3 (n=28, 31%), PI-RADS 4 (n=40, 45%) and PI-RADS 5 (n=21, 24%). Of these, 47/89 (53%) showed anterior zone prostate cancer (Gleason Score ≥ 3+3). The best model on the test set exploited six first-order features of ADC maps and three first-order features of T2-weighted images reaching accuracy, sensitivity, specificity, precision and ROC-AUC of 0.74 [95% C.I. 0.73-0.75], 0.72 [0.70-0.75], 0.77 [0.74-0.80], 0.80 [0.78-0.82], 0.82 [0.80-0.83], respectively.

**Conclusion:** The proposed radiomic model reached satisfactory performance in predicting anterior zone prostate cancer and may be a useful supportive tool in the diagnostic pathway.
**Limitations:** Monocentric retrospective cohort and no external validation
**Ethics committee approval:** This study was approved by CE AVEN : AVEN873/2018/OSS/UNIPR.
**Funding for this study:** No funding was received for this study.
**Author Disclosures:**
Marzia Russomando: Nothing to disclose
Paolo Canale: Nothing to disclose
Ludovica Leo: Nothing to disclose
Camilla Roberti: Nothing to disclose
Nicola Sverzellati: Nothing to disclose
Valentina Casale: Nothing to disclose
Carmelinda Manna: Nothing to disclose
Silvia Schirò: Nothing to disclose
Carlotta Zilioli: Nothing to disclose

### RPS 2007-10

**The role of in-bore prostate biopsy in the diagnosis of prostate cancer**
*C. Pizzi*, C. Sattin, S. Alessi, Q. Nguyen, G. Musi, G. Petralia; Milan/IT
*(caterina.pizzi@unimi.it)*

**Purpose:** The goal of this study was to evaluate the detection rates of prostate cancer (PCa) and clinically significant prostate cancer (csPCa) with in-bore MRI-guided prostate biopsy.
**Methods or Backround:** We considered in-bore MRI-guided prostate biopsies performed at our institution from November 2014 to January 2023, using two different systems: one manual and one robotic. Differences in detection rates of PCa and csPCa were assessed between the two systems and between patients with lesions ≤10 mm and >10 mm.
**Results or Findings:** Of the 831 biopsies included, 493 were positive for PCa and 281 for csPCa (59.3% and 33.8%). The detection rate of PCa and csPCa did not differ between manual and robotic systems (55.3% versus 63.2% and 33.2% versus 34.4%, respectively), neither for lesions ≤10 mm and >10 mm (32.9% versus 25.5% and 18.1% versus 16%, respectively).
**Conclusion:** The observed detection rates of PCa and csPCa (59.3% and 33.8%) agree with the literature. In the absence of significant differences in detection rates of PCa and csPCa, MRI-guided bore biopsy can be performed effectively on lesions of any size using either commercially available system (manual or robotic).
**Limitations:** Not applicable.
**Ethics committee approval:** This institutional review board approved study was a single-centre retrospective analysis, and the specific consent was waived for this audit of clinical procedures that had been performed with informed consent.
**Funding for this study:** No funding was provided for this study.
**Author Disclosures:**
Gennaro Musi: Nothing to disclose
Giuseppe Petralia: Nothing to disclose
Caterina Pizzi: Nothing to disclose
Sarah Alessi: Nothing to disclose
Quyen Nguyen: Nothing to disclose
Caterina Sattin: Nothing to disclose

### RPS 2007-11

**Ultrasound/ MRI fusion-guided transperineal laser ablation (TPLA) in the treatment of localised radiotherapy-resistant prostate cancer**
*B. Carreri*, P. E. Gigliotti, F. R. Fraioli, M. Nezzo, F. G. Garaci, G. Manenti; Rome/IT

**Purpose:** The aim of this prospective interventional study is assessing the feasibility, safety and treatment success of US/ MRI fusion-guided transperineal laser ablation (TPLA) as salvage treatment for radiation therapy refractory focal PCa, evaluating clinical and functional outcomes and defining post-procedural imaging findings using 3T multi-parametric MRI.
**Methods or Backround:** A cohort of five patients over 70 years old who had undergone RT as primary treatment, with a single, Gleason score ≤ 7 (3 + 4), local recurrence for PCa, underwent TPLA in outpatient setting (SoracteLite, ECHOLASER, Elesta). Post-ablation follow-up included regular PSA sampling and 3T mpMRI at one hour, three, six, 12, and 18 months and systematic and targeted ultrasound/MR fusion-guided biopsies at 18 months. International Prostate Symptom Score (IPSS) and the five-item version of the International Index of Erectile Function (IIEF-5) questionnaires were completed at baseline and at 18-month follow-up to investigate any procedure-related erectile dysfunction or urinary symptoms.
**Results or Findings:** All procedures were successfully completed with no significant complications (Clavien-Dindo Grade I). The procedure achieved optimal outcomes, with a statistically relevant reduction of PSA and ablation cavity volume trends at the end of follow-up (>70%). IIEF-5 and IPSS scores showed no significant difference between pre-procedural and 18 months values. Ultrasound/MRI fusion-guided biopsies at 18 months also confirmed the absence of recurrence.
**Conclusion:** Preliminary results demonstrated that TPLA can effectively and safely treat local recurrences of RT refractory PCa over a medium-term period, without side-effects and functional complications.
**Limitations:** The limitations of our study are that it’s single-centred and no preliminary sample size calculation was conducted. Instead, we only included a small cohort of patients who met our specific criteria, such as having received radiation therapy, having a low-risk score, no extraglandular extension.
**Ethics committee approval:** The study was approved the ethics committee: UID RS 68/19.
**Funding for this study:** No funding was provided for this study.
**Author Disclosures:**
Guglielmo Manenti: Nothing to disclose
Beatrice Carreri: Nothing to disclose
Paola Elda Gigliotti: Nothing to disclose
Francesco Giuseppe Garaci: Nothing to disclose
Marco Nezzo: Nothing to disclose
Federico Romeo Fraioli: Nothing to disclose

### RPS 2007-12

**Deep-learning-based image reconstruction of T2W images of the prostate and compressed sensing for one half of the acquisition time**
*L. Lambert*[1], M. Jurka[1], M. Wagnerová[1], O. Capoun[1], R. Jakubicek[2], P. Ourednicek[2], A. Burgetova[1]; [1]Prague/CZ, [2]Brno/CZ
*(lambert.lukas@gmail.com)*

**Purpose:** Deep-learning-based reconstruction (DLR) is being developed by major MRI vendors in order to improve image quality and accelerate image acquisition. We used DLR combined with compressed sensing (CS), a method of K-space subsampling, the aim was to accelerate acquisition of T2-weighted MRI of the prostate gland and maintain original image quality.
**Methods or Backround:** In this prospective study, forty-seven patients underwent biparametric prostate MRI with two T2 acquisitions in the transverse plane- a standard acquisition (4:27 min) and short acquisition (2:18) accelerated by increasing the CS factor from 1.3 to 2.8. The images were reconstructed with and without DLR. The image quality was rated in six domains, contrast-to-noise ratio and image sharpness were measured.
**Results or Findings:** The image quality of short-DLR was rated better in all categories compared to the standard sequence ($p<0.0001$ to $p=0.0044$). DLR images had higher sharpness compared to non-DLR. Both short and short-DLR images had lower calculated CNR. Subjective evaluation correlated both with prostate volume and image sharpness ($p<0.0001$).
**Conclusion:** The combination of DLR and CS results in accelerated acquisition of T2 images of the prostate with maintained perceived image quality, higher sharpness, and lower contrast-to-noise ratio.
**Limitations:** Study limitations: single center, single vendor, optimization of one sequence, technical level study.
**Ethics committee approval:** The study was approved by the Ethics Committee of the General University Hospital in Prague.
**Funding for this study:** This study was supported by the Ministry of Health of the Czech Republic (MH CZ-DRO, General University Hospital in Prague, 00064165) and by the institutional funding of the Charles University in Prague (Cooperatio, Medical Diagnostics and Basic Medical Sciences).
**Author Disclosures:**
Petr Ourednicek: Employee: Philips, Best, NL
Lukas Lambert: Nothing to disclose
Martin Jurka: Nothing to disclose
Andrea Burgetova: Nothing to disclose
Roman Jakubicek: Nothing to disclose
Otakar Capoun: Nothing to disclose
Monika Wagnerová: Nothing to disclose

### RPS 2007-13

**PSMA PET-based radiomic features for non-invasive discrimination of intraprostatic tumours**
*L. Luo*, R. Chang, Y. Li, Y. Liao, Z. Wang, X. Duan; Xi’an/CN
*(luoliang@stu.xjtu.edu.cn)*

**Purpose:** This study aims to investigate the utility of machine learning-based radiomics models derived from PSMA PET/CT images in differentiating between benign and malignant intraprostatic lesions detected by [18F]-PSMA-1007 PET/CT.
**Methods or Backround:** We retrospectively analyzed consecutive patients with prostate cancer (PCa) who underwent [18F]-PSMA-1007 PET/CT imaging and biopsy. A total of 1316 radiomic features were extracted from volumes of interest on PET and CT images respectively. Feature selection was performed using the Max-Relevance and Min-Redundancy (mRMR) algorithm and the least absolute shrinkage and selection operator (LASSO). Two radiomics models (PET model and PET/CT model) were generated using logistic regression in the training set. In addition, two baseline models were developed using clinical data including fPSA and tPSA, as well as prostate cancer molecular imaging evaluation standards (PROMISE), namely Clinical model and PROMISE model. ROC and Delong test were employed for model evaluation and comparisons.
**Results or Findings:** A total of 75 patients (50 with PCa and 25 with benign prostate hyperplasia) were included, with 53 patients for training and 22 for testing. In the training set, the area under curve (AUC) of the PET and PET/CT models were 0.94 (95% CI: 0.88-1.00) and 0.97 (95% CI: 0.92-1.00), respectively. The best-performing model (PET/CT model) demonstrated an accuracy of 92.5%, sensitivity of 100%, and specificity of 77.8%. Although the AUC of the PET/CT model was not significantly better than that of the PET model, it was significantly outperformed the Clinical model and PROMISE model (P < 0.001 and P = 0.002, respectively.).
**Conclusion:** Our findings highlight the potential clinical relevance of [18F]-PSMA-1007 PET-based radiomics models in the non-invasively prediction of intraprostatic lesions in patients with PCa, and show better diagnostic performance compared to baseline models.
**Limitations:** Not applicable
**Ethics committee approval:** Approval was granted by the Ethics Committee of the First Affiliated Hospital of Xi'an Jiaotong University.
**Funding for this study:** No funding was obtained for this study,
**Author Disclosures:**
Xiaoyi Duan: Nothing to disclose
Yunxuan Li: Nothing to disclose
Ruxi Chang: Nothing to disclose
Zhuonan Wang: Nothing to disclose
Liang Luo: Nothing to disclose
Yuting Liao: Nothing to disclose

**16:00-17:30** **Research Stage 1**

### Research Presentation Session: Artificial Intelligence & Machine Learning & Imaging Informatics

### RPS 2105

### Exploring the latest frontiers in machine learning techniques for MDCT advancements

**Moderator**
R. Mirón Mombiela; Herlev/DK
*(mirona@ufm.edu)*

### RPS 2105-2

**Coronary artery calcium scoring on the segment-level using deep active multi-task learning for time-efficient annotation**
*B. Föllmer*[1], S. Tsogias[1], F. Biavati[1], M. Bosserdt[1], K. F. Kofoed[2], P. Maurovich-Horvat[3], P. Donnelly[4], T. Benedek[5], M. Dewey[1]; [1]Berlin/DE, [2]Copenhagen/DK, [3]Budapest/HU, [4]Belfast/UK, [5]Targu Mures/RO
*(bernhard.foellmer@charite.de)*

**Purpose:** The aim of this study was to develop and evaluate a time-efficient annotation strategy for multi-task segment-level coronary artery calcium scoring (CACS) on non-contrast CT for the improvement of localisation and quantification of calcifications in the coronary artery tree.
**Methods or Backround:** This study included 1514 patients (mean age 60.0 ± 10.2 years, 55.7% female) with stable chest pain from the multicentre DISCHARGE trial (NCT02400229), which were randomly divided into a training/validation set (1514), and a test set (455). We developed a deep active learning strategy for time-efficient annotation of coronary artery segment-regions for auxiliary task learning in a multi-task model for segmentation of CACs on segment-level. We compared the model with a baseline U-Net in terms of micro-average sensitivity and micro-average specificity for assigning detected calcification to the correct segment and analysed interobserver variability in a subset of 150 patients.
**Results or Findings:** The micro-average sensitivity and micro-average specificity for assigning detected calcification to the correct segment improved from 0.581 (95% CI, 0.550-0.613) to 0.732 (95% CI, 0.711-0.754, p<0.001) and from 0.965 (95% CI, 0.962-0.968) to 0.978 (95% CI, 0.976-0.980), respectively (p<.001), compared to the baseline model, with an additional annotation time of less than 12 hours for annotation of coronary artery segment-regions. The agreement between the model and the reference standard (first observer) for segment class assignment was good with a weighted Cohen's κ of 0.806 (95% CI, 0.782-0.828) and only slightly lower compared to the second observer (weighted Cohen's κ of 0.819 [95% CI, 0.789-0.849] (p<.001).
**Conclusion:** Deep active learning can be used for time-efficient annotation of coronary artery regions to improve the performance of a multi-task model for segment-level CAC scoring.
**Limitations:** A consensus between the two observers that can serve as a reference standard was not available.
**Ethics committee approval:** The DISCHARGE trial was approved by the ethics committee at Charité–Universitätsmedizin Berlin as the coordinating center, by the German Federal Office for Radiation Protection, and by local or national ethics committees.
**Funding for this study:** This work was funded by the German Research Foundation through the graduate program BIOQIC (GRK2260, project-ID: 289347353), and the DISCHARGE project (603266-2, HEALTH-2012.2.4.-2) funded by the FP7 Program of the European Commission.
**Author Disclosures:**
Theodora Benedek: Grant Recipient: Grant from the European Commission, during the conduct of the DISCHARGE study; Grant Recipient: Grants from Romanian Ministry of European Funds; the Romanian Government and the European Union; and grants from Romanian Ministry of European Funds, the Romanian Government, and the European Union
Klaus F. Kofoed: Grant Recipient: Received financial support from Rigshospitalet, University of Copenhagen, during the conduct of the study. Grant Recipient: Received grants from AP Møller og hustru Chastine McKinney Møllers Fond, Danish Heart Foundation, The Danish Agency for Science, Technology and Innovation by The Danish Council for Strategic Research, Health Insurance Company Denmark. Grant Recipient: He additionally received unrestricted research grant from Canon Medical Corporation and GE Healthcare. Grant Recipient: Received funding from EU-FP7 Framework Program (DISCHARGE EU FP EC-GA 603266)
Pál Maurovich-Horvat: Speaker: Personal payment or honoraria for lectures, presentations, speakers bureaus, manuscript writing or educational events from Biotronik, Microport, Milestone Pharmaceutical, and Zoll. Grant Recipient: Grants or contracts through his institution from Abbott and Medtronic; Shareholder: Shares from Neumann Medical Ltd. J.L.M.; Consultant: Personal consulting fees from Medtronic and Sanofi
Marc Dewey: Author: Cardiac CT (Springer Nature). Other: Institutional research agreements: Siemens, General Electric, Philips, Canon. Patent Holder: Patent on fractal analysis of perfusion imaging (jointly with Florian Michallek, EPO 2022 EP3350773A1, and USPTO 2021 10,991,109, approved); Other: Hands-on cardiac CT courses (www.ct-kurs.de); Grant Recipient: EU (EC-GA 603266 in HEALTH.2013.2.4.2-2) DFG (DE 1361/14-1, DE 1361/18-1, BIOQIC GRK 2260/1, Radiomics DE 1361/19-1 [428222922] and 20-1 [428223139] in SPP 2177/1), GUIDE-IT (DE 1361/24-1), Berlin University Alliance (GC_SC_PC 27), Berlin Institute of Health (Digital Health Accelerator). Other: M.D. is European Society of Radiology (ESR) Publications Chair (2022-2025); the opinions expressed in this presentation are the author's own and do not represent the view of ESR.
Sotirios Tsogias: Nothing to disclose
Federico Biavati: Nothing to disclose
Maria Bosserdt: Grant Recipient: Received funding from EU-FP7 Framework Program (DISCHARGE EU FP EC-GA 603266).
Patrick Donnelly: Grant Recipient: Grant from the European Commission, during the conduct of the DISCHARGE study; Grant Recipient: Grants from Southeastern Health and Social Care Trust Innovation Research Development Group Fund.
Bernhard Föllmer: Nothing to disclose

### RPS 2105-3

**Auto-LSN: fully automated liver surface nodularity quantification for the diagnosis of advanced fibrosis in CT imaging**
*S. Yang*[1], A. Bône[2], T. Decaens[3], A. J. Glaunes[1]; [1]Paris/FR, [2]Villepinte/FR, [3]Grenoble/FR

**Purpose:** The liver surface nodularity score was proposed in 2016 to measure the irregularities of the surface of the liver in imaging and was shown to positively correlate with fibrosis, graded by the Metavir score. The LSN software requires the user to manually draw regions of interest before automatically segmenting the liver contour and measuring the score. We propose an entirely automated alternative (auto-LSN), and we compare its potential for the diagnosis of advanced fibrosis with LSN.
**Methods or Backround:** It was a monocentric retrospective study on portal phase CT images in patients with suspected primary hepatic tumors and an available Metavir score between 2015 and 2020. With respect to LSN, our auto-LSN method innovates on three key components: (i) automatic segmentation of the liver contour with deep learning, (ii) exclusion of low-contrast, high-curvature or inner regions of this liver contour, (iii) refinement and smoothing of the contours with Savitzky-Golay filter in the remaining regions of interest. The potential of auto-LSN to diagnose advanced fibrosis was measured with the area under curve (AUC), and compared with LSN, measured with the commercially available software by a radiologist (8 years of experience). The Delong's test was used for the comparison of AUCs.

**Results or Findings:** One hundred and two patients were included (88 males, age 72y ± 9.89),75 (74%) in the advanced fibrosis group (Metavir F3/F4). Auto-LSN and LSN scores were 2.64 (± 0.47) and 3.50 (± 0.86) respectively in the advanced fibrosis group, 2.20 (± 0.51) and 3.00 (± 0.67) in the other. The AUC of auto-LSN and LSN were 76% and 68%, respectively (p = 0.18).
**Conclusion:** The performance of auto-LSN and LSN to diagnose advanced fibrosis were similar. Auto-LSN has the advantage to be fully automated.
**Limitations:** Monocentric retrospective study.
**Ethics committee approval:** Not applicable for this study.
**Funding for this study:** This work is supported by a public grant overseen by the French National Research Agency (ANR) as part of the Investments for the Future programme (PIA) under grant agreement No. ANR-21-RHUS-01. This work is also funded by Guerbet.
**Author Disclosures:**
Alexis Joan Glaunes: Nothing to disclose
Sisi Yang: Employee: Guerbet
Alexandre Bône: Employee: Guerbet
Thomas Decaens: Nothing to disclose

### RPS 2105-4

**Assessing the accuracy of an AI-based coronary artery calcium score algorithm on non-gated chest CT images with varying slice thicknesses**
*D. Mu*[1], K. Yin[1], W. Chen[1], X. Chen[2], B. Zhang[1]; [1]Nanjing/CN, [2]Shanghai/CN

**Purpose:** This study aimed to evaluate the accuracy of an artificial intelligence-based coronary artery calcium score (AI-CACS) algorithm when applied to non-gated chest computed tomography (CT) images with varying slice width thickness.
**Methods or Backround:** A total of 112 patients who underwent both chest CT and simultaneous electrocardiogram (ECG)-gated non-contrast enhanced cardiac CT were prospectively enrolled. Different image thicknesses (1 mm, 3 mm, and 5 mm) were reconstructed from the same chest CT scan. The coronary artery calcium score (CACS) was obtained semi-automatically from ECG-gated cardiac CT scans using a conventional CAD method, serving as the reference (ECG-CACS). An AI-based algorithm was developed to automatically calculate CACS from non-gated chest CT images (AI-CACS). Agreement and correlation were assessed using Bland-Altman analysis and Spearman correlation coefficients. Risk stratification was also performed and compared.
**Results or Findings:** AI-CACS demonstrated strong correlations with ECG-CACS for the three different slice thicknesses (1 mm: 0.973, 3 mm: 0.941, 5 mm: 0.834; all p < 0.001). AI-CACS with a 1 mm slice thickness showed no statistically significant difference compared to ECG-CACS (p=0.085). The Bland-Altman plot revealed mean differences of -6.5, 15.4, and 53.1 for the AI-CACS 1 mm, 3 mm, and 5 mm groups, respectively, with 95% limits of agreement of -95.0 to 81.9, -96.6 to 127.4, and -187.8 to 294.0, respectively. Agreement of risk categories for CACS was measured by kappa (κ) values (AI-CACS-1mm: 0.868; AI-CACS-3mm: 0.772; AI-CACS-5 mm: 0.412; all p < 0.001), and the concordance rate was 91%, 84.8%, and 62.5%, respectively.
**Conclusion:** The AI-based algorithm proved to be feasible for calculating CACS from chest CT scans, with images having a 1mm slice width thickness yielding the best results.
**Limitations:** Only slice thicknesses of 1 mm, 3 mm, and 5 mm were evaluated in this study. A larger multi-centre, multi-vendor cohort study shall be conducted.
**Ethics committee approval:** This study was approved by the IRB with approval number: 2022-547-01.
**Funding for this study:** No funding was obtained for this study.
**Author Disclosures:**
Bing Zhang: Nothing to disclose
Kejie Yin: Nothing to disclose
Xingbiao Chen: Employee: Philips Healthcare
Wenping Chen: Nothing to disclose
Dan Mu: Nothing to disclose

### RPS 2105-5

**Deep learning decision-making research software prototype for automated inner ear classification**
*A. Kedves*[1], A. A. Almasri[2], S. Sugarova[3], A. Alsanosi[4], F. Almuhawas[4], L. Hofmeyr[5], F. Wagner[6], K. Sriperumbudur[1], A. Dhanasingh[1]; [1]Innsbruck/AT, [2]Pecs/HU, [3]St. Petersburg/RU, [4]Riyadh/SA, [5]Stellenbosch/ZA, [6]Bern/CH

**Purpose:** The aim of this study was to create an efficient DICOM viewer program, that automatically crops the inner ear, and classifies inner ear malformations (IEM), based on computed tomography (CT).
**Methods or Backround:** Retrospectively we evaluated 2053 patients from three hospitals and extracted 1200 inner ear CTs to import, crop, and the artificial intelligence (AI) to train, test, and validate. An automated cropping algorithm based on K-means clustering was created to crop the inner ear volume, along with a simple graphical user interface (GUI). Using the crops as an input, we created a deep learning convolution neural network (DL CNN) (5-fold cross-validation) to determine whether the inner ear anatomy is abnormal or normal (data equally distributed). Abnormal anatomy consists of cochlear hypoplasia, ossification, incomplete partition type I, incomplete partition type III, and common cavity (data equally distributed) were selected. Both the cropping tool and the AI model were validated.
**Results or Findings:** We developed an efficient research software prototype that can read CT files and crops a volume that contains the inner ear. Based on that volume of interest, the AI model makes the classification. The cropping was 92.25% accurate. The area under the curve (AUC) is 0.86 (95% CI: 0.81-0.91) on DL. Accuracy, precision, recall, and F1 scores are 0.812, 0.791, 0.8, and 0.766, respectively.
**Conclusion:** We present a fully automatised workflow of software development and validation tool. Our solution could provide good diagnostic accuracy during risk stratification; however, must be supervised by the decision-maker.
**Limitations:** The most important limitation to make the AI model more robust is the number of samples available at the time of this study.
**Ethics committee approval:** Approved bythe independent ethics committee of three hospitals IRB Nos. 22/0084/IRB, 23_001/IRB, and S_23_001/IRB, respectively.
**Funding for this study:** No funding was received for this study.
**Author Disclosures:**
Andras Kedves: Employee: MED-EL Medical Electronics GmbH.
Fida Almuhawas: Nothing to disclose
Kiran Sriperumbudur: Employee: MED-EL Medical Electronics GmbH.
Abdulrahman Alsanosi: Nothing to disclose
Abdulrahman Alkojak Almasri: Nothing to disclose
Louis Hofmeyr: Nothing to disclose
Franca Wagner: Nothing to disclose
Sima Sugarova: Nothing to disclose
Anandhan Dhanasingh: Employee: MED-EL Medical Electronics GmbH.

### RPS 2105-6

**Deep learning for estimating pulmonary nodule malignancy risk: how much data does AI need to reach radiologist level performance?**
*B. Obreja*[1], K. V. Venkadesh[1], W. Hendrix[1], Z. Saghir[2], M. Prokop[1], C. Jacobs[1]; [1]Nijmegen/NL, [2]Hellerup/DK
*(bogdan.obreja@radboudumc.nl)*

**Purpose:** Deep learning algorithms require large training datasets to achieve optimal performance. For many AI tasks, it is unclear whether algorithm performance would improve further if more training data was added. The aim of this study is to quantify the number of CT training samples required to achieve radiologist-level performance for a deep learning AI algorithm that estimates pulmonary nodule malignancy risk.
**Methods or Backround:** For estimating pulmonary nodule malignancy risk, we used the NLST dataset (malignant nodules:1249, benign nodules:14828) to train a deep learning algorithm. The dataset was split: 80% training and 20% internal validation. The algorithm was trained on random subsets of the training set with subset sizes ranging from 10% to 100%, with a class distribution of malignant≈7.77% and benign≈92.23%. The trained AI algorithms were validated on a size-matched cancer-enriched cohort (malignant:59, benign:118) from DLCST. The performance was compared against a group of 11 clinicians that also scored the test set, which included 4 thoracic radiologists.
**Results or Findings:** Using training data subsets of 10%, 20%, and 30%, the AI achieved AUC values of 0.74 (95%CI:0.67-0.82), 0.79 (95%CI:0.72-0.85), and 0.81 (95%CI:0.74-0.87) respectively. When the training data set size reached 60% (malignant:602, benign:7112), the performance saturated, reaching an AUC of 0.82 (95%CI:0.75-0.88). This was comparable to the average AUC of all clinicians (0.82,95%CI:0.77-0.86,p>0.99) and of the four thoracic radiologists (0.82,95%CI:0.74-0.89,p>0.99).
**Conclusion:** The AI was able to reach the level of an experienced thoracic radiologist when it was trained on 7714 nodules (malignant:602) from the NLST dataset. These findings have potential implications for the allocation of resources in developing deep learning algorithms for lung cancer medical imaging diagnostics.
**Limitations:** The generalizability of these findings is constrained by heterogeneity and geographical limitations of the datasets used in this study.
**Ethics committee approval:** This study included data collected from the National Lung Screening Trial (NLST) and the Danish Lung Cancer Screening Trial (DLCST). For NLST, IRB approval was obtained at all 33 participating sites and all participants provided informed consent. For DLCST, the Ethics Committee of Copenhagen County approved the study, and informed consent was obtained from all participants.
**Funding for this study:** Public private consortium with funding from NWO, Dutch Ministry of Economic Affairs, and MeVis Medical Solutions, Bremen, Germany.

**Author Disclosures:**
Mathias Prokop: Research/Grant Support: MeVis Medical Solutions AG, Bremen, Germany, and Siemens Healthineers, Erlangen, Germany, and Canon Medical Systems. Other: Royalties to institution from MeVis Medical Solutions AG, Bremen, Germany.
Kiran Vaidhya Venkadesh: Nothing to disclose
Zaigham Saghir: Nothing to disclose
Ward Hendrix: Nothing to disclose
Bogdan Obreja: Research/Grant Support: Dutch Science Foundation (NWO), Dutch Ministry of Economic Affairs, and MeVis Medical Solutions AG, Bremen, Germany.
Colin Jacobs: Other: Royalties to institution from MeVis Medical Solutions AG, Bremen, Germany; Research/Grant Support: MeVis Medical Solutions AG, Bremen, Germany and Philips Medical System, Best, Netherlands.

### RPS 2105-7

**SALT: Softmax for Arbitrary Label Trees**

*G. Baldini*[1], S. Koitka[1], C. M. Friedrich[2], J. Haubold[1], B. M. Schaarschmidt[1], M. Forsting[1], F. Nensa[1], R. Hosch[1]; [1]Essen/DE, [2]Dortmund/DE
*(giulia.baldini@uk-essen.de)*

**Purpose:** Segmentation networks treat anatomical structures as isolated entities, neglecting their inherent hierarchical relationships. We aimed to develop Softmax for Arbitrary Label Trees (SALT) that leverages these properties to enhance segmentation speed and interpretability.
**Methods or Backround:** This study proposes a segmentation method for CT-imaging that employs conditional probabilities to model the hierarchical structure of anatomical landmarks (such as, the lungs can be split in left/right and in upper/middle/lower lobe). This study utilizes 900 body region segmentations (883 patients) of the SAROS dataset from The Cancer Imaging Archive (TCIA). The TotalSegmentator was used to generate additional segmentations for a total of 117 labels. SALT was trained on 600 CTs, and 150 CTs were used for validation and testing. The model was evaluated on SAROS and on the TCIA dataset LCTSC using the Dice-Similarity-Coefficient (DSC).
**Results or Findings:** On the SAROS test set, the model obtained a DSC of 0.99 for abdominal and thoracic cavities, 0.98 for mediastinum, bones, and pericardium, 0.97 for muscles, 0.93 for subcutaneous tissue, 0.86 for brain, and 0.81 for spinal cord. On the LCTSC dataset, the model exhibited a DSC of 0.94 for right lung, 0.91 for left lung, 0.93 for both lungs, 0.89 for pericardium, 0.83 for spinal cord. Furthermore, SALT demonstrated remarkable computational efficiency, being capable of segmenting a whole-body CT in 20 seconds. This feature allows for its integration into clinical workflows, as a full-body segmentation could be automatically and efficiently computed whenever a CT scan is performed.
**Conclusion:** SALT used the hierarchical structures inherent in the human body to achieve high-quality segmentations while delivering exceptional speed. Additionally, this method also allows training using multiple, incompatible datasets.
**Limitations:** SALT was only evaluated on two TCIA datasets and its performance should be assessed on more datasets and conditions.
**Ethics committee approval:** Ethical approval was not required for this study, as it exclusively utilizes data already publicly available on The Cancer Imaging Archive (TCIA).
**Funding for this study:** This study did not receive external funding.
**Author Disclosures:**
Giulia Baldini: Nothing to disclose
Michael Forsting: Nothing to disclose
Benedikt Michael Schaarschmidt: Nothing to disclose
Johannes Haubold: Nothing to disclose
Christoph M. Friedrich: Nothing to disclose
Sven Koitka: Nothing to disclose
René Hosch: Nothing to disclose
Felix Nensa: Nothing to disclose

### RPS 2105-8

**Validation of an artificial intelligence software for automatic pulmonary nodule volumetry using micro-CT determined ground truth nodule volumes**

*L. D'hondt*[1], P-J. Kellens[1], K. Torfs[2], H. Bosmans[2], A. Snoeckx[3], K. Bacher[1]; [1]Ghent/BE, [2]Leuven/BE, [3]Antwerp/BE
*(louisdho.dhondt@ugent.be)*

**Purpose:** Validation of pulmonary nodule volumetry in clinically available software for automatic nodule detection and volumetry is currently either underrepresented or based on a consensus-driven ground truth in patient images, introducing uncertainties due to intrinsic structural differences between readers. Purpose of the study was to validate the nodule volumetry of a computer-aided detection (CAD) software using objective micro-CT determined ground truth nodule volumes.
**Methods or Backround:** Eighteen 3D-printed solid pulmonary nodules, including six diameters and three morphology classes, were subjected to high-resolution µCT scanning to establish objective ground truth volumes. The anthropomorphic Lungman phantom, containing the nodules, was scanned using a 256-slice CT scanner at three CTDIvol-values (6.04, 1.54, 0.20 mGy), and subsequently reconstructed with both iterative and deep learning image reconstruction, along with either soft or hard kernels. Volumetric accuracy of a commercially available automatic volumetry software (AVIEW LCS+) was assessed through multiple linear regression, identifying which predictors (reconstruction algorithm, kernel, dose, morphology, and diameter) significantly influence the outcome (% error).
**Results or Findings:** Volumes of nodules larger than six mm in diameter were accurate within 10% of their ground truth volume. Accuracy of volume measurements was significantly influenced by smaller and irregular morphologies ($p<0.001$). Notably, variation in reconstruction algorithm exerted no significant influence ($p>0.05$). Radiation dose and reconstruction kernel emerged as crucial parameters for accuracy. However, regression analysis showed diminished impact of the latter through significant interaction with nodule characteristics, resulting in measured volumes closer to the real volumes. Considerable discrepancies were observed between objective pulmonary nodule volumes and volumes determined through consensus readings.
**Conclusion:** Robustness of the volumetry software for variations in CT acquisition parameters in a phantom makes it a valuable candidate application for diverse imaging protocols across multiple centers.
**Limitations:** This is a phantom study.
**Ethics committee approval:** No ethics committee approval was needed, since this study used phantom images.
**Funding for this study:** Funding was provided by the FWO “Kom op tegen Kanker”-project for lung cancer screening research in Belgium. (Project number: G0B1922N).
**Author Disclosures:**
Klaus Bacher: Nothing to disclose
Kwinten Torfs: Nothing to disclose
Louise D'hondt: Nothing to disclose
Annemiek Snoeckx: Nothing to disclose
Hilde Bosmans: Nothing to disclose
Pieter-Jan Kellens: Nothing to disclose

### RPS 2105-9

**AI-assisted detection of abdominal pathologies in chest CT scans**

E. Petrash, M. Dugova, R. Gareeva, E. Kochina, A. Shevtsov, V. Samokhin, F. Yaushev, *M. Belyaev*; London/UK
*(m.belyaev@ira-labs.com)*

**Purpose:** Amid intensified focus on chest pathologies, abdominal findings on chest CT scans may be overlooked. Detecting non-alcoholic fatty liver disease (NAFLD) is crucial due to its hepatocellular carcinoma risk. Early identification of liver, kidney, and adrenal incidentalomas aids in timely cancer interventions. Swift urolithiasis recognition prevents ureteral complications. Identifying aortic aneurysms is vital due to its high mortality. Our research assesses an AI solution's efficacy in enhancing these detection rates on chest CT.
**Methods or Backround:** The retrospective study used data from the national lung screening trial, consisting of 2408 chest CTs from primary patients. We excluded 130 cases with a pathology below the diaphragm mentioned in the report. The remaining 2278 CTs without described abdominal findings were auto-analysed by a comprehensive AI product, AUCT-Abdomen, which detects adrenal mass, NAFLD, liver & kidney lesions, urolithiasis, and abdominal aortic dilatation. CTs with AI findings were then independently reviewed by two radiologists with 12 and 14 years of experience.
**Results or Findings:** AI identified 270 (11.2%) previously unreported patients on top of 130 (5.3%) reported initially. AI findings include 125 adrenal mass, 21 NAFLD, 68 liver lesions, 17 kidney lesions, 5 urolithiasis, 20 aortic dilatation, and 5 aortic aneurysm. AI results were false positives in 17 (0.7%) cases.
**Conclusion:** Integrating AI into the radiological evaluation of chest CT scans may ensure the thorough detection of abdominal incidental findings, including adrenal mass, hepatic steatosis, liver and kidney lesions, urolithiasis, aortic dilatation and aneurysm. Our research may highlight a potential deprioritisation of findings peripheral to the chest and the potential of AI in bridging this diagnostic gap. The potential benefits from deploying AI products for incidental abdominal findings may be exceptionally high for departments with strictly limited turnaround time.
**Limitations:** No limitations were identified for this study.
**Ethics committee approval:** The study is retrospective so no ethical approval was sought.
**Funding for this study:** Funding for this study was provided by AUMI AI Limited.

**Author Disclosures:**
Alexey Shevtsov: Employee: AUMI AI Limited
Regina Gareeva: Employee: AUMI AI Limited
Evgeniya Kochina: Employee: AUMI AI Limited
Mariya Dugova: Employee: AUMI AI Limited
Mikhail Belyaev: Founder: AUMI AI Limited
Valentin Samokhin: Employee: AUMI AI Limited
Ekaterina Petrash: Employee: AUMI AI Limited
Farukh Yaushev: Employee: AUMI AI Limited

### RPS 2105-10

**Fully automatic AI-driven assessment in coronary CT angiography for intermediate stenosis: a comparative study with quantitative coronary angiography and fractional flow reserve**

*J-I. Jo*, H. J. J. Koo, J-W. Kang, D. H. Yang; Seoul/KR
*(sweetjjo86@gmail.com)*

**Purpose:** Limited data exists on direct comparison of AI-driven automatic coronary stenosis assessment in coronary CT angiography (CCTA) to quantitative coronary angiography (QCA). This study aims to compare AI-based coronary stenosis evaluation in CCTA with its quantitative counterpart of coronary angiography and invasive fractional flow reserve (FFR).
**Methods or Backround:** In this single-center retrospective study, 215 intermediate coronary lesions, with QCA diameter stenosis between 20% and 80%, were assessed from 195 symptomatic patients (mean age 61 years, 149 men, 585 coronary arteries). For stenosis quantification in CCTA, an AI-driven research prototype (Siemens Healthineers, Germany) was used (AI-CCTA). Diagnostic accuracy of AI-CCTA on per-vessel basis was assessed, using invasive coronary angiography stenosis grading (with > 50% stenosis) or invasive FFR (< 0.80) as reference standards. AI-driven diameter stenosis was then correlated with QCA results and expert manual measurements.
**Results or Findings:** Among 585 coronary arteries, disease prevalence as determined by invasive angiography (≥ 50%) was 46.5%. AI-CCTA showed sensitivity of 71.7%, specificity of 89.8%, positive predictive value of 85.9%, negative predictive value of 78.5%, and area under the curve (AUC) of 0.81. For 215 intermediate lesions assessed using QCA and FFR, diagnostic performance of AI-CCTA was moderate, with AUC of 0.63 for both QCA and FFR. In measuring degree of stenosis, AI-CCTA demonstrated a moderate correlation with QCA ($r = 0.42$, $p < 0.001$), which was notably better than results from manual quantification versus QCA ($r = 0.26$, $p=0.001$).
**Conclusion:** The AI-powered automated CCTA analysis showed promising results when compared to invasive angiography. While AI-CCTA demonstrated a moderate relationship with QCA in intermediate coronary stenosis lesions, its results appeared to surpass those of manual evaluations.
**Limitations:** Individuals with stents or a history of coronary-artery bypass grafting were excluded from analysis.
**Ethics committee approval:** Approval from the Institutional Review Board of Asan Medical Center, Seoul, Korea
**Funding for this study:** No funding was provided for this study.
**Author Disclosures:**
Joon-Won Kang: Nothing to disclose
Dong Hyun Yang: Nothing to disclose
Hyun Jung J. Koo: Nothing to disclose
Jung-In Jo: Nothing to disclose

### RPS 2105-11

**Artificial intelligence-based non-contrast-enhanced CT images for diagnosis of hepatic lesions: a multicentre study**

*Z. Ma*, L. Jin, M. Li; Shanghai/CN
*(zxma21@m.fudan.edu.cn)*

**Purpose:** We aimed to realise the identification and classification diagnosis of intrahepatic space-occupying lesions on non-contrast enhanced CT (NCCT).
**Methods or Backround:** In this retrospective study, patients who suspected with space-occupying lesions in liver undergoing both NCCT and enhanced CT/MRI multi-phase enhancement from January 2017 to March 2023 in our hospital and from January 2020 to August 2023 in another medical center. Each liver lesions in NCCT were confirmed by enhanced CT/MRI multi-phase enhancement or pathology as the golden reference. The lesion contours of the patients in NCCT images were manually delineated by radiologists, and radiomics features were extracted in 3D Slicer. An automatic machine learning algorithm was used to screen out the most relevant radiomics features and establish a classification model for differential diagnosis to classify type of intrahepatic space-occupying lesions.
**Results or Findings:** A total of 252 liver lesions in 230 patients from our hospital including 79 hepatic cysts, 81 haemangiomas, 52 malignant tumours and 40 liver abscesses. A total of 33 liver lesions in 230 patients from another medical centre including 12 hepatic cysts, 8 haemangiomas, 8 malignant tumours and 5 liver abscesses. The sensitivity of the nnDetection to detect the lesion was 0.81, the AUC of classification model for lesions were 1.0 (hepatic cysts), 0.99 (hemangiomas), 0.9 8(malignant tumours) and 0.98 (liver abscesses) in our internal test dataset while the AUC were 1.0 (hepatic cysts), 0.97 (haemangiomas), 0.9 3 (malignant tumours) and 0.92 (liver abscesses) in outside test dataset.
**Conclusion:** Our proposed model showed better performance of the identification and classification diagnosis of intrahepatic space-occupying lesions on NCCT.
**Limitations:** The small sample size may lead the bias of this study.
**Ethics committee approval:** The study was approved by Huadong hospital (reference number: 20230089).
**Funding for this study:** No funding was received for this study.
**Author Disclosures:**
Ming Li: Nothing to disclose
Liang Jin: Nothing to disclose
Zhuangxuan Ma: Nothing to disclose

**16:00-17:30** **Research Stage 2**

### Research Presentation Session: Abdominal Viscera & GI Tract

### RPS 2101

### Acute abdominal pathologies: diagnosis and prediction

**Moderator**
M. Scharitzer; Vienna/AT
*(martina.scharitzer@meduniwien.ac.at)*

### RPS 2101-2

**Dual-Energy CT of acute bowel ischaemia: influence on diagnostic accuracy and reader confidence**

*M. Oberparleiter*, J. Vosshenrich, H-C. Breit, B. Friebe, D. Harder, D. Boll, C. J. Zech, M. Obmann; Basel/CH
*(moritzoberparleiter@gmail.com)*

**Purpose:** Current guidelines do not recommend the use of Dual-Energy CT (DECT) for suspected acute mesenteric ischaemia due to a lack of clinical studies. The purpose of this study was to investigate the diagnostic accuracy, reader confidence and reading time of DECT compared to conventional CT.
**Methods or Backround:** 25 patients with surgically proven acute mesenteric ischaemia and 25 gender- and age-matched controls, who underwent arterial and portal venous phase DECT of the abdomen were included in this retrospective study. Two fellowship-trained abdominal radiologists evaluated all cases with and without the use of DECT-derived virtual non-contrast images and iodine maps for mesenteric ischaemia. Reading time was recorded and diagnostic confidence was rated on a 10-point Likert scale. The inter-reader agreement was assessed using Cohen's kappa. Sensitivity and specificity were compared using McNemar's test, reading time and reader confidence with the Wilcoxon rank-sum test.
**Results or Findings:** Inter-reader agreement was good ($\kappa=0.72$). Sensitivity and specificity for diagnosing acute mesenteric ischaemia were 78% and 100% using conventional image data alone. Utilising additional DECT data, sensitivity was significantly higher at 94% ($p=0.02$), while specificity remained 100%. Diagnostic confidence increased significantly from 8 (IQR, 7-10) to 9 (IQR, 8-10) ($p<0.01$). Mean reading time per case increased significantly from 154 to 183 s ($p=0.02$) using additional DECT images.
**Conclusion:** Additional use of DECT should be considered when examining for suspected acute mesenteric ischaemia as DECT increased reader diagnostic accuracy and confidence for mesenteric ischaemia with only a moderate increase in reading time.
**Limitations:** Only two DECT scanner types were used in this study and results may not be transferable to other DECT platforms. Readers who are familiar with DECT technology may benefit while inexperienced readers may benefit less from DECT images.
**Ethics committee approval:** The need for informed consent was waived due to the retrospective nature of this study.
**Funding for this study:** This research received no specific grant from any funding agency in the public, commercial or non-profit sectors.
**Author Disclosures:**
Markus Obmann: Nothing to disclose
Hanns-Christian Breit: Nothing to disclose
Björn Friebe: Nothing to disclose
Christoph Johannes Zech: Nothing to disclose
Daniel Boll: Nothing to disclose
Dorothee Harder: Nothing to disclose
Jan Vosshenrich: Nothing to disclose
Moritz Oberparleiter: Nothing to disclose

### RPS 2101-3

**Hit and miss: locating the exact site of gastrointestinal tract perforations as a challenge**

*T. B. Plojović*, A. Pavlović, D. Marković, D. Janjic, J. Vukmirovic, K. Lazarevic, B. Jovandić, D. Vasin, S. Hasanagic; Belgrade/RS
*(tarik95@hotmail.rs)*

**Purpose:** The objective of our study was to analyse the capacity of CT (Computerized Tomography) to identify the site of gastrointestinal perforation (GI) and to determine which radiological signs, either direct or indirect, are the most predictive.
**Methods or Backround:** Between September 2022 and September 2023, we retrospectively studied 100 patients presenting with pneumoperitoneum on CT. All patients had surgically proven gastrointestinal tract perforation. Two expert radiologists, with no previous knowledge of the clinical histories or the surgical results, evaluated the CT scans.
**Results or Findings:** The locations of the perforations found during surgery in the 100 patients were as follows: 36 stomach or duodenum; 15 small intestine; 12 appendix; 16 ascending, transverse or descending colon; and 21 sigma/rectum. The Kappa correlation coefficient between radiologists for predicting the localisation of the perforation in our study was high. The two most frequent signs observed in our study were free extraluminal air in the supramesocolic space and gas bubbles adjacent to the wall. The prediction of the perforation site in the gastrointestinal tract using CT coincided with the surgical findings in 80 out of 100 patients. In 20 patients, the prediction did not concur with the findings. In 15 cases, CT identified an incorrect perforation site while in 5 patients, CT did not identify the location of the GI perforation. The most sensitive sign in our study was the presence of free extraluminal air in the supramesocolic compartment. The most specific ones were the presence of abscesses and focal wall defects.
**Conclusion:** CT can locate gastrointestinal perforation sites with a high sensitivity and excellent interobserver correlation.
**Limitations:** This was a retrospective study.
**Ethics committee approval:** This study was approved by the Institutional Ethics Committee.
**Funding for this study:** No funding was received for this study.
**Author Disclosures:**
Katarina Lazarevic: Nothing to disclose
Aleksandar Pavlović: Nothing to disclose
Danilo Marković: Nothing to disclose
Sanela Hasanagic: Nothing to disclose
Doroteja Janjic: Nothing to disclose
Dragan Vasin: Nothing to disclose
Tarik Binasa Plojović: Nothing to disclose
Biljana Jovandić: Nothing to disclose
Jelica Vukmirovic: Nothing to disclose

### RPS 2101-4

**CT prognostic signs in emergency surgery for acute obstructive colonic cancer (AOCC)**

F. Pezzullo[1], S. Tamburrini[1], *R. Comune*[1], R. D'avino[1], C. Liguori[1], M. Scaglione[2], F. Tamburro[1]; [1]Naples/IT, [2]Sassari/IT
*(ros.comune@gmail.com)*

**Purpose:** This study aimed to identify CT prognostic signs of poor outcomes of emergency surgery in AOCC.
**Methods or Backround:** Demographic, clinical, laboratory, radiological and surgical data of 65 consecutive patients with AOCC who underwent emergency surgery were analysed. CTs were reviewed to assess the diameters of the cecum, ascending, transverse, descending, and sigmoid proximal to the tumour. Furthermore, colon segments' CD/L1-VD ratios, continence of the ileocecal valve, small bowel over-distension, presence of faecal signs and cecal pneumatosis were also analysed. Postoperative complications, according to Clavien-Dindo classifications, were used.
**Results or Findings:** Preoperative transverse and descending colon CD/L1-VD ratios were significantly associated with the development of postoperative complications with a cut-off value of >/=1.4 and 1.3 (p=0.157 and p=0.008 - Clavien-Dindo classification major - grade ≥III-V), respectively. Postoperative complications within 30 days after surgery occurred in 18/65 patients, with 12 patients developing surgical complications (18.5%), 3 patients developing medical complications (4.6%) and 3 patients dying (4.6%). Of the 18 patients, 15 (23,1%) developed severe complications (grade ≥III-V). A cecum distension >/= 9 cm represented the critical dimension beyond which perforation and cecal necrosis were found at surgery (11/65 patients). Cecal pneumatosis was detected in 5/11 patients.
**Conclusion:** CT is a valid tool to select patients at higher risk of complications. A CD/L1-VD ratio with cut-off values of 1.4 (transverse) and 1.3 (descending) predicted major complications (grade ≥III-V). A cecum diameter >/= 9 cm and continence of ileo cecal valve were predictive factors of poor outcome and cecal necrosis. The CT sign of cecal pneumatosis was not pathognomonic for cecal necrosis.
**Limitations:** This was a retrospective study and prospective multicenter studies are needed to validate our results.
**Ethics committee approval:** This study was approved by the local Institutional Review Board. The Ethical Committee approval was obtained (2022030). Patient consent was waived (Retrospective Observational Study).
**Funding for this study:** No funding was received for this study.
**Author Disclosures:**
Stefania Tamburrini: Nothing to disclose
Filomena Pezzullo: Nothing to disclose
Fabio Tamburro: Nothing to disclose
Mariano Scaglione: Nothing to disclose
Carlo Liguori: Nothing to disclose
Rosita Comune: Nothing to disclose
Raffaele D'avino: Nothing to disclose

### RPS 2101-5

**(HU)nt for truth: psoas muscle evaluation on CT imaging predicts 30-day survival in variceal bleed patients**

*J. Krawczyk*, D. Leon, L. D. Tyson, T. Haq, D. Saba, R. H. Thomas, A. Dhar; London/UK
*(jejabig.psn@gmail.com)*

**Purpose:** This study aimed to assess if sarcopenia defined on cross-sectional imaging predicts 30-day mortality in the setting of variceal bleeding, independent of baseline liver disease severity.
**Methods or Backround:** A retrospective study was performed of all patients with decompensated cirrhosis admitted with a variceal bleed over a 24-month period to our tertiary centre. CT imaging acquired within 3 months of admission was reviewed to determine psoas muscle thickness, width, area and Hounsfield Units (HU: a surrogate measure of psoas density) at the level of the inferior L4 endplate. The area of the psoas was calculated using a generic lesion segmentation tool and the average HU value was calculated from circular ROI in the area of the muscle. Association with 30-day mortality was tested by univariate and multivariate binary logistic regression; and the utility of identified prognostic biomarkers by AUROC analyses (SPSS v 27).
**Results or Findings:** 104 patients with decompensated cirrhosis were identified with variceal bleed episodes. 15/104 died within 30 days. As expected, a higher MELD score was associated with higher mortality (OR 1.106, 95% CI 1.034-1.183, P=0.003). Higher Psoas HU and PMTH were both associated with lower mortality, independent of MELD (Adjusted OR 0.891, 0.821-0.967, P=0.006; AOR 0.810, 0.665-0.987, P=0.036 respectively). Psoas HU was predictive of 30-day survival (AUROC 0.790, 0.673-0.907, P=0.004) as was PMTH (AUROC 0.743, 0.571-0.914, P=0.017), and MELD (AUROC 0.719, 0.534-0.904, P=0.008).
**Conclusion:** Psoas HU and PMTH are negatively associated with mortality in patients with decompensated cirrhosis and variceal bleeding. Crucially, this relationship appears independent of baseline liver disease severity (MELD score).
**Limitations:** Larger studies are needed to confirm the association of Psoas HU and PMTH with 30-day mortality and to further define the role of these parameters as prognostic indexes in this clinical setting.
**Ethics committee approval:** No information provided by the submitter.
**Funding for this study:** No funding was received for this study.
**Author Disclosures:**
Jędrzej Krawczyk: Nothing to disclose
Dylan Leon: Nothing to disclose
Robert Hywel Thomas: Nothing to disclose
Ameet Dhar: Nothing to disclose
Luke David Tyson: Nothing to disclose
Taha Haq: Nothing to disclose
Dana Saba: Nothing to disclose

### RPS 2101-6

**Diagnostic impact of AAST injury scale in the assessment of hepatic traumatic lesions**

*C. Maino*, C. Talei Franzesi, M. Ragusi, D. G. Gandola, T. P. Giandola, P. N. Franco, D. Ippolito; Monza/IT
*(mainocesare@gmail.com)*

**Purpose:** This study aimed to determine the diagnostic impact of computed tomography in the evaluation and management of patients with traumatic liver lesions following the American Association for Surgery of Trauma (AAST) scale and its relation to clinical and laboratory data.
**Methods or Backround:** A total of 103 hemodynamically stable patients, with traumatic liver injury, who underwent contrast-enhanced CT scan to assess and quantify liver damage, were enrolled. Imaging data were independently evaluated by a general surgeon and a radiologist (both with more than 15 years of experience). The reviewers first graded liver lesions, according to the AAST scale, blinded to the clinical data. During the second revision session, the reviewers reconsidered the CT findings along with the support of clinical data. The primary study outcome was to determine the patient's management

[operative (OM) or not-operative (NOM)] based exclusively on imaging CT findings and by adding laboratory data.
**Results or Findings:** A good inter-reader agreement was found for AAST grades I, II, III, and V (k= 0.870, k=0.880, k=0.900, and k=1); while in grade IV the agreement was fair (k=0.455). According to the first revision section, the accuracy in determining the management was higher for the radiologist (AUC=0.850, 95% CI 0.770-0.950) than the surgeon (AUC=0.700 95% CI 0.550-0.820), achieving a statistically significant difference (p=0.025). During the second revision session, after correlation with clinical and laboratory data, the overall accuracy between the two readers was statistically comparable (AUC=0.880 and AUC=0.850, p>0.05).
**Conclusion:** The CT liver damage score, according to the AAST scale, represents a useful and fast approach to correctly address the management of liver trauma patients.
**Limitations:** This was a retrospective study.
**Ethics committee approval:** The study was retrospective.
**Funding for this study:** No funding was received for this study.
**Author Disclosures:**
Davide Giacomo Gandola: Nothing to disclose
Cesare Maino: Nothing to disclose
Maria Ragusi: Nothing to disclose
Teresa Paola Giandola: Nothing to disclose
Paolo Niccolò Franco: Nothing to disclose
Davide Ippolito: Nothing to disclose
Cammillo Talei Franzesi: Nothing to disclose

### RPS 2101-7

### Early IVIM-DWI for the prediction of post-pancreatectomy acute pancreatitis

*L. Fortuna*, L. Costa, B. Maris, G. Zamboni; Verona/IT
*(lucafortuna96@gmail.com)*

**Purpose:** This study aimed to evaluate early DW-MRI radiological findings and texture analysis parameters that will predict the development of post-pancreatectomy acute pancreatitis (PPAP) in patients undergoing pancreaticoduodenectomy.
**Methods or Backround:** In this IRB-approved prospective study, 65 patients underwent MRI on the third postoperative day after pancreaticoduodenectomy. Scan protocol included standard sequences, post-gadolinium acquisitions, and IVIM-DWI. IVIM DICOM images were analysed with in-house software that produced F, D, and D* maps and allowed us to calculate texture parameters of three different ROIs (stump, tail, entire pancreatic remnant). By retrospectively applying the 2021 ISGPS definition of PPAP, patients were defined as with or without POH/PPAP. Texture parameters and radiological findings were compared between the two groups (Kruskal-Wallis, ANOVA tests).
**Results or Findings:** The patient population included 33 females and 32 males. 20 patients developed postoperative hyperamylasemia (POH) and 6 of these grade B or C PPAP. Significant differences in texture parameters were identified between the POH/PPAP and the non-POH/PPAP groups for mean ADC (1.33±0.22 vs 1.56±0.28) × (10−3 mm2/□; p=0.006) a□d D valu□ (0.11±0.14 vs 0.22±0.15; p=0.03), F entropy (4.5±0.10 vs 4.1±0.08 (SE); p=0.004) and D* entropy (2.7±0.73 vs 1.3±0.38 (SE); p=0.01) of ROIs including the pancreatic stump. Similar results were found evaluating ROIs of the tail (mean D, p=0.01; entropy F, p=0.02; entropy D*, p=0.01) and entire pancreatic remnant (mean D, p=0.01; entropy F, p=0.0001; entropy D*, p=0.0001). No macroscopic features consistent with PPAP were identified.
**Conclusion:** Early postoperative MRI texture analysis of IVIM-derived parameters might predict who will develop PPAP after pancreaticoduodenectomy.
**Limitations:** The limitations of the study are the limited sample size, the difference in slice thickness between post-contrast T1 images and those in DWI IVIM; the exclusion of patients unable to perform MRI due to early clinical worsening; and POH and PPAP groups union.
**Ethics committee approval:** This study was approved by the Ethics Committee. The notification can be found under the number 2130 CESC VR-RO.
**Funding for this study:** No funding was provided for this study.
**Author Disclosures:**
Giulia Zamboni: Nothing to disclose
Luca Fortuna: Nothing to disclose
Bogdan Maris: Nothing to disclose
Lorenzo Costa: Nothing to disclose

### RPS 2101-8

### Time dependency and risk factors of splanchnic vein thrombosis development in the early phase of acute pancreatitis: a systematic review and meta-analysis

*Z. Z. Borbély Ruben*[1], B. M. Philip[1], E. Á. Szalai[1], B. Gellért[1], D. Veres Sandor[1], B. Teutsch[1], B. Erőss[1], P. Hegyi[1], N. Faluhelyi[2]; [1]Budapest/HU, [2]Pécs/HU
*(drborbelyruben@gmail.com)*

**Purpose:** Splanchnic vein thrombosis (SVT) is a local complication of acute pancreatitis (AP) that may lead to subsequent complications such as portal hypertension, gastrointestinal bleeding, and mesenteric ischemia. This study aimed to analyse the temporal progression and contributing risk factors of SVT occurrence during the early phase of AP.
**Methods or Backround:** We systematically searched medical databases (Embase, MEDLINE via PubMed, Scopus, and CENTRAL) on 27.10.2022. Inclusion criteria were studies using appropriate diagnostic modalities to identify SVT in patients from the early phase of AP. We performed a random-effects meta-analysis, calculated SVT-affected patient proportions with 95% confidence intervals (CI) and conducted subgroup analyses. The protocol was prospectively registered in PROSPERO: CRD42022367578.
**Results or Findings:** The proportion of patients with SVT within 12 days after symptom onset was 0.13 (CI 0.07-0.23). The occurrence was lowest at 0.06 (CI 0.03-0.1) between 0-3 days after symptom onset and increased fourfold to 0.23 (CI 0.16-0.31) between 3-11 days. The proportion of patients affected on hospital admission was 0.12 (CI 0.02-0.49), and it was 0.17 (CI 0.03-0.58) 1-5 days after admission. Alcoholic aetiology (0.31, CI 0.13-0.58) and pancreatic necrosis (0.55, CI 0.29-0.78, necrosis above 30%) correlated with increased SVT prevalence.
**Conclusion:** The risk of developing SVT is significant in AP, affecting up to a quarter of patients. The risk of occurrence increases with time in the early stages of AP. Alcoholic aetiology and pancreatic necrosis elevate the risk for SVT in AP. Our findings highlight the need for anticoagulation therapy and advanced imaging (CT, MRI) to become a routine component of AP therapy.
**Limitations:** The limitations were the lack of individual patient data and aggregate data from published studies limited our ability to control for potential confounders and explore effect modifiers beyond subgroup analyses.
**Ethics committee approval:** No ethical approval was required for this systematic review with meta-analysis, as all data were already published in peer-reviewed journals. No patients were directly involved in the design, conduct, or interpretation of our study.
**Funding for this study:** The research was supported by the Hungarian Ministry of Innovation and Technology, National Research, Development and Innovation Fund (TKP2021-EGA-23 to PH), Translational Neuroscience National Laboratory program (RRF-2.3.1-21-2022-00011 to PH), a project grant (K131996 to PH) and the Translational Medicine Foundation. Funding for Brigitta Teutsch was provided by the ÚNKP-22-3 New National Excellence Program of the Ministry for Innovation and Technology from the source of the National Research, Development and Innovation Fund (to BT - ÚNKP-22-3-I-PTE-1693). The funders did not affect the concept, data collection, analysis, or writing of the manuscript.
**Author Disclosures:**
Bálint Erőss: Nothing to disclose
Nándor Faluhelyi: Nothing to disclose
Péter Hegyi: Nothing to disclose
Bálint Gellért: Nothing to disclose
Bryan Mangalath Philip: Nothing to disclose
Zsolt Zsolt Borbély Ruben: Nothing to disclose
Eszter Ágnes Szalai: Nothing to disclose
Brigitta Teutsch: Nothing to disclose
Daniel Veres Sandor: Nothing to disclose

### RPS 2101-9

### Extracellular volume fraction derived from dual-energy CT: a potential predictor for postpancreatectomy acute pancreatitis after pancreatoduodenectomy

*X. Bai*, J. Yin, H. Shi, K. Jiang, Q. Xu; Nanjing/CN
*(xiaohan_bai98@163.com)*

**Purpose:** This study aimed to investigate the value of extracellular volume (ECV) fraction and fat fraction (FF) derived from dual-energy CT (DECT) for predicting postpancreatectomy acute pancreatitis (PPAP) after pancreatoduodenectomy (PD).
**Methods or Backround:** This retrospective study included patients who underwent DECT and PD between April 2022 and September 2022. PPAP was determined according to the International Study Group for Pancreatic Surgery (ISGPS) definition. Iodine concentration (IC) and fat fraction of the pancreatic parenchyma were measured on preoperative DECT. The ECV fraction was calculated from iodine map images of the equilibrium phase. The independent predictors for PPAP were assessed by univariate and multivariate logistic regression analyses and receiver operating characteristic (ROC) curve analyses.

**Results or Findings:** 69 patients were retrospectively enrolled (median age, 60 years; interquartile range, 55-70 years; 47 men). Of these, nine patients (13.0%) developed PPAP. These patients had lower portal venous phase IC, equilibrium phase IC, FF and ECV fraction, compared with patients without PPAP. After multivariate analysis, ECV fraction was independently associated with PPAP (odd ratio [OR], 0.87; 95% confidence interval [CI]: 0.79, 0.96; p<0.001), with an area under the curve (AUC) of 0.839 (sensitivity 100.0%, specificity 58.3%).
**Conclusion:** A lower ECV fraction is independently associated with the occurrence of PPAP. ECV fraction may serve as a potential predictor for PPAP.
**Limitations:** No limitations were identified.
**Ethics committee approval:** No information provided by the submitter.
**Funding for this study:** No funding was received for this study.
**Author Disclosures:**
Qing Xu: Nothing to disclose
Hongyuan Shi: Nothing to disclose
Xiaohan Bai: Nothing to disclose
Jie Yin: Nothing to disclose
Kuirong Jiang: Nothing to disclose

**16:00-17:30** **Research Stage 3**

### Research Presentation Session: Neuro

### RPS 2111
### Neurodegeneration and cognition

**Moderator**
E. E. Bron; Rotterdam/NL
*(e.bron@erasmusmc.nl)*

### RPS 2111-2
**Relationship between hippocampal subfield volumes and cognitive decline in healthy subjects**
*S. Doran*, R. A. Kenny, J. F. Meaney, C. De Looze; Dublin/IE

**Purpose:** We examined the relationship between hippocampal subfield volumes and cognitive decline over a four-year period in a healthy older adult population with the goal of identifying subjects at risk of progressive cognitive impairment which could potentially guide therapeutic interventions and monitoring.
**Methods or Backround:** 482 subjects (68.1 years +/- 7.4; 52.9% female) from the Irish Longitudinal Study on Ageing underwent magnetic resonance brain imaging and a series of cognitive tests. Using K-means longitudinal clustering, subjects were first grouped into three separate global and domain-specific cognitive function trajectories; high-stable, mid-stable and low-declining. Linear mixed effects models were then used to establish associations between hippocampal subfield volumes and cognitive groups.
**Results or Findings:** Decline in multiple hippocampal subfields was associated with global cognitive decline, specifically the presubiculum (estimate -0.20; 95% confidence interval (CI) -0.78 – -0.02; p=.03), subiculum (-0.44; -0.82 – -0.06; p=.02), CA1 (-0.34; -0.78 – -0.02; p=.04), CA4 (-0.55; -0.93 – -0.17; p=.005), molecular layer (-0.49; -0.87 – -0.11; p=.01), dentate gyrus (-0.57; -0.94 – -0.19; p=.003), hippocampal tail (-0.53; -0.91 – -0.15; p=.006) and HATA (-0.41; -0.79 – -0.03; p=.04), with smaller volumes for the Low-Declining cognition group compared to the High-Stable cognition group. In contrast to global cognitive decline, when specifically assessing the memory domain, cornu ammonis 1 subfield was not found to be associated with low declining cognition (-0.14; -0.37 – 0.10; p=.26).
**Conclusion:** Previously published data shows that atrophy of specific hippocampal subfields is associated with cognitive decline but our study confirms the same effect in subjects asymptomatic at time of enrolment. This strengthens the predictive value of hippocampal subfield atrophy in risk of cognitive decline and may provide a biomarker for monitoring treatment efficacy.
**Limitations:** T1 imaging can be unreliable for hippocampal segmentation.
**Ethics committee approval:** This study was approved by the Trinity College Faculty of Health Sciences Research Ethics Committee, Dublin, Ireland. Protocols conformed with the Declaration of Helsinki. Signed informed consent was obtained from all respondents prior to participation. Additional ethics approval was received for the magnetic resonance imaging (MRI) sub-study from the St James's Hospital/Adelaide and Meath Hospital, Inc. National Children's Hospital, Tallaght (SJH/AMNCH) Research Ethic Committee, Dublin, Ireland. Those attending for MRI also completed an additional MRI-specific consent form. (De Looze et al)
**Funding for this study:** Funding for The Irish Longitudinal Study on Ageing (TILDA) is provided by the Irish Government, the Health Research Board (HRB), The Atlantic Philanthropies, and the Irish Life Plc.
**Author Disclosures:**
Simon Doran: Nothing to disclose
Celine De Looze: Nothing to disclose
Rose Anne Kenny: Nothing to disclose
James F. Meaney: Nothing to disclose

### RPS 2111-3
**Differential atrophy along the longitudinal axis of the hippocampus in Alzheimer's disease and suspected non-Alzheimer's disease pathophysiology (SNAP)**
*T. Meira*, R. Morais-Ribeiro, T. Jesus, M. Dias, A. Coelho, T. G. Oliveira; Braga/PT
*(torcatomeira14@gmail.com)*

**Purpose:** Cerebrospinal fluid (CSF) biomarkers have been increasingly used to support diagnosis of Alzheimer's disease (AD). Suspected non-AD pathophysiology (SNAP) refers to normal CSF levels of amyloid-beta (AB) with increased tau, whereas Alzheimer's Disease continuum (ADc) is defined by AB pathology evidence. Since hippocampus studies have highlighted differential properties along its longitudinal axis, we aim to evaluate how its various subregional volumetric markers differ between ADc, SNAP and controls, as well as their association with clinical presentation.
**Methods or Backround:** We included 1242 participants from the Alzheimer's Disease Neuroimaging Initiative. Controls (n=234) were defined as having normal CSF AB (≥192 pg/ml), total tau (<93 pg/ml) and phosphorylated tau (<23 pg/ml). ADc individuals (n=784) were abnormal for AB, whereas SNAP subjects (n=224) had normal AB with either abnormal total or phosphorylated tau. Structural MRI acquisitions were analyzed with a method developed in our laboratory for segmenting the hippocampus in anterior, intermediate and posterior parts. Controlling for age, sex and multiple comparisons, groups were compared with one-way ANOVA and Pearson coefficients were calculated to assess correlations between volumetric variables and age, CSF biomarkers or neuropsychological scores.
**Results or Findings:** ADc showed lower total and subregional hippocampal volumes than SNAP or controls (P<0.001). When normalizing the volume of each subregion for its total ipsilateral hippocampus volume, ADc showed increased posterior atrophy (P<0.001) and higher relative anterior volume (P<0.01) when compared to the other groups. SNAP and controls showed greater correlations between volumetric measures and age, with attenuated differences over age. Several subregional hippocampal volumes correlated significantly with altered levels of CSF biomarkers, cognition, neuropsychiatric features and functional impairment.
**Conclusion:** Our work contributes to better understanding of ADc and SNAP pathophysiology and to predicting their respective clinical features.
**Limitations:** No limitations were identified.
**Ethics committee approval:** As per ADNI protocols, all procedures performed in studies involving human participants were in accordance with the ethical standards of the institutional and/or national research committee and with the 1964 Helsinki declaration and its later amendments or comparable ethical standards. More details can be found at adni.loni.usc.edu.
**Funding for this study:** No funding was received for this study.
**Author Disclosures:**
Tiago Gil Oliveira: Nothing to disclose
Marcelo Dias: Nothing to disclose
Torcato Meira: Nothing to disclose
Rafaela Morais-Ribeiro: Nothing to disclose
Ana Coelho: Nothing to disclose
Tiago Jesus: Nothing to disclose

### RPS 2111-4
**Grey matter atrophy mediates the association between tau pathology and cognition in Alzheimer's disease: a simultaneous PET/MRI study**
*X. Xu*, B. Zhang; Nanjing/CN
*(xiaoxu960815@163.com)*

**Purpose:** This study aimed to investigate the area-specific causal pathways between regional tau pathology, grey matter atrophy, and cognitive impairment in MCI/AD patients.
**Methods or Backround:** Thirty-four MCI/AD patients covering the prodromal to dementia spectrum and 23 cognitive normal controls experienced standardised neuropsychological assessments followed by 18F-fortaucipir (FTP) PET and 3D T1 magnetic resonance imaging (MRI). We explored voxel-wise and region of interest- based inter-group differences in grey matter volume (GMV) and regional tau standardised uptake value ratio (SUVR) in the two groups. Multimodal correlation analyses and mediation analyses were carried out to assess the relationship between GMV and the SUVR and cognition scores in MCI/AD group.

**Results or Findings:** The 18F-fortaucipir retention was observed in the fusiform, lateral temporal lobe, supramarginal gyrus, precuneus and posterior cingulate in MCI/AD groups. Mediation analyses showed the GMV of the medial parietal lobes and medial temporal lobes mediated the effect of local and distant region tau on cognitive impairment. Besides, in the medial temporal, GMV of the entorhinal cortex mediated the effect of local region tau on cognitive impairment.
**Conclusion:** Our finding help to clarify the spatial relationship of tau propagation and gray matter atrophy. Local and distant atrophy played a important mediating role between tauand cognitive impairment in MCI/AD patients. Besides, temporoparietal region is an important hub linking gray matter atrophy, tau pathology, and cognitive impairment for AD, and might be a potential treatment target region for AD.
**Limitations:** Firstly, the 18F-fortaucipir PET tracer demonstrating a low degree of off-target binding in the choroid plexus may lead to inaccurate quantification of tau protein in the hippocampus ,Another limitation is the small sample size, longitudinal measurements utilising a larger sample size will be conducted in future studies.
**Ethics committee approval:** Northern Theater General Hospital Ethics Committee: No.2021(024).
**Funding for this study:** This work was supported by Clinical Research Special Funded Project of Nanjing Drum Tower Hospital (No. 2023-LCYJ-MS-11).
**Author Disclosures:**
Bing Zhang: Nothing to disclose
Xinru Xu: Nothing to disclose

### RPS 2111-5

**Compensatory deregulation of attention and executive networks in prodromal Lewy body dementia. A resting-state functional magnetic resonance imaging study**

*V. Onofrj*[1], R. Franciotti[2], A. Ferretti[2], C. Padulo[2], S. Sansi[2], I. Rektorova[3]; [1]Brussels/IT, [2]Chieti/IT, [3]Brno/CZ
*(valeriaonofrj@gmail.com)*

**Purpose:** Early diagnosis of Lewy Body Dementia (MCI-LBD) is challenging due to the lack of specific diagnostic tests and imaging findings. MCI-LBD subjects show attentional, executive, spatial perception, verbal, spatial memory, and intelligence deficits. These occur early and imply the dysfunction of selected sensory and associative networks.
**Methods or Backround:** We performed a global analysis of resting-state functional MRI data on 38 MCI-LBD subjects and 24 healthy controls (HC) and extracted the connectivity matrices of regions included in the Cingulo-opercular Network (CON), Fronto-Parietal Network (FPN), Default Mode Network (DMN), Dorsal Attention Network (DAN), Somato-Motor Network (SMN), Visual Network (VN) and Language Network (LN). We compared intra- and inter-network connectivity between the two groups of subjects and correlated neuropsychological test scores with intra- and inter-network connectivity of MCI-LBD subjects.
**Results or Findings:** The data revealed increased between-networks connectivity ($p<0.05$) between the DAN and SMN, the CON and FPN, and the FPN and DMN in MCI-LBD subjects vs. HC. Decreased between-network and intra-network connectivity ($p<0.05$) was found between the SMN and DMN, the DMN and DAN, and between the right rostral prefrontal and right anterior insular nodes of the CON in MCI-LBD subjects vs. HC. Significant correlations ($p<0.05$) were found among intra-network and between-network FC values and attention, executive, visuo-perceptual, verbal memory, spatial memory deficit, and intelligence tests.
**Conclusion:** Overall, we found early demodulation of intra- and inter-network connectivity starting with the internal dissociation of the CON. Intra- and inter-network connectivity correlates with neuropsychological tests results and may potentially serve as early imaging biomarkers of LBD.
**Limitations:** Our study limitation is mainly the limited number of subjects.
**Ethics committee approval:** This study was approved by the Ethics Committee of Masaryk University and was performed according to the Declaration of Helsinki (1997) and subsequent revisions. All participants gave written informed consent.
**Funding for this study:** The authors received no financial support for the research, authorship, and publication of this article.
**Author Disclosures:**
Caterina Padulo: Nothing to disclose
Irena Rektorova: Nothing to disclose
Valeria Onofrj: Nothing to disclose
Raffaella Franciotti: Nothing to disclose
Stefano Sansi: Nothing to disclose
Antonio Ferretti: Nothing to disclose

### RPS 2111-6

**Can AI augmented MRI replace FDG PET CT brain in the evaluation of patients with cognitive impairment**

*H. Dillon*, B. S. Kelly, G. J. Mcneill, R. O'Donohoe, A. Stone, C. Hickie, M. Colombie, J. Kinsella, R. P. Killeen; Dublin/IE

**Purpose:** Cerebral atrophy and hypometabolism are crucial indicators of neurodegenerative diseases such as Alzheimer's and other cognitive disorders. Assessing cerebral atrophy on MRI is time consuming and subjective. This study aims to compare cerebral atrophy detected on AI augmented MRI with patterns of hypometabolism on FDG PET brain, the current gold standard. This software has the potential to reduce the need for costly FDG PET brain studies as well as increase the number of radiologists who can interpret neurodegenerative studies.
**Methods or Backround:** There is currently limited availability of FDG PET brain in the diagnosis of neurodegenerative conditions worldwide. A new commercially available AI software available through Siemens known as RadCompanion utilises artificial intelligence to detect cerebral atrophy on T1w MRI brain using a simplistic traffic light colour coded system while also providing quantitative analyses. We compared atrophy detected on this new software with the pattern of hypometabolism on FDG PET brain. The images were interpreted by a dual specialised neuromolecular radiologist, specialist neuroradiologist and neuroradiology fellow.
**Results or Findings:** Twenty-three patients were assessed. The patten of cerebral atrophy on MRI was compared to corresponding FDG PET brain and a pattern of neurodegeneration suggested. In 78% of patients the pattern of neurodegeneration suggested matched the suggested diagnosis on the corresponding FDG PET brain. In 100% of patients a normal MRI correlated with a normal FDG PET brain.
**Conclusion:** Our study demonstrates good correlation between AI augmented MRI and FDG PET Brain. This software could provide a useful screening test in centres with limited availability to PET CT and perhaps a suitable alternative in centres with no access. We recommend further evaluation in future using a larger sample size.
**Limitations:** Limitations included small sample size, single centre and retrospective study.
**Ethics committee approval:** This study as ethically approved by through local hospital audit committee.
**Funding for this study:** No funding was obtained for this study.
**Author Disclosures:**
Rory O'Donohoe: Nothing to disclose
Justin Kinsella: Nothing to disclose
Graeme John Mcneill: Nothing to disclose
Mathilde Colombie: Nothing to disclose
Henry Dillon: Nothing to disclose
Alan Stone: Nothing to disclose
Ronan P. Killeen: Nothing to disclose
Brendan S. Kelly: Nothing to disclose
Conor Hickie: Nothing to disclose

### RPS 2111-7

**Brain tissue atrophy and cognitive decline in relation to serum uric acid variance**

*J. Sun*, H. Lv, Z. Wang; Beijing/CN

**Purpose:** This study aimed to evaluate the long-term associations between serum uric acid (SUA) variance and neuroimaging indices of brain health.
**Methods or Backround:** This cohort study recruited 1,111 participants aged 25-83 years from the subset study of brain MRI acquisition within the Kailuan study from 2020 onward. The SUA concentrations were measured every two years from 2006 to 2018. We primarily assessed SUA variance as the average slope incorporating seven measurements and further defined the direction of changes. The multivariate-adjusted associations of SUA variance with MRI markers of brain tissue volumes and microstructural integrity and cognitive function were examined using generalised linear models.
**Results or Findings:** Compared with the stable group, brain white matter (WM) volume decreased irrespective of the increase or decrease in SUA levels (beta=−0.26, 95% CI −0.40 to 0.12 and beta=−0.15, 95% CI −0.28 to −0.02). Elevated SUA levels were also associated with smaller cerebral parenchyma volume (beta=−0.01, 95% CI −0.01 to 0). Participants with progressively increased SUA levels exhibited lower global fractional anisotropy and higher radial diffusivity (beta=−0.27, 95% CI −0.41 to −0.13 and beta=0.23, 95% CI 0.10 to 0.36), which remained significant after further adjustment for white matter and white matter hyperintensity volume. Elevated SUA concentration was also associated with lower MoCA scores (beta=−0.20, 95% CI −0.33 to −0.06).
**Conclusion:** The SUA changes were associated with decreased WM volume. Progressively increased SUA levels detrimentally affect brain health, manifested by smaller brain tissue volume, impaired microstructural integrity, and poorer cognitive performance. Long-term prevention of SUA fluctuation is essential for protecting brain health and preventing early-stage dementia.

**Limitations:** The effects of SUA variance in a more recent period on neuroanatomical features were still unknown and warrant future exploration.
**Ethics committee approval:** The META-KLS cohort study was ethically approved by the Medical Ethics Committee of Kailuan General Hospital (No.2021002). Written informed consent was obtained from all participants. Participants did not receive a stipend.
**Funding for this study:** This study was supported by grants No. 62171297 and 61931013 from the National Natural Science Foundation of China, No. [2015] 160 from the Beijing Scholars Program, No. ZYLX202101 from Beijing Hospitals Authority Clinical Medicine Development of Special Funding Support, No. 2021-135 from Beijing Municipal Health Commission-Beijing Key Clinical Discipline Funding.
**Author Disclosures:**
ZhenChang Wang: Nothing to disclose
Han Lv: Nothing to disclose
Jing Sun: Nothing to disclose

### RPS 2111-8

**White matter abnormalities in patients with mild cognitive impairment and post-acute sequelae of COVID- 19: preliminary results of a radiomics study**

*M. Stubos*, M. Ukmar, G. Pizzamiglio, I. Zorzenon, L. Bottaro, E. Zulian, N. Fiotti, G. Furlanis, M. A. A. Cova; Trieste/IT
*(melaniastubos@gmail.com)*

**Purpose:** The purpose of this study was to assess the contribution of radiomics in the analysis of the normal appearing white matter (NAWM) in patients with cognitive decline related to mild cognitive impairment (MCI) and to post-acute sequelae of COVID-19 (PASC) in comparison to the normal white matter (NMW) of healthy controls. Furthermore, we compared the NAMW of the patients with MCI to the one of patients with PASC.
**Methods or Backround:** A retrospective study on ten patients with MCI, ten patients with PASC and 23 healthy controls who underwent a 3T MRI was performed, and the 3D-FLAIR sequence was selected. Fourteen different brain areas (bilateral frontal lobes, temporal lobes, parietal lobes, thalami, cerebellar peduncles, lateral ventricles, and genu and splenium of the corpus callosum) were selected for the ROIs placement: features were extracted on a radiomics software and a statistical analysis was carried out through a binary logistic regression model.
**Results or Findings:** In the comparison between the NAWM of MCI patients and the NWM of healthy controls, statistically significant differences ($p<0.05$) were found in all the 14 analysed areas, with a good performance in the temporal, frontal and parietal lobes. Significant differences were also found in the comparison between the NAWM of PASC patients and the NWM of healthy controls, with a good performance in the frontal lobes, temporal lobes and thalami. No significant differences were found in the comparison between the NAWM of patients with MCI and PASC.
**Conclusion:** This study shows that radiomics can be a useful tool in the analysis of the NAMW in patients with cognitive decline related to MCI and PASC.
**Limitations:** The low number of patients included in the study and the heterogeneity of the MCI group of patients.
**Ethics committee approval:** The study is retrospective study, hence ethical approval was not sought.
**Funding for this study:** No funding was received for this study.
**Author Disclosures:**
Giovanni Furlanis: Nothing to disclose
Lorella Bottaro: Nothing to disclose
Irene Zorzenon: Nothing to disclose
Maja Ukmar: Nothing to disclose
Giulia Pizzamiglio: Nothing to disclose
Elisa Zulian: Nothing to disclose
Maria Assunta A. Cova: Nothing to disclose
Nicola Fiotti: Nothing to disclose
Melania Stubos: Nothing to disclose

### RPS 2111-9

**COVID-19 induces grey matter atrophy in patients with cognitive but also with only olfactory disorders**

S. Capelli[1], A. Caroli[1], A. Arrigoni[1], A. Napolitano[2], G. Pezzetti[2], A. Remuzzi[3], F. L. Lorini[2], M. Sessa[2], *S. Gerevini*[2]; [1]Ranica/IT, [2]Bergamo/IT, [3]Dalmine/IT

**Purpose:** The aim of this study was to evaluate grey matter (GM) structural alterations related to COVID-19 in two separate groups of patients with the most frequent and distinctive COVID-19-related neurological manifestations - isolated olfactory disorders (COVID-OD) and cognitive disorders (COVID-CM) - compared to a control group of healthy individuals.
**Methods or Backround:** Sixty-one COVID-CM patients (57[60–63] years, 62% females), 84 COVID-OD patients (49[35–57] years, 60% females) and 17 controls (51[41–52] years, 41% females) were included in the study. To investigate differences between patients and controls in terms of GM regional volume and voxel-wise density, Region-Based Morphometry (RBM) and Voxel-Based Morphometry (VBM) were applied to T1-weighted MRI scans. Surface-Based Morphometry (SBM) was used to investigate changes in cortical thickness. Total intracranial volume and age were included as nuisance variables in the statistical model assessing group differences.
**Results or Findings:** The multi-morphometric analysis revealed statistically significant reduction in GM regional volume (RBM) and density (VBM) as well as lower cortical thickness in both COVID-CM and COVID-OD groups compared to controls. Notably, COVID-CM patients showed more widespread and severe tissue loss than COVID-OD patients. The most affected GM regions were hippocampus, putamen, cingulate cortex, praecuneus, amygdala, lingual gyrus, and caudate nucleus. Most of the atrophic regions are known to be involved in memory processes, in the sense of smell, or both.
**Conclusion:** Current MRI findings indicate that, with varying degrees of severity, both COVID-19-related olfactory and cognitive disorders lead to GM atrophy, possibly reflecting neurodegeneration and neuroinflammation. The COVID-CM group showed more pronounced GM changes, suggesting a stronger inflammatory response.
**Limitations:** This was a retrospective and monocentric study, with a small control population. Quantitative clinical data assessing the severity of olfactory or cognitive disorders was not available.
**Ethics committee approval:** The use of patient data was granted ethical approval by the local ethics committee as part of a broader observational study protocol (Reg. 118/22). Informed consent was acquired from patients or from their next of kin (in the case of ICU patients).
**Funding for this study:** This study received partial support from Brembo SpA (Curno, Bergamo, Italy) through the "Progetto TrexUno" initiative.
**Author Disclosures:**
Serena Capelli: Nothing to disclose
Giulio Pezzetti: Nothing to disclose
Alberto Arrigoni: Nothing to disclose
Ferdinando Luca Lorini: Nothing to disclose
Maria Sessa: Nothing to disclose
Anna Caroli: Nothing to disclose
Angela Napolitano: Nothing to disclose
Andrea Remuzzi: Nothing to disclose
Simonetta Gerevini: Nothing to disclose

### RPS 2111-10

**Brain age in healthy individuals and across multiple neurological disorders**

*L. Chai*, Z. Zhuo, Y. Duan, Y. Liu; Beijing/CN
*(chailia456@163.com)*

**Purpose:** The aim of this study was to investigate brain age in healthy individuals and across multiple neurological disorders and its association with MRI measures and clinical variables.
**Methods or Backround:** MRI and clinical data of 2,913 HC and 331 MS patients, 189 NMOSD patients, 239 AD patients, 244 PD patients, and 338 cSVD patients were collected. Brain age gap (BAG) was defined as the difference between brain age predicted using 3D T1w with deep learning and chronological age. Brain regions volumes and WMH and clinical measures were compared between HC with advanced brain aging (BAG ≥ 5 years) and resilient brain agers (BAG ≤ −5 years). Associations between BAG, WMH and clinical variables were examined in patients.
**Results or Findings:** Increased BAG was in patients with MS (10.30 ± 12.6 years), NMOSD (2.96 ± 7.8 years), AD (6.50 ± 6.6 years), PD (4.24 ± 4.8 years), and cSVD (3.24 ± 5.9 years). WMH was higher and regional brain volume was lower in advanced brain agers ($p<0.001$) than in resilient brain agers. The specific brain regions associated with increased BAG differed across the various neurological disorders. Increased BAG was correlated with WMH and cognitive decline in neurological disorders. Increased BAG was correlated with higher disability scores in MS patients but not in NMOSD patients.
**Conclusion:** The BAG shows utility as an imaging marker for monitoring cognitive and physical impairment across different neurological disorders. Advanced brain age was associated with atrophy and WMH, suggesting an increased risk of neurological problems.
**Limitations:** First, it was cross-sectional study. Second, deep learning methods are a “black box”, and the interpretability of brain age predictions needs further improvement. Third, we did not consider other factors that influence brain age. Fourth, the cognitive and clinical assessments were relatively limited.

Ethics committee approval: This study was ethically approved by the Beijing Tiantan Hospital, Capital Medical University, No. KY-2019-050-02 and KY-2019-140-02.
**Funding for this study:** This study was funded by the Beijing Tiantan Hospital, Capital Medical University, No. KY-2019-050-02 and KY-2019-140-02.
**Author Disclosures:**
Yaou Liu: Nothing to disclose
Li Chai: Nothing to disclose
Zhizheng Zhuo: Nothing to disclose
Yunyun Duan: Nothing to disclose

**16:00-17:30** **Research Stage 4**

### Research Presentation Session: Chest

### RPS 2104

### Pulmonary nodules and lung cancer screening

**Moderator**
C. M. Schaefer-Prokop; Amersfoort/NL
*(cornelia.schaeferprokop@gmail.com)*

### RPS 2104-2

**Quality assurance of national lung cancer screening in Taiwan**
*Y-C. Chang*, Y-S. Huang, H-H. Hsu, A. M-F. Yen, H. J. Chiou, W. P. Chan, P-C. Yang; Taipei/TW
*(ycc5566@ntu.edu.tw)*

**Purpose:** This study aimed to describe the preliminary experience in quality assurance of LDCT in national lung cancer screening in Taiwan.
**Methods or Backround:** From 1st July 2022, the National Lung Cancer Screening (LCS) program in Taiwan was launched on a biennial basis. Enrollment criteria include heavy smokers (>30 py, 50-74 years old) and subjects with a family history of lung cancer (male 50-74 years and female 45-74 years old) according to international guidelines and evidence from TALENT (Taiwan Lung Cancer Screening for Never Smoker Trial) study. Modified Lung-RADS (Version 1.1) was used for categorising the LDCT interpretation results after adjusting the size criteria of non-solid nodules >20mm diameter as Category 3 (probably benign findings). This is the first national lung cancer screening to include non-smoker subjects with a family history in the world. Quality evaluation parameters included radiation exposure dose, interpretation results based on modified Lung-RADS, cancer detection rate, positive predication rate (PPV) in different groups, and proven lung cancer stages in 2 groups.
**Results or Findings:** Until 30th June 2023 (data estimated in June 2023), there were a total of 48,372 subjects (male 56.27%, female 43.73%) receiving LDCT LCS in 163 hospitals. 531 lung cancers (1.11%) were identified (data from the Health Promotion Administration, Ministry of Health and Welfare). The majority of lung cancers detected were in the early stage (stage 0 12.5%, stage I 72.58%). Quality assurance (QA) was performed according to the result of positive findings of LDCT (category 3, 4) (11%), positive evaluation after visiting chest specialists (9.23%), cancer detection rate, radiation exposure, etc.
**Conclusion:** The preliminary results of QA showed the importance of screening subjects with a family history of lung cancer in addition to heavy smokers in Taiwan.
**Limitations:** Data analysis is subjected to change because of different time for statistical estimation.
**Ethics committee approval:** No information provided by the submitter.
**Funding for this study:** Funding for this study was received from the Health Promotion Administration, Ministry of Health and Welfare, Taiwan.
**Author Disclosures:**
Hsian-He Hsu: Nothing to disclose
Yeun-Chung Chang: Nothing to disclose
Yu-Sen Huang: Nothing to disclose
Pan-Chyr Yang: Nothing to disclose
Wing P. Chan: Nothing to disclose
Hong Jen Chiou: Nothing to disclose
Amy Ming-Fang Yen: Nothing to disclose

### RPS 2104-3

**Impact of perifissural nodules on false positive rates in lung cancer screening with AI as the initial reader**
*D. Han*[1], M. A. Heuvelmans[1], H. L. Lancaster[1], J. W. C. Gratama[2], M. Silva[3], J. Field[4], M. Oudkerk[1]; [1]Groningen/NL, [2]Apeldoorn/NL, [3]Parma/IT, [4]Liverpool/UK
*(han@i-dna.org)*

**Purpose:** The primary objective of this study is to assess the false positive rate in lung cancer screening attributable to PFNs, at the participant level. Perifissural nodules (PFNs) have been definitively established as benign in lung cancer screening (LCS) trials. Their prevalence, accounting for 20-30% of all nodules, could significantly impact the false positive rate of lung cancer screening, potentially leading to an unnecessary number of follow-ups. This issue is particularly relevant when AI systems are employed as the primary readers, as they currently struggle with accurate classification of PFNs.
**Methods or Backround:** We selected 1,253 baseline scans from the UK Lung Cancer Screening Trial based on the presence of pulmonary nodules exceeding 15 mm³ in volume. We employed the AI-based software AVIEW to automatically detect and volumetrically quantify solid pulmonary nodules. Subsequently, all AI-detected pulmonary nodules with a volume of ≥30 mm³ underwent visual classification by an experienced reader, distinguishing between PFNs and non-PFNs. Pulmonary nodules measuring <100 mm³ were considered negative, while those ≥100 mm³ were categorized as positive.
**Results or Findings:** At the nodule level, a total of 375 pulmonary nodules were classified as PFNs, comprising 296 (78.9%) measuring <100 mm³ and 79 (21.1%) measuring ≥100 mm³. At the participant level, out of 1253 participants, 316 (25.2%) were found to have PFNs. Among these, 250 (20.0%) participants had only negative PFNs, while 66 (5.2%) participants had positive PFNs. Notably, 33 (2.6%) participants with positive PFNs did not have concurrent pulmonary nodules measuring ≥100 mm³.
**Conclusion:** Using AI-based software as the primary reader results in a few false positive PFNs in lung cancer screening.
**Limitations:** The false positive rate attributable to PFNs may be influenced by the performance of AI.
**Ethics committee approval:** This study was waived by the ethics committee due to the retrospective nature of this study.
**Funding for this study:** No funding was received for this study.
**Author Disclosures:**
Jan Willem C. Gratama: Nothing to disclose
Matthijs Oudkerk: Nothing to disclose
Marjolein A. Heuvelmans: Nothing to disclose
Harriet Louise Lancaster: Nothing to disclose
Mario Silva: Nothing to disclose
John Field: Nothing to disclose
Daiwei Han: Nothing to disclose

### RPS 2104-4

**Prevalence of bronchiectasis, airway wall thickening and emphysema in Chinese low-dose CT Screening**
M. Vonder[1], X. Yang[1], H. Groen[1], M. Oudkerk[1], Z. Ye[2], M. Dorrius[1], G. De Bock[1], *Z. Nie*[1]; [1]Groningen/NL, [2]Tianjin/CN
*(z.nie@umcg.nl)*

**Purpose:** This study aimed to assess the prevalence of lung CT findings in a general Chinese population. In lung cancer CT screening, other lung findings like bronchiectasis, airway wall thickening and emphysema are associated with more exacerbations and hospitalisations, as well as increased mortality rate.
**Methods or Backround:** This study included Nelcin-B3 participants aged 40-74 years in China who received low-dose CT lung cancer screening. Baseline characteristics of participants were described. Fleischner criteria were applied to assess bronchiectasis, airway wall thickening, and emphysema (at least mild). The prevalence and combined prevalence for lung findings were determined. Multivariable logistic regression analysis was performed to examine factors associated with the prevalence of these lung CT findings.
**Results or Findings:** In total, 978 participants (mean age 61.3 years ±6.8; 54.6% women) were included. Bronchiectasis was identified in 9.2% of participants, 35.7% showed airway wall thickening, and 19.9% had emphysema. 2.1% of participants showed all three CT findings. 50% of participants with emphysema were more likely to be current smokers. Multivariable logistic regression showed age (OR=1.04; CI: 1.01-1.07), smoking (OR=3.03; CI: 1.87- 4.93), bronchiectasis (OR=1.68; CI: 1.00-2.83) and airway wall thickening (OR=2.06; CI: 1.46-2.92) were positively associated with the presence of emphysema.
**Conclusion:** In the general Chinese population, at least 48% have one lung CT finding for lung cancer screening. Only 2% have all three lung CT findings. Smoking is the strongest predictor for the presence of emphysema. The relevance of these CT findings should be considered in future lung cancer screening guidelines.

**Limitations:** Firstly, no detailed clinical information could be provided. Secondly, CT diagnosis of mild bronchiectasis and airway wall thickening remains challenging which potentially leads to false-positive diagnoses. Lastly, it only analysed patient data collected at one singular medical centre, which limits generalisability to the whole Chinese population.
**Ethics committee approval:** This study was approved by the Ethics of Biomedicine Research of Second Military Medical University.
**Funding for this study:** Funding for this study was received from The Royal Netherlands Academy of Arts and Sciences and the Ministry of Science and Technology of the People's Republic of China.
**Author Disclosures:**
Marleen Vonder: Nothing to disclose
Monique Dorrius: Nothing to disclose
Matthijs Oudkerk: Nothing to disclose
Geertruida De Bock: Nothing to disclose
Zhaoxiang Ye: Nothing to disclose
Xiaofei Yang: Nothing to disclose
Henk Groen: Nothing to disclose
Zhenhui Nie: Nothing to disclose

### RPS 2104-5

**External validation of an AI algorithm for pulmonary nodule malignancy risk estimation on a dataset of incidentally detected pulmonary nodules**

*R. Dinnessen*[1], K. V. Venkadesh[1], D. Peeters[1], H. A. Piggelen-Gietema[2], E. Scholten[1], C. M. Schaefer-Prokop[3], C. Jacobs[1]; [1]Nijmegen/NL, [2]Maastricht/NL, [3]Amersfoort/NL
*(renate.dinnessen@radboudumc.nl)*

**Purpose:** An AI algorithm for malignancy risk estimation was developed and validated on screen-detected pulmonary nodules. We aimed to test the AI algorithm in clinical data and compare the results to the Brock model.
**Methods or Backround:** A size-matched dataset of solid incidentally detected pulmonary nodules with a diameter range between 5-15 mm was collected, consisting of 53 malignant nodules from CT scans performed at least two months prior to a lung cancer diagnosis, and 53 benign nodules. Differences in patient and nodule characteristics between the malignant and benign groups were assessed. AUCs and 95% confidence intervals were determined and compared using the DeLong method. Sensitivity and specificity were determined at a 10% malignancy risk threshold for the AI algorithm and Brock model, according to the British Thoracic Society guidelines.
**Results or Findings:** No statistical difference in size was detected between the malignant and benign nodules (median [range]: 10.8 [5.8, 15.4]; 10.4 [5.8, 15.1]; respectively). Cases with malignant nodules had a significantly lower number of nodules (p=0.001). The AI algorithm significantly outperformed the Brock model (p<0.001). AUC [95% CI] of the AI algorithm and Brock model were 0.87 [0.80-0.94] and 0.59 [0.48-0.69], respectively. The AI algorithm had a higher sensitivity (0.60 [0.46-0.74]) and specificity (0.87 [0.75-0.95]) than the Brock model (0.42 [0.28-0.56]; 0.75 [0.62-0.86]; respectively).
**Conclusion:** The AI algorithm outperformed the Brock model in a clinical dataset with a more heterogeneous population than a screening population. The AI algorithm demonstrated the potential for nodule risk stratification in a clinical setting, which can aid clinicians in decisions in nodule management, thereby potentially reducing unnecessary follow-up.
**Limitations:** This is a retrospective validation on a single-centre dataset. More research is needed to test the performance in larger and multi-centre data.
**Ethics committee approval:** This study included data collected retrospectively from one university medical centre. The local IRB board waived the need for informed consent because of the retrospective design and the use of anonymized data in this study.
**Funding for this study:** Funding was provided by the Dutch Cancer Society (KWF Kankerbestrijding, project number 14113).
**Author Disclosures:**
Renate Dinnessen: Nothing to disclose
Cornelia M. Schaefer-Prokop: Nothing to disclose
Dre Peeters: Nothing to disclose
Kiran Vaidhya Venkadesh: Nothing to disclose
Ernst Scholten: Nothing to disclose
Hester Alexandra Piggelen-Gietema: Nothing to disclose
Colin Jacobs: Research/Grant Support: MeVis Medical Solutions AG, Bremen, Germany; Philips Medical System, Best, Netherlands; Other: Royalties to institution from MeVis Medical Solutions AG, Bremen, Germany

### RPS 2104-6

**Deep learning for growth prediction of pulmonary ground-glass nodules**

*Y. Sun*, L. Jin, C. Li, M. Li; Shanghai/CN
*(sunyingli208ok@126.com)*

**Purpose:** The diagnosis of pulmonary ground glass nodules(GGNs) remains a challenge in clinical practice. The growth rate of nodules is heterogeneous, although generally slower than that of other diseases. The purpose of this study is to attempt to predict the long-term stability or growth of GGNs using deep learning based on baseline CT imaging.
**Methods or Backround:** In this retrospective study, 575 GGNs from 456 patients were recruited. Five hundred and seventy-five GGNs were randomized into training (70%) and validation sets (30%). A deep learning-based algorithm was developed and validated using baseline CT imaging and clinical features. The deep learning prediction network model was compared with the traditional radiographic features. Also, the first follow-up imaging was also added as input to improve the performance of the deep learning model.
**Results or Findings:** The growth and stable groups contained 233 and 342 GGNs, respectively. Traditional radiographic features have limited value in the prediction of growth or long-term stability of GGNs(AUC=0.70 ± 0.06). Size, density, and age were independent predictors of GGN growth. Comparing with traditional radiographic model, our deep learning model yielded a significant higher AUC value of 0.80 ± 0.05 (P < 0.01). The addition of first follow up CT images improved the model performance (AUC=0.84 ± 0.06).
**Conclusion:** We developed and validated a deep learning model to predict the natural growth pattern of GGNs basing on baseline and first follow up CT imaging. The model achieved good performance and may provide a basis for the improvement of follow-up management of GGNs.
**Limitations:** First, in this retrospective study, the variety of CT scan protocols may have affected the characteristics of GGNs and deep features. Second, the conclusions require further validation using external large-scale datasets.
**Ethics committee approval:** This study was approved ethically with the approval code: 2019K134.
**Funding for this study:** Funding was received from these agencies: National Natural Science Foundation of China 61976238(Ming Li), Science and Technology Planning Project of Shanghai Science and Technology Commission 20Y11902900 (Ming Li), Shanghai "Rising Stars of Medical Talent" Youth Development Program "Outstanding Youth Medical Talents" SHWJRS [2021]-99 (Ming Li), National key research and development program 2022YFF1203301(Ming Li), Cancer Society of Shanghai SACACY21C12 (Yingli Sun), Emerging Talent Program of Huadong Hospital Grant numbers XXRC2213(Ming Li), Leading Talent Program of Huadong Hospital Grant numbers LJRC2202(Ming Li) , and Excellent Academic Leaders of Shanghai 2022XD042(Ming Li).
**Author Disclosures:**
Ming Li: Nothing to disclose
Liang Jin: Nothing to disclose
Yingli Sun: Nothing to disclose
Cheng Li: Nothing to disclose

### RPS 2104-7

**Real world impact of deep-learning supported CAD for routine thoracic CT showed higher agreements with expert peer review on management recommendations for incidental lung nodules**

R. Ramaesh[1], *M. P. Engbersen*[2], M. Javidi[1], J. Rodrigues[3], E. Van Beek[1], M. Bernabeu[1]; [1]Edinburgh/UK, [2]Amsterdam/NL, [3]Bath/UK
*(maurits@aidence.com)*

**Purpose:** Incidental lung nodules at CT provide an opportunity for the timely detection of early-stage lung cancer. Computer-aided detection (CAD) supported by deep learning aims to assist radiologists in the detection and further assessment of nodules. This study investigates the effect of CAD on agreements in management recommendations between the reporting radiologist and an expert peer reviewer.
**Methods or Backround:** In this multicentre implementation study, participating radiologists from four centres reported on chest CTs in adult patients without a history of malignancy or nodules, during routine practice with the CAD as 'second reader'. The reporters documented management recommendations concerning lung nodules sequentially, without CAD and with CAD. All cases with nodules found either by CAD or the reporter were independently reviewed by a thoracic radiologist with at least eight years experience. Recommendations of the reporter, with and without CAD, were compared to the recommendations of the reviewer. Agreement between recommendations was assessed with quadratic weighted kappa, and the difference was tested with a William's test.
**Results or Findings:** Nineteen percent (237/1264) of CTs had lung nodules found and confirmed by the reviewer. In 11.4% (27/237) of nodule cases, the reporting radiologist had changed their recommendation after CAD. The most frequent reason given for this change was an initially missed nodule (8.4%; 20/237). The weighted kappa between the reporters' and the reviewers' recommendations was 0.49 and 0.55, unaided and aided by CAD (p = 0.04).
**Conclusion:** Aided by CAD, reporting radiologists found more incidental lung nodules and provided management recommendations which are more in agreement with an expert reviewer, suggesting a significant contribution of CAD to the clinical management of incidental nodules.
**Limitations:** Limitations include a lack of reference standard by longitudinal data or a consensus of multiple reviewers.
**Ethics committee approval:** The study was approved by the EM-REC (Edinburgh Medical School Research Ethics Committee). Reference number: 22-EMREC-015 dd. 14-04-2022.
**Funding for this study:** This study was funded by the NHS AI in Health and Care Award (grant number: 2119-C25043).

**Author Disclosures:**
Malihe Javidi: Nothing to disclose
Rishi Ramaesh: Nothing to disclose
Maurits Peter Engbersen: Shareholder: RadNet
Edwin Van Beek: Advisory Board: Aidence BV; Owner: QCTS Ltd.; Consultant: Lunit; Speaker: Astra Zeneca; Consultant: Astra Zeneca
Jonathan Rodrigues: Speaker: HeartFlow; Speaker: Aidence BV; Employee: Heart and Lung Imaging LTD; Founder: Heart and Lung Imaging LTD; Shareholder: Heart and Lung Imaging LTD; Speaker: Sanofi; Consultant: NHSx
Miguel Bernabeu: Research/Grant Support: Eisai

### RPS 2104-8

**Comparison of two different DL-based CAD systems regarding pulmonary nodule detection, localisation and classification: a multi-reader study**

A. A. Peters[1], *N. Wiescholek*[1], J. Klaus[1], F. Strodka[1], A. Macek[1], E. C. Primetis[2], D. D. Drakopoulos[2], A. Christe[1], L. Ebner[1]; [1]Bern/CH, [2]Muri bei Bern/CH

**Purpose:** The aim of this study was to evaluate and compare the performance of two DL-CAD systems regarding detection, localisation and classification of pulmonary nodules.
**Methods or Backround:** The main study cohort contained 122 proven T1 tumors of the lung and was extended by 83 cases (subsolid, n=13; solid<6mm, n=40; controls, n=30), resulting in a primary cohort of n=205. Two different DL-CAD systems analyzed all cases. Five independent blinded readers with different experience levels (residents, n=3; seniors, n=2) performed two readout sessions, first stand-alone and then with access to the results of one of the DL-CAD systems. Two readers used software 1 and the three readers used software 2 and scored nodule size, density and localisation. LungRADS categories were calculated and compared.
**Results or Findings:** After application of the eligibility criteria, the final cohort consisted of 198 subjects with 221 pulmonary nodules. Residents' mean detection rate increased from 64% to 77% (p<0.001) using the respective DL-CAD (table 2), while the seniors' detection rates did not improve (p=0.25). Regarding the correct localization of the nodules, the residents' rates for lobar (73% vs. 77%; p<0.001) and segmental (64% vs. 68%; p<0.001) nodule localisation improved significantly, the seniors showed no significant benefit. Regarding software comparison, software 2 lead to a slightly higher increase in detection rates (software 1, 80% to 86% and software 2, 67% to 77%; both p<0.001). Both systems showed no significant effect on the rate of correct LungRADS classification.
**Conclusion:** Less experienced readers have more benefits from using DL-CAD systems regarding detection and localisation of pulmonary nodules. There is no effect on correct LungRADS classification. Both systems performed comparably, software 2 lead to a higher increase in detection rates.
**Limitations:** Selection bias (high cancer prevalence). Nodule size groups categorized instead of exact measures.
**Ethics committee approval:** This study was approved by the Kantonale Ethikkommision (KEK) Bern.
**Funding for this study:** No funding was obtained for the current study.
**Author Disclosures:**
Felix Strodka: Nothing to disclose
Alan Arthur Peters: Nothing to disclose
Jeremias Klaus: Nothing to disclose
Ana Macek: Nothing to disclose
Dionysios Dionysios Drakopoulos: Nothing to disclose
Elias C. Primetis: Nothing to disclose
Lukas Ebner: Nothing to disclose
Andreas Christe: Nothing to disclose
Nina Wiescholek: Nothing to disclose

### RPS 2104-9

**Incidental detection of ground-glass nodules and primary lung cancer in patients with primary breast cancer: incidence and long-term follow-up on chest CT**

*H. S. Ryu*, H. N. Lee, J. I. Kim, J. K. Ryu, Y. J. Lim; Seoul/KR
*(bebenignsun@gmail.com)*

**Purpose:** Patients with breast cancer have a higher risk of developing lung cancer than the general population. The study aimed to evaluate the incidence of GGN and risk factors for GGN growth in patients with breast cancer and to evaluate the incidence and pathologic features of lung cancer.
**Methods or Backround:** We retrospectively reviewed the clinical data and chest CTs of 1384 patients diagnosed with breast cancer who underwent chest CT between January 2008 and December 2022. We evaluated the incidence and size change of GGNs during follow-up and identified independent risk factors for their growth using multivariate analysis. Furthermore, the incidence and pathologic features of lung cancer were also evaluated.
**Results or Findings:** We detected persistent GGNs in 69 of 1384 (5.0%) patients. The initial diameter of GGNs was 6.3 ± 3.6 mm on average, with primarily (85.5%) pure GGNs. Among them, 27 (39.1%) exhibited interval growth with a median volume doubling time of 1006.0 days (interquartile range, 622.0–1528.0 days) during the median 959 days (interquartile range, 612.0–1645.0 days) follow-up period. Older age (P = 0.026), part-solid nodules (P = 0.006), and total number of GGNs (≥2) (P = 0.007) were significant factors for GGN growth. Lung cancer was confirmed in 13 of 1384 patients (0.9%), all with adenocarcinoma, including one case of minimally invasive adenocarcinoma. The cancers demonstrated a high rate of epidermal growth factor receptor mutation (69.2%).
**Conclusion:** Persistent GGNs in breast cancer patients with high-risk factors should be monitored for early detection and treatment of lung cancer.
**Limitations:** This retrospective study was conducted at a single centre with a small sample size, manual measurement of GGNs was subject to errors, and chest CT was not dedicated to automated volume measurement.
**Ethics committee approval:** The study was approved by institutional review board of Kyung Hee University Hospital at Gangdong (2023-02-016) and informed consent was waived owing to the retrospective nature of the study.
**Funding for this study:** No funding was received for this study.
**Author Disclosures:**
Yu Jin Lim: Nothing to disclose
Han Na Lee: Nothing to disclose
Hye Sun Ryu: Nothing to disclose
Jung Kyu Ryu: Nothing to disclose
Jung Im Kim: Nothing to disclose

### RPS 2104-10

**Construction and validation of a risk score system for diagnosing invasive adenocarcinoma presenting as pulmonary pure ground-glass nodules: a multicentre cohort study in China**

*Q. Meng*, P. Gao; Zhengzhou/CN
*(zlyymengqingcheng1865@zzu.edu.cn)*

**Purpose:** Pure ground-glass nodule (pGGN) invisibility drives clinical intervention. Radiomics and radiogenomics aid pGGN diagnosis but lack of standardised acquisition parameters, reproducibility and inconsistent methods. We aim to evaluate a risk score system for diagnosing invasive adenocarcinoma presenting as pGGN.
**Methods or Backround:** Seven hundred and seventy-two pGGNs from 707 individuals were grouped into training (509 patients/558 observations) and validation (198 patients/214 observations) sets. A test set with 143 observations was also analysed. The quantitative parameters were obtained using AI. The positive pGGN cutoff score was ≥3. Risk score systems3 were calculated as the history of carcinoma*1+chronic obstructive pulmonary disease (COPD)*1 + long diameters*1 + volume of nodule*1 + mean CT values*1 + type II vascular supply sign*1 or type III*2 + other variables of radiographic characteristics*1. The risk score system and AI model were evaluated using areas under the receiver operating characteristics curve (AUCs), accuracy, sensitivity, specificity, and positive predictive values.
**Results or Findings:** Risk score system 3 (AUC, 0.840) performed better than the AI model (AUC, 0.553), risk score system 1 (AUC, 0.802, and risk score system 2 (AUC, 0.816), with 88.0% (0.850–0.904) accuracy, 95.6% (0.932–0.972) PPV, 89.6% (0.864–0.920) sensitivity, and 80.6% (0.717–0.872) specificity in the training sets. Risk score system 3 yielded the best performance in the validation and test set, with AUCs of 0.769 and 0.801.
**Conclusion:** The risk scoring system based on AI-based quantitative image parameters, clinical features, and radiographic characteristics can effectively predict the invasive adenocarcinoma of pulmonary pGGNs.
**Limitations:** First, the study was retrospective, with some selection bias. Second, the improved AI algorithm may enhance the diagnosis of the invasiveness of pGGNs in clinical practice, and iterative upgrading of the algorithm is needed in the future.
**Ethics committee approval:** This multi-centre, retrospective cohort study was conducted in accordance with the Declaration of Helsinki, and the protocol was approved by the The Affiliated Cancer Hospital of Zhengzhou University & Henan Cancer Hospital Medical and the The Affiliated Cancer Hospital of Zhengzhou University & Henan Cancer Hospital Ethics Committee (2021-KY-0022).
**Funding for this study:** Funding of this study was received from the Key project of Medical science and Technology of Henan Province in China (No: SBGJ202102057).
**Author Disclosures:**
Pengrui Gao: Nothing to disclose
Qingcheng Meng: Nothing to disclose

### Sunday, March 3

08:00-09:00 **Research Stage 1**

### Research Presentation Session: Neuro

### RPS 2211

### Brain tumour imaging: from radiology to pathology

**Moderator**
J. Walecki; Warsaw/PL
*(jerzywalecki1@gmail.com)*

### RPS 2211-2

**Default mode network functional connectivity is sensitive to glioma WHO-grade**

*A. M. Radwan*, H. Vandermeulen, F. Samardzic, J. M. Sousa, S. Sunaert; Leuven/BE

**Purpose:** This study examined the impact of glioma characteristics (WHO grade and volume), patient age, and gender on the functional connectivity (FC) of seven canonical resting-state networks (RSNs). Specifically, FC difference in the default mode (DMN), dorsal attention (DAN), fronto-parietal (FP), language (Lang), salience (SAL), sensory-motor (SMN), and visual (Vis) networks.
**Methods or Backround:** BOLD resting-state functional magnetic resonance imaging (rs-fMRI) data were collected at 3 Tesla from 40 neurosurgery-naïve patients, newly diagnosed with cerebral glioma. Data were preprocessed using fmriprep, and denoising and seed-to-voxel functional connectivity mapping (SBA-FC) were performed with CONN. Hypothesis testing was conducted using FSL's Randomise for nonparametric two-group t-tests. This excluded subject-specific lesioned voxels and controlled for lesion volume, patient age, and gender. SBA-FC beta maps were compared between high-grade gliomas (HGG, WHO grades III-IV, N=21) and low-grade gliomas (LGG, WHO grades I-II, N=19). Separate statistical contrasts also evaluated the effects of lesion volume, patient age, and gender.
**Results or Findings:** Global DMN connectivity was significantly reduced in HGG patients compared to LGG (579 voxels, t-value mean=3, PFDR<.05), involving the posterior cingulate and precuneus gyri. A small focus (18 voxels) of significantly increasing FC with age was identified in the right supplementary motor area (SMA) (t-value mean=4.45, PFDR<0.05). Tumour volume and patient gender did not significantly influence FC in any of the seven RSNs.
**Conclusion:** DMN functional connectivity is notably sensitive to glioma WHO grade. FC in the right SMA appeared to increase with patient age, while lesion volume and patient gender did not impact FC in the other RSNs.
**Limitations:** This study had a limited sample size and a lack of neuropsychological data.
**Ethics committee approval:** This study was approved by the UZ/KU Leuven ethics committee study number is S61759.
**Funding for this study:** No funding was provided for this study.
**Author Disclosures:**
José Maria Sousa: Nothing to disclose
Henri Vandermeulen: Nothing to disclose
Ahmed M. Radwan: Nothing to disclose
Filip Samardzic: Nothing to disclose
Stefan Sunaert: Nothing to disclose

### RPS 2211-3

**IDH status prediction in gliomas using machine-learning analysis of multiparametric MRI**

*V. Sedlák*, T. Belsan, D. Netuka, A. Kavková; Prague/CZ

**Purpose:** This study aimed to explore the efficacy of machine-learning algorithms in accurately predicting Isocitrate Dehydrogenase (IDH) mutation status in adult-type diffuse brain gliomas, utilising quantitative data extracted from multiparametric MRI, to enhance diagnostic precision and potentially guide personalized treatment strategies.
**Methods or Backround:** A cohort of 100 patients underwent comprehensive multimodal MRI, encompassing ASL perfusion, DSC perfusion, advanced diffusion imaging (including DKI, SMT and other models) and standard morphological imaging (i.e. T2, FLAIR, SWI, pre and postcontrast T1). Quantitative features were then extracted from these scans and fed into machine-learning algorithms, with the objective of developing a predictive model for IDH status in gliomas. Investigated algorithms included random forest, XGBoost, AdaBoost, logistic regression and support vector machine models.
**Results or Findings:** Various performance metrics were assessed for each model with emphasis on accuracy and AUC. The investigated machine-learning models achieved high diagnostic accuracies in determining the IDH mutation status, with areas-under-the-curve ranging from 89% for Random Forrest to 97% in the case of the Logistic Regression model.
**Conclusion:** The integration of machine-learning algorithms with multiparametric MRI data demonstrates a promising avenue for the accurate prediction of IDH status in glioma patients. This approach not only substantiates the pivotal role of advanced imaging techniques in diagnostic neuro-oncology but also underscores the transformative impact of machine-learning in medical diagnostics and patient stratification.
**Limitations:** The main limitation of the study is the still relatively modest sample size in combination with the inherent heterogeneity of glioma characteristics, which in combination might introduce potential bias in algorithm training. Further studies with larger cohorts and external validation are imperative to ascertain the generalisability of these models.
**Ethics committee approval:** This study was approved by the Ethics Committee of the Military University Hospital in Prague.
**Funding for this study:** This research was financially supported by the Charles University Grant Agency (project no. 222623) entitled "Advanced Diffusion MR Imaging in Diagnosis of Brain Tumors", implemented at the Second Faculty of Medicine of Charles University.
**Author Disclosures:**
Vojtěch Sedlák: Nothing to disclose
Anna Kavková: Nothing to disclose
David Netuka: Nothing to disclose
Tomas Belsan: Nothing to disclose

### RPS 2211-4

**DSC-PWI presurgical differentiation of Grade 4 astrocytoma and glioblastoma in young adults: unsupervised percentilic rCBV analysis across enhancing and non-enhancing regions**

*A. M. Viveros*, P. Naval-Baudin, S. Flores Casaperalta, F. A. Garay Buitron, S. Septién Rivera, M. Cos Domingo, C. Majós, A. Pons Escoda; Barcelona/ES
*(mildred.viveros@hotmail.com)*

**Purpose:** This research aimed to evaluate the differentiation ability of relative-cerebral-blood-volume (rCBV) percentile values for the enhancing and non-enhancing tumour regions compared to the more commonly used mean or maximum preselected rCBV values. The presurgical differentiation between IDH-mutant astrocytoma-grade-4 and IDH-wildtype-glioblastoma is relevant for patient management, especially in young adults. It provides prognostic information and aids in guiding the molecular diagnostic work-up or in identifying patients for trials on IDH-directed treatments. While DSC-PWI has demonstrated potential for this task, its full capabilities may not yet have been realised.
**Methods or Backround:** Patients with grade 4 astrocytic tumours, known IDH-mutation status, available presurgical MR with DSC-PWI, and under 55 years old (threshold below which IDH-mutations are evenly balanced) were retrospectively retrieved from 2016- 2023. Both enhancing and non-enhancing regions were 3D-segmented. Voxel-level rCBV was calculated to derive mean, maximum, and percentile values. Statistical comparisons were performed using the Mann-Whitney U test and AUC-ROC.
**Results or Findings:** The study comprised 59 patients: 11 astrocytoma-4, and 48 glioblastoma. The enhancing regions of glioblastoma displayed a higher rCBV, though the differences were not statistically significant. The non-enhancing components of astrocytoma-4 exhibited significantly higher rCBV, more pronounced when assessing lower percentiles. The 30th rCBV percentile values for the non-enhancing region were 0.705 in astrocytoma-4 and 0.458 in glioblastoma, with a p-value of 0.001 and AUC-ROC of 0.811. This outperformed the results derived from the commonly used mean and maximum.
**Conclusion:** An unsupervised percentile-based approach to select rCBV values enhances the differentiation outcomes over the traditional mean and maximum. The non-enhancing region offers more valuable insights than the enhancing region. Elevated rCBV values in the lower percentiles of the non-enhancing component in astrocytoma-4 are the most distinguishable characteristic and may represent very-low vascularized infiltrated tissue, versus pure oedema in glioblastoma.
**Limitations:** This was a single-site and retrospective investigation.
**Ethics committee approval:** This study was approved by the Ethics Committee of Hospital Universitari de Bellvitge.
**Funding for this study:** No funding was received for this study.
**Author Disclosures:**
Carlos Majós: Nothing to disclose
Pablo Naval-Baudin: Nothing to disclose
Albert Pons Escoda: Nothing to disclose
Sara Septién Rivera: Nothing to disclose
Francis Astrid Garay Buitron: Nothing to disclose
Susanie Flores Casaperalta: Nothing to disclose
Mónica Cos Domingo: Nothing to disclose
Ady Mildred Viveros: Nothing to disclose

RPS 2211-5
**Tumour-ipsilateral hemisphere T2 relaxometry predicts progression-free survival in patients with primary glioblastoma**
*J. Vymazal*, A. Rulseh; Prague/CZ
*(josef.vymazal@homolka.cz)*

**Purpose:** This study aimed to assess whether repeated MR T2 relaxometry of the tumour-ipsilateral hemisphere can predict the progression-free survival (PFS) of patients with primary glioblastoma (GBM).
**Methods or Backround:** 299 MR examinations in 32 GBM patients were included in this study. T2 in the tumour ipsilateral hemisphere was calculated and plotted against PFS. The evaluation period ranged between 4 months and 19 years. Patients with no disease progression were included only if they exceeded 2.5 years of follow-up; only data from the first 2.5 years were used if PFS was longer. 1/T2 was plotted against PFS with linear regression and the slope was calculated. No post-progression data were used.
**Results or Findings:** Seven patients with PFS longer than 6 years (6-19 years, three of them still with no progression) had an average slope of 0.0018 (-0.1294 to 0.4256). Seven patients with PFS less than 1 year (0.329 to 0.944) had an average slope of -1.2659 (-3.7109 to -0.1964), p=0.025. Eight patients with PFS between 1-2 years had an average slope of -0.619 (-0.1412 to -1,3223) p=0. Ten patients with PFS between 2-5 years had an average slope of -0.4032 (-0.6595 to -0.1331), compared with PFS longer than 6 years p=0.0015.
**Conclusion:** T2 relaxometry from the tumour-ipsilateral hemisphere reliably predicted PFS longer than 6 years, based on data from the first 2.5 years. The average linear regression slope was dependent on PFS: No patients with PFS longer than 6 years had a slope lower than -0.12, and no patients with a slope less than -0.8 had PFS longer than 1.67 years. This methodology may select patients with a high risk of early recurrence and also those where long-term PFS can be expected.
**Limitations:** This was a partially retrospective study.
**Ethics committee approval:** This study was approved by the Ethics Committee of the Na Homolce Hospital, Prague, The Czech Republic.
**Funding for this study:** This study was supported by MH CZ – DRO (Na Homolce Hospital – NNH, 00023884) IG204301, IG204302.
**Author Disclosures:**
Josef Vymazal: Research/Grant Support: Supported by MH CZ – DRO (Na Homolce Hospital – NNH, 00023884) IG204301, IG204302
Aaron Rulseh: Nothing to disclose

RPS 2211-6
**Quantitative histopathological analysis of the periphery of glioblastoma based on ADC and rCBV**
*J. Romero Coronado*, A. Ramos Gonzalez, A. Hilario Barrio, E. Salvador Alvarez, C. Lechuga Vázquez, Z. H. Chen Zhou, A. C. Martinez De Aragón Calvo, A. Cardenas; Madrid/ES
*(juan.romero.ct@gmail.com)*

**Purpose:** In glioblastoma, non-enhancing areas with FLAIR hyperintensity represent both vasogenic oedema and tumour infiltration. The purpose of this study is to correlate the degree of tumour infiltration in histological samples obtained from the FLAIR hyperintense area, targeted by ADC and rCBV in a pre-surgical analysis.
**Methods or Backround:** A total of 33 biopsies performed on 11 patients diagnosed with glioblastoma were analysed. Samples were obtained in their first surgical procedure in patients without any prior treatment. MRI with DWI and DSC sequences were performed and analyzed with Olea Sphere software. We obtained different quantitative parameters using ROIs in the periphery of the non-enhancing tumour. During surgery, before the enhancing tumour was removed, biopsies were obtained from the periphery, previously targeted based on ADC and rCBV. To assess the proliferative potential of our samples, we performed immunohistochemical staining of p53 and MIB1. All slides underwent digital scanning using a NanoZoomer-SQ scanner, and positive cells were quantified using QuPath-0.4.2 software.
**Results or Findings:** In this study, we have obtained a valuable correlation between ADC and rCBV data, and the presence of tumour infiltration in the non-enhancing peripheral tumour with high signal in T2 and FLAIR sequences. The rCBV does not correlate when the value is above 1, in which case we always found tumour infiltration. The ADC correlates with infiltration when the values are low, while we have unexpectedly found areas with high ADC with dense tumour infiltration presumably due to the presence of large vasogenic oedema.
**Conclusion:** Our findings provide valuable insights into the nature of the peripheral zone of glioblastomas. Future studies will help us to correlate radiomic parameters with the degree of tumour infiltration, considering pathological, biological and immune biomarkers.
**Limitations:** No limitations were identified.
**Ethics committee approval:** No information was provided by the submitter.
**Funding for this study:** This study was funded by the Fondo de Investigación en Salud (FIS).
**Author Disclosures:**
Amaya Hilario Barrio: Nothing to disclose
Carmen Lechuga Vázquez: Nothing to disclose
Elena Salvador Alvarez: Nothing to disclose
Ana Carmen Martinez De Aragón Calvo: Nothing to disclose
Agustin Cardenas: Nothing to disclose
Ana Ramos Gonzalez: Nothing to disclose
Juan Romero Coronado: Nothing to disclose
Zhao Hui Chen Zhou: Nothing to disclose

RPS 2211-7
**Differentiating brain metastases, glioblastoma and primary central nervous system lymphoma non-invasively using artificial intelligence-based multiparametric MRI**
*J. Li*, L. Chai, Z. Zhuo, Y. Duan, Y. Liu; Beijing/CN
*(19834515120@163.com)*

**Purpose:** This study aimed to differentiate brain metastases, glioblastoma and primary central nervous system lymphoma non-invasively using artificial intelligence-based multiparametric MRI. Accurate differentiation of brain metastases (BM), glioblastoma (GBM), and primary central nervous system lymphoma (PCNSL) is crucial in clinical practice. However, most studies on artificial intelligence (AI) only focus on the differentiation of two types of tumours, lacking research on AI methods for the simultaneous differentiation of all three tumours.
**Methods or Backround:** This study included preoperative multiparametric MRI images of BM (n=375), GBM (n=391), and PCNSL (n=361). The MRI sequences consisted of T1w, T2w, FLAIR, ADC, and contrast-enhanced T1 images. The data were randomly divided into a training set (n=788) and a test set (n=339) in a 7:3 ratio. A fully automated differentiation model was developed based on the multiparametric MRI for tumour differentiation. The results of the model were compared with those of junior and senior neuro-radiologists.
**Results or Findings:** The accuracy of the model in differentiating BM, GBM, and PCNSL was 0.73, with corresponding AUC values of 0.82, 0.88, and 0.88. The results were similar to those of senior neuro-radiologists (accuracy: 0.74; AUC: 0.87, 0.90, 0.90) and higher than those of junior neuro-radiologists (accuracy: 0.60; AUC: 0.60, 0.73, 0.71). The accuracy improved for the junior neuro-radiologists after re-evaluating the cases using the model's results (accuracy: 0.70; AUC: 0.71, 0.81, 0.77).
**Conclusion:** The model utilises multiparametric MRI for non-invasive differentiation of BM, GBM, and PCNSL. The results of the model are similar to those of senior neuro-radiologists and superior to those of junior neuro-radiologists. Thus, the diagnostic proficiency of junior neuro-radiologists was improved.
**Limitations:** Firstly, all data were obtained from a single centre. Additionally, The number of PCNSLs was relatively lower in all data sets, and this class imbalance may impart statistical bias in model performance. Lastly, the model was trained only with axial MRI slices.
**Ethics committee approval:** This study was approved by the Institutional Review Board of our hospital, and written informed consent was obtained from all patients or their legal guardians.
**Funding for this study:** This work was supported by the Beijing Municipal Natural Science Foundation for Distinguished Young Scholars (No. JQ20035), Capital Health Development Research Project (NO. 2022-1-2042); Radiographic Standard Database Construction Project (NO. YXFSC2022JJSJ004).
**Author Disclosures:**
Yaou Liu: Nothing to disclose
Li Chai: Nothing to disclose
Junjie Li: Nothing to disclose
Zhizheng Zhuo: Nothing to disclose
Yunyun Duan: Nothing to disclose

**08:00-09:00** **Research Stage 2**

### Research Presentation Session: Musculoskeletal

### RPS 2210

### Bone density and spine imaging

**Moderator**
U. Aydingoz; Ankara/TR
*(uaydingo@hacettepe.edu.tr)*
**Author Disclosures:**
Ustun Aydingoz: Advisory Board: GE HealthCare; Speaker: GE HealthCare

### RPS 2210-2

**Application of a novel ultra-high resolution photon counting detector CT in assessing thoracolumbar vertebral anatomic structures**
*Y. Huang*; Shanghai/CN
*(hyf199812222@163.com)*

**Purpose:** The purpose of this study was to evaluate the feasibility of ultra-high resolution (UHR) scanning on photon counting detector CT (PCD-CT) for assessing thoracolumbar vertebral anatomic structures.
**Methods or Backround:** During September 2023, 20 patients underwent the UHR thoracolumbar scanning on PCD-CT utilising a standard resolution (SR) reconstruction with a thickness of 1 mm and an increment of 0.7 mm, and a UHR reconstruction of 0.2 mm for both thickness and increment. All images were reconstructed by the kernel of Br60, Br80, Br84 and Br89. The radiomic features were extracted and the slope of grey values across the vertebral cortex were calculated. The image quality was subjectively analysed by a Likert 5-point scale.
**Results or Findings:** The radiomic features associated with the demonstration of anatomical structures indicated that UHR images outperformed SR images with statistical significance (p<0.001). The slope of grey values across the vertebral cortex of UHR images was highest in UHR images with Br89 (1553.4 [1169.0], p<0.001) and Br84 (1643.5 [1402.5], p<0.001). Image noise significantly increased in UHR images with a high level of kernel. The subjective evaluation revealed that UHR images with Br84 had the best display of bone trabeculae and cortex (5 [0], p<0.001). Moreover, Br84 (5 [0]) and Br89 (5 [0]) had the highest demonstration of spatial resolution (p<0.001).
**Conclusion:** It is feasible to perform thoracolumbar UHR scanning on PCD-CT, and Br84 is the best kernel to improve the spatial resolution and visualisation of vertebral trabecular microstructure. Thoracolumbar vertebral imaging seems particularly likely to benefit from the advantages of PCD-CT in the clinical scenario in the future.
**Limitations:** Due to ethical issues, it is not possible to compare the image quality of conventional scanning images on energy-integrating detector CT and UHR scanning images on PCD-CT in the same patient.
**Ethics committee approval:** The study was approved by the ethics committee reference number: 186
**Funding for this study:** No funding was received for this study.
**Author Disclosures:**
Yufei Huang: Nothing to disclose

### RPS 2210-3

**Association between haemochromatosis genetic variants and vertebral fracture: a UK Biobank study**
*L. Banfield*, K. Knapp, D. Melzer, J. Atkins; Exeter/UK
*(l.r.banfield@exeter.ac.uk)*

**Purpose:** Haemochromatosis is caused by the p.C282Y homozygous variant resulting in iron overload. Previous studies demonstrate increased risks of osteoporosis, back pain and femoral fracture in male homozygotes. The objective of this study was to review the prevalence of vertebral fracture (identified on iDXA) by genotype, in a large community cohort.
**Methods or Backround:** Lateral spine iDXA scans (GE-Lunar) acquired from UK Biobank were assessed. A subset of 292 participants; 146 pC282Y homozygotes and 146 age, BMI and sex matched controls were randomly selected. Images were reviewed for radiological evidence of vertebral fracture using the Genant Semi-Quantitative Scale. Descriptive analysis and logistic regression models assessed for associations between genotypes and incidence of vertebral fracture, adjusting for age and stratified by sex. Bone mineral density (BMD) scores were also reviewed for participants with reported fractures.
**Results or Findings:** 37 vertebral fractures (12.9%) were identified with 20 (14.0%) in the p.C282Y homozygotes and 17 (11.8%) in the wildtype. Vertebral fractures were twice as common in C282Y homozygous men (n=10, 18.2% vs n=5, 8.9%) but not higher in women (n=10, 11.4% vs n=12, 13.6%) however, age-adjusted logistic regression suggests this is not statistically significant in males (OR: 2.34 [0.73-7.42, p=0.15]) although numbers were small. There was also no significant difference in BMD between the homozygotes and the wildtypes: Lumbar spine T-score –0.21 versus 0.34; p=0.50, femoral neck T-score –1.03 versus -1.09 respectively; p=0.94.
**Conclusion:** There may be an increased prevalence of vertebral fracture in the male p.C282Y homozygotes in the absence of diminished BMD when compared to those with no genetic mutations, but this was not statistically significant and further work is needed in a larger cohort.
**Limitations:** The study used a volunteer population, so may be biased towards healthier individuals.
**Ethics committee approval:** Ethical approval for UK Biobank was obtained from the North West Multi-Centre Research Ethics Committee.
**Funding for this study:** Janice L. Atkins is supported by a National Institute for Health and Care Research (NIHR) Advanced Fellowship (NIHR301844). David Melzer, Karen M. Knapp, and Lucy R. Banfield are supported by the University of Exeter.
**Author Disclosures:**
Karen Knapp: Nothing to disclose
David Melzer: Nothing to disclose
Janice Atkins: Nothing to disclose
Lucy Banfield: Nothing to disclose

### RPS 2210-4

**MRI or low-dose CT in suspected axial spondyloarthritis: preliminary results of a comparative study**
*D. Deppe*, S. T. Ulas, M. Koka, F. N. Proft, M. Protopopov, L. Spiller, V. Rios Rodriguez, D. Poddubnyy, T. Diekhoff; Berlin/DE

**Purpose:** This study aimed to evaluate and compare the diagnostic efficacy of magnetic resonance imaging (MRI) and low-dose computed tomography (CT) in patients with suspected axial spondyloarthritis (axSpA). In a standard setting, patients undergo radiography and subsequent MRI, when the radiograph is negative. CT is used in unclear cases, however, it's gaining more attention since the introduction of low-dose techniques.
**Methods or Backround:** 86 patients with suspected axSpA were randomised into two arms: 1. MRI-first (followed by CT if MRI was negative) and 2. CT-first (followed by MRI if CT was negative). Positive imaging was defined by the unambiguous identification of active inflammatory (bone marrow oedema according to the updated ASAS definition suggestive of axSpA) or structural lesions (e.g. extensive erosions or ankylosis suggestive of axSpA), rated in consensus reading by two radiologists specialised in musculoskeletal imaging.
**Results or Findings:** In the MRI-first arm (48 patients), MRI was positive in 11/48 (23%) patients. Subsequent CT in 37 patients showed no positive results (0/37; 0%). In the CT-first arm (38 patients), CT was positive in 12/38 (32%). Subsequent MRI (26 patients) was positive in one (1/26; 4%).
**Conclusion:** MRI and low-dose CT demonstrated comparable diagnostic performance in patients with suspected axSpA.
**Limitations:** If initial imaging was positive, subsequent imaging was not performed which limits the analysis.
**Ethics committee approval:** The study was approved by the local ethics committee (EA1/145/22).
**Funding for this study:** No funding was provided for this study.
**Author Disclosures:**
Dominik Deppe: Nothing to disclose
Denis Poddubnyy: Nothing to disclose
Torsten Diekhoff: Nothing to disclose
Mikhail Protopopov: Nothing to disclose
Mariam Koka: Nothing to disclose
Laura Spiller: Nothing to disclose
Fabian N. Proft: Nothing to disclose
Valeria Rios Rodriguez: Nothing to disclose
Sevtap Tugce Ulas: Nothing to disclose

### RPS 2210-5

**Straightening out scoliosis: how artificial intelligence assisted Cobb angle measurements on spine radiographs can support the clinical user**
*W. Grootjans*[1], S. Challiui[1], A. Bubberman[1], C. Salzlechner[2], D. Ehinger[2], K. van Langevelde[1]; [1]Leiden/NL, [2]Vienna/AT
*(w.grootjans@lumc.nl)*

**Purpose:** Quantitative assessment of scoliosis using Cobb angles on spine radiographs is, albeit labour-intensive, commonly performed in radiology practices. This study aimed to assess the added value and clinical impact of automating Cobb angle measurements using artificial intelligence (AI).
**Methods or Backround:** A total of 100 anteroposterior spine radiographs were retrospectively analysed with a fully automated software solution based on deep learning (SQUIRREL, version 1.0, ImageBiopsy Lab, Vienna, Austria). Automatically determined Cobb angles and range of the spinal curvature were compared to manual measurements. User interaction for AI (un)assisted Cobb angle measurements was monitored using specially designed mouse-tracking software in 20 cases. Time per case, number of mouse clicks, and mousing distance were recorded. Differences were reported as mean±SD (change %).
**Results or Findings:** The acceptance rate of automated Cobb angle measurements was 93%. Rejected cases were due to the presence of metal implants or unrealistic measurements. For accepted cases, the mean difference in automatic and manually determined Cobb angles was 3.7°±3.4, while the mean difference in upper and lower vertebral position was 1.2±1.5 and 0.8±1.1 respectively. Without AI, the mean number of left, right and middle mouse button (LMB, RMB, MMB) clicks was 32.2±7.0, 1.9±0.5, and 1.9±2.0 respectively. Mean mousing distance and time per case were 48620±22698 pixels and 66.9±18.0 seconds respectively. Supporting the user with AI resulted in a mousing distance and time per case of 13868±2486 (-29%) and 29.2±4.7 (-44%) seconds respectively. The mean number of LMB and RMB clicks were 11.3±2.2 (-35%) and 0.4±0.9 (-21%) respectively, while MMB was not used.
**Conclusion:** Automated analysis of Cobb angles by AI-software showed excellent performance. Automation with AI modifies user behaviour and reduces the physical effort required to report a case in clinical practice.
**Limitations:** Data on a single experienced user was reported. Further studies extend these experiments to different users.
**Ethics committee approval:** The study was approved by the institutional ethics commitee (reference number: 20231010001).
**Funding for this study:** No funding was received for this study.
**Author Disclosures:**
Daniel Ehinger: Employee: ImageBiopsy Lab
Willem Grootjans: Nothing to disclose
Kirsten van Langevelde: Nothing to disclose
Adriaan Bubberman: Nothing to disclose
Christoph Salzlechner: Employee: ImageBiopsy Lab
Samir Challiui: Nothing to disclose

### RPS 2210-6

**Assessment of the predictability of vertebral fractures in multiple myeloma in dual-layer CT (DLCT) with virtual non-calcium (VNCa) CT images and calculation of virtual calcium-only (VCa) images**

S. Brandelik[1], S. Rahn[1], M. Merz[2], W. Stiller[1], S. Skornitzke[1], C. Melzig[1], H-U. Kauczor[1], T. F. Weber[1], *T. D. Do*[1]; [1]Heidelberg/DE, [2]Leipzig/DE

**Purpose:** This study aimed to assess the predictability of vertebral fractures in multiple myeloma in dual-layer CT (DLCT) with virtual non-calcium (VNCa) CT images and the calculation of virtual calcium-only (VCa) images.
**Methods or Backround:** 81 patients with plasma cell dyscrasia and whole-body DLCT at the time of diagnosis and follow-up spine imaging were included in the study. Conventional CT images (CI), VNCa images with calcium suppression (CaSupp) indices 25 and 100, and the novel method of calculated VCa images by subtraction of CaSupp100 - CaSupp25 were quantitatively analysed using region-of-interests in the vertebral bodies L1-L5 and all vertebral bodies with fractures on baseline or follow-up imaging. Logistic regression analyses were performed to assess the predictability of imminent spine fractures. For model comparisons, the Akaike information criterion and $R^2$ were consulted.
**Results or Findings:** New fractures were seen in 24 patients' follow-up imaging. Predictability of new vertebral fractures was significant for baseline assessment of CT numbers in CI, CaSupp 25 VNCa, and VCa (p=0.01, respectively) with a higher risk for new fractures in case of lower CT numbers in CI and VCa (Odds ratio 0.982 [0.969;0.994], 0.987 [0.978;0.995]) and in case of higher CT numbers in CaSupp 25 (Odds ratio 1.015 [1.006;1.026]). Direct model comparisons implied that CT numbers in CaSupp 25 and VCa might show better fracture prediction than in CI (R2=0.18 both vs. 0.15; AICc=91.95, 91.79 vs. 93.62). Neither age, gender nor pre-existing fractures improved the fracture predictability when included in the calculation.
**Conclusion:** VNCa and calculated VCa images in DLCT are feasible to predict imminent vertebral fracture risk in MM patients.
**Limitations:** This study was limited by the number of patients.
**Ethics committee approval:** This retrospective exploratory single-centre study was approved by the local review board (application number: S-348/2019). The need for written informed consent was waived.
**Funding for this study:** No funding was received for this study.
**Author Disclosures:**
Maximilian Merz: Nothing to disclose
Thuy Duong Do: Nothing to disclose
Wolfram Stiller: Advisory Board: Philips Medical System
Hans-Ulrich Kauczor: Nothing to disclose
Stefanie Rahn: Nothing to disclose
Tim Frederik Weber: Nothing to disclose
Claudius Melzig: Nothing to disclose
Simone Brandelik: Nothing to disclose
Stephan Skornitzke: Shareholder: ownership interests in investment funds containing stock of healthcare companies

### RPS 2210-7

**The role of dynamic magnetic resonance imaging in improving the diagnostic accuracy of degenerative diseases of the lumbosacral spine**

N. Ibrahim, *A. Saoud*, M. Yosry, A. Yassin; Cairo/EG

**Purpose:** This study aimed to evaluate the role of dynamic MRI in the assessment of lumbar spine degenerative diseases compared to conventional MRI regarding the detection of spinal instability and evaluating the degenerative changes in flexion and extension in comparison to a neutral position to determine the best clinical management and outcome for the patients.
**Methods or Backround:** A prospective study conducted on symptomatic patients with degenerative spine referred to MRI. They underwent imaging in neutral, flexion and extension positions. AP diameter of the spinal canal, foraminal height and area, lumbosacral angle and lordotic angle were measured and used as a comparative index. Correlation with patients' symptomatology and outcome were analysed.
**Results or Findings:** Dynamic MRI evoked radiological findings explaining the patients' clinical picture in 32.4% of the patients. 25% showed a narrowing of the neural foramina dimensions that could be elicited on dynamic images only. 25% of spinal canal stenosis was induced by the positional change and 50% showed spinal instability either in flexion or extension position. 70.3% had conservative management with pain improvement after 3 months of compliance. In 24.3% of the patients, the treatment plan was changed from conservative to surgical intervention after performing dynamic MRI of which 75% showed improvement after surgery. The evaluation of the patient's pain level and outcome after management taken according to the dynamic MRI results showed significant improvement (p-value 0.01).
**Conclusion:** Dynamic MRI of the lumbar spine allows better detection of hidden pathologies in conventional MRI in patients with clinical/radiological mismatches. It gives the surgeon a tool to understand patients' symptomatology. It also provides evidence of neural compression and facilitates accurate preoperative planning regards the procedure and the target level, and accordingly better postoperative outcome.
**Limitations:** This study had a small patient population and there was a lack of long-term follow-up of some of the patients.
**Ethics committee approval:** This study has the approval number MS 85/2023.
**Funding for this study:** No funding was received for this study.
**Author Disclosures:**
Naiyra Ibrahim: Nothing to disclose
Mohamed Yosry: Nothing to disclose
Abdelfattah Saoud: Nothing to disclose
Aya Yassin: Nothing to disclose

### RPS 2210-8

**Opportunistic screening of osteoporosis using routine lumbar spine MRI: a retrospective comparative study**

L. Rousan, *N. M. I. Obeidat*, N. Abdo, L. Alshraa, T. Ajam, H. Al-Zoubi, H. Saif, A. Albaqshi; Irbid/JO
*(nmobeidat8@just.edu.jo)*

**Purpose:** Multiple studies compared T-scores obtained from BMD with different MRI protocols depending on the SNR ratio of the lumbar vertebral bodies. This study aimed to assess whether routine sequences of the lumbar spine MRI can predict the presence of osteoporosis by visually assessing the alteration of the signal intensity alone.
**Methods or Backround:** A 322 cohort of patients who underwent both lumbar spine MRI and DEXA scan within a 6-month interval between January 2015 and December 2022 were selected. Three radiologists graded L4 (or L3) vertebral body fat content into ≤50% or >50% based on visual estimation of its bone marrow signal intensity on sagittal T1 weighted sequences. Average vertebral body bone marrow signal intensity was also obtained. MRI interpretation results were compared with DEXA scan reports after grouping patients into three categories (normal, osteopenia, and osteoporosis). Statistical analysis was performed and a p-value of <0.05 was considered significant.
**Results or Findings:** 322 patients were enrolled in the study (80.4% females, average age 57 years). 10.2% of which had osteoporosis, but the majority had a normal T-score (54.7%). MRI interpretation yielded 167 and 155 patients with a score of ≤50% and >50%, respectively, with an average signal intensity of 727. There was a significant statistical correlation between each of the MRI readings (estimated fat percentage and bone marrow signal intensity) with the diagnosis of reduced bone density on the DEXA scan (p-value of 0.0008 and 0.028, respectively).
**Conclusion:** Our results showed that routine lumbar spine MRI studies can predict reduced bone density and, hence proved to be beneficial in opportunistic screening for osteoporosis. These results are of considerable interest in suggesting the silent disease of osteoporosis in the most requested musculoskeletal MRI scan.
**Limitations:** No limitations were identified.

Ethics committee approval: The institutional review board approved this study and waived the need for written consent (Approval No. 38/123/2019).
**Funding for this study:** No funding was received for this study.
**Author Disclosures:**
Hamzeh Al-Zoubi: Nothing to disclose
Hamad Saif: Nothing to disclose
Leena Alshraa: Nothing to disclose
Nour Abdo: Nothing to disclose
Tarek Ajam: Nothing to disclose
Naser Mohammad Issa Obeidat: Nothing to disclose
Liqa Rousan: Nothing to disclose
Abdulla Albaqshi: Nothing to disclose

**08:00-09:00** **Research Stage 3**

### Research Presentation Session: Vascular

### RPS 2215

### Aortic aneurysm and dissection research: from classification to management

**Moderator**
K. Müller-Peltzer; Freiburg/DE
*(katharina.mueller-peltzer@uniklinik-freiburg.de)*

### RPS 2215-2

**Uncommon anatomy, uncommon ailment: right-sided aortic arch with aberrant retroesophageal subclavian artery and dissecting aortic aneurysms in youth**
*A. M. Sherif*; Abu Dhabi/AE

**Purpose:** To pose the question of whether there exists any association between right-sided aortic arch and aberrant left retroesophageal subclavian artery and dissecting aortic aneurysms in young patients. To encourage innovative thinking and the exploration of new theories regarding potential links between this unique anatomical variant and aortic pathology in the absence of traditional risk factors. To discuss imaging findings and management strategies for these unique patients.
**Methods or Backround:** We present three compelling cases of young patients, aged 25 to 40, each afflicted with dissecting aortic aneurysms involving the aortic arch. They exhibited no known predisposing factors for aortic pathology, such as hypertension or connective tissue disorders. What distinguishes these cases is the presence of a right-sided aortic arch with an aberrant retroesophageal left subclavian artery. The literature has yet to establish any association between right-sided aortic arch and dissecting aneurysms.
**Results or Findings:** Imaging studies consistently revealed a right-sided aortic arch and an aberrant retroesophageal left subclavian artery in all cases. We pose the question of whether this unique anatomical configuration may introduce hemodynamic stress, thereby potentially contributing to the development of aortic dissection in these patients.
**Conclusion:** This series invites exploration of novel ideas and associations, opening doors for fresh discussions and theories surrounding the possible connection between right-sided aortic arch with aberrant retroesophageal left subclavian artery and dissecting aortic aneurysms in young, otherwise healthy patients. While the exact etiological mechanisms remain elusive, this observation underscores the importance of innovative thinking and further research to elucidate the embryological origins and hemodynamic implications of this distinctive anatomical variant. Additionally, it underscores the need for comprehensive imaging and multidisciplinary management approaches to guide patient care in this intriguing context.
**Limitations:** No limitations were identified.
**Ethics committee approval:** The study is educational and so no ethical approval was required.
**Funding for this study:** No funding was received for this study.
**Author Disclosures:**
Ahmed Monier Sherif: Nothing to disclose

### RPS 2215-3

**Comparative analysis of computational workstations for stent planning of thoracic aortic aneurysms**
*V. Koch*, L. D. Grünewald, S. Mahmoudi, S. Martin, C. Booz, J. Gotta, K. Eichler, T. Vogl, T. Gruber-Rouh; Frankfurt a. Main/DE

**Purpose:** The aim of this study was to evaluate the accuracy of three different computer workstations in measuring thoracic aortic aneurysms (TAAs) both in live patients and in laboratory settings, using either pre-interventional computed tomography angiography scans (CTAs) or a specially designed phantom model.
**Methods or Backround:** This retrospective study involved 23 patients with confirmed TAAs detected during routine CTA scans. Alongside measurements of the phantom, an experienced radiologist, unaware of the true dimensions, assessed TAA sizes using three different workstations in two separate rounds. Measurement accuracy was determined by calculating measurement errors, and a Pearson correlation analysis was conducted.
**Results or Findings:** Measurements obtained from the Siemens workstation had a deviation of 3.54% (range, 2.78 to 4.03%; p=0.14) from the true size, while those from General Electric deviated by 4.05% (range, 1.46 to 7.09%; p<0.0001), and TeraRecon had a deviation of 4.86% (range, 3.22 to 6.45%; p<0.0001). Siemens demonstrated the highest precision among the workstations, despite having the most variable measurements (with a range of 4.46%). TeraRecon showed the least variability (with a range of 2.83%) but had the largest deviation from the true phantom size. General Electric's workstation exhibited a variability range of 2.94%. Siemens displayed the strongest correlation between measurements from the 1st and 2nd rounds (r=0.898), followed by TeraRecon (r=0.799) and General Electric (r=0.703). Repeating measurements reduced processing times by 40% with General Electric, 20% with Siemens, and 18% with TeraRecon.
**Conclusion:** In summary, all three workstations demonstrated accurate dimension assessment in the majority of cases with consistently high reproducibility. This ensures precise pre-interventional planning for thoracic endovascular aortic repair.
**Limitations:** Retrospective study. Vendor-specific setup.
**Ethics committee approval:** The institutional ethical review board approved this retrospective study that complies with the Declaration of Helsinki. The need for written informed consent was waived.
**Funding for this study:** No funding was received for this study.
**Author Disclosures:**
Simon Martin: Nothing to disclose
Christian Booz: Nothing to disclose
Thomas Vogl: Nothing to disclose
Vitali Koch: Nothing to disclose
Scherwin Mahmoudi: Nothing to disclose
Tatjana Gruber-Rouh: Nothing to disclose
Leon David Grünewald: Nothing to disclose
Katrin Eichler: Nothing to disclose
Jennifer Gotta: Nothing to disclose

### RPS 2215-4

**Assessment of AAA morphology using relaxation-enhanced angiography without contrast and triggering (REACT): comparison with CTA**
*W. Zeng*, Z. Li, C. Xia; Chengdu/CN
*(zengwen2020@163.com)*

**Purpose:** The aim of the study was to assess the accuracy of relaxation-enhanced angiography without contrast and triggering (REACT) for measuring abdominal aortic aneurysm.
**Methods or Backround:** Contrast-enhanced computed tomography (CT) is usually the gold standard for AAA diagnosis. A novel REACT is proposed, which can be used to diagnose aorta diseases. In this study, patients were recruited consecutively and received REACT using 3T MRI and CTA. Two radiologists independently assessed scans for the morphology of AAA, including the diameter of neck of AAA, the maximum diameter of AAA, and the thickness of lateral thrombus.
**Results or Findings:** A total of 15 consecutive AAA patients (53.27±13.68 years, 11 males) were included. Considering CT as the standard of reference, REACT provided a good correlation. There was no significant difference between REACT and CT in measuring the diameter of neck of AAA(25.30±4.71 mm vs. 26.93±5.06 mm, P=0.028) and the maximum diameter of AAA (46.75±19.43 mm vs. 48.52±20.46 mm, P=0.009). When measuring the thickness of lateral thrombus, REACT slightly underestimated it than CT (6.48±2.17 mm vs. 7.86±3.18 mm, P=0.073). The interobserver agreement for contrast-enhance CT and REACT is relatively good (0.846 for neck of AAA, 0.751 for maximum diameter of AAA, and 0.56 for thickness of lateral thrombus). Further, REACT achieved a substantial accordance with contrast-enhanced CT regarding the morphology of AAA [Cohen’s Kappa 0.776 (95% CI: 0.88–0.93)].
**Conclusion:** REACT has high accuracy in AAA morphological measurements without any contrast agent. This novel sequence is also sensitive to the thickness of thrombosis, which provides a new diagnostic method for AAA patients.
**Limitations:** First, this was a single-center study with a relatively small number of patients enrolled. Second, the readers were not blinded to the CTA and REACT, which might have influenced observer bias.
**Ethics committee approval:** All the subjects provided written informed consent, and this study was approved by the ethics committee of West China Hospital.
**Funding for this study:** This study was supported by the National Key R&D Program of China (2022YFC2009905)

**Author Disclosures:**
Chunchao Xia: Nothing to disclose
Wen Zeng: Nothing to disclose
Zhenli Li: Nothing to disclose

### RPS 2215-5

**Revealing aortic health: a radiomic approach for early acute aortic syndrome detection on non-contrast computed tomography**

*C. Zanon*, A. Toniolo, A. Spertino, C. Bini, G. Cabrelle, M. Antonello, E. Quaia, A. Pepe; Padua/IT

**Purpose:** Acute Aortic Syndrome (AAS) is a life-threatening condition that frequently leads to a high mortality rate for delayed diagnosis. Routine computed tomography (CT) screening programs depend mainly on subjective and qualitative analyses, but Texture Analysis (TA) can extract quantitative data that are not visible to the human eye. This study aimed to evaluate the use of Aortic TA parameters on non-contrast computed tomography (CT) to differentiate between patients with AAS and healthy subjects.
**Methods or Backround:** We retrospectively included 22 patients (mean age, 58 ± 11 years; 13 (59%) male) with a confirmed diagnosis of ASS (12 Type B dissection, 6 intramural hematomas, 4 penetrating aortic ulcers) who underwent emergency CT. Two expert radiologists used specific software to draw aortic wall volumes of interest (VOIs) on unenhanced CT images (axial plane: ascending aorta, isthmus, descending aorta) in patients with AAD and a healthy control group (n=10). We extracted 118 texture parameters (first- and second-order parameters). The Mann-Whitney Test was used, and a p-value of 0.05 was considered statistically significant.
**Results or Findings:** The groups showed statistically significant differences in 17 features, 4 in the ascending aorta ($p < 0.04$), 4 in the isthmus ($p <0.02$), and 9 in the thoracic aorta ($p<0.04$).
**Conclusion:** Texture Analysis may provide valuable insights into the possibility of detecting early aortic wall deterioration to prevent AAS.
**Limitations:** This study is retrospective and involves a limited number of patients.
**Ethics committee approval:** The institutional review board of the University of Padua approved this retrospective study.
**Funding for this study:** No funding was received for this study.
**Author Disclosures:**
Alessia Pepe: Nothing to disclose
Giulio Cabrelle: Nothing to disclose
Andrea Spertino: Nothing to disclose
Chiara Zanon: Nothing to disclose
Michele Antonello: Nothing to disclose
Costanza Bini: Nothing to disclose
Emilio Quaia: Nothing to disclose
Alessandro Toniolo: Nothing to disclose

### RPS 2215-6

**Dual-source photon-counting CTA of the thorax: impact of low energy virtual monoenergetic imaging on image quality, vascular contrast and diagnostic assessability**

*C. Booz*[1], I. Yel[1], V. Koch[1], L. D. Gruenewald[1], L. S. Alizadeh[1], S. Martin[1], T. J. Vogl[1], D. P. Overhoff[2], S. Waldeck[2]; [1]Frankfurt a. Main/DE, [2]Koblenz/DE
*(boozchristian@gmail.com)*

**Purpose:** The aim of this study was to evaluate the impact of low energy VMI+ reconstructions on quantitative and qualitative image quality, vascular contrast and diagnostic assessability of thoracic arteries in photon-counting CTA.
**Methods or Backround:** A total of 120 patients (66 male) who had undergone dual-source photon-counting CTA scans of the thorax were retrospectively analyzed. Standard 120 kV CT images and low keV VMI+ series from 40 to 100 keV with an interval of 15 keV were reconstructed. Quantitative analyses included evaluation of vascular CT numbers, signal-to-noise ratio (SNR) and contrast-to-noise ratio (CNR). CT number measurements were performed in the ascending and descending aorta, the aortic arch, the common carotid artery, the subclavian artery and the coronaries. Qualitative analyses were performed by three board-certified radiologists independently using five-point scales to evaluate image quality, vascular contrast and diagnostic assessability of thoracic arteries.
**Results or Findings:** Mean attenuation, CNR and SNR values were highest in 40 keV VMI+ reconstructions (HU, 1205 ± 11; CNR, 29 ± 7; SNR, 30 ± 9) followed by 55-keV VMI+ reconstructions (HU, 679 ± 8; CNR, 23 ± 6; SNR, 24 ± 7); all three mean values at these keV levels were significantly higher compared with the remaining VMI+ series and standard 120 kV CT series (HU, 169 ± 7; CNR, 19 ± 5; SNR, 27 ± 7) ($p<.0001$). The qualitative analysis showed highest rating scores for 55 keV VMI+ reconstructions followed by 40 keV and 70 keV VMI+ series with a significant difference compared to standard 120 kV CT images series ($p<.0001$).
**Conclusion:** Low keV VMI+ reconstructions at a level of 40-55 keV significantly improve image quality, vascular contrast and the diagnostic assessability of the thoracic arteries compared with standard CT series in photon-counting CTA.
**Limitations:** Single-center retrospective study design
**Ethics committee approval:** The local IRB approved this study.
**Funding for this study:** No funding was receivded for this study.
**Author Disclosures:**
Simon Martin: Nothing to disclose
Christian Booz: Speaker: Siemens Healthineers
Leon D. Gruenewald: Nothing to disclose
Ibrahim Yel: Speaker: Siemens Healthineers
Vitali Koch: Nothing to disclose
Thomas J. Vogl: Nothing to disclose
Daniel Pasqual Overhoff: Nothing to disclose
Stephan Waldeck: Nothing to disclose
Leona Soraja Alizadeh: Nothing to disclose

### RPS 2215-7

**Early insights into the use of dynamic computed tomography angiography for classifying challenging endoleaks**

*Á. Bérczi*, Z. Jokkel, A. Jermendy, M. Berczeli, F. Szablics, Z. Szeberin, B. Merkely, C. Csobay-Novák; Budapest/HU
*(bercziak@gmail.com)*

**Purpose:** The aim of this study was to implement and validate our dynamic CTA (d-CTA) protocol, designed to characterize endoleaks that were previously unclassified or of uncertain origin with triphasic CTA approach.
**Methods or Backround:** Between January 2022 and January 2023, d-CTA scans were performed on specific patients who had uncertain endoleak types or showed ongoing expansion of the aneurysm sac following endovascular aneurysm repair. A total of 12-18 scans were completed, with two different scan protocols: one with 16 cm cranio-caudal coverage and 4.4 seconds between acquisitions, and another with 8 cm coverage and acquisition times ranging from 0.8 to 2 seconds. The scanning protocol was individually tailored based on the region of interest (ROI) and suspected endoleak types. Quantitative data analysis was focused on changes in Hounsfield units within a defined ROI across multiple contrast-enhanced scans.
**Results or Findings:** A total of 18 patients underwent d-CTA scans, out of which 15 individuals met the inclusion criteria. In two cases type V endoleaks were reclassified as type II by identifying inflow arteries. Additionally, for five patients with confirmed type II endoleaks, the quantitative analysis identified the inflow vessels as either the inferior mesenteric artery (IMA) or lumbar arteries. Furthermore, indistinct types of I, II, and/or III endoleaks in seven patients were successfully characterized. In one patient with suspected type II endoleak from IMA on triphasic CTA, the d-CTA scans showed an occluded IMA with no endoleak a month later.
**Conclusion:** D-CTA has the potential to serve as a valuable supplement to the standard triphasic CTA follow-up, aiding in the characterization of difficult-to-diagnose endoleaks and offering valuable information for the development of precise and targeted treatment strategies.
**Limitations:** The main limitation of the study is the small sample size.
**Ethics committee approval:** The present study has been approved by the regional and institutional committee of science and research ethics of our University (SE-RKEB 96/2023).
**Funding for this study:** No funding was received for this study.
**Author Disclosures:**
Bela Merkely: Nothing to disclose
Márton Berczeli: Nothing to disclose
Ákos Bérczi: Nothing to disclose
Fanni Szablics: Nothing to disclose
Csaba Csobay-Novák: Nothing to disclose
Adam Jermendy: Nothing to disclose
Zoltán Szeberin: Nothing to disclose
Zsofia Jokkel: Nothing to disclose

### RPS 2215-8

**Value of dark-blood computed tomography angiography for the assessment of aortic wall disease in patients with acute aortic syndrome**

*N. Li*, W. Sun, Q. Luo, S. Gui, H. Shuishi; Wuhan/CN
*(ncln1001@163.com)*

**Purpose:** The objective of this study was to assess the capability of dark-blood images derived from contrast-enhanced dual-energy CT scans in visualizing aortic wall disease in patients with acute aortic syndrome (AAS).
**Methods or Backround:** Ninety-seven patients with computed tomography angiography (CTA)-confirmed AAS, including acute aortic dissection (n = 35), intramural hematoma (n = 31), and atherosclerotic aortic ulcers (n = 31), were enrolled using IQon spectral CT (Philips Healthcare). Dark-blood images, polyenergetic images (PI), and 40–120 keV virtual monoenergetic images (VMI) were reconstructed. Objective image analysis was performed on PI and VMI images using quantitative values, such as image signal-to-noise ratio (SNRimage), contrast-to-noise ratio (CNRimage), lesion signal-to-noise ratio (SNRlesion), wall-to-fat ratio (WFR), and wall-to-lumen ratio (WLR). Subjective image analyses were conducted by two readers who assessed the diagnostic quality on 4-point Likert scales and the inner/outer vessel wall conspicuity on 5-

Sunday point Likert scales for dark-blood images, PI, VMI40keV, VMI70keV, and VMI100keV.
**Results or Findings:** The SNRimage, CNRimage, SNRlesion, WFR, and WLR gradually decreased as the keV levels increased, while the highest values were found in VMI40keV (all P < 0.05). The subjective scores of the different images were statistically significant (P < 0.05), with the dark-blood image ranked highest in Likert scores regarding diagnostic quality and both inner and outer wall conspicuity, followed by VMI40keV, VMI70keV, and PI, with the lowest scores at VMI100keV. Inter-observer agreement of dark-blood image ranged between moderate and substantial (weighted kappa values 0.60-0.68).
**Conclusion:** Similar to previous studies, low keV levels of VMI enhanced the identification and outlining of wall lesions. Compared to other enhanced images derived from spectral detector CTA, dark-blood images improve the subjective identification of aortic wall lesions and enhance the contrast of wall lesions.
**Limitations:** It was a single-centre trial with a small sample size.
**Ethics committee approval:** The study was approved by the Institutional Review Board of Tongji Medical College, Huazhong University of Science and Technology.
**Funding for this study:** This research was supported by the National Natural Science Foundation of China under Grant 81271570
**Author Disclosures:**
Qinyue Luo: Nothing to disclose
Shen Gui: Nothing to disclose
He Shuishi: Nothing to disclose
Wengang Sun: Nothing to disclose
Na Li: Nothing to disclose

### RPS 2215-9
**Relaxation-enhanced angiography without contrast and triggering (REACT) MRA with deep learning-constrained compressed sensing in aorta aneurysm: a large field of view angiography**
*W. Zeng*, C. Xia, Z. Li; Chengdu/CN
*(zengwen2020@163.com)*

**Purpose:** In this study, free-breathing relaxation-enhanced angiography without contrast and triggering (REACT) MRA was applied to aortic diseases to achieve large field imaging of the entire aorta. Deep learning-constrained compressed sensing was used to reduce scanning time and improve image quality, and this study aimed to examine the feasibility of DLCS for shortening examination time and improving image quality.
**Methods or Backround:** Prospectively recruited patients with aortic disease, including aneurysms and dissections. All the patients underwent 3.0T MR including REACT-CS6, REACT-CS9 and REACT-CS12 between June 2023 and September 2023. The images of REACT-DLCS6, REACT-DLCS9 and REACT-DLCS12 were reconstructed. ROIs were placed at ascending aorta, arch, descending aorta, abdominal aorta and liver. The signal-to-noise ratio of the aorta and the contrast-to-noise ratio between the aorta and the liver were calculated. Quality grading was performed on a 5-point scale for the assessment of overall image quality, artifact removal and background suppression.
**Results or Findings:** Twenty-three patients were included in this study. Acquisition times were CS6 (4 min 46 s), CS9 (3 min 14 s), and CS12 (2 min 06 s). DLCS6 showed the highest SNR ($p < 0.001$) and CNR ($p < 0.001$). There was no evidence of differences among DLCS6, DLCS9 and DLCS12 for CNR (DLCS6 vs. DLCS9, $p = 1.00$; DLCS6 vs. DLCS12, $p = 1.00$; DLCS9 vs. DLCS12, $p = 1.00$). DLCS6 obtained higher scores than other sequences (all $p < 0.01$) and the AICS9 followed.
**Conclusion:** In conclusion, REACT is suitable for large FOV angiography to aid in the diagnosis of patients with aortic disease without the hassle of radiation and gadolinium contrast agents. DLCS9 reduces scanning time to 3 minutes while maintaining relatively good image quality.
**Limitations:** The current work did not fully evaluate the diagnostic performances of these sequences.
**Ethics committee approval:** Ethics approval has been obtained from the Ethics Committee on Biomedical Research, West China Hospital of Sichuan University (approval number: 2021-1171).
**Funding for this study:** The study was funded by the National Key R&D Program of China (grant number: 2022YFC2009905).
**Author Disclosures:**
Chunchao Xia: Nothing to disclose
Wen Zeng: Nothing to disclose
Zhenli Li: Nothing to disclose

**08:00-09:00** **Research Stage 4**

### Research Presentation Session: Genitourinary

### RPS 2207
### Non-oncological genitourinary diseases

**Moderator**
G. Heinz-Peer; St. Pölten/AT

### RPS 2207-2
**Revolutionising the diagnosis of congenital cervical malformations: a novel magnetic resonance imaging-based classification for streamlined standardised assessment**
*Z. Yuan*, Y-L. He, Y. Li, J. Ren, M. Huang, X. Liu, C. Wang, Z. Y. Jin, H-D. Xue; Beijing/CN
*(zhiliny0027@163.com)*

**Purpose:** The aim of this study was to develop an innovative MRI-Based classification method to optimise standardised diagnosis of congenital cervical malformations (CCMs) and summarise their unique MRI Features.
**Methods or Backround:** A total of 79 consecutive patients with CCMs, pre-treatment pelvic MRI scans were conducted and subsequently reviewed by three experienced gynaecological radiologists. Various characteristics, such as signal patterns, morphological classifications, and other relevant factors, were meticulously documented. Furthermore, any accompanying abnormalities were noted for subsequent analysis.
**Results or Findings:** Morphologically, CCMs can be categorised into three types as follows: Type I (42,53%), characterised by the presence of a cervix with visible cervical canals; Type II (18,23%), featuring an existing cervix with concealed cervical canals; and Type III (19,24%), indicating cervical aplasia, which involves a blind end in the lower part of the uterine corpus. Haematometra was significantly more prevalent in Type I compared to Type II patients ($p<0.001$), and Type I patients exhibited a significantly longer mean sagittal length than Type II patients ($p<0.001$). Cervical detection had three signal patterns: no signal (27%), no-evident layer differentiation (21%), and multiple-evident layer differentiation with haematocele (52%). Most patients (94%) had complete vaginal atresia.
**Conclusion:** The MRI-based classification system proposed for CCMs, along with the highlighted key MRI features, demonstrates substantial potential to improve the effectiveness of clinical diagnoses for individuals with CCMs.
**Limitations:** The current study has some inherent limitations. Firstly, it was a retrospective observational study conducted at a single centre, which may have introduced certain sampling biases. Second, the number of enrolled patients was relatively small. Given the rarity of CCMs, collecting larger datasets from multiple centres would be advantageous for the continued improvement of this classification system.
**Ethics committee approval:** Ethical approval for this study was obtained from the Institutional Review Board.
**Funding for this study:** This work was supported by grants from National High Level Hospital Clinical Research Funding (grant No. 2022-PUMCH-A-004) and Natural Science Foundation of China (grant No. 82271886)
**Author Disclosures:**
Yong-Lan He: Nothing to disclose
Zheng Yu Jin: Nothing to disclose
Hua-Dan Xue: Nothing to disclose
Jing Ren: Nothing to disclose
Chen Wang: Nothing to disclose
Menglin Huang: Nothing to disclose
Zhilin Yuan: Nothing to disclose
Xinyu Liu: Nothing to disclose
Yuan Li: Nothing to disclose

### RPS 2207-3
**Correlation between total fibrotic kidney volume and parasympathetic activity in autosomal-dominant polycystic kidney disease patients: a pilot study**
*A. Borrelli*, L. Laschena, S. Lucciola, A. Dehghanpour, C. Catalano, V. Panebianco; Rome/IT
*(antonella.borrelli@uniroma1.it)*

**Purpose:** In patients with autosomal-dominant polycystic kidney disease (ADPKD) cystic enlargement may cause parenchymal hypoxia, renin secretion, and endothelial dysfunction, causing hyperactivation of renin–angiotensin system and early hypertension, affecting blood pressure circadian rhythm. Aim of this study is to evaluate renal damage progression, indicated by MRI-based parameter, in ADPKD patients, correlating with sympathetic/parasympathetic balance using heart rate variability (HRV) parameters.
**Methods or Backround:** Sixteen adult ADPKD patients were enrolled, undergoing MRI with three Tesla scanner, to evaluate especially total kidney volume (TKV) and total fibrotic volume (TFV). A post-processed slice-by-slice renal segmentation, with colorimetric maps was obtained. A software was used for three-dimensional volume rendering reconstruction, which resulted in semiquantitative estimation of parenchymal perfused tissue and fibrotic areas, giving indication of functional parenchymal areas. These data were correlated with HRV parameters, calculated using 24 h-ECG Holter (low frequency, LF, and high frequency, HF).
**Results or Findings:** A statistically significant positive linear correlation was observed between length of kidneys and LF ($r = 0.595$, $p < 0.05$), and LF-day ($r = 0.587$, $p < 0.05$). Moreover, a statistically significant positive linear correlation exists between HF and TFV ($r = 0.804$, $p < 0.01$) and height- adjusted (ha) TFV ($r = 0.801$, $p < 0.01$). Finally, we found a statistically significant positive linear correlation between HFnight and TKV ($r = 0.608$, $p < 0.05$), ha-TKV ($r = 0.685$, $p < 0.01$), TFV ($r = 0.594$, $p < 0.05$) and ha-TFV ($r = 0.615$, $p < 0.05$).
**Conclusion:** The increase in TKV and TFV could lead to parasympathetic tone hyperactivation, probably in response to hypoxic stress and vasoconstriction due to cystic enlargement.
**Limitations:** Limited sample size: however, ADPKD represents a rare disease, therefore the sample may be considered representative
**Ethics committee approval:** Ethical approval for this study was obtained from the Institutional Ehtics committee.
**Funding for this study:** No funding was available for this study.
**Author Disclosures:**
Valeria Panebianco: Nothing to disclose
Antonella Borrelli: Nothing to disclose
Ailin Dehghanpour: Nothing to disclose
Ludovica Laschena: Nothing to disclose
Sara Lucciola: Nothing to disclose
Carlo Catalano: Nothing to disclose

### RPS 2207-4

**MRI of pelvic endometriosis: evaluation of the mr#Enzian classification and the importance of adenomyosis subtypes**
*A-M. Pausch*, V. Filleböck, M. Benli, I. Witzel, A. M. Hötker; Zurich/CH

**Purpose:** This study aimed to investigate the utility of the #Enzian classification in magnetic resonance imaging (MRI) for endometriosis assessment, focusing on inter-reader agreement, diagnostic accuracy, and the correlation of adenomyosis with deep infiltrating endometriosis (DIE).
**Methods or Backround:** This IRB-approved retrospective single-centre study included 412 women who underwent MRI evaluation for endometriosis between February 2017 and June 2022. Two experienced radiologists independently analysed MRI images using the #Enzian classification and assessed the type of adenomyosis, if any. The surgical #Enzian classification served as the gold standard for evaluating preoperative MRI results. Statistical analysis was performed to assess inter-reader agreement and diagnostic accuracy.
**Results or Findings:** Inter-reader agreement was substantial to excellent (Cohen's kappa 0.75 – 0.96) for most compartments except peritoneal involvement (0.39). The preoperative MRI showed mostly substantial to excellent accuracy (0.84 – 0.98), sensitivity (0.62 – 1.00), specificity (0.87 – 1.00), positive (0.58 – 1.00) and negative predictive values (0.86 – 1.00) for most compartments, except for peritoneal lesions (0.36, 0.17, 1.00, 1.00, 0.26 respectively). A trend with a higher prevalence of concordant DIE in women with external compared to those with internal adenomyosis was visible ($p = 0.067$).
**Conclusion:** The mr#Enzian showed mostly high inter-reader agreement and diagnostic accuracy for various endometriosis compartments. MRI's role is particularly significant in the context of the current paradigm shift towards medical endometriosis treatment. The inclusion of information about the type of adenomyosis in the mr#Enzian classification could enhance diagnostic accuracy and inform treatment planning.
**Limitations:** The study may have selection bias due to the highly subspecialised gynaecology department as referral source, moreover, the use of laparoscopy with histological confirmation as the gold standard, despite its known limitations, could introduce further bias.
**Ethics committee approval:** Cantonal Ethics Committee Zurich gave the ethical approval for this study.
**Funding for this study:** No funding was obtained for this study.
**Author Disclosures:**
Isabell Witzel: Nothing to disclose
Vivien Filleböck: Nothing to disclose
Antonia-Maria Pausch: Nothing to disclose
Merve Benli: Nothing to disclose
Andreas M. Hötker: Nothing to disclose

### RPS 2207-5

**The hidden connections: the relationship between endometrioma size, adenomyosis and deep infiltrating endometriosis**
*H. Özen Atalay*, M. S. Sogut, U. Kalkan, A. Durur Karakaya; Istanbul/TR
*(handeozen15@gmail.com)*

**Purpose:** The purpose of this study was to evaluate the association between endometrioma size, the presence of adenomyosis, and deep-infiltrating endometriosis.
**Methods or Backround:** The present retrospective study was performed at a single center, involving a total of 118 patients who were diagnosed with endometrioma. The longest diameter of the biggest endometrioma, the presence of adenomyosis, and the presence of deep-infiltrating endometriosis were evaluated in the pelvic MRI by two radiologists with consensus. The Kruskal-Wallis test, Pearson correlation coefficient and Fisher's exact test were used to calculate the correlation coefficient, and to detect possible relationships.
**Results or Findings:** Seventy-one patients have deep-infiltrating endometriosis, and thirty-one patients have adenomyosis, in total of 118 patients with endometrioma. The mean diameter of the endometriomas was 40.79±18.9 mm. The mean diameter of the endometrioma did not have a statistically significant effect on the presence of adenomyosis or deep-infiltrating endometriosis ($p=0.812$). There were weak correlations between the mean diameter of the endometrioma and the presence of adenomyosis and DIE ($r=0.028$ and $r=0.060$, $p>0.05$ respectively). However, there was a significant association between the presence of adenomyosis and deep-infiltrating endometriosis ($p=0.001$). The Pearson's r correlation test yielded a moderately positive correlation ($r=0.301$, $p=0.001$).
**Conclusion:** This study indicates that endometrioma size is not significantly correlated with the presence of adenomyosis or deep-infiltrating endometriosis. However, a significant association exists between the presence of adenomyosis and deep-infiltrating endometriosis. These findings suggest that while endometrioma size may not be a predictive factor, the co-occurrence of adenomyosis and deep-infiltrating endometriosis should be considered in clinical evaluations.
**Limitations:** This retrospective study included a limited number of patients from a single institution.
**Ethics committee approval:** Koc University Biomedical Research Ethics Committee, Istanbul/Turkey ethically approved this study.
**Funding for this study:** No funding was obtained for this study.
**Author Disclosures:**
Afak Durur Karakaya: Nothing to disclose
Hande Özen Atalay: Nothing to disclose
Muhammet Selman Sogut: Nothing to disclose
Uzeyir Kalkan: Nothing to disclose

### RPS 2207-6

**Photon-counting detector CT for kidney stone detection in contrast-enhanced urography: a comparison between virtual non-contrast and virtual non-iodine reconstructions in a 3D printed kidney phantom**
P. S. Breiding[1], M. Turrion[2], K. Martini[2], D. N. Nakhostin[2], H. Alkadhi[2], *A. Euler*[3]; [1]Bern/CH, [2]Zurich/CH, [3]Baden/CH

**Purpose:** The aim of this study was to compare contrast media subtraction, kidney stone visibility and subjective image quality between a novel virtual non-iodine reconstruction algorithm (VNI; PureCalcium) and a virtual non-contrast (VNC) algorithm on photon-counting detector CT (PCD-CT).
**Methods or Backround:** Calcium oxalate and uric acid stones were placed into contrast-filled calyces of a 3D printed kidney phantom and imaged on PCD-CT. Two blinded readers assessed quality of PC and VNC images using a five-point visual scale. The readers were asked to rate contrast media subtraction and erroneous kidney stone subtraction in each cylinder using a three-point visual scale.
**Results or Findings:** Overall image quality was rated higher for VNI images compared to VNC images by one reader (4.9 vs. 4.0; $p < 0.05$). Inter-reader agreement for how well contrast was subtracted was substantial for both VNC and PC images (Krippendorff's alpha = 0.628 and 0.731). Incomplete contrast subtraction was observed more frequently for VNI images compared to VNC images by one reader (29% vs. 15%; $p < 0.05$). Inter-reader agreement for kidney stone subtraction was substantial for both VNC and PC images (Krippendorff's alpha = 0.748 and 0.668). Kidney stones were subtracted more frequently with VNI compared to VNC (Reader 1: 22% vs. 16%; Reader 2: 25% vs. 10%; $p < 0.05$). Smaller stones were subtracted more frequently than larger stones irrespective of reconstruction method for both readers ($p < 0.05$).
**Conclusion:** Overall subjective image quality was higher for VNI images compared to VNC images. Incomplete contrast subtraction and subtraction of kidney stones was more frequent for VNI images.
**Limitations:** The phantom model may be prone to artifacts and does not reflect true tissue heterogenicitiy or dynamic tissue properties. We only used two keV levels to reconstruct the monoenergetic PC images.
**Ethics committee approval:** This study was ethically approved the Ethics committee - University of Zürich.

**Funding for this study:** No funding was obtained for this study.
**Author Disclosures:**
Katharina Martini: Nothing to disclose
Philipe Sebastian Breiding: Nothing to disclose
Dominik Nader Nakhostin: Nothing to disclose
Maria Turrion: Nothing to disclose
Andre Euler: Nothing to disclose
Hatem Alkadhi: Nothing to disclose

### RPS 2207-7

**Quantitative measurement of renal artery spin labelling imaging: a non-invasive index to evaluate perfusion in patients with renal artery stenosis**

*X. Zhang*, G. Zhang, L. Xu, X. Bai, J. Zhang, L. Chen, H. Sun, Z. Jin; Beijing/CN
*(zhang_xiaoxiao23@163.com)*

**Purpose:** The objective of this study was to evaluate the effect of interventional therapy on renal blood flow (RBF) in renal artery stenosis (RAS) patients and to explore the correlation between RBF and kidney glomerular filtration rate (GFR).
**Methods or Backround:** Eleven RAS patients underwent preoperative arterial spin labeling mapping (ASL) exam and interventional therapy were enrolled, and seven patients underwent postoperative ASL examination. We analysed 22 renal arteries and kidneys as independent subjects, dividing them into no renal artery stenosis, mild renal artery stenosis (≤75%), and severe renal artery stenosis (＞75%) groups based on the degree of luminal stenosis. We evaluated the correlation between preoperative cortical RBF and preoperative single kidney GFR, and differences between preoperative and postoperative RBF were compared.
**Results or Findings:** All RAS patients experienced a decrease in systolic and diastolic blood pressure post-interventional therapy. The RBF without renal artery stenosis was higher than that in the renal artery stenosis group (273.68 ±62.77 vs 172.05±62.44), and the RBF in the mild renal artery stenosis group was higher than that in the severe renal artery stenosis group (187.33±49.66 vs 156.77±75.21). A positive correlation was found between preoperative cortical RBF and preoperative single kidney GFR, with no significant correlation between preoperative cortical RBF and preoperative estimated GFR. The renal cortical RBF was higher than preoperative RBF (203.19±51.42 vs.164.00±68.10). The postoperative RBF in the region of abnormal perfusion was also higher than the preoperative RBF (159.92±46.10 vs. 108.55±39.57).
**Conclusion:** Renal RBF obtained by ASL images was significantly correlated with single renal function and could be used to evaluate the perfusion improvement in RAS patients after interventional therapy.
**Limitations:** The main limitations of this study are that the sample size is too small and that some patients did not undergo postoperative review.
**Ethics committee approval:** Ethical approval was sought from the Institutional Review Board of Peking Union Medical College Hospital.
**Funding for this study:** No funding was obtained for this study.
**Author Disclosures:**
Jiahui Zhang: Nothing to disclose
Gumuyang Zhang: Nothing to disclose
Hao Sun: Nothing to disclose
Lili Xu: Nothing to disclose
Xin Bai: Nothing to disclose
Li Chen: Nothing to disclose
Zhengyu Jin: Nothing to disclose
Xiaoxiao Zhang: Nothing to disclose

### RPS 2207-8

**Follow-up in acute pyelonephritis (PNA) using DWI sequences in MRI: our experience**

*G. Di Guardia*, P. Lasciarrea, F. Testa, V. Verna, S. Rubiolo, X. Kaci, F. Lucio, A. Cappelletti; Verduno/IT
*(diguardia.giuseppe@gmail.com)*

**Purpose:** Several studies have highlighted the role of MRI and diffusion-weighted sequences (MRI-DW) in the diagnosis of PNA and its impact regarding the planning of an adequate therapeutic strategy by nephrologists. The aim of our study is to evaluate the changes in signal alteration in diffusion-weighted sequences (DWI) and ADC values in patients (pts) with PNA, by comparison with the mean ADC value (ADCm) of both the pathological kidney and the contralateral, in acute and during follow-up.
**Methods or Backround:** From June 2016 to February 2020, 101 patients were studied (10 males, 91 females, average age 40 years, minimum age 18 years, maximum age 68 years) with clinical-laboratory diagnosis of PNA. 26/101 patients were evaluated retrospectively by evaluating the DWI sequences, demarcating circular regions of interest (ROI) on the areas of impaired signal and comparing them both with the ADCm value of the ipsilateral healthy renal parenchyma, and with the contralateral, obtaining an ADC ratio ( ADCr).
**Results or Findings:** In the acute phase (t0), 94 outbreaks of PNA were identified, with ADCm 1.26 ± 0.17 x 10-3 mm2 / s, ADCr0 = 0.64 ± 0.08. At follow-up after therapy (mean follow-up time 36.4 ± 11.7 days) we observed clinical-laboratory resolution in all patients, but only 39/94 foci demonstrated complete resolution on MRI (41%) in agreement with the data in the literature.
**Conclusion:** RM-DW allows a valid monitoring of PNA outbreaks, also identifying the different degrees of evolution. For the calculation of the ADCr we did not detect statistically significant changes using the free parenchyma of the pathological kidney compared to the contralateral kidney.
**Limitations:** No limitations have been identified for this study.
**Ethics committee approval:** No ethics committee approval was required.
**Funding for this study:** No funds were sought for this study.
**Author Disclosures:**
Xhorxhi Kaci: Nothing to disclose
Giuseppe Di Guardia: Nothing to disclose
Francesco Testa: Nothing to disclose
Agnese Cappelletti: Nothing to disclose
Stefano Rubiolo: Nothing to disclose
Francesco Lucio: Nothing to disclose
Valter Verna: Nothing to disclose
Paolo Lasciarrea: Nothing to disclose

### RPS 2207-9

**Reliable volume measurements in polycystic kidneys: a comparison between 3D volumetry and Mayo clinic's kidney volume calculator based on the ellipsoid equation**

*C. Gerlotti Slusnys*[1], M. Paniagua González[1], D. D´Elia Torrence[2], P. López Gómez[1], M. Camarena Gea[1], M. Poida[1], A. Garcia Perez[1], C. Calles Blanco[1], J. Amorim Sortino[1]; [1]Madrid/ES, [2]Logroño/ES
*(claudia.gerlotti@salud.madrid.org)*

**Purpose:** This study focuses on the precise determination of total kidney volume (TKV) in individuals with polycystic kidney disease (PKD). Accurate TKV measurement is important for predicting renal functional decline and assessing Tolvaptan treatment candidates. The widely used Mayo clinic ellipsoid equation is simpler but tends to underestimate TKV. We aim to assess its accuracy compared to the more precise 3D volumetry method.
**Methods or Backround:** We retrospectively studied 15 PKD patients (30 kidneys) who had CT scans from 2019 to 2023. TKV was calculated using the Mayo Clinic method (MCM) and 3D volumetry method (3DM) with manual segmentation. In this study, a predetermined threshold was set, deeming a difference of less than 10 percent between the two volumes as clinically not relevant according to expert nephrologists. The paired Student's T-test, dispersion, and Pearson's correlation analysis were employed for hypothesis testing.
**Results or Findings:** The paired Student's T-test revealed a statistically significant difference between the means of both methods, with an 8.55% variation. Dispersion analysis showed a range from -140 cc to +286 cc. Pearson's correlation analysis demonstrated a strong linear correlation, enabling the creation of a formula to predict 3DM volumetric values based on MCM calculations.
**Conclusion:** Our study, consistent with existing literature, found that the statistical difference between methods is below 10%. Although 3DM is more precise, MCM remains a simpler and practical TKV calculation option. The linear correlation allows us to predict 3DM values based on MCM results. In conclusion, both tests are functional, and we introduce a tool that could potentially reduce the need for 3D reconstruction.
**Limitations:** No limitations were identified.
**Ethics committee approval:** Not applicable
**Funding for this study:** No funding was received for this study.
**Author Disclosures:**
Claudia Gerlotti Slusnys: Nothing to disclose
Paola López Gómez: Nothing to disclose
Carlos Calles Blanco: Nothing to disclose
María Camarena Gea: Nothing to disclose
Mihail Poida: Nothing to disclose
Joaquim Amorim Sortino: Nothing to disclose
Miguel Paniagua González: Nothing to disclose
Angela Garcia Perez: Nothing to disclose
Diana D´Elia Torrence: Nothing to disclose

**08:00-09:00** **Room G1**

### Research Presentation Session: Radiographers

### RPS 2214

### A focus on MRI and mammography practice and care

**Moderators**
C. Aynes; Sabadell/ES
*(caynes@cspt.es)*
E. Divjak; Zagreb/HR
*(edivjak@gmail.com)*

### RPS 2214-3

**Comparing radiologists' and radiographers' assessment of quality and justification of lower back MRI referrals**

*C. C. Chilanga*[1], M. Heggelund[1], E. Kjelle[2]; [1]Drammen/NO, [2]Gjøvik/NO
*(catherine.chilanga@usn.no)*

**Purpose:** This study aimed to compare radiologists' and radiographers' assessment of the quality of Magnetic Resonance Imaging (MRI) lumbosacral spine referrals for low back pain (LBP).
**Methods or Backround:** A total of N=300 lumbosacral spine MRI referrals for LBP for adults aged 16–90 years were obtained. A registration form was designed consisting of seven statements on relevant information in the referral rated on a three-point scale: 'Yes', 'No' or 'Uncertain'. In addition, the referral was rated as 'Justified', 'Unjustified' or 'Need more information'. The registration form was pilot-tested on two radiologists to check for validity and reliability. Four radiologists and two radiographers were recruited and assigned the task to assess the same 300 referrals individually and complete the registration form. STATA Statistics packages were utilised to perform mixed model analysis to show variability in ratings between groups.
**Results or Findings:** Overall, an average of 65% (n=196) of the referrals were assessed as justified. There was a difference between radiographers and radiologists in the assessment of referral justification with 73.5% (95% CI [69.4%-77.7%]) and 58.4% (95% CI [54.5%-62.1%]) rated 'Justified' respectively. This variation was statistically significant (p-value <0.001). In referral quality, an average of 7% (n=22) received a 'good' quality score. Compared to the radiologists, the radiographers rated a statistically significant (p<0.001) higher percentage of the referrals as good quality, with radiologists rating 6.2% (95% CI [4.5%-8.0%] as good quality and radiographers rating 10% (95% CI [7.3%-12.6%]) to be of good quality.
**Conclusion:** The radiographers rated more LBP MRI referrals as 'Justified' and gave the referrals an overall higher quality score than radiologists.
**Limitations:** The limitations of the study are that data is only from a few raters, with an added unequal amount representation of professional groups, limiting generalising of the findings.
**Ethics committee approval:** The study was approved by the Regional Committees for Medical and Health Research Ethics (REK) reference number 378396 and the Norwegian Agency for Shared Services in Education and Research (SIKT) reference 261461.
**Funding for this study:** Funding was provided by The Research Council of Norway (Project number 302503).
**Author Disclosures:**
Mina Heggelund: Nothing to disclose
Elin Kjelle: Nothing to disclose
Catherine Chilute Chilanga: Nothing to disclose

### RPS 2214-4

**The most common patient-related and technical artefacts on breast MRI: how to recognise and overcome?**

*Z. Ljepoja*, M. M. Nadrljanski, I. B. Krušac, D. Dimitrijevic, L. Raspopovic, A. Djajic, M. Mihajlović; Belgrade/RS
*(zeljka_ljepoja@yahoo.com)*

**Purpose:** This study aimed to recognise the most common artefacts and misinterpretations of the objects in the field of view: patient-related (motion artefacts, artefacts due to positioning, metal artefacts) and technical (zipper artefacts, wrap-around artefacts, chemical shift artefacts and zebra artefacts).
**Methods or Backround:** 300 consecutive breast MRI exams, all realised with a full diagnostic protocol (T2W-STIR, T2W-TSE, T1W-TSE, FLASH 3D with the application of the same contrast medium: gadobutrol) on 1.5T MRI unit (realised: October 2020 –October 2023) were analysed for the preselected sets of artefacts (patient-related and technical).
**Results or Findings:** Artefacts were detected on 19.67% of all analysed MRI breast exams. Artefacts predominantly belonging to patients were 84.74%. Motion artefacts were 45.76%; artefacts due to positioning were 23.73%; metal artefacts were 15.25%. Only 15.25% of all artefacts were technical artefacts. Zebra artefacts were 5.08%; wrap-around artefacts were 3.39%; zipper artefacts were 3.39%, and chemical shift artefacts were 3.39%. Significantly more patient-related artefacts were detected (motion artefacts, p=4.45e-7), with the pval for distribution of 0.046, favouring the presence of motion artefacts. The diagnostic interpretation was affected by patient-related motion artefacts with 7 exams being rescheduled and repeated, i.e. 11.86% of all detected artefacts.
**Conclusion:** Technologists and radiologists need to recognise and understand the artefacts on breast MRI in order to provide the satisfactory and permanent quality of the images. Adequate patient preparation is important for the adequate image quality.
**Limitations:** This was a retrospective analysis.
**Ethics committee approval:** This was technical research, exempted from the decision of the ethical committee.
**Funding for this study:** No funding was received for this study.
**Author Disclosures:**
Iva B. Krušac: Nothing to disclose
Dejan Dimitrijevic: Nothing to disclose
Zeljka Ljepoja: Nothing to disclose
Marko Mihajlović: Nothing to disclose
Andjela Djajic: Nothing to disclose
Mirjan M. Nadrljanski: Nothing to disclose
Luka Raspopovic: Nothing to disclose

### RPS 2214-5

**A safety questionnaire to assess participant (paediatric and adult) experience of ultra-high-field MRI: theory, design, and implementation considerations**

*P. Bridgen*[1], K. Colford[1], B. Hansson[2], I. M. Björkman-Burtscher[3], T. Arichi[1], S. Malik[1], S. Giles[1], C. Malamateniou[1], G. Turner[1]; [1]London/UK, [2]Lund/SE, [3]Gothenburg/SE
*(philippa.bridgen@kcl.ac.uk)*

**Purpose:** The aim of this research was to create a robust, user-informed, evidence-based questionnaire for patient safety aspects at 7T, using local expertise and previous literature.
**Methods or Backround:** 7T MRI increases signal-to-noise-ratio (SNR) and improves contrast in comparison to standard magnetic field strengths, giving the potential for additional information clinically. However, patients can experience transient sensory effects during 7T examinations, which may impact patient experience and acceptability. Previous research has primarily focused on adult perception, with children perceptions so far only being extrapolated from 3T data. Expanding these questionnaires to children to gain further necessary information is needed. Literature searches were carried out looking for both MRI transient effects and questionnaire designs at all MRI field strengths. Content analysis of 32 articles was completed to identify common themes, directed the subject of questions asked, following the patients' MRI-scan journey. Answer formats included free comments and 5-point Likert scales. Questionnaires were adapted to be age-appropriate for 5-8 year and 8-11 olds, and adults. Language level was verified using the Flesch-Kincaid method. All questions were piloted (n=10) to gain feedback from intended users on content, design, and flow.
**Results or Findings:** Three comparable age-appropriate questionnaires were constructed, reflecting identified common themes from literature. Pictures aided understanding for children aged 5-8 years old. Questionnaires were divided into six sections: initial overview, positioning, entering, during the scan, exiting and post scan.
**Conclusion:** Newly designed questionnaires will allow a better understanding of how children or adults may experience 7T MRI and enhance safety strategies. Evidence collected from future use will support change of current practice.
**Limitations:** 7T is self-limiting due to the narrow scope, as such there is the potential for a lack of diversity of participants.
**Ethics committee approval:** Ethical approval granted from Committee of the School of Health Sciences, City, University of London under ETH2223-1703
**Funding for this study:** No funding was obtained for this study.
**Author Disclosures:**
Philippa Bridgen: Nothing to disclose
Tomoki Arichi: Nothing to disclose
Boel Hansson: Nothing to disclose
Isabella Maria Björkman-Burtscher: Nothing to disclose
Sharon Giles: Nothing to disclose
Shaihan Malik: Nothing to disclose
Kathleen Colford: Nothing to disclose
Christina Malamateniou: Nothing to disclose
Georgina Turner: Nothing to disclose

### RPS 2214-6

**Comparison between 0.3 Tesla and 1.5 Tesla MRI modalities in knee imaging**

*M. Jurjević*, N. Mekis, J. Izlakar; Ljubljana/SI
*(jurjevicmatej1997@gmail.com)*

**Purpose:** This study compared knee imaging at 0.3T and 1.5T field densities. We examined differences in signal-to-noise ratio (SNR), contrast-to-noise ratio (CNR), and image quality assessed by three experienced radiologists.
**Methods or Backround:** A sample of 25 left knees was examined using both MR devices with a 3 mm slice thickness. The volunteers were healthy and had no previous knee injuries. SNR and CNR measurements were performed on the medial meniscus, the distal part of the femur, articular cartilage, and the background. In the second part of the study, three radiologists assessed the image quality of the ACL, PCL, menisci, articular cartilage, and the overall image.
**Results or Findings:** In the SNR and CNR measurements, we recorded statistically significant differences in the area of the medial meniscus in favor of the 1.5T modality for both SNR ($p < 0.001$) and CNR ($p < 0.001$). Similar results were observed in the area of the distal part of the femur, with better values for the 0.3T modality for both SNR ($p < 0.001$) and CNR ($p < 0.001$). However, in the measurements of the distal femur, we did not find statistically significant differences in the values of SNR ($p = 0.677$) and CNR ($p = 0.861$). For all selected structures, radiologists rated the 1.5T modality higher, and this is supported by the statistically significant differences. The agreement between radiologists was moderate for the ratings of menisci, articular cartilage, and the overall image, and poor for the quality of cruciate ligaments.
**Conclusion:** Our results show superior image quality on 1.5T MRI, while indicating the potential for 0.3T open-type scanners in knee diagnostics.
**Limitations:** Smaller FOV in one plain on 0.3T to reduce wrap-around artifact. Surface array coil used on 1.5T scanner.
**Ethics committee approval:** This study received ethical approval from the National Medical ethics committee, approval number: 0120-356/2022/3.
**Funding for this study:** Funds for this study were obtained from the Institute of Oncology Ljubljana, Medisken Trbovlje.
**Author Disclosures:**
Nejc Mekis: Nothing to disclose
Jani Izlakar: Nothing to disclose
Matej Jurjević: Nothing to disclose

### RPS 2214-7

**Effect of noise exposure during 7 Tesla MRI**

*L. M. V. Wennberg*, P. C. Maly Sundgren, S. Waechter, J. Brännström, A. Jönsson, B. Hansson, J. Mårtensson; Lund/SE
*(linda.wennberg@med.lu.se)*

**Purpose:** This study aimed to assess the potential impact of noise exposure during a 7 Tesla (T) brain MRI in healthy adults.
**Methods or Backround:** Excessive noise can harm the cochlear outer hair cells, leading to auditory damage. In this study, we used otoacoustic emission (OAE) to evaluate the effects of noise exposure in 39 healthy adults after a 7T MRI scanning session utilizing currently accepted hearing protection. The participants were enrolled in a research project involving two one-hour MRI scanning sessions on the same day. OAE assessments were performed before and after each scan, with a follow-up performed one week later.
**Results or Findings:** Our analysis revealed no significant differences in outer hair cell function between the baseline measurements and the first MRI scan. A significant difference was observed at 1.5 kHz and 2 kHz in the left ear, as well as at 4 kHz in the right ear after the second MRI scan. However, the follow-up OAE measurement showed no significant difference compared to the baseline at any of the frequencies in either ear.
**Conclusion:** Our study's findings suggest no lasting effects on outer hair cell function in adults who undergo two one-hour MRI scanning sessions in a single day while using appropriate hearing protection.
**Limitations:** It should be noted that the participants in this study were restricted to healthy young adults who met specific criteria. Therefore, further investigations involving a more diverse group of individuals would be beneficial to gain a more comprehensive understanding of the impact of 7T MRI scanning sessions on different populations.
**Ethics committee approval:** This study was approved by the local ethics committee (registration numbers 2019-05387, 2016/126, and 2020-06907).
**Funding for this study:** This work was supported by grants from the Swedish Research Council (2017-00896) and the LMK Foundation. Both were awarded to Johan Mårtensson.
**Author Disclosures:**
Jonas Brännström: Nothing to disclose
Johan Mårtensson: Nothing to disclose
Boel Hansson: Nothing to disclose
Linda Maria Viviann Wennberg: Nothing to disclose
Pia C. Maly Sundgren: Nothing to disclose
Sebastian Waechter: Nothing to disclose
Anders Jönsson: Nothing to disclose

### RPS 2214-8

**Supplementary breast cancer screening in women with dense breasts: insights from European radiographers and radiologists**

*D. Mizzi*[1], C. Allely[2], F. Zarb[1], C. Mercer[2]; [1]Msida/MT, [2]Salford/UK
*(deborah.mizzi@um.edu.mt)*

**Purpose:** This study explored the understanding of challenges and requirements for implementing supplementary breast cancer screening for women with dense breasts among clinical radiographers and radiologists in Europe.
**Methods or Backround:** Fourteen semi-structured online interviews were conducted with European clinical radiologists (n=5) and radiographers (n=9) specializing in breast cancer screening from eight different countries including United Kingdom, Malta, Italy, the Netherlands, Greece, Finland, Denmark and Switzerland. The interview schedule comprised questions regarding professional background and demographics and 13 key questions divided into six subgroups, namely supplementary imaging; training; resources and guidelines; challenges; implementing supplementary screening and the women's perspective of supplementary imaging. Data analysis followed the six phases of reflexive thematic analysis.
**Results or Findings:** Six significant themes emerged from the data analysis: understanding and experiences of supplementary imaging for women with dense breasts; challenges and requirements related to training among clinical radiographers and radiologists; awareness among radiographers and radiologists of guidelines on imaging women with dense breasts; challenges to implement supplementary screening; predictors of Implementing supplementary screening and Views of radiologists and radiographers on women's perception towards supplementary screening.
**Conclusion:** The interviews with radiographers and radiologists provided valuable insights into the challenges and potential strategies for implementing supplementary breast cancer screening. These challenges included cost and logistics problems and patient and staff related challenges. Implementing multifaceted solutions such as Artificial Intelligence integration, specialised training and resource investment can address these challenges and promote the successful implementation of supplementary screening in women with dense breasts. Further research and collaboration are needed to refine and implement these strategies effectively.
**Limitations:** The data collection period coincided with the reopening of screening units after COVID-19 closures. During this period, participants were exceptionally busy, which limited their availability to partake in the study.
**Ethics committee approval:** Ethical permission was attained from the University of Salford School of Health and Society Research Ethics Committee.
**Funding for this study:** This work is part of a PhD programme which is part-financed by the Tertiary Education Scholarship Scheme (TESS), Government of Malta (TESS Contract MEDE 417/2018/61).
**Author Disclosures:**
Deborah Mizzi: Nothing to disclose
Clare Allely: Nothing to disclose
Francis Zarb: Nothing to disclose
Claire Mercer: Nothing to disclose

### RPS 2214-9

**Mean glandular dose in mammography with breast implants**

*E. Alukić*, A. Simunovic, N. Mekis, M. Marolt Music; Ljubljana/SI
*(erna.alukic@zf.uni-lj.si)*

**Purpose:** The aim was to evaluate the mean glandular dose (MGD) in screening mammography in patients with breast implants and to investigate the differences in MGD depending on the type of exposure setting - automatic exposure control (AEC) or manual exposure technique.
**Methods or Backround:** A retrospective study with secondary data analysis included 536 patients with breast implants who completed screening mammography between 2008 and 2021. MGD, breast thickness, and type of exposure setting were assessed and compared using standard mammography and the Eklund technique. Image quality was assessed by three radiologists experienced in mammography assessment.
**Results or Findings:** MGD is statistically significantly lower for breast thicknesses of 6 < 8 cm, 8 < 10 cm, and > 10 cm for standard images with manual exposure technique. For additional images with the AEC system, the mean MGD is statistically significantly lower for breast thicknesses of < 4 cm and 4 < 6 cm. For additional images with breast thickness of 6 < 8 cm, the MGD value is significantly lower with the manual exposure technique. There are statistically significant differences in MGD between images taken with standard mammography and those taken with the Eklund technique for both types of exposure settings. The overall assessment of all quality criteria images was statistically significantly higher for the manual exposure technique.
**Conclusion:** For additional images with breast thickness < 4 cm and 4 < 6 cm, the AEC system is better due to the lower MGD values. The manual mode is more suitable for breast thicknesses of 6 > 8 cm.
**Limitations:** No limitations.

**Ethics committee approval:** This study was ethically approved by the Commission of the Republic of Slovenia for Medical Ethics, Document number: ERIDEK-0047/2022.
**Funding for this study:** No funding was obtained for this study.
**Author Disclosures:**
Nejc Mekis: Nothing to disclose
Anja Simunovic: Nothing to disclose
Erna Alukić: Nothing to disclose
Maja Marolt Music: Nothing to disclose

### RPS 2214-10

**Assessing the potential of digital breast tomosynthesis as a primary breast cancer screening tool in Malta: identifying beneficiaries and advancements**

*M. Pule*[1], D. Mizzi[1], K. Borg Grima[2]; [1]Msida/MT, [2]Naxxar/MT
*(maria.pule.13@um.edu.mt)*

**Purpose:** The aim of the study was to prospectively identify who would benefit from having a digital breast tomosynthesis (DBT) as a first line screening. This was done by identifying different characteristics of patients being referred for a DBT at the local screening centre during further assessment clinics. The objectives of phase 1 were to audit the referrals for further assessment clinics within the local screening centre. In phase 2, data on the reason of referral together with the different patient characteristics, and the imaging results was collated to be able to reach set objectives.
**Methods or Backround:** The study involved two sequential phases. Phase 1 consisted of a retrospective analysis of statistical figures on the use of DBT locally from 2015 to 2021. Phase 2 was a prospective study composed of a self-designed patient questionnaire distributed between March and April 2022, required to evaluate any link between the different patient characteristics and the clinical outcome following a DBT.
**Results or Findings:** Phase 1 included 2,756 cases, where 35.9% had their first mammogram while 64.1% had a subsequent mammogram. First time mammogram cases were statistically the most likely to be returned back to normal screening (37.8% n=990). In both phases, asymmetric densities was the most common reason of referral. For phase 2, 53 participants were recruited. Results indicated that the most common types of Mammographic Breast Densities were type BIRADS A (30.2 %) and B (50.9%).
**Conclusion:** From the results collected in both phases it was shown that women screened for the first-time would benefit from DBT as a first line screening tool since they are more likely to be returned back to normal screening.
**Limitations:** Due to COVID-19 restrictions, the sample population for phase 2 was limited.
**Ethics committee approval:** Approval for this research was then obtained from the Faculty Research Ethics Committee (FHS-2021-00023) and from the University Research Ethics Committee within the University of Malta.
**Funding for this study:** No funding was received for this study.
**Author Disclosures:**
Karen Borg Grima: Nothing to disclose
Deborah Mizzi: Nothing to disclose
Maria Pule: Nothing to disclose

**09:30-11:00 Research Stage 1**

### Research Presentation Session: Artificial Intelligence & Machine Learning & Imaging Informatics

### RPS 2305

### Cutting edge advances in AI for radiological imaging

**Moderator**
S. Volpe; Milan/IT
*(stefania.volpe@ieo.it)*

### RPS 2305-2

**Automatic detection of bone fragility in radiographic images using deep-learning with multicentre cohort datasets**

*G. Gatineau*[1], G. Nguyen[2], M. De Gruttola[2], K. Hind[2], M. Kuzma[3], J. Payer[3], G. Guglielmi[4], A. Fahrleitner-Pammer[5], D. Hans[1]; [1]Lausanne/CH, [2]Geneva/CH, [3]Bratislava/SK, [4]Foggia/IT, [5]Graz/AT

**Purpose:** This study aimed to assess the accuracy and efficacy of an novel AI-driven radiographic processing tool designed to opportunistically identify individuals predisposed to very high bone fragility risk, addressing a prevailing clinical challenge in the field.
**Methods or Backround:** From four multinational cohorts, 4,764 paired lumbar-spine X-ray DICOM and DXA scans (GE and Hologic systems) were acquired within 6 months. A total of 3,369 cases from three cohorts were allocated for training and validation of a new AI-bone fragility detection tool (Medimaps Group, Switzerland). Three hundred cases were designated as internal test set. Two hundred and seventy-one cases from the fourth cohort acted as an external test set. Very high fracture risk was defined using DXA parameters as the ground truth: BMD T-score≤-2.5 and a trabecular bone score (TBS)<1.23.
**Results or Findings:** The mean age and BMI of the sample (5.2% male) were 66.1±10.8 y and 26.4±5.0 kg/m2 respectively. Using the combination of DXA-derived BMD and TBS, 17.5% were identified at very high fracture risk. Uncertainties were obtained with a 95% confidence interval (CI) using binomial distribution approximations. The accuracy of the AI tool for the internal test set, was 0.85 (95% CI: 0.76-0.94), specificity 0.91 (0.8-0.99), and sensitivity 0.69 (0.53-0.84). For external validation, the accuracy, specificity, and sensitivity were 0.80 (0.69-0.87), 0.88 (0.77-0.99), and 0.62 (0.47-0.77) respectively.
**Conclusion:** This AI-enhanced radiographic tool exhibits potential in accurately detecting individuals at very high risk of bone fragility. Its robust specificity underscores its capacity to reduce false-positive rates, emphasising its clinical utility for efficient patient screening.
**Limitations:** While this study demonstrates promise, further development and validation will be beneficial, using larger and more diverse samples.
**Ethics committee approval:** This study was ethically approved by the WMA declaration of Helsinki. Ethical Principles For Medical Research Involving Human Subjects
**Funding for this study:** This study was funded by the Fond National Suisse 32473B_156978 and 320030_188886.
**Author Disclosures:**
Astrid Fahrleitner-Pammer: Nothing to disclose
Michele De Gruttola: Employee: Medimaps group
Guillaume Gatineau: Nothing to disclose
Giuseppe Guglielmi: Nothing to disclose
Didier Hans: CEO: Medimaps group
Giao Nguyen: Employee: Medimaps group
Martin Kuzma: Nothing to disclose
Karen Hind: Employee: Medimaps group
Juraj Payer: Nothing to disclose

### RPS 2305-3

**Spectral CT fingerprinting: AI-based clustering of tissue signatures: proof of concept**

*N. Große Hokamp*, M. M. Thakur, D. Maintz, M. Schöneck, L. Caldeira; Cologne/DE
*(nils@grossehokamp.de)*

**Purpose:** Spectral computed tomography (CT) allows for Material Decomposition by assessing attenuation caused by photoelectric effect and compton scattering seperately. Followed by dedicated reconstruction algorithms, dedicated spectral results can be generated that highlight different material characteristics. We claim that combining these quantitative characteristics will result in a characteristic CT-signature or fingerprint of

Sunday tissues and that this clustering can be automated using artificial intelligence. This study served as proof-of-concept of this approach in a phantom setup.
**Methods or Backround:** A 3D-printed phantom filled with different solutions containing iodine (2 different concentrations), gadolinium, iron oxide and water was scanned using a dual layer spectral CT system. Conventional images, iodine maps and low energy virtual monoenergetic images were reconstructed. An unsupervised, high dimensional, k-means clustering algorithm was developed and used to automatically provide clusterings. These clusters were then forward-projected into the image and visually checked for agreement.
**Results or Findings:** Aforementioned high-dimensional, AI-based clustering allowed for cluster formation (mean RMSE = 0.68, range [0.06-1.63]). Forward projection revealed, that clusters match very closely to actual phantom compartments as indicated by DICE coefficients (all >0.8).
**Conclusion:** CT Fingerprinting, i.e. the AI-based derivation of a tissue signature ist feasible from a technology standpoint. Future research needs to focus on elevating the dimension of the clustering algorithm and on clinical translation.
**Limitations:** The study has been carried out in a phantom only, as it should serve as proof-of-concept. Only 3 dimensions have been introduced in the clustering approach to date.
**Ethics committee approval:** This was a phantom study and hence, no ethical approval was sought.
**Funding for this study:** This study was funded by the Advanced Clinician Scientist Program (AdCCSP), University of Cologne.
**Author Disclosures:**
Manali Mahadeo Thakur: Nothing to disclose
Mirjam Schöneck: Nothing to disclose
Liliana Caldeira: Nothing to disclose
David Maintz: Speaker: Philips
Nils Große Hokamp: Research/Grant Support: Philips Speaker: Amboss Speaker: Philips Consultant: Bristol Myers Squibb

### RPS 2305-4

**Pre-operative prediction of axillary lymph node status using ML applied to breast US: a multicentre study**

*M. Caruso*, A. De Giorgio, R. Cuocolo, L. R. La Rocca, M. Ferrante, A. Stanzione, S. Maurea, V. Romeo, A. Brunetti; Naples/IT

**Purpose:** To assess whether a machine-learning (ML) algorithm could empower the ability of US to preoperatively define axillary lymph node (ALN) status in breast cancer (BC) using a multicentric dataset.
**Methods or Backround:** Patients with at least one histologically proven BC lesion, who underwent preoperative breast US, were retrospectively selected in three different Institutions. BC lesions were segmented on US images by three different operators and radiomics features (first, second higher order) were extracted. Multi-step feature selection was performed using Intraclass Correlation Coefficient (ICC) analysis and principal component analysis (PCA). Thereafter, a Random Forest (RF) ML classifier was applied to the dataset to predict the ALN status (positive/negative for metastasis) and its performance assessed through the Matthews Correlation Coefficient (MCC).
**Results or Findings:** A total of 293 BCs (ALN negative: 176; ALN positive: 117) were included in the study. Three datasets were identified as follows: 1) training set, composed of 235 BCs (ALN-: 141; ALN+: 94); 2) validation set including 30 BCs (ALN-: 17; ALN+: 13); and 3) test set made of 30 BCs (ALN-: 18; ALN+: 12). 549 radiomics features were extracted from US images; of these, 280 were discarded according to ICC analysis, with a total of 5 features finally selected by PCA. RF classifier showed a MCC of 0.97, 0.11 and 0.08 in the training, validation, and test set, respectively.
**Conclusion:** ML applied to a multicentric dataset showed promises in the preoperative assessment of ALN status in BC. However, further efforts are necessary to improve the generalisability of the model when applied to external datasets.
**Limitations:** Limited sample size
**Ethics committee approval:** The study was approved by local ethics committee.
**Funding for this study:** No funding was obtained for this study.
**Author Disclosures:**
Ludovica Rita La Rocca: Nothing to disclose
Andrea De Giorgio: Nothing to disclose
Marilina Ferrante: Nothing to disclose
Valeria Romeo: Nothing to disclose
Arnaldo Stanzione: Nothing to disclose
Arturo Brunetti: Nothing to disclose
Renato Cuocolo: Nothing to disclose
Martina Caruso: Nothing to disclose
Simone Maurea: Nothing to disclose

### RPS 2305-5

**Prediction of patient survival with residual convolutional neural network (ResNet) in unresectable synchronous liver-only metastatic colorectal cancer treated with bevacizumab-based chemotherapy**

*S-H. Chiu*, C-C. Wu, W-C. Chang, P-Y. Chang; Taipei/TW
*(doc62207@gmail.com)*

**Purpose:** To verify the prediction of survival with residual convolutional neural network (ResNet)-determined morphological response (ResNet-MR) in patients with unresectable synchronous liver-only metastatic colorectal cancer (mCRC) treated with bevacizumab-based chemotherapy (BBC).
**Methods or Backround:** A retrospective review of liver-only mCRC patients treated with BBC from December 2011 to April 2021 was performed. Patients with metachronous liver metastases or receiving locoregional treatment before initiation of BBC were excluded. Downstaging to curative treatment and overall survival (OS) were recorded. Two abdominal radiologists evaluated CT images based on the morphological criteria and divided images into group 1, 2, and 3. These divided images established the radiologists-determined morphological response (RD-MR), which classified patients into responders and non-responders based on the morphological change 3 months after initiation of BBC. Then, group 1 and 3 images divided by radiologists were inputted into ResNet as training dataset. The trained ResNet redivided group 2 images into group 1 and 3. ResNet-MR was determined by these redivided images and initial group 1 and 3 images determined by radiologists.
**Results or Findings:** Eighty-four patients were enrolled (53 male with median age 60.0 years old). The follow-up time ranged from 10-86 months. There were 407 group 1 and 3 images imputed into ResNet as training dataset. Both RD-MR and ResNet-MR correlated with OS (p-value = 0.0167 and 0.0225, respectively). RD-MR classified 28 patients (33.3%) as responders, and ResNet-MR classified additional 16 patients (19.0%) as responders; these 16 patients showed longer OS than the rest of non-responders in RD-MR (27.49 versus 21.20 months, p-value=0.043), and had higher percentage to reach downstaging (37.5% versus 17.5%, p-value=0.1610).
**Conclusion:** ResNet showed it ability to predict therapeutic effect of BBC in mCRC patients, which will optimise individualised cancer treatment.
**Limitations:** A single-centre retrospective study .
**Ethics committee approval:** The study was approved with the approval protocol number: TSGHIRB No. 2-108-05-153, under the protocol title "Development of artificial intelligence approaches for Detection and Characterization of Hepatic Lesion and Evaluation of HCC treatment".
**Funding for this study:** Funding was obtained for this study with the funding number: Nsc 109-2314-B-016-012.
**Author Disclosures:**
Wei-Chou Chang: Nothing to disclose
Sung-Hua Chiu: Nothing to disclose
Chao-Cheng Wu: Nothing to disclose
Ping-Ying Chang: Nothing to disclose

### RPS 2305-6

**Predicting histological response in pancreatic adenocarcinoma treated with neoadjuvant chemotherapy: conventional clinicoradiological variables and added value of radiomics**

*J. Mushtaq*, D. Palumbo, F. Prato, M. Mori, S. Crippa, M. Falconi, C. Fiorino, F. De Cobelli; Milan/IT
*(mushtaq.junaid@gmail.com)*

**Purpose:** Pancreatic cancer prognosis remains abysmal due to late-stage diagnosis and limited treatment options. Neoadjuvant chemotherapy (NAT) is increasingly used to improve outcomes. However, it complicates radiological assessment, making it difficult to accurately determine tumor status. This study aims to explore radiomics as a non-invasive tool for predicting important prognostic factors and compares its performance with traditional radiological assessments.
**Methods or Backround:** This retrospective single-centre study included patients with pancreatic adenocarcinoma who underwent NAT and pancreaticoduodenectomy between January 2015 and December 2021. Clinical, radiological and pathological data were collected, and endpoints identified: disease recurrence, N2, Tumor Regression Grade (TRG). Radiomic features were extracted from pre-NAT and post-NAT CT images. Machine learning approaches, including bootstrapping, were used to develop predictive models. Two models were built: a purely radiomic model and a combined model including clinical and radiological data. The population was divided in a training and a validation cohorts. Balanced groups ensured robust analysis following established guidelines.
**Results or Findings:** The study included 156 patients (training n=103, validation n=53). Radiological and clinical variables, except for delta Ca19.9 levels, failed to predict the endpoints. Radiomics, using delta values to capture changes in the tumor microenvironment, showed promise.

Delta radiomic models were successfully validated for the N2 and TRG endpoints and performed moderately well with AUCs of 0.749 (p=0.031) and 0.710 (p=0.046) respectively. Excellent negative predictive values, 82% and 79% respectively for N2 and TRG, indicate the model's ability to identify low-risk patients. A combined model with delta CA19.9 did not significantly improve performance.
**Conclusion:** Radiomics may hold potential for improved patient selection in pancreatic cancer treatment.
**Limitations:** The study is limited by its retrospective nature and single-center design. External validation is necessary for confirming the results.
**Ethics committee approval:** This retrospective study was conducted at the San Raffaele Scientific Institute (Milan, Italy) and was carried out within the framework of an approved Ethics Committee study (28/INT/2015).
**Funding for this study:** No funding was obtained for this study.
**Author Disclosures:**
Claudio Fiorino: Nothing to disclose
Junaid Mushtaq: Nothing to disclose
Diego Palumbo: Nothing to disclose
Massimo Falconi: Nothing to disclose
Francesco De Cobelli: Nothing to disclose
Martina Mori: Nothing to disclose
Francesco Prato: Nothing to disclose
Stefano Crippa: Nothing to disclose

### RPS 2305-7

**Monitoring over time of pathological complete response to neoadjuvant chemotherapy in breast cancer patients through an ensemble vision transformers-based model**

*M. C. Comes*, S. Bove, A. Fanizzi, R. Massafra; Bari/IT
*(m.c.comes@oncologico.bari.it)*

**Purpose:** Morphological and vascular peculiarities of breast cancer can change during neoadjuvant chemotherapy (NAC). Dynamic contrast-enhanced magnetic resonance imaging (DCE-MRI) acquired pre- and mid-treatment quantitatively capture information about tumour heterogeneity as potential earlier indicators of pathological complete response (pCR) to NAC in breast cancer. This study aimed to develop an ensemble deep learning-based model, exploiting a Vision Transformer (ViT) architecture, which merges features automatically extracted from five segmented slices of both pre- and mid-treatment exams containing the maximum tumour area, to predict and monitor pCR to NAC.
**Methods or Backround:** Imaging data analysed in this study referred to a cohort of 86 breast cancer patients, randomly split into training and test cohorts at a ratio of 8:2, who underwent NAC and for which information regarding the pCR achievement was available (37.2% of patients achieved pCR). As far as we know, our research is the first proposal using ViTs on DCE-MRI exams to monitor pCR over time during NAC.
**Results or Findings:** The performances of the proposed model were assessed using standard evaluation metrics and promising results were achieved: AUC value of 91.4%, accuracy value of 82.4%, a specificity value of 80.0%, a sensitivity value of 85.7%, precision value of 75.0%, F-score value of 80.0%, G-mean value of 82.8%.
**Conclusion:** Finally, the heterogeneity changes in DCE-MRI at pre- and mid-treatment could affect the accuracy of pCR prediction to NAC.
**Limitations:** The study needs to be validated in a larger cohort.
**Ethics committee approval:** The study was approved by the Ethic Commitee of Istituto Tumori 'Giovanni Paolo II', Bari, Italy.
**Funding for this study:** The funding for this study was obtained by the Ministery of Health, Italy.
**Author Disclosures:**
Samantha Bove: Nothing to disclose
Raffaella Massafra: Nothing to disclose
Maria Colomba Comes: Nothing to disclose
Annarita Fanizzi: Nothing to disclose

### RPS 2305-8

**Retrospective evaluation of interval breast cancer: can the number of interval carcinomas be reduced utilising AI diagnostic software?**

*J. Subelack*[1], A. Geissler[1], J. Vogel[1], M. Blum[1], A. Eichenberger[1], R. Morant[1], A. Gräwingholt[2], D. Kuklinski[1]; [1]St. Gallen/CH, [2]Paderborn/DE
*(jonas.subelack@unisg.ch)*

**Purpose:** We investigate whether an artificial intelligence (AI) powered mammography screening software can support radiologists and screening programs to reduce interval carcinomas (ICs).
**Methods or Backround:** Combining data from the cancer registry of eastern Switzerland and the "donna" screening program, we include data from 151,245 screening mammograms between 2010 and 2019. Hereby, 264 ICs were identified when a carcinoma was detected opportunistically within 24 months after screening. Mammograms of the 264 ICs which in the screening round prior to detection were considered as normal and 90 randomly selected true normals were reviewed retrospectively by three independent radiologists and an AI-diagnostic software (ProFound-AI, iCAD). The software calculates three measures: (1) lesion-score: probability of a marked lesion in a mammogram to be a cancer; (2) case-score: probability whether the set of mammograms of one woman contains a cancer; (3) risk-category: woman's risk to be detected with breast cancer within two years. We evaluate which measure (combination) finds best signs of later detected ICs in the prior mammograms that were read as normal and also compare it to the review of the three radiologists. We further estimate the accuracy of the software depending on thresholds.
**Results or Findings:** We expect that both retrospective assessments have an improved detection rate of ICs compared to the initial screening. Detection probability of ICs and the number of false-positives is highly dependent on the thresholds set for the measure(s). Using predefined thresholds, we expect that the software is superior to the retrospective assessments of radiologists in detecting ICs.
**Conclusion:** If the software detects significantly more cancer signs than the radiologists, it needs to be discussed how best to integrate the software into the mammography screening process to reduce ICs.
**Limitations:** Only ICs are analysed in depth, limiting the scope of this study.
**Ethics committee approval:** A declaration of non-responsibility is at hand.
**Funding for this study:** The cancer league of eastern Switzerland funds this cooperative research project.
**Author Disclosures:**
David Kuklinski: Nothing to disclose
Alena Eichenberger: Nothing to disclose
Jonas Subelack: Nothing to disclose
Alexander Geissler: Nothing to disclose
Justus Vogel: Nothing to disclose
Axel Gräwingholt: Nothing to disclose
Rudolf Morant: Nothing to disclose
Marcel Blum: Nothing to disclose

### RPS 2305-9

**Exploring the potential of ChatGPT and a context-aware ChatGPT in gastrointestinal radiology: a differential diagnostic accuracy assessment**

*S. Rau*, A. Rau, J. Nattenmüller, A. Fink, F. Bamberg, M. Russe; Freiburg im Breisgau/DE
*(stephan.rau@uniklinik-freiburg.de)*

**Purpose:** The growing volume of imaging studies necessitates tools to assist radiologists in delivering accurate and effective diagnoses. We aimed to investigate the potential of ChatGPT by OpenAI and a context-aware chatbot based on ChatGPT in providing differential diagnoses.
**Methods or Backround:** Utilizing the LlamaIndex framework, which integrates datasets into large language models, ChatGPT 4 was enhanced using the 96 documents from the Radiographics Top 10 Reading list on gastrointestinal imaging, creating a gastrointestinal imaging-aware chatbot (giaGPT). To assess the differential diagnostic capability, a set of fifty case-files on abdominal pathologies was created, comprising of radiological findings in fluoroscopy, MRI and CT. We compared the giaGPT to the generic ChatGPT 4 (gGPT) in terms of offering the top three differential diagnoses using interpretations from senior level radiologists as ground truth. Additionally, the trustworthiness of the giaGPT was evaluated by investigating the utilized source document as provided by the embedded context-retrieval mechanism.
**Results or Findings:** Within the evaluated dataset, the giaGPT demonstrated a high capability in identifying the most appropriate differential diagnosis in a vast majority of cases with a sensitivity of 78%, significantly surpassing the gGPT with 54%. Notably, giaGPT offered the primary differential in the top 3 differential diagnoses in 90% of the cases, gGPT in only 74%.
**Conclusion:** Context-aware ChatGPT-based algorithms may provide a promising tool for supporting radiologists in the task of differential diagnostics. It offers accurate differentials and direct access to the employed source documents, providing insight into the decision-making process, providing trustworthy and evidence-based clinical decision-support.
**Limitations:** Although the findings are promising, they call for a more extensive evaluation using datasets from clinical routine covering a more diverse spectrum of pathologies. Additionally, future research should focus on the impacts of diagnostic confidence and time efficiencies.
**Ethics committee approval:** No ethics committee decision was needed in the study because no patient data were used.
**Funding for this study:** This study was financially supported by internal funds.
**Author Disclosures:**
Johanna Nattenmüller: Nothing to disclose
Alexander Rau: Research/Grant Support: Supported by Berta-Ottenstein-Programme for Clinician Scientists, Faculty of Medicine, University of Freiburg.
Anna Fink: Nothing to disclose
Maximilian Russe: Nothing to disclose
Fabian Bamberg: Speaker: Bureau of Siemens Healthineers; Grant Recipient: Bureau of Siemens Healthineers.
Stephan Rau: Nothing to disclose

### RPS 2305-10

**CT effective dose in intensive care unit patients: comparison between deep learning image reconstruction, filtered back projection and iterative algorithms**

*E. Agostini*, E. K. Lanza De Cristoforis, C. Zanon, E. Quaia; Padua/IT
*(elena.agostini.1@studenti.unipd.it)*

**Purpose:** The aim of this study was to assess the effective dose reduction and image quality improvement provided by deep learning image reconstruction (DLIR) in comparison to filtered back projection (FBP) and iterative reconstruction (IR) algorithms.
**Methods or Backround:** Eighty-three critical care patients who underwent CT imaging of the chest, abdomen and trunk (chest + abdomen) within a period of 30 days using both DLIR (TrueFidelity) and FBP or IR hybrid (AIDR3D) and model-based IR algorithm (ADMIRE) were included. All examinations were performed using automatic exposure control (AEC) which modulates the tube current and hence radiation exposure according to the algorithm applied. Radiation dose was assessed using CT dose index volume (CTDI volume), dose-length product (DLP) and Effective Dose. For the quantification of image quality, noise and signal to noise ratio (SNR) were used. All parameters were compared across the different reconstruction methods for each patient using both parametric (t test) and non-parametric (Wilcoxon) tests. In cases of contrast-enhanced CT (CECT), all parameters were retrieved for every acquisition phase (direct, arterial, venous or delayed) as well as for their total value as stated in the patient protocol.
**Results or Findings:** DLIR vs FBP improved both CTDIvol (9.56 ± 5.86 vs 24.67 ± 61.01), DLP (1085.33 ± 626.30 vs 1350.62 ± 1191.68), and effective dose (16.13 ± 9.55 vs 20.19 ± 17.9), while DLIR vs FBP and vs IR improved both image noise (8.45 ± 3.24 vs 28.85 ± 32.77 vs 14.85 ± 2.73 HU) and SNR (3.99 ± 1.23 HU vs 11.53 ± 9.28 vs 4.84 ± 2.74 HU).
**Conclusion:** DLIR provides benefits in terms of effective dose and image quality over the traditional FBP. It also outperforms IR methods for image quality, but not for effective dose.
**Limitations:** No limitations were identified.
**Ethics committee approval:** The study was approved by the institutional review board.
**Funding for this study:** No funding was received for this study.
**Author Disclosures:**
Elena Agostini: Nothing to disclose
Chiara Zanon: Nothing to disclose
Emilio Quaia: Nothing to disclose
Elena Kiyomi Lanza De Cristoforis: Nothing to disclose

### RPS 2305-11

**Insights from a commercial AI tool's performance in detecting common acute and chronic chest radiograph abnormalities in a multi-ethnic Singapore dataset using AimSG's automated auditing tools**

*T. S. E. Tan *, G. Han Leong, J. Zou, C. Liew; Singapore/SG
*(timothy.tan.shao.ern@doctors.org.uk)*

**Purpose:** To clinically validate the performance of a machine learning-based algorithm tool in identifying abnormal findings on chest radiographs (CXRs).
**Methods or Backround:** Two consultant radiologists with between 5 and 15 years of experience read 300 adult CXRs performed in 2021 at a high-volume tertiary Singapore acute hospital to establish ground truth. The Lunit INSIGHT CXR (South Korea) artificial intelligence (AI) algorithm was integrated into the hospital RISPACS on a national vendor-neutral AI imaging platform (AimSG, Singapore), which contains an integrated data stack and performance monitoring data tools (Carpl.AI, New Delhi). Audit of the AI algorithm's performance on the CXRs was compared with the radiologists' analyses on AimSG. The area under the receiver operating characteristic curve (AUROC) was calculated for ten specific abnormalities.
**Results or Findings:** Three hundred adults (median age 75.7, range +/- 15.6 ; 182 males, 61%) comprising 183 (61% Chinese), 96 (32% Malays), 16 (5% Indians), and 5 (2%) other ethnicities had CXRs performed for acute respiratory and thoracic symptoms. A total of 281 (93.7%) of 300 CXRs demonstrated at least one acute or chronic abnormality. The algorithm achieved an AUROC of between 0.95 and 0.99 on this dataset at a threshold of 50%. The F1 score ranged from 0.3-0.76 for chronic findings such as lung nodules, calcifications, and fibrosis to between 0.86-0.93 for acute/life-threatening findings such as pleural effusion, pneumothorax, and consolidation, with the highest positive predictive value of 0.97 for consolidation.
**Conclusion:** The AI algorithm performed better in identifying acute or life-threatening findings compared to chronic findings on CXRs and can be a useful triage tool in acute clinical settings in multi-ethnic South-East Asian cohorts. This ongoing study also demonstrates the utility of algorithmic monitoring of AI model performance through AimSG.
**Limitations:** Small sample size.
**Ethics committee approval:** This study was ethically approved by the Singhealth CIRB Ref: 2021/2337.
**Funding for this study:** No funding was obtained for this study.
**Author Disclosures:**
Charlene Liew: Nothing to disclose
Timothy Shao Ern Tan: Nothing to disclose
Goh Han Leong: Nothing to disclose
Jenny Zou: Nothing to disclose

**09:30-11:00** **Research Stage 2**

### Research Presentation Session: Cardiac

### RPS 2303

### Pre- and periprocedural imaging and new techniques

**Moderator**
F. Wolf; Vienna/AT
*(florian.wolf@meduniwien.ac.at)*

### RPS 2303-2

**Early myocardial extracellular volume at CT imaging: prognostic value in patients undergoing transcatheter aortic valve implantation**

M. L. Lampus[1], *F. Rizzetto*[2], C. B. Monti[2], D. Artioli[2], P. Pedrotti[2], F. Musca[2], A. Vanzulli[2]; [1]Sassari/IT, [2]Milan/IT
*(francesco.rizzetto@unimi.it)*

**Purpose:** The objective of this study was to investigate the prognostic value of early myocardial extracellular volume (eECV) at CT imaging in patients undergoing transcatheter aortic valve implantation (TAVI).
**Methods or Backround:** Consecutive patients who underwent angio-CT examinations for TAVI procedure planning between January 2021 and December 2022 were retrospectively identified. Pre-TAVI CT scans, demographics, and death records, when present, were retrieved for each patient. Region-of-interests in the septal midventricular wall and left ventricular blood pool were delineated on axial images from unenhanced scan and venous phase (acquired 70-90 seconds after contrast injection). Myocardial eECV was calculated as the ratio between myocardium and blood pool differential attenuations, adjusted for patient hematocrit. Myocardial relative enhancement (MRE), computed as the ratio between myocardium and blood pool attenuation at venous phase, and septal wall thickness were also calculated. The correlation between eECV and MRE was assessed with Spearman's rho. Kaplan-Meier analysis and hazard ratios (HR) were used to evaluate the association of eECV and MRE with overall survival to all-cause mortality during a 30-month follow-up period.
**Results or Findings:** The study included 244 patients, with median (1st-3rd quartile) eECV, MRE, and septal wall thickness of 32% (29-36%), 68% (64-73%), and 15 mm (13-17 mm), respectively. A strong positive correlation was observed between eECV and MRE (rho=0.67; $p<0.001$). Patients with eECV≥34% had an increased risk of mortality (HR=2.7, 95% confidence interval: 1.3-5.4, p=0.006), as well as those with MRE≥74% (HR=3.3, 95% confidence interval: 1.4-7.5, p=0.005). No significant difference in demographics and septal wall thickness was observed between high- and low-risk patients.
**Conclusion:** Myocardial eECV and MRE obtained from venous phase CT images are associated with all-cause mortality in patients undergoing TAVI.
**Limitations:** The main study limitation is the retrospective single-centre design.
**Ethics committee approval:** Institutional Review Board approved the retrospective data collection in an anonymous, aggregated form.
**Funding for this study:** No funding was received for this study.
**Author Disclosures:**
Francesco Rizzetto: Other: Bracco Imaging
Diana Artioli: Nothing to disclose
Maria Luisa Lampus: Nothing to disclose
Caterina Beatrice Monti: Other: Bracco Imaging
Angelo Vanzulli: Nothing to disclose
Patrizia Pedrotti: Nothing to disclose
Francesco Musca: Nothing to disclose

### RPS 2303-3

**Interrater variability of ML-based CT-FFR during TAVR-planning: influence of image quality and coronary artery calcifications**

*R. F. Gohmann*, A. Schug, C. Lücke, P. Seitz, M. Gutberlet; Leipzig/DE

**Purpose:** The purpose of this study was to compare machine learning (ML)-based CT-derived fractional flow reserve (CT-FFR) in patients before transcatheter aortic valve replacement (TAVR) by observers with differing training and to assess the influence of image quality and coronary artery calcium score (CAC).
**Methods or Backround:** Patients considered for TAVR have a high prevalence of coronary artery disease (CAD). Coronary computed tomography angiography (cCTA) can effectively exclude CAD, but remains limited by its specificity. CT-FFR may mitigate this limitation also in patients prior to TAVR. While a high reliability of CT-FFR is presumed, little is known about the reproducibility of ML-based CT-FFR.
**Results or Findings:** CT-FFR was successfully performed on 214/272 examinations by both observers. The median difference of CT-FFR was -0.05(-0.12-0.02) (p<0.001). Differences were smaller with high values and larger with low values. Categorization into CAD was different in 37/214 examinations, resulting in a net recategorizations of Δ13 (13/214) examinations and a difference in accuracy of Δ6.1%. Categorization into CAD was independent of quantitative image quality or CAC.
**Conclusion:** CT-FFR values measured by two observers with different experience were different, with smaller differences of high and much larger differences of low values. Categorization into CAD was different in several patients, but only led to a small net difference and ultimately had a moderate influence on diagnostic accuracy, independently of image quality or CAC.
**Limitations:** Retrospective study. Assessment of coronary artery disease (CAD) is recommended prior to transcatheter aortic valve replacement (TAVR). Invasive coronary angiography (ICA) may be omitted, if significant CAD can be excluded on coronary computed tomography angiography (cCTA). Despite its high sensitivity, cCTA is limited by relatively low specificity and positive predictive value, particularly in high-risk patients. CT-derived fractional flow reserve (CT-FFR) is a promising technique with the potential to increase the diagnostic accuracy and specificity of cCTA for ruling-out CAD during pre-TAVR evaluation. However, the significance of the segmentation process and reader experience on the reliability of CT-FFR is not well investigated.
**Ethics committee approval:** The local ethics committee approved the study and written informed consent was waived.
**Funding for this study:** No information was provided for this section by the submitter.
**Author Disclosures:**
Christian Lücke: Nothing to disclose
Matthias Gutberlet: Nothing to disclose
Patrick Seitz: Nothing to disclose
Adrian Schug: Nothing to disclose
Robin F. Gohmann: Nothing to disclose

### RPS 2303-4

**CT fluoroscopy-guided percutaneous biopsy of cardiac and pericardial masses**

*P. Rogalla*, M. Pourafkari, B. Karasfi, S. Kandel, C. O'Brien, R. Cusimano; Toronto, ON/CA

**Purpose:** Multiple routes exist for obtaining tissue samples from cardio-pericardial masses (CPM), including percutaneous transvenous catheter biopsy techniques, minimally invasive methods such as mediastinoscopy and open surgical approaches. This study aims to assess the feasibility, success rate, and complications of CT-guided biopsy of CPM.
**Methods or Backround:** Institutional Research Ethics Board approval was obtained. Twenty-four biopsies of cardio-pericardial masses and pericardial thickening were performed in 23 patients under CT-fluoroscopy guidance with or without EKG gating on a 320-slice scanner (Canon Medical Systems) under conscious sedation. 3D mapping of the coronary arteries prior to the intervention, mediastinal widening and capnothorax techniques were applied when appropriate. The clinical presentation, details of the procedure, the success rate of obtaining diagnostic tissue, and potential complications and mitigation strategies, and in-room time were recorded.
**Results or Findings:** Twenty-three patients (12 women, 11 men; mean age, 52.3 years) were included in this study. The CT fluoroscopy-guided procedure was successful in obtaining diagnostic tissue samples in all patients. The target lesions ranged from 1 cm of pericardial thickening to a 15 cm intrapericardial mass. Histology revealed 12 malignant and 12 benign diagnoses. One patient developed a small hemothorax, and another patient developed a small pneumomediastinum immediately post-procedure, both of which were successfully treated conservatively. Post-procedure hemopericardium or arrhythmia were not observed in any of the patients. One patient with encasing sarcomatoid mesothelioma passed away from cardiac arrest in the ICU two hours after the procedure. The mean in-room time was 34.2 min.
**Conclusion:** CT-guided percutaneous biopsy of cardio-pericardial masses is a safe procedure that provides an accurate diagnosis and may be considered when a histological diagnosis is crucial for clinical management decisions.
**Limitations:** Single-centre evaluation, no alternative sampling methods tested in comparison.
**Ethics committee approval:** Institutional Research Ethics Board approval was obtained for this study.
**Funding for this study:** No funding was obtained for this study.
**Author Disclosures:**
Robert Cusimano: Nothing to disclose
Marina Pourafkari: Nothing to disclose
Behruz Karasfi: Nothing to disclose
Sonja Kandel: Nothing to disclose
Patrik Rogalla: Nothing to disclose
Ciara O'Brien: Nothing to disclose

### RPS 2303-5

**Diagnostic accuracy of detecting significant mitral valve regurgitation on non-contrast thoracic CT**

*B. M. Suwita*[1], R. Khattar[2], K. Vakalis[1], W. Banya[2], T. Mittal[1], A. Baltabaeva[1], S. Mirsadraee[2]; [1]Harefield/UK, [2]London/UK
*(benedicta.mutiara.s@gmail.com)*

**Purpose:** Previous studies suggested that mitral regurgitation (MR) is an under-diagnosed disease. Earlier detection results in appropriate management and a better prognosis. Non-contrast thoracic CT for non-cardiovascular indications may be used for mitral annulus measurement. This study aimed to assess CT markers of MR on non-contrast thoracic CT and to determine cut-off values for detecting moderate/severe MR on this modality.
**Methods or Backround:** This is a retrospective study of adult subjects who underwent non-contrast thoracic CT and echocardiography between 2013 and 2023. Exclusion criteria were poor image quality, prior intervention on mitral/aortic valve, and >12 months period between CT and echocardiography. Using double-oblique multiplanar projects, 4 parameters were measured on the thoracic CT: mitral valve anterior-posterior (MVAP), mitral valve septal-lateral (MVSL), left ventricle anterior-posterior (LVAP) and left ventricle apical-basal diameters. The left ventricle sphericity index (LVSI) was measured by dividing LVAP and LV apical-basal. Subjects were divided into “normal”, “mild MR”, “moderate MR”, and “severe MR” categories based on echocardiography.
**Results or Findings:** 220 subjects were included (120 normal, 44 mild, 27 moderate, 29 severe). Kruskal-Wallis test and Bonferroni post-hoc analysis showed no statistically significant difference between the normal and mild MR. However, there is a significant difference between normal and moderate/severe MR groups in all CT parameters. Cut-off points to detect moderate/severe MR were: MVAP 41.5 mm, MVSL 37.5 mm, LVAP 72.5 mm, LV apical-basal 87.5 mm, and LVSI 0.83. The accuracy (with 95% confidence interval) for each cut-off point was: 84% (78–89%), 82% (76–87%), 78% (72–84%), 63% (55–70%) and 72% (66–79%).
**Conclusion:** Simple measurements on non-contrast thoracic CT can predict the presence of moderate/severe MR with good diagnostic accuracy. These results are important for early detection of undiagnosed significant MR.
**Limitations:** The limitations of the study are no sub-group analysis for different MR causes.
**Ethics committee approval:** The study is retrospective.
**Funding for this study:** No funding was provided for this study
**Author Disclosures:**
Saeed Mirsadraee: Nothing to disclose
Benedicta Mutiara Suwita: Nothing to disclose
Tarun Mittal: Nothing to disclose
Aigul Baltabaeva: Nothing to disclose
Winston Banya: Nothing to disclose
Konstantinos Vakalis: Nothing to disclose
Rajdeep Khattar: Nothing to disclose

### RPS 2303-6

**Impact of postprocedural CT evaluation of lead to valve angle on the development of cardiac implantable electrical devices related tricuspid regurgitation**

A. Caracciolo, *V. Scialò*, C. Lisi, F. Catapano, L. Monti, M. Francone; Milan/IT

**Purpose:** Research shows that direct injury from CIED leads is related to the development of tricuspid regurgitation (TR), with a prevalence of up to 40% of patients. This study aimed to explore the role of CT in identifying predictors of the development or worsening of TR secondary to CIED.
**Methods or Backround:** 374 patients who underwent CIED implantation between January 2014 and December 2021 and had at least one chest CT after implantation and repeated transthoracic echocardiograms (TTE) were selected. The angle between the tricuspid valve plane and the lead was measured in a two-chamber view CT scan. Worsening TR was defined as an increased TR severity compared to baseline while worsening right ventricular (RV) function was defined as the new occurrence of RV dysfunction, as indicated by a tricuspid annular plane excursion (TAPSE) <18 mm. Univariate

Sunday and multivariate regression analyses were performed to identify the predictors of development or TR worsening.
**Results or Findings:** After implantation, 169 individuals (45.18%) had new-onset or worsening TR and 60 (16.04%) had a worse RV function. Regression analysis reported that statistically significant predictors of development or worsening of TR were a lead-valve angle >90° (HR 2.03, CI 1.09-3.76; p 0.025) and worsening right ventricular function (HR 2.22, CI 1.26-3.91; p 0.006); other strongly related variables, although without a statistical significance were moderate-to-severe mitral regurgitation (HR 0.74, CI 0.47 - 1.16; p 0.184), baseline RV dysfunction (HR 1.36; CI 0.83-2.24; p 0.2).
**Conclusion:** Our study demonstrates that lead positioning has a significant impact on patient outcomes, with a two-fold increased risk of worsening TR when the lead-valve angle exceeds 90°. Postimplantation CT scans may help identify patients at risk of developing TR due to suboptimal lead positioning.
**Limitations:** No limitations were identified.
**Ethics committee approval:** Not applicable because the study is retrospective.
**Funding for this study:** No funding was provided for this study.
**Author Disclosures:**
Lorenzo Monti: Nothing to disclose
Costanza Lisi: Nothing to disclose
Alessandra Caracciolo: Nothing to disclose
Vincenzo Scialò: Nothing to disclose
Federica Catapano: Nothing to disclose
Marco Francone: Nothing to disclose

### RPS 2303-7

**Myocardial tissue characterisation with late contrast enhancement (LCE-CT) in patients presenting with acute symptoms and mild troponin rise**

*D. Vignale*[1], A. Palmisano[1], M. Gatti[2], R. Faletti[2], A. Esposito[1]; [1]Milan/IT, [2]Turin/IT
*(vignale.davide@hsr.it)*

**Purpose:** This study aimed to investigate the added diagnostic value of myocardial tissue characterisation with Late Contrast Enhancement (LCE) scan in patients with acute symptoms, slight troponin increases but no acute myocardial infarction diagnosis.
**Methods or Backround:** A total of 144 patients (M: F=80:64; median age=67, IQR 50-77) with elevated troponin but no ECG or lab signs of acute myocardial infarction were enrolled. Initial CT angiography (CTA) screened for coronary artery disease (CAD), pulmonary embolism, and thoracic aortic conditions. Those with negative CTA results underwent a low kVp LCE scan 7 min after an iodinated contrast injection to detect myocardial damage. A subset of patients with obstructive CAD underwent LCE too. Diagnoses were mostly confirmed through gold-standard methods.
**Results or Findings:** Median troponin was 69 ng/L (IQR 39-200; normal <14 ng/L). Out of 144 patients, 64 (44%) had CAD, two (1%) aortic syndrome, and eight (6%) pulmonary embolism. LCE was performed in the remaining 70 (49%) with negative CTA, diagnosing myocarditis in 35/70 (50%), Takotsubo in 8/70 (11%), MINOCA in 4/70 (6%), dilated cardiomyopathy in 4/70 (6%), and amyloidosis in 3/70 (4%). For 16/70 (23%), LCE was normal. Among the 27 with CAD also imaged with LCE, 12 (44%) had no LCE, four (15%) had subendocardial LCE, and 11 (41%) had transmural LCE, with 3/11 (27%) showing microvascular obstruction.
**Conclusion:** In patients with acute symptoms and elevated troponin but negative CTA, supplementary LCE improved diagnostic accuracy from 51% to 89% (p<0.001), mainly identifying myocarditis as the aetiology. In patients with CAD, LCE revealed different extents of myocardial damage, offering valuable insights into the severity of acute coronary syndromes.
**Limitations:** This study had a relatively small sample size and not all patients underwent CMR. There was also a lack of follow-up data on LCE capabilities of risk stratification.
**Ethics committee approval:** This study was approved by protocol CT-MyoC.
**Funding for this study:** No funding was received for this study.
**Author Disclosures:**
Davide Vignale: Nothing to disclose
Marco Gatti: Nothing to disclose
Antonio Esposito: Nothing to disclose
Riccardo Faletti: Nothing to disclose
Anna Palmisano: Nothing to disclose

### RPS 2303-8

**Feasibility of T2 mapping for the detection of myocardial injury in hypertrophic cardiomyopathy**

S. Yang, S. Zhao, *J. Wang*; Beijing/CN
*(watermelonwjx@163.com)*

**Purpose:** This study aimed to explore the association between T2 value and myocardial injury in patients with hypertrophic cardiomyopathy (HCM).
**Methods or Backround:** All patients underwent laboratory testing and CMR. Hs-cTnI was obtained as a marker of myocardial injury (>0.016 ng/mL). Global T2 values were quantified for three LV short-axis slices. The maximum T2 value and ConSept T2 were measured from the maximal value of 16 segments and the middle septum, respectively.
**Results or Findings:** In the HCM group, elevated hs-cTn I was found in 25 (20 males, 50±13 years) and normal hs-cTn I in 25 (17 males, 49±14 years). The hs-cTnI elevated group had higher T2 global, ConSept T2, T2 max, T1 global, ConSept T1 values, and LGE extent (P<0.05) than the normal hs-cTnI group. The ConSept T2 value (r=0.52, P<0.001) and LGE extent (r=0.52, P<0.001) were all moderately correlated with hs-cTnI. Among all parametric parameters, the ConSept T2 value showed the best performance in identifying myocardial injury (AUC=0.83). In multivariate logistic regression analyses, after adjusting LV ejection fraction, LV end-diastolic volume index, maximal ventricular wall thickness, LV myocardial mass index, LGE extent, T1 global, and ConSept T1 values, respectively, ConSept T2 value was still significantly associated with elevated hs-cTnI (P<0.01).
**Conclusion:** We confirmed the feasibility of T2 mapping for detecting myocardial injury in HCM. T2 value was associated with an elevated hs-cTnI, which provided in-vivo evidence by CMR for the ongoing myocardial injury in HCM.
**Limitations:** The study was conducted at a tertiary referral centre with a small sample size. Since the mapping values depend on the sequence and vendor, the results observed in our study might not be generalised to the other centres.
**Ethics committee approval:** The study complied with the Declaration of Helsinki and was approved by the institutional review boards of Fuwai Hospital.
**Funding for this study:** This study was supported by grants from the National Key Research and Development Program of China (No. 2021YFF0501404, No. 2021YFF0501400) and the key projects of the National Natural Science Foundation of China (No. 81930044).
**Author Disclosures:**
Shihua Zhao: Nothing to disclose
Jiaxin Wang: Nothing to disclose
Shujuan Yang: Nothing to disclose

### RPS 2303-9

**Fractal analysis on CMR: trabecular complexity as a new marker to predict outcomes in patients with dilated cardiomyopathy**

*X. Xiang*, S. Yu, K. Yang, C. Cui, X. Chen, M. Lu, S. Zhao; Beijing/CN

**Purpose:** Trabecular complexity is a unique biometric marker like a fingerprint, but its prognostic impact in dilated cardiomyopathy (DCM) remains unclear. This study investigated the prognostic value of trabecular complexity by fractal analysis in patients with DCM.
**Methods or Backround:** Consecutive 276 patients with DCM were enrolled in this study. Comprehensive clinical evaluation and cardiovascular magnetic resonance (CMR) imaging investigation were obtained. Trabecular complexity was quantified with fractal analysis of cine images to estimate the global, basal, and apical fractal dimensions (FD). All patients were followed up for major adverse cardiac events (MACE) of all-cause mortality, aborted sudden cardiac death, and heart transplantation.
**Results or Findings:** Over a 5.37-year median follow-up, 103 (37.32%) patients experienced MACE. All left ventricular FD parameters were higher in patients with events than those without events (all P<0.05). Max Basal FD emerged as the strongest MACE prognosticator among FD parameters (AUC: 0.84 [95% CI, 0.78–0.88]), and the optimal cutoff value was 1.27. Furthermore, Cox hazards analysis revealed that Max Basal FD was independently associated with MACE (HR: 1.07 per %, p=0.002) after adjustment for clinical and imaging risk factors including NT-proBNP, LVEF, and LGE presence. By Kaplan-Meier analysis, the risk of MACE increased significantly with increased Global FD, Mean Basal FD, and Max Basal FD (all log-rank p<0.001).
**Conclusion:** LV max basal FD was an independent predictor of adverse outcomes, and fractal analysis may contribute to improving the risk stratification for patients with DCM.
**Limitations:** This was a single-centre study with no genetic characterization of the subjects. Despite the limitations, it is the first study to apply Fractal Analysis to predict outcomes in DCM, demonstrating that myocardial trabecular complexity as a new marker has the potential to improve DCM risk stratification algorithms.
**Ethics committee approval:** This study was approved by the hospital's Institutional Review Board (IRB).
**Funding for this study:** This study was funded by the National Key Research and Development Program of China (No. 2021YFF0501400) and the Key Project of the National Natural Science Foundation of China (No. 81930044).
**Author Disclosures:**
Minjie Lu: Nothing to disclose
Kai Yang: Nothing to disclose
Shiqin Yu: Nothing to disclose
Xiuyu Chen: Nothing to disclose
Xiaorui Xiang: Nothing to disclose
Shihua Zhao: Nothing to disclose
Chen Cui: Nothing to disclose

**RPS 2303-10**

**Computed tomography angiography reconstructed with MBIR for TAVR planning as a comprehensive tool for the assessment of coronary arteries**

*C. R. G. L. O. M. Talei Franzesi*, C. Maino, D. G. Gandola, M. Ragusi, T. P. Giandola, P. N. Franco, R. Corso, D. Ippolito; Milan/IT
*(ctfdoc@hotmail.com)*

**Purpose:** This study aimed to evaluate the diagnostic performance of a model-based iterative algorithm (MBIR) in the assessment of coronary arteries in an angiographic-CT (CTA) study during TAVR planning.
**Methods or Backround:** A total of 82 patients who underwent CTA for TAVR planning were included in this study. All exams were obtained using a 256-slice MDCT scanner with low-dose and low-contrast media volume CTA protocol (80 kV, 50 mL) with ECG-gated technique and reconstructed with MBIR algorithm. All coronary arteries were divided into 3 segments and evaluated in terms of image quality and contrast enhancement. Mean attenuation values (HU) of coronary arteries and thoracic aorta were calculated by drawing a manual region of interest (ROI) in the lumen of vessels.
**Results or Findings:** The mean attenuation value in the thoracic aorta was 478 HU and in the abdominal aorta was 447 HU. The proximal segment of the left anterior descending artery was identified and analysed in all (100%) cases, while the medium segment was identified in 89% and the distal tract only in 65%. The proximal segment of the left circumflex artery was recognisable in 89%, the medium segment in 70%, and only in 29% it was possible to assess the distal segment. In 95% of CT scans, we were able to evaluate the proximal segment of the right coronary artery, in 79% of the medium and 52% of the distal segment.
**Conclusion:** Low-dose and low-contrast media volume CTA performed for TAVR planning offers important insight information on coronary status when combined with the IMR reconstruction algorithm.
**Limitations:** This was a retrospective and a single-centre study.
**Ethics committee approval:** This was a retrospective study.
**Funding for this study:** No funding was received for this study.
**Author Disclosures:**
Davide Giacomo Gandola: Nothing to disclose
Cesare Maino: Nothing to disclose
Maria Ragusi: Nothing to disclose
Cammillo Roberto Giovanni Leopoldo Oreste Massimiliano Talei Franzesi: Nothing to disclose
Rocco Corso: Nothing to disclose
Teresa Paola Giandola: Nothing to disclose
Paolo Niccolò Franco: Nothing to disclose
Davide Ippolito: Nothing to disclose

**09:30-11:00 Research Stage 3**

### Research Presentation Session: Oncologic Imaging

### RPS 2316

### Innovation in cancer research

**Moderator**
D. M. Lambregts; Amsterdam/NL

**RPS 2316-2**

**Clinical added value of MRI to CT in patients scheduled for local therapy of colorectal liver metastases (CAMINO): an international multicentre prospective diagnostic accuracy trial**

*B. Görgec*[1], I. Schrøder-Hansen[2], G. Kemmerich[2], T. Syversveen[2], J. P. Sijberden[1], Å. Fretland[2], C. Verhoef[3], M. G. Besselink[1], J. Stoker[1]; [1]Amsterdam/NL, [2]Oslo/NO, [3]Rotterdam/NL
*(b.gorgec@amsterdamumc.nl)*

**Purpose:** Guidelines are inconclusive on whether contrast enhanced magnetic resonance imaging(ceMRI) should be added routinely to computed tomography(ceCT) in the workup of patients with colorectal liver metastases(CRLM) scheduled for curative liver resection and/or thermal ablation. Although ceMRI is reportedly superior in the detection and characterization of CRLM, its impact on actual clinical patient management is unknown and through this project, we aim to understand that.
**Methods or Backround:** This was an international multicentre prospective incremental diagnostic accuracy trial in patients with primary or recurrent CRLM scheduled for local therapy based on ceCT. All patients had ceCT and liver ceMRI including gadoxetic acid as contrast agent and diffusion-weighted imaging(DWI). Primary outcome was change in the local clinical treatment plan based on liver ceMRI findings. A multidisciplinary blinded expert panel performed a post-hoc analysis.
**Results or Findings:** Between December 2019, and July 2021, we enrolled 298 patients with CRLM planned for local therapy based on ceCT. A change in the local clinical treatment plan based on liver ceMRI findings was observed in 92/298 patients(31%;95% CI: 26% to 36%). This concerned 40 patients(13%) requiring more extensive local therapy, 11 patients(3.7%) requiring less extensive local therapy, and 34 patients(11.4%) in whom the indication for curative-intent local therapy was revoked, including 26 patients(8.7%) with too extensive disease and 8 patients(2.7%) with benign lesions on liver ceMRI(confirmed by follow-up). Upon assessment by the expert panel, liver ceMRI changed clinical management in 101/297 patients(34%;95% CI: 29% to 40%).
**Conclusion:** Liver ceMRI changed the local treatment plan in one third of patients scheduled for intervention for CRLM based on ceCT imaging.
**Limitations:** First, primary outcome change in local treatment plan could not be blinded for physicians. Second, ceCT- and ceMRI-scan protocols were not completely standardized between participating centres.
**Ethics committee approval:** The Medical Ethical Review Board (METc) of the Amsterdam UMC, location AMC, has assessed primarily that the CAMINO study was NOT subject to the Medical Research Involving Human Subjects Act (WMO).
**Funding for this study:** This study was funded by the The Dutch Cancer Society and Bayer AG – Pharmaceuticals.
**Author Disclosures:**
Jasper Paul Sijberden: Nothing to disclose
Trygve Syversveen: Nothing to disclose
Jaap Stoker: Grant Recipient: Bayer AG – Pharmaceuticals; Grant Recipient: The Dutch Cancer Society
Åsmund Fretland: Nothing to disclose
Gunter Kemmerich: Nothing to disclose
Ingrid Schrøder-Hansen: Nothing to disclose
Cornelis Verhoef: Grant Recipient: Bayer AG – Pharmaceuticals; Grant Recipient: The Dutch Cancer Society
Marc G. Besselink: Grant Recipient: The Dutch Cancer Society; Grant Recipient: Bayer AG – Pharmaceuticals
Burak Görgec: Grant Recipient: Bayer AG – Pharmaceuticals; Grant Recipient: The Dutch Cancer Society

**RPS 2316-3**

**Diagnostic value of very early diffusion weighted changes at MRI after single-dose ablative radiation therapy (SDART) for organ-confined prostate cancer**

*C. R. G. L. O. M. Talei Franzesi*, D. G. Gandola, P. N. Franco, C. Maino, D. Panizza, R. Lucchini, S. Arcangeli, R. Corso, D. Ippolito; Milan/IT
*(ctfdoc@hotmail.com)*

**Purpose:** The objective of this study was to investigate diagnostic value of diffusion-weighted MRI early changes, an hour after treatment, in patients with organ confined unfavorable prostate cancer(PCa) treated with single-dose ablative radiation therapy(SDART), in comparison with biochemical markers(PSA and testosterone).
**Methods or Backround:** Twenty-four patients treated with a single fraction of 24Gy to the whole prostate with urethra sparing in association with androgen deprivation therapy(ADT) were enrolled. MRI was performed before SDART(time0), one-hour post-SDART(time1), and 3-month after treatment(time2). All the patients were examined on a 3.0-T-MRI(Ingenia;Philips Healthcare) with a phased-array external coil, with bowel preparations and 20 mg of butyl-scopolamine(Buscopan); the bladder was filled with 120 cc of saline solution, to simulate the same conditions during irradiation. MRI was performed with axial T1-weighted TSE and high resolution multi-planar T2-weighted TSE sequences. DWI was acquired with six b-values (0,50,100,150,800,1600mm2/s) and Apparent Diffusion Coefficient(ADC) (0,800) maps were calculated. Finally, axial contrast-enhanced dynamic imaging was obtained during intravenous injection of gadobutrol. ADC values were calculated at time 0,1,and 2 by placing region-of-interests(ROI) on ADC maps and the results were compared with PSA and testosterone blood levels at time 0 and 2.
**Results or Findings:** Median patient's age was 78 years. Median prostate volume was 36.2 cc. An increase of ADC value of tumour lesion of 27%(range 7%-69%) and 54%(range 20%-83%) was registered at time 1 and time 2 respectively, compared to the baseline. Median prostate volume was found unchanged at time 1, while decreased by about 25%(range 9%-59%) at time 2. At 3-months follow-up,all patients were found bNED with PSA and testosterone levels of<0.01 ng/ml and<0.20 ng/ml, respectively, and nine of them obtained a complete response.

**Conclusion:** Our findings demonstrated high diagnostic value of DWI imaging with good correlation between very early changes (one-hour after treatment) in ADC values after SDART and later tumour response (biochemical and imaging) in patients with unfavorable PCa.
**Limitations:** One limitation was that this was a single-centre study.
**Ethics committee approval:** This study utilised retrospective anonymous data analysis.
**Funding for this study:** No funding was received for this study.
**Author Disclosures:**
Raffaella Lucchini: Nothing to disclose
Denis Panizza: Nothing to disclose
Davide Giacomo Gandola: Nothing to disclose
Cesare Maino: Nothing to disclose
Cammillo Roberto Giovanni Leopoldo Oreste Massimiliano Talei Franzesi: Nothing to disclose
Rocco Corso: Nothing to disclose
Stefano Arcangeli: Nothing to disclose
Paolo Niccolò Franco: Nothing to disclose
Davide Ippolito: Nothing to disclose

### RPS 2316-4

**Comparison between MRI and conventional CT scans in the prediction of peritoneal cancer index**

*M. Polici*, B. Masci, F. Palmeri, P. Sammartino, A. Del Gaudio, M. Zerunian, D. De Santis, D. Caruso, A. Laghi; Rome/IT
*(policimichela28@gmail.com)*

**Purpose:** Peritoneal cancer index (PCI) has been recognised as an independent prognostic indicator for long-term outcomes, however the preoperative assessment represents a clinical challenge. So, the aim of the study was to compare the performance of MRI to CT having the surgery as reference standard.
**Methods or Backround:** Twenty-three patients affected by peritoneal carcinomatosis with availability of pre-operative MRI and CT scans of abdomen and pelvis, were prospectively enrolled, between July 2021 and May 2023. All CT and MRI scans were performed in the same day, within one week from the surgery. Two expert abdominal radiologists blinded, with two different experience levels (expert and inexperienced), evaluated PCI in both MRI and CT scans. The agreements between the radiologists' assessment with surgical results, sensitivity, and specificity were evaluated in total and per site.
**Results or Findings:** In total PCI, for the expert radiologist the assessment was quite better for MRI (kappa=0.89) than CT (kappa = 0.73). Similarly, for the inexperienced radiologist MRI was better (kappa = 0.79) than CT (kappa = 0.69). In the sub analysis of per site, both expert and inexperienced radiologists showed higher agreement for MRI (kappa = 0.83 and 0.75, respectively) than for CT (kappa=0.68 and 0.63, respectively). Overall, MRI showed higher value of per-site sensitivity and specificity compared to CT (0.95 vs 0.65 and 0.79 vs 0.75, respectively).
**Conclusion:** MRI shows better results in terms of PCI assessment performance compared to CT, with quite higher agreement for the expert radiologist.
**Limitations:** This study was performed on a small population; it was of retrospective nature; it lacks survival analysis.
**Ethics committee approval:** Written informed consent was acquired for all patients and Institutional Review Board approval was obtained
**Funding for this study:** No funding was received for this study.
**Author Disclosures:**
Damiano Caruso: Nothing to disclose
Benedetta Masci: Nothing to disclose
Marta Zerunian: Nothing to disclose
Domenico De Santis: Nothing to disclose
Federica Palmeri: Nothing to disclose
Paolo Sammartino: Nothing to disclose
Andrea Laghi: Nothing to disclose
Antonella Del Gaudio: Nothing to disclose
Michela Polici: Nothing to disclose

### RPS 2316-5

**CT morphological phenotype of a tumour is influenced more by the host tissue environment than tumour type**

*S. Rostami*, C. Guerendel, S. H. Benson, Z. Bodalal, R. G. H. Beets-Tan; Amsterdam/NL

**Purpose:** Tumour morphology is influenced by numerous genetic and environmental factors. Tumour type and origin, oncogenic mutational processes, and host tissue (micro)environment all impact how a tumour grows. In this study, we aimed to assess the effects of the tumour type and the host tissue (micro)environment on tumour morphology, using radiomics and unsupervised clustering techniques.
**Methods or Backround:** Manually segmented baseline CT scans of 1659 cancer patients of various tumour types were used to extract the radiomic features of 11,164 lesions. TSNE, an unsupervised clustering approach, was used to derive the components that encapsulated the entire tumour morphology, as measured by radiomics. We visualised all the lesions on a TSNE plot to explore the presence of morphological clustering. To assess the association between the observed morphological clusters and our endpoints, we applied the labels of tumour type and host tissue (micro)environment (e.g., liver, lung, bone, lymphatics, etc.) to the plot.
**Results or Findings:** On the basis of morphology, TSNE identified several distinct clusters. Tumour type failed to explain the clustering pattern observed, when all the lesions (primary and metastases) were included. Looking at only primary lesions, lung cancer formed a distinct morphological cluster apart from non-lung cancers. We observed that the morphological clusters identified could be largely explained by the host tissue (micro)environment.
**Conclusion:** In this study, we utilised routinely available clinical imaging data to explore the biological influence on tumour morphology. Considering that tumour morphology is a multifactorial attribute, the host tissue (micro)environment might substantially affect how a tumour appears, more than its very own origin.
**Limitations:** External validation of the results is not done yet, limiting this study.
**Ethics committee approval:** This study was ethically approved by the IRB Code: IRBd20-213.
**Funding for this study:** No funding was received for this study.
**Author Disclosures:**
Zuhir Bodalal: Nothing to disclose
Corentin Guerendel: Nothing to disclose
Sajjad Rostami: Nothing to disclose
Sean Harry Benson: Nothing to disclose
Regina G. H. Beets-Tan: Nothing to disclose

### RPS 2316-6

**Patient eligibility for trials with imaging response assessment at the time of molecular tumour board presentation**

*N. Mansour*, B. Westphalen, M. Bergwelt-Baildon, W. G. Kunz; Munich/DE
*(nabeel.mansour@med.uni-muenchen.de)*

**Purpose:** The goal of this study was to assess the eligibility of patients with advanced or recurrent solid malignancies presented in a molecular tumour board (MTB) at a large precision oncology centre for inclusion in trials with the endpoints objective response rate (ORR) or duration of response (DOR) based on Response Evaluation Criteria in Solid Tumours (RECIST 1.1).
**Methods or Backround:** Prospective patients with available imaging at the time of presentation in the MTB were included. Imaging data was reviewed for objectifiable measurable disease (MD) according to RECIST version 1.1. Additionally, we evaluated the patients with MD for representativeness of the identified measurable lesion(s) in relation to the overall tumour burden.
**Results or Findings:** Two hundred and sixty-two patients with different solid malignancies were included. 177 patients (68%) had MD and 85 (32%) had non-measurable disease (NMD) at the timepoint of MTB presentation in accordance with RECIST 1.1. MD was not representative of the overall tumour burden in eleven patients (6%). The main reasons for NMD were lesions with longest diameter shorter than 10 mm (22%) and non-measurable peritoneal carcinomatosis (18%). Colorectal cancer and malignant melanoma displayed the highest rates of MD (>75%). In contrast, gastric cancer, head and neck malignancies, and ovarian carcinoma had the lowest rates of MD (<55%).
**Conclusion:** Approximately a third of cancer patients with advanced solid malignancies are not eligible for treatment response assessment in trials with endpoints ORR or DOR at the time of MTB presentation. The rate of patients eligible for trials with imaging endpoints differs significantly based on the underlying malignancy and should be taken under consideration in the planning stages of new basket trials.
**Limitations:** The data is limited as a single-center study with a limited sample size, hence the representation of tumour entities may differ in larger cohorts.
**Ethics committee approval:** All procedures performed in studies involving human participants were in accordance with the ethical standards of the institutional research committee and with the 1964 Helsinki Declaration and its later amendments or comparable ethical standards. The study was approved by the institutional review board. Informed consent was waived due to the retrospective character of the study.
**Funding for this study:** This study was not supported by any funding.
**Author Disclosures:**
Michael Bergwelt-Baildon: Nothing to disclose
Benedikt Westphalen: Nothing to disclose
Wolfgang Gerhard Kunz: Nothing to disclose
Nabeel Mansour: Nothing to disclose

### RPS 2316-7

**Iodine maps concentration and LI-RADS classification: to a quantitative evaluation of HCC**

*A. Barbaro*, P. A. Bonaffini, A. Celestino, G. Muscogiuri, P. Marra, S. Sironi; 24127 Bergamo/IT
*(a.barbaro1@campus.unimib.it)*

**Purpose:** The LI-RADS classification is universally recognized as a valid instrument for evaluating hepatocellular carcinoma (HCC), but it is a qualitative evaluation. This study aims to investigate the possibility of having a quantitative assessment of HCC, by using the Iodine maps of dual-energy CT (DECT).
**Methods or Backround:** This retrospective study included 75 patients with HCC who underwent DECT between March 2022 and April 2023. Some of them underwent more than one DECT in that period, for a total of 90 exams. We included all those lesions classified as LI-RADS 5 and all those classified as LI-RADS 3 and 4 that were subsequently confirmed as HCC by other exams such as MRI or CEUS. We measured the Iodine Concentration in the arterial phase (ICa), in the venous phase (ICv) and normalized them for iodine concentration in a non-pathologic part of liver parenchyma (NICa and NICv). We calculated the ratio between NICa and NICv, and performed a Kruskal-Willis test.
**Results or Findings:** The median of ICa was 2.95 mg/ml for LI-RADS 3, 3.40 mg/ml for LI-RADS 4 and 3.30 mg/ml for LI-RADS 5. The median of ICv was 2.55 mg/ml for LI-RADS 3, 2.90 mg/ml for LI-RADS 4, and 2.40 mg/ml for LI-RADS 5. The median of the NICa/NICv ratio was 1.793 for LI-RADS 3, 3.495 for LI-RADS 4, and 2.611 for LI-RADS 5 (P = 0,000429), demonstrating that the NICa/NICv ratio is significantly lower in the LI-RADS 3 sample.
**Conclusion:** Quantitative assessment of wash-in/was-out for HCC nodules on iodine concentration maps and, particularly, the NICa/NICv ratio may increase radiologists' confidence in LI-RADS class allocation.
**Limitations:** The main limitations are the small sample size and the selection bias due to the retrospective design of this study.
**Ethics committee approval:** No ethical approval was sought for this study.
**Funding for this study:** No funding was received for this study.
**Author Disclosures:**
Antonio Celestino: Nothing to disclose
Sandro Sironi: Nothing to disclose
Paolo Marra: Nothing to disclose
Pietro Andrea Bonaffini: Nothing to disclose
Alessandro Barbaro: Nothing to disclose
Giuseppe Muscogiuri: Nothing to disclose

### RPS 2316-8

**Stage IIIN2 NSCLC undergoing induction chemotherapy and subsequent surgery: preliminary results of CT morphologic prognostic factors**

*N. Di Meglio*, A. Perrella, G. Bagnacci, V. Di Martino, L. Volterrani, L. Luzzi, M. A. Mazzei; Siena/IT
*(dimeglionunzia@gmail.com)*

**Purpose:** Patients diagnosed with stage IIIN2 Non-Small Cell Lung Carcinoma (NSCLC) have various treatment options, including surgery following induction chemotherapy (CHT) or concurrent chemoradiotherapy followed by immunotherapy. This study aimed to identify radiological prognostic factors in a cohort of stage IIIN2-NSCLC patients who received induction CHT and subsequent surgery.
**Methods or Backround:** Among 93 patients with locally advanced NSCLC who underwent surgical resection following induction CHT between 2013 and 2020, we retrospectively selected 72 patients. We assessed histological parameters (pTNM stage, pathological regression grade, mutational profile) and radiological features (tumour size, margin characteristics, location, presence of necrosis or carcinomatous lymphangitis for T-stage, and lymph node size, location, structure, and enhancement pattern for N-stage) from staging and restaging CT scans. We correlated these parameters with overall survival (OS) to determine their prognostic significance. We also evaluated the performance of different radiological analysis methods
**Results or Findings:** The mean OS was 65.54±6.170 months, with disease recurrence occurring in 32 out of 72 cases (44.5%). Among clinical, histological, and radiological factors, a significant correlation with OS was observed only for the presence of the KRAS mutation (p<0.001), regression of clinical nodal stage after CHT (cN2 to ypN0/1; p 0.02), and nodal stage regression based on multiparametric criteria (p 0.001). Multiparametric criteria exhibited high sensitivity (95%), specificity (85%), positive predictive value (PPV) (91%), negative predictive value (NPV) (92%), and an overall accuracy of 91%.
**Conclusion:** Nodal downstaging after induction CHT result to be a good prognostic factor. Radiological nodal involvement could be established with high accuracy by the multiparametric criteria at CT examination.
**Limitations:** The main limitation of this study is that it is of retrospective design.
**Ethics committee approval:** No ethical approval was sought for this study.
**Funding for this study:** No funding was obtained for this study.
**Author Disclosures:**
Maria Antonietta Mazzei: Nothing to disclose
Vito Di Martino: Nothing to disclose
Luca Luzzi: Nothing to disclose
Armando Perrella: Nothing to disclose
Nunzia Di Meglio: Nothing to disclose
Luca Volterrani: Nothing to disclose
Giulio Bagnacci: Nothing to disclose

### RPS 2316-9

**Dynamic contrast-enhanced ultrasound analysis in preoperative diagnosis between hepatocellular carcinoma and intrahepatic cholangiocarcinoma in non-cirrhotic liver**

*Y. Dong*, S. Chen, Y. Huang, J. Chen, Y. Wang, W-P. Wang; Shanghai/CN
*(dr_mimi@163.com)*

**Purpose:** The purpose of this study was to investigate the value of dynamic contrast-enhanced ultrasound (DCE-US) and quantitative analysis in preoperative differential diagnosis of intrahepatic cholangiocarcinoma (ICC) and hepatocellular carcinoma (HCC) in non-cirrhotic liver.
**Methods or Backround:** In this retrospective study, patients with histopathologically proven ICC and HCC lesions in non-cirrhotic liver were included. All patients underwent contrast enhanced ultrasound (CEUS) examinations within one week before surgery. B mode ultrasound (BMUS) features and CEUS enhancement patterns were analysed. DCE-US analysis was performed by VueBox® software (Bracco, Italy). Time intensity curves (TICs) were generated, and quantitative perfusion parameters were obtained and compared between ICC and HCC groups using the Student t test or Mann-Whitney U test.
**Results or Findings:** From November 2020 to February 2022, patients with histopathologically confirmed ICC (n = 30) and HCC (n = 24) lesions in non-cirrhotic liver were included. Compared with HCC lesions, TICs of ICCs showed earlier and lower enhancement during the AP, faster decline during the PVP, and reduced area under the curve (AUC). The combined area under the receiver operating characteristic curve (AUROC) of all significant parameters was 0.946, with 86.7 % sensitivity, 95.8 % specificity, and 90.7 % accuracy in differential diagnosis between ICC and HCC lesions in non-cirrhotic liver, which improved the diagnostic efficacy of CEUS (58.3 % sensitivity, 90.0 % specificity, and 75.9 % accuracy).
**Conclusion:** ICC and HCC lesions in non-cirrhotic liver might show some overlap of CEUS features in diagnosis. DCE-US with quantitative analysis is helpful for preoperative differential diagnosis.
**Limitations:** Single centre study with relatively small sample size and retrospective nature of the study were predominant limitations.
**Ethics committee approval:** This was a retrospective observational study approved by the institutional review board of our university hospital (ID: B2020-424R). The informed consent was waived. All aspects of this study were performed in accordance with the Declaration of Helsinki.
**Funding for this study:** This study was supported by National Natural Science Foundation of China (Grant No. 82071942).
**Author Disclosures:**
Wen-Ping Wang: Nothing to disclose
Yunlin Huang: Nothing to disclose
Sheng Chen: Nothing to disclose
Yi Dong: Nothing to disclose
Juan Chen: Nothing to disclose
Ying Wang: Nothing to disclose

### RPS 2316-10

**Body CT scan for oncologic patients: the impact of subspecialty radiology in the clinical practice: a quality care study**

S. Rizzo[1], L. Bellesi[2], A. D'Ermo[2], *L. Bonomo*[1], O. D'Ecclesiis[3], S. Presilla[2], E. Rezzonico[1], M. Del Grande[2], F. Del Grande[1]; [1]Lugano/CH, [2]Bellinzona/CH, [3]Milan/IT

**Purpose:** The primary objective of this study was to assess if the CT dose delivered to oncological patients was different in a subspecialty radiology department, compared to a general radiology department. Secondary explorative objective was to assess if the objective image quality of CT examinations was different in the two settings.
**Methods or Backround:** Chest and abdomen CT scans performed for oncological indications were selected from a general radiology department and a subspecialty radiology department. By using a radiation dose management platform, we extracted and compared CT dose index (CTDIvol) and dose length product (DLP) both for each phase and for the entire CT exams. For objective image quality evaluation, we calculated the signal-to-noise ratio (SNR) and the contrast-to-noise ratio (CNR) at the level of the liver and of the aorta. Appropriate statistical analysis was performed. P-value < 0.05 was considered significant. The statistical analyses were performed with R software, version 4.2.3.

**Results or Findings:** Sven thousand and ninety-eight CT examinations were included. CTDIvol was evaluated in 12804 phases; DLP in 10713 phases and in 6714 examinations. The CTDIvol and DLP overall as well as in the native, arterial and portal venous phases were significantly lower in the subspecialty radiology department. The objective image quality showed no significant difference in the general and subspecialty radiology departments.
**Conclusion:** In a subspecialty radiology department, CT protocols are optimized compared to general radiology department leading to lower dose to oncologic patients without significant objective image quality degradation.
**Limitations:** We did not evaluate the patients' exposure through the effective dose; we cannot exclude that other factors may have influenced the difference in radiation dose; we did not evaluate the effects of the reduction of radiation dose and the effects of the choice of a different imaging.
**Ethics committee approval:** The present study was considered a quality care control study by our Ethical Committee and did not fall under the Swiss law of the human research. As such, specific approval and informed consent were waived.
**Funding for this study:** No funding was received for this study.
**Author Disclosures:**
Ermidio Rezzonico: Nothing to disclose
Stefano Presilla: Nothing to disclose
Maria Del Grande: Nothing to disclose
Luca Bellesi: Nothing to disclose
Oriana D'Ecclesiis: Nothing to disclose
Stefania Rizzo: Nothing to disclose
Filippo Del Grande: Nothing to disclose
Andrea D'Ermo: Nothing to disclose
Luca Bonomo: Nothing to disclose

### RPS 2316-11

**Comparison of iRECIST and RECIST 1.1 for response assessment of immune checkpoint inhibitor therapy**
*C. Nelles*, P. Bernard, N. Große Hokamp, M. Gräf, T. Persigehl, P. J. Bröckelmann, S. Lennartz; Cologne/DE

**Purpose:** To compare response assessment of patients undergoing immune checkpoint inhibitor (ICI) therapy between iRECIST and RECIST 1.1 in a clinical cohort of patients with non-small cell lung cancer (NSCLC) or melanoma.
**Methods or Backround:** 216 patients with NSCLC or melanoma who received PD-1 inhibitors (Nivolumab or Pembrolizumab) or CTLA-4 inhibitor Ipilimumab between January 2015 and May 2020 and who underwent oncologic staging and follow-up CT of the chest and abdomen were included in this retrospective analysis. Treatment response assessment in compliance with the RECIST 1.1 and iRECIST guidelines was performed for all patients for the time period of ICI therapy. Response patterns as well as overall response rate (ORR) and progression-free survival time (PFS) were compared between iRECIST and RECIST 1.1.
**Results or Findings:** Out of 110 (50.9%) patients with progressive disease (PD) according to RECIST 1.1, 34 did not reach confirmation of progression (iCPD) in iRECIST and for 9 patients, iCPD was reached at a later point in time compared to RECIST 1.1, with a resulting difference of mean PFS (225.3 ± 92.8 days for RECIST 1.1 vs. 270.7 ± 91.9 days for iRECIST). In iRECIST, unconfirmed progressive disease (iUPD) transitioned to stable disease (iSD) in the subsequent follow-up in 17 patients and to partial response (iPR) in 12 patients, leading to a difference in ORR (31.0% for RECIST 1.1 and 35.2% for iRECIST).
**Conclusion:** Our analysis suggests that iRECIST is more suitable than RECIST 1.1 for correctly capturing atypical response to ICI therapy in patients with melanoma and NSCLC outside of clinical trials, resulting in differences in clinical outcome evaluation.
**Limitations:** This was a retrospective and a monocentre study design, we did not assess response patterns separately for melanoma and NSCLC.
**Ethics committee approval:** After reviewing the study design, the institutional review board waived the need for informed patient consent and approved this retrospective monocentre study.
**Funding for this study:** No funding was received for this study.
**Author Disclosures:**
Simon Lennartz: Speaker: Amboss GmbH Author: Amboss GmbH
Nils Große Hokamp: Research/Grant Support: Philips Healthcare; Speaker: Philips Healthcare
Paul J. Bröckelmann: Nothing to disclose
Pascale Bernard: Nothing to disclose
Christian Nelles: Nothing to disclose
Moritz Gräf: Nothing to disclose
Thorsten Persigehl: Nothing to disclose

**09:30-11:00 — Research Stage 4**

### Research Presentation Session: Neuro

### RPS 2311

### Stroke: improving diagnosis through imaging

**Moderator**
K. Sklinda; Warsaw/PL

### RPS 2311-2

**Cerebral collaterals are associated with pre-treatment brain-blood barrier permeability in acute ischaemic stroke patients**
*A. Bani Sadr*, L. Mechtouff, M. Hermier, C. De Bourguignon, A. Martin, E. Tommasino, T-H. Cho, N. Nighoghossian, Y. Berthezene; Lyon/FR
*(apbanisadr@gmail.com)*

**Purpose:** The aim of this study was to investigate the relationship between cerebral collateral status and blood-brain barrier (BBB) permeability at admission MRI in a cohort of acute ischemic stroke (AIS) patients treated with mechanical thrombectomy.
**Methods or Backround:** The HIBISCUS-STROKE cohort is a single-centre observational study that prospectively included AIS patients with anterior circulation occlusion treated with mechanical thrombectomy. Admission dynamic-susceptibility MRI were post-processed to provide K2 maps with arrival-time correction as a marker of BBB permeability. After co-registration with ADC maps, 90th percentiles of K2 were extracted in the infarct core and normalised to contralateral white matter. Cerebral collateral status was assessed by the hypoperfusion intensity ratio (HIR). Good collaterals were defined by HIR<0.4. Multiple variable logistic regression analysis was adjusted to investigate factors associated with poor collaterals.
**Results or Findings:** One hundred and fifty seven patients were included (67.9□16.6 years, 52.8% male) with a median HIR of 0.43 (interquartile range (IQR): [0.32; 0.65]) and a median K2 of 1.67 (IQR: [0.45; 65.94]). Patients with poor collaterals (n=81, 51.6%) had worse NIHSS score (P=0.01), larger infarct core (P<0.0001) and higher K2 (median: 3.1, IQR: [0.5; 85.1] versus 1.2; IQR: [0.5; 3.4]; P=0.002). They were less likely to achieve successful recanalization (P=0.004) and had a higher rate of haemorrhagic transformation (P=0.02). On multiple variable analysis, poor collaterals were independently associated with larger infarct core volume (odds ratio (OR)=1.10; 95% confidence interval (CI): [1.06; 1.13]; P=0.002) and K2 (OR=1.46; 95% CI: [1.18; 1.84]; P=0.004).
**Conclusion:** Poor collaterals are independently associated with a larger infarct core and increased BBB permeability at admission MRI.
**Limitations:** This study was a retrospective analysis of a single-centre cohort, limiting its scope.
**Ethics committee approval:** This study was ethically approved by the IRB number: 00009118.
**Funding for this study:** This study was funded by the RHU MARVELOUS (ANR-16-RHUS-0009) of Université de Lyon, within the program "Investissements d'Avenir" operated by the French National Research Agency.
**Author Disclosures:**
Yves Berthezene: Nothing to disclose
Marc Hermier: Nothing to disclose
Laura Mechtouff: Nothing to disclose
Emanuele Tommasino: Nothing to disclose
Norbert Nighoghossian: Nothing to disclose
Anna Martin: Nothing to disclose
Tae-Hee Cho: Nothing to disclose
Charles De Bourguignon: Nothing to disclose
Alexandre Bani Sadr: Nothing to disclose

### RPS 2311-3

**Accuracy of artificial intelligence for diagnosing intracranial haemorrhage: results of a year-long multicentre clinical monitoring study**
*A. N. Khoruzhaya*, K. M. Arzamasov, D. V. Burenchev, E. I. Kremneva, A. Vladzymyrskyy, Y. Vasilev; Moscow/RU
*(khoruanna69@yandex.ru)*

**Purpose:** The objective of this study was to evaluate the performance metrics of an artificial intelligence (AI) service aimed at diagnosing intracranial haemorrhage on head CT during a year-long multicentre clinical follow-up.
**Methods or Backround:** As part of the Moscow Computer Vision Experiment, an AI service with known characteristics obtained from calibration testing (sensitivity - 0.89, specificity - 0.96, ROC AUC - 0.96) was connected on 28 April 2022 to the CT machines of 56 inpatient medical institutions.

At the end of April 2023, it had analysed a total of 133,506 head CT. In order to assess accuracy rates, monthly clinical monitoring was conducted throughout the year, during which 1112 randomly selected head CT scans were independently assessed by three radiologists with more than 3 years' experience, assessing the actual presence of ICH (0/1) and the result provided by the AI service (trigger threshold 0.75). According to the results of physician judgement (GT), 440 CT studies (39%) contained signs of ICH.
**Results or Findings:** Full concordance of brain CT assessment by radiologists and AI service was achieved in 57% (633 CTs), partial concordance in 22% (249). The number of false positive responses by the AI service was 19% (212) and false negative responses were 1% (11). Thus, sensitivity was 97.5%, specificity was 68.4%, and ROC AUC was 0.94.
**Conclusion:** The AI service in a year-long multicentre clinical monitoring demonstrated a higher sensitivity than calibration testing, but lost significantly in specificity. This suggests good potential for the service to be used in acute care and to perform triage.
**Limitations:** The decrease in AI performance metrics should be taken into account for long-term use in practice and feedback should be given to developers for further retraining of AI services to reduce the number of FP results.
**Ethics committee approval:** The study was conducted in accordance with the Declaration of Helsinki, and approved by the Independent Ethics Committee of MRO RORR (protocol code 2/2020, the date of approval February 20, 2020). Clinical trial: NCT04489992.
**Funding for this study:** This study was funded by the Program of the Moscow Healthcare Department “Scientific Support of the Capital's Healthcare” for 2023–2025: AAAA-A21-121012290079-3.
**Author Disclosures:**
Yurii Vasilev: Nothing to disclose
Elena Igorevna Kremneva: Nothing to disclose
Dmitry V Burenchev: Nothing to disclose
Anton Vladzymyrskyy: Nothing to disclose
Kirill Mihajlovich Arzamasov: Nothing to disclose
Anna Nikolaevna Khoruzhaya: Nothing to disclose

### RPS 2311-4

**Deep-learning augmented contrast enhancement improves the detection of cerebral vessel occlusions in CT-angiography of acute stroke patients**

*S. Steinmetz*, M. A. Abello Mercado, A. Sanner, M. A. Brockmann, A. Othman; Mainz/DE
*(steinmetz.sebastian@icloud.com)*

**Purpose:** The aim of this study was to examine the impact of deep-learning augmented contrast enhancement on diagnostic performance of poorly contrasted computed tomography angiography (CTA) in acute stroke.
**Methods or Backround:** This retrospective single centre study included patients with suspected ischaemic stroke who underwent comprehensive CT imaging including cranial computed tomography (CCT), whole brain volume perfusion CT (VPCT) and computed tomography angiography (CTA) and had poorly contrasted CTA (defined as <350HU in the proximal MCA) between 01/2021 and 12/2022. 58/102 patients had vascular occlusion with correlate in perfusion. Datasets were reconstructed both conventional iterative methods (conventional CTA) and additionally using a pre-trained deep learning model allowing selective boosting of iodine-based contrast agents (enhanced CTA). Subjective image analysis was conducted by three readers, who rated general and vessel specific parameters on a 4-point Likert-scale. Furthermore, they evaluated both datasets for presence / absence of cerebral vessel occlusions. VPCT served as reference standard for calculating sensitivity and specificity.
**Results or Findings:** Enhanced CTA revealed significantly higher subjective image quality parameters (p<.001). Readers significantly improved sensitivity by enhanced CTA compared to conventional CTA (Reader 1 and 3: 55/58 [95%; 95% CI: 85.62% to 98.92%] vs. 48/58 [83%; 95% CI: 70.57% to 91.41%]; Reader 2: 53/58 [91%; 95% CI: 81.02% to 97.14%] vs. 46/58 [79%; 95% CI: 66.65% to 88.83%]. Reader 1 and 3 yielded no false positive findings on conventional CTA or enhanced CTA (specificity 44/44 [100%; 95% CI: 91.96% to 100%]), reader 2 yielded one false positive on enhanced CTA (specificity 43/44) [98%; 95% CI: 87.98% to 99.94%].
**Conclusion:** Deep-learning augmented contrast enhancements improves image quality and diagnostic performance in poorly contrasted CT angiographies. This could contribute to improve diagnostic certainty in acute stroke.
**Limitations:** Retrospective study design limits this study.
**Ethics committee approval:** This was a retrospective study and was approved by the Ethics committee, application number: 2022-16477.
**Funding for this study:** No funding was obtained for this study.
**Author Disclosures:**
Marc A. Brockmann: Nothing to disclose
Mario Alberto Abello Mercado: Nothing to disclose
Sebastian Steinmetz: Nothing to disclose
Antoine Sanner: Nothing to disclose
Ahmed Othman: Nothing to disclose

### RPS 2311-5

**Risk identification for the development of large-artery vasospasm after aneurysmatic subarachnoid haemorrhage: a multivariate, risk- and location-adjusted prediction model**

*J. Schwarting*, D. Trost, C. Albrecht, C. Zimmer, M. Wostrack, B. Meyer, J. H. W. Bodden, T. Boeckh-Behrens; Munich/DE

**Purpose:** Large-artery vasospasm (CVS) after aneurysmatic subarachnoid hemorrhage (aSAH) can reduce cerebral perfusion and cause severe neurological deficits. Delayed recognition of CVS risks the success of endovascular spasmolysis. An analysis of potential risk factors could be used to enable risk stratification for early therapeutic interventions. Therefore, the aim of this study was to confirm established and identify unknown risk factors for CVS at the time of aneurysm occlusion.
**Methods or Backround:** In a single-center retrospective cohort study design, we compared 853 SAH patients (mean age 57; 67% female) between 01/2006 and 03/2020. Patients with and without CVS were compared based on demographic, clinical, and radiographic parameters at the time of aneurysm occlusion. CVS was defined as a blood flow velocity of >200 cm/s in large intracranial arteries or the occurrence of secondary neurological deficits without competing causes. Cohort differences were included as predictors in a multivariate analysis to address confounding. Logistic regression models were used to determine odds ratios (OR) for the presence of CVS for each predictor.
**Results or Findings:** 32% of SAH patients developed CVS. CVS was associated with age, female sex, aneurysm location, modified Fisher score, Barrow Neurological Institute (BNI) score, and surgical interventions in univariate analysis. Multivariable regression analysis identified multiple risk factors (Table 1). BNI score (OR: 1.33, p = 0.002), de-compressive craniectomy (OR: 1.93, p = 0.005), and aneurysm clipping (OR: 2.22, p < 0.001), were identified as only independent risk factors after correction for age, sex, aneurysm site and clinical severity.
**Conclusion:** Patients undergoing surgical interventions or patients with thick layers of subarachnoid blood should be monitored most intensively after aneurysm occlusion for early detection of CVS and endovascular intervention.
**Limitations:** The most important limitation was the retrospective, single-center study design.
**Ethics committee approval:** The study was approved by the local Research Ethics committee of the Technical University of Munich (186/20S).
**Funding for this study:** We did not receive any 3rd party funding for this study.
**Author Disclosures:**
Bernhard Meyer: Nothing to disclose
Claus Zimmer: Nothing to disclose
Dominik Trost: Nothing to disclose
Carolin Albrecht: Nothing to disclose
Julian Schwarting: Nothing to disclose
Jannis Hubert Wilhelm Bodden: Nothing to disclose
Tobias Boeckh-Behrens: Nothing to disclose
Maria Wostrack: Nothing to disclose

### RPS 2311-6

**Cost-effectiveness of endovascular thrombectomy in acute ischaemic stroke with large infarct**

*J. Schwarting*[1], M. Froelich[2], J. S. Kirschke[1], J. H. W. Bodden[1], K. Dimitriadis[1], J. Ricke[1], C. Zimmer[1], T. Boeckh-Behrens[1], W. G. Kunz[1]; [1]Munich/DE, [2]Mannheim/DE

**Purpose:** Endovascular thrombectomy (EVT) is the standard of care for acute large vessel occlusion stroke. Recently, the ANGEL-ASPECT and SELECT 2 trials showed improved outcomes in patients with acute ischaemic Stroke presenting with large infarcts. The cost-effectiveness of EVT for this subpopulation of stroke patients has only been calculated with data from the previously published RESCUE-Japan LIMIT trial. It is therefore limited in its generalizability to an international population. The aim of this study was, therefore, to simulate patient-level costs, analyze the economic potential of EVT for patients with large ischaemic stroke from a public health payer perspective based on the recently published data, and identify significant determinants of cost-effectiveness.
**Methods or Backround:** A Markov model was developed to compare outcome and cost parameters for patients treated with EVT or only with the best medical care based on the three recent prospective clinical trials ANGEL-ASPECT, SELECT2 and RESCUE-Japan LIMIT. Treatment outcomes were derived from the most recent literature. Deterministic and probabilistic sensitivity analyses addressed uncertainty. Willingness to pay was set at $100,000 per quality-adjusted life year (QALY).
**Results or Findings:** Endovascular treatment yielded an incremental gain of 1.32 QALYs per procedure with cost savings of $17,318 per patient. Lifetime costs were most sensitive to the costs of the endovascular procedure.

**Conclusion:** EVT is a cost-saving (i.e., dominant) strategy for patients presenting with large ischemic cores defined by inclusion criteria of the recently published ANGEL-ASPECT, SELECT2, and RESCUE-Japan LIMIT trials in comparison to best medical care in our simulation. Prospective data of individual patients need to be collected to validate these results.
**Limitations:** Simulation based on simplified linear pathways for diagnostics and therapy with limitations derived from the availability, quality, and validity of input variables.
**Ethics committee approval:** This was a Cost-effectiveness analysis without the use of individual patient data.
**Funding for this study:** No third party funding was used for this study.
**Author Disclosures:**
Konstantinos Dimitriadis: Nothing to disclose
Matthias Froelich: Nothing to disclose
Jan S. Kirschke: Nothing to disclose
Claus Zimmer: Nothing to disclose
Julian Schwarting: Nothing to disclose
Wolfgang Gerhard Kunz: Nothing to disclose
Jannis Hubert Wilhelm Bodden: Nothing to disclose
Tobias Boeckh-Behrens: Nothing to disclose
Jens Ricke: Nothing to disclose

### RPS 2311-7

### Comparative evaluation of 5.0T and 3.0T TOF MRA in assessing collateral circulation in moyamoya disease: a focus on disease progression and diagnostic precision

*Y. Zhou*, Y. Zhai, K. Xue, Y. Yang, D. Zhang, J. Ni, F. Feng, Y. Wang; Beijing/CN

**Purpose:** The aim of this study was to compare the efficacy of 5.0T and 3.0T TOF MRA in evaluating collateral circulation and visualising abnormal vascular networks in patients diagnosed with moyamoya disease (MMD).
**Methods or Backround:** Using both 5.0T and 3.0T MRI systems, a retrospective analysis was conducted on 30 symptomatic hemispheres from 21 ischemic-type MMD patients (10 males, age 7–39 years). A 0-5 grading system evaluated the visibility of moyamoya vessels across 5 regions (basal ganglion, anterior communicating artery, MCA–ICA tip, posterior communicating artery-PCA, basilar artery tip areas). Leptomeningeal anastomoses were evaluated with scores ranging from 0 to 6, comprising three parts of the collateral networks (pPCA→ ACA, the anterior temporal branch of the PCA anastomoses to the temporal branch of the MCA, pPCA→ MCA ). High-signal-intensity regions in basal ganglia was manually counted. Comparative analysis of moyamoya vessels and leptomeningeal system scores between 3.0 and 5.0T MRA was executed using the Wilcoxon matched-pair signed-rank test. A paired t-test was employed to juxtapose the number of high-signal-intensity regions between 3.0T and 5.0T MRA.
**Results or Findings:** The 5.0T TOF MRA demonstrated enhanced detection capabilities, showing a more significant number of moyamoya vessels and leptomeningeal anastomoses compared to 3.0T MRA. Furthermore, 5.0T MRA was more adept at detecting high-signal-intensity regions in the basal ganglia. Overall, 5.0T MRA provided clearer visualisation of the abnormal vascular networks associated with MMD.
**Conclusion:** The 5.0T TOF MRA presents a promising diagnostic tool for MMD, offering superior visualization of abnormal vascular networks and potentially aiding in more accurate disease assessment and prognosis.
**Limitations:** The parameters for 3.0T and 5.0T MRI couldn't be perfectly matched. The small sample size might lead to false-negative findings.
**Ethics committee approval:** The study was approved by the Ethics Committee at Peking Union Medical College Hospital (Reference number: K3147).
**Funding for this study:** This study was funded by the Major International (Regional) Joint Research Project of National Natural Science Foundation of China (Grant Nos. 82020108018, 2020), the Beijing Natural Science Foundation (Grant Nos. Z210013, 2021) and National High Level Hospital Clinical Research Funding (2022-PUMCH-B-027).
**Author Disclosures:**
Yuanren Zhai: Nothing to disclose
Yining Wang: Grant Recipient: This study was funded by the Major International (Regional) Joint Research Project of National Natural Science Foundation of China (Grant Nos. 82020108018, 2020), the Beijing Natural Science Foundation (Grant Nos. Z210013, 2021) and National High Level Hospital Clinical Research Funding (2022-PUMCH-B-027).
Yuxin Yang: Employee: MR Collaboration, United Imaging Research Institute of Intelligent Imaging.
Ke Xue: Employee: MR Collaboration, United Imaging Research Institute of Intelligent Imaging.
Yijun Zhou: Nothing to disclose
Jun Ni: Nothing to disclose
Dong Zhang: Nothing to disclose
Feng Feng: Nothing to disclose

### RPS 2311-8

### Generalisation to ultra low-field MRI of an algorithm for detection of acute cerebral infarcts

*S. Ingala*[1], M. Perslev[1], S. Yamazaki Jensen[1], K. Dmitriev[2], J. Schlemper[2], C. Truwit[2], M. B. Bachmann Nielsen[1], M. Sofka[2], A. Pai[1]; [1]Copenhagen/DK, [2]Palo Alto, CA/US
*(silvia.ingala@gmail.com)*

**Purpose:** Cerebriu apollo brain is a software designed to detect acute cerebral infarcts on standard (1.5T and 3T) magnetic resonance images (MRIs). In this work, we aim to investigate the generalisation of the out-of-the-box software in detecting acute cerebral infarcts on ultra-low field MRI (ULF-MRI).
**Methods or Backround:** Background: Apollo Brain was trained on 1300 standard MRI scans to detect acute cerebral infarcts, intracranial tumors, and intracranial haemorrhages. In this work, only the cerebral infarct detection feature is evaluated. Apollo brain accepts T2-FLAIR and DWI sequences with an option to add either T2* or susceptibility weighted images (SWI). No additional training or fine-tuning was performed for generalisation to ULF-MRI sequences. Methods: MRI images using Hyperfine Swoop scanner (software version > 8.4.0) collected between July 27, 2022, and June 13, 2023 were gathered. Inclusion criteria were a human non-contrast MRI imaging study indicating ischaemic stroke or intracranial hemorrhage (intraparenchymal or subarachnoid haemorrhage). We excluded MRI images displaying significant motion and other artifacts. All the scans were reviewed by an experienced radiologist. Then, Apollo brain performance was tested.
**Results or Findings:** A total of 30 subjects were included. Of these, 19 were diagnosed with acute cerebral infarct, including one with signs of haemorrhagic transformation of the infarct. Apollo brain software was able to detect the infarcts with a sensitivity of 94% and specificity of 83%. The software had one false negative , and two false positives.
**Conclusion:** Out-of-the-box Apollo Brain software for detection of acute intracerebral infarcts exhibited excellent performance with ULF-MRI images. These results demonstrate the possibility for an accelerated adoption of ULF-MRI in low resource settings for detecting acute cerebral infarcts coupled with Apollo brain software.
**Limitations:** We expect to improve the study by increasing the sample size.
**Ethics committee approval:** No information was provided by the submitter.
**Funding for this study:** No information was provided by the submitter.
**Author Disclosures:**
Chip Truwit: Consultant: Hyperfine
Simon Yamazaki Jensen: Employee: Cerebriu A/S
Jo Schlemper: Employee: Hyperfine
Michael B. Bachmann Nielsen: Nothing to disclose
Akshay Pai: Shareholder: Cerebriu A/S
Silvia Ingala: Consultant: Cerebriu A/S
Konstantin Dmitriev: Employee: Hyperfine
Michal Sofka: Employee: Hyperfine
Mathias Perslev: Employee: Cerebriu A/S

### RPS 2311-9

### Spectral imaging and analysis of monophasic CT angiography for assessment of penumbra and infarct core in acute stroke

*S. Aludin*, L-P. Schmill, P. Langguth, O. Jansen, N. Larsen, F. Wodarg, T. Klintz, S. Seehafer, A. Horr; Kiel/DE

**Purpose:** CT-angiography (CTA) and CT-perfusion (CTP) are part of acute stroke imaging. CTP thereby estimates the irreversibly damaged parenchyma, the infarct core (IC), and the potentially salvageable parenchyma, the penumbra (PEN), but requires additional contrast and radiation. Spectral-detector-CT (SDCT) enables spectral imaging like iodine-density imaging or virtual-monoenergetic imaging and the aim of this study was to assess its potential in differentiating IC and PEN using only monophasic CTA.
**Methods or Backround:** Twenty patients with stroke in the media territory were analysed. Areas of IC and PEN derived from CTP-analysis as well as their healthy hemisphere's counterparts were transferred to spectral maps of the CTA as regions of interest. The areas were compared to each other and between the different spectral maps (measurements of Hounsfield-Unit in monoenergetic images (MonoE) at 40 keV, 70 keV, and 120 keV, plus iodine-density (ID) and electron-density (ED) values). Unilateral absolute values and ratios formed to the healthy hemisphere's values were evaluated. Visual infarct delineation was also rated in each map.
**Results or Findings:** IC and PEN could be distinguished from their healthy counterpart by absolute values ($p<0.01$). IC could be differentiated from PEN by absolute values ($p<0.0001$, except for ED) and by the ratio formed to the healthy counterpart ($p<0.01$). Thereby, discrimination of IC and PEN by ratio was good in MonoE40 (AUC=0.74, $p<0.0001$) and best in ID (AUC=0.92, $p<0.0001$). Visual delineation was best in ID and MonoE40.
**Conclusion:** SDCT-derived spectral maps from monophasic CTA, such as ID or MonoE40, allow detection and differentiation of IC and PEN. As a result, additional CTP may be removed from acute stroke CT protocols in the future, potentially saving time, contrast and radiation exposure.

**Limitations:** The limitations of the study are the low number of individuals, thus further studies are needed.
**Ethics committee approval:** The study was ethically approved by the institutional review board and written informed consent was available from all participants.
**Funding for this study:** No funding was sought for this study.
**Author Disclosures:**
Tristan Klintz: Nothing to disclose
Fritz Wodarg: Nothing to disclose
Lars-Patrick Schmill: Nothing to disclose
Patrick Langguth: Speaker: Philips GmbH Market DACH
Schekeb Aludin: Nothing to disclose
Svea Seehafer: Nothing to disclose
Agreen Horr: Nothing to disclose
Olav Jansen: Nothing to disclose
Naomi Larsen: Nothing to disclose

### RPS 2311-10

**Validation of the novel combined marker Haematoma Maturity Score for intracerebral haemorrhages**

*B. T. Catelani*[1], A. Lopez Rueda[2], M. E. Santos Armentia[3], G. B. Harvey[1], A. Pérez Fernandez[3]; [1]Rosario/AR, [2]Barcelona/ES, [3]Vigo/ES
*(barbaracatelani@gmail.com)*

**Purpose:** This study aimed to validate the novel combined indicator Hematoma Maturity Score using non-contrast computed tomography (NCCT) scans in patients with intracerebral haemorrhage (ICH) for predicting functional outcomes at discharge, in comparison to individual radiological NCCT signs.
**Methods or Backround:** A retrospective analysis of patients with ICH who had undergone baseline CT scans was performed. The Black Hole Sign, Blend Sign, Island Sign, Swirl Sign, and the Hematoma Maturity Score were independently assessed by two radiologists who were blinded to clinical information. Patients were dichotomised based on dependency and mortality at discharge using the Modified Rankin Scale (mRS): no symptoms or no significant/mild disability (mRS 0–2); moderate/severe disability or mortality (mRS 3-6).
**Results or Findings:** Sixty-four patients with confirmed spontaneous ICH identified on NCCT were included. The Swirl Sign, Island Sign, and Hematoma Maturity Score exhibited statistically significant associations with the clinical outcomes of the patients ($p < 0.01$). There was almost perfect agreement between readers regarding the maturity score (kappa = 0.84), and substantial agreement for the other signs.
**Conclusion:** The novel concept of the Hematoma Maturity Score demonstrated the highest impact on clinical outcomes when compared to other evaluated radiological signs, with almost perfect agreement between readers.
**Limitations:** No limitations have been identified for this study.
**Ethics committee approval:** This study has been approved by the local hospital Ethics Committee.
**Funding for this study:** No funding was received for this study.
**Author Disclosures:**
Guillermina Beatriz Harvey: Nothing to disclose
Bárbara Teresa Catelani: Nothing to disclose
Mª Eloisa Santos Armentia: Nothing to disclose
Antonio Lopez Rueda: Nothing to disclose
Anabel Pérez Fernandez: Nothing to disclose

### RPS 2311-11

**Improved neurovascular imaging using Advanced intelligent Clear-IQ Engine (AiCE)**

M. A. Abello Mercado, *S. Steinmetz*, A. Sanner, A. Kronfeld, M. A. Brockmann, A. Othman; Mainz/DE
*(steinmetz.sebastian@icloud.com)*

**Purpose:** The aim of this study was to evaluate the effects of deep-learning image reconstruction on image quality and diagnostic confidence of ultra-high-resolution computed tomography (UHRCT).
**Methods or Backround:** In this single-center study, 100 consecutive patients with acute neurological symptoms underwent CT imaging including cranial computed tomography (CCT) and computed tomography angiography (CTA) using an ultra-high resolution CT scanner. CTA images were reconstructed with normal resolution mode and ultra-high resolution mode using iterative reconstruction. A deep-learning reconstruction algorithm (advanced intelligent clear-IQ engine, AiCE); specifically trained for ultra-high resolution CT-angiography of the brain was utilized to generate a further UHR-CTA datasets (DL-UHR-CTA, matrix 1024 x 1024, slice thickness 0.25 mm). Image quality for all three reconstructions was evaluated visually by two blinded radiologists using a 4-point Likert-scale. Therefore, general (overall image quality, contrast in general, artifacts, diagnostic confidence and image noise) and vessel specific (assessability of proximal, intermediate and subcortical vessels as well as perforators) criteria were assessed. The quantitative features including slope, signal-to-noise ratio (SNR), contrast-to-noise ratio (CNR), noise, entropy and co-occurrence matrix (COOC) were examined and compared using an in-house tool.
**Results or Findings:** Qualitative analysis revealed highest scores for DL-UHR-CTA, followed by UHR-CTA and NR-CTA, whereas DL-UHR-CTA yielded excellent results for all qualitative parameters and was significantly superior to UHR-CTA and NR-CTA (all $p<0.001$). The quantitative analysis was in line with the qualitative findings with significantly superior results for DL-UHR-CTA (slope: $p<.01$, SNR/CNR: $p=0.004$, entropy $p<.01$, COOC: $p<.01$).
**Conclusion:** Deep-Learning image reconstruction significantly improves image quality of ultra-high resolution neurovascular CT-angiography allowing for higher diagnostic confidence, potentially improving the detection of subtile but oftentimes-significant pathologies.
**Limitations:** Deep-learning image reconstruction improves the quality of UHR-CTA images, leading to higher diagnostic confidence and potentially aiding in the detection of subtile but clinically significant pathologies.
**Ethics committee approval:** Approval was received from the Ethics committee, application number: 2021-15948:1-retrospektiv.
**Funding for this study:** No funding was received for this study.
**Author Disclosures:**
Marc A. Brockmann: Nothing to disclose
Mario Alberto Abello Mercado: Nothing to disclose
Andrea Kronfeld: Nothing to disclose
Sebastian Steinmetz: Nothing to disclose
Antoine Sanner: Nothing to disclose
Ahmed Othman: Nothing to disclose

### RPS 2311-12

**Assessment of cerebral blood flow distribution at different phases between different types of the circle of Willis using 4D Ffow imaging**

*B. Sui*, B. B. XiaoYan; Beijing/CN
*(reneesui@163.com)*

**Purpose:** Haemodynamic factors have been proved to play essential roles in the formation and development of vascular diseases. This study aimed to investigate the blood flow distribution in the different types of circle of Willis and the changes of blood flow at different phases of the cardiac cycle using 4D Ffow imaging.
**Methods or Backround:** Four-dimensional flow magnetic resonance imaging (4D flow MRI) was performed in 30 healthy subjects (mean 27.5±4.1 years; range 24-38 years old). CoW was classified into five types according to the vessel anatomical structures. The cerebral blood flow distribution at different arteries and the total cerebral blood flow (tCBF) were analyzed and compared between different types of CoWs. The changes of cerebral blood flow during different phases of the cardiac cycle were observed and compared between different types of CoWs.
**Results or Findings:** Twelve subjects (40%) were found with complete CoW, and eighteen (60%) with incomplete CoW. No significant difference was found in tCBF between different types of CoWs ($P=0.787$). In subjects with a fetal-type PCA (Type V), the average flow rates (FRavg) at contralateral ICA was higher than that of the ipsilateral ICA ($P=0.003$). During the peak systolic and end diastole, compared with Type I, in subjects with unilateral PCoA opening (Type IV), the Δflow rates (ΔFR) at bilateral MCA were significantly higher than that of Type I ($P=0.012$ and $P=0.006$, respectively); in subjects with unilateral fetal-type PCA (Type V), the ΔFR at contralateral MCA were significantly higher than that of Type I ($P=0.007$).
**Conclusion:** Blood flow distribution exists a difference in different types of CoWs during the different phases of the cardiac cycle, especially in type IV and type V at peak systolic.
**Limitations:** small samples for type V and VI.
**Ethics committee approval:** This study was approved by the local ethics committee of Beijing Tiantan Hospital, and all volunteers gave written informed consent.
**Funding for this study:** This study was funded by the Beijing Municipal Natural Science Foundation (NO. 7162056, 7212028).
**Author Disclosures:**
Bai Bai XiaoYan: Nothing to disclose
Binbin Sui: Nothing to disclose

11:30-12:30 Research Stage 1

### Research Presentation Session: Artificial Intelligence & Machine Learning & Imaging Informatics

### RPS 2405

### AI in breast cancer screening

**Moderator**
S. Astley; Manchester/UK
*(sue.astley@manchester.ac.uk)*

### RPS 2405-2

**Bimodal CADx assessment in mammography and tomosynthesis: a standalone study for breast screening**

*H. Beaumont*, A. Iannessi, S. Pacilè, T. Louis; Nice/FR
*(hubertbeaumont@hotmail.com)*

**Purpose:** Digital mammography (FFDM) is the standard for breast cancer screening. Digital breast tomosynthesis (DBT), compared to FFDM, enhances cancer detection and reduces unnecessary biopsies. Despite DBT's adoption, critical questions remain—higher radiation, time, cost, and clinical benefits, particularly for systematic breast screening. In the era of AI CADx (Computer-Aided Diagnosis) for breast screening, one unresolved question is the role of bimodal algorithms in predicting cancer risk and offering guidance when opinions differ, and we aim to understand this.
**Methods or Backround:** We analysed a cohort of 1071 screened patients for breast cancer who underwent both mammography and tomography. A CADx software assigned a score of malignancy to each tumour, allowing to compute the joint distribution of the paired mammography/tomography scorings. From the joint distribution, we defined areas of “perpendicular diagnosis” (PD) as the areas of highly discordant scoring. We evaluated the potential of systematic reclassifications of perpendicular scoring through sensitivity and specificity both for tumoural mass and calcifications.
**Results or Findings:** We observed a modest inter-modality agreement, indicated by a kappa of 0.19 (95% CI: 0.18; 0.22). PD scoring was present in 32.7% (95% CI: 29.7; 35.8) of mass cases and 38.6% (95% CI: 30.1; 47.6) of calcification cases. Specific reclassification rules significantly increased mass sensitivity from 0.74 (95% CI: 0.71; 0.77) to 0.80 (95% CI: 0.77; 0.83), resolving 29.1% of PD cases. For calcifications, reclassification addressed 12% of discrepancies, improving specificity from 0.85 (95% CI: 0.76; 0.91) to 0.86 (95% CI: 0.78; 0.92).
**Conclusion:** Our analysis revealed a significant proportion of CADx algorithmic discrepancies. In cases where FFDM classified masses as benign while DBT CADx suggested malignancy, DBT CADx aided decision-making. Conversely, for calcifications initially assessed as negative with tomosynthesis, FFDM CADx proved valuable. Exploring alternative reclassification methods is essential.
**Limitations:** No limitations were applicable for this study.
**Ethics committee approval:** Not applicable for this study.
**Funding for this study:** No funding was obtained for this study.
**Author Disclosures:**
Serena Pacilè: Employee: Therapixel SA
Thomas Louis: Employee: Therapixel SA
Antoine Iannessi: Shareholder: Therapixel SA
Hubert Beaumont: Nothing to disclose

### RPS 2405-3

**Are AI-detected interval cancers actionable for recall in a real screening setting? An informed review of 120 interval cancer cases with high AI scores in breast screen Norway**

*H. W. Koch*[1], M. Larsen[2], S. Hofvind[2]; [1]Stavanger/NO, [2]Oslo/NO

**Purpose:** Retrospective studies have suggested that using artificial intelligence (AI) systems in breast cancer screening might help us detect 30-40% of interval cancers. However, it is uncertain whether the AI-markings match the location of the tumour on diagnostic mammograms, and if the findings are actionable for recall in a real screening setting, which, is the aim of this study.
**Methods or Backround:** In 2022, we conducted a retrospective study comparing the performance of an AI-system with independent double reading by radiologists according to cancer detection. The AI-system (Transpara v.1.7.0) scored mammograms from 1-10 based on risk of malignancy. 42% (120/289) of the interval cancers had an AI-score of 10. In this study, four radiologists did a consensus review of the interval cancers with AI-score 10 and compared AI-markings with cancer location on diagnostic mammograms. Interval cancers were classified as false negative, minimal sign (actionable or non-actionable) or true negative. Mammographic breast density was classified as BI-RADS a-d.
**Results or Findings:** Of 120 interval cancers with AI-score 10 (group1), 77.5% (93/120) had AI-markings matching the cancer location (group2). 20.8% (25/120) had AI-markings matching cancer location and were considered actionable for recall (false negative/minimal sign actionable) (group3). Density distribution as percentage of all 289 interval cancers: Group1: a: 17% (1/6), b: 42% (46/110), c: 41% (56/138), d: 49% (17/35), Group2: a: 17% (1/6), b: 33% (36/110), c: 38% (53/138), d: 9% (3/35), Group3: a: 0% (0/6), b: 10% (11/110), c: 10% (14/138), d: 0% (0/35).
**Conclusion:** Our results indicate that the true effect of AI in screen reading regarding earlier detection of interval cancers is still uncertain. Although 49% of interval cancers in extremely dense breasts had AI-score 10, none were considered actionable for recall in an informed consensus review.
**Limitations:** Retrospective study design and informed consensus review was the limitation of this study.
**Ethics committee approval:** The study was approved by the Regional Committee for Medical and Health Research Ethics (#13294).
**Funding for this study:** No funding was obtained for this study.
**Author Disclosures:**
Henrik Wethe Koch: Nothing to disclose
Solveig Hofvind: Nothing to disclose
Marthe Larsen: Nothing to disclose

### RPS 2405-4

**Integration of artificial intelligence (AI) in double-read population-based mammography screening: simulated replacement of one reader and beyond**

*M. T. Elhakim*[1], S. Wordenskjold Stougaard[1], O. Graumann[2], M. Nielsen[3], O. Gerke[1], L. B. Larsen[1], B. Schnack Brandt Rasmussen[1]; [1]Odense/DK, [2]Aarhus/DK, [3]Copenhagen/DK
*(mohammad.talal.elhakim2@rsyd.dk)*

**Purpose:** The aim of this study was to compare the accuracy and feasibility of three AI-integrated screening scenarios compared to double reading with arbitration (combined reading).
**Methods or Backround:** A study sample of 249,402 consecutive screening mammograms representative of an entire screening population was obtained from the Region of Southern Denmark. The AI system Lunit INSIGHT MMG v.1.1.7.1 (Lunit Inc.) processed all mammograms. In Scenario 1, AI replaced first reader. In Scenario 2, AI replaced second reader when it agreed with the decision of the original first reader. In Scenario 3, AI was applied as a standalone triage tool, with AI replacing both readers for assessing low- and high-risk screenings, while moderate-risk screenings were assessed by the original combined reading. AI cut-offs were chosen partly based on a previous validated threshold and partly based on maintaining a workload reduction at around 50% for comparability across scenarios. Sensitivity, specificity, positive predictive value (PPV), negative predictive value (NPV), recall rate (RR), and arbitration rate (AR) were calculated.
**Results or Findings:** AI cut-off scores were 80.99% (Scenarios 1 and 2), and <3.36% and ≥95.29% for low-risk and high-risk screenings, respectively (Scenario 3). Compared to combined reading, AI-integrated screening showed no statistically significant difference in any outcome measures other than a higher AR by +1.0% (p<0.0001) in Scenario 1, and a higher sensitivity by +1.5% (p=0.001) and lower AR by -0.8% (p<0.0001) in Scenario 3. In Scenario 2, AI-integrated screening had statistically significantly lower sensitivity (-4.5%; p<0.0001), NPV (-0.1; p=0.001), RR (-0.6%; p<0.001), and AR (-1.5; p<0.0001), and higher specificity (+0.6%; p<0.0001) and PPV (+4.7; p<0.0001).
**Conclusion:** Partial or full replacement of one or both readers in double reading with AI seems feasible without markedly affecting accuracy in screening.
**Limitations:** Retrospective design and correlated radiologist readings with reference standard were the limitations of this study.
**Ethics committee approval:** This study was approved by the Danish National Committee on Health Research Ethics (identifier D1763009).
**Funding for this study:** Region of Southern Denmark funded this study.
**Author Disclosures:**
Ole Graumann: Nothing to disclose
Mohammad Talal Elhakim: Nothing to disclose
Lisbet Brønsro Larsen: Nothing to disclose
Sarah Wordenskjold Stougaard: Nothing to disclose
Benjamin Schnack Brandt Rasmussen: Nothing to disclose
Mads Nielsen: Shareholder: Biomediq A/S Board Member: Biomediq A/S
Oke Gerke: Nothing to disclose

### RPS 2405-5

**RSNA 2023 screening mammography breast cancer detection AI challenge results**

G. Partridge[1], M. Vazirabad[2], R. Ball[3], H. Trivedi[4], F. C. Kitamura[5], H. Frazer[6], R. M. Mann[7], L. Moy[8], *Y. Chen*[1]; [1]Nottingham/UK, [2]Chicago, IL/US, [3]Bar Harbor, ME/US, [4]Atlanta, GA/US, [5]São Paulo/BR, [6]South Yarra/AU, [7]Nijmegen/NL, [8]New York, NY/US

**Purpose:** Artificial intelligence (AI), used alongside human readers, in breast cancer screening could revolutionise the screening workflow. The RSNA hosted the 2023 screening mammography breast cancer detection AI challenge, where participants were invited to develop AI algorithms to interpret mammograms. The purpose was to assess the performance of submitted algorithms and explore the potential for improving performance by combining high-performing algorithms.
**Methods or Backround:** Teams were provided a training-set of 11,913 2-view 2D digital mammogram (2DDM) screening cases, from two institutions (US and Australia) for AI training. AI performance was evaluated using an independent test-set of 5,415 2DDM cases from the same source. Cancer cases were pathology proven and non-cancer cases had at least 1-year of normal follow-up. Algorithms were ranked in the challenge using the pF1 accuracy score (incorporating sensitivity and PPV). In the current study, all algorithms were assessed independently. In addition, combined models were constructed from top-ranked algorithms.
**Results or Findings:** One thousand six hundred and eighty seven teams participated in the challenge, each submitting their own algorithm. Median specificity and NPV were high across algorithms (98.7% and 98.5%, respectively), yet median cancer detection was low (sensitivity: 27.6%, PPV: 36.9%), with a median recall rate (RR) of 1.7%. The highest ranked algorithm (as per pF1) had a RR of 1.5%, specificity of 99.5%, NPV of 99.0%, sensitivity of 48.6%, and a PPV of 64.6%. Combining the top 3 and top 10 ranked algorithms demonstrated an increased RR (2.4% and 3.5%), while achieving a marked improvement in sensitivity (60.7% and 67.8%), while specificity remained more stable (98.7% and 97.8%, respectively).
**Conclusion:** Variation in performance of submitted AI algorithms to the RSNA challenge is substantial. Combining the highest-performing algorithms demonstrated improvement in performance.
**Limitations:** Relatively small size of evaluation test-set, low cancer prevalence (but screening setting) limit this study.
**Ethics committee approval:** Not applicable for this study.
**Funding for this study:** No funding was provided for this study.
**Author Disclosures:**
Linda Moy: Nothing to disclose
Felipe Campos Kitamura: Nothing to disclose
Helen Frazer: Nothing to disclose
Robyn Ball: Nothing to disclose
George Partridge: Nothing to disclose
Ritse Maarten Mann: Nothing to disclose
Hari Trivedi: Nothing to disclose
Maryam Vazirabad: Nothing to disclose
Yan Chen: Nothing to disclose

### RPS 2405-6

**Improving breast cancer recurrence forecasts: combining multi-time-point mammography and medical reports**

*C. Lu*[1], X. Wang[1], L. Han[1], T. Zhang[1], Y. Gao[1], T. Tan[1], R. M. Mann[2]; [1]Amsterdam/NL, [2]Nijmegen/NL
*(yangluuu2020@gmail.com)*

**Purpose:** Predicting the risk of recurrence post-breast cancer surgery continues to be a challenging task, despite having access to complete medical records. The purpose of this study was to develop a deep learning model based on multi-time-point mammogram breast images and medical reports to predict the risk of postoperative recurrence of breast cancer.
**Methods or Backround:** At a large academic medical center, we collected consecutive digital screening mammograms and medical reports in 3188 patients between January 1, 2000, and December 31, 2020. Our model synergistically integrates risk factors derived from multi-time-point mammograms with patients' preoperative clinical data. We compared our method with commonly used machine learning methods based on clinical data and image-based deep learning models.
**Results or Findings:** We conducted a comprehensive comparison between our model and common machine learning models as well as deep learning methods, demonstrating that our model attained the highest AUCs in three datasets of patients who relapsed at different times, with scores of 0.72, 0.76, and 0.83 within 5, 10, and 20 years respectively. We discovered that while traditional risk factors are significant contributors, our model enhances the accuracy of predicting cancer recurrence risk by deducing potential risk factors from multi-time-point mammography images.
**Conclusion:** Our model underscores the advantages of incorporating complete and consecutive medical data into predictive algorithms, enhancing accuracy in forecasting recurrence and informing health policies for post-surgical treatment of breast cancer patients.
**Limitations:** This study needs to be validated in more external datasets.
**Ethics committee approval:** Not applicable for this study.
**Funding for this study:** This study is supported by the Chinese Scholarship Council Studentship and the Guangzhou Elite Program (TZ-JY201948).
**Author Disclosures:**
Tao Tan: Nothing to disclose
Yuan Gao: Nothing to disclose
Ritse Maarten Mann: Nothing to disclose
Luyi Han: Nothing to disclose
Xin Wang: Nothing to disclose
Chunyao Lu: Nothing to disclose
Tianyu Zhang: Nothing to disclose

### RPS 2405-7

**Microwave breast cancer screening and early detection: SAFE clinical study**

*A. Janjic*, I. Akduman, M. Cayoren, A. Yurtseven, O. Bugdayci, M. E. Aribal; Istanbul/TR
*(aleksandarjanjic@mitosmedikal.com)*

**Purpose:** The SAFE (Scan and find early) system is a microwave breast cancer imaging (MBI) device designed for non-invasive and non-ionising breast cancer screening and early detection. This technology relies on distinguishing dielectric properties between cancerous and healthy tissue to provide valuable clinical insights. This study aims to evaluate SAFE's capability to precisely identify lesions within the patient's breast.
**Methods or Backround:** This study exclusively enrolled patients scheduled for biopsy, following approval from the ethics committee of Marmara University School of Medicine. The approach utilised to identify breast lesions was based on analysing the difference in backscattered signals between healthy and cancerous breast tissue. Furthermore, we employed a machine learning approach, specifically utilising extreme gradient boosting (XGBOOST), to discern the presence of cancerous tissue within the breast. In tandem, a qualitative microwave imaging method was employed to precisely pinpoint the location of the tumour.
**Results or Findings:** Our dataset comprised 394 samples, with 284 originating from healthy tissue and 110 from cancerous tissue. Among the 110 cancerous cases, 69 were identified as benign and 41 as malignant findings. The devised detection model exhibited commendable performance, with a sensitivity, specificity, and accuracy of 91%, 92%, and 92%, respectively.
**Conclusion:** The findings from our study demonstrate the capability of our MBI system in detecting a significant majority of breast lesions. This suggests that SAFE holds promise in positively influencing breast cancer screening and early detection, given its non-invasive and safe characteristics. We are in the process of planning further clinical studies to validate the results obtained.
**Limitations:** The study's limitations encompass a modest sample size, potential selection bias from exclusive enrollment, reliance on a single machine learning algorithm, and the suggestion for external validation to fortify the findings.
**Ethics committee approval:** The study was approved by the Ethics committee of Marmara University School of Medicine (Protocol number: 70737436-050.06.04; Date of approval: Jun. 09, 2014). All protocols and procedures were in accordance with both institutional and national ethical standards in research and with the World Medical Association Declaration of Helsinki.
**Funding for this study:** This research was funded by the Scientific and Technology Research Council of Turkey (TUBITAK) grant number 120N388 and by the European Union's Horizon 2020 research and in-novation program under the Marie Sklodowska Curie grant agreement No. 764479.
**Author Disclosures:**
Mehmet Cayoren: Nothing to disclose
Ali Yurtseven: Nothing to disclose
Aleksandar Janjic: Nothing to disclose
Mustafa Erkin Aribal: Nothing to disclose
Onur Bugdayci: Nothing to disclose
Ibrahim Akduman: Nothing to disclose

### RPS 2405-8

**Clinical and operational benefits of artificial intelligence (AI) in prospective UK breast screening service evaluation**

*G. Lip*[1], A. Ng[2], C. F. De Vries[1], L. A. Anderson[1], R. Staff[1], G. Fox[2], C. Oberije[2], P. Kecskemethy[2]; [1]Aberdeen/UK, [2]London/UK
*(gerald.lip@nhs.scot)*

**Purpose:** The objective of this study was to assess the clinical and operational benefits of AI workflows in a live breast screening service.

**Methods or Backround:** An AI system has been evaluated in a prospective paired design at NHS Grampian to assess the impact of using AI in a combination of workflows that use AI as an: (1) Independent reader when it agrees on 'no recall' with Reader 1 (i.e. Double Reader Triage (DRT) workflow) or when it agrees on 'recall'/'no recall' with Reader 1 (i.e. Supporting Independent Reader (sIR)) to provide workload savings, and (2) Extra Reader (XR) to triage positives for additional arbitration (not recalled by standard double reading (DR)) to provide an opportunity for increased cancer detection. Over 10,000 non-opt-out women who had a four-view FFDM processed by the AI were included. All screens were human double-read, maintaining the standard of care. DRT and sIR performance outcomes were simulated, while XR outcomes were measured from live use. Planned analyses included assessment of recall rate, cancer detection rate, arbitration rate, positive predictive value, sensitivity, specificity, and workload savings for DR, DRT, sIR, and XR and combination workflows DRT+XR and sIR+XR, as well as non-inferiority and superiority tests for the combination workflows against DR.
**Results or Findings:** Interim analyses have identified that cancers from at least six women have been found through the XR process, resulting in a relative 13% (6/47) increase in cancer detection (0.9/1000 absolute) compared to DR. The sIR and DRT workflows are expected to provide significant workload savings that offset the additional arbitration workload that XR requires by 6x.
**Conclusion:** Interim analyses indicate clinical and operational benefits when using AI in breast cancer screening, which will be confirmed and presented in the final evaluation analysis (expected Q1 2024).
**Limitations:** Single-site evaluation limits this study.
**Ethics committee approval:** Ethics review not required for service evaluations
**Funding for this study:** NHSE/AAC/NIHR AI in Health and Care Award funded this study.
**Author Disclosures:**
Cary Oberije: Employee: Kheiron Medical Technologies Ltd
Roger Staff: Nothing to disclose
Gerald Lip: Nothing to disclose
Clarisse Florence De Vries: Nothing to disclose
Georgia Fox: Employee: Kheiron Medical Technologies Ltd
Peter Kecskemethy: CEO: Kheiron Medical Technologies Ltd
Annie Ng: Employee: Kheiron Medical Technologies Ltd
Lesley Ann Anderson: Other: University of Aberdeen

### RPS 2405-9

**Adding artificial intelligence (AI) case scoring in a breast screening programme to overcame delay in most probably true positive cases: a retrospective study**

*A. Nitrosi*, R. Vacondio, C. Coriani, L. Verzellesi, N. Cucurachi, C. Campari, M. Bertolini, P. Pattacini, M. Iori; Reggio Emilia/IT
*(nitrosi.andrea@ausl.re.it)*

**Purpose:** The objective of this study was to retrospectively evaluate an AI case score based strategy to anticipate the readings of most probably true positive cases to keep reading times within two weeks as would be required by local regulations.
**Methods or Backround:** We analyzed 32,012 2D mammography screening exams including 71 proven tumours and 61 pending diagnosis, consequentially acquired in Reggio Emilia Breast Screening Program (BSP) starting from October 2022 to July 2023 and elaborated by iCAD Inc. ProFound AI 2D system. ICAD Case Scores represent the AI algorithm's relative confidence that a case is malignant on a scale of 0% to 100%. A pool of nine radiologists performs double blinded plus arbitration readings; each reads about 200 mammograms per 6 hours work-shift, exams are evaluated in "virtual sessions" defined by mammograph unit and exam date. Due to a known chronic shortage of medical personnel, the readings can exceed local regulations standards of 15 days (even to more than 30). An AI-based prioritised reading protocol was elaborated analyzing the tumour incidence in function of Case Score.
**Results or Findings:** Among cases respectively having a Case Score evaluated greater than 30%, 40%, 50% and 60% the percentage of total tumours found were 89%, 85%, 69% and 61%, while the percentage of cases to be read were 20%, 13.8%, 9.8%, 5.4%. It's worth noting that prioritizing the readings of "case score based virtual session" comprehending only exams with case score >40% (4406 over 32012) per week (an average of 174 exams per week), the majority of the true positives (85%) women could be recalled within very short time.
**Conclusion:** This scenario would not undermine the reading screening workflow while guarantee early diagnosis and hopefully nor influence readers competence.
**Limitations:** Cases refer only to Reggio Emilia BSP limiting this study.
**Ethics committee approval:** Compliance with Ethical Standards Institutional Review Board approval was not required because it is a Clinical Audit about a technical development. This study was conducted in accordance with the routine quality assurance procedures established by the Local Health Authority for its screening programmes. The Reggio Emilia Cancer Registry, which routinely collects the screening history of each case of breast cancer, has been approved by the Provincial Ethic Committee.
**Funding for this study:** This study was partially supported by the Italian Ministry of Health—Ricerca Corrente.
**Author Disclosures:**
Laura Verzellesi: Nothing to disclose
Cinzia Campari: Nothing to disclose
Marco Bertolini: Nothing to disclose
Pierpaolo Pattacini: Nothing to disclose
Chiara Coriani: Nothing to disclose
Noemi Cucurachi: Nothing to disclose
Andrea Nitrosi: Nothing to disclose
Mauro Iori: Nothing to disclose
Rita Vacondio: Speaker: Invited speaker

**11:30-12:30 Research Stage 2**

### Research Presentation Session: Oncologic Imaging

### RPS 2416

### Imaging advances in oncology: from diagnosis to therapy

**Moderator**
R. Balaji; Chennai/IN
*(ravikanthbalaji@gmail.com)*

### RPS 2416-2

**An external validation study of the Birmingham atypical cartilage tumour imaging protocol for the management of solitary central cartilage tumours of the proximal humerus and around the knee**

*T. Van Den Berghe*, F. Delbare, E. Candries, M. Lejoly, C. Algoet, F. Laloo, W. Huysse, D. Creytens, K. Verstraete; Ghent/BE
*(thomasdanae@hotmail.com)*

**Purpose:** This study aimed to externally validate the Birmingham atypical cartilage tumour imaging protocol (BACTIP) recommendations for differentiation and follow-up of central cartilage tumours (CCTs) of the proximal humerus, distal femur and proximal tibia and to recommend BACTIP adaptations to reduce diagnostic delay in high-risk CCTs.
**Methods or Backround:** MRIs of 123 patients (45±11 years, 37 men) with an untreated CCT with MRI follow-up (n=62) or histopathological confirmation (n=61) were retrospectively and consecutively included and categorised following the BACTIP (2003-2020/Ghent University Hospital/Belgium). Tumour length and endosteal scalloping differences between enchondroma, atypical cartilaginous tumour (ACT) and high-grade chondrosarcoma (CS II/III/dedifferentiated) were evaluated. ROC-curve analysis for differentiating benign from malignant CCTs and for evaluating the BACTIP was performed.
**Results or Findings:** For lesion length and endosteal scalloping, ROC-AUCs were poor and fair-excellent, respectively, for differentiating between different CCT groups (0.59-0.69 versus 0.73-0.91). The diagnostic performance of endosteal scalloping and the BACTIP was higher than that of lesion length. A 1° endosteal scalloping angle cut-off differentiated the enchondroma group from the ACT/high-grade CS group with a sensitivity of 90%, reducing diagnostic delay. However, the specificity was only 29%, inducing overmedicalisation with excessive follow-up. ROC-AUC of the BACTIP was poor for differentiating enchondroma from ACT (ROC-AUC=0.69; 95%CI=0.51-0.87; p=0.041) and fair-good for differentiation between other CCT groups (ROC-AUC=0.72-0.81). BACTIP recommendations were incorrect/unsafe in five ACTs and one CS II which were all discharged from follow-up, inducing diagnostic delay. Eleven enchondromas received unnecessary referral and/or follow-up.
**Conclusion:** Although promising as a useful tool for the management and follow-up of CCTs of the proximal humerus, distal femur and proximal tibia, five ACTs and one CS grade II were discharged, inducing diagnostic delay, which could be reduced by adapting BACTIP cut-off values.
**Limitations:** No limitations were identified.
**Ethics committee approval:** This study was approved by the IRB (BC-08631).

**Funding for this study:** No funding was received for this study.
**Author Disclosures:**
Frederiek Laloo: Nothing to disclose
Koenraad Verstraete: Nothing to disclose
Maryse Lejoly: Nothing to disclose
Esther Candries: Nothing to disclose
Wouter Huysse: Nothing to disclose
Felix Delbare: Nothing to disclose
David Creytens: Nothing to disclose
Thomas Van Den Berghe: Nothing to disclose
Chloé Algoet: Nothing to disclose

### RPS 2416-3

**Evaluation of the value of (dynamic) contrast-enhanced MRI for the diagnosis and follow-up of central cartilaginous tumours**

*T. Van Den Berghe*, M. Lejoly, F. Delbare, W. Huysse, D. Creytens, G. Sys, K. Verstraete; Ghent/BE
*(thomasdanae@hotmail.com)*

**Purpose:** The objective of this study was to evaluate the value of (dynamic) contrast-enhanced MRI for the diagnosis and follow-up of central cartilaginous tumours of the proximal humerus, distal femur and proximal tibia.
**Methods or Backround:** Ninety-seven patients (44±11 years, 31 men) with a central cartilaginous tumour (histopathologically proven or more than two years follow-up (5±3 years)) were retrospectively and consecutively included. Thickness of the enhancing rim and dynamic contrast-enhanced MRI parameters were calculated. Tumour volumes were calculated for the assessment of tumour growth. Significant differences between enchondromas, atypical cartilaginous tumours, high-grade chondrosarcomas, tumours with and without growth at follow-up were searched for and ROC-curves were analysed.
**Results or Findings:** There was a significant difference (p=0.015) in enhancing rim thickness between high-grade chondrosarcomas and other diagnostic central cartilaginous tumour groups. A ROC-curve with an AUC of 0.89 and cut-off value of 1.2 mm had a sensitivity of 100% and a specificity of 64% to identify high-grade chondrosarcomas. A significant correlation was found between the relative maximal enhancement of the whole tumour on dynamic contrast-enhanced MRI and the absolute growth rate (ml/year) (ρ=0.75; p<0.001). Lesions with a relative maximal enhancement <1 compared to muscle remained stable or showed regression during follow-up (mean -0.1±0.3 ml/year). Lesions with a relative maximal enhancement between one and two showed little growth (mean +0.2±0.2 ml/year), and lesions with a relative maximal enhancement >2 had the highest growth rate (mean +0.4±0.2 ml/year).
**Conclusion:** A thick enhancing rim of more than 1.2 mm has a high sensitivity to detect high-grade chondrosarcomas. The higher the relative maximal enhancement of the whole tumour on dynamic contrast-enhanced MRI, the higher the growth rate of a central cartilaginous tumour.
**Limitations:** No limitations were identified.
**Ethics committee approval:** This study was approved by the IRB (BC-08631).
**Funding for this study:** No funding was received for this study.
**Author Disclosures:**
Koenraad Verstraete: Nothing to disclose
Maryse Lejoly: Nothing to disclose
Wouter Huysse: Nothing to disclose
Felix Delbare: Nothing to disclose
David Creytens: Nothing to disclose
Thomas Van Den Berghe: Nothing to disclose
Gwen Sys: Nothing to disclose

### RPS 2416-4

**Changes in Deauville scores on delayed imaging with total-body 18F-FDG PET/CT scans: any association with outcome?**

*Y. G. Abdelhafez*, C. Mingels, H. Nalbant, M. Rokni, F. Sen, R. Badawi, N. S. Esteghamat, J. Tuscano, L. Nardo; Sacramento, CA/US
*(yabdelhafez@ucdavis.edu)*

**Purpose:** Deauville score (DS) relies on comparing lesion uptake to reference tissues (liver and blood pool) that demonstrate tracer washout over time. We aimed to evaluate DS changes for 18F-FDG total-body PET/CT scans acquired at 2-hr compared to 1-hr post-injection and the association of these changes with clinical outcome data.
**Methods or Backround:** Forty-five patients (54.3±19.1 years; 25 females) with lymphoma (Hodgkin's lymphoma=12; non-Hodgkin's=33 [11 DLBCL, 13 follicular, and 9 marginal-zone]) underwent a total of 64 18F-FDG total-body PET/CT studies (36 staging/restaging, 22 interim, and 6 end-of-treatment [EOT] scans). All studies were acquired as dual-timepoint total-body scans at 1- and 2-hr post-injection. Changes in scan-based DSs between the two timepoints were recorded by a single radiologist blinded to clinical notes. Interim and EOT scans were evaluated for therapy response according to Lugano criteria. Follow-up data (progression and/or all-cause mortality) was collected.
**Results or Findings:** The median follow-up from diagnosis was 32.6 months. A total of 15 out of 45 patients progressed, seven of them died. One patient (2%) was upstaged from DS3 on 1-hr to DS4 on 2-hr interim PET scan. This upstaging would have changed the response category from complete to partial metabolic response. Subsequent follow-up revealed complete response.
**Conclusion:** DSs assessed on delayed 2-hr 18F-FDG total-body PET/CT scans may potentially change the response category of a small fraction (~2%) of lymphoma patients. Further validation of these initial findings in a larger cohort, with multiple readers, and stratification according to the pathologic subtypes is ongoing.
**Limitations:** Small cohort limits this study.
**Ethics committee approval:** This single-centre study was approved by the Institutional Review Board at University of California Davis (IRB#1470016) and all included subjects signed an informed consent form.
**Funding for this study:** US National Institutes of Health R01-CA249422; R01-CA206187 funded with this study.
**Author Disclosures:**
Fatma Sen: Nothing to disclose
Naseem S. Esteghamat: Nothing to disclose
Mehrad Rokni: Nothing to disclose
Joseph Tuscano: Nothing to disclose
Hande Nalbant: Nothing to disclose
Clemens Mingels: Nothing to disclose
Ramsey Badawi: Research/Grant Support: United Imaging Healthcare
Lorenzo Nardo: Research/Grant Support: United Imaging Healthcare
Yasser Gaber Abdelhafez: Nothing to disclose

### RPS 2416-5

**Relaxation-compensated CEST imaging of the APT has greater predictive value in patients with glioma at baseline before radiotherapy, compared to imaging of the APTw and ssMT at 3T**

*N. von Knebel Doeberitz*, F. Kroh, S. Graß, L. König, C. Bauspieß, M. E. Ladd, H-P. Schlemmer, A. Korzowski, D. Paech; Heidelberg/DE
*(n.knebel@dkfz-heidelberg.de)*

**Purpose:** Chemical exchange saturation transfer (CEST) MRI can be applied to use endogenous compounds as peptides and semi-solid macromolecules as imaging contrasts. However, CEST-contrasts are heavily dependent on the field strength and the applied metrics for contrast reconstruction from the Z-spectrum. Therefore, for the purpose of this study was to compare the predictive value of different CEST contrast of the amid proton transfer (APT) and semi-solid magnetization transfer (ssMT) in a larger clinical cohort of patients with glioma before radiotherapy at 3T.
**Methods or Backround:** Seventy-nine patients who had received biopsy or surgery for diffuse glioma prospectively underwent CEST imaging of the APT and ssMT at the baseline MRI before radiotherapy, applying Lorentzian-fit- and asymmetry-based reconstruction metrics first described by Zou et al. (APTw_asym), Mehrabian et al. (MTconst) and Zaiß et al. (APT_MTRRex and MT_MTRRex). Contrast-enhancing (CE) and whole tumour (WT) volumes were segmented on contrast-enhanced T1w-CE and T2w-FLAIR images. Therapy response at the first follow-up four to six weeks after the completion of radiotherapy and progression-free survival (PFS) were assessed by longitudinal follow-up according to the response assessment in neuro-oncology (RANO) criteria. Statistical testing included receiver-operator-characteristic (ROC) and Kaplan-Meier analyses.
**Results or Findings:** Imaging of the APT_MTRRex (CE: AUC=0.73, p=0.01) and MT_MTRRex (CE: AUC=0.67, p=0.05) was associated with therapy response. The APT_MTRRex (WT: HR=2.75, p<0.01) was furthermore associated with PFS. Imaging of the APTw_asym and MTconst were not associated with any outcome.
**Conclusion:** Relaxation-compensated CEST imaging of the APT has great predictive value in patients with glioma at the baseline MRI at 3T, and might help to inform clinical decision making in the future.
**Limitations:** Single centre study. Median overall survival was not reached at the time of data analysis (05/07/2023), leading to limitations.
**Ethics committee approval:** The local institutional review board committee approved this prospective study. Written informed consent was obtained from each participant prior to study inclusion.
**Funding for this study:** German Cancer Research Foundation (Grant No. 445704496) funded this study.
**Author Disclosures:**
Andreas Korzowski: Nothing to disclose
Florian Kroh: Nothing to disclose
Mark E. Ladd: CEO: Head of department
Daniel Paech: Grant Recipient: DFG-Grant Receipient that founded the study
Cora Bauspieß: Nothing to disclose
Nikolaus von Knebel Doeberitz: Author: First author
Heinz-Peter Schlemmer: CEO: Head of department
Svenja Graß: Nothing to disclose
Laila König: Nothing to disclose

### RPS 2416-6

**Therapeutic application of single-atom iron nanozymes in magnetic resonance imaging, synergistic chemodynamic therapy, and photothermal therapy in colorectal carcinoma**

*J. Zhang*, L. Wang; Shanghai/CN

**Purpose:** This study was aimed at designing a nanotherapeutic strategy that incorporates nanoscale chemodynamic therapy while integrating efficient photothermal conversion performance in colorectal cancer treatment.
**Methods or Backround:** We constructed a novel and simple Fe single-atom coordinated N-doped porous carbon nanozyme (FeCA SAzyme) at the atomic level for catalytic anti-tumour treatment coupled with photothermal therapy. The catalytic performance of FeCA SAzyme was verified by cell experiment and animal experiment.
**Results or Findings:** The FeCA SAzymes exhibited a superior catalytic kinetic Km value (4.62 mM) compared with natural catalase and other reported nanozymes. In addition, the dual-mode strategy of chemodynamic therapy in combination with photothermal therapy further increased the tumour treatment effect. Moreover, the FeCA SAzymes exhibited favourable performance in magnetic resonance imaging. The r2 value of FeCA SAzymes was approximately 149.8 mM-1S-1, which highlighting their potential as negative-contrast media for imaging purposes.
**Conclusion:** Our study shows that single-atom nanozymes can increase the Fenton reaction at the atomic level, which provides a new research direction for exploring multi-modal biological applications in tumour therapy and diagnosis.
**Limitations:** No limitations identified for this study.
**Ethics committee approval:** Fudan University Ethics committee approved this study.
**Funding for this study:** No funding was received for this study.
**Author Disclosures:**
Jun Zhang: Nothing to disclose
Lulu Wang: Nothing to disclose

### RPS 2416-7

**The value of DLSCT-based multi-parameters for assessment of VETC and MVI in hepatocellular carcinoma**

*A. Li*[1], M. Luo[1], M. Li[1], H. You[1], J. Zhou[1], W. Deng[2], X. Yu[2], Z. Zhang[2], J. Wang[1]; [1]Guangzhou/CN, [2]Shanghai/CN
*(lianqi0520@163.com)*

**Purpose:** The objective of this study was to explore the diagnostic performance of multi-parameters derived from dual-layer spectral detector CT (DLSCT) to preoperatively identify vessels encapsulating tumour clusters (VETC) and microvascular invasion (MVI) of hepatocellular carcinoma (HCC).
**Methods or Backround:** Patients with histopathology-confirmed HCC who preoperatively underwent dual-phase contrast-enhanced DLSCT were enrolled between May 2020 and June 2022 in this retrospective single-center study. Multi-parameters, including effective atomic number (Zeff), electron density (ED), iodine density (ID), and the slope of the spectral attenuation curve (λ) were obtained from DLSCT in arterial-phase (AP) and portal-venous-phase (PVP). The parameters of Zeff, ID and ED were then normalised with three different normalization approaches, N1=tumor-to-aorta ratio; N2=tumor-to-liver parenchyma; N3=[tumourPVP-tumourAP]-to-tumourAP ratio. The diagnostic performance of multi-parameters was analyzed by the area under the receiver operating characteristic curve (AUC), and was compared using the Delong test.
**Results or Findings:** There were 122 HCC patients (positive/negative VETC, n=57/65; positive/negative MVI, n=54/68). Compared with ZeffAP (AUC:0.64) and IDAP (AUC:0.64), the performance of N1-ZeffAP (AUC:0.77) and N1-IDAP (AUC:0.70) for VETC diagnosis of HCC had been significantly improved, respectively (both p<0.05), and N1-ZeffAP had very high sensitivity (98.2%). The AUCs of N1-ZeffPVP (0.68) and N1-IDPVP (0.70), N2-ZeffPVP (0.71) and N2-IDPVP (0.71) for MVI diagnosis of HCC non-significantly increased compared with ZeffPVP (0.65) and IDPVP (0.65) (all p>0.05). N1-EDPVP showed high sensitivity (88.2%) and N1-IDPVP showed high specificity (88.9%) for identifying MVI. λAP was used to diagnose VETC with good sensitivity (84.2%), and λPVP was used to diagnose MVI with good specificity (79.6%).
**Conclusion:** Multi-parameters derived from dual-phase contrast-enhanced DLSCT could be the promising biomarkers for noninvasively identifying VETC and MVI of HCC, and the tumour-to-aorta ratio is recommended for the normalization of parameters to improve the diagnostic performance.
**Limitations:** Not applicable for this study.
**Ethics committee approval:** Not applicable for this study.
**Funding for this study:** This work was supported by the National Natural Science Foundation of China grant (82271973, JW; 91959118, Jin Wang), Guangdong Basic and Applied Research Foundation (2021A1515010582, Jin Wang), Key Research and Development Program of Guangdong Province (2019B020235002, Jin Wang), China International Medical Foundation SKY Research Fund for Medical Imaging (Z-2014-07-2101 and Z-2014-07-1912-15, Jin Wang) and Clinical Research Foundation of the 3rd Affiliated Hospital of Sun Yat-Sen University (YHJH201901, Jin Wang)
**Author Disclosures:**
Xiao Yu: Nothing to disclose
Ma Luo: Nothing to disclose
Jinhui Zhou: Nothing to disclose
Jin Wang: Nothing to disclose
Zhongping Zhang: Nothing to disclose
Huayu You: Nothing to disclose
Mengsi Li: Nothing to disclose
Weiwei Deng: Nothing to disclose
Anqi Li: Nothing to disclose

### RPS 2416-8

**Node reporting and data system 1.0 (Node-RADS): validation on abdominal lymph nodes**

*E. Grimaldi*[1], F. Rigiroli[2], O. Hamam[2], B. Aslan[2], A. Brook[2], B. Siewert[2], S. Nougaret[2], O. R. Brook[2]; [1]Milan/IT, [2]Boston, MA/US

**Purpose:** The aim of the study is to validate the Node Reporting and Data System 1.0 (Node-RADS) by assigning biopsied abdominal lymph nodes to one of the Node-RADS categories and calculating malignancy rates for each.
**Methods or Backround:** This IRB-approved, HIPAA-compliant study included 505 consecutive patients who underwent CT-guided biopsy of abdominal lymph nodes between May 2016 and July 2023 at a single tertiary institution. A third-year radiology resident, blinded to pathology results, reviewed the lymph node's size and configuration on a pre-procedure contrast-enhanced CT or MRI study acquired within 30 days prior to biopsy. This resulted in a malignancy suspicion score summarized in five Node-RADS categories: "1-very low"; "2-low"; "3-equivocal"; "4-high"; "5-very high". This score was then compared to the pathology result. Clinical or imaging follow-up was used as the reference standard for inconclusive pathology results.
**Results or Findings:** Five hundred and five consecutive CT-guided abdominal lymph node biopsies were included (median age 66 years, IQR 58-75 years), of which 326/505 (65%) were retroperitoneal, 123/505 (24%) pelvic and 56/505 (11%) mesenteric lymph nodes. 10/505 (2%) lymph nodes were classified as Node-RADS 2, 61/505 (12%) Node-RADS 3, 66/505 (13%) Node-RADS 4 and 367/505 (73%) Node-RADS 5. Pathology was the reference standard in 476/505 (94%) patients, imaging follow-up in 25/505 (5%), and clinical follow-up in 4/505 (1%). Malignancy rates were 1/10 (10%, 95% CI: 0%-31%) of Node-RADS 2, 26/61 (43%, 95%CI: 30%-55%) of Node-RADS 3, 53/66 (80%, 95%CI: 71%-90%) of Node-RADS 4, and 332/367 (90%, 95% CI: 87%-93%) of Node-RADS 5.
**Conclusion:** Node-RADS categories were validated in the largest cohort of abdominal lymphadenopathies with pathology. This system can provide a numeric risk score for malignant involvement of abdominal lymph nodes.
**Limitations:** The limitation is that the inter-reader agreement is still under evaluation.
**Ethics committee approval:** The study was approved by IRB (reference number: 2018P000099).
**Funding for this study:** No funding was received for this study.
**Author Disclosures:**
Bulent Aslan: Nothing to disclose
Alexander Brook: Nothing to disclose
Omar Hamam: Nothing to disclose
Elena Grimaldi: Nothing to disclose
Bettina Siewert: Nothing to disclose
Olga Rachel Brook: Nothing to disclose
Stephanie Nougaret: Nothing to disclose
Francesca Rigiroli: Nothing to disclose

### RPS 2416-9

**Evaluation of sarcopenia as predicting factor of perioperative chemotherapy toxicity and response to treatment in patients with locally advanced gastric cancer**

*S. Nardacci*, M. Zerunian, G. Arrivi, C. Santangeli, F. Mazzuca, D. De Santis, D. Caruso, A. Laghi; Rome/IT
*(stefano.nardacci@uniroma1.it)*

**Purpose:** In the management of patients with locally advanced gastric cancer, treatment is gastrectomy with perioperative chemotherapy (p-ChT). Not all patients tolerate chemotherapy, therefore it would be useful to identify a non-invasive biomarker to predict the outcome of p-CHT. Thus, we aimed to test the role of sarcopenia in predicting p-ChT toxicity and treatment response in patients with gastric cancer.

**Methods or Backround:** Patients with advanced gastric cancer who underwent contrast-enhanced CT both before and after the p-ChT and had images available were retrospectively enrolled. Twenty-nine patients were enrolled, six excluded for CT unavailability. Skeletal muscle mass (SMM) was assessed by manually segmented specific abdominal body region on unenhanced CT at L3 lumbar vertebra level with a dedicated software (ImageJ). Skeletal muscle index was obtained by normalizing SMM for patient height in metres squared. SMI<52.4 and 38.5 cm2/m2 was considered as sarcopenic for male and female respectively. Data on haematological, gastrointestinal and neurological toxicity and response to treatment were recorded. Rank correlation and receiver operating characteristics (ROC) curve were obtained to assess sarcopenia status performance for p-Cht toxicity and response to treatment prediction; P<0.05 considered significant.
**Results or Findings:** Among the toxicities of p-Cht, sarcopenia showed significant ability to predict both haematologic and neurologic toxicity (AUC=0.929 and 0.972 respectively, all P<0.001). In addition, sarcopenia was shown to be a significant predictor of postoperative complications in the sarcopenic population (p=0.033). Gastrointestinal toxicity and response therapy did not show significant results (P>0.05).
**Conclusion:** Evaluation of sarcopenia might represent an important non-invasive imaging biomarker to predict haematologic and neurologic toxicity in patients with gastric cancer before the beginning of chemotherapy to allow a personalised treatment management.
**Limitations:** The limitations of the study are the poor population and the manual segmentation method.
**Ethics committee approval:** The study is retrospective hence, no ethical approval was required.
**Funding for this study:** No funding was received for this study.
**Author Disclosures:**
Damiano Caruso: Nothing to disclose
Curzio Santangeli: Nothing to disclose
Marta Zerunian: Nothing to disclose
Domenico De Santis: Nothing to disclose
Andrea Laghi: Nothing to disclose
Giulia Arrivi: Nothing to disclose
Federica Mazzuca: Nothing to disclose
Stefano Nardacci: Nothing to disclose

**11:30-12:30** **Research Stage 3**

### Research Presentation Session: Abdominal Viscera & GI Tract

### RPS 2401

### Artificial intelligence in abdominal imaging: current directions

**Moderator**
P. A. Bonaffini; Bergamo/IT
*(pa.bonaffini@gmail.com)*

### RPS 2401-2

**Risk stratification of gallbladder masses by ML-based ultrasound radiomics models: a prospective and multi-institutional study**
*C-K. Zhao*; Shanghai/CN
*(798434308@qq.com)*

**Purpose:** This study aimed to evaluate the diagnostic performance of machine learning (ML)–based ultrasound (US) radiomics models for risk stratification of gallbladder (GB) masses.
**Methods or Backround:** We prospectively examined 640 pathologically confirmed GB masses obtained from 640 patients between August 2019 and October 2022 at four institutions. Radiomics features were extracted from greyscale US images and germane features were selected. Subsequently, 11 ML algorithms were separately used with the selected features to construct optimum US radiomics models for risk stratification of the GB masses. Furthermore, we compared the diagnostic performance of these models with the conventional US and contrast-enhanced US (CEUS) models.
**Results or Findings:** The optimal XGBoost-based US radiomics model for discriminating neoplastic from non-neoplastic GB lesions showed higher diagnostic performance in terms of areas under the curves (AUCs) than the conventional US model (0.822–0.853 vs. 0.642–0.706, p < 0.05) and potentially decreased unnecessary cholecystectomy rate in a speculative comparison with performing cholecystectomy for lesions sized over 10 mm (2.7–13.8% vs. 53.6–64.9%, p < 0.05) in the validation and test sets. The AUCs of the XGBoost-based US radiomics model for discriminating carcinomas from benign GB lesions were higher than the conventional US model (0.904–0.979 vs. 0.706–0.766, p < 0.05). The XGBoost-US radiomics model performed better than the CEUS model in discriminating GB carcinomas (AUC: 0.995 vs. 0.902, p = 0.011).
**Conclusion:** The proposed ML-based US radiomics models possess the potential capacity for risk stratification of GB masses and may reduce the unnecessary cholecystectomy rate and use of CEUS.
**Limitations:** First, the sample size was relatively limited. Second, since surgery-confirmed pathology was used as the gold standard, GB lesions sized less than 6 mm were not included, which are commonly not recommended for cholecystectomy.
**Ethics committee approval:** This study was approved by the ethics committee of the institution (No: SHSYIEC–4.1/21–263/01; 2022-187R).
**Funding for this study:** This work was supported in part by the National Natural Science Foundation of China (Grant 82202174).
**Author Disclosures:**
Chong-Ke Zhao: Nothing to disclose

### RPS 2401-3

**Development and validation of comprehensive nomogram based on imaging features and MRI radiomics to predict microvascular invasion and overall survival in patients with intrahepatic cholangiocarcinoma**
*G. Miao*, X. Qian, Y. Zhang, C. Yang, M. Zeng; Shanghai/CN
*(miaogengyun@163.com)*

**Purpose:** Microvascular invasion (MVI) is a predictor of poor prognosis in intrahepatic cholangiocarcinoma (ICC). The aim of this study was to establish a comprehensive model based on MR radiomics for MVI status stratification and overall survival prediction in ICC patients preoperatively.
**Methods or Backround:** A total of 249 ICC patients were randomized into training and validation cohorts (174:75), and a time-independent test cohort with 47 ICC patients was enrolled. Independent clinical and imaging predictors were identified by univariate and multivariate logistic regression analyses. The radiomic model was based on the robust radiomic features extracted by a logistic regression classifier and the least absolute shrinkage and selection operator algorithm. The imaging-radiomics (IR) model integrated the independent predictors and robust radiomics features. The predictive efficacy of the models was evaluated by receiver operating characteristic curves, calibration curves and decision curves. Multivariate Cox analysis identified the independent risk factors for overall survival, Kaplan–Meier curves were plotted, and a nomogram was used to visualize the predictive model.
**Results or Findings:** The imaging model comprised tumour size and intrahepatic duct dilatation. The radiomics model comprises 25 stable radiomics features. The IR model shows desirable performance (AUCtraining= 0.890, AUCvalidation= 0.885 and AUCtest= 0.815). The calibration curve and decision curve validate the clinical utility. Overall survival predicted by histological and IR model-predicted MVI groups exhibited similar predictive efficacy.
**Conclusion:** The IR model and nomogram based on IR model-predicted MVI status may be a potential tool in MVI status stratification and overall survival prediction of ICC patients preoperatively.
**Limitations:** The models are based on retrospectively collected data from a single institution.
**Ethics committee approval:** Approval for this retrospective study was granted by the Ethics Committee of our Hospital.
**Funding for this study:** This work was supported by 1. Shanghai Municipal Health Commission (Grant number 202240152); 2. National Natural Science Foundation of China (Grant number 82171897); 3. Shanghai Municipal Key Clinical Specialty (Grant number shslczdzk03202); 4. Clinical Research Plan of SHDC (Grant number SHDC2020CR1029B).
**Author Disclosures:**
Mengsu Zeng: Nothing to disclose
Gengyun Miao: Nothing to disclose
Chun Yang: Nothing to disclose
Xianling Qian: Nothing to disclose
Yunfei Zhang: Nothing to disclose

### RPS 2401-4

**Automatic detection and segmentation of IPMN pancreatic cysts in MRI with a multi-sequence cascaded deep learning pipeline**
N. Mazor, N. Lev-Cohain, R. Lederman, J. Sosna, *L. Joskowicz*; Jerusalem/IL

**Purpose:** Radiological detection and follow-up of IPMN pancreatic cysts in multi-sequence MRI studies is required to assess their malignancy potential. The evaluation requires expertise and is not automated. This study evaluates a novel multi-sequence cascaded deep learning pipeline for the detection and segmentation of IPMN pancreatic cysts in abdominal MRI.
**Methods or Backround:** The pipeline consists of three steps: (1) pancreas Region of Interest (ROI) segmentation in the axial MRI TSE; (2) transfer and masking of the computed pancreas ROI to the coronal MRI MRCP; (3) detection and segmentation of cysts in the masked MRCP. Both steps 1 and 3 use 3D U-Net models with Hard Negative Patch Mining, a new technique for class imbalance correction and reduction of false positives. The pipeline was evaluated on 158 MRI patient studies of patients with pancreatic cysts undergoing follow-up. The training/validation/testing sets split was 118/17/23. Ground truth segmentations of a total of 840 cysts were manually obtained by an expert radiologist: Six hundred and nineteen cysts were >5 mm, 221 cysts were >10 mm with a mean number of cysts/scan of 5.3±2.6 and mean cyst diameter (volume) of 7.4 mm (0.91cc). The computed test set results were then compared to their respective manual ground truth delineations.
**Results or Findings:** The pipeline achieved mean recall of 0.80±0.19 and 0.99±0.05, precision of 0.75±0.26, and 0.95±0.16, and dice score of 0.80±0.19 and and 0.81±0.11 for pancreatic cysts of diameter > 5 mm and > 10mm respectively, which is the clinically relevant endpoint. The cyst inclusion in the pancreas ROI Recall is 0.94±0.22 and 0.98±0.07, respectively.
**Conclusion:** Automatic pancreatic cyst detection and segmentation in multi-sequence MRI may provide an accurate and reliable method for precise disease evaluation and save time.
**Limitations:** The major limitation of this study was this was of single observer annotation, one centre.
**Ethics committee approval:** No ethical approval was obtained for this study.
**Funding for this study:** No funding was obtained for this study.
**Author Disclosures:**
Richard Lederman: Consultant: HighRAD Ltd
Leo Joskowicz: Founder: HighRAD Ltd
Jacob Sosna: Founder: HighRAD Ltd
Nir Mazor: Nothing to disclose
Naama Lev-Cohain: Nothing to disclose

### RPS 2401-5

### Delta CT-radiomics derived response prediction in advanced pancreatic ductal adenocarcinoma

*F. N. Harder*[1], G. O'Kane[2], E. Salinas-Miranda[2], K. Lajkosz[2], A. Farooq[2], S. Gallinger[2], J. Knox[2], M. Haider[2]; [1]Munich/DE, [2]Toronto, ON/CA

**Purpose:** The aim of this study was to evaluate multi-time point kinetic delta radiomics for response and overall survival (OS) prediction in advanced PDAC.
**Methods or Backround:** One hundred and fifty-seven patients with advanced PDAC (108/157 with synchronous liver metastases) were retrospectively enrolled serving as the training cohort. Twenty-eight patients with metastatic PDAC from a second prospective study served as an external validation cohort. All patients received mFOLFIRINOX or gemcitabine/nab-paclitaxel as first-line chemotherapy. Baseline CT-models and delta radiomics models reflecting the kinetic between baseline and first follow-up CT were build based on size-related, non-size and combined size and non-size features from the primary tumour and largest liver metastasis to predict progression under chemotherapy based on RECIST1.1 and OS at 9 months in the overall cohort and liver metastases subgroup. Baseline and delta radiomics models were compared against each other and established Moffitt RNA-signature.
**Results or Findings:** Non-size and combined delta-radiomics models significantly discriminated between responders (complete/partial response and stable disease) and non-responders (progressive disease) in the training cohort and external validation cohort (AUC 0.714-0.873, p = < 0.001-0.01) outperforming baseline-only models (AUC 0.55-0.645) and Moffitt RNA signature (0.551-0.675). Radiomics models represented an independent survival predictor at 9 months in the training and the external validation cohort, with non-size models yielding the highest AUC in the training cohort, yet not significantly outperforming Moffitt RNA signature (training: 0.726 vs 0.588, p = 0.16; validation: 0.713 vs. 0.567, p = 0.35).
**Conclusion:** Delta-radiomics models outperformed baseline-models and Moffitt RNA signature as predictive biomarkers for response and OS prediction in advanced PDAC. In particular non-size feature models from the primary tumour and the largest liver metastasis provided additive value.
**Limitations:** Although including the largest cohort for radiomics-based response prediction in advanced PDAC so far, further studies need to validate the herein-found results.
**Ethics committee approval:** This study was approved by our institutional research ethics board and informed consent was obtained.
**Funding for this study:** Funding for this study was provided by the Ontario Institute of Cancer Research, The Sinai Health Foundation and University Medical Imaging Toronto. F.N.H. received funding from Deutsche Forschungsgemeinschaft, HA 9949/1-1.
**Author Disclosures:**
Grainne O'Kane: Nothing to disclose
Jennifer Knox: Nothing to disclose
Katherine Lajkosz: Nothing to disclose
Abdul Farooq: Nothing to disclose
Steven Gallinger: Nothing to disclose
Masoom Haider: Nothing to disclose
Felix N. Harder: Nothing to disclose
Emmanuel Salinas-Miranda: Nothing to disclose

### RPS 2401-6

### Radiomic feature profiles to define treatment response in rectal cancer: not just a tumour matter

*A. Marhuenda*[1], A. Nogue[1], M. Domingo Pomar[1], I. Machado[1], R. Garcia Figueiras[2], F. Bellvis[1], A. Fuster Matanzo[1], A. Jimenez-Pastor[1], A. Alberich-Bayarri[1]; [1]Valencia/ES, [2]Santiago de Compostela/ES
*(amarflu@gmail.com)*

**Purpose:** In rectal cancer (RC), predicting response to specific treatments is essential for defining appropriate therapeutic strategies. Moreover, the involvement of peritumoral regions and/or the presence of certain histopathological conditions are associated with poor outcomes. The project aimed to assess whether radiomics may help stratify patients at baseline based on treatment response and involvement of peritumoural regions.
**Methods or Backround:** A retrospective, single-centre study was conducted. Baseline T2W MRIs of RC patients receiving neoadjuvant treatment (NT) and surgery were included. Manual delineation of seven labels was performed: tumour, extramural venous invasion (EMVI), tumoural deposits (TD), lymph nodes (LN) including intra- and extra- mesorectum nodes, peritumoural wall (PW), mesorectum fat, and presacral space (PS). Radiomic features were extracted for each segmentation, and four models were evaluated: tumour [model 1], tumour surroundings (EMVI, TD, LN, and mesorectum) [model 2], model 1 plus model 2 [model 3], and model 3 with PW and PS [model 4]. Univariate and multivariate (logistic regression) analyses were performed.
**Results or Findings:** A total of 50 RC patients who received any type of NT and underwent surgery were included. In the univariate analysis, the greatest differences between responders and non-responders were found in model 4. Statistical differences (p < 0.05) were noted in four radiomic features—Kurtosis, GLRLM Run Entropy, NGTDM_Busyness and NGTDM_Strength. In the multivariate analysis, model 4 outperformed the other models, with an AUC of 0.787.
**Conclusion:** Radiomic features could assist oncologists in therapeutic decision-making by predicting treatment responses. Segmentations including tumour and peritumoral regions provide more solid results. This highlights the relevance of a more holistic approach that would simplify segmentation´s tasks. Further studies with larger sample sizes are required.
**Limitations:** The limitations of the study are basically focused on the reduced number of patients.
**Ethics committee approval:** The study was approved by 2018-27.
**Funding for this study:** No funding was provided for this study.
**Author Disclosures:**
Ana Marhuenda: Nothing to disclose
Almudena Fuster Matanzo: Nothing to disclose
Ana Jimenez-Pastor: Nothing to disclose
Anna Nogue: Nothing to disclose
Angel Alberich-Bayarri: Shareholder: Shareholder at Quibim CEO: CEO at Quibim
Roberto Garcia Figueiras: Nothing to disclose
Marcos Domingo Pomar: Nothing to disclose
Isidro Machado: Nothing to disclose
Fuensanta Bellvis: Nothing to disclose

### RPS 2401-7

### Effect of artificial intelligence-aided differentiation of benign and premalignant colorectal polyps as a second reader at CT colonography

*S. Grosu*, M. P. Fabritius, M. Winkelmann, S. Maurus, A. Graser, J. Ricke, P. M. Kazmierczak, M. Ingrisch, P. Wesp; Munich/DE

**Purpose:** Premalignant adenomatous colorectal polyps require endoscopic resection, as opposed to benign hyperplastic colorectal polyps. The aim of this study was to evaluate the effect of artificial intelligence (AI)-assisted differentiation of benign and premalignant colorectal polyps as a second reader for general radiologists at CT colonography.
**Methods or Backround:** CT colonography images with colorectal polyps of all sizes and morphologies were retrospectively evaluated by three independent board-certified radiologists with moderate experience in CT colonography. The readers' task was to decide whether the depicted polyps required endoscopic resection. After a primary unassisted read, a second read with access to the classification of a radiomics-based random forest AI model labelling each polyp as “adenomatous” or “hyperplastic” was performed. No polyp used for training the AI model was included in this study. The performance of the unassisted and AI-assisted reading was evaluated using polyp histopathology as the reference standard.
**Results or Findings:** Seventy-seven polyps in 59 patients comprising 118 polyp image series (47% supine position, 53% prone position) were evaluated unassisted and AI-assisted by three radiologists, resulting in a total of 708 readings (subsequent polypectomy: yes or no). Compared with unassisted reading, the AI-assisted reading had a significantly higher accuracy (76% +/- 1% vs. 84% +/- 1%, p < 0.001), sensitivity (76% +/- 2% vs. 85% +/- 0%, p < 0.001), and specificity (75% +/- 1% vs. 81% +/- 2%, p < 0.001) in selecting polyps eligible for polypectomy.

**Conclusion:** In this proof-of-concept study, AI-based characterisation of colorectal polyps at CT colonography as a second reader enabled a more precise selection of polyps eligible for subsequent endoscopic resection.
**Limitations:** The limitation of this study is the rather small sample size.
**Ethics committee approval:** This study was approved by the ethics committe of the Ludwig-Maximilians University, Munich (18-401).
**Funding for this study:** No funding was received for this study.
**Author Disclosures:**
Anno Graser: Nothing to disclose
Matthias Philipp Fabritius: Nothing to disclose
Philipp M. Kazmierczak: Nothing to disclose
Sergio Grosu: Nothing to disclose
Stefan Maurus: Nothing to disclose
Michael Winkelmann: Nothing to disclose
Michael Ingrisch: Nothing to disclose
Philipp Wesp: Nothing to disclose
Jens Ricke: Nothing to disclose

### RPS 2401-8

**A radiomics model for the preoperative prediction of lymph node metastasis in colorectal carcinoma**

*A. Salgado-Parente*[1], L. González Campo[1], A. M. Vera Carmona[1], A. Martínez Caballero[1], I. De Vicente Bernal[1], L. D. Juez Saez[1], P. Abadía Barnó[1], A. Torrado-Carvajal[2], J. Blazquez Sanchez[1]; [1]Madrid/ES, [2]Mostoles/ES
*(alba.salgado@salud.madrid.org)*

**Purpose:** The aim of this study was to develop and validate a radiomics nomogram for the preoperative prediction of lymph node (LN) metastasis in colorectal carcinoma (CRC).
**Methods or Backround:** The prediction model was developed in a primary cohort that consisted of 110 patients with clinicopathologically confirmed CRC with data collected from January 2013 to September 2017 in a single institution. The patients were divided into a training set (n = 88) and a validation set (n = 22) with statistically comparable demographic features. Radiomics features of the primary tumor and lymph node were extracted from portal venous phase CT images of each patient. Mutual information was used on the whole dataset for feature selection. The following models were trained: Random Forest, Logistic Regression, Naive Bayes, Gaussian Process, Support Vector Machine, MultiLayer Perceptron, K-Nearest Neighbors, Gradient Boosting, Neural Network. A crossvalidated fine-tuning of the hyperparameters was performed on each model to enhance the overall performance. A majority voting approach was followed assessing the different combinations of the individual classifiers. The combinations with the best F1 score performance were then selected to present the results.
**Results or Findings:** The radiomics signature demonstrated favorable discriminatory ability in predicting lymph node involvement based on both tumor segmentation (ROC AUC 0.88, sensitivity 1, specificity 0.73) and lymph node segmentation (ROC AUC 0.94, sensitivity 1, specificity 0.83). Accuracy was highly discriminative with values of 0.86 for tumor-based segmentations and 0.91 for lymph node-based segmentations.
**Conclusion:** The CT-based radiomics nomogram has the potential to be used as a non-invasive tool for individualised preoperative prediction of LN metastasis in CRC. External validation is further required prior to clinical implementation.
**Limitations:** The major limitations were that this was a single-centre and retrospective analysis.
**Ethics committee approval:** Not applicable for this study.
**Funding for this study:** No funding was received for this study.
**Author Disclosures:**
Alberto Martínez Caballero: Nothing to disclose
Alba Salgado-Parente: Nothing to disclose
Luz Divina Juez Saez: Nothing to disclose
Ana María Vera Carmona: Nothing to disclose
Luis González Campo: Nothing to disclose
Pedro Abadía Barnó: Nothing to disclose
Javier Blazquez Sanchez: Nothing to disclose
Irene De Vicente Bernal: Nothing to disclose
Angel Torrado-Carvajal: Nothing to disclose

### RPS 2401-9

**Development and validation of CT-based radiomics deep-learning signatures to preoperatively predict lymph node metastasis in non-functional pancreatic neuroendocrine tumour: a multi-cohort study**

*W. Tang*; Shanghai/CN
*(tangwei12@fudan.edu.cn)*

**Purpose:** Lymph node status is an important factor for the patients with non-functional pancreatic neuroendocrine tumours (NF-PanNETs) with respect to surgical methods, prognosis, recurrence. Our aim is to develop and validate a combination model based on contrast-enhanced CT images to preoperatively predict the lymph node metastasis (LNM) in NF-PanNETs.
**Methods or Backround:** Retrospective data were gathered for 320 patients with NF-PanNETs who underwent curative pancreatic resection and CT imaging at two institutions (Centre 1, n= 236 and Centre 2, n=84) between January 2010 and March 2022. RDPs (Radiomics deep learning signature) were developed based on ten machine-learning techniques. These signatures were integrated with the clinicopathological factors into a nomogram for clinical applications. The evaluation of the model's performance was conducted through the metrics of the area under the curve (AUC).
**Results or Findings:** The RDP signature showed excellent performance in both centres with a high AUC for predicting LNM and DFS in centre 1 (AUC, 0.88; 95% CI: 0.84, 0.92; DFS, p <.05) and centre 2 (AUC, 0.91; 95% CI: 0.85, 0.97; DFS, p <.05). The clinical factors of vascular invasion, perineural invasion, and tumour grade were associated with LNM (p <.05). The combination nomogram showed better prediction capability for LNM (AUC, 0.93; 95% CI: 0.89, 0.96). Notably, our model maintained a satisfactory predictive ability for tumours at the 2-cm threshold, demonstrating its effectiveness across different tumour sizes in centre 1 (≤2 cm: AUC, 0.90 and >2 cm: AUC, 0.86) and centre 2 (≤2 cm: AUC, 0.93 and >2 cm: AUC, 0.91).
**Conclusion:** Our RDPs may have the potential to preoperatively predict LNM in NF-PanNETs, address the insufficiency of clinical guidelines concerning the 2-cm threshold for tumour lymph node dissection, and provide precise therapeutic strategies.
**Limitations:** Small sample size was a limitation of this study.
**Ethics committee approval:** This two-centre retrospective study received approval from the Research Ethics Committee of the Institutional Review Boards from all participating hospitals, and the need for informed consent was exempted.
**Funding for this study:** This work was supported by Project of Shanghai Municipal Health Commission (202340123) and The Rare Tumour Research Special Project of the National Natural Science Foundation of China (82141104).
**Author Disclosures:**
Wei Tang: Nothing to disclose

**11:30-12:30** **Research Stage 4**

### Research Presentation Session: Cardiac

### RPS 2403

### Spectral CT: new developments and clinical applications (part 2)

**Moderator**
M. Halfmann; Mainz/DE
*(moritz.halfmann@unimedizin-mainz.de)*

### RPS 2403-2

**Coronary artery calcium scoring using virtual vs true non-contrast images from photon-counting coronary CT angiography**

N. Haag[1], A. E. Michael[1], S. Lennartz[2], C. Panknin[3], I. Shahzadi[3], J. H. Niehoff[1], J. Borggrefe[1], *J. R. Kröger*[1]; [1]Minden/DE, [2]Cologne/DE, [3]Forchheim/DE
*(janrobertkroeger@gmail.com)*

**Purpose:** Coronary artery calcium scoring (CACS) requires true non-contrast (TNC) CT. Photon-counting CT provides an algorithm (PureCalcium) for reconstructing virtual non-contrast images from contrast-enhanced coronary CT angiography (CCTA) specifically for CACS. The aim of this study was to assess the CACS differences based on PureCalcium images derived from contrast-enhanced CCTA compared with TNC images and evaluate the impact of these differences on the classification of patients into plaque burden categories.
**Methods or Backround:** Images from patients who underwent photon-counting CCTA between August 2022 and May 2023 were identified and retrospectively analysed. Agatston scores were derived from TNC and PureCalcium images and tested for agreement using the intra-class correlation coefficient. Patients were categorised into different plaque burden groups based on Agatston scores and agreement was evaluated using weighted Cohen’s kappa. Wilcoxon signed-rank test and Bland-Altman analysis were used to evaluate differences, the DLP for the entire examination and TNC were analysed.
**Results or Findings:** Median Agatston scores were comparable between TNC and PureCalcium (4.8; IQR= 83.6 [0.0 – 2151.8] vs 2.7; IQR =89.2 [0.0-2377.1], P=.99) and showed a strong agreement and relationship (ICC = 0.98, 95% CI [0.97, 0.99]. On Bland-Altman analysis only two patients showed a bias outside of the limits of agreement. With PureCalcium CACS, 74% of patients were correctly classified into plaque burden groups agreement excellent

(κ = 0.88). TNC contributed a mean of 19.7 ± 8.8% SD of the radiation dose of the entire examination.
**Conclusion:** Agatston scores for coronary artery disease derived from PureCalcium and TNC images from contrast-enhanced photon counting CCTA high agreement and did not differ significantly. PureCalcium CACS correctly classified most patients into plaque burden groups. Omitting TNC scans reduced the radiation dose.
**Limitations:** Distribution of Agatston scores was skewed and lower than expected in our study cohort.
**Ethics committee approval:** This study was approved by the local ethics committee as a retrospective study
**Funding for this study:** No funding was received for this study.
**Author Disclosures:**
Simon Lennartz: Speaker: Amboss
Julius Henning Niehoff: Nothing to disclose
Christoph Panknin: Employee: Siemens Healthineers
Nina Haag: Nothing to disclose
Jan Borggrefe: Speaker: Stryker; Speaker: Siemens Healthineers; Speaker: Philips Healthcare
Jan Robert Kröger: Advisory Board: Siemens Healthineers; Speaker: GE Healthcare
Arwed E. Michael: Nothing to disclose
Iram Shahzadi: Employee: Siemens Healthineers

### RPS 2403-3

**In vitro study of the quality of multi-parameter coronary stent images on second-generation dual-layer detector spectral CT**
*L. Chen*, Z. Jian; Xi An/CN

**Purpose:** The aim of this study was to evaluate the image quality of the multi-parameter coronary stent images on second-generation dual-layer detector spectral CT.
**Methods or Backround:** Eighteen coronary stents of different materials and diameters were placed in simulated coronary vessels containing a concentration of 11 mg/L iodine contrast agent. Scanning was performed using second generation dual-layer detector spectral CT. 50~190 keV monoenergetic (20 keV interval), conventional, and iodine map images were reconstructed. Stent lumen score, stent structure score, and degree of blooming artefact were subjectively evaluated (Likert 4-score). Stent lumen contrast noise ratios (CNRs), non-stent lumen contrast noise ratios (CNRc), inner diameter differences (IDD), and blooming artifact index (BAI) were used for objective evaluation. The stents were divided into small diameter subgroups (<3 mm) and large diameter subgroups (≥3 mm) according to their diameters. Repeated-measure ANOVA and Tukey's test were used for multi-comparison.
**Results or Findings:** For all stents and the large diameter subgroup, the optimal monoenergetic images had the highest stent lumen score, which was significantly greater than those of iodine map images. The iodine map images had the highest stent structure score, which was significantly greater than those of iodine maps (for all stents and small diameter subgroup) and conventional images (for all stents and both subgroups). CNRs and CNRc decreased with increasing mono-energy level. For all stents and both subgroups, the CNRs and CNRc of optimal monoenergetic images were greater than for conventional and iodine map images. For all stents, iodine map images had the smallest IDD and BAI.
**Conclusion:** Monoenergetic imaging on the second-generation dual-layer detector spectral CT has advantages in assessing stent lumen and improving CNR. Meanwhile, iodine map imaging has advantages in assessing stent structure and internal diameter.
Keywords: Stent; Computed tomography; Coronary vessels
**Limitations:** The main limitation was that this was a phantom study.
**Ethics committee approval:** No ethical clearance was sought for this study.
**Funding for this study:** No funding was obtained for this study.
**Author Disclosures:**
Zhijie Jian: Nothing to disclose
Lihong Chen: Nothing to disclose

### RPS 2403-4

**Ultra-high resolution coronary CT angiography on photon-counting detector CT: impact of quantum iterative reconstruction**
*M. Vecsey-Nagy*[1], G. Tremamunno[1], U. J. Schoepf[1], N. Fink[1], E. Zsarnóczay[1], P. Maurovich-Horvat[2], B. Szilveszter[2], A. Varga-Szemes[1], T. S. Emrich[1]; [1]Charleston, SC/US, [2]Budapest/HU

**Purpose:** The objective of this study was to assess the impact of different quantum iterative reconstruction (QIR) levels on objective and subjective image quality of ultra-high resolution (UHR) coronary CT angiography (CCTA) images and to determine the accuracy of stenosis quantification using photon-counting detector CT (PCD-CT) .
**Methods or Backround:** A dynamic vessel phantom containing two calcified lesions (25% and 50% stenosis) was scanned at 60, 80 and 100 beats per minute with a PCD-CT system. In vivo examinations were performed in 102 CCTA patients. All scans were acquired in UHR mode (slice thickness: 0.2 mm, kernel: bv64) and reconstructed with four different QIR levels (1-4). Image noise, signal-to-noise ratio (SNR), sharpness, and percent diameter stenosis (PDS) were quantified on the phantom, while subjective quality metrics (graininess, sharpness, overall image quality) were assessed on clinical scans.
**Results or Findings:** Increasing QIR levels resulted in significantly lower objective image noise and higher SNR (p<0.001), while sharpness and PDS values did not differ significantly between QIRs (all pairwise p>0.013). Subjective graininess of in vivo images significantly decreased with increasing QIR levels, with significantly higher image quality scores for each stepwise increase (all pairwise p<0.001). Qualitative sharpness, on the other hand, did not differ across different levels of QIR (p=0.15).
**Conclusion:** The novel QIR algorithm may enhance image quality of CCTA datasets without compromising image sharpness or accurate stenosis measurements, with the most prominent benefits at the highest strength level.
**Limitations:** The subjective aspect of the qualitative analysis introduces the possibility of bias into the study. Moreover, although potential usefulness of QIR in reducing radiation dose may be proposed, we have not yet examined methods of radiation dose reduction with QIR.
**Ethics committee approval:** The protocol for this prospective, HIPAA-compliant study was approved by the local Institutional Review Board (#6030).
**Funding for this study:** No funding was received for this study.
**Author Disclosures:**
Emese Zsarnóczay: Nothing to disclose
Nicola Fink: Nothing to disclose
Pál Maurovich-Horvat: Nothing to disclose
Milán Vecsey-Nagy: Nothing to disclose
Uwe Joseph Schoepf: Nothing to disclose
Giuseppe Tremamunno: Nothing to disclose
Bálint Szilveszter: Nothing to disclose
Tilman Stephan Emrich: Nothing to disclose
Akos Varga-Szemes: Nothing to disclose

### RPS 2403-5

**Feasibility of coronary CT angiography-based fractional flow reserve using a clinical photon-counting detector CT system**
*E. Zsarnóczay*[1], D. Pinos[2], U. J. Schoepf[2], N. Fink[2], J. O'Doherty[2], M. Vecsey-Nagy[2], P. Maurovich-Horvat[1], T. S. Emrich[2], A. Varga-Szemes[2]; [1]Budapest/HU, [2]Charleston, SC/US

**Purpose:** The aim of this study was to intra-individually compare CT-FFR between photon-counting detector (PCD) and conventional energy-integrating detector (EID) CT systems.
**Methods or Backround:** In this single-centre prospective study, adults who underwent CCTA on an EID-CT system were recruited for research PCD-CT scans between July 2021 to March 2022. EID-CT images were reconstructed (Bv36 kernel, iterative reconstruction level 3, 0.5 mm slice thickness). PCD-CT images were post-processed with settings matched to EID-CT as close as possible (Bv36 kernel, quantum iterative reconstruction level 3, virtual mono-energetic level 55 keV, 0.6 mm slice thickness). CT-FFR was obtained semi-automatically using a prototype on-site machine learning algorithm by two readers. The lowest CT-FFR value was used for the per-patient analysis and a CT-FFR ≤0.75 was considered haemodynamically significant. Correlation and reliability between CT-FFREID-CT and CT-FFRPCD-CT were assessed with Spearman (r) and intraclass correlation coefficients (ICC).
**Results or Findings:** A total of 22 patients (63.3±9.2 years; 7 women) were included. The median time between EID-CT and PCD-CT was 5.5 days. Comparison of CT-FFR values showed no significant difference and strong agreement between EID-CT and PCD-CT in the per-vessel analysis (0.88 [0.74-0.94] vs. 0.87 [0.76-0.93], P=0.096, mean bias 0.02, limits of agreement [LoA] -0.14/0.19, r=0.83, ICC=0.92), and in the per-patient analysis (0.81 [0.60-0.86] vs. 0.76 [0.64-0.86], P=0.768, mean bias 0.02, LoA -0.15/0.19, r=0.90, ICC=0.93). All included patients were classified into the same category (CT-FFR>0.75 vs ≤0.75) with both CT systems.
**Conclusion:** PCD-CT-based CT-FFR evaluation is feasible and correlates well with EID-CT-based CT-FFR.
**Limitations:** CT-FFR values from EID- and PCD-CT were not compared with invasively measured FFR. Only one reconstruction setting was used for image postprocessing with EID- and PCD-CT.
**Ethics committee approval:** The protocol of this prospective, Health Insurance Portability and Accountability Act–compliant, single-centre study was approved by the local Institutional Review Board, and written informed consent was obtained from each participant.
**Funding for this study:** This research was partially funded by a grant from Siemens Healthineers.

**Author Disclosures:**
Emese Zsarnóczay: Nothing to disclose
Daniel Pinos: Nothing to disclose
Nicola Fink: Nothing to disclose
Pál Maurovich-Horvat: Nothing to disclose
Milán Vecsey-Nagy: Nothing to disclose
Jim O'Doherty: Employee: Siemens Medical Solutions USA Inc
Uwe Joseph Schoepf: Nothing to disclose
Tilman Stephan Emrich: Speaker: Siemens Medical Solutions USA Inc
Akos Varga-Szemes: Nothing to disclose

### RPS 2403-6

**Impact of reconstruction parameters on the accuracy of myocardial extracellular volume quantification on a first-generation photon-counting detector CT**

*C. Gnasso*[1], D. Pinos[2], U. J. Schoepf[2], M. Vecsey-Nagy[2], E. Zsarnóczay[2], N. Fink[2], R. J. Holtackers[3], A. Varga-Szemes[2], T. S. Emrich[2]; [1]Milan/IT, [2]Charleston, SC/US, [3]Maastricht/NL
*(gnasso.chiara@hsr.it)*

**Purpose:** The potential role of cardiac CT has increasingly been demonstrated for the assessment of diffuse myocardial fibrosis through the quantification of extracellular volume (ECV). The recently introduced photon-counting detector (PCD)-CT technology may deliver more accurate ECV quantification compared to energy-integrating detector CT. This study aimed to establish the image reconstruction settings for PCD-CT delayed acquisitions yielding the highest agreement in ECV quantification compared to MRI-ECV.
**Methods or Backround:** Patients (n=27, 53.1±17.2 years; 14 women) prospectively underwent same-day cardiac MRI and PCD-CT. Delayed CT scans were reconstructed with different quantum iterative reconstruction levels (QIR 1-4), slice thicknesses (s.t. 0.4-8 mm), and virtual monoenergetic imaging levels (VMI, 40-90 keV), as shown in Figure 1. ECV was quantified for each reconstruction setting and compared using ANOVA for repeated measures and t-test for pairwise comparisons. The agreement with MRI was assessed with Bland-Altman plots and Lin's concordance correlation coefficient (CCC).
**Results or Findings:** ECV values didn't differ significantly among QIR levels (p=1.00), whereas a significant difference was observed throughout different slice thicknesses, with 0.4 mm yielding the highest agreement (CCC 0.944). 45 keV VMI reconstructions had the lowest mean bias (0.6, 95%CI 0.1–1.4) compared to MRI. Using the most optimal reconstruction settings (QIR4; s.t. 0.4 mm; VMI 45 keV), a 63% reduction in mean bias and a 6% increase in concordance with MRI were achieved compared to the standard settings used in previous publications (QIR3; s.t. 1.5 mm; VMI 65 keV).
**Conclusion:** The appropriate selection of CT reconstruction parameters improves the agreement between PCD-CT and MRI ECV values.
**Limitations:** Our study has the following limitations: first, it is a single-centre study with a relatively small sample size; second, the lack of a histological reference, and third, the use of MRI-derived synthetic Hct.
**Ethics committee approval:** The study is HIPAA-compliant.
**Funding for this study:** This study was supported by an institutional research grant from Siemens Healthcare.
**Author Disclosures:**
Emese Zsarnóczay: Nothing to disclose
Daniel Pinos: Nothing to disclose
Nicola Fink: Nothing to disclose
Milán Vecsey-Nagy: Nothing to disclose
Uwe Joseph Schoepf: Other: Institutional research support and/or personal fees from Bayer, Elucid Bioimaging, Bracco, Guerbet, HeartFlow and Siemens
Robert J. Holtackers: Nothing to disclose
Tilman Stephan Emrich: Other: Speaker fee and travel support from Siemens
Chiara Gnasso: Nothing to disclose
Akos Varga-Szemes: Other: Receives institutional research support and/or personal fees from Elucid BioImaging and Siemens

### RPS 2403-7

**Impact of photon-counting CT-based virtual mono-energetic imaging on detecting myocardial late iodine enhancement: comparison with MRI reference**

*C. Gnasso*[1], M. Vecsey-Nagy[2], U. J. Schoepf[2], D. Pinos[2], E. Zsarnóczay[2], G. Tremamunno[2], R. J. Holtackers[3], T. S. Emrich[4], A. Varga-Szemes[2]; [1]Milan/IT, [2]Charleston, SC/US, [3]Maastricht/NL, [4]Frankfurt/DE
*(gnasso.chiara@hsr.it)*

**Purpose:** Cardiac CT has the ability to detect myocardial scar, which can potentially be improved by the use of the recently-introduced photon-counting detector (PCD)-CT technology. This study aims to investigate the image quality and diagnostic performance of late iodine enhancement (LIE)-CT scans at different virtual monoenergetic image (VMI) levels in detecting and characterising myocardial scars, using late gadolinium enhancement (LGE)-MRI as the reference standard.
**Methods or Backround:** This is a post-hoc analysis of a prospective research study including patients with various cardiomyopathies who underwent clinical LGE-MRI and same-day research LIE-CT scan between July 2021 and January 2022. LIE PCD-CT scans were reconstructed at different VMI levels (40, 45, 50, 60, 70, and 90 keV). Two blinded readers evaluated subjective and objective image quality, presence of scar and scar pattern on a per-segment level. The diagnostic performance was evaluated as sensitivity, specificity, and accuracy. Agreement with MRI in scar detection and pattern identification were evaluated with Cohen's κ statistic.
**Results or Findings:** The LIE-CT scans of 27 patients (48% male, mean age 52.9 ± 17.2 years) were analyzed. VMI at 50 keV demonstrated an adequate objective and subjective image quality and the best trade-off between sensitivity (87.4%), specificity (97.8%), and accuracy (95.9%), in scar detection, with an excellent agreement with MRI (κ = 0.86). 50 keV also showed the highest concordance in discriminating different scar patterns, with a 100% rate for detecting subepicardial scars and patchy fibrosis.
**Conclusion:** PCD-CT VMI reconstructions at 50 keV are a valuable tool for myocardial scar detection and characterisation, demonstrating excellent diagnostic performance and agreement with cardiac MRI.
**Limitations:** Due to the sub-analysis nature of this study, selection bias needs to be considered. Due to the limited number of patients, per-patient analysis was not performed.
**Ethics committee approval:** The study is HIPAA-compliant.
**Funding for this study:** No funding was obtained for this study.
**Author Disclosures:**
Emese Zsarnóczay: Nothing to disclose
Daniel Pinos: Nothing to disclose
Milán Vecsey-Nagy: Nothing to disclose
Uwe Joseph Schoepf: Other: Institutional research support and/or personal fees from Bayer, Elucid Bioimaging, Bracco, Guerbet, HeartFlow and Siemens
Giuseppe Tremamunno: Nothing to disclose
Robert J. Holtackers: Nothing to disclose
Tilman Stephan Emrich: Other: Speaker fee and travel support from Siemens
Chiara Gnasso: Nothing to disclose
Akos Varga-Szemes: Other: Receives institutional research support and/or personal fees from Elucid BioImaging and Siemens

### RPS 2403-8

**Dual-layer spectral CT-based quantification of myocardial extracellular volume fraction using CMR as a reference in aortic valve disease**

Y. Li[1], X. He[1], J. Nan[1], W. Tian[1], X. Lu[1], L. Cao[1], Y. Wang[2], *G. Wang*[1], Z. Chen[1]; [1]Lanzhou/CN, [2]Shanghai/CN

**Purpose:** The objective of this study was to assess the agreement of myocardial extracellular volume fraction (ECV) measurements in patients with aortic valve disease (AVD) between dual-layer spectral detector CT (DLCT) and cardiovascular magnetic resonance (CMR) measurements.
**Methods or Backround:** Twenty-three patients diagnosed with AVD who underwent both simultaneous DLCT late iodine enhancement and CMR examinations were retrospectively enrolled. These examinations performed within a week. ECV values were calculated from the iodine map (CT-ECV-Iodine) and the conventional CT images (CT-ECV-HU (Hounsfield units)) derived from DLCT. CT-ECV-Iodine, CT-ECV-HU, and CMR-ECV were measured at three short axes (base, middle, and apex), according to the American Heart Association 16-segment model. Global and segmental ECVSs of cardiac is recorded. The Pearson correlation coefficient and Bland-Altman plot were used to calculate the correlation and agreements among these measurements.
**Results or Findings:** The correlation analysis revealed a strong correlation between CT-ECV-Iodine and CT-ECV-HU (r =0.954, 95% confidence interval (CI) [0.89-0.98], p <0.001), a relatively high correlation between CT-ECV-Iodine and CMR-ECV (r =0.862, 95% CI [0.70-0.94], p <0.001), and a significant correlation between CT-ECV-HU and CMR-ECV (r =0.812, 95% CI [0.60-0.92], p <0.001). The mean differences between these measurements were as follows: CT-ECV-Iodine and CT-ECV-HU exhibited a mean difference of -0.40% (with a 95% limit of agreement (LOA) ranging from -3.2% to 2.4%), CT-ECV-Iodine and CMR-ECV showed a mean difference of 0.76% (95% LOA: -3.5-5.0%), and CT-ECV-HU and CMR-ECV had a mean difference of 1.17 (95% LOA: -4.22-6.55%).
**Conclusion:** DLCT-based ECV measurements showed a strong correlation with CMR in patients with AVD. Furthermore, it is a potential alternative way for assessment of myocardial interstitial fibrosis in AVD.
**Limitations:** Small sample size is the limitation of this study.
**Ethics committee approval:** The study was conducted according to the guidelines of the Declaration of Helsinki and approved by the Ethical Committee of the first hospital of Lanzhou University.
**Funding for this study:** This study is supported by the Lanzhou Science and technology project Foundation (2020-2D-80) and First Hospital of Lanzhou University Hospital Foundation (ldyyyn2019-78).

**Author Disclosures:**
Xiande Lu: Nothing to disclose
Gang Wang: Nothing to disclose
Yu Wang: Nothing to disclose
Wei Tian: Nothing to disclose
ZiXian Chen: Nothing to disclose
Jiang Nan: Nothing to disclose
Yuxi Li: Nothing to disclose
Xiangui He: Nothing to disclose
Liang Cao: Nothing to disclose

### RPS 2403-9

**Quantification of myocardial extracellular volume and aortic valve calcification scores using dual-layer spectral CT to identify severe aortic regurgitation in patients with aortic valve disease**

Y. Li[1], X. He[1], L. Cao[1], W. Tian[1], X. Lu[1], J. Xu[1], Y. Wang[2], G. Wang[1], *Z. Chen*[1]; [1]Lan Zhou city/CN, [2]Shanghai/CN

**Purpose:** To investigate the diagnostic performance of a combination of myocardial extracellular volume fraction (ECV) and aortic valve calcification scores (AVCS) measured using dual-layer spectral detector CT (DLCT) in the identifying of severe aortic regurgitation (sAR) in patients with aortic valve disease (AVD).
**Methods or Backround:** A total of 54 patients with AVD (37 aortic regurgitation (AR), including 16 severe AR (sAR) and 21 with mild to moderate AR (mAR); 17 aortic stenosis (AS)) who underwent non-contrast and late iodine enhancement DLCT examinations were retrospectively included. The ECV map was reconstructed using a dedicated workstation, and AVCS was evaluated semi-automatically using multiplanar reconstruction by Agatston algorithm from the non-contrast phase of DLCT. AVCS scores were categorized into four grades. Student’s t-test and Mann-Whitney U test were performed. The feasibility of CT-ECV combined with AVCS grades in identifying sAR was evaluated using the receiver operating characteristic curve.
**Results or Findings:** In the sAR group, the ECV was significantly higher (32.29 [30.26-35.57]% vs 29.30 [27.08, 32.71]% , p=0.03) than non-sAR group, while the AVCS grades were significantly lower (1.88±0.48 vs 2.5±0.94, p=0.02). Using an ECV cutoff of 29.13%, the AUC for discriminating sAR was 0.75. The combined model of ECV and AVCS grades demonstrated notable diagnostic performance in distinguishing sAR from non-sAR, with an AUC of 0.82, sensitivity of 94% and specificity of 55%. To differentiate between AR severity levels, there was a significant difference in ECV between the sAR group and the mAR group (p =0.02). The AUC was 0.79, with a sensitivity of 75% and specificity of 72%.
**Conclusion:** The combination of DLCT-ECV and AVCS grades, assessed via spectral CT, shows promise for identifying severe aortic regurgitation in patients with aortic valve disease.
**Limitations:** Small sample size was a limitation of this study.
**Ethics committee approval:** The study was conducted according to the guidelines of the Declaration of Helsinki and approved by the Ethical Committee of the first hospital of Lanzhou University.
**Funding for this study:** This study is supported by the Lanzhou Science and technology project Foundation (2020-2D-80) and First Hospital of Lanzhou University Hospital Foundation (ldyyyn2019-78).
**Author Disclosures:**
Xiande Lu: Nothing to disclose
Gang Wang: Nothing to disclose
Yu Wang: Nothing to disclose
Wei Tian: Nothing to disclose
ZiXian Chen: Nothing to disclose
Jizhe Xu: Nothing to disclose
Yuxi Li: Nothing to disclose
Xiangui He: Nothing to disclose
Liang Cao: Nothing to disclose
